# Supplementary material for: Exploring online consumer behavior on fraudulent energy-saving products
Source: Sci Rep. 2024 Jun 21;14:14304. doi: 10.1038/s41598-024-65210-1 (PMC11192901; doi:10.1038/s41598-024-65210-1)
Supplement: Supplementary file 4 — Supplementary Table 4. [file 41598_2024_65210_MOESM4_ESM.pdf]

Happy Fan Shop	King of Power Saving	I just got it home and haven't used it for long, so I'll use it for a couple months and see what happens. Seeing that most people have good reviews, it should be fine.	Jingdong prefecture level city in Shandong
Happy Fan Shop	King of Power Saving	I'm looking forward to the results, so I'll give you a good review out of habit! Follow up review after a while	Jingdong prefecture level city in Shandong
Happy Fan Shop	King of Power Saving	Use a dish first to know if it will help save energy	Jingdong prefecture level city in Shandong
Happy Fan Shop	King of Power Saving	Whether it saves electricity or not, we don't know yet.	Jingdong prefecture level city in Shandong
Happy Fan Shop	King of Power Saving	Goods have been received, use a period of time to know how oh. The courier did not deliver to the door ah! Sent to the station ah! The heat is inconvenient ah!	Jingdong prefecture level city in Shandong
Happy Fan Shop	King of Power Saving	A little effect, before an hour a power, now an hour 0.76 degrees, a dollar a power = save 24 cents, although not a lot, first use it!	Jingdong prefecture level city in Shandong
Happy Fan Shop	King of Power Saving	Just received the goods, use a month to evaluate	Jingdong prefecture level city in Shandong
Happy Fan Shop	King of Power Saving	Home every month with a lot of electricity, inadvertently found this good thing online that can save the beginning of a skeptical attitude to consult customer service, customer service replied very professional, said 120 days can be refunded that first try it!	Jingdong prefecture level city in Shandong
Happy Fan Shop	King of Power Saving	After receiving the goods this afternoon, immediately installed in the bathroom Smith energy storage electric water heater on the power outlet (instead of connecting with a plug board), when used, found that the "Power Saving King" LCD display, is the value of the voltage, not the store's video said so	Jingdong prefecture level city in Shandong
Happy Fan Shop	King of Power Saving	Has made many days, did not feel the power saving, the effect is not obvious.	Jingdong prefecture level city in Shandong
Happy Fan Shop	King of Power Saving	Energy savers do not save energy at all, but rather consume a lot of electricity.	Jingdong prefecture level city in Shandong
Happy Fan Shop	King of Power Saving	It's useless. It's a waste of money.	Jingdong prefecture level city in Shandong
Happy Fan Shop	King of Power Saving	Last month saved more than 500, which came back to buy, the store bought three, and then buy 1 home use, my family is dry beauty salon, more instruments, especially electricity, a month almost 1000. or so, with this did not expect to save so much, great, five-star praise!	Jingdong prefecture level city in Shandong
Happy Fan Shop	King of Power Saving	It rained a couple days ago and I just went to get the item back today , not bad, hey I looked at the meter and that red display light takes several seconds to jump, did not make a mistake buying it. After a period of time to evaluate.	Jingdong prefecture level city in Shandong
Happy Fan Shop	King of Power Saving	Without further ado, the electricity bill came down, this month less 102 yuan, has been repurchased, I also recommended to my friends, all said that there are effects, the product is okay, the effect is quite	Jingdong prefecture level city in Shandong
Happy Fan Shop	King of Power Saving	After buying and using two months really see the effect, so only to evaluate! Originally held the mindset of trying, I did not expect really effective, worth having!	Jingdong prefecture level city in Shandong
Happy Fan Shop	King of Power Saving	In the use of this energy saver before the monthly electricity bill is more than 150 yuan or so, now save thirty percent, every day to work, six or seven o'clock in the afternoon to go home, I do not know why the electricity bill is so expensive, energy-saving appliances really have the effect of recommending	Jingdong prefecture level city in Shandong
Happy Fan Shop	King of Power Saving	It saves about 100 bucks a month. A friend recommended it to me, and he's been using it for a while, and the effect is so obvious that I bought one too.	Jingdong prefecture level city in Shandong
Happy Fan Shop	King of Power Saving	My dad asked me to buy one, but after I paid the bill, I asked and he said it was a lot less this month, so he asked me to buy another one.	Jingdong prefecture level city in Shandong
Happy Fan Shop	King of Power Saving	The specific effect will have to be tested for at least two months to compare whether there is a power saving effect or not.	Jingdong prefecture level city in Shandong
Happy Fan Shop	King of Power Saving	'The goods have been received, immediately go to use, please ask the boss you instructions do not allow to withdraw, there are two fuses installed there!	Jingdong prefecture level city in Shandong
Happy Fan Shop	King of Power Saving	First praise is using, after using if good then follow up, now because I have not yet seen the results	Jingdong prefecture level city in Shandong
Happy Fan Shop	King of Power Saving	I've just been using it, and I haven't seen if it has a power-saving effect yet, so I'll follow up after 10 days and half a month.	Jingdong prefecture level city in Shandong
Happy Fan Shop	King of Power Saving	Just received the intelligent power saving meter, immediately plugged in to use, see it is not bad, and order to sell the second time	Jingdong prefecture level city in Shandong
Happy Fan Shop	King of Power Saving	This power saver is a bit effective, used for half a month, the effect is still visible.	Jingdong prefecture level city in Shandong
Happy Fan Shop	King of Power Saving	I received the baby and it looks great, I'm trying it out now and will keep an eye on it afterward.	Jingdong prefecture level city in Shandong
Happy Fan Shop	King of Power Saving	I've just received it, but I'm still trying to see how it works, so I'll come back in a little while.	Jingdong prefecture level city in Shandong
Happy Fan Shop	King of Power Saving	Just used it wait to use it for a while and then comment thanks, all first five stars.	Jingdong prefecture level city in Shandong
Happy Fan Shop	King of Power Saving	Very trustworthy brand, quality is guaranteed, the price is still affordable, quite satisfied!	Jingdong prefecture level city in Shandong
Happy Fan Shop	King of Power Saving	Things received, I hope that the platform of Jingdong do not sell fake goods are available	Jingdong prefecture level city in Shandong
Happy Fan Shop	King of Power Saving	Energy saver received, the use of the effect is good and the business description of the same	Jingdong prefecture level city in Shandong
Happy Fan Shop	King of Power Saving	The surface looks beautiful, the actual effect is not known, after using additional evaluation.	Jingdong prefecture level city in Shandong
Happy Fan Shop	King of Power Saving	Tried it for a few months, it really saves electricity, and I bought it for a friend.	Jingdong prefecture level city in Shandong
Happy Fan Shop	King of Power Saving	It didn't work and wanted to return it, it really didn't work at all.	Jingdong prefecture level city in Shandong
Happy Fan Shop	King of Power Saving	Just used it, don't know how effective it is, looking forward to usefulness	Jingdong prefecture level city in Shandong
Happy Fan Shop	King of Power Saving	After two months before the effect I do not know if it is true!	Jingdong prefecture level city in Shandong
Happy Fan Shop	King of Power Saving	So fast! Ordered yesterday and received today 👍👍👍👍👍👍👍👍👍 🌹🌹🌹🌹🌹🌹🌹🌹🌹	Jingdong prefecture level city in Shandong
Happy Fan Shop	King of Power Saving	Things are more perfect than I imagined, very powerful, it is worth getting Packaging is very strong, things are well protected, it is worth the praise things are very satisfied Oh, beautiful and stylish atmosphere, customer service is super patient, service is meticulous and attentive, the big brand is not	Jingdong prefecture level city in Shandong
Happy Fan Shop	King of Power Saving	The effect is too good, the family has now several units, back to buy a number of times, the power saver received, power-saving effect is very good,, fine workmanship, long warranty, but also purify the air, before buying a used for a period of time, with the okay, and then bought a, and only now come to the	Jingdong prefecture level city in Shandong
Happy Fan Shop	King of Power Saving	Quite good, and imagine the same, casual ride are very good, satisfied! Baby received it 👌, really great ah! 🌈 quality is really nothing to say, very satisfied! 🆗 happy happy 😃 genuine, great, the price is very good, in short, quite good, will buy again a very good shopping experience, attentive service, fast logistics, distribution is also thoughtful, the attitude is also very good, value for money, ah,	Jingdong prefecture level city in Shandong
Happy Fan Shop	King of Power Saving	Power-saving effect: strong Cost-effective: quite good Appearance style: beautiful Workmanship material: meticulous Experience: looking forward to power-saving effect	Jingdong prefecture level city in Shandong
Happy Fan Shop	King of Power Saving	Cost-effective is okay, the appearance of the style of meticulous workmanship, looking forward to energy-saving effect.	Jingdong prefecture level city in Shandong
Happy Fan Shop	King of Power Saving	Very Good Very Power Saving This Power Saver is really power saving As soon as the power is turned on, the Power Saver starts the power saving function for you.	Jingdong prefecture level city in Shandong
Happy Fan Shop	King of Power Saving	Logistics is very fast, the price is favorable, friends introduced that it is very good to use, bought 2 at once, looking forward to the effect, used after the follow-up comment.	Jingdong prefecture level city in Shandong
Happy Fan Shop	King of Power Saving	It's all fake. It doesn't save any electricity.	Jingdong prefecture level city in Shandong
Happy Fan Shop	King of Power Saving	This Power Saver is so much better than the one I bought before, not only the effect is good, the quality is very good in all aspects, the customer service attitude is also very good, recommended to buy!	Jingdong prefecture level city in Shandong

Happy Fan Shop	King of Power Saving	Logistics is very fast, buy for parents, good quality, once bought 2, power-saving effect is good to buy 2 more, look forward to using the effect, satisfied will recommend to friends.	Jingdong prefecture level city in Shandong
Happy Fan Shop	King of Power Saving	Things are more perfect than I imagined, very powerful, it is worth getting Packaging is very strong, things are well protected, it is worth the praise things are very satisfied Oh, beautiful and stylish atmosphere, customer service is super patient, service is meticulous and attentive, the big brand is not	Jingdong prefecture level city in Shandong
Happy Fan Shop	King of Power Saving	The effect is too good, the family has now several units, back to buy a number of times, the power saver received, power-saving effect is very good,, fine workmanship, long warranty, but also purify the air, before buying a used for a period of time, with the okay, and then bought a, and only now come to the	Jingdong prefecture level city in Shandong
Happy Fan Shop	King of Power Saving	Quite good, and imagine the same, casual ride are very good, satisfied! Baby received it 👌, really great ah! 🌈 quality is really nothing to say, very satisfied! 🆗 happy happy 😀 genuine, great, the price is very good, in short, quite good, will buy again a very good shopping experience, attentive service, fast logistics, distribution is also thoughtful, the attitude is also very good, value for money, ah,	Jingdong prefecture level city in Shandong
Happy Fan Shop	King of Power Saving	Power-saving effect: strong Cost-effective: quite good Appearance style: beautiful Workmanship material: meticulous Experience: looking forward to power-saving effect	Jingdong prefecture level city in Shandong
Happy Fan Shop	King of Power Saving	Cost-effective is okay, the appearance of the style of meticulous workmanship, looking forward to energy-saving effect.	Jingdong prefecture level city in Shandong
Happy Fan Shop	King of Power Saving	Very Good Very Power Saving This Power Saver is really power saving As soon as the power is turned on, the Power Saver starts the power saving function for you.	Jingdong prefecture level city in Shandong
Happy Fan Shop	King of Power Saving	Logistics is very fast, the price is favorable, friends introduced that it is very good to use, bought 2 at once, looking forward to the effect, used after the follow-up comment.	Jingdong prefecture level city in Shandong
Happy Fan Shop	King of Power Saving	It's all fake. It doesn't save any electricity.	Jingdong prefecture level city in Shandong
Happy Fan Shop	King of Power Saving	This Power Saver is so much better than the one I bought before, not only the effect is good, the quality is very good in all aspects, the customer service attitude is also very good, recommended to buy!	Jingdong prefecture level city in Shandong
Happy Fan Shop	King of Power Saving	Logistics is very fast, buy for parents, good quality, once bought 2, power-saving effect is good to buy 2 more, look forward to using the effect, satisfied will recommend to friends.	Jingdong prefecture level city in Shandong
Happy Fan Shop	King of Power Saving	Things are more perfect than I imagined, very powerful, it is worth getting Packaging is very strong, things are well protected, it is worth the praise things are very satisfied Oh, beautiful and stylish atmosphere, customer service is super patient, service is meticulous and attentive, the big brand is not	Jingdong prefecture level city in Shandong
Happy Fan Shop	King of Power Saving	The effect is too good, the family has now several units, back to buy a number of times, the power saver received, power-saving effect is very good,, fine workmanship, long warranty, but also purify the air, before buying a used for a period of time, with the okay, and then bought a, and only now come to the	Jingdong prefecture level city in Shandong
Happy Fan Shop	King of Power Saving	Quite good, and imagine the same, casual ride are very good, satisfied! Baby received it 👌, really great ah! 🌈 quality is really nothing to say, very satisfied! 🆗 happy happy 😀 genuine, great, the price is very good, in short, quite good, will buy again a very good shopping experience, attentive service, fast logistics, distribution is also thoughtful, the attitude is also very good, value for money, ah,	Jingdong prefecture level city in Shandong
Happy Fan Shop	King of Power Saving	Power-saving effect: strong Cost-effective: quite good Appearance style: beautiful Workmanship material: meticulous Experience: looking forward to power-saving effect	Jingdong prefecture level city in Shandong
Happy Fan Shop	King of Power Saving	Cost-effective is okay, the appearance of the style of meticulous workmanship, looking forward to energy-saving effect.	Jingdong prefecture level city in Shandong
Happy Fan Shop	King of Power Saving	Very Good Very Power Saving This Power Saver is really power saving As soon as the power is turned on, the Power Saver starts the power saving function for you.	Jingdong prefecture level city in Shandong
Happy Fan Shop	King of Power Saving	Logistics is very fast, the price is favorable, friends introduced that it is very good to use, bought 2 at once, looking forward to the effect, used after the follow-up comment.	Jingdong prefecture level city in Shandong
Happy Fan Shop	King of Power Saving	It's all fake. It doesn't save any electricity.	Jingdong prefecture level city in Shandong
Happy Fan Shop	King of Power Saving	This Power Saver is so much better than the one I bought before, not only the effect is good, the quality is very good in all aspects, the customer service attitude is also very good, recommended to buy!	Jingdong prefecture level city in Shandong
Happy Fan Shop	King of Power Saving	Logistics is very fast, buy for parents, good quality, once bought 2, power-saving effect is good to buy 2 more, look forward to using the effect, satisfied will recommend to friends.	Jingdong prefecture level city in Shandong
Happy Fan Shop	King of Power Saving	With the description is the same, very high-grade, good value, to buy friends can order Oh!	Jingdong prefecture level city in Shandong
Happy Fan Shop	King of Power Saving	Not using much electricity in the winter saving over 100 per month! Ready to buy 2 more units in the summer ! ! ! ! ! Good quality, good price, power saving effect is obvious, cost-effective, worth buying, can be recommended!	Jingdong prefecture level city in Shandong
Happy Fan Shop	King of Power Saving	Purchased a power saver received on the put into use, rent a room with many electrical appliances, power consumption is very large, we have a piece of electricity or 1.2 dollars a time, with this power saver is much better, every day can save a lot of money, very convenient and practical.	Jingdong prefecture level city in Shandong
Happy Fan Shop	King of Power Saving	The power saving effect is very good: my supermarket monthly electricity bill is more than 7000 yuan. On May 1st someone recommended buying a couple of power savers, and I bought 5 of them first. The electricity bill was about 2000 less in June. This time, I bought 5 more. The seller said 800 degrees of electricity with one. I feel it's basically enough. A total of 10 units	Jingdong prefecture level city in Shandong
Happy Fan Shop	King of Power Saving	I never thought I could get such a good baby at such an affordable price, it's very good value for money, I love it!	Jingdong prefecture level city in Shandong
Happy Fan Shop	King of Power Saving	Previously bought other brands of power savers, but found no effect at home, this time to see how the products on the Jingdong, inserted in the home socket, the LED display is blue, the night light is very nice to see, look at the instructions that have to be half a year in order to achieve the best results, try it, I believe that the quality of the Jingdong, it should not be wrong.	Jingdong prefecture level city in Shandong
Happy Fan Shop	King of Power Saving	Superb, inexpensive, saves a lot of points, been using it for a long time!	Jingdong prefecture level city in Shandong
Happy Fan Shop	King of Power Saving	There are a lot of appliances in the house, the summer air conditioning is very costly, I bought this in front of the air conditioning effect is very good, I explain that I bought three in one breath, I use more electricity a month down to 200 or so, very satisfied.	Jingdong prefecture level city in Shandong
Happy Fan Shop	King of Power Saving	A month can save more than 200, home monthly electricity bill of more than 600 dollars, inserted 3 or so, an amount of the balance of the average can save more than 200. Product is very good, has been buying this one, has given a lot of relatives recommended, the quality is also very good, before buying a look with this same, who knows to buy home has not been used for more than a week on the bad, the quality of this is really good with more than two years!	Jingdong prefecture level city in Shandong
Happy Fan Shop	King of Power Saving	Whole house power saving king, can really save electricity, friends recommended, high power saving appliances, only replacement, no repair, after-sales worry-free!	Jingdong prefecture level city in Shandong
Happy Fan Shop	King of Power Saving	Great item love it, great customer service, great shopping experience!	Jingdong prefecture level city in Shandong
Happy Fan Shop	King of Power Saving	Power saving, very good use, much cheaper than the physical store, satisfaction	Jingdong prefecture level city in Shandong
Happy Fan Shop	King of Power Saving	In use, very well packaged and fast delivery of credit!	Jingdong prefecture level city in Shandong
Happy Fan Shop	King of Power Saving	Product quality, customer service attitude is particularly good, like, praise!	Jingdong prefecture level city in Shandong
Happy Fan Shop	King of Power Saving	The logistics is very fast, the packaging is not broken, the quality is very good, I like it very much!	Jingdong prefecture level city in Shandong
Happy Fan Shop	King of Power Saving	The saver was well packaged and was as described when plugged in	Jingdong prefecture level city in Shandong
Happy Fan Shop	King of Power Saving	Energy efficient and very satisfied	Jingdong prefecture level city in Shandong
Happy Fan Shop	King of Power Saving	Great quality for the price and fast shipping.	Jingdong prefecture level city in Shandong
Happy Fan Shop	King of Power Saving	It's great, just as described. Perfect.	Jingdong prefecture level city in Shandong
Happy Fan Shop	King of Power Saving	There is no power saving effect, but rather ring up an increase in electricity costs, because this machine is on continuously all day long, uninterruptedly, its own electricity and increased costs, plus the cost of purchasing the machine, so I feel that the use of this machine instead of increasing the	Jingdong prefecture level city in Shandong
Happy Fan Shop	King of Power Saving	Recommended by the owner of the store next door, looked at his home does have to save electricity, decided to get. Indeed quite can, the voltage is much more stable, there is a reduction in electricity	Jingdong prefecture level city in Shandong

Happy Fan Shop	King of Power Saving	Goods have been received value for money, affordable and reasonable price, and the description of the same, power-saving safe and reliable!	Jingdong prefecture level city in Shandong
Happy Fan Shop	King of Power Saving	Listen to friends introduced to buy, tried to save a lot of electricity is good, quite good, worth buying.	Jingdong prefecture level city in Shandong
Happy Fan Shop	King of Power Saving	The product is easy to use, safe and environmentally friendly, only used for a short time, to the next month, after the electricity bill status display to know how the performance of the power saver efficacy.	Jingdong prefecture level city in Shandong
Happy Fan Shop	King of Power Saving	Last month's electricity bill is 178 yuan, with this power saver is 92 yuan, ready to buy two more back home, I was introduced to buy a friend, just started to not believe that I did not expect the effect!	Jingdong prefecture level city in Shandong
Happy Fan Shop	King of Power Saving	It looks nice, but after using it for a few days I can't see how it saves power?	Jingdong prefecture level city in Shandong
Happy Fan Shop	King of Power Saving	Recommended by a friend, it is genuine, as described by the merchant, cost-effective	Jingdong prefecture level city in Shandong
Happy Fan Shop	King of Power Saving	Works well, affordable and saves money.	Jingdong prefecture level city in Shandong
Happy Fan Shop	King of Power Saving	Feels good haven't compared it yet, the air conditioning has been on a lot in the past two months	Jingdong prefecture level city in Shandong
Happy Fan Shop	King of Power Saving	Saving about 5 kWh per day is a good thing	Jingdong prefecture level city in Shandong
Happy Fan Shop	King of Power Saving	Not bad, affordable, best value for money	Jingdong prefecture level city in Shandong
Happy Fan Shop	King of Power Saving	The goods are genuine and the logistics are faster.	Jingdong prefecture level city in Shandong
Happy Fan Shop	King of Power Saving	Goods have been received today, has been plugged into the outlet, looking forward to saving electricity, after a period of time to see how the effect in the follow-up comment!	Jingdong prefecture level city in Shandong
Happy Fan Shop	King of Power Saving	I just got it home and haven't used it for long, so I'll use it for a couple months and see what happens. Seeing that most people have good reviews, it should be fine.	Jingdong prefecture level city in Shandong
Happy Fan Shop	King of Power Saving	I'm looking forward to the results, so I'll give you a good review out of habit! Follow up review after a while	Jingdong prefecture level city in Shandong
Happy Fan Shop	King of Power Saving	Use a dish first to know if it will help save energy	Jingdong prefecture level city in Shandong
Happy Fan Shop	King of Power Saving	Whether it saves electricity or not, we don't know yet.	Jingdong prefecture level city in Shandong
Happy Fan Shop	King of Power Saving	Goods have been received, use a period of time to know how oh. The courier did not deliver to the door ah! Sent to the station ah! The heat is inconvenient ah!	Jingdong prefecture level city in Shandong
Happy Fan Shop	King of Power Saving	A little effect, before an hour a power, now an hour 0.76 degrees, a dollar a power = save 24 cents, although not a lot, first use it!	Jingdong prefecture level city in Shandong
Happy Fan Shop	King of Power Saving	Just received the goods, use a month to evaluate	Jingdong prefecture level city in Shandong
Happy Fan Shop	King of Power Saving	Home every month with a lot of electricity, inadvertently found this good thing online that can save the beginning of a skeptical attitude to consult customer service, customer service replied very professional, said 120 days can be refunded that first try it!	Jingdong prefecture level city in Shandong
Happy Fan Shop	King of Power Saving	After receiving the goods this afternoon, immediately installed in the bathroom Smith energy storage electric water heater on the power outlet (instead of connecting with a plug board), when used, found that the "Power Saving King" LCD display, is the value of the voltage, not the store's video said so	Jingdong prefecture level city in Shandong
Happy Fan Shop	King of Power Saving	Has made many days, did not feel the power saving, the effect is not obvious.	Jingdong prefecture level city in Shandong
Happy Fan Shop	King of Power Saving	Energy savers do not save energy at all, but rather consume a lot of electricity.	Jingdong prefecture level city in Shandong
Happy Fan Shop	King of Power Saving	It's useless. It's a waste of money.	Jingdong prefecture level city in Shandong
Happy Fan Shop	King of Power Saving	Last month saved more than 500, which came back to buy, the store bought three, and then buy 1 home use, my family is dry beauty salon, more instruments, especially electricity, a month almost 1000. or so, with this did not expect to save so much, great, five-star praise!	Jingdong prefecture level city in Shandong
Happy Fan Shop	King of Power Saving	It rained a couple days ago and I just went to get the item back today , not bad, hey I looked at the meter and that red display light takes several seconds to jump, did not make a mistake buying it. After a period of time to evaluate.	Jingdong prefecture level city in Shandong
Happy Fan Shop	King of Power Saving	Without further ado, the electricity bill came down, this month less 102 yuan, has been repurchased, I also recommended to my friends, all said that there are effects, the product is okay, the effect is quite	Jingdong prefecture level city in Shandong
Happy Fan Shop	King of Power Saving	After buying and using two months really see the effect, so only to evaluate! Originally held the mindset of trying, I did not expect really effective, worth having!	Jingdong prefecture level city in Shandong
Happy Fan Shop	King of Power Saving	In the use of this energy saver before the monthly electricity bill is more than 150 yuan or so, now save thirty percent, every day to work, six or seven o'clock in the afternoon to go home, I do not know why the electricity bill is so expensive, energy-saving appliances really have the effect of recommending	Jingdong prefecture level city in Shandong
Happy Fan Shop	King of Power Saving	It saves about 100 bucks a month. A friend recommended it to me, and he's been using it for a while, and the effect is so obvious that I bought one too.	Jingdong prefecture level city in Shandong
Happy Fan Shop	King of Power Saving	My dad asked me to buy one, but after I paid the bill, I asked and he said it was a lot less this month, so he asked me to buy another one.	Jingdong prefecture level city in Shandong
Happy Fan Shop	King of Power Saving	The specific effect will have to be tested for at least two months to compare whether there is a power saving effect or not.	Jingdong prefecture level city in Shandong
Happy Fan Shop	King of Power Saving	'The goods have been received, immediately go to use, please ask the boss you instructions do not allow to withdraw, there are two fuses installed there!	Jingdong prefecture level city in Shandong
Happy Fan Shop	King of Power Saving	First praise is using, after using if good then follow up, now because I have not yet seen the results	Jingdong prefecture level city in Shandong
Happy Fan Shop	King of Power Saving	I've just been using it, and I haven't seen if it has a power-saving effect yet, so I'll follow up after 10 days and half a month.	Jingdong prefecture level city in Shandong
Happy Fan Shop	King of Power Saving	Just received the intelligent power saving meter, immediately plugged in to use, see it is not bad, and order to sell the second time	Jingdong prefecture level city in Shandong
Happy Fan Shop	King of Power Saving	This power saver is a bit effective, used for half a month, the effect is still visible.	Jingdong prefecture level city in Shandong
Happy Fan Shop	King of Power Saving	I received the baby and it looks great, I'm trying it out now and will keep an eye on it afterward.	Jingdong prefecture level city in Shandong
Happy Fan Shop	King of Power Saving	I've just received it, but I'm still trying to see how it works, so I'll come back in a little while.	Jingdong prefecture level city in Shandong
Happy Fan Shop	King of Power Saving	Just used it wait to use it for a while and then comment thanks, all first five stars.	Jingdong prefecture level city in Shandong
Happy Fan Shop	King of Power Saving	Very trustworthy brand, quality is guaranteed, the price is still affordable, quite satisfied!	Jingdong prefecture level city in Shandong
Happy Fan Shop	King of Power Saving	Things received, I hope that the platform of Jingdong do not sell fake goods are available	Jingdong prefecture level city in Shandong
Happy Fan Shop	King of Power Saving	Energy saver received, the use of the effect is good and the business description of the same	Jingdong prefecture level city in Shandong
Happy Fan Shop	King of Power Saving	The surface looks beautiful, the actual effect is not known, after using additional evaluation.	Jingdong prefecture level city in Shandong
Happy Fan Shop	King of Power Saving	Tried it for a few months, it really saves electricity, and I bought it for a friend.	Jingdong prefecture level city in Shandong
Happy Fan Shop	King of Power Saving	It didn't work and wanted to return it, it really didn't work at all.	Jingdong prefecture level city in Shandong
Happy Fan Shop	King of Power Saving	Just used it, don't know how effective it is, looking forward to usefulness	Jingdong prefecture level city in Shandong
Happy Fan Shop	King of Power Saving	After two months before the effect I do not know if it is true!	Jingdong prefecture level city in Shandong
Happy Fan Shop	King of Power Saving	So fast! Ordered yesterday and received today 👍👍👍👍👍👍👍👍👍 🌹🌹🌹🌹🌹🌹🌹🌹🌹	Jingdong prefecture level city in Shandong

Happy Fan Shop	King of Power Saving	I've started using it. We'll see how it works.	Jingdong prefecture level city in Shandong
Happy Fan Shop	King of Power Saving	I just received the goods, I won't know the effect until after two.	Jingdong prefecture level city in Shandong
Happy Fan Shop	King of Power Saving	Just received the goods, the specific effect will be evaluated later	Jingdong prefecture level city in Shandong
Happy Fan Shop	King of Power Saving	I've received the goods, I don't know if it's any good yet	Jingdong prefecture level city in Shandong
Happy Fan Shop	King of Power Saving	Try it for a month and then come back with a concrete evaluation	Jingdong prefecture level city in Shandong
Happy Fan Shop	King of Power Saving	In use, after two months to see the effect of power saving	Jingdong prefecture level city in Shandong
Happy Fan Shop	King of Power Saving	Power Saver. It's authentic. It's black. It works.	Jingdong prefecture level city in Shandong
Happy Fan Shop	King of Power Saving	I've been using it for a week now and it's not working.	Jingdong prefecture level city in Shandong
Happy Fan Shop	King of Power Saving	I'm just using it. I don't know how it's going to work.	Jingdong prefecture level city in Shandong
Happy Fan Shop	King of Power Saving	Products are better looking out for power savings	Jingdong prefecture level city in Shandong
Happy Fan Shop	King of Power Saving	I received it and I've kept it on. I hope it works.	Jingdong prefecture level city in Shandong
Happy Fan Shop	King of Power Saving	It's really good! Worth recommending to buy!	Jingdong prefecture level city in Shandong
Happy Fan Shop	King of Power Saving	Try it for a few days and see if it doesn't work, then return it.	Jingdong prefecture level city in Shandong
Happy Fan Shop	King of Power Saving	It's in use and I hope to be pleasantly surprised.	Jingdong prefecture level city in Shandong
Happy Fan Shop	King of Power Saving	We'll see how it works when we use it	Jingdong prefecture level city in Shandong
Happy Fan Shop	King of Power Saving	Let's see if it works.	Jingdong prefecture level city in Shandong
Happy Fan Shop	King of Power Saving	I'm using it and will follow up with a review	Jingdong prefecture level city in Shandong
Happy Fan Shop	King of Power Saving	It really works. It saves energy.	Jingdong prefecture level city in Shandong
Happy Fan Shop	King of Power Saving	It does have some power savings!	Jingdong prefecture level city in Shandong
Happy Fan Shop	King of Power Saving	Jingdong shopping speed is convenient and timely	Jingdong prefecture level city in Shandong
Happy Fan Shop	King of Power Saving	Second time I've bought it. Everything's fine.	Jingdong prefecture level city in Shandong
Happy Fan Shop	King of Power Saving	I don't know if it works yet.	Jingdong prefecture level city in Shandong
Happy Fan Shop	King of Power Saving	Not used, don't know how well it works.	Jingdong prefecture level city in Shandong
Happy Fan Shop	King of Power Saving	No visible results so far	Jingdong prefecture level city in Shandong
Happy Fan Shop	King of Power Saving	It doesn't feel like it's working.	Jingdong prefecture level city in Shandong
Happy Fan Shop	King of Power Saving	Where's the effect? Where are the power savings?	Jingdong prefecture level city in Shandong
Happy Fan Shop	King of Power Saving	Let's see how it works in practice!	Jingdong prefecture level city in Shandong
Happy Fan Shop	King of Power Saving	I'll wait until I use it to give you a good review	Jingdong prefecture level city in Shandong
Happy Fan Shop	King of Power Saving	I'll follow up after I've used it	Jingdong prefecture level city in Shandong
Happy Fan Shop	King of Power Saving	Well, that's good for saving power.	Jingdong prefecture level city in Shandong
Happy Fan Shop	King of Power Saving	Received. Used in the know.	Jingdong prefecture level city in Shandong
Happy Fan Shop	King of Power Saving	It doesn't do much good.	Jingdong prefecture level city in Shandong
Happy Fan Shop	King of Power Saving	It's not working.	Jingdong prefecture level city in Shandong
Happy Fan Shop	King of Power Saving	I don't know. I just used it.	Jingdong prefecture level city in Shandong
Happy Fan Shop	King of Power Saving	Use it and come back to evaluate.	Jingdong prefecture level city in Shandong
Happy Fan Shop	King of Power Saving	Two words: useless.	Jingdong prefecture level city in Shandong
Happy Fan Shop	King of Power Saving	To be used and then evaluated	Jingdong prefecture level city in Shandong
Happy Fan Shop	King of Power Saving	No shipment.	Jingdong prefecture level city in Shandong
Happy Fan Shop	King of Power Saving	Fake. Speechless.	Jingdong prefecture level city in Shandong
Happy Fan Shop	King of Power Saving	fantastic (idiom); awesome	Jingdong prefecture level city in Shandong
Happy Fan Shop	King of Power Saving	an excellent	Jingdong prefecture level city in Shandong
Happy Fan Shop	King of Power Saving	an excellent	Jingdong prefecture level city in Shandong
Happy Fan Shop	King of Power Saving	Want to buy! Recommended not to buy! No effect	Jingdong prefecture level city in Shandong
Happy Fan Shop	King of Power Saving	That's all there is to it. I can't figure out how to save electricity.	Jingdong prefecture level city in Shandong
Happy Fan Shop	King of Power Saving	It's no use.	Jingdong prefecture level city in Shandong
Happy Fan Shop	King of Power Saving	Things are more perfect than I imagined, very powerful, it is worth getting Packaging is very strong, things are well protected, it is worth the praise things are very satisfied Oh, beautiful and stylish atmosphere, customer service is super patient, service is meticulous and attentive, the big brand is not	Jingdong prefecture level city in Shandong
Happy Fan Shop	King of Power Saving	The effect is too good, the family has now several units, back to buy a number of times, the power saver received, power-saving effect is very good,, fine workmanship, long warranty, but also purify the air, before buying a used for a period of time, with the okay, and then bought a, and only now come to the	Jingdong prefecture level city in Shandong
Happy Fan Shop	King of Power Saving	Quite good, and imagine the same, casual ride are very good, satisfied! Baby received it 👌, really great ah! 🌈 quality is really nothing to say, very satisfied! 🆗 happy happy 😃 genuine, great, the price is very good, in short, quite good, will buy again a very good shopping experience, attentive service, fast logistics, distribution is also thoughtful, the attitude is also very good, value for money, ah,	Jingdong prefecture level city in Shandong
Happy Fan Shop	King of Power Saving	Power-saving effect: strong Cost-effective: quite good Appearance style: beautiful Workmanship material: meticulous Experience: looking forward to power-saving effect	Jingdong prefecture level city in Shandong

Happy Fan Shop	King of Power Saving	Cost-effective is okay, the appearance of the style of meticulous workmanship, looking forward to energy-saving effect.	Jingdong prefecture level city in Shandong
Happy Fan Shop	King of Power Saving	Very Good Very Power Saving This Power Saver is really power saving As soon as the power is turned on, the Power Saver starts the power saving function for you.	Jingdong prefecture level city in Shandong
Happy Fan Shop	King of Power Saving	Logistics is very fast, the price is favorable, friends introduced that it is very good to use, bought 2 at once, looking forward to the effect, used after the follow-up comment.	Jingdong prefecture level city in Shandong
Happy Fan Shop	King of Power Saving	It's all fake. It doesn't save any electricity.	Jingdong prefecture level city in Shandong
Happy Fan Shop	King of Power Saving	This Power Saver is so much better than the one I bought before, not only the effect is good, the quality is very good in all aspects, the customer service attitude is also very good, recommended to buy!	Jingdong prefecture level city in Shandong
Happy Fan Shop	King of Power Saving	Logistics is very fast, buy for parents, good quality, once bought 2, power-saving effect is good to buy 2 more, look forward to using the effect, satisfied will recommend to friends.	Jingdong prefecture level city in Shandong
Happy Fan Shop	King of Power Saving	With the description is the same, very high-grade, good value, to buy friends can order Oh!	Jingdong prefecture level city in Shandong
Happy Fan Shop	King of Power Saving	Not using much electricity in the winter saving over 100 per month! Ready to buy 2 more units in the summer ！！！！！ Good quality, good price, power saving effect is obvious, cost-effective, worth buying, can be recommended!	Jingdong prefecture level city in Shandong
Happy Fan Shop	King of Power Saving	Purchased a power saver received on the put into use, rent a room with many electrical appliances, power consumption is very large, we have a piece of electricity or 1.2 dollars a time, with this power saver is much better, every day can save a lot of money, very convenient and practical.	Jingdong prefecture level city in Shandong
Happy Fan Shop	King of Power Saving	The power saving effect is very good: my supermarket monthly electricity bill is more than 7000 yuan. On May 1st someone recommended buying a couple of power savers, and I bought 5 of them first. The electricity bill was about 2000 less in June. This time, I bought 5 more. The seller said 800 degrees of electricity with one. I feel it's basically enough. A total of 10 units	Jingdong prefecture level city in Shandong
Happy Fan Shop	King of Power Saving	I never thought I could get such a good baby at such an affordable price, it's very good value for money, I love it!	Jingdong prefecture level city in Shandong
Happy Fan Shop	King of Power Saving	Previously bought other brands of power savers, but found no effect at home, this time to see how the products on the Jingdong, inserted in the home socket, the LED display is blue, the night light is very nice to see, look at the instructions that have to be half a year in order to achieve the best results, try it, I believe that the quality of the Jingdong, it should not be wrong.	Jingdong prefecture level city in Shandong
Happy Fan Shop	King of Power Saving	Superb, inexpensive, saves a lot of points, been using it for a long time!	Jingdong prefecture level city in Shandong
Happy Fan Shop	King of Power Saving	There are a lot of appliances in the house, the summer air conditioning is very costly, I bought this in front of the air conditioning effect is very good, I explain that I bought three in one breath, I use more electricity a month down to 200 or so, very satisfied.	Jingdong prefecture level city in Shandong
Happy Fan Shop	King of Power Saving	A month can save more than 200, home monthly electricity bill of more than 600 dollars, inserted 3 or so, an amount of the balance of the average can save more than 200. Product is very good, has been buying this one, has given a lot of relatives recommended, the quality is also very good, before buying a look with this same, who knows to buy home has not been used for more than a week on the bad, the quality of this is really good with more than two years!	Jingdong prefecture level city in Shandong
Happy Fan Shop	King of Power Saving	Whole house power saving king, can really save electricity, friends recommended, high power saving appliances, only replacement, no repair, after-sales worry-free!	Jingdong prefecture level city in Shandong
Happy Fan Shop	King of Power Saving	Great item love it, great customer service, great shopping experience!	Jingdong prefecture level city in Shandong
Happy Fan Shop	King of Power Saving	Power saving, very good use, much cheaper than the physical store, satisfaction	Jingdong prefecture level city in Shandong
Happy Fan Shop	King of Power Saving	In use, very well packaged and fast delivery of credit!	Jingdong prefecture level city in Shandong
Happy Fan Shop	King of Power Saving	Product quality, customer service attitude is particularly good, like, praise!	Jingdong prefecture level city in Shandong
Happy Fan Shop	King of Power Saving	The logistics is very fast, the packaging is not broken, the quality is very good, I like it very much!	Jingdong prefecture level city in Shandong
Happy Fan Shop	King of Power Saving	The saver was well packaged and was as described when plugged in	Jingdong prefecture level city in Shandong
Happy Fan Shop	King of Power Saving	Energy efficient and very satisfied	Jingdong prefecture level city in Shandong
Happy Fan Shop	King of Power Saving	Great quality for the price and fast shipping.	Jingdong prefecture level city in Shandong
Happy Fan Shop	King of Power Saving	It's great, just as described. Perfect.	Jingdong prefecture level city in Shandong
Happy Fan Shop	King of Power Saving	There is no power saving effect, but rather ring up an increase in electricity costs, because this machine is on continuously all day long, uninterruptedly, its own electricity and increased costs, plus the cost of purchasing the machine, so I feel that the use of this machine instead of increasing the	Jingdong prefecture level city in Shandong
Happy Fan Shop	King of Power Saving	Recommended by the owner of the store next door, looked at his home does have to save electricity, decided to get. Indeed quite can, the voltage is much more stable, there is a reduction in electricity	Jingdong prefecture level city in Shandong
Happy Fan Shop	King of Power Saving	Goods have been received value for money, affordable and reasonable price, and the description of the same, power-saving safe and reliable!	Jingdong prefecture level city in Shandong
Happy Fan Shop	King of Power Saving	Listen to friends introduced to buy, tried to save a lot of electricity is good, quite good, worth buying.	Jingdong prefecture level city in Shandong
Happy Fan Shop	King of Power Saving	The product is easy to use, safe and environmentally friendly, only used for a short time, to the next month, after the electricity bill status display to know how the performance of the power saver efficacy.	Jingdong prefecture level city in Shandong
Happy Fan Shop	King of Power Saving	Last month's electricity bill is 178 yuan, with this power saver is 92 yuan, ready to buy two more back home, I was introduced to buy a friend, just started to not believe that I did not expect the effect!	Jingdong prefecture level city in Shandong
Happy Fan Shop	King of Power Saving	It looks nice, but after using it for a few days I can't see how it saves power?	Jingdong prefecture level city in Shandong
Happy Fan Shop	King of Power Saving	Recommended by a friend, it is genuine, as described by the merchant, cost-effective	Jingdong prefecture level city in Shandong
Happy Fan Shop	King of Power Saving	Works well, affordable and saves money.	Jingdong prefecture level city in Shandong
Happy Fan Shop	King of Power Saving	Feels good haven't compared it yet, the air conditioning has been on a lot in the past two months	Jingdong prefecture level city in Shandong
Happy Fan Shop	King of Power Saving	Saving about 5 kWh per day is a good thing	Jingdong prefecture level city in Shandong
Happy Fan Shop	King of Power Saving	Not bad, affordable, best value for money	Jingdong prefecture level city in Shandong
Happy Fan Shop	King of Power Saving	The goods are genuine and the logistics are faster.	Jingdong prefecture level city in Shandong
Happy Fan Shop	King of Power Saving	Goods have been received today, has been plugged into the outlet, looking forward to saving electricity, after a period of time to see how the effect in the follow-up comment!	Jingdong prefecture level city in Shandong
Happy Fan Shop	King of Power Saving	I just got it home and haven't used it for long, so I'll use it for a couple months and see what happens. Seeing that most people have good reviews, it should be fine.	Jingdong prefecture level city in Shandong
Happy Fan Shop	King of Power Saving	I'm looking forward to the results, so I'll give you a good review out of habit! Follow up review after a while	Jingdong prefecture level city in Shandong
Happy Fan Shop	King of Power Saving	Use a dish first to know if it will help save energy	Jingdong prefecture level city in Shandong
Happy Fan Shop	King of Power Saving	Whether it saves electricity or not, we don't know yet.	Jingdong prefecture level city in Shandong
Happy Fan Shop	King of Power Saving	Goods have been received, use a period of time to know how oh. The courier did not deliver to the door ah! Sent to the station ah! The heat is inconvenient ah!	Jingdong prefecture level city in Shandong
Happy Fan Shop	King of Power Saving	A little effect, before an hour a power, now an hour 0.76 degrees, a dollar a power = save 24 cents, although not a lot, first use it!	Jingdong prefecture level city in Shandong
Happy Fan Shop	King of Power Saving	Just received the goods, use a month to evaluate	Jingdong prefecture level city in Shandong

Happy Fan Shop	King of Power Saving	Home every month with a lot of electricity, inadvertently found this good thing online that can save the beginning of a skeptical attitude to consult customer service, customer service replied very professional, said 120 days can be refunded that first try it!	Jingdong prefecture level city in Shandong
Happy Fan Shop	King of Power Saving	After receiving the goods this afternoon, immediately installed in the bathroom Smith energy storage electric water heater on the power outlet (instead of connecting with a plug board), when used, found that the "Power Saving King" LCD display, is the value of the voltage, not the store's video said so	Jingdong prefecture level city in Shandong
Happy Fan Shop	King of Power Saving	Has made many days, did not feel the power saving, the effect is not obvious.	Jingdong prefecture level city in Shandong
Happy Fan Shop	King of Power Saving	Energy savers do not save energy at all, but rather consume a lot of electricity.	Jingdong prefecture level city in Shandong
Happy Fan Shop	King of Power Saving	It's useless. It's a waste of money.	Jingdong prefecture level city in Shandong
Happy Fan Shop	King of Power Saving	Last month saved more than 500, which came back to buy, the store bought three, and then buy 1 home use, my family is dry beauty salon, more instruments, especially electricity, a month almost 1000. or so, with this did not expect to save so much, great, five-star praise!	Jingdong prefecture level city in Shandong
Happy Fan Shop	King of Power Saving	It rained a couple days ago and I just went to get the item back today , not bad, hey I looked at the meter and that red display light takes several seconds to jump, did not make a mistake buying it. After a period of time to evaluate.	Jingdong prefecture level city in Shandong
Happy Fan Shop	King of Power Saving	Without further ado, the electricity bill came down, this month less 102 yuan, has been repurchased, I also recommended to my friends, all said that there are effects, the product is okay, the effect is quite	Jingdong prefecture level city in Shandong
Happy Fan Shop	King of Power Saving	After buying and using two months really see the effect, so only to evaluate! Originally held the mindset of trying, I did not expect really effective, worth having!	Jingdong prefecture level city in Shandong
Happy Fan Shop	King of Power Saving	In the use of this energy saver before the monthly electricity bill is more than 150 yuan or so, now save thirty percent, every day to work, six or seven o'clock in the afternoon to go home, I do not know why the electricity bill is so expensive, energy-saving appliances really have the effect of recommending	Jingdong prefecture level city in Shandong
Happy Fan Shop	King of Power Saving	It saves about 100 bucks a month. A friend recommended it to me, and he's been using it for a while, and the effect is so obvious that I bought one too.	Jingdong prefecture level city in Shandong
Happy Fan Shop	King of Power Saving	My dad asked me to buy one, but after I paid the bill, I asked and he said it was a lot less this month, so he asked me to buy another one.	Jingdong prefecture level city in Shandong
Happy Fan Shop	King of Power Saving	The specific effect will have to be tested for at least two months to compare whether there is a power saving effect or not.	Jingdong prefecture level city in Shandong
Happy Fan Shop	King of Power Saving	'The goods have been received, immediately go to use, please ask the boss you instructions do not allow to withdraw, there are two fuses installed there!	Jingdong prefecture level city in Shandong
Happy Fan Shop	King of Power Saving	First praise is using, after using if good then follow up, now because I have not yet seen the results	Jingdong prefecture level city in Shandong
Happy Fan Shop	King of Power Saving	I've just been using it, and I haven't seen if it has a power-saving effect yet, so I'll follow up after 10 days and half a month.	Jingdong prefecture level city in Shandong
Happy Fan Shop	King of Power Saving	Just received the intelligent power saving meter, immediately plugged in to use, see it is not bad, and order to sell the second time	Jingdong prefecture level city in Shandong
Happy Fan Shop	King of Power Saving	This power saver is a bit effective, used for half a month, the effect is still visible.	Jingdong prefecture level city in Shandong
Happy Fan Shop	King of Power Saving	I received the baby and it looks great, I'm trying it out now and will keep an eye on it afterward.	Jingdong prefecture level city in Shandong
Happy Fan Shop	King of Power Saving	I've just received it, but I'm still trying to see how it works, so I'll come back in a little while.	Jingdong prefecture level city in Shandong
Happy Fan Shop	King of Power Saving	Just used it wait to use it for a while and then comment thanks, all first five stars.	Jingdong prefecture level city in Shandong
Happy Fan Shop	King of Power Saving	Very trustworthy brand, quality is guaranteed, the price is still affordable, quite satisfied!	Jingdong prefecture level city in Shandong
Happy Fan Shop	King of Power Saving	Things received, I hope that the platform of Jingdong do not sell fake goods are available	Jingdong prefecture level city in Shandong
Happy Fan Shop	King of Power Saving	Energy saver received, the use of the effect is good and the business description of the same	Jingdong prefecture level city in Shandong
Happy Fan Shop	King of Power Saving	The surface looks beautiful, the actual effect is not known, after using additional evaluation.	Jingdong prefecture level city in Shandong
Happy Fan Shop	King of Power Saving	Tried it for a few months, it really saves electricity, and I bought it for a friend.	Jingdong prefecture level city in Shandong
Happy Fan Shop	King of Power Saving	It didn't work and wanted to return it, it really didn't work at all.	Jingdong prefecture level city in Shandong
Happy Fan Shop	King of Power Saving	Just used it, don't know how effective it is, looking forward to usefulness	Jingdong prefecture level city in Shandong
Happy Fan Shop	King of Power Saving	After two months before the effect I do not know if it is true!	Jingdong prefecture level city in Shandong
Happy Fan Shop	King of Power Saving	So fast! Ordered yesterday and received today 👍👍👍👍👍👍👍👍👍 🌹🌹🌹🌹🌹🌹🌹🌹🌹	Jingdong prefecture level city in Shandong
Happy Fan Shop	King of Power Saving	I've started using it. We'll see how it works.	Jingdong prefecture level city in Shandong
Happy Fan Shop	King of Power Saving	I just received the goods, I won't know the effect until after two.	Jingdong prefecture level city in Shandong
Happy Fan Shop	King of Power Saving	Just received the goods, the specific effect will be evaluated later	Jingdong prefecture level city in Shandong
Happy Fan Shop	King of Power Saving	I've received the goods, I don't know if it's any good yet	Jingdong prefecture level city in Shandong
Happy Fan Shop	King of Power Saving	Try it for a month and then come back with a concrete evaluation	Jingdong prefecture level city in Shandong
Happy Fan Shop	King of Power Saving	In use, after two months to see the effect of power saving	Jingdong prefecture level city in Shandong
Happy Fan Shop	King of Power Saving	Power Saver. It's authentic. It's black. It works.	Jingdong prefecture level city in Shandong
Happy Fan Shop	King of Power Saving	I've been using it for a week now and it's not working.	Jingdong prefecture level city in Shandong
Happy Fan Shop	King of Power Saving	I'm just using it. I don't know how it's going to work.	Jingdong prefecture level city in Shandong
Happy Fan Shop	King of Power Saving	Products are better looking out for power savings	Jingdong prefecture level city in Shandong
Happy Fan Shop	King of Power Saving	I received it and I've kept it on. I hope it works.	Jingdong prefecture level city in Shandong
Happy Fan Shop	King of Power Saving	It's really good! Worth recommending to buy!	Jingdong prefecture level city in Shandong
Happy Fan Shop	King of Power Saving	Try it for a few days and see if it doesn't work, then return it.	Jingdong prefecture level city in Shandong
Happy Fan Shop	King of Power Saving	It's in use and I hope to be pleasantly surprised.	Jingdong prefecture level city in Shandong
Happy Fan Shop	King of Power Saving	We'll see how it works when we use it	Jingdong prefecture level city in Shandong
Happy Fan Shop	King of Power Saving	Let's see if it works.	Jingdong prefecture level city in Shandong
Happy Fan Shop	King of Power Saving	I'm using it and will follow up with a review	Jingdong prefecture level city in Shandong
Happy Fan Shop	King of Power Saving	It really works. It saves energy.	Jingdong prefecture level city in Shandong
Happy Fan Shop	King of Power Saving	It does have some power savings!	Jingdong prefecture level city in Shandong

Happy Fan Shop	King of Power Saving	Jingdong shopping speed is convenient and timely	Jingdong prefecture level city in Shandong
Happy Fan Shop	King of Power Saving	Second time I've bought it. Everything's fine.	Jingdong prefecture level city in Shandong
Happy Fan Shop	King of Power Saving	I don't know if it works yet.	Jingdong prefecture level city in Shandong
Happy Fan Shop	King of Power Saving	Not used, don't know how well it works.	Jingdong prefecture level city in Shandong
Happy Fan Shop	King of Power Saving	No visible results so far	Jingdong prefecture level city in Shandong
Happy Fan Shop	King of Power Saving	It doesn't feel like it's working.	Jingdong prefecture level city in Shandong
Happy Fan Shop	King of Power Saving	Where's the effect? Where are the power savings?	Jingdong prefecture level city in Shandong
Happy Fan Shop	King of Power Saving	Let's see how it works in practice!	Jingdong prefecture level city in Shandong
Happy Fan Shop	King of Power Saving	I'll wait until I use it to give you a good review	Jingdong prefecture level city in Shandong
Happy Fan Shop	King of Power Saving	I'll follow up after I've used it	Jingdong prefecture level city in Shandong
Happy Fan Shop	King of Power Saving	Well, that's good for saving power.	Jingdong prefecture level city in Shandong
Happy Fan Shop	King of Power Saving	Received. Used in the know.	Jingdong prefecture level city in Shandong
Happy Fan Shop	King of Power Saving	It doesn't do much good.	Jingdong prefecture level city in Shandong
Happy Fan Shop	King of Power Saving	In line with the physical object, fast logistics, good service attitude of the deliveryman! The quality is very good! It will take up to 2 months to see if it can save power! Hope it's as good as advertised!	Jingdong prefecture level city in Shandong
Happy Fan Shop	King of Power Saving	My family three rooms and one hall, more than 100 square meters, two air conditioners! TV, washing machine, water heater, what is very costly electricity, bought even a that a gold color, currently using the feeling is quite good, scanning code to verify that it is genuine, the key is really save	Jingdong prefecture level city in Shandong
Happy Fan Shop	In-situ compensated economizer	A month to save more than 300, the saver is really too good to use, at once bought 3, commercial electricity, electricity high daily power consumption, before a month of more than 600, would have just wanted to try, did not want to use more after a month to save so much, happy!	Jingdong prefecture level city in Shandong
Happy Fan Shop	In-situ compensated economizer	More than 130 less than last month's electricity bill. Installed some time, bought 3 currently still stable power saving in the product no noise no hot phenomenon, rest assured with! Worth buying!	Jingdong prefecture level city in Shandong
Happy Fan Shop	In-situ compensated economizer	Things received, just plugged in last night to try, have not seen the results, business service attitude is very good, logistics is very fast, things look good!	Jingdong prefecture level city in Shandong
Happy Fan Shop	In-situ compensated economizer	Arrived and could not wait to open, really did not let me disappointed, the quality is particularly good, the value is also quite high, satisfied with a shopping	Jingdong prefecture level city in Shandong
Happy Fan Shop	In-situ compensated economizer	This month only paid more than 20 fast money, before all 100 dollars, the effect is really good, this product is so good, recommended to buy!	Jingdong prefecture level city in Shandong
Happy Fan Shop	In-situ compensated economizer	What if it doesn't save power and doesn't work?	Jingdong prefecture level city in Shandong
Happy Fan Shop	In-situ compensated economizer	Didn't feel like saving power for a few days, didn't experience any service issues.	Jingdong prefecture level city in Shandong
Happy Fan Shop	In-situ compensated economizer	phoney	Jingdong prefecture level city in Shandong
Fortune Shop	Green Intelligent Power Saver	First try a period of time teachers are really useful, Jingdong logistics is very fast!	Jingdong prefecture level city in Shandong
Fortune Shop	Green Intelligent Power Saver	Absolutely good stuff, with particularly convenient and simple, directly inserted into the home plug board on it. Now look at the meter does turn much slower than before, I now still open the air conditioning as usual to watch TV, and the original did not change at all, but the power really less.	Jingdong prefecture level city in Shandong
Fortune Shop	Green Intelligent Power Saver	Very satisfied with a shopping, logistics is very fast first night order the next day to the home, the family likes to use this product, will buy again and recommended to friends, should say that all aspects are good must give five-star praise!	Jingdong prefecture level city in Shandong
Fortune Shop	Green Intelligent Power Saver	I received the goods, but I don't know whether it can really save or not, I won't know until I pay the rent next month.	Jingdong prefecture level city in Shandong
Fortune Shop	Green Intelligent Power Saver	Logistics quickly goods received to the business description of the same, I am the third purchase, really save electricity and compact does not take up space	Jingdong prefecture level city in Shandong
Fortune Shop	Green Intelligent Power Saver	High-power super saver received, logistics is very fast packaging is very good, the price is not expensive quality is very good, save electricity and save money, genuine, service attitude is very good, very enthusiastic and thoughtful, there is a need for friends, please rest assured that the order to buy	Jingdong prefecture level city in Shandong
Fortune Shop	Green Intelligent Power Saver	Things received, small and exquisite, immediately plugged in to use, looking forward to the effect!	Jingdong prefecture level city in Shandong
Fortune Shop	Green Intelligent Power Saver	Today received the baby, beautiful appearance, environmental protection and energy saving, benefit the country and the people and their own, value for money, logistics is super fast, the service attitude is very good, very satisfied with a shopping experience.	Jingdong prefecture level city in Shandong
Fortune Shop	Green Intelligent Power Saver	Last night before leaving work to look at the table, and then plugged into the node device, this morning and then look at the table, than the original save a few degrees of electricity, very good to have a power-saving effect, easy to use, satisfied with the shopping praise.	Jingdong prefecture level city in Shandong
Fortune Shop	Green Intelligent Power Saver	The product is real and effective, the electricity bill is reduced, really good, thanks to the merchants have so many good products, the product is real and effective, the electricity bill is reduced, want to save electricity friends quickly buy it, early use early to save money.	Jingdong prefecture level city in Shandong
Fortune Shop	Green Intelligent Power Saver	Saver saves time and money, baby appearance is beautiful, the quality of goods is good, and the same as the business description, good service attitude, packaging is complete, logistics is very fast, praise to	Jingdong prefecture level city in Shandong
Fortune Shop	Green Intelligent Power Saver	The effect is good, summer, home electricity is relatively large, buy and use a few days, feel the effect is very good, originally ten dollars a day electricity bill, now eight dollars a day, quite good	Jingdong prefecture level city in Shandong
Fortune Shop	Green Intelligent Power Saver	Indeed can save electricity and money, the first time I know this or from a friend's home to see, but also do not believe it, and now they use it to know that there is this good thing.	Jingdong prefecture level city in Shandong
Fortune Shop	Green Intelligent Power Saver	I have received the Smart Power Saver. Perfectly packaged. Fast delivery. Boss customer service attitude is also very good. It's worth buying. I plugged it in and tried it and it really saves more power than the original. I'll buy it again next time.	Jingdong prefecture level city in Shandong
Fortune Shop	Green Intelligent Power Saver	It's nice to save a dozen bucks a night on two air conditioners that used to take 30 or 40 degrees a night, but now it's only 10 or so degrees a night	Jingdong prefecture level city in Shandong
Fortune Shop	Green Intelligent Power Saver	Product no manufacturer's name no product qualification certificate, try two days to see the effect of	Jingdong prefecture level city in Shandong
Fortune Shop	Green Intelligent Power Saver	I've been using it for over a month and have saved over $80, so I've repurchased a few more back to save together	Jingdong prefecture level city in Shandong
Fortune Shop	Green Intelligent Power Saver	I haven't used it yet and will comment when I do, but it's well made.	Jingdong prefecture level city in Shandong
Fortune Shop	Green Intelligent Power Saver	It's quite energy efficient. It's really a boon for the home.	Jingdong prefecture level city in Shandong
Fortune Shop	Green Intelligent Power Saver	Energy-saving socket received a month, after using it for a month proved that it is indeed energy-saving, electricity bill saving, will buy it again, thank you!	Jingdong prefecture level city in Shandong
Fortune Shop	Green Intelligent Power Saver	There was a similar product many years ago. Now technology has evolved, buy one to try, according to the instructions in the box, it takes one or two dozen days to show results. Check your electric bill and see what happens.	Jingdong prefecture level city in Shandong
Fortune Shop	Green Intelligent Power Saver	Jingdong logistics is really fast, the same day the order arrived on the same day, more convenient than the supermarket, and a wide range of choices, the	Jingdong prefecture level city in Shandong
Fortune Shop	Green Intelligent Power Saver	I've been using this for a month or so and I can't see any results, it really is a scam as they say.	Jingdong prefecture level city in Shandong

Fortune Shop	Green Intelligent Power Saver	Very good use, a month can save a few degrees of power loss of electricity, introduced to the relatives to buy back to try.	Jingdong prefecture level city in Shandong
Fortune Shop	Green Intelligent Power Saver	It's really powerful! With this air conditioner can be turned on at any time without the pressure of paying the electricity bill! It's good for the people!	Jingdong prefecture level city in Shandong
Fortune Shop	Green Intelligent Power Saver	This business is a big ** products are not qualified, with a month of electricity more than one hundred percent of thirty, the purchase agreed to return the goods but do not receive a refund after receiving the goods, looking for the official side does not help, the Jingdong is a big **, I have never shopped in	Jingdong prefecture level city in Shandong
Fortune Shop	Green Intelligent Power Saver	It's good. I tried it. It saves power.	Jingdong prefecture level city in Shandong
Fortune Shop	Green Intelligent Power Saver	Nothing**, it seems like there's more	Jingdong prefecture level city in Shandong
Fortune Shop	Green Intelligent Power Saver	Power saving device received, well packaged, good quality, fast logistics	Jingdong prefecture level city in Shandong
Fortune Shop	Green Intelligent Power Saver	The service is okay, is that when I bought five, the desirable pieces are three, and then wait a few days and send me two replacement, I do not know if it is a mistake of the shipper, if I was not in the courier there to see, go home to say no, the	Jingdong prefecture level city in Shandong
Fortune Shop	Green Intelligent Power Saver	This thing will only cost me more if I use it, but before I used it, it was less than a ~ electricity a month, and after I used it, it was more than two hundred kWh a month.	Jingdong prefecture level city in Shandong
Fortune Shop	Green Intelligent Power Saver	I just used it. I don't know how it is.	Jingdong prefecture level city in Shandong
Fortune Shop	Green Intelligent Power Saver	I've received the product and I'm using it, so I'll see how it goes in a month!	Jingdong prefecture level city in Shandong
Fortune Shop	Green Intelligent Power Saver	The goods have just received the effect do not know how, please look forward to the results.	Jingdong prefecture level city in Shandong
Fortune Shop	Green Intelligent Power Saver	Power saving device, the material is very good practical and durable, the size is suitable, packaging is very good, the price is cheap, logistics is very fast!	Jingdong prefecture level city in Shandong
Fortune Shop	Green Intelligent Power Saver	Very nice, will comment in detail after using it for a while.	Jingdong prefecture level city in Shandong
Fortune Shop	Green Intelligent Power Saver	Good stuff to trust and support	Jingdong prefecture level city in Shandong
Fortune Shop	Green Intelligent Power Saver	I'll comment after I use it, doubt if it really saves power no	Jingdong prefecture level city in Shandong
Fortune Shop	Green Intelligent Power Saver	It's good quality and works well!	Jingdong prefecture level city in Shandong
Fortune Shop	Green Intelligent Power Saver	hahaha just right hahahahahaha hiccup hahahaha	Jingdong prefecture level city in Shandong
Fortune Shop	Green Intelligent Power Saver	The real thing matches the picture and looks great!	Jingdong prefecture level city in Shandong
Fortune Shop	Green Intelligent Power Saver	Plugged it in and we'll see when we pay the bills at the end of the year!	Jingdong prefecture level city in Shandong
Fortune Shop	Green Intelligent Power Saver	False advertising. It doesn't work.	Jingdong prefecture level city in Shandong
Fortune Shop	Green Intelligent Power Saver	Goods received, good effect	Jingdong prefecture level city in Shandong
Fortune Shop	Green Intelligent Power Saver	It looks okay, we'll see if it saves electricity when the time comes	Jingdong prefecture level city in Shandong
Fortune Shop	Green Intelligent Power Saver	Logistics is very fast quality is very good, the price is very affordable, accessories are also complete, installed, satisfied with good use	Jingdong prefecture level city in Shandong
Fortune Shop	Green Intelligent Power Saver	I've received the power saver. I'm looking forward to the savings.	Jingdong prefecture level city in Shandong
Fortune Shop	Green Intelligent Power Saver	Received, have not yet used, after use and then evaluate	Jingdong prefecture level city in Shandong
Fortune Shop	Green Intelligent Power Saver	I received the item, but I don't know if it saves power or not.	Jingdong prefecture level city in Shandong
Fortune Shop	Green Intelligent Power Saver	On Trial	Jingdong prefecture level city in Shandong
Fortune Shop	Green Intelligent Power Saver	Use it and see how it goes.	Jingdong prefecture level city in Shandong
Fortune Shop	Green Intelligent Power Saver	Quality is very good, no odor, fine workmanship, grain color are very like, satisfied!	Jingdong prefecture level city in Shandong
Fortune Shop	Green Intelligent Power Saver	I'll give you a good review. I don't know if it'll work.	Jingdong prefecture level city in Shandong
Fortune Shop	Green Intelligent Power Saver	The logistics is very fast and the package is intact. Good use of affordable. The effect is very good, is genuine	Jingdong prefecture level city in Shandong
Fortune Shop	Green Intelligent Power Saver	Nothing happens when you plug it in.	Jingdong prefecture level city in Shandong
Fortune Shop	Green Intelligent Power Saver	I don't know if it's as good as it sounds.	Jingdong prefecture level city in Shandong
Fortune Shop	Green Intelligent Power Saver	The quality of the stuff is very good, exactly the same as the seller's description, very satisfied!	Jingdong prefecture level city in Shandong
Fortune Shop	Green Intelligent Power Saver	The saver works great, it really works, it's affordable and worth buying.	Jingdong prefecture level city in Shandong
Fortune Shop	Green Intelligent Power Saver	Just used it for a couple days. I don't know yet.	Jingdong prefecture level city in Shandong
Fortune Shop	Green Intelligent Power Saver	The quality is quite good, cheaper than the physical store, good, next time will come again!	Jingdong prefecture level city in Shandong
Fortune Shop	Green Intelligent Power Saver	Good quality, cheap and affordable worth recommending to buy!	Jingdong prefecture level city in Shandong
Fortune Shop	Green Intelligent Power Saver	Very good quality, well made, works well! Satisfied	Jingdong prefecture level city in Shandong
Fortune Shop	Green Intelligent Power Saver	The logistics is very fast quality is very good very pleasant a shopping	Jingdong prefecture level city in Shandong
Fortune Shop	Green Intelligent Power Saver	Use it and then come back to evaluate!	Jingdong prefecture level city in Shandong
Fortune Shop	Green Intelligent Power Saver	# It's no use lying at all #	Jingdong prefecture level city in Shandong
Fortune Shop	Green Intelligent Power Saver	I can't get a voltage drop.	Jingdong prefecture level city in Shandong
Fortune Shop	Green Intelligent Power Saver	Evaluate after use	Jingdong prefecture level city in Shandong
Fortune Shop	Green Intelligent Power Saver	Still on trial.	Jingdong prefecture level city in Shandong
Fortune Shop	Green Intelligent Power Saver	ineffective	Jingdong prefecture level city in Shandong
Fortune Shop	Green Intelligent Power Saver	Satisfaction Shopping	Jingdong prefecture level city in Shandong
Fortune Shop	Green Intelligent Power Saver	Lies.	Jingdong prefecture level city in Shandong
Fortune Shop	Green Intelligent Power Saver	It's a real power saver.	Jingdong prefecture level city in Shandong
Fortune Shop	Green Intelligent Power Saver	in use	Jingdong prefecture level city in Shandong

Fortune Shop	Green Intelligent Power Saver	I don't know yet.	Jingdong prefecture level city in Shandong
Fortune Shop	Green Intelligent Power Saver	not care about anything	Jingdong prefecture level city in Shandong
Fortune Shop	Green Intelligent Power Saver	It doesn't seem to have much effect	Jingdong prefecture level city in Shandong
Fortune Shop	Green Intelligent Power Saver	Let the merchant see for himself, does the plug positive and negative terminals look like this?	Jingdong prefecture level city in Shandong
Fortune Shop	Green Intelligent Power Saver	It doesn't work well, the electricity bill is less before using it, but more after using it, contacting customer service, the service is still not good, and returning it is slow	Jingdong prefecture level city in Shandong
Fortune Shop	Green Intelligent Power Saver	Doesn't save any power at all. Not much use.	Jingdong prefecture level city in Shandong
Fortune Shop	Green Intelligent Power Saver	With the advertisement product does not match, above the effective negative, power saving rate of two rows of words it, the product looks very thin, with 3 days to see the meter number is still with the previous no difference, which is obviously fraudulent behavior!	Jingdong prefecture level city in Shandong
Fortune Shop	Green Intelligent Power Saver	The plugs are all loose, they're all the same length and they don't work at all	Jingdong prefecture level city in Shandong
Fortune Shop	Green Intelligent Power Saver	It's bad 👎 it's not effective at all, you'd be crazy to sell this garbage on the internet.	Jingdong prefecture level city in Shandong
Fortune Shop	Green Intelligent Power Saver	It doesn't save any power at all, so I hope the old timers don't fall for this scam again	Jingdong prefecture level city in Shandong
Fortune Shop	Green Intelligent Power Saver	I've been using it for over a month now and haven't noticed any power savings.	Jingdong prefecture level city in Shandong
Fortune Shop	Green Intelligent Power Saver	It doesn't work at all. The electricity bill is higher when you use it.	Jingdong prefecture level city in Shandong
Fortune Shop	Green Intelligent Power Saver	It doesn't save any power, it's still as bad as ever.	Jingdong prefecture level city in Shandong
Fortune Shop	Green Intelligent Power Saver	Last night before leaving work to look at the table, and then plugged into the node device, this morning and then look at the table, than the original save a few degrees of electricity, very good to have a power-saving effect, easy to use, satisfied with the shopping praise.	Jingdong prefecture level city in Shandong
Fortune Shop	Green Intelligent Power Saver	The product is real and effective, the electricity bill is reduced, really good, thanks to the merchants have so many good products, the product is real and effective, the electricity bill is reduced, want to save electricity friends quickly buy it, early use early to save money.	Jingdong prefecture level city in Shandong
Fortune Shop	Green Intelligent Power Saver	Saver saves time and money, baby appearance is beautiful, the quality of goods is good, and the same as the business description, good service attitude, packaging is complete, logistics is very fast, praise to	Jingdong prefecture level city in Shandong
Fortune Shop	Green Intelligent Power Saver	The effect is good, summer, home electricity is relatively large, buy and use a few days, feel the effect is very good, originally ten dollars a day electricity bill, now eight dollars a day, quite good	Jingdong prefecture level city in Shandong
Fortune Shop	Green Intelligent Power Saver	Indeed can save electricity and money, the first time I know this or from a friend's home to see, but also do not believe it, and now they use it to know that there is this good thing.	Jingdong prefecture level city in Shandong
Fortune Shop	Green Intelligent Power Saver	I have received the Smart Power Saver. Perfectly packaged. Fast delivery. Boss customer service attitude is also very good. It's worth buying. I plugged it in and tried it and it really saves more power than the original. I'll buy it again next time.	Jingdong prefecture level city in Shandong
Fortune Shop	Green Intelligent Power Saver	It's nice to save a dozen bucks a night on two air conditioners that used to take 30 or 40 degrees a night, but now it's only 10 or so degrees a night	Jingdong prefecture level city in Shandong
Fortune Shop	Green Intelligent Power Saver	Product no manufacturer's name no product qualification certificate, try two days to see the effect of	Jingdong prefecture level city in Shandong
Fortune Shop	Green Intelligent Power Saver	I've been using it for over a month and have saved over $80, so I've repurchased a few more back to save together	Jingdong prefecture level city in Shandong
Fortune Shop	Green Intelligent Power Saver	I haven't used it yet and will comment when I do, but it's well made.	Jingdong prefecture level city in Shandong
Fortune Shop	Green Intelligent Power Saver	It's quite energy efficient. It's really a boon for the home.	Jingdong prefecture level city in Shandong
Fortune Shop	Green Intelligent Power Saver	Energy-saving socket received a month, after using it for a month proved that it is indeed energy-saving, electricity bill saving, will buy it again, thank you!	Jingdong prefecture level city in Shandong
Fortune Shop	Green Intelligent Power Saver	There was a similar product many years ago. Now technology has evolved, buy one to try, according to the instructions in the box, it takes one or two dozen days to show results. Check your electric bill and see what happens.	Jingdong prefecture level city in Shandong
Fortune Shop	Green Intelligent Power Saver	Jingdong logistics is really fast, the same day the order arrived on the same day, more convenient than the supermarket, and a wide range of choices, the	Jingdong prefecture level city in Shandong
Fortune Shop	Green Intelligent Power Saver	I've been using this for a month or so and I can't see any results, it really is a scam as they say.	Jingdong prefecture level city in Shandong
Fortune Shop	Green Intelligent Power Saver	Very good use, a month can save a few degrees of power loss of electricity, introduced to the relatives to buy back to try.	Jingdong prefecture level city in Shandong
Fortune Shop	Green Intelligent Power Saver	It's really powerful! With this air conditioner can be turned on at any time without the pressure of paying the electricity bill! It's good for the people!	Jingdong prefecture level city in Shandong
Fortune Shop	Green Intelligent Power Saver	This business is a big ** products are not qualified, with a month of electricity more than one hundred percent of thirty, the purchase agreed to return the goods but do not receive a refund after receiving the goods, looking for the official side does not help, the Jingdong is a big **, I have never shopped in	Jingdong prefecture level city in Shandong
Fortune Shop	Green Intelligent Power Saver	It's good. I tried it. It saves power.	Jingdong prefecture level city in Shandong
Fortune Shop	Green Intelligent Power Saver	Nothing**, it seems like there's more	Jingdong prefecture level city in Shandong
Fortune Shop	Green Intelligent Power Saver	Power saving device received, well packaged, good quality, fast logistics	Jingdong prefecture level city in Shandong
Fortune Shop	Green Intelligent Power Saver	The service is okay, is that when I bought five, the desirable pieces are three, and then wait a few days and send me two replacement, I do not know if it is a mistake of the shipper, if I was not in the courier there to see, go home to say no, the	Jingdong prefecture level city in Shandong
Fortune Shop	Green Intelligent Power Saver	This thing will only cost me more if I use it, but before I used it, it was less than a ~ electricity a month, and after I used it, it was more than two hundred kWh a month.	Jingdong prefecture level city in Shandong
Fortune Shop	Green Intelligent Power Saver	I just used it. I don't know how it is.	Jingdong prefecture level city in Shandong
Fortune Shop	Green Intelligent Power Saver	I've received the product and I'm using it, so I'll see how it goes in a month!	Jingdong prefecture level city in Shandong
Fortune Shop	Green Intelligent Power Saver	The goods have just received the effect do not know how, please look forward to the results.	Jingdong prefecture level city in Shandong
Fortune Shop	Green Intelligent Power Saver	Power saving device, the material is very good practical and durable, the size is suitable, packaging is very good, the price is cheap, logistics is very fast!	Jingdong prefecture level city in Shandong
Fortune Shop	Green Intelligent Power Saver	Very nice, will comment in detail after using it for a while.	Jingdong prefecture level city in Shandong
Fortune Shop	Green Intelligent Power Saver	Good stuff to trust and support	Jingdong prefecture level city in Shandong
Fortune Shop	Green Intelligent Power Saver	I'll comment after I use it, doubt if it really saves power no	Jingdong prefecture level city in Shandong
Fortune Shop	Green Intelligent Power Saver	It's good quality and works well!	Jingdong prefecture level city in Shandong
Fortune Shop	Green Intelligent Power Saver	hahaha just right hahahahahaha hiccup hahahaha	Jingdong prefecture level city in Shandong
Fortune Shop	Green Intelligent Power Saver	The real thing matches the picture and looks great!	Jingdong prefecture level city in Shandong
Fortune Shop	Green Intelligent Power Saver	Plugged it in and we'll see when we pay the bills at the end of the year!	Jingdong prefecture level city in Shandong
Fortune Shop	Green Intelligent Power Saver	False advertising. It doesn't work.	Jingdong prefecture level city in Shandong

Fortune Shop	Green Intelligent Power Saver	Goods received, good effect	Jingdong prefecture level city in Shandong
Fortune Shop	Green Intelligent Power Saver	It looks okay, we'll see if it saves electricity when the time comes	Jingdong prefecture level city in Shandong
Fortune Shop	Green Intelligent Power Saver	Logistics is very fast quality is very good, the price is very affordable, accessories are also complete, installed, satisfied with good use	Jingdong prefecture level city in Shandong
Fortune Shop	Green Intelligent Power Saver	I've received the power saver. I'm looking forward to the savings.	Jingdong prefecture level city in Shandong
Fortune Shop	Green Intelligent Power Saver	Received, have not yet used, after use and then evaluate	Jingdong prefecture level city in Shandong
Fortune Shop	Green Intelligent Power Saver	I received the item, but I don't know if it saves power or not.	Jingdong prefecture level city in Shandong
Fortune Shop	Green Intelligent Power Saver	On Trial	Jingdong prefecture level city in Shandong
Fortune Shop	Green Intelligent Power Saver	Use it and see how it goes.	Jingdong prefecture level city in Shandong
Fortune Shop	Green Intelligent Power Saver	Quality is very good, no odor, fine workmanship, grain color are very like, satisfied!	Jingdong prefecture level city in Shandong
Fortune Shop	Green Intelligent Power Saver	I'll give you a good review. I don't know if it'll work.	Jingdong prefecture level city in Shandong
Fortune Shop	Green Intelligent Power Saver	The logistics is very fast and the package is intact. Good use of affordable. The effect is very good, is genuine	Jingdong prefecture level city in Shandong
Fortune Shop	Green Intelligent Power Saver	Nothing happens when you plug it in.	Jingdong prefecture level city in Shandong
Fortune Shop	Green Intelligent Power Saver	I don't know if it's as good as it sounds.	Jingdong prefecture level city in Shandong
Fortune Shop	Green Intelligent Power Saver	The quality of the stuff is very good, exactly the same as the seller's description, very satisfied!	Jingdong prefecture level city in Shandong
Fortune Shop	Green Intelligent Power Saver	The saver works great, it really works, it's affordable and worth buying.	Jingdong prefecture level city in Shandong
Fortune Shop	Green Intelligent Power Saver	Just used it for a couple days. I don't know yet.	Jingdong prefecture level city in Shandong
Fortune Shop	Green Intelligent Power Saver	The quality is quite good, cheaper than the physical store, good, next time will come again!	Jingdong prefecture level city in Shandong
Fortune Shop	Green Intelligent Power Saver	Good quality, cheap and affordable worth recommending to buy!	Jingdong prefecture level city in Shandong
Fortune Shop	Green Intelligent Power Saver	Very good quality, well made, works well! Satisfied	Jingdong prefecture level city in Shandong
Fortune Shop	Green Intelligent Power Saver	The logistics is very fast quality is very good very pleasant a shopping	Jingdong prefecture level city in Shandong
Fortune Shop	Green Intelligent Power Saver	Use it and then come back to evaluate!	Jingdong prefecture level city in Shandong
Fortune Shop	Green Intelligent Power Saver	# It's no use lying at all #	Jingdong prefecture level city in Shandong
Fortune Shop	Green Intelligent Power Saver	I can't get a voltage drop.	Jingdong prefecture level city in Shandong
Fortune Shop	Green Intelligent Power Saver	Evaluate after use	Jingdong prefecture level city in Shandong
Fortune Shop	Green Intelligent Power Saver	Still on trial.	Jingdong prefecture level city in Shandong
Fortune Shop	Green Intelligent Power Saver	ineffective	Jingdong prefecture level city in Shandong
Fortune Shop	Green Intelligent Power Saver	Satisfaction Shopping	Jingdong prefecture level city in Shandong
Fortune Shop	Green Intelligent Power Saver	Lies.	Jingdong prefecture level city in Shandong
Fortune Shop	Green Intelligent Power Saver	in use	Jingdong prefecture level city in Shandong
Fortune Shop	Green Intelligent Power Saver	It's a real power saver.	Jingdong prefecture level city in Shandong
Fortune Shop	Green Intelligent Power Saver	I don't know yet.	Jingdong prefecture level city in Shandong
Fortune Shop	Green Intelligent Power Saver	not care about anything	Jingdong prefecture level city in Shandong
Fortune Shop	Green Intelligent Power Saver	It doesn't seem to have much effect	Jingdong prefecture level city in Shandong
Fortune Shop	Green Intelligent Power Saver	Let the merchant see for himself, does the plug positive and negative terminals look like this?	Jingdong prefecture level city in Shandong
Fortune Shop	Green Intelligent Power Saver	It doesn't work well, the electricity bill is less before using it, but more after using it, contacting customer service, the service is still not good, and returning it is slow	Jingdong prefecture level city in Shandong
Fortune Shop	Green Intelligent Power Saver	With the advertisement product does not match, above the effective negative, power saving rate of two rows of words it, the product looks very thin, with 3 days to see the meter number is still with the previous no difference, which is obviously fraudulent behavior!	Jingdong prefecture level city in Shandong
Fortune Shop	Green Intelligent Power Saver	Doesn't save any power at all. Not much use.	Jingdong prefecture level city in Shandong
Fortune Shop	Green Intelligent Power Saver	The plugs are all loose, they're all the same length and they don't work at all	Jingdong prefecture level city in Shandong
Fortune Shop	Green Intelligent Power Saver	It's bad 👎 it's not effective at all, you'd be crazy to sell this garbage on the internet.	Jingdong prefecture level city in Shandong
Fortune Shop	Green Intelligent Power Saver	It doesn't save any power at all, so I hope the old timers don't fall for this scam again	Jingdong prefecture level city in Shandong
Fortune Shop	Green Intelligent Power Saver	I've been using it for over a month now and haven't noticed any power savings.	Jingdong prefecture level city in Shandong
Fortune Shop	Green Intelligent Power Saver	It doesn't work at all. The electricity bill is higher when you use it.	Jingdong prefecture level city in Shandong
Fortune Shop	Green Intelligent Power Saver	It doesn't save any power, it's still as bad as ever.	Jingdong prefecture level city in Shandong
Fortune Shop	Green Intelligent Power Saver	Last night before leaving work to look at the table, and then plugged into the node device, this morning and then look at the table, than the original save a few degrees of electricity, very good to have a power-saving effect, easy to use, satisfied with the shopping praise.	Jingdong prefecture level city in Shandong
Fortune Shop	Green Intelligent Power Saver	The product is real and effective, the electricity bill is reduced, really good, thanks to the merchants have so many good products, the product is real and effective, the electricity bill is reduced, want to save electricity friends quickly buy it, early use early to save money.	Jingdong prefecture level city in Shandong
Fortune Shop	Green Intelligent Power Saver	Saver saves time and money, baby appearance is beautiful, the quality of goods is good, and the same as the business description, good service attitude, packaging is complete, logistics is very fast, praise to	Jingdong prefecture level city in Shandong
Fortune Shop	Green Intelligent Power Saver	The effect is good, summer, home electricity is relatively large, buy and use a few days, feel the effect is very good, originally ten dollars a day electricity bill, now eight dollars a day, quite good	Jingdong prefecture level city in Shandong
Fortune Shop	Green Intelligent Power Saver	Indeed can save electricity and money, the first time I know this or from a friend's home to see, but also do not believe it, and now they use it to know that there is this good thing.	Jingdong prefecture level city in Shandong
Fortune Shop	Green Intelligent Power Saver	I have received the Smart Power Saver. Perfectly packaged. Fast delivery. Boss customer service attitude is also very good. It's worth buying. I plugged it in and tried it and it really saves more power than the original. I'll buy it again next time.	Jingdong prefecture level city in Shandong

Fortune Shop	Green Intelligent Power Saver	It's nice to save a dozen bucks a night on two air conditioners that used to take 30 or 40 degrees a night, but now it's only 10 or so degrees a night	Jingdong prefecture level city in Shandong
Fortune Shop	Green Intelligent Power Saver	Product no manufacturer's name no product qualification certificate, try two days to see the effect of	Jingdong prefecture level city in Shandong
Fortune Shop	Green Intelligent Power Saver	I've been using it for over a month and have saved over $80, so I've repurchased a few more back to save together	Jingdong prefecture level city in Shandong
Fortune Shop	Green Intelligent Power Saver	It's quite energy efficient. It's really a boon for the home.	Jingdong prefecture level city in Shandong
Fortune Shop	Green Intelligent Power Saver	I haven't used it yet and will comment when I do, but it's well made.	Jingdong prefecture level city in Shandong
Fortune Shop	Green Intelligent Power Saver	Energy-saving socket received a month, after using it for a month proved that it is indeed energy-saving, electricity bill saving, will buy it again, thank you!	Jingdong prefecture level city in Shandong
Fortune Shop	Green Intelligent Power Saver	There was a similar product many years ago. Now technology has evolved, buy one to try, according to the instructions in the box, it takes one or two dozen days to show results. Check your electric bill and see what happens.	Jingdong prefecture level city in Shandong
Fortune Shop	Green Intelligent Power Saver	Jingdong logistics is really fast, the same day the order arrived on the same day, more convenient than the supermarket, and a wide range of choices, the	Jingdong prefecture level city in Shandong
Fortune Shop	Green Intelligent Power Saver	I've been using this for a month or so and I can't see any results, it really is a scam as they say.	Jingdong prefecture level city in Shandong
Fortune Shop	Green Intelligent Power Saver	Very good use, a month can save a few degrees of power loss of electricity, introduced to the relatives to buy back to try.	Jingdong prefecture level city in Shandong
Fortune Shop	Green Intelligent Power Saver	It's really powerful! With this air conditioner can be turned on at any time without the pressure of paying the electricity bill! It's good for the people!	Jingdong prefecture level city in Shandong
Fortune Shop	Green Intelligent Power Saver	This business is a big ** products are not qualified, with a month of electricity more than one hundred percent of thirty, the purchase agreed to return the goods but do not receive a refund after receiving the goods, looking for the official side does not help, the Jingdong is a big **, I have never shopped in	Jingdong prefecture level city in Shandong
Fortune Shop	Green Intelligent Power Saver	It's good. I tried it. It saves power.	Jingdong prefecture level city in Shandong
Fortune Shop	Green Intelligent Power Saver	Nothing**, it seems like there's more	Jingdong prefecture level city in Shandong
Fortune Shop	Green Intelligent Power Saver	Power saving device received, well packaged, good quality, fast logistics	Jingdong prefecture level city in Shandong
Fortune Shop	Green Intelligent Power Saver	The service is okay, is that when I bought five, the desirable pieces are three, and then wait a few days and send me two replacement, I do not know if it is a mistake of the shipper, if I was not in the courier there to see, go home to say no, the	Jingdong prefecture level city in Shandong
Fortune Shop	Green Intelligent Power Saver	This thing will only cost me more if I use it, but before I used it, it was less than a ~ electricity a month, and after I used it, it was more than two hundred kWh a month.	Jingdong prefecture level city in Shandong
Fortune Shop	Green Intelligent Power Saver	I just used it. I don't know how it is.	Jingdong prefecture level city in Shandong
Fortune Shop	Green Intelligent Power Saver	I've received the product and I'm using it, so I'll see how it goes in a month!	Jingdong prefecture level city in Shandong
Fortune Shop	Green Intelligent Power Saver	The goods have just received the effect do not know how, please look forward to the results.	Jingdong prefecture level city in Shandong
Fortune Shop	Green Intelligent Power Saver	Power saving device, the material is very good practical and durable, the size is suitable, packaging is very good, the price is cheap, logistics is very fast!	Jingdong prefecture level city in Shandong
Fortune Shop	Green Intelligent Power Saver	Very nice, will comment in detail after using it for a while.	Jingdong prefecture level city in Shandong
Fortune Shop	Green Intelligent Power Saver	Good stuff to trust and support	Jingdong prefecture level city in Shandong
Fortune Shop	Green Intelligent Power Saver	I'll comment after I use it, doubt if it really saves power no	Jingdong prefecture level city in Shandong
Fortune Shop	Green Intelligent Power Saver	It's good quality and works well!	Jingdong prefecture level city in Shandong
Fortune Shop	Green Intelligent Power Saver	hahaha just right hahahahahaha hiccup hahahaha	Jingdong prefecture level city in Shandong
Fortune Shop	Green Intelligent Power Saver	The real thing matches the picture and looks great!	Jingdong prefecture level city in Shandong
Fortune Shop	Green Intelligent Power Saver	Plugged it in and we'll see when we pay the bills at the end of the year!	Jingdong prefecture level city in Shandong
Fortune Shop	Green Intelligent Power Saver	False advertising. It doesn't work.	Jingdong prefecture level city in Shandong
Fortune Shop	Green Intelligent Power Saver	Goods received, good effect	Jingdong prefecture level city in Shandong
Fortune Shop	Green Intelligent Power Saver	It looks okay, we'll see if it saves electricity when the time comes	Jingdong prefecture level city in Shandong
Fortune Shop	Green Intelligent Power Saver	Logistics is very fast quality is very good, the price is very affordable, accessories are also complete, installed, satisfied with good use	Jingdong prefecture level city in Shandong
Fortune Shop	Green Intelligent Power Saver	I've received the power saver. I'm looking forward to the savings.	Jingdong prefecture level city in Shandong
Fortune Shop	Green Intelligent Power Saver	Received, have not yet used, after use and then evaluate	Jingdong prefecture level city in Shandong
Fortune Shop	Green Intelligent Power Saver	I received the item, but I don't know if it saves power or not.	Jingdong prefecture level city in Shandong
Fortune Shop	Green Intelligent Power Saver	On Trial	Jingdong prefecture level city in Shandong
Fortune Shop	Green Intelligent Power Saver	Use it and see how it goes.	Jingdong prefecture level city in Shandong
Fortune Shop	Green Intelligent Power Saver	Quality is very good, no odor, fine workmanship, grain color are very like, satisfied!	Jingdong prefecture level city in Shandong
Fortune Shop	Green Intelligent Power Saver	I'll give you a good review. I don't know if it'll work.	Jingdong prefecture level city in Shandong
Fortune Shop	Green Intelligent Power Saver	The logistics is very fast and the package is intact. Good use of affordable. The effect is very good, is genuine	Jingdong prefecture level city in Shandong
Fortune Shop	Green Intelligent Power Saver	Nothing happens when you plug it in.	Jingdong prefecture level city in Shandong
Fortune Shop	Green Intelligent Power Saver	I don't know if it's as good as it sounds.	Jingdong prefecture level city in Shandong
Fortune Shop	Green Intelligent Power Saver	The quality of the stuff is very good, exactly the same as the seller's description, very satisfied!	Jingdong prefecture level city in Shandong
Fortune Shop	Green Intelligent Power Saver	The saver works great, it really works, it's affordable and worth buying.	Jingdong prefecture level city in Shandong
Fortune Shop	Green Intelligent Power Saver	Just used it for a couple days. I don't know yet.	Jingdong prefecture level city in Shandong
Fortune Shop	Green Intelligent Power Saver	The quality is quite good, cheaper than the physical store, good, next time will come again!	Jingdong prefecture level city in Shandong
Fortune Shop	Green Intelligent Power Saver	Good quality, cheap and affordable worth recommending to buy!	Jingdong prefecture level city in Shandong
Fortune Shop	Green Intelligent Power Saver	Very good quality, well made, works well! Satisfied	Jingdong prefecture level city in Shandong
Fortune Shop	Green Intelligent Power Saver	The logistics is very fast quality is very good very pleasant a shopping	Jingdong prefecture level city in Shandong
Fortune Shop	Green Intelligent Power Saver	Use it and then come back to evaluate!	Jingdong prefecture level city in Shandong

Fortune Shop	Green Intelligent Power Saver	# It's no use lying at all #	Jingdong prefecture level city in Shandong
Fortune Shop	Green Intelligent Power Saver	I can't get a voltage drop.	Jingdong prefecture level city in Shandong
Fortune Shop	Green Intelligent Power Saver	Evaluate after use	Jingdong prefecture level city in Shandong
Fortune Shop	Green Intelligent Power Saver	Still on trial.	Jingdong prefecture level city in Shandong
Fortune Shop	Green Intelligent Power Saver	ineffective	Jingdong prefecture level city in Shandong
Fortune Shop	Green Intelligent Power Saver	Satisfaction Shopping	Jingdong prefecture level city in Shandong
Fortune Shop	Green Intelligent Power Saver	Lies.	Jingdong prefecture level city in Shandong
Fortune Shop	Green Intelligent Power Saver	It's a real power saver.	Jingdong prefecture level city in Shandong
Fortune Shop	Green Intelligent Power Saver	in use	Jingdong prefecture level city in Shandong
Fortune Shop	Green Intelligent Power Saver	I don't know yet.	Jingdong prefecture level city in Shandong
Fortune Shop	Green Intelligent Power Saver	not care about anything	Jingdong prefecture level city in Shandong
Fortune Shop	Green Intelligent Power Saver	It doesn't seem to have much effect	Jingdong prefecture level city in Shandong
Fortune Shop	Green Intelligent Power Saver	Let the merchant see for himself, does the plug positive and negative terminals look like this?	Jingdong prefecture level city in Shandong
Fortune Shop	Green Intelligent Power Saver	It doesn't work well, the electricity bill is less before using it, but more after using it, contacting customer service, the service is still not good, and returning it is slow	Jingdong prefecture level city in Shandong
Fortune Shop	Green Intelligent Power Saver	With the advertisement product does not match, above the effective negative, power saving rate of two rows of words it, the product looks very thin, with 3 days to see the meter number is still with the previous no difference, which is obviously fraudulent behavior!	Jingdong prefecture level city in Shandong
Fortune Shop	Green Intelligent Power Saver	Doesn't save any power at all. Not much use.	Jingdong prefecture level city in Shandong
Fortune Shop	Green Intelligent Power Saver	The plugs are all loose, they're all the same length and they don't work at all	Jingdong prefecture level city in Shandong
Fortune Shop	Green Intelligent Power Saver	It's bad 👎 it's not effective at all, you'd be crazy to sell this garbage on the internet.	Jingdong prefecture level city in Shandong
Fortune Shop	Green Intelligent Power Saver	It doesn't save any power at all, so I hope the old timers don't fall for this scam again	Jingdong prefecture level city in Shandong
Fortune Shop	Green Intelligent Power Saver	I've been using it for over a month now and haven't noticed any power savings.	Jingdong prefecture level city in Shandong
Fortune Shop	Green Intelligent Power Saver	It doesn't work at all. The electricity bill is higher when you use it.	Jingdong prefecture level city in Shandong
Fortune Shop	Green Intelligent Power Saver	It doesn't save any power, it's still as bad as ever.	Jingdong prefecture level city in Shandong
Fortune Shop	Green Intelligent Power Saver	Last night before leaving work to look at the table, and then plugged into the node device, this morning and then look at the table, than the original save a few degrees of electricity, very good to have a power-saving effect, easy to use, satisfied with the shopping praise.	Jingdong prefecture level city in Shandong
Fortune Shop	Green Intelligent Power Saver	The product is real and effective, the electricity bill is reduced, really good, thanks to the merchants have so many good products, the product is real and effective, the electricity bill is reduced, want to save electricity friends quickly buy it, early use early to save money.	Jingdong prefecture level city in Shandong
Fortune Shop	Green Intelligent Power Saver	Saver saves time and money, baby appearance is beautiful, the quality of goods is good, and the same as the business description, good service attitude, packaging is complete, logistics is very fast, praise to	Jingdong prefecture level city in Shandong
Fortune Shop	Green Intelligent Power Saver	The effect is good, summer, home electricity is relatively large, buy and use a few days, feel the effect is very good, originally ten dollars a day electricity bill, now eight dollars a day, quite good	Jingdong prefecture level city in Shandong
Fortune Shop	Green Intelligent Power Saver	Indeed can save electricity and money, the first time I know this or from a friend's home to see, but also do not believe it, and now they use it to know that there is this good thing.	Jingdong prefecture level city in Shandong
Fortune Shop	Green Intelligent Power Saver	I have received the Smart Power Saver. Perfectly packaged. Fast delivery. Boss customer service attitude is also very good. It's worth buying. I plugged it in and tried it and it really saves more power than the original. I'll buy it again next time.	Jingdong prefecture level city in Shandong
Fortune Shop	Green Intelligent Power Saver	Product no manufacturer's name no product qualification certificate, try two days to see the effect of	Jingdong prefecture level city in Shandong
Fortune Shop	Green Intelligent Power Saver	It's nice to save a dozen bucks a night on two air conditioners that used to take 30 or 40 degrees a night, but now it's only 10 or so degrees a night	Jingdong prefecture level city in Shandong
Fortune Shop	Green Intelligent Power Saver	I've been using it for over a month and have saved over $80, so I've repurchased a few more back to save together	Jingdong prefecture level city in Shandong
Fortune Shop	Green Intelligent Power Saver	I haven't used it yet and will comment when I do, but it's well made.	Jingdong prefecture level city in Shandong
Fortune Shop	Green Intelligent Power Saver	It's quite energy efficient. It's really a boon for the home.	Jingdong prefecture level city in Shandong
Fortune Shop	Green Intelligent Power Saver	Energy-saving socket received a month, after using it for a month proved that it is indeed energy-saving, electricity bill saving, will buy it again, thank you!	Jingdong prefecture level city in Shandong
Fortune Shop	Green Intelligent Power Saver	Jingdong logistics is really fast, the same day the order arrived on the same day, more convenient than the supermarket, and a wide range of choices, the	Jingdong prefecture level city in Shandong
Fortune Shop	Green Intelligent Power Saver	There was a similar product many years ago. Now technology has evolved, buy one to try, according to the instructions in the box, it takes one or two dozen days to show results. Check your electric bill and see what happens.	Jingdong prefecture level city in Shandong
Fortune Shop	Green Intelligent Power Saver	I've been using this for a month or so and I can't see any results, it really is a scam as they say.	Jingdong prefecture level city in Shandong
Fortune Shop	Green Intelligent Power Saver	Very good use, a month can save a few degrees of power loss of electricity, introduced to the relatives to buy back to try.	Jingdong prefecture level city in Shandong
Fortune Shop	Green Intelligent Power Saver	This business is a big ** products are not qualified, with a month of electricity more than one hundred percent of thirty, the purchase agreed to return the goods but do not receive a refund after receiving the goods, looking for the official side does not help, the Jingdong is a big **, I have never shopped in	Jingdong prefecture level city in Shandong
Fortune Shop	Green Intelligent Power Saver	It's good. I tried it. It saves power.	Jingdong prefecture level city in Shandong
Fortune Shop	Green Intelligent Power Saver	It's really powerful! With this air conditioner can be turned on at any time without the pressure of paying the electricity bill! It's good for the people!	Jingdong prefecture level city in Shandong
Fortune Shop	Green Intelligent Power Saver	Nothing**, it seems like there's more	Jingdong prefecture level city in Shandong
Fortune Shop	Green Intelligent Power Saver	Power saving device received, well packaged, good quality, fast logistics	Jingdong prefecture level city in Shandong
Fortune Shop	Green Intelligent Power Saver	The service is okay, is that when I bought five, the desirable pieces are three, and then wait a few days and send me two replacement, I do not know if it is a mistake of the shipper, if I was not in the courier there to see, go home to say no, the	Jingdong prefecture level city in Shandong
Fortune Shop	Green Intelligent Power Saver	This thing will only cost me more if I use it, but before I used it, it was less than a ~ electricity a month, and after I used it, it was more than two hundred kWh a month.	Jingdong prefecture level city in Shandong
Fortune Shop	Green Intelligent Power Saver	I just used it. I don't know how it is.	Jingdong prefecture level city in Shandong
Fortune Shop	Green Intelligent Power Saver	I've received the product and I'm using it, so I'll see how it goes in a month!	Jingdong prefecture level city in Shandong
Fortune Shop	Green Intelligent Power Saver	The goods have just received the effect do not know how, please look forward to the results.	Jingdong prefecture level city in Shandong

Fortune Shop	Green Intelligent Power Saver	Very nice, will comment in detail after using it for a while.	Jingdong prefecture level city in Shandong
Fortune Shop	Green Intelligent Power Saver	Good stuff to trust and support	Jingdong prefecture level city in Shandong
Fortune Shop	Green Intelligent Power Saver	Power saving device, the material is very good practical and durable, the size is suitable, packaging is very good, the price is cheap, logistics is very fast!	Jingdong prefecture level city in Shandong
Fortune Shop	Green Intelligent Power Saver	I'll comment after I use it, doubt if it really saves power no	Jingdong prefecture level city in Shandong
Fortune Shop	Green Intelligent Power Saver	It's good quality and works well!	Jingdong prefecture level city in Shandong
Fortune Shop	Green Intelligent Power Saver	The real thing matches the picture and looks great!	Jingdong prefecture level city in Shandong
Fortune Shop	Green Intelligent Power Saver	hahaha just right hahahahahaha hiccup hahahaha	Jingdong prefecture level city in Shandong
Fortune Shop	Green Intelligent Power Saver	Plugged it in and we'll see when we pay the bills at the end of the year!	Jingdong prefecture level city in Shandong
Fortune Shop	Green Intelligent Power Saver	False advertising. It doesn't work.	Jingdong prefecture level city in Shandong
Fortune Shop	Green Intelligent Power Saver	Goods received, good effect	Jingdong prefecture level city in Shandong
Fortune Shop	Green Intelligent Power Saver	It looks okay, we'll see if it saves electricity when the time comes	Jingdong prefecture level city in Shandong
Fortune Shop	Green Intelligent Power Saver	Logistics is very fast quality is very good, the price is very affordable, accessories are also complete, installed, satisfied with good use	Jingdong prefecture level city in Shandong
Fortune Shop	Green Intelligent Power Saver	I've received the power saver. I'm looking forward to the savings.	Jingdong prefecture level city in Shandong
Fortune Shop	Green Intelligent Power Saver	Received, have not yet used, after use and then evaluate	Jingdong prefecture level city in Shandong
Fortune Shop	Green Intelligent Power Saver	I received the item, but I don't know if it saves power or not.	Jingdong prefecture level city in Shandong
Fortune Shop	Green Intelligent Power Saver	Quality is very good, no odor, fine workmanship, grain color are very like, satisfied!	Jingdong prefecture level city in Shandong
Fortune Shop	Green Intelligent Power Saver	Use it and see how it goes.	Jingdong prefecture level city in Shandong
Fortune Shop	Green Intelligent Power Saver	On Trial	Jingdong prefecture level city in Shandong
Fortune Shop	Green Intelligent Power Saver	I'll give you a good review. I don't know if it'll work.	Jingdong prefecture level city in Shandong
Fortune Shop	Green Intelligent Power Saver	The logistics is very fast and the package is intact. Good use of affordable. The effect is very good, is genuine	Jingdong prefecture level city in Shandong
Fortune Shop	Green Intelligent Power Saver	I don't know if it's as good as it sounds.	Jingdong prefecture level city in Shandong
Fortune Shop	Green Intelligent Power Saver	Nothing happens when you plug it in.	Jingdong prefecture level city in Shandong
Fortune Shop	Green Intelligent Power Saver	The quality of the stuff is very good, exactly the same as the seller's description, very satisfied!	Jingdong prefecture level city in Shandong
Fortune Shop	Green Intelligent Power Saver	The saver works great, it really works, it's affordable and worth buying.	Jingdong prefecture level city in Shandong
Fortune Shop	Green Intelligent Power Saver	Just used it for a couple days. I don't know yet.	Jingdong prefecture level city in Shandong
Fortune Shop	Green Intelligent Power Saver	The quality is quite good, cheaper than the physical store, good, next time will come again!	Jingdong prefecture level city in Shandong
Fortune Shop	Green Intelligent Power Saver	Good quality, cheap and affordable worth recommending to buy!	Jingdong prefecture level city in Shandong
Fortune Shop	Green Intelligent Power Saver	The logistics is very fast quality is very good very pleasant a shopping	Jingdong prefecture level city in Shandong
Fortune Shop	Green Intelligent Power Saver	Use it and then come back to evaluate!	Jingdong prefecture level city in Shandong
Fortune Shop	Green Intelligent Power Saver	Very good quality, well made, works well! Satisfied	Jingdong prefecture level city in Shandong
Fortune Shop	Green Intelligent Power Saver	# It's no use lying at all #	Jingdong prefecture level city in Shandong
Fortune Shop	Green Intelligent Power Saver	I can't get a voltage drop.	Jingdong prefecture level city in Shandong
Fortune Shop	Green Intelligent Power Saver	Evaluate after use	Jingdong prefecture level city in Shandong
Fortune Shop	Green Intelligent Power Saver	Still on trial.	Jingdong prefecture level city in Shandong
Fortune Shop	Green Intelligent Power Saver	ineffective	Jingdong prefecture level city in Shandong
Fortune Shop	Green Intelligent Power Saver	Satisfaction Shopping	Jingdong prefecture level city in Shandong
Fortune Shop	Green Intelligent Power Saver	It's a real power saver.	Jingdong prefecture level city in Shandong
Fortune Shop	Green Intelligent Power Saver	in use	Jingdong prefecture level city in Shandong
Fortune Shop	Green Intelligent Power Saver	Lies.	Jingdong prefecture level city in Shandong
Fortune Shop	Green Intelligent Power Saver	I don't know yet.	Jingdong prefecture level city in Shandong
Fortune Shop	Green Intelligent Power Saver	not care about anything	Jingdong prefecture level city in Shandong
Fortune Shop	Green Intelligent Power Saver	It doesn't seem to have much effect	Jingdong prefecture level city in Shandong
Fortune Shop	Green Intelligent Power Saver	Let the merchant see for himself, does the plug positive and negative terminals look like this?	Jingdong prefecture level city in Shandong
Fortune Shop	Green Intelligent Power Saver	It doesn't work well, the electricity bill is less before using it, but more after using it, contacting customer service, the service is still not good, and returning it is slow	Jingdong prefecture level city in Shandong
Fortune Shop	Green Intelligent Power Saver	With the advertisement product does not match, above the effective negative, power saving rate of two rows of words it, the product looks very thin, with 3 days to see the meter number is still with the previous no difference, which is obviously fraudulent behavior!	Jingdong prefecture level city in Shandong
Fortune Shop	Green Intelligent Power Saver	Doesn't save any power at all. Not much use.	Jingdong prefecture level city in Shandong
Fortune Shop	Green Intelligent Power Saver	The plugs are all loose, they're all the same length and they don't work at all	Jingdong prefecture level city in Shandong
Fortune Shop	Green Intelligent Power Saver	It's bad 👎 it's not effective at all, you'd be crazy to sell this garbage on the internet.	Jingdong prefecture level city in Shandong
Fortune Shop	Green Intelligent Power Saver	It doesn't save any power at all, so I hope the old timers don't fall for this scam again	Jingdong prefecture level city in Shandong
Fortune Shop	Green Intelligent Power Saver	I've been using it for over a month now and haven't noticed any power savings.	Jingdong prefecture level city in Shandong
Fortune Shop	Green Intelligent Power Saver	It doesn't work at all. The electricity bill is higher when you use it.	Jingdong prefecture level city in Shandong

Fortune Shop	Green Intelligent Power Saver	It doesn't save any power, it's still as bad as ever.	Jingdong prefecture level city in Shandong
Fortune Shop	Green Intelligent Power Saver	Last night before leaving work to look at the table, and then plugged into the node device, this morning and then look at the table, than the original save a few degrees of electricity, very good to have a power-saving effect, easy to use, satisfied with the shopping praise.	Jingdong prefecture level city in Shandong
Fortune Shop	Green Intelligent Power Saver	The product is real and effective, the electricity bill is reduced, really good, thanks to the merchants have so many good products, the product is real and effective, the electricity bill is reduced, want to save electricity friends quickly buy it, early use early to save money.	Jingdong prefecture level city in Shandong
Fortune Shop	Green Intelligent Power Saver	Saver saves time and money, baby appearance is beautiful, the quality of goods is good, and the same as the business description, good service attitude, packaging is complete, logistics is very fast, praise to	Jingdong prefecture level city in Shandong
Fortune Shop	Green Intelligent Power Saver	The effect is good, summer, home electricity is relatively large, buy and use a few days, feel the effect is very good, originally ten dollars a day electricity bill, now eight dollars a day, quite good	Jingdong prefecture level city in Shandong
Fortune Shop	Green Intelligent Power Saver	Indeed can save electricity and money, the first time I know this or from a friend's home to see, but also do not believe it, and now they use it to know that there is this good thing.	Jingdong prefecture level city in Shandong
Fortune Shop	Green Intelligent Power Saver	I have received the Smart Power Saver. Perfectly packaged. Fast delivery. Boss customer service attitude is also very good. It's worth buying. I plugged it in and tried it and it really saves more power than the original. I'll buy it again next time.	Jingdong prefecture level city in Shandong
Fortune Shop	Green Intelligent Power Saver	It's nice to save a dozen bucks a night on two air conditioners that used to take 30 or 40 degrees a night, but now it's only 10 or so degrees a night	Jingdong prefecture level city in Shandong
Fortune Shop	Green Intelligent Power Saver	Product no manufacturer's name no product qualification certificate, try two days to see the effect of	Jingdong prefecture level city in Shandong
Fortune Shop	Green Intelligent Power Saver	I've been using it for over a month and have saved over $80, so I've repurchased a few more back to save together	Jingdong prefecture level city in Shandong
Fortune Shop	Green Intelligent Power Saver	I haven't used it yet and will comment when I do, but it's well made.	Jingdong prefecture level city in Shandong
Fortune Shop	Green Intelligent Power Saver	It's quite energy efficient. It's really a boon for the home.	Jingdong prefecture level city in Shandong
Fortune Shop	Green Intelligent Power Saver	Energy-saving socket received a month, after using it for a month proved that it is indeed energy-saving, electricity bill saving, will buy it again, thank you!	Jingdong prefecture level city in Shandong
Fortune Shop	Green Intelligent Power Saver	There was a similar product many years ago. Now technology has evolved, buy one to try, according to the instructions in the box, it takes one or two dozen days to show results. Check your electric bill and see what happens.	Jingdong prefecture level city in Shandong
Fortune Shop	Green Intelligent Power Saver	Jingdong logistics is really fast, the same day the order arrived on the same day, more convenient than the supermarket, and a wide range of choices, the	Jingdong prefecture level city in Shandong
Fortune Shop	Green Intelligent Power Saver	I've been using this for a month or so and I can't see any results, it really is a scam as they say.	Jingdong prefecture level city in Shandong
Fortune Shop	Green Intelligent Power Saver	Very good use, a month can save a few degrees of power loss of electricity, introduced to the relatives to buy back to try.	Jingdong prefecture level city in Shandong
Fortune Shop	Green Intelligent Power Saver	It's really powerful! With this air conditioner can be turned on at any time without the pressure of paying the electricity bill! It's good for the people!	Jingdong prefecture level city in Shandong
Fortune Shop	Green Intelligent Power Saver	This business is a big ** products are not qualified, with a month of electricity more than one hundred percent of thirty, the purchase agreed to return the goods but do not receive a refund after receiving the goods, looking for the official side does not help, the Jingdong is a big **, I have never shopped in	Jingdong prefecture level city in Shandong
Fortune Shop	Green Intelligent Power Saver	It's good. I tried it. It saves power.	Jingdong prefecture level city in Shandong
Fortune Shop	Green Intelligent Power Saver	Nothing**, it seems like there's more	Jingdong prefecture level city in Shandong
Fortune Shop	Green Intelligent Power Saver	Power saving device received, well packaged, good quality, fast logistics	Jingdong prefecture level city in Shandong
Fortune Shop	Green Intelligent Power Saver	The service is okay, is that when I bought five, the desirable pieces are three, and then wait a few days and send me two replacement, I do not know if it is a mistake of the shipper, if I was not in the courier there to see, go home to say no, the	Jingdong prefecture level city in Shandong
Fortune Shop	Green Intelligent Power Saver	This thing will only cost me more if I use it, but before I used it, it was less than a ~ electricity a month, and after I used it, it was more than two hundred kWh a month.	Jingdong prefecture level city in Shandong
Fortune Shop	Green Intelligent Power Saver	I just used it. I don't know how it is.	Jingdong prefecture level city in Shandong
Fortune Shop	Green Intelligent Power Saver	I've received the product and I'm using it, so I'll see how it goes in a month!	Jingdong prefecture level city in Shandong
Fortune Shop	Green Intelligent Power Saver	The goods have just received the effect do not know how, please look forward to the results.	Jingdong prefecture level city in Shandong
Fortune Shop	Green Intelligent Power Saver	Power saving device, the material is very good practical and durable, the size is suitable, packaging is very good, the price is cheap, logistics is very fast!	Jingdong prefecture level city in Shandong
Fortune Shop	Green Intelligent Power Saver	Very nice, will comment in detail after using it for a while.	Jingdong prefecture level city in Shandong
Fortune Shop	Green Intelligent Power Saver	Good stuff to trust and support	Jingdong prefecture level city in Shandong
Fortune Shop	Green Intelligent Power Saver	I'll comment after I use it, doubt if it really saves power no	Jingdong prefecture level city in Shandong
Fortune Shop	Green Intelligent Power Saver	It's good quality and works well!	Jingdong prefecture level city in Shandong
Fortune Shop	Green Intelligent Power Saver	hahaha just right hahahahahaha hiccup hahahaha	Jingdong prefecture level city in Shandong
Fortune Shop	Green Intelligent Power Saver	The real thing matches the picture and looks great!	Jingdong prefecture level city in Shandong
Fortune Shop	Green Intelligent Power Saver	Plugged it in and we'll see when we pay the bills at the end of the year!	Jingdong prefecture level city in Shandong
Fortune Shop	Green Intelligent Power Saver	False advertising. It doesn't work.	Jingdong prefecture level city in Shandong
Fortune Shop	Green Intelligent Power Saver	Goods received, good effect	Jingdong prefecture level city in Shandong
Fortune Shop	Green Intelligent Power Saver	It looks okay, we'll see if it saves electricity when the time comes	Jingdong prefecture level city in Shandong
Fortune Shop	Green Intelligent Power Saver	Logistics is very fast quality is very good, the price is very affordable, accessories are also complete, installed, satisfied with good use	Jingdong prefecture level city in Shandong
Fortune Shop	Green Intelligent Power Saver	I've received the power saver. I'm looking forward to the savings.	Jingdong prefecture level city in Shandong
Fortune Shop	Green Intelligent Power Saver	Received, have not yet used, after use and then evaluate	Jingdong prefecture level city in Shandong
Fortune Shop	Green Intelligent Power Saver	I received the item, but I don't know if it saves power or not.	Jingdong prefecture level city in Shandong
Fortune Shop	Green Intelligent Power Saver	On Trial	Jingdong prefecture level city in Shandong
Fortune Shop	Green Intelligent Power Saver	Use it and see how it goes.	Jingdong prefecture level city in Shandong
Fortune Shop	Green Intelligent Power Saver	Quality is very good, no odor, fine workmanship, grain color are very like, satisfied!	Jingdong prefecture level city in Shandong
Fortune Shop	Green Intelligent Power Saver	I'll give you a good review. I don't know if it'll work.	Jingdong prefecture level city in Shandong
Fortune Shop	Green Intelligent Power Saver	The logistics is very fast and the package is intact. Good use of affordable. The effect is very good, is genuine	Jingdong prefecture level city in Shandong
Fortune Shop	Green Intelligent Power Saver	Nothing happens when you plug it in.	Jingdong prefecture level city in Shandong

Fortune Shop	Green Intelligent Power Saver	I don't know if it's as good as it sounds.	Jingdong prefecture level city in Shandong
Fortune Shop	Green Intelligent Power Saver	The quality of the stuff is very good, exactly the same as the seller's description, very satisfied!	Jingdong prefecture level city in Shandong
Fortune Shop	Green Intelligent Power Saver	The saver works great, it really works, it's affordable and worth buying.	Jingdong prefecture level city in Shandong
Fortune Shop	Green Intelligent Power Saver	Just used it for a couple days. I don't know yet.	Jingdong prefecture level city in Shandong
Fortune Shop	Green Intelligent Power Saver	The quality is quite good, cheaper than the physical store, good, next time will come again!	Jingdong prefecture level city in Shandong
Fortune Shop	Green Intelligent Power Saver	Good quality, cheap and affordable worth recommending to buy!	Jingdong prefecture level city in Shandong
Fortune Shop	Green Intelligent Power Saver	Very good quality, well made, works well! Satisfied	Jingdong prefecture level city in Shandong
Fortune Shop	Green Intelligent Power Saver	The logistics is very fast quality is very good very pleasant a shopping	Jingdong prefecture level city in Shandong
Fortune Shop	Green Intelligent Power Saver	Use it and then come back to evaluate!	Jingdong prefecture level city in Shandong
Fortune Shop	Green Intelligent Power Saver	# It's no use lying at all #	Jingdong prefecture level city in Shandong
Fortune Shop	Green Intelligent Power Saver	I can't get a voltage drop.	Jingdong prefecture level city in Shandong
Fortune Shop	Green Intelligent Power Saver	Evaluate after use	Jingdong prefecture level city in Shandong
Fortune Shop	Green Intelligent Power Saver	Still on trial.	Jingdong prefecture level city in Shandong
Fortune Shop	Green Intelligent Power Saver	ineffective	Jingdong prefecture level city in Shandong
Fortune Shop	Green Intelligent Power Saver	Satisfaction Shopping	Jingdong prefecture level city in Shandong
Fortune Shop	Green Intelligent Power Saver	Lies.	Jingdong prefecture level city in Shandong
Fortune Shop	Green Intelligent Power Saver	It's a real power saver.	Jingdong prefecture level city in Shandong
Fortune Shop	Green Intelligent Power Saver	in use	Jingdong prefecture level city in Shandong
Fortune Shop	Green Intelligent Power Saver	I don't know yet.	Jingdong prefecture level city in Shandong
Fortune Shop	Green Intelligent Power Saver	not care about anything	Jingdong prefecture level city in Shandong
Fortune Shop	Green Intelligent Power Saver	It doesn't seem to have much effect	Jingdong prefecture level city in Shandong
Fortune Shop	Green Intelligent Power Saver	Let the merchant see for himself, does the plug positive and negative terminals look like this?	Jingdong prefecture level city in Shandong
Fortune Shop	Green Intelligent Power Saver	It doesn't work well, the electricity bill is less before using it, but more after using it, contacting customer service, the service is still not good, and returning it is slow	Jingdong prefecture level city in Shandong
Fortune Shop	Green Intelligent Power Saver	With the advertisement product does not match, above the effective negative, power saving rate of two rows of words it, the product looks very thin, with 3 days to see the meter number is still with the previous no difference, which is obviously fraudulent behavior!	Jingdong prefecture level city in Shandong
Fortune Shop	Green Intelligent Power Saver	Doesn't save any power at all. Not much use.	Jingdong prefecture level city in Shandong
Fortune Shop	Green Intelligent Power Saver	The plugs are all loose, they're all the same length and they don't work at all	Jingdong prefecture level city in Shandong
Fortune Shop	Green Intelligent Power Saver	It's bad 👎 it's not effective at all, you'd be crazy to sell this garbage on the internet.	Jingdong prefecture level city in Shandong
Fortune Shop	Green Intelligent Power Saver	It doesn't save any power at all, so I hope the old timers don't fall for this scam again	Jingdong prefecture level city in Shandong
Fortune Shop	Green Intelligent Power Saver	I've been using it for over a month now and haven't noticed any power savings.	Jingdong prefecture level city in Shandong
Fortune Shop	Green Intelligent Power Saver	It doesn't work at all. The electricity bill is higher when you use it.	Jingdong prefecture level city in Shandong
Fortune Shop	Green Intelligent Power Saver	It doesn't save any power, it's still as bad as ever.	Jingdong prefecture level city in Shandong
Fortune Shop	Green Intelligent Power Saver	Last night before leaving work to look at the table, and then plugged into the node device, this morning and then look at the table, than the original save a few degrees of electricity, very good to have a power-saving effect, easy to use, satisfied with the shopping praise.	Jingdong prefecture level city in Shandong
Fortune Shop	Green Intelligent Power Saver	The product is real and effective, the electricity bill is reduced, really good, thanks to the merchants have so many good products, the product is real and effective, the electricity bill is reduced, want to save electricity friends quickly buy it, early use early to save money.	Jingdong prefecture level city in Shandong
Fortune Shop	Green Intelligent Power Saver	Saver saves time and money, baby appearance is beautiful, the quality of goods is good, and the same as the business description, good service attitude, packaging is complete, logistics is very fast, praise to	Jingdong prefecture level city in Shandong
Fortune Shop	Green Intelligent Power Saver	The effect is good, summer, home electricity is relatively large, buy and use a few days, feel the effect is very good, originally ten dollars a day electricity bill, now eight dollars a day, quite good	Jingdong prefecture level city in Shandong
Fortune Shop	Green Intelligent Power Saver	Indeed can save electricity and money, the first time I know this or from a friend's home to see, but also do not believe it, and now they use it to know that there is this good thing.	Jingdong prefecture level city in Shandong
Fortune Shop	Green Intelligent Power Saver	I have received the Smart Power Saver. Perfectly packaged. Fast delivery. Boss customer service attitude is also very good. It's worth buying. I plugged it in and tried it and it really saves more power than the original. I'll buy it again next time.	Jingdong prefecture level city in Shandong
Fortune Shop	Green Intelligent Power Saver	It's nice to save a dozen bucks a night on two air conditioners that used to take 30 or 40 degrees a night, but now it's only 10 or so degrees a night	Jingdong prefecture level city in Shandong
Fortune Shop	Green Intelligent Power Saver	Product no manufacturer's name no product qualification certificate, try two days to see the effect of	Jingdong prefecture level city in Shandong
Fortune Shop	Green Intelligent Power Saver	I've been using it for over a month and have saved over $80, so I've repurchased a few more back to save together	Jingdong prefecture level city in Shandong
Fortune Shop	Green Intelligent Power Saver	I haven't used it yet and will comment when I do, but it's well made.	Jingdong prefecture level city in Shandong
Fortune Shop	Green Intelligent Power Saver	It's quite energy efficient. It's really a boon for the home.	Jingdong prefecture level city in Shandong
Fortune Shop	Green Intelligent Power Saver	Energy-saving socket received a month, after using it for a month proved that it is indeed energy-saving, electricity bill saving, will buy it again, thank you!	Jingdong prefecture level city in Shandong
Fortune Shop	Green Intelligent Power Saver	There was a similar product many years ago. Now technology has evolved, buy one to try, according to the instructions in the box, it takes one or two dozen days to show results. Check your electric bill and see what happens.	Jingdong prefecture level city in Shandong
Fortune Shop	Green Intelligent Power Saver	Jingdong logistics is really fast, the same day the order arrived on the same day, more convenient than the supermarket, and a wide range of choices, the	Jingdong prefecture level city in Shandong
Fortune Shop	Green Intelligent Power Saver	I've been using this for a month or so and I can't see any results, it really is a scam as they say.	Jingdong prefecture level city in Shandong
Fortune Shop	Green Intelligent Power Saver	Very good use, a month can save a few degrees of power loss of electricity, introduced to the relatives to buy back to try.	Jingdong prefecture level city in Shandong
Fortune Shop	Green Intelligent Power Saver	It's really powerful! With this air conditioner can be turned on at any time without the pressure of paying the electricity bill! It's good for the people!	Jingdong prefecture level city in Shandong

Fortune Shop	Green Intelligent Power Saver	This business is a big ** products are not qualified, with a month of electricity more than one hundred percent of thirty, the purchase agreed to return the goods but do not receive a refund after receiving the goods, looking for the official side does not help, the Jingdong is a big **, I have never shopped in	Jingdong prefecture level city in Shandong
Fortune Shop	Green Intelligent Power Saver	It's good. I tried it. It saves power.	Jingdong prefecture level city in Shandong
Fortune Shop	Green Intelligent Power Saver	Nothing**, it seems like there's more	Jingdong prefecture level city in Shandong
Fortune Shop	Green Intelligent Power Saver	Power saving device received, well packaged, good quality, fast logistics	Jingdong prefecture level city in Shandong
Fortune Shop	Green Intelligent Power Saver	The service is okay, is that when I bought five, the desirable pieces are three, and then wait a few days and send me two replacement, I do not know if it is a mistake of the shipper, if I was not in the courier there to see, go home to say no, the	Jingdong prefecture level city in Shandong
Fortune Shop	Green Intelligent Power Saver	This thing will only cost me more if I use it, but before I used it, it was less than a ~ electricity a month, and after I used it, it was more than two hundred kWh a month.	Jingdong prefecture level city in Shandong
Fortune Shop	Green Intelligent Power Saver	I just used it. I don't know how it is.	Jingdong prefecture level city in Shandong
Fortune Shop	Green Intelligent Power Saver	I've received the product and I'm using it, so I'll see how it goes in a month!	Jingdong prefecture level city in Shandong
Fortune Shop	Green Intelligent Power Saver	The goods have just received the effect do not know how, please look forward to the results.	Jingdong prefecture level city in Shandong
Fortune Shop	Green Intelligent Power Saver	Power saving device, the material is very good practical and durable, the size is suitable, packaging is very good, the price is cheap, logistics is very fast!	Jingdong prefecture level city in Shandong
Fortune Shop	Green Intelligent Power Saver	Very nice, will comment in detail after using it for a while.	Jingdong prefecture level city in Shandong
Fortune Shop	Green Intelligent Power Saver	Good stuff to trust and support	Jingdong prefecture level city in Shandong
Fortune Shop	Green Intelligent Power Saver	I'll comment after I use it, doubt if it really saves power no	Jingdong prefecture level city in Shandong
Fortune Shop	Green Intelligent Power Saver	It's good quality and works well!	Jingdong prefecture level city in Shandong
Fortune Shop	Green Intelligent Power Saver	hahaha just right hahahahahaha hiccup hahahaha	Jingdong prefecture level city in Shandong
Fortune Shop	Green Intelligent Power Saver	The real thing matches the picture and looks great!	Jingdong prefecture level city in Shandong
Fortune Shop	Green Intelligent Power Saver	Plugged it in and we'll see when we pay the bills at the end of the year!	Jingdong prefecture level city in Shandong
Fortune Shop	Green Intelligent Power Saver	False advertising. It doesn't work.	Jingdong prefecture level city in Shandong
Fortune Shop	Green Intelligent Power Saver	Goods received, good effect	Jingdong prefecture level city in Shandong
Fortune Shop	Green Intelligent Power Saver	It looks okay, we'll see if it saves electricity when the time comes	Jingdong prefecture level city in Shandong
Fortune Shop	Green Intelligent Power Saver	Logistics is very fast quality is very good, the price is very affordable, accessories are also complete, installed, satisfied with good use	Jingdong prefecture level city in Shandong
Fortune Shop	Green Intelligent Power Saver	I've received the power saver. I'm looking forward to the savings.	Jingdong prefecture level city in Shandong
Fortune Shop	Green Intelligent Power Saver	Received, have not yet used, after use and then evaluate	Jingdong prefecture level city in Shandong
Fortune Shop	Green Intelligent Power Saver	I received the item, but I don't know if it saves power or not.	Jingdong prefecture level city in Shandong
Fortune Shop	Green Intelligent Power Saver	On Trial	Jingdong prefecture level city in Shandong
Fortune Shop	Green Intelligent Power Saver	Use it and see how it goes.	Jingdong prefecture level city in Shandong
Fortune Shop	Green Intelligent Power Saver	Quality is very good, no odor, fine workmanship, grain color are very like, satisfied!	Jingdong prefecture level city in Shandong
Fortune Shop	Green Intelligent Power Saver	I'll give you a good review. I don't know if it'll work.	Jingdong prefecture level city in Shandong
Fortune Shop	Green Intelligent Power Saver	The logistics is very fast and the package is intact. Good use of affordable. The effect is very good, is genuine	Jingdong prefecture level city in Shandong
Fortune Shop	Green Intelligent Power Saver	Nothing happens when you plug it in.	Jingdong prefecture level city in Shandong
Fortune Shop	Green Intelligent Power Saver	I don't know if it's as good as it sounds.	Jingdong prefecture level city in Shandong
Fortune Shop	Green Intelligent Power Saver	The quality of the stuff is very good, exactly the same as the seller's description, very satisfied!	Jingdong prefecture level city in Shandong
Fortune Shop	Green Intelligent Power Saver	The saver works great, it really works, it's affordable and worth buying.	Jingdong prefecture level city in Shandong
Fortune Shop	Green Intelligent Power Saver	Just used it for a couple days. I don't know yet.	Jingdong prefecture level city in Shandong
Fortune Shop	Green Intelligent Power Saver	The quality is quite good, cheaper than the physical store, good, next time will come again!	Jingdong prefecture level city in Shandong
Fortune Shop	Green Intelligent Power Saver	Good quality, cheap and affordable worth recommending to buy!	Jingdong prefecture level city in Shandong
Fortune Shop	Green Intelligent Power Saver	Very good quality, well made, works well! Satisfied	Jingdong prefecture level city in Shandong
Fortune Shop	Green Intelligent Power Saver	The logistics is very fast quality is very good very pleasant a shopping	Jingdong prefecture level city in Shandong
Fortune Shop	Green Intelligent Power Saver	Use it and then come back to evaluate!	Jingdong prefecture level city in Shandong
Fortune Shop	Green Intelligent Power Saver	# It's no use lying at all #	Jingdong prefecture level city in Shandong
Fortune Shop	Green Intelligent Power Saver	I can't get a voltage drop.	Jingdong prefecture level city in Shandong
Fortune Shop	Green Intelligent Power Saver	Evaluate after use	Jingdong prefecture level city in Shandong
Fortune Shop	Green Intelligent Power Saver	Still on trial.	Jingdong prefecture level city in Shandong
Fortune Shop	Green Intelligent Power Saver	ineffective	Jingdong prefecture level city in Shandong
Fortune Shop	Green Intelligent Power Saver	Satisfaction Shopping	Jingdong prefecture level city in Shandong
Fortune Shop	Green Intelligent Power Saver	Lies.	Jingdong prefecture level city in Shandong
Fortune Shop	Green Intelligent Power Saver	It's a real power saver.	Jingdong prefecture level city in Shandong
Fortune Shop	Green Intelligent Power Saver	in use	Jingdong prefecture level city in Shandong
Fortune Shop	Green Intelligent Power Saver	I don't know yet.	Jingdong prefecture level city in Shandong
Fortune Shop	Green Intelligent Power Saver	not care about anything	Jingdong prefecture level city in Shandong

Fortune Shop	Green Intelligent Power Saver	It doesn't seem to have much effect	Jingdong prefecture level city in Shandong
Fortune Shop	Green Intelligent Power Saver	Let the merchant see for himself, does the plug positive and negative terminals look like this?	Jingdong prefecture level city in Shandong
Fortune Shop	Green Intelligent Power Saver	It doesn't work well, the electricity bill is less before using it, but more after using it, contacting customer service, the service is still not good, and returning it is slow	Jingdong prefecture level city in Shandong
Fortune Shop	Green Intelligent Power Saver	With the advertisement product does not match, above the effective negative, power saving rate of two rows of words it, the product looks very thin, with 3 days to see the meter number is still with the previous no difference, which is obviously fraudulent behavior!	Jingdong prefecture level city in Shandong
Fortune Shop	Green Intelligent Power Saver	Doesn't save any power at all. Not much use.	Jingdong prefecture level city in Shandong
Fortune Shop	Green Intelligent Power Saver	The plugs are all loose, they're all the same length and they don't work at all	Jingdong prefecture level city in Shandong
Fortune Shop	Green Intelligent Power Saver	It's bad 👎 it's not effective at all, you'd be crazy to sell this garbage on the internet.	Jingdong prefecture level city in Shandong
Fortune Shop	Green Intelligent Power Saver	It doesn't save any power at all, so I hope the old timers don't fall for this scam again	Jingdong prefecture level city in Shandong
Fortune Shop	Green Intelligent Power Saver	I've been using it for over a month now and haven't noticed any power savings.	Jingdong prefecture level city in Shandong
Fortune Shop	Green Intelligent Power Saver	It doesn't work at all. The electricity bill is higher when you use it.	Jingdong prefecture level city in Shandong
Fortune Shop	Green Intelligent Power Saver	It doesn't save any power, it's still as bad as ever.	Jingdong prefecture level city in Shandong
Fortune Shop	Green Intelligent Power Saver	Last night before leaving work to look at the table, and then plugged into the node device, this morning and then look at the table, than the original save a few degrees of electricity, very good to have a power-saving effect, easy to use, satisfied with the shopping praise.	Jingdong prefecture level city in Shandong
Fortune Shop	Green Intelligent Power Saver	The product is real and effective, the electricity bill is reduced, really good, thanks to the merchants have so many good products, the product is real and effective, the electricity bill is reduced, want to save electricity friends quickly buy it, early use early to save money.	Jingdong prefecture level city in Shandong
Fortune Shop	Green Intelligent Power Saver	Saver saves time and money, baby appearance is beautiful, the quality of goods is good, and the same as the business description, good service attitude, packaging is complete, logistics is very fast, praise to	Jingdong prefecture level city in Shandong
Fortune Shop	Green Intelligent Power Saver	The effect is good, summer, home electricity is relatively large, buy and use a few days, feel the effect is very good, originally ten dollars a day electricity bill, now eight dollars a day, quite good	Jingdong prefecture level city in Shandong
Fortune Shop	Green Intelligent Power Saver	Indeed can save electricity and money, the first time I know this or from a friend's home to see, but also do not believe it, and now they use it to know that there is this good thing.	Jingdong prefecture level city in Shandong
Fortune Shop	Green Intelligent Power Saver	I have received the Smart Power Saver. Perfectly packaged. Fast delivery. Boss customer service attitude is also very good. It's worth buying. I plugged it in and tried it and it really saves more power than the original. I'll buy it again next time.	Jingdong prefecture level city in Shandong
Fortune Shop	Green Intelligent Power Saver	It's nice to save a dozen bucks a night on two air conditioners that used to take 30 or 40 degrees a night, but now it's only 10 or so degrees a night	Jingdong prefecture level city in Shandong
Fortune Shop	Green Intelligent Power Saver	Product no manufacturer's name no product qualification certificate, try two days to see the effect of	Jingdong prefecture level city in Shandong
Fortune Shop	Green Intelligent Power Saver	I've been using it for over a month and have saved over $80, so I've repurchased a few more back to save together	Jingdong prefecture level city in Shandong
Fortune Shop	Green Intelligent Power Saver	I haven't used it yet and will comment when I do, but it's well made.	Jingdong prefecture level city in Shandong
Fortune Shop	Green Intelligent Power Saver	It's quite energy efficient. It's really a boon for the home.	Jingdong prefecture level city in Shandong
Fortune Shop	Green Intelligent Power Saver	Energy-saving socket received a month, after using it for a month proved that it is indeed energy-saving, electricity bill saving, will buy it again, thank you!	Jingdong prefecture level city in Shandong
Fortune Shop	Green Intelligent Power Saver	There was a similar product many years ago. Now technology has evolved, buy one to try, according to the instructions in the box, it takes one or two dozen days to show results. Check your electric bill and see what happens.	Jingdong prefecture level city in Shandong
Fortune Shop	Green Intelligent Power Saver	Jingdong logistics is really fast, the same day the order arrived on the same day, more convenient than the supermarket, and a wide range of choices, the	Jingdong prefecture level city in Shandong
Fortune Shop	Green Intelligent Power Saver	I've been using this for a month or so and I can't see any results, it really is a scam as they say.	Jingdong prefecture level city in Shandong
Fortune Shop	Green Intelligent Power Saver	Very good use, a month can save a few degrees of power loss of electricity, introduced to the relatives to buy back to try.	Jingdong prefecture level city in Shandong
Fortune Shop	Green Intelligent Power Saver	It's really powerful! With this air conditioner can be turned on at any time without the pressure of paying the electricity bill! It's good for the people!	Jingdong prefecture level city in Shandong
Fortune Shop	Green Intelligent Power Saver	This business is a big ** products are not qualified, with a month of electricity more than one hundred percent of thirty, the purchase agreed to return the goods but do not receive a refund after receiving the goods, looking for the official side does not help, the Jingdong is a big **, I have never shopped in	Jingdong prefecture level city in Shandong
Fortune Shop	Green Intelligent Power Saver	It's good. I tried it. It saves power.	Jingdong prefecture level city in Shandong
Fortune Shop	Green Intelligent Power Saver	Nothing**, it seems like there's more	Jingdong prefecture level city in Shandong
Fortune Shop	Green Intelligent Power Saver	Power saving device received, well packaged, good quality, fast logistics	Jingdong prefecture level city in Shandong
Fortune Shop	Green Intelligent Power Saver	The service is okay, is that when I bought five, the desirable pieces are three, and then wait a few days and send me two replacement, I do not know if it is a mistake of the shipper, if I was not in the courier there to see, go home to say no, the	Jingdong prefecture level city in Shandong
Fortune Shop	Green Intelligent Power Saver	This thing will only cost me more if I use it, but before I used it, it was less than a ~ electricity a month, and after I used it, it was more than two hundred kWh a month.	Jingdong prefecture level city in Shandong
Fortune Shop	Green Intelligent Power Saver	I just used it. I don't know how it is.	Jingdong prefecture level city in Shandong
Fortune Shop	Green Intelligent Power Saver	I've received the product and I'm using it, so I'll see how it goes in a month!	Jingdong prefecture level city in Shandong
Fortune Shop	Green Intelligent Power Saver	The goods have just received the effect do not know how, please look forward to the results.	Jingdong prefecture level city in Shandong
Fortune Shop	Green Intelligent Power Saver	Power saving device, the material is very good practical and durable, the size is suitable, packaging is very good, the price is cheap, logistics is very fast!	Jingdong prefecture level city in Shandong
Fortune Shop	Green Intelligent Power Saver	Very nice, will comment in detail after using it for a while.	Jingdong prefecture level city in Shandong
Fortune Shop	Green Intelligent Power Saver	Good stuff to trust and support	Jingdong prefecture level city in Shandong
Fortune Shop	Green Intelligent Power Saver	I'll comment after I use it, doubt if it really saves power no	Jingdong prefecture level city in Shandong
Fortune Shop	Green Intelligent Power Saver	It's good quality and works well!	Jingdong prefecture level city in Shandong
Fortune Shop	Green Intelligent Power Saver	hahaha just right hahahahahaha hiccup hahahaha	Jingdong prefecture level city in Shandong
Fortune Shop	Green Intelligent Power Saver	The real thing matches the picture and looks great!	Jingdong prefecture level city in Shandong
Fortune Shop	Green Intelligent Power Saver	Plugged it in and we'll see when we pay the bills at the end of the year!	Jingdong prefecture level city in Shandong
Fortune Shop	Green Intelligent Power Saver	False advertising. It doesn't work.	Jingdong prefecture level city in Shandong
Fortune Shop	Green Intelligent Power Saver	Goods received, good effect	Jingdong prefecture level city in Shandong
Fortune Shop	Green Intelligent Power Saver	It looks okay, we'll see if it saves electricity when the time comes	Jingdong prefecture level city in Shandong

Fortune Shop	Green Intelligent Power Saver	Logistics is very fast quality is very good, the price is very affordable, accessories are also complete, installed, satisfied with good use	Jingdong prefecture level city in Shandong
Fortune Shop	Green Intelligent Power Saver	I've received the power saver. I'm looking forward to the savings.	Jingdong prefecture level city in Shandong
Fortune Shop	Green Intelligent Power Saver	Received, have not yet used, after use and then evaluate	Jingdong prefecture level city in Shandong
Fortune Shop	Green Intelligent Power Saver	I received the item, but I don't know if it saves power or not.	Jingdong prefecture level city in Shandong
Fortune Shop	Green Intelligent Power Saver	On Trial	Jingdong prefecture level city in Shandong
Fortune Shop	Green Intelligent Power Saver	Use it and see how it goes.	Jingdong prefecture level city in Shandong
Fortune Shop	Green Intelligent Power Saver	Quality is very good, no odor, fine workmanship, grain color are very like, satisfied!	Jingdong prefecture level city in Shandong
Fortune Shop	Green Intelligent Power Saver	I'll give you a good review. I don't know if it'll work.	Jingdong prefecture level city in Shandong
Fortune Shop	Green Intelligent Power Saver	The logistics is very fast and the package is intact. Good use of affordable. The effect is very good, is genuine	Jingdong prefecture level city in Shandong
Fortune Shop	Green Intelligent Power Saver	Nothing happens when you plug it in.	Jingdong prefecture level city in Shandong
Fortune Shop	Green Intelligent Power Saver	I don't know if it's as good as it sounds.	Jingdong prefecture level city in Shandong
Fortune Shop	Green Intelligent Power Saver	The quality of the stuff is very good, exactly the same as the seller's description, very satisfied!	Jingdong prefecture level city in Shandong
Fortune Shop	Green Intelligent Power Saver	The saver works great, it really works, it's affordable and worth buying.	Jingdong prefecture level city in Shandong
Fortune Shop	Green Intelligent Power Saver	Just used it for a couple days. I don't know yet.	Jingdong prefecture level city in Shandong
Fortune Shop	Green Intelligent Power Saver	The quality is quite good, cheaper than the physical store, good, next time will come again!	Jingdong prefecture level city in Shandong
Fortune Shop	Green Intelligent Power Saver	Good quality, cheap and affordable worth recommending to buy!	Jingdong prefecture level city in Shandong
Fortune Shop	Green Intelligent Power Saver	Very good quality, well made, works well! Satisfied	Jingdong prefecture level city in Shandong
Fortune Shop	Green Intelligent Power Saver	The logistics is very fast quality is very good very pleasant a shopping	Jingdong prefecture level city in Shandong
Fortune Shop	Green Intelligent Power Saver	Use it and then come back to evaluate!	Jingdong prefecture level city in Shandong
Fortune Shop	Green Intelligent Power Saver	# It's no use lying at all #	Jingdong prefecture level city in Shandong
Fortune Shop	Green Intelligent Power Saver	I can't get a voltage drop.	Jingdong prefecture level city in Shandong
Fortune Shop	Green Intelligent Power Saver	Evaluate after use	Jingdong prefecture level city in Shandong
Fortune Shop	Green Intelligent Power Saver	Still on trial.	Jingdong prefecture level city in Shandong
Fortune Shop	Green Intelligent Power Saver	ineffective	Jingdong prefecture level city in Shandong
Fortune Shop	Green Intelligent Power Saver	Satisfaction Shopping	Jingdong prefecture level city in Shandong
Fortune Shop	Green Intelligent Power Saver	Lies.	Jingdong prefecture level city in Shandong
Fortune Shop	Green Intelligent Power Saver	It's a real power saver.	Jingdong prefecture level city in Shandong
Fortune Shop	Green Intelligent Power Saver	in use	Jingdong prefecture level city in Shandong
Fortune Shop	Green Intelligent Power Saver	I don't know yet.	Jingdong prefecture level city in Shandong
Fortune Shop	Green Intelligent Power Saver	not care about anything	Jingdong prefecture level city in Shandong
Fortune Shop	Green Intelligent Power Saver	It doesn't seem to have much effect	Jingdong prefecture level city in Shandong
Fortune Shop	Green Intelligent Power Saver	Let the merchant see for himself, does the plug positive and negative terminals look like this?	Jingdong prefecture level city in Shandong
Fortune Shop	Green Intelligent Power Saver	It doesn't work well, the electricity bill is less before using it, but more after using it, contacting customer service, the service is still not good, and returning it is slow	Jingdong prefecture level city in Shandong
Fortune Shop	Green Intelligent Power Saver	With the advertisement product does not match, above the effective negative, power saving rate of two rows of words it, the product looks very thin, with 3 days to see the meter number is still with the previous no difference, which is obviously fraudulent behavior!	Jingdong prefecture level city in Shandong
Fortune Shop	Green Intelligent Power Saver	Doesn't save any power at all. Not much use.	Jingdong prefecture level city in Shandong
Fortune Shop	Green Intelligent Power Saver	The plugs are all loose, they're all the same length and they don't work at all	Jingdong prefecture level city in Shandong
Fortune Shop	Green Intelligent Power Saver	It's bad 👎 it's not effective at all, you'd be crazy to sell this garbage on the internet.	Jingdong prefecture level city in Shandong
Fortune Shop	Green Intelligent Power Saver	It doesn't save any power at all, so I hope the old timers don't fall for this scam again	Jingdong prefecture level city in Shandong
Fortune Shop	Green Intelligent Power Saver	I've been using it for over a month now and haven't noticed any power savings.	Jingdong prefecture level city in Shandong
Fortune Shop	Green Intelligent Power Saver	It doesn't work at all. The electricity bill is higher when you use it.	Jingdong prefecture level city in Shandong
Fortune Shop	Green Intelligent Power Saver	It doesn't save any power, it's still as bad as ever.	Jingdong prefecture level city in Shandong
Fortune Shop	Green Intelligent Power Saver	Last night before leaving work to look at the table, and then plugged into the node device, this morning and then look at the table, than the original save a few degrees of electricity, very good to have a power-saving effect, easy to use, satisfied with the shopping praise.	Jingdong prefecture level city in Shandong
Fortune Shop	Green Intelligent Power Saver	The product is real and effective, the electricity bill is reduced, really good, thanks to the merchants have so many good products, the product is real and effective, the electricity bill is reduced, want to save electricity friends quickly buy it, early use early to save money.	Jingdong prefecture level city in Shandong
Fortune Shop	Green Intelligent Power Saver	Saver saves time and money, baby appearance is beautiful, the quality of goods is good, and the same as the business description, good service attitude, packaging is complete, logistics is very fast, praise to	Jingdong prefecture level city in Shandong
Fortune Shop	Green Intelligent Power Saver	The effect is good, summer, home electricity is relatively large, buy and use a few days, feel the effect is very good, originally ten dollars a day electricity bill, now eight dollars a day, quite good	Jingdong prefecture level city in Shandong
Fortune Shop	Green Intelligent Power Saver	Indeed can save electricity and money, the first time I know this or from a friend's home to see, but also do not believe it, and now they use it to know that there is this good thing.	Jingdong prefecture level city in Shandong
Fortune Shop	Green Intelligent Power Saver	I have received the Smart Power Saver. Perfectly packaged. Fast delivery. Boss customer service attitude is also very good. It's worth buying. I plugged it in and tried it and it really saves more power than the original. I'll buy it again next time.	Jingdong prefecture level city in Shandong
Fortune Shop	Green Intelligent Power Saver	It's nice to save a dozen bucks a night on two air conditioners that used to take 30 or 40 degrees a night, but now it's only 10 or so degrees a night	Jingdong prefecture level city in Shandong
Fortune Shop	Green Intelligent Power Saver	Product no manufacturer's name no product qualification certificate, try two days to see the effect of	Jingdong prefecture level city in Shandong

Fortune Shop	Green Intelligent Power Saver	I've been using it for over a month and have saved over $80, so I've repurchased a few more back to save together	Jingdong prefecture level city in Shandong
Fortune Shop	Green Intelligent Power Saver	I haven't used it yet and will comment when I do, but it's well made.	Jingdong prefecture level city in Shandong
Fortune Shop	Green Intelligent Power Saver	It's quite energy efficient. It's really a boon for the home.	Jingdong prefecture level city in Shandong
Fortune Shop	Green Intelligent Power Saver	Energy-saving socket received a month, after using it for a month proved that it is indeed energy-saving, electricity bill saving, will buy it again, thank you!	Jingdong prefecture level city in Shandong
Fortune Shop	Green Intelligent Power Saver	There was a similar product many years ago. Now technology has evolved, buy one to try, according to the instructions in the box, it takes one or two dozen days to show results. Check your electric bill and see what happens.	Jingdong prefecture level city in Shandong
Fortune Shop	Green Intelligent Power Saver	Jingdong logistics is really fast, the same day the order arrived on the same day, more convenient than the supermarket, and a wide range of choices, the	Jingdong prefecture level city in Shandong
Fortune Shop	Green Intelligent Power Saver	I've been using this for a month or so and I can't see any results, it really is a scam as they say.	Jingdong prefecture level city in Shandong
Fortune Shop	Green Intelligent Power Saver	Very good use, a month can save a few degrees of power loss of electricity, introduced to the relatives to buy back to try.	Jingdong prefecture level city in Shandong
Fortune Shop	Green Intelligent Power Saver	It's really powerful! With this air conditioner can be turned on at any time without the pressure of paying the electricity bill! It's good for the people!	Jingdong prefecture level city in Shandong
Fortune Shop	Green Intelligent Power Saver	This business is a big ** products are not qualified, with a month of electricity more than one hundred percent of thirty, the purchase agreed to return the goods but do not receive a refund after receiving the goods, looking for the official side does not help, the Jingdong is a big **, I have never shopped in	Jingdong prefecture level city in Shandong
Fortune Shop	Green Intelligent Power Saver	It's good. I tried it. It saves power.	Jingdong prefecture level city in Shandong
Fortune Shop	Green Intelligent Power Saver	Nothing**, it seems like there's more	Jingdong prefecture level city in Shandong
Fortune Shop	Green Intelligent Power Saver	Power saving device received, well packaged, good quality, fast logistics	Jingdong prefecture level city in Shandong
Fortune Shop	Green Intelligent Power Saver	The service is okay, is that when I bought five, the desirable pieces are three, and then wait a few days and send me two replacement, I do not know if it is a mistake of the shipper, if I was not in the courier there to see, go home to say no, the	Jingdong prefecture level city in Shandong
Fortune Shop	Green Intelligent Power Saver	This thing will only cost me more if I use it, but before I used it, it was less than a ~ electricity a month, and after I used it, it was more than two hundred kWh a month.	Jingdong prefecture level city in Shandong
Fortune Shop	Green Intelligent Power Saver	I just used it. I don't know how it is.	Jingdong prefecture level city in Shandong
Fortune Shop	Green Intelligent Power Saver	I've received the product and I'm using it, so I'll see how it goes in a month!	Jingdong prefecture level city in Shandong
Fortune Shop	Green Intelligent Power Saver	The goods have just received the effect do not know how, please look forward to the results.	Jingdong prefecture level city in Shandong
Fortune Shop	Green Intelligent Power Saver	Power saving device, the material is very good practical and durable, the size is suitable, packaging is very good, the price is cheap, logistics is very fast!	Jingdong prefecture level city in Shandong
Fortune Shop	Green Intelligent Power Saver	Very nice, will comment in detail after using it for a while.	Jingdong prefecture level city in Shandong
Fortune Shop	Green Intelligent Power Saver	Good stuff to trust and support	Jingdong prefecture level city in Shandong
Fortune Shop	Green Intelligent Power Saver	I'll comment after I use it, doubt if it really saves power no	Jingdong prefecture level city in Shandong
Fortune Shop	Green Intelligent Power Saver	It's good quality and works well!	Jingdong prefecture level city in Shandong
Fortune Shop	Green Intelligent Power Saver	hahaha just right hahahahahaha hiccup hahahaha	Jingdong prefecture level city in Shandong
Fortune Shop	Green Intelligent Power Saver	The real thing matches the picture and looks great!	Jingdong prefecture level city in Shandong
Fortune Shop	Green Intelligent Power Saver	Plugged it in and we'll see when we pay the bills at the end of the year!	Jingdong prefecture level city in Shandong
Fortune Shop	Green Intelligent Power Saver	False advertising. It doesn't work.	Jingdong prefecture level city in Shandong
Fortune Shop	Green Intelligent Power Saver	Goods received, good effect	Jingdong prefecture level city in Shandong
Fortune Shop	Green Intelligent Power Saver	It looks okay, we'll see if it saves electricity when the time comes	Jingdong prefecture level city in Shandong
Fortune Shop	Green Intelligent Power Saver	Logistics is very fast quality is very good, the price is very affordable, accessories are also complete, installed, satisfied with good use	Jingdong prefecture level city in Shandong
Fortune Shop	Green Intelligent Power Saver	I've received the power saver. I'm looking forward to the savings.	Jingdong prefecture level city in Shandong
Fortune Shop	Green Intelligent Power Saver	Received, have not yet used, after use and then evaluate	Jingdong prefecture level city in Shandong
Fortune Shop	Green Intelligent Power Saver	I received the item, but I don't know if it saves power or not.	Jingdong prefecture level city in Shandong
Fortune Shop	Green Intelligent Power Saver	On Trial	Jingdong prefecture level city in Shandong
Fortune Shop	Green Intelligent Power Saver	Use it and see how it goes.	Jingdong prefecture level city in Shandong
Fortune Shop	Green Intelligent Power Saver	Quality is very good, no odor, fine workmanship, grain color are very like, satisfied!	Jingdong prefecture level city in Shandong
Fortune Shop	Green Intelligent Power Saver	I'll give you a good review. I don't know if it'll work.	Jingdong prefecture level city in Shandong
Fortune Shop	Green Intelligent Power Saver	The logistics is very fast and the package is intact. Good use of affordable. The effect is very good, is genuine	Jingdong prefecture level city in Shandong
Fortune Shop	Green Intelligent Power Saver	Nothing happens when you plug it in.	Jingdong prefecture level city in Shandong
Fortune Shop	Green Intelligent Power Saver	I don't know if it's as good as it sounds.	Jingdong prefecture level city in Shandong
Fortune Shop	Green Intelligent Power Saver	The quality of the stuff is very good, exactly the same as the seller's description, very satisfied!	Jingdong prefecture level city in Shandong
Fortune Shop	Green Intelligent Power Saver	The saver works great, it really works, it's affordable and worth buying.	Jingdong prefecture level city in Shandong
Fortune Shop	Green Intelligent Power Saver	Just used it for a couple days. I don't know yet.	Jingdong prefecture level city in Shandong
Fortune Shop	Green Intelligent Power Saver	The quality is quite good, cheaper than the physical store, good, next time will come again!	Jingdong prefecture level city in Shandong
Fortune Shop	Green Intelligent Power Saver	Good quality, cheap and affordable worth recommending to buy!	Jingdong prefecture level city in Shandong
Fortune Shop	Green Intelligent Power Saver	Very good quality, well made, works well! Satisfied	Jingdong prefecture level city in Shandong
Fortune Shop	Green Intelligent Power Saver	The logistics is very fast quality is very good very pleasant a shopping	Jingdong prefecture level city in Shandong
Fortune Shop	Green Intelligent Power Saver	Use it and then come back to evaluate!	Jingdong prefecture level city in Shandong
Fortune Shop	Green Intelligent Power Saver	# It's no use lying at all #	Jingdong prefecture level city in Shandong
Fortune Shop	Green Intelligent Power Saver	I can't get a voltage drop.	Jingdong prefecture level city in Shandong

Fortune Shop	Green Intelligent Power Saver	Evaluate after use	Jingdong prefecture level city in Shandong
Fortune Shop	Green Intelligent Power Saver	Still on trial.	Jingdong prefecture level city in Shandong
Fortune Shop	Green Intelligent Power Saver	ineffective	Jingdong prefecture level city in Shandong
Fortune Shop	Green Intelligent Power Saver	Satisfaction Shopping	Jingdong prefecture level city in Shandong
Fortune Shop	Green Intelligent Power Saver	Lies.	Jingdong prefecture level city in Shandong
Fortune Shop	Green Intelligent Power Saver	It's a real power saver.	Jingdong prefecture level city in Shandong
Fortune Shop	Green Intelligent Power Saver	in use	Jingdong prefecture level city in Shandong
Fortune Shop	Green Intelligent Power Saver	I don't know yet.	Jingdong prefecture level city in Shandong
Fortune Shop	Green Intelligent Power Saver	not care about anything	Jingdong prefecture level city in Shandong
Fortune Shop	Green Intelligent Power Saver	It doesn't seem to have much effect	Jingdong prefecture level city in Shandong
Fortune Shop	Green Intelligent Power Saver	Let the merchant see for himself, does the plug positive and negative terminals look like this?	Jingdong prefecture level city in Shandong
Fortune Shop	Green Intelligent Power Saver	It doesn't work well, the electricity bill is less before using it, but more after using it, contacting customer service, the service is still not good, and returning it is slow	Jingdong prefecture level city in Shandong
Fortune Shop	Green Intelligent Power Saver	With the advertisement product does not match, above the effective negative, power saving rate of two rows of words it, the product looks very thin, with 3 days to see the meter number is still with the previous no difference, which is obviously fraudulent behavior!	Jingdong prefecture level city in Shandong
Fortune Shop	Green Intelligent Power Saver	Doesn't save any power at all. Not much use.	Jingdong prefecture level city in Shandong
Fortune Shop	Green Intelligent Power Saver	The plugs are all loose, they're all the same length and they don't work at all	Jingdong prefecture level city in Shandong
Fortune Shop	Green Intelligent Power Saver	It's bad 👎 it's not effective at all, you'd be crazy to sell this garbage on the internet.	Jingdong prefecture level city in Shandong
Fortune Shop	Green Intelligent Power Saver	It doesn't save any power at all, so I hope the old timers don't fall for this scam again	Jingdong prefecture level city in Shandong
Fortune Shop	Green Intelligent Power Saver	I've been using it for over a month now and haven't noticed any power savings.	Jingdong prefecture level city in Shandong
Fortune Shop	Green Intelligent Power Saver	It doesn't work at all. The electricity bill is higher when you use it.	Jingdong prefecture level city in Shandong
Fortune Shop	Green Intelligent Power Saver	It doesn't save any power, it's still as bad as ever.	Jingdong prefecture level city in Shandong
Fortune Shop	Green Intelligent Power Saver	Last night before leaving work to look at the table, and then plugged into the node device, this morning and then look at the table, than the original save a few degrees of electricity, very good to have a power-saving effect, easy to use, satisfied with the shopping praise.	Jingdong prefecture level city in Shandong
Fortune Shop	Green Intelligent Power Saver	The product is real and effective, the electricity bill is reduced, really good, thanks to the merchants have so many good products, the product is real and effective, the electricity bill is reduced, want to save electricity friends quickly buy it, early use early to save money.	Jingdong prefecture level city in Shandong
Fortune Shop	Green Intelligent Power Saver	Saver saves time and money, baby appearance is beautiful, the quality of goods is good, and the same as the business description, good service attitude, packaging is complete, logistics is very fast, praise to	Jingdong prefecture level city in Shandong
Fortune Shop	Green Intelligent Power Saver	The effect is good, summer, home electricity is relatively large, buy and use a few days, feel the effect is very good, originally ten dollars a day electricity bill, now eight dollars a day, quite good	Jingdong prefecture level city in Shandong
Fortune Shop	Green Intelligent Power Saver	Indeed can save electricity and money, the first time I know this or from a friend's home to see, but also do not believe it, and now they use it to know that there is this good thing.	Jingdong prefecture level city in Shandong
Fortune Shop	Green Intelligent Power Saver	I have received the Smart Power Saver. Perfectly packaged. Fast delivery. Boss customer service attitude is also very good. It's worth buying. I plugged it in and tried it and it really saves more power than the original. I'll buy it again next time.	Jingdong prefecture level city in Shandong
Fortune Shop	Green Intelligent Power Saver	It's nice to save a dozen bucks a night on two air conditioners that used to take 30 or 40 degrees a night, but now it's only 10 or so degrees a night	Jingdong prefecture level city in Shandong
Fortune Shop	Green Intelligent Power Saver	Product no manufacturer's name no product qualification certificate, try two days to see the effect of	Jingdong prefecture level city in Shandong
Fortune Shop	Green Intelligent Power Saver	I've been using it for over a month and have saved over $80, so I've repurchased a few more back to save together	Jingdong prefecture level city in Shandong
Fortune Shop	Green Intelligent Power Saver	I haven't used it yet and will comment when I do, but it's well made.	Jingdong prefecture level city in Shandong
Fortune Shop	Green Intelligent Power Saver	It's quite energy efficient. It's really a boon for the home.	Jingdong prefecture level city in Shandong
Fortune Shop	Green Intelligent Power Saver	Energy-saving socket received a month, after using it for a month proved that it is indeed energy-saving, electricity bill saving, will buy it again, thank you!	Jingdong prefecture level city in Shandong
Fortune Shop	Green Intelligent Power Saver	There was a similar product many years ago. Now technology has evolved, buy one to try, according to the instructions in the box, it takes one or two dozen days to show results. Check your electric bill and see what happens.	Jingdong prefecture level city in Shandong
Fortune Shop	Green Intelligent Power Saver	Jingdong logistics is really fast, the same day the order arrived on the same day, more convenient than the supermarket, and a wide range of choices, the	Jingdong prefecture level city in Shandong
Fortune Shop	Green Intelligent Power Saver	I've been using this for a month or so and I can't see any results, it really is a scam as they say.	Jingdong prefecture level city in Shandong
Fortune Shop	Green Intelligent Power Saver	Very good use, a month can save a few degrees of power loss of electricity, introduced to the relatives to buy back to try.	Jingdong prefecture level city in Shandong
Fortune Shop	Green Intelligent Power Saver	It's really powerful! With this air conditioner can be turned on at any time without the pressure of paying the electricity bill! It's good for the people!	Jingdong prefecture level city in Shandong
Fortune Shop	Green Intelligent Power Saver	This business is a big ** products are not qualified, with a month of electricity more than one hundred percent of thirty, the purchase agreed to return the goods but do not receive a refund after receiving the goods, looking for the official side does not help, the Jingdong is a big **, I have never shopped in	Jingdong prefecture level city in Shandong
Fortune Shop	Green Intelligent Power Saver	It's good. I tried it. It saves power.	Jingdong prefecture level city in Shandong
Fortune Shop	Green Intelligent Power Saver	Nothing**, it seems like there's more	Jingdong prefecture level city in Shandong
Fortune Shop	Green Intelligent Power Saver	Power saving device received, well packaged, good quality, fast logistics	Jingdong prefecture level city in Shandong
Fortune Shop	Green Intelligent Power Saver	The service is okay, is that when I bought five, the desirable pieces are three, and then wait a few days and send me two replacement, I do not know if it is a mistake of the shipper, if I was not in the courier there to see, go home to say no, the	Jingdong prefecture level city in Shandong
Fortune Shop	Green Intelligent Power Saver	This thing will only cost me more if I use it, but before I used it, it was less than a ~ electricity a month, and after I used it, it was more than two hundred kWh a month.	Jingdong prefecture level city in Shandong
Fortune Shop	Green Intelligent Power Saver	I just used it. I don't know how it is.	Jingdong prefecture level city in Shandong
Fortune Shop	Green Intelligent Power Saver	I've received the product and I'm using it, so I'll see how it goes in a month!	Jingdong prefecture level city in Shandong
Fortune Shop	Green Intelligent Power Saver	The goods have just received the effect do not know how, please look forward to the results.	Jingdong prefecture level city in Shandong
Fortune Shop	Green Intelligent Power Saver	Power saving device, the material is very good practical and durable, the size is suitable, packaging is very good, the price is cheap, logistics is very fast!	Jingdong prefecture level city in Shandong
Fortune Shop	Green Intelligent Power Saver	Very nice, will comment in detail after using it for a while.	Jingdong prefecture level city in Shandong

Fortune Shop	Green Intelligent Power Saver	Good stuff to trust and support	Jingdong prefecture level city in Shandong
Fortune Shop	Green Intelligent Power Saver	I'll comment after I use it, doubt if it really saves power no	Jingdong prefecture level city in Shandong
Fortune Shop	Green Intelligent Power Saver	It's good quality and works well!	Jingdong prefecture level city in Shandong
Fortune Shop	Green Intelligent Power Saver	hahaha just right hahahahahaha hiccup hahahaha	Jingdong prefecture level city in Shandong
Fortune Shop	Green Intelligent Power Saver	The real thing matches the picture and looks great!	Jingdong prefecture level city in Shandong
Fortune Shop	Green Intelligent Power Saver	Plugged it in and we'll see when we pay the bills at the end of the year!	Jingdong prefecture level city in Shandong
Fortune Shop	Green Intelligent Power Saver	False advertising. It doesn't work.	Jingdong prefecture level city in Shandong
Fortune Shop	Green Intelligent Power Saver	Goods received, good effect	Jingdong prefecture level city in Shandong
Fortune Shop	Green Intelligent Power Saver	It looks okay, we'll see if it saves electricity when the time comes	Jingdong prefecture level city in Shandong
Fortune Shop	Green Intelligent Power Saver	Logistics is very fast quality is very good, the price is very affordable, accessories are also complete, installed, satisfied with good use	Jingdong prefecture level city in Shandong
Fortune Shop	Green Intelligent Power Saver	I've received the power saver. I'm looking forward to the savings.	Jingdong prefecture level city in Shandong
Fortune Shop	Green Intelligent Power Saver	Received, have not yet used, after use and then evaluate	Jingdong prefecture level city in Shandong
Fortune Shop	Green Intelligent Power Saver	I received the item, but I don't know if it saves power or not.	Jingdong prefecture level city in Shandong
Fortune Shop	Green Intelligent Power Saver	On Trial	Jingdong prefecture level city in Shandong
Fortune Shop	Green Intelligent Power Saver	Use it and see how it goes.	Jingdong prefecture level city in Shandong
Fortune Shop	Green Intelligent Power Saver	Quality is very good, no odor, fine workmanship, grain color are very like, satisfied!	Jingdong prefecture level city in Shandong
Fortune Shop	Green Intelligent Power Saver	I'll give you a good review. I don't know if it'll work.	Jingdong prefecture level city in Shandong
Fortune Shop	Green Intelligent Power Saver	The logistics is very fast and the package is intact. Good use of affordable. The effect is very good, is genuine	Jingdong prefecture level city in Shandong
Fortune Shop	Green Intelligent Power Saver	Nothing happens when you plug it in.	Jingdong prefecture level city in Shandong
Fortune Shop	Green Intelligent Power Saver	I don't know if it's as good as it sounds.	Jingdong prefecture level city in Shandong
Fortune Shop	Green Intelligent Power Saver	The quality of the stuff is very good, exactly the same as the seller's description, very satisfied!	Jingdong prefecture level city in Shandong
Fortune Shop	Green Intelligent Power Saver	The saver works great, it really works, it's affordable and worth buying.	Jingdong prefecture level city in Shandong
Fortune Shop	Green Intelligent Power Saver	Just used it for a couple days. I don't know yet.	Jingdong prefecture level city in Shandong
Fortune Shop	Green Intelligent Power Saver	The quality is quite good, cheaper than the physical store, good, next time will come again!	Jingdong prefecture level city in Shandong
Fortune Shop	Green Intelligent Power Saver	Good quality, cheap and affordable worth recommending to buy!	Jingdong prefecture level city in Shandong
Fortune Shop	Green Intelligent Power Saver	Very good quality, well made, works well! Satisfied	Jingdong prefecture level city in Shandong
Fortune Shop	Green Intelligent Power Saver	The logistics is very fast quality is very good very pleasant a shopping	Jingdong prefecture level city in Shandong
Fortune Shop	Green Intelligent Power Saver	Use it and then come back to evaluate!	Jingdong prefecture level city in Shandong
Fortune Shop	Green Intelligent Power Saver	# It's no use lying at all #	Jingdong prefecture level city in Shandong
Fortune Shop	Green Intelligent Power Saver	I can't get a voltage drop.	Jingdong prefecture level city in Shandong
Fortune Shop	Green Intelligent Power Saver	Evaluate after use	Jingdong prefecture level city in Shandong
Fortune Shop	Green Intelligent Power Saver	Still on trial.	Jingdong prefecture level city in Shandong
Fortune Shop	Green Intelligent Power Saver	ineffective	Jingdong prefecture level city in Shandong
Fortune Shop	Green Intelligent Power Saver	Satisfaction Shopping	Jingdong prefecture level city in Shandong
Fortune Shop	Green Intelligent Power Saver	Lies.	Jingdong prefecture level city in Shandong
Fortune Shop	Green Intelligent Power Saver	It's a real power saver.	Jingdong prefecture level city in Shandong
Fortune Shop	Green Intelligent Power Saver	in use	Jingdong prefecture level city in Shandong
Fortune Shop	Green Intelligent Power Saver	I don't know yet.	Jingdong prefecture level city in Shandong
Fortune Shop	Green Intelligent Power Saver	not care about anything	Jingdong prefecture level city in Shandong
Fortune Shop	Green Intelligent Power Saver	It doesn't seem to have much effect	Jingdong prefecture level city in Shandong
Fortune Shop	Green Intelligent Power Saver	Let the merchant see for himself, does the plug positive and negative terminals look like this?	Jingdong prefecture level city in Shandong
Fortune Shop	Green Intelligent Power Saver	It doesn't work well, the electricity bill is less before using it, but more after using it, contacting customer service, the service is still not good, and returning it is slow	Jingdong prefecture level city in Shandong
Fortune Shop	Green Intelligent Power Saver	With the advertisement product does not match, above the effective negative, power saving rate of two rows of words it, the product looks very thin, with 3 days to see the meter number is still with the previous no difference, which is obviously fraudulent behavior!	Jingdong prefecture level city in Shandong
Fortune Shop	Green Intelligent Power Saver	Doesn't save any power at all. Not much use.	Jingdong prefecture level city in Shandong
Fortune Shop	Green Intelligent Power Saver	The plugs are all loose, they're all the same length and they don't work at all	Jingdong prefecture level city in Shandong
Fortune Shop	Green Intelligent Power Saver	It's bad 👎 it's not effective at all, you'd be crazy to sell this garbage on the internet.	Jingdong prefecture level city in Shandong
Fortune Shop	Green Intelligent Power Saver	It doesn't save any power at all, so I hope the old timers don't fall for this scam again	Jingdong prefecture level city in Shandong
Fortune Shop	Green Intelligent Power Saver	I've been using it for over a month now and haven't noticed any power savings.	Jingdong prefecture level city in Shandong
Fortune Shop	Green Intelligent Power Saver	It doesn't work at all. The electricity bill is higher when you use it.	Jingdong prefecture level city in Shandong
Fortune Shop	Green Intelligent Power Saver	It doesn't save any power, it's still as bad as ever.	Jingdong prefecture level city in Shandong

Fortune Shop	Green Intelligent Power Saver	Last night before leaving work to look at the table, and then plugged into the node device, this morning and then look at the table, than the original save a few degrees of electricity, very good to have a power-saving effect, easy to use, satisfied with the shopping praise.	Jingdong prefecture level city in Shandong
Fortune Shop	Green Intelligent Power Saver	The product is real and effective, the electricity bill is reduced, really good, thanks to the merchants have so many good products, the product is real and effective, the electricity bill is reduced, want to save electricity friends quickly buy it, early use early to save money.	Jingdong prefecture level city in Shandong
Fortune Shop	Green Intelligent Power Saver	Saver saves time and money, baby appearance is beautiful, the quality of goods is good, and the same as the business description, good service attitude, packaging is complete, logistics is very fast, praise to	Jingdong prefecture level city in Shandong
Fortune Shop	Green Intelligent Power Saver	The effect is good, summer, home electricity is relatively large, buy and use a few days, feel the effect is very good, originally ten dollars a day electricity bill, now eight dollars a day, quite good	Jingdong prefecture level city in Shandong
Fortune Shop	Green Intelligent Power Saver	Indeed can save electricity and money, the first time I know this or from a friend's home to see, but also do not believe it, and now they use it to know that there is this good thing.	Jingdong prefecture level city in Shandong
Fortune Shop	Green Intelligent Power Saver	I have received the Smart Power Saver. Perfectly packaged. Fast delivery. Boss customer service attitude is also very good. It's worth buying. I plugged it in and tried it and it really saves more power than the original. I'll buy it again next time.	Jingdong prefecture level city in Shandong
Fortune Shop	Green Intelligent Power Saver	It's nice to save a dozen bucks a night on two air conditioners that used to take 30 or 40 degrees a night, but now it's only 10 or so degrees a night	Jingdong prefecture level city in Shandong
Fortune Shop	Green Intelligent Power Saver	Product no manufacturer's name no product qualification certificate, try two days to see the effect of	Jingdong prefecture level city in Shandong
Fortune Shop	Green Intelligent Power Saver	I've been using it for over a month and have saved over $80, so I've repurchased a few more back to save together	Jingdong prefecture level city in Shandong
Fortune Shop	Green Intelligent Power Saver	I haven't used it yet and will comment when I do, but it's well made.	Jingdong prefecture level city in Shandong
Fortune Shop	Green Intelligent Power Saver	It's quite energy efficient. It's really a boon for the home.	Jingdong prefecture level city in Shandong
Fortune Shop	Green Intelligent Power Saver	Energy-saving socket received a month, after using it for a month proved that it is indeed energy-saving, electricity bill saving, will buy it again, thank you!	Jingdong prefecture level city in Shandong
Fortune Shop	Green Intelligent Power Saver	There was a similar product many years ago. Now technology has evolved, buy one to try, according to the instructions in the box, it takes one or two dozen days to show results. Check your electric bill and see what happens.	Jingdong prefecture level city in Shandong
Fortune Shop	Green Intelligent Power Saver	Jingdong logistics is really fast, the same day the order arrived on the same day, more convenient than the supermarket, and a wide range of choices, the	Jingdong prefecture level city in Shandong
Fortune Shop	Green Intelligent Power Saver	I've been using this for a month or so and I can't see any results, it really is a scam as they say.	Jingdong prefecture level city in Shandong
Fortune Shop	Green Intelligent Power Saver	Very good use, a month can save a few degrees of power loss of electricity, introduced to the relatives to buy back to try.	Jingdong prefecture level city in Shandong
Fortune Shop	Green Intelligent Power Saver	It's really powerful! With this air conditioner can be turned on at any time without the pressure of paying the electricity bill! It's good for the people!	Jingdong prefecture level city in Shandong
Fortune Shop	Green Intelligent Power Saver	This business is a big ** products are not qualified, with a month of electricity more than one hundred percent of thirty, the purchase agreed to return the goods but do not receive a refund after receiving the goods, looking for the official side does not help, the Jingdong is a big **, I have never shopped in	Jingdong prefecture level city in Shandong
Fortune Shop	Green Intelligent Power Saver	It's good. I tried it. It saves power.	Jingdong prefecture level city in Shandong
Fortune Shop	Green Intelligent Power Saver	Nothing**, it seems like there's more	Jingdong prefecture level city in Shandong
Fortune Shop	Green Intelligent Power Saver	Power saving device received, well packaged, good quality, fast logistics	Jingdong prefecture level city in Shandong
Fortune Shop	Green Intelligent Power Saver	The service is okay, is that when I bought five, the desirable pieces are three, and then wait a few days and send me two replacement, I do not know if it is a mistake of the shipper, if I was not in the courier there to see, go home to say no, the	Jingdong prefecture level city in Shandong
Fortune Shop	Green Intelligent Power Saver	This thing will only cost me more if I use it, but before I used it, it was less than a ~ electricity a month, and after I used it, it was more than two hundred kWh a month.	Jingdong prefecture level city in Shandong
Fortune Shop	Green Intelligent Power Saver	I just used it. I don't know how it is.	Jingdong prefecture level city in Shandong
Fortune Shop	Green Intelligent Power Saver	I've received the product and I'm using it, so I'll see how it goes in a month!	Jingdong prefecture level city in Shandong
Fortune Shop	Green Intelligent Power Saver	The goods have just received the effect do not know how, please look forward to the results.	Jingdong prefecture level city in Shandong
Fortune Shop	Green Intelligent Power Saver	Power saving device, the material is very good practical and durable, the size is suitable, packaging is very good, the price is cheap, logistics is very fast!	Jingdong prefecture level city in Shandong
Fortune Shop	Green Intelligent Power Saver	Very nice, will comment in detail after using it for a while.	Jingdong prefecture level city in Shandong
Fortune Shop	Green Intelligent Power Saver	Good stuff to trust and support	Jingdong prefecture level city in Shandong
Fortune Shop	Green Intelligent Power Saver	I'll comment after I use it, doubt if it really saves power no	Jingdong prefecture level city in Shandong
Fortune Shop	Green Intelligent Power Saver	It's good quality and works well!	Jingdong prefecture level city in Shandong
Fortune Shop	Green Intelligent Power Saver	hahaha just right hahahahahaha hiccup hahahaha	Jingdong prefecture level city in Shandong
Fortune Shop	Green Intelligent Power Saver	The real thing matches the picture and looks great!	Jingdong prefecture level city in Shandong
Fortune Shop	Green Intelligent Power Saver	Plugged it in and we'll see when we pay the bills at the end of the year!	Jingdong prefecture level city in Shandong
Fortune Shop	Green Intelligent Power Saver	False advertising. It doesn't work.	Jingdong prefecture level city in Shandong
Fortune Shop	Green Intelligent Power Saver	Goods received, good effect	Jingdong prefecture level city in Shandong
Fortune Shop	Green Intelligent Power Saver	It looks okay, we'll see if it saves electricity when the time comes	Jingdong prefecture level city in Shandong
Fortune Shop	Green Intelligent Power Saver	Logistics is very fast quality is very good, the price is very affordable, accessories are also complete, installed, satisfied with good use	Jingdong prefecture level city in Shandong
Fortune Shop	Green Intelligent Power Saver	I've received the power saver. I'm looking forward to the savings.	Jingdong prefecture level city in Shandong
Fortune Shop	Green Intelligent Power Saver	Received, have not yet used, after use and then evaluate	Jingdong prefecture level city in Shandong
Fortune Shop	Green Intelligent Power Saver	I received the item, but I don't know if it saves power or not.	Jingdong prefecture level city in Shandong
Fortune Shop	Green Intelligent Power Saver	On Trial	Jingdong prefecture level city in Shandong
Fortune Shop	Green Intelligent Power Saver	Use it and see how it goes.	Jingdong prefecture level city in Shandong
Fortune Shop	Green Intelligent Power Saver	Quality is very good, no odor, fine workmanship, grain color are very like, satisfied!	Jingdong prefecture level city in Shandong
Fortune Shop	Green Intelligent Power Saver	I'll give you a good review. I don't know if it'll work.	Jingdong prefecture level city in Shandong
Fortune Shop	Green Intelligent Power Saver	The logistics is very fast and the package is intact. Good use of affordable. The effect is very good, is genuine	Jingdong prefecture level city in Shandong
Fortune Shop	Green Intelligent Power Saver	Nothing happens when you plug it in.	Jingdong prefecture level city in Shandong
Fortune Shop	Green Intelligent Power Saver	I don't know if it's as good as it sounds.	Jingdong prefecture level city in Shandong

Fortune Shop	Green Intelligent Power Saver	The quality of the stuff is very good, exactly the same as the seller's description, very satisfied!	Jingdong prefecture level city in Shandong
Fortune Shop	Green Intelligent Power Saver	The saver works great, it really works, it's affordable and worth buying.	Jingdong prefecture level city in Shandong
Fortune Shop	Green Intelligent Power Saver	Just used it for a couple days. I don't know yet.	Jingdong prefecture level city in Shandong
Fortune Shop	Green Intelligent Power Saver	The quality is quite good, cheaper than the physical store, good, next time will come again!	Jingdong prefecture level city in Shandong
Fortune Shop	Green Intelligent Power Saver	Good quality, cheap and affordable worth recommending to buy!	Jingdong prefecture level city in Shandong
Fortune Shop	Green Intelligent Power Saver	Very good quality, well made, works well! Satisfied	Jingdong prefecture level city in Shandong
Fortune Shop	Green Intelligent Power Saver	The logistics is very fast quality is very good very pleasant a shopping	Jingdong prefecture level city in Shandong
Fortune Shop	Green Intelligent Power Saver	Use it and then come back to evaluate!	Jingdong prefecture level city in Shandong
Fortune Shop	Green Intelligent Power Saver	# It's no use lying at all #	Jingdong prefecture level city in Shandong
Fortune Shop	Green Intelligent Power Saver	I can't get a voltage drop.	Jingdong prefecture level city in Shandong
Fortune Shop	Green Intelligent Power Saver	Evaluate after use	Jingdong prefecture level city in Shandong
Fortune Shop	Green Intelligent Power Saver	Still on trial.	Jingdong prefecture level city in Shandong
Fortune Shop	Green Intelligent Power Saver	ineffective	Jingdong prefecture level city in Shandong
Fortune Shop	Green Intelligent Power Saver	Satisfaction Shopping	Jingdong prefecture level city in Shandong
Fortune Shop	Green Intelligent Power Saver	Lies.	Jingdong prefecture level city in Shandong
Fortune Shop	Green Intelligent Power Saver	It's a real power saver.	Jingdong prefecture level city in Shandong
Fortune Shop	Green Intelligent Power Saver	in use	Jingdong prefecture level city in Shandong
Fortune Shop	Green Intelligent Power Saver	I don't know yet.	Jingdong prefecture level city in Shandong
Fortune Shop	Green Intelligent Power Saver	not care about anything	Jingdong prefecture level city in Shandong
Fortune Shop	Green Intelligent Power Saver	It doesn't seem to have much effect	Jingdong prefecture level city in Shandong
Fortune Shop	Green Intelligent Power Saver	Let the merchant see for himself, does the plug positive and negative terminals look like this?	Jingdong prefecture level city in Shandong
Fortune Shop	Green Intelligent Power Saver	It doesn't work well, the electricity bill is less before using it, but more after using it, contacting customer service, the service is still not good, and returning it is slow	Jingdong prefecture level city in Shandong
Fortune Shop	Green Intelligent Power Saver	With the advertisement product does not match, above the effective negative, power saving rate of two rows of words it, the product looks very thin, with 3 days to see the meter number is still with the previous no difference, which is obviously fraudulent behavior!	Jingdong prefecture level city in Shandong
Fortune Shop	Green Intelligent Power Saver	Doesn't save any power at all. Not much use.	Jingdong prefecture level city in Shandong
Fortune Shop	Green Intelligent Power Saver	The plugs are all loose, they're all the same length and they don't work at all	Jingdong prefecture level city in Shandong
Fortune Shop	Green Intelligent Power Saver	It's bad 👎 it's not effective at all, you'd be crazy to sell this garbage on the internet.	Jingdong prefecture level city in Shandong
Fortune Shop	Green Intelligent Power Saver	It doesn't save any power at all, so I hope the old timers don't fall for this scam again	Jingdong prefecture level city in Shandong
Fortune Shop	Green Intelligent Power Saver	I've been using it for over a month now and haven't noticed any power savings.	Jingdong prefecture level city in Shandong
Fortune Shop	Green Intelligent Power Saver	It doesn't work at all. The electricity bill is higher when you use it.	Jingdong prefecture level city in Shandong
Fortune Shop	Green Intelligent Power Saver	It doesn't save any power, it's still as bad as ever.	Jingdong prefecture level city in Shandong
Fortune Shop	Green Intelligent Power Saver	Last night before leaving work to look at the table, and then plugged into the node device, this morning and then look at the table, the original save a few degrees of electricity, very good to have a power-saving effect, easy to use, satisfied with the shopping praise.	Jingdong prefecture level city in Shandong
Fortune Shop	Green Intelligent Power Saver	The product is real and effective, the electricity bill is reduced, really good, thanks to the merchants have so many good products, the product is real and effective, the electricity bill is reduced, want to save electricity friends quickly buy it, early use early to save money.	Jingdong prefecture level city in Shandong
Fortune Shop	Green Intelligent Power Saver	Saver saves time and money, baby appearance is beautiful, the quality of goods is good, and the same as the business description, good service attitude, packaging is complete, logistics is very fast, praise to	Jingdong prefecture level city in Shandong
Fortune Shop	Green Intelligent Power Saver	The effect is good, summer, home electricity is relatively large, buy and use a few days, feel the effect is very good, originally ten dollars a day electricity bill, now eight dollars a day, quite good	Jingdong prefecture level city in Shandong
Fortune Shop	Green Intelligent Power Saver	Indeed can save electricity and money, the first time I know this or from a friend's home to see, but also do not believe it, and now they use it to know that there is this good thing.	Jingdong prefecture level city in Shandong
Fortune Shop	Green Intelligent Power Saver	I have received the Smart Power Saver. Perfectly packaged. Fast delivery. Boss customer service attitude is also very good. It's worth buying. I plugged it in and tried it and it really saves more power than the original. I'll buy it again next time.	Jingdong prefecture level city in Shandong
Fortune Shop	Green Intelligent Power Saver	It's nice to save a dozen bucks a night on two air conditioners that used to take 30 or 40 degrees a night, but now it's only 10 or so degrees a night	Jingdong prefecture level city in Shandong
Fortune Shop	Green Intelligent Power Saver	Product no manufacturer's name no product qualification certificate, try two days to see the effect of	Jingdong prefecture level city in Shandong
Fortune Shop	Green Intelligent Power Saver	I've been using it for over a month and have saved over $80, so I've repurchased a few more back to save together	Jingdong prefecture level city in Shandong
Fortune Shop	Green Intelligent Power Saver	I haven't used it yet and will comment when I do, but it's well made.	Jingdong prefecture level city in Shandong
Fortune Shop	Green Intelligent Power Saver	It's quite energy efficient. It's really a boon for the home.	Jingdong prefecture level city in Shandong
Fortune Shop	Green Intelligent Power Saver	Energy-saving socket received a month, after using it for a month proved that it is indeed energy-saving, electricity bill saving, will buy it again, thank you!	Jingdong prefecture level city in Shandong
Fortune Shop	Green Intelligent Power Saver	There was a similar product many years ago. Now technology has evolved, buy one to try, according to the instructions in the box, it takes one or two dozen days to show results. Check your electric bill and see what happens.	Jingdong prefecture level city in Shandong
Fortune Shop	Green Intelligent Power Saver	Jingdong logistics is really fast, the same day the order arrived on the same day, more convenient than the supermarket, and a wide range of choices, the	Jingdong prefecture level city in Shandong
Fortune Shop	Green Intelligent Power Saver	I've been using this for a month or so and I can't see any results, it really is a scam as they say.	Jingdong prefecture level city in Shandong
Fortune Shop	Green Intelligent Power Saver	Very good use, a month can save a few degrees of power loss of electricity, introduced to the relatives to buy back to try.	Jingdong prefecture level city in Shandong
Fortune Shop	Green Intelligent Power Saver	It's really powerful! With this air conditioner can be turned on at any time without the pressure of paying the electricity bill! It's good for the people!	Jingdong prefecture level city in Shandong
Fortune Shop	Green Intelligent Power Saver	This business is a big ** products are not qualified, with a month of electricity more than one hundred percent of thirty, the purchase agreed to return the goods but do not receive a refund after receiving the goods, looking for the official side does not help, the Jingdong is a big **, I have never shopped in	Jingdong prefecture level city in Shandong

Fortune Shop	Green Intelligent Power Saver	It's good. I tried it. It saves power.	Jingdong prefecture level city in Shandong
Fortune Shop	Green Intelligent Power Saver	Nothing**, it seems like there's more	Jingdong prefecture level city in Shandong
Fortune Shop	Green Intelligent Power Saver	Power saving device received, well packaged, good quality, fast logistics	Jingdong prefecture level city in Shandong
Fortune Shop	Green Intelligent Power Saver	The service is okay, is that when I bought five, the desirable pieces are three, and then wait a few days and send me two replacement, I do not know if it is a mistake of the shipper, if I was not in the courier there to see, go home to say no, the	Jingdong prefecture level city in Shandong
Fortune Shop	Green Intelligent Power Saver	This thing will only cost me more if I use it, but before I used it, it was less than a ~ electricity a month, and after I used it, it was more than two hundred kWh a month.	Jingdong prefecture level city in Shandong
Fortune Shop	Green Intelligent Power Saver	I just used it. I don't know how it is.	Jingdong prefecture level city in Shandong
Fortune Shop	Green Intelligent Power Saver	I've received the product and I'm using it, so I'll see how it goes in a month!	Jingdong prefecture level city in Shandong
Fortune Shop	Green Intelligent Power Saver	The goods have just received the effect do not know how, please look forward to the results.	Jingdong prefecture level city in Shandong
Fortune Shop	Green Intelligent Power Saver	Power saving device, the material is very good practical and durable, the size is suitable, packaging is very good, the price is cheap, logistics is very fast!	Jingdong prefecture level city in Shandong
Fortune Shop	Green Intelligent Power Saver	Very nice, will comment in detail after using it for a while.	Jingdong prefecture level city in Shandong
Fortune Shop	Green Intelligent Power Saver	Good stuff to trust and support	Jingdong prefecture level city in Shandong
Fortune Shop	Green Intelligent Power Saver	I'll comment after I use it, doubt if it really saves power no	Jingdong prefecture level city in Shandong
Fortune Shop	Green Intelligent Power Saver	It's good quality and works well!	Jingdong prefecture level city in Shandong
Fortune Shop	Green Intelligent Power Saver	hahaha just right hahahahahaha hiccup hahahaha	Jingdong prefecture level city in Shandong
Fortune Shop	Green Intelligent Power Saver	The real thing matches the picture and looks great!	Jingdong prefecture level city in Shandong
Fortune Shop	Green Intelligent Power Saver	Plugged it in and we'll see when we pay the bills at the end of the year!	Jingdong prefecture level city in Shandong
Fortune Shop	Green Intelligent Power Saver	False advertising. It doesn't work.	Jingdong prefecture level city in Shandong
Fortune Shop	Green Intelligent Power Saver	Goods received, good effect	Jingdong prefecture level city in Shandong
Fortune Shop	Green Intelligent Power Saver	It looks okay, we'll see if it saves electricity when the time comes	Jingdong prefecture level city in Shandong
Fortune Shop	Green Intelligent Power Saver	Logistics is very fast quality is very good, the price is very affordable, accessories are also complete, installed, satisfied with good use	Jingdong prefecture level city in Shandong
Fortune Shop	Green Intelligent Power Saver	I've received the power saver. I'm looking forward to the savings.	Jingdong prefecture level city in Shandong
Fortune Shop	Green Intelligent Power Saver	Received, have not yet used, after use and then evaluate	Jingdong prefecture level city in Shandong
Fortune Shop	Green Intelligent Power Saver	I received the item, but I don't know if it saves power or not.	Jingdong prefecture level city in Shandong
Fortune Shop	Green Intelligent Power Saver	On Trial	Jingdong prefecture level city in Shandong
Fortune Shop	Green Intelligent Power Saver	Use it and see how it goes.	Jingdong prefecture level city in Shandong
Fortune Shop	Green Intelligent Power Saver	Quality is very good, no odor, fine workmanship, grain color are very like, satisfied!	Jingdong prefecture level city in Shandong
Fortune Shop	Green Intelligent Power Saver	I'll give you a good review. I don't know if it'll work.	Jingdong prefecture level city in Shandong
Fortune Shop	Green Intelligent Power Saver	The logistics is very fast and the package is intact. Good use of affordable. The effect is very good, is genuine	Jingdong prefecture level city in Shandong
Fortune Shop	Green Intelligent Power Saver	Nothing happens when you plug it in.	Jingdong prefecture level city in Shandong
Fortune Shop	Green Intelligent Power Saver	I don't know if it's as good as it sounds.	Jingdong prefecture level city in Shandong
Fortune Shop	Green Intelligent Power Saver	The quality of the stuff is very good, exactly the same as the seller's description, very satisfied!	Jingdong prefecture level city in Shandong
Fortune Shop	Green Intelligent Power Saver	The saver works great, it really works, it's affordable and worth buying.	Jingdong prefecture level city in Shandong
Fortune Shop	Green Intelligent Power Saver	Just used it for a couple days. I don't know yet.	Jingdong prefecture level city in Shandong
Fortune Shop	Green Intelligent Power Saver	The quality is quite good, cheaper than the physical store, good, next time will come again!	Jingdong prefecture level city in Shandong
Fortune Shop	Green Intelligent Power Saver	Good quality, cheap and affordable worth recommending to buy!	Jingdong prefecture level city in Shandong
Fortune Shop	Green Intelligent Power Saver	Very good quality, well made, works well! Satisfied	Jingdong prefecture level city in Shandong
Fortune Shop	Green Intelligent Power Saver	The logistics is very fast quality is very good very pleasant a shopping	Jingdong prefecture level city in Shandong
Fortune Shop	Green Intelligent Power Saver	Use it and then come back to evaluate!	Jingdong prefecture level city in Shandong
Fortune Shop	Green Intelligent Power Saver	# It's no use lying at all #	Jingdong prefecture level city in Shandong
Fortune Shop	Green Intelligent Power Saver	I can't get a voltage drop.	Jingdong prefecture level city in Shandong
Fortune Shop	Green Intelligent Power Saver	Evaluate after use	Jingdong prefecture level city in Shandong
Fortune Shop	Green Intelligent Power Saver	Still on trial.	Jingdong prefecture level city in Shandong
Fortune Shop	Green Intelligent Power Saver	ineffective	Jingdong prefecture level city in Shandong
Fortune Shop	Green Intelligent Power Saver	Satisfaction Shopping	Jingdong prefecture level city in Shandong
Fortune Shop	Green Intelligent Power Saver	Lies.	Jingdong prefecture level city in Shandong
Fortune Shop	Green Intelligent Power Saver	It's a real power saver.	Jingdong prefecture level city in Shandong
Fortune Shop	Green Intelligent Power Saver	in use	Jingdong prefecture level city in Shandong
Fortune Shop	Green Intelligent Power Saver	I don't know yet.	Jingdong prefecture level city in Shandong
Fortune Shop	Green Intelligent Power Saver	not care about anything	Jingdong prefecture level city in Shandong
Fortune Shop	Green Intelligent Power Saver	It doesn't seem to have much effect	Jingdong prefecture level city in Shandong
Fortune Shop	Green Intelligent Power Saver	Let the merchant see for himself, does the plug positive and negative terminals look like this?	Jingdong prefecture level city in Shandong

Fortune Shop	Green Intelligent Power Saver	It doesn't work well, the electricity bill is less before using it, but more after using it, contacting customer service, the service is still not good, and returning it is slow	Jingdong prefecture level city in Shandong
Fortune Shop	Green Intelligent Power Saver	With the advertisement product does not match, above the effective negative, power saving rate of two rows of words it, the product looks very thin, with 3 days to see the meter number is still with the previous no difference, which is obviously fraudulent behavior!	Jingdong prefecture level city in Shandong
Fortune Shop	Green Intelligent Power Saver	Doesn't save any power at all. Not much use.	Jingdong prefecture level city in Shandong
Fortune Shop	Green Intelligent Power Saver	The plugs are all loose, they're all the same length and they don't work at all	Jingdong prefecture level city in Shandong
Fortune Shop	Green Intelligent Power Saver	It's bad 👎 it's not effective at all, you'd be crazy to sell this garbage on the internet.	Jingdong prefecture level city in Shandong
Fortune Shop	Green Intelligent Power Saver	It doesn't save any power at all, so I hope the old timers don't fall for this scam again	Jingdong prefecture level city in Shandong
Fortune Shop	Green Intelligent Power Saver	I've been using it for over a month now and haven't noticed any power savings.	Jingdong prefecture level city in Shandong
Fortune Shop	Green Intelligent Power Saver	It doesn't work at all. The electricity bill is higher when you use it.	Jingdong prefecture level city in Shandong
Fortune Shop	Green Intelligent Power Saver	It doesn't save any power, it's still as bad as ever.	Jingdong prefecture level city in Shandong
Fortune Shop	Green Intelligent Power Saver	Last night before leaving work to look at the table, and then plugged into the node device, this morning and then look at the table, than the original save a few degrees of electricity, very good to have a power-saving effect, easy to use, satisfied with the shopping praise.	Jingdong prefecture level city in Shandong
Fortune Shop	Green Intelligent Power Saver	The product is real and effective, the electricity bill is reduced, really good, thanks to the merchants have so many good products, the product is real and effective, the electricity bill is reduced, want to save electricity friends quickly buy it, early use early to save money.	Jingdong prefecture level city in Shandong
Fortune Shop	Green Intelligent Power Saver	Saver saves time and money, baby appearance is beautiful, the quality of goods is good, and the same as the business description, good service attitude, packaging is complete, logistics is very fast, praise to	Jingdong prefecture level city in Shandong
Fortune Shop	Green Intelligent Power Saver	The effect is good, summer, home electricity is relatively large, buy and use a few days, feel the effect is very good, originally ten dollars a day electricity bill, now eight dollars a day, quite good	Jingdong prefecture level city in Shandong
Fortune Shop	Green Intelligent Power Saver	Indeed can save electricity and money, the first time I know this or from a friend's home to see, but also do not believe it, and now they use it to know that there is this good thing.	Jingdong prefecture level city in Shandong
Fortune Shop	Green Intelligent Power Saver	I have received the Smart Power Saver. Perfectly packaged. Fast delivery. Boss customer service attitude is also very good. It's worth buying. I plugged it in and tried it and it really saves more power than the original. I'll buy it again next time.	Jingdong prefecture level city in Shandong
Fortune Shop	Green Intelligent Power Saver	It's nice to save a dozen bucks a night on two air conditioners that used to take 30 or 40 degrees a night, but now it's only 10 or so degrees a night	Jingdong prefecture level city in Shandong
Fortune Shop	Green Intelligent Power Saver	Product no manufacturer's name no product qualification certificate, try two days to see the effect of	Jingdong prefecture level city in Shandong
Fortune Shop	Green Intelligent Power Saver	I've been using it for over a month and have saved over $80, so I've repurchased a few more back to save together	Jingdong prefecture level city in Shandong
Fortune Shop	Green Intelligent Power Saver	I haven't used it yet and will comment when I do, but it's well made.	Jingdong prefecture level city in Shandong
Fortune Shop	Green Intelligent Power Saver	It's quite energy efficient. It's really a boon for the home.	Jingdong prefecture level city in Shandong
Fortune Shop	Green Intelligent Power Saver	Energy-saving socket received a month, after using it for a month proved that it is indeed energy-saving, electricity bill saving, will buy it again, thank you!	Jingdong prefecture level city in Shandong
Fortune Shop	Green Intelligent Power Saver	There was a similar product many years ago. Now technology has evolved, buy one to try, according to the instructions in the box, it takes one or two dozen days to show results. Check your electric bill and see what happens.	Jingdong prefecture level city in Shandong
Fortune Shop	Green Intelligent Power Saver	Jingdong logistics is really fast, the same day the order arrived on the same day, more convenient than the supermarket, and a wide range of choices, the	Jingdong prefecture level city in Shandong
Fortune Shop	Green Intelligent Power Saver	I've been using this for a month or so and I can't see any results, it really is a scam as they say.	Jingdong prefecture level city in Shandong
Fortune Shop	Green Intelligent Power Saver	Very good use, a month can save a few degrees of power loss of electricity, introduced to the relatives to buy back to try.	Jingdong prefecture level city in Shandong
Fortune Shop	Green Intelligent Power Saver	It's really powerful! With this air conditioner can be turned on at any time without the pressure of paying the electricity bill! It's good for the people!	Jingdong prefecture level city in Shandong
Fortune Shop	Green Intelligent Power Saver	This business is a big ** products are not qualified, with a month of electricity more than one hundred percent of thirty, the purchase agreed to return the goods but do not receive a refund after receiving the goods, looking for the official side does not help, the Jingdong is a big **, I have never shopped in	Jingdong prefecture level city in Shandong
Fortune Shop	Green Intelligent Power Saver	It's good. I tried it. It saves power.	Jingdong prefecture level city in Shandong
Fortune Shop	Green Intelligent Power Saver	Nothing**, it seems like there's more	Jingdong prefecture level city in Shandong
Fortune Shop	Green Intelligent Power Saver	Power saving device received, well packaged, good quality, fast logistics	Jingdong prefecture level city in Shandong
Fortune Shop	Green Intelligent Power Saver	The service is okay, is that when I bought five, the desirable pieces are three, and then wait a few days and send me two replacement, I do not know if it is a mistake of the shipper, if I was not in the courier there to see, go home to say no, the	Jingdong prefecture level city in Shandong
Fortune Shop	Green Intelligent Power Saver	This thing will only cost me more if I use it, but before I used it, it was less than a ~ electricity a month, and after I used it, it was more than two hundred kWh a month.	Jingdong prefecture level city in Shandong
Fortune Shop	Green Intelligent Power Saver	I just used it. I don't know how it is.	Jingdong prefecture level city in Shandong
Fortune Shop	Green Intelligent Power Saver	I've received the product and I'm using it, so I'll see how it goes in a month!	Jingdong prefecture level city in Shandong
Fortune Shop	Green Intelligent Power Saver	The goods have just received the effect do not know how, please look forward to the results.	Jingdong prefecture level city in Shandong
Fortune Shop	Green Intelligent Power Saver	Power saving device, the material is very good practical and durable, the size is suitable, packaging is very good, the price is cheap, logistics is very fast!	Jingdong prefecture level city in Shandong
Fortune Shop	Green Intelligent Power Saver	Very nice, will comment in detail after using it for a while.	Jingdong prefecture level city in Shandong
Fortune Shop	Green Intelligent Power Saver	Good stuff to trust and support	Jingdong prefecture level city in Shandong
Fortune Shop	Green Intelligent Power Saver	I'll comment after I use it, doubt if it really saves power no	Jingdong prefecture level city in Shandong
Fortune Shop	Green Intelligent Power Saver	It's good quality and works well!	Jingdong prefecture level city in Shandong
Fortune Shop	Green Intelligent Power Saver	hahaha just right hahahahahaha hiccup hahahaha	Jingdong prefecture level city in Shandong
Fortune Shop	Green Intelligent Power Saver	The real thing matches the picture and looks great!	Jingdong prefecture level city in Shandong
Fortune Shop	Green Intelligent Power Saver	Plugged it in and we'll see when we pay the bills at the end of the year!	Jingdong prefecture level city in Shandong
Fortune Shop	Green Intelligent Power Saver	False advertising. It doesn't work.	Jingdong prefecture level city in Shandong
Fortune Shop	Green Intelligent Power Saver	Goods received, good effect	Jingdong prefecture level city in Shandong
Fortune Shop	Green Intelligent Power Saver	It looks okay, we'll see if it saves electricity when the time comes	Jingdong prefecture level city in Shandong
Fortune Shop	Green Intelligent Power Saver	Logistics is very fast quality is very good, the price is very affordable, accessories are also complete, installed, satisfied with good use	Jingdong prefecture level city in Shandong
Fortune Shop	Green Intelligent Power Saver	I've received the power saver. I'm looking forward to the savings.	Jingdong prefecture level city in Shandong

Fortune Shop	Green Intelligent Power Saver	Received, have not yet used, after use and then evaluate	Jingdong prefecture level city in Shandong
Fortune Shop	Green Intelligent Power Saver	I received the item, but I don't know if it saves power or not.	Jingdong prefecture level city in Shandong
Fortune Shop	Green Intelligent Power Saver	On Trial	Jingdong prefecture level city in Shandong
Fortune Shop	Green Intelligent Power Saver	Use it and see how it goes.	Jingdong prefecture level city in Shandong
Fortune Shop	Green Intelligent Power Saver	Quality is very good, no odor, fine workmanship, grain color are very like, satisfied!	Jingdong prefecture level city in Shandong
Fortune Shop	Green Intelligent Power Saver	I'll give you a good review. I don't know if it'll work.	Jingdong prefecture level city in Shandong
Fortune Shop	Green Intelligent Power Saver	The logistics is very fast and the package is intact. Good use of affordable. The effect is very good, is genuine	Jingdong prefecture level city in Shandong
Fortune Shop	Green Intelligent Power Saver	Nothing happens when you plug it in.	Jingdong prefecture level city in Shandong
Fortune Shop	Green Intelligent Power Saver	I don't know if it's as good as it sounds.	Jingdong prefecture level city in Shandong
Fortune Shop	Green Intelligent Power Saver	The quality of the stuff is very good, exactly the same as the seller's description, very satisfied!	Jingdong prefecture level city in Shandong
Fortune Shop	Green Intelligent Power Saver	The saver works great, it really works, it's affordable and worth buying.	Jingdong prefecture level city in Shandong
Fortune Shop	Green Intelligent Power Saver	Just used it for a couple days. I don't know yet.	Jingdong prefecture level city in Shandong
Fortune Shop	Green Intelligent Power Saver	The quality is quite good, cheaper than the physical store, good, next time will come again!	Jingdong prefecture level city in Shandong
Fortune Shop	Green Intelligent Power Saver	Good quality, cheap and affordable worth recommending to buy!	Jingdong prefecture level city in Shandong
Fortune Shop	Green Intelligent Power Saver	Very good quality, well made, works well! Satisfied	Jingdong prefecture level city in Shandong
Fortune Shop	Green Intelligent Power Saver	The logistics is very fast quality is very good very pleasant a shopping	Jingdong prefecture level city in Shandong
Fortune Shop	Green Intelligent Power Saver	Use it and then come back to evaluate!	Jingdong prefecture level city in Shandong
Fortune Shop	Green Intelligent Power Saver	# It's no use lying at all #	Jingdong prefecture level city in Shandong
Fortune Shop	Green Intelligent Power Saver	I can't get a voltage drop.	Jingdong prefecture level city in Shandong
Fortune Shop	Green Intelligent Power Saver	Evaluate after use	Jingdong prefecture level city in Shandong
Fortune Shop	Green Intelligent Power Saver	Still on trial.	Jingdong prefecture level city in Shandong
Fortune Shop	Green Intelligent Power Saver	ineffective	Jingdong prefecture level city in Shandong
Fortune Shop	Green Intelligent Power Saver	Satisfaction Shopping	Jingdong prefecture level city in Shandong
Fortune Shop	Green Intelligent Power Saver	Lies.	Jingdong prefecture level city in Shandong
Fortune Shop	Green Intelligent Power Saver	It's a real power saver.	Jingdong prefecture level city in Shandong
Fortune Shop	Green Intelligent Power Saver	in use	Jingdong prefecture level city in Shandong
Fortune Shop	Green Intelligent Power Saver	I don't know yet.	Jingdong prefecture level city in Shandong
Fortune Shop	Green Intelligent Power Saver	not care about anything	Jingdong prefecture level city in Shandong
Fortune Shop	Green Intelligent Power Saver	It doesn't seem to have much effect	Jingdong prefecture level city in Shandong
Fortune Shop	Green Intelligent Power Saver	Let the merchant see for himself, does the plug positive and negative terminals look like this?	Jingdong prefecture level city in Shandong
Fortune Shop	Green Intelligent Power Saver	It doesn't work well, the electricity bill is less before using it, but more after using it, contacting customer service, the service is still not good, and returning it is slow	Jingdong prefecture level city in Shandong
Fortune Shop	Green Intelligent Power Saver	With the advertisement product does not match, above the effective negative, power saving rate of two rows of words it, the product looks very thin, with 3 days to see the meter number is still with the previous no difference, which is obviously fraudulent behavior!	Jingdong prefecture level city in Shandong
Fortune Shop	Green Intelligent Power Saver	Doesn't save any power at all. Not much use.	Jingdong prefecture level city in Shandong
Fortune Shop	Green Intelligent Power Saver	The plugs are all loose, they're all the same length and they don't work at all	Jingdong prefecture level city in Shandong
Fortune Shop	Green Intelligent Power Saver	It's bad 👎 it's not effective at all, you'd be crazy to sell this garbage on the internet.	Jingdong prefecture level city in Shandong
Fortune Shop	Green Intelligent Power Saver	It doesn't save any power at all, so I hope the old timers don't fall for this scam again	Jingdong prefecture level city in Shandong
Fortune Shop	Green Intelligent Power Saver	I've been using it for over a month now and haven't noticed any power savings.	Jingdong prefecture level city in Shandong
Fortune Shop	Green Intelligent Power Saver	It doesn't work at all. The electricity bill is higher when you use it.	Jingdong prefecture level city in Shandong
Fortune Shop	Green Intelligent Power Saver	It doesn't save any power, it's still as bad as ever.	Jingdong prefecture level city in Shandong
Fortune Shop	Green Intelligent Power Saver	Last night before leaving work to look at the table, and then plugged into the node device, this morning and then look at the table, than the original save a few degrees of electricity, very good to have a power-saving effect, easy to use, satisfied with the shopping praise.	Jingdong prefecture level city in Shandong
Fortune Shop	Green Intelligent Power Saver	The product is real and effective, the electricity bill is reduced, really good, thanks to the merchants have so many good products, the product is real and effective, the electricity bill is reduced, want to save electricity friends quickly buy it, early use early to save money.	Jingdong prefecture level city in Shandong
Fortune Shop	Green Intelligent Power Saver	Saver saves time and money, baby appearance is beautiful, the quality of goods is good, and the same as the business description, good service attitude, packaging is complete, logistics is very fast, praise to	Jingdong prefecture level city in Shandong
Fortune Shop	Green Intelligent Power Saver	The effect is good, summer, home electricity is relatively large, buy and use a few days, feel the effect is very good, originally ten dollars a day electricity bill, now eight dollars a day, quite good	Jingdong prefecture level city in Shandong
Fortune Shop	Green Intelligent Power Saver	Indeed can save electricity and money, the first time I know this or from a friend's home to see, but also do not believe it, and now they use it to know that there is this good thing.	Jingdong prefecture level city in Shandong
Fortune Shop	Green Intelligent Power Saver	I have received the Smart Power Saver. Perfectly packaged. Fast delivery. Boss customer service attitude is also very good. It's worth buying. I plugged it in and tried it and it really saves more power than the original. I'll buy it again next time.	Jingdong prefecture level city in Shandong
Fortune Shop	Green Intelligent Power Saver	It's nice to save a dozen bucks a night on two air conditioners that used to take 30 or 40 degrees a night, but now it's only 10 or so degrees a night	Jingdong prefecture level city in Shandong
Fortune Shop	Green Intelligent Power Saver	Product no manufacturer's name no product qualification certificate, try two days to see the effect of	Jingdong prefecture level city in Shandong
Fortune Shop	Green Intelligent Power Saver	I've been using it for over a month and have saved over $80, so I've repurchased a few more back to save together	Jingdong prefecture level city in Shandong
Fortune Shop	Green Intelligent Power Saver	I haven't used it yet and will comment when I do, but it's well made.	Jingdong prefecture level city in Shandong

Fortune Shop	Green Intelligent Power Saver	It's quite energy efficient. It's really a boon for the home.	Jingdong prefecture level city in Shandong
Fortune Shop	Green Intelligent Power Saver	Energy-saving socket received a month, after using it for a month proved that it is indeed energy-saving, electricity bill saving, will buy it again, thank you!	Jingdong prefecture level city in Shandong
Fortune Shop	Green Intelligent Power Saver	There was a similar product many years ago. Now technology has evolved, buy one to try, according to the instructions in the box, it takes one or two dozen days to show results. Check your electric bill and see what happens.	Jingdong prefecture level city in Shandong
Fortune Shop	Green Intelligent Power Saver	Jingdong logistics is really fast, the same day the order arrived on the same day, more convenient than the supermarket, and a wide range of choices, the	Jingdong prefecture level city in Shandong
Fortune Shop	Green Intelligent Power Saver	I've been using this for a month or so and I can't see any results, it really is a scam as they say.	Jingdong prefecture level city in Shandong
Fortune Shop	Green Intelligent Power Saver	Very good use, a month can save a few degrees of power loss of electricity, introduced to the relatives to buy back to try.	Jingdong prefecture level city in Shandong
Fortune Shop	Green Intelligent Power Saver	It's really powerful! With this air conditioner can be turned on at any time without the pressure of paying the electricity bill! It's good for the people!	Jingdong prefecture level city in Shandong
Fortune Shop	Green Intelligent Power Saver	This business is a big ** products are not qualified, with a month of electricity more than one hundred percent of thirty, the purchase agreed to return the goods but do not receive a refund after receiving the goods, looking for the official side does not help, the Jingdong is a big **, I have never shopped in	Jingdong prefecture level city in Shandong
Fortune Shop	Green Intelligent Power Saver	It's good. I tried it. It saves power.	Jingdong prefecture level city in Shandong
Fortune Shop	Green Intelligent Power Saver	Nothing**, it seems like there's more	Jingdong prefecture level city in Shandong
Fortune Shop	Green Intelligent Power Saver	Power saving device received, well packaged, good quality, fast logistics	Jingdong prefecture level city in Shandong
Fortune Shop	Green Intelligent Power Saver	The service is okay, is that when I bought five, the desirable pieces are three, and then wait a few days and send me two replacement, I do not know if it is a mistake of the shipper, if I was not in the courier there to see, go home to say no, the	Jingdong prefecture level city in Shandong
Fortune Shop	Green Intelligent Power Saver	This thing will only cost me more if I use it, but before I used it, it was less than a ~ electricity a month, and after I used it, it was more than two hundred kWh a month.	Jingdong prefecture level city in Shandong
Fortune Shop	Green Intelligent Power Saver	I just used it. I don't know how it is.	Jingdong prefecture level city in Shandong
Fortune Shop	Green Intelligent Power Saver	I've received the product and I'm using it, so I'll see how it goes in a month!	Jingdong prefecture level city in Shandong
Fortune Shop	Green Intelligent Power Saver	The goods have just received the effect do not know how, please look forward to the results.	Jingdong prefecture level city in Shandong
Fortune Shop	Green Intelligent Power Saver	Power saving device, the material is very good practical and durable, the size is suitable, packaging is very good, the price is cheap, logistics is very fast!	Jingdong prefecture level city in Shandong
Fortune Shop	Green Intelligent Power Saver	Very nice, will comment in detail after using it for a while.	Jingdong prefecture level city in Shandong
Fortune Shop	Green Intelligent Power Saver	Good stuff to trust and support	Jingdong prefecture level city in Shandong
Fortune Shop	Green Intelligent Power Saver	I'll comment after I use it, doubt if it really saves power no	Jingdong prefecture level city in Shandong
Fortune Shop	Green Intelligent Power Saver	It's good quality and works well!	Jingdong prefecture level city in Shandong
Fortune Shop	Green Intelligent Power Saver	hahaha just right hahahahahaha hiccup hahahaha	Jingdong prefecture level city in Shandong
Fortune Shop	Green Intelligent Power Saver	The real thing matches the picture and looks great!	Jingdong prefecture level city in Shandong
Fortune Shop	Green Intelligent Power Saver	Plugged it in and we'll see when we pay the bills at the end of the year!	Jingdong prefecture level city in Shandong
Fortune Shop	Green Intelligent Power Saver	False advertising. It doesn't work.	Jingdong prefecture level city in Shandong
Fortune Shop	Green Intelligent Power Saver	Goods received, good effect	Jingdong prefecture level city in Shandong
Fortune Shop	Green Intelligent Power Saver	It looks okay, we'll see if it saves electricity when the time comes	Jingdong prefecture level city in Shandong
Fortune Shop	Green Intelligent Power Saver	Logistics is very fast quality is very good, the price is very affordable, accessories are also complete, installed, satisfied with good use	Jingdong prefecture level city in Shandong
Fortune Shop	Green Intelligent Power Saver	I've received the power saver. I'm looking forward to the savings.	Jingdong prefecture level city in Shandong
Fortune Shop	Green Intelligent Power Saver	Received, have not yet used, after use and then evaluate	Jingdong prefecture level city in Shandong
Fortune Shop	Green Intelligent Power Saver	I received the item, but I don't know if it saves power or not.	Jingdong prefecture level city in Shandong
Fortune Shop	Green Intelligent Power Saver	On Trial	Jingdong prefecture level city in Shandong
Fortune Shop	Green Intelligent Power Saver	Use it and see how it goes.	Jingdong prefecture level city in Shandong
Fortune Shop	Green Intelligent Power Saver	Quality is very good, no odor, fine workmanship, grain color are very like, satisfied!	Jingdong prefecture level city in Shandong
Fortune Shop	Green Intelligent Power Saver	I'll give you a good review. I don't know if it'll work.	Jingdong prefecture level city in Shandong
Fortune Shop	Green Intelligent Power Saver	The logistics is very fast and the package is intact. Good use of affordable. The effect is very good, is genuine	Jingdong prefecture level city in Shandong
Fortune Shop	Green Intelligent Power Saver	Nothing happens when you plug it in.	Jingdong prefecture level city in Shandong
Fortune Shop	Green Intelligent Power Saver	I don't know if it's as good as it sounds.	Jingdong prefecture level city in Shandong
Fortune Shop	Green Intelligent Power Saver	The quality of the stuff is very good, exactly the same as the seller's description, very satisfied!	Jingdong prefecture level city in Shandong
Fortune Shop	Green Intelligent Power Saver	The saver works great, it really works, it's affordable and worth buying.	Jingdong prefecture level city in Shandong
Fortune Shop	Green Intelligent Power Saver	Just used it for a couple days. I don't know yet.	Jingdong prefecture level city in Shandong
Fortune Shop	Green Intelligent Power Saver	The quality is quite good, cheaper than the physical store, good, next time will come again!	Jingdong prefecture level city in Shandong
Fortune Shop	Green Intelligent Power Saver	Good quality, cheap and affordable worth recommending to buy!	Jingdong prefecture level city in Shandong
Fortune Shop	Green Intelligent Power Saver	Very good quality, well made, works well! Satisfied	Jingdong prefecture level city in Shandong
Fortune Shop	Green Intelligent Power Saver	The logistics is very fast quality is very good very pleasant a shopping	Jingdong prefecture level city in Shandong
Fortune Shop	Green Intelligent Power Saver	Use it and then come back to evaluate!	Jingdong prefecture level city in Shandong
Fortune Shop	Green Intelligent Power Saver	# It's no use lying at all #	Jingdong prefecture level city in Shandong
Fortune Shop	Green Intelligent Power Saver	I can't get a voltage drop.	Jingdong prefecture level city in Shandong
Fortune Shop	Green Intelligent Power Saver	Evaluate after use	Jingdong prefecture level city in Shandong
Fortune Shop	Green Intelligent Power Saver	Still on trial.	Jingdong prefecture level city in Shandong

Fortune Shop	Green Intelligent Power Saver	ineffective	Jingdong prefecture level city in Shandong
Fortune Shop	Green Intelligent Power Saver	Satisfaction Shopping	Jingdong prefecture level city in Shandong
Fortune Shop	Green Intelligent Power Saver	Lies.	Jingdong prefecture level city in Shandong
Fortune Shop	Green Intelligent Power Saver	It's a real power saver.	Jingdong prefecture level city in Shandong
Fortune Shop	Green Intelligent Power Saver	in use	Jingdong prefecture level city in Shandong
Fortune Shop	Green Intelligent Power Saver	I don't know yet.	Jingdong prefecture level city in Shandong
Fortune Shop	Green Intelligent Power Saver	not care about anything	Jingdong prefecture level city in Shandong
Fortune Shop	Green Intelligent Power Saver	It doesn't seem to have much effect	Jingdong prefecture level city in Shandong
Fortune Shop	Green Intelligent Power Saver	Let the merchant see for himself, does the plug positive and negative terminals look like this?	Jingdong prefecture level city in Shandong
Fortune Shop	Green Intelligent Power Saver	It doesn't work well, the electricity bill is less before using it, but more after using it, contacting customer service, the service is still not good, and returning it is slow	Jingdong prefecture level city in Shandong
Fortune Shop	Green Intelligent Power Saver	With the advertisement product does not match, above the effective negative, power saving rate of two rows of words it, the product looks very thin, with 3 days to see the meter number is still with the previous no difference, which is obviously fraudulent behavior!	Jingdong prefecture level city in Shandong
Fortune Shop	Green Intelligent Power Saver	Doesn't save any power at all. Not much use.	Jingdong prefecture level city in Shandong
Fortune Shop	Green Intelligent Power Saver	The plugs are all loose, they're all the same length and they don't work at all	Jingdong prefecture level city in Shandong
Fortune Shop	Green Intelligent Power Saver	It's bad 👎 it's not effective at all, you'd be crazy to sell this garbage on the internet.	Jingdong prefecture level city in Shandong
Fortune Shop	Green Intelligent Power Saver	It doesn't save any power at all, so I hope the old timers don't fall for this scam again	Jingdong prefecture level city in Shandong
Fortune Shop	Green Intelligent Power Saver	I've been using it for over a month now and haven't noticed any power savings.	Jingdong prefecture level city in Shandong
Fortune Shop	Green Intelligent Power Saver	It doesn't work at all. The electricity bill is higher when you use it.	Jingdong prefecture level city in Shandong
Fortune Shop	Green Intelligent Power Saver	It doesn't save any power, it's still as bad as ever.	Jingdong prefecture level city in Shandong
Fortune Shop	Green Intelligent Power Saver	Last night before leaving work to look at the table, and then plugged into the node device, this morning and then look at the table, than the original save a few degrees of electricity, very good to have a power-saving effect, easy to use, satisfied with the shopping praise.	Jingdong prefecture level city in Shandong
Fortune Shop	Green Intelligent Power Saver	The product is real and effective, the electricity bill is reduced, really good, thanks to the merchants have so many good products, the product is real and effective, the electricity bill is reduced, want to save electricity friends quickly buy it, early use early to save money.	Jingdong prefecture level city in Shandong
Fortune Shop	Green Intelligent Power Saver	Saver saves time and money, baby appearance is beautiful, the quality of goods is good, and the same as the business description, good service attitude, packaging is complete, logistics is very fast, praise to	Jingdong prefecture level city in Shandong
Fortune Shop	Green Intelligent Power Saver	The effect is good, summer, home electricity is relatively large, buy and use a few days, feel the effect is very good, originally ten dollars a day electricity bill, now eight dollars a day, quite good	Jingdong prefecture level city in Shandong
Fortune Shop	Green Intelligent Power Saver	Indeed can save electricity and money, the first time I know this or from a friend's home to see, but also do not believe it, and now they use it to know that there is this good thing.	Jingdong prefecture level city in Shandong
Fortune Shop	Green Intelligent Power Saver	I have received the Smart Power Saver. Perfectly packaged. Fast delivery. Boss customer service attitude is also very good. It's worth buying. I plugged it in and tried it and it really saves more power than the original. I'll buy it again next time.	Jingdong prefecture level city in Shandong
Fortune Shop	Green Intelligent Power Saver	It's nice to save a dozen bucks a night on two air conditioners that used to take 30 or 40 degrees a night, but now it's only 10 or so degrees a night	Jingdong prefecture level city in Shandong
Fortune Shop	Green Intelligent Power Saver	Product no manufacturer's name no product qualification certificate, try two days to see the effect of	Jingdong prefecture level city in Shandong
Fortune Shop	Green Intelligent Power Saver	I've been using it for over a month and have saved over $80, so I've repurchased a few more back to save together	Jingdong prefecture level city in Shandong
Fortune Shop	Green Intelligent Power Saver	I haven't used it yet and will comment when I do, but it's well made.	Jingdong prefecture level city in Shandong
Fortune Shop	Green Intelligent Power Saver	It's quite energy efficient. It's really a boon for the home.	Jingdong prefecture level city in Shandong
Fortune Shop	Green Intelligent Power Saver	Energy-saving socket received a month, after using it for a month proved that it is indeed energy-saving, electricity bill saving, will buy it again, thank you!	Jingdong prefecture level city in Shandong
Fortune Shop	Green Intelligent Power Saver	There was a similar product many years ago. Now technology has evolved, buy one to try, according to the instructions in the box, it takes one or two dozen days to show results. Check your electric bill and see what happens.	Jingdong prefecture level city in Shandong
Fortune Shop	Green Intelligent Power Saver	Jingdong logistics is really fast, the same day the order arrived on the same day, more convenient than the supermarket, and a wide range of choices, the	Jingdong prefecture level city in Shandong
Fortune Shop	Green Intelligent Power Saver	I've been using this for a month or so and I can't see any results, it really is a scam as they say.	Jingdong prefecture level city in Shandong
Fortune Shop	Green Intelligent Power Saver	Very good use, a month can save a few degrees of power loss of electricity, introduced to the relatives to buy back to try.	Jingdong prefecture level city in Shandong
Fortune Shop	Green Intelligent Power Saver	It's really powerful! With this air conditioner can be turned on at any time without the pressure of paying the electricity bill! It's good for the people!	Jingdong prefecture level city in Shandong
Fortune Shop	Green Intelligent Power Saver	This business is a big ** products are not qualified, with a month of electricity more than one hundred percent of thirty, the purchase agreed to return the goods but do not receive a refund after receiving the goods, looking for the official side does not help, the Jingdong is a big **, I have never shopped in	Jingdong prefecture level city in Shandong
Fortune Shop	Green Intelligent Power Saver	It's good. I tried it. It saves power.	Jingdong prefecture level city in Shandong
Fortune Shop	Green Intelligent Power Saver	Nothing**, it seems like there's more	Jingdong prefecture level city in Shandong
Fortune Shop	Green Intelligent Power Saver	Power saving device received, well packaged, good quality, fast logistics	Jingdong prefecture level city in Shandong
Fortune Shop	Green Intelligent Power Saver	The service is okay, is that when I bought five, the desirable pieces are three, and then wait a few days and send me two replacement, I do not know if it is a mistake of the shipper, if I was not in the courier there to see, go home to say no, the	Jingdong prefecture level city in Shandong
Fortune Shop	Green Intelligent Power Saver	This thing will only cost me more if I use it, but before I used it, it was less than a ~ electricity a month, and after I used it, it was more than two hundred kWh a month.	Jingdong prefecture level city in Shandong
Fortune Shop	Green Intelligent Power Saver	I just used it. I don't know how it is.	Jingdong prefecture level city in Shandong
Fortune Shop	Green Intelligent Power Saver	I've received the product and I'm using it, so I'll see how it goes in a month!	Jingdong prefecture level city in Shandong
Fortune Shop	Green Intelligent Power Saver	The goods have just received the effect do not know how, please look forward to the results.	Jingdong prefecture level city in Shandong
Fortune Shop	Green Intelligent Power Saver	Power saving device, the material is very good practical and durable, the size is suitable, packaging is very good, the price is cheap, logistics is very fast!	Jingdong prefecture level city in Shandong
Fortune Shop	Green Intelligent Power Saver	Very nice, will comment in detail after using it for a while.	Jingdong prefecture level city in Shandong
Fortune Shop	Green Intelligent Power Saver	Good stuff to trust and support	Jingdong prefecture level city in Shandong
Fortune Shop	Green Intelligent Power Saver	I'll comment after I use it, doubt if it really saves power no	Jingdong prefecture level city in Shandong

Fortune Shop	Green Intelligent Power Saver	It's good quality and works well!	Jingdong prefecture level city in Shandong
Fortune Shop	Green Intelligent Power Saver	hahaha just right hahahahahaha hiccup hahahaha	Jingdong prefecture level city in Shandong
Fortune Shop	Green Intelligent Power Saver	The real thing matches the picture and looks great!	Jingdong prefecture level city in Shandong
Fortune Shop	Green Intelligent Power Saver	Plugged it in and we'll see when we pay the bills at the end of the year!	Jingdong prefecture level city in Shandong
Fortune Shop	Green Intelligent Power Saver	False advertising. It doesn't work.	Jingdong prefecture level city in Shandong
Fortune Shop	Green Intelligent Power Saver	Goods received, good effect	Jingdong prefecture level city in Shandong
Fortune Shop	Green Intelligent Power Saver	It looks okay, we'll see if it saves electricity when the time comes	Jingdong prefecture level city in Shandong
Fortune Shop	Green Intelligent Power Saver	Logistics is very fast quality is very good, the price is very affordable, accessories are also complete, installed, satisfied with good use	Jingdong prefecture level city in Shandong
Fortune Shop	Green Intelligent Power Saver	I've received the power saver. I'm looking forward to the savings.	Jingdong prefecture level city in Shandong
Fortune Shop	Green Intelligent Power Saver	Received, have not yet used, after use and then evaluate	Jingdong prefecture level city in Shandong
Fortune Shop	Green Intelligent Power Saver	I received the item, but I don't know if it saves power or not.	Jingdong prefecture level city in Shandong
Fortune Shop	Green Intelligent Power Saver	On Trial	Jingdong prefecture level city in Shandong
Fortune Shop	Green Intelligent Power Saver	Use it and see how it goes.	Jingdong prefecture level city in Shandong
Fortune Shop	Green Intelligent Power Saver	Quality is very good, no odor, fine workmanship, grain color are very like, satisfied!	Jingdong prefecture level city in Shandong
Fortune Shop	Green Intelligent Power Saver	I'll give you a good review. I don't know if it'll work.	Jingdong prefecture level city in Shandong
Fortune Shop	Green Intelligent Power Saver	The logistics is very fast and the package is intact. Good use of affordable. The effect is very good, is genuine	Jingdong prefecture level city in Shandong
Fortune Shop	Green Intelligent Power Saver	Nothing happens when you plug it in.	Jingdong prefecture level city in Shandong
Fortune Shop	Green Intelligent Power Saver	I don't know if it's as good as it sounds.	Jingdong prefecture level city in Shandong
Fortune Shop	Green Intelligent Power Saver	The quality of the stuff is very good, exactly the same as the seller's description, very satisfied!	Jingdong prefecture level city in Shandong
Fortune Shop	Green Intelligent Power Saver	The saver works great, it really works, it's affordable and worth buying.	Jingdong prefecture level city in Shandong
Fortune Shop	Green Intelligent Power Saver	Just used it for a couple days. I don't know yet.	Jingdong prefecture level city in Shandong
Fortune Shop	Green Intelligent Power Saver	The quality is quite good, cheaper than the physical store, good, next time will come again!	Jingdong prefecture level city in Shandong
Fortune Shop	Green Intelligent Power Saver	Good quality, cheap and affordable worth recommending to buy!	Jingdong prefecture level city in Shandong
Fortune Shop	Green Intelligent Power Saver	Very good quality, well made, works well! Satisfied	Jingdong prefecture level city in Shandong
Fortune Shop	Green Intelligent Power Saver	The logistics is very fast quality is very good very pleasant a shopping	Jingdong prefecture level city in Shandong
Fortune Shop	Green Intelligent Power Saver	Use it and then come back to evaluate!	Jingdong prefecture level city in Shandong
Fortune Shop	Green Intelligent Power Saver	# It's no use lying at all #	Jingdong prefecture level city in Shandong
Fortune Shop	Green Intelligent Power Saver	I can't get a voltage drop.	Jingdong prefecture level city in Shandong
Fortune Shop	Green Intelligent Power Saver	Evaluate after use	Jingdong prefecture level city in Shandong
Fortune Shop	Green Intelligent Power Saver	Still on trial.	Jingdong prefecture level city in Shandong
Fortune Shop	Green Intelligent Power Saver	ineffective	Jingdong prefecture level city in Shandong
Fortune Shop	Green Intelligent Power Saver	Satisfaction Shopping	Jingdong prefecture level city in Shandong
Fortune Shop	Green Intelligent Power Saver	Lies.	Jingdong prefecture level city in Shandong
Fortune Shop	Green Intelligent Power Saver	It's a real power saver.	Jingdong prefecture level city in Shandong
Fortune Shop	Green Intelligent Power Saver	in use	Jingdong prefecture level city in Shandong
Fortune Shop	Green Intelligent Power Saver	I don't know yet.	Jingdong prefecture level city in Shandong
Fortune Shop	Green Intelligent Power Saver	not care about anything	Jingdong prefecture level city in Shandong
Fortune Shop	Green Intelligent Power Saver	It doesn't seem to have much effect	Jingdong prefecture level city in Shandong
Fortune Shop	Green Intelligent Power Saver	Let the merchant see for himself, does the plug positive and negative terminals look like this?	Jingdong prefecture level city in Shandong
Fortune Shop	Green Intelligent Power Saver	It doesn't work well, the electricity bill is less before using it, but more after using it, contacting customer service, the service is still not good, and returning it is slow	Jingdong prefecture level city in Shandong
Fortune Shop	Green Intelligent Power Saver	With the advertisement product does not match, above the effective negative, power saving rate of two rows of words it, the product looks very thin, with 3 days to see the meter number is still with the previous no difference, which is obviously fraudulent behavior!	Jingdong prefecture level city in Shandong
Fortune Shop	Green Intelligent Power Saver	Doesn't save any power at all. Not much use.	Jingdong prefecture level city in Shandong
Fortune Shop	Green Intelligent Power Saver	The plugs are all loose, they're all the same length and they don't work at all	Jingdong prefecture level city in Shandong
Fortune Shop	Green Intelligent Power Saver	It's bad 👎 it's not effective at all, you'd be crazy to sell this garbage on the internet.	Jingdong prefecture level city in Shandong
Fortune Shop	Green Intelligent Power Saver	It doesn't save any power at all, so I hope the old timers don't fall for this scam again	Jingdong prefecture level city in Shandong
Fortune Shop	Green Intelligent Power Saver	I've been using it for over a month now and haven't noticed any power savings.	Jingdong prefecture level city in Shandong
Fortune Shop	Green Intelligent Power Saver	It doesn't work at all. The electricity bill is higher when you use it.	Jingdong prefecture level city in Shandong
Fortune Shop	Green Intelligent Power Saver	It doesn't save any power, it's still as bad as ever.	Jingdong prefecture level city in Shandong
Fortune Shop	Green Intelligent Power Saver	Last night before leaving work to look at the table, and then plugged into the node device, this morning and then look at the table, than the original save a few degrees of electricity, very good to have a power-saving effect, easy to use, satisfied with the shopping praise.	Jingdong prefecture level city in Shandong
Fortune Shop	Green Intelligent Power Saver	The product is real and effective, the electricity bill is reduced, really good, thanks to the merchants have so many good products, the product is real and effective, the electricity bill is reduced, want to save electricity friends quickly buy it, early use early to save money.	Jingdong prefecture level city in Shandong

Fortune Shop	Green Intelligent Power Saver	Saver saves time and money, baby appearance is beautiful, the quality of goods is good, and the same as the business description, good service attitude, packaging is complete, logistics is very fast, praise to	Jingdong prefecture level city in Shandong
Fortune Shop	Green Intelligent Power Saver	The effect is good, summer, home electricity is relatively large, buy and use a few days, feel the effect is very good, originally ten dollars a day electricity bill, now eight dollars a day, quite good	Jingdong prefecture level city in Shandong
Fortune Shop	Green Intelligent Power Saver	Indeed can save electricity and money, the first time I know this or from a friend's home to see, but also do not believe it, and now they use it to know that there is this good thing.	Jingdong prefecture level city in Shandong
Fortune Shop	Green Intelligent Power Saver	I have received the Smart Power Saver. Perfectly packaged. Fast delivery. Boss customer service attitude is also very good. It's worth buying. I plugged it in and tried it and it really saves more power than the original. I'll buy it again next time.	Jingdong prefecture level city in Shandong
Fortune Shop	Green Intelligent Power Saver	It's nice to save a dozen bucks a night on two air conditioners that used to take 30 or 40 degrees a night, but now it's only 10 or so degrees a night	Jingdong prefecture level city in Shandong
Fortune Shop	Green Intelligent Power Saver	Product no manufacturer's name no product qualification certificate, try two days to see the effect of	Jingdong prefecture level city in Shandong
Fortune Shop	Green Intelligent Power Saver	I've been using it for over a month and have saved over $80, so I've repurchased a few more back to save together	Jingdong prefecture level city in Shandong
Fortune Shop	Green Intelligent Power Saver	I haven't used it yet and will comment when I do, but it's well made.	Jingdong prefecture level city in Shandong
Fortune Shop	Green Intelligent Power Saver	It's quite energy efficient. It's really a boon for the home.	Jingdong prefecture level city in Shandong
Fortune Shop	Green Intelligent Power Saver	Energy-saving socket received a month, after using it for a month proved that it is indeed energy-saving, electricity bill saving, will buy it again, thank you!	Jingdong prefecture level city in Shandong
Fortune Shop	Green Intelligent Power Saver	There was a similar product many years ago. Now technology has evolved, buy one to try, according to the instructions in the box, it takes one or two dozen days to show results. Check your electric bill and see what happens.	Jingdong prefecture level city in Shandong
Fortune Shop	Green Intelligent Power Saver	Jingdong logistics is really fast, the same day the order arrived on the same day, more convenient than the supermarket, and a wide range of choices, the	Jingdong prefecture level city in Shandong
Fortune Shop	Green Intelligent Power Saver	I've been using this for a month or so and I can't see any results, it really is a scam as they say.	Jingdong prefecture level city in Shandong
Fortune Shop	Green Intelligent Power Saver	Very good use, a month can save a few degrees of power loss of electricity, introduced to the relatives to buy back to try.	Jingdong prefecture level city in Shandong
Fortune Shop	Green Intelligent Power Saver	It's really powerful! With this air conditioner can be turned on at any time without the pressure of paying the electricity bill! It's good for the people!	Jingdong prefecture level city in Shandong
Fortune Shop	Green Intelligent Power Saver	This business is a big ** products are not qualified, with a month of electricity more than one hundred percent of thirty, the purchase agreed to return the goods but do not receive a refund after receiving the goods, looking for the official side does not help, the Jingdong is a big **, I have never shopped in	Jingdong prefecture level city in Shandong
Fortune Shop	Green Intelligent Power Saver	It's good. I tried it. It saves power.	Jingdong prefecture level city in Shandong
Fortune Shop	Green Intelligent Power Saver	Nothing**, it seems like there's more	Jingdong prefecture level city in Shandong
Fortune Shop	Green Intelligent Power Saver	Power saving device received, well packaged, good quality, fast logistics	Jingdong prefecture level city in Shandong
Fortune Shop	Green Intelligent Power Saver	The service is okay, is that when I bought five, the desirable pieces are three, and then wait a few days and send me two replacement, I do not know if it is a mistake of the shipper, if I was not in the courier there to see, go home to say no, the	Jingdong prefecture level city in Shandong
Fortune Shop	Green Intelligent Power Saver	This thing will only cost me more if I use it, but before I used it, it was less than a ~ electricity a month, and after I used it, it was more than two hundred kWh a month.	Jingdong prefecture level city in Shandong
Fortune Shop	Green Intelligent Power Saver	I just used it. I don't know how it is.	Jingdong prefecture level city in Shandong
Fortune Shop	Green Intelligent Power Saver	I've received the product and I'm using it, so I'll see how it goes in a month!	Jingdong prefecture level city in Shandong
Fortune Shop	Green Intelligent Power Saver	The goods have just received the effect do not know how, please look forward to the results.	Jingdong prefecture level city in Shandong
Fortune Shop	Green Intelligent Power Saver	Power saving device, the material is very good practical and durable, the size is suitable, packaging is very good, the price is cheap, logistics is very fast!	Jingdong prefecture level city in Shandong
Fortune Shop	Green Intelligent Power Saver	Very nice, will comment in detail after using it for a while.	Jingdong prefecture level city in Shandong
Fortune Shop	Green Intelligent Power Saver	Good stuff to trust and support	Jingdong prefecture level city in Shandong
Fortune Shop	Green Intelligent Power Saver	I'll comment after I use it, doubt if it really saves power no	Jingdong prefecture level city in Shandong
Fortune Shop	Green Intelligent Power Saver	It's good quality and works well!	Jingdong prefecture level city in Shandong
Fortune Shop	Green Intelligent Power Saver	hahaha just right hahahahahaha hiccup hahahaha	Jingdong prefecture level city in Shandong
Fortune Shop	Green Intelligent Power Saver	The real thing matches the picture and looks great!	Jingdong prefecture level city in Shandong
Fortune Shop	Green Intelligent Power Saver	Plugged it in and we'll see when we pay the bills at the end of the year!	Jingdong prefecture level city in Shandong
Fortune Shop	Green Intelligent Power Saver	False advertising. It doesn't work.	Jingdong prefecture level city in Shandong
Fortune Shop	Green Intelligent Power Saver	Goods received, good effect	Jingdong prefecture level city in Shandong
Fortune Shop	Green Intelligent Power Saver	It looks okay, we'll see if it saves electricity when the time comes	Jingdong prefecture level city in Shandong
Fortune Shop	Green Intelligent Power Saver	Logistics is very fast quality is very good, the price is very affordable, accessories are also complete, installed, satisfied with good use	Jingdong prefecture level city in Shandong
Fortune Shop	Green Intelligent Power Saver	I've received the power saver. I'm looking forward to the savings.	Jingdong prefecture level city in Shandong
Fortune Shop	Green Intelligent Power Saver	Received, have not yet used, after use and then evaluate	Jingdong prefecture level city in Shandong
Fortune Shop	Green Intelligent Power Saver	I received the item, but I don't know if it saves power or not.	Jingdong prefecture level city in Shandong
Fortune Shop	Green Intelligent Power Saver	On Trial	Jingdong prefecture level city in Shandong
Fortune Shop	Green Intelligent Power Saver	Use it and see how it goes.	Jingdong prefecture level city in Shandong
Fortune Shop	Green Intelligent Power Saver	Quality is very good, no odor, fine workmanship, grain color are very like, satisfied!	Jingdong prefecture level city in Shandong
Fortune Shop	Green Intelligent Power Saver	I'll give you a good review. I don't know if it'll work.	Jingdong prefecture level city in Shandong
Fortune Shop	Green Intelligent Power Saver	The logistics is very fast and the package is intact. Good use of affordable. The effect is very good, is genuine	Jingdong prefecture level city in Shandong
Fortune Shop	Green Intelligent Power Saver	Nothing happens when you plug it in.	Jingdong prefecture level city in Shandong
Fortune Shop	Green Intelligent Power Saver	I don't know if it's as good as it sounds.	Jingdong prefecture level city in Shandong
Fortune Shop	Green Intelligent Power Saver	The quality of the stuff is very good, exactly the same as the seller's description, very satisfied!	Jingdong prefecture level city in Shandong
Fortune Shop	Green Intelligent Power Saver	The saver works great, it really works, it's affordable and worth buying.	Jingdong prefecture level city in Shandong
Fortune Shop	Green Intelligent Power Saver	Just used it for a couple days. I don't know yet.	Jingdong prefecture level city in Shandong

Fortune Shop	Green Intelligent Power Saver	The quality is quite good, cheaper than the physical store, good, next time will come again!	Jingdong prefecture level city in Shandong
Fortune Shop	Green Intelligent Power Saver	Good quality, cheap and affordable worth recommending to buy!	Jingdong prefecture level city in Shandong
Fortune Shop	Green Intelligent Power Saver	Very good quality, well made, works well! Satisfied	Jingdong prefecture level city in Shandong
Fortune Shop	Green Intelligent Power Saver	The logistics is very fast quality is very good very pleasant a shopping	Jingdong prefecture level city in Shandong
Fortune Shop	Green Intelligent Power Saver	Use it and then come back to evaluate!	Jingdong prefecture level city in Shandong
Fortune Shop	Green Intelligent Power Saver	# It's no use lying at all #	Jingdong prefecture level city in Shandong
Fortune Shop	Green Intelligent Power Saver	I can't get a voltage drop.	Jingdong prefecture level city in Shandong
Fortune Shop	Green Intelligent Power Saver	Evaluate after use	Jingdong prefecture level city in Shandong
Fortune Shop	Green Intelligent Power Saver	Still on trial.	Jingdong prefecture level city in Shandong
Fortune Shop	Green Intelligent Power Saver	ineffective	Jingdong prefecture level city in Shandong
Fortune Shop	Green Intelligent Power Saver	Satisfaction Shopping	Jingdong prefecture level city in Shandong
Fortune Shop	Green Intelligent Power Saver	Lies.	Jingdong prefecture level city in Shandong
Fortune Shop	Green Intelligent Power Saver	It's a real power saver.	Jingdong prefecture level city in Shandong
Fortune Shop	Green Intelligent Power Saver	in use	Jingdong prefecture level city in Shandong
Fortune Shop	Green Intelligent Power Saver	I don't know yet.	Jingdong prefecture level city in Shandong
Fortune Shop	Green Intelligent Power Saver	not care about anything	Jingdong prefecture level city in Shandong
Fortune Shop	Green Intelligent Power Saver	It doesn't seem to have much effect	Jingdong prefecture level city in Shandong
Fortune Shop	Green Intelligent Power Saver	Let the merchant see for himself, does the plug positive and negative terminals look like this?	Jingdong prefecture level city in Shandong
Fortune Shop	Green Intelligent Power Saver	It doesn't work well, the electricity bill is less before using it, but more after using it, contacting customer service, the service is still not good, and returning it is slow	Jingdong prefecture level city in Shandong
Fortune Shop	Green Intelligent Power Saver	With the advertisement product does not match, above the effective negative, power saving rate of two rows of words it, the product looks very thin, with 3 days to see the meter number is still with the previous no difference, which is obviously fraudulent behavior!	Jingdong prefecture level city in Shandong
Fortune Shop	Green Intelligent Power Saver	Doesn't save any power at all. Not much use.	Jingdong prefecture level city in Shandong
Fortune Shop	Green Intelligent Power Saver	The plugs are all loose, they're all the same length and they don't work at all	Jingdong prefecture level city in Shandong
Fortune Shop	Green Intelligent Power Saver	It's bad 👎 it's not effective at all, you'd be crazy to sell this garbage on the internet.	Jingdong prefecture level city in Shandong
Fortune Shop	Green Intelligent Power Saver	It doesn't save any power at all, so I hope the old timers don't fall for this scam again	Jingdong prefecture level city in Shandong
Fortune Shop	Green Intelligent Power Saver	I've been using it for over a month now and haven't noticed any power savings.	Jingdong prefecture level city in Shandong
Fortune Shop	Green Intelligent Power Saver	It doesn't work at all. The electricity bill is higher when you use it.	Jingdong prefecture level city in Shandong
Fortune Shop	Green Intelligent Power Saver	It doesn't save any power, it's still as bad as ever.	Jingdong prefecture level city in Shandong
Fortune Shop	Green Intelligent Power Saver	Last night before leaving work to look at the table, and then plugged into the node device, this morning and then look at the table, than the original save a few degrees of electricity, very good to have a power-saving effect, easy to use, satisfied with the shopping praise.	Jingdong prefecture level city in Shandong
Fortune Shop	Green Intelligent Power Saver	The product is real and effective, the electricity bill is reduced, really good, thanks to the merchants have so many good products, the product is real and effective, the electricity bill is reduced, want to save electricity friends quickly buy it, early use early to save money.	Jingdong prefecture level city in Shandong
Fortune Shop	Green Intelligent Power Saver	Saver saves time and money, baby appearance is beautiful, the quality of goods is good, and the same as the business description, good service attitude, packaging is complete, logistics is very fast, praise to	Jingdong prefecture level city in Shandong
Fortune Shop	Green Intelligent Power Saver	The effect is good, summer, home electricity is relatively large, buy and use a few days, feel the effect is very good, originally ten dollars a day electricity bill, now eight dollars a day, quite good	Jingdong prefecture level city in Shandong
Fortune Shop	Green Intelligent Power Saver	Indeed can save electricity and money, the first time I know this or from a friend's home to see, but also do not believe it, and now they use it to know that there is this good thing.	Jingdong prefecture level city in Shandong
Fortune Shop	Green Intelligent Power Saver	I have received the Smart Power Saver. Perfectly packaged. Fast delivery. Boss customer service attitude is also very good. It's worth buying. I plugged it in and tried it and it really saves more power than the original. I'll buy it again next time.	Jingdong prefecture level city in Shandong
Fortune Shop	Green Intelligent Power Saver	It's nice to save a dozen bucks a night on two air conditioners that used to take 30 or 40 degrees a night, but now it's only 10 or so degrees a night	Jingdong prefecture level city in Shandong
Fortune Shop	Green Intelligent Power Saver	Product no manufacturer's name no product qualification certificate, try two days to see the effect of	Jingdong prefecture level city in Shandong
Fortune Shop	Green Intelligent Power Saver	I've been using it for over a month and have saved over $80, so I've repurchased a few more back to save together	Jingdong prefecture level city in Shandong
Fortune Shop	Green Intelligent Power Saver	I haven't used it yet and will comment when I do, but it's well made.	Jingdong prefecture level city in Shandong
Fortune Shop	Green Intelligent Power Saver	It's quite energy efficient. It's really a boon for the home.	Jingdong prefecture level city in Shandong
Fortune Shop	Green Intelligent Power Saver	Energy-saving socket received a month, after using it for a month proved that it is indeed energy-saving, electricity bill saving, will buy it again, thank you!	Jingdong prefecture level city in Shandong
Fortune Shop	Green Intelligent Power Saver	There was a similar product many years ago. Now technology has evolved, buy one to try, according to the instructions in the box, it takes one or two dozen days to show results. Check your electric bill and see what happens.	Jingdong prefecture level city in Shandong
Fortune Shop	Green Intelligent Power Saver	Jingdong logistics is really fast, the same day the order arrived on the same day, more convenient than the supermarket, and a wide range of choices, the	Jingdong prefecture level city in Shandong
Fortune Shop	Green Intelligent Power Saver	I've been using this for a month or so and I can't see any results, it really is a scam as they say.	Jingdong prefecture level city in Shandong
Fortune Shop	Green Intelligent Power Saver	Very good use, a month can save a few degrees of power loss of electricity, introduced to the relatives to buy back to try.	Jingdong prefecture level city in Shandong
Fortune Shop	Green Intelligent Power Saver	It's really powerful! With this air conditioner can be turned on at any time without the pressure of paying the electricity bill! It's good for the people!	Jingdong prefecture level city in Shandong
Fortune Shop	Green Intelligent Power Saver	This business is a big ** products are not qualified, with a month of electricity more than one hundred percent of thirty, the purchase agreed to return the goods but do not receive a refund after receiving the goods, looking for the official side does not help, the Jingdong is a big **, I have never shopped in	Jingdong prefecture level city in Shandong
Fortune Shop	Green Intelligent Power Saver	It's good. I tried it. It saves power.	Jingdong prefecture level city in Shandong
Fortune Shop	Green Intelligent Power Saver	Nothing**, it seems like there's more	Jingdong prefecture level city in Shandong
Fortune Shop	Green Intelligent Power Saver	Power saving device received, well packaged, good quality, fast logistics	Jingdong prefecture level city in Shandong

Fortune Shop	Green Intelligent Power Saver	The service is okay, is that when I bought five, the desirable pieces are three, and then wait a few days and send me two replacement, I do not know if it is a mistake of the shipper, if I was not in the courier there to see, go home to say no, the	Jingdong prefecture level city in Shandong
Fortune Shop	Green Intelligent Power Saver	This thing will only cost me more if I use it, but before I used it, it was less than a ~ electricity a month, and after I used it, it was more than two hundred kWh a month.	Jingdong prefecture level city in Shandong
Fortune Shop	Green Intelligent Power Saver	I just used it. I don't know how it is.	Jingdong prefecture level city in Shandong
Fortune Shop	Green Intelligent Power Saver	I've received the product and I'm using it, so I'll see how it goes in a month!	Jingdong prefecture level city in Shandong
Fortune Shop	Green Intelligent Power Saver	The goods have just received the effect do not know how, please look forward to the results.	Jingdong prefecture level city in Shandong
Fortune Shop	Green Intelligent Power Saver	Power saving device, the material is very good practical and durable, the size is suitable, packaging is very good, the price is cheap, logistics is very fast!	Jingdong prefecture level city in Shandong
Fortune Shop	Green Intelligent Power Saver	Very nice, will comment in detail after using it for a while.	Jingdong prefecture level city in Shandong
Fortune Shop	Green Intelligent Power Saver	Good stuff to trust and support	Jingdong prefecture level city in Shandong
Fortune Shop	Green Intelligent Power Saver	I'll comment after I use it, doubt if it really saves power no	Jingdong prefecture level city in Shandong
Fortune Shop	Green Intelligent Power Saver	It's good quality and works well!	Jingdong prefecture level city in Shandong
Fortune Shop	Green Intelligent Power Saver	hahaha just right hahahahahaha hiccup hahahaha	Jingdong prefecture level city in Shandong
Fortune Shop	Green Intelligent Power Saver	The real thing matches the picture and looks great!	Jingdong prefecture level city in Shandong
Fortune Shop	Green Intelligent Power Saver	Plugged it in and we'll see when we pay the bills at the end of the year!	Jingdong prefecture level city in Shandong
Fortune Shop	Green Intelligent Power Saver	False advertising. It doesn't work.	Jingdong prefecture level city in Shandong
Fortune Shop	Green Intelligent Power Saver	Goods received, good effect	Jingdong prefecture level city in Shandong
Fortune Shop	Green Intelligent Power Saver	It looks okay, we'll see if it saves electricity when the time comes	Jingdong prefecture level city in Shandong
Fortune Shop	Green Intelligent Power Saver	Logistics is very fast quality is very good, the price is very affordable, accessories are also complete, installed, satisfied with good use	Jingdong prefecture level city in Shandong
Fortune Shop	Green Intelligent Power Saver	I've received the power saver. I'm looking forward to the savings.	Jingdong prefecture level city in Shandong
Fortune Shop	Green Intelligent Power Saver	Received, have not yet used, after use and then evaluate	Jingdong prefecture level city in Shandong
Fortune Shop	Green Intelligent Power Saver	I received the item, but I don't know if it saves power or not.	Jingdong prefecture level city in Shandong
Fortune Shop	Green Intelligent Power Saver	On Trial	Jingdong prefecture level city in Shandong
Fortune Shop	Green Intelligent Power Saver	Use it and see how it goes.	Jingdong prefecture level city in Shandong
Fortune Shop	Green Intelligent Power Saver	Quality is very good, no odor, fine workmanship, grain color are very like, satisfied!	Jingdong prefecture level city in Shandong
Fortune Shop	Green Intelligent Power Saver	I'll give you a good review. I don't know if it'll work.	Jingdong prefecture level city in Shandong
Fortune Shop	Green Intelligent Power Saver	The logistics is very fast and the package is intact. Good use of affordable. The effect is very good, is genuine	Jingdong prefecture level city in Shandong
Fortune Shop	Green Intelligent Power Saver	Nothing happens when you plug it in.	Jingdong prefecture level city in Shandong
Fortune Shop	Green Intelligent Power Saver	I don't know if it's as good as it sounds.	Jingdong prefecture level city in Shandong
Fortune Shop	Green Intelligent Power Saver	The quality of the stuff is very good, exactly the same as the seller's description, very satisfied!	Jingdong prefecture level city in Shandong
Fortune Shop	Green Intelligent Power Saver	The saver works great, it really works, it's affordable and worth buying.	Jingdong prefecture level city in Shandong
Fortune Shop	Green Intelligent Power Saver	Just used it for a couple days. I don't know yet.	Jingdong prefecture level city in Shandong
Fortune Shop	Green Intelligent Power Saver	The quality is quite good, cheaper than the physical store, good, next time will come again!	Jingdong prefecture level city in Shandong
Fortune Shop	Green Intelligent Power Saver	Good quality, cheap and affordable worth recommending to buy!	Jingdong prefecture level city in Shandong
Fortune Shop	Green Intelligent Power Saver	Very good quality, well made, works well! Satisfied	Jingdong prefecture level city in Shandong
Fortune Shop	Green Intelligent Power Saver	The logistics is very fast quality is very good very pleasant a shopping	Jingdong prefecture level city in Shandong
Fortune Shop	Green Intelligent Power Saver	Use it and then come back to evaluate!	Jingdong prefecture level city in Shandong
Fortune Shop	Green Intelligent Power Saver	# It's no use lying at all #	Jingdong prefecture level city in Shandong
Fortune Shop	Green Intelligent Power Saver	I can't get a voltage drop.	Jingdong prefecture level city in Shandong
Fortune Shop	Green Intelligent Power Saver	Evaluate after use	Jingdong prefecture level city in Shandong
Fortune Shop	Green Intelligent Power Saver	Still on trial.	Jingdong prefecture level city in Shandong
Fortune Shop	Green Intelligent Power Saver	ineffective	Jingdong prefecture level city in Shandong
Fortune Shop	Green Intelligent Power Saver	Satisfaction Shopping	Jingdong prefecture level city in Shandong
Fortune Shop	Green Intelligent Power Saver	Lies.	Jingdong prefecture level city in Shandong
Fortune Shop	Green Intelligent Power Saver	It's a real power saver.	Jingdong prefecture level city in Shandong
Fortune Shop	Green Intelligent Power Saver	in use	Jingdong prefecture level city in Shandong
Fortune Shop	Green Intelligent Power Saver	I don't know yet.	Jingdong prefecture level city in Shandong
Fortune Shop	Green Intelligent Power Saver	not care about anything	Jingdong prefecture level city in Shandong
Fortune Shop	Green Intelligent Power Saver	It doesn't seem to have much effect	Jingdong prefecture level city in Shandong
Fortune Shop	Green Intelligent Power Saver	Let the merchant see for himself, does the plug positive and negative terminals look like this?	Jingdong prefecture level city in Shandong
Fortune Shop	Green Intelligent Power Saver	It doesn't work well, the electricity bill is less before using it, but more after using it, contacting customer service, the service is still not good, and returning it is slow	Jingdong prefecture level city in Shandong
Fortune Shop	Green Intelligent Power Saver	With the advertisement product does not match, above the effective negative, power saving rate of two rows of words it, the product looks very thin, with 3 days to see the meter number is still with the previous no difference, which is obviously fraudulent behavior!	Jingdong prefecture level city in Shandong

Fortune Shop	Green Intelligent Power Saver	Doesn't save any power at all. Not much use.	Jingdong prefecture level city in Shandong
Fortune Shop	Green Intelligent Power Saver	The plugs are all loose, they're all the same length and they don't work at all	Jingdong prefecture level city in Shandong
Fortune Shop	Green Intelligent Power Saver	It's bad 👎 it's not effective at all, you'd be crazy to sell this garbage on the internet.	Jingdong prefecture level city in Shandong
Fortune Shop	Green Intelligent Power Saver	It doesn't save any power at all, so I hope the old timers don't fall for this scam again	Jingdong prefecture level city in Shandong
Fortune Shop	Green Intelligent Power Saver	I've been using it for over a month now and haven't noticed any power savings.	Jingdong prefecture level city in Shandong
Fortune Shop	Green Intelligent Power Saver	It doesn't work at all. The electricity bill is higher when you use it.	Jingdong prefecture level city in Shandong
Fortune Shop	Green Intelligent Power Saver	It doesn't save any power, it's still as bad as ever.	Jingdong prefecture level city in Shandong
Fortune Shop	Green Intelligent Power Saver	Last night before leaving work to look at the table, and then plugged into the node device, this morning and then look at the table, than the original save a few degrees of electricity, very good to have a power-saving effect, easy to use, satisfied with the shopping praise.	Jingdong prefecture level city in Shandong
Fortune Shop	Green Intelligent Power Saver	The product is real and effective, the electricity bill is reduced, really good, thanks to the merchants have so many good products, the product is real and effective, the electricity bill is reduced, want to save electricity friends quickly buy it, early use early to save money.	Jingdong prefecture level city in Shandong
Fortune Shop	Green Intelligent Power Saver	Saver saves time and money, baby appearance is beautiful, the quality of goods is good, and the same as the business description, good service attitude, packaging is complete, logistics is very fast, praise to	Jingdong prefecture level city in Shandong
Fortune Shop	Green Intelligent Power Saver	The effect is good, summer, home electricity is relatively large, buy and use a few days, feel the effect is very good, originally ten dollars a day electricity bill, now eight dollars a day, quite good	Jingdong prefecture level city in Shandong
Fortune Shop	Green Intelligent Power Saver	Indeed can save electricity and money, the first time I know this or from a friend's home to see, but also do not believe it, and now they use it to know that there is this good thing.	Jingdong prefecture level city in Shandong
Fortune Shop	Green Intelligent Power Saver	I have received the Smart Power Saver. Perfectly packaged. Fast delivery. Boss customer service attitude is also very good. It's worth buying. I plugged it in and tried it and it really saves more power than the original. I'll buy it again next time.	Jingdong prefecture level city in Shandong
Fortune Shop	Green Intelligent Power Saver	It's nice to save a dozen bucks a night on two air conditioners that used to take 30 or 40 degrees a night, but now it's only 10 or so degrees a night	Jingdong prefecture level city in Shandong
Fortune Shop	Green Intelligent Power Saver	Product no manufacturer's name no product qualification certificate, try two days to see the effect of	Jingdong prefecture level city in Shandong
Fortune Shop	Green Intelligent Power Saver	I've been using it for over a month and have saved over $80, so I've repurchased a few more back to save together	Jingdong prefecture level city in Shandong
Fortune Shop	Green Intelligent Power Saver	I haven't used it yet and will comment when I do, but it's well made.	Jingdong prefecture level city in Shandong
Fortune Shop	Green Intelligent Power Saver	It's quite energy efficient. It's really a boon for the home.	Jingdong prefecture level city in Shandong
Fortune Shop	Green Intelligent Power Saver	Energy-saving socket received a month, after using it for a month proved that it is indeed energy-saving, electricity bill saving, will buy it again, thank you!	Jingdong prefecture level city in Shandong
Fortune Shop	Green Intelligent Power Saver	There was a similar product many years ago. Now technology has evolved, buy one to try, according to the instructions in the box, it takes one or two dozen days to show results. Check your electric bill and see what happens.	Jingdong prefecture level city in Shandong
Fortune Shop	Green Intelligent Power Saver	Jingdong logistics is really fast, the same day the order arrived on the same day, more convenient than the supermarket, and a wide range of choices, the	Jingdong prefecture level city in Shandong
Fortune Shop	Green Intelligent Power Saver	I've been using this for a month or so and I can't see any results, it really is a scam as they say.	Jingdong prefecture level city in Shandong
Fortune Shop	Green Intelligent Power Saver	Very good use, a month can save a few degrees of power loss of electricity, introduced to the relatives to buy back to try.	Jingdong prefecture level city in Shandong
Fortune Shop	Green Intelligent Power Saver	It's really powerful! With this air conditioner can be turned on at any time without the pressure of paying the electricity bill! It's good for the people!	Jingdong prefecture level city in Shandong
Fortune Shop	Green Intelligent Power Saver	This business is a big ** products are not qualified, with a month of electricity more than one hundred percent of thirty, the purchase agreed to return the goods but do not receive a refund after receiving the goods, looking for the official side does not help, the Jingdong is a big **, I have never shopped in	Jingdong prefecture level city in Shandong
Fortune Shop	Green Intelligent Power Saver	It's good. I tried it. It saves power.	Jingdong prefecture level city in Shandong
Fortune Shop	Green Intelligent Power Saver	Nothing**, it seems like there's more	Jingdong prefecture level city in Shandong
Fortune Shop	Green Intelligent Power Saver	Power saving device received, well packaged, good quality, fast logistics	Jingdong prefecture level city in Shandong
Fortune Shop	Green Intelligent Power Saver	The service is okay, is that when I bought five, the desirable pieces are three, and then wait a few days and send me two replacement, I do not know if it is a mistake of the shipper, if I was not in the courier there to see, go home to say no, the	Jingdong prefecture level city in Shandong
Fortune Shop	Green Intelligent Power Saver	This thing will only cost me more if I use it, but before I used it, it was less than a ~ electricity a month, and after I used it, it was more than two hundred kWh a month.	Jingdong prefecture level city in Shandong
Fortune Shop	Green Intelligent Power Saver	I just used it. I don't know how it is.	Jingdong prefecture level city in Shandong
Fortune Shop	Green Intelligent Power Saver	I've received the product and I'm using it, so I'll see how it goes in a month!	Jingdong prefecture level city in Shandong
Fortune Shop	Green Intelligent Power Saver	The goods have just received the effect do not know how, please look forward to the results.	Jingdong prefecture level city in Shandong
Fortune Shop	Green Intelligent Power Saver	Power saving device, the material is very good practical and durable, the size is suitable, packaging is very good, the price is cheap, logistics is very fast!	Jingdong prefecture level city in Shandong
Fortune Shop	Green Intelligent Power Saver	Very nice, will comment in detail after using it for a while.	Jingdong prefecture level city in Shandong
Fortune Shop	Green Intelligent Power Saver	Good stuff to trust and support	Jingdong prefecture level city in Shandong
Fortune Shop	Green Intelligent Power Saver	I'll comment after I use it, doubt if it really saves power no	Jingdong prefecture level city in Shandong
Fortune Shop	Green Intelligent Power Saver	It's good quality and works well!	Jingdong prefecture level city in Shandong
Fortune Shop	Green Intelligent Power Saver	hahaha just right hahahahahaha hiccup hahahaha	Jingdong prefecture level city in Shandong
Fortune Shop	Green Intelligent Power Saver	The real thing matches the picture and looks great!	Jingdong prefecture level city in Shandong
Fortune Shop	Green Intelligent Power Saver	Plugged it in and we'll see when we pay the bills at the end of the year!	Jingdong prefecture level city in Shandong
Fortune Shop	Green Intelligent Power Saver	False advertising. It doesn't work.	Jingdong prefecture level city in Shandong
Fortune Shop	Green Intelligent Power Saver	Goods received, good effect	Jingdong prefecture level city in Shandong
Fortune Shop	Green Intelligent Power Saver	It looks okay, we'll see if it saves electricity when the time comes	Jingdong prefecture level city in Shandong
Fortune Shop	Green Intelligent Power Saver	Logistics is very fast quality is very good, the price is very affordable, accessories are also complete, installed, satisfied with good use	Jingdong prefecture level city in Shandong
Fortune Shop	Green Intelligent Power Saver	I've received the power saver. I'm looking forward to the savings.	Jingdong prefecture level city in Shandong
Fortune Shop	Green Intelligent Power Saver	Received, have not yet used, after use and then evaluate	Jingdong prefecture level city in Shandong
Fortune Shop	Green Intelligent Power Saver	I received the item, but I don't know if it saves power or not.	Jingdong prefecture level city in Shandong

Fortune Shop	Green Intelligent Power Saver	On Trial	Jingdong prefecture level city in Shandong
Fortune Shop	Green Intelligent Power Saver	Use it and see how it goes.	Jingdong prefecture level city in Shandong
Fortune Shop	Green Intelligent Power Saver	Quality is very good, no odor, fine workmanship, grain color are very like, satisfied!	Jingdong prefecture level city in Shandong
Fortune Shop	Green Intelligent Power Saver	I'll give you a good review. I don't know if it'll work.	Jingdong prefecture level city in Shandong
Fortune Shop	Green Intelligent Power Saver	The logistics is very fast and the package is intact. Good use of affordable. The effect is very good, is genuine	Jingdong prefecture level city in Shandong
Fortune Shop	Green Intelligent Power Saver	Nothing happens when you plug it in.	Jingdong prefecture level city in Shandong
Fortune Shop	Green Intelligent Power Saver	I don't know if it's as good as it sounds.	Jingdong prefecture level city in Shandong
Fortune Shop	Green Intelligent Power Saver	The quality of the stuff is very good, exactly the same as the seller's description, very satisfied!	Jingdong prefecture level city in Shandong
Fortune Shop	Green Intelligent Power Saver	The saver works great, it really works, it's affordable and worth buying.	Jingdong prefecture level city in Shandong
Fortune Shop	Green Intelligent Power Saver	Just used it for a couple days. I don't know yet.	Jingdong prefecture level city in Shandong
Fortune Shop	Green Intelligent Power Saver	The quality is quite good, cheaper than the physical store, good, next time will come again!	Jingdong prefecture level city in Shandong
Fortune Shop	Green Intelligent Power Saver	Good quality, cheap and affordable worth recommending to buy!	Jingdong prefecture level city in Shandong
Fortune Shop	Green Intelligent Power Saver	Very good quality, well made, works well! Satisfied	Jingdong prefecture level city in Shandong
Fortune Shop	Green Intelligent Power Saver	The logistics is very fast quality is very good very pleasant a shopping	Jingdong prefecture level city in Shandong
Fortune Shop	Green Intelligent Power Saver	Use it and then come back to evaluate!	Jingdong prefecture level city in Shandong
Fortune Shop	Green Intelligent Power Saver	# It's no use lying at all #	Jingdong prefecture level city in Shandong
Fortune Shop	Green Intelligent Power Saver	I can't get a voltage drop.	Jingdong prefecture level city in Shandong
Fortune Shop	Green Intelligent Power Saver	Evaluate after use	Jingdong prefecture level city in Shandong
Fortune Shop	Green Intelligent Power Saver	Still on trial.	Jingdong prefecture level city in Shandong
Fortune Shop	Green Intelligent Power Saver	ineffective	Jingdong prefecture level city in Shandong
Fortune Shop	Green Intelligent Power Saver	Satisfaction Shopping	Jingdong prefecture level city in Shandong
Fortune Shop	Green Intelligent Power Saver	Lies.	Jingdong prefecture level city in Shandong
Fortune Shop	Green Intelligent Power Saver	It's a real power saver.	Jingdong prefecture level city in Shandong
Fortune Shop	Green Intelligent Power Saver	in use	Jingdong prefecture level city in Shandong
Fortune Shop	Green Intelligent Power Saver	I don't know yet.	Jingdong prefecture level city in Shandong
Fortune Shop	Green Intelligent Power Saver	not care about anything	Jingdong prefecture level city in Shandong
Fortune Shop	Green Intelligent Power Saver	It doesn't seem to have much effect	Jingdong prefecture level city in Shandong
Fortune Shop	Green Intelligent Power Saver	Let the merchant see for himself, does the plug positive and negative terminals look like this?	Jingdong prefecture level city in Shandong
Fortune Shop	Green Intelligent Power Saver	It doesn't work well, the electricity bill is less before using it, but more after using it, contacting customer service, the service is still not good, and returning it is slow	Jingdong prefecture level city in Shandong
Fortune Shop	Green Intelligent Power Saver	With the advertisement product does not match, above the effective negative, power saving rate of two rows of words it, the product looks very thin, with 3 days to see the meter number is still with the previous no difference, which is obviously fraudulent behavior!	Jingdong prefecture level city in Shandong
Fortune Shop	Green Intelligent Power Saver	Doesn't save any power at all. Not much use.	Jingdong prefecture level city in Shandong
Fortune Shop	Green Intelligent Power Saver	The plugs are all loose, they're all the same length and they don't work at all	Jingdong prefecture level city in Shandong
Fortune Shop	Green Intelligent Power Saver	It's bad 👎 it's not effective at all, you'd be crazy to sell this garbage on the internet.	Jingdong prefecture level city in Shandong
Fortune Shop	Green Intelligent Power Saver	It doesn't save any power at all, so I hope the old timers don't fall for this scam again	Jingdong prefecture level city in Shandong
Fortune Shop	Green Intelligent Power Saver	I've been using it for over a month now and haven't noticed any power savings.	Jingdong prefecture level city in Shandong
Fortune Shop	Green Intelligent Power Saver	It doesn't work at all. The electricity bill is higher when you use it.	Jingdong prefecture level city in Shandong
Fortune Shop	Green Intelligent Power Saver	It doesn't save any power, it's still as bad as ever.	Jingdong prefecture level city in Shandong
Fortune Shop	Green Intelligent Power Saver	Last night before leaving work to look at the table, and then plugged into the node device, this morning and then look at the table, than the original save a few degrees of electricity, very good to have a power-saving effect, easy to use, satisfied with the shopping praise.	Jingdong prefecture level city in Shandong
Fortune Shop	Green Intelligent Power Saver	The product is real and effective, the electricity bill is reduced, really good, thanks to the merchants have so many good products, the product is real and effective, the electricity bill is reduced, want to save electricity friends quickly buy it, early use early to save money.	Jingdong prefecture level city in Shandong
Fortune Shop	Green Intelligent Power Saver	Saver saves time and money, baby appearance is beautiful, the quality of goods is good, and the same as the business description, good service attitude, packaging is complete, logistics is very fast, praise to	Jingdong prefecture level city in Shandong
Fortune Shop	Green Intelligent Power Saver	The effect is good, summer, home electricity is relatively large, buy and use a few days, feel the effect is very good, originally ten dollars a day electricity bill, now eight dollars a day, quite good	Jingdong prefecture level city in Shandong
Fortune Shop	Green Intelligent Power Saver	Indeed can save electricity and money, the first time I know this or from a friend's home to see, but also do not believe it, and now they use it to know that there is this good thing.	Jingdong prefecture level city in Shandong
Fortune Shop	Green Intelligent Power Saver	I have received the Smart Power Saver. Perfectly packaged. Fast delivery. Boss customer service attitude is also very good. It's worth buying. I plugged it in and tried it and it really saves more power than the original. I'll buy it again next time.	Jingdong prefecture level city in Shandong
Fortune Shop	Green Intelligent Power Saver	It's nice to save a dozen bucks a night on two air conditioners that used to take 30 or 40 degrees a night, but now it's only 10 or so degrees a night	Jingdong prefecture level city in Shandong
Fortune Shop	Green Intelligent Power Saver	Product no manufacturer's name no product qualification certificate, try two days to see the effect of	Jingdong prefecture level city in Shandong
Fortune Shop	Green Intelligent Power Saver	I've been using it for over a month and have saved over $80, so I've repurchased a few more back to save together	Jingdong prefecture level city in Shandong
Fortune Shop	Green Intelligent Power Saver	I haven't used it yet and will comment when I do, but it's well made.	Jingdong prefecture level city in Shandong
Fortune Shop	Green Intelligent Power Saver	It's quite energy efficient. It's really a boon for the home.	Jingdong prefecture level city in Shandong
Fortune Shop	Green Intelligent Power Saver	Energy-saving socket received a month, after using it for a month proved that it is indeed energy-saving, electricity bill saving, will buy it again, thank you!	Jingdong prefecture level city in Shandong

Fortune Shop	Green Intelligent Power Saver	There was a similar product many years ago. Now technology has evolved, buy one to try, according to the instructions in the box, it takes one or two dozen days to show results. Check your electric bill and see what happens.	Jingdong prefecture level city in Shandong
Fortune Shop	Green Intelligent Power Saver	Jingdong logistics is really fast, the same day the order arrived on the same day, more convenient than the supermarket, and a wide range of choices, the	Jingdong prefecture level city in Shandong
Fortune Shop	Green Intelligent Power Saver	I've been using this for a month or so and I can't see any results, it really is a scam as they say.	Jingdong prefecture level city in Shandong
Fortune Shop	Green Intelligent Power Saver	Very good use, a month can save a few degrees of power loss of electricity, introduced to the relatives to buy back to try.	Jingdong prefecture level city in Shandong
Fortune Shop	Green Intelligent Power Saver	It's really powerful! With this air conditioner can be turned on at any time without the pressure of paying the electricity bill! It's good for the people!	Jingdong prefecture level city in Shandong
Fortune Shop	Green Intelligent Power Saver	This business is a big ** products are not qualified, with a month of electricity more than one hundred percent of thirty, the purchase agreed to return the goods but do not receive a refund after receiving the goods, looking for the official side does not help, the Jingdong is a big **, I have never shopped in	Jingdong prefecture level city in Shandong
Fortune Shop	Green Intelligent Power Saver	It's good. I tried it. It saves power.	Jingdong prefecture level city in Shandong
Fortune Shop	Green Intelligent Power Saver	Nothing**, it seems like there's more	Jingdong prefecture level city in Shandong
Fortune Shop	Green Intelligent Power Saver	Power saving device received, well packaged, good quality, fast logistics	Jingdong prefecture level city in Shandong
Fortune Shop	Green Intelligent Power Saver	The service is okay, is that when I bought five, the desirable pieces are three, and then wait a few days and send me two replacement, I do not know if it is a mistake of the shipper, if I was not in the courier there to see, go home to say no, the	Jingdong prefecture level city in Shandong
Fortune Shop	Green Intelligent Power Saver	This thing will only cost me more if I use it, but before I used it, it was less than a ~ electricity a month, and after I used it, it was more than two hundred kWh a month.	Jingdong prefecture level city in Shandong
Fortune Shop	Green Intelligent Power Saver	I just used it. I don't know how it is.	Jingdong prefecture level city in Shandong
Fortune Shop	Green Intelligent Power Saver	I've received the product and I'm using it, so I'll see how it goes in a month!	Jingdong prefecture level city in Shandong
Fortune Shop	Green Intelligent Power Saver	The goods have just received the effect do not know how, please look forward to the results.	Jingdong prefecture level city in Shandong
Fortune Shop	Green Intelligent Power Saver	Power saving device, the material is very good practical and durable, the size is suitable, packaging is very good, the price is cheap, logistics is very fast!	Jingdong prefecture level city in Shandong
Fortune Shop	Green Intelligent Power Saver	Very nice, will comment in detail after using it for a while.	Jingdong prefecture level city in Shandong
Fortune Shop	Green Intelligent Power Saver	Good stuff to trust and support	Jingdong prefecture level city in Shandong
Fortune Shop	Green Intelligent Power Saver	I'll comment after I use it, doubt if it really saves power no	Jingdong prefecture level city in Shandong
Fortune Shop	Green Intelligent Power Saver	It's good quality and works well!	Jingdong prefecture level city in Shandong
Fortune Shop	Green Intelligent Power Saver	hahaha just right hahahahahaha hiccup hahahaha	Jingdong prefecture level city in Shandong
Fortune Shop	Green Intelligent Power Saver	The real thing matches the picture and looks great!	Jingdong prefecture level city in Shandong
Fortune Shop	Green Intelligent Power Saver	Plugged it in and we'll see when we pay the bills at the end of the year!	Jingdong prefecture level city in Shandong
Fortune Shop	Green Intelligent Power Saver	False advertising. It doesn't work.	Jingdong prefecture level city in Shandong
Fortune Shop	Green Intelligent Power Saver	Goods received, good effect	Jingdong prefecture level city in Shandong
Fortune Shop	Green Intelligent Power Saver	It looks okay, we'll see if it saves electricity when the time comes	Jingdong prefecture level city in Shandong
Fortune Shop	Green Intelligent Power Saver	Logistics is very fast quality is very good, the price is very affordable, accessories are also complete, installed, satisfied with good use	Jingdong prefecture level city in Shandong
Fortune Shop	Green Intelligent Power Saver	I've received the power saver. I'm looking forward to the savings.	Jingdong prefecture level city in Shandong
Fortune Shop	Green Intelligent Power Saver	Received, have not yet used, after use and then evaluate	Jingdong prefecture level city in Shandong
Fortune Shop	Green Intelligent Power Saver	I received the item, but I don't know if it saves power or not.	Jingdong prefecture level city in Shandong
Fortune Shop	Green Intelligent Power Saver	On Trial	Jingdong prefecture level city in Shandong
Fortune Shop	Green Intelligent Power Saver	Use it and see how it goes.	Jingdong prefecture level city in Shandong
Fortune Shop	Green Intelligent Power Saver	Quality is very good, no odor, fine workmanship, grain color are very like, satisfied!	Jingdong prefecture level city in Shandong
Fortune Shop	Green Intelligent Power Saver	I'll give you a good review. I don't know if it'll work.	Jingdong prefecture level city in Shandong
Fortune Shop	Green Intelligent Power Saver	The logistics is very fast and the package is intact. Good use of affordable. The effect is very good, is genuine	Jingdong prefecture level city in Shandong
Fortune Shop	Green Intelligent Power Saver	Nothing happens when you plug it in.	Jingdong prefecture level city in Shandong
Fortune Shop	Green Intelligent Power Saver	I don't know if it's as good as it sounds.	Jingdong prefecture level city in Shandong
Fortune Shop	Green Intelligent Power Saver	The quality of the stuff is very good, exactly the same as the seller's description, very satisfied!	Jingdong prefecture level city in Shandong
Fortune Shop	Green Intelligent Power Saver	The saver works great, it really works, it's affordable and worth buying.	Jingdong prefecture level city in Shandong
Fortune Shop	Green Intelligent Power Saver	Just used it for a couple days. I don't know yet.	Jingdong prefecture level city in Shandong
Fortune Shop	Green Intelligent Power Saver	The quality is quite good, cheaper than the physical store, good, next time will come again!	Jingdong prefecture level city in Shandong
Fortune Shop	Green Intelligent Power Saver	Good quality, cheap and affordable worth recommending to buy!	Jingdong prefecture level city in Shandong
Fortune Shop	Green Intelligent Power Saver	Very good quality, well made, works well! Satisfied	Jingdong prefecture level city in Shandong
Fortune Shop	Green Intelligent Power Saver	The logistics is very fast quality is very good very pleasant a shopping	Jingdong prefecture level city in Shandong
Fortune Shop	Green Intelligent Power Saver	Use it and then come back to evaluate!	Jingdong prefecture level city in Shandong
Fortune Shop	Green Intelligent Power Saver	# It's no use lying at all #	Jingdong prefecture level city in Shandong
Fortune Shop	Green Intelligent Power Saver	I can't get a voltage drop.	Jingdong prefecture level city in Shandong
Fortune Shop	Green Intelligent Power Saver	Evaluate after use	Jingdong prefecture level city in Shandong
Fortune Shop	Green Intelligent Power Saver	Still on trial.	Jingdong prefecture level city in Shandong
Fortune Shop	Green Intelligent Power Saver	ineffective	Jingdong prefecture level city in Shandong
Fortune Shop	Green Intelligent Power Saver	Satisfaction Shopping	Jingdong prefecture level city in Shandong

Fortune Shop	Green Intelligent Power Saver	Lies.	Jingdong prefecture level city in Shandong
Fortune Shop	Green Intelligent Power Saver	It's a real power saver.	Jingdong prefecture level city in Shandong
Fortune Shop	Green Intelligent Power Saver	in use	Jingdong prefecture level city in Shandong
Fortune Shop	Green Intelligent Power Saver	I don't know yet.	Jingdong prefecture level city in Shandong
Fortune Shop	Green Intelligent Power Saver	not care about anything	Jingdong prefecture level city in Shandong
Fortune Shop	Green Intelligent Power Saver	It doesn't seem to have much effect	Jingdong prefecture level city in Shandong
Fortune Shop	Green Intelligent Power Saver	Let the merchant see for himself, does the plug positive and negative terminals look like this?	Jingdong prefecture level city in Shandong
Fortune Shop	Green Intelligent Power Saver	It doesn't work well, the electricity bill is less before using it, but more after using it, contacting customer service, the service is still not good, and returning it is slow	Jingdong prefecture level city in Shandong
Fortune Shop	Green Intelligent Power Saver	With the advertisement product does not match, above the effective negative, power saving rate of two rows of words it, the product looks very thin, with 3 days to see the meter number is still with the previous no difference, which is obviously fraudulent behavior!	Jingdong prefecture level city in Shandong
Fortune Shop	Green Intelligent Power Saver	Doesn't save any power at all. Not much use.	Jingdong prefecture level city in Shandong
Fortune Shop	Green Intelligent Power Saver	The plugs are all loose, they're all the same length and they don't work at all	Jingdong prefecture level city in Shandong
Fortune Shop	Green Intelligent Power Saver	It's bad 👎 it's not effective at all, you'd be crazy to sell this garbage on the internet.	Jingdong prefecture level city in Shandong
Fortune Shop	Green Intelligent Power Saver	It doesn't save any power at all, so I hope the old timers don't fall for this scam again	Jingdong prefecture level city in Shandong
Fortune Shop	Green Intelligent Power Saver	I've been using it for over a month now and haven't noticed any power savings.	Jingdong prefecture level city in Shandong
Fortune Shop	Green Intelligent Power Saver	It doesn't work at all. The electricity bill is higher when you use it.	Jingdong prefecture level city in Shandong
Fortune Shop	Green Intelligent Power Saver	It doesn't save any power, it's still as bad as ever.	Jingdong prefecture level city in Shandong
Fortune Shop	Green Intelligent Power Saver	Last night before leaving work to look at the table, and then plugged into the node device, this morning and then look at the table, than the original save a few degrees of electricity, very good to have a power-saving effect, easy to use, satisfied with the shopping praise.	Jingdong prefecture level city in Shandong
Fortune Shop	Green Intelligent Power Saver	The product is real and effective, the electricity bill is reduced, really good, thanks to the merchants have so many good products, the product is real and effective, the electricity bill is reduced, want to save electricity friends quickly buy it, early use early to save money.	Jingdong prefecture level city in Shandong
Fortune Shop	Green Intelligent Power Saver	Saver saves time and money, baby appearance is beautiful, the quality of goods is good, and the same as the business description, good service attitude, packaging is complete, logistics is very fast, praise to	Jingdong prefecture level city in Shandong
Fortune Shop	Green Intelligent Power Saver	The effect is good, summer, home electricity is relatively large, buy and use a few days, feel the effect is very good, originally ten dollars a day electricity bill, now eight dollars a day, quite good	Jingdong prefecture level city in Shandong
Fortune Shop	Green Intelligent Power Saver	Indeed can save electricity and money, the first time I know this or from a friend's home to see, but also do not believe it, and now they use it to know that there is this good thing.	Jingdong prefecture level city in Shandong
Fortune Shop	Green Intelligent Power Saver	I have received the Smart Power Saver. Perfectly packaged. Fast delivery. Boss customer service attitude is also very good. It's worth buying. I plugged it in and tried it and it really saves more power than the original. I'll buy it again next time.	Jingdong prefecture level city in Shandong
Fortune Shop	Green Intelligent Power Saver	It's nice to save a dozen bucks a night on two air conditioners that used to take 30 or 40 degrees a night, but now it's only 10 or so degrees a night	Jingdong prefecture level city in Shandong
Fortune Shop	Green Intelligent Power Saver	Product no manufacturer's name no product qualification certificate, try two days to see the effect of	Jingdong prefecture level city in Shandong
Fortune Shop	Green Intelligent Power Saver	I've been using it for over a month and have saved over $80, so I've repurchased a few more back to save together	Jingdong prefecture level city in Shandong
Fortune Shop	Green Intelligent Power Saver	I haven't used it yet and will comment when I do, but it's well made.	Jingdong prefecture level city in Shandong
Fortune Shop	Green Intelligent Power Saver	It's quite energy efficient. It's really a boon for the home.	Jingdong prefecture level city in Shandong
Fortune Shop	Green Intelligent Power Saver	Energy-saving socket received a month, after using it for a month proved that it is indeed energy-saving, electricity bill saving, will buy it again, thank you!	Jingdong prefecture level city in Shandong
Fortune Shop	Green Intelligent Power Saver	There was a similar product many years ago. Now technology has evolved, buy one to try, according to the instructions in the box, it takes one or two dozen days to show results. Check your electric bill and see what happens.	Jingdong prefecture level city in Shandong
Fortune Shop	Green Intelligent Power Saver	Jingdong logistics is really fast, the same day the order arrived on the same day, more convenient than the supermarket, and a wide range of choices, the	Jingdong prefecture level city in Shandong
Fortune Shop	Green Intelligent Power Saver	I've been using this for a month or so and I can't see any results, it really is a scam as they say.	Jingdong prefecture level city in Shandong
Fortune Shop	Green Intelligent Power Saver	Very good use, a month can save a few degrees of power loss of electricity, introduced to the relatives to buy back to try.	Jingdong prefecture level city in Shandong
Fortune Shop	Green Intelligent Power Saver	It's really powerful! With this air conditioner can be turned on at any time without the pressure of paying the electricity bill! It's good for the people!	Jingdong prefecture level city in Shandong
Fortune Shop	Green Intelligent Power Saver	This business is a big ** products are not qualified, with a month of electricity more than one hundred percent of thirty, the purchase agreed to return the goods but do not receive a refund after receiving the goods, looking for the official side does not help, the Jingdong is a big **, I have never shopped in	Jingdong prefecture level city in Shandong
Fortune Shop	Green Intelligent Power Saver	It's good. I tried it. It saves power.	Jingdong prefecture level city in Shandong
Fortune Shop	Green Intelligent Power Saver	Nothing**, it seems like there's more	Jingdong prefecture level city in Shandong
Fortune Shop	Green Intelligent Power Saver	Power saving device received, well packaged, good quality, fast logistics	Jingdong prefecture level city in Shandong
Fortune Shop	Green Intelligent Power Saver	The service is okay, is that when I bought five, the desirable pieces are three, and then wait a few days and send me two replacement, I do not know if it is a mistake of the shipper, if I was not in the courier there to see, go home to say no, the	Jingdong prefecture level city in Shandong
Fortune Shop	Green Intelligent Power Saver	This thing will only cost me more if I use it, but before I used it, it was less than a ~ electricity a month, and after I used it, it was more than two hundred kWh a month.	Jingdong prefecture level city in Shandong
Fortune Shop	Green Intelligent Power Saver	I just used it. I don't know how it is.	Jingdong prefecture level city in Shandong
Fortune Shop	Green Intelligent Power Saver	I've received the product and I'm using it, so I'll see how it goes in a month!	Jingdong prefecture level city in Shandong
Fortune Shop	Green Intelligent Power Saver	The goods have just received the effect do not know how, please look forward to the results.	Jingdong prefecture level city in Shandong
Fortune Shop	Green Intelligent Power Saver	Power saving device, the material is very good practical and durable, the size is suitable, packaging is very good, the price is cheap, logistics is very fast!	Jingdong prefecture level city in Shandong
Fortune Shop	Green Intelligent Power Saver	Very nice, will comment in detail after using it for a while.	Jingdong prefecture level city in Shandong
Fortune Shop	Green Intelligent Power Saver	Good stuff to trust and support	Jingdong prefecture level city in Shandong
Fortune Shop	Green Intelligent Power Saver	I'll comment after I use it, doubt if it really saves power no	Jingdong prefecture level city in Shandong
Fortune Shop	Green Intelligent Power Saver	It's good quality and works well!	Jingdong prefecture level city in Shandong
Fortune Shop	Green Intelligent Power Saver	hahaha just right hahahahahaha hiccup hahahaha	Jingdong prefecture level city in Shandong

Fortune Shop	Green Intelligent Power Saver	The real thing matches the picture and looks great!	Jingdong prefecture level city in Shandong
Fortune Shop	Green Intelligent Power Saver	Plugged it in and we'll see when we pay the bills at the end of the year!	Jingdong prefecture level city in Shandong
Fortune Shop	Green Intelligent Power Saver	False advertising. It doesn't work.	Jingdong prefecture level city in Shandong
Fortune Shop	Green Intelligent Power Saver	Goods received, good effect	Jingdong prefecture level city in Shandong
Fortune Shop	Green Intelligent Power Saver	It looks okay, we'll see if it saves electricity when the time comes	Jingdong prefecture level city in Shandong
Fortune Shop	Green Intelligent Power Saver	Logistics is very fast quality is very good, the price is very affordable, accessories are also complete, installed, satisfied with good use	Jingdong prefecture level city in Shandong
Fortune Shop	Green Intelligent Power Saver	I've received the power saver. I'm looking forward to the savings.	Jingdong prefecture level city in Shandong
Fortune Shop	Green Intelligent Power Saver	Received, have not yet used, after use and then evaluate	Jingdong prefecture level city in Shandong
Fortune Shop	Green Intelligent Power Saver	I received the item, but I don't know if it saves power or not.	Jingdong prefecture level city in Shandong
Fortune Shop	Green Intelligent Power Saver	On Trial	Jingdong prefecture level city in Shandong
Fortune Shop	Green Intelligent Power Saver	Use it and see how it goes.	Jingdong prefecture level city in Shandong
Fortune Shop	Green Intelligent Power Saver	Quality is very good, no odor, fine workmanship, grain color are very like, satisfied!	Jingdong prefecture level city in Shandong
Fortune Shop	Green Intelligent Power Saver	I'll give you a good review. I don't know if it'll work.	Jingdong prefecture level city in Shandong
Fortune Shop	Green Intelligent Power Saver	The logistics is very fast and the package is intact. Good use of affordable. The effect is very good, is genuine	Jingdong prefecture level city in Shandong
Fortune Shop	Green Intelligent Power Saver	Nothing happens when you plug it in.	Jingdong prefecture level city in Shandong
Fortune Shop	Green Intelligent Power Saver	I don't know if it's as good as it sounds.	Jingdong prefecture level city in Shandong
Fortune Shop	Green Intelligent Power Saver	The quality of the stuff is very good, exactly the same as the seller's description, very satisfied!	Jingdong prefecture level city in Shandong
Fortune Shop	Green Intelligent Power Saver	The saver works great, it really works, it's affordable and worth buying.	Jingdong prefecture level city in Shandong
Fortune Shop	Green Intelligent Power Saver	Just used it for a couple days. I don't know yet.	Jingdong prefecture level city in Shandong
Fortune Shop	Green Intelligent Power Saver	The quality is quite good, cheaper than the physical store, good, next time will come again!	Jingdong prefecture level city in Shandong
Fortune Shop	Green Intelligent Power Saver	Good quality, cheap and affordable worth recommending to buy!	Jingdong prefecture level city in Shandong
Fortune Shop	Green Intelligent Power Saver	Very good quality, well made, works well! Satisfied	Jingdong prefecture level city in Shandong
Fortune Shop	Green Intelligent Power Saver	The logistics is very fast quality is very good very pleasant a shopping	Jingdong prefecture level city in Shandong
Fortune Shop	Green Intelligent Power Saver	Use it and then come back to evaluate!	Jingdong prefecture level city in Shandong
Fortune Shop	Green Intelligent Power Saver	# It's no use lying at all #	Jingdong prefecture level city in Shandong
Fortune Shop	Green Intelligent Power Saver	I can't get a voltage drop.	Jingdong prefecture level city in Shandong
Fortune Shop	Green Intelligent Power Saver	Evaluate after use	Jingdong prefecture level city in Shandong
Fortune Shop	Green Intelligent Power Saver	Still on trial.	Jingdong prefecture level city in Shandong
Fortune Shop	Green Intelligent Power Saver	ineffective	Jingdong prefecture level city in Shandong
Fortune Shop	Green Intelligent Power Saver	Satisfaction Shopping	Jingdong prefecture level city in Shandong
Fortune Shop	Green Intelligent Power Saver	Lies.	Jingdong prefecture level city in Shandong
Fortune Shop	Green Intelligent Power Saver	It's a real power saver.	Jingdong prefecture level city in Shandong
Fortune Shop	Green Intelligent Power Saver	in use	Jingdong prefecture level city in Shandong
Fortune Shop	Green Intelligent Power Saver	I don't know yet.	Jingdong prefecture level city in Shandong
Fortune Shop	Green Intelligent Power Saver	not care about anything	Jingdong prefecture level city in Shandong
Fortune Shop	Green Intelligent Power Saver	It doesn't seem to have much effect	Jingdong prefecture level city in Shandong
Fortune Shop	Green Intelligent Power Saver	Let the merchant see for himself, does the plug positive and negative terminals look like this?	Jingdong prefecture level city in Shandong
Fortune Shop	Green Intelligent Power Saver	It doesn't work well, the electricity bill is less before using it, but more after using it, contacting customer service, the service is still not good, and returning it is slow	Jingdong prefecture level city in Shandong
Fortune Shop	Green Intelligent Power Saver	With the advertisement product does not match, above the effective negative, power saving rate of two rows of words it, the product looks very thin, with 3 days to see the meter number is still with the previous no difference, which is obviously fraudulent behavior!	Jingdong prefecture level city in Shandong
Fortune Shop	Green Intelligent Power Saver	Doesn't save any power at all. Not much use.	Jingdong prefecture level city in Shandong
Fortune Shop	Green Intelligent Power Saver	The plugs are all loose, they're all the same length and they don't work at all	Jingdong prefecture level city in Shandong
Fortune Shop	Green Intelligent Power Saver	It's bad 👎 it's not effective at all, you'd be crazy to sell this garbage on the internet.	Jingdong prefecture level city in Shandong
Fortune Shop	Green Intelligent Power Saver	It doesn't save any power at all, so I hope the old timers don't fall for this scam again	Jingdong prefecture level city in Shandong
Fortune Shop	Green Intelligent Power Saver	I've been using it for over a month now and haven't noticed any power savings.	Jingdong prefecture level city in Shandong
Fortune Shop	Green Intelligent Power Saver	It doesn't work at all. The electricity bill is higher when you use it.	Jingdong prefecture level city in Shandong
Fortune Shop	Green Intelligent Power Saver	It doesn't save any power, it's still as bad as ever.	Jingdong prefecture level city in Shandong
Fortune Shop	Green Intelligent Power Saver	Last night before leaving work to look at the table, and then plugged into the node device, this morning and then look at the table, than the original save a few degrees of electricity, very good to have a power-saving effect, easy to use, satisfied with the shopping praise.	Jingdong prefecture level city in Shandong
Fortune Shop	Green Intelligent Power Saver	The product is real and effective, the electricity bill is reduced, really good, thanks to the merchants have so many good products, the product is real and effective, the electricity bill is reduced, want to save electricity friends quickly buy it, early use early to save money.	Jingdong prefecture level city in Shandong
Fortune Shop	Green Intelligent Power Saver	Saver saves time and money, baby appearance is beautiful, the quality of goods is good, and the same as the business description, good service attitude, packaging is complete, logistics is very fast, praise to	Jingdong prefecture level city in Shandong
Fortune Shop	Green Intelligent Power Saver	The effect is good, summer, home electricity is relatively large, buy and use a few days, feel the effect is very good, originally ten dollars a day electricity bill, now eight dollars a day, quite good	Jingdong prefecture level city in Shandong

Fortune Shop	Green Intelligent Power Saver	Indeed can save electricity and money, the first time I know this or from a friend's home to see, but also do not believe it, and now they use it to know that there is this good thing.	Jingdong prefecture level city in Shandong
Fortune Shop	Green Intelligent Power Saver	I have received the Smart Power Saver. Perfectly packaged. Fast delivery. Boss customer service attitude is also very good. It's worth buying. I plugged it in and tried it and it really saves more power than the original. I'll buy it again next time.	Jingdong prefecture level city in Shandong
Fortune Shop	Green Intelligent Power Saver	It's nice to save a dozen bucks a night on two air conditioners that used to take 30 or 40 degrees a night, but now it's only 10 or so degrees a night	Jingdong prefecture level city in Shandong
Fortune Shop	Green Intelligent Power Saver	Product no manufacturer's name no product qualification certificate, try two days to see the effect of	Jingdong prefecture level city in Shandong
Fortune Shop	Green Intelligent Power Saver	I've been using it for over a month and have saved over $80, so I've repurchased a few more back to save together	Jingdong prefecture level city in Shandong
Fortune Shop	Green Intelligent Power Saver	I haven't used it yet and will comment when I do, but it's well made.	Jingdong prefecture level city in Shandong
Fortune Shop	Green Intelligent Power Saver	It's quite energy efficient. It's really a boon for the home.	Jingdong prefecture level city in Shandong
Fortune Shop	Green Intelligent Power Saver	Energy-saving socket received a month, after using it for a month proved that it is indeed energy-saving, electricity bill saving, will buy it again, thank you!	Jingdong prefecture level city in Shandong
Fortune Shop	Green Intelligent Power Saver	There was a similar product many years ago. Now technology has evolved, buy one to try, according to the instructions in the box, it takes one or two dozen days to show results. Check your electric bill and see what happens.	Jingdong prefecture level city in Shandong
Fortune Shop	Green Intelligent Power Saver	Jingdong logistics is really fast, the same day the order arrived on the same day, more convenient than the supermarket, and a wide range of choices, the	Jingdong prefecture level city in Shandong
Fortune Shop	Green Intelligent Power Saver	I've been using this for a month or so and I can't see any results, it really is a scam as they say.	Jingdong prefecture level city in Shandong
Fortune Shop	Green Intelligent Power Saver	Very good use, a month can save a few degrees of power loss of electricity, introduced to the relatives to buy back to try.	Jingdong prefecture level city in Shandong
Fortune Shop	Green Intelligent Power Saver	It's really powerful! With this air conditioner can be turned on at any time without the pressure of paying the electricity bill! It's good for the people!	Jingdong prefecture level city in Shandong
Fortune Shop	Green Intelligent Power Saver	This business is a big ** products are not qualified, with a month of electricity more than one hundred percent of thirty, the purchase agreed to return the goods but do not receive a refund after receiving the goods, looking for the official side does not help, the Jingdong is a big **, I have never shopped in	Jingdong prefecture level city in Shandong
Fortune Shop	Green Intelligent Power Saver	It's good. I tried it. It saves power.	Jingdong prefecture level city in Shandong
Fortune Shop	Green Intelligent Power Saver	Nothing**, it seems like there's more	Jingdong prefecture level city in Shandong
Fortune Shop	Green Intelligent Power Saver	Power saving device received, well packaged, good quality, fast logistics	Jingdong prefecture level city in Shandong
Fortune Shop	Green Intelligent Power Saver	The service is okay, is that when I bought five, the desirable pieces are three, and then wait a few days and send me two replacement, I do not know if it is a mistake of the shipper, if I was not in the courier there to see, go home to say no, the	Jingdong prefecture level city in Shandong
Fortune Shop	Green Intelligent Power Saver	This thing will only cost me more if I use it, but before I used it, it was less than a ~ electricity a month, and after I used it, it was more than two hundred kWh a month.	Jingdong prefecture level city in Shandong
Fortune Shop	Green Intelligent Power Saver	I just used it. I don't know how it is.	Jingdong prefecture level city in Shandong
Fortune Shop	Green Intelligent Power Saver	I've received the product and I'm using it, so I'll see how it goes in a month!	Jingdong prefecture level city in Shandong
Fortune Shop	Green Intelligent Power Saver	The goods have just received the effect do not know how, please look forward to the results.	Jingdong prefecture level city in Shandong
Fortune Shop	Green Intelligent Power Saver	Power saving device, the material is very good practical and durable, the size is suitable, packaging is very good, the price is cheap, logistics is very fast!	Jingdong prefecture level city in Shandong
Fortune Shop	Green Intelligent Power Saver	Very nice, will comment in detail after using it for a while.	Jingdong prefecture level city in Shandong
Fortune Shop	Green Intelligent Power Saver	Good stuff to trust and support	Jingdong prefecture level city in Shandong
Fortune Shop	Green Intelligent Power Saver	I'll comment after I use it, doubt if it really saves power no	Jingdong prefecture level city in Shandong
Fortune Shop	Green Intelligent Power Saver	It's good quality and works well!	Jingdong prefecture level city in Shandong
Fortune Shop	Green Intelligent Power Saver	hahaha just right hahahahahaha hiccup hahahaha	Jingdong prefecture level city in Shandong
Fortune Shop	Green Intelligent Power Saver	The real thing matches the picture and looks great!	Jingdong prefecture level city in Shandong
Fortune Shop	Green Intelligent Power Saver	Plugged it in and we'll see when we pay the bills at the end of the year!	Jingdong prefecture level city in Shandong
Fortune Shop	Green Intelligent Power Saver	False advertising. It doesn't work.	Jingdong prefecture level city in Shandong
Fortune Shop	Green Intelligent Power Saver	Goods received, good effect	Jingdong prefecture level city in Shandong
Fortune Shop	Green Intelligent Power Saver	It looks okay, we'll see if it saves electricity when the time comes	Jingdong prefecture level city in Shandong
Fortune Shop	Green Intelligent Power Saver	Logistics is very fast quality is very good, the price is very affordable, accessories are also complete, installed, satisfied with good use	Jingdong prefecture level city in Shandong
Fortune Shop	Green Intelligent Power Saver	I've received the power saver. I'm looking forward to the savings.	Jingdong prefecture level city in Shandong
Fortune Shop	Green Intelligent Power Saver	Received, have not yet used, after use and then evaluate	Jingdong prefecture level city in Shandong
Fortune Shop	Green Intelligent Power Saver	I received the item, but I don't know if it saves power or not.	Jingdong prefecture level city in Shandong
Fortune Shop	Green Intelligent Power Saver	On Trial	Jingdong prefecture level city in Shandong
Fortune Shop	Green Intelligent Power Saver	Use it and see how it goes.	Jingdong prefecture level city in Shandong
Fortune Shop	Green Intelligent Power Saver	Quality is very good, no odor, fine workmanship, grain color are very like, satisfied!	Jingdong prefecture level city in Shandong
Fortune Shop	Green Intelligent Power Saver	I'll give you a good review. I don't know if it'll work.	Jingdong prefecture level city in Shandong
Fortune Shop	Green Intelligent Power Saver	The logistics is very fast and the package is intact. Good use of affordable. The effect is very good, is genuine	Jingdong prefecture level city in Shandong
Fortune Shop	Green Intelligent Power Saver	Nothing happens when you plug it in.	Jingdong prefecture level city in Shandong
Fortune Shop	Green Intelligent Power Saver	I don't know if it's as good as it sounds.	Jingdong prefecture level city in Shandong
Fortune Shop	Green Intelligent Power Saver	The quality of the stuff is very good, exactly the same as the seller's description, very satisfied!	Jingdong prefecture level city in Shandong
Fortune Shop	Green Intelligent Power Saver	The saver works great, it really works, it's affordable and worth buying.	Jingdong prefecture level city in Shandong
Fortune Shop	Green Intelligent Power Saver	Just used it for a couple days. I don't know yet.	Jingdong prefecture level city in Shandong
Fortune Shop	Green Intelligent Power Saver	The quality is quite good, cheaper than the physical store, good, next time will come again!	Jingdong prefecture level city in Shandong
Fortune Shop	Green Intelligent Power Saver	Good quality, cheap and affordable worth recommending to buy!	Jingdong prefecture level city in Shandong

Fortune Shop	Green Intelligent Power Saver	Very good quality, well made, works well! Satisfied	Jingdong prefecture level city in Shandong
Fortune Shop	Green Intelligent Power Saver	The logistics is very fast quality is very good very pleasant a shopping	Jingdong prefecture level city in Shandong
Fortune Shop	Green Intelligent Power Saver	Use it and then come back to evaluate!	Jingdong prefecture level city in Shandong
Fortune Shop	Green Intelligent Power Saver	# It's no use lying at all #	Jingdong prefecture level city in Shandong
Fortune Shop	Green Intelligent Power Saver	I can't get a voltage drop.	Jingdong prefecture level city in Shandong
Fortune Shop	Green Intelligent Power Saver	Evaluate after use	Jingdong prefecture level city in Shandong
Fortune Shop	Green Intelligent Power Saver	Still on trial.	Jingdong prefecture level city in Shandong
Fortune Shop	Green Intelligent Power Saver	ineffective	Jingdong prefecture level city in Shandong
Fortune Shop	Green Intelligent Power Saver	Satisfaction Shopping	Jingdong prefecture level city in Shandong
Fortune Shop	Green Intelligent Power Saver	Lies.	Jingdong prefecture level city in Shandong
Fortune Shop	Green Intelligent Power Saver	It's a real power saver.	Jingdong prefecture level city in Shandong
Fortune Shop	Green Intelligent Power Saver	in use	Jingdong prefecture level city in Shandong
Fortune Shop	Green Intelligent Power Saver	I don't know yet.	Jingdong prefecture level city in Shandong
Fortune Shop	Green Intelligent Power Saver	not care about anything	Jingdong prefecture level city in Shandong
Fortune Shop	Green Intelligent Power Saver	It doesn't seem to have much effect	Jingdong prefecture level city in Shandong
Fortune Shop	Green Intelligent Power Saver	Let the merchant see for himself, does the plug positive and negative terminals look like this?	Jingdong prefecture level city in Shandong
Fortune Shop	Green Intelligent Power Saver	It doesn't work well, the electricity bill is less before using it, but more after using it, contacting customer service, the service is still not good, and returning it is slow	Jingdong prefecture level city in Shandong
Fortune Shop	Green Intelligent Power Saver	With the advertisement product does not match, above the effective negative, power saving rate of two rows of words it, the product looks very thin, with 3 days to see the meter number is still with the previous no difference, which is obviously fraudulent behavior!	Jingdong prefecture level city in Shandong
Fortune Shop	Green Intelligent Power Saver	Doesn't save any power at all. Not much use.	Jingdong prefecture level city in Shandong
Fortune Shop	Green Intelligent Power Saver	The plugs are all loose, they're all the same length and they don't work at all	Jingdong prefecture level city in Shandong
Fortune Shop	Green Intelligent Power Saver	It's bad 👎 it's not effective at all, you'd be crazy to sell this garbage on the internet.	Jingdong prefecture level city in Shandong
Fortune Shop	Green Intelligent Power Saver	It doesn't save any power at all, so I hope the old timers don't fall for this scam again	Jingdong prefecture level city in Shandong
Fortune Shop	Green Intelligent Power Saver	I've been using it for over a month now and haven't noticed any power savings.	Jingdong prefecture level city in Shandong
Fortune Shop	Green Intelligent Power Saver	It doesn't work at all. The electricity bill is higher when you use it.	Jingdong prefecture level city in Shandong
Fortune Shop	Green Intelligent Power Saver	It doesn't save any power, it's still as bad as ever.	Jingdong prefecture level city in Shandong
Fortune Shop	Green Intelligent Power Saver	Last night before leaving work to look at the table, and then plugged into the node device, this morning and then look at the table, than the original save a few degrees of electricity, very good to have a power-saving effect, easy to use, satisfied with the shopping praise.	Jingdong prefecture level city in Shandong
Fortune Shop	Green Intelligent Power Saver	The product is real and effective, the electricity bill is reduced, really good, thanks to the merchants have so many good products, the product is real and effective, the electricity bill is reduced, want to save electricity friends quickly buy it, early use early to save money.	Jingdong prefecture level city in Shandong
Fortune Shop	Green Intelligent Power Saver	Saver saves time and money, baby appearance is beautiful, the quality of goods is good, and the same as the business description, good service attitude, packaging is complete, logistics is very fast, praise to	Jingdong prefecture level city in Shandong
Fortune Shop	Green Intelligent Power Saver	The effect is good, summer, home electricity is relatively large, buy and use a few days, feel the effect is very good, originally ten dollars a day electricity bill, now eight dollars a day, quite good	Jingdong prefecture level city in Shandong
Fortune Shop	Green Intelligent Power Saver	Indeed can save electricity and money, the first time I know this or from a friend's home to see, but also do not believe it, and now they use it to know that there is this good thing.	Jingdong prefecture level city in Shandong
Fortune Shop	Green Intelligent Power Saver	I have received the Smart Power Saver. Perfectly packaged. Fast delivery. Boss customer service attitude is also very good. It's worth buying. I plugged it in and tried it and it really saves more power than the original. I'll buy it again next time.	Jingdong prefecture level city in Shandong
Fortune Shop	Green Intelligent Power Saver	It's nice to save a dozen bucks a night on two air conditioners that used to take 30 or 40 degrees a night, but now it's only 10 or so degrees a night	Jingdong prefecture level city in Shandong
Fortune Shop	Green Intelligent Power Saver	Product no manufacturer's name no product qualification certificate, try two days to see the effect of	Jingdong prefecture level city in Shandong
Fortune Shop	Green Intelligent Power Saver	I've been using it for over a month and have saved over $80, so I've repurchased a few more back to save together	Jingdong prefecture level city in Shandong
Fortune Shop	Green Intelligent Power Saver	I haven't used it yet and will comment when I do, but it's well made.	Jingdong prefecture level city in Shandong
Fortune Shop	Green Intelligent Power Saver	It's quite energy efficient. It's really a boon for the home.	Jingdong prefecture level city in Shandong
Fortune Shop	Green Intelligent Power Saver	Energy-saving socket received a month, after using it for a month proved that it is indeed energy-saving, electricity bill saving, will buy it again, thank you!	Jingdong prefecture level city in Shandong
Fortune Shop	Green Intelligent Power Saver	There was a similar product many years ago. Now technology has evolved, buy one to try, according to the instructions in the box, it takes one or two dozen days to show results. Check your electric bill and see what happens.	Jingdong prefecture level city in Shandong
Fortune Shop	Green Intelligent Power Saver	Jingdong logistics is really fast, the same day the order arrived on the same day, more convenient than the supermarket, and a wide range of choices, the	Jingdong prefecture level city in Shandong
Fortune Shop	Green Intelligent Power Saver	I've been using this for a month or so and I can't see any results, it really is a scam as they say.	Jingdong prefecture level city in Shandong
Fortune Shop	Green Intelligent Power Saver	Very good use, a month can save a few degrees of power loss of electricity, introduced to the relatives to buy back to try.	Jingdong prefecture level city in Shandong
Fortune Shop	Green Intelligent Power Saver	It's really powerful! With this air conditioner can be turned on at any time without the pressure of paying the electricity bill! It's good for the people!	Jingdong prefecture level city in Shandong
Fortune Shop	Green Intelligent Power Saver	This business is a big ** products are not qualified, with a month of electricity more than one hundred percent of thirty, the purchase agreed to return the goods but do not receive a refund after receiving the goods, looking for the official side does not help, the Jingdong is a big **, I have never shopped in	Jingdong prefecture level city in Shandong
Fortune Shop	Green Intelligent Power Saver	It's good. I tried it. It saves power.	Jingdong prefecture level city in Shandong
Fortune Shop	Green Intelligent Power Saver	Nothing**, it seems like there's more	Jingdong prefecture level city in Shandong
Fortune Shop	Green Intelligent Power Saver	Power saving device received, well packaged, good quality, fast logistics	Jingdong prefecture level city in Shandong
Fortune Shop	Green Intelligent Power Saver	The service is okay, is that when I bought five, the desirable pieces are three, and then wait a few days and send me two replacement, I do not know if it is a mistake of the shipper, if I was not in the courier there to see, go home to say no, the	Jingdong prefecture level city in Shandong
Fortune Shop	Green Intelligent Power Saver	This thing will only cost me more if I use it, but before I used it, it was less than a ~ electricity a month, and after I used it, it was more than two hundred kWh a month.	Jingdong prefecture level city in Shandong

Fortune Shop	Green Intelligent Power Saver	I just used it. I don't know how it is.	Jingdong prefecture level city in Shandong
Fortune Shop	Green Intelligent Power Saver	I've received the product and I'm using it, so I'll see how it goes in a month!	Jingdong prefecture level city in Shandong
Fortune Shop	Green Intelligent Power Saver	The goods have just received the effect do not know how, please look forward to the results.	Jingdong prefecture level city in Shandong
Fortune Shop	Green Intelligent Power Saver	Power saving device, the material is very good practical and durable, the size is suitable, packaging is very good, the price is cheap, logistics is very fast!	Jingdong prefecture level city in Shandong
Fortune Shop	Green Intelligent Power Saver	Very nice, will comment in detail after using it for a while.	Jingdong prefecture level city in Shandong
Fortune Shop	Green Intelligent Power Saver	Good stuff to trust and support	Jingdong prefecture level city in Shandong
Fortune Shop	Green Intelligent Power Saver	I'll comment after I use it, doubt if it really saves power no	Jingdong prefecture level city in Shandong
Fortune Shop	Green Intelligent Power Saver	It's good quality and works well!	Jingdong prefecture level city in Shandong
Fortune Shop	Green Intelligent Power Saver	hahaha just right hahahahahaha hiccup hahahaha	Jingdong prefecture level city in Shandong
Fortune Shop	Green Intelligent Power Saver	The real thing matches the picture and looks great!	Jingdong prefecture level city in Shandong
Fortune Shop	Green Intelligent Power Saver	Plugged it in and we'll see when we pay the bills at the end of the year!	Jingdong prefecture level city in Shandong
Fortune Shop	Green Intelligent Power Saver	False advertising. It doesn't work.	Jingdong prefecture level city in Shandong
Fortune Shop	Green Intelligent Power Saver	Goods received, good effect	Jingdong prefecture level city in Shandong
Fortune Shop	Green Intelligent Power Saver	It looks okay, we'll see if it saves electricity when the time comes	Jingdong prefecture level city in Shandong
Fortune Shop	Green Intelligent Power Saver	Logistics is very fast quality is very good, the price is very affordable, accessories are also complete, installed, satisfied with good use	Jingdong prefecture level city in Shandong
Fortune Shop	Green Intelligent Power Saver	I've received the power saver. I'm looking forward to the savings.	Jingdong prefecture level city in Shandong
Fortune Shop	Green Intelligent Power Saver	Received, have not yet used, after use and then evaluate	Jingdong prefecture level city in Shandong
Fortune Shop	Green Intelligent Power Saver	I received the item, but I don't know if it saves power or not.	Jingdong prefecture level city in Shandong
Fortune Shop	Green Intelligent Power Saver	On Trial	Jingdong prefecture level city in Shandong
Fortune Shop	Green Intelligent Power Saver	Use it and see how it goes.	Jingdong prefecture level city in Shandong
Fortune Shop	Green Intelligent Power Saver	Quality is very good, no odor, fine workmanship, grain color are very like, satisfied!	Jingdong prefecture level city in Shandong
Fortune Shop	Green Intelligent Power Saver	I'll give you a good review. I don't know if it'll work.	Jingdong prefecture level city in Shandong
Fortune Shop	Green Intelligent Power Saver	The logistics is very fast and the package is intact. Good use of affordable. The effect is very good, is genuine	Jingdong prefecture level city in Shandong
Fortune Shop	Green Intelligent Power Saver	Nothing happens when you plug it in.	Jingdong prefecture level city in Shandong
Fortune Shop	Green Intelligent Power Saver	I don't know if it's as good as it sounds.	Jingdong prefecture level city in Shandong
Fortune Shop	Green Intelligent Power Saver	The quality of the stuff is very good, exactly the same as the seller's description, very satisfied!	Jingdong prefecture level city in Shandong
Fortune Shop	Green Intelligent Power Saver	The saver works great, it really works, it's affordable and worth buying.	Jingdong prefecture level city in Shandong
Fortune Shop	Green Intelligent Power Saver	Just used it for a couple days. I don't know yet.	Jingdong prefecture level city in Shandong
Fortune Shop	Green Intelligent Power Saver	The quality is quite good, cheaper than the physical store, good, next time will come again!	Jingdong prefecture level city in Shandong
Fortune Shop	Green Intelligent Power Saver	Good quality, cheap and affordable worth recommending to buy!	Jingdong prefecture level city in Shandong
Fortune Shop	Green Intelligent Power Saver	Very good quality, well made, works well! Satisfied	Jingdong prefecture level city in Shandong
Fortune Shop	Green Intelligent Power Saver	The logistics is very fast quality is very good very pleasant a shopping	Jingdong prefecture level city in Shandong
Fortune Shop	Green Intelligent Power Saver	Use it and then come back to evaluate!	Jingdong prefecture level city in Shandong
Fortune Shop	Green Intelligent Power Saver	# It's no use lying at all #	Jingdong prefecture level city in Shandong
Fortune Shop	Green Intelligent Power Saver	I can't get a voltage drop.	Jingdong prefecture level city in Shandong
Fortune Shop	Green Intelligent Power Saver	Evaluate after use	Jingdong prefecture level city in Shandong
Fortune Shop	Green Intelligent Power Saver	Still on trial.	Jingdong prefecture level city in Shandong
Fortune Shop	Green Intelligent Power Saver	ineffective	Jingdong prefecture level city in Shandong
Fortune Shop	Green Intelligent Power Saver	Satisfaction Shopping	Jingdong prefecture level city in Shandong
Fortune Shop	Green Intelligent Power Saver	Lies.	Jingdong prefecture level city in Shandong
Fortune Shop	Green Intelligent Power Saver	It's a real power saver.	Jingdong prefecture level city in Shandong
Fortune Shop	Green Intelligent Power Saver	in use	Jingdong prefecture level city in Shandong
Fortune Shop	Green Intelligent Power Saver	I don't know yet.	Jingdong prefecture level city in Shandong
Fortune Shop	Green Intelligent Power Saver	not care about anything	Jingdong prefecture level city in Shandong
Fortune Shop	Green Intelligent Power Saver	It doesn't seem to have much effect	Jingdong prefecture level city in Shandong
Fortune Shop	Green Intelligent Power Saver	Let the merchant see for himself, does the plug positive and negative terminals look like this?	Jingdong prefecture level city in Shandong
Fortune Shop	Green Intelligent Power Saver	It doesn't work well, the electricity bill is less before using it, but more after using it, contacting customer service, the service is still not good, and returning it is slow	Jingdong prefecture level city in Shandong
Fortune Shop	Green Intelligent Power Saver	With the advertisement product does not match, above the effective negative, power saving rate of two rows of words it, the product looks very thin, with 3 days to see the meter number is still with the previous no difference, which is obviously fraudulent behavior!	Jingdong prefecture level city in Shandong
Fortune Shop	Green Intelligent Power Saver	Doesn't save any power at all. Not much use.	Jingdong prefecture level city in Shandong
Fortune Shop	Green Intelligent Power Saver	The plugs are all loose, they're all the same length and they don't work at all	Jingdong prefecture level city in Shandong
Fortune Shop	Green Intelligent Power Saver	It's bad 👎 it's not effective at all, you'd be crazy to sell this garbage on the internet.	Jingdong prefecture level city in Shandong

Fortune Shop	Green Intelligent Power Saver	It doesn't save any power at all, so I hope the old timers don't fall for this scam again	Jingdong prefecture level city in Shandong
Fortune Shop	Green Intelligent Power Saver	I've been using it for over a month now and haven't noticed any power savings.	Jingdong prefecture level city in Shandong
Fortune Shop	Green Intelligent Power Saver	It doesn't work at all. The electricity bill is higher when you use it.	Jingdong prefecture level city in Shandong
Fortune Shop	Green Intelligent Power Saver	It doesn't save any power, it's still as bad as ever.	Jingdong prefecture level city in Shandong
Fortune Shop	Green Intelligent Power Saver	Last night before leaving work to look at the table, and then plugged into the node device, this morning and then look at the table, than the original save a few degrees of electricity, very good to have a power-saving effect, easy to use, satisfied with the shopping praise.	Jingdong prefecture level city in Shandong
Fortune Shop	Green Intelligent Power Saver	The product is real and effective, the electricity bill is reduced, really good, thanks to the merchants have so many good products, the product is real and effective, the electricity bill is reduced, want to save electricity friends quickly buy it, early use early to save money.	Jingdong prefecture level city in Shandong
Fortune Shop	Green Intelligent Power Saver	Saver saves time and money, baby appearance is beautiful, the quality of goods is good, and the same as the business description, good service attitude, packaging is complete, logistics is very fast, praise to	Jingdong prefecture level city in Shandong
Fortune Shop	Green Intelligent Power Saver	The effect is good, summer, home electricity is relatively large, buy and use a few days, feel the effect is very good, originally ten dollars a day electricity bill, now eight dollars a day, quite good	Jingdong prefecture level city in Shandong
Fortune Shop	Green Intelligent Power Saver	Indeed can save electricity and money, the first time I know this or from a friend's home to see, but also do not believe it, and now they use it to know that there is this good thing.	Jingdong prefecture level city in Shandong
Fortune Shop	Green Intelligent Power Saver	I have received the Smart Power Saver. Perfectly packaged. Fast delivery. Boss customer service attitude is also very good. It's worth buying. I plugged it in and tried it and it really saves more power than the original. I'll buy it again next time.	Jingdong prefecture level city in Shandong
Fortune Shop	Green Intelligent Power Saver	It's nice to save a dozen bucks a night on two air conditioners that used to take 30 or 40 degrees a night, but now it's only 10 or so degrees a night	Jingdong prefecture level city in Shandong
Fortune Shop	Green Intelligent Power Saver	Product no manufacturer's name no product qualification certificate, try two days to see the effect of	Jingdong prefecture level city in Shandong
Fortune Shop	Green Intelligent Power Saver	I've been using it for over a month and have saved over $80, so I've repurchased a few more back to save together	Jingdong prefecture level city in Shandong
Fortune Shop	Green Intelligent Power Saver	I haven't used it yet and will comment when I do, but it's well made.	Jingdong prefecture level city in Shandong
Fortune Shop	Green Intelligent Power Saver	It's quite energy efficient. It's really a boon for the home.	Jingdong prefecture level city in Shandong
Fortune Shop	Green Intelligent Power Saver	Energy-saving socket received a month, after using it for a month proved that it is indeed energy-saving, electricity bill saving, will buy it again, thank you!	Jingdong prefecture level city in Shandong
Fortune Shop	Green Intelligent Power Saver	There was a similar product many years ago. Now technology has evolved, buy one to try, according to the instructions in the box, it takes one or two dozen days to show results. Check your electric bill and see what happens.	Jingdong prefecture level city in Shandong
Fortune Shop	Green Intelligent Power Saver	Jingdong logistics is really fast, the same day the order arrived on the same day, more convenient than the supermarket, and a wide range of choices, the	Jingdong prefecture level city in Shandong
Fortune Shop	Green Intelligent Power Saver	I've been using this for a month or so and I can't see any results, it really is a scam as they say.	Jingdong prefecture level city in Shandong
Fortune Shop	Green Intelligent Power Saver	Very good use, a month can save a few degrees of power loss of electricity, introduced to the relatives to buy back to try.	Jingdong prefecture level city in Shandong
Fortune Shop	Green Intelligent Power Saver	It's really powerful! With this air conditioner can be turned on at any time without the pressure of paying the electricity bill! It's good for the people!	Jingdong prefecture level city in Shandong
Fortune Shop	Green Intelligent Power Saver	This business is a big ** products are not qualified, with a month of electricity more than one hundred percent of thirty, the purchase agreed to return the goods but do not receive a refund after receiving the goods, looking for the official side does not help, the Jingdong is a big **, I have never shopped in	Jingdong prefecture level city in Shandong
Fortune Shop	Green Intelligent Power Saver	It's good. I tried it. It saves power.	Jingdong prefecture level city in Shandong
Fortune Shop	Green Intelligent Power Saver	Nothing**, it seems like there's more	Jingdong prefecture level city in Shandong
Fortune Shop	Green Intelligent Power Saver	Power saving device received, well packaged, good quality, fast logistics	Jingdong prefecture level city in Shandong
Fortune Shop	Green Intelligent Power Saver	The service is okay, is that when I bought five, the desirable pieces are three, and then wait a few days and send me two replacement, I do not know if it is a mistake of the shipper, if I was not in the courier there to see, go home to say no, the	Jingdong prefecture level city in Shandong
Fortune Shop	Green Intelligent Power Saver	This thing will only cost me more if I use it, but before I used it, it was less than a ~ electricity a month, and after I used it, it was more than two hundred kWh a month.	Jingdong prefecture level city in Shandong
Fortune Shop	Green Intelligent Power Saver	I just used it. I don't know how it is.	Jingdong prefecture level city in Shandong
Fortune Shop	Green Intelligent Power Saver	I've received the product and I'm using it, so I'll see how it goes in a month!	Jingdong prefecture level city in Shandong
Fortune Shop	Green Intelligent Power Saver	The goods have just received the effect do not know how, please look forward to the results.	Jingdong prefecture level city in Shandong
Fortune Shop	Green Intelligent Power Saver	Power saving device, the material is very good practical and durable, the size is suitable, packaging is very good, the price is cheap, logistics is very fast!	Jingdong prefecture level city in Shandong
Fortune Shop	Green Intelligent Power Saver	Very nice, will comment in detail after using it for a while.	Jingdong prefecture level city in Shandong
Fortune Shop	Green Intelligent Power Saver	Good stuff to trust and support	Jingdong prefecture level city in Shandong
Fortune Shop	Green Intelligent Power Saver	I'll comment after I use it, doubt if it really saves power no	Jingdong prefecture level city in Shandong
Fortune Shop	Green Intelligent Power Saver	It's good quality and works well!	Jingdong prefecture level city in Shandong
Fortune Shop	Green Intelligent Power Saver	hahaha just right hahahahahaha hiccup hahahaha	Jingdong prefecture level city in Shandong
Fortune Shop	Green Intelligent Power Saver	The real thing matches the picture and looks great!	Jingdong prefecture level city in Shandong
Fortune Shop	Green Intelligent Power Saver	Plugged it in and we'll see when we pay the bills at the end of the year!	Jingdong prefecture level city in Shandong
Fortune Shop	Green Intelligent Power Saver	False advertising. It doesn't work.	Jingdong prefecture level city in Shandong
Fortune Shop	Green Intelligent Power Saver	Goods received, good effect	Jingdong prefecture level city in Shandong
Fortune Shop	Green Intelligent Power Saver	It looks okay, we'll see if it saves electricity when the time comes	Jingdong prefecture level city in Shandong
Fortune Shop	Green Intelligent Power Saver	Logistics is very fast quality is very good, the price is very affordable, accessories are also complete, installed, satisfied with good use	Jingdong prefecture level city in Shandong
Fortune Shop	Green Intelligent Power Saver	I've received the power saver. I'm looking forward to the savings.	Jingdong prefecture level city in Shandong
Fortune Shop	Green Intelligent Power Saver	Received, have not yet used, after use and then evaluate	Jingdong prefecture level city in Shandong
Fortune Shop	Green Intelligent Power Saver	I received the item, but I don't know if it saves power or not.	Jingdong prefecture level city in Shandong
Fortune Shop	Green Intelligent Power Saver	On Trial	Jingdong prefecture level city in Shandong
Fortune Shop	Green Intelligent Power Saver	Use it and see how it goes.	Jingdong prefecture level city in Shandong
Fortune Shop	Green Intelligent Power Saver	Quality is very good, no odor, fine workmanship, grain color are very like, satisfied!	Jingdong prefecture level city in Shandong

Fortune Shop	Green Intelligent Power Saver	I'll give you a good review. I don't know if it'll work.	Jingdong prefecture level city in Shandong
Fortune Shop	Green Intelligent Power Saver	The logistics is very fast and the package is intact. Good use of affordable. The effect is very good, is genuine	Jingdong prefecture level city in Shandong
Fortune Shop	Green Intelligent Power Saver	Nothing happens when you plug it in.	Jingdong prefecture level city in Shandong
Fortune Shop	Green Intelligent Power Saver	I don't know if it's as good as it sounds.	Jingdong prefecture level city in Shandong
Fortune Shop	Green Intelligent Power Saver	The quality of the stuff is very good, exactly the same as the seller's description, very satisfied!	Jingdong prefecture level city in Shandong
Fortune Shop	Green Intelligent Power Saver	The saver works great, it really works, it's affordable and worth buying.	Jingdong prefecture level city in Shandong
Fortune Shop	Green Intelligent Power Saver	Just used it for a couple days. I don't know yet.	Jingdong prefecture level city in Shandong
Fortune Shop	Green Intelligent Power Saver	The quality is quite good, cheaper than the physical store, good, next time will come again!	Jingdong prefecture level city in Shandong
Fortune Shop	Green Intelligent Power Saver	Good quality, cheap and affordable worth recommending to buy!	Jingdong prefecture level city in Shandong
Fortune Shop	Green Intelligent Power Saver	Very good quality, well made, works well! Satisfied	Jingdong prefecture level city in Shandong
Fortune Shop	Green Intelligent Power Saver	The logistics is very fast quality is very good very pleasant a shopping	Jingdong prefecture level city in Shandong
Fortune Shop	Green Intelligent Power Saver	Use it and then come back to evaluate!	Jingdong prefecture level city in Shandong
Fortune Shop	Green Intelligent Power Saver	# It's no use lying at all #	Jingdong prefecture level city in Shandong
Fortune Shop	Green Intelligent Power Saver	I can't get a voltage drop.	Jingdong prefecture level city in Shandong
Fortune Shop	Green Intelligent Power Saver	Evaluate after use	Jingdong prefecture level city in Shandong
Fortune Shop	Green Intelligent Power Saver	Still on trial.	Jingdong prefecture level city in Shandong
Fortune Shop	Green Intelligent Power Saver	ineffective	Jingdong prefecture level city in Shandong
Fortune Shop	Green Intelligent Power Saver	Satisfaction Shopping	Jingdong prefecture level city in Shandong
Fortune Shop	Green Intelligent Power Saver	Lies.	Jingdong prefecture level city in Shandong
Fortune Shop	Green Intelligent Power Saver	It's a real power saver.	Jingdong prefecture level city in Shandong
Fortune Shop	Green Intelligent Power Saver	in use	Jingdong prefecture level city in Shandong
Fortune Shop	Green Intelligent Power Saver	I don't know yet.	Jingdong prefecture level city in Shandong
Fortune Shop	Green Intelligent Power Saver	not care about anything	Jingdong prefecture level city in Shandong
Fortune Shop	Green Intelligent Power Saver	It doesn't seem to have much effect	Jingdong prefecture level city in Shandong
Fortune Shop	Green Intelligent Power Saver	Let the merchant see for himself, does the plug positive and negative terminals look like this?	Jingdong prefecture level city in Shandong
Fortune Shop	Green Intelligent Power Saver	It doesn't work well, the electricity bill is less before using it, but more after using it, contacting customer service, the service is still not good, and returning it is slow	Jingdong prefecture level city in Shandong
Fortune Shop	Green Intelligent Power Saver	With the advertisement product does not match, above the effective negative, power saving rate of two rows of words it, the product looks very thin, with 3 days to see the meter number is still with the previous no difference, which is obviously fraudulent behavior!	Jingdong prefecture level city in Shandong
Fortune Shop	Green Intelligent Power Saver	Doesn't save any power at all. Not much use.	Jingdong prefecture level city in Shandong
Fortune Shop	Green Intelligent Power Saver	The plugs are all loose, they're all the same length and they don't work at all	Jingdong prefecture level city in Shandong
Fortune Shop	Green Intelligent Power Saver	It's bad 👎 it's not effective at all, you'd be crazy to sell this garbage on the internet.	Jingdong prefecture level city in Shandong
Fortune Shop	Green Intelligent Power Saver	It doesn't save any power at all, so I hope the old timers don't fall for this scam again	Jingdong prefecture level city in Shandong
Fortune Shop	Green Intelligent Power Saver	I've been using it for over a month now and haven't noticed any power savings.	Jingdong prefecture level city in Shandong
Fortune Shop	Green Intelligent Power Saver	It doesn't work at all. The electricity bill is higher when you use it.	Jingdong prefecture level city in Shandong
Fortune Shop	Green Intelligent Power Saver	It doesn't save any power, it's still as bad as ever.	Jingdong prefecture level city in Shandong
Fortune Shop	Green Intelligent Power Saver	Last night before leaving work to look at the table, and then plugged into the node device, this morning and then look at the table, than the original save a few degrees of electricity, very good to have a power-saving effect, easy to use, satisfied with the shopping praise.	Jingdong prefecture level city in Shandong
Fortune Shop	Green Intelligent Power Saver	The product is real and effective, the electricity bill is reduced, really good, thanks to the merchants have so many good products, the product is real and effective, the electricity bill is reduced, want to save electricity friends quickly buy it, early use early to save money.	Jingdong prefecture level city in Shandong
Fortune Shop	Green Intelligent Power Saver	Saver saves time and money, baby appearance is beautiful, the quality of goods is good, and the same as the business description, good service attitude, packaging is complete, logistics is very fast, praise to	Jingdong prefecture level city in Shandong
Fortune Shop	Green Intelligent Power Saver	The effect is good, summer, home electricity is relatively large, buy and use a few days, feel the effect is very good, originally ten dollars a day electricity bill, now eight dollars a day, quite good	Jingdong prefecture level city in Shandong
Fortune Shop	Green Intelligent Power Saver	Indeed can save electricity and money, the first time I know this or from a friend's home to see, but also do not believe it, and now they use it to know that there is this good thing.	Jingdong prefecture level city in Shandong
Fortune Shop	Green Intelligent Power Saver	I have received the Smart Power Saver. Perfectly packaged. Fast delivery. Boss customer service attitude is also very good. It's worth buying. I plugged it in and tried it and it really saves more power than the original. I'll buy it again next time.	Jingdong prefecture level city in Shandong
Fortune Shop	Green Intelligent Power Saver	It's nice to save a dozen bucks a night on two air conditioners that used to take 30 or 40 degrees a night, but now it's only 10 or so degrees a night	Jingdong prefecture level city in Shandong
Fortune Shop	Green Intelligent Power Saver	Product no manufacturer's name no product qualification certificate, try two days to see the effect of	Jingdong prefecture level city in Shandong
Fortune Shop	Green Intelligent Power Saver	I've been using it for over a month and have saved over $80, so I've repurchased a few more back to save together	Jingdong prefecture level city in Shandong
Fortune Shop	Green Intelligent Power Saver	I haven't used it yet and will comment when I do, but it's well made.	Jingdong prefecture level city in Shandong
Fortune Shop	Green Intelligent Power Saver	It's quite energy efficient. It's really a boon for the home.	Jingdong prefecture level city in Shandong
Fortune Shop	Green Intelligent Power Saver	Energy-saving socket received a month, after using it for a month proved that it is indeed energy-saving, electricity bill saving, will buy it again, thank you!	Jingdong prefecture level city in Shandong
Fortune Shop	Green Intelligent Power Saver	There was a similar product many years ago. Now technology has evolved, buy one to try, according to the instructions in the box, it takes one or two dozen days to show results. Check your electric bill and see what happens.	Jingdong prefecture level city in Shandong
Fortune Shop	Green Intelligent Power Saver	Jingdong logistics is really fast, the same day the order arrived on the same day, more convenient than the supermarket, and a wide range of choices, the	Jingdong prefecture level city in Shandong
Fortune Shop	Green Intelligent Power Saver	I've been using this for a month or so and I can't see any results, it really is a scam as they say.	Jingdong prefecture level city in Shandong

Fortune Shop	Green Intelligent Power Saver	Very good use, a month can save a few degrees of power loss of electricity, introduced to the relatives to buy back to try.	Jingdong prefecture level city in Shandong
Fortune Shop	Green Intelligent Power Saver	It's really powerful! With this air conditioner can be turned on at any time without the pressure of paying the electricity bill! It's good for the people!	Jingdong prefecture level city in Shandong
Fortune Shop	Green Intelligent Power Saver	This business is a big ** products are not qualified, with a month of electricity more than one hundred percent of thirty, the purchase agreed to return the goods but do not receive a refund after receiving the goods, looking for the official side does not help, the Jingdong is a big **, I have never shopped in	Jingdong prefecture level city in Shandong
Fortune Shop	Green Intelligent Power Saver	It's good. I tried it. It saves power.	Jingdong prefecture level city in Shandong
Fortune Shop	Green Intelligent Power Saver	Nothing**, it seems like there's more	Jingdong prefecture level city in Shandong
Fortune Shop	Green Intelligent Power Saver	Power saving device received, well packaged, good quality, fast logistics	Jingdong prefecture level city in Shandong
Fortune Shop	Green Intelligent Power Saver	The service is okay, is that when I bought five, the desirable pieces are three, and then wait a few days and send me two replacement, I do not know if it is a mistake of the shipper, if I was not in the courier there to see, go home to say no, the	Jingdong prefecture level city in Shandong
Fortune Shop	Green Intelligent Power Saver	This thing will only cost me more if I use it, but before I used it, it was less than a ~ electricity a month, and after I used it, it was more than two hundred kWh a month.	Jingdong prefecture level city in Shandong
Fortune Shop	Green Intelligent Power Saver	I just used it. I don't know how it is.	Jingdong prefecture level city in Shandong
Fortune Shop	Green Intelligent Power Saver	I've received the product and I'm using it, so I'll see how it goes in a month!	Jingdong prefecture level city in Shandong
Fortune Shop	Green Intelligent Power Saver	The goods have just received the effect do not know how, please look forward to the results.	Jingdong prefecture level city in Shandong
Fortune Shop	Green Intelligent Power Saver	Power saving device, the material is very good practical and durable, the size is suitable, packaging is very good, the price is cheap, logistics is very fast!	Jingdong prefecture level city in Shandong
Fortune Shop	Green Intelligent Power Saver	Very nice, will comment in detail after using it for a while.	Jingdong prefecture level city in Shandong
Fortune Shop	Green Intelligent Power Saver	Good stuff to trust and support	Jingdong prefecture level city in Shandong
Fortune Shop	Green Intelligent Power Saver	I'll comment after I use it, doubt if it really saves power no	Jingdong prefecture level city in Shandong
Fortune Shop	Green Intelligent Power Saver	It's good quality and works well!	Jingdong prefecture level city in Shandong
Fortune Shop	Green Intelligent Power Saver	hahaha just right hahahahahaha hiccup hahahaha	Jingdong prefecture level city in Shandong
Fortune Shop	Green Intelligent Power Saver	The real thing matches the picture and looks great!	Jingdong prefecture level city in Shandong
Fortune Shop	Green Intelligent Power Saver	Plugged it in and we'll see when we pay the bills at the end of the year!	Jingdong prefecture level city in Shandong
Fortune Shop	Green Intelligent Power Saver	False advertising. It doesn't work.	Jingdong prefecture level city in Shandong
Fortune Shop	Green Intelligent Power Saver	Goods received, good effect	Jingdong prefecture level city in Shandong
Fortune Shop	Green Intelligent Power Saver	It looks okay, we'll see if it saves electricity when the time comes	Jingdong prefecture level city in Shandong
Fortune Shop	Green Intelligent Power Saver	Logistics is very fast quality is very good, the price is very affordable, accessories are also complete, installed, satisfied with good use	Jingdong prefecture level city in Shandong
Fortune Shop	Green Intelligent Power Saver	I've received the power saver. I'm looking forward to the savings.	Jingdong prefecture level city in Shandong
Fortune Shop	Green Intelligent Power Saver	Received, have not yet used, after use and then evaluate	Jingdong prefecture level city in Shandong
Fortune Shop	Green Intelligent Power Saver	I received the item, but I don't know if it saves power or not.	Jingdong prefecture level city in Shandong
Fortune Shop	Green Intelligent Power Saver	On Trial	Jingdong prefecture level city in Shandong
Fortune Shop	Green Intelligent Power Saver	Use it and see how it goes.	Jingdong prefecture level city in Shandong
Fortune Shop	Green Intelligent Power Saver	Quality is very good, no odor, fine workmanship, grain color are very like, satisfied!	Jingdong prefecture level city in Shandong
Fortune Shop	Green Intelligent Power Saver	I'll give you a good review. I don't know if it'll work.	Jingdong prefecture level city in Shandong
Fortune Shop	Green Intelligent Power Saver	The logistics is very fast and the package is intact. Good use of affordable. The effect is very good, is genuine	Jingdong prefecture level city in Shandong
Fortune Shop	Green Intelligent Power Saver	Nothing happens when you plug it in.	Jingdong prefecture level city in Shandong
Fortune Shop	Green Intelligent Power Saver	I don't know if it's as good as it sounds.	Jingdong prefecture level city in Shandong
Fortune Shop	Green Intelligent Power Saver	The quality of the stuff is very good, exactly the same as the seller's description, very satisfied!	Jingdong prefecture level city in Shandong
Fortune Shop	Green Intelligent Power Saver	The saver works great, it really works, it's affordable and worth buying.	Jingdong prefecture level city in Shandong
Fortune Shop	Green Intelligent Power Saver	Just used it for a couple days. I don't know yet.	Jingdong prefecture level city in Shandong
Fortune Shop	Green Intelligent Power Saver	The quality is quite good, cheaper than the physical store, good, next time will come again!	Jingdong prefecture level city in Shandong
Fortune Shop	Green Intelligent Power Saver	Good quality, cheap and affordable worth recommending to buy!	Jingdong prefecture level city in Shandong
Fortune Shop	Green Intelligent Power Saver	Very good quality, well made, works well! Satisfied	Jingdong prefecture level city in Shandong
Fortune Shop	Green Intelligent Power Saver	The logistics is very fast quality is very good very pleasant a shopping	Jingdong prefecture level city in Shandong
Fortune Shop	Green Intelligent Power Saver	Use it and then come back to evaluate!	Jingdong prefecture level city in Shandong
Fortune Shop	Green Intelligent Power Saver	# It's no use lying at all #	Jingdong prefecture level city in Shandong
Fortune Shop	Green Intelligent Power Saver	I can't get a voltage drop.	Jingdong prefecture level city in Shandong
Fortune Shop	Green Intelligent Power Saver	Evaluate after use	Jingdong prefecture level city in Shandong
Fortune Shop	Green Intelligent Power Saver	Still on trial.	Jingdong prefecture level city in Shandong
Fortune Shop	Green Intelligent Power Saver	ineffective	Jingdong prefecture level city in Shandong
Fortune Shop	Green Intelligent Power Saver	Satisfaction Shopping	Jingdong prefecture level city in Shandong
Fortune Shop	Green Intelligent Power Saver	Lies.	Jingdong prefecture level city in Shandong
Fortune Shop	Green Intelligent Power Saver	It's a real power saver.	Jingdong prefecture level city in Shandong
Fortune Shop	Green Intelligent Power Saver	in use	Jingdong prefecture level city in Shandong

Fortune Shop	Green Intelligent Power Saver	I don't know yet.	Jingdong prefecture level city in Shandong
Fortune Shop	Green Intelligent Power Saver	not care about anything	Jingdong prefecture level city in Shandong
Fortune Shop	Green Intelligent Power Saver	It doesn't seem to have much effect	Jingdong prefecture level city in Shandong
Fortune Shop	Green Intelligent Power Saver	Let the merchant see for himself, does the plug positive and negative terminals look like this?	Jingdong prefecture level city in Shandong
Fortune Shop	Green Intelligent Power Saver	It doesn't work well, the electricity bill is less before using it, but more after using it, contacting customer service, the service is still not good, and returning it is slow	Jingdong prefecture level city in Shandong
Fortune Shop	Green Intelligent Power Saver	With the advertisement product does not match, above the effective negative, power saving rate of two rows of words it, the product looks very thin, with 3 days to see the meter number is still with the previous no difference, which is obviously fraudulent behavior!	Jingdong prefecture level city in Shandong
Fortune Shop	Green Intelligent Power Saver	Doesn't save any power at all. Not much use.	Jingdong prefecture level city in Shandong
Fortune Shop	Green Intelligent Power Saver	The plugs are all loose, they're all the same length and they don't work at all	Jingdong prefecture level city in Shandong
Fortune Shop	Green Intelligent Power Saver	It's bad 👎 it's not effective at all, you'd be crazy to sell this garbage on the internet.	Jingdong prefecture level city in Shandong
Fortune Shop	Green Intelligent Power Saver	It doesn't save any power at all, so I hope the old timers don't fall for this scam again	Jingdong prefecture level city in Shandong
Fortune Shop	Green Intelligent Power Saver	I've been using it for over a month now and haven't noticed any power savings.	Jingdong prefecture level city in Shandong
Fortune Shop	Green Intelligent Power Saver	It doesn't work at all. The electricity bill is higher when you use it.	Jingdong prefecture level city in Shandong
Fortune Shop	Green Intelligent Power Saver	It doesn't save any power, it's still as bad as ever.	Jingdong prefecture level city in Shandong
Fortune Shop	Green Intelligent Power Saver	Last night before leaving work to look at the table, and then plugged into the node device, this morning and then look at the table, than the original save a few degrees of electricity, very good to have a power-saving effect, easy to use, satisfied with the shopping praise.	Jingdong prefecture level city in Shandong
Fortune Shop	Green Intelligent Power Saver	The product is real and effective, the electricity bill is reduced, really good, thanks to the merchants have so many good products, the product is real and effective, the electricity bill is reduced, want to save electricity friends quickly buy it, early use early to save money.	Jingdong prefecture level city in Shandong
Fortune Shop	Green Intelligent Power Saver	Saver saves time and money, baby appearance is beautiful, the quality of goods is good, and the same as the business description, good service attitude, packaging is complete, logistics is very fast, praise to	Jingdong prefecture level city in Shandong
Fortune Shop	Green Intelligent Power Saver	The effect is good, summer, home electricity is relatively large, buy and use a few days, feel the effect is very good, originally ten dollars a day electricity bill, now eight dollars a day, quite good	Jingdong prefecture level city in Shandong
Fortune Shop	Green Intelligent Power Saver	Indeed can save electricity and money, the first time I know this or from a friend's home to see, but also do not believe it, and now they use it to know that there is this good thing.	Jingdong prefecture level city in Shandong
Fortune Shop	Green Intelligent Power Saver	I have received the Smart Power Saver. Perfectly packaged. Fast delivery. Boss customer service attitude is also very good. It's worth buying. I plugged it in and tried it and it really saves more power than the original. I'll buy it again next time.	Jingdong prefecture level city in Shandong
Fortune Shop	Green Intelligent Power Saver	It's nice to save a dozen bucks a night on two air conditioners that used to take 30 or 40 degrees a night, but now it's only 10 or so degrees a night	Jingdong prefecture level city in Shandong
Fortune Shop	Green Intelligent Power Saver	Product no manufacturer's name no product qualification certificate, try two days to see the effect of	Jingdong prefecture level city in Shandong
Fortune Shop	Green Intelligent Power Saver	I've been using it for over a month and have saved over $80, so I've repurchased a few more back to save together	Jingdong prefecture level city in Shandong
Fortune Shop	Green Intelligent Power Saver	I haven't used it yet and will comment when I do, but it's well made.	Jingdong prefecture level city in Shandong
Fortune Shop	Green Intelligent Power Saver	It's quite energy efficient. It's really a boon for the home.	Jingdong prefecture level city in Shandong
Fortune Shop	Green Intelligent Power Saver	Energy-saving socket received a month, after using it for a month proved that it is indeed energy-saving, electricity bill saving, will buy it again, thank you!	Jingdong prefecture level city in Shandong
Fortune Shop	Green Intelligent Power Saver	There was a similar product many years ago. Now technology has evolved, buy one to try, according to the instructions in the box, it takes one or two dozen days to show results. Check your electric bill and see what happens.	Jingdong prefecture level city in Shandong
Fortune Shop	Green Intelligent Power Saver	Jingdong logistics is really fast, the same day the order arrived on the same day, more convenient than the supermarket, and a wide range of choices, the	Jingdong prefecture level city in Shandong
Fortune Shop	Green Intelligent Power Saver	I've been using this for a month or so and I can't see any results, it really is a scam as they say.	Jingdong prefecture level city in Shandong
Fortune Shop	Green Intelligent Power Saver	Very good use, a month can save a few degrees of power loss of electricity, introduced to the relatives to buy back to try.	Jingdong prefecture level city in Shandong
Fortune Shop	Green Intelligent Power Saver	It's really powerful! With this air conditioner can be turned on at any time without the pressure of paying the electricity bill! It's good for the people!	Jingdong prefecture level city in Shandong
Fortune Shop	Green Intelligent Power Saver	This business is a big ** products are not qualified, with a month of electricity more than one hundred percent of thirty, the purchase agreed to return the goods but do not receive a refund after receiving the goods, looking for the official side does not help, the Jingdong is a big **, I have never shopped in	Jingdong prefecture level city in Shandong
Fortune Shop	Green Intelligent Power Saver	It's good. I tried it. It saves power.	Jingdong prefecture level city in Shandong
Fortune Shop	Green Intelligent Power Saver	Nothing**, it seems like there's more	Jingdong prefecture level city in Shandong
Fortune Shop	Green Intelligent Power Saver	Power saving device received, well packaged, good quality, fast logistics	Jingdong prefecture level city in Shandong
Fortune Shop	Green Intelligent Power Saver	The service is okay, is that when I bought five, the desirable pieces are three, and then wait a few days and send me two replacement, I do not know if it is a mistake of the shipper, if I was not in the courier there to see, go home to say no, the	Jingdong prefecture level city in Shandong
Fortune Shop	Green Intelligent Power Saver	This thing will only cost me more if I use it, but before I used it, it was less than a ~ electricity a month, and after I used it, it was more than two hundred kWh a month.	Jingdong prefecture level city in Shandong
Fortune Shop	Green Intelligent Power Saver	I just used it. I don't know how it is.	Jingdong prefecture level city in Shandong
Fortune Shop	Green Intelligent Power Saver	I've received the product and I'm using it, so I'll see how it goes in a month!	Jingdong prefecture level city in Shandong
Fortune Shop	Green Intelligent Power Saver	The goods have just received the effect do not know how, please look forward to the results.	Jingdong prefecture level city in Shandong
Fortune Shop	Green Intelligent Power Saver	Power saving device, the material is very good practical and durable, the size is suitable, packaging is very good, the price is cheap, logistics is very fast!	Jingdong prefecture level city in Shandong
Fortune Shop	Green Intelligent Power Saver	Very nice, will comment in detail after using it for a while.	Jingdong prefecture level city in Shandong
Fortune Shop	Green Intelligent Power Saver	Good stuff to trust and support	Jingdong prefecture level city in Shandong
Fortune Shop	Green Intelligent Power Saver	I'll comment after I use it, doubt if it really saves power no	Jingdong prefecture level city in Shandong
Fortune Shop	Green Intelligent Power Saver	It's good quality and works well!	Jingdong prefecture level city in Shandong
Fortune Shop	Green Intelligent Power Saver	hahaha just right hahahahahaha hiccup hahahaha	Jingdong prefecture level city in Shandong
Fortune Shop	Green Intelligent Power Saver	The real thing matches the picture and looks great!	Jingdong prefecture level city in Shandong
Fortune Shop	Green Intelligent Power Saver	Plugged it in and we'll see when we pay the bills at the end of the year!	Jingdong prefecture level city in Shandong
Fortune Shop	Green Intelligent Power Saver	False advertising. It doesn't work.	Jingdong prefecture level city in Shandong

Fortune Shop	Green Intelligent Power Saver	Goods received, good effect	Jingdong prefecture level city in Shandong
Fortune Shop	Green Intelligent Power Saver	It looks okay, we'll see if it saves electricity when the time comes	Jingdong prefecture level city in Shandong
Fortune Shop	Green Intelligent Power Saver	Logistics is very fast quality is very good, the price is very affordable, accessories are also complete, installed, satisfied with good use	Jingdong prefecture level city in Shandong
Fortune Shop	Green Intelligent Power Saver	I've received the power saver. I'm looking forward to the savings.	Jingdong prefecture level city in Shandong
Fortune Shop	Green Intelligent Power Saver	Received, have not yet used, after use and then evaluate	Jingdong prefecture level city in Shandong
Fortune Shop	Green Intelligent Power Saver	I received the item, but I don't know if it saves power or not.	Jingdong prefecture level city in Shandong
Fortune Shop	Green Intelligent Power Saver	On Trial	Jingdong prefecture level city in Shandong
Fortune Shop	Green Intelligent Power Saver	Use it and see how it goes.	Jingdong prefecture level city in Shandong
Fortune Shop	Green Intelligent Power Saver	Quality is very good, no odor, fine workmanship, grain color are very like, satisfied!	Jingdong prefecture level city in Shandong
Fortune Shop	Green Intelligent Power Saver	I'll give you a good review. I don't know if it'll work.	Jingdong prefecture level city in Shandong
Fortune Shop	Green Intelligent Power Saver	The logistics is very fast and the package is intact. Good use of affordable. The effect is very good, is genuine	Jingdong prefecture level city in Shandong
Fortune Shop	Green Intelligent Power Saver	Nothing happens when you plug it in.	Jingdong prefecture level city in Shandong
Fortune Shop	Green Intelligent Power Saver	I don't know if it's as good as it sounds.	Jingdong prefecture level city in Shandong
Fortune Shop	Green Intelligent Power Saver	The quality of the stuff is very good, exactly the same as the seller's description, very satisfied!	Jingdong prefecture level city in Shandong
Fortune Shop	Green Intelligent Power Saver	The saver works great, it really works, it's affordable and worth buying.	Jingdong prefecture level city in Shandong
Fortune Shop	Green Intelligent Power Saver	Just used it for a couple days. I don't know yet.	Jingdong prefecture level city in Shandong
Fortune Shop	Green Intelligent Power Saver	The quality is quite good, cheaper than the physical store, good, next time will come again!	Jingdong prefecture level city in Shandong
Fortune Shop	Green Intelligent Power Saver	Good quality, cheap and affordable worth recommending to buy!	Jingdong prefecture level city in Shandong
Fortune Shop	Green Intelligent Power Saver	Very good quality, well made, works well! Satisfied	Jingdong prefecture level city in Shandong
Fortune Shop	Green Intelligent Power Saver	The logistics is very fast quality is very good very pleasant a shopping	Jingdong prefecture level city in Shandong
Fortune Shop	Green Intelligent Power Saver	Use it and then come back to evaluate!	Jingdong prefecture level city in Shandong
Fortune Shop	Green Intelligent Power Saver	# It's no use lying at all #	Jingdong prefecture level city in Shandong
Fortune Shop	Green Intelligent Power Saver	I can't get a voltage drop.	Jingdong prefecture level city in Shandong
Fortune Shop	Green Intelligent Power Saver	Evaluate after use	Jingdong prefecture level city in Shandong
Fortune Shop	Green Intelligent Power Saver	Still on trial.	Jingdong prefecture level city in Shandong
Fortune Shop	Green Intelligent Power Saver	ineffective	Jingdong prefecture level city in Shandong
Fortune Shop	Green Intelligent Power Saver	Satisfaction Shopping	Jingdong prefecture level city in Shandong
Fortune Shop	Green Intelligent Power Saver	Lies.	Jingdong prefecture level city in Shandong
Fortune Shop	Green Intelligent Power Saver	It's a real power saver.	Jingdong prefecture level city in Shandong
Fortune Shop	Green Intelligent Power Saver	in use	Jingdong prefecture level city in Shandong
Fortune Shop	Green Intelligent Power Saver	I don't know yet.	Jingdong prefecture level city in Shandong
Fortune Shop	Green Intelligent Power Saver	not care about anything	Jingdong prefecture level city in Shandong
Fortune Shop	Green Intelligent Power Saver	It doesn't seem to have much effect	Jingdong prefecture level city in Shandong
Fortune Shop	Green Intelligent Power Saver	Let the merchant see for himself, does the plug positive and negative terminals look like this?	Jingdong prefecture level city in Shandong
Fortune Shop	Green Intelligent Power Saver	It doesn't work well, the electricity bill is less before using it, but more after using it, contacting customer service, the service is still not good, and returning it is slow	Jingdong prefecture level city in Shandong
Fortune Shop	Green Intelligent Power Saver	With the advertisement product does not match, above the effective negative, power saving rate of two rows of words it, the product looks very thin, with 3 days to see the meter number is still with the previous no difference, which is obviously fraudulent behavior!	Jingdong prefecture level city in Shandong
Fortune Shop	Green Intelligent Power Saver	Doesn't save any power at all. Not much use.	Jingdong prefecture level city in Shandong
Fortune Shop	Green Intelligent Power Saver	The plugs are all loose, they're all the same length and they don't work at all	Jingdong prefecture level city in Shandong
Fortune Shop	Green Intelligent Power Saver	It's bad 👎 it's not effective at all, you'd be crazy to sell this garbage on the internet.	Jingdong prefecture level city in Shandong
Fortune Shop	Green Intelligent Power Saver	It doesn't save any power at all, so I hope the old timers don't fall for this scam again	Jingdong prefecture level city in Shandong
Fortune Shop	Green Intelligent Power Saver	I've been using it for over a month now and haven't noticed any power savings.	Jingdong prefecture level city in Shandong
Fortune Shop	Green Intelligent Power Saver	It doesn't work at all. The electricity bill is higher when you use it.	Jingdong prefecture level city in Shandong
Fortune Shop	Green Intelligent Power Saver	It doesn't save any power, it's still as bad as ever.	Jingdong prefecture level city in Shandong
Fortune Shop	Green Intelligent Power Saver	Last night before leaving work to look at the table, and then plugged into the node device, this morning and then look at the table, than the original save a few degrees of electricity, very good to have a power-saving effect, easy to use, satisfied with the shopping praise.	Jingdong prefecture level city in Shandong
Fortune Shop	Green Intelligent Power Saver	The product is real and effective, the electricity bill is reduced, really good, thanks to the merchants have so many good products, the product is real and effective, the electricity bill is reduced, want to save electricity friends quickly buy it, early use early to save money.	Jingdong prefecture level city in Shandong
Fortune Shop	Green Intelligent Power Saver	Saver saves time and money, baby appearance is beautiful, the quality of goods is good, and the same as the business description, good service attitude, packaging is complete, logistics is very fast, praise to	Jingdong prefecture level city in Shandong
Fortune Shop	Green Intelligent Power Saver	The effect is good, summer, home electricity is relatively large, buy and use a few days, feel the effect is very good, originally ten dollars a day electricity bill, now eight dollars a day, quite good	Jingdong prefecture level city in Shandong
Fortune Shop	Green Intelligent Power Saver	Indeed can save electricity and money, the first time I know this or from a friend's home to see, but also do not believe it, and now they use it to know that there is this good thing.	Jingdong prefecture level city in Shandong
Fortune Shop	Green Intelligent Power Saver	I have received the Smart Power Saver. Perfectly packaged. Fast delivery. Boss customer service attitude is also very good. It's worth buying. I plugged it in and tried it and it really saves more power than the original. I'll buy it again next time.	Jingdong prefecture level city in Shandong

Fortune Shop	Green Intelligent Power Saver	It's nice to save a dozen bucks a night on two air conditioners that used to take 30 or 40 degrees a night, but now it's only 10 or so degrees a night	Jingdong prefecture level city in Shandong
Fortune Shop	Green Intelligent Power Saver	Product no manufacturer's name no product qualification certificate, try two days to see the effect of	Jingdong prefecture level city in Shandong
Fortune Shop	Green Intelligent Power Saver	I've been using it for over a month and have saved over $80, so I've repurchased a few more back to save together	Jingdong prefecture level city in Shandong
Fortune Shop	Green Intelligent Power Saver	I haven't used it yet and will comment when I do, but it's well made.	Jingdong prefecture level city in Shandong
Fortune Shop	Green Intelligent Power Saver	It's quite energy efficient. It's really a boon for the home.	Jingdong prefecture level city in Shandong
Fortune Shop	Green Intelligent Power Saver	Energy-saving socket received a month, after using it for a month proved that it is indeed energy-saving, electricity bill saving, will buy it again, thank you!	Jingdong prefecture level city in Shandong
Fortune Shop	Green Intelligent Power Saver	There was a similar product many years ago. Now technology has evolved, buy one to try, according to the instructions in the box, it takes one or two dozen days to show results. Check your electric bill and see what happens.	Jingdong prefecture level city in Shandong
Fortune Shop	Green Intelligent Power Saver	Jingdong logistics is really fast, the same day the order arrived on the same day, more convenient than the supermarket, and a wide range of choices, the	Jingdong prefecture level city in Shandong
Fortune Shop	Green Intelligent Power Saver	I've been using this for a month or so and I can't see any results, it really is a scam as they say.	Jingdong prefecture level city in Shandong
Fortune Shop	Green Intelligent Power Saver	Very good use, a month can save a few degrees of power loss of electricity, introduced to the relatives to buy back to try.	Jingdong prefecture level city in Shandong
Fortune Shop	Green Intelligent Power Saver	It's really powerful! With this air conditioner can be turned on at any time without the pressure of paying the electricity bill! It's good for the people!	Jingdong prefecture level city in Shandong
Fortune Shop	Green Intelligent Power Saver	This business is a big ** products are not qualified, with a month of electricity more than one hundred percent of thirty, the purchase agreed to return the goods but do not receive a refund after receiving the goods, looking for the official side does not help, the Jingdong is a big **, I have never shopped in	Jingdong prefecture level city in Shandong
Fortune Shop	Green Intelligent Power Saver	It's good. I tried it. It saves power.	Jingdong prefecture level city in Shandong
Fortune Shop	Green Intelligent Power Saver	Nothing**, it seems like there's more	Jingdong prefecture level city in Shandong
Fortune Shop	Green Intelligent Power Saver	Power saving device received, well packaged, good quality, fast logistics	Jingdong prefecture level city in Shandong
Fortune Shop	Green Intelligent Power Saver	The service is okay, is that when I bought five, the desirable pieces are three, and then wait a few days and send me two replacement, I do not know if it is a mistake of the shipper, if I was not in the courier there to see, go home to say no, the	Jingdong prefecture level city in Shandong
Fortune Shop	Green Intelligent Power Saver	This thing will only cost me more if I use it, but before I used it, it was less than a ~ electricity a month, and after I used it, it was more than two hundred kWh a month.	Jingdong prefecture level city in Shandong
Fortune Shop	Green Intelligent Power Saver	I just used it. I don't know how it is.	Jingdong prefecture level city in Shandong
Fortune Shop	Green Intelligent Power Saver	I've received the product and I'm using it, so I'll see how it goes in a month!	Jingdong prefecture level city in Shandong
Fortune Shop	Green Intelligent Power Saver	The goods have just received the effect do not know how, please look forward to the results.	Jingdong prefecture level city in Shandong
Fortune Shop	Green Intelligent Power Saver	Power saving device, the material is very good practical and durable, the size is suitable, packaging is very good, the price is cheap, logistics is very fast!	Jingdong prefecture level city in Shandong
Fortune Shop	Green Intelligent Power Saver	Very nice, will comment in detail after using it for a while.	Jingdong prefecture level city in Shandong
Fortune Shop	Green Intelligent Power Saver	Good stuff to trust and support	Jingdong prefecture level city in Shandong
Fortune Shop	Green Intelligent Power Saver	I'll comment after I use it, doubt if it really saves power no	Jingdong prefecture level city in Shandong
Fortune Shop	Green Intelligent Power Saver	It's good quality and works well!	Jingdong prefecture level city in Shandong
Fortune Shop	Green Intelligent Power Saver	hahaha just right hahahahahaha hiccup hahahaha	Jingdong prefecture level city in Shandong
Fortune Shop	Green Intelligent Power Saver	The real thing matches the picture and looks great!	Jingdong prefecture level city in Shandong
Fortune Shop	Green Intelligent Power Saver	Plugged it in and we'll see when we pay the bills at the end of the year!	Jingdong prefecture level city in Shandong
Fortune Shop	Green Intelligent Power Saver	False advertising. It doesn't work.	Jingdong prefecture level city in Shandong
Fortune Shop	Green Intelligent Power Saver	Goods received, good effect	Jingdong prefecture level city in Shandong
Fortune Shop	Green Intelligent Power Saver	It looks okay, we'll see if it saves electricity when the time comes	Jingdong prefecture level city in Shandong
Fortune Shop	Green Intelligent Power Saver	Logistics is very fast quality is very good, the price is very affordable, accessories are also complete, installed, satisfied with good use	Jingdong prefecture level city in Shandong
Fortune Shop	Green Intelligent Power Saver	I've received the power saver. I'm looking forward to the savings.	Jingdong prefecture level city in Shandong
Fortune Shop	Green Intelligent Power Saver	Received, have not yet used, after use and then evaluate	Jingdong prefecture level city in Shandong
Fortune Shop	Green Intelligent Power Saver	I received the item, but I don't know if it saves power or not.	Jingdong prefecture level city in Shandong
Fortune Shop	Green Intelligent Power Saver	On Trial	Jingdong prefecture level city in Shandong
Fortune Shop	Green Intelligent Power Saver	Use it and see how it goes.	Jingdong prefecture level city in Shandong
Fortune Shop	Green Intelligent Power Saver	Quality is very good, no odor, fine workmanship, grain color are very like, satisfied!	Jingdong prefecture level city in Shandong
Fortune Shop	Green Intelligent Power Saver	I'll give you a good review. I don't know if it'll work.	Jingdong prefecture level city in Shandong
Fortune Shop	Green Intelligent Power Saver	The logistics is very fast and the package is intact. Good use of affordable. The effect is very good, is genuine	Jingdong prefecture level city in Shandong
Fortune Shop	Green Intelligent Power Saver	Nothing happens when you plug it in.	Jingdong prefecture level city in Shandong
Fortune Shop	Green Intelligent Power Saver	I don't know if it's as good as it sounds.	Jingdong prefecture level city in Shandong
Fortune Shop	Green Intelligent Power Saver	The quality of the stuff is very good, exactly the same as the seller's description, very satisfied!	Jingdong prefecture level city in Shandong
Fortune Shop	Green Intelligent Power Saver	The saver works great, it really works, it's affordable and worth buying.	Jingdong prefecture level city in Shandong
Fortune Shop	Green Intelligent Power Saver	Just used it for a couple days. I don't know yet.	Jingdong prefecture level city in Shandong
Fortune Shop	Green Intelligent Power Saver	The quality is quite good, cheaper than the physical store, good, next time will come again!	Jingdong prefecture level city in Shandong
Fortune Shop	Green Intelligent Power Saver	Good quality, cheap and affordable worth recommending to buy!	Jingdong prefecture level city in Shandong
Fortune Shop	Green Intelligent Power Saver	Very good quality, well made, works well! Satisfied	Jingdong prefecture level city in Shandong
Fortune Shop	Green Intelligent Power Saver	The logistics is very fast quality is very good very pleasant a shopping	Jingdong prefecture level city in Shandong
Fortune Shop	Green Intelligent Power Saver	Use it and then come back to evaluate!	Jingdong prefecture level city in Shandong

Fortune Shop	Green Intelligent Power Saver	# It's no use lying at all #	Jingdong prefecture level city in Shandong
Fortune Shop	Green Intelligent Power Saver	I can't get a voltage drop.	Jingdong prefecture level city in Shandong
Fortune Shop	Green Intelligent Power Saver	Evaluate after use	Jingdong prefecture level city in Shandong
Fortune Shop	Green Intelligent Power Saver	Still on trial.	Jingdong prefecture level city in Shandong
Fortune Shop	Green Intelligent Power Saver	ineffective	Jingdong prefecture level city in Shandong
Fortune Shop	Green Intelligent Power Saver	Satisfaction Shopping	Jingdong prefecture level city in Shandong
Fortune Shop	Green Intelligent Power Saver	Lies.	Jingdong prefecture level city in Shandong
Fortune Shop	Green Intelligent Power Saver	It's a real power saver.	Jingdong prefecture level city in Shandong
Fortune Shop	Green Intelligent Power Saver	in use	Jingdong prefecture level city in Shandong
Fortune Shop	Green Intelligent Power Saver	I don't know yet.	Jingdong prefecture level city in Shandong
Fortune Shop	Green Intelligent Power Saver	not care about anything	Jingdong prefecture level city in Shandong
Fortune Shop	Green Intelligent Power Saver	It doesn't seem to have much effect	Jingdong prefecture level city in Shandong
Fortune Shop	Green Intelligent Power Saver	Let the merchant see for himself, does the plug positive and negative terminals look like this?	Jingdong prefecture level city in Shandong
Fortune Shop	Green Intelligent Power Saver	It doesn't work well, the electricity bill is less before using it, but more after using it, contacting customer service, the service is still not good, and returning it is slow	Jingdong prefecture level city in Shandong
Fortune Shop	Green Intelligent Power Saver	With the advertisement product does not match, above the effective negative, power saving rate of two rows of words it, the product looks very thin, with 3 days to see the meter number is still with the previous no difference, which is obviously fraudulent behavior!	Jingdong prefecture level city in Shandong
Fortune Shop	Green Intelligent Power Saver	Doesn't save any power at all. Not much use.	Jingdong prefecture level city in Shandong
Fortune Shop	Green Intelligent Power Saver	The plugs are all loose, they're all the same length and they don't work at all	Jingdong prefecture level city in Shandong
Fortune Shop	Green Intelligent Power Saver	It's bad 👎 it's not effective at all, you'd be crazy to sell this garbage on the internet.	Jingdong prefecture level city in Shandong
Fortune Shop	Green Intelligent Power Saver	It doesn't save any power at all, so I hope the old timers don't fall for this scam again	Jingdong prefecture level city in Shandong
Fortune Shop	Green Intelligent Power Saver	I've been using it for over a month now and haven't noticed any power savings.	Jingdong prefecture level city in Shandong
Fortune Shop	Green Intelligent Power Saver	It doesn't work at all. The electricity bill is higher when you use it.	Jingdong prefecture level city in Shandong
Fortune Shop	Green Intelligent Power Saver	It doesn't save any power, it's still as bad as ever.	Jingdong prefecture level city in Shandong
Fortune Shop	Green Intelligent Power Saver	Last night before leaving work to look at the table, and then plugged into the node device, this morning and then look at the table, than the original save a few degrees of electricity, very good to have a power-saving effect, easy to use, satisfied with the shopping praise.	Jingdong prefecture level city in Shandong
Fortune Shop	Green Intelligent Power Saver	The product is real and effective, the electricity bill is reduced, really good, thanks to the merchants have so many good products, the product is real and effective, the electricity bill is reduced, want to save electricity friends quickly buy it, early use early to save money.	Jingdong prefecture level city in Shandong
Fortune Shop	Green Intelligent Power Saver	Saver saves time and money, baby appearance is beautiful, the quality of goods is good, and the same as the business description, good service attitude, packaging is complete, logistics is very fast, praise to	Jingdong prefecture level city in Shandong
Fortune Shop	Green Intelligent Power Saver	The effect is good, summer, home electricity is relatively large, buy and use a few days, feel the effect is very good, originally ten dollars a day electricity bill, now eight dollars a day, quite good	Jingdong prefecture level city in Shandong
Fortune Shop	Green Intelligent Power Saver	Indeed can save electricity and money, the first time I know this or from a friend's home to see, but also do not believe it, and now they use it to know that there is this good thing.	Jingdong prefecture level city in Shandong
Fortune Shop	Green Intelligent Power Saver	I have received the Smart Power Saver. Perfectly packaged. Fast delivery. Boss customer service attitude is also very good. It's worth buying. I plugged it in and tried it and it really saves more power than the original. I'll buy it again next time.	Jingdong prefecture level city in Shandong
Fortune Shop	Green Intelligent Power Saver	It's nice to save a dozen bucks a night on two air conditioners that used to take 30 or 40 degrees a night, but now it's only 10 or so degrees a night	Jingdong prefecture level city in Shandong
Fortune Shop	Green Intelligent Power Saver	Product no manufacturer's name no product qualification certificate, try two days to see the effect of	Jingdong prefecture level city in Shandong
Fortune Shop	Green Intelligent Power Saver	I've been using it for over a month and have saved over $80, so I've repurchased a few more back to save together	Jingdong prefecture level city in Shandong
Fortune Shop	Green Intelligent Power Saver	I haven't used it yet and will comment when I do, but it's well made.	Jingdong prefecture level city in Shandong
Fortune Shop	Green Intelligent Power Saver	It's quite energy efficient. It's really a boon for the home.	Jingdong prefecture level city in Shandong
Fortune Shop	Green Intelligent Power Saver	Energy-saving socket received a month, after using it for a month proved that it is indeed energy-saving, electricity bill saving, will buy it again, thank you!	Jingdong prefecture level city in Shandong
Fortune Shop	Green Intelligent Power Saver	There was a similar product many years ago. Now technology has evolved, buy one to try, according to the instructions in the box, it takes one or two dozen days to show results. Check your electric bill and see what happens.	Jingdong prefecture level city in Shandong
Fortune Shop	Green Intelligent Power Saver	Jingdong logistics is really fast, the same day the order arrived on the same day, more convenient than the supermarket, and a wide range of choices, the	Jingdong prefecture level city in Shandong
Fortune Shop	Green Intelligent Power Saver	I've been using this for a month or so and I can't see any results, it really is a scam as they say.	Jingdong prefecture level city in Shandong
Fortune Shop	Green Intelligent Power Saver	Very good use, a month can save a few degrees of power loss of electricity, introduced to the relatives to buy back to try.	Jingdong prefecture level city in Shandong
Fortune Shop	Green Intelligent Power Saver	It's really powerful! With this air conditioner can be turned on at any time without the pressure of paying the electricity bill! It's good for the people!	Jingdong prefecture level city in Shandong
Fortune Shop	Green Intelligent Power Saver	This business is a big ** products are not qualified, with a month of electricity more than one hundred percent of thirty, the purchase agreed to return the goods but do not receive a refund after receiving the goods, looking for the official side does not help, the Jingdong is a big **, I have never shopped in	Jingdong prefecture level city in Shandong
Fortune Shop	Green Intelligent Power Saver	It's good. I tried it. It saves power.	Jingdong prefecture level city in Shandong
Fortune Shop	Green Intelligent Power Saver	Nothing**, it seems like there's more	Jingdong prefecture level city in Shandong
Fortune Shop	Green Intelligent Power Saver	Power saving device received, well packaged, good quality, fast logistics	Jingdong prefecture level city in Shandong
Fortune Shop	Green Intelligent Power Saver	The service is okay, is that when I bought five, the desirable pieces are three, and then wait a few days and send me two replacement, I do not know if it is a mistake of the shipper, if I was not in the courier there to see, go home to say no, the	Jingdong prefecture level city in Shandong
Fortune Shop	Green Intelligent Power Saver	This thing will only cost me more if I use it, but before I used it, it was less than a ~ electricity a month, and after I used it, it was more than two hundred kWh a month.	Jingdong prefecture level city in Shandong
Fortune Shop	Green Intelligent Power Saver	I just used it. I don't know how it is.	Jingdong prefecture level city in Shandong
Fortune Shop	Green Intelligent Power Saver	I've received the product and I'm using it, so I'll see how it goes in a month!	Jingdong prefecture level city in Shandong
Fortune Shop	Green Intelligent Power Saver	The goods have just received the effect do not know how, please look forward to the results.	Jingdong prefecture level city in Shandong

Fortune Shop	Green Intelligent Power Saver	Power saving device, the material is very good practical and durable, the size is suitable, packaging is very good, the price is cheap, logistics is very fast!	Jingdong prefecture level city in Shandong
Fortune Shop	Green Intelligent Power Saver	Very nice, will comment in detail after using it for a while.	Jingdong prefecture level city in Shandong
Fortune Shop	Green Intelligent Power Saver	Good stuff to trust and support	Jingdong prefecture level city in Shandong
Fortune Shop	Green Intelligent Power Saver	I'll comment after I use it, doubt if it really saves power no	Jingdong prefecture level city in Shandong
Fortune Shop	Green Intelligent Power Saver	It's good quality and works well!	Jingdong prefecture level city in Shandong
Fortune Shop	Green Intelligent Power Saver	hahaha just right hahahahahaha hiccup hahahaha	Jingdong prefecture level city in Shandong
Fortune Shop	Green Intelligent Power Saver	The real thing matches the picture and looks great!	Jingdong prefecture level city in Shandong
Fortune Shop	Green Intelligent Power Saver	Plugged it in and we'll see when we pay the bills at the end of the year!	Jingdong prefecture level city in Shandong
Fortune Shop	Green Intelligent Power Saver	False advertising. It doesn't work.	Jingdong prefecture level city in Shandong
Fortune Shop	Green Intelligent Power Saver	Goods received, good effect	Jingdong prefecture level city in Shandong
Fortune Shop	Green Intelligent Power Saver	It looks okay, we'll see if it saves electricity when the time comes	Jingdong prefecture level city in Shandong
Fortune Shop	Green Intelligent Power Saver	Logistics is very fast quality is very good, the price is very affordable, accessories are also complete, installed, satisfied with good use	Jingdong prefecture level city in Shandong
Fortune Shop	Green Intelligent Power Saver	I've received the power saver. I'm looking forward to the savings.	Jingdong prefecture level city in Shandong
Fortune Shop	Green Intelligent Power Saver	Received, have not yet used, after use and then evaluate	Jingdong prefecture level city in Shandong
Fortune Shop	Green Intelligent Power Saver	I received the item, but I don't know if it saves power or not.	Jingdong prefecture level city in Shandong
Fortune Shop	Green Intelligent Power Saver	On Trial	Jingdong prefecture level city in Shandong
Fortune Shop	Green Intelligent Power Saver	Use it and see how it goes.	Jingdong prefecture level city in Shandong
Fortune Shop	Green Intelligent Power Saver	Quality is very good, no odor, fine workmanship, grain color are very like, satisfied!	Jingdong prefecture level city in Shandong
Fortune Shop	Green Intelligent Power Saver	I'll give you a good review. I don't know if it'll work.	Jingdong prefecture level city in Shandong
Fortune Shop	Green Intelligent Power Saver	The logistics is very fast and the package is intact. Good use of affordable. The effect is very good, is genuine	Jingdong prefecture level city in Shandong
Fortune Shop	Green Intelligent Power Saver	Nothing happens when you plug it in.	Jingdong prefecture level city in Shandong
Fortune Shop	Green Intelligent Power Saver	I don't know if it's as good as it sounds.	Jingdong prefecture level city in Shandong
Fortune Shop	Green Intelligent Power Saver	The quality of the stuff is very good, exactly the same as the seller's description, very satisfied!	Jingdong prefecture level city in Shandong
Fortune Shop	Green Intelligent Power Saver	The saver works great, it really works, it's affordable and worth buying.	Jingdong prefecture level city in Shandong
Fortune Shop	Green Intelligent Power Saver	Just used it for a couple days. I don't know yet.	Jingdong prefecture level city in Shandong
Fortune Shop	Green Intelligent Power Saver	The quality is quite good, cheaper than the physical store, good, next time will come again!	Jingdong prefecture level city in Shandong
Fortune Shop	Green Intelligent Power Saver	Good quality, cheap and affordable worth recommending to buy!	Jingdong prefecture level city in Shandong
Fortune Shop	Green Intelligent Power Saver	Very good quality, well made, works well! Satisfied	Jingdong prefecture level city in Shandong
Fortune Shop	Green Intelligent Power Saver	The logistics is very fast quality is very good very pleasant a shopping	Jingdong prefecture level city in Shandong
Fortune Shop	Green Intelligent Power Saver	Use it and then come back to evaluate!	Jingdong prefecture level city in Shandong
Fortune Shop	Green Intelligent Power Saver	# It's no use lying at all #	Jingdong prefecture level city in Shandong
Fortune Shop	Green Intelligent Power Saver	I can't get a voltage drop.	Jingdong prefecture level city in Shandong
Fortune Shop	Green Intelligent Power Saver	Evaluate after use	Jingdong prefecture level city in Shandong
Fortune Shop	Green Intelligent Power Saver	Still on trial.	Jingdong prefecture level city in Shandong
Fortune Shop	Green Intelligent Power Saver	ineffective	Jingdong prefecture level city in Shandong
Fortune Shop	Green Intelligent Power Saver	Satisfaction Shopping	Jingdong prefecture level city in Shandong
Fortune Shop	Green Intelligent Power Saver	Lies.	Jingdong prefecture level city in Shandong
Fortune Shop	Green Intelligent Power Saver	It's a real power saver.	Jingdong prefecture level city in Shandong
Fortune Shop	Green Intelligent Power Saver	in use	Jingdong prefecture level city in Shandong
Fortune Shop	Green Intelligent Power Saver	I don't know yet.	Jingdong prefecture level city in Shandong
Fortune Shop	Green Intelligent Power Saver	not care about anything	Jingdong prefecture level city in Shandong
Fortune Shop	Green Intelligent Power Saver	It doesn't seem to have much effect	Jingdong prefecture level city in Shandong
Fortune Shop	Green Intelligent Power Saver	Let the merchant see for himself, does the plug positive and negative terminals look like this?	Jingdong prefecture level city in Shandong
Fortune Shop	Green Intelligent Power Saver	It doesn't work well, the electricity bill is less before using it, but more after using it, contacting customer service, the service is still not good, and returning it is slow	Jingdong prefecture level city in Shandong
Fortune Shop	Green Intelligent Power Saver	With the advertisement product does not match, above the effective negative, power saving rate of two rows of words it, the product looks very thin, with 3 days to see the meter number is still with the previous no difference, which is obviously fraudulent behavior!	Jingdong prefecture level city in Shandong
Fortune Shop	Green Intelligent Power Saver	Doesn't save any power at all. Not much use.	Jingdong prefecture level city in Shandong
Fortune Shop	Green Intelligent Power Saver	The plugs are all loose, they're all the same length and they don't work at all	Jingdong prefecture level city in Shandong
Fortune Shop	Green Intelligent Power Saver	It's bad 👎 it's not effective at all, you'd be crazy to sell this garbage on the internet.	Jingdong prefecture level city in Shandong
Fortune Shop	Green Intelligent Power Saver	It doesn't save any power at all, so I hope the old timers don't fall for this scam again	Jingdong prefecture level city in Shandong
Fortune Shop	Green Intelligent Power Saver	I've been using it for over a month now and haven't noticed any power savings.	Jingdong prefecture level city in Shandong
Fortune Shop	Green Intelligent Power Saver	It doesn't work at all. The electricity bill is higher when you use it.	Jingdong prefecture level city in Shandong

Fortune Shop	Green Intelligent Power Saver	It doesn't save any power, it's still as bad as ever.	Jingdong prefecture level city in Shandong
Fortune Shop	Green Intelligent Power Saver	Last night before leaving work to look at the table, and then plugged into the node device, this morning and then look at the table, than the original save a few degrees of electricity, very good to have a power-saving effect, easy to use, satisfied with the shopping praise.	Jingdong prefecture level city in Shandong
Fortune Shop	Green Intelligent Power Saver	The product is real and effective, the electricity bill is reduced, really good, thanks to the merchants have so many good products, the product is real and effective, the electricity bill is reduced, want to save electricity friends quickly buy it, early use early to save money.	Jingdong prefecture level city in Shandong
Fortune Shop	Green Intelligent Power Saver	Saver saves time and money, baby appearance is beautiful, the quality of goods is good, and the same as the business description, good service attitude, packaging is complete, logistics is very fast, praise to	Jingdong prefecture level city in Shandong
Fortune Shop	Green Intelligent Power Saver	The effect is good, summer, home electricity is relatively large, buy and use a few days, feel the effect is very good, originally ten dollars a day electricity bill, now eight dollars a day, quite good	Jingdong prefecture level city in Shandong
Fortune Shop	Green Intelligent Power Saver	Indeed can save electricity and money, the first time I know this or from a friend's home to see, but also do not believe it, and now they use it to know that there is this good thing.	Jingdong prefecture level city in Shandong
Fortune Shop	Green Intelligent Power Saver	I have received the Smart Power Saver. Perfectly packaged. Fast delivery. Boss customer service attitude is also very good. It's worth buying. I plugged it in and tried it and it really saves more power than the original. I'll buy it again next time.	Jingdong prefecture level city in Shandong
Fortune Shop	Green Intelligent Power Saver	It's nice to save a dozen bucks a night on two air conditioners that used to take 30 or 40 degrees a night, but now it's only 10 or so degrees a night	Jingdong prefecture level city in Shandong
Fortune Shop	Green Intelligent Power Saver	Product no manufacturer's name no product qualification certificate, try two days to see the effect of	Jingdong prefecture level city in Shandong
Fortune Shop	Green Intelligent Power Saver	I've been using it for over a month and have saved over $80, so I've repurchased a few more back to save together	Jingdong prefecture level city in Shandong
Fortune Shop	Green Intelligent Power Saver	I haven't used it yet and will comment when I do, but it's well made.	Jingdong prefecture level city in Shandong
Fortune Shop	Green Intelligent Power Saver	It's quite energy efficient. It's really a boon for the home.	Jingdong prefecture level city in Shandong
Fortune Shop	Green Intelligent Power Saver	Energy-saving socket received a month, after using it for a month proved that it is indeed energy-saving, electricity bill saving, will buy it again, thank you!	Jingdong prefecture level city in Shandong
Fortune Shop	Green Intelligent Power Saver	There was a similar product many years ago. Now technology has evolved, buy one to try, according to the instructions in the box, it takes one or two dozen days to show results. Check your electric bill and see what happens.	Jingdong prefecture level city in Shandong
Fortune Shop	Green Intelligent Power Saver	Jingdong logistics is really fast, the same day the order arrived on the same day, more convenient than the supermarket, and a wide range of choices, the	Jingdong prefecture level city in Shandong
Fortune Shop	Green Intelligent Power Saver	I've been using this for a month or so and I can't see any results, it really is a scam as they say.	Jingdong prefecture level city in Shandong
Fortune Shop	Green Intelligent Power Saver	Very good use, a month can save a few degrees of power loss of electricity, introduced to the relatives to buy back to try.	Jingdong prefecture level city in Shandong
Fortune Shop	Green Intelligent Power Saver	It's really powerful! With this air conditioner can be turned on at any time without the pressure of paying the electricity bill! It's good for the people!	Jingdong prefecture level city in Shandong
Fortune Shop	Green Intelligent Power Saver	This business is a big ** products are not qualified, with a month of electricity more than one hundred percent of thirty, the purchase agreed to return the goods but do not receive a refund after receiving the goods, looking for the official side does not help, the Jingdong is a big **, I have never shopped in	Jingdong prefecture level city in Shandong
Fortune Shop	Green Intelligent Power Saver	It's good. I tried it. It saves power.	Jingdong prefecture level city in Shandong
Fortune Shop	Green Intelligent Power Saver	Nothing**, it seems like there's more	Jingdong prefecture level city in Shandong
Fortune Shop	Green Intelligent Power Saver	Power saving device received, well packaged, good quality, fast logistics	Jingdong prefecture level city in Shandong
Fortune Shop	Green Intelligent Power Saver	The service is okay, is that when I bought five, the desirable pieces are three, and then wait a few days and send me two replacement, I do not know if it is a mistake of the shipper, if I was not in the courier there to see, go home to say no, the	Jingdong prefecture level city in Shandong
Fortune Shop	Green Intelligent Power Saver	This thing will only cost me more if I use it, but before I used it, it was less than a ~ electricity a month, and after I used it, it was more than two hundred kWh a month.	Jingdong prefecture level city in Shandong
Fortune Shop	Green Intelligent Power Saver	I just used it. I don't know how it is.	Jingdong prefecture level city in Shandong
Fortune Shop	Green Intelligent Power Saver	I've received the product and I'm using it, so I'll see how it goes in a month!	Jingdong prefecture level city in Shandong
Fortune Shop	Green Intelligent Power Saver	The goods have just received the effect do not know how, please look forward to the results.	Jingdong prefecture level city in Shandong
Fortune Shop	Green Intelligent Power Saver	Power saving device, the material is very good practical and durable, the size is suitable, packaging is very good, the price is cheap, logistics is very fast!	Jingdong prefecture level city in Shandong
Fortune Shop	Green Intelligent Power Saver	Very nice, will comment in detail after using it for a while.	Jingdong prefecture level city in Shandong
Fortune Shop	Green Intelligent Power Saver	Good stuff to trust and support	Jingdong prefecture level city in Shandong
Fortune Shop	Green Intelligent Power Saver	I'll comment after I use it, doubt if it really saves power no	Jingdong prefecture level city in Shandong
Fortune Shop	Green Intelligent Power Saver	It's good quality and works well!	Jingdong prefecture level city in Shandong
Fortune Shop	Green Intelligent Power Saver	hahaha just right hahahahahaha hiccup hahahaha	Jingdong prefecture level city in Shandong
Fortune Shop	Green Intelligent Power Saver	The real thing matches the picture and looks great!	Jingdong prefecture level city in Shandong
Fortune Shop	Green Intelligent Power Saver	Plugged it in and we'll see when we pay the bills at the end of the year!	Jingdong prefecture level city in Shandong
Fortune Shop	Green Intelligent Power Saver	False advertising. It doesn't work.	Jingdong prefecture level city in Shandong
Fortune Shop	Green Intelligent Power Saver	Goods received, good effect	Jingdong prefecture level city in Shandong
Fortune Shop	Green Intelligent Power Saver	It looks okay, we'll see if it saves electricity when the time comes	Jingdong prefecture level city in Shandong
Fortune Shop	Green Intelligent Power Saver	Logistics is very fast quality is very good, the price is very affordable, accessories are also complete, installed, satisfied with good use	Jingdong prefecture level city in Shandong
Fortune Shop	Green Intelligent Power Saver	I've received the power saver. I'm looking forward to the savings.	Jingdong prefecture level city in Shandong
Fortune Shop	Green Intelligent Power Saver	Received, have not yet used, after use and then evaluate	Jingdong prefecture level city in Shandong
Fortune Shop	Green Intelligent Power Saver	I received the item, but I don't know if it saves power or not.	Jingdong prefecture level city in Shandong
Fortune Shop	Green Intelligent Power Saver	On Trial	Jingdong prefecture level city in Shandong
Fortune Shop	Green Intelligent Power Saver	Use it and see how it goes.	Jingdong prefecture level city in Shandong
Fortune Shop	Green Intelligent Power Saver	Quality is very good, no odor, fine workmanship, grain color are very like, satisfied!	Jingdong prefecture level city in Shandong
Fortune Shop	Green Intelligent Power Saver	I'll give you a good review. I don't know if it'll work.	Jingdong prefecture level city in Shandong
Fortune Shop	Green Intelligent Power Saver	The logistics is very fast and the package is intact. Good use of affordable. The effect is very good, is genuine	Jingdong prefecture level city in Shandong
Fortune Shop	Green Intelligent Power Saver	Nothing happens when you plug it in.	Jingdong prefecture level city in Shandong

Fortune Shop	Green Intelligent Power Saver	I don't know if it's as good as it sounds.	Jingdong prefecture level city in Shandong
Fortune Shop	Green Intelligent Power Saver	The quality of the stuff is very good, exactly the same as the seller's description, very satisfied!	Jingdong prefecture level city in Shandong
Fortune Shop	Green Intelligent Power Saver	The saver works great, it really works, it's affordable and worth buying.	Jingdong prefecture level city in Shandong
Fortune Shop	Green Intelligent Power Saver	Just used it for a couple days. I don't know yet.	Jingdong prefecture level city in Shandong
Fortune Shop	Green Intelligent Power Saver	The quality is quite good, cheaper than the physical store, good, next time will come again!	Jingdong prefecture level city in Shandong
Fortune Shop	Green Intelligent Power Saver	Good quality, cheap and affordable worth recommending to buy!	Jingdong prefecture level city in Shandong
Fortune Shop	Green Intelligent Power Saver	Very good quality, well made, works well! Satisfied	Jingdong prefecture level city in Shandong
Fortune Shop	Green Intelligent Power Saver	The logistics is very fast quality is very good very pleasant a shopping	Jingdong prefecture level city in Shandong
Fortune Shop	Green Intelligent Power Saver	Use it and then come back to evaluate!	Jingdong prefecture level city in Shandong
Fortune Shop	Green Intelligent Power Saver	# It's no use lying at all #	Jingdong prefecture level city in Shandong
Fortune Shop	Green Intelligent Power Saver	I can't get a voltage drop.	Jingdong prefecture level city in Shandong
Fortune Shop	Green Intelligent Power Saver	Evaluate after use	Jingdong prefecture level city in Shandong
Fortune Shop	Green Intelligent Power Saver	Still on trial.	Jingdong prefecture level city in Shandong
Fortune Shop	Green Intelligent Power Saver	ineffective	Jingdong prefecture level city in Shandong
Fortune Shop	Green Intelligent Power Saver	Satisfaction Shopping	Jingdong prefecture level city in Shandong
Fortune Shop	Green Intelligent Power Saver	Lies.	Jingdong prefecture level city in Shandong
Fortune Shop	Green Intelligent Power Saver	It's a real power saver.	Jingdong prefecture level city in Shandong
Fortune Shop	Green Intelligent Power Saver	in use	Jingdong prefecture level city in Shandong
Fortune Shop	Green Intelligent Power Saver	I don't know yet.	Jingdong prefecture level city in Shandong
Fortune Shop	Green Intelligent Power Saver	not care about anything	Jingdong prefecture level city in Shandong
Fortune Shop	Green Intelligent Power Saver	It doesn't seem to have much effect	Jingdong prefecture level city in Shandong
Fortune Shop	Green Intelligent Power Saver	Let the merchant see for himself, does the plug positive and negative terminals look like this?	Jingdong prefecture level city in Shandong
Fortune Shop	Green Intelligent Power Saver	It doesn't work well, the electricity bill is less before using it, but more after using it, contacting customer service, the service is still not good, and returning it is slow	Jingdong prefecture level city in Shandong
Fortune Shop	Green Intelligent Power Saver	With the advertisement product does not match, above the effective negative, power saving rate of two rows of words it, the product looks very thin, with 3 days to see the meter number is still with the previous no difference, which is obviously fraudulent behavior!	Jingdong prefecture level city in Shandong
Fortune Shop	Green Intelligent Power Saver	Doesn't save any power at all. Not much use.	Jingdong prefecture level city in Shandong
Fortune Shop	Green Intelligent Power Saver	The plugs are all loose, they're all the same length and they don't work at all	Jingdong prefecture level city in Shandong
Fortune Shop	Green Intelligent Power Saver	It's bad 👎 it's not effective at all, you'd be crazy to sell this garbage on the internet.	Jingdong prefecture level city in Shandong
Fortune Shop	Green Intelligent Power Saver	It doesn't save any power at all, so I hope the old timers don't fall for this scam again	Jingdong prefecture level city in Shandong
Fortune Shop	Green Intelligent Power Saver	I've been using it for over a month now and haven't noticed any power savings.	Jingdong prefecture level city in Shandong
Fortune Shop	Green Intelligent Power Saver	It doesn't work at all. The electricity bill is higher when you use it.	Jingdong prefecture level city in Shandong
Fortune Shop	Green Intelligent Power Saver	It doesn't save any power, it's still as bad as ever.	Jingdong prefecture level city in Shandong
Fortune Shop	Green Intelligent Power Saver	Last night before leaving work to look at the table, and then plugged into the node device, this morning and then look at the table, than the original save a few degrees of electricity, very good to have a power-saving effect, easy to use, satisfied with the shopping praise.	Jingdong prefecture level city in Shandong
Fortune Shop	Green Intelligent Power Saver	The product is real and effective, the electricity bill is reduced, really good, thanks to the merchants have so many good products, the product is real and effective, the electricity bill is reduced, want to save electricity friends quickly buy it, early use early to save money.	Jingdong prefecture level city in Shandong
Fortune Shop	Green Intelligent Power Saver	Saver saves time and money, baby appearance is beautiful, the quality of goods is good, and the same as the business description, good service attitude, packaging is complete, logistics is very fast, praise to	Jingdong prefecture level city in Shandong
Fortune Shop	Green Intelligent Power Saver	The effect is good, summer, home electricity is relatively large, buy and use a few days, feel the effect is very good, originally ten dollars a day electricity bill, now eight dollars a day, quite good	Jingdong prefecture level city in Shandong
Fortune Shop	Green Intelligent Power Saver	Indeed can save electricity and money, the first time I know this or from a friend's home to see, but also do not believe it, and now they use it to know that there is this good thing.	Jingdong prefecture level city in Shandong
Fortune Shop	Green Intelligent Power Saver	I have received the Smart Power Saver. Perfectly packaged. Fast delivery. Boss customer service attitude is also very good. It's worth buying. I plugged it in and tried it and it really saves more power than the original. I'll buy it again next time.	Jingdong prefecture level city in Shandong
Fortune Shop	Green Intelligent Power Saver	It's nice to save a dozen bucks a night on two air conditioners that used to take 30 or 40 degrees a night, but now it's only 10 or so degrees a night	Jingdong prefecture level city in Shandong
Fortune Shop	Green Intelligent Power Saver	Product no manufacturer's name no product qualification certificate, try two days to see the effect of	Jingdong prefecture level city in Shandong
Fortune Shop	Green Intelligent Power Saver	I've been using it for over a month and have saved over $80, so I've repurchased a few more back to save together	Jingdong prefecture level city in Shandong
Fortune Shop	Green Intelligent Power Saver	I haven't used it yet and will comment when I do, but it's well made.	Jingdong prefecture level city in Shandong
Fortune Shop	Green Intelligent Power Saver	It's quite energy efficient. It's really a boon for the home.	Jingdong prefecture level city in Shandong
Fortune Shop	Green Intelligent Power Saver	Energy-saving socket received a month, after using it for a month proved that it is indeed energy-saving, electricity bill saving, will buy it again, thank you!	Jingdong prefecture level city in Shandong
Fortune Shop	Green Intelligent Power Saver	There was a similar product many years ago. Now technology has evolved, buy one to try, according to the instructions in the box, it takes one or two dozen days to show results. Check your electric bill and see what happens.	Jingdong prefecture level city in Shandong
Fortune Shop	Green Intelligent Power Saver	Jingdong logistics is really fast, the same day the order arrived on the same day, more convenient than the supermarket, and a wide range of choices, the	Jingdong prefecture level city in Shandong
Fortune Shop	Green Intelligent Power Saver	I've been using this for a month or so and I can't see any results, it really is a scam as they say.	Jingdong prefecture level city in Shandong
Fortune Shop	Green Intelligent Power Saver	Very good use, a month can save a few degrees of power loss of electricity, introduced to the relatives to buy back to try.	Jingdong prefecture level city in Shandong
Fortune Shop	Green Intelligent Power Saver	It's really powerful! With this air conditioner can be turned on at any time without the pressure of paying the electricity bill! It's good for the people!	Jingdong prefecture level city in Shandong

Fortune Shop	Green Intelligent Power Saver	This business is a big ** products are not qualified, with a month of electricity more than one hundred percent of thirty, the purchase agreed to return the goods but do not receive a refund after receiving the goods, looking for the official side does not help, the Jingdong is a big **, I have never shopped in	Jingdong prefecture level city in Shandong
Fortune Shop	Green Intelligent Power Saver	It's good. I tried it. It saves power.	Jingdong prefecture level city in Shandong
Fortune Shop	Green Intelligent Power Saver	Nothing**, it seems like there's more	Jingdong prefecture level city in Shandong
Fortune Shop	Green Intelligent Power Saver	Power saving device received, well packaged, good quality, fast logistics	Jingdong prefecture level city in Shandong
Fortune Shop	Green Intelligent Power Saver	The service is okay, is that when I bought five, the desirable pieces are three, and then wait a few days and send me two replacement, I do not know if it is a mistake of the shipper, if I was not in the courier there to see, go home to say no, the	Jingdong prefecture level city in Shandong
Fortune Shop	Green Intelligent Power Saver	This thing will only cost me more if I use it, but before I used it, it was less than a ~ electricity a month, and after I used it, it was more than two hundred kWh a month.	Jingdong prefecture level city in Shandong
Fortune Shop	Green Intelligent Power Saver	I just used it. I don't know how it is.	Jingdong prefecture level city in Shandong
Fortune Shop	Green Intelligent Power Saver	I've received the product and I'm using it, so I'll see how it goes in a month!	Jingdong prefecture level city in Shandong
Fortune Shop	Green Intelligent Power Saver	The goods have just received the effect do not know how, please look forward to the results.	Jingdong prefecture level city in Shandong
Fortune Shop	Green Intelligent Power Saver	Power saving device, the material is very good practical and durable, the size is suitable, packaging is very good, the price is cheap, logistics is very fast!	Jingdong prefecture level city in Shandong
Fortune Shop	Green Intelligent Power Saver	Very nice, will comment in detail after using it for a while.	Jingdong prefecture level city in Shandong
Fortune Shop	Green Intelligent Power Saver	Good stuff to trust and support	Jingdong prefecture level city in Shandong
Fortune Shop	Green Intelligent Power Saver	I'll comment after I use it, doubt if it really saves power no	Jingdong prefecture level city in Shandong
Fortune Shop	Green Intelligent Power Saver	It's good quality and works well!	Jingdong prefecture level city in Shandong
Fortune Shop	Green Intelligent Power Saver	hahaha just right hahahahahaha hiccup hahahaha	Jingdong prefecture level city in Shandong
Fortune Shop	Green Intelligent Power Saver	The real thing matches the picture and looks great!	Jingdong prefecture level city in Shandong
Fortune Shop	Green Intelligent Power Saver	Plugged it in and we'll see when we pay the bills at the end of the year!	Jingdong prefecture level city in Shandong
Fortune Shop	Green Intelligent Power Saver	False advertising. It doesn't work.	Jingdong prefecture level city in Shandong
Fortune Shop	Green Intelligent Power Saver	Goods received, good effect	Jingdong prefecture level city in Shandong
Fortune Shop	Green Intelligent Power Saver	It looks okay, we'll see if it saves electricity when the time comes	Jingdong prefecture level city in Shandong
Fortune Shop	Green Intelligent Power Saver	Logistics is very fast quality is very good, the price is very affordable, accessories are also complete, installed, satisfied with good use	Jingdong prefecture level city in Shandong
Fortune Shop	Green Intelligent Power Saver	I've received the power saver. I'm looking forward to the savings.	Jingdong prefecture level city in Shandong
Fortune Shop	Green Intelligent Power Saver	Received, have not yet used, after use and then evaluate	Jingdong prefecture level city in Shandong
Fortune Shop	Green Intelligent Power Saver	I received the item, but I don't know if it saves power or not.	Jingdong prefecture level city in Shandong
Fortune Shop	Green Intelligent Power Saver	On Trial	Jingdong prefecture level city in Shandong
Fortune Shop	Green Intelligent Power Saver	Use it and see how it goes.	Jingdong prefecture level city in Shandong
Fortune Shop	Green Intelligent Power Saver	Quality is very good, no odor, fine workmanship, grain color are very like, satisfied!	Jingdong prefecture level city in Shandong
Fortune Shop	Green Intelligent Power Saver	I'll give you a good review. I don't know if it'll work.	Jingdong prefecture level city in Shandong
Fortune Shop	Green Intelligent Power Saver	The logistics is very fast and the package is intact. Good use of affordable. The effect is very good, is genuine	Jingdong prefecture level city in Shandong
Fortune Shop	Green Intelligent Power Saver	Nothing happens when you plug it in.	Jingdong prefecture level city in Shandong
Fortune Shop	Green Intelligent Power Saver	I don't know if it's as good as it sounds.	Jingdong prefecture level city in Shandong
Fortune Shop	Green Intelligent Power Saver	The quality of the stuff is very good, exactly the same as the seller's description, very satisfied!	Jingdong prefecture level city in Shandong
Fortune Shop	Green Intelligent Power Saver	The saver works great, it really works, it's affordable and worth buying.	Jingdong prefecture level city in Shandong
Fortune Shop	Green Intelligent Power Saver	Just used it for a couple days. I don't know yet.	Jingdong prefecture level city in Shandong
Fortune Shop	Green Intelligent Power Saver	The quality is quite good, cheaper than the physical store, good, next time will come again!	Jingdong prefecture level city in Shandong
Fortune Shop	Green Intelligent Power Saver	Good quality, cheap and affordable worth recommending to buy!	Jingdong prefecture level city in Shandong
Fortune Shop	Green Intelligent Power Saver	Very good quality, well made, works well! Satisfied	Jingdong prefecture level city in Shandong
Fortune Shop	Green Intelligent Power Saver	The logistics is very fast quality is very good very pleasant a shopping	Jingdong prefecture level city in Shandong
Fortune Shop	Green Intelligent Power Saver	Use it and then come back to evaluate!	Jingdong prefecture level city in Shandong
Fortune Shop	Green Intelligent Power Saver	# It's no use lying at all #	Jingdong prefecture level city in Shandong
Fortune Shop	Green Intelligent Power Saver	I can't get a voltage drop.	Jingdong prefecture level city in Shandong
Fortune Shop	Green Intelligent Power Saver	Evaluate after use	Jingdong prefecture level city in Shandong
Fortune Shop	Green Intelligent Power Saver	Still on trial.	Jingdong prefecture level city in Shandong
Fortune Shop	Green Intelligent Power Saver	ineffective	Jingdong prefecture level city in Shandong
Fortune Shop	Green Intelligent Power Saver	Satisfaction Shopping	Jingdong prefecture level city in Shandong
Fortune Shop	Green Intelligent Power Saver	Lies.	Jingdong prefecture level city in Shandong
Fortune Shop	Green Intelligent Power Saver	It's a real power saver.	Jingdong prefecture level city in Shandong
Fortune Shop	Green Intelligent Power Saver	in use	Jingdong prefecture level city in Shandong
Fortune Shop	Green Intelligent Power Saver	I don't know yet.	Jingdong prefecture level city in Shandong
Fortune Shop	Green Intelligent Power Saver	not care about anything	Jingdong prefecture level city in Shandong

Fortune Shop	Green Intelligent Power Saver	It doesn't seem to have much effect	Jingdong prefecture level city in Shandong
Fortune Shop	Green Intelligent Power Saver	Let the merchant see for himself, does the plug positive and negative terminals look like this?	Jingdong prefecture level city in Shandong
Fortune Shop	Green Intelligent Power Saver	It doesn't work well, the electricity bill is less before using it, but more after using it, contacting customer service, the service is still not good, and returning it is slow	Jingdong prefecture level city in Shandong
Fortune Shop	Green Intelligent Power Saver	With the advertisement product does not match, above the effective negative, power saving rate of two rows of words it, the product looks very thin, with 3 days to see the meter number is still with the previous no difference, which is obviously fraudulent behavior!	Jingdong prefecture level city in Shandong
Fortune Shop	Green Intelligent Power Saver	Doesn't save any power at all. Not much use.	Jingdong prefecture level city in Shandong
Fortune Shop	Green Intelligent Power Saver	The plugs are all loose, they're all the same length and they don't work at all	Jingdong prefecture level city in Shandong
Fortune Shop	Green Intelligent Power Saver	It's bad 👎 it's not effective at all, you'd be crazy to sell this garbage on the internet.	Jingdong prefecture level city in Shandong
Fortune Shop	Green Intelligent Power Saver	It doesn't save any power at all, so I hope the old timers don't fall for this scam again	Jingdong prefecture level city in Shandong
Fortune Shop	Green Intelligent Power Saver	I've been using it for over a month now and haven't noticed any power savings.	Jingdong prefecture level city in Shandong
Fortune Shop	Green Intelligent Power Saver	It doesn't work at all. The electricity bill is higher when you use it.	Jingdong prefecture level city in Shandong
Fortune Shop	Green Intelligent Power Saver	It doesn't save any power, it's still as bad as ever.	Jingdong prefecture level city in Shandong
Fortune Shop	Green Intelligent Power Saver	Last night before leaving work to look at the table, and then plugged into the node device, this morning and then look at the table, than the original save a few degrees of electricity, very good to have a power-saving effect, easy to use, satisfied with the shopping praise.	Jingdong prefecture level city in Shandong
Fortune Shop	Green Intelligent Power Saver	The product is real and effective, the electricity bill is reduced, really good, thanks to the merchants have so many good products, the product is real and effective, the electricity bill is reduced, want to save electricity friends quickly buy it, early use early to save money.	Jingdong prefecture level city in Shandong
Fortune Shop	Green Intelligent Power Saver	Saver saves time and money, baby appearance is beautiful, the quality of goods is good, and the same as the business description, good service attitude, packaging is complete, logistics is very fast, praise to	Jingdong prefecture level city in Shandong
Fortune Shop	Green Intelligent Power Saver	The effect is good, summer, home electricity is relatively large, buy and use a few days, feel the effect is very good, originally ten dollars a day electricity bill, now eight dollars a day, quite good	Jingdong prefecture level city in Shandong
Fortune Shop	Green Intelligent Power Saver	Indeed can save electricity and money, the first time I know this or from a friend's home to see, but also do not believe it, and now they use it to know that there is this good thing.	Jingdong prefecture level city in Shandong
Fortune Shop	Green Intelligent Power Saver	I have received the Smart Power Saver. Perfectly packaged. Fast delivery. Boss customer service attitude is also very good. It's worth buying. I plugged it in and tried it and it really saves more power than the original. I'll buy it again next time.	Jingdong prefecture level city in Shandong
Fortune Shop	Green Intelligent Power Saver	It's nice to save a dozen bucks a night on two air conditioners that used to take 30 or 40 degrees a night, but now it's only 10 or so degrees a night	Jingdong prefecture level city in Shandong
Fortune Shop	Green Intelligent Power Saver	Product no manufacturer's name no product qualification certificate, try two days to see the effect of	Jingdong prefecture level city in Shandong
Fortune Shop	Green Intelligent Power Saver	I've been using it for over a month and have saved over $80, so I've repurchased a few more back to save together	Jingdong prefecture level city in Shandong
Fortune Shop	Green Intelligent Power Saver	I haven't used it yet and will comment when I do, but it's well made.	Jingdong prefecture level city in Shandong
Fortune Shop	Green Intelligent Power Saver	It's quite energy efficient. It's really a boon for the home.	Jingdong prefecture level city in Shandong
Fortune Shop	Green Intelligent Power Saver	Energy-saving socket received a month, after using it for a month proved that it is indeed energy-saving, electricity bill saving, will buy it again, thank you!	Jingdong prefecture level city in Shandong
Fortune Shop	Green Intelligent Power Saver	There was a similar product many years ago. Now technology has evolved, buy one to try, according to the instructions in the box, it takes one or two dozen days to show results. Check your electric bill and see what happens.	Jingdong prefecture level city in Shandong
Fortune Shop	Green Intelligent Power Saver	Jingdong logistics is really fast, the same day the order arrived on the same day, more convenient than the supermarket, and a wide range of choices, the	Jingdong prefecture level city in Shandong
Fortune Shop	Green Intelligent Power Saver	I've been using this for a month or so and I can't see any results, it really is a scam as they say.	Jingdong prefecture level city in Shandong
Fortune Shop	Green Intelligent Power Saver	Very good use, a month can save a few degrees of power loss of electricity, introduced to the relatives to buy back to try.	Jingdong prefecture level city in Shandong
Fortune Shop	Green Intelligent Power Saver	It's really powerful! With this air conditioner can be turned on at any time without the pressure of paying the electricity bill! It's good for the people!	Jingdong prefecture level city in Shandong
Fortune Shop	Green Intelligent Power Saver	This business is a big ** products are not qualified, with a month of electricity more than one hundred percent of thirty, the purchase agreed to return the goods but do not receive a refund after receiving the goods, looking for the official side does not help, the Jingdong is a big **, I have never shopped in	Jingdong prefecture level city in Shandong
Fortune Shop	Green Intelligent Power Saver	It's good. I tried it. It saves power.	Jingdong prefecture level city in Shandong
Fortune Shop	Green Intelligent Power Saver	Nothing**, it seems like there's more	Jingdong prefecture level city in Shandong
Fortune Shop	Green Intelligent Power Saver	Power saving device received, well packaged, good quality, fast logistics	Jingdong prefecture level city in Shandong
Fortune Shop	Green Intelligent Power Saver	The service is okay, is that when I bought five, the desirable pieces are three, and then wait a few days and send me two replacement, I do not know if it is a mistake of the shipper, if I was not in the courier there to see, go home to say no, the	Jingdong prefecture level city in Shandong
Fortune Shop	Green Intelligent Power Saver	This thing will only cost me more if I use it, but before I used it, it was less than a ~ electricity a month, and after I used it, it was more than two hundred kWh a month.	Jingdong prefecture level city in Shandong
Fortune Shop	Green Intelligent Power Saver	I just used it. I don't know how it is.	Jingdong prefecture level city in Shandong
Fortune Shop	Green Intelligent Power Saver	I've received the product and I'm using it, so I'll see how it goes in a month!	Jingdong prefecture level city in Shandong
Fortune Shop	Green Intelligent Power Saver	The goods have just received the effect do not know how, please look forward to the results.	Jingdong prefecture level city in Shandong
Fortune Shop	Green Intelligent Power Saver	Power saving device, the material is very good practical and durable, the size is suitable, packaging is very good, the price is cheap, logistics is very fast!	Jingdong prefecture level city in Shandong
Fortune Shop	Green Intelligent Power Saver	Very nice, will comment in detail after using it for a while.	Jingdong prefecture level city in Shandong
Fortune Shop	Green Intelligent Power Saver	Good stuff to trust and support	Jingdong prefecture level city in Shandong
Fortune Shop	Green Intelligent Power Saver	I'll comment after I use it, doubt if it really saves power no	Jingdong prefecture level city in Shandong
Fortune Shop	Green Intelligent Power Saver	It's good quality and works well!	Jingdong prefecture level city in Shandong
Fortune Shop	Green Intelligent Power Saver	hahaha just right hahahahahaha hiccup hahahaha	Jingdong prefecture level city in Shandong
Fortune Shop	Green Intelligent Power Saver	The real thing matches the picture and looks great!	Jingdong prefecture level city in Shandong
Fortune Shop	Green Intelligent Power Saver	Plugged it in and we'll see when we pay the bills at the end of the year!	Jingdong prefecture level city in Shandong
Fortune Shop	Green Intelligent Power Saver	False advertising. It doesn't work.	Jingdong prefecture level city in Shandong
Fortune Shop	Green Intelligent Power Saver	Goods received, good effect	Jingdong prefecture level city in Shandong
Fortune Shop	Green Intelligent Power Saver	It looks okay, we'll see if it saves electricity when the time comes	Jingdong prefecture level city in Shandong

Fortune Shop	Green Intelligent Power Saver	Logistics is very fast quality is very good, the price is very affordable, accessories are also complete, installed, satisfied with good use	Jingdong prefecture level city in Shandong
Fortune Shop	Green Intelligent Power Saver	I've received the power saver. I'm looking forward to the savings.	Jingdong prefecture level city in Shandong
Fortune Shop	Green Intelligent Power Saver	Received, have not yet used, after use and then evaluate	Jingdong prefecture level city in Shandong
Fortune Shop	Green Intelligent Power Saver	I received the item, but I don't know if it saves power or not.	Jingdong prefecture level city in Shandong
Fortune Shop	Green Intelligent Power Saver	On Trial	Jingdong prefecture level city in Shandong
Fortune Shop	Green Intelligent Power Saver	Use it and see how it goes.	Jingdong prefecture level city in Shandong
Fortune Shop	Green Intelligent Power Saver	Quality is very good, no odor, fine workmanship, grain color are very like, satisfied!	Jingdong prefecture level city in Shandong
Fortune Shop	Green Intelligent Power Saver	I'll give you a good review. I don't know if it'll work.	Jingdong prefecture level city in Shandong
Fortune Shop	Green Intelligent Power Saver	The logistics is very fast and the package is intact. Good use of affordable. The effect is very good, is genuine	Jingdong prefecture level city in Shandong
Fortune Shop	Green Intelligent Power Saver	Nothing happens when you plug it in.	Jingdong prefecture level city in Shandong
Fortune Shop	Green Intelligent Power Saver	I don't know if it's as good as it sounds.	Jingdong prefecture level city in Shandong
Fortune Shop	Green Intelligent Power Saver	The quality of the stuff is very good, exactly the same as the seller's description, very satisfied!	Jingdong prefecture level city in Shandong
Fortune Shop	Green Intelligent Power Saver	The saver works great, it really works, it's affordable and worth buying.	Jingdong prefecture level city in Shandong
Fortune Shop	Green Intelligent Power Saver	Just used it for a couple days. I don't know yet.	Jingdong prefecture level city in Shandong
Fortune Shop	Green Intelligent Power Saver	The quality is quite good, cheaper than the physical store, good, next time will come again!	Jingdong prefecture level city in Shandong
Fortune Shop	Green Intelligent Power Saver	Good quality, cheap and affordable worth recommending to buy!	Jingdong prefecture level city in Shandong
Fortune Shop	Green Intelligent Power Saver	Very good quality, well made, works well! Satisfied	Jingdong prefecture level city in Shandong
Fortune Shop	Green Intelligent Power Saver	The logistics is very fast quality is very good very pleasant a shopping	Jingdong prefecture level city in Shandong
Fortune Shop	Green Intelligent Power Saver	Use it and then come back to evaluate!	Jingdong prefecture level city in Shandong
Fortune Shop	Green Intelligent Power Saver	# It's no use lying at all #	Jingdong prefecture level city in Shandong
Fortune Shop	Green Intelligent Power Saver	I can't get a voltage drop.	Jingdong prefecture level city in Shandong
Fortune Shop	Green Intelligent Power Saver	Evaluate after use	Jingdong prefecture level city in Shandong
Fortune Shop	Green Intelligent Power Saver	Still on trial.	Jingdong prefecture level city in Shandong
Fortune Shop	Green Intelligent Power Saver	ineffective	Jingdong prefecture level city in Shandong
Fortune Shop	Green Intelligent Power Saver	Satisfaction Shopping	Jingdong prefecture level city in Shandong
Fortune Shop	Green Intelligent Power Saver	Lies.	Jingdong prefecture level city in Shandong
Fortune Shop	Green Intelligent Power Saver	It's a real power saver.	Jingdong prefecture level city in Shandong
Fortune Shop	Green Intelligent Power Saver	in use	Jingdong prefecture level city in Shandong
Fortune Shop	Green Intelligent Power Saver	I don't know yet.	Jingdong prefecture level city in Shandong
Fortune Shop	Green Intelligent Power Saver	not care about anything	Jingdong prefecture level city in Shandong
Fortune Shop	Green Intelligent Power Saver	It doesn't seem to have much effect	Jingdong prefecture level city in Shandong
Fortune Shop	Green Intelligent Power Saver	Let the merchant see for himself, does the plug positive and negative terminals look like this?	Jingdong prefecture level city in Shandong
Fortune Shop	Green Intelligent Power Saver	It doesn't work well, the electricity bill is less before using it, but more after using it, contacting customer service, the service is still not good, and returning it is slow	Jingdong prefecture level city in Shandong
Fortune Shop	Green Intelligent Power Saver	With the advertisement product does not match, above the effective negative, power saving rate of two rows of words it, the product looks very thin, with 3 days to see the meter number is still with the previous no difference, which is obviously fraudulent behavior!	Jingdong prefecture level city in Shandong
Fortune Shop	Green Intelligent Power Saver	Doesn't save any power at all. Not much use.	Jingdong prefecture level city in Shandong
Fortune Shop	Green Intelligent Power Saver	The plugs are all loose, they're all the same length and they don't work at all	Jingdong prefecture level city in Shandong
Fortune Shop	Green Intelligent Power Saver	It's bad 👎 it's not effective at all, you'd be crazy to sell this garbage on the internet.	Jingdong prefecture level city in Shandong
Fortune Shop	Green Intelligent Power Saver	It doesn't save any power at all, so I hope the old timers don't fall for this scam again	Jingdong prefecture level city in Shandong
Fortune Shop	Green Intelligent Power Saver	I've been using it for over a month now and haven't noticed any power savings.	Jingdong prefecture level city in Shandong
Fortune Shop	Green Intelligent Power Saver	It doesn't work at all. The electricity bill is higher when you use it.	Jingdong prefecture level city in Shandong
Fortune Shop	Green Intelligent Power Saver	It doesn't save any power, it's still as bad as ever.	Jingdong prefecture level city in Shandong
Fortune Shop	Green Intelligent Power Saver	Last night before leaving work to look at the table, and then plugged into the node device, this morning and then look at the table, than the original save a few degrees of electricity, very good to have a power-saving effect, easy to use, satisfied with the shopping praise.	Jingdong prefecture level city in Shandong
Fortune Shop	Green Intelligent Power Saver	The product is real and effective, the electricity bill is reduced, really good, thanks to the merchants have so many good products, the product is real and effective, the electricity bill is reduced, want to save electricity friends quickly buy it, early use early to save money.	Jingdong prefecture level city in Shandong
Fortune Shop	Green Intelligent Power Saver	Saver saves time and money, baby appearance is beautiful, the quality of goods is good, and the same as the business description, good service attitude, packaging is complete, logistics is very fast, praise to	Jingdong prefecture level city in Shandong
Fortune Shop	Green Intelligent Power Saver	The effect is good, summer, home electricity is relatively large, buy and use a few days, feel the effect is very good, originally ten dollars a day electricity bill, now eight dollars a day, quite good	Jingdong prefecture level city in Shandong
Fortune Shop	Green Intelligent Power Saver	Indeed can save electricity and money, the first time I know this or from a friend's home to see, but also do not believe it, and now they use it to know that there is this good thing.	Jingdong prefecture level city in Shandong
Fortune Shop	Green Intelligent Power Saver	I have received the Smart Power Saver. Perfectly packaged. Fast delivery. Boss customer service attitude is also very good. It's worth buying. I plugged it in and tried it and it really saves more power than the original. I'll buy it again next time.	Jingdong prefecture level city in Shandong
Fortune Shop	Green Intelligent Power Saver	It's nice to save a dozen bucks a night on two air conditioners that used to take 30 or 40 degrees a night, but now it's only 10 or so degrees a night	Jingdong prefecture level city in Shandong
Fortune Shop	Green Intelligent Power Saver	Product no manufacturer's name no product qualification certificate, try two days to see the effect of	Jingdong prefecture level city in Shandong

Fortune Shop	Green Intelligent Power Saver	I've been using it for over a month and have saved over $80, so I've repurchased a few more back to save together	Jingdong prefecture level city in Shandong
Fortune Shop	Green Intelligent Power Saver	I haven't used it yet and will comment when I do, but it's well made.	Jingdong prefecture level city in Shandong
Fortune Shop	Green Intelligent Power Saver	It's quite energy efficient. It's really a boon for the home.	Jingdong prefecture level city in Shandong
Fortune Shop	Green Intelligent Power Saver	Energy-saving socket received a month, after using it for a month proved that it is indeed energy-saving, electricity bill saving, will buy it again, thank you!	Jingdong prefecture level city in Shandong
Fortune Shop	Green Intelligent Power Saver	There was a similar product many years ago. Now technology has evolved, buy one to try, according to the instructions in the box, it takes one or two dozen days to show results. Check your electric bill and see what happens.	Jingdong prefecture level city in Shandong
Fortune Shop	Green Intelligent Power Saver	Jingdong logistics is really fast, the same day the order arrived on the same day, more convenient than the supermarket, and a wide range of choices, the	Jingdong prefecture level city in Shandong
Fortune Shop	Green Intelligent Power Saver	I've been using this for a month or so and I can't see any results, it really is a scam as they say.	Jingdong prefecture level city in Shandong
Fortune Shop	Green Intelligent Power Saver	Very good use, a month can save a few degrees of power loss of electricity, introduced to the relatives to buy back to try.	Jingdong prefecture level city in Shandong
Fortune Shop	Green Intelligent Power Saver	It's really powerful! With this air conditioner can be turned on at any time without the pressure of paying the electricity bill! It's good for the people!	Jingdong prefecture level city in Shandong
Fortune Shop	Green Intelligent Power Saver	This business is a big ** products are not qualified, with a month of electricity more than one hundred percent of thirty, the purchase agreed to return the goods but do not receive a refund after receiving the goods, looking for the official side does not help, the Jingdong is a big **, I have never shopped in	Jingdong prefecture level city in Shandong
Fortune Shop	Green Intelligent Power Saver	It's good. I tried it. It saves power.	Jingdong prefecture level city in Shandong
Fortune Shop	Green Intelligent Power Saver	Nothing**, it seems like there's more	Jingdong prefecture level city in Shandong
Fortune Shop	Green Intelligent Power Saver	Power saving device received, well packaged, good quality, fast logistics	Jingdong prefecture level city in Shandong
Fortune Shop	Green Intelligent Power Saver	The service is okay, is that when I bought five, the desirable pieces are three, and then wait a few days and send me two replacement, I do not know if it is a mistake of the shipper, if I was not in the courier there to see, go home to say no, the	Jingdong prefecture level city in Shandong
Fortune Shop	Green Intelligent Power Saver	This thing will only cost me more if I use it, but before I used it, it was less than a ~ electricity a month, and after I used it, it was more than two hundred kWh a month.	Jingdong prefecture level city in Shandong
Fortune Shop	Green Intelligent Power Saver	I just used it. I don't know how it is.	Jingdong prefecture level city in Shandong
Fortune Shop	Green Intelligent Power Saver	I've received the product and I'm using it, so I'll see how it goes in a month!	Jingdong prefecture level city in Shandong
Fortune Shop	Green Intelligent Power Saver	The goods have just received the effect do not know how, please look forward to the results.	Jingdong prefecture level city in Shandong
Fortune Shop	Green Intelligent Power Saver	Power saving device, the material is very good practical and durable, the size is suitable, packaging is very good, the price is cheap, logistics is very fast!	Jingdong prefecture level city in Shandong
Fortune Shop	Green Intelligent Power Saver	Very nice, will comment in detail after using it for a while.	Jingdong prefecture level city in Shandong
Fortune Shop	Green Intelligent Power Saver	Good stuff to trust and support	Jingdong prefecture level city in Shandong
Fortune Shop	Green Intelligent Power Saver	I'll comment after I use it, doubt if it really saves power no	Jingdong prefecture level city in Shandong
Fortune Shop	Green Intelligent Power Saver	It's good quality and works well!	Jingdong prefecture level city in Shandong
Fortune Shop	Green Intelligent Power Saver	hahaha just right hahahahahaha hiccup hahahaha	Jingdong prefecture level city in Shandong
Fortune Shop	Green Intelligent Power Saver	The real thing matches the picture and looks great!	Jingdong prefecture level city in Shandong
Fortune Shop	Green Intelligent Power Saver	Plugged it in and we'll see when we pay the bills at the end of the year!	Jingdong prefecture level city in Shandong
Fortune Shop	Green Intelligent Power Saver	False advertising. It doesn't work.	Jingdong prefecture level city in Shandong
Fortune Shop	Green Intelligent Power Saver	Goods received, good effect	Jingdong prefecture level city in Shandong
Fortune Shop	Green Intelligent Power Saver	It looks okay, we'll see if it saves electricity when the time comes	Jingdong prefecture level city in Shandong
Fortune Shop	Green Intelligent Power Saver	Logistics is very fast quality is very good, the price is very affordable, accessories are also complete, installed, satisfied with good use	Jingdong prefecture level city in Shandong
Fortune Shop	Green Intelligent Power Saver	I've received the power saver. I'm looking forward to the savings.	Jingdong prefecture level city in Shandong
Fortune Shop	Green Intelligent Power Saver	Received, have not yet used, after use and then evaluate	Jingdong prefecture level city in Shandong
Fortune Shop	Green Intelligent Power Saver	I received the item, but I don't know if it saves power or not.	Jingdong prefecture level city in Shandong
Fortune Shop	Green Intelligent Power Saver	On Trial	Jingdong prefecture level city in Shandong
Fortune Shop	Green Intelligent Power Saver	Use it and see how it goes.	Jingdong prefecture level city in Shandong
Fortune Shop	Green Intelligent Power Saver	Quality is very good, no odor, fine workmanship, grain color are very like, satisfied!	Jingdong prefecture level city in Shandong
Fortune Shop	Green Intelligent Power Saver	I'll give you a good review. I don't know if it'll work.	Jingdong prefecture level city in Shandong
Fortune Shop	Green Intelligent Power Saver	The logistics is very fast and the package is intact. Good use of affordable. The effect is very good, is genuine	Jingdong prefecture level city in Shandong
Fortune Shop	Green Intelligent Power Saver	Nothing happens when you plug it in.	Jingdong prefecture level city in Shandong
Fortune Shop	Green Intelligent Power Saver	I don't know if it's as good as it sounds.	Jingdong prefecture level city in Shandong
Fortune Shop	Green Intelligent Power Saver	The quality of the stuff is very good, exactly the same as the seller's description, very satisfied!	Jingdong prefecture level city in Shandong
Fortune Shop	Green Intelligent Power Saver	The saver works great, it really works, it's affordable and worth buying.	Jingdong prefecture level city in Shandong
Fortune Shop	Green Intelligent Power Saver	Just used it for a couple days. I don't know yet.	Jingdong prefecture level city in Shandong
Fortune Shop	Green Intelligent Power Saver	The quality is quite good, cheaper than the physical store, good, next time will come again!	Jingdong prefecture level city in Shandong
Fortune Shop	Green Intelligent Power Saver	Good quality, cheap and affordable worth recommending to buy!	Jingdong prefecture level city in Shandong
Fortune Shop	Green Intelligent Power Saver	Very good quality, well made, works well! Satisfied	Jingdong prefecture level city in Shandong
Fortune Shop	Green Intelligent Power Saver	The logistics is very fast quality is very good very pleasant a shopping	Jingdong prefecture level city in Shandong
Fortune Shop	Green Intelligent Power Saver	Use it and then come back to evaluate!	Jingdong prefecture level city in Shandong
Fortune Shop	Green Intelligent Power Saver	# It's no use lying at all #	Jingdong prefecture level city in Shandong
Fortune Shop	Green Intelligent Power Saver	I can't get a voltage drop.	Jingdong prefecture level city in Shandong

Fortune Shop	Green Intelligent Power Saver	Evaluate after use	Jingdong prefecture level city in Shandong
Fortune Shop	Green Intelligent Power Saver	Still on trial.	Jingdong prefecture level city in Shandong
Fortune Shop	Green Intelligent Power Saver	ineffective	Jingdong prefecture level city in Shandong
Fortune Shop	Green Intelligent Power Saver	Satisfaction Shopping	Jingdong prefecture level city in Shandong
Fortune Shop	Green Intelligent Power Saver	Lies.	Jingdong prefecture level city in Shandong
Fortune Shop	Green Intelligent Power Saver	It's a real power saver.	Jingdong prefecture level city in Shandong
Fortune Shop	Green Intelligent Power Saver	in use	Jingdong prefecture level city in Shandong
Fortune Shop	Green Intelligent Power Saver	I don't know yet.	Jingdong prefecture level city in Shandong
Fortune Shop	Green Intelligent Power Saver	not care about anything	Jingdong prefecture level city in Shandong
Fortune Shop	Green Intelligent Power Saver	It doesn't seem to have much effect	Jingdong prefecture level city in Shandong
Fortune Shop	Green Intelligent Power Saver	Let the merchant see for himself, does the plug positive and negative terminals look like this?	Jingdong prefecture level city in Shandong
Fortune Shop	Green Intelligent Power Saver	It doesn't work well, the electricity bill is less before using it, but more after using it, contacting customer service, the service is still not good, and returning it is slow	Jingdong prefecture level city in Shandong
Fortune Shop	Green Intelligent Power Saver	With the advertisement product does not match, above the effective negative, power saving rate of two rows of words it, the product looks very thin, with 3 days to see the meter number is still with the previous no difference, which is obviously fraudulent behavior!	Jingdong prefecture level city in Shandong
Fortune Shop	Green Intelligent Power Saver	Doesn't save any power at all. Not much use.	Jingdong prefecture level city in Shandong
Fortune Shop	Green Intelligent Power Saver	The plugs are all loose, they're all the same length and they don't work at all	Jingdong prefecture level city in Shandong
Fortune Shop	Green Intelligent Power Saver	It's bad 👎 it's not effective at all, you'd be crazy to sell this garbage on the internet.	Jingdong prefecture level city in Shandong
Fortune Shop	Green Intelligent Power Saver	It doesn't save any power at all, so I hope the old timers don't fall for this scam again	Jingdong prefecture level city in Shandong
Fortune Shop	Green Intelligent Power Saver	I've been using it for over a month now and haven't noticed any power savings.	Jingdong prefecture level city in Shandong
Fortune Shop	Green Intelligent Power Saver	It doesn't work at all. The electricity bill is higher when you use it.	Jingdong prefecture level city in Shandong
Fortune Shop	Green Intelligent Power Saver	It doesn't save any power, it's still as bad as ever.	Jingdong prefecture level city in Shandong
Fortune Shop	Green Intelligent Power Saver	Last night before leaving work to look at the table, and then plugged into the node device, this morning and then look at the table, than the original save a few degrees of electricity, very good to have a power-saving effect, easy to use, satisfied with the shopping praise.	Jingdong prefecture level city in Shandong
Fortune Shop	Green Intelligent Power Saver	The product is real and effective, the electricity bill is reduced, really good, thanks to the merchants have so many good products, the product is real and effective, the electricity bill is reduced, want to save electricity friends quickly buy it, early use early to save money.	Jingdong prefecture level city in Shandong
Fortune Shop	Green Intelligent Power Saver	Saver saves time and money, baby appearance is beautiful, the quality of goods is good, and the same as the business description, good service attitude, packaging is complete, logistics is very fast, praise to	Jingdong prefecture level city in Shandong
Fortune Shop	Green Intelligent Power Saver	The effect is good, summer, home electricity is relatively large, buy and use a few days, feel the effect is very good, originally ten dollars a day electricity bill, now eight dollars a day, quite good	Jingdong prefecture level city in Shandong
Fortune Shop	Green Intelligent Power Saver	Indeed can save electricity and money, the first time I know this or from a friend's home to see, but also do not believe it, and now they use it to know that there is this good thing.	Jingdong prefecture level city in Shandong
Fortune Shop	Green Intelligent Power Saver	I have received the Smart Power Saver. Perfectly packaged. Fast delivery. Boss customer service attitude is also very good. It's worth buying. I plugged it in and tried it and it really saves more power than the original. I'll buy it again next time.	Jingdong prefecture level city in Shandong
Fortune Shop	Green Intelligent Power Saver	It's nice to save a dozen bucks a night on two air conditioners that used to take 30 or 40 degrees a night, but now it's only 10 or so degrees a night	Jingdong prefecture level city in Shandong
Fortune Shop	Green Intelligent Power Saver	Product no manufacturer's name no product qualification certificate, try two days to see the effect of	Jingdong prefecture level city in Shandong
Fortune Shop	Green Intelligent Power Saver	I've been using it for over a month and have saved over $80, so I've repurchased a few more back to save together	Jingdong prefecture level city in Shandong
Fortune Shop	Green Intelligent Power Saver	I haven't used it yet and will comment when I do, but it's well made.	Jingdong prefecture level city in Shandong
Fortune Shop	Green Intelligent Power Saver	It's quite energy efficient. It's really a boon for the home.	Jingdong prefecture level city in Shandong
Fortune Shop	Green Intelligent Power Saver	Energy-saving socket received a month, after using it for a month proved that it is indeed energy-saving, electricity bill saving, will buy it again, thank you!	Jingdong prefecture level city in Shandong
Fortune Shop	Green Intelligent Power Saver	There was a similar product many years ago. Now technology has evolved, buy one to try, according to the instructions in the box, it takes one or two dozen days to show results. Check your electric bill and see what happens.	Jingdong prefecture level city in Shandong
Fortune Shop	Green Intelligent Power Saver	Jingdong logistics is really fast, the same day the order arrived on the same day, more convenient than the supermarket, and a wide range of choices, the	Jingdong prefecture level city in Shandong
Fortune Shop	Green Intelligent Power Saver	I've been using this for a month or so and I can't see any results, it really is a scam as they say.	Jingdong prefecture level city in Shandong
Fortune Shop	Green Intelligent Power Saver	Very good use, a month can save a few degrees of power loss of electricity, introduced to the relatives to buy back to try.	Jingdong prefecture level city in Shandong
Fortune Shop	Green Intelligent Power Saver	It's really powerful! With this air conditioner can be turned on at any time without the pressure of paying the electricity bill! It's good for the people!	Jingdong prefecture level city in Shandong
Fortune Shop	Green Intelligent Power Saver	This business is a big ** products are not qualified, with a month of electricity more than one hundred percent of thirty, the purchase agreed to return the goods but do not receive a refund after receiving the goods, looking for the official side does not help, the Jingdong is a big **, I have never shopped in	Jingdong prefecture level city in Shandong
Fortune Shop	Green Intelligent Power Saver	It's good. I tried it. It saves power.	Jingdong prefecture level city in Shandong
Fortune Shop	Green Intelligent Power Saver	Nothing**, it seems like there's more	Jingdong prefecture level city in Shandong
Fortune Shop	Green Intelligent Power Saver	Power saving device received, well packaged, good quality, fast logistics	Jingdong prefecture level city in Shandong
Fortune Shop	Green Intelligent Power Saver	The service is okay, is that when I bought five, the desirable pieces are three, and then wait a few days and send me two replacement, I do not know if it is a mistake of the shipper, if I was not in the courier there to see, go home to say no, the	Jingdong prefecture level city in Shandong
Fortune Shop	Green Intelligent Power Saver	This thing will only cost me more if I use it, but before I used it, it was less than a ~ electricity a month, and after I used it, it was more than two hundred kWh a month.	Jingdong prefecture level city in Shandong
Fortune Shop	Green Intelligent Power Saver	I just used it. I don't know how it is.	Jingdong prefecture level city in Shandong
Fortune Shop	Green Intelligent Power Saver	I've received the product and I'm using it, so I'll see how it goes in a month!	Jingdong prefecture level city in Shandong
Fortune Shop	Green Intelligent Power Saver	The goods have just received the effect do not know how, please look forward to the results.	Jingdong prefecture level city in Shandong
Fortune Shop	Green Intelligent Power Saver	Power saving device, the material is very good practical and durable, the size is suitable, packaging is very good, the price is cheap, logistics is very fast!	Jingdong prefecture level city in Shandong
Fortune Shop	Green Intelligent Power Saver	Very nice, will comment in detail after using it for a while.	Jingdong prefecture level city in Shandong

Fortune Shop	Green Intelligent Power Saver	Good stuff to trust and support	Jingdong prefecture level city in Shandong
Fortune Shop	Green Intelligent Power Saver	I'll comment after I use it, doubt if it really saves power no	Jingdong prefecture level city in Shandong
Fortune Shop	Green Intelligent Power Saver	It's good quality and works well!	Jingdong prefecture level city in Shandong
Fortune Shop	Green Intelligent Power Saver	hahaha just right hahahahahaha hiccup hahahaha	Jingdong prefecture level city in Shandong
Fortune Shop	Green Intelligent Power Saver	The real thing matches the picture and looks great!	Jingdong prefecture level city in Shandong
Fortune Shop	Green Intelligent Power Saver	Plugged it in and we'll see when we pay the bills at the end of the year!	Jingdong prefecture level city in Shandong
Fortune Shop	Green Intelligent Power Saver	False advertising. It doesn't work.	Jingdong prefecture level city in Shandong
Fortune Shop	Green Intelligent Power Saver	Goods received, good effect	Jingdong prefecture level city in Shandong
Fortune Shop	Green Intelligent Power Saver	It looks okay, we'll see if it saves electricity when the time comes	Jingdong prefecture level city in Shandong
Fortune Shop	Green Intelligent Power Saver	Logistics is very fast quality is very good, the price is very affordable, accessories are also complete, installed, satisfied with good use	Jingdong prefecture level city in Shandong
Fortune Shop	Green Intelligent Power Saver	I've received the power saver. I'm looking forward to the savings.	Jingdong prefecture level city in Shandong
Fortune Shop	Green Intelligent Power Saver	Received, have not yet used, after use and then evaluate	Jingdong prefecture level city in Shandong
Fortune Shop	Green Intelligent Power Saver	I received the item, but I don't know if it saves power or not.	Jingdong prefecture level city in Shandong
Fortune Shop	Green Intelligent Power Saver	On Trial	Jingdong prefecture level city in Shandong
Fortune Shop	Green Intelligent Power Saver	Use it and see how it goes.	Jingdong prefecture level city in Shandong
Fortune Shop	Green Intelligent Power Saver	Quality is very good, no odor, fine workmanship, grain color are very like, satisfied!	Jingdong prefecture level city in Shandong
Fortune Shop	Green Intelligent Power Saver	I'll give you a good review. I don't know if it'll work.	Jingdong prefecture level city in Shandong
Fortune Shop	Green Intelligent Power Saver	The logistics is very fast and the package is intact. Good use of affordable. The effect is very good, is genuine	Jingdong prefecture level city in Shandong
Fortune Shop	Green Intelligent Power Saver	Nothing happens when you plug it in.	Jingdong prefecture level city in Shandong
Fortune Shop	Green Intelligent Power Saver	I don't know if it's as good as it sounds.	Jingdong prefecture level city in Shandong
Fortune Shop	Green Intelligent Power Saver	The quality of the stuff is very good, exactly the same as the seller's description, very satisfied!	Jingdong prefecture level city in Shandong
Fortune Shop	Green Intelligent Power Saver	The saver works great, it really works, it's affordable and worth buying.	Jingdong prefecture level city in Shandong
Fortune Shop	Green Intelligent Power Saver	Just used it for a couple days. I don't know yet.	Jingdong prefecture level city in Shandong
Fortune Shop	Green Intelligent Power Saver	The quality is quite good, cheaper than the physical store, good, next time will come again!	Jingdong prefecture level city in Shandong
Fortune Shop	Green Intelligent Power Saver	Good quality, cheap and affordable worth recommending to buy!	Jingdong prefecture level city in Shandong
Fortune Shop	Green Intelligent Power Saver	Very good quality, well made, works well! Satisfied	Jingdong prefecture level city in Shandong
Fortune Shop	Green Intelligent Power Saver	The logistics is very fast quality is very good very pleasant a shopping	Jingdong prefecture level city in Shandong
Fortune Shop	Green Intelligent Power Saver	Use it and then come back to evaluate!	Jingdong prefecture level city in Shandong
Fortune Shop	Green Intelligent Power Saver	# It's no use lying at all #	Jingdong prefecture level city in Shandong
Fortune Shop	Green Intelligent Power Saver	I can't get a voltage drop.	Jingdong prefecture level city in Shandong
Fortune Shop	Green Intelligent Power Saver	Evaluate after use	Jingdong prefecture level city in Shandong
Fortune Shop	Green Intelligent Power Saver	Still on trial.	Jingdong prefecture level city in Shandong
Fortune Shop	Green Intelligent Power Saver	ineffective	Jingdong prefecture level city in Shandong
Fortune Shop	Green Intelligent Power Saver	Satisfaction Shopping	Jingdong prefecture level city in Shandong
Fortune Shop	Green Intelligent Power Saver	Lies.	Jingdong prefecture level city in Shandong
Fortune Shop	Green Intelligent Power Saver	It's a real power saver.	Jingdong prefecture level city in Shandong
Fortune Shop	Green Intelligent Power Saver	in use	Jingdong prefecture level city in Shandong
Fortune Shop	Green Intelligent Power Saver	I don't know yet.	Jingdong prefecture level city in Shandong
Fortune Shop	Green Intelligent Power Saver	not care about anything	Jingdong prefecture level city in Shandong
Fortune Shop	Green Intelligent Power Saver	It doesn't seem to have much effect	Jingdong prefecture level city in Shandong
Fortune Shop	Green Intelligent Power Saver	Let the merchant see for himself, does the plug positive and negative terminals look like this?	Jingdong prefecture level city in Shandong
Fortune Shop	Green Intelligent Power Saver	It doesn't work well, the electricity bill is less before using it, but more after using it, contacting customer service, the service is still not good, and returning it is slow	Jingdong prefecture level city in Shandong
Fortune Shop	Green Intelligent Power Saver	With the advertisement product does not match, above the effective negative, power saving rate of two rows of words it, the product looks very thin, with 3 days to see the meter number is still with the previous no difference, which is obviously fraudulent behavior!	Jingdong prefecture level city in Shandong
Fortune Shop	Green Intelligent Power Saver	Doesn't save any power at all. Not much use.	Jingdong prefecture level city in Shandong
Fortune Shop	Green Intelligent Power Saver	The plugs are all loose, they're all the same length and they don't work at all	Jingdong prefecture level city in Shandong
Fortune Shop	Green Intelligent Power Saver	It's bad 👎 it's not effective at all, you'd be crazy to sell this garbage on the internet.	Jingdong prefecture level city in Shandong
Fortune Shop	Green Intelligent Power Saver	It doesn't save any power at all, so I hope the old timers don't fall for this scam again	Jingdong prefecture level city in Shandong
Fortune Shop	Green Intelligent Power Saver	I've been using it for over a month now and haven't noticed any power savings.	Jingdong prefecture level city in Shandong
Fortune Shop	Green Intelligent Power Saver	It doesn't work at all. The electricity bill is higher when you use it.	Jingdong prefecture level city in Shandong
Fortune Shop	Green Intelligent Power Saver	It doesn't save any power, it's still as bad as ever.	Jingdong prefecture level city in Shandong

Fortune Shop	Green Intelligent Power Saver	Last night before leaving work to look at the table, and then plugged into the node device, this morning and then look at the table, than the original save a few degrees of electricity, very good to have a power-saving effect, easy to use, satisfied with the shopping praise.	Jingdong prefecture level city in Shandong
Fortune Shop	Green Intelligent Power Saver	The product is real and effective, the electricity bill is reduced, really good, thanks to the merchants have so many good products, the product is real and effective, the electricity bill is reduced, want to save electricity friends quickly buy it, early use early to save money.	Jingdong prefecture level city in Shandong
Fortune Shop	Green Intelligent Power Saver	Saver saves time and money, baby appearance is beautiful, the quality of goods is good, and the same as the business description, good service attitude, packaging is complete, logistics is very fast, praise to	Jingdong prefecture level city in Shandong
Fortune Shop	Green Intelligent Power Saver	The effect is good, summer, home electricity is relatively large, buy and use a few days, feel the effect is very good, originally ten dollars a day electricity bill, now eight dollars a day, quite good	Jingdong prefecture level city in Shandong
Fortune Shop	Green Intelligent Power Saver	Indeed can save electricity and money, the first time I know this or from a friend's home to see, but also do not believe it, and now they use it to know that there is this good thing.	Jingdong prefecture level city in Shandong
Fortune Shop	Green Intelligent Power Saver	I have received the Smart Power Saver. Perfectly packaged. Fast delivery. Boss customer service attitude is also very good. It's worth buying. I plugged it in and tried it and it really saves more power than the original. I'll buy it again next time.	Jingdong prefecture level city in Shandong
Fortune Shop	Green Intelligent Power Saver	It's nice to save a dozen bucks a night on two air conditioners that used to take 30 or 40 degrees a night, but now it's only 10 or so degrees a night	Jingdong prefecture level city in Shandong
Fortune Shop	Green Intelligent Power Saver	Product no manufacturer's name no product qualification certificate, try two days to see the effect of	Jingdong prefecture level city in Shandong
Fortune Shop	Green Intelligent Power Saver	I've been using it for over a month and have saved over $80, so I've repurchased a few more back to save together	Jingdong prefecture level city in Shandong
Fortune Shop	Green Intelligent Power Saver	I haven't used it yet and will comment when I do, but it's well made.	Jingdong prefecture level city in Shandong
Fortune Shop	Green Intelligent Power Saver	It's quite energy efficient. It's really a boon for the home.	Jingdong prefecture level city in Shandong
Fortune Shop	Green Intelligent Power Saver	Energy-saving socket received a month, after using it for a month proved that it is indeed energy-saving, electricity bill saving, will buy it again, thank you!	Jingdong prefecture level city in Shandong
Fortune Shop	Green Intelligent Power Saver	There was a similar product many years ago. Now technology has evolved, buy one to try, according to the instructions in the box, it takes one or two dozen days to show results. Check your electric bill and see what happens.	Jingdong prefecture level city in Shandong
Fortune Shop	Green Intelligent Power Saver	Jingdong logistics is really fast, the same day the order arrived on the same day, more convenient than the supermarket, and a wide range of choices, the	Jingdong prefecture level city in Shandong
Fortune Shop	Green Intelligent Power Saver	I've been using this for a month or so and I can't see any results, it really is a scam as they say.	Jingdong prefecture level city in Shandong
Fortune Shop	Green Intelligent Power Saver	Very good use, a month can save a few degrees of power loss of electricity, introduced to the relatives to buy back to try.	Jingdong prefecture level city in Shandong
Fortune Shop	Green Intelligent Power Saver	It's really powerful! With this air conditioner can be turned on at any time without the pressure of paying the electricity bill! It's good for the people!	Jingdong prefecture level city in Shandong
Fortune Shop	Green Intelligent Power Saver	This business is a big ** products are not qualified, with a month of electricity more than one hundred percent of thirty, the purchase agreed to return the goods but do not receive a refund after receiving the goods, looking for the official side does not help, the Jingdong is a big **, I have never shopped in	Jingdong prefecture level city in Shandong
Fortune Shop	Green Intelligent Power Saver	It's good. I tried it. It saves power.	Jingdong prefecture level city in Shandong
Fortune Shop	Green Intelligent Power Saver	Nothing**, it seems like there's more	Jingdong prefecture level city in Shandong
Fortune Shop	Green Intelligent Power Saver	Power saving device received, well packaged, good quality, fast logistics	Jingdong prefecture level city in Shandong
Fortune Shop	Green Intelligent Power Saver	The service is okay, is that when I bought five, the desirable pieces are three, and then wait a few days and send me two replacement, I do not know if it is a mistake of the shipper, if I was not in the courier there to see, go home to say no, the	Jingdong prefecture level city in Shandong
Fortune Shop	Green Intelligent Power Saver	This thing will only cost me more if I use it, but before I used it, it was less than a ~ electricity a month, and after I used it, it was more than two hundred kWh a month.	Jingdong prefecture level city in Shandong
Fortune Shop	Green Intelligent Power Saver	I just used it. I don't know how it is.	Jingdong prefecture level city in Shandong
Fortune Shop	Green Intelligent Power Saver	I've received the product and I'm using it, so I'll see how it goes in a month!	Jingdong prefecture level city in Shandong
Fortune Shop	Green Intelligent Power Saver	The goods have just received the effect do not know how, please look forward to the results.	Jingdong prefecture level city in Shandong
Fortune Shop	Green Intelligent Power Saver	Power saving device, the material is very good practical and durable, the size is suitable, packaging is very good, the price is cheap, logistics is very fast!	Jingdong prefecture level city in Shandong
Fortune Shop	Green Intelligent Power Saver	Very nice, will comment in detail after using it for a while.	Jingdong prefecture level city in Shandong
Fortune Shop	Green Intelligent Power Saver	Good stuff to trust and support	Jingdong prefecture level city in Shandong
Fortune Shop	Green Intelligent Power Saver	I'll comment after I use it, doubt if it really saves power no	Jingdong prefecture level city in Shandong
Fortune Shop	Green Intelligent Power Saver	It's good quality and works well!	Jingdong prefecture level city in Shandong
Fortune Shop	Green Intelligent Power Saver	hahaha just right hahahahahaha hiccup hahahaha	Jingdong prefecture level city in Shandong
Fortune Shop	Green Intelligent Power Saver	The real thing matches the picture and looks great!	Jingdong prefecture level city in Shandong
Fortune Shop	Green Intelligent Power Saver	Plugged it in and we'll see when we pay the bills at the end of the year!	Jingdong prefecture level city in Shandong
Fortune Shop	Green Intelligent Power Saver	False advertising. It doesn't work.	Jingdong prefecture level city in Shandong
Fortune Shop	Green Intelligent Power Saver	Goods received, good effect	Jingdong prefecture level city in Shandong
Fortune Shop	Green Intelligent Power Saver	It looks okay, we'll see if it saves electricity when the time comes	Jingdong prefecture level city in Shandong
Fortune Shop	Green Intelligent Power Saver	Logistics is very fast quality is very good, the price is very affordable, accessories are also complete, installed, satisfied with good use	Jingdong prefecture level city in Shandong
Fortune Shop	Green Intelligent Power Saver	I've received the power saver. I'm looking forward to the savings.	Jingdong prefecture level city in Shandong
Fortune Shop	Green Intelligent Power Saver	Received, have not yet used, after use and then evaluate	Jingdong prefecture level city in Shandong
Fortune Shop	Green Intelligent Power Saver	I received the item, but I don't know if it saves power or not.	Jingdong prefecture level city in Shandong
Fortune Shop	Green Intelligent Power Saver	On Trial	Jingdong prefecture level city in Shandong
Fortune Shop	Green Intelligent Power Saver	Use it and see how it goes.	Jingdong prefecture level city in Shandong
Fortune Shop	Green Intelligent Power Saver	Quality is very good, no odor, fine workmanship, grain color are very like, satisfied!	Jingdong prefecture level city in Shandong
Fortune Shop	Green Intelligent Power Saver	I'll give you a good review. I don't know if it'll work.	Jingdong prefecture level city in Shandong
Fortune Shop	Green Intelligent Power Saver	The logistics is very fast and the package is intact. Good use of affordable. The effect is very good, is genuine	Jingdong prefecture level city in Shandong
Fortune Shop	Green Intelligent Power Saver	Nothing happens when you plug it in.	Jingdong prefecture level city in Shandong
Fortune Shop	Green Intelligent Power Saver	I don't know if it's as good as it sounds.	Jingdong prefecture level city in Shandong

Fortune Shop	Green Intelligent Power Saver	The quality of the stuff is very good, exactly the same as the seller's description, very satisfied!	Jingdong prefecture level city in Shandong
Fortune Shop	Green Intelligent Power Saver	The saver works great, it really works, it's affordable and worth buying.	Jingdong prefecture level city in Shandong
Fortune Shop	Green Intelligent Power Saver	Just used it for a couple days. I don't know yet.	Jingdong prefecture level city in Shandong
Fortune Shop	Green Intelligent Power Saver	The quality is quite good, cheaper than the physical store, good, next time will come again!	Jingdong prefecture level city in Shandong
Fortune Shop	Green Intelligent Power Saver	Good quality, cheap and affordable worth recommending to buy!	Jingdong prefecture level city in Shandong
Fortune Shop	Green Intelligent Power Saver	Very good quality, well made, works well! Satisfied	Jingdong prefecture level city in Shandong
Fortune Shop	Green Intelligent Power Saver	The logistics is very fast quality is very good very pleasant a shopping	Jingdong prefecture level city in Shandong
Fortune Shop	Green Intelligent Power Saver	Use it and then come back to evaluate!	Jingdong prefecture level city in Shandong
Fortune Shop	Green Intelligent Power Saver	# It's no use lying at all #	Jingdong prefecture level city in Shandong
Fortune Shop	Green Intelligent Power Saver	I can't get a voltage drop.	Jingdong prefecture level city in Shandong
Fortune Shop	Green Intelligent Power Saver	Evaluate after use	Jingdong prefecture level city in Shandong
Fortune Shop	Green Intelligent Power Saver	Still on trial.	Jingdong prefecture level city in Shandong
Fortune Shop	Green Intelligent Power Saver	ineffective	Jingdong prefecture level city in Shandong
Fortune Shop	Green Intelligent Power Saver	Satisfaction Shopping	Jingdong prefecture level city in Shandong
Fortune Shop	Green Intelligent Power Saver	Lies.	Jingdong prefecture level city in Shandong
Fortune Shop	Green Intelligent Power Saver	It's a real power saver.	Jingdong prefecture level city in Shandong
Fortune Shop	Green Intelligent Power Saver	in use	Jingdong prefecture level city in Shandong
Fortune Shop	Green Intelligent Power Saver	I don't know yet.	Jingdong prefecture level city in Shandong
Fortune Shop	Green Intelligent Power Saver	not care about anything	Jingdong prefecture level city in Shandong
Fortune Shop	Green Intelligent Power Saver	It doesn't seem to have much effect	Jingdong prefecture level city in Shandong
Fortune Shop	Green Intelligent Power Saver	Let the merchant see for himself, does the plug positive and negative terminals look like this?	Jingdong prefecture level city in Shandong
Fortune Shop	Green Intelligent Power Saver	It doesn't work well, the electricity bill is less before using it, but more after using it, contacting customer service, the service is still not good, and returning it is slow	Jingdong prefecture level city in Shandong
Fortune Shop	Green Intelligent Power Saver	With the advertisement product does not match, above the effective negative, power saving rate of two rows of words it, the product looks very thin, with 3 days to see the meter number is still with the previous no difference, which is obviously fraudulent behavior!	Jingdong prefecture level city in Shandong
Fortune Shop	Green Intelligent Power Saver	Doesn't save any power at all. Not much use.	Jingdong prefecture level city in Shandong
Fortune Shop	Green Intelligent Power Saver	The plugs are all loose, they're all the same length and they don't work at all	Jingdong prefecture level city in Shandong
Fortune Shop	Green Intelligent Power Saver	It's bad 👎 it's not effective at all, you'd be crazy to sell this garbage on the internet.	Jingdong prefecture level city in Shandong
Fortune Shop	Green Intelligent Power Saver	It doesn't save any power at all, so I hope the old timers don't fall for this scam again	Jingdong prefecture level city in Shandong
Fortune Shop	Green Intelligent Power Saver	I've been using it for over a month now and haven't noticed any power savings.	Jingdong prefecture level city in Shandong
Fortune Shop	Green Intelligent Power Saver	It doesn't work at all. The electricity bill is higher when you use it.	Jingdong prefecture level city in Shandong
Fortune Shop	Green Intelligent Power Saver	It doesn't save any power, it's still as bad as ever.	Jingdong prefecture level city in Shandong
Fortune Shop	Green Intelligent Power Saver	Last night before leaving work to look at the table, and then plugged into the node device, this morning and then look at the table, the original save a few degrees of electricity, very good to have a power-saving effect, easy to use, satisfied with the shopping praise.	Jingdong prefecture level city in Shandong
Fortune Shop	Green Intelligent Power Saver	The product is real and effective, the electricity bill is reduced, really good, thanks to the merchants have so many good products, the product is real and effective, the electricity bill is reduced, want to save electricity friends quickly buy it, early use early to save money.	Jingdong prefecture level city in Shandong
Fortune Shop	Green Intelligent Power Saver	Saver saves time and money, baby appearance is beautiful, the quality of goods is good, and the same as the business description, good service attitude, packaging is complete, logistics is very fast, praise to	Jingdong prefecture level city in Shandong
Fortune Shop	Green Intelligent Power Saver	The effect is good, summer, home electricity is relatively large, buy and use a few days, feel the effect is very good, originally ten dollars a day electricity bill, now eight dollars a day, quite good	Jingdong prefecture level city in Shandong
Fortune Shop	Green Intelligent Power Saver	Indeed can save electricity and money, the first time I know this or from a friend's home to see, but also do not believe it, and now they use it to know that there is this good thing.	Jingdong prefecture level city in Shandong
Fortune Shop	Green Intelligent Power Saver	I have received the Smart Power Saver. Perfectly packaged. Fast delivery. Boss customer service attitude is also very good. It's worth buying. I plugged it in and tried it and it really saves more power than the original. I'll buy it again next time.	Jingdong prefecture level city in Shandong
Fortune Shop	Green Intelligent Power Saver	It's nice to save a dozen bucks a night on two air conditioners that used to take 30 or 40 degrees a night, but now it's only 10 or so degrees a night	Jingdong prefecture level city in Shandong
Fortune Shop	Green Intelligent Power Saver	Product no manufacturer's name no product qualification certificate, try two days to see the effect of	Jingdong prefecture level city in Shandong
Fortune Shop	Green Intelligent Power Saver	I've been using it for over a month and have saved over $80, so I've repurchased a few more back to save together	Jingdong prefecture level city in Shandong
Fortune Shop	Green Intelligent Power Saver	I haven't used it yet and will comment when I do, but it's well made.	Jingdong prefecture level city in Shandong
Fortune Shop	Green Intelligent Power Saver	It's quite energy efficient. It's really a boon for the home.	Jingdong prefecture level city in Shandong
Fortune Shop	Green Intelligent Power Saver	Energy-saving socket received a month, after using it for a month proved that it is indeed energy-saving, electricity bill saving, will buy it again, thank you!	Jingdong prefecture level city in Shandong
Fortune Shop	Green Intelligent Power Saver	There was a similar product many years ago. Now technology has evolved, buy one to try, according to the instructions in the box, it takes one or two dozen days to show results. Check your electric bill and see what happens.	Jingdong prefecture level city in Shandong
Fortune Shop	Green Intelligent Power Saver	Jingdong logistics is really fast, the same day the order arrived on the same day, more convenient than the supermarket, and a wide range of choices, the	Jingdong prefecture level city in Shandong
Fortune Shop	Green Intelligent Power Saver	I've been using this for a month or so and I can't see any results, it really is a scam as they say.	Jingdong prefecture level city in Shandong
Fortune Shop	Green Intelligent Power Saver	Very good use, a month can save a few degrees of power loss of electricity, introduced to the relatives to buy back to try.	Jingdong prefecture level city in Shandong
Fortune Shop	Green Intelligent Power Saver	It's really powerful! With this air conditioner can be turned on at any time without the pressure of paying the electricity bill! It's good for the people!	Jingdong prefecture level city in Shandong
Fortune Shop	Green Intelligent Power Saver	This business is a big ** products are not qualified, with a month of electricity more than one hundred percent of thirty, the purchase agreed to return the goods but do not receive a refund after receiving the goods, looking for the official side does not help, the Jingdong is a big **, I have never shopped in	Jingdong prefecture level city in Shandong

Fortune Shop	Green Intelligent Power Saver	It's good. I tried it. It saves power.	Jingdong prefecture level city in Shandong
Fortune Shop	Green Intelligent Power Saver	Nothing**, it seems like there's more	Jingdong prefecture level city in Shandong
Fortune Shop	Green Intelligent Power Saver	Power saving device received, well packaged, good quality, fast logistics	Jingdong prefecture level city in Shandong
Fortune Shop	Green Intelligent Power Saver	The service is okay, is that when I bought five, the desirable pieces are three, and then wait a few days and send me two replacement, I do not know if it is a mistake of the shipper, if I was not in the courier there to see, go home to say no, the	Jingdong prefecture level city in Shandong
Fortune Shop	Green Intelligent Power Saver	This thing will only cost me more if I use it, but before I used it, it was less than a ~ electricity a month, and after I used it, it was more than two hundred kWh a month.	Jingdong prefecture level city in Shandong
Fortune Shop	Green Intelligent Power Saver	I just used it. I don't know how it is.	Jingdong prefecture level city in Shandong
Fortune Shop	Green Intelligent Power Saver	I've received the product and I'm using it, so I'll see how it goes in a month!	Jingdong prefecture level city in Shandong
Fortune Shop	Green Intelligent Power Saver	The goods have just received the effect do not know how, please look forward to the results.	Jingdong prefecture level city in Shandong
Fortune Shop	Green Intelligent Power Saver	Power saving device, the material is very good practical and durable, the size is suitable, packaging is very good, the price is cheap, logistics is very fast!	Jingdong prefecture level city in Shandong
Fortune Shop	Green Intelligent Power Saver	Very nice, will comment in detail after using it for a while.	Jingdong prefecture level city in Shandong
Fortune Shop	Green Intelligent Power Saver	Good stuff to trust and support	Jingdong prefecture level city in Shandong
Fortune Shop	Green Intelligent Power Saver	I'll comment after I use it, doubt if it really saves power no	Jingdong prefecture level city in Shandong
Fortune Shop	Green Intelligent Power Saver	It's good quality and works well!	Jingdong prefecture level city in Shandong
Fortune Shop	Green Intelligent Power Saver	hahaha just right hahahahahaha hiccup hahahaha	Jingdong prefecture level city in Shandong
Fortune Shop	Green Intelligent Power Saver	The real thing matches the picture and looks great!	Jingdong prefecture level city in Shandong
Fortune Shop	Green Intelligent Power Saver	Plugged it in and we'll see when we pay the bills at the end of the year!	Jingdong prefecture level city in Shandong
Fortune Shop	Green Intelligent Power Saver	False advertising. It doesn't work.	Jingdong prefecture level city in Shandong
Fortune Shop	Green Intelligent Power Saver	Goods received, good effect	Jingdong prefecture level city in Shandong
Fortune Shop	Green Intelligent Power Saver	It looks okay, we'll see if it saves electricity when the time comes	Jingdong prefecture level city in Shandong
Fortune Shop	Green Intelligent Power Saver	Logistics is very fast quality is very good, the price is very affordable, accessories are also complete, installed, satisfied with good use	Jingdong prefecture level city in Shandong
Fortune Shop	Green Intelligent Power Saver	I've received the power saver. I'm looking forward to the savings.	Jingdong prefecture level city in Shandong
Fortune Shop	Green Intelligent Power Saver	Received, have not yet used, after use and then evaluate	Jingdong prefecture level city in Shandong
Fortune Shop	Green Intelligent Power Saver	I received the item, but I don't know if it saves power or not.	Jingdong prefecture level city in Shandong
Fortune Shop	Green Intelligent Power Saver	On Trial	Jingdong prefecture level city in Shandong
Fortune Shop	Green Intelligent Power Saver	Use it and see how it goes.	Jingdong prefecture level city in Shandong
Fortune Shop	Green Intelligent Power Saver	Quality is very good, no odor, fine workmanship, grain color are very like, satisfied!	Jingdong prefecture level city in Shandong
Fortune Shop	Green Intelligent Power Saver	I'll give you a good review. I don't know if it'll work.	Jingdong prefecture level city in Shandong
Fortune Shop	Green Intelligent Power Saver	The logistics is very fast and the package is intact. Good use of affordable. The effect is very good, is genuine	Jingdong prefecture level city in Shandong
Fortune Shop	Green Intelligent Power Saver	Nothing happens when you plug it in.	Jingdong prefecture level city in Shandong
Fortune Shop	Green Intelligent Power Saver	I don't know if it's as good as it sounds.	Jingdong prefecture level city in Shandong
Fortune Shop	Green Intelligent Power Saver	The quality of the stuff is very good, exactly the same as the seller's description, very satisfied!	Jingdong prefecture level city in Shandong
Fortune Shop	Green Intelligent Power Saver	The saver works great, it really works, it's affordable and worth buying.	Jingdong prefecture level city in Shandong
Fortune Shop	Green Intelligent Power Saver	Just used it for a couple days. I don't know yet.	Jingdong prefecture level city in Shandong
Fortune Shop	Green Intelligent Power Saver	The quality is quite good, cheaper than the physical store, good, next time will come again!	Jingdong prefecture level city in Shandong
Fortune Shop	Green Intelligent Power Saver	Good quality, cheap and affordable worth recommending to buy!	Jingdong prefecture level city in Shandong
Fortune Shop	Green Intelligent Power Saver	Very good quality, well made, works well! Satisfied	Jingdong prefecture level city in Shandong
Fortune Shop	Green Intelligent Power Saver	The logistics is very fast quality is very good very pleasant a shopping	Jingdong prefecture level city in Shandong
Fortune Shop	Green Intelligent Power Saver	Use it and then come back to evaluate!	Jingdong prefecture level city in Shandong
Fortune Shop	Green Intelligent Power Saver	# It's no use lying at all #	Jingdong prefecture level city in Shandong
Fortune Shop	Green Intelligent Power Saver	I can't get a voltage drop.	Jingdong prefecture level city in Shandong
Fortune Shop	Green Intelligent Power Saver	Evaluate after use	Jingdong prefecture level city in Shandong
Fortune Shop	Green Intelligent Power Saver	Still on trial.	Jingdong prefecture level city in Shandong
Fortune Shop	Green Intelligent Power Saver	ineffective	Jingdong prefecture level city in Shandong
Fortune Shop	Green Intelligent Power Saver	Satisfaction Shopping	Jingdong prefecture level city in Shandong
Fortune Shop	Green Intelligent Power Saver	Lies.	Jingdong prefecture level city in Shandong
Fortune Shop	Green Intelligent Power Saver	It's a real power saver.	Jingdong prefecture level city in Shandong
Fortune Shop	Green Intelligent Power Saver	in use	Jingdong prefecture level city in Shandong
Fortune Shop	Green Intelligent Power Saver	I don't know yet.	Jingdong prefecture level city in Shandong
Fortune Shop	Green Intelligent Power Saver	not care about anything	Jingdong prefecture level city in Shandong
Fortune Shop	Green Intelligent Power Saver	It doesn't seem to have much effect	Jingdong prefecture level city in Shandong
Fortune Shop	Green Intelligent Power Saver	Let the merchant see for himself, does the plug positive and negative terminals look like this?	Jingdong prefecture level city in Shandong

Fortune Shop	Green Intelligent Power Saver	It doesn't work well, the electricity bill is less before using it, but more after using it, contacting customer service, the service is still not good, and returning it is slow	Jingdong prefecture level city in Shandong
Fortune Shop	Green Intelligent Power Saver	With the advertisement product does not match, above the effective negative, power saving rate of two rows of words it, the product looks very thin, with 3 days to see the meter number is still with the previous no difference, which is obviously fraudulent behavior!	Jingdong prefecture level city in Shandong
Fortune Shop	Green Intelligent Power Saver	Doesn't save any power at all. Not much use.	Jingdong prefecture level city in Shandong
Fortune Shop	Green Intelligent Power Saver	The plugs are all loose, they're all the same length and they don't work at all	Jingdong prefecture level city in Shandong
Fortune Shop	Green Intelligent Power Saver	It's bad 👎 it's not effective at all, you'd be crazy to sell this garbage on the internet.	Jingdong prefecture level city in Shandong
Fortune Shop	Green Intelligent Power Saver	It doesn't save any power at all, so I hope the old timers don't fall for this scam again	Jingdong prefecture level city in Shandong
Fortune Shop	Green Intelligent Power Saver	I've been using it for over a month now and haven't noticed any power savings.	Jingdong prefecture level city in Shandong
Fortune Shop	Green Intelligent Power Saver	It doesn't work at all. The electricity bill is higher when you use it.	Jingdong prefecture level city in Shandong
Fortune Shop	Green Intelligent Power Saver	It doesn't save any power, it's still as bad as ever.	Jingdong prefecture level city in Shandong
Fortune Shop	Green Intelligent Power Saver	Last night before leaving work to look at the table, and then plugged into the node device, this morning and then look at the table, than the original save a few degrees of electricity, very good to have a power-saving effect, easy to use, satisfied with the shopping praise.	Jingdong prefecture level city in Shandong
Fortune Shop	Green Intelligent Power Saver	The product is real and effective, the electricity bill is reduced, really good, thanks to the merchants have so many good products, the product is real and effective, the electricity bill is reduced, want to save electricity friends quickly buy it, early use early to save money.	Jingdong prefecture level city in Shandong
Fortune Shop	Green Intelligent Power Saver	Saver saves time and money, baby appearance is beautiful, the quality of goods is good, and the same as the business description, good service attitude, packaging is complete, logistics is very fast, praise to	Jingdong prefecture level city in Shandong
Fortune Shop	Green Intelligent Power Saver	The effect is good, summer, home electricity is relatively large, buy and use a few days, feel the effect is very good, originally ten dollars a day electricity bill, now eight dollars a day, quite good	Jingdong prefecture level city in Shandong
Fortune Shop	Green Intelligent Power Saver	Indeed can save electricity and money, the first time I know this or from a friend's home to see, but also do not believe it, and now they use it to know that there is this good thing.	Jingdong prefecture level city in Shandong
Fortune Shop	Green Intelligent Power Saver	I have received the Smart Power Saver. Perfectly packaged. Fast delivery. Boss customer service attitude is also very good. It's worth buying. I plugged it in and tried it and it really saves more power than the original. I'll buy it again next time.	Jingdong prefecture level city in Shandong
Fortune Shop	Green Intelligent Power Saver	It's nice to save a dozen bucks a night on two air conditioners that used to take 30 or 40 degrees a night, but now it's only 10 or so degrees a night	Jingdong prefecture level city in Shandong
Fortune Shop	Green Intelligent Power Saver	Product no manufacturer's name no product qualification certificate, try two days to see the effect of	Jingdong prefecture level city in Shandong
Fortune Shop	Green Intelligent Power Saver	I've been using it for over a month and have saved over $80, so I've repurchased a few more back to save together	Jingdong prefecture level city in Shandong
Fortune Shop	Green Intelligent Power Saver	I haven't used it yet and will comment when I do, but it's well made.	Jingdong prefecture level city in Shandong
Fortune Shop	Green Intelligent Power Saver	It's quite energy efficient. It's really a boon for the home.	Jingdong prefecture level city in Shandong
Fortune Shop	Green Intelligent Power Saver	Energy-saving socket received a month, after using it for a month proved that it is indeed energy-saving, electricity bill saving, will buy it again, thank you!	Jingdong prefecture level city in Shandong
Fortune Shop	Green Intelligent Power Saver	There was a similar product many years ago. Now technology has evolved, buy one to try, according to the instructions in the box, it takes one or two dozen days to show results. Check your electric bill and see what happens.	Jingdong prefecture level city in Shandong
Fortune Shop	Green Intelligent Power Saver	Jingdong logistics is really fast, the same day the order arrived on the same day, more convenient than the supermarket, and a wide range of choices, the	Jingdong prefecture level city in Shandong
Fortune Shop	Green Intelligent Power Saver	I've been using this for a month or so and I can't see any results, it really is a scam as they say.	Jingdong prefecture level city in Shandong
Fortune Shop	Green Intelligent Power Saver	Very good use, a month can save a few degrees of power loss of electricity, introduced to the relatives to buy back to try.	Jingdong prefecture level city in Shandong
Fortune Shop	Green Intelligent Power Saver	It's really powerful! With this air conditioner can be turned on at any time without the pressure of paying the electricity bill! It's good for the people!	Jingdong prefecture level city in Shandong
Fortune Shop	Green Intelligent Power Saver	This business is a big ** products are not qualified, with a month of electricity more than one hundred percent of thirty, the purchase agreed to return the goods but do not receive a refund after receiving the goods, looking for the official side does not help, the Jingdong is a big **, I have never shopped in	Jingdong prefecture level city in Shandong
Fortune Shop	Green Intelligent Power Saver	It's good. I tried it. It saves power.	Jingdong prefecture level city in Shandong
Fortune Shop	Green Intelligent Power Saver	Nothing**, it seems like there's more	Jingdong prefecture level city in Shandong
Fortune Shop	Green Intelligent Power Saver	Power saving device received, well packaged, good quality, fast logistics	Jingdong prefecture level city in Shandong
Fortune Shop	Green Intelligent Power Saver	The service is okay, is that when I bought five, the desirable pieces are three, and then wait a few days and send me two replacement, I do not know if it is a mistake of the shipper, if I was not in the courier there to see, go home to say no, the	Jingdong prefecture level city in Shandong
Fortune Shop	Green Intelligent Power Saver	This thing will only cost me more if I use it, but before I used it, it was less than a ~ electricity a month, and after I used it, it was more than two hundred kWh a month.	Jingdong prefecture level city in Shandong
Fortune Shop	Green Intelligent Power Saver	I just used it. I don't know how it is.	Jingdong prefecture level city in Shandong
Fortune Shop	Green Intelligent Power Saver	I've received the product and I'm using it, so I'll see how it goes in a month!	Jingdong prefecture level city in Shandong
Fortune Shop	Green Intelligent Power Saver	The goods have just received the effect do not know how, please look forward to the results.	Jingdong prefecture level city in Shandong
Fortune Shop	Green Intelligent Power Saver	Power saving device, the material is very good practical and durable, the size is suitable, packaging is very good, the price is cheap, logistics is very fast!	Jingdong prefecture level city in Shandong
Fortune Shop	Green Intelligent Power Saver	Very nice, will comment in detail after using it for a while.	Jingdong prefecture level city in Shandong
Fortune Shop	Green Intelligent Power Saver	Good stuff to trust and support	Jingdong prefecture level city in Shandong
Fortune Shop	Green Intelligent Power Saver	I'll comment after I use it, doubt if it really saves power no	Jingdong prefecture level city in Shandong
Fortune Shop	Green Intelligent Power Saver	It's good quality and works well!	Jingdong prefecture level city in Shandong
Fortune Shop	Green Intelligent Power Saver	hahaha just right hahahahahaha hiccup hahahaha	Jingdong prefecture level city in Shandong
Fortune Shop	Green Intelligent Power Saver	The real thing matches the picture and looks great!	Jingdong prefecture level city in Shandong
Fortune Shop	Green Intelligent Power Saver	Plugged it in and we'll see when we pay the bills at the end of the year!	Jingdong prefecture level city in Shandong
Fortune Shop	Green Intelligent Power Saver	False advertising. It doesn't work.	Jingdong prefecture level city in Shandong
Fortune Shop	Green Intelligent Power Saver	Goods received, good effect	Jingdong prefecture level city in Shandong
Fortune Shop	Green Intelligent Power Saver	It looks okay, we'll see if it saves electricity when the time comes	Jingdong prefecture level city in Shandong
Fortune Shop	Green Intelligent Power Saver	Logistics is very fast quality is very good, the price is very affordable, accessories are also complete, installed, satisfied with good use	Jingdong prefecture level city in Shandong
Fortune Shop	Green Intelligent Power Saver	I've received the power saver. I'm looking forward to the savings.	Jingdong prefecture level city in Shandong

Fortune Shop	Green Intelligent Power Saver	Received, have not yet used, after use and then evaluate	Jingdong prefecture level city in Shandong
Fortune Shop	Green Intelligent Power Saver	I received the item, but I don't know if it saves power or not.	Jingdong prefecture level city in Shandong
Fortune Shop	Green Intelligent Power Saver	On Trial	Jingdong prefecture level city in Shandong
Fortune Shop	Green Intelligent Power Saver	Use it and see how it goes.	Jingdong prefecture level city in Shandong
Fortune Shop	Green Intelligent Power Saver	Quality is very good, no odor, fine workmanship, grain color are very like, satisfied!	Jingdong prefecture level city in Shandong
Fortune Shop	Green Intelligent Power Saver	I'll give you a good review. I don't know if it'll work.	Jingdong prefecture level city in Shandong
Fortune Shop	Green Intelligent Power Saver	The logistics is very fast and the package is intact. Good use of affordable. The effect is very good, is genuine	Jingdong prefecture level city in Shandong
Fortune Shop	Green Intelligent Power Saver	Nothing happens when you plug it in.	Jingdong prefecture level city in Shandong
Fortune Shop	Green Intelligent Power Saver	I don't know if it's as good as it sounds.	Jingdong prefecture level city in Shandong
Fortune Shop	Green Intelligent Power Saver	The quality of the stuff is very good, exactly the same as the seller's description, very satisfied!	Jingdong prefecture level city in Shandong
Fortune Shop	Green Intelligent Power Saver	The saver works great, it really works, it's affordable and worth buying.	Jingdong prefecture level city in Shandong
Fortune Shop	Green Intelligent Power Saver	Just used it for a couple days. I don't know yet.	Jingdong prefecture level city in Shandong
Fortune Shop	Green Intelligent Power Saver	The quality is quite good, cheaper than the physical store, good, next time will come again!	Jingdong prefecture level city in Shandong
Fortune Shop	Green Intelligent Power Saver	Good quality, cheap and affordable worth recommending to buy!	Jingdong prefecture level city in Shandong
Fortune Shop	Green Intelligent Power Saver	Very good quality, well made, works well! Satisfied	Jingdong prefecture level city in Shandong
Fortune Shop	Green Intelligent Power Saver	The logistics is very fast quality is very good very pleasant a shopping	Jingdong prefecture level city in Shandong
Fortune Shop	Green Intelligent Power Saver	Use it and then come back to evaluate!	Jingdong prefecture level city in Shandong
Fortune Shop	Green Intelligent Power Saver	# It's no use lying at all #	Jingdong prefecture level city in Shandong
Fortune Shop	Green Intelligent Power Saver	I can't get a voltage drop.	Jingdong prefecture level city in Shandong
Fortune Shop	Green Intelligent Power Saver	Evaluate after use	Jingdong prefecture level city in Shandong
Fortune Shop	Green Intelligent Power Saver	Still on trial.	Jingdong prefecture level city in Shandong
Fortune Shop	Green Intelligent Power Saver	ineffective	Jingdong prefecture level city in Shandong
Fortune Shop	Green Intelligent Power Saver	Satisfaction Shopping	Jingdong prefecture level city in Shandong
Fortune Shop	Green Intelligent Power Saver	Lies.	Jingdong prefecture level city in Shandong
Fortune Shop	Green Intelligent Power Saver	It's a real power saver.	Jingdong prefecture level city in Shandong
Fortune Shop	Green Intelligent Power Saver	in use	Jingdong prefecture level city in Shandong
Fortune Shop	Green Intelligent Power Saver	I don't know yet.	Jingdong prefecture level city in Shandong
Fortune Shop	Green Intelligent Power Saver	not care about anything	Jingdong prefecture level city in Shandong
Fortune Shop	Green Intelligent Power Saver	It doesn't seem to have much effect	Jingdong prefecture level city in Shandong
Fortune Shop	Green Intelligent Power Saver	Let the merchant see for himself, does the plug positive and negative terminals look like this?	Jingdong prefecture level city in Shandong
Fortune Shop	Green Intelligent Power Saver	It doesn't work well, the electricity bill is less before using it, but more after using it, contacting customer service, the service is still not good, and returning it is slow	Jingdong prefecture level city in Shandong
Fortune Shop	Green Intelligent Power Saver	With the advertisement product does not match, above the effective negative, power saving rate of two rows of words it, the product looks very thin, with 3 days to see the meter number is still with the previous no difference, which is obviously fraudulent behavior!	Jingdong prefecture level city in Shandong
Fortune Shop	Green Intelligent Power Saver	Doesn't save any power at all. Not much use.	Jingdong prefecture level city in Shandong
Fortune Shop	Green Intelligent Power Saver	The plugs are all loose, they're all the same length and they don't work at all	Jingdong prefecture level city in Shandong
Fortune Shop	Green Intelligent Power Saver	It's bad 👎 it's not effective at all, you'd be crazy to sell this garbage on the internet.	Jingdong prefecture level city in Shandong
Fortune Shop	Green Intelligent Power Saver	It doesn't save any power at all, so I hope the old timers don't fall for this scam again	Jingdong prefecture level city in Shandong
Fortune Shop	Green Intelligent Power Saver	I've been using it for over a month now and haven't noticed any power savings.	Jingdong prefecture level city in Shandong
Fortune Shop	Green Intelligent Power Saver	It doesn't work at all. The electricity bill is higher when you use it.	Jingdong prefecture level city in Shandong
Fortune Shop	Green Intelligent Power Saver	It doesn't save any power, it's still as bad as ever.	Jingdong prefecture level city in Shandong
Fortune Shop	Green Intelligent Power Saver	Last night before leaving work to look at the table, and then plugged into the node device, this morning and then look at the table, than the original save a few degrees of electricity, very good to have a power-saving effect, easy to use, satisfied with the shopping praise.	Jingdong prefecture level city in Shandong
Fortune Shop	Green Intelligent Power Saver	The product is real and effective, the electricity bill is reduced, really good, thanks to the merchants have so many good products, the product is real and effective, the electricity bill is reduced, want to save electricity friends quickly buy it, early use early to save money.	Jingdong prefecture level city in Shandong
Fortune Shop	Green Intelligent Power Saver	Saver saves time and money, baby appearance is beautiful, the quality of goods is good, and the same as the business description, good service attitude, packaging is complete, logistics is very fast, praise to	Jingdong prefecture level city in Shandong
Fortune Shop	Green Intelligent Power Saver	The effect is good, summer, home electricity is relatively large, buy and use a few days, feel the effect is very good, originally ten dollars a day electricity bill, now eight dollars a day, quite good	Jingdong prefecture level city in Shandong
Fortune Shop	Green Intelligent Power Saver	Indeed can save electricity and money, the first time I know this or from a friend's home to see, but also do not believe it, and now they use it to know that there is this good thing.	Jingdong prefecture level city in Shandong
Fortune Shop	Green Intelligent Power Saver	I have received the Smart Power Saver. Perfectly packaged. Fast delivery. Boss customer service attitude is also very good. It's worth buying. I plugged it in and tried it and it really saves more power than the original. I'll buy it again next time.	Jingdong prefecture level city in Shandong
Fortune Shop	Green Intelligent Power Saver	It's nice to save a dozen bucks a night on two air conditioners that used to take 30 or 40 degrees a night, but now it's only 10 or so degrees a night	Jingdong prefecture level city in Shandong
Fortune Shop	Green Intelligent Power Saver	Product no manufacturer's name no product qualification certificate, try two days to see the effect of	Jingdong prefecture level city in Shandong
Fortune Shop	Green Intelligent Power Saver	I've been using it for over a month and have saved over $80, so I've repurchased a few more back to save together	Jingdong prefecture level city in Shandong
Fortune Shop	Green Intelligent Power Saver	I haven't used it yet and will comment when I do, but it's well made.	Jingdong prefecture level city in Shandong

Fortune Shop	Green Intelligent Power Saver	It's quite energy efficient. It's really a boon for the home.	Jingdong prefecture level city in Shandong
Fortune Shop	Green Intelligent Power Saver	Energy-saving socket received a month, after using it for a month proved that it is indeed energy-saving, electricity bill saving, will buy it again, thank you!	Jingdong prefecture level city in Shandong
Fortune Shop	Green Intelligent Power Saver	There was a similar product many years ago. Now technology has evolved, buy one to try, according to the instructions in the box, it takes one or two dozen days to show results. Check your electric bill and see what happens.	Jingdong prefecture level city in Shandong
Fortune Shop	Green Intelligent Power Saver	Jingdong logistics is really fast, the same day the order arrived on the same day, more convenient than the supermarket, and a wide range of choices, the	Jingdong prefecture level city in Shandong
Fortune Shop	Green Intelligent Power Saver	I've been using this for a month or so and I can't see any results, it really is a scam as they say.	Jingdong prefecture level city in Shandong
Fortune Shop	Green Intelligent Power Saver	Very good use, a month can save a few degrees of power loss of electricity, introduced to the relatives to buy back to try.	Jingdong prefecture level city in Shandong
Fortune Shop	Green Intelligent Power Saver	It's really powerful! With this air conditioner can be turned on at any time without the pressure of paying the electricity bill! It's good for the people!	Jingdong prefecture level city in Shandong
Fortune Shop	Green Intelligent Power Saver	This business is a big ** products are not qualified, with a month of electricity more than one hundred percent of thirty, the purchase agreed to return the goods but do not receive a refund after receiving the goods, looking for the official side does not help, the Jingdong is a big **, I have never shopped in	Jingdong prefecture level city in Shandong
Fortune Shop	Green Intelligent Power Saver	It's good. I tried it. It saves power.	Jingdong prefecture level city in Shandong
Fortune Shop	Green Intelligent Power Saver	Nothing**, it seems like there's more	Jingdong prefecture level city in Shandong
Fortune Shop	Green Intelligent Power Saver	Power saving device received, well packaged, good quality, fast logistics	Jingdong prefecture level city in Shandong
Fortune Shop	Green Intelligent Power Saver	The service is okay, is that when I bought five, the desirable pieces are three, and then wait a few days and send me two replacement, I do not know if it is a mistake of the shipper, if I was not in the courier there to see, go home to say no, the	Jingdong prefecture level city in Shandong
Fortune Shop	Green Intelligent Power Saver	This thing will only cost me more if I use it, but before I used it, it was less than a ~ electricity a month, and after I used it, it was more than two hundred kWh a month.	Jingdong prefecture level city in Shandong
Fortune Shop	Green Intelligent Power Saver	I just used it. I don't know how it is.	Jingdong prefecture level city in Shandong
Fortune Shop	Green Intelligent Power Saver	I've received the product and I'm using it, so I'll see how it goes in a month!	Jingdong prefecture level city in Shandong
Fortune Shop	Green Intelligent Power Saver	The goods have just received the effect do not know how, please look forward to the results.	Jingdong prefecture level city in Shandong
Fortune Shop	Green Intelligent Power Saver	Power saving device, the material is very good practical and durable, the size is suitable, packaging is very good, the price is cheap, logistics is very fast!	Jingdong prefecture level city in Shandong
Fortune Shop	Green Intelligent Power Saver	Very nice, will comment in detail after using it for a while.	Jingdong prefecture level city in Shandong
Fortune Shop	Green Intelligent Power Saver	Good stuff to trust and support	Jingdong prefecture level city in Shandong
Fortune Shop	Green Intelligent Power Saver	I'll comment after I use it, doubt if it really saves power no	Jingdong prefecture level city in Shandong
Fortune Shop	Green Intelligent Power Saver	It's good quality and works well!	Jingdong prefecture level city in Shandong
Fortune Shop	Green Intelligent Power Saver	hahaha just right hahahahahaha hiccup hahahaha	Jingdong prefecture level city in Shandong
Fortune Shop	Green Intelligent Power Saver	The real thing matches the picture and looks great!	Jingdong prefecture level city in Shandong
Fortune Shop	Green Intelligent Power Saver	Plugged it in and we'll see when we pay the bills at the end of the year!	Jingdong prefecture level city in Shandong
Fortune Shop	Green Intelligent Power Saver	False advertising. It doesn't work.	Jingdong prefecture level city in Shandong
Fortune Shop	Green Intelligent Power Saver	Goods received, good effect	Jingdong prefecture level city in Shandong
Fortune Shop	Green Intelligent Power Saver	It looks okay, we'll see if it saves electricity when the time comes	Jingdong prefecture level city in Shandong
Fortune Shop	Green Intelligent Power Saver	Logistics is very fast quality is very good, the price is very affordable, accessories are also complete, installed, satisfied with good use	Jingdong prefecture level city in Shandong
Fortune Shop	Green Intelligent Power Saver	I've received the power saver. I'm looking forward to the savings.	Jingdong prefecture level city in Shandong
Fortune Shop	Green Intelligent Power Saver	Received, have not yet used, after use and then evaluate	Jingdong prefecture level city in Shandong
Fortune Shop	Green Intelligent Power Saver	I received the item, but I don't know if it saves power or not.	Jingdong prefecture level city in Shandong
Fortune Shop	Green Intelligent Power Saver	On Trial	Jingdong prefecture level city in Shandong
Fortune Shop	Green Intelligent Power Saver	Use it and see how it goes.	Jingdong prefecture level city in Shandong
Fortune Shop	Green Intelligent Power Saver	Quality is very good, no odor, fine workmanship, grain color are very like, satisfied!	Jingdong prefecture level city in Shandong
Fortune Shop	Green Intelligent Power Saver	I'll give you a good review. I don't know if it'll work.	Jingdong prefecture level city in Shandong
Fortune Shop	Green Intelligent Power Saver	The logistics is very fast and the package is intact. Good use of affordable. The effect is very good, is genuine	Jingdong prefecture level city in Shandong
Fortune Shop	Green Intelligent Power Saver	Nothing happens when you plug it in.	Jingdong prefecture level city in Shandong
Fortune Shop	Green Intelligent Power Saver	I don't know if it's as good as it sounds.	Jingdong prefecture level city in Shandong
Fortune Shop	Green Intelligent Power Saver	The quality of the stuff is very good, exactly the same as the seller's description, very satisfied!	Jingdong prefecture level city in Shandong
Fortune Shop	Green Intelligent Power Saver	The saver works great, it really works, it's affordable and worth buying.	Jingdong prefecture level city in Shandong
Fortune Shop	Green Intelligent Power Saver	Just used it for a couple days. I don't know yet.	Jingdong prefecture level city in Shandong
Fortune Shop	Green Intelligent Power Saver	The quality is quite good, cheaper than the physical store, good, next time will come again!	Jingdong prefecture level city in Shandong
Fortune Shop	Green Intelligent Power Saver	Good quality, cheap and affordable worth recommending to buy!	Jingdong prefecture level city in Shandong
Fortune Shop	Green Intelligent Power Saver	Very good quality, well made, works well! Satisfied	Jingdong prefecture level city in Shandong
Fortune Shop	Green Intelligent Power Saver	The logistics is very fast quality is very good very pleasant a shopping	Jingdong prefecture level city in Shandong
Fortune Shop	Green Intelligent Power Saver	Use it and then come back to evaluate!	Jingdong prefecture level city in Shandong
Fortune Shop	Green Intelligent Power Saver	# It's no use lying at all #	Jingdong prefecture level city in Shandong
Fortune Shop	Green Intelligent Power Saver	I can't get a voltage drop.	Jingdong prefecture level city in Shandong
Fortune Shop	Green Intelligent Power Saver	Evaluate after use	Jingdong prefecture level city in Shandong
Fortune Shop	Green Intelligent Power Saver	Still on trial.	Jingdong prefecture level city in Shandong

Fortune Shop	Green Intelligent Power Saver	ineffective	Jingdong prefecture level city in Shandong
Fortune Shop	Green Intelligent Power Saver	Satisfaction Shopping	Jingdong prefecture level city in Shandong
Fortune Shop	Green Intelligent Power Saver	Lies.	Jingdong prefecture level city in Shandong
Fortune Shop	Green Intelligent Power Saver	It's a real power saver.	Jingdong prefecture level city in Shandong
Fortune Shop	Green Intelligent Power Saver	in use	Jingdong prefecture level city in Shandong
Fortune Shop	Green Intelligent Power Saver	I don't know yet.	Jingdong prefecture level city in Shandong
Fortune Shop	Green Intelligent Power Saver	not care about anything	Jingdong prefecture level city in Shandong
Fortune Shop	Green Intelligent Power Saver	It doesn't seem to have much effect	Jingdong prefecture level city in Shandong
Fortune Shop	Green Intelligent Power Saver	Let the merchant see for himself, does the plug positive and negative terminals look like this?	Jingdong prefecture level city in Shandong
Fortune Shop	Green Intelligent Power Saver	It doesn't work well, the electricity bill is less before using it, but more after using it, contacting customer service, the service is still not good, and returning it is slow	Jingdong prefecture level city in Shandong
Fortune Shop	Green Intelligent Power Saver	With the advertisement product does not match, above the effective negative, power saving rate of two rows of words it, the product looks very thin, with 3 days to see the meter number is still with the previous no difference, which is obviously fraudulent behavior!	Jingdong prefecture level city in Shandong
Fortune Shop	Green Intelligent Power Saver	Doesn't save any power at all. Not much use.	Jingdong prefecture level city in Shandong
Fortune Shop	Green Intelligent Power Saver	The plugs are all loose, they're all the same length and they don't work at all	Jingdong prefecture level city in Shandong
Fortune Shop	Green Intelligent Power Saver	It's bad 👎 it's not effective at all, you'd be crazy to sell this garbage on the internet.	Jingdong prefecture level city in Shandong
Fortune Shop	Green Intelligent Power Saver	It doesn't save any power at all, so I hope the old timers don't fall for this scam again	Jingdong prefecture level city in Shandong
Fortune Shop	Green Intelligent Power Saver	I've been using it for over a month now and haven't noticed any power savings.	Jingdong prefecture level city in Shandong
Fortune Shop	Green Intelligent Power Saver	It doesn't work at all. The electricity bill is higher when you use it.	Jingdong prefecture level city in Shandong
Fortune Shop	Green Intelligent Power Saver	It doesn't save any power, it's still as bad as ever.	Jingdong prefecture level city in Shandong
Fortune Shop	Green Intelligent Power Saver	Last night before leaving work to look at the table, and then plugged into the node device, this morning and then look at the table, than the original save a few degrees of electricity, very good to have a power-saving effect, easy to use, satisfied with the shopping praise.	Jingdong prefecture level city in Shandong
Fortune Shop	Green Intelligent Power Saver	The product is real and effective, the electricity bill is reduced, really good, thanks to the merchants have so many good products, the product is real and effective, the electricity bill is reduced, want to save electricity friends quickly buy it, early use early to save money.	Jingdong prefecture level city in Shandong
Fortune Shop	Green Intelligent Power Saver	Saver saves time and money, baby appearance is beautiful, the quality of goods is good, and the same as the business description, good service attitude, packaging is complete, logistics is very fast, praise to	Jingdong prefecture level city in Shandong
Fortune Shop	Green Intelligent Power Saver	The effect is good, summer, home electricity is relatively large, buy and use a few days, feel the effect is very good, originally ten dollars a day electricity bill, now eight dollars a day, quite good	Jingdong prefecture level city in Shandong
Fortune Shop	Green Intelligent Power Saver	Indeed can save electricity and money, the first time I know this or from a friend's home to see, but also do not believe it, and now they use it to know that there is this good thing.	Jingdong prefecture level city in Shandong
Fortune Shop	Green Intelligent Power Saver	I have received the Smart Power Saver. Perfectly packaged. Fast delivery. Boss customer service attitude is also very good. It's worth buying. I plugged it in and tried it and it really saves more power than the original. I'll buy it again next time.	Jingdong prefecture level city in Shandong
Fortune Shop	Green Intelligent Power Saver	It's nice to save a dozen bucks a night on two air conditioners that used to take 30 or 40 degrees a night, but now it's only 10 or so degrees a night	Jingdong prefecture level city in Shandong
Fortune Shop	Green Intelligent Power Saver	Product no manufacturer's name no product qualification certificate, try two days to see the effect of	Jingdong prefecture level city in Shandong
Fortune Shop	Green Intelligent Power Saver	I've been using it for over a month and have saved over $80, so I've repurchased a few more back to save together	Jingdong prefecture level city in Shandong
Fortune Shop	Green Intelligent Power Saver	I haven't used it yet and will comment when I do, but it's well made.	Jingdong prefecture level city in Shandong
Fortune Shop	Green Intelligent Power Saver	It's quite energy efficient. It's really a boon for the home.	Jingdong prefecture level city in Shandong
Fortune Shop	Green Intelligent Power Saver	Energy-saving socket received a month, after using it for a month proved that it is indeed energy-saving, electricity bill saving, will buy it again, thank you!	Jingdong prefecture level city in Shandong
Fortune Shop	Green Intelligent Power Saver	There was a similar product many years ago. Now technology has evolved, buy one to try, according to the instructions in the box, it takes one or two dozen days to show results. Check your electric bill and see what happens.	Jingdong prefecture level city in Shandong
Fortune Shop	Green Intelligent Power Saver	Jingdong logistics is really fast, the same day the order arrived on the same day, more convenient than the supermarket, and a wide range of choices, the	Jingdong prefecture level city in Shandong
Fortune Shop	Green Intelligent Power Saver	I've been using this for a month or so and I can't see any results, it really is a scam as they say.	Jingdong prefecture level city in Shandong
Fortune Shop	Green Intelligent Power Saver	Very good use, a month can save a few degrees of power loss of electricity, introduced to the relatives to buy back to try.	Jingdong prefecture level city in Shandong
Fortune Shop	Green Intelligent Power Saver	It's really powerful! With this air conditioner can be turned on at any time without the pressure of paying the electricity bill! It's good for the people!	Jingdong prefecture level city in Shandong
Fortune Shop	Green Intelligent Power Saver	This business is a big ** products are not qualified, with a month of electricity more than one hundred percent of thirty, the purchase agreed to return the goods but do not receive a refund after receiving the goods, looking for the official side does not help, the Jingdong is a big **, I have never shopped in	Jingdong prefecture level city in Shandong
Fortune Shop	Green Intelligent Power Saver	It's good. I tried it. It saves power.	Jingdong prefecture level city in Shandong
Fortune Shop	Green Intelligent Power Saver	Nothing**, it seems like there's more	Jingdong prefecture level city in Shandong
Fortune Shop	Green Intelligent Power Saver	Power saving device received, well packaged, good quality, fast logistics	Jingdong prefecture level city in Shandong
Fortune Shop	Green Intelligent Power Saver	The service is okay, is that when I bought five, the desirable pieces are three, and then wait a few days and send me two replacement, I do not know if it is a mistake of the shipper, if I was not in the courier there to see, go home to say no, the	Jingdong prefecture level city in Shandong
Fortune Shop	Green Intelligent Power Saver	This thing will only cost me more if I use it, but before I used it, it was less than a ~ electricity a month, and after I used it, it was more than two hundred kWh a month.	Jingdong prefecture level city in Shandong
Fortune Shop	Green Intelligent Power Saver	I just used it. I don't know how it is.	Jingdong prefecture level city in Shandong
Fortune Shop	Green Intelligent Power Saver	I've received the product and I'm using it, so I'll see how it goes in a month!	Jingdong prefecture level city in Shandong
Fortune Shop	Green Intelligent Power Saver	The goods have just received the effect do not know how, please look forward to the results.	Jingdong prefecture level city in Shandong
Fortune Shop	Green Intelligent Power Saver	Power saving device, the material is very good practical and durable, the size is suitable, packaging is very good, the price is cheap, logistics is very fast!	Jingdong prefecture level city in Shandong
Fortune Shop	Green Intelligent Power Saver	Very nice, will comment in detail after using it for a while.	Jingdong prefecture level city in Shandong
Fortune Shop	Green Intelligent Power Saver	Good stuff to trust and support	Jingdong prefecture level city in Shandong
Fortune Shop	Green Intelligent Power Saver	I'll comment after I use it, doubt if it really saves power no	Jingdong prefecture level city in Shandong

Fortune Shop	Green Intelligent Power Saver	It's good quality and works well!	Jingdong prefecture level city in Shandong
Fortune Shop	Green Intelligent Power Saver	hahaha just right hahahahahaha hiccup hahahaha	Jingdong prefecture level city in Shandong
Fortune Shop	Green Intelligent Power Saver	The real thing matches the picture and looks great!	Jingdong prefecture level city in Shandong
Fortune Shop	Green Intelligent Power Saver	Plugged it in and we'll see when we pay the bills at the end of the year!	Jingdong prefecture level city in Shandong
Fortune Shop	Green Intelligent Power Saver	False advertising. It doesn't work.	Jingdong prefecture level city in Shandong
Fortune Shop	Green Intelligent Power Saver	Goods received, good effect	Jingdong prefecture level city in Shandong
Fortune Shop	Green Intelligent Power Saver	It looks okay, we'll see if it saves electricity when the time comes	Jingdong prefecture level city in Shandong
Fortune Shop	Green Intelligent Power Saver	Logistics is very fast quality is very good, the price is very affordable, accessories are also complete, installed, satisfied with good use	Jingdong prefecture level city in Shandong
Fortune Shop	Green Intelligent Power Saver	I've received the power saver. I'm looking forward to the savings.	Jingdong prefecture level city in Shandong
Fortune Shop	Green Intelligent Power Saver	Received, have not yet used, after use and then evaluate	Jingdong prefecture level city in Shandong
Fortune Shop	Green Intelligent Power Saver	I received the item, but I don't know if it saves power or not.	Jingdong prefecture level city in Shandong
Fortune Shop	Green Intelligent Power Saver	On Trial	Jingdong prefecture level city in Shandong
Fortune Shop	Green Intelligent Power Saver	Use it and see how it goes.	Jingdong prefecture level city in Shandong
Fortune Shop	Green Intelligent Power Saver	Quality is very good, no odor, fine workmanship, grain color are very like, satisfied!	Jingdong prefecture level city in Shandong
Fortune Shop	Green Intelligent Power Saver	I'll give you a good review. I don't know if it'll work.	Jingdong prefecture level city in Shandong
Fortune Shop	Green Intelligent Power Saver	The logistics is very fast and the package is intact. Good use of affordable. The effect is very good, is genuine	Jingdong prefecture level city in Shandong
Fortune Shop	Green Intelligent Power Saver	Nothing happens when you plug it in.	Jingdong prefecture level city in Shandong
Fortune Shop	Green Intelligent Power Saver	I don't know if it's as good as it sounds.	Jingdong prefecture level city in Shandong
Fortune Shop	Green Intelligent Power Saver	The quality of the stuff is very good, exactly the same as the seller's description, very satisfied!	Jingdong prefecture level city in Shandong
Fortune Shop	Green Intelligent Power Saver	The saver works great, it really works, it's affordable and worth buying.	Jingdong prefecture level city in Shandong
Fortune Shop	Green Intelligent Power Saver	Just used it for a couple days. I don't know yet.	Jingdong prefecture level city in Shandong
Fortune Shop	Green Intelligent Power Saver	The quality is quite good, cheaper than the physical store, good, next time will come again!	Jingdong prefecture level city in Shandong
Fortune Shop	Green Intelligent Power Saver	Good quality, cheap and affordable worth recommending to buy!	Jingdong prefecture level city in Shandong
Fortune Shop	Green Intelligent Power Saver	Very good quality, well made, works well! Satisfied	Jingdong prefecture level city in Shandong
Fortune Shop	Green Intelligent Power Saver	The logistics is very fast quality is very good very pleasant a shopping	Jingdong prefecture level city in Shandong
Fortune Shop	Green Intelligent Power Saver	Use it and then come back to evaluate!	Jingdong prefecture level city in Shandong
Fortune Shop	Green Intelligent Power Saver	# It's no use lying at all #	Jingdong prefecture level city in Shandong
Fortune Shop	Green Intelligent Power Saver	I can't get a voltage drop.	Jingdong prefecture level city in Shandong
Fortune Shop	Green Intelligent Power Saver	Evaluate after use	Jingdong prefecture level city in Shandong
Fortune Shop	Green Intelligent Power Saver	Still on trial.	Jingdong prefecture level city in Shandong
Fortune Shop	Green Intelligent Power Saver	ineffective	Jingdong prefecture level city in Shandong
Fortune Shop	Green Intelligent Power Saver	Satisfaction Shopping	Jingdong prefecture level city in Shandong
Fortune Shop	Green Intelligent Power Saver	Lies.	Jingdong prefecture level city in Shandong
Fortune Shop	Green Intelligent Power Saver	It's a real power saver.	Jingdong prefecture level city in Shandong
Fortune Shop	Green Intelligent Power Saver	in use	Jingdong prefecture level city in Shandong
Fortune Shop	Green Intelligent Power Saver	I don't know yet.	Jingdong prefecture level city in Shandong
Fortune Shop	Green Intelligent Power Saver	not care about anything	Jingdong prefecture level city in Shandong
Fortune Shop	Green Intelligent Power Saver	It doesn't seem to have much effect	Jingdong prefecture level city in Shandong
Fortune Shop	Green Intelligent Power Saver	Let the merchant see for himself, does the plug positive and negative terminals look like this?	Jingdong prefecture level city in Shandong
Fortune Shop	Green Intelligent Power Saver	It doesn't work well, the electricity bill is less before using it, but more after using it, contacting customer service, the service is still not good, and returning it is slow	Jingdong prefecture level city in Shandong
Fortune Shop	Green Intelligent Power Saver	With the advertisement product does not match, above the effective negative, power saving rate of two rows of words it, the product looks very thin, with 3 days to see the meter number is still with the previous no difference, which is obviously fraudulent behavior!	Jingdong prefecture level city in Shandong
Fortune Shop	Green Intelligent Power Saver	Doesn't save any power at all. Not much use.	Jingdong prefecture level city in Shandong
Fortune Shop	Green Intelligent Power Saver	The plugs are all loose, they're all the same length and they don't work at all	Jingdong prefecture level city in Shandong
Fortune Shop	Green Intelligent Power Saver	It's bad 👎 it's not effective at all, you'd be crazy to sell this garbage on the internet.	Jingdong prefecture level city in Shandong
Fortune Shop	Green Intelligent Power Saver	It doesn't save any power at all, so I hope the old timers don't fall for this scam again	Jingdong prefecture level city in Shandong
Fortune Shop	Green Intelligent Power Saver	I've been using it for over a month now and haven't noticed any power savings.	Jingdong prefecture level city in Shandong
Fortune Shop	Green Intelligent Power Saver	It doesn't work at all. The electricity bill is higher when you use it.	Jingdong prefecture level city in Shandong
Fortune Shop	Green Intelligent Power Saver	It doesn't save any power, it's still as bad as ever.	Jingdong prefecture level city in Shandong
Fortune Shop	Green Intelligent Power Saver	Last night before leaving work to look at the table, and then plugged into the node device, this morning and then look at the table, than the original save a few degrees of electricity, very good to have a power-saving effect, easy to use, satisfied with the shopping praise.	Jingdong prefecture level city in Shandong
Fortune Shop	Green Intelligent Power Saver	The product is real and effective, the electricity bill is reduced, really good, thanks to the merchants have so many good products, the product is real and effective, the electricity bill is reduced, want to save electricity friends quickly buy it, early use early to save money.	Jingdong prefecture level city in Shandong

Fortune Shop	Green Intelligent Power Saver	Saver saves time and money, baby appearance is beautiful, the quality of goods is good, and the same as the business description, good service attitude, packaging is complete, logistics is very fast, praise to	Jingdong prefecture level city in Shandong
Fortune Shop	Green Intelligent Power Saver	The effect is good, summer, home electricity is relatively large, buy and use a few days, feel the effect is very good, originally ten dollars a day electricity bill, now eight dollars a day, quite good	Jingdong prefecture level city in Shandong
Fortune Shop	Green Intelligent Power Saver	Indeed can save electricity and money, the first time I know this or from a friend's home to see, but also do not believe it, and now they use it to know that there is this good thing.	Jingdong prefecture level city in Shandong
Fortune Shop	Green Intelligent Power Saver	I have received the Smart Power Saver. Perfectly packaged. Fast delivery. Boss customer service attitude is also very good. It's worth buying. I plugged it in and tried it and it really saves more power than the original. I'll buy it again next time.	Jingdong prefecture level city in Shandong
Fortune Shop	Green Intelligent Power Saver	It's nice to save a dozen bucks a night on two air conditioners that used to take 30 or 40 degrees a night, but now it's only 10 or so degrees a night	Jingdong prefecture level city in Shandong
Fortune Shop	Green Intelligent Power Saver	Product no manufacturer's name no product qualification certificate, try two days to see the effect of	Jingdong prefecture level city in Shandong
Fortune Shop	Green Intelligent Power Saver	I've been using it for over a month and have saved over $80, so I've repurchased a few more back to save together	Jingdong prefecture level city in Shandong
Fortune Shop	Green Intelligent Power Saver	I haven't used it yet and will comment when I do, but it's well made.	Jingdong prefecture level city in Shandong
Fortune Shop	Green Intelligent Power Saver	It's quite energy efficient. It's really a boon for the home.	Jingdong prefecture level city in Shandong
Fortune Shop	Green Intelligent Power Saver	Energy-saving socket received a month, after using it for a month proved that it is indeed energy-saving, electricity bill saving, will buy it again, thank you!	Jingdong prefecture level city in Shandong
Fortune Shop	Green Intelligent Power Saver	There was a similar product many years ago. Now technology has evolved, buy one to try, according to the instructions in the box, it takes one or two dozen days to show results. Check your electric bill and see what happens.	Jingdong prefecture level city in Shandong
Fortune Shop	Green Intelligent Power Saver	Jingdong logistics is really fast, the same day the order arrived on the same day, more convenient than the supermarket, and a wide range of choices, the	Jingdong prefecture level city in Shandong
Fortune Shop	Green Intelligent Power Saver	I've been using this for a month or so and I can't see any results, it really is a scam as they say.	Jingdong prefecture level city in Shandong
Fortune Shop	Green Intelligent Power Saver	Very good use, a month can save a few degrees of power loss of electricity, introduced to the relatives to buy back to try.	Jingdong prefecture level city in Shandong
Fortune Shop	Green Intelligent Power Saver	It's really powerful! With this air conditioner can be turned on at any time without the pressure of paying the electricity bill! It's good for the people!	Jingdong prefecture level city in Shandong
Fortune Shop	Green Intelligent Power Saver	This business is a big ** products are not qualified, with a month of electricity more than one hundred percent of thirty, the purchase agreed to return the goods but do not receive a refund after receiving the goods, looking for the official side does not help, the Jingdong is a big **, I have never shopped in	Jingdong prefecture level city in Shandong
Fortune Shop	Green Intelligent Power Saver	It's good. I tried it. It saves power.	Jingdong prefecture level city in Shandong
Fortune Shop	Green Intelligent Power Saver	Nothing**, it seems like there's more	Jingdong prefecture level city in Shandong
Fortune Shop	Green Intelligent Power Saver	Power saving device received, well packaged, good quality, fast logistics	Jingdong prefecture level city in Shandong
Fortune Shop	Green Intelligent Power Saver	The service is okay, is that when I bought five, the desirable pieces are three, and then wait a few days and send me two replacement, I do not know if it is a mistake of the shipper, if I was not in the courier there to see, go home to say no, the	Jingdong prefecture level city in Shandong
Fortune Shop	Green Intelligent Power Saver	This thing will only cost me more if I use it, but before I used it, it was less than a ` electricity a month, and after I used it, it was more than two hundred kWh a month.	Jingdong prefecture level city in Shandong
Fortune Shop	Green Intelligent Power Saver	I just used it. I don't know how it is.	Jingdong prefecture level city in Shandong
Fortune Shop	Green Intelligent Power Saver	I've received the product and I'm using it, so I'll see how it goes in a month!	Jingdong prefecture level city in Shandong
Fortune Shop	Green Intelligent Power Saver	The goods have just received the effect do not know how, please look forward to the results.	Jingdong prefecture level city in Shandong
Fortune Shop	Green Intelligent Power Saver	Power saving device, the material is very good practical and durable, the size is suitable, packaging is very good, the price is cheap, logistics is very fast!	Jingdong prefecture level city in Shandong
Fortune Shop	Green Intelligent Power Saver	Very nice, will comment in detail after using it for a while.	Jingdong prefecture level city in Shandong
Fortune Shop	Green Intelligent Power Saver	Good stuff to trust and support	Jingdong prefecture level city in Shandong
Fortune Shop	Green Intelligent Power Saver	I'll comment after I use it, doubt if it really saves power no	Jingdong prefecture level city in Shandong
Fortune Shop	Green Intelligent Power Saver	It's good quality and works well!	Jingdong prefecture level city in Shandong
Fortune Shop	Green Intelligent Power Saver	hahaha just right hahahahahaha hiccup hahahaha	Jingdong prefecture level city in Shandong
Fortune Shop	Green Intelligent Power Saver	The real thing matches the picture and looks great!	Jingdong prefecture level city in Shandong
Fortune Shop	Green Intelligent Power Saver	Plugged it in and we'll see when we pay the bills at the end of the year!	Jingdong prefecture level city in Shandong
Fortune Shop	Green Intelligent Power Saver	False advertising. It doesn't work.	Jingdong prefecture level city in Shandong
Fortune Shop	Green Intelligent Power Saver	Goods received, good effect	Jingdong prefecture level city in Shandong
Fortune Shop	Green Intelligent Power Saver	It looks okay, we'll see if it saves electricity when the time comes	Jingdong prefecture level city in Shandong
Fortune Shop	Green Intelligent Power Saver	Logistics is very fast quality is very good, the price is very affordable, accessories are also complete, installed, satisfied with good use	Jingdong prefecture level city in Shandong
Fortune Shop	Green Intelligent Power Saver	I've received the power saver. I'm looking forward to the savings.	Jingdong prefecture level city in Shandong
Fortune Shop	Green Intelligent Power Saver	Received, have not yet used, after use and then evaluate	Jingdong prefecture level city in Shandong
Fortune Shop	Green Intelligent Power Saver	I received the item, but I don't know if it saves power or not.	Jingdong prefecture level city in Shandong
Fortune Shop	Green Intelligent Power Saver	On Trial	Jingdong prefecture level city in Shandong
Fortune Shop	Green Intelligent Power Saver	Use it and see how it goes.	Jingdong prefecture level city in Shandong
Fortune Shop	Green Intelligent Power Saver	Quality is very good, no odor, fine workmanship, grain color are very like, satisfied!	Jingdong prefecture level city in Shandong
Fortune Shop	Green Intelligent Power Saver	I'll give you a good review. I don't know if it'll work.	Jingdong prefecture level city in Shandong
Fortune Shop	Green Intelligent Power Saver	The logistics is very fast and the package is intact. Good use of affordable. The effect is very good, is genuine	Jingdong prefecture level city in Shandong
Fortune Shop	Green Intelligent Power Saver	Nothing happens when you plug it in.	Jingdong prefecture level city in Shandong
Fortune Shop	Green Intelligent Power Saver	I don't know if it's as good as it sounds.	Jingdong prefecture level city in Shandong
Fortune Shop	Green Intelligent Power Saver	The quality of the stuff is very good, exactly the same as the seller's description, very satisfied!	Jingdong prefecture level city in Shandong
Fortune Shop	Green Intelligent Power Saver	The saver works great, it really works, it's affordable and worth buying.	Jingdong prefecture level city in Shandong
Fortune Shop	Green Intelligent Power Saver	Just used it for a couple days. I don't know yet.	Jingdong prefecture level city in Shandong

Fortune Shop	Green Intelligent Power Saver	The quality is quite good, cheaper than the physical store, good, next time will come again!	Jingdong prefecture level city in Shandong
Fortune Shop	Green Intelligent Power Saver	Good quality, cheap and affordable worth recommending to buy!	Jingdong prefecture level city in Shandong
Fortune Shop	Green Intelligent Power Saver	Very good quality, well made, works well! Satisfied	Jingdong prefecture level city in Shandong
Fortune Shop	Green Intelligent Power Saver	The logistics is very fast quality is very good very pleasant a shopping	Jingdong prefecture level city in Shandong
Fortune Shop	Green Intelligent Power Saver	Use it and then come back to evaluate!	Jingdong prefecture level city in Shandong
Fortune Shop	Green Intelligent Power Saver	# It's no use lying at all #	Jingdong prefecture level city in Shandong
Fortune Shop	Green Intelligent Power Saver	I can't get a voltage drop.	Jingdong prefecture level city in Shandong
Fortune Shop	Green Intelligent Power Saver	Evaluate after use	Jingdong prefecture level city in Shandong
Fortune Shop	Green Intelligent Power Saver	Still on trial.	Jingdong prefecture level city in Shandong
Fortune Shop	Green Intelligent Power Saver	ineffective	Jingdong prefecture level city in Shandong
Fortune Shop	Green Intelligent Power Saver	Satisfaction Shopping	Jingdong prefecture level city in Shandong
Fortune Shop	Green Intelligent Power Saver	Lies.	Jingdong prefecture level city in Shandong
Fortune Shop	Green Intelligent Power Saver	It's a real power saver.	Jingdong prefecture level city in Shandong
Fortune Shop	Green Intelligent Power Saver	in use	Jingdong prefecture level city in Shandong
Fortune Shop	Green Intelligent Power Saver	I don't know yet.	Jingdong prefecture level city in Shandong
Fortune Shop	Green Intelligent Power Saver	not care about anything	Jingdong prefecture level city in Shandong
Fortune Shop	Green Intelligent Power Saver	It doesn't seem to have much effect	Jingdong prefecture level city in Shandong
Fortune Shop	Green Intelligent Power Saver	Let the merchant see for himself, does the plug positive and negative terminals look like this?	Jingdong prefecture level city in Shandong
Fortune Shop	Green Intelligent Power Saver	It doesn't work well, the electricity bill is less before using it, but more after using it, contacting customer service, the service is still not good, and returning it is slow	Jingdong prefecture level city in Shandong
Fortune Shop	Green Intelligent Power Saver	With the advertisement product does not match, above the effective negative, power saving rate of two rows of words it, the product looks very thin, with 3 days to see the meter number is still with the previous no difference, which is obviously fraudulent behavior!	Jingdong prefecture level city in Shandong
Fortune Shop	Green Intelligent Power Saver	Doesn't save any power at all. Not much use.	Jingdong prefecture level city in Shandong
Fortune Shop	Green Intelligent Power Saver	The plugs are all loose, they're all the same length and they don't work at all	Jingdong prefecture level city in Shandong
Fortune Shop	Green Intelligent Power Saver	It's bad 👎 it's not effective at all, you'd be crazy to sell this garbage on the internet.	Jingdong prefecture level city in Shandong
Fortune Shop	Green Intelligent Power Saver	It doesn't save any power at all, so I hope the old timers don't fall for this scam again	Jingdong prefecture level city in Shandong
Fortune Shop	Green Intelligent Power Saver	I've been using it for over a month now and haven't noticed any power savings.	Jingdong prefecture level city in Shandong
Fortune Shop	Green Intelligent Power Saver	It doesn't work at all. The electricity bill is higher when you use it.	Jingdong prefecture level city in Shandong
Fortune Shop	Green Intelligent Power Saver	It doesn't save any power, it's still as bad as ever.	Jingdong prefecture level city in Shandong
Fortune Shop	Green Intelligent Power Saver	Last night before leaving work to look at the table, and then plugged into the node device, this morning and then look at the table, than the original save a few degrees of electricity, very good to have a power-saving effect, easy to use, satisfied with the shopping praise.	Jingdong prefecture level city in Shandong
Fortune Shop	Green Intelligent Power Saver	The product is real and effective, the electricity bill is reduced, really good, thanks to the merchants have so many good products, the product is real and effective, the electricity bill is reduced, want to save electricity friends quickly buy it, early use early to save money.	Jingdong prefecture level city in Shandong
Fortune Shop	Green Intelligent Power Saver	Saver saves time and money, baby appearance is beautiful, the quality of goods is good, and the same as the business description, good service attitude, packaging is complete, logistics is very fast, praise to	Jingdong prefecture level city in Shandong
Fortune Shop	Green Intelligent Power Saver	The effect is good, summer, home electricity is relatively large, buy and use a few days, feel the effect is very good, originally ten dollars a day electricity bill, now eight dollars a day, quite good	Jingdong prefecture level city in Shandong
Fortune Shop	Green Intelligent Power Saver	Indeed can save electricity and money, the first time I know this or from a friend's home to see, but also do not believe it, and now they use it to know that there is this good thing.	Jingdong prefecture level city in Shandong
Fortune Shop	Green Intelligent Power Saver	I have received the Smart Power Saver. Perfectly packaged. Fast delivery. Boss customer service attitude is also very good. It's worth buying. I plugged it in and tried it and it really saves more power than the original. I'll buy it again next time.	Jingdong prefecture level city in Shandong
Fortune Shop	Green Intelligent Power Saver	It's nice to save a dozen bucks a night on two air conditioners that used to take 30 or 40 degrees a night, but now it's only 10 or so degrees a night	Jingdong prefecture level city in Shandong
Fortune Shop	Green Intelligent Power Saver	Product no manufacturer's name no product qualification certificate, try two days to see the effect of	Jingdong prefecture level city in Shandong
Fortune Shop	Green Intelligent Power Saver	I've been using it for over a month and have saved over $80, so I've repurchased a few more back to save together	Jingdong prefecture level city in Shandong
Fortune Shop	Green Intelligent Power Saver	I haven't used it yet and will comment when I do, but it's well made.	Jingdong prefecture level city in Shandong
Fortune Shop	Green Intelligent Power Saver	It's quite energy efficient. It's really a boon for the home.	Jingdong prefecture level city in Shandong
Fortune Shop	Green Intelligent Power Saver	Energy-saving socket received a month, after using it for a month proved that it is indeed energy-saving, electricity bill saving, will buy it again, thank you!	Jingdong prefecture level city in Shandong
Fortune Shop	Green Intelligent Power Saver	There was a similar product many years ago. Now technology has evolved, buy one to try, according to the instructions in the box, it takes one or two dozen days to show results. Check your electric bill and see what happens.	Jingdong prefecture level city in Shandong
Fortune Shop	Green Intelligent Power Saver	Jingdong logistics is really fast, the same day the order arrived on the same day, more convenient than the supermarket, and a wide range of choices, the	Jingdong prefecture level city in Shandong
Fortune Shop	Green Intelligent Power Saver	I've been using this for a month or so and I can't see any results, it really is a scam as they say.	Jingdong prefecture level city in Shandong
Fortune Shop	Green Intelligent Power Saver	Very good use, a month can save a few degrees of power loss of electricity, introduced to the relatives to buy back to try.	Jingdong prefecture level city in Shandong
Fortune Shop	Green Intelligent Power Saver	It's really powerful! With this air conditioner can be turned on at any time without the pressure of paying the electricity bill! It's good for the people!	Jingdong prefecture level city in Shandong
Fortune Shop	Green Intelligent Power Saver	This business is a big ** products are not qualified, with a month of electricity more than one hundred percent of thirty, the purchase agreed to return the goods but do not receive a refund after receiving the goods, looking for the official side does not help, the Jingdong is a big **, I have never shopped in	Jingdong prefecture level city in Shandong
Fortune Shop	Green Intelligent Power Saver	It's good. I tried it. It saves power.	Jingdong prefecture level city in Shandong
Fortune Shop	Green Intelligent Power Saver	Nothing**, it seems like there's more	Jingdong prefecture level city in Shandong
Fortune Shop	Green Intelligent Power Saver	Power saving device received, well packaged, good quality, fast logistics	Jingdong prefecture level city in Shandong

Fortune Shop	Green Intelligent Power Saver	The service is okay, is that when I bought five, the desirable pieces are three, and then wait a few days and send me two replacement, I do not know if it is a mistake of the shipper, if I was not in the courier there to see, go home to say no, the	Jingdong prefecture level city in Shandong
Fortune Shop	Green Intelligent Power Saver	This thing will only cost me more if I use it, but before I used it, it was less than a ~ electricity a month, and after I used it, it was more than two hundred kWh a month.	Jingdong prefecture level city in Shandong
Fortune Shop	Green Intelligent Power Saver	I just used it. I don't know how it is.	Jingdong prefecture level city in Shandong
Fortune Shop	Green Intelligent Power Saver	I've received the product and I'm using it, so I'll see how it goes in a month!	Jingdong prefecture level city in Shandong
Fortune Shop	Green Intelligent Power Saver	The goods have just received the effect do not know how, please look forward to the results.	Jingdong prefecture level city in Shandong
Fortune Shop	Green Intelligent Power Saver	Power saving device, the material is very good practical and durable, the size is suitable, packaging is very good, the price is cheap, logistics is very fast!	Jingdong prefecture level city in Shandong
Fortune Shop	Green Intelligent Power Saver	Very nice, will comment in detail after using it for a while.	Jingdong prefecture level city in Shandong
Fortune Shop	Green Intelligent Power Saver	Good stuff to trust and support	Jingdong prefecture level city in Shandong
Fortune Shop	Green Intelligent Power Saver	I'll comment after I use it, doubt if it really saves power no	Jingdong prefecture level city in Shandong
Fortune Shop	Green Intelligent Power Saver	It's good quality and works well!	Jingdong prefecture level city in Shandong
Fortune Shop	Green Intelligent Power Saver	hahaha just right hahahahahaha hiccup hahahaha	Jingdong prefecture level city in Shandong
Fortune Shop	Green Intelligent Power Saver	The real thing matches the picture and looks great!	Jingdong prefecture level city in Shandong
Fortune Shop	Green Intelligent Power Saver	Plugged it in and we'll see when we pay the bills at the end of the year!	Jingdong prefecture level city in Shandong
Fortune Shop	Green Intelligent Power Saver	False advertising. It doesn't work.	Jingdong prefecture level city in Shandong
Fortune Shop	Green Intelligent Power Saver	Goods received, good effect	Jingdong prefecture level city in Shandong
Fortune Shop	Green Intelligent Power Saver	It looks okay, we'll see if it saves electricity when the time comes	Jingdong prefecture level city in Shandong
Fortune Shop	Green Intelligent Power Saver	Logistics is very fast quality is very good, the price is very affordable, accessories are also complete, installed, satisfied with good use	Jingdong prefecture level city in Shandong
Fortune Shop	Green Intelligent Power Saver	I've received the power saver. I'm looking forward to the savings.	Jingdong prefecture level city in Shandong
Fortune Shop	Green Intelligent Power Saver	Received, have not yet used, after use and then evaluate	Jingdong prefecture level city in Shandong
Fortune Shop	Green Intelligent Power Saver	I received the item, but I don't know if it saves power or not.	Jingdong prefecture level city in Shandong
Fortune Shop	Green Intelligent Power Saver	On Trial	Jingdong prefecture level city in Shandong
Fortune Shop	Green Intelligent Power Saver	Use it and see how it goes.	Jingdong prefecture level city in Shandong
Fortune Shop	Green Intelligent Power Saver	Quality is very good, no odor, fine workmanship, grain color are very like, satisfied!	Jingdong prefecture level city in Shandong
Fortune Shop	Green Intelligent Power Saver	I'll give you a good review. I don't know if it'll work.	Jingdong prefecture level city in Shandong
Fortune Shop	Green Intelligent Power Saver	The logistics is very fast and the package is intact. Good use of affordable. The effect is very good, is genuine	Jingdong prefecture level city in Shandong
Fortune Shop	Green Intelligent Power Saver	Nothing happens when you plug it in.	Jingdong prefecture level city in Shandong
Fortune Shop	Green Intelligent Power Saver	I don't know if it's as good as it sounds.	Jingdong prefecture level city in Shandong
Fortune Shop	Green Intelligent Power Saver	The quality of the stuff is very good, exactly the same as the seller's description, very satisfied!	Jingdong prefecture level city in Shandong
Fortune Shop	Green Intelligent Power Saver	The saver works great, it really works, it's affordable and worth buying.	Jingdong prefecture level city in Shandong
Fortune Shop	Green Intelligent Power Saver	Just used it for a couple days. I don't know yet.	Jingdong prefecture level city in Shandong
Fortune Shop	Green Intelligent Power Saver	The quality is quite good, cheaper than the physical store, good, next time will come again!	Jingdong prefecture level city in Shandong
Fortune Shop	Green Intelligent Power Saver	Good quality, cheap and affordable worth recommending to buy!	Jingdong prefecture level city in Shandong
Fortune Shop	Green Intelligent Power Saver	Very good quality, well made, works well! Satisfied	Jingdong prefecture level city in Shandong
Fortune Shop	Green Intelligent Power Saver	The logistics is very fast quality is very good very pleasant a shopping	Jingdong prefecture level city in Shandong
Fortune Shop	Green Intelligent Power Saver	Use it and then come back to evaluate!	Jingdong prefecture level city in Shandong
Fortune Shop	Green Intelligent Power Saver	# It's no use lying at all #	Jingdong prefecture level city in Shandong
Fortune Shop	Green Intelligent Power Saver	I can't get a voltage drop.	Jingdong prefecture level city in Shandong
Fortune Shop	Green Intelligent Power Saver	Evaluate after use	Jingdong prefecture level city in Shandong
Fortune Shop	Green Intelligent Power Saver	Still on trial.	Jingdong prefecture level city in Shandong
Fortune Shop	Green Intelligent Power Saver	ineffective	Jingdong prefecture level city in Shandong
Fortune Shop	Green Intelligent Power Saver	Satisfaction Shopping	Jingdong prefecture level city in Shandong
Fortune Shop	Green Intelligent Power Saver	Lies.	Jingdong prefecture level city in Shandong
Fortune Shop	Green Intelligent Power Saver	It's a real power saver.	Jingdong prefecture level city in Shandong
Fortune Shop	Green Intelligent Power Saver	in use	Jingdong prefecture level city in Shandong
Fortune Shop	Green Intelligent Power Saver	I don't know yet.	Jingdong prefecture level city in Shandong
Fortune Shop	Green Intelligent Power Saver	not care about anything	Jingdong prefecture level city in Shandong
Fortune Shop	Green Intelligent Power Saver	It doesn't seem to have much effect	Jingdong prefecture level city in Shandong
Fortune Shop	Green Intelligent Power Saver	Let the merchant see for himself, does the plug positive and negative terminals look like this?	Jingdong prefecture level city in Shandong
Fortune Shop	Green Intelligent Power Saver	It doesn't work well, the electricity bill is less before using it, but more after using it, contacting customer service, the service is still not good, and returning it is slow	Jingdong prefecture level city in Shandong
Fortune Shop	Green Intelligent Power Saver	With the advertisement product does not match, above the effective negative, power saving rate of two rows of words it, the product looks very thin, with 3 days to see the meter number is still with the previous no difference, which is obviously fraudulent behavior!	Jingdong prefecture level city in Shandong

Fortune Shop	Green Intelligent Power Saver	Doesn't save any power at all. Not much use.	Jingdong prefecture level city in Shandong
Fortune Shop	Green Intelligent Power Saver	The plugs are all loose, they're all the same length and they don't work at all	Jingdong prefecture level city in Shandong
Fortune Shop	Green Intelligent Power Saver	It's bad 👎 it's not effective at all, you'd be crazy to sell this garbage on the internet.	Jingdong prefecture level city in Shandong
Fortune Shop	Green Intelligent Power Saver	It doesn't save any power at all, so I hope the old timers don't fall for this scam again	Jingdong prefecture level city in Shandong
Fortune Shop	Green Intelligent Power Saver	I've been using it for over a month now and haven't noticed any power savings.	Jingdong prefecture level city in Shandong
Fortune Shop	Green Intelligent Power Saver	It doesn't work at all. The electricity bill is higher when you use it.	Jingdong prefecture level city in Shandong
Fortune Shop	Green Intelligent Power Saver	It doesn't save any power, it's still as bad as ever.	Jingdong prefecture level city in Shandong
Fortune Shop	Green Intelligent Power Saver	Last night before leaving work to look at the table, and then plugged into the node device, this morning and then look at the table, than the original save a few degrees of electricity, very good to have a power-saving effect, easy to use, satisfied with the shopping praise.	Jingdong prefecture level city in Shandong
Fortune Shop	Green Intelligent Power Saver	The product is real and effective, the electricity bill is reduced, really good, thanks to the merchants have so many good products, the product is real and effective, the electricity bill is reduced, want to save electricity friends quickly buy it, early use early to save money.	Jingdong prefecture level city in Shandong
Fortune Shop	Green Intelligent Power Saver	Saver saves time and money, baby appearance is beautiful, the quality of goods is good, and the same as the business description, good service attitude, packaging is complete, logistics is very fast, praise to	Jingdong prefecture level city in Shandong
Fortune Shop	Green Intelligent Power Saver	The effect is good, summer, home electricity is relatively large, buy and use a few days, feel the effect is very good, originally ten dollars a day electricity bill, now eight dollars a day, quite good	Jingdong prefecture level city in Shandong
Fortune Shop	Green Intelligent Power Saver	Indeed can save electricity and money, the first time I know this or from a friend's home to see, but also do not believe it, and now they use it to know that there is this good thing.	Jingdong prefecture level city in Shandong
Fortune Shop	Green Intelligent Power Saver	I have received the Smart Power Saver. Perfectly packaged. Fast delivery. Boss customer service attitude is also very good. It's worth buying. I plugged it in and tried it and it really saves more power than the original. I'll buy it again next time.	Jingdong prefecture level city in Shandong
Fortune Shop	Green Intelligent Power Saver	It's nice to save a dozen bucks a night on two air conditioners that used to take 30 or 40 degrees a night, but now it's only 10 or so degrees a night	Jingdong prefecture level city in Shandong
Fortune Shop	Green Intelligent Power Saver	Product no manufacturer's name no product qualification certificate, try two days to see the effect of	Jingdong prefecture level city in Shandong
Fortune Shop	Green Intelligent Power Saver	I've been using it for over a month and have saved over $80, so I've repurchased a few more back to save together	Jingdong prefecture level city in Shandong
Fortune Shop	Green Intelligent Power Saver	I haven't used it yet and will comment when I do, but it's well made.	Jingdong prefecture level city in Shandong
Fortune Shop	Green Intelligent Power Saver	It's quite energy efficient. It's really a boon for the home.	Jingdong prefecture level city in Shandong
Fortune Shop	Green Intelligent Power Saver	Energy-saving socket received a month, after using it for a month proved that it is indeed energy-saving, electricity bill saving, will buy it again, thank you!	Jingdong prefecture level city in Shandong
Fortune Shop	Green Intelligent Power Saver	There was a similar product many years ago. Now technology has evolved, buy one to try, according to the instructions in the box, it takes one or two dozen days to show results. Check your electric bill and see what happens.	Jingdong prefecture level city in Shandong
Fortune Shop	Green Intelligent Power Saver	Jingdong logistics is really fast, the same day the order arrived on the same day, more convenient than the supermarket, and a wide range of choices, the	Jingdong prefecture level city in Shandong
Fortune Shop	Green Intelligent Power Saver	I've been using this for a month or so and I can't see any results, it really is a scam as they say.	Jingdong prefecture level city in Shandong
Fortune Shop	Green Intelligent Power Saver	Very good use, a month can save a few degrees of power loss of electricity, introduced to the relatives to buy back to try.	Jingdong prefecture level city in Shandong
Fortune Shop	Green Intelligent Power Saver	It's really powerful! With this air conditioner can be turned on at any time without the pressure of paying the electricity bill! It's good for the people!	Jingdong prefecture level city in Shandong
Fortune Shop	Green Intelligent Power Saver	This business is a big ** products are not qualified, with a month of electricity more than one hundred percent of thirty, the purchase agreed to return the goods but do not receive a refund after receiving the goods, looking for the official side does not help, the Jingdong is a big **, I have never shopped in	Jingdong prefecture level city in Shandong
Fortune Shop	Green Intelligent Power Saver	It's good. I tried it. It saves power.	Jingdong prefecture level city in Shandong
Fortune Shop	Green Intelligent Power Saver	Nothing**, it seems like there's more	Jingdong prefecture level city in Shandong
Fortune Shop	Green Intelligent Power Saver	Power saving device received, well packaged, good quality, fast logistics	Jingdong prefecture level city in Shandong
Fortune Shop	Green Intelligent Power Saver	The service is okay, is that when I bought five, the desirable pieces are three, and then wait a few days and send me two replacement, I do not know if it is a mistake of the shipper, if I was not in the courier there to see, go home to say no, the	Jingdong prefecture level city in Shandong
Fortune Shop	Green Intelligent Power Saver	This thing will only cost me more if I use it, but before I used it, it was less than a ~ electricity a month, and after I used it, it was more than two hundred kWh a month.	Jingdong prefecture level city in Shandong
Fortune Shop	Green Intelligent Power Saver	I just used it. I don't know how it is.	Jingdong prefecture level city in Shandong
Fortune Shop	Green Intelligent Power Saver	I've received the product and I'm using it, so I'll see how it goes in a month!	Jingdong prefecture level city in Shandong
Fortune Shop	Green Intelligent Power Saver	The goods have just received the effect do not know how, please look forward to the results.	Jingdong prefecture level city in Shandong
Fortune Shop	Green Intelligent Power Saver	Power saving device, the material is very good practical and durable, the size is suitable, packaging is very good, the price is cheap, logistics is very fast!	Jingdong prefecture level city in Shandong
Fortune Shop	Green Intelligent Power Saver	Very nice, will comment in detail after using it for a while.	Jingdong prefecture level city in Shandong
Fortune Shop	Green Intelligent Power Saver	Good stuff to trust and support	Jingdong prefecture level city in Shandong
Fortune Shop	Green Intelligent Power Saver	I'll comment after I use it, doubt if it really saves power no	Jingdong prefecture level city in Shandong
Fortune Shop	Green Intelligent Power Saver	It's good quality and works well!	Jingdong prefecture level city in Shandong
Fortune Shop	Green Intelligent Power Saver	hahaha just right hahahahahaha hiccup hahahaha	Jingdong prefecture level city in Shandong
Fortune Shop	Green Intelligent Power Saver	The real thing matches the picture and looks great!	Jingdong prefecture level city in Shandong
Fortune Shop	Green Intelligent Power Saver	Plugged it in and we'll see when we pay the bills at the end of the year!	Jingdong prefecture level city in Shandong
Fortune Shop	Green Intelligent Power Saver	False advertising. It doesn't work.	Jingdong prefecture level city in Shandong
Fortune Shop	Green Intelligent Power Saver	Goods received, good effect	Jingdong prefecture level city in Shandong
Fortune Shop	Green Intelligent Power Saver	It looks okay, we'll see if it saves electricity when the time comes	Jingdong prefecture level city in Shandong
Fortune Shop	Green Intelligent Power Saver	Logistics is very fast quality is very good, the price is very affordable, accessories are also complete, installed, satisfied with good use	Jingdong prefecture level city in Shandong
Fortune Shop	Green Intelligent Power Saver	I've received the power saver. I'm looking forward to the savings.	Jingdong prefecture level city in Shandong
Fortune Shop	Green Intelligent Power Saver	Received, have not yet used, after use and then evaluate	Jingdong prefecture level city in Shandong
Fortune Shop	Green Intelligent Power Saver	I received the item, but I don't know if it saves power or not.	Jingdong prefecture level city in Shandong

Fortune Shop	Green Intelligent Power Saver	On Trial	Jingdong prefecture level city in Shandong
Fortune Shop	Green Intelligent Power Saver	Use it and see how it goes.	Jingdong prefecture level city in Shandong
Fortune Shop	Green Intelligent Power Saver	Quality is very good, no odor, fine workmanship, grain color are very like, satisfied!	Jingdong prefecture level city in Shandong
Fortune Shop	Green Intelligent Power Saver	I'll give you a good review. I don't know if it'll work.	Jingdong prefecture level city in Shandong
Fortune Shop	Green Intelligent Power Saver	The logistics is very fast and the package is intact. Good use of affordable. The effect is very good, is genuine	Jingdong prefecture level city in Shandong
Fortune Shop	Green Intelligent Power Saver	Nothing happens when you plug it in.	Jingdong prefecture level city in Shandong
Fortune Shop	Green Intelligent Power Saver	I don't know if it's as good as it sounds.	Jingdong prefecture level city in Shandong
Fortune Shop	Green Intelligent Power Saver	The quality of the stuff is very good, exactly the same as the seller's description, very satisfied!	Jingdong prefecture level city in Shandong
Fortune Shop	Green Intelligent Power Saver	The saver works great, it really works, it's affordable and worth buying.	Jingdong prefecture level city in Shandong
Fortune Shop	Green Intelligent Power Saver	Just used it for a couple days. I don't know yet.	Jingdong prefecture level city in Shandong
Fortune Shop	Green Intelligent Power Saver	The quality is quite good, cheaper than the physical store, good, next time will come again!	Jingdong prefecture level city in Shandong
Fortune Shop	Green Intelligent Power Saver	Good quality, cheap and affordable worth recommending to buy!	Jingdong prefecture level city in Shandong
Fortune Shop	Green Intelligent Power Saver	Very good quality, well made, works well! Satisfied	Jingdong prefecture level city in Shandong
Fortune Shop	Green Intelligent Power Saver	The logistics is very fast quality is very good very pleasant a shopping	Jingdong prefecture level city in Shandong
Fortune Shop	Green Intelligent Power Saver	Use it and then come back to evaluate!	Jingdong prefecture level city in Shandong
Fortune Shop	Green Intelligent Power Saver	# It's no use lying at all #	Jingdong prefecture level city in Shandong
Fortune Shop	Green Intelligent Power Saver	I can't get a voltage drop.	Jingdong prefecture level city in Shandong
Fortune Shop	Green Intelligent Power Saver	Evaluate after use	Jingdong prefecture level city in Shandong
Fortune Shop	Green Intelligent Power Saver	Still on trial.	Jingdong prefecture level city in Shandong
Fortune Shop	Green Intelligent Power Saver	ineffective	Jingdong prefecture level city in Shandong
Fortune Shop	Green Intelligent Power Saver	Satisfaction Shopping	Jingdong prefecture level city in Shandong
Fortune Shop	Green Intelligent Power Saver	Lies.	Jingdong prefecture level city in Shandong
Fortune Shop	Green Intelligent Power Saver	It's a real power saver.	Jingdong prefecture level city in Shandong
Fortune Shop	Green Intelligent Power Saver	in use	Jingdong prefecture level city in Shandong
Fortune Shop	Green Intelligent Power Saver	I don't know yet.	Jingdong prefecture level city in Shandong
Fortune Shop	Green Intelligent Power Saver	not care about anything	Jingdong prefecture level city in Shandong
Fortune Shop	Green Intelligent Power Saver	It doesn't seem to have much effect	Jingdong prefecture level city in Shandong
Fortune Shop	Green Intelligent Power Saver	Let the merchant see for himself, does the plug positive and negative terminals look like this?	Jingdong prefecture level city in Shandong
Fortune Shop	Green Intelligent Power Saver	It doesn't work well, the electricity bill is less before using it, but more after using it, contacting customer service, the service is still not good, and returning it is slow	Jingdong prefecture level city in Shandong
Fortune Shop	Green Intelligent Power Saver	With the advertisement product does not match, above the effective negative, power saving rate of two rows of words it, the product looks very thin, with 3 days to see the meter number is still with the previous no difference, which is obviously fraudulent behavior!	Jingdong prefecture level city in Shandong
Fortune Shop	Green Intelligent Power Saver	Doesn't save any power at all. Not much use.	Jingdong prefecture level city in Shandong
Fortune Shop	Green Intelligent Power Saver	The plugs are all loose, they're all the same length and they don't work at all	Jingdong prefecture level city in Shandong
Fortune Shop	Green Intelligent Power Saver	It's bad 👎 it's not effective at all, you'd be crazy to sell this garbage on the internet.	Jingdong prefecture level city in Shandong
Fortune Shop	Green Intelligent Power Saver	It doesn't save any power at all, so I hope the old timers don't fall for this scam again	Jingdong prefecture level city in Shandong
Fortune Shop	Green Intelligent Power Saver	I've been using it for over a month now and haven't noticed any power savings.	Jingdong prefecture level city in Shandong
Fortune Shop	Green Intelligent Power Saver	It doesn't work at all. The electricity bill is higher when you use it.	Jingdong prefecture level city in Shandong
Fortune Shop	Green Intelligent Power Saver	It doesn't save any power, it's still as bad as ever.	Jingdong prefecture level city in Shandong
Fortune Shop	Green Intelligent Power Saver	Last night before leaving work to look at the table, and then plugged into the node device, this morning and then look at the table, than the original save a few degrees of electricity, very good to have a power-saving effect, easy to use, satisfied with the shopping praise.	Jingdong prefecture level city in Shandong
Fortune Shop	Green Intelligent Power Saver	The product is real and effective, the electricity bill is reduced, really good, thanks to the merchants have so many good products, the product is real and effective, the electricity bill is reduced, want to save electricity friends quickly buy it, early use early to save money.	Jingdong prefecture level city in Shandong
Fortune Shop	Green Intelligent Power Saver	Saver saves time and money, baby appearance is beautiful, the quality of goods is good, and the same as the business description, good service attitude, packaging is complete, logistics is very fast, praise to	Jingdong prefecture level city in Shandong
Fortune Shop	Green Intelligent Power Saver	The effect is good, summer, home electricity is relatively large, buy and use a few days, feel the effect is very good, originally ten dollars a day electricity bill, now eight dollars a day, quite good	Jingdong prefecture level city in Shandong
Fortune Shop	Green Intelligent Power Saver	Indeed can save electricity and money, the first time I know this or from a friend's home to see, but also do not believe it, and now they use it to know that there is this good thing.	Jingdong prefecture level city in Shandong
Fortune Shop	Green Intelligent Power Saver	I have received the Smart Power Saver. Perfectly packaged. Fast delivery. Boss customer service attitude is also very good. It's worth buying. I plugged it in and tried it and it really saves more power than the original. I'll buy it again next time.	Jingdong prefecture level city in Shandong
Fortune Shop	Green Intelligent Power Saver	It's nice to save a dozen bucks a night on two air conditioners that used to take 30 or 40 degrees a night, but now it's only 10 or so degrees a night	Jingdong prefecture level city in Shandong
Fortune Shop	Green Intelligent Power Saver	Product no manufacturer's name no product qualification certificate, try two days to see the effect of	Jingdong prefecture level city in Shandong
Fortune Shop	Green Intelligent Power Saver	I've been using it for over a month and have saved over $80, so I've repurchased a few more back to save together	Jingdong prefecture level city in Shandong
Fortune Shop	Green Intelligent Power Saver	I haven't used it yet and will comment when I do, but it's well made.	Jingdong prefecture level city in Shandong
Fortune Shop	Green Intelligent Power Saver	It's quite energy efficient. It's really a boon for the home.	Jingdong prefecture level city in Shandong
Fortune Shop	Green Intelligent Power Saver	Energy-saving socket received a month, after using it for a month proved that it is indeed energy-saving, electricity bill saving, will buy it again, thank you!	Jingdong prefecture level city in Shandong

Fortune Shop	Green Intelligent Power Saver	There was a similar product many years ago. Now technology has evolved, buy one to try, according to the instructions in the box, it takes one or two dozen days to show results. Check your electric bill and see what happens.	Jingdong prefecture level city in Shandong
Fortune Shop	Green Intelligent Power Saver	Jingdong logistics is really fast, the same day the order arrived on the same day, more convenient than the supermarket, and a wide range of choices, the	Jingdong prefecture level city in Shandong
Fortune Shop	Green Intelligent Power Saver	I've been using this for a month or so and I can't see any results, it really is a scam as they say.	Jingdong prefecture level city in Shandong
Fortune Shop	Green Intelligent Power Saver	Very good use, a month can save a few degrees of power loss of electricity, introduced to the relatives to buy back to try.	Jingdong prefecture level city in Shandong
Fortune Shop	Green Intelligent Power Saver	It's really powerful! With this air conditioner can be turned on at any time without the pressure of paying the electricity bill! It's good for the people!	Jingdong prefecture level city in Shandong
Fortune Shop	Green Intelligent Power Saver	This business is a big ** products are not qualified, with a month of electricity more than one hundred percent of thirty, the purchase agreed to return the goods but do not receive a refund after receiving the goods, looking for the official side does not help, the Jingdong is a big **, I have never shopped in	Jingdong prefecture level city in Shandong
Fortune Shop	Green Intelligent Power Saver	It's good. I tried it. It saves power.	Jingdong prefecture level city in Shandong
Fortune Shop	Green Intelligent Power Saver	Nothing**, it seems like there's more	Jingdong prefecture level city in Shandong
Fortune Shop	Green Intelligent Power Saver	Power saving device received, well packaged, good quality, fast logistics	Jingdong prefecture level city in Shandong
Fortune Shop	Green Intelligent Power Saver	The service is okay, is that when I bought five, the desirable pieces are three, and then wait a few days and send me two replacement, I do not know if it is a mistake of the shipper, if I was not in the courier there to see, go home to say no, the	Jingdong prefecture level city in Shandong
Fortune Shop	Green Intelligent Power Saver	This thing will only cost me more if I use it, but before I used it, it was less than a ~ electricity a month, and after I used it, it was more than two hundred kWh a month.	Jingdong prefecture level city in Shandong
Fortune Shop	Green Intelligent Power Saver	I just used it. I don't know how it is.	Jingdong prefecture level city in Shandong
Fortune Shop	Green Intelligent Power Saver	I've received the product and I'm using it, so I'll see how it goes in a month!	Jingdong prefecture level city in Shandong
Fortune Shop	Green Intelligent Power Saver	The goods have just received the effect do not know how, please look forward to the results.	Jingdong prefecture level city in Shandong
Fortune Shop	Green Intelligent Power Saver	Power saving device, the material is very good practical and durable, the size is suitable, packaging is very good, the price is cheap, logistics is very fast!	Jingdong prefecture level city in Shandong
Fortune Shop	Green Intelligent Power Saver	Very nice, will comment in detail after using it for a while.	Jingdong prefecture level city in Shandong
Fortune Shop	Green Intelligent Power Saver	Good stuff to trust and support	Jingdong prefecture level city in Shandong
Fortune Shop	Green Intelligent Power Saver	I'll comment after I use it, doubt if it really saves power no	Jingdong prefecture level city in Shandong
Fortune Shop	Green Intelligent Power Saver	It's good quality and works well!	Jingdong prefecture level city in Shandong
Fortune Shop	Green Intelligent Power Saver	hahaha just right hahahahahaha hiccup hahahaha	Jingdong prefecture level city in Shandong
Fortune Shop	Green Intelligent Power Saver	The real thing matches the picture and looks great!	Jingdong prefecture level city in Shandong
Fortune Shop	Green Intelligent Power Saver	Plugged it in and we'll see when we pay the bills at the end of the year!	Jingdong prefecture level city in Shandong
Fortune Shop	Green Intelligent Power Saver	False advertising. It doesn't work.	Jingdong prefecture level city in Shandong
Fortune Shop	Green Intelligent Power Saver	Goods received, good effect	Jingdong prefecture level city in Shandong
Fortune Shop	Green Intelligent Power Saver	It looks okay, we'll see if it saves electricity when the time comes	Jingdong prefecture level city in Shandong
Fortune Shop	Green Intelligent Power Saver	Logistics is very fast quality is very good, the price is very affordable, accessories are also complete, installed, satisfied with good use	Jingdong prefecture level city in Shandong
Fortune Shop	Green Intelligent Power Saver	I've received the power saver. I'm looking forward to the savings.	Jingdong prefecture level city in Shandong
Fortune Shop	Green Intelligent Power Saver	Received, have not yet used, after use and then evaluate	Jingdong prefecture level city in Shandong
Fortune Shop	Green Intelligent Power Saver	I received the item, but I don't know if it saves power or not.	Jingdong prefecture level city in Shandong
Fortune Shop	Green Intelligent Power Saver	On Trial	Jingdong prefecture level city in Shandong
Fortune Shop	Green Intelligent Power Saver	Use it and see how it goes.	Jingdong prefecture level city in Shandong
Fortune Shop	Green Intelligent Power Saver	Quality is very good, no odor, fine workmanship, grain color are very like, satisfied!	Jingdong prefecture level city in Shandong
Fortune Shop	Green Intelligent Power Saver	I'll give you a good review. I don't know if it'll work.	Jingdong prefecture level city in Shandong
Fortune Shop	Green Intelligent Power Saver	The logistics is very fast and the package is intact. Good use of affordable. The effect is very good, is genuine	Jingdong prefecture level city in Shandong
Fortune Shop	Green Intelligent Power Saver	Nothing happens when you plug it in.	Jingdong prefecture level city in Shandong
Fortune Shop	Green Intelligent Power Saver	I don't know if it's as good as it sounds.	Jingdong prefecture level city in Shandong
Fortune Shop	Green Intelligent Power Saver	The quality of the stuff is very good, exactly the same as the seller's description, very satisfied!	Jingdong prefecture level city in Shandong
Fortune Shop	Green Intelligent Power Saver	The saver works great, it really works, it's affordable and worth buying.	Jingdong prefecture level city in Shandong
Fortune Shop	Green Intelligent Power Saver	Just used it for a couple days. I don't know yet.	Jingdong prefecture level city in Shandong
Fortune Shop	Green Intelligent Power Saver	The quality is quite good, cheaper than the physical store, good, next time will come again!	Jingdong prefecture level city in Shandong
Fortune Shop	Green Intelligent Power Saver	Good quality, cheap and affordable worth recommending to buy!	Jingdong prefecture level city in Shandong
Fortune Shop	Green Intelligent Power Saver	Very good quality, well made, works well! Satisfied	Jingdong prefecture level city in Shandong
Fortune Shop	Green Intelligent Power Saver	The logistics is very fast quality is very good very pleasant a shopping	Jingdong prefecture level city in Shandong
Fortune Shop	Green Intelligent Power Saver	Use it and then come back to evaluate!	Jingdong prefecture level city in Shandong
Fortune Shop	Green Intelligent Power Saver	# It's no use lying at all #	Jingdong prefecture level city in Shandong
Fortune Shop	Green Intelligent Power Saver	I can't get a voltage drop.	Jingdong prefecture level city in Shandong
Fortune Shop	Green Intelligent Power Saver	Evaluate after use	Jingdong prefecture level city in Shandong
Fortune Shop	Green Intelligent Power Saver	Still on trial.	Jingdong prefecture level city in Shandong
Fortune Shop	Green Intelligent Power Saver	ineffective	Jingdong prefecture level city in Shandong
Fortune Shop	Green Intelligent Power Saver	Satisfaction Shopping	Jingdong prefecture level city in Shandong

Fortune Shop	Green Intelligent Power Saver	Lies.	Jingdong prefecture level city in Shandong
Fortune Shop	Green Intelligent Power Saver	It's a real power saver.	Jingdong prefecture level city in Shandong
Fortune Shop	Green Intelligent Power Saver	in use	Jingdong prefecture level city in Shandong
Fortune Shop	Green Intelligent Power Saver	I don't know yet.	Jingdong prefecture level city in Shandong
Fortune Shop	Green Intelligent Power Saver	not care about anything	Jingdong prefecture level city in Shandong
Fortune Shop	Green Intelligent Power Saver	It doesn't seem to have much effect	Jingdong prefecture level city in Shandong
Fortune Shop	Green Intelligent Power Saver	Let the merchant see for himself, does the plug positive and negative terminals look like this?	Jingdong prefecture level city in Shandong
Fortune Shop	Green Intelligent Power Saver	It doesn't work well, the electricity bill is less before using it, but more after using it, contacting customer service, the service is still not good, and returning it is slow	Jingdong prefecture level city in Shandong
Fortune Shop	Green Intelligent Power Saver	With the advertisement product does not match, above the effective negative, power saving rate of two rows of words it, the product looks very thin, with 3 days to see the meter number is still with the previous no difference, which is obviously fraudulent behavior!	Jingdong prefecture level city in Shandong
Fortune Shop	Green Intelligent Power Saver	Doesn't save any power at all. Not much use.	Jingdong prefecture level city in Shandong
Fortune Shop	Green Intelligent Power Saver	The plugs are all loose, they're all the same length and they don't work at all	Jingdong prefecture level city in Shandong
Fortune Shop	Green Intelligent Power Saver	It's bad 👎 it's not effective at all, you'd be crazy to sell this garbage on the internet.	Jingdong prefecture level city in Shandong
Fortune Shop	Green Intelligent Power Saver	It doesn't save any power at all, so I hope the old timers don't fall for this scam again	Jingdong prefecture level city in Shandong
Fortune Shop	Green Intelligent Power Saver	I've been using it for over a month now and haven't noticed any power savings.	Jingdong prefecture level city in Shandong
Fortune Shop	Green Intelligent Power Saver	It doesn't work at all. The electricity bill is higher when you use it.	Jingdong prefecture level city in Shandong
Fortune Shop	Green Intelligent Power Saver	It doesn't save any power, it's still as bad as ever.	Jingdong prefecture level city in Shandong
Fortune Shop	Green Intelligent Power Saver	Last night before leaving work to look at the table, and then plugged into the node device, this morning and then look at the table, than the original save a few degrees of electricity, very good to have a power-saving effect, easy to use, satisfied with the shopping praise.	Jingdong prefecture level city in Shandong
Fortune Shop	Green Intelligent Power Saver	The product is real and effective, the electricity bill is reduced, really good, thanks to the merchants have so many good products, the product is real and effective, the electricity bill is reduced, want to save electricity friends quickly buy it, early use early to save money.	Jingdong prefecture level city in Shandong
Fortune Shop	Green Intelligent Power Saver	Saver saves time and money, baby appearance is beautiful, the quality of goods is good, and the same as the business description, good service attitude, packaging is complete, logistics is very fast, praise to	Jingdong prefecture level city in Shandong
Fortune Shop	Green Intelligent Power Saver	The effect is good, summer, home electricity is relatively large, buy and use a few days, feel the effect is very good, originally ten dollars a day electricity bill, now eight dollars a day, quite good	Jingdong prefecture level city in Shandong
Fortune Shop	Green Intelligent Power Saver	Indeed can save electricity and money, the first time I know this or from a friend's home to see, but also do not believe it, and now they use it to know that there is this good thing.	Jingdong prefecture level city in Shandong
Fortune Shop	Green Intelligent Power Saver	I have received the Smart Power Saver. Perfectly packaged. Fast delivery. Boss customer service attitude is also very good. It's worth buying. I plugged it in and tried it and it really saves more power than the original. I'll buy it again next time.	Jingdong prefecture level city in Shandong
Fortune Shop	Green Intelligent Power Saver	It's nice to save a dozen bucks a night on two air conditioners that used to take 30 or 40 degrees a night, but now it's only 10 or so degrees a night	Jingdong prefecture level city in Shandong
Fortune Shop	Green Intelligent Power Saver	Product no manufacturer's name no product qualification certificate, try two days to see the effect of	Jingdong prefecture level city in Shandong
Fortune Shop	Green Intelligent Power Saver	I've been using it for over a month and have saved over $80, so I've repurchased a few more back to save together	Jingdong prefecture level city in Shandong
Fortune Shop	Green Intelligent Power Saver	I haven't used it yet and will comment when I do, but it's well made.	Jingdong prefecture level city in Shandong
Fortune Shop	Green Intelligent Power Saver	It's quite energy efficient. It's really a boon for the home.	Jingdong prefecture level city in Shandong
Fortune Shop	Green Intelligent Power Saver	Energy-saving socket received a month, after using it for a month proved that it is indeed energy-saving, electricity bill saving, will buy it again, thank you!	Jingdong prefecture level city in Shandong
Fortune Shop	Green Intelligent Power Saver	There was a similar product many years ago. Now technology has evolved, buy one to try, according to the instructions in the box, it takes one or two dozen days to show results. Check your electric bill and see what happens.	Jingdong prefecture level city in Shandong
Fortune Shop	Green Intelligent Power Saver	Jingdong logistics is really fast, the same day the order arrived on the same day, more convenient than the supermarket, and a wide range of choices, the	Jingdong prefecture level city in Shandong
Fortune Shop	Green Intelligent Power Saver	I've been using this for a month or so and I can't see any results, it really is a scam as they say.	Jingdong prefecture level city in Shandong
Fortune Shop	Green Intelligent Power Saver	Very good use, a month can save a few degrees of power loss of electricity, introduced to the relatives to buy back to try.	Jingdong prefecture level city in Shandong
Fortune Shop	Green Intelligent Power Saver	It's really powerful! With this air conditioner can be turned on at any time without the pressure of paying the electricity bill! It's good for the people!	Jingdong prefecture level city in Shandong
Fortune Shop	Green Intelligent Power Saver	This business is a big ** products are not qualified, with a month of electricity more than one hundred percent of thirty, the purchase agreed to return the goods but do not receive a refund after receiving the goods, looking for the official side does not help, the Jingdong is a big **, I have never shopped in	Jingdong prefecture level city in Shandong
Fortune Shop	Green Intelligent Power Saver	It's good. I tried it. It saves power.	Jingdong prefecture level city in Shandong
Fortune Shop	Green Intelligent Power Saver	Nothing**, it seems like there's more	Jingdong prefecture level city in Shandong
Fortune Shop	Green Intelligent Power Saver	Power saving device received, well packaged, good quality, fast logistics	Jingdong prefecture level city in Shandong
Fortune Shop	Green Intelligent Power Saver	The service is okay, is that when I bought five, the desirable pieces are three, and then wait a few days and send me two replacement, I do not know if it is a mistake of the shipper, if I was not in the courier there to see, go home to say no, the	Jingdong prefecture level city in Shandong
Fortune Shop	Green Intelligent Power Saver	This thing will only cost me more if I use it, but before I used it, it was less than a ~ electricity a month, and after I used it, it was more than two hundred kWh a month.	Jingdong prefecture level city in Shandong
Fortune Shop	Green Intelligent Power Saver	I just used it. I don't know how it is.	Jingdong prefecture level city in Shandong
Fortune Shop	Green Intelligent Power Saver	I've received the product and I'm using it, so I'll see how it goes in a month!	Jingdong prefecture level city in Shandong
Fortune Shop	Green Intelligent Power Saver	The goods have just received the effect do not know how, please look forward to the results.	Jingdong prefecture level city in Shandong
Fortune Shop	Green Intelligent Power Saver	Power saving device, the material is very good practical and durable, the size is suitable, packaging is very good, the price is cheap, logistics is very fast!	Jingdong prefecture level city in Shandong
Fortune Shop	Green Intelligent Power Saver	Very nice, will comment in detail after using it for a while.	Jingdong prefecture level city in Shandong
Fortune Shop	Green Intelligent Power Saver	Good stuff to trust and support	Jingdong prefecture level city in Shandong
Fortune Shop	Green Intelligent Power Saver	I'll comment after I use it, doubt if it really saves power no	Jingdong prefecture level city in Shandong
Fortune Shop	Green Intelligent Power Saver	It's good quality and works well!	Jingdong prefecture level city in Shandong
Fortune Shop	Green Intelligent Power Saver	hahaha just right hahahahahaha hiccup hahahaha	Jingdong prefecture level city in Shandong

Fortune Shop	Green Intelligent Power Saver	The real thing matches the picture and looks great!	Jingdong prefecture level city in Shandong
Fortune Shop	Green Intelligent Power Saver	Plugged it in and we'll see when we pay the bills at the end of the year!	Jingdong prefecture level city in Shandong
Fortune Shop	Green Intelligent Power Saver	False advertising. It doesn't work.	Jingdong prefecture level city in Shandong
Fortune Shop	Green Intelligent Power Saver	Goods received, good effect	Jingdong prefecture level city in Shandong
Fortune Shop	Green Intelligent Power Saver	It looks okay, we'll see if it saves electricity when the time comes	Jingdong prefecture level city in Shandong
Fortune Shop	Green Intelligent Power Saver	Logistics is very fast quality is very good, the price is very affordable, accessories are also complete, installed, satisfied with good use	Jingdong prefecture level city in Shandong
Fortune Shop	Green Intelligent Power Saver	I've received the power saver. I'm looking forward to the savings.	Jingdong prefecture level city in Shandong
Fortune Shop	Green Intelligent Power Saver	Received, have not yet used, after use and then evaluate	Jingdong prefecture level city in Shandong
Fortune Shop	Green Intelligent Power Saver	I received the item, but I don't know if it saves power or not.	Jingdong prefecture level city in Shandong
Fortune Shop	Green Intelligent Power Saver	On Trial	Jingdong prefecture level city in Shandong
Fortune Shop	Green Intelligent Power Saver	Use it and see how it goes.	Jingdong prefecture level city in Shandong
Fortune Shop	Green Intelligent Power Saver	Quality is very good, no odor, fine workmanship, grain color are very like, satisfied!	Jingdong prefecture level city in Shandong
Fortune Shop	Green Intelligent Power Saver	I'll give you a good review. I don't know if it'll work.	Jingdong prefecture level city in Shandong
Fortune Shop	Green Intelligent Power Saver	The logistics is very fast and the package is intact. Good use of affordable. The effect is very good, is genuine	Jingdong prefecture level city in Shandong
Fortune Shop	Green Intelligent Power Saver	Nothing happens when you plug it in.	Jingdong prefecture level city in Shandong
Fortune Shop	Green Intelligent Power Saver	I don't know if it's as good as it sounds.	Jingdong prefecture level city in Shandong
Fortune Shop	Green Intelligent Power Saver	The quality of the stuff is very good, exactly the same as the seller's description, very satisfied!	Jingdong prefecture level city in Shandong
Fortune Shop	Green Intelligent Power Saver	The saver works great, it really works, it's affordable and worth buying.	Jingdong prefecture level city in Shandong
Fortune Shop	Green Intelligent Power Saver	Just used it for a couple days. I don't know yet.	Jingdong prefecture level city in Shandong
Fortune Shop	Green Intelligent Power Saver	The quality is quite good, cheaper than the physical store, good, next time will come again!	Jingdong prefecture level city in Shandong
Fortune Shop	Green Intelligent Power Saver	Good quality, cheap and affordable worth recommending to buy!	Jingdong prefecture level city in Shandong
Fortune Shop	Green Intelligent Power Saver	Very good quality, well made, works well! Satisfied	Jingdong prefecture level city in Shandong
Fortune Shop	Green Intelligent Power Saver	The logistics is very fast quality is very good very pleasant a shopping	Jingdong prefecture level city in Shandong
Fortune Shop	Green Intelligent Power Saver	Use it and then come back to evaluate!	Jingdong prefecture level city in Shandong
Fortune Shop	Green Intelligent Power Saver	# It's no use lying at all #	Jingdong prefecture level city in Shandong
Fortune Shop	Green Intelligent Power Saver	I can't get a voltage drop.	Jingdong prefecture level city in Shandong
Fortune Shop	Green Intelligent Power Saver	Evaluate after use	Jingdong prefecture level city in Shandong
Fortune Shop	Green Intelligent Power Saver	Still on trial.	Jingdong prefecture level city in Shandong
Fortune Shop	Green Intelligent Power Saver	ineffective	Jingdong prefecture level city in Shandong
Fortune Shop	Green Intelligent Power Saver	Satisfaction Shopping	Jingdong prefecture level city in Shandong
Fortune Shop	Green Intelligent Power Saver	Lies.	Jingdong prefecture level city in Shandong
Fortune Shop	Green Intelligent Power Saver	It's a real power saver.	Jingdong prefecture level city in Shandong
Fortune Shop	Green Intelligent Power Saver	in use	Jingdong prefecture level city in Shandong
Fortune Shop	Green Intelligent Power Saver	I don't know yet.	Jingdong prefecture level city in Shandong
Fortune Shop	Green Intelligent Power Saver	not care about anything	Jingdong prefecture level city in Shandong
Fortune Shop	Green Intelligent Power Saver	It doesn't seem to have much effect	Jingdong prefecture level city in Shandong
Fortune Shop	Green Intelligent Power Saver	Let the merchant see for himself, does the plug positive and negative terminals look like this?	Jingdong prefecture level city in Shandong
Fortune Shop	Green Intelligent Power Saver	It doesn't work well, the electricity bill is less before using it, but more after using it, contacting customer service, the service is still not good, and returning it is slow	Jingdong prefecture level city in Shandong
Fortune Shop	Green Intelligent Power Saver	With the advertisement product does not match, above the effective negative, power saving rate of two rows of words it, the product looks very thin, with 3 days to see the meter number is still with the previous no difference, which is obviously fraudulent behavior!	Jingdong prefecture level city in Shandong
Fortune Shop	Green Intelligent Power Saver	Doesn't save any power at all. Not much use.	Jingdong prefecture level city in Shandong
Fortune Shop	Green Intelligent Power Saver	The plugs are all loose, they're all the same length and they don't work at all	Jingdong prefecture level city in Shandong
Fortune Shop	Green Intelligent Power Saver	It's bad 👎 it's not effective at all, you'd be crazy to sell this garbage on the internet.	Jingdong prefecture level city in Shandong
Fortune Shop	Green Intelligent Power Saver	It doesn't save any power at all, so I hope the old timers don't fall for this scam again	Jingdong prefecture level city in Shandong
Fortune Shop	Green Intelligent Power Saver	I've been using it for over a month now and haven't noticed any power savings.	Jingdong prefecture level city in Shandong
Fortune Shop	Green Intelligent Power Saver	It doesn't work at all. The electricity bill is higher when you use it.	Jingdong prefecture level city in Shandong
Fortune Shop	Green Intelligent Power Saver	It doesn't save any power, it's still as bad as ever.	Jingdong prefecture level city in Shandong
Fortune Shop	Green Intelligent Power Saver	Last night before leaving work to look at the table, and then plugged into the node device, this morning and then look at the table, than the original save a few degrees of electricity, very good to have a power-saving effect, easy to use, satisfied with the shopping praise.	Jingdong prefecture level city in Shandong
Fortune Shop	Green Intelligent Power Saver	The product is real and effective, the electricity bill is reduced, really good, thanks to the merchants have so many good products, the product is real and effective, the electricity bill is reduced, want to save electricity friends quickly buy it, early use early to save money.	Jingdong prefecture level city in Shandong
Fortune Shop	Green Intelligent Power Saver	Saver saves time and money, baby appearance is beautiful, the quality of goods is good, and the same as the business description, good service attitude, packaging is complete, logistics is very fast, praise to	Jingdong prefecture level city in Shandong
Fortune Shop	Green Intelligent Power Saver	The effect is good, summer, home electricity is relatively large, buy and use a few days, feel the effect is very good, originally ten dollars a day electricity bill, now eight dollars a day, quite good	Jingdong prefecture level city in Shandong

Fortune Shop	Green Intelligent Power Saver	Indeed can save electricity and money, the first time I know this or from a friend's home to see, but also do not believe it, and now they use it to know that there is this good thing.	Jingdong prefecture level city in Shandong
Fortune Shop	Green Intelligent Power Saver	I have received the Smart Power Saver. Perfectly packaged. Fast delivery. Boss customer service attitude is also very good. It's worth buying. I plugged it in and tried it and it really saves more power than the original. I'll buy it again next time.	Jingdong prefecture level city in Shandong
Fortune Shop	Green Intelligent Power Saver	It's nice to save a dozen bucks a night on two air conditioners that used to take 30 or 40 degrees a night, but now it's only 10 or so degrees a night	Jingdong prefecture level city in Shandong
Fortune Shop	Green Intelligent Power Saver	Product no manufacturer's name no product qualification certificate, try two days to see the effect of	Jingdong prefecture level city in Shandong
Fortune Shop	Green Intelligent Power Saver	I've been using it for over a month and have saved over $80, so I've repurchased a few more back to save together	Jingdong prefecture level city in Shandong
Fortune Shop	Green Intelligent Power Saver	I haven't used it yet and will comment when I do, but it's well made.	Jingdong prefecture level city in Shandong
Fortune Shop	Green Intelligent Power Saver	It's quite energy efficient. It's really a boon for the home.	Jingdong prefecture level city in Shandong
Fortune Shop	Green Intelligent Power Saver	Energy-saving socket received a month, after using it for a month proved that it is indeed energy-saving, electricity bill saving, will buy it again, thank you!	Jingdong prefecture level city in Shandong
Fortune Shop	Green Intelligent Power Saver	There was a similar product many years ago. Now technology has evolved, buy one to try, according to the instructions in the box, it takes one or two dozen days to show results. Check your electric bill and see what happens.	Jingdong prefecture level city in Shandong
Fortune Shop	Green Intelligent Power Saver	Jingdong logistics is really fast, the same day the order arrived on the same day, more convenient than the supermarket, and a wide range of choices, the	Jingdong prefecture level city in Shandong
Fortune Shop	Green Intelligent Power Saver	I've been using this for a month or so and I can't see any results, it really is a scam as they say.	Jingdong prefecture level city in Shandong
Fortune Shop	Green Intelligent Power Saver	Very good use, a month can save a few degrees of power loss of electricity, introduced to the relatives to buy back to try.	Jingdong prefecture level city in Shandong
Fortune Shop	Green Intelligent Power Saver	It's really powerful! With this air conditioner can be turned on at any time without the pressure of paying the electricity bill! It's good for the people!	Jingdong prefecture level city in Shandong
Fortune Shop	Green Intelligent Power Saver	This business is a big ** products are not qualified, with a month of electricity more than one hundred percent of thirty, the purchase agreed to return the goods but do not receive a refund after receiving the goods, looking for the official side does not help, the Jingdong is a big **, I have never shopped in	Jingdong prefecture level city in Shandong
Fortune Shop	Green Intelligent Power Saver	It's good. I tried it. It saves power.	Jingdong prefecture level city in Shandong
Fortune Shop	Green Intelligent Power Saver	Nothing**, it seems like there's more	Jingdong prefecture level city in Shandong
Fortune Shop	Green Intelligent Power Saver	Power saving device received, well packaged, good quality, fast logistics	Jingdong prefecture level city in Shandong
Fortune Shop	Green Intelligent Power Saver	The service is okay, is that when I bought five, the desirable pieces are three, and then wait a few days and send me two replacement, I do not know if it is a mistake of the shipper, if I was not in the courier there to see, go home to say no, the	Jingdong prefecture level city in Shandong
Fortune Shop	Green Intelligent Power Saver	This thing will only cost me more if I use it, but before I used it, it was less than a ~ electricity a month, and after I used it, it was more than two hundred kWh a month.	Jingdong prefecture level city in Shandong
Fortune Shop	Green Intelligent Power Saver	I just used it. I don't know how it is.	Jingdong prefecture level city in Shandong
Fortune Shop	Green Intelligent Power Saver	I've received the product and I'm using it, so I'll see how it goes in a month!	Jingdong prefecture level city in Shandong
Fortune Shop	Green Intelligent Power Saver	The goods have just received the effect do not know how, please look forward to the results.	Jingdong prefecture level city in Shandong
Fortune Shop	Green Intelligent Power Saver	Power saving device, the material is very good practical and durable, the size is suitable, packaging is very good, the price is cheap, logistics is very fast!	Jingdong prefecture level city in Shandong
Fortune Shop	Green Intelligent Power Saver	Very nice, will comment in detail after using it for a while.	Jingdong prefecture level city in Shandong
Fortune Shop	Green Intelligent Power Saver	Good stuff to trust and support	Jingdong prefecture level city in Shandong
Fortune Shop	Green Intelligent Power Saver	I'll comment after I use it, doubt if it really saves power no	Jingdong prefecture level city in Shandong
Fortune Shop	Green Intelligent Power Saver	It's good quality and works well!	Jingdong prefecture level city in Shandong
Fortune Shop	Green Intelligent Power Saver	hahaha just right hahahahahaha hiccup hahahaha	Jingdong prefecture level city in Shandong
Fortune Shop	Green Intelligent Power Saver	The real thing matches the picture and looks great!	Jingdong prefecture level city in Shandong
Fortune Shop	Green Intelligent Power Saver	Plugged it in and we'll see when we pay the bills at the end of the year!	Jingdong prefecture level city in Shandong
Fortune Shop	Green Intelligent Power Saver	False advertising. It doesn't work.	Jingdong prefecture level city in Shandong
Fortune Shop	Green Intelligent Power Saver	Goods received, good effect	Jingdong prefecture level city in Shandong
Fortune Shop	Green Intelligent Power Saver	It looks okay, we'll see if it saves electricity when the time comes	Jingdong prefecture level city in Shandong
Fortune Shop	Green Intelligent Power Saver	Logistics is very fast quality is very good, the price is very affordable, accessories are also complete, installed, satisfied with good use	Jingdong prefecture level city in Shandong
Fortune Shop	Green Intelligent Power Saver	I've received the power saver. I'm looking forward to the savings.	Jingdong prefecture level city in Shandong
Fortune Shop	Green Intelligent Power Saver	Received, have not yet used, after use and then evaluate	Jingdong prefecture level city in Shandong
Fortune Shop	Green Intelligent Power Saver	I received the item, but I don't know if it saves power or not.	Jingdong prefecture level city in Shandong
Fortune Shop	Green Intelligent Power Saver	On Trial	Jingdong prefecture level city in Shandong
Fortune Shop	Green Intelligent Power Saver	Use it and see how it goes.	Jingdong prefecture level city in Shandong
Fortune Shop	Green Intelligent Power Saver	Quality is very good, no odor, fine workmanship, grain color are very like, satisfied!	Jingdong prefecture level city in Shandong
Fortune Shop	Green Intelligent Power Saver	I'll give you a good review. I don't know if it'll work.	Jingdong prefecture level city in Shandong
Fortune Shop	Green Intelligent Power Saver	The logistics is very fast and the package is intact. Good use of affordable. The effect is very good, is genuine	Jingdong prefecture level city in Shandong
Fortune Shop	Green Intelligent Power Saver	Nothing happens when you plug it in.	Jingdong prefecture level city in Shandong
Fortune Shop	Green Intelligent Power Saver	I don't know if it's as good as it sounds.	Jingdong prefecture level city in Shandong
Fortune Shop	Green Intelligent Power Saver	The quality of the stuff is very good, exactly the same as the seller's description, very satisfied!	Jingdong prefecture level city in Shandong
Fortune Shop	Green Intelligent Power Saver	The saver works great, it really works, it's affordable and worth buying.	Jingdong prefecture level city in Shandong
Fortune Shop	Green Intelligent Power Saver	Just used it for a couple days. I don't know yet.	Jingdong prefecture level city in Shandong
Fortune Shop	Green Intelligent Power Saver	The quality is quite good, cheaper than the physical store, good, next time will come again!	Jingdong prefecture level city in Shandong
Fortune Shop	Green Intelligent Power Saver	Good quality, cheap and affordable worth recommending to buy!	Jingdong prefecture level city in Shandong

Fortune Shop	Green Intelligent Power Saver	Very good quality, well made, works well! Satisfied	Jingdong prefecture level city in Shandong
Fortune Shop	Green Intelligent Power Saver	The logistics is very fast quality is very good very pleasant a shopping	Jingdong prefecture level city in Shandong
Fortune Shop	Green Intelligent Power Saver	Use it and then come back to evaluate!	Jingdong prefecture level city in Shandong
Fortune Shop	Green Intelligent Power Saver	# It's no use lying at all #	Jingdong prefecture level city in Shandong
Fortune Shop	Green Intelligent Power Saver	I can't get a voltage drop.	Jingdong prefecture level city in Shandong
Fortune Shop	Green Intelligent Power Saver	Evaluate after use	Jingdong prefecture level city in Shandong
Fortune Shop	Green Intelligent Power Saver	Still on trial.	Jingdong prefecture level city in Shandong
Fortune Shop	Green Intelligent Power Saver	ineffective	Jingdong prefecture level city in Shandong
Fortune Shop	Green Intelligent Power Saver	Satisfaction Shopping	Jingdong prefecture level city in Shandong
Fortune Shop	Green Intelligent Power Saver	Lies.	Jingdong prefecture level city in Shandong
Fortune Shop	Green Intelligent Power Saver	It's a real power saver.	Jingdong prefecture level city in Shandong
Fortune Shop	Green Intelligent Power Saver	in use	Jingdong prefecture level city in Shandong
Fortune Shop	Green Intelligent Power Saver	I don't know yet.	Jingdong prefecture level city in Shandong
Fortune Shop	Green Intelligent Power Saver	not care about anything	Jingdong prefecture level city in Shandong
Fortune Shop	Green Intelligent Power Saver	It doesn't seem to have much effect	Jingdong prefecture level city in Shandong
Fortune Shop	Green Intelligent Power Saver	Let the merchant see for himself, does the plug positive and negative terminals look like this?	Jingdong prefecture level city in Shandong
Fortune Shop	Green Intelligent Power Saver	It doesn't work well, the electricity bill is less before using it, but more after using it, contacting customer service, the service is still not good, and returning it is slow	Jingdong prefecture level city in Shandong
Fortune Shop	Green Intelligent Power Saver	With the advertisement product does not match, above the effective negative, power saving rate of two rows of words it, the product looks very thin, with 3 days to see the meter number is still with the previous no difference, which is obviously fraudulent behavior!	Jingdong prefecture level city in Shandong
Fortune Shop	Green Intelligent Power Saver	Doesn't save any power at all. Not much use.	Jingdong prefecture level city in Shandong
Fortune Shop	Green Intelligent Power Saver	The plugs are all loose, they're all the same length and they don't work at all	Jingdong prefecture level city in Shandong
Fortune Shop	Green Intelligent Power Saver	It's bad 👎 it's not effective at all, you'd be crazy to sell this garbage on the internet.	Jingdong prefecture level city in Shandong
Fortune Shop	Green Intelligent Power Saver	It doesn't save any power at all, so I hope the old timers don't fall for this scam again	Jingdong prefecture level city in Shandong
Fortune Shop	Green Intelligent Power Saver	I've been using it for over a month now and haven't noticed any power savings.	Jingdong prefecture level city in Shandong
Fortune Shop	Green Intelligent Power Saver	It doesn't work at all. The electricity bill is higher when you use it.	Jingdong prefecture level city in Shandong
Fortune Shop	Green Intelligent Power Saver	It doesn't save any power, it's still as bad as ever.	Jingdong prefecture level city in Shandong
Fortune Shop	Green Intelligent Power Saver	Last night before leaving work to look at the table, and then plugged into the node device, this morning and then look at the table, than the original save a few degrees of electricity, very good to have a power-saving effect, easy to use, satisfied with the shopping praise.	Jingdong prefecture level city in Shandong
Fortune Shop	Green Intelligent Power Saver	The product is real and effective, the electricity bill is reduced, really good, thanks to the merchants have so many good products, the product is real and effective, the electricity bill is reduced, want to save electricity friends quickly buy it, early use early to save money.	Jingdong prefecture level city in Shandong
Fortune Shop	Green Intelligent Power Saver	Saver saves time and money, baby appearance is beautiful, the quality of goods is good, and the same as the business description, good service attitude, packaging is complete, logistics is very fast, praise to	Jingdong prefecture level city in Shandong
Fortune Shop	Green Intelligent Power Saver	The effect is good, summer, home electricity is relatively large, buy and use a few days, feel the effect is very good, originally ten dollars a day electricity bill, now eight dollars a day, quite good	Jingdong prefecture level city in Shandong
Fortune Shop	Green Intelligent Power Saver	Indeed can save electricity and money, the first time I know this or from a friend's home to see, but also do not believe it, and now they use it to know that there is this good thing.	Jingdong prefecture level city in Shandong
Fortune Shop	Green Intelligent Power Saver	I have received the Smart Power Saver. Perfectly packaged. Fast delivery. Boss customer service attitude is also very good. It's worth buying. I plugged it in and tried it and it really saves more power than the original. I'll buy it again next time.	Jingdong prefecture level city in Shandong
Fortune Shop	Green Intelligent Power Saver	It's nice to save a dozen bucks a night on two air conditioners that used to take 30 or 40 degrees a night, but now it's only 10 or so degrees a night	Jingdong prefecture level city in Shandong
Fortune Shop	Green Intelligent Power Saver	Product no manufacturer's name no product qualification certificate, try two days to see the effect of	Jingdong prefecture level city in Shandong
Fortune Shop	Green Intelligent Power Saver	I've been using it for over a month and have saved over $80, so I've repurchased a few more back to save together	Jingdong prefecture level city in Shandong
Fortune Shop	Green Intelligent Power Saver	I haven't used it yet and will comment when I do, but it's well made.	Jingdong prefecture level city in Shandong
Fortune Shop	Green Intelligent Power Saver	It's quite energy efficient. It's really a boon for the home.	Jingdong prefecture level city in Shandong
Fortune Shop	Green Intelligent Power Saver	Energy-saving socket received a month, after using it for a month proved that it is indeed energy-saving, electricity bill saving, will buy it again, thank you!	Jingdong prefecture level city in Shandong
Fortune Shop	Green Intelligent Power Saver	There was a similar product many years ago. Now technology has evolved, buy one to try, according to the instructions in the box, it takes one or two dozen days to show results. Check your electric bill and see what happens.	Jingdong prefecture level city in Shandong
Fortune Shop	Green Intelligent Power Saver	Jingdong logistics is really fast, the same day the order arrived on the same day, more convenient than the supermarket, and a wide range of choices, the	Jingdong prefecture level city in Shandong
Fortune Shop	Green Intelligent Power Saver	I've been using this for a month or so and I can't see any results, it really is a scam as they say.	Jingdong prefecture level city in Shandong
Fortune Shop	Green Intelligent Power Saver	Very good use, a month can save a few degrees of power loss of electricity, introduced to the relatives to buy back to try.	Jingdong prefecture level city in Shandong
Fortune Shop	Green Intelligent Power Saver	It's really powerful! With this air conditioner can be turned on at any time without the pressure of paying the electricity bill! It's good for the people!	Jingdong prefecture level city in Shandong
Fortune Shop	Green Intelligent Power Saver	This business is a big ** products are not qualified, with a month of electricity more than one hundred percent of thirty, the purchase agreed to return the goods but do not receive a refund after receiving the goods, looking for the official side does not help, the Jingdong is a big **, I have never shopped in	Jingdong prefecture level city in Shandong
Fortune Shop	Green Intelligent Power Saver	It's good. I tried it. It saves power.	Jingdong prefecture level city in Shandong
Fortune Shop	Green Intelligent Power Saver	Nothing**, it seems like there's more	Jingdong prefecture level city in Shandong
Fortune Shop	Green Intelligent Power Saver	Power saving device received, well packaged, good quality, fast logistics	Jingdong prefecture level city in Shandong
Fortune Shop	Green Intelligent Power Saver	The service is okay, is that when I bought five, the desirable pieces are three, and then wait a few days and send me two replacement, I do not know if it is a mistake of the shipper, if I was not in the courier there to see, go home to say no, the	Jingdong prefecture level city in Shandong
Fortune Shop	Green Intelligent Power Saver	This thing will only cost me more if I use it, but before I used it, it was less than a ~ electricity a month, and after I used it, it was more than two hundred kWh a month.	Jingdong prefecture level city in Shandong

Fortune Shop	Green Intelligent Power Saver	I just used it. I don't know how it is.	Jingdong prefecture level city in Shandong
Fortune Shop	Green Intelligent Power Saver	I've received the product and I'm using it, so I'll see how it goes in a month!	Jingdong prefecture level city in Shandong
Fortune Shop	Green Intelligent Power Saver	The goods have just received the effect do not know how, please look forward to the results.	Jingdong prefecture level city in Shandong
Fortune Shop	Green Intelligent Power Saver	Power saving device, the material is very good practical and durable, the size is suitable, packaging is very good, the price is cheap, logistics is very fast!	Jingdong prefecture level city in Shandong
Fortune Shop	Green Intelligent Power Saver	Very nice, will comment in detail after using it for a while.	Jingdong prefecture level city in Shandong
Fortune Shop	Green Intelligent Power Saver	Good stuff to trust and support	Jingdong prefecture level city in Shandong
Fortune Shop	Green Intelligent Power Saver	I'll comment after I use it, doubt if it really saves power no	Jingdong prefecture level city in Shandong
Fortune Shop	Green Intelligent Power Saver	It's good quality and works well!	Jingdong prefecture level city in Shandong
Fortune Shop	Green Intelligent Power Saver	hahaha just right hahahahahaha hiccup hahahaha	Jingdong prefecture level city in Shandong
Fortune Shop	Green Intelligent Power Saver	The real thing matches the picture and looks great!	Jingdong prefecture level city in Shandong
Fortune Shop	Green Intelligent Power Saver	Plugged it in and we'll see when we pay the bills at the end of the year!	Jingdong prefecture level city in Shandong
Fortune Shop	Green Intelligent Power Saver	False advertising. It doesn't work.	Jingdong prefecture level city in Shandong
Fortune Shop	Green Intelligent Power Saver	Goods received, good effect	Jingdong prefecture level city in Shandong
Fortune Shop	Green Intelligent Power Saver	It looks okay, we'll see if it saves electricity when the time comes	Jingdong prefecture level city in Shandong
Fortune Shop	Green Intelligent Power Saver	Logistics is very fast quality is very good, the price is very affordable, accessories are also complete, installed, satisfied with good use	Jingdong prefecture level city in Shandong
Fortune Shop	Green Intelligent Power Saver	I've received the power saver. I'm looking forward to the savings.	Jingdong prefecture level city in Shandong
Fortune Shop	Green Intelligent Power Saver	Received, have not yet used, after use and then evaluate	Jingdong prefecture level city in Shandong
Fortune Shop	Green Intelligent Power Saver	I received the item, but I don't know if it saves power or not.	Jingdong prefecture level city in Shandong
Fortune Shop	Green Intelligent Power Saver	On Trial	Jingdong prefecture level city in Shandong
Fortune Shop	Green Intelligent Power Saver	Use it and see how it goes.	Jingdong prefecture level city in Shandong
Fortune Shop	Green Intelligent Power Saver	Quality is very good, no odor, fine workmanship, grain color are very like, satisfied!	Jingdong prefecture level city in Shandong
Fortune Shop	Green Intelligent Power Saver	I'll give you a good review. I don't know if it'll work.	Jingdong prefecture level city in Shandong
Fortune Shop	Green Intelligent Power Saver	The logistics is very fast and the package is intact. Good use of affordable. The effect is very good, is genuine	Jingdong prefecture level city in Shandong
Fortune Shop	Green Intelligent Power Saver	Nothing happens when you plug it in.	Jingdong prefecture level city in Shandong
Fortune Shop	Green Intelligent Power Saver	I don't know if it's as good as it sounds.	Jingdong prefecture level city in Shandong
Fortune Shop	Green Intelligent Power Saver	The quality of the stuff is very good, exactly the same as the seller's description, very satisfied!	Jingdong prefecture level city in Shandong
Fortune Shop	Green Intelligent Power Saver	The saver works great, it really works, it's affordable and worth buying.	Jingdong prefecture level city in Shandong
Fortune Shop	Green Intelligent Power Saver	Just used it for a couple days. I don't know yet.	Jingdong prefecture level city in Shandong
Fortune Shop	Green Intelligent Power Saver	The quality is quite good, cheaper than the physical store, good, next time will come again!	Jingdong prefecture level city in Shandong
Fortune Shop	Green Intelligent Power Saver	Good quality, cheap and affordable worth recommending to buy!	Jingdong prefecture level city in Shandong
Fortune Shop	Green Intelligent Power Saver	Very good quality, well made, works well! Satisfied	Jingdong prefecture level city in Shandong
Fortune Shop	Green Intelligent Power Saver	The logistics is very fast quality is very good very pleasant a shopping	Jingdong prefecture level city in Shandong
Fortune Shop	Green Intelligent Power Saver	Use it and then come back to evaluate!	Jingdong prefecture level city in Shandong
Fortune Shop	Green Intelligent Power Saver	# It's no use lying at all #	Jingdong prefecture level city in Shandong
Fortune Shop	Green Intelligent Power Saver	I can't get a voltage drop.	Jingdong prefecture level city in Shandong
Fortune Shop	Green Intelligent Power Saver	Evaluate after use	Jingdong prefecture level city in Shandong
Fortune Shop	Green Intelligent Power Saver	Still on trial.	Jingdong prefecture level city in Shandong
Fortune Shop	Green Intelligent Power Saver	ineffective	Jingdong prefecture level city in Shandong
Fortune Shop	Green Intelligent Power Saver	Satisfaction Shopping	Jingdong prefecture level city in Shandong
Fortune Shop	Green Intelligent Power Saver	Lies.	Jingdong prefecture level city in Shandong
Fortune Shop	Green Intelligent Power Saver	It's a real power saver.	Jingdong prefecture level city in Shandong
Fortune Shop	Green Intelligent Power Saver	in use	Jingdong prefecture level city in Shandong
Fortune Shop	Green Intelligent Power Saver	I don't know yet.	Jingdong prefecture level city in Shandong
Fortune Shop	Green Intelligent Power Saver	not care about anything	Jingdong prefecture level city in Shandong
Fortune Shop	Green Intelligent Power Saver	It doesn't seem to have much effect	Jingdong prefecture level city in Shandong
Fortune Shop	Green Intelligent Power Saver	Let the merchant see for himself, does the plug positive and negative terminals look like this?	Jingdong prefecture level city in Shandong
Fortune Shop	Green Intelligent Power Saver	It doesn't work well, the electricity bill is less before using it, but more after using it, contacting customer service, the service is still not good, and returning it is slow	Jingdong prefecture level city in Shandong
Fortune Shop	Green Intelligent Power Saver	With the advertisement product does not match, above the effective negative, power saving rate of two rows of words it, the product looks very thin, with 3 days to see the meter number is still with the previous no difference, which is obviously fraudulent behavior!	Jingdong prefecture level city in Shandong
Fortune Shop	Green Intelligent Power Saver	Doesn't save any power at all. Not much use.	Jingdong prefecture level city in Shandong
Fortune Shop	Green Intelligent Power Saver	The plugs are all loose, they're all the same length and they don't work at all	Jingdong prefecture level city in Shandong
Fortune Shop	Green Intelligent Power Saver	It's bad 👎 it's not effective at all, you'd be crazy to sell this garbage on the internet.	Jingdong prefecture level city in Shandong

Fortune Shop	Green Intelligent Power Saver	It doesn't save any power at all, so I hope the old timers don't fall for this scam again	Jingdong prefecture level city in Shandong
Fortune Shop	Green Intelligent Power Saver	I've been using it for over a month now and haven't noticed any power savings.	Jingdong prefecture level city in Shandong
Fortune Shop	Green Intelligent Power Saver	It doesn't work at all. The electricity bill is higher when you use it.	Jingdong prefecture level city in Shandong
Fortune Shop	Green Intelligent Power Saver	It doesn't save any power, it's still as bad as ever.	Jingdong prefecture level city in Shandong
Fortune Shop	Green Intelligent Power Saver	Last night before leaving work to look at the table, and then plugged into the node device, this morning and then look at the table, than the original save a few degrees of electricity, very good to have a power-saving effect, easy to use, satisfied with the shopping praise.	Jingdong prefecture level city in Shandong
Fortune Shop	Green Intelligent Power Saver	The product is real and effective, the electricity bill is reduced, really good, thanks to the merchants have so many good products, the product is real and effective, the electricity bill is reduced, want to save electricity friends quickly buy it, early use early to save money.	Jingdong prefecture level city in Shandong
Fortune Shop	Green Intelligent Power Saver	Saver saves time and money, baby appearance is beautiful, the quality of goods is good, and the same as the business description, good service attitude, packaging is complete, logistics is very fast, praise to	Jingdong prefecture level city in Shandong
Fortune Shop	Green Intelligent Power Saver	The effect is good, summer, home electricity is relatively large, buy and use a few days, feel the effect is very good, originally ten dollars a day electricity bill, now eight dollars a day, quite good	Jingdong prefecture level city in Shandong
Fortune Shop	Green Intelligent Power Saver	Indeed can save electricity and money, the first time I know this or from a friend's home to see, but also do not believe it, and now they use it to know that there is this good thing.	Jingdong prefecture level city in Shandong
Fortune Shop	Green Intelligent Power Saver	I have received the Smart Power Saver. Perfectly packaged. Fast delivery. Boss customer service attitude is also very good. It's worth buying. I plugged it in and tried it and it really saves more power than the original. I'll buy it again next time.	Jingdong prefecture level city in Shandong
Fortune Shop	Green Intelligent Power Saver	It's nice to save a dozen bucks a night on two air conditioners that used to take 30 or 40 degrees a night, but now it's only 10 or so degrees a night	Jingdong prefecture level city in Shandong
Fortune Shop	Green Intelligent Power Saver	Product no manufacturer's name no product qualification certificate, try two days to see the effect of	Jingdong prefecture level city in Shandong
Fortune Shop	Green Intelligent Power Saver	I've been using it for over a month and have saved over $80, so I've repurchased a few more back to save together	Jingdong prefecture level city in Shandong
Fortune Shop	Green Intelligent Power Saver	I haven't used it yet and will comment when I do, but it's well made.	Jingdong prefecture level city in Shandong
Fortune Shop	Green Intelligent Power Saver	It's quite energy efficient. It's really a boon for the home.	Jingdong prefecture level city in Shandong
Fortune Shop	Green Intelligent Power Saver	Energy-saving socket received a month, after using it for a month proved that it is indeed energy-saving, electricity bill saving, will buy it again, thank you!	Jingdong prefecture level city in Shandong
Fortune Shop	Green Intelligent Power Saver	There was a similar product many years ago. Now technology has evolved, buy one to try, according to the instructions in the box, it takes one or two dozen days to show results. Check your electric bill and see what happens.	Jingdong prefecture level city in Shandong
Fortune Shop	Green Intelligent Power Saver	Jingdong logistics is really fast, the same day the order arrived on the same day, more convenient than the supermarket, and a wide range of choices, the	Jingdong prefecture level city in Shandong
Fortune Shop	Green Intelligent Power Saver	I've been using this for a month or so and I can't see any results, it really is a scam as they say.	Jingdong prefecture level city in Shandong
Fortune Shop	Green Intelligent Power Saver	Very good use, a month can save a few degrees of power loss of electricity, introduced to the relatives to buy back to try.	Jingdong prefecture level city in Shandong
Fortune Shop	Green Intelligent Power Saver	It's really powerful! With this air conditioner can be turned on at any time without the pressure of paying the electricity bill! It's good for the people!	Jingdong prefecture level city in Shandong
Fortune Shop	Green Intelligent Power Saver	This business is a big ** products are not qualified, with a month of electricity more than one hundred percent of thirty, the purchase agreed to return the goods but do not receive a refund after receiving the goods, looking for the official side does not help, the Jingdong is a big **, I have never shopped in	Jingdong prefecture level city in Shandong
Fortune Shop	Green Intelligent Power Saver	It's good. I tried it. It saves power.	Jingdong prefecture level city in Shandong
Fortune Shop	Green Intelligent Power Saver	Nothing**, it seems like there's more	Jingdong prefecture level city in Shandong
Fortune Shop	Green Intelligent Power Saver	Power saving device received, well packaged, good quality, fast logistics	Jingdong prefecture level city in Shandong
Fortune Shop	Green Intelligent Power Saver	The service is okay, is that when I bought five, the desirable pieces are three, and then wait a few days and send me two replacement, I do not know if it is a mistake of the shipper, if I was not in the courier there to see, go home to say no, the	Jingdong prefecture level city in Shandong
Fortune Shop	Green Intelligent Power Saver	This thing will only cost me more if I use it, but before I used it, it was less than a ~ electricity a month, and after I used it, it was more than two hundred kWh a month.	Jingdong prefecture level city in Shandong
Fortune Shop	Green Intelligent Power Saver	I just used it. I don't know how it is.	Jingdong prefecture level city in Shandong
Fortune Shop	Green Intelligent Power Saver	I've received the product and I'm using it, so I'll see how it goes in a month!	Jingdong prefecture level city in Shandong
Fortune Shop	Green Intelligent Power Saver	The goods have just received the effect do not know how, please look forward to the results.	Jingdong prefecture level city in Shandong
Fortune Shop	Green Intelligent Power Saver	Power saving device, the material is very good practical and durable, the size is suitable, packaging is very good, the price is cheap, logistics is very fast!	Jingdong prefecture level city in Shandong
Fortune Shop	Green Intelligent Power Saver	Very nice, will comment in detail after using it for a while.	Jingdong prefecture level city in Shandong
Fortune Shop	Green Intelligent Power Saver	Good stuff to trust and support	Jingdong prefecture level city in Shandong
Fortune Shop	Green Intelligent Power Saver	I'll comment after I use it, doubt if it really saves power no	Jingdong prefecture level city in Shandong
Fortune Shop	Green Intelligent Power Saver	It's good quality and works well!	Jingdong prefecture level city in Shandong
Fortune Shop	Green Intelligent Power Saver	hahaha just right hahahahahaha hiccup hahahaha	Jingdong prefecture level city in Shandong
Fortune Shop	Green Intelligent Power Saver	The real thing matches the picture and looks great!	Jingdong prefecture level city in Shandong
Fortune Shop	Green Intelligent Power Saver	Plugged it in and we'll see when we pay the bills at the end of the year!	Jingdong prefecture level city in Shandong
Fortune Shop	Green Intelligent Power Saver	False advertising. It doesn't work.	Jingdong prefecture level city in Shandong
Fortune Shop	Green Intelligent Power Saver	Goods received, good effect	Jingdong prefecture level city in Shandong
Fortune Shop	Green Intelligent Power Saver	It looks okay, we'll see if it saves electricity when the time comes	Jingdong prefecture level city in Shandong
Fortune Shop	Green Intelligent Power Saver	Logistics is very fast quality is very good, the price is very affordable, accessories are also complete, installed, satisfied with good use	Jingdong prefecture level city in Shandong
Fortune Shop	Green Intelligent Power Saver	I've received the power saver. I'm looking forward to the savings.	Jingdong prefecture level city in Shandong
Fortune Shop	Green Intelligent Power Saver	Received, have not yet used, after use and then evaluate	Jingdong prefecture level city in Shandong
Fortune Shop	Green Intelligent Power Saver	I received the item, but I don't know if it saves power or not.	Jingdong prefecture level city in Shandong
Fortune Shop	Green Intelligent Power Saver	On Trial	Jingdong prefecture level city in Shandong
Fortune Shop	Green Intelligent Power Saver	Use it and see how it goes.	Jingdong prefecture level city in Shandong
Fortune Shop	Green Intelligent Power Saver	Quality is very good, no odor, fine workmanship, grain color are very like, satisfied!	Jingdong prefecture level city in Shandong

Fortune Shop	Green Intelligent Power Saver	I'll give you a good review. I don't know if it'll work.	Jingdong prefecture level city in Shandong
Fortune Shop	Green Intelligent Power Saver	The logistics is very fast and the package is intact. Good use of affordable. The effect is very good, is genuine	Jingdong prefecture level city in Shandong
Fortune Shop	Green Intelligent Power Saver	Nothing happens when you plug it in.	Jingdong prefecture level city in Shandong
Fortune Shop	Green Intelligent Power Saver	I don't know if it's as good as it sounds.	Jingdong prefecture level city in Shandong
Fortune Shop	Green Intelligent Power Saver	The quality of the stuff is very good, exactly the same as the seller's description, very satisfied!	Jingdong prefecture level city in Shandong
Fortune Shop	Green Intelligent Power Saver	The saver works great, it really works, it's affordable and worth buying.	Jingdong prefecture level city in Shandong
Fortune Shop	Green Intelligent Power Saver	Just used it for a couple days. I don't know yet.	Jingdong prefecture level city in Shandong
Fortune Shop	Green Intelligent Power Saver	The quality is quite good, cheaper than the physical store, good, next time will come again!	Jingdong prefecture level city in Shandong
Fortune Shop	Green Intelligent Power Saver	Good quality, cheap and affordable worth recommending to buy!	Jingdong prefecture level city in Shandong
Fortune Shop	Green Intelligent Power Saver	Very good quality, well made, works well! Satisfied	Jingdong prefecture level city in Shandong
Fortune Shop	Green Intelligent Power Saver	The logistics is very fast quality is very good very pleasant a shopping	Jingdong prefecture level city in Shandong
Fortune Shop	Green Intelligent Power Saver	Use it and then come back to evaluate!	Jingdong prefecture level city in Shandong
Fortune Shop	Green Intelligent Power Saver	# It's no use lying at all #	Jingdong prefecture level city in Shandong
Fortune Shop	Green Intelligent Power Saver	I can't get a voltage drop.	Jingdong prefecture level city in Shandong
Fortune Shop	Green Intelligent Power Saver	Evaluate after use	Jingdong prefecture level city in Shandong
Fortune Shop	Green Intelligent Power Saver	Still on trial.	Jingdong prefecture level city in Shandong
Fortune Shop	Green Intelligent Power Saver	ineffective	Jingdong prefecture level city in Shandong
Fortune Shop	Green Intelligent Power Saver	Satisfaction Shopping	Jingdong prefecture level city in Shandong
Fortune Shop	Green Intelligent Power Saver	Lies.	Jingdong prefecture level city in Shandong
Fortune Shop	Green Intelligent Power Saver	It's a real power saver.	Jingdong prefecture level city in Shandong
Fortune Shop	Green Intelligent Power Saver	in use	Jingdong prefecture level city in Shandong
Fortune Shop	Green Intelligent Power Saver	I don't know yet.	Jingdong prefecture level city in Shandong
Fortune Shop	Green Intelligent Power Saver	not care about anything	Jingdong prefecture level city in Shandong
Fortune Shop	Green Intelligent Power Saver	It doesn't seem to have much effect	Jingdong prefecture level city in Shandong
Fortune Shop	Green Intelligent Power Saver	Let the merchant see for himself, does the plug positive and negative terminals look like this?	Jingdong prefecture level city in Shandong
Fortune Shop	Green Intelligent Power Saver	It doesn't work well, the electricity bill is less before using it, but more after using it, contacting customer service, the service is still not good, and returning it is slow	Jingdong prefecture level city in Shandong
Fortune Shop	Green Intelligent Power Saver	With the advertisement product does not match, above the effective negative, power saving rate of two rows of words it, the product looks very thin, with 3 days to see the meter number is still with the previous no difference, which is obviously fraudulent behavior!	Jingdong prefecture level city in Shandong
Fortune Shop	Green Intelligent Power Saver	Doesn't save any power at all. Not much use.	Jingdong prefecture level city in Shandong
Fortune Shop	Green Intelligent Power Saver	The plugs are all loose, they're all the same length and they don't work at all	Jingdong prefecture level city in Shandong
Fortune Shop	Green Intelligent Power Saver	It's bad 👎 it's not effective at all, you'd be crazy to sell this garbage on the internet.	Jingdong prefecture level city in Shandong
Fortune Shop	Green Intelligent Power Saver	It doesn't save any power at all, so I hope the old timers don't fall for this scam again	Jingdong prefecture level city in Shandong
Fortune Shop	Green Intelligent Power Saver	I've been using it for over a month now and haven't noticed any power savings.	Jingdong prefecture level city in Shandong
Fortune Shop	Green Intelligent Power Saver	It doesn't work at all. The electricity bill is higher when you use it.	Jingdong prefecture level city in Shandong
Fortune Shop	Green Intelligent Power Saver	It doesn't save any power, it's still as bad as ever.	Jingdong prefecture level city in Shandong
Fortune Shop	Green Intelligent Power Saver	Last night before leaving work to look at the table, and then plugged into the node device, this morning and then look at the table, than the original save a few degrees of electricity, very good to have a power-saving effect, easy to use, satisfied with the shopping praise.	Jingdong prefecture level city in Shandong
Fortune Shop	Green Intelligent Power Saver	The product is real and effective, the electricity bill is reduced, really good, thanks to the merchants have so many good products, the product is real and effective, the electricity bill is reduced, want to save electricity friends quickly buy it, early use early to save money.	Jingdong prefecture level city in Shandong
Fortune Shop	Green Intelligent Power Saver	Saver saves time and money, baby appearance is beautiful, the quality of goods is good, and the same as the business description, good service attitude, packaging is complete, logistics is very fast, praise to	Jingdong prefecture level city in Shandong
Fortune Shop	Green Intelligent Power Saver	The effect is good, summer, home electricity is relatively large, buy and use a few days, feel the effect is very good, originally ten dollars a day electricity bill, now eight dollars a day, quite good	Jingdong prefecture level city in Shandong
Fortune Shop	Green Intelligent Power Saver	Indeed can save electricity and money, the first time I know this or from a friend's home to see, but also do not believe it, and now they use it to know that there is this good thing.	Jingdong prefecture level city in Shandong
Fortune Shop	Green Intelligent Power Saver	I have received the Smart Power Saver. Perfectly packaged. Fast delivery. Boss customer service attitude is also very good. It's worth buying. I plugged it in and tried it and it really saves more power than the original. I'll buy it again next time.	Jingdong prefecture level city in Shandong
Fortune Shop	Green Intelligent Power Saver	It's nice to save a dozen bucks a night on two air conditioners that used to take 30 or 40 degrees a night, but now it's only 10 or so degrees a night	Jingdong prefecture level city in Shandong
Fortune Shop	Green Intelligent Power Saver	Product no manufacturer's name no product qualification certificate, try two days to see the effect of	Jingdong prefecture level city in Shandong
Fortune Shop	Green Intelligent Power Saver	I've been using it for over a month and have saved over $80, so I've repurchased a few more back to save together	Jingdong prefecture level city in Shandong
Fortune Shop	Green Intelligent Power Saver	I haven't used it yet and will comment when I do, but it's well made.	Jingdong prefecture level city in Shandong
Fortune Shop	Green Intelligent Power Saver	It's quite energy efficient. It's really a boon for the home.	Jingdong prefecture level city in Shandong
Fortune Shop	Green Intelligent Power Saver	Energy-saving socket received a month, after using it for a month proved that it is indeed energy-saving, electricity bill saving, will buy it again, thank you!	Jingdong prefecture level city in Shandong
Fortune Shop	Green Intelligent Power Saver	There was a similar product many years ago. Now technology has evolved, buy one to try, according to the instructions in the box, it takes one or two dozen days to show results. Check your electric bill and see what happens.	Jingdong prefecture level city in Shandong
Fortune Shop	Green Intelligent Power Saver	Jingdong logistics is really fast, the same day the order arrived on the same day, more convenient than the supermarket, and a wide range of choices, the	Jingdong prefecture level city in Shandong
Fortune Shop	Green Intelligent Power Saver	I've been using this for a month or so and I can't see any results, it really is a scam as they say.	Jingdong prefecture level city in Shandong

Fortune Shop	Green Intelligent Power Saver	Very good use, a month can save a few degrees of power loss of electricity, introduced to the relatives to buy back to try.	Jingdong prefecture level city in Shandong
Fortune Shop	Green Intelligent Power Saver	It's really powerful! With this air conditioner can be turned on at any time without the pressure of paying the electricity bill! It's good for the people!	Jingdong prefecture level city in Shandong
Fortune Shop	Green Intelligent Power Saver	This business is a big ** products are not qualified, with a month of electricity more than one hundred percent of thirty, the purchase agreed to return the goods but do not receive a refund after receiving the goods, looking for the official side does not help, the Jingdong is a big **, I have never shopped in	Jingdong prefecture level city in Shandong
Fortune Shop	Green Intelligent Power Saver	It's good. I tried it. It saves power.	Jingdong prefecture level city in Shandong
Fortune Shop	Green Intelligent Power Saver	Nothing**, it seems like there's more	Jingdong prefecture level city in Shandong
Fortune Shop	Green Intelligent Power Saver	Power saving device received, well packaged, good quality, fast logistics	Jingdong prefecture level city in Shandong
Fortune Shop	Green Intelligent Power Saver	The service is okay, is that when I bought five, the desirable pieces are three, and then wait a few days and send me two replacement, I do not know if it is a mistake of the shipper, if I was not in the courier there to see, go home to say no, the	Jingdong prefecture level city in Shandong
Fortune Shop	Green Intelligent Power Saver	This thing will only cost me more if I use it, but before I used it, it was less than a ~ electricity a month, and after I used it, it was more than two hundred kWh a month.	Jingdong prefecture level city in Shandong
Fortune Shop	Green Intelligent Power Saver	I just used it. I don't know how it is.	Jingdong prefecture level city in Shandong
Fortune Shop	Green Intelligent Power Saver	I've received the product and I'm using it, so I'll see how it goes in a month!	Jingdong prefecture level city in Shandong
Fortune Shop	Green Intelligent Power Saver	The goods have just received the effect do not know how, please look forward to the results.	Jingdong prefecture level city in Shandong
Fortune Shop	Green Intelligent Power Saver	Power saving device, the material is very good practical and durable, the size is suitable, packaging is very good, the price is cheap, logistics is very fast!	Jingdong prefecture level city in Shandong
Fortune Shop	Green Intelligent Power Saver	Very nice, will comment in detail after using it for a while.	Jingdong prefecture level city in Shandong
Fortune Shop	Green Intelligent Power Saver	Good stuff to trust and support	Jingdong prefecture level city in Shandong
Fortune Shop	Green Intelligent Power Saver	I'll comment after I use it, doubt if it really saves power no	Jingdong prefecture level city in Shandong
Fortune Shop	Green Intelligent Power Saver	It's good quality and works well!	Jingdong prefecture level city in Shandong
Fortune Shop	Green Intelligent Power Saver	hahaha just right hahahahahaha hiccup hahahaha	Jingdong prefecture level city in Shandong
Fortune Shop	Green Intelligent Power Saver	The real thing matches the picture and looks great!	Jingdong prefecture level city in Shandong
Fortune Shop	Green Intelligent Power Saver	Plugged it in and we'll see when we pay the bills at the end of the year!	Jingdong prefecture level city in Shandong
Fortune Shop	Green Intelligent Power Saver	False advertising. It doesn't work.	Jingdong prefecture level city in Shandong
Fortune Shop	Green Intelligent Power Saver	Goods received, good effect	Jingdong prefecture level city in Shandong
Fortune Shop	Green Intelligent Power Saver	It looks okay, we'll see if it saves electricity when the time comes	Jingdong prefecture level city in Shandong
Fortune Shop	Green Intelligent Power Saver	Logistics is very fast quality is very good, the price is very affordable, accessories are also complete, installed, satisfied with good use	Jingdong prefecture level city in Shandong
Fortune Shop	Green Intelligent Power Saver	I've received the power saver. I'm looking forward to the savings.	Jingdong prefecture level city in Shandong
Fortune Shop	Green Intelligent Power Saver	Received, have not yet used, after use and then evaluate	Jingdong prefecture level city in Shandong
Fortune Shop	Green Intelligent Power Saver	I received the item, but I don't know if it saves power or not.	Jingdong prefecture level city in Shandong
Fortune Shop	Green Intelligent Power Saver	On Trial	Jingdong prefecture level city in Shandong
Fortune Shop	Green Intelligent Power Saver	Use it and see how it goes.	Jingdong prefecture level city in Shandong
Fortune Shop	Green Intelligent Power Saver	Quality is very good, no odor, fine workmanship, grain color are very like, satisfied!	Jingdong prefecture level city in Shandong
Fortune Shop	Green Intelligent Power Saver	I'll give you a good review. I don't know if it'll work.	Jingdong prefecture level city in Shandong
Fortune Shop	Green Intelligent Power Saver	The logistics is very fast and the package is intact. Good use of affordable. The effect is very good, is genuine	Jingdong prefecture level city in Shandong
Fortune Shop	Green Intelligent Power Saver	Nothing happens when you plug it in.	Jingdong prefecture level city in Shandong
Fortune Shop	Green Intelligent Power Saver	I don't know if it's as good as it sounds.	Jingdong prefecture level city in Shandong
Fortune Shop	Green Intelligent Power Saver	The quality of the stuff is very good, exactly the same as the seller's description, very satisfied!	Jingdong prefecture level city in Shandong
Fortune Shop	Green Intelligent Power Saver	The saver works great, it really works, it's affordable and worth buying.	Jingdong prefecture level city in Shandong
Fortune Shop	Green Intelligent Power Saver	Just used it for a couple days. I don't know yet.	Jingdong prefecture level city in Shandong
Fortune Shop	Green Intelligent Power Saver	The quality is quite good, cheaper than the physical store, good, next time will come again!	Jingdong prefecture level city in Shandong
Fortune Shop	Green Intelligent Power Saver	Good quality, cheap and affordable worth recommending to buy!	Jingdong prefecture level city in Shandong
Fortune Shop	Green Intelligent Power Saver	Very good quality, well made, works well! Satisfied	Jingdong prefecture level city in Shandong
Fortune Shop	Green Intelligent Power Saver	The logistics is very fast quality is very good very pleasant a shopping	Jingdong prefecture level city in Shandong
Fortune Shop	Green Intelligent Power Saver	Use it and then come back to evaluate!	Jingdong prefecture level city in Shandong
Fortune Shop	Green Intelligent Power Saver	# It's no use lying at all #	Jingdong prefecture level city in Shandong
Fortune Shop	Green Intelligent Power Saver	I can't get a voltage drop.	Jingdong prefecture level city in Shandong
Fortune Shop	Green Intelligent Power Saver	Evaluate after use	Jingdong prefecture level city in Shandong
Fortune Shop	Green Intelligent Power Saver	Still on trial.	Jingdong prefecture level city in Shandong
Fortune Shop	Green Intelligent Power Saver	ineffective	Jingdong prefecture level city in Shandong
Fortune Shop	Green Intelligent Power Saver	Satisfaction Shopping	Jingdong prefecture level city in Shandong
Fortune Shop	Green Intelligent Power Saver	Lies.	Jingdong prefecture level city in Shandong
Fortune Shop	Green Intelligent Power Saver	It's a real power saver.	Jingdong prefecture level city in Shandong
Fortune Shop	Green Intelligent Power Saver	in use	Jingdong prefecture level city in Shandong

Fortune Shop	Green Intelligent Power Saver	I don't know yet.	Jingdong prefecture level city in Shandong
Fortune Shop	Green Intelligent Power Saver	not care about anything	Jingdong prefecture level city in Shandong
Fortune Shop	Green Intelligent Power Saver	It doesn't seem to have much effect	Jingdong prefecture level city in Shandong
Fortune Shop	Green Intelligent Power Saver	Let the merchant see for himself, does the plug positive and negative terminals look like this?	Jingdong prefecture level city in Shandong
Fortune Shop	Green Intelligent Power Saver	It doesn't work well, the electricity bill is less before using it, but more after using it, contacting customer service, the service is still not good, and returning it is slow	Jingdong prefecture level city in Shandong
Fortune Shop	Green Intelligent Power Saver	With the advertisement product does not match, above the effective negative, power saving rate of two rows of words it, the product looks very thin, with 3 days to see the meter number is still with the previous no difference, which is obviously fraudulent behavior!	Jingdong prefecture level city in Shandong
Fortune Shop	Green Intelligent Power Saver	Doesn't save any power at all. Not much use.	Jingdong prefecture level city in Shandong
Fortune Shop	Green Intelligent Power Saver	The plugs are all loose, they're all the same length and they don't work at all	Jingdong prefecture level city in Shandong
Fortune Shop	Green Intelligent Power Saver	It's bad 👎 it's not effective at all, you'd be crazy to sell this garbage on the internet.	Jingdong prefecture level city in Shandong
Fortune Shop	Green Intelligent Power Saver	It doesn't save any power at all, so I hope the old timers don't fall for this scam again	Jingdong prefecture level city in Shandong
Fortune Shop	Green Intelligent Power Saver	I've been using it for over a month now and haven't noticed any power savings.	Jingdong prefecture level city in Shandong
Fortune Shop	Green Intelligent Power Saver	It doesn't work at all. The electricity bill is higher when you use it.	Jingdong prefecture level city in Shandong
Fortune Shop	Green Intelligent Power Saver	It doesn't save any power, it's still as bad as ever.	Jingdong prefecture level city in Shandong
Fortune Shop	Green Intelligent Power Saver	Last night before leaving work to look at the table, and then plugged into the node device, this morning and then look at the table, than the original save a few degrees of electricity, very good to have a power-saving effect, easy to use, satisfied with the shopping praise.	Jingdong prefecture level city in Shandong
Fortune Shop	Green Intelligent Power Saver	The product is real and effective, the electricity bill is reduced, really good, thanks to the merchants have so many good products, the product is real and effective, the electricity bill is reduced, want to save electricity friends quickly buy it, early use early to save money.	Jingdong prefecture level city in Shandong
Fortune Shop	Green Intelligent Power Saver	Saver saves time and money, baby appearance is beautiful, the quality of goods is good, and the same as the business description, good service attitude, packaging is complete, logistics is very fast, praise to	Jingdong prefecture level city in Shandong
Fortune Shop	Green Intelligent Power Saver	The effect is good, summer, home electricity is relatively large, buy and use a few days, feel the effect is very good, originally ten dollars a day electricity bill, now eight dollars a day, quite good	Jingdong prefecture level city in Shandong
Fortune Shop	Green Intelligent Power Saver	Indeed can save electricity and money, the first time I know this or from a friend's home to see, but also do not believe it, and now they use it to know that there is this good thing.	Jingdong prefecture level city in Shandong
Fortune Shop	Green Intelligent Power Saver	I have received the Smart Power Saver. Perfectly packaged. Fast delivery. Boss customer service attitude is also very good. It's worth buying. I plugged it in and tried it and it really saves more power than the original. I'll buy it again next time.	Jingdong prefecture level city in Shandong
Fortune Shop	Green Intelligent Power Saver	It's nice to save a dozen bucks a night on two air conditioners that used to take 30 or 40 degrees a night, but now it's only 10 or so degrees a night	Jingdong prefecture level city in Shandong
Fortune Shop	Green Intelligent Power Saver	Product no manufacturer's name no product qualification certificate, try two days to see the effect of	Jingdong prefecture level city in Shandong
Fortune Shop	Green Intelligent Power Saver	I've been using it for over a month and have saved over $80, so I've repurchased a few more back to save together	Jingdong prefecture level city in Shandong
Fortune Shop	Green Intelligent Power Saver	I haven't used it yet and will comment when I do, but it's well made.	Jingdong prefecture level city in Shandong
Fortune Shop	Green Intelligent Power Saver	It's quite energy efficient. It's really a boon for the home.	Jingdong prefecture level city in Shandong
Fortune Shop	Green Intelligent Power Saver	Energy-saving socket received a month, after using it for a month proved that it is indeed energy-saving, electricity bill saving, will buy it again, thank you!	Jingdong prefecture level city in Shandong
Fortune Shop	Green Intelligent Power Saver	There was a similar product many years ago. Now technology has evolved, buy one to try, according to the instructions in the box, it takes one or two dozen days to show results. Check your electric bill and see what happens.	Jingdong prefecture level city in Shandong
Fortune Shop	Green Intelligent Power Saver	Jingdong logistics is really fast, the same day the order arrived on the same day, more convenient than the supermarket, and a wide range of choices, the	Jingdong prefecture level city in Shandong
Fortune Shop	Green Intelligent Power Saver	I've been using this for a month or so and I can't see any results, it really is a scam as they say.	Jingdong prefecture level city in Shandong
Fortune Shop	Green Intelligent Power Saver	Very good use, a month can save a few degrees of power loss of electricity, introduced to the relatives to buy back to try.	Jingdong prefecture level city in Shandong
Fortune Shop	Green Intelligent Power Saver	It's really powerful! With this air conditioner can be turned on at any time without the pressure of paying the electricity bill! It's good for the people!	Jingdong prefecture level city in Shandong
Fortune Shop	Green Intelligent Power Saver	This business is a big ** products are not qualified, with a month of electricity more than one hundred percent of thirty, the purchase agreed to return the goods but do not receive a refund after receiving the goods, looking for the official side does not help, the Jingdong is a big **, I have never shopped in	Jingdong prefecture level city in Shandong
Fortune Shop	Green Intelligent Power Saver	It's good. I tried it. It saves power.	Jingdong prefecture level city in Shandong
Fortune Shop	Green Intelligent Power Saver	Nothing**, it seems like there's more	Jingdong prefecture level city in Shandong
Fortune Shop	Green Intelligent Power Saver	Power saving device received, well packaged, good quality, fast logistics	Jingdong prefecture level city in Shandong
Fortune Shop	Green Intelligent Power Saver	The service is okay, is that when I bought five, the desirable pieces are three, and then wait a few days and send me two replacement, I do not know if it is a mistake of the shipper, if I was not in the courier there to see, go home to say no, the	Jingdong prefecture level city in Shandong
Fortune Shop	Green Intelligent Power Saver	This thing will only cost me more if I use it, but before I used it, it was less than a ~ electricity a month, and after I used it, it was more than two hundred kWh a month.	Jingdong prefecture level city in Shandong
Fortune Shop	Green Intelligent Power Saver	I just used it. I don't know how it is.	Jingdong prefecture level city in Shandong
Fortune Shop	Green Intelligent Power Saver	I've received the product and I'm using it, so I'll see how it goes in a month!	Jingdong prefecture level city in Shandong
Fortune Shop	Green Intelligent Power Saver	The goods have just received the effect do not know how, please look forward to the results.	Jingdong prefecture level city in Shandong
Fortune Shop	Green Intelligent Power Saver	Power saving device, the material is very good practical and durable, the size is suitable, packaging is very good, the price is cheap, logistics is very fast!	Jingdong prefecture level city in Shandong
Fortune Shop	Green Intelligent Power Saver	Very nice, will comment in detail after using it for a while.	Jingdong prefecture level city in Shandong
Fortune Shop	Green Intelligent Power Saver	Good stuff to trust and support	Jingdong prefecture level city in Shandong
Fortune Shop	Green Intelligent Power Saver	I'll comment after I use it, doubt if it really saves power no	Jingdong prefecture level city in Shandong
Fortune Shop	Green Intelligent Power Saver	It's good quality and works well!	Jingdong prefecture level city in Shandong
Fortune Shop	Green Intelligent Power Saver	hahaha just right hahahahahaha hiccup hahahaha	Jingdong prefecture level city in Shandong
Fortune Shop	Green Intelligent Power Saver	The real thing matches the picture and looks great!	Jingdong prefecture level city in Shandong
Fortune Shop	Green Intelligent Power Saver	Plugged it in and we'll see when we pay the bills at the end of the year!	Jingdong prefecture level city in Shandong
Fortune Shop	Green Intelligent Power Saver	False advertising. It doesn't work.	Jingdong prefecture level city in Shandong

Fortune Shop	Green Intelligent Power Saver	Goods received, good effect	Jingdong prefecture level city in Shandong
Fortune Shop	Green Intelligent Power Saver	It looks okay, we'll see if it saves electricity when the time comes	Jingdong prefecture level city in Shandong
Fortune Shop	Green Intelligent Power Saver	Logistics is very fast quality is very good, the price is very affordable, accessories are also complete, installed, satisfied with good use	Jingdong prefecture level city in Shandong
Fortune Shop	Green Intelligent Power Saver	I've received the power saver. I'm looking forward to the savings.	Jingdong prefecture level city in Shandong
Fortune Shop	Green Intelligent Power Saver	Received, have not yet used, after use and then evaluate	Jingdong prefecture level city in Shandong
Fortune Shop	Green Intelligent Power Saver	I received the item, but I don't know if it saves power or not.	Jingdong prefecture level city in Shandong
Fortune Shop	Green Intelligent Power Saver	On Trial	Jingdong prefecture level city in Shandong
Fortune Shop	Green Intelligent Power Saver	Use it and see how it goes.	Jingdong prefecture level city in Shandong
Fortune Shop	Green Intelligent Power Saver	Quality is very good, no odor, fine workmanship, grain color are very like, satisfied!	Jingdong prefecture level city in Shandong
Fortune Shop	Green Intelligent Power Saver	I'll give you a good review. I don't know if it'll work.	Jingdong prefecture level city in Shandong
Fortune Shop	Green Intelligent Power Saver	The logistics is very fast and the package is intact. Good use of affordable. The effect is very good, is genuine	Jingdong prefecture level city in Shandong
Fortune Shop	Green Intelligent Power Saver	Nothing happens when you plug it in.	Jingdong prefecture level city in Shandong
Fortune Shop	Green Intelligent Power Saver	I don't know if it's as good as it sounds.	Jingdong prefecture level city in Shandong
Fortune Shop	Green Intelligent Power Saver	The quality of the stuff is very good, exactly the same as the seller's description, very satisfied!	Jingdong prefecture level city in Shandong
Fortune Shop	Green Intelligent Power Saver	The saver works great, it really works, it's affordable and worth buying.	Jingdong prefecture level city in Shandong
Fortune Shop	Green Intelligent Power Saver	Just used it for a couple days. I don't know yet.	Jingdong prefecture level city in Shandong
Fortune Shop	Green Intelligent Power Saver	The quality is quite good, cheaper than the physical store, good, next time will come again!	Jingdong prefecture level city in Shandong
Fortune Shop	Green Intelligent Power Saver	Good quality, cheap and affordable worth recommending to buy!	Jingdong prefecture level city in Shandong
Fortune Shop	Green Intelligent Power Saver	Very good quality, well made, works well! Satisfied	Jingdong prefecture level city in Shandong
Fortune Shop	Green Intelligent Power Saver	The logistics is very fast quality is very good very pleasant a shopping	Jingdong prefecture level city in Shandong
Fortune Shop	Green Intelligent Power Saver	Use it and then come back to evaluate!	Jingdong prefecture level city in Shandong
Fortune Shop	Green Intelligent Power Saver	# It's no use lying at all #	Jingdong prefecture level city in Shandong
Fortune Shop	Green Intelligent Power Saver	I can't get a voltage drop.	Jingdong prefecture level city in Shandong
Fortune Shop	Green Intelligent Power Saver	Evaluate after use	Jingdong prefecture level city in Shandong
Fortune Shop	Green Intelligent Power Saver	Still on trial.	Jingdong prefecture level city in Shandong
Fortune Shop	Green Intelligent Power Saver	ineffective	Jingdong prefecture level city in Shandong
Fortune Shop	Green Intelligent Power Saver	Satisfaction Shopping	Jingdong prefecture level city in Shandong
Fortune Shop	Green Intelligent Power Saver	Lies.	Jingdong prefecture level city in Shandong
Fortune Shop	Green Intelligent Power Saver	It's a real power saver.	Jingdong prefecture level city in Shandong
Fortune Shop	Green Intelligent Power Saver	in use	Jingdong prefecture level city in Shandong
Fortune Shop	Green Intelligent Power Saver	I don't know yet.	Jingdong prefecture level city in Shandong
Fortune Shop	Green Intelligent Power Saver	not care about anything	Jingdong prefecture level city in Shandong
Fortune Shop	Green Intelligent Power Saver	It doesn't seem to have much effect	Jingdong prefecture level city in Shandong
Fortune Shop	Green Intelligent Power Saver	Let the merchant see for himself, does the plug positive and negative terminals look like this?	Jingdong prefecture level city in Shandong
Fortune Shop	Green Intelligent Power Saver	It doesn't work well, the electricity bill is less before using it, but more after using it, contacting customer service, the service is still not good, and returning it is slow	Jingdong prefecture level city in Shandong
Fortune Shop	Green Intelligent Power Saver	With the advertisement product does not match, above the effective negative, power saving rate of two rows of words it, the product looks very thin, with 3 days to see the meter number is still with the previous no difference, which is obviously fraudulent behavior!	Jingdong prefecture level city in Shandong
Fortune Shop	Green Intelligent Power Saver	Doesn't save any power at all. Not much use.	Jingdong prefecture level city in Shandong
Fortune Shop	Green Intelligent Power Saver	The plugs are all loose, they're all the same length and they don't work at all	Jingdong prefecture level city in Shandong
Fortune Shop	Green Intelligent Power Saver	It's bad 👎 it's not effective at all, you'd be crazy to sell this garbage on the internet.	Jingdong prefecture level city in Shandong
Fortune Shop	Green Intelligent Power Saver	It doesn't save any power at all, so I hope the old timers don't fall for this scam again	Jingdong prefecture level city in Shandong
Fortune Shop	Green Intelligent Power Saver	I've been using it for over a month now and haven't noticed any power savings.	Jingdong prefecture level city in Shandong
Fortune Shop	Green Intelligent Power Saver	It doesn't work at all. The electricity bill is higher when you use it.	Jingdong prefecture level city in Shandong
Fortune Shop	Green Intelligent Power Saver	It doesn't save any power, it's still as bad as ever.	Jingdong prefecture level city in Shandong
Fortune Shop	Green Intelligent Power Saver	Last night before leaving work to look at the table, and then plugged into the node device, this morning and then look at the table, than the original save a few degrees of electricity, very good to have a power-saving effect, easy to use, satisfied with the shopping praise.	Jingdong prefecture level city in Shandong
Fortune Shop	Green Intelligent Power Saver	The product is real and effective, the electricity bill is reduced, really good, thanks to the merchants have so many good products, the product is real and effective, the electricity bill is reduced, want to save electricity friends quickly buy it, early use early to save money.	Jingdong prefecture level city in Shandong
Fortune Shop	Green Intelligent Power Saver	Saver saves time and money, baby appearance is beautiful, the quality of goods is good, and the same as the business description, good service attitude, packaging is complete, logistics is very fast, praise to	Jingdong prefecture level city in Shandong
Fortune Shop	Green Intelligent Power Saver	The effect is good, summer, home electricity is relatively large, buy and use a few days, feel the effect is very good, originally ten dollars a day electricity bill, now eight dollars a day, quite good	Jingdong prefecture level city in Shandong
Fortune Shop	Green Intelligent Power Saver	Indeed can save electricity and money, the first time I know this or from a friend's home to see, but also do not believe it, and now they use it to know that there is this good thing.	Jingdong prefecture level city in Shandong
Fortune Shop	Green Intelligent Power Saver	I have received the Smart Power Saver. Perfectly packaged. Fast delivery. Boss customer service attitude is also very good. It's worth buying. I plugged it in and tried it and it really saves more power than the original. I'll buy it again next time.	Jingdong prefecture level city in Shandong

Fortune Shop	Green Intelligent Power Saver	It's nice to save a dozen bucks a night on two air conditioners that used to take 30 or 40 degrees a night, but now it's only 10 or so degrees a night	Jingdong prefecture level city in Shandong
Fortune Shop	Green Intelligent Power Saver	Product no manufacturer's name no product qualification certificate, try two days to see the effect of	Jingdong prefecture level city in Shandong
Fortune Shop	Green Intelligent Power Saver	I've been using it for over a month and have saved over $80, so I've repurchased a few more back to save together	Jingdong prefecture level city in Shandong
Fortune Shop	Green Intelligent Power Saver	I haven't used it yet and will comment when I do, but it's well made.	Jingdong prefecture level city in Shandong
Fortune Shop	Green Intelligent Power Saver	It's quite energy efficient. It's really a boon for the home.	Jingdong prefecture level city in Shandong
Fortune Shop	Green Intelligent Power Saver	Energy-saving socket received a month, after using it for a month proved that it is indeed energy-saving, electricity bill saving, will buy it again, thank you!	Jingdong prefecture level city in Shandong
Fortune Shop	Green Intelligent Power Saver	There was a similar product many years ago. Now technology has evolved, buy one to try, according to the instructions in the box, it takes one or two dozen days to show results. Check your electric bill and see what happens.	Jingdong prefecture level city in Shandong
Fortune Shop	Green Intelligent Power Saver	Jingdong logistics is really fast, the same day the order arrived on the same day, more convenient than the supermarket, and a wide range of choices, the	Jingdong prefecture level city in Shandong
Fortune Shop	Green Intelligent Power Saver	I've been using this for a month or so and I can't see any results, it really is a scam as they say.	Jingdong prefecture level city in Shandong
Fortune Shop	Green Intelligent Power Saver	Very good use, a month can save a few degrees of power loss of electricity, introduced to the relatives to buy back to try.	Jingdong prefecture level city in Shandong
Fortune Shop	Green Intelligent Power Saver	It's really powerful! With this air conditioner can be turned on at any time without the pressure of paying the electricity bill! It's good for the people!	Jingdong prefecture level city in Shandong
Fortune Shop	Green Intelligent Power Saver	This business is a big ** products are not qualified, with a month of electricity more than one hundred percent of thirty, the purchase agreed to return the goods but do not receive a refund after receiving the goods, looking for the official side does not help, the Jingdong is a big **, I have never shopped in	Jingdong prefecture level city in Shandong
Fortune Shop	Green Intelligent Power Saver	It's good. I tried it. It saves power.	Jingdong prefecture level city in Shandong
Fortune Shop	Green Intelligent Power Saver	Nothing**, it seems like there's more	Jingdong prefecture level city in Shandong
Fortune Shop	Green Intelligent Power Saver	Power saving device received, well packaged, good quality, fast logistics	Jingdong prefecture level city in Shandong
Fortune Shop	Green Intelligent Power Saver	The service is okay, is that when I bought five, the desirable pieces are three, and then wait a few days and send me two replacement, I do not know if it is a mistake of the shipper, if I was not in the courier there to see, go home to say no, the	Jingdong prefecture level city in Shandong
Fortune Shop	Green Intelligent Power Saver	This thing will only cost me more if I use it, but before I used it, it was less than a ~ electricity a month, and after I used it, it was more than two hundred kWh a month.	Jingdong prefecture level city in Shandong
Fortune Shop	Green Intelligent Power Saver	I just used it. I don't know how it is.	Jingdong prefecture level city in Shandong
Fortune Shop	Green Intelligent Power Saver	I've received the product and I'm using it, so I'll see how it goes in a month!	Jingdong prefecture level city in Shandong
Fortune Shop	Green Intelligent Power Saver	The goods have just received the effect do not know how, please look forward to the results.	Jingdong prefecture level city in Shandong
Fortune Shop	Green Intelligent Power Saver	Power saving device, the material is very good practical and durable, the size is suitable, packaging is very good, the price is cheap, logistics is very fast!	Jingdong prefecture level city in Shandong
Fortune Shop	Green Intelligent Power Saver	Very nice, will comment in detail after using it for a while.	Jingdong prefecture level city in Shandong
Fortune Shop	Green Intelligent Power Saver	Good stuff to trust and support	Jingdong prefecture level city in Shandong
Fortune Shop	Green Intelligent Power Saver	I'll comment after I use it, doubt if it really saves power no	Jingdong prefecture level city in Shandong
Fortune Shop	Green Intelligent Power Saver	It's good quality and works well!	Jingdong prefecture level city in Shandong
Fortune Shop	Green Intelligent Power Saver	hahaha just right hahahahahaha hiccup hahahaha	Jingdong prefecture level city in Shandong
Fortune Shop	Green Intelligent Power Saver	The real thing matches the picture and looks great!	Jingdong prefecture level city in Shandong
Fortune Shop	Green Intelligent Power Saver	Plugged it in and we'll see when we pay the bills at the end of the year!	Jingdong prefecture level city in Shandong
Fortune Shop	Green Intelligent Power Saver	False advertising. It doesn't work.	Jingdong prefecture level city in Shandong
Fortune Shop	Green Intelligent Power Saver	Goods received, good effect	Jingdong prefecture level city in Shandong
Fortune Shop	Green Intelligent Power Saver	It looks okay, we'll see if it saves electricity when the time comes	Jingdong prefecture level city in Shandong
Fortune Shop	Green Intelligent Power Saver	Logistics is very fast quality is very good, the price is very affordable, accessories are also complete, installed, satisfied with good use	Jingdong prefecture level city in Shandong
Fortune Shop	Green Intelligent Power Saver	I've received the power saver. I'm looking forward to the savings.	Jingdong prefecture level city in Shandong
Fortune Shop	Green Intelligent Power Saver	Received, have not yet used, after use and then evaluate	Jingdong prefecture level city in Shandong
Fortune Shop	Green Intelligent Power Saver	I received the item, but I don't know if it saves power or not.	Jingdong prefecture level city in Shandong
Fortune Shop	Green Intelligent Power Saver	On Trial	Jingdong prefecture level city in Shandong
Fortune Shop	Green Intelligent Power Saver	Use it and see how it goes.	Jingdong prefecture level city in Shandong
Fortune Shop	Green Intelligent Power Saver	Quality is very good, no odor, fine workmanship, grain color are very like, satisfied!	Jingdong prefecture level city in Shandong
Fortune Shop	Green Intelligent Power Saver	I'll give you a good review. I don't know if it'll work.	Jingdong prefecture level city in Shandong
Fortune Shop	Green Intelligent Power Saver	The logistics is very fast and the package is intact. Good use of affordable. The effect is very good, is genuine	Jingdong prefecture level city in Shandong
Fortune Shop	Green Intelligent Power Saver	Nothing happens when you plug it in.	Jingdong prefecture level city in Shandong
Fortune Shop	Green Intelligent Power Saver	I don't know if it's as good as it sounds.	Jingdong prefecture level city in Shandong
Fortune Shop	Green Intelligent Power Saver	The quality of the stuff is very good, exactly the same as the seller's description, very satisfied!	Jingdong prefecture level city in Shandong
Fortune Shop	Green Intelligent Power Saver	The saver works great, it really works, it's affordable and worth buying.	Jingdong prefecture level city in Shandong
Fortune Shop	Green Intelligent Power Saver	Just used it for a couple days. I don't know yet.	Jingdong prefecture level city in Shandong
Fortune Shop	Green Intelligent Power Saver	The quality is quite good, cheaper than the physical store, good, next time will come again!	Jingdong prefecture level city in Shandong
Fortune Shop	Green Intelligent Power Saver	Good quality, cheap and affordable worth recommending to buy!	Jingdong prefecture level city in Shandong
Fortune Shop	Green Intelligent Power Saver	Very good quality, well made, works well! Satisfied	Jingdong prefecture level city in Shandong
Fortune Shop	Green Intelligent Power Saver	The logistics is very fast quality is very good very pleasant a shopping	Jingdong prefecture level city in Shandong
Fortune Shop	Green Intelligent Power Saver	Use it and then come back to evaluate!	Jingdong prefecture level city in Shandong

Fortune Shop	Green Intelligent Power Saver	# It's no use lying at all #	Jingdong prefecture level city in Shandong
Fortune Shop	Green Intelligent Power Saver	I can't get a voltage drop.	Jingdong prefecture level city in Shandong
Fortune Shop	Green Intelligent Power Saver	Evaluate after use	Jingdong prefecture level city in Shandong
Fortune Shop	Green Intelligent Power Saver	Still on trial.	Jingdong prefecture level city in Shandong
Fortune Shop	Green Intelligent Power Saver	ineffective	Jingdong prefecture level city in Shandong
Fortune Shop	Green Intelligent Power Saver	Satisfaction Shopping	Jingdong prefecture level city in Shandong
Fortune Shop	Green Intelligent Power Saver	Lies.	Jingdong prefecture level city in Shandong
Fortune Shop	Green Intelligent Power Saver	It's a real power saver.	Jingdong prefecture level city in Shandong
Fortune Shop	Green Intelligent Power Saver	in use	Jingdong prefecture level city in Shandong
Fortune Shop	Green Intelligent Power Saver	I don't know yet.	Jingdong prefecture level city in Shandong
Fortune Shop	Green Intelligent Power Saver	not care about anything	Jingdong prefecture level city in Shandong
Fortune Shop	Green Intelligent Power Saver	It doesn't seem to have much effect	Jingdong prefecture level city in Shandong
Fortune Shop	Green Intelligent Power Saver	Let the merchant see for himself, does the plug positive and negative terminals look like this?	Jingdong prefecture level city in Shandong
Fortune Shop	Green Intelligent Power Saver	It doesn't work well, the electricity bill is less before using it, but more after using it, contacting customer service, the service is still not good, and returning it is slow	Jingdong prefecture level city in Shandong
Fortune Shop	Green Intelligent Power Saver	With the advertisement product does not match, above the effective negative, power saving rate of two rows of words it, the product looks very thin, with 3 days to see the meter number is still with the previous no difference, which is obviously fraudulent behavior!	Jingdong prefecture level city in Shandong
Fortune Shop	Green Intelligent Power Saver	Doesn't save any power at all. Not much use.	Jingdong prefecture level city in Shandong
Fortune Shop	Green Intelligent Power Saver	The plugs are all loose, they're all the same length and they don't work at all	Jingdong prefecture level city in Shandong
Fortune Shop	Green Intelligent Power Saver	It's bad 👎 it's not effective at all, you'd be crazy to sell this garbage on the internet.	Jingdong prefecture level city in Shandong
Fortune Shop	Green Intelligent Power Saver	It doesn't save any power at all, so I hope the old timers don't fall for this scam again	Jingdong prefecture level city in Shandong
Fortune Shop	Green Intelligent Power Saver	I've been using it for over a month now and haven't noticed any power savings.	Jingdong prefecture level city in Shandong
Fortune Shop	Green Intelligent Power Saver	It doesn't work at all. The electricity bill is higher when you use it.	Jingdong prefecture level city in Shandong
Fortune Shop	Green Intelligent Power Saver	It doesn't save any power, it's still as bad as ever.	Jingdong prefecture level city in Shandong
Fortune Shop	Green Intelligent Power Saver	Last night before leaving work to look at the table, and then plugged into the node device, this morning and then look at the table, than the original save a few degrees of electricity, very good to have a power-saving effect, easy to use, satisfied with the shopping praise.	Jingdong prefecture level city in Shandong
Fortune Shop	Green Intelligent Power Saver	The product is real and effective, the electricity bill is reduced, really good, thanks to the merchants have so many good products, the product is real and effective, the electricity bill is reduced, want to save electricity friends quickly buy it, early use early to save money.	Jingdong prefecture level city in Shandong
Fortune Shop	Green Intelligent Power Saver	Saver saves time and money, baby appearance is beautiful, the quality of goods is good, and the same as the business description, good service attitude, packaging is complete, logistics is very fast, praise to	Jingdong prefecture level city in Shandong
Fortune Shop	Green Intelligent Power Saver	The effect is good, summer, home electricity is relatively large, buy and use a few days, feel the effect is very good, originally ten dollars a day electricity bill, now eight dollars a day, quite good	Jingdong prefecture level city in Shandong
Fortune Shop	Green Intelligent Power Saver	Indeed can save electricity and money, the first time I know this or from a friend's home to see, but also do not believe it, and now they use it to know that there is this good thing.	Jingdong prefecture level city in Shandong
Fortune Shop	Green Intelligent Power Saver	I have received the Smart Power Saver. Perfectly packaged. Fast delivery. Boss customer service attitude is also very good. It's worth buying. I plugged it in and tried it and it really saves more power than the original. I'll buy it again next time.	Jingdong prefecture level city in Shandong
Fortune Shop	Green Intelligent Power Saver	It's nice to save a dozen bucks a night on two air conditioners that used to take 30 or 40 degrees a night, but now it's only 10 or so degrees a night	Jingdong prefecture level city in Shandong
Fortune Shop	Green Intelligent Power Saver	Product no manufacturer's name no product qualification certificate, try two days to see the effect of	Jingdong prefecture level city in Shandong
Fortune Shop	Green Intelligent Power Saver	I've been using it for over a month and have saved over $80, so I've repurchased a few more back to save together	Jingdong prefecture level city in Shandong
Fortune Shop	Green Intelligent Power Saver	I haven't used it yet and will comment when I do, but it's well made.	Jingdong prefecture level city in Shandong
Fortune Shop	Green Intelligent Power Saver	It's quite energy efficient. It's really a boon for the home.	Jingdong prefecture level city in Shandong
Fortune Shop	Green Intelligent Power Saver	Energy-saving socket received a month, after using it for a month proved that it is indeed energy-saving, electricity bill saving, will buy it again, thank you!	Jingdong prefecture level city in Shandong
Fortune Shop	Green Intelligent Power Saver	There was a similar product many years ago. Now technology has evolved, buy one to try, according to the instructions in the box, it takes one or two dozen days to show results. Check your electric bill and see what happens.	Jingdong prefecture level city in Shandong
Fortune Shop	Green Intelligent Power Saver	Jingdong logistics is really fast, the same day the order arrived on the same day, more convenient than the supermarket, and a wide range of choices, the	Jingdong prefecture level city in Shandong
Fortune Shop	Green Intelligent Power Saver	I've been using this for a month or so and I can't see any results, it really is a scam as they say.	Jingdong prefecture level city in Shandong
Fortune Shop	Green Intelligent Power Saver	Very good use, a month can save a few degrees of power loss of electricity, introduced to the relatives to buy back to try.	Jingdong prefecture level city in Shandong
Fortune Shop	Green Intelligent Power Saver	It's really powerful! With this air conditioner can be turned on at any time without the pressure of paying the electricity bill! It's good for the people!	Jingdong prefecture level city in Shandong
Fortune Shop	Green Intelligent Power Saver	This business is a big ** products are not qualified, with a month of electricity more than one hundred percent of thirty, the purchase agreed to return the goods but do not receive a refund after receiving the goods, looking for the official side does not help, the Jingdong is a big **, I have never shopped in	Jingdong prefecture level city in Shandong
Fortune Shop	Green Intelligent Power Saver	It's good. I tried it. It saves power.	Jingdong prefecture level city in Shandong
Fortune Shop	Green Intelligent Power Saver	Nothing**, it seems like there's more	Jingdong prefecture level city in Shandong
Fortune Shop	Green Intelligent Power Saver	Power saving device received, well packaged, good quality, fast logistics	Jingdong prefecture level city in Shandong
Fortune Shop	Green Intelligent Power Saver	The service is okay, is that when I bought five, the desirable pieces are three, and then wait a few days and send me two replacement, I do not know if it is a mistake of the shipper, if I was not in the courier there to see, go home to say no, the	Jingdong prefecture level city in Shandong
Fortune Shop	Green Intelligent Power Saver	This thing will only cost me more if I use it, but before I used it, it was less than a ~ electricity a month, and after I used it, it was more than two hundred kWh a month.	Jingdong prefecture level city in Shandong
Fortune Shop	Green Intelligent Power Saver	I just used it. I don't know how it is.	Jingdong prefecture level city in Shandong
Fortune Shop	Green Intelligent Power Saver	I've received the product and I'm using it, so I'll see how it goes in a month!	Jingdong prefecture level city in Shandong
Fortune Shop	Green Intelligent Power Saver	The goods have just received the effect do not know how, please look forward to the results.	Jingdong prefecture level city in Shandong

Fortune Shop	Green Intelligent Power Saver	Power saving device, the material is very good practical and durable, the size is suitable, packaging is very good, the price is cheap, logistics is very fast!	Jingdong prefecture level city in Shandong
Fortune Shop	Green Intelligent Power Saver	Very nice, will comment in detail after using it for a while.	Jingdong prefecture level city in Shandong
Fortune Shop	Green Intelligent Power Saver	Good stuff to trust and support	Jingdong prefecture level city in Shandong
Fortune Shop	Green Intelligent Power Saver	I’ll comment after I use it, doubt if it really saves power no	Jingdong prefecture level city in Shandong
Fortune Shop	Green Intelligent Power Saver	It’s good quality and works well!	Jingdong prefecture level city in Shandong
Fortune Shop	Green Intelligent Power Saver	hahaha just right hahahahahaha hiccup hahahaha	Jingdong prefecture level city in Shandong
Fortune Shop	Green Intelligent Power Saver	The real thing matches the picture and looks great!	Jingdong prefecture level city in Shandong
Fortune Shop	Green Intelligent Power Saver	Plugged it in and we’ll see when we pay the bills at the end of the year!	Jingdong prefecture level city in Shandong
Fortune Shop	Green Intelligent Power Saver	False advertising. It doesn’t work.	Jingdong prefecture level city in Shandong
Fortune Shop	Green Intelligent Power Saver	Goods received, good effect	Jingdong prefecture level city in Shandong
Fortune Shop	Green Intelligent Power Saver	It looks okay, we’ll see if it saves electricity when the time comes	Jingdong prefecture level city in Shandong
Fortune Shop	Green Intelligent Power Saver	Logistics is very fast quality is very good, the price is very affordable, accessories are also complete, installed, satisfied with good use	Jingdong prefecture level city in Shandong
Fortune Shop	Green Intelligent Power Saver	I’ve received the power saver. I’m looking forward to the savings.	Jingdong prefecture level city in Shandong
Fortune Shop	Green Intelligent Power Saver	Received, have not yet used, after use and then evaluate	Jingdong prefecture level city in Shandong
Fortune Shop	Green Intelligent Power Saver	I received the item, but I don’t know if it saves power or not.	Jingdong prefecture level city in Shandong
Fortune Shop	Green Intelligent Power Saver	On Trial	Jingdong prefecture level city in Shandong
Fortune Shop	Green Intelligent Power Saver	Use it and see how it goes.	Jingdong prefecture level city in Shandong
Fortune Shop	Green Intelligent Power Saver	Quality is very good, no odor, fine workmanship, grain color are very like, satisfied!	Jingdong prefecture level city in Shandong
Fortune Shop	Green Intelligent Power Saver	I’ll give you a good review. I don’t know if it’ll work.	Jingdong prefecture level city in Shandong
Fortune Shop	Green Intelligent Power Saver	The logistics is very fast and the package is intact. Good use of affordable. The effect is very good, is genuine	Jingdong prefecture level city in Shandong
Fortune Shop	Green Intelligent Power Saver	Nothing happens when you plug it in.	Jingdong prefecture level city in Shandong
Fortune Shop	Green Intelligent Power Saver	I don’t know if it’s as good as it sounds.	Jingdong prefecture level city in Shandong
Fortune Shop	Green Intelligent Power Saver	The quality of the stuff is very good, exactly the same as the seller’s description, very satisfied!	Jingdong prefecture level city in Shandong
Fortune Shop	Green Intelligent Power Saver	The saver works great, it really works, it’s affordable and worth buying.	Jingdong prefecture level city in Shandong
Fortune Shop	Green Intelligent Power Saver	Just used it for a couple days. I don’t know yet.	Jingdong prefecture level city in Shandong
Fortune Shop	Green Intelligent Power Saver	The quality is quite good, cheaper than the physical store, good, next time will come again!	Jingdong prefecture level city in Shandong
Fortune Shop	Green Intelligent Power Saver	Good quality, cheap and affordable worth recommending to buy!	Jingdong prefecture level city in Shandong
Fortune Shop	Green Intelligent Power Saver	Very good quality, well made, works well! Satisfied	Jingdong prefecture level city in Shandong
Fortune Shop	Green Intelligent Power Saver	The logistics is very fast quality is very good very pleasant a shopping	Jingdong prefecture level city in Shandong
Fortune Shop	Green Intelligent Power Saver	Use it and then come back to evaluate!	Jingdong prefecture level city in Shandong
Fortune Shop	Green Intelligent Power Saver	# It’s no use lying at all #	Jingdong prefecture level city in Shandong
Fortune Shop	Green Intelligent Power Saver	I can’t get a voltage drop.	Jingdong prefecture level city in Shandong
Fortune Shop	Green Intelligent Power Saver	Evaluate after use	Jingdong prefecture level city in Shandong
Fortune Shop	Green Intelligent Power Saver	Still on trial.	Jingdong prefecture level city in Shandong
Fortune Shop	Green Intelligent Power Saver	ineffective	Jingdong prefecture level city in Shandong
Fortune Shop	Green Intelligent Power Saver	Satisfaction Shopping	Jingdong prefecture level city in Shandong
Fortune Shop	Green Intelligent Power Saver	Lies.	Jingdong prefecture level city in Shandong
Fortune Shop	Green Intelligent Power Saver	It’s a real power saver.	Jingdong prefecture level city in Shandong
Fortune Shop	Green Intelligent Power Saver	in use	Jingdong prefecture level city in Shandong
Fortune Shop	Green Intelligent Power Saver	I don’t know yet.	Jingdong prefecture level city in Shandong
Fortune Shop	Green Intelligent Power Saver	not care about anything	Jingdong prefecture level city in Shandong
Fortune Shop	Green Intelligent Power Saver	It doesn’t seem to have much effect	Jingdong prefecture level city in Shandong
Fortune Shop	Green Intelligent Power Saver	Let the merchant see for himself, does the plug positive and negative terminals look like this?	Jingdong prefecture level city in Shandong
Fortune Shop	Green Intelligent Power Saver	It doesn’t work well, the electricity bill is less before using it, but more after using it, contacting customer service, the service is still not good, and returning it is slow	Jingdong prefecture level city in Shandong
Fortune Shop	Green Intelligent Power Saver	With the advertisement product does not match, above the effective negative, power saving rate of two rows of words it, the product looks very thin, with 3 days to see the meter number is still with the previous no difference, which is obviously fraudulent behavior!	Jingdong prefecture level city in Shandong
Fortune Shop	Green Intelligent Power Saver	Doesn’t save any power at all. Not much use.	Jingdong prefecture level city in Shandong
Fortune Shop	Green Intelligent Power Saver	The plugs are all loose, they’re all the same length and they don’t work at all	Jingdong prefecture level city in Shandong
Fortune Shop	Green Intelligent Power Saver	It’s bad 👎 it’s not effective at all, you’d be crazy to sell this garbage on the internet.	Jingdong prefecture level city in Shandong
Fortune Shop	Green Intelligent Power Saver	It doesn’t save any power at all, so I hope the old timers don’t fall for this scam again	Jingdong prefecture level city in Shandong
Fortune Shop	Green Intelligent Power Saver	I’ve been using it for over a month now and haven’t noticed any power savings.	Jingdong prefecture level city in Shandong
Fortune Shop	Green Intelligent Power Saver	It doesn’t work at all. The electricity bill is higher when you use it.	Jingdong prefecture level city in Shandong

Fortune Shop	Green Intelligent Power Saver	It doesn't save any power, it's still as bad as ever.	Jingdong prefecture level city in Shandong
Fortune Shop	Green Intelligent Power Saver	Last night before leaving work to look at the table, and then plugged into the node device, this morning and then look at the table, than the original save a few degrees of electricity, very good to have a power-saving effect, easy to use, satisfied with the shopping praise.	Jingdong prefecture level city in Shandong
Fortune Shop	Green Intelligent Power Saver	The product is real and effective, the electricity bill is reduced, really good, thanks to the merchants have so many good products, the product is real and effective, the electricity bill is reduced, want to save electricity friends quickly buy it, early use early to save money.	Jingdong prefecture level city in Shandong
Fortune Shop	Green Intelligent Power Saver	Saver saves time and money, baby appearance is beautiful, the quality of goods is good, and the same as the business description, good service attitude, packaging is complete, logistics is very fast, praise to	Jingdong prefecture level city in Shandong
Fortune Shop	Green Intelligent Power Saver	The effect is good, summer, home electricity is relatively large, buy and use a few days, feel the effect is very good, originally ten dollars a day electricity bill, now eight dollars a day, quite good	Jingdong prefecture level city in Shandong
Fortune Shop	Green Intelligent Power Saver	Indeed can save electricity and money, the first time I know this or from a friend's home to see, but also do not believe it, and now they use it to know that there is this good thing.	Jingdong prefecture level city in Shandong
Fortune Shop	Green Intelligent Power Saver	I have received the Smart Power Saver. Perfectly packaged. Fast delivery. Boss customer service attitude is also very good. It's worth buying. I plugged it in and tried it and it really saves more power than the original. I'll buy it again next time.	Jingdong prefecture level city in Shandong
Fortune Shop	Green Intelligent Power Saver	It's nice to save a dozen bucks a night on two air conditioners that used to take 30 or 40 degrees a night, but now it's only 10 or so degrees a night	Jingdong prefecture level city in Shandong
Fortune Shop	Green Intelligent Power Saver	Product no manufacturer's name no product qualification certificate, try two days to see the effect of	Jingdong prefecture level city in Shandong
Fortune Shop	Green Intelligent Power Saver	I've been using it for over a month and have saved over $80, so I've repurchased a few more back to save together	Jingdong prefecture level city in Shandong
Fortune Shop	Green Intelligent Power Saver	I haven't used it yet and will comment when I do, but it's well made.	Jingdong prefecture level city in Shandong
Fortune Shop	Green Intelligent Power Saver	It's quite energy efficient. It's really a boon for the home.	Jingdong prefecture level city in Shandong
Fortune Shop	Green Intelligent Power Saver	Energy-saving socket received a month, after using it for a month proved that it is indeed energy-saving, electricity bill saving, will buy it again, thank you!	Jingdong prefecture level city in Shandong
Fortune Shop	Green Intelligent Power Saver	There was a similar product many years ago. Now technology has evolved, buy one to try, according to the instructions in the box, it takes one or two dozen days to show results. Check your electric bill and see what happens.	Jingdong prefecture level city in Shandong
Fortune Shop	Green Intelligent Power Saver	Jingdong logistics is really fast, the same day the order arrived on the same day, more convenient than the supermarket, and a wide range of choices, the	Jingdong prefecture level city in Shandong
Fortune Shop	Green Intelligent Power Saver	I've been using this for a month or so and I can't see any results, it really is a scam as they say.	Jingdong prefecture level city in Shandong
Fortune Shop	Green Intelligent Power Saver	Very good use, a month can save a few degrees of power loss of electricity, introduced to the relatives to buy back to try.	Jingdong prefecture level city in Shandong
Fortune Shop	Green Intelligent Power Saver	It's really powerful! With this air conditioner can be turned on at any time without the pressure of paying the electricity bill! It's good for the people!	Jingdong prefecture level city in Shandong
Fortune Shop	Green Intelligent Power Saver	This business is a big ** products are not qualified, with a month of electricity more than one hundred percent of thirty, the purchase agreed to return the goods but do not receive a refund after receiving the goods, looking for the official side does not help, the Jingdong is a big **, I have never shopped in	Jingdong prefecture level city in Shandong
Fortune Shop	Green Intelligent Power Saver	It's good. I tried it. It saves power.	Jingdong prefecture level city in Shandong
Fortune Shop	Green Intelligent Power Saver	Nothing**, it seems like there's more	Jingdong prefecture level city in Shandong
Fortune Shop	Green Intelligent Power Saver	Power saving device received, well packaged, good quality, fast logistics	Jingdong prefecture level city in Shandong
Fortune Shop	Green Intelligent Power Saver	The service is okay, is that when I bought five, the desirable pieces are three, and then wait a few days and send me two replacement, I do not know if it is a mistake of the shipper, if I was not in the courier there to see, go home to say no, the	Jingdong prefecture level city in Shandong
Fortune Shop	Green Intelligent Power Saver	This thing will only cost me more if I use it, but before I used it, it was less than a ~ electricity a month, and after I used it, it was more than two hundred kWh a month.	Jingdong prefecture level city in Shandong
Fortune Shop	Green Intelligent Power Saver	I just used it. I don't know how it is.	Jingdong prefecture level city in Shandong
Fortune Shop	Green Intelligent Power Saver	I've received the product and I'm using it, so I'll see how it goes in a month!	Jingdong prefecture level city in Shandong
Fortune Shop	Green Intelligent Power Saver	The goods have just received the effect do not know how, please look forward to the results.	Jingdong prefecture level city in Shandong
Fortune Shop	Green Intelligent Power Saver	Power saving device, the material is very good practical and durable, the size is suitable, packaging is very good, the price is cheap, logistics is very fast!	Jingdong prefecture level city in Shandong
Fortune Shop	Green Intelligent Power Saver	Very nice, will comment in detail after using it for a while.	Jingdong prefecture level city in Shandong
Fortune Shop	Green Intelligent Power Saver	Good stuff to trust and support	Jingdong prefecture level city in Shandong
Fortune Shop	Green Intelligent Power Saver	I'll comment after I use it, doubt if it really saves power no	Jingdong prefecture level city in Shandong
Fortune Shop	Green Intelligent Power Saver	It's good quality and works well!	Jingdong prefecture level city in Shandong
Fortune Shop	Green Intelligent Power Saver	hahaha just right hahahahahaha hiccup hahahaha	Jingdong prefecture level city in Shandong
Fortune Shop	Green Intelligent Power Saver	The real thing matches the picture and looks great!	Jingdong prefecture level city in Shandong
Fortune Shop	Green Intelligent Power Saver	Plugged it in and we'll see when we pay the bills at the end of the year!	Jingdong prefecture level city in Shandong
Fortune Shop	Green Intelligent Power Saver	False advertising. It doesn't work.	Jingdong prefecture level city in Shandong
Fortune Shop	Green Intelligent Power Saver	Goods received, good effect	Jingdong prefecture level city in Shandong
Fortune Shop	Green Intelligent Power Saver	It looks okay, we'll see if it saves electricity when the time comes	Jingdong prefecture level city in Shandong
Fortune Shop	Green Intelligent Power Saver	Logistics is very fast quality is very good, the price is very affordable, accessories are also complete, installed, satisfied with good use	Jingdong prefecture level city in Shandong
Fortune Shop	Green Intelligent Power Saver	I've received the power saver. I'm looking forward to the savings.	Jingdong prefecture level city in Shandong
Fortune Shop	Green Intelligent Power Saver	Received, have not yet used, after use and then evaluate	Jingdong prefecture level city in Shandong
Fortune Shop	Green Intelligent Power Saver	I received the item, but I don't know if it saves power or not.	Jingdong prefecture level city in Shandong
Fortune Shop	Green Intelligent Power Saver	On Trial	Jingdong prefecture level city in Shandong
Fortune Shop	Green Intelligent Power Saver	Use it and see how it goes.	Jingdong prefecture level city in Shandong
Fortune Shop	Green Intelligent Power Saver	Quality is very good, no odor, fine workmanship, grain color are very like, satisfied!	Jingdong prefecture level city in Shandong
Fortune Shop	Green Intelligent Power Saver	I'll give you a good review. I don't know if it'll work.	Jingdong prefecture level city in Shandong
Fortune Shop	Green Intelligent Power Saver	The logistics is very fast and the package is intact. Good use of affordable. The effect is very good, is genuine	Jingdong prefecture level city in Shandong
Fortune Shop	Green Intelligent Power Saver	Nothing happens when you plug it in.	Jingdong prefecture level city in Shandong

Fortune Shop	Green Intelligent Power Saver	I don't know if it's as good as it sounds.	Jingdong prefecture level city in Shandong
Fortune Shop	Green Intelligent Power Saver	The quality of the stuff is very good, exactly the same as the seller's description, very satisfied!	Jingdong prefecture level city in Shandong
Fortune Shop	Green Intelligent Power Saver	The saver works great, it really works, it's affordable and worth buying.	Jingdong prefecture level city in Shandong
Fortune Shop	Green Intelligent Power Saver	Just used it for a couple days. I don't know yet.	Jingdong prefecture level city in Shandong
Fortune Shop	Green Intelligent Power Saver	The quality is quite good, cheaper than the physical store, good, next time will come again!	Jingdong prefecture level city in Shandong
Fortune Shop	Green Intelligent Power Saver	Good quality, cheap and affordable worth recommending to buy!	Jingdong prefecture level city in Shandong
Fortune Shop	Green Intelligent Power Saver	Very good quality, well made, works well! Satisfied	Jingdong prefecture level city in Shandong
Fortune Shop	Green Intelligent Power Saver	The logistics is very fast quality is very good very pleasant a shopping	Jingdong prefecture level city in Shandong
Fortune Shop	Green Intelligent Power Saver	Use it and then come back to evaluate!	Jingdong prefecture level city in Shandong
Fortune Shop	Green Intelligent Power Saver	# It's no use lying at all #	Jingdong prefecture level city in Shandong
Fortune Shop	Green Intelligent Power Saver	I can't get a voltage drop.	Jingdong prefecture level city in Shandong
Fortune Shop	Green Intelligent Power Saver	Evaluate after use	Jingdong prefecture level city in Shandong
Fortune Shop	Green Intelligent Power Saver	Still on trial.	Jingdong prefecture level city in Shandong
Fortune Shop	Green Intelligent Power Saver	ineffective	Jingdong prefecture level city in Shandong
Fortune Shop	Green Intelligent Power Saver	Satisfaction Shopping	Jingdong prefecture level city in Shandong
Fortune Shop	Green Intelligent Power Saver	Lies.	Jingdong prefecture level city in Shandong
Fortune Shop	Green Intelligent Power Saver	It's a real power saver.	Jingdong prefecture level city in Shandong
Fortune Shop	Green Intelligent Power Saver	in use	Jingdong prefecture level city in Shandong
Fortune Shop	Green Intelligent Power Saver	I don't know yet.	Jingdong prefecture level city in Shandong
Fortune Shop	Green Intelligent Power Saver	not care about anything	Jingdong prefecture level city in Shandong
Fortune Shop	Green Intelligent Power Saver	It doesn't seem to have much effect	Jingdong prefecture level city in Shandong
Fortune Shop	Green Intelligent Power Saver	Let the merchant see for himself, does the plug positive and negative terminals look like this?	Jingdong prefecture level city in Shandong
Fortune Shop	Green Intelligent Power Saver	It doesn't work well, the electricity bill is less before using it, but more after using it, contacting customer service, the service is still not good, and returning it is slow	Jingdong prefecture level city in Shandong
Fortune Shop	Green Intelligent Power Saver	With the advertisement product does not match, above the effective negative, power saving rate of two rows of words it, the product looks very thin, with 3 days to see the meter number is still with the previous no difference, which is obviously fraudulent behavior!	Jingdong prefecture level city in Shandong
Fortune Shop	Green Intelligent Power Saver	Doesn't save any power at all. Not much use.	Jingdong prefecture level city in Shandong
Fortune Shop	Green Intelligent Power Saver	The plugs are all loose, they're all the same length and they don't work at all	Jingdong prefecture level city in Shandong
Fortune Shop	Green Intelligent Power Saver	It's bad 👎 it's not effective at all, you'd be crazy to sell this garbage on the internet.	Jingdong prefecture level city in Shandong
Fortune Shop	Green Intelligent Power Saver	It doesn't save any power at all, so I hope the old timers don't fall for this scam again	Jingdong prefecture level city in Shandong
Fortune Shop	Green Intelligent Power Saver	I've been using it for over a month now and haven't noticed any power savings.	Jingdong prefecture level city in Shandong
Fortune Shop	Green Intelligent Power Saver	It doesn't work at all. The electricity bill is higher when you use it.	Jingdong prefecture level city in Shandong
Fortune Shop	Green Intelligent Power Saver	It doesn't save any power, it's still as bad as ever.	Jingdong prefecture level city in Shandong
Fortune Shop	Green Intelligent Power Saver	Last night before leaving work to look at the table, and then plugged into the node device, this morning and then look at the table, the original save a few degrees of electricity, very good to have a power-saving effect, easy to use, satisfied with the shopping praise.	Jingdong prefecture level city in Shandong
Fortune Shop	Green Intelligent Power Saver	The product is real and effective, the electricity bill is reduced, really good, thanks to the merchants have so many good products, the product is real and effective, the electricity bill is reduced, want to save electricity friends quickly buy it, early use early to save money.	Jingdong prefecture level city in Shandong
Fortune Shop	Green Intelligent Power Saver	Saver saves time and money, baby appearance is beautiful, the quality of goods is good, and the same as the business description, good service attitude, packaging is complete, logistics is very fast, praise to	Jingdong prefecture level city in Shandong
Fortune Shop	Green Intelligent Power Saver	The effect is good, summer, home electricity is relatively large, buy and use a few days, feel the effect is very good, originally ten dollars a day electricity bill, now eight dollars a day, quite good	Jingdong prefecture level city in Shandong
Fortune Shop	Green Intelligent Power Saver	Indeed can save electricity and money, the first time I know this or from a friend's home to see, but also do not believe it, and now they use it to know that there is this good thing.	Jingdong prefecture level city in Shandong
Fortune Shop	Green Intelligent Power Saver	I have received the Smart Power Saver. Perfectly packaged. Fast delivery. Boss customer service attitude is also very good. It's worth buying. I plugged it in and tried it and it really saves more power than the original. I'll buy it again next time.	Jingdong prefecture level city in Shandong
Fortune Shop	Green Intelligent Power Saver	It's nice to save a dozen bucks a night on two air conditioners that used to take 30 or 40 degrees a night, but now it's only 10 or so degrees a night	Jingdong prefecture level city in Shandong
Fortune Shop	Green Intelligent Power Saver	Product no manufacturer's name no product qualification certificate, try two days to see the effect of	Jingdong prefecture level city in Shandong
Fortune Shop	Green Intelligent Power Saver	I've been using it for over a month and have saved over $80, so I've repurchased a few more back to save together	Jingdong prefecture level city in Shandong
Fortune Shop	Green Intelligent Power Saver	I haven't used it yet and will comment when I do, but it's well made.	Jingdong prefecture level city in Shandong
Fortune Shop	Green Intelligent Power Saver	It's quite energy efficient. It's really a boon for the home.	Jingdong prefecture level city in Shandong
Fortune Shop	Green Intelligent Power Saver	Energy-saving socket received a month, after using it for a month proved that it is indeed energy-saving, electricity bill saving, will buy it again, thank you!	Jingdong prefecture level city in Shandong
Fortune Shop	Green Intelligent Power Saver	There was a similar product many years ago. Now technology has evolved, buy one to try, according to the instructions in the box, it takes one or two dozen days to show results. Check your electric bill and see what happens.	Jingdong prefecture level city in Shandong
Fortune Shop	Green Intelligent Power Saver	Jingdong logistics is really fast, the same day the order arrived on the same day, more convenient than the supermarket, and a wide range of choices, the	Jingdong prefecture level city in Shandong
Fortune Shop	Green Intelligent Power Saver	I've been using this for a month or so and I can't see any results, it really is a scam as they say.	Jingdong prefecture level city in Shandong
Fortune Shop	Green Intelligent Power Saver	Very good use, a month can save a few degrees of power loss of electricity, introduced to the relatives to buy back to try.	Jingdong prefecture level city in Shandong
Fortune Shop	Green Intelligent Power Saver	It's really powerful! With this air conditioner can be turned on at any time without the pressure of paying the electricity bill! It's good for the people!	Jingdong prefecture level city in Shandong

Fortune Shop	Green Intelligent Power Saver	This business is a big ** products are not qualified, with a month of electricity more than one hundred percent of thirty, the purchase agreed to return the goods but do not receive a refund after receiving the goods, looking for the official side does not help, the Jingdong is a big **, I have never shopped in	Jingdong prefecture level city in Shandong
Fortune Shop	Green Intelligent Power Saver	It's good. I tried it. It saves power.	Jingdong prefecture level city in Shandong
Fortune Shop	Green Intelligent Power Saver	Nothing**, it seems like there's more	Jingdong prefecture level city in Shandong
Fortune Shop	Green Intelligent Power Saver	Power saving device received, well packaged, good quality, fast logistics	Jingdong prefecture level city in Shandong
Fortune Shop	Green Intelligent Power Saver	The service is okay, is that when I bought five, the desirable pieces are three, and then wait a few days and send me two replacement, I do not know if it is a mistake of the shipper, if I was not in the courier there to see, go home to say no, the	Jingdong prefecture level city in Shandong
Fortune Shop	Green Intelligent Power Saver	This thing will only cost me more if I use it, but before I used it, it was less than a ~ electricity a month, and after I used it, it was more than two hundred kWh a month.	Jingdong prefecture level city in Shandong
Fortune Shop	Green Intelligent Power Saver	I just used it. I don't know how it is.	Jingdong prefecture level city in Shandong
Fortune Shop	Green Intelligent Power Saver	I've received the product and I'm using it, so I'll see how it goes in a month!	Jingdong prefecture level city in Shandong
Fortune Shop	Green Intelligent Power Saver	The goods have just received the effect do not know how, please look forward to the results.	Jingdong prefecture level city in Shandong
Fortune Shop	Green Intelligent Power Saver	Power saving device, the material is very good practical and durable, the size is suitable, packaging is very good, the price is cheap, logistics is very fast!	Jingdong prefecture level city in Shandong
Fortune Shop	Green Intelligent Power Saver	Very nice, will comment in detail after using it for a while.	Jingdong prefecture level city in Shandong
Fortune Shop	Green Intelligent Power Saver	Good stuff to trust and support	Jingdong prefecture level city in Shandong
Fortune Shop	Green Intelligent Power Saver	I'll comment after I use it, doubt if it really saves power no	Jingdong prefecture level city in Shandong
Fortune Shop	Green Intelligent Power Saver	It's good quality and works well!	Jingdong prefecture level city in Shandong
Fortune Shop	Green Intelligent Power Saver	hahaha just right hahahahahaha hiccup hahahaha	Jingdong prefecture level city in Shandong
Fortune Shop	Green Intelligent Power Saver	The real thing matches the picture and looks great!	Jingdong prefecture level city in Shandong
Fortune Shop	Green Intelligent Power Saver	Plugged it in and we'll see when we pay the bills at the end of the year!	Jingdong prefecture level city in Shandong
Fortune Shop	Green Intelligent Power Saver	False advertising. It doesn't work.	Jingdong prefecture level city in Shandong
Fortune Shop	Green Intelligent Power Saver	Goods received, good effect	Jingdong prefecture level city in Shandong
Fortune Shop	Green Intelligent Power Saver	It looks okay, we'll see if it saves electricity when the time comes	Jingdong prefecture level city in Shandong
Fortune Shop	Green Intelligent Power Saver	Logistics is very fast quality is very good, the price is very affordable, accessories are also complete, installed, satisfied with good use	Jingdong prefecture level city in Shandong
Fortune Shop	Green Intelligent Power Saver	I've received the power saver. I'm looking forward to the savings.	Jingdong prefecture level city in Shandong
Fortune Shop	Green Intelligent Power Saver	Received, have not yet used, after use and then evaluate	Jingdong prefecture level city in Shandong
Fortune Shop	Green Intelligent Power Saver	I received the item, but I don't know if it saves power or not.	Jingdong prefecture level city in Shandong
Fortune Shop	Green Intelligent Power Saver	On Trial	Jingdong prefecture level city in Shandong
Fortune Shop	Green Intelligent Power Saver	Use it and see how it goes.	Jingdong prefecture level city in Shandong
Fortune Shop	Green Intelligent Power Saver	Quality is very good, no odor, fine workmanship, grain color are very like, satisfied!	Jingdong prefecture level city in Shandong
Fortune Shop	Green Intelligent Power Saver	I'll give you a good review. I don't know if it'll work.	Jingdong prefecture level city in Shandong
Fortune Shop	Green Intelligent Power Saver	The logistics is very fast and the package is intact. Good use of affordable. The effect is very good, is genuine	Jingdong prefecture level city in Shandong
Fortune Shop	Green Intelligent Power Saver	Nothing happens when you plug it in.	Jingdong prefecture level city in Shandong
Fortune Shop	Green Intelligent Power Saver	I don't know if it's as good as it sounds.	Jingdong prefecture level city in Shandong
Fortune Shop	Green Intelligent Power Saver	The quality of the stuff is very good, exactly the same as the seller's description, very satisfied!	Jingdong prefecture level city in Shandong
Fortune Shop	Green Intelligent Power Saver	The saver works great, it really works, it's affordable and worth buying.	Jingdong prefecture level city in Shandong
Fortune Shop	Green Intelligent Power Saver	Just used it for a couple days. I don't know yet.	Jingdong prefecture level city in Shandong
Fortune Shop	Green Intelligent Power Saver	The quality is quite good, cheaper than the physical store, good, next time will come again!	Jingdong prefecture level city in Shandong
Fortune Shop	Green Intelligent Power Saver	Good quality, cheap and affordable worth recommending to buy!	Jingdong prefecture level city in Shandong
Fortune Shop	Green Intelligent Power Saver	Very good quality, well made, works well! Satisfied	Jingdong prefecture level city in Shandong
Fortune Shop	Green Intelligent Power Saver	The logistics is very fast quality is very good very pleasant a shopping	Jingdong prefecture level city in Shandong
Fortune Shop	Green Intelligent Power Saver	Use it and then come back to evaluate!	Jingdong prefecture level city in Shandong
Fortune Shop	Green Intelligent Power Saver	# It's no use lying at all #	Jingdong prefecture level city in Shandong
Fortune Shop	Green Intelligent Power Saver	I can't get a voltage drop.	Jingdong prefecture level city in Shandong
Fortune Shop	Green Intelligent Power Saver	Evaluate after use	Jingdong prefecture level city in Shandong
Fortune Shop	Green Intelligent Power Saver	Still on trial.	Jingdong prefecture level city in Shandong
Fortune Shop	Green Intelligent Power Saver	ineffective	Jingdong prefecture level city in Shandong
Fortune Shop	Green Intelligent Power Saver	Satisfaction Shopping	Jingdong prefecture level city in Shandong
Fortune Shop	Green Intelligent Power Saver	Lies.	Jingdong prefecture level city in Shandong
Fortune Shop	Green Intelligent Power Saver	It's a real power saver.	Jingdong prefecture level city in Shandong
Fortune Shop	Green Intelligent Power Saver	in use	Jingdong prefecture level city in Shandong
Fortune Shop	Green Intelligent Power Saver	I don't know yet.	Jingdong prefecture level city in Shandong
Fortune Shop	Green Intelligent Power Saver	not care about anything	Jingdong prefecture level city in Shandong

Fortune Shop	Green Intelligent Power Saver	It doesn't seem to have much effect	Jingdong prefecture level city in Shandong
Fortune Shop	Green Intelligent Power Saver	Let the merchant see for himself, does the plug positive and negative terminals look like this?	Jingdong prefecture level city in Shandong
Fortune Shop	Green Intelligent Power Saver	It doesn't work well, the electricity bill is less before using it, but more after using it, contacting customer service, the service is still not good, and returning it is slow	Jingdong prefecture level city in Shandong
Fortune Shop	Green Intelligent Power Saver	With the advertisement product does not match, above the effective negative, power saving rate of two rows of words it, the product looks very thin, with 3 days to see the meter number is still with the previous no difference, which is obviously fraudulent behavior!	Jingdong prefecture level city in Shandong
Fortune Shop	Green Intelligent Power Saver	Doesn't save any power at all. Not much use.	Jingdong prefecture level city in Shandong
Fortune Shop	Green Intelligent Power Saver	The plugs are all loose, they're all the same length and they don't work at all	Jingdong prefecture level city in Shandong
Fortune Shop	Green Intelligent Power Saver	It's bad 👎 it's not effective at all, you'd be crazy to sell this garbage on the internet.	Jingdong prefecture level city in Shandong
Fortune Shop	Green Intelligent Power Saver	It doesn't save any power at all, so I hope the old timers don't fall for this scam again	Jingdong prefecture level city in Shandong
Fortune Shop	Green Intelligent Power Saver	I've been using it for over a month now and haven't noticed any power savings.	Jingdong prefecture level city in Shandong
Fortune Shop	Green Intelligent Power Saver	It doesn't work at all. The electricity bill is higher when you use it.	Jingdong prefecture level city in Shandong
Fortune Shop	Green Intelligent Power Saver	It doesn't save any power, it's still as bad as ever.	Jingdong prefecture level city in Shandong
Fortune Shop	Green Intelligent Power Saver	Last night before leaving work to look at the table, and then plugged into the node device, this morning and then look at the table, than the original save a few degrees of electricity, very good to have a power-saving effect, easy to use, satisfied with the shopping praise.	Jingdong prefecture level city in Shandong
Fortune Shop	Green Intelligent Power Saver	The product is real and effective, the electricity bill is reduced, really good, thanks to the merchants have so many good products, the product is real and effective, the electricity bill is reduced, want to save electricity friends quickly buy it, early use early to save money.	Jingdong prefecture level city in Shandong
Fortune Shop	Green Intelligent Power Saver	Saver saves time and money, baby appearance is beautiful, the quality of goods is good, and the same as the business description, good service attitude, packaging is complete, logistics is very fast, praise to	Jingdong prefecture level city in Shandong
Fortune Shop	Green Intelligent Power Saver	The effect is good, summer, home electricity is relatively large, buy and use a few days, feel the effect is very good, originally ten dollars a day electricity bill, now eight dollars a day, quite good	Jingdong prefecture level city in Shandong
Fortune Shop	Green Intelligent Power Saver	Indeed can save electricity and money, the first time I know this or from a friend's home to see, but also do not believe it, and now they use it to know that there is this good thing.	Jingdong prefecture level city in Shandong
Fortune Shop	Green Intelligent Power Saver	I have received the Smart Power Saver. Perfectly packaged. Fast delivery. Boss customer service attitude is also very good. It's worth buying. I plugged it in and tried it and it really saves more power than the original. I'll buy it again next time.	Jingdong prefecture level city in Shandong
Fortune Shop	Green Intelligent Power Saver	It's nice to save a dozen bucks a night on two air conditioners that used to take 30 or 40 degrees a night, but now it's only 10 or so degrees a night	Jingdong prefecture level city in Shandong
Fortune Shop	Green Intelligent Power Saver	Product no manufacturer's name no product qualification certificate, try two days to see the effect of	Jingdong prefecture level city in Shandong
Fortune Shop	Green Intelligent Power Saver	I've been using it for over a month and have saved over $80, so I've repurchased a few more back to save together	Jingdong prefecture level city in Shandong
Fortune Shop	Green Intelligent Power Saver	I haven't used it yet and will comment when I do, but it's well made.	Jingdong prefecture level city in Shandong
Fortune Shop	Green Intelligent Power Saver	It's quite energy efficient. It's really a boon for the home.	Jingdong prefecture level city in Shandong
Fortune Shop	Green Intelligent Power Saver	Energy-saving socket received a month, after using it for a month proved that it is indeed energy-saving, electricity bill saving, will buy it again, thank you!	Jingdong prefecture level city in Shandong
Fortune Shop	Green Intelligent Power Saver	There was a similar product many years ago. Now technology has evolved, buy one to try, according to the instructions in the box, it takes one or two dozen days to show results. Check your electric bill and see what happens.	Jingdong prefecture level city in Shandong
Fortune Shop	Green Intelligent Power Saver	Jingdong logistics is really fast, the same day the order arrived on the same day, more convenient than the supermarket, and a wide range of choices, the	Jingdong prefecture level city in Shandong
Fortune Shop	Green Intelligent Power Saver	I've been using this for a month or so and I can't see any results, it really is a scam as they say.	Jingdong prefecture level city in Shandong
Fortune Shop	Green Intelligent Power Saver	Very good use, a month can save a few degrees of power loss of electricity, introduced to the relatives to buy back to try.	Jingdong prefecture level city in Shandong
Fortune Shop	Green Intelligent Power Saver	It's really powerful! With this air conditioner can be turned on at any time without the pressure of paying the electricity bill! It's good for the people!	Jingdong prefecture level city in Shandong
Fortune Shop	Green Intelligent Power Saver	This business is a big ** products are not qualified, with a month of electricity more than one hundred percent of thirty, the purchase agreed to return the goods but do not receive a refund after receiving the goods, looking for the official side does not help, the Jingdong is a big **, I have never shopped in	Jingdong prefecture level city in Shandong
Fortune Shop	Green Intelligent Power Saver	It's good. I tried it. It saves power.	Jingdong prefecture level city in Shandong
Fortune Shop	Green Intelligent Power Saver	Nothing**, it seems like there's more	Jingdong prefecture level city in Shandong
Fortune Shop	Green Intelligent Power Saver	Power saving device received, well packaged, good quality, fast logistics	Jingdong prefecture level city in Shandong
Fortune Shop	Green Intelligent Power Saver	The service is okay, is that when I bought five, the desirable pieces are three, and then wait a few days and send me two replacement, I do not know if it is a mistake of the shipper, if I was not in the courier there to see, go home to say no, the	Jingdong prefecture level city in Shandong
Fortune Shop	Green Intelligent Power Saver	This thing will only cost me more if I use it, but before I used it, it was less than a ~ electricity a month, and after I used it, it was more than two hundred kWh a month.	Jingdong prefecture level city in Shandong
Fortune Shop	Green Intelligent Power Saver	I just used it. I don't know how it is.	Jingdong prefecture level city in Shandong
Fortune Shop	Green Intelligent Power Saver	I've received the product and I'm using it, so I'll see how it goes in a month!	Jingdong prefecture level city in Shandong
Fortune Shop	Green Intelligent Power Saver	The goods have just received the effect do not know how, please look forward to the results.	Jingdong prefecture level city in Shandong
Fortune Shop	Green Intelligent Power Saver	Power saving device, the material is very good practical and durable, the size is suitable, packaging is very good, the price is cheap, logistics is very fast!	Jingdong prefecture level city in Shandong
Fortune Shop	Green Intelligent Power Saver	Very nice, will comment in detail after using it for a while.	Jingdong prefecture level city in Shandong
Fortune Shop	Green Intelligent Power Saver	Good stuff to trust and support	Jingdong prefecture level city in Shandong
Fortune Shop	Green Intelligent Power Saver	I'll comment after I use it, doubt if it really saves power no	Jingdong prefecture level city in Shandong
Fortune Shop	Green Intelligent Power Saver	It's good quality and works well!	Jingdong prefecture level city in Shandong
Fortune Shop	Green Intelligent Power Saver	hahaha just right hahahahahaha hiccup hahahaha	Jingdong prefecture level city in Shandong
Fortune Shop	Green Intelligent Power Saver	The real thing matches the picture and looks great!	Jingdong prefecture level city in Shandong
Fortune Shop	Green Intelligent Power Saver	Plugged it in and we'll see when we pay the bills at the end of the year!	Jingdong prefecture level city in Shandong
Fortune Shop	Green Intelligent Power Saver	False advertising. It doesn't work.	Jingdong prefecture level city in Shandong
Fortune Shop	Green Intelligent Power Saver	Goods received, good effect	Jingdong prefecture level city in Shandong
Fortune Shop	Green Intelligent Power Saver	It looks okay, we'll see if it saves electricity when the time comes	Jingdong prefecture level city in Shandong

Fortune Shop	Green Intelligent Power Saver	Logistics is very fast quality is very good, the price is very affordable, accessories are also complete, installed, satisfied with good use	Jingdong prefecture level city in Shandong
Fortune Shop	Green Intelligent Power Saver	I've received the power saver. I'm looking forward to the savings.	Jingdong prefecture level city in Shandong
Fortune Shop	Green Intelligent Power Saver	Received, have not yet used, after use and then evaluate	Jingdong prefecture level city in Shandong
Fortune Shop	Green Intelligent Power Saver	I received the item, but I don't know if it saves power or not.	Jingdong prefecture level city in Shandong
Fortune Shop	Green Intelligent Power Saver	On Trial	Jingdong prefecture level city in Shandong
Fortune Shop	Green Intelligent Power Saver	Use it and see how it goes.	Jingdong prefecture level city in Shandong
Fortune Shop	Green Intelligent Power Saver	Quality is very good, no odor, fine workmanship, grain color are very like, satisfied!	Jingdong prefecture level city in Shandong
Fortune Shop	Green Intelligent Power Saver	I'll give you a good review. I don't know if it'll work.	Jingdong prefecture level city in Shandong
Fortune Shop	Green Intelligent Power Saver	The logistics is very fast and the package is intact. Good use of affordable. The effect is very good, is genuine	Jingdong prefecture level city in Shandong
Fortune Shop	Green Intelligent Power Saver	Nothing happens when you plug it in.	Jingdong prefecture level city in Shandong
Fortune Shop	Green Intelligent Power Saver	I don't know if it's as good as it sounds.	Jingdong prefecture level city in Shandong
Fortune Shop	Green Intelligent Power Saver	The quality of the stuff is very good, exactly the same as the seller's description, very satisfied!	Jingdong prefecture level city in Shandong
Fortune Shop	Green Intelligent Power Saver	The saver works great, it really works, it's affordable and worth buying.	Jingdong prefecture level city in Shandong
Fortune Shop	Green Intelligent Power Saver	Just used it for a couple days. I don't know yet.	Jingdong prefecture level city in Shandong
Fortune Shop	Green Intelligent Power Saver	The quality is quite good, cheaper than the physical store, good, next time will come again!	Jingdong prefecture level city in Shandong
Fortune Shop	Green Intelligent Power Saver	Good quality, cheap and affordable worth recommending to buy!	Jingdong prefecture level city in Shandong
Fortune Shop	Green Intelligent Power Saver	Very good quality, well made, works well! Satisfied	Jingdong prefecture level city in Shandong
Fortune Shop	Green Intelligent Power Saver	The logistics is very fast quality is very good very pleasant a shopping	Jingdong prefecture level city in Shandong
Fortune Shop	Green Intelligent Power Saver	Use it and then come back to evaluate!	Jingdong prefecture level city in Shandong
Fortune Shop	Green Intelligent Power Saver	# It's no use lying at all #	Jingdong prefecture level city in Shandong
Fortune Shop	Green Intelligent Power Saver	I can't get a voltage drop.	Jingdong prefecture level city in Shandong
Fortune Shop	Green Intelligent Power Saver	Evaluate after use	Jingdong prefecture level city in Shandong
Fortune Shop	Green Intelligent Power Saver	Still on trial.	Jingdong prefecture level city in Shandong
Fortune Shop	Green Intelligent Power Saver	ineffective	Jingdong prefecture level city in Shandong
Fortune Shop	Green Intelligent Power Saver	Satisfaction Shopping	Jingdong prefecture level city in Shandong
Fortune Shop	Green Intelligent Power Saver	Lies.	Jingdong prefecture level city in Shandong
Fortune Shop	Green Intelligent Power Saver	It's a real power saver.	Jingdong prefecture level city in Shandong
Fortune Shop	Green Intelligent Power Saver	in use	Jingdong prefecture level city in Shandong
Fortune Shop	Green Intelligent Power Saver	I don't know yet.	Jingdong prefecture level city in Shandong
Fortune Shop	Green Intelligent Power Saver	not care about anything	Jingdong prefecture level city in Shandong
Fortune Shop	Green Intelligent Power Saver	It doesn't seem to have much effect	Jingdong prefecture level city in Shandong
Fortune Shop	Green Intelligent Power Saver	Let the merchant see for himself, does the plug positive and negative terminals look like this?	Jingdong prefecture level city in Shandong
Fortune Shop	Green Intelligent Power Saver	It doesn't work well, the electricity bill is less before using it, but more after using it, contacting customer service, the service is still not good, and returning it is slow	Jingdong prefecture level city in Shandong
Fortune Shop	Green Intelligent Power Saver	With the advertisement product does not match, above the effective negative, power saving rate of two rows of words it, the product looks very thin, with 3 days to see the meter number is still with the previous no difference, which is obviously fraudulent behavior!	Jingdong prefecture level city in Shandong
Fortune Shop	Green Intelligent Power Saver	Doesn't save any power at all. Not much use.	Jingdong prefecture level city in Shandong
Fortune Shop	Green Intelligent Power Saver	The plugs are all loose, they're all the same length and they don't work at all	Jingdong prefecture level city in Shandong
Fortune Shop	Green Intelligent Power Saver	It's bad 👎 it's not effective at all, you'd be crazy to sell this garbage on the internet.	Jingdong prefecture level city in Shandong
Fortune Shop	Green Intelligent Power Saver	It doesn't save any power at all, so I hope the old timers don't fall for this scam again	Jingdong prefecture level city in Shandong
Fortune Shop	Green Intelligent Power Saver	I've been using it for over a month now and haven't noticed any power savings.	Jingdong prefecture level city in Shandong
Fortune Shop	Green Intelligent Power Saver	It doesn't work at all. The electricity bill is higher when you use it.	Jingdong prefecture level city in Shandong
Fortune Shop	Green Intelligent Power Saver	It doesn't save any power, it's still as bad as ever.	Jingdong prefecture level city in Shandong
Fortune Shop	Green Intelligent Power Saver	Last night before leaving work to look at the table, and then plugged into the node device, this morning and then look at the table, than the original save a few degrees of electricity, very good to have a power-saving effect, easy to use, satisfied with the shopping praise.	Jingdong prefecture level city in Shandong
Fortune Shop	Green Intelligent Power Saver	The product is real and effective, the electricity bill is reduced, really good, thanks to the merchants have so many good products, the product is real and effective, the electricity bill is reduced, want to save electricity friends quickly buy it, early use early to save money.	Jingdong prefecture level city in Shandong
Fortune Shop	Green Intelligent Power Saver	Saver saves time and money, baby appearance is beautiful, the quality of goods is good, and the same as the business description, good service attitude, packaging is complete, logistics is very fast, praise to	Jingdong prefecture level city in Shandong
Fortune Shop	Green Intelligent Power Saver	The effect is good, summer, home electricity is relatively large, buy and use a few days, feel the effect is very good, originally ten dollars a day electricity bill, now eight dollars a day, quite good	Jingdong prefecture level city in Shandong
Fortune Shop	Green Intelligent Power Saver	Indeed can save electricity and money, the first time I know this or from a friend's home to see, but also do not believe it, and now they use it to know that there is this good thing.	Jingdong prefecture level city in Shandong
Fortune Shop	Green Intelligent Power Saver	I have received the Smart Power Saver. Perfectly packaged. Fast delivery. Boss customer service attitude is also very good. It's worth buying. I plugged it in and tried it and it really saves more power than the original. I'll buy it again next time.	Jingdong prefecture level city in Shandong
Fortune Shop	Green Intelligent Power Saver	It's nice to save a dozen bucks a night on two air conditioners that used to take 30 or 40 degrees a night, but now it's only 10 or so degrees a night	Jingdong prefecture level city in Shandong
Fortune Shop	Green Intelligent Power Saver	Product no manufacturer's name no product qualification certificate, try two days to see the effect of	Jingdong prefecture level city in Shandong

Fortune Shop	Green Intelligent Power Saver	I've been using it for over a month and have saved over $80, so I've repurchased a few more back to save together	Jingdong prefecture level city in Shandong
Fortune Shop	Green Intelligent Power Saver	I haven't used it yet and will comment when I do, but it's well made.	Jingdong prefecture level city in Shandong
Fortune Shop	Green Intelligent Power Saver	It's quite energy efficient. It's really a boon for the home.	Jingdong prefecture level city in Shandong
Fortune Shop	Green Intelligent Power Saver	Energy-saving socket received a month, after using it for a month proved that it is indeed energy-saving, electricity bill saving, will buy it again, thank you!	Jingdong prefecture level city in Shandong
Fortune Shop	Green Intelligent Power Saver	There was a similar product many years ago. Now technology has evolved, buy one to try, according to the instructions in the box, it takes one or two dozen days to show results. Check your electric bill and see what happens.	Jingdong prefecture level city in Shandong
Fortune Shop	Green Intelligent Power Saver	Jingdong logistics is really fast, the same day the order arrived on the same day, more convenient than the supermarket, and a wide range of choices, the	Jingdong prefecture level city in Shandong
Fortune Shop	Green Intelligent Power Saver	I've been using this for a month or so and I can't see any results, it really is a scam as they say.	Jingdong prefecture level city in Shandong
Fortune Shop	Green Intelligent Power Saver	Very good use, a month can save a few degrees of power loss of electricity, introduced to the relatives to buy back to try.	Jingdong prefecture level city in Shandong
Fortune Shop	Green Intelligent Power Saver	It's really powerful! With this air conditioner can be turned on at any time without the pressure of paying the electricity bill! It's good for the people!	Jingdong prefecture level city in Shandong
Fortune Shop	Green Intelligent Power Saver	This business is a big ** products are not qualified, with a month of electricity more than one hundred percent of thirty, the purchase agreed to return the goods but do not receive a refund after receiving the goods, looking for the official side does not help, the Jingdong is a big **, I have never shopped in	Jingdong prefecture level city in Shandong
Fortune Shop	Green Intelligent Power Saver	It's good. I tried it. It saves power.	Jingdong prefecture level city in Shandong
Fortune Shop	Green Intelligent Power Saver	Nothing**, it seems like there's more	Jingdong prefecture level city in Shandong
Fortune Shop	Green Intelligent Power Saver	Power saving device received, well packaged, good quality, fast logistics	Jingdong prefecture level city in Shandong
Fortune Shop	Green Intelligent Power Saver	The service is okay, is that when I bought five, the desirable pieces are three, and then wait a few days and send me two replacement, I do not know if it is a mistake of the shipper, if I was not in the courier there to see, go home to say no, the	Jingdong prefecture level city in Shandong
Fortune Shop	Green Intelligent Power Saver	This thing will only cost me more if I use it, but before I used it, it was less than a ~ electricity a month, and after I used it, it was more than two hundred kWh a month.	Jingdong prefecture level city in Shandong
Fortune Shop	Green Intelligent Power Saver	I just used it. I don't know how it is.	Jingdong prefecture level city in Shandong
Fortune Shop	Green Intelligent Power Saver	I've received the product and I'm using it, so I'll see how it goes in a month!	Jingdong prefecture level city in Shandong
Fortune Shop	Green Intelligent Power Saver	The goods have just received the effect do not know how, please look forward to the results.	Jingdong prefecture level city in Shandong
Fortune Shop	Green Intelligent Power Saver	Power saving device, the material is very good practical and durable, the size is suitable, packaging is very good, the price is cheap, logistics is very fast!	Jingdong prefecture level city in Shandong
Fortune Shop	Green Intelligent Power Saver	Very nice, will comment in detail after using it for a while.	Jingdong prefecture level city in Shandong
Fortune Shop	Green Intelligent Power Saver	Good stuff to trust and support	Jingdong prefecture level city in Shandong
Fortune Shop	Green Intelligent Power Saver	I'll comment after I use it, doubt if it really saves power no	Jingdong prefecture level city in Shandong
Fortune Shop	Green Intelligent Power Saver	It's good quality and works well!	Jingdong prefecture level city in Shandong
Fortune Shop	Green Intelligent Power Saver	hahaha just right hahahahahaha hiccup hahahaha	Jingdong prefecture level city in Shandong
Fortune Shop	Green Intelligent Power Saver	The real thing matches the picture and looks great!	Jingdong prefecture level city in Shandong
Fortune Shop	Green Intelligent Power Saver	Plugged it in and we'll see when we pay the bills at the end of the year!	Jingdong prefecture level city in Shandong
Fortune Shop	Green Intelligent Power Saver	False advertising. It doesn't work.	Jingdong prefecture level city in Shandong
Fortune Shop	Green Intelligent Power Saver	Goods received, good effect	Jingdong prefecture level city in Shandong
Fortune Shop	Green Intelligent Power Saver	It looks okay, we'll see if it saves electricity when the time comes	Jingdong prefecture level city in Shandong
Fortune Shop	Green Intelligent Power Saver	Logistics is very fast quality is very good, the price is very affordable, accessories are also complete, installed, satisfied with good use	Jingdong prefecture level city in Shandong
Fortune Shop	Green Intelligent Power Saver	I've received the power saver. I'm looking forward to the savings.	Jingdong prefecture level city in Shandong
Fortune Shop	Green Intelligent Power Saver	Received, have not yet used, after use and then evaluate	Jingdong prefecture level city in Shandong
Fortune Shop	Green Intelligent Power Saver	I received the item, but I don't know if it saves power or not.	Jingdong prefecture level city in Shandong
Fortune Shop	Green Intelligent Power Saver	On Trial	Jingdong prefecture level city in Shandong
Fortune Shop	Green Intelligent Power Saver	Use it and see how it goes.	Jingdong prefecture level city in Shandong
Fortune Shop	Green Intelligent Power Saver	Quality is very good, no odor, fine workmanship, grain color are very like, satisfied!	Jingdong prefecture level city in Shandong
Fortune Shop	Green Intelligent Power Saver	I'll give you a good review. I don't know if it'll work.	Jingdong prefecture level city in Shandong
Fortune Shop	Green Intelligent Power Saver	The logistics is very fast and the package is intact. Good use of affordable. The effect is very good, is genuine	Jingdong prefecture level city in Shandong
Fortune Shop	Green Intelligent Power Saver	Nothing happens when you plug it in.	Jingdong prefecture level city in Shandong
Fortune Shop	Green Intelligent Power Saver	I don't know if it's as good as it sounds.	Jingdong prefecture level city in Shandong
Fortune Shop	Green Intelligent Power Saver	The quality of the stuff is very good, exactly the same as the seller's description, very satisfied!	Jingdong prefecture level city in Shandong
Fortune Shop	Green Intelligent Power Saver	The saver works great, it really works, it's affordable and worth buying.	Jingdong prefecture level city in Shandong
Fortune Shop	Green Intelligent Power Saver	Just used it for a couple days. I don't know yet.	Jingdong prefecture level city in Shandong
Fortune Shop	Green Intelligent Power Saver	The quality is quite good, cheaper than the physical store, good, next time will come again!	Jingdong prefecture level city in Shandong
Fortune Shop	Green Intelligent Power Saver	Good quality, cheap and affordable worth recommending to buy!	Jingdong prefecture level city in Shandong
Fortune Shop	Green Intelligent Power Saver	Very good quality, well made, works well! Satisfied	Jingdong prefecture level city in Shandong
Fortune Shop	Green Intelligent Power Saver	The logistics is very fast quality is very good very pleasant a shopping	Jingdong prefecture level city in Shandong
Fortune Shop	Green Intelligent Power Saver	Use it and then come back to evaluate!	Jingdong prefecture level city in Shandong
Fortune Shop	Green Intelligent Power Saver	# It's no use lying at all #	Jingdong prefecture level city in Shandong
Fortune Shop	Green Intelligent Power Saver	I can't get a voltage drop.	Jingdong prefecture level city in Shandong

Fortune Shop	Green Intelligent Power Saver	Evaluate after use	Jingdong prefecture level city in Shandong
Fortune Shop	Green Intelligent Power Saver	Still on trial.	Jingdong prefecture level city in Shandong
Fortune Shop	Green Intelligent Power Saver	ineffective	Jingdong prefecture level city in Shandong
Fortune Shop	Green Intelligent Power Saver	Satisfaction Shopping	Jingdong prefecture level city in Shandong
Fortune Shop	Green Intelligent Power Saver	Lies.	Jingdong prefecture level city in Shandong
Fortune Shop	Green Intelligent Power Saver	It's a real power saver.	Jingdong prefecture level city in Shandong
Fortune Shop	Green Intelligent Power Saver	in use	Jingdong prefecture level city in Shandong
Fortune Shop	Green Intelligent Power Saver	I don't know yet.	Jingdong prefecture level city in Shandong
Fortune Shop	Green Intelligent Power Saver	not care about anything	Jingdong prefecture level city in Shandong
Fortune Shop	Green Intelligent Power Saver	It doesn't seem to have much effect	Jingdong prefecture level city in Shandong
Fortune Shop	Green Intelligent Power Saver	Let the merchant see for himself, does the plug positive and negative terminals look like this?	Jingdong prefecture level city in Shandong
Fortune Shop	Green Intelligent Power Saver	It doesn't work well, the electricity bill is less before using it, but more after using it, contacting customer service, the service is still not good, and returning it is slow	Jingdong prefecture level city in Shandong
Fortune Shop	Green Intelligent Power Saver	With the advertisement product does not match, above the effective negative, power saving rate of two rows of words it, the product looks very thin, with 3 days to see the meter number is still with the previous no difference, which is obviously fraudulent behavior!	Jingdong prefecture level city in Shandong
Fortune Shop	Green Intelligent Power Saver	Doesn't save any power at all. Not much use.	Jingdong prefecture level city in Shandong
Fortune Shop	Green Intelligent Power Saver	The plugs are all loose, they're all the same length and they don't work at all	Jingdong prefecture level city in Shandong
Fortune Shop	Green Intelligent Power Saver	It's bad 👎 it's not effective at all, you'd be crazy to sell this garbage on the internet.	Jingdong prefecture level city in Shandong
Fortune Shop	Green Intelligent Power Saver	It doesn't save any power at all, so I hope the old timers don't fall for this scam again	Jingdong prefecture level city in Shandong
Fortune Shop	Green Intelligent Power Saver	I've been using it for over a month now and haven't noticed any power savings.	Jingdong prefecture level city in Shandong
Fortune Shop	Green Intelligent Power Saver	It doesn't work at all. The electricity bill is higher when you use it.	Jingdong prefecture level city in Shandong
Fortune Shop	Green Intelligent Power Saver	It doesn't save any power, it's still as bad as ever.	Jingdong prefecture level city in Shandong
Fortune Shop	Green Intelligent Power Saver	Last night before leaving work to look at the table, and then plugged into the node device, this morning and then look at the table, than the original save a few degrees of electricity, very good to have a power-saving effect, easy to use, satisfied with the shopping praise.	Jingdong prefecture level city in Shandong
Fortune Shop	Green Intelligent Power Saver	The product is real and effective, the electricity bill is reduced, really good, thanks to the merchants have so many good products, the product is real and effective, the electricity bill is reduced, want to save electricity friends quickly buy it, early use early to save money.	Jingdong prefecture level city in Shandong
Fortune Shop	Green Intelligent Power Saver	Saver saves time and money, baby appearance is beautiful, the quality of goods is good, and the same as the business description, good service attitude, packaging is complete, logistics is very fast, praise to	Jingdong prefecture level city in Shandong
Fortune Shop	Green Intelligent Power Saver	The effect is good, summer, home electricity is relatively large, buy and use a few days, feel the effect is very good, originally ten dollars a day electricity bill, now eight dollars a day, quite good	Jingdong prefecture level city in Shandong
Fortune Shop	Green Intelligent Power Saver	Indeed can save electricity and money, the first time I know this or from a friend's home to see, but also do not believe it, and now they use it to know that there is this good thing.	Jingdong prefecture level city in Shandong
Fortune Shop	Green Intelligent Power Saver	I have received the Smart Power Saver. Perfectly packaged. Fast delivery. Boss customer service attitude is also very good. It's worth buying. I plugged it in and tried it and it really saves more power than the original. I'll buy it again next time.	Jingdong prefecture level city in Shandong
Fortune Shop	Green Intelligent Power Saver	It's nice to save a dozen bucks a night on two air conditioners that used to take 30 or 40 degrees a night, but now it's only 10 or so degrees a night	Jingdong prefecture level city in Shandong
Fortune Shop	Green Intelligent Power Saver	Product no manufacturer's name no product qualification certificate, try two days to see the effect of	Jingdong prefecture level city in Shandong
Fortune Shop	Green Intelligent Power Saver	I've been using it for over a month and have saved over $80, so I've repurchased a few more back to save together	Jingdong prefecture level city in Shandong
Fortune Shop	Green Intelligent Power Saver	I haven't used it yet and will comment when I do, but it's well made.	Jingdong prefecture level city in Shandong
Fortune Shop	Green Intelligent Power Saver	It's quite energy efficient. It's really a boon for the home.	Jingdong prefecture level city in Shandong
Fortune Shop	Green Intelligent Power Saver	Energy-saving socket received a month, after using it for a month proved that it is indeed energy-saving, electricity bill saving, will buy it again, thank you!	Jingdong prefecture level city in Shandong
Fortune Shop	Green Intelligent Power Saver	There was a similar product many years ago. Now technology has evolved, buy one to try, according to the instructions in the box, it takes one or two dozen days to show results. Check your electric bill and see what happens.	Jingdong prefecture level city in Shandong
Fortune Shop	Green Intelligent Power Saver	Jingdong logistics is really fast, the same day the order arrived on the same day, more convenient than the supermarket, and a wide range of choices, the	Jingdong prefecture level city in Shandong
Fortune Shop	Green Intelligent Power Saver	I've been using this for a month or so and I can't see any results, it really is a scam as they say.	Jingdong prefecture level city in Shandong
Fortune Shop	Green Intelligent Power Saver	Very good use, a month can save a few degrees of power loss of electricity, introduced to the relatives to buy back to try.	Jingdong prefecture level city in Shandong
Fortune Shop	Green Intelligent Power Saver	It's really powerful! With this air conditioner can be turned on at any time without the pressure of paying the electricity bill! It's good for the people!	Jingdong prefecture level city in Shandong
Fortune Shop	Green Intelligent Power Saver	This business is a big ** products are not qualified, with a month of electricity more than one hundred percent of thirty, the purchase agreed to return the goods but do not receive a refund after receiving the goods, looking for the official side does not help, the Jingdong is a big **, I have never shopped in	Jingdong prefecture level city in Shandong
Fortune Shop	Green Intelligent Power Saver	It's good. I tried it. It saves power.	Jingdong prefecture level city in Shandong
Fortune Shop	Green Intelligent Power Saver	Nothing**, it seems like there's more	Jingdong prefecture level city in Shandong
Fortune Shop	Green Intelligent Power Saver	Power saving device received, well packaged, good quality, fast logistics	Jingdong prefecture level city in Shandong
Fortune Shop	Green Intelligent Power Saver	The service is okay, is that when I bought five, the desirable pieces are three, and then wait a few days and send me two replacement, I do not know if it is a mistake of the shipper, if I was not in the courier there to see, go home to say no, the	Jingdong prefecture level city in Shandong
Fortune Shop	Green Intelligent Power Saver	This thing will only cost me more if I use it, but before I used it, it was less than a ~ electricity a month, and after I used it, it was more than two hundred kWh a month.	Jingdong prefecture level city in Shandong
Fortune Shop	Green Intelligent Power Saver	I just used it. I don't know how it is.	Jingdong prefecture level city in Shandong
Fortune Shop	Green Intelligent Power Saver	I've received the product and I'm using it, so I'll see how it goes in a month!	Jingdong prefecture level city in Shandong
Fortune Shop	Green Intelligent Power Saver	The goods have just received the effect do not know how, please look forward to the results.	Jingdong prefecture level city in Shandong
Fortune Shop	Green Intelligent Power Saver	Power saving device, the material is very good practical and durable, the size is suitable, packaging is very good, the price is cheap, logistics is very fast!	Jingdong prefecture level city in Shandong
Fortune Shop	Green Intelligent Power Saver	Very nice, will comment in detail after using it for a while.	Jingdong prefecture level city in Shandong

Fortune Shop	Green Intelligent Power Saver	Good stuff to trust and support	Jingdong prefecture level city in Shandong
Fortune Shop	Green Intelligent Power Saver	I'll comment after I use it, doubt if it really saves power no	Jingdong prefecture level city in Shandong
Fortune Shop	Green Intelligent Power Saver	It's good quality and works well!	Jingdong prefecture level city in Shandong
Fortune Shop	Green Intelligent Power Saver	hahaha just right hahahahahaha hiccup hahahaha	Jingdong prefecture level city in Shandong
Fortune Shop	Green Intelligent Power Saver	The real thing matches the picture and looks great!	Jingdong prefecture level city in Shandong
Fortune Shop	Green Intelligent Power Saver	Plugged it in and we'll see when we pay the bills at the end of the year!	Jingdong prefecture level city in Shandong
Fortune Shop	Green Intelligent Power Saver	False advertising. It doesn't work.	Jingdong prefecture level city in Shandong
Fortune Shop	Green Intelligent Power Saver	Goods received, good effect	Jingdong prefecture level city in Shandong
Fortune Shop	Green Intelligent Power Saver	It looks okay, we'll see if it saves electricity when the time comes	Jingdong prefecture level city in Shandong
Fortune Shop	Green Intelligent Power Saver	Logistics is very fast quality is very good, the price is very affordable, accessories are also complete, installed, satisfied with good use	Jingdong prefecture level city in Shandong
Fortune Shop	Green Intelligent Power Saver	I've received the power saver. I'm looking forward to the savings.	Jingdong prefecture level city in Shandong
Fortune Shop	Green Intelligent Power Saver	Received, have not yet used, after use and then evaluate	Jingdong prefecture level city in Shandong
Fortune Shop	Green Intelligent Power Saver	I received the item, but I don't know if it saves power or not.	Jingdong prefecture level city in Shandong
Fortune Shop	Green Intelligent Power Saver	On Trial	Jingdong prefecture level city in Shandong
Fortune Shop	Green Intelligent Power Saver	Use it and see how it goes.	Jingdong prefecture level city in Shandong
Fortune Shop	Green Intelligent Power Saver	Quality is very good, no odor, fine workmanship, grain color are very like, satisfied!	Jingdong prefecture level city in Shandong
Fortune Shop	Green Intelligent Power Saver	I'll give you a good review. I don't know if it'll work.	Jingdong prefecture level city in Shandong
Fortune Shop	Green Intelligent Power Saver	The logistics is very fast and the package is intact. Good use of affordable. The effect is very good, is genuine	Jingdong prefecture level city in Shandong
Fortune Shop	Green Intelligent Power Saver	Nothing happens when you plug it in.	Jingdong prefecture level city in Shandong
Fortune Shop	Green Intelligent Power Saver	I don't know if it's as good as it sounds.	Jingdong prefecture level city in Shandong
Fortune Shop	Green Intelligent Power Saver	The quality of the stuff is very good, exactly the same as the seller's description, very satisfied!	Jingdong prefecture level city in Shandong
Fortune Shop	Green Intelligent Power Saver	The saver works great, it really works, it's affordable and worth buying.	Jingdong prefecture level city in Shandong
Fortune Shop	Green Intelligent Power Saver	Just used it for a couple days. I don't know yet.	Jingdong prefecture level city in Shandong
Fortune Shop	Green Intelligent Power Saver	The quality is quite good, cheaper than the physical store, good, next time will come again!	Jingdong prefecture level city in Shandong
Fortune Shop	Green Intelligent Power Saver	Good quality, cheap and affordable worth recommending to buy!	Jingdong prefecture level city in Shandong
Fortune Shop	Green Intelligent Power Saver	Very good quality, well made, works well! Satisfied	Jingdong prefecture level city in Shandong
Fortune Shop	Green Intelligent Power Saver	The logistics is very fast quality is very good very pleasant a shopping	Jingdong prefecture level city in Shandong
Fortune Shop	Green Intelligent Power Saver	Use it and then come back to evaluate!	Jingdong prefecture level city in Shandong
Fortune Shop	Green Intelligent Power Saver	# It's no use lying at all #	Jingdong prefecture level city in Shandong
Fortune Shop	Green Intelligent Power Saver	I can't get a voltage drop.	Jingdong prefecture level city in Shandong
Fortune Shop	Green Intelligent Power Saver	Evaluate after use	Jingdong prefecture level city in Shandong
Fortune Shop	Green Intelligent Power Saver	Still on trial.	Jingdong prefecture level city in Shandong
Fortune Shop	Green Intelligent Power Saver	ineffective	Jingdong prefecture level city in Shandong
Fortune Shop	Green Intelligent Power Saver	Satisfaction Shopping	Jingdong prefecture level city in Shandong
Fortune Shop	Green Intelligent Power Saver	Lies.	Jingdong prefecture level city in Shandong
Fortune Shop	Green Intelligent Power Saver	It's a real power saver.	Jingdong prefecture level city in Shandong
Fortune Shop	Green Intelligent Power Saver	in use	Jingdong prefecture level city in Shandong
Fortune Shop	Green Intelligent Power Saver	I don't know yet.	Jingdong prefecture level city in Shandong
Fortune Shop	Green Intelligent Power Saver	not care about anything	Jingdong prefecture level city in Shandong
Fortune Shop	Green Intelligent Power Saver	It doesn't seem to have much effect	Jingdong prefecture level city in Shandong
Fortune Shop	Green Intelligent Power Saver	Let the merchant see for himself, does the plug positive and negative terminals look like this?	Jingdong prefecture level city in Shandong
Fortune Shop	Green Intelligent Power Saver	It doesn't work well, the electricity bill is less before using it, but more after using it, contacting customer service, the service is still not good, and returning it is slow	Jingdong prefecture level city in Shandong
Fortune Shop	Green Intelligent Power Saver	With the advertisement product does not match, above the effective negative, power saving rate of two rows of words it, the product looks very thin, with 3 days to see the meter number is still with the previous no difference, which is obviously fraudulent behavior!	Jingdong prefecture level city in Shandong
Fortune Shop	Green Intelligent Power Saver	Doesn't save any power at all. Not much use.	Jingdong prefecture level city in Shandong
Fortune Shop	Green Intelligent Power Saver	The plugs are all loose, they're all the same length and they don't work at all	Jingdong prefecture level city in Shandong
Fortune Shop	Green Intelligent Power Saver	It's bad 👎 it's not effective at all, you'd be crazy to sell this garbage on the internet.	Jingdong prefecture level city in Shandong
Fortune Shop	Green Intelligent Power Saver	It doesn't save any power at all, so I hope the old timers don't fall for this scam again	Jingdong prefecture level city in Shandong
Fortune Shop	Green Intelligent Power Saver	I've been using it for over a month now and haven't noticed any power savings.	Jingdong prefecture level city in Shandong
Fortune Shop	Green Intelligent Power Saver	It doesn't work at all. The electricity bill is higher when you use it.	Jingdong prefecture level city in Shandong
Fortune Shop	Green Intelligent Power Saver	It doesn't save any power, it's still as bad as ever.	Jingdong prefecture level city in Shandong

Fortune Shop	Green Intelligent Power Saver	Last night before leaving work to look at the table, and then plugged into the node device, this morning and then look at the table, than the original save a few degrees of electricity, very good to have a power-saving effect, easy to use, satisfied with the shopping praise.	Jingdong prefecture level city in Shandong
Fortune Shop	Green Intelligent Power Saver	The product is real and effective, the electricity bill is reduced, really good, thanks to the merchants have so many good products, the product is real and effective, the electricity bill is reduced, want to save electricity friends quickly buy it, early use early to save money.	Jingdong prefecture level city in Shandong
Fortune Shop	Green Intelligent Power Saver	Saver saves time and money, baby appearance is beautiful, the quality of goods is good, and the same as the business description, good service attitude, packaging is complete, logistics is very fast, praise to	Jingdong prefecture level city in Shandong
Fortune Shop	Green Intelligent Power Saver	The effect is good, summer, home electricity is relatively large, buy and use a few days, feel the effect is very good, originally ten dollars a day electricity bill, now eight dollars a day, quite good	Jingdong prefecture level city in Shandong
Fortune Shop	Green Intelligent Power Saver	Indeed can save electricity and money, the first time I know this or from a friend's home to see, but also do not believe it, and now they use it to know that there is this good thing.	Jingdong prefecture level city in Shandong
Fortune Shop	Green Intelligent Power Saver	I have received the Smart Power Saver. Perfectly packaged. Fast delivery. Boss customer service attitude is also very good. It's worth buying. I plugged it in and tried it and it really saves more power than the original. I'll buy it again next time.	Jingdong prefecture level city in Shandong
Fortune Shop	Green Intelligent Power Saver	It's nice to save a dozen bucks a night on two air conditioners that used to take 30 or 40 degrees a night, but now it's only 10 or so degrees a night	Jingdong prefecture level city in Shandong
Fortune Shop	Green Intelligent Power Saver	Product no manufacturer's name no product qualification certificate, try two days to see the effect of	Jingdong prefecture level city in Shandong
Fortune Shop	Green Intelligent Power Saver	I've been using it for over a month and have saved over $80, so I've repurchased a few more back to save together	Jingdong prefecture level city in Shandong
Fortune Shop	Green Intelligent Power Saver	I haven't used it yet and will comment when I do, but it's well made.	Jingdong prefecture level city in Shandong
Fortune Shop	Green Intelligent Power Saver	It's quite energy efficient. It's really a boon for the home.	Jingdong prefecture level city in Shandong
Fortune Shop	Green Intelligent Power Saver	Energy-saving socket received a month, after using it for a month proved that it is indeed energy-saving, electricity bill saving, will buy it again, thank you!	Jingdong prefecture level city in Shandong
Fortune Shop	Green Intelligent Power Saver	There was a similar product many years ago. Now technology has evolved, buy one to try, according to the instructions in the box, it takes one or two dozen days to show results. Check your electric bill and see what happens.	Jingdong prefecture level city in Shandong
Fortune Shop	Green Intelligent Power Saver	Jingdong logistics is really fast, the same day the order arrived on the same day, more convenient than the supermarket, and a wide range of choices, the	Jingdong prefecture level city in Shandong
Fortune Shop	Green Intelligent Power Saver	I've been using this for a month or so and I can't see any results, it really is a scam as they say.	Jingdong prefecture level city in Shandong
Fortune Shop	Green Intelligent Power Saver	Very good use, a month can save a few degrees of power loss of electricity, introduced to the relatives to buy back to try.	Jingdong prefecture level city in Shandong
Fortune Shop	Green Intelligent Power Saver	It's really powerful! With this air conditioner can be turned on at any time without the pressure of paying the electricity bill! It's good for the people!	Jingdong prefecture level city in Shandong
Fortune Shop	Green Intelligent Power Saver	This business is a big ** products are not qualified, with a month of electricity more than one hundred percent of thirty, the purchase agreed to return the goods but do not receive a refund after receiving the goods, looking for the official side does not help, the Jingdong is a big **, I have never shopped in	Jingdong prefecture level city in Shandong
Fortune Shop	Green Intelligent Power Saver	It's good. I tried it. It saves power.	Jingdong prefecture level city in Shandong
Fortune Shop	Green Intelligent Power Saver	Nothing**, it seems like there's more	Jingdong prefecture level city in Shandong
Fortune Shop	Green Intelligent Power Saver	Power saving device received, well packaged, good quality, fast logistics	Jingdong prefecture level city in Shandong
Fortune Shop	Green Intelligent Power Saver	The service is okay, is that when I bought five, the desirable pieces are three, and then wait a few days and send me two replacement, I do not know if it is a mistake of the shipper, if I was not in the courier there to see, go home to say no, the	Jingdong prefecture level city in Shandong
Fortune Shop	Green Intelligent Power Saver	This thing will only cost me more if I use it, but before I used it, it was less than a ~ electricity a month, and after I used it, it was more than two hundred kWh a month.	Jingdong prefecture level city in Shandong
Fortune Shop	Green Intelligent Power Saver	I just used it. I don't know how it is.	Jingdong prefecture level city in Shandong
Fortune Shop	Green Intelligent Power Saver	I've received the product and I'm using it, so I'll see how it goes in a month!	Jingdong prefecture level city in Shandong
Fortune Shop	Green Intelligent Power Saver	The goods have just received the effect do not know how, please look forward to the results.	Jingdong prefecture level city in Shandong
Fortune Shop	Green Intelligent Power Saver	Power saving device, the material is very good practical and durable, the size is suitable, packaging is very good, the price is cheap, logistics is very fast!	Jingdong prefecture level city in Shandong
Fortune Shop	Green Intelligent Power Saver	Very nice, will comment in detail after using it for a while.	Jingdong prefecture level city in Shandong
Fortune Shop	Green Intelligent Power Saver	Good stuff to trust and support	Jingdong prefecture level city in Shandong
Fortune Shop	Green Intelligent Power Saver	I'll comment after I use it, doubt if it really saves power no	Jingdong prefecture level city in Shandong
Fortune Shop	Green Intelligent Power Saver	It's good quality and works well!	Jingdong prefecture level city in Shandong
Fortune Shop	Green Intelligent Power Saver	hahaha just right hahahahahaha hiccup hahahaha	Jingdong prefecture level city in Shandong
Fortune Shop	Green Intelligent Power Saver	The real thing matches the picture and looks great!	Jingdong prefecture level city in Shandong
Fortune Shop	Green Intelligent Power Saver	Plugged it in and we'll see when we pay the bills at the end of the year!	Jingdong prefecture level city in Shandong
Fortune Shop	Green Intelligent Power Saver	False advertising. It doesn't work.	Jingdong prefecture level city in Shandong
Fortune Shop	Green Intelligent Power Saver	Goods received, good effect	Jingdong prefecture level city in Shandong
Fortune Shop	Green Intelligent Power Saver	It looks okay, we'll see if it saves electricity when the time comes	Jingdong prefecture level city in Shandong
Fortune Shop	Green Intelligent Power Saver	Logistics is very fast quality is very good, the price is very affordable, accessories are also complete, installed, satisfied with good use	Jingdong prefecture level city in Shandong
Fortune Shop	Green Intelligent Power Saver	I've received the power saver. I'm looking forward to the savings.	Jingdong prefecture level city in Shandong
Fortune Shop	Green Intelligent Power Saver	Received, have not yet used, after use and then evaluate	Jingdong prefecture level city in Shandong
Fortune Shop	Green Intelligent Power Saver	I received the item, but I don't know if it saves power or not.	Jingdong prefecture level city in Shandong
Fortune Shop	Green Intelligent Power Saver	On Trial	Jingdong prefecture level city in Shandong
Fortune Shop	Green Intelligent Power Saver	Use it and see how it goes.	Jingdong prefecture level city in Shandong
Fortune Shop	Green Intelligent Power Saver	Quality is very good, no odor, fine workmanship, grain color are very like, satisfied!	Jingdong prefecture level city in Shandong
Fortune Shop	Green Intelligent Power Saver	I'll give you a good review. I don't know if it'll work.	Jingdong prefecture level city in Shandong
Fortune Shop	Green Intelligent Power Saver	The logistics is very fast and the package is intact. Good use of affordable. The effect is very good, is genuine	Jingdong prefecture level city in Shandong
Fortune Shop	Green Intelligent Power Saver	Nothing happens when you plug it in.	Jingdong prefecture level city in Shandong
Fortune Shop	Green Intelligent Power Saver	I don't know if it's as good as it sounds.	Jingdong prefecture level city in Shandong

Fortune Shop	Green Intelligent Power Saver	The quality of the stuff is very good, exactly the same as the seller's description, very satisfied!	Jingdong prefecture level city in Shandong
Fortune Shop	Green Intelligent Power Saver	The saver works great, it really works, it's affordable and worth buying.	Jingdong prefecture level city in Shandong
Fortune Shop	Green Intelligent Power Saver	Just used it for a couple days. I don't know yet.	Jingdong prefecture level city in Shandong
Fortune Shop	Green Intelligent Power Saver	The quality is quite good, cheaper than the physical store, good, next time will come again!	Jingdong prefecture level city in Shandong
Fortune Shop	Green Intelligent Power Saver	Good quality, cheap and affordable worth recommending to buy!	Jingdong prefecture level city in Shandong
Fortune Shop	Green Intelligent Power Saver	Very good quality, well made, works well! Satisfied	Jingdong prefecture level city in Shandong
Fortune Shop	Green Intelligent Power Saver	The logistics is very fast quality is very good very pleasant a shopping	Jingdong prefecture level city in Shandong
Fortune Shop	Green Intelligent Power Saver	Use it and then come back to evaluate!	Jingdong prefecture level city in Shandong
Fortune Shop	Green Intelligent Power Saver	# It's no use lying at all #	Jingdong prefecture level city in Shandong
Fortune Shop	Green Intelligent Power Saver	I can't get a voltage drop.	Jingdong prefecture level city in Shandong
Fortune Shop	Green Intelligent Power Saver	Evaluate after use	Jingdong prefecture level city in Shandong
Fortune Shop	Green Intelligent Power Saver	Still on trial.	Jingdong prefecture level city in Shandong
Fortune Shop	Green Intelligent Power Saver	ineffective	Jingdong prefecture level city in Shandong
Fortune Shop	Green Intelligent Power Saver	Satisfaction Shopping	Jingdong prefecture level city in Shandong
Fortune Shop	Green Intelligent Power Saver	Lies.	Jingdong prefecture level city in Shandong
Fortune Shop	Green Intelligent Power Saver	It's a real power saver.	Jingdong prefecture level city in Shandong
Fortune Shop	Green Intelligent Power Saver	in use	Jingdong prefecture level city in Shandong
Fortune Shop	Green Intelligent Power Saver	I don't know yet.	Jingdong prefecture level city in Shandong
Fortune Shop	Green Intelligent Power Saver	not care about anything	Jingdong prefecture level city in Shandong
Fortune Shop	Green Intelligent Power Saver	It doesn't seem to have much effect	Jingdong prefecture level city in Shandong
Fortune Shop	Green Intelligent Power Saver	Let the merchant see for himself, does the plug positive and negative terminals look like this?	Jingdong prefecture level city in Shandong
Fortune Shop	Green Intelligent Power Saver	It doesn't work well, the electricity bill is less before using it, but more after using it, contacting customer service, the service is still not good, and returning it is slow	Jingdong prefecture level city in Shandong
Fortune Shop	Green Intelligent Power Saver	With the advertisement product does not match, above the effective negative, power saving rate of two rows of words it, the product looks very thin, with 3 days to see the meter number is still with the previous no difference, which is obviously fraudulent behavior!	Jingdong prefecture level city in Shandong
Fortune Shop	Green Intelligent Power Saver	Doesn't save any power at all. Not much use.	Jingdong prefecture level city in Shandong
Fortune Shop	Green Intelligent Power Saver	The plugs are all loose, they're all the same length and they don't work at all	Jingdong prefecture level city in Shandong
Fortune Shop	Green Intelligent Power Saver	It's bad 👎 it's not effective at all, you'd be crazy to sell this garbage on the internet.	Jingdong prefecture level city in Shandong
Fortune Shop	Green Intelligent Power Saver	It doesn't save any power at all, so I hope the old timers don't fall for this scam again	Jingdong prefecture level city in Shandong
Fortune Shop	Green Intelligent Power Saver	I've been using it for over a month now and haven't noticed any power savings.	Jingdong prefecture level city in Shandong
Fortune Shop	Green Intelligent Power Saver	It doesn't work at all. The electricity bill is higher when you use it.	Jingdong prefecture level city in Shandong
Fortune Shop	Green Intelligent Power Saver	It doesn't save any power, it's still as bad as ever.	Jingdong prefecture level city in Shandong
Fortune Shop	Green Intelligent Power Saver	Last night before leaving work to look at the table, and then plugged into the node device, this morning and then look at the table, the original save a few degrees of electricity, very good to have a power-saving effect, easy to use, satisfied with the shopping praise.	Jingdong prefecture level city in Shandong
Fortune Shop	Green Intelligent Power Saver	The product is real and effective, the electricity bill is reduced, really good, thanks to the merchants have so many good products, the product is real and effective, the electricity bill is reduced, want to save electricity friends quickly buy it, early use early to save money.	Jingdong prefecture level city in Shandong
Fortune Shop	Green Intelligent Power Saver	Saver saves time and money, baby appearance is beautiful, the quality of goods is good, and the same as the business description, good service attitude, packaging is complete, logistics is very fast, praise to	Jingdong prefecture level city in Shandong
Fortune Shop	Green Intelligent Power Saver	The effect is good, summer, home electricity is relatively large, buy and use a few days, feel the effect is very good, originally ten dollars a day electricity bill, now eight dollars a day, quite good	Jingdong prefecture level city in Shandong
Fortune Shop	Green Intelligent Power Saver	Indeed can save electricity and money, the first time I know this or from a friend's home to see, but also do not believe it, and now they use it to know that there is this good thing.	Jingdong prefecture level city in Shandong
Fortune Shop	Green Intelligent Power Saver	I have received the Smart Power Saver. Perfectly packaged. Fast delivery. Boss customer service attitude is also very good. It's worth buying. I plugged it in and tried it and it really saves more power than the original. I'll buy it again next time.	Jingdong prefecture level city in Shandong
Fortune Shop	Green Intelligent Power Saver	It's nice to save a dozen bucks a night on two air conditioners that used to take 30 or 40 degrees a night, but now it's only 10 or so degrees a night	Jingdong prefecture level city in Shandong
Fortune Shop	Green Intelligent Power Saver	Product no manufacturer's name no product qualification certificate, try two days to see the effect of	Jingdong prefecture level city in Shandong
Fortune Shop	Green Intelligent Power Saver	I've been using it for over a month and have saved over $80, so I've repurchased a few more back to save together	Jingdong prefecture level city in Shandong
Fortune Shop	Green Intelligent Power Saver	I haven't used it yet and will comment when I do, but it's well made.	Jingdong prefecture level city in Shandong
Fortune Shop	Green Intelligent Power Saver	It's quite energy efficient. It's really a boon for the home.	Jingdong prefecture level city in Shandong
Fortune Shop	Green Intelligent Power Saver	Energy-saving socket received a month, after using it for a month proved that it is indeed energy-saving, electricity bill saving, will buy it again, thank you!	Jingdong prefecture level city in Shandong
Fortune Shop	Green Intelligent Power Saver	There was a similar product many years ago. Now technology has evolved, buy one to try, according to the instructions in the box, it takes one or two dozen days to show results. Check your electric bill and see what happens.	Jingdong prefecture level city in Shandong
Fortune Shop	Green Intelligent Power Saver	Jingdong logistics is really fast, the same day the order arrived on the same day, more convenient than the supermarket, and a wide range of choices, the	Jingdong prefecture level city in Shandong
Fortune Shop	Green Intelligent Power Saver	I've been using this for a month or so and I can't see any results, it really is a scam as they say.	Jingdong prefecture level city in Shandong
Fortune Shop	Green Intelligent Power Saver	Very good use, a month can save a few degrees of power loss of electricity, introduced to the relatives to buy back to try.	Jingdong prefecture level city in Shandong
Fortune Shop	Green Intelligent Power Saver	It's really powerful! With this air conditioner can be turned on at any time without the pressure of paying the electricity bill! It's good for the people!	Jingdong prefecture level city in Shandong
Fortune Shop	Green Intelligent Power Saver	This business is a big ** products are not qualified, with a month of electricity more than one hundred percent of thirty, the purchase agreed to return the goods but do not receive a refund after receiving the goods, looking for the official side does not help, the Jingdong is a big **, I have never shopped in	Jingdong prefecture level city in Shandong

Fortune Shop	Green Intelligent Power Saver	It's good. I tried it. It saves power.	Jingdong prefecture level city in Shandong
Fortune Shop	Green Intelligent Power Saver	Nothing**, it seems like there's more	Jingdong prefecture level city in Shandong
Fortune Shop	Green Intelligent Power Saver	Power saving device received, well packaged, good quality, fast logistics	Jingdong prefecture level city in Shandong
Fortune Shop	Green Intelligent Power Saver	The service is okay, is that when I bought five, the desirable pieces are three, and then wait a few days and send me two replacement, I do not know if it is a mistake of the shipper, if I was not in the courier there to see, go home to say no, the	Jingdong prefecture level city in Shandong
Fortune Shop	Green Intelligent Power Saver	This thing will only cost me more if I use it, but before I used it, it was less than a ~ electricity a month, and after I used it, it was more than two hundred kWh a month.	Jingdong prefecture level city in Shandong
Fortune Shop	Green Intelligent Power Saver	I just used it. I don't know how it is.	Jingdong prefecture level city in Shandong
Fortune Shop	Green Intelligent Power Saver	I've received the product and I'm using it, so I'll see how it goes in a month!	Jingdong prefecture level city in Shandong
Fortune Shop	Green Intelligent Power Saver	The goods have just received the effect do not know how, please look forward to the results.	Jingdong prefecture level city in Shandong
Fortune Shop	Green Intelligent Power Saver	Power saving device, the material is very good practical and durable, the size is suitable, packaging is very good, the price is cheap, logistics is very fast!	Jingdong prefecture level city in Shandong
Fortune Shop	Green Intelligent Power Saver	Very nice, will comment in detail after using it for a while.	Jingdong prefecture level city in Shandong
Fortune Shop	Green Intelligent Power Saver	Good stuff to trust and support	Jingdong prefecture level city in Shandong
Fortune Shop	Green Intelligent Power Saver	I'll comment after I use it, doubt if it really saves power no	Jingdong prefecture level city in Shandong
Fortune Shop	Green Intelligent Power Saver	It's good quality and works well!	Jingdong prefecture level city in Shandong
Fortune Shop	Green Intelligent Power Saver	hahaha just right hahahahahaha hiccup hahahaha	Jingdong prefecture level city in Shandong
Fortune Shop	Green Intelligent Power Saver	The real thing matches the picture and looks great!	Jingdong prefecture level city in Shandong
Fortune Shop	Green Intelligent Power Saver	Plugged it in and we'll see when we pay the bills at the end of the year!	Jingdong prefecture level city in Shandong
Fortune Shop	Green Intelligent Power Saver	False advertising. It doesn't work.	Jingdong prefecture level city in Shandong
Fortune Shop	Green Intelligent Power Saver	Goods received, good effect	Jingdong prefecture level city in Shandong
Fortune Shop	Green Intelligent Power Saver	It looks okay, we'll see if it saves electricity when the time comes	Jingdong prefecture level city in Shandong
Fortune Shop	Green Intelligent Power Saver	Logistics is very fast quality is very good, the price is very affordable, accessories are also complete, installed, satisfied with good use	Jingdong prefecture level city in Shandong
Fortune Shop	Green Intelligent Power Saver	I've received the power saver. I'm looking forward to the savings.	Jingdong prefecture level city in Shandong
Fortune Shop	Green Intelligent Power Saver	Received, have not yet used, after use and then evaluate	Jingdong prefecture level city in Shandong
Fortune Shop	Green Intelligent Power Saver	I received the item, but I don't know if it saves power or not.	Jingdong prefecture level city in Shandong
Fortune Shop	Green Intelligent Power Saver	On Trial	Jingdong prefecture level city in Shandong
Fortune Shop	Green Intelligent Power Saver	Use it and see how it goes.	Jingdong prefecture level city in Shandong
Fortune Shop	Green Intelligent Power Saver	Quality is very good, no odor, fine workmanship, grain color are very like, satisfied!	Jingdong prefecture level city in Shandong
Fortune Shop	Green Intelligent Power Saver	I'll give you a good review. I don't know if it'll work.	Jingdong prefecture level city in Shandong
Fortune Shop	Green Intelligent Power Saver	The logistics is very fast and the package is intact. Good use of affordable. The effect is very good, is genuine	Jingdong prefecture level city in Shandong
Fortune Shop	Green Intelligent Power Saver	Nothing happens when you plug it in.	Jingdong prefecture level city in Shandong
Fortune Shop	Green Intelligent Power Saver	I don't know if it's as good as it sounds.	Jingdong prefecture level city in Shandong
Fortune Shop	Green Intelligent Power Saver	The quality of the stuff is very good, exactly the same as the seller's description, very satisfied!	Jingdong prefecture level city in Shandong
Fortune Shop	Green Intelligent Power Saver	The saver works great, it really works, it's affordable and worth buying.	Jingdong prefecture level city in Shandong
Fortune Shop	Green Intelligent Power Saver	Just used it for a couple days. I don't know yet.	Jingdong prefecture level city in Shandong
Fortune Shop	Green Intelligent Power Saver	The quality is quite good, cheaper than the physical store, good, next time will come again!	Jingdong prefecture level city in Shandong
Fortune Shop	Green Intelligent Power Saver	Good quality, cheap and affordable worth recommending to buy!	Jingdong prefecture level city in Shandong
Fortune Shop	Green Intelligent Power Saver	Very good quality, well made, works well! Satisfied	Jingdong prefecture level city in Shandong
Fortune Shop	Green Intelligent Power Saver	The logistics is very fast quality is very good very pleasant a shopping	Jingdong prefecture level city in Shandong
Fortune Shop	Green Intelligent Power Saver	Use it and then come back to evaluate!	Jingdong prefecture level city in Shandong
Fortune Shop	Green Intelligent Power Saver	# It's no use lying at all #	Jingdong prefecture level city in Shandong
Fortune Shop	Green Intelligent Power Saver	I can't get a voltage drop.	Jingdong prefecture level city in Shandong
Fortune Shop	Green Intelligent Power Saver	Evaluate after use	Jingdong prefecture level city in Shandong
Fortune Shop	Green Intelligent Power Saver	Still on trial.	Jingdong prefecture level city in Shandong
Fortune Shop	Green Intelligent Power Saver	ineffective	Jingdong prefecture level city in Shandong
Fortune Shop	Green Intelligent Power Saver	Satisfaction Shopping	Jingdong prefecture level city in Shandong
Fortune Shop	Green Intelligent Power Saver	Lies.	Jingdong prefecture level city in Shandong
Fortune Shop	Green Intelligent Power Saver	It's a real power saver.	Jingdong prefecture level city in Shandong
Fortune Shop	Green Intelligent Power Saver	in use	Jingdong prefecture level city in Shandong
Fortune Shop	Green Intelligent Power Saver	I don't know yet.	Jingdong prefecture level city in Shandong
Fortune Shop	Green Intelligent Power Saver	not care about anything	Jingdong prefecture level city in Shandong
Fortune Shop	Green Intelligent Power Saver	It doesn't seem to have much effect	Jingdong prefecture level city in Shandong
Fortune Shop	Green Intelligent Power Saver	Let the merchant see for himself, does the plug positive and negative terminals look like this?	Jingdong prefecture level city in Shandong

Fortune Shop	Green Intelligent Power Saver	It doesn't work well, the electricity bill is less before using it, but more after using it, contacting customer service, the service is still not good, and returning it is slow	Jingdong prefecture level city in Shandong
Fortune Shop	Green Intelligent Power Saver	With the advertisement product does not match, above the effective negative, power saving rate of two rows of words it, the product looks very thin, with 3 days to see the meter number is still with the previous no difference, which is obviously fraudulent behavior!	Jingdong prefecture level city in Shandong
Fortune Shop	Green Intelligent Power Saver	Doesn't save any power at all. Not much use.	Jingdong prefecture level city in Shandong
Fortune Shop	Green Intelligent Power Saver	The plugs are all loose, they're all the same length and they don't work at all	Jingdong prefecture level city in Shandong
Fortune Shop	Green Intelligent Power Saver	It's bad 👎 it's not effective at all, you'd be crazy to sell this garbage on the internet.	Jingdong prefecture level city in Shandong
Fortune Shop	Green Intelligent Power Saver	It doesn't save any power at all, so I hope the old timers don't fall for this scam again	Jingdong prefecture level city in Shandong
Fortune Shop	Green Intelligent Power Saver	I've been using it for over a month now and haven't noticed any power savings.	Jingdong prefecture level city in Shandong
Fortune Shop	Green Intelligent Power Saver	It doesn't work at all. The electricity bill is higher when you use it.	Jingdong prefecture level city in Shandong
Fortune Shop	Green Intelligent Power Saver	It doesn't save any power, it's still as bad as ever.	Jingdong prefecture level city in Shandong
Fortune Shop	Green Intelligent Power Saver	Last night before leaving work to look at the table, and then plugged into the node device, this morning and then look at the table, than the original save a few degrees of electricity, very good to have a power-saving effect, easy to use, satisfied with the shopping praise.	Jingdong prefecture level city in Shandong
Fortune Shop	Green Intelligent Power Saver	The product is real and effective, the electricity bill is reduced, really good, thanks to the merchants have so many good products, the product is real and effective, the electricity bill is reduced, want to save electricity friends quickly buy it, early use early to save money.	Jingdong prefecture level city in Shandong
Fortune Shop	Green Intelligent Power Saver	Saver saves time and money, baby appearance is beautiful, the quality of goods is good, and the same as the business description, good service attitude, packaging is complete, logistics is very fast, praise to	Jingdong prefecture level city in Shandong
Fortune Shop	Green Intelligent Power Saver	The effect is good, summer, home electricity is relatively large, buy and use a few days, feel the effect is very good, originally ten dollars a day electricity bill, now eight dollars a day, quite good	Jingdong prefecture level city in Shandong
Fortune Shop	Green Intelligent Power Saver	Indeed can save electricity and money, the first time I know this or from a friend's home to see, but also do not believe it, and now they use it to know that there is this good thing.	Jingdong prefecture level city in Shandong
Fortune Shop	Green Intelligent Power Saver	I have received the Smart Power Saver. Perfectly packaged. Fast delivery. Boss customer service attitude is also very good. It's worth buying. I plugged it in and tried it and it really saves more power than the original. I'll buy it again next time.	Jingdong prefecture level city in Shandong
Fortune Shop	Green Intelligent Power Saver	It's nice to save a dozen bucks a night on two air conditioners that used to take 30 or 40 degrees a night, but now it's only 10 or so degrees a night	Jingdong prefecture level city in Shandong
Fortune Shop	Green Intelligent Power Saver	Product no manufacturer's name no product qualification certificate, try two days to see the effect of	Jingdong prefecture level city in Shandong
Fortune Shop	Green Intelligent Power Saver	I've been using it for over a month and have saved over $80, so I've repurchased a few more back to save together	Jingdong prefecture level city in Shandong
Fortune Shop	Green Intelligent Power Saver	I haven't used it yet and will comment when I do, but it's well made.	Jingdong prefecture level city in Shandong
Fortune Shop	Green Intelligent Power Saver	It's quite energy efficient. It's really a boon for the home.	Jingdong prefecture level city in Shandong
Fortune Shop	Green Intelligent Power Saver	Energy-saving socket received a month, after using it for a month proved that it is indeed energy-saving, electricity bill saving, will buy it again, thank you!	Jingdong prefecture level city in Shandong
Fortune Shop	Green Intelligent Power Saver	There was a similar product many years ago. Now technology has evolved, buy one to try, according to the instructions in the box, it takes one or two dozen days to show results. Check your electric bill and see what happens.	Jingdong prefecture level city in Shandong
Fortune Shop	Green Intelligent Power Saver	Jingdong logistics is really fast, the same day the order arrived on the same day, more convenient than the supermarket, and a wide range of choices, the	Jingdong prefecture level city in Shandong
Fortune Shop	Green Intelligent Power Saver	I've been using this for a month or so and I can't see any results, it really is a scam as they say.	Jingdong prefecture level city in Shandong
Fortune Shop	Green Intelligent Power Saver	Very good use, a month can save a few degrees of power loss of electricity, introduced to the relatives to buy back to try.	Jingdong prefecture level city in Shandong
Fortune Shop	Green Intelligent Power Saver	It's really powerful! With this air conditioner can be turned on at any time without the pressure of paying the electricity bill! It's good for the people!	Jingdong prefecture level city in Shandong
Fortune Shop	Green Intelligent Power Saver	This business is a big ** products are not qualified, with a month of electricity more than one hundred percent of thirty, the purchase agreed to return the goods but do not receive a refund after receiving the goods, looking for the official side does not help, the Jingdong is a big **, I have never shopped in	Jingdong prefecture level city in Shandong
Fortune Shop	Green Intelligent Power Saver	It's good. I tried it. It saves power.	Jingdong prefecture level city in Shandong
Fortune Shop	Green Intelligent Power Saver	Nothing**, it seems like there's more	Jingdong prefecture level city in Shandong
Fortune Shop	Green Intelligent Power Saver	Power saving device received, well packaged, good quality, fast logistics	Jingdong prefecture level city in Shandong
Fortune Shop	Green Intelligent Power Saver	The service is okay, is that when I bought five, the desirable pieces are three, and then wait a few days and send me two replacement, I do not know if it is a mistake of the shipper, if I was not in the courier there to see, go home to say no, the	Jingdong prefecture level city in Shandong
Fortune Shop	Green Intelligent Power Saver	This thing will only cost me more if I use it, but before I used it, it was less than a ~ electricity a month, and after I used it, it was more than two hundred kWh a month.	Jingdong prefecture level city in Shandong
Fortune Shop	Green Intelligent Power Saver	I just used it. I don't know how it is.	Jingdong prefecture level city in Shandong
Fortune Shop	Green Intelligent Power Saver	I've received the product and I'm using it, so I'll see how it goes in a month!	Jingdong prefecture level city in Shandong
Fortune Shop	Green Intelligent Power Saver	The goods have just received the effect do not know how, please look forward to the results.	Jingdong prefecture level city in Shandong
Fortune Shop	Green Intelligent Power Saver	Power saving device, the material is very good practical and durable, the size is suitable, packaging is very good, the price is cheap, logistics is very fast!	Jingdong prefecture level city in Shandong
Fortune Shop	Green Intelligent Power Saver	Very nice, will comment in detail after using it for a while.	Jingdong prefecture level city in Shandong
Fortune Shop	Green Intelligent Power Saver	Good stuff to trust and support	Jingdong prefecture level city in Shandong
Fortune Shop	Green Intelligent Power Saver	I'll comment after I use it, doubt if it really saves power no	Jingdong prefecture level city in Shandong
Fortune Shop	Green Intelligent Power Saver	It's good quality and works well!	Jingdong prefecture level city in Shandong
Fortune Shop	Green Intelligent Power Saver	hahaha just right hahahahahaha hiccup hahahaha	Jingdong prefecture level city in Shandong
Fortune Shop	Green Intelligent Power Saver	The real thing matches the picture and looks great!	Jingdong prefecture level city in Shandong
Fortune Shop	Green Intelligent Power Saver	Plugged it in and we'll see when we pay the bills at the end of the year!	Jingdong prefecture level city in Shandong
Fortune Shop	Green Intelligent Power Saver	False advertising. It doesn't work.	Jingdong prefecture level city in Shandong
Fortune Shop	Green Intelligent Power Saver	Goods received, good effect	Jingdong prefecture level city in Shandong
Fortune Shop	Green Intelligent Power Saver	It looks okay, we'll see if it saves electricity when the time comes	Jingdong prefecture level city in Shandong
Fortune Shop	Green Intelligent Power Saver	Logistics is very fast quality is very good, the price is very affordable, accessories are also complete, installed, satisfied with good use	Jingdong prefecture level city in Shandong
Fortune Shop	Green Intelligent Power Saver	I've received the power saver. I'm looking forward to the savings.	Jingdong prefecture level city in Shandong

Fortune Shop	Green Intelligent Power Saver	Received, have not yet used, after use and then evaluate	Jingdong prefecture level city in Shandong
Fortune Shop	Green Intelligent Power Saver	I received the item, but I don't know if it saves power or not.	Jingdong prefecture level city in Shandong
Fortune Shop	Green Intelligent Power Saver	On Trial	Jingdong prefecture level city in Shandong
Fortune Shop	Green Intelligent Power Saver	Use it and see how it goes.	Jingdong prefecture level city in Shandong
Fortune Shop	Green Intelligent Power Saver	Quality is very good, no odor, fine workmanship, grain color are very like, satisfied!	Jingdong prefecture level city in Shandong
Fortune Shop	Green Intelligent Power Saver	I'll give you a good review. I don't know if it'll work.	Jingdong prefecture level city in Shandong
Fortune Shop	Green Intelligent Power Saver	The logistics is very fast and the package is intact. Good use of affordable. The effect is very good, is genuine	Jingdong prefecture level city in Shandong
Fortune Shop	Green Intelligent Power Saver	Nothing happens when you plug it in.	Jingdong prefecture level city in Shandong
Fortune Shop	Green Intelligent Power Saver	I don't know if it's as good as it sounds.	Jingdong prefecture level city in Shandong
Fortune Shop	Green Intelligent Power Saver	The quality of the stuff is very good, exactly the same as the seller's description, very satisfied!	Jingdong prefecture level city in Shandong
Fortune Shop	Green Intelligent Power Saver	The saver works great, it really works, it's affordable and worth buying.	Jingdong prefecture level city in Shandong
Fortune Shop	Green Intelligent Power Saver	Just used it for a couple days. I don't know yet.	Jingdong prefecture level city in Shandong
Fortune Shop	Green Intelligent Power Saver	The quality is quite good, cheaper than the physical store, good, next time will come again!	Jingdong prefecture level city in Shandong
Fortune Shop	Green Intelligent Power Saver	Good quality, cheap and affordable worth recommending to buy!	Jingdong prefecture level city in Shandong
Fortune Shop	Green Intelligent Power Saver	Very good quality, well made, works well! Satisfied	Jingdong prefecture level city in Shandong
Fortune Shop	Green Intelligent Power Saver	The logistics is very fast quality is very good very pleasant a shopping	Jingdong prefecture level city in Shandong
Fortune Shop	Green Intelligent Power Saver	Use it and then come back to evaluate!	Jingdong prefecture level city in Shandong
Fortune Shop	Green Intelligent Power Saver	# It's no use lying at all #	Jingdong prefecture level city in Shandong
Fortune Shop	Green Intelligent Power Saver	I can't get a voltage drop.	Jingdong prefecture level city in Shandong
Fortune Shop	Green Intelligent Power Saver	Evaluate after use	Jingdong prefecture level city in Shandong
Fortune Shop	Green Intelligent Power Saver	Still on trial.	Jingdong prefecture level city in Shandong
Fortune Shop	Green Intelligent Power Saver	ineffective	Jingdong prefecture level city in Shandong
Fortune Shop	Green Intelligent Power Saver	Satisfaction Shopping	Jingdong prefecture level city in Shandong
Fortune Shop	Green Intelligent Power Saver	Lies.	Jingdong prefecture level city in Shandong
Fortune Shop	Green Intelligent Power Saver	It's a real power saver.	Jingdong prefecture level city in Shandong
Fortune Shop	Green Intelligent Power Saver	in use	Jingdong prefecture level city in Shandong
Fortune Shop	Green Intelligent Power Saver	I don't know yet.	Jingdong prefecture level city in Shandong
Fortune Shop	Green Intelligent Power Saver	not care about anything	Jingdong prefecture level city in Shandong
Fortune Shop	Green Intelligent Power Saver	It doesn't seem to have much effect	Jingdong prefecture level city in Shandong
Fortune Shop	Green Intelligent Power Saver	Let the merchant see for himself, does the plug positive and negative terminals look like this?	Jingdong prefecture level city in Shandong
Fortune Shop	Green Intelligent Power Saver	It doesn't work well, the electricity bill is less before using it, but more after using it, contacting customer service, the service is still not good, and returning it is slow	Jingdong prefecture level city in Shandong
Fortune Shop	Green Intelligent Power Saver	With the advertisement product does not match, above the effective negative, power saving rate of two rows of words it, the product looks very thin, with 3 days to see the meter number is still with the previous no difference, which is obviously fraudulent behavior!	Jingdong prefecture level city in Shandong
Fortune Shop	Green Intelligent Power Saver	Doesn't save any power at all. Not much use.	Jingdong prefecture level city in Shandong
Fortune Shop	Green Intelligent Power Saver	The plugs are all loose, they're all the same length and they don't work at all	Jingdong prefecture level city in Shandong
Fortune Shop	Green Intelligent Power Saver	It's bad 👎 it's not effective at all, you'd be crazy to sell this garbage on the internet.	Jingdong prefecture level city in Shandong
Fortune Shop	Green Intelligent Power Saver	It doesn't save any power at all, so I hope the old timers don't fall for this scam again	Jingdong prefecture level city in Shandong
Fortune Shop	Green Intelligent Power Saver	I've been using it for over a month now and haven't noticed any power savings.	Jingdong prefecture level city in Shandong
Fortune Shop	Green Intelligent Power Saver	It doesn't work at all. The electricity bill is higher when you use it.	Jingdong prefecture level city in Shandong
Fortune Shop	Green Intelligent Power Saver	It doesn't save any power, it's still as bad as ever.	Jingdong prefecture level city in Shandong
Fortune Shop	Green Intelligent Power Saver	Last night before leaving work to look at the table, and then plugged into the node device, this morning and then look at the table, than the original save a few degrees of electricity, very good to have a power-saving effect, easy to use, satisfied with the shopping praise.	Jingdong prefecture level city in Shandong
Fortune Shop	Green Intelligent Power Saver	The product is real and effective, the electricity bill is reduced, really good, thanks to the merchants have so many good products, the product is real and effective, the electricity bill is reduced, want to save electricity friends quickly buy it, early use early to save money.	Jingdong prefecture level city in Shandong
Fortune Shop	Green Intelligent Power Saver	Saver saves time and money, baby appearance is beautiful, the quality of goods is good, and the same as the business description, good service attitude, packaging is complete, logistics is very fast, praise to	Jingdong prefecture level city in Shandong
Fortune Shop	Green Intelligent Power Saver	The effect is good, summer, home electricity is relatively large, buy and use a few days, feel the effect is very good, originally ten dollars a day electricity bill, now eight dollars a day, quite good	Jingdong prefecture level city in Shandong
Fortune Shop	Green Intelligent Power Saver	Indeed can save electricity and money, the first time I know this or from a friend's home to see, but also do not believe it, and now they use it to know that there is this good thing.	Jingdong prefecture level city in Shandong
Fortune Shop	Green Intelligent Power Saver	I have received the Smart Power Saver. Perfectly packaged. Fast delivery. Boss customer service attitude is also very good. It's worth buying. I plugged it in and tried it and it really saves more power than the original. I'll buy it again next time.	Jingdong prefecture level city in Shandong
Fortune Shop	Green Intelligent Power Saver	It's nice to save a dozen bucks a night on two air conditioners that used to take 30 or 40 degrees a night, but now it's only 10 or so degrees a night	Jingdong prefecture level city in Shandong
Fortune Shop	Green Intelligent Power Saver	Product no manufacturer's name no product qualification certificate, try two days to see the effect of	Jingdong prefecture level city in Shandong
Fortune Shop	Green Intelligent Power Saver	I've been using it for over a month and have saved over $80, so I've repurchased a few more back to save together	Jingdong prefecture level city in Shandong
Fortune Shop	Green Intelligent Power Saver	I haven't used it yet and will comment when I do, but it's well made.	Jingdong prefecture level city in Shandong

Fortune Shop	Green Intelligent Power Saver	It's quite energy efficient. It's really a boon for the home.	Jingdong prefecture level city in Shandong
Fortune Shop	Green Intelligent Power Saver	Energy-saving socket received a month, after using it for a month proved that it is indeed energy-saving, electricity bill saving, will buy it again, thank you!	Jingdong prefecture level city in Shandong
Fortune Shop	Green Intelligent Power Saver	There was a similar product many years ago. Now technology has evolved, buy one to try, according to the instructions in the box, it takes one or two dozen days to show results. Check your electric bill and see what happens.	Jingdong prefecture level city in Shandong
Fortune Shop	Green Intelligent Power Saver	Jingdong logistics is really fast, the same day the order arrived on the same day, more convenient than the supermarket, and a wide range of choices, the	Jingdong prefecture level city in Shandong
Fortune Shop	Green Intelligent Power Saver	I've been using this for a month or so and I can't see any results, it really is a scam as they say.	Jingdong prefecture level city in Shandong
Fortune Shop	Green Intelligent Power Saver	Very good use, a month can save a few degrees of power loss of electricity, introduced to the relatives to buy back to try.	Jingdong prefecture level city in Shandong
Fortune Shop	Green Intelligent Power Saver	It's really powerful! With this air conditioner can be turned on at any time without the pressure of paying the electricity bill! It's good for the people!	Jingdong prefecture level city in Shandong
Fortune Shop	Green Intelligent Power Saver	This business is a big ** products are not qualified, with a month of electricity more than one hundred percent of thirty, the purchase agreed to return the goods but do not receive a refund after receiving the goods, looking for the official side does not help, the Jingdong is a big **, I have never shopped in	Jingdong prefecture level city in Shandong
Fortune Shop	Green Intelligent Power Saver	It's good. I tried it. It saves power.	Jingdong prefecture level city in Shandong
Fortune Shop	Green Intelligent Power Saver	Nothing**, it seems like there's more	Jingdong prefecture level city in Shandong
Fortune Shop	Green Intelligent Power Saver	Power saving device received, well packaged, good quality, fast logistics	Jingdong prefecture level city in Shandong
Fortune Shop	Green Intelligent Power Saver	The service is okay, is that when I bought five, the desirable pieces are three, and then wait a few days and send me two replacement, I do not know if it is a mistake of the shipper, if I was not in the courier there to see, go home to say no, the	Jingdong prefecture level city in Shandong
Fortune Shop	Green Intelligent Power Saver	This thing will only cost me more if I use it, but before I used it, it was less than a ~ electricity a month, and after I used it, it was more than two hundred kWh a month.	Jingdong prefecture level city in Shandong
Fortune Shop	Green Intelligent Power Saver	I just used it. I don't know how it is.	Jingdong prefecture level city in Shandong
Fortune Shop	Green Intelligent Power Saver	I've received the product and I'm using it, so I'll see how it goes in a month!	Jingdong prefecture level city in Shandong
Fortune Shop	Green Intelligent Power Saver	The goods have just received the effect do not know how, please look forward to the results.	Jingdong prefecture level city in Shandong
Fortune Shop	Green Intelligent Power Saver	Power saving device, the material is very good practical and durable, the size is suitable, packaging is very good, the price is cheap, logistics is very fast!	Jingdong prefecture level city in Shandong
Fortune Shop	Green Intelligent Power Saver	Very nice, will comment in detail after using it for a while.	Jingdong prefecture level city in Shandong
Fortune Shop	Green Intelligent Power Saver	Good stuff to trust and support	Jingdong prefecture level city in Shandong
Fortune Shop	Green Intelligent Power Saver	I'll comment after I use it, doubt if it really saves power no	Jingdong prefecture level city in Shandong
Fortune Shop	Green Intelligent Power Saver	It's good quality and works well!	Jingdong prefecture level city in Shandong
Fortune Shop	Green Intelligent Power Saver	hahaha just right hahahahahaha hiccup hahahaha	Jingdong prefecture level city in Shandong
Fortune Shop	Green Intelligent Power Saver	The real thing matches the picture and looks great!	Jingdong prefecture level city in Shandong
Fortune Shop	Green Intelligent Power Saver	Plugged it in and we'll see when we pay the bills at the end of the year!	Jingdong prefecture level city in Shandong
Fortune Shop	Green Intelligent Power Saver	False advertising. It doesn't work.	Jingdong prefecture level city in Shandong
Fortune Shop	Green Intelligent Power Saver	Goods received, good effect	Jingdong prefecture level city in Shandong
Fortune Shop	Green Intelligent Power Saver	It looks okay, we'll see if it saves electricity when the time comes	Jingdong prefecture level city in Shandong
Fortune Shop	Green Intelligent Power Saver	Logistics is very fast quality is very good, the price is very affordable, accessories are also complete, installed, satisfied with good use	Jingdong prefecture level city in Shandong
Fortune Shop	Green Intelligent Power Saver	I've received the power saver. I'm looking forward to the savings.	Jingdong prefecture level city in Shandong
Fortune Shop	Green Intelligent Power Saver	Received, have not yet used, after use and then evaluate	Jingdong prefecture level city in Shandong
Fortune Shop	Green Intelligent Power Saver	I received the item, but I don't know if it saves power or not.	Jingdong prefecture level city in Shandong
Fortune Shop	Green Intelligent Power Saver	On Trial	Jingdong prefecture level city in Shandong
Fortune Shop	Green Intelligent Power Saver	Use it and see how it goes.	Jingdong prefecture level city in Shandong
Fortune Shop	Green Intelligent Power Saver	Quality is very good, no odor, fine workmanship, grain color are very like, satisfied!	Jingdong prefecture level city in Shandong
Fortune Shop	Green Intelligent Power Saver	I'll give you a good review. I don't know if it'll work.	Jingdong prefecture level city in Shandong
Fortune Shop	Green Intelligent Power Saver	The logistics is very fast and the package is intact. Good use of affordable. The effect is very good, is genuine	Jingdong prefecture level city in Shandong
Fortune Shop	Green Intelligent Power Saver	Nothing happens when you plug it in.	Jingdong prefecture level city in Shandong
Fortune Shop	Green Intelligent Power Saver	I don't know if it's as good as it sounds.	Jingdong prefecture level city in Shandong
Fortune Shop	Green Intelligent Power Saver	The quality of the stuff is very good, exactly the same as the seller's description, very satisfied!	Jingdong prefecture level city in Shandong
Fortune Shop	Green Intelligent Power Saver	The saver works great, it really works, it's affordable and worth buying.	Jingdong prefecture level city in Shandong
Fortune Shop	Green Intelligent Power Saver	Just used it for a couple days. I don't know yet.	Jingdong prefecture level city in Shandong
Fortune Shop	Green Intelligent Power Saver	The quality is quite good, cheaper than the physical store, good, next time will come again!	Jingdong prefecture level city in Shandong
Fortune Shop	Green Intelligent Power Saver	Good quality, cheap and affordable worth recommending to buy!	Jingdong prefecture level city in Shandong
Fortune Shop	Green Intelligent Power Saver	Very good quality, well made, works well! Satisfied	Jingdong prefecture level city in Shandong
Fortune Shop	Green Intelligent Power Saver	The logistics is very fast quality is very good very pleasant a shopping	Jingdong prefecture level city in Shandong
Fortune Shop	Green Intelligent Power Saver	Use it and then come back to evaluate!	Jingdong prefecture level city in Shandong
Fortune Shop	Green Intelligent Power Saver	# It's no use lying at all #	Jingdong prefecture level city in Shandong
Fortune Shop	Green Intelligent Power Saver	I can't get a voltage drop.	Jingdong prefecture level city in Shandong
Fortune Shop	Green Intelligent Power Saver	Evaluate after use	Jingdong prefecture level city in Shandong
Fortune Shop	Green Intelligent Power Saver	Still on trial.	Jingdong prefecture level city in Shandong

Fortune Shop	Green Intelligent Power Saver	ineffective	Jingdong prefecture level city in Shandong
Fortune Shop	Green Intelligent Power Saver	Satisfaction Shopping	Jingdong prefecture level city in Shandong
Fortune Shop	Green Intelligent Power Saver	Lies.	Jingdong prefecture level city in Shandong
Fortune Shop	Green Intelligent Power Saver	It's a real power saver.	Jingdong prefecture level city in Shandong
Fortune Shop	Green Intelligent Power Saver	in use	Jingdong prefecture level city in Shandong
Fortune Shop	Green Intelligent Power Saver	I don't know yet.	Jingdong prefecture level city in Shandong
Fortune Shop	Green Intelligent Power Saver	not care about anything	Jingdong prefecture level city in Shandong
Fortune Shop	Green Intelligent Power Saver	It doesn't seem to have much effect	Jingdong prefecture level city in Shandong
Fortune Shop	Green Intelligent Power Saver	Let the merchant see for himself, does the plug positive and negative terminals look like this?	Jingdong prefecture level city in Shandong
Fortune Shop	Green Intelligent Power Saver	It doesn't work well, the electricity bill is less before using it, but more after using it, contacting customer service, the service is still not good, and returning it is slow	Jingdong prefecture level city in Shandong
Fortune Shop	Green Intelligent Power Saver	With the advertisement product does not match, above the effective negative, power saving rate of two rows of words it, the product looks very thin, with 3 days to see the meter number is still with the previous no difference, which is obviously fraudulent behavior!	Jingdong prefecture level city in Shandong
Fortune Shop	Green Intelligent Power Saver	Doesn't save any power at all. Not much use.	Jingdong prefecture level city in Shandong
Fortune Shop	Green Intelligent Power Saver	The plugs are all loose, they're all the same length and they don't work at all	Jingdong prefecture level city in Shandong
Fortune Shop	Green Intelligent Power Saver	It's bad 👎 it's not effective at all, you'd be crazy to sell this garbage on the internet.	Jingdong prefecture level city in Shandong
Fortune Shop	Green Intelligent Power Saver	It doesn't save any power at all, so I hope the old timers don't fall for this scam again	Jingdong prefecture level city in Shandong
Fortune Shop	Green Intelligent Power Saver	I've been using it for over a month now and haven't noticed any power savings.	Jingdong prefecture level city in Shandong
Fortune Shop	Green Intelligent Power Saver	It doesn't work at all. The electricity bill is higher when you use it.	Jingdong prefecture level city in Shandong
Fortune Shop	Green Intelligent Power Saver	It doesn't save any power, it's still as bad as ever.	Jingdong prefecture level city in Shandong
Fortune Shop	Green Intelligent Power Saver	Last night before leaving work to look at the table, and then plugged into the node device, this morning and then look at the table, than the original save a few degrees of electricity, very good to have a power-saving effect, easy to use, satisfied with the shopping praise.	Jingdong prefecture level city in Shandong
Fortune Shop	Green Intelligent Power Saver	The product is real and effective, the electricity bill is reduced, really good, thanks to the merchants have so many good products, the product is real and effective, the electricity bill is reduced, want to save electricity friends quickly buy it, early use early to save money.	Jingdong prefecture level city in Shandong
Fortune Shop	Green Intelligent Power Saver	Saver saves time and money, baby appearance is beautiful, the quality of goods is good, and the same as the business description, good service attitude, packaging is complete, logistics is very fast, praise to	Jingdong prefecture level city in Shandong
Fortune Shop	Green Intelligent Power Saver	The effect is good, summer, home electricity is relatively large, buy and use a few days, feel the effect is very good, originally ten dollars a day electricity bill, now eight dollars a day, quite good	Jingdong prefecture level city in Shandong
Fortune Shop	Green Intelligent Power Saver	Indeed can save electricity and money, the first time I know this or from a friend's home to see, but also do not believe it, and now they use it to know that there is this good thing.	Jingdong prefecture level city in Shandong
Fortune Shop	Green Intelligent Power Saver	I have received the Smart Power Saver. Perfectly packaged. Fast delivery. Boss customer service attitude is also very good. It's worth buying. I plugged it in and tried it and it really saves more power than the original. I'll buy it again next time.	Jingdong prefecture level city in Shandong
Fortune Shop	Green Intelligent Power Saver	It's nice to save a dozen bucks a night on two air conditioners that used to take 30 or 40 degrees a night, but now it's only 10 or so degrees a night	Jingdong prefecture level city in Shandong
Fortune Shop	Green Intelligent Power Saver	Product no manufacturer's name no product qualification certificate, try two days to see the effect of	Jingdong prefecture level city in Shandong
Fortune Shop	Green Intelligent Power Saver	I've been using it for over a month and have saved over $80, so I've repurchased a few more back to save together	Jingdong prefecture level city in Shandong
Fortune Shop	Green Intelligent Power Saver	I haven't used it yet and will comment when I do, but it's well made.	Jingdong prefecture level city in Shandong
Fortune Shop	Green Intelligent Power Saver	It's quite energy efficient. It's really a boon for the home.	Jingdong prefecture level city in Shandong
Fortune Shop	Green Intelligent Power Saver	Energy-saving socket received a month, after using it for a month proved that it is indeed energy-saving, electricity bill saving, will buy it again, thank you!	Jingdong prefecture level city in Shandong
Fortune Shop	Green Intelligent Power Saver	There was a similar product many years ago. Now technology has evolved, buy one to try, according to the instructions in the box, it takes one or two dozen days to show results. Check your electric bill and see what happens.	Jingdong prefecture level city in Shandong
Fortune Shop	Green Intelligent Power Saver	Jingdong logistics is really fast, the same day the order arrived on the same day, more convenient than the supermarket, and a wide range of choices, the	Jingdong prefecture level city in Shandong
Fortune Shop	Green Intelligent Power Saver	I've been using this for a month or so and I can't see any results, it really is a scam as they say.	Jingdong prefecture level city in Shandong
Fortune Shop	Green Intelligent Power Saver	Very good use, a month can save a few degrees of power loss of electricity, introduced to the relatives to buy back to try.	Jingdong prefecture level city in Shandong
Fortune Shop	Green Intelligent Power Saver	It's really powerful! With this air conditioner can be turned on at any time without the pressure of paying the electricity bill! It's good for the people!	Jingdong prefecture level city in Shandong
Fortune Shop	Green Intelligent Power Saver	This business is a big ** products are not qualified, with a month of electricity more than one hundred percent of thirty, the purchase agreed to return the goods but do not receive a refund after receiving the goods, looking for the official side does not help, the Jingdong is a big **, I have never shopped in	Jingdong prefecture level city in Shandong
Fortune Shop	Green Intelligent Power Saver	It's good. I tried it. It saves power.	Jingdong prefecture level city in Shandong
Fortune Shop	Green Intelligent Power Saver	Nothing**, it seems like there's more	Jingdong prefecture level city in Shandong
Fortune Shop	Green Intelligent Power Saver	Power saving device received, well packaged, good quality, fast logistics	Jingdong prefecture level city in Shandong
Fortune Shop	Green Intelligent Power Saver	The service is okay, is that when I bought five, the desirable pieces are three, and then wait a few days and send me two replacement, I do not know if it is a mistake of the shipper, if I was not in the courier there to see, go home to say no, the	Jingdong prefecture level city in Shandong
Fortune Shop	Green Intelligent Power Saver	This thing will only cost me more if I use it, but before I used it, it was less than a ~ electricity a month, and after I used it, it was more than two hundred kWh a month.	Jingdong prefecture level city in Shandong
Fortune Shop	Green Intelligent Power Saver	I just used it. I don't know how it is.	Jingdong prefecture level city in Shandong
Fortune Shop	Green Intelligent Power Saver	I've received the product and I'm using it, so I'll see how it goes in a month!	Jingdong prefecture level city in Shandong
Fortune Shop	Green Intelligent Power Saver	The goods have just received the effect do not know how, please look forward to the results.	Jingdong prefecture level city in Shandong
Fortune Shop	Green Intelligent Power Saver	Power saving device, the material is very good practical and durable, the size is suitable, packaging is very good, the price is cheap, logistics is very fast!	Jingdong prefecture level city in Shandong
Fortune Shop	Green Intelligent Power Saver	Very nice, will comment in detail after using it for a while.	Jingdong prefecture level city in Shandong
Fortune Shop	Green Intelligent Power Saver	Good stuff to trust and support	Jingdong prefecture level city in Shandong
Fortune Shop	Green Intelligent Power Saver	I'll comment after I use it, doubt if it really saves power no	Jingdong prefecture level city in Shandong

Fortune Shop	Green Intelligent Power Saver	It's good quality and works well!	Jingdong prefecture level city in Shandong
Fortune Shop	Green Intelligent Power Saver	hahaha just right hahahahahaha hiccup hahahaha	Jingdong prefecture level city in Shandong
Fortune Shop	Green Intelligent Power Saver	The real thing matches the picture and looks great!	Jingdong prefecture level city in Shandong
Fortune Shop	Green Intelligent Power Saver	Plugged it in and we'll see when we pay the bills at the end of the year!	Jingdong prefecture level city in Shandong
Fortune Shop	Green Intelligent Power Saver	False advertising. It doesn't work.	Jingdong prefecture level city in Shandong
Fortune Shop	Green Intelligent Power Saver	Goods received, good effect	Jingdong prefecture level city in Shandong
Fortune Shop	Green Intelligent Power Saver	It looks okay, we'll see if it saves electricity when the time comes	Jingdong prefecture level city in Shandong
Fortune Shop	Green Intelligent Power Saver	Logistics is very fast quality is very good, the price is very affordable, accessories are also complete, installed, satisfied with good use	Jingdong prefecture level city in Shandong
Fortune Shop	Green Intelligent Power Saver	I've received the power saver. I'm looking forward to the savings.	Jingdong prefecture level city in Shandong
Fortune Shop	Green Intelligent Power Saver	Received, have not yet used, after use and then evaluate	Jingdong prefecture level city in Shandong
Fortune Shop	Green Intelligent Power Saver	I received the item, but I don't know if it saves power or not.	Jingdong prefecture level city in Shandong
Fortune Shop	Green Intelligent Power Saver	On Trial	Jingdong prefecture level city in Shandong
Fortune Shop	Green Intelligent Power Saver	Use it and see how it goes.	Jingdong prefecture level city in Shandong
Fortune Shop	Green Intelligent Power Saver	Quality is very good, no odor, fine workmanship, grain color are very like, satisfied!	Jingdong prefecture level city in Shandong
Fortune Shop	Green Intelligent Power Saver	I'll give you a good review. I don't know if it'll work.	Jingdong prefecture level city in Shandong
Fortune Shop	Green Intelligent Power Saver	The logistics is very fast and the package is intact. Good use of affordable. The effect is very good, is genuine	Jingdong prefecture level city in Shandong
Fortune Shop	Green Intelligent Power Saver	Nothing happens when you plug it in.	Jingdong prefecture level city in Shandong
Fortune Shop	Green Intelligent Power Saver	I don't know if it's as good as it sounds.	Jingdong prefecture level city in Shandong
Fortune Shop	Green Intelligent Power Saver	The quality of the stuff is very good, exactly the same as the seller's description, very satisfied!	Jingdong prefecture level city in Shandong
Fortune Shop	Green Intelligent Power Saver	The saver works great, it really works, it's affordable and worth buying.	Jingdong prefecture level city in Shandong
Fortune Shop	Green Intelligent Power Saver	Just used it for a couple days. I don't know yet.	Jingdong prefecture level city in Shandong
Fortune Shop	Green Intelligent Power Saver	The quality is quite good, cheaper than the physical store, good, next time will come again!	Jingdong prefecture level city in Shandong
Fortune Shop	Green Intelligent Power Saver	Good quality, cheap and affordable worth recommending to buy!	Jingdong prefecture level city in Shandong
Fortune Shop	Green Intelligent Power Saver	Very good quality, well made, works well! Satisfied	Jingdong prefecture level city in Shandong
Fortune Shop	Green Intelligent Power Saver	The logistics is very fast quality is very good very pleasant a shopping	Jingdong prefecture level city in Shandong
Fortune Shop	Green Intelligent Power Saver	Use it and then come back to evaluate!	Jingdong prefecture level city in Shandong
Fortune Shop	Green Intelligent Power Saver	# It's no use lying at all #	Jingdong prefecture level city in Shandong
Fortune Shop	Green Intelligent Power Saver	I can't get a voltage drop.	Jingdong prefecture level city in Shandong
Fortune Shop	Green Intelligent Power Saver	Evaluate after use	Jingdong prefecture level city in Shandong
Fortune Shop	Green Intelligent Power Saver	Still on trial.	Jingdong prefecture level city in Shandong
Fortune Shop	Green Intelligent Power Saver	ineffective	Jingdong prefecture level city in Shandong
Fortune Shop	Green Intelligent Power Saver	Satisfaction Shopping	Jingdong prefecture level city in Shandong
Fortune Shop	Green Intelligent Power Saver	Lies.	Jingdong prefecture level city in Shandong
Fortune Shop	Green Intelligent Power Saver	It's a real power saver.	Jingdong prefecture level city in Shandong
Fortune Shop	Green Intelligent Power Saver	in use	Jingdong prefecture level city in Shandong
Fortune Shop	Green Intelligent Power Saver	I don't know yet.	Jingdong prefecture level city in Shandong
Fortune Shop	Green Intelligent Power Saver	not care about anything	Jingdong prefecture level city in Shandong
Fortune Shop	Green Intelligent Power Saver	It doesn't seem to have much effect	Jingdong prefecture level city in Shandong
Fortune Shop	Green Intelligent Power Saver	Let the merchant see for himself, does the plug positive and negative terminals look like this?	Jingdong prefecture level city in Shandong
Fortune Shop	Green Intelligent Power Saver	It doesn't work well, the electricity bill is less before using it, but more after using it, contacting customer service, the service is still not good, and returning it is slow	Jingdong prefecture level city in Shandong
Fortune Shop	Green Intelligent Power Saver	With the advertisement product does not match, above the effective negative, power saving rate of two rows of words it, the product looks very thin, with 3 days to see the meter number is still with the previous no difference, which is obviously fraudulent behavior!	Jingdong prefecture level city in Shandong
Fortune Shop	Green Intelligent Power Saver	Doesn't save any power at all. Not much use.	Jingdong prefecture level city in Shandong
Fortune Shop	Green Intelligent Power Saver	The plugs are all loose, they're all the same length and they don't work at all	Jingdong prefecture level city in Shandong
Fortune Shop	Green Intelligent Power Saver	It's bad 👎 it's not effective at all, you'd be crazy to sell this garbage on the internet.	Jingdong prefecture level city in Shandong
Fortune Shop	Green Intelligent Power Saver	It doesn't save any power at all, so I hope the old timers don't fall for this scam again	Jingdong prefecture level city in Shandong
Fortune Shop	Green Intelligent Power Saver	I've been using it for over a month now and haven't noticed any power savings.	Jingdong prefecture level city in Shandong
Fortune Shop	Green Intelligent Power Saver	It doesn't work at all. The electricity bill is higher when you use it.	Jingdong prefecture level city in Shandong
Fortune Shop	Green Intelligent Power Saver	It doesn't save any power, it's still as bad as ever.	Jingdong prefecture level city in Shandong
Fortune Shop	Green Intelligent Power Saver	Last night before leaving work to look at the table, and then plugged into the node device, this morning and then look at the table, than the original save a few degrees of electricity, very good to have a power-saving effect, easy to use, satisfied with the shopping praise.	Jingdong prefecture level city in Shandong
Fortune Shop	Green Intelligent Power Saver	The product is real and effective, the electricity bill is reduced, really good, thanks to the merchants have so many good products, the product is real and effective, the electricity bill is reduced, want to save electricity friends quickly buy it, early use early to save money.	Jingdong prefecture level city in Shandong

Fortune Shop	Green Intelligent Power Saver	Saver saves time and money, baby appearance is beautiful, the quality of goods is good, and the same as the business description, good service attitude, packaging is complete, logistics is very fast, praise to	Jingdong prefecture level city in Shandong
Fortune Shop	Green Intelligent Power Saver	The effect is good, summer, home electricity is relatively large, buy and use a few days, feel the effect is very good, originally ten dollars a day electricity bill, now eight dollars a day, quite good	Jingdong prefecture level city in Shandong
Fortune Shop	Green Intelligent Power Saver	Indeed can save electricity and money, the first time I know this or from a friend's home to see, but also do not believe it, and now they use it to know that there is this good thing.	Jingdong prefecture level city in Shandong
Fortune Shop	Green Intelligent Power Saver	I have received the Smart Power Saver. Perfectly packaged. Fast delivery. Boss customer service attitude is also very good. It's worth buying. I plugged it in and tried it and it really saves more power than the original. I'll buy it again next time.	Jingdong prefecture level city in Shandong
Fortune Shop	Green Intelligent Power Saver	It's nice to save a dozen bucks a night on two air conditioners that used to take 30 or 40 degrees a night, but now it's only 10 or so degrees a night	Jingdong prefecture level city in Shandong
Fortune Shop	Green Intelligent Power Saver	Product no manufacturer's name no product qualification certificate, try two days to see the effect of	Jingdong prefecture level city in Shandong
Fortune Shop	Green Intelligent Power Saver	I've been using it for over a month and have saved over $80, so I've repurchased a few more back to save together	Jingdong prefecture level city in Shandong
Fortune Shop	Green Intelligent Power Saver	I haven't used it yet and will comment when I do, but it's well made.	Jingdong prefecture level city in Shandong
Fortune Shop	Green Intelligent Power Saver	It's quite energy efficient. It's really a boon for the home.	Jingdong prefecture level city in Shandong
Fortune Shop	Green Intelligent Power Saver	Energy-saving socket received a month, after using it for a month proved that it is indeed energy-saving, electricity bill saving, will buy it again, thank you!	Jingdong prefecture level city in Shandong
Fortune Shop	Green Intelligent Power Saver	There was a similar product many years ago. Now technology has evolved, buy one to try, according to the instructions in the box, it takes one or two dozen days to show results. Check your electric bill and see what happens.	Jingdong prefecture level city in Shandong
Fortune Shop	Green Intelligent Power Saver	Jingdong logistics is really fast, the same day the order arrived on the same day, more convenient than the supermarket, and a wide range of choices, the	Jingdong prefecture level city in Shandong
Fortune Shop	Green Intelligent Power Saver	I've been using this for a month or so and I can't see any results, it really is a scam as they say.	Jingdong prefecture level city in Shandong
Fortune Shop	Green Intelligent Power Saver	Very good use, a month can save a few degrees of power loss of electricity, introduced to the relatives to buy back to try.	Jingdong prefecture level city in Shandong
Fortune Shop	Green Intelligent Power Saver	It's really powerful! With this air conditioner can be turned on at any time without the pressure of paying the electricity bill! It's good for the people!	Jingdong prefecture level city in Shandong
Fortune Shop	Green Intelligent Power Saver	This business is a big ** products are not qualified, with a month of electricity more than one hundred percent of thirty, the purchase agreed to return the goods but do not receive a refund after receiving the goods, looking for the official side does not help, the Jingdong is a big **, I have never shopped in	Jingdong prefecture level city in Shandong
Fortune Shop	Green Intelligent Power Saver	It's good. I tried it. It saves power.	Jingdong prefecture level city in Shandong
Fortune Shop	Green Intelligent Power Saver	Nothing**, it seems like there's more	Jingdong prefecture level city in Shandong
Fortune Shop	Green Intelligent Power Saver	Power saving device received, well packaged, good quality, fast logistics	Jingdong prefecture level city in Shandong
Fortune Shop	Green Intelligent Power Saver	The service is okay, is that when I bought five, the desirable pieces are three, and then wait a few days and send me two replacement, I do not know if it is a mistake of the shipper, if I was not in the courier there to see, go home to say no, the	Jingdong prefecture level city in Shandong
Fortune Shop	Green Intelligent Power Saver	This thing will only cost me more if I use it, but before I used it, it was less than a ` electricity a month, and after I used it, it was more than two hundred kWh a month.	Jingdong prefecture level city in Shandong
Fortune Shop	Green Intelligent Power Saver	I just used it. I don't know how it is.	Jingdong prefecture level city in Shandong
Fortune Shop	Green Intelligent Power Saver	I've received the product and I'm using it, so I'll see how it goes in a month!	Jingdong prefecture level city in Shandong
Fortune Shop	Green Intelligent Power Saver	The goods have just received the effect do not know how, please look forward to the results.	Jingdong prefecture level city in Shandong
Fortune Shop	Green Intelligent Power Saver	Power saving device, the material is very good practical and durable, the size is suitable, packaging is very good, the price is cheap, logistics is very fast!	Jingdong prefecture level city in Shandong
Fortune Shop	Green Intelligent Power Saver	Very nice, will comment in detail after using it for a while.	Jingdong prefecture level city in Shandong
Fortune Shop	Green Intelligent Power Saver	Good stuff to trust and support	Jingdong prefecture level city in Shandong
Fortune Shop	Green Intelligent Power Saver	I'll comment after I use it, doubt if it really saves power no	Jingdong prefecture level city in Shandong
Fortune Shop	Green Intelligent Power Saver	It's good quality and works well!	Jingdong prefecture level city in Shandong
Fortune Shop	Green Intelligent Power Saver	hahaha just right hahahahahaha hiccup hahahaha	Jingdong prefecture level city in Shandong
Fortune Shop	Green Intelligent Power Saver	The real thing matches the picture and looks great!	Jingdong prefecture level city in Shandong
Fortune Shop	Green Intelligent Power Saver	Plugged it in and we'll see when we pay the bills at the end of the year!	Jingdong prefecture level city in Shandong
Fortune Shop	Green Intelligent Power Saver	False advertising. It doesn't work.	Jingdong prefecture level city in Shandong
Fortune Shop	Green Intelligent Power Saver	Goods received, good effect	Jingdong prefecture level city in Shandong
Fortune Shop	Green Intelligent Power Saver	It looks okay, we'll see if it saves electricity when the time comes	Jingdong prefecture level city in Shandong
Fortune Shop	Green Intelligent Power Saver	Logistics is very fast quality is very good, the price is very affordable, accessories are also complete, installed, satisfied with good use	Jingdong prefecture level city in Shandong
Fortune Shop	Green Intelligent Power Saver	I've received the power saver. I'm looking forward to the savings.	Jingdong prefecture level city in Shandong
Fortune Shop	Green Intelligent Power Saver	Received, have not yet used, after use and then evaluate	Jingdong prefecture level city in Shandong
Fortune Shop	Green Intelligent Power Saver	I received the item, but I don't know if it saves power or not.	Jingdong prefecture level city in Shandong
Fortune Shop	Green Intelligent Power Saver	On Trial	Jingdong prefecture level city in Shandong
Fortune Shop	Green Intelligent Power Saver	Use it and see how it goes.	Jingdong prefecture level city in Shandong
Fortune Shop	Green Intelligent Power Saver	Quality is very good, no odor, fine workmanship, grain color are very like, satisfied!	Jingdong prefecture level city in Shandong
Fortune Shop	Green Intelligent Power Saver	I'll give you a good review. I don't know if it'll work.	Jingdong prefecture level city in Shandong
Fortune Shop	Green Intelligent Power Saver	The logistics is very fast and the package is intact. Good use of affordable. The effect is very good, is genuine	Jingdong prefecture level city in Shandong
Fortune Shop	Green Intelligent Power Saver	Nothing happens when you plug it in.	Jingdong prefecture level city in Shandong
Fortune Shop	Green Intelligent Power Saver	I don't know if it's as good as it sounds.	Jingdong prefecture level city in Shandong
Fortune Shop	Green Intelligent Power Saver	The quality of the stuff is very good, exactly the same as the seller's description, very satisfied!	Jingdong prefecture level city in Shandong
Fortune Shop	Green Intelligent Power Saver	The saver works great, it really works, it's affordable and worth buying.	Jingdong prefecture level city in Shandong
Fortune Shop	Green Intelligent Power Saver	Just used it for a couple days. I don't know yet.	Jingdong prefecture level city in Shandong

Fortune Shop	Green Intelligent Power Saver	The quality is quite good, cheaper than the physical store, good, next time will come again!	Jingdong prefecture level city in Shandong
Fortune Shop	Green Intelligent Power Saver	Good quality, cheap and affordable worth recommending to buy!	Jingdong prefecture level city in Shandong
Fortune Shop	Green Intelligent Power Saver	Very good quality, well made, works well! Satisfied	Jingdong prefecture level city in Shandong
Fortune Shop	Green Intelligent Power Saver	The logistics is very fast quality is very good very pleasant a shopping	Jingdong prefecture level city in Shandong
Fortune Shop	Green Intelligent Power Saver	Use it and then come back to evaluate!	Jingdong prefecture level city in Shandong
Fortune Shop	Green Intelligent Power Saver	# It's no use lying at all #	Jingdong prefecture level city in Shandong
Fortune Shop	Green Intelligent Power Saver	I can't get a voltage drop.	Jingdong prefecture level city in Shandong
Fortune Shop	Green Intelligent Power Saver	Evaluate after use	Jingdong prefecture level city in Shandong
Fortune Shop	Green Intelligent Power Saver	Still on trial.	Jingdong prefecture level city in Shandong
Fortune Shop	Green Intelligent Power Saver	ineffective	Jingdong prefecture level city in Shandong
Fortune Shop	Green Intelligent Power Saver	Satisfaction Shopping	Jingdong prefecture level city in Shandong
Fortune Shop	Green Intelligent Power Saver	Lies.	Jingdong prefecture level city in Shandong
Fortune Shop	Green Intelligent Power Saver	It's a real power saver.	Jingdong prefecture level city in Shandong
Fortune Shop	Green Intelligent Power Saver	in use	Jingdong prefecture level city in Shandong
Fortune Shop	Green Intelligent Power Saver	I don't know yet.	Jingdong prefecture level city in Shandong
Fortune Shop	Green Intelligent Power Saver	not care about anything	Jingdong prefecture level city in Shandong
Fortune Shop	Green Intelligent Power Saver	It doesn't seem to have much effect	Jingdong prefecture level city in Shandong
Fortune Shop	Green Intelligent Power Saver	Let the merchant see for himself, does the plug positive and negative terminals look like this?	Jingdong prefecture level city in Shandong
Fortune Shop	Green Intelligent Power Saver	It doesn't work well, the electricity bill is less before using it, but more after using it, contacting customer service, the service is still not good, and returning it is slow	Jingdong prefecture level city in Shandong
Fortune Shop	Green Intelligent Power Saver	With the advertisement product does not match, above the effective negative, power saving rate of two rows of words it, the product looks very thin, with 3 days to see the meter number is still with the previous no difference, which is obviously fraudulent behavior!	Jingdong prefecture level city in Shandong
Fortune Shop	Green Intelligent Power Saver	Doesn't save any power at all. Not much use.	Jingdong prefecture level city in Shandong
Fortune Shop	Green Intelligent Power Saver	The plugs are all loose, they're all the same length and they don't work at all	Jingdong prefecture level city in Shandong
Fortune Shop	Green Intelligent Power Saver	It's bad 👎 it's not effective at all, you'd be crazy to sell this garbage on the internet.	Jingdong prefecture level city in Shandong
Fortune Shop	Green Intelligent Power Saver	It doesn't save any power at all, so I hope the old timers don't fall for this scam again	Jingdong prefecture level city in Shandong
Fortune Shop	Green Intelligent Power Saver	I've been using it for over a month now and haven't noticed any power savings.	Jingdong prefecture level city in Shandong
Fortune Shop	Green Intelligent Power Saver	It doesn't work at all. The electricity bill is higher when you use it.	Jingdong prefecture level city in Shandong
Fortune Shop	Green Intelligent Power Saver	It doesn't save any power, it's still as bad as ever.	Jingdong prefecture level city in Shandong
Fortune Shop	Green Intelligent Power Saver	Last night before leaving work to look at the table, and then plugged into the node device, this morning and then look at the table, than the original save a few degrees of electricity, very good to have a power-saving effect, easy to use, satisfied with the shopping praise.	Jingdong prefecture level city in Shandong
Fortune Shop	Green Intelligent Power Saver	The product is real and effective, the electricity bill is reduced, really good, thanks to the merchants have so many good products, the product is real and effective, the electricity bill is reduced, want to save electricity friends quickly buy it, early use early to save money.	Jingdong prefecture level city in Shandong
Fortune Shop	Green Intelligent Power Saver	Saver saves time and money, baby appearance is beautiful, the quality of goods is good, and the same as the business description, good service attitude, packaging is complete, logistics is very fast, praise to	Jingdong prefecture level city in Shandong
Fortune Shop	Green Intelligent Power Saver	The effect is good, summer, home electricity is relatively large, buy and use a few days, feel the effect is very good, originally ten dollars a day electricity bill, now eight dollars a day, quite good	Jingdong prefecture level city in Shandong
Fortune Shop	Green Intelligent Power Saver	Indeed can save electricity and money, the first time I know this or from a friend's home to see, but also do not believe it, and now they use it to know that there is this good thing.	Jingdong prefecture level city in Shandong
Fortune Shop	Green Intelligent Power Saver	I have received the Smart Power Saver. Perfectly packaged. Fast delivery. Boss customer service attitude is also very good. It's worth buying. I plugged it in and tried it and it really saves more power than the original. I'll buy it again next time.	Jingdong prefecture level city in Shandong
Fortune Shop	Green Intelligent Power Saver	It's nice to save a dozen bucks a night on two air conditioners that used to take 30 or 40 degrees a night, but now it's only 10 or so degrees a night	Jingdong prefecture level city in Shandong
Fortune Shop	Green Intelligent Power Saver	Product no manufacturer's name no product qualification certificate, try two days to see the effect of	Jingdong prefecture level city in Shandong
Fortune Shop	Green Intelligent Power Saver	I've been using it for over a month and have saved over $80, so I've repurchased a few more back to save together	Jingdong prefecture level city in Shandong
Fortune Shop	Green Intelligent Power Saver	I haven't used it yet and will comment when I do, but it's well made.	Jingdong prefecture level city in Shandong
Fortune Shop	Green Intelligent Power Saver	It's quite energy efficient. It's really a boon for the home.	Jingdong prefecture level city in Shandong
Fortune Shop	Green Intelligent Power Saver	Energy-saving socket received a month, after using it for a month proved that it is indeed energy-saving, electricity bill saving, will buy it again, thank you!	Jingdong prefecture level city in Shandong
Fortune Shop	Green Intelligent Power Saver	There was a similar product many years ago. Now technology has evolved, buy one to try, according to the instructions in the box, it takes one or two dozen days to show results. Check your electric bill and see what happens.	Jingdong prefecture level city in Shandong
Fortune Shop	Green Intelligent Power Saver	Jingdong logistics is really fast, the same day the order arrived on the same day, more convenient than the supermarket, and a wide range of choices, the	Jingdong prefecture level city in Shandong
Fortune Shop	Green Intelligent Power Saver	I've been using this for a month or so and I can't see any results, it really is a scam as they say.	Jingdong prefecture level city in Shandong
Fortune Shop	Green Intelligent Power Saver	Very good use, a month can save a few degrees of power loss of electricity, introduced to the relatives to buy back to try.	Jingdong prefecture level city in Shandong
Fortune Shop	Green Intelligent Power Saver	It's really powerful! With this air conditioner can be turned on at any time without the pressure of paying the electricity bill! It's good for the people!	Jingdong prefecture level city in Shandong
Fortune Shop	Green Intelligent Power Saver	This business is a big ** products are not qualified, with a month of electricity more than one hundred percent of thirty, the purchase agreed to return the goods but do not receive a refund after receiving the goods, looking for the official side does not help, the Jingdong is a big **, I have never shopped in	Jingdong prefecture level city in Shandong
Fortune Shop	Green Intelligent Power Saver	It's good. I tried it. It saves power.	Jingdong prefecture level city in Shandong
Fortune Shop	Green Intelligent Power Saver	Nothing**, it seems like there's more	Jingdong prefecture level city in Shandong
Fortune Shop	Green Intelligent Power Saver	Power saving device received, well packaged, good quality, fast logistics	Jingdong prefecture level city in Shandong

Fortune Shop	Green Intelligent Power Saver	The service is okay, is that when I bought five, the desirable pieces are three, and then wait a few days and send me two replacement, I do not know if it is a mistake of the shipper, if I was not in the courier there to see, go home to say no, the	Jingdong prefecture level city in Shandong
Fortune Shop	Green Intelligent Power Saver	This thing will only cost me more if I use it, but before I used it, it was less than a ~ electricity a month, and after I used it, it was more than two hundred kWh a month.	Jingdong prefecture level city in Shandong
Fortune Shop	Green Intelligent Power Saver	I just used it. I don't know how it is.	Jingdong prefecture level city in Shandong
Fortune Shop	Green Intelligent Power Saver	I've received the product and I'm using it, so I'll see how it goes in a month!	Jingdong prefecture level city in Shandong
Fortune Shop	Green Intelligent Power Saver	The goods have just received the effect do not know how, please look forward to the results.	Jingdong prefecture level city in Shandong
Fortune Shop	Green Intelligent Power Saver	Power saving device, the material is very good practical and durable, the size is suitable, packaging is very good, the price is cheap, logistics is very fast!	Jingdong prefecture level city in Shandong
Fortune Shop	Green Intelligent Power Saver	Very nice, will comment in detail after using it for a while.	Jingdong prefecture level city in Shandong
Fortune Shop	Green Intelligent Power Saver	Good stuff to trust and support	Jingdong prefecture level city in Shandong
Fortune Shop	Green Intelligent Power Saver	I'll comment after I use it, doubt if it really saves power no	Jingdong prefecture level city in Shandong
Fortune Shop	Green Intelligent Power Saver	It's good quality and works well!	Jingdong prefecture level city in Shandong
Fortune Shop	Green Intelligent Power Saver	hahaha just right hahahahahaha hiccup hahahaha	Jingdong prefecture level city in Shandong
Fortune Shop	Green Intelligent Power Saver	The real thing matches the picture and looks great!	Jingdong prefecture level city in Shandong
Fortune Shop	Green Intelligent Power Saver	Plugged it in and we'll see when we pay the bills at the end of the year!	Jingdong prefecture level city in Shandong
Fortune Shop	Green Intelligent Power Saver	False advertising. It doesn't work.	Jingdong prefecture level city in Shandong
Fortune Shop	Green Intelligent Power Saver	Goods received, good effect	Jingdong prefecture level city in Shandong
Fortune Shop	Green Intelligent Power Saver	It looks okay, we'll see if it saves electricity when the time comes	Jingdong prefecture level city in Shandong
Fortune Shop	Green Intelligent Power Saver	Logistics is very fast quality is very good, the price is very affordable, accessories are also complete, installed, satisfied with good use	Jingdong prefecture level city in Shandong
Fortune Shop	Green Intelligent Power Saver	I've received the power saver. I'm looking forward to the savings.	Jingdong prefecture level city in Shandong
Fortune Shop	Green Intelligent Power Saver	Received, have not yet used, after use and then evaluate	Jingdong prefecture level city in Shandong
Fortune Shop	Green Intelligent Power Saver	I received the item, but I don't know if it saves power or not.	Jingdong prefecture level city in Shandong
Fortune Shop	Green Intelligent Power Saver	On Trial	Jingdong prefecture level city in Shandong
Fortune Shop	Green Intelligent Power Saver	Use it and see how it goes.	Jingdong prefecture level city in Shandong
Fortune Shop	Green Intelligent Power Saver	Quality is very good, no odor, fine workmanship, grain color are very like, satisfied!	Jingdong prefecture level city in Shandong
Fortune Shop	Green Intelligent Power Saver	I'll give you a good review. I don't know if it'll work.	Jingdong prefecture level city in Shandong
Fortune Shop	Green Intelligent Power Saver	The logistics is very fast and the package is intact. Good use of affordable. The effect is very good, is genuine	Jingdong prefecture level city in Shandong
Fortune Shop	Green Intelligent Power Saver	Nothing happens when you plug it in.	Jingdong prefecture level city in Shandong
Fortune Shop	Green Intelligent Power Saver	I don't know if it's as good as it sounds.	Jingdong prefecture level city in Shandong
Fortune Shop	Green Intelligent Power Saver	The quality of the stuff is very good, exactly the same as the seller's description, very satisfied!	Jingdong prefecture level city in Shandong
Fortune Shop	Green Intelligent Power Saver	The saver works great, it really works, it's affordable and worth buying.	Jingdong prefecture level city in Shandong
Fortune Shop	Green Intelligent Power Saver	Just used it for a couple days. I don't know yet.	Jingdong prefecture level city in Shandong
Fortune Shop	Green Intelligent Power Saver	The quality is quite good, cheaper than the physical store, good, next time will come again!	Jingdong prefecture level city in Shandong
Fortune Shop	Green Intelligent Power Saver	Good quality, cheap and affordable worth recommending to buy!	Jingdong prefecture level city in Shandong
Fortune Shop	Green Intelligent Power Saver	Very good quality, well made, works well! Satisfied	Jingdong prefecture level city in Shandong
Fortune Shop	Green Intelligent Power Saver	The logistics is very fast quality is very good very pleasant a shopping	Jingdong prefecture level city in Shandong
Fortune Shop	Green Intelligent Power Saver	Use it and then come back to evaluate!	Jingdong prefecture level city in Shandong
Fortune Shop	Green Intelligent Power Saver	# It's no use lying at all #	Jingdong prefecture level city in Shandong
Fortune Shop	Green Intelligent Power Saver	I can't get a voltage drop.	Jingdong prefecture level city in Shandong
Fortune Shop	Green Intelligent Power Saver	Evaluate after use	Jingdong prefecture level city in Shandong
Fortune Shop	Green Intelligent Power Saver	Still on trial.	Jingdong prefecture level city in Shandong
Fortune Shop	Green Intelligent Power Saver	ineffective	Jingdong prefecture level city in Shandong
Fortune Shop	Green Intelligent Power Saver	Satisfaction Shopping	Jingdong prefecture level city in Shandong
Fortune Shop	Green Intelligent Power Saver	Lies.	Jingdong prefecture level city in Shandong
Fortune Shop	Green Intelligent Power Saver	It's a real power saver.	Jingdong prefecture level city in Shandong
Fortune Shop	Green Intelligent Power Saver	in use	Jingdong prefecture level city in Shandong
Fortune Shop	Green Intelligent Power Saver	I don't know yet.	Jingdong prefecture level city in Shandong
Fortune Shop	Green Intelligent Power Saver	not care about anything	Jingdong prefecture level city in Shandong
Fortune Shop	Green Intelligent Power Saver	It doesn't seem to have much effect	Jingdong prefecture level city in Shandong
Fortune Shop	Green Intelligent Power Saver	Let the merchant see for himself, does the plug positive and negative terminals look like this?	Jingdong prefecture level city in Shandong
Fortune Shop	Green Intelligent Power Saver	It doesn't work well, the electricity bill is less before using it, but more after using it, contacting customer service, the service is still not good, and returning it is slow	Jingdong prefecture level city in Shandong
Fortune Shop	Green Intelligent Power Saver	With the advertisement product does not match, above the effective negative, power saving rate of two rows of words it, the product looks very thin, with 3 days to see the meter number is still with the previous no difference, which is obviously fraudulent behavior!	Jingdong prefecture level city in Shandong

Fortune Shop	Green Intelligent Power Saver	Doesn't save any power at all. Not much use.	Jingdong prefecture level city in Shandong
Fortune Shop	Green Intelligent Power Saver	The plugs are all loose, they're all the same length and they don't work at all	Jingdong prefecture level city in Shandong
Fortune Shop	Green Intelligent Power Saver	It's bad 👎 it's not effective at all, you'd be crazy to sell this garbage on the internet.	Jingdong prefecture level city in Shandong
Fortune Shop	Green Intelligent Power Saver	It doesn't save any power at all, so I hope the old timers don't fall for this scam again	Jingdong prefecture level city in Shandong
Fortune Shop	Green Intelligent Power Saver	I've been using it for over a month now and haven't noticed any power savings.	Jingdong prefecture level city in Shandong
Fortune Shop	Green Intelligent Power Saver	It doesn't work at all. The electricity bill is higher when you use it.	Jingdong prefecture level city in Shandong
Fortune Shop	Green Intelligent Power Saver	It doesn't save any power, it's still as bad as ever.	Jingdong prefecture level city in Shandong
Fortune Shop	Green Intelligent Power Saver	Last night before leaving work to look at the table, and then plugged into the node device, this morning and then look at the table, than the original save a few degrees of electricity, very good to have a power-saving effect, easy to use, satisfied with the shopping praise.	Jingdong prefecture level city in Shandong
Fortune Shop	Green Intelligent Power Saver	The product is real and effective, the electricity bill is reduced, really good, thanks to the merchants have so many good products, the product is real and effective, the electricity bill is reduced, want to save electricity friends quickly buy it, early use early to save money.	Jingdong prefecture level city in Shandong
Fortune Shop	Green Intelligent Power Saver	Saver saves time and money, baby appearance is beautiful, the quality of goods is good, and the same as the business description, good service attitude, packaging is complete, logistics is very fast, praise to	Jingdong prefecture level city in Shandong
Fortune Shop	Green Intelligent Power Saver	The effect is good, summer, home electricity is relatively large, buy and use a few days, feel the effect is very good, originally ten dollars a day electricity bill, now eight dollars a day, quite good	Jingdong prefecture level city in Shandong
Fortune Shop	Green Intelligent Power Saver	Indeed can save electricity and money, the first time I know this or from a friend's home to see, but also do not believe it, and now they use it to know that there is this good thing.	Jingdong prefecture level city in Shandong
Fortune Shop	Green Intelligent Power Saver	I have received the Smart Power Saver. Perfectly packaged. Fast delivery. Boss customer service attitude is also very good. It's worth buying. I plugged it in and tried it and it really saves more power than the original. I'll buy it again next time.	Jingdong prefecture level city in Shandong
Fortune Shop	Green Intelligent Power Saver	It's nice to save a dozen bucks a night on two air conditioners that used to take 30 or 40 degrees a night, but now it's only 10 or so degrees a night	Jingdong prefecture level city in Shandong
Fortune Shop	Green Intelligent Power Saver	Product no manufacturer's name no product qualification certificate, try two days to see the effect of	Jingdong prefecture level city in Shandong
Fortune Shop	Green Intelligent Power Saver	I've been using it for over a month and have saved over $80, so I've repurchased a few more back to save together	Jingdong prefecture level city in Shandong
Fortune Shop	Green Intelligent Power Saver	I haven't used it yet and will comment when I do, but it's well made.	Jingdong prefecture level city in Shandong
Fortune Shop	Green Intelligent Power Saver	It's quite energy efficient. It's really a boon for the home.	Jingdong prefecture level city in Shandong
Fortune Shop	Green Intelligent Power Saver	Energy-saving socket received a month, after using it for a month proved that it is indeed energy-saving, electricity bill saving, will buy it again, thank you!	Jingdong prefecture level city in Shandong
Fortune Shop	Green Intelligent Power Saver	There was a similar product many years ago. Now technology has evolved, buy one to try, according to the instructions in the box, it takes one or two dozen days to show results. Check your electric bill and see what happens.	Jingdong prefecture level city in Shandong
Fortune Shop	Green Intelligent Power Saver	Jingdong logistics is really fast, the same day the order arrived on the same day, more convenient than the supermarket, and a wide range of choices, the	Jingdong prefecture level city in Shandong
Fortune Shop	Green Intelligent Power Saver	I've been using this for a month or so and I can't see any results, it really is a scam as they say.	Jingdong prefecture level city in Shandong
Fortune Shop	Green Intelligent Power Saver	Very good use, a month can save a few degrees of power loss of electricity, introduced to the relatives to buy back to try.	Jingdong prefecture level city in Shandong
Fortune Shop	Green Intelligent Power Saver	It's really powerful! With this air conditioner can be turned on at any time without the pressure of paying the electricity bill! It's good for the people!	Jingdong prefecture level city in Shandong
Fortune Shop	Green Intelligent Power Saver	This business is a big ** products are not qualified, with a month of electricity more than one hundred percent of thirty, the purchase agreed to return the goods but do not receive a refund after receiving the goods, looking for the official side does not help, the Jingdong is a big **, I have never shopped in	Jingdong prefecture level city in Shandong
Fortune Shop	Green Intelligent Power Saver	It's good. I tried it. It saves power.	Jingdong prefecture level city in Shandong
Fortune Shop	Green Intelligent Power Saver	Nothing**, it seems like there's more	Jingdong prefecture level city in Shandong
Fortune Shop	Green Intelligent Power Saver	Power saving device received, well packaged, good quality, fast logistics	Jingdong prefecture level city in Shandong
Fortune Shop	Green Intelligent Power Saver	The service is okay, is that when I bought five, the desirable pieces are three, and then wait a few days and send me two replacement, I do not know if it is a mistake of the shipper, if I was not in the courier there to see, go home to say no, the	Jingdong prefecture level city in Shandong
Fortune Shop	Green Intelligent Power Saver	This thing will only cost me more if I use it, but before I used it, it was less than a ~ electricity a month, and after I used it, it was more than two hundred kWh a month.	Jingdong prefecture level city in Shandong
Fortune Shop	Green Intelligent Power Saver	I just used it. I don't know how it is.	Jingdong prefecture level city in Shandong
Fortune Shop	Green Intelligent Power Saver	I've received the product and I'm using it, so I'll see how it goes in a month!	Jingdong prefecture level city in Shandong
Fortune Shop	Green Intelligent Power Saver	The goods have just received the effect do not know how, please look forward to the results.	Jingdong prefecture level city in Shandong
Fortune Shop	Green Intelligent Power Saver	Power saving device, the material is very good practical and durable, the size is suitable, packaging is very good, the price is cheap, logistics is very fast!	Jingdong prefecture level city in Shandong
Fortune Shop	Green Intelligent Power Saver	Very nice, will comment in detail after using it for a while.	Jingdong prefecture level city in Shandong
Fortune Shop	Green Intelligent Power Saver	Good stuff to trust and support	Jingdong prefecture level city in Shandong
Fortune Shop	Green Intelligent Power Saver	I'll comment after I use it, doubt if it really saves power no	Jingdong prefecture level city in Shandong
Fortune Shop	Green Intelligent Power Saver	It's good quality and works well!	Jingdong prefecture level city in Shandong
Fortune Shop	Green Intelligent Power Saver	hahaha just right hahahahahaha hiccup hahahaha	Jingdong prefecture level city in Shandong
Fortune Shop	Green Intelligent Power Saver	The real thing matches the picture and looks great!	Jingdong prefecture level city in Shandong
Fortune Shop	Green Intelligent Power Saver	Plugged it in and we'll see when we pay the bills at the end of the year!	Jingdong prefecture level city in Shandong
Fortune Shop	Green Intelligent Power Saver	False advertising. It doesn't work.	Jingdong prefecture level city in Shandong
Fortune Shop	Green Intelligent Power Saver	Goods received, good effect	Jingdong prefecture level city in Shandong
Fortune Shop	Green Intelligent Power Saver	It looks okay, we'll see if it saves electricity when the time comes	Jingdong prefecture level city in Shandong
Fortune Shop	Green Intelligent Power Saver	Logistics is very fast quality is very good, the price is very affordable, accessories are also complete, installed, satisfied with good use	Jingdong prefecture level city in Shandong
Fortune Shop	Green Intelligent Power Saver	I've received the power saver. I'm looking forward to the savings.	Jingdong prefecture level city in Shandong
Fortune Shop	Green Intelligent Power Saver	Received, have not yet used, after use and then evaluate	Jingdong prefecture level city in Shandong
Fortune Shop	Green Intelligent Power Saver	I received the item, but I don't know if it saves power or not.	Jingdong prefecture level city in Shandong

Fortune Shop	Green Intelligent Power Saver	On Trial	Jingdong prefecture level city in Shandong
Fortune Shop	Green Intelligent Power Saver	Use it and see how it goes.	Jingdong prefecture level city in Shandong
Fortune Shop	Green Intelligent Power Saver	Quality is very good, no odor, fine workmanship, grain color are very like, satisfied!	Jingdong prefecture level city in Shandong
Fortune Shop	Green Intelligent Power Saver	I'll give you a good review. I don't know if it'll work.	Jingdong prefecture level city in Shandong
Fortune Shop	Green Intelligent Power Saver	The logistics is very fast and the package is intact. Good use of affordable. The effect is very good, is genuine	Jingdong prefecture level city in Shandong
Fortune Shop	Green Intelligent Power Saver	Nothing happens when you plug it in.	Jingdong prefecture level city in Shandong
Fortune Shop	Green Intelligent Power Saver	I don't know if it's as good as it sounds.	Jingdong prefecture level city in Shandong
Fortune Shop	Green Intelligent Power Saver	The quality of the stuff is very good, exactly the same as the seller's description, very satisfied!	Jingdong prefecture level city in Shandong
Fortune Shop	Green Intelligent Power Saver	The saver works great, it really works, it's affordable and worth buying.	Jingdong prefecture level city in Shandong
Fortune Shop	Green Intelligent Power Saver	Just used it for a couple days. I don't know yet.	Jingdong prefecture level city in Shandong
Fortune Shop	Green Intelligent Power Saver	The quality is quite good, cheaper than the physical store, good, next time will come again!	Jingdong prefecture level city in Shandong
Fortune Shop	Green Intelligent Power Saver	Good quality, cheap and affordable worth recommending to buy!	Jingdong prefecture level city in Shandong
Fortune Shop	Green Intelligent Power Saver	Very good quality, well made, works well! Satisfied	Jingdong prefecture level city in Shandong
Fortune Shop	Green Intelligent Power Saver	The logistics is very fast quality is very good very pleasant a shopping	Jingdong prefecture level city in Shandong
Fortune Shop	Green Intelligent Power Saver	Use it and then come back to evaluate!	Jingdong prefecture level city in Shandong
Fortune Shop	Green Intelligent Power Saver	# It's no use lying at all #	Jingdong prefecture level city in Shandong
Fortune Shop	Green Intelligent Power Saver	I can't get a voltage drop.	Jingdong prefecture level city in Shandong
Fortune Shop	Green Intelligent Power Saver	Evaluate after use	Jingdong prefecture level city in Shandong
Fortune Shop	Green Intelligent Power Saver	Still on trial.	Jingdong prefecture level city in Shandong
Fortune Shop	Green Intelligent Power Saver	ineffective	Jingdong prefecture level city in Shandong
Fortune Shop	Green Intelligent Power Saver	Satisfaction Shopping	Jingdong prefecture level city in Shandong
Fortune Shop	Green Intelligent Power Saver	Lies.	Jingdong prefecture level city in Shandong
Fortune Shop	Green Intelligent Power Saver	It's a real power saver.	Jingdong prefecture level city in Shandong
Fortune Shop	Green Intelligent Power Saver	in use	Jingdong prefecture level city in Shandong
Fortune Shop	Green Intelligent Power Saver	I don't know yet.	Jingdong prefecture level city in Shandong
Fortune Shop	Green Intelligent Power Saver	not care about anything	Jingdong prefecture level city in Shandong
Fortune Shop	Green Intelligent Power Saver	It doesn't seem to have much effect	Jingdong prefecture level city in Shandong
Fortune Shop	Green Intelligent Power Saver	Let the merchant see for himself, does the plug positive and negative terminals look like this?	Jingdong prefecture level city in Shandong
Fortune Shop	Green Intelligent Power Saver	It doesn't work well, the electricity bill is less before using it, but more after using it, contacting customer service, the service is still not good, and returning it is slow	Jingdong prefecture level city in Shandong
Fortune Shop	Green Intelligent Power Saver	With the advertisement product does not match, above the effective negative, power saving rate of two rows of words it, the product looks very thin, with 3 days to see the meter number is still with the previous no difference, which is obviously fraudulent behavior!	Jingdong prefecture level city in Shandong
Fortune Shop	Green Intelligent Power Saver	Doesn't save any power at all. Not much use.	Jingdong prefecture level city in Shandong
Fortune Shop	Green Intelligent Power Saver	The plugs are all loose, they're all the same length and they don't work at all	Jingdong prefecture level city in Shandong
Fortune Shop	Green Intelligent Power Saver	It's bad 👎 it's not effective at all, you'd be crazy to sell this garbage on the internet.	Jingdong prefecture level city in Shandong
Fortune Shop	Green Intelligent Power Saver	It doesn't save any power at all, so I hope the old timers don't fall for this scam again	Jingdong prefecture level city in Shandong
Fortune Shop	Green Intelligent Power Saver	I've been using it for over a month now and haven't noticed any power savings.	Jingdong prefecture level city in Shandong
Fortune Shop	Green Intelligent Power Saver	It doesn't work at all. The electricity bill is higher when you use it.	Jingdong prefecture level city in Shandong
Fortune Shop	Green Intelligent Power Saver	It doesn't save any power, it's still as bad as ever.	Jingdong prefecture level city in Shandong
Fortune Shop	Green Intelligent Power Saver	Last night before leaving work to look at the table, and then plugged into the node device, this morning and then look at the table, than the original save a few degrees of electricity, very good to have a power-saving effect, easy to use, satisfied with the shopping praise.	Jingdong prefecture level city in Shandong
Fortune Shop	Green Intelligent Power Saver	The product is real and effective, the electricity bill is reduced, really good, thanks to the merchants have so many good products, the product is real and effective, the electricity bill is reduced, want to save electricity friends quickly buy it, early use early to save money.	Jingdong prefecture level city in Shandong
Fortune Shop	Green Intelligent Power Saver	Saver saves time and money, baby appearance is beautiful, the quality of goods is good, and the same as the business description, good service attitude, packaging is complete, logistics is very fast, praise to	Jingdong prefecture level city in Shandong
Fortune Shop	Green Intelligent Power Saver	The effect is good, summer, home electricity is relatively large, buy and use a few days, feel the effect is very good, originally ten dollars a day electricity bill, now eight dollars a day, quite good	Jingdong prefecture level city in Shandong
Fortune Shop	Green Intelligent Power Saver	Indeed can save electricity and money, the first time I know this or from a friend's home to see, but also do not believe it, and now they use it to know that there is this good thing.	Jingdong prefecture level city in Shandong
Fortune Shop	Green Intelligent Power Saver	I have received the Smart Power Saver. Perfectly packaged. Fast delivery. Boss customer service attitude is also very good. It's worth buying. I plugged it in and tried it and it really saves more power than the original. I'll buy it again next time.	Jingdong prefecture level city in Shandong
Fortune Shop	Green Intelligent Power Saver	It's nice to save a dozen bucks a night on two air conditioners that used to take 30 or 40 degrees a night, but now it's only 10 or so degrees a night	Jingdong prefecture level city in Shandong
Fortune Shop	Green Intelligent Power Saver	Product no manufacturer's name no product qualification certificate, try two days to see the effect of	Jingdong prefecture level city in Shandong
Fortune Shop	Green Intelligent Power Saver	I've been using it for over a month and have saved over $80, so I've repurchased a few more back to save together	Jingdong prefecture level city in Shandong
Fortune Shop	Green Intelligent Power Saver	I haven't used it yet and will comment when I do, but it's well made.	Jingdong prefecture level city in Shandong
Fortune Shop	Green Intelligent Power Saver	It's quite energy efficient. It's really a boon for the home.	Jingdong prefecture level city in Shandong
Fortune Shop	Green Intelligent Power Saver	Energy-saving socket received a month, after using it for a month proved that it is indeed energy-saving, electricity bill saving, will buy it again, thank you!	Jingdong prefecture level city in Shandong

Fortune Shop	Green Intelligent Power Saver	There was a similar product many years ago. Now technology has evolved, buy one to try, according to the instructions in the box, it takes one or two dozen days to show results. Check your electric bill and see what happens.	Jingdong prefecture level city in Shandong
Fortune Shop	Green Intelligent Power Saver	Jingdong logistics is really fast, the same day the order arrived on the same day, more convenient than the supermarket, and a wide range of choices, the	Jingdong prefecture level city in Shandong
Fortune Shop	Green Intelligent Power Saver	I've been using this for a month or so and I can't see any results, it really is a scam as they say.	Jingdong prefecture level city in Shandong
Fortune Shop	Green Intelligent Power Saver	Very good use, a month can save a few degrees of power loss of electricity, introduced to the relatives to buy back to try.	Jingdong prefecture level city in Shandong
Fortune Shop	Green Intelligent Power Saver	It's really powerful! With this air conditioner can be turned on at any time without the pressure of paying the electricity bill! It's good for the people!	Jingdong prefecture level city in Shandong
Fortune Shop	Green Intelligent Power Saver	This business is a big ** products are not qualified, with a month of electricity more than one hundred percent of thirty, the purchase agreed to return the goods but do not receive a refund after receiving the goods, looking for the official side does not help, the Jingdong is a big **, I have never shopped in	Jingdong prefecture level city in Shandong
Fortune Shop	Green Intelligent Power Saver	It's good. I tried it. It saves power.	Jingdong prefecture level city in Shandong
Fortune Shop	Green Intelligent Power Saver	Nothing**, it seems like there's more	Jingdong prefecture level city in Shandong
Fortune Shop	Green Intelligent Power Saver	Power saving device received, well packaged, good quality, fast logistics	Jingdong prefecture level city in Shandong
Fortune Shop	Green Intelligent Power Saver	The service is okay, is that when I bought five, the desirable pieces are three, and then wait a few days and send me two replacement, I do not know if it is a mistake of the shipper, if I was not in the courier there to see, go home to say no, the	Jingdong prefecture level city in Shandong
Fortune Shop	Green Intelligent Power Saver	This thing will only cost me more if I use it, but before I used it, it was less than a ~ electricity a month, and after I used it, it was more than two hundred kWh a month.	Jingdong prefecture level city in Shandong
Fortune Shop	Green Intelligent Power Saver	I just used it. I don't know how it is.	Jingdong prefecture level city in Shandong
Fortune Shop	Green Intelligent Power Saver	I've received the product and I'm using it, so I'll see how it goes in a month!	Jingdong prefecture level city in Shandong
Fortune Shop	Green Intelligent Power Saver	The goods have just received the effect do not know how, please look forward to the results.	Jingdong prefecture level city in Shandong
Fortune Shop	Green Intelligent Power Saver	Power saving device, the material is very good practical and durable, the size is suitable, packaging is very good, the price is cheap, logistics is very fast!	Jingdong prefecture level city in Shandong
Fortune Shop	Green Intelligent Power Saver	Very nice, will comment in detail after using it for a while.	Jingdong prefecture level city in Shandong
Fortune Shop	Green Intelligent Power Saver	Good stuff to trust and support	Jingdong prefecture level city in Shandong
Fortune Shop	Green Intelligent Power Saver	I'll comment after I use it, doubt if it really saves power no	Jingdong prefecture level city in Shandong
Fortune Shop	Green Intelligent Power Saver	It's good quality and works well!	Jingdong prefecture level city in Shandong
Fortune Shop	Green Intelligent Power Saver	hahaha just right hahahahahaha hiccup hahahaha	Jingdong prefecture level city in Shandong
Fortune Shop	Green Intelligent Power Saver	The real thing matches the picture and looks great!	Jingdong prefecture level city in Shandong
Fortune Shop	Green Intelligent Power Saver	Plugged it in and we'll see when we pay the bills at the end of the year!	Jingdong prefecture level city in Shandong
Fortune Shop	Green Intelligent Power Saver	False advertising. It doesn't work.	Jingdong prefecture level city in Shandong
Fortune Shop	Green Intelligent Power Saver	Goods received, good effect	Jingdong prefecture level city in Shandong
Fortune Shop	Green Intelligent Power Saver	It looks okay, we'll see if it saves electricity when the time comes	Jingdong prefecture level city in Shandong
Fortune Shop	Green Intelligent Power Saver	Logistics is very fast quality is very good, the price is very affordable, accessories are also complete, installed, satisfied with good use	Jingdong prefecture level city in Shandong
Fortune Shop	Green Intelligent Power Saver	I've received the power saver. I'm looking forward to the savings.	Jingdong prefecture level city in Shandong
Fortune Shop	Green Intelligent Power Saver	Received, have not yet used, after use and then evaluate	Jingdong prefecture level city in Shandong
Fortune Shop	Green Intelligent Power Saver	I received the item, but I don't know if it saves power or not.	Jingdong prefecture level city in Shandong
Fortune Shop	Green Intelligent Power Saver	On Trial	Jingdong prefecture level city in Shandong
Fortune Shop	Green Intelligent Power Saver	Use it and see how it goes.	Jingdong prefecture level city in Shandong
Fortune Shop	Green Intelligent Power Saver	Quality is very good, no odor, fine workmanship, grain color are very like, satisfied!	Jingdong prefecture level city in Shandong
Fortune Shop	Green Intelligent Power Saver	I'll give you a good review. I don't know if it'll work.	Jingdong prefecture level city in Shandong
Fortune Shop	Green Intelligent Power Saver	The logistics is very fast and the package is intact. Good use of affordable. The effect is very good, is genuine	Jingdong prefecture level city in Shandong
Fortune Shop	Green Intelligent Power Saver	Nothing happens when you plug it in.	Jingdong prefecture level city in Shandong
Fortune Shop	Green Intelligent Power Saver	I don't know if it's as good as it sounds.	Jingdong prefecture level city in Shandong
Fortune Shop	Green Intelligent Power Saver	The quality of the stuff is very good, exactly the same as the seller's description, very satisfied!	Jingdong prefecture level city in Shandong
Fortune Shop	Green Intelligent Power Saver	The saver works great, it really works, it's affordable and worth buying.	Jingdong prefecture level city in Shandong
Fortune Shop	Green Intelligent Power Saver	Just used it for a couple days. I don't know yet.	Jingdong prefecture level city in Shandong
Fortune Shop	Green Intelligent Power Saver	The quality is quite good, cheaper than the physical store, good, next time will come again!	Jingdong prefecture level city in Shandong
Fortune Shop	Green Intelligent Power Saver	Good quality, cheap and affordable worth recommending to buy!	Jingdong prefecture level city in Shandong
Fortune Shop	Green Intelligent Power Saver	Very good quality, well made, works well! Satisfied	Jingdong prefecture level city in Shandong
Fortune Shop	Green Intelligent Power Saver	The logistics is very fast quality is very good very pleasant a shopping	Jingdong prefecture level city in Shandong
Fortune Shop	Green Intelligent Power Saver	Use it and then come back to evaluate!	Jingdong prefecture level city in Shandong
Fortune Shop	Green Intelligent Power Saver	# It's no use lying at all #	Jingdong prefecture level city in Shandong
Fortune Shop	Green Intelligent Power Saver	I can't get a voltage drop.	Jingdong prefecture level city in Shandong
Fortune Shop	Green Intelligent Power Saver	Evaluate after use	Jingdong prefecture level city in Shandong
Fortune Shop	Green Intelligent Power Saver	Still on trial.	Jingdong prefecture level city in Shandong
Fortune Shop	Green Intelligent Power Saver	ineffective	Jingdong prefecture level city in Shandong
Fortune Shop	Green Intelligent Power Saver	Satisfaction Shopping	Jingdong prefecture level city in Shandong

Fortune Shop	Green Intelligent Power Saver	Lies.	Jingdong prefecture level city in Shandong
Fortune Shop	Green Intelligent Power Saver	It's a real power saver.	Jingdong prefecture level city in Shandong
Fortune Shop	Green Intelligent Power Saver	in use	Jingdong prefecture level city in Shandong
Fortune Shop	Green Intelligent Power Saver	I don't know yet.	Jingdong prefecture level city in Shandong
Fortune Shop	Green Intelligent Power Saver	not care about anything	Jingdong prefecture level city in Shandong
Fortune Shop	Green Intelligent Power Saver	It doesn't seem to have much effect	Jingdong prefecture level city in Shandong
Fortune Shop	Green Intelligent Power Saver	Let the merchant see for himself, does the plug positive and negative terminals look like this?	Jingdong prefecture level city in Shandong
Fortune Shop	Green Intelligent Power Saver	It doesn't work well, the electricity bill is less before using it, but more after using it, contacting customer service, the service is still not good, and returning it is slow	Jingdong prefecture level city in Shandong
Fortune Shop	Green Intelligent Power Saver	With the advertisement product does not match, above the effective negative, power saving rate of two rows of words it, the product looks very thin, with 3 days to see the meter number is still with the previous no difference, which is obviously fraudulent behavior!	Jingdong prefecture level city in Shandong
Fortune Shop	Green Intelligent Power Saver	Doesn't save any power at all. Not much use.	Jingdong prefecture level city in Shandong
Fortune Shop	Green Intelligent Power Saver	The plugs are all loose, they're all the same length and they don't work at all	Jingdong prefecture level city in Shandong
Fortune Shop	Green Intelligent Power Saver	It's bad 👎 it's not effective at all, you'd be crazy to sell this garbage on the internet.	Jingdong prefecture level city in Shandong
Fortune Shop	Green Intelligent Power Saver	It doesn't save any power at all, so I hope the old timers don't fall for this scam again	Jingdong prefecture level city in Shandong
Fortune Shop	Green Intelligent Power Saver	I've been using it for over a month now and haven't noticed any power savings.	Jingdong prefecture level city in Shandong
Fortune Shop	Green Intelligent Power Saver	It doesn't work at all. The electricity bill is higher when you use it.	Jingdong prefecture level city in Shandong
Fortune Shop	Green Intelligent Power Saver	It doesn't save any power, it's still as bad as ever.	Jingdong prefecture level city in Shandong
Fortune Shop	Green Intelligent Power Saver	Last night before leaving work to look at the table, and then plugged into the node device, this morning and then look at the table, than the original save a few degrees of electricity, very good to have a power-saving effect, easy to use, satisfied with the shopping praise.	Jingdong prefecture level city in Shandong
Fortune Shop	Green Intelligent Power Saver	The product is real and effective, the electricity bill is reduced, really good, thanks to the merchants have so many good products, the product is real and effective, the electricity bill is reduced, want to save electricity friends quickly buy it, early use early to save money.	Jingdong prefecture level city in Shandong
Fortune Shop	Green Intelligent Power Saver	Saver saves time and money, baby appearance is beautiful, the quality of goods is good, and the same as the business description, good service attitude, packaging is complete, logistics is very fast, praise to	Jingdong prefecture level city in Shandong
Fortune Shop	Green Intelligent Power Saver	The effect is good, summer, home electricity is relatively large, buy and use a few days, feel the effect is very good, originally ten dollars a day electricity bill, now eight dollars a day, quite good	Jingdong prefecture level city in Shandong
Fortune Shop	Green Intelligent Power Saver	Indeed can save electricity and money, the first time I know this or from a friend's home to see, but also do not believe it, and now they use it to know that there is this good thing.	Jingdong prefecture level city in Shandong
Fortune Shop	Green Intelligent Power Saver	I have received the Smart Power Saver. Perfectly packaged. Fast delivery. Boss customer service attitude is also very good. It's worth buying. I plugged it in and tried it and it really saves more power than the original. I'll buy it again next time.	Jingdong prefecture level city in Shandong
Fortune Shop	Green Intelligent Power Saver	It's nice to save a dozen bucks a night on two air conditioners that used to take 30 or 40 degrees a night, but now it's only 10 or so degrees a night	Jingdong prefecture level city in Shandong
Fortune Shop	Green Intelligent Power Saver	Product no manufacturer's name no product qualification certificate, try two days to see the effect of	Jingdong prefecture level city in Shandong
Fortune Shop	Green Intelligent Power Saver	I've been using it for over a month and have saved over $80, so I've repurchased a few more back to save together	Jingdong prefecture level city in Shandong
Fortune Shop	Green Intelligent Power Saver	I haven't used it yet and will comment when I do, but it's well made.	Jingdong prefecture level city in Shandong
Fortune Shop	Green Intelligent Power Saver	It's quite energy efficient. It's really a boon for the home.	Jingdong prefecture level city in Shandong
Fortune Shop	Green Intelligent Power Saver	Energy-saving socket received a month, after using it for a month proved that it is indeed energy-saving, electricity bill saving, will buy it again, thank you!	Jingdong prefecture level city in Shandong
Fortune Shop	Green Intelligent Power Saver	There was a similar product many years ago. Now technology has evolved, buy one to try, according to the instructions in the box, it takes one or two dozen days to show results. Check your electric bill and see what happens.	Jingdong prefecture level city in Shandong
Fortune Shop	Green Intelligent Power Saver	Jingdong logistics is really fast, the same day the order arrived on the same day, more convenient than the supermarket, and a wide range of choices, the	Jingdong prefecture level city in Shandong
Fortune Shop	Green Intelligent Power Saver	I've been using this for a month or so and I can't see any results, it really is a scam as they say.	Jingdong prefecture level city in Shandong
Fortune Shop	Green Intelligent Power Saver	Very good use, a month can save a few degrees of power loss of electricity, introduced to the relatives to buy back to try.	Jingdong prefecture level city in Shandong
Fortune Shop	Green Intelligent Power Saver	It's really powerful! With this air conditioner can be turned on at any time without the pressure of paying the electricity bill! It's good for the people!	Jingdong prefecture level city in Shandong
Fortune Shop	Green Intelligent Power Saver	This business is a big ** products are not qualified, with a month of electricity more than one hundred percent of thirty, the purchase agreed to return the goods but do not receive a refund after receiving the goods, looking for the official side does not help, the Jingdong is a big **, I have never shopped in	Jingdong prefecture level city in Shandong
Fortune Shop	Green Intelligent Power Saver	It's good. I tried it. It saves power.	Jingdong prefecture level city in Shandong
Fortune Shop	Green Intelligent Power Saver	Nothing**, it seems like there's more	Jingdong prefecture level city in Shandong
Fortune Shop	Green Intelligent Power Saver	Power saving device received, well packaged, good quality, fast logistics	Jingdong prefecture level city in Shandong
Fortune Shop	Green Intelligent Power Saver	The service is okay, is that when I bought five, the desirable pieces are three, and then wait a few days and send me two replacement, I do not know if it is a mistake of the shipper, if I was not in the courier there to see, go home to say no, the	Jingdong prefecture level city in Shandong
Fortune Shop	Green Intelligent Power Saver	This thing will only cost me more if I use it, but before I used it, it was less than a ~ electricity a month, and after I used it, it was more than two hundred kWh a month.	Jingdong prefecture level city in Shandong
Fortune Shop	Green Intelligent Power Saver	I just used it. I don't know how it is.	Jingdong prefecture level city in Shandong
Fortune Shop	Green Intelligent Power Saver	I've received the product and I'm using it, so I'll see how it goes in a month!	Jingdong prefecture level city in Shandong
Fortune Shop	Green Intelligent Power Saver	The goods have just received the effect do not know how, please look forward to the results.	Jingdong prefecture level city in Shandong
Fortune Shop	Green Intelligent Power Saver	Power saving device, the material is very good practical and durable, the size is suitable, packaging is very good, the price is cheap, logistics is very fast!	Jingdong prefecture level city in Shandong
Fortune Shop	Green Intelligent Power Saver	Very nice, will comment in detail after using it for a while.	Jingdong prefecture level city in Shandong
Fortune Shop	Green Intelligent Power Saver	Good stuff to trust and support	Jingdong prefecture level city in Shandong
Fortune Shop	Green Intelligent Power Saver	I'll comment after I use it, doubt if it really saves power no	Jingdong prefecture level city in Shandong
Fortune Shop	Green Intelligent Power Saver	It's good quality and works well!	Jingdong prefecture level city in Shandong
Fortune Shop	Green Intelligent Power Saver	hahaha just right hahahahahaha hiccup hahahaha	Jingdong prefecture level city in Shandong

Fortune Shop	Green Intelligent Power Saver	The real thing matches the picture and looks great!	Jingdong prefecture level city in Shandong
Fortune Shop	Green Intelligent Power Saver	Plugged it in and we'll see when we pay the bills at the end of the year!	Jingdong prefecture level city in Shandong
Fortune Shop	Green Intelligent Power Saver	False advertising. It doesn't work.	Jingdong prefecture level city in Shandong
Fortune Shop	Green Intelligent Power Saver	Goods received, good effect	Jingdong prefecture level city in Shandong
Fortune Shop	Green Intelligent Power Saver	It looks okay, we'll see if it saves electricity when the time comes	Jingdong prefecture level city in Shandong
Fortune Shop	Green Intelligent Power Saver	Logistics is very fast quality is very good, the price is very affordable, accessories are also complete, installed, satisfied with good use	Jingdong prefecture level city in Shandong
Fortune Shop	Green Intelligent Power Saver	I've received the power saver. I'm looking forward to the savings.	Jingdong prefecture level city in Shandong
Fortune Shop	Green Intelligent Power Saver	Received, have not yet used, after use and then evaluate	Jingdong prefecture level city in Shandong
Fortune Shop	Green Intelligent Power Saver	I received the item, but I don't know if it saves power or not.	Jingdong prefecture level city in Shandong
Fortune Shop	Green Intelligent Power Saver	On Trial	Jingdong prefecture level city in Shandong
Fortune Shop	Green Intelligent Power Saver	Use it and see how it goes.	Jingdong prefecture level city in Shandong
Fortune Shop	Green Intelligent Power Saver	Quality is very good, no odor, fine workmanship, grain color are very like, satisfied!	Jingdong prefecture level city in Shandong
Fortune Shop	Green Intelligent Power Saver	I'll give you a good review. I don't know if it'll work.	Jingdong prefecture level city in Shandong
Fortune Shop	Green Intelligent Power Saver	The logistics is very fast and the package is intact. Good use of affordable. The effect is very good, is genuine	Jingdong prefecture level city in Shandong
Fortune Shop	Green Intelligent Power Saver	Nothing happens when you plug it in.	Jingdong prefecture level city in Shandong
Fortune Shop	Green Intelligent Power Saver	I don't know if it's as good as it sounds.	Jingdong prefecture level city in Shandong
Fortune Shop	Green Intelligent Power Saver	The quality of the stuff is very good, exactly the same as the seller's description, very satisfied!	Jingdong prefecture level city in Shandong
Fortune Shop	Green Intelligent Power Saver	The saver works great, it really works, it's affordable and worth buying.	Jingdong prefecture level city in Shandong
Fortune Shop	Green Intelligent Power Saver	Just used it for a couple days. I don't know yet.	Jingdong prefecture level city in Shandong
Fortune Shop	Green Intelligent Power Saver	The quality is quite good, cheaper than the physical store, good, next time will come again!	Jingdong prefecture level city in Shandong
Fortune Shop	Green Intelligent Power Saver	Good quality, cheap and affordable worth recommending to buy!	Jingdong prefecture level city in Shandong
Fortune Shop	Green Intelligent Power Saver	Very good quality, well made, works well! Satisfied	Jingdong prefecture level city in Shandong
Fortune Shop	Green Intelligent Power Saver	The logistics is very fast quality is very good very pleasant a shopping	Jingdong prefecture level city in Shandong
Fortune Shop	Green Intelligent Power Saver	Use it and then come back to evaluate!	Jingdong prefecture level city in Shandong
Fortune Shop	Green Intelligent Power Saver	# It's no use lying at all #	Jingdong prefecture level city in Shandong
Fortune Shop	Green Intelligent Power Saver	I can't get a voltage drop.	Jingdong prefecture level city in Shandong
Fortune Shop	Green Intelligent Power Saver	Evaluate after use	Jingdong prefecture level city in Shandong
Fortune Shop	Green Intelligent Power Saver	Still on trial.	Jingdong prefecture level city in Shandong
Fortune Shop	Green Intelligent Power Saver	ineffective	Jingdong prefecture level city in Shandong
Fortune Shop	Green Intelligent Power Saver	Satisfaction Shopping	Jingdong prefecture level city in Shandong
Fortune Shop	Green Intelligent Power Saver	Lies.	Jingdong prefecture level city in Shandong
Fortune Shop	Green Intelligent Power Saver	It's a real power saver.	Jingdong prefecture level city in Shandong
Fortune Shop	Green Intelligent Power Saver	in use	Jingdong prefecture level city in Shandong
Fortune Shop	Green Intelligent Power Saver	I don't know yet.	Jingdong prefecture level city in Shandong
Fortune Shop	Green Intelligent Power Saver	not care about anything	Jingdong prefecture level city in Shandong
Fortune Shop	Green Intelligent Power Saver	It doesn't seem to have much effect	Jingdong prefecture level city in Shandong
Fortune Shop	Green Intelligent Power Saver	Let the merchant see for himself, does the plug positive and negative terminals look like this?	Jingdong prefecture level city in Shandong
Fortune Shop	Green Intelligent Power Saver	It doesn't work well, the electricity bill is less before using it, but more after using it, contacting customer service, the service is still not good, and returning it is slow	Jingdong prefecture level city in Shandong
Fortune Shop	Green Intelligent Power Saver	With the advertisement product does not match, above the effective negative, power saving rate of two rows of words it, the product looks very thin, with 3 days to see the meter number is still with the previous no difference, which is obviously fraudulent behavior!	Jingdong prefecture level city in Shandong
Fortune Shop	Green Intelligent Power Saver	Doesn't save any power at all. Not much use.	Jingdong prefecture level city in Shandong
Fortune Shop	Green Intelligent Power Saver	The plugs are all loose, they're all the same length and they don't work at all	Jingdong prefecture level city in Shandong
Fortune Shop	Green Intelligent Power Saver	It's bad 👎 it's not effective at all, you'd be crazy to sell this garbage on the internet.	Jingdong prefecture level city in Shandong
Fortune Shop	Green Intelligent Power Saver	It doesn't save any power at all, so I hope the old timers don't fall for this scam again	Jingdong prefecture level city in Shandong
Fortune Shop	Green Intelligent Power Saver	I've been using it for over a month now and haven't noticed any power savings.	Jingdong prefecture level city in Shandong
Fortune Shop	Green Intelligent Power Saver	It doesn't work at all. The electricity bill is higher when you use it.	Jingdong prefecture level city in Shandong
Fortune Shop	Green Intelligent Power Saver	It doesn't save any power, it's still as bad as ever.	Jingdong prefecture level city in Shandong
Fortune Shop	Green Intelligent Power Saver	Last night before leaving work to look at the table, and then plugged into the node device, this morning and then look at the table, than the original save a few degrees of electricity, very good to have a power-saving effect, easy to use, satisfied with the shopping praise.	Jingdong prefecture level city in Shandong
Fortune Shop	Green Intelligent Power Saver	The product is real and effective, the electricity bill is reduced, really good, thanks to the merchants have so many good products, the product is real and effective, the electricity bill is reduced, want to save electricity friends quickly buy it, early use early to save money.	Jingdong prefecture level city in Shandong
Fortune Shop	Green Intelligent Power Saver	Saver saves time and money, baby appearance is beautiful, the quality of goods is good, and the same as the business description, good service attitude, packaging is complete, logistics is very fast, praise to	Jingdong prefecture level city in Shandong
Fortune Shop	Green Intelligent Power Saver	The effect is good, summer, home electricity is relatively large, buy and use a few days, feel the effect is very good, originally ten dollars a day electricity bill, now eight dollars a day, quite good	Jingdong prefecture level city in Shandong

Fortune Shop	Green Intelligent Power Saver	Indeed can save electricity and money, the first time I know this or from a friend's home to see, but also do not believe it, and now they use it to know that there is this good thing.	Jingdong prefecture level city in Shandong
Fortune Shop	Green Intelligent Power Saver	I have received the Smart Power Saver. Perfectly packaged. Fast delivery. Boss customer service attitude is also very good. It's worth buying. I plugged it in and tried it and it really saves more power than the original. I'll buy it again next time.	Jingdong prefecture level city in Shandong
Fortune Shop	Green Intelligent Power Saver	It's nice to save a dozen bucks a night on two air conditioners that used to take 30 or 40 degrees a night, but now it's only 10 or so degrees a night	Jingdong prefecture level city in Shandong
Fortune Shop	Green Intelligent Power Saver	Product no manufacturer's name no product qualification certificate, try two days to see the effect of	Jingdong prefecture level city in Shandong
Fortune Shop	Green Intelligent Power Saver	I've been using it for over a month and have saved over $80, so I've repurchased a few more back to save together	Jingdong prefecture level city in Shandong
Fortune Shop	Green Intelligent Power Saver	I haven't used it yet and will comment when I do, but it's well made.	Jingdong prefecture level city in Shandong
Fortune Shop	Green Intelligent Power Saver	It's quite energy efficient. It's really a boon for the home.	Jingdong prefecture level city in Shandong
Fortune Shop	Green Intelligent Power Saver	Energy-saving socket received a month, after using it for a month proved that it is indeed energy-saving, electricity bill saving, will buy it again, thank you!	Jingdong prefecture level city in Shandong
Fortune Shop	Green Intelligent Power Saver	There was a similar product many years ago. Now technology has evolved, buy one to try, according to the instructions in the box, it takes one or two dozen days to show results. Check your electric bill and see what happens.	Jingdong prefecture level city in Shandong
Fortune Shop	Green Intelligent Power Saver	Jingdong logistics is really fast, the same day the order arrived on the same day, more convenient than the supermarket, and a wide range of choices, the	Jingdong prefecture level city in Shandong
Fortune Shop	Green Intelligent Power Saver	I've been using this for a month or so and I can't see any results, it really is a scam as they say.	Jingdong prefecture level city in Shandong
Fortune Shop	Green Intelligent Power Saver	Very good use, a month can save a few degrees of power loss of electricity, introduced to the relatives to buy back to try.	Jingdong prefecture level city in Shandong
Fortune Shop	Green Intelligent Power Saver	It's really powerful! With this air conditioner can be turned on at any time without the pressure of paying the electricity bill! It's good for the people!	Jingdong prefecture level city in Shandong
Fortune Shop	Green Intelligent Power Saver	This business is a big ** products are not qualified, with a month of electricity more than one hundred percent of thirty, the purchase agreed to return the goods but do not receive a refund after receiving the goods, looking for the official side does not help, the Jingdong is a big **, I have never shopped in	Jingdong prefecture level city in Shandong
Fortune Shop	Green Intelligent Power Saver	It's good. I tried it. It saves power.	Jingdong prefecture level city in Shandong
Fortune Shop	Green Intelligent Power Saver	Nothing**, it seems like there's more	Jingdong prefecture level city in Shandong
Fortune Shop	Green Intelligent Power Saver	Power saving device received, well packaged, good quality, fast logistics	Jingdong prefecture level city in Shandong
Fortune Shop	Green Intelligent Power Saver	The service is okay, is that when I bought five, the desirable pieces are three, and then wait a few days and send me two replacement, I do not know if it is a mistake of the shipper, if I was not in the courier there to see, go home to say no, the	Jingdong prefecture level city in Shandong
Fortune Shop	Green Intelligent Power Saver	This thing will only cost me more if I use it, but before I used it, it was less than a ~ electricity a month, and after I used it, it was more than two hundred kWh a month.	Jingdong prefecture level city in Shandong
Fortune Shop	Green Intelligent Power Saver	I just used it. I don't know how it is.	Jingdong prefecture level city in Shandong
Fortune Shop	Green Intelligent Power Saver	I've received the product and I'm using it, so I'll see how it goes in a month!	Jingdong prefecture level city in Shandong
Fortune Shop	Green Intelligent Power Saver	The goods have just received the effect do not know how, please look forward to the results.	Jingdong prefecture level city in Shandong
Fortune Shop	Green Intelligent Power Saver	Power saving device, the material is very good practical and durable, the size is suitable, packaging is very good, the price is cheap, logistics is very fast!	Jingdong prefecture level city in Shandong
Fortune Shop	Green Intelligent Power Saver	Very nice, will comment in detail after using it for a while.	Jingdong prefecture level city in Shandong
Fortune Shop	Green Intelligent Power Saver	Good stuff to trust and support	Jingdong prefecture level city in Shandong
Fortune Shop	Green Intelligent Power Saver	I'll comment after I use it, doubt if it really saves power no	Jingdong prefecture level city in Shandong
Fortune Shop	Green Intelligent Power Saver	It's good quality and works well!	Jingdong prefecture level city in Shandong
Fortune Shop	Green Intelligent Power Saver	hahaha just right hahahahahaha hiccup hahahaha	Jingdong prefecture level city in Shandong
Fortune Shop	Green Intelligent Power Saver	The real thing matches the picture and looks great!	Jingdong prefecture level city in Shandong
Fortune Shop	Green Intelligent Power Saver	Plugged it in and we'll see when we pay the bills at the end of the year!	Jingdong prefecture level city in Shandong
Fortune Shop	Green Intelligent Power Saver	False advertising. It doesn't work.	Jingdong prefecture level city in Shandong
Fortune Shop	Green Intelligent Power Saver	Goods received, good effect	Jingdong prefecture level city in Shandong
Fortune Shop	Green Intelligent Power Saver	It looks okay, we'll see if it saves electricity when the time comes	Jingdong prefecture level city in Shandong
Fortune Shop	Green Intelligent Power Saver	Logistics is very fast quality is very good, the price is very affordable, accessories are also complete, installed, satisfied with good use	Jingdong prefecture level city in Shandong
Fortune Shop	Green Intelligent Power Saver	I've received the power saver. I'm looking forward to the savings.	Jingdong prefecture level city in Shandong
Fortune Shop	Green Intelligent Power Saver	Received, have not yet used, after use and then evaluate	Jingdong prefecture level city in Shandong
Fortune Shop	Green Intelligent Power Saver	I received the item, but I don't know if it saves power or not.	Jingdong prefecture level city in Shandong
Fortune Shop	Green Intelligent Power Saver	On Trial	Jingdong prefecture level city in Shandong
Fortune Shop	Green Intelligent Power Saver	Use it and see how it goes.	Jingdong prefecture level city in Shandong
Fortune Shop	Green Intelligent Power Saver	Quality is very good, no odor, fine workmanship, grain color are very like, satisfied!	Jingdong prefecture level city in Shandong
Fortune Shop	Green Intelligent Power Saver	I'll give you a good review. I don't know if it'll work.	Jingdong prefecture level city in Shandong
Fortune Shop	Green Intelligent Power Saver	The logistics is very fast and the package is intact. Good use of affordable. The effect is very good, is genuine	Jingdong prefecture level city in Shandong
Fortune Shop	Green Intelligent Power Saver	Nothing happens when you plug it in.	Jingdong prefecture level city in Shandong
Fortune Shop	Green Intelligent Power Saver	I don't know if it's as good as it sounds.	Jingdong prefecture level city in Shandong
Fortune Shop	Green Intelligent Power Saver	The quality of the stuff is very good, exactly the same as the seller's description, very satisfied!	Jingdong prefecture level city in Shandong
Fortune Shop	Green Intelligent Power Saver	The saver works great, it really works, it's affordable and worth buying.	Jingdong prefecture level city in Shandong
Fortune Shop	Green Intelligent Power Saver	Just used it for a couple days. I don't know yet.	Jingdong prefecture level city in Shandong
Fortune Shop	Green Intelligent Power Saver	The quality is quite good, cheaper than the physical store, good, next time will come again!	Jingdong prefecture level city in Shandong
Fortune Shop	Green Intelligent Power Saver	Good quality, cheap and affordable worth recommending to buy!	Jingdong prefecture level city in Shandong

Fortune Shop	Green Intelligent Power Saver	Very good quality, well made, works well! Satisfied	Jingdong prefecture level city in Shandong
Fortune Shop	Green Intelligent Power Saver	The logistics is very fast quality is very good very pleasant a shopping	Jingdong prefecture level city in Shandong
Fortune Shop	Green Intelligent Power Saver	Use it and then come back to evaluate!	Jingdong prefecture level city in Shandong
Fortune Shop	Green Intelligent Power Saver	# It's no use lying at all #	Jingdong prefecture level city in Shandong
Fortune Shop	Green Intelligent Power Saver	I can't get a voltage drop.	Jingdong prefecture level city in Shandong
Fortune Shop	Green Intelligent Power Saver	Evaluate after use	Jingdong prefecture level city in Shandong
Fortune Shop	Green Intelligent Power Saver	Still on trial.	Jingdong prefecture level city in Shandong
Fortune Shop	Green Intelligent Power Saver	ineffective	Jingdong prefecture level city in Shandong
Fortune Shop	Green Intelligent Power Saver	Satisfaction Shopping	Jingdong prefecture level city in Shandong
Fortune Shop	Green Intelligent Power Saver	Lies.	Jingdong prefecture level city in Shandong
Fortune Shop	Green Intelligent Power Saver	It's a real power saver.	Jingdong prefecture level city in Shandong
Fortune Shop	Green Intelligent Power Saver	in use	Jingdong prefecture level city in Shandong
Fortune Shop	Green Intelligent Power Saver	I don't know yet.	Jingdong prefecture level city in Shandong
Fortune Shop	Green Intelligent Power Saver	not care about anything	Jingdong prefecture level city in Shandong
Fortune Shop	Green Intelligent Power Saver	It doesn't seem to have much effect	Jingdong prefecture level city in Shandong
Fortune Shop	Green Intelligent Power Saver	Let the merchant see for himself, does the plug positive and negative terminals look like this?	Jingdong prefecture level city in Shandong
Fortune Shop	Green Intelligent Power Saver	It doesn't work well, the electricity bill is less before using it, but more after using it, contacting customer service, the service is still not good, and returning it is slow	Jingdong prefecture level city in Shandong
Fortune Shop	Green Intelligent Power Saver	With the advertisement product does not match, above the effective negative, power saving rate of two rows of words it, the product looks very thin, with 3 days to see the meter number is still with the previous no difference, which is obviously fraudulent behavior!	Jingdong prefecture level city in Shandong
Fortune Shop	Green Intelligent Power Saver	Doesn't save any power at all. Not much use.	Jingdong prefecture level city in Shandong
Fortune Shop	Green Intelligent Power Saver	The plugs are all loose, they're all the same length and they don't work at all	Jingdong prefecture level city in Shandong
Fortune Shop	Green Intelligent Power Saver	It's bad 👎 it's not effective at all, you'd be crazy to sell this garbage on the internet.	Jingdong prefecture level city in Shandong
Fortune Shop	Green Intelligent Power Saver	It doesn't save any power at all, so I hope the old timers don't fall for this scam again	Jingdong prefecture level city in Shandong
Fortune Shop	Green Intelligent Power Saver	I've been using it for over a month now and haven't noticed any power savings.	Jingdong prefecture level city in Shandong
Fortune Shop	Green Intelligent Power Saver	It doesn't work at all. The electricity bill is higher when you use it.	Jingdong prefecture level city in Shandong
Fortune Shop	Green Intelligent Power Saver	It doesn't save any power, it's still as bad as ever.	Jingdong prefecture level city in Shandong
Fortune Shop	Green Intelligent Power Saver	Last night before leaving work to look at the table, and then plugged into the node device, this morning and then look at the table, than the original save a few degrees of electricity, very good to have a power-saving effect, easy to use, satisfied with the shopping praise.	Jingdong prefecture level city in Shandong
Fortune Shop	Green Intelligent Power Saver	The product is real and effective, the electricity bill is reduced, really good, thanks to the merchants have so many good products, the product is real and effective, the electricity bill is reduced, want to save electricity friends quickly buy it, early use early to save money.	Jingdong prefecture level city in Shandong
Fortune Shop	Green Intelligent Power Saver	Saver saves time and money, baby appearance is beautiful, the quality of goods is good, and the same as the business description, good service attitude, packaging is complete, logistics is very fast, praise to	Jingdong prefecture level city in Shandong
Fortune Shop	Green Intelligent Power Saver	The effect is good, summer, home electricity is relatively large, buy and use a few days, feel the effect is very good, originally ten dollars a day electricity bill, now eight dollars a day, quite good	Jingdong prefecture level city in Shandong
Fortune Shop	Green Intelligent Power Saver	Indeed can save electricity and money, the first time I know this or from a friend's home to see, but also do not believe it, and now they use it to know that there is this good thing.	Jingdong prefecture level city in Shandong
Fortune Shop	Green Intelligent Power Saver	I have received the Smart Power Saver. Perfectly packaged. Fast delivery. Boss customer service attitude is also very good. It's worth buying. I plugged it in and tried it and it really saves more power than the original. I'll buy it again next time.	Jingdong prefecture level city in Shandong
Fortune Shop	Green Intelligent Power Saver	It's nice to save a dozen bucks a night on two air conditioners that used to take 30 or 40 degrees a night, but now it's only 10 or so degrees a night	Jingdong prefecture level city in Shandong
Fortune Shop	Green Intelligent Power Saver	Product no manufacturer's name no product qualification certificate, try two days to see the effect of	Jingdong prefecture level city in Shandong
Fortune Shop	Green Intelligent Power Saver	I've been using it for over a month and have saved over $80, so I've repurchased a few more back to save together	Jingdong prefecture level city in Shandong
Fortune Shop	Green Intelligent Power Saver	I haven't used it yet and will comment when I do, but it's well made.	Jingdong prefecture level city in Shandong
Fortune Shop	Green Intelligent Power Saver	It's quite energy efficient. It's really a boon for the home.	Jingdong prefecture level city in Shandong
Fortune Shop	Green Intelligent Power Saver	Energy-saving socket received a month, after using it for a month proved that it is indeed energy-saving, electricity bill saving, will buy it again, thank you!	Jingdong prefecture level city in Shandong
Fortune Shop	Green Intelligent Power Saver	There was a similar product many years ago. Now technology has evolved, buy one to try, according to the instructions in the box, it takes one or two dozen days to show results. Check your electric bill and see what happens.	Jingdong prefecture level city in Shandong
Fortune Shop	Green Intelligent Power Saver	Jingdong logistics is really fast, the same day the order arrived on the same day, more convenient than the supermarket, and a wide range of choices, the	Jingdong prefecture level city in Shandong
Fortune Shop	Green Intelligent Power Saver	I've been using this for a month or so and I can't see any results, it really is a scam as they say.	Jingdong prefecture level city in Shandong
Fortune Shop	Green Intelligent Power Saver	Very good use, a month can save a few degrees of power loss of electricity, introduced to the relatives to buy back to try.	Jingdong prefecture level city in Shandong
Fortune Shop	Green Intelligent Power Saver	It's really powerful! With this air conditioner can be turned on at any time without the pressure of paying the electricity bill! It's good for the people!	Jingdong prefecture level city in Shandong
Fortune Shop	Green Intelligent Power Saver	This business is a big ** products are not qualified, with a month of electricity more than one hundred percent of thirty, the purchase agreed to return the goods but do not receive a refund after receiving the goods, looking for the official side does not help, the Jingdong is a big **, I have never shopped in	Jingdong prefecture level city in Shandong
Fortune Shop	Green Intelligent Power Saver	It's good. I tried it. It saves power.	Jingdong prefecture level city in Shandong
Fortune Shop	Green Intelligent Power Saver	Nothing**, it seems like there's more	Jingdong prefecture level city in Shandong
Fortune Shop	Green Intelligent Power Saver	Power saving device received, well packaged, good quality, fast logistics	Jingdong prefecture level city in Shandong
Fortune Shop	Green Intelligent Power Saver	The service is okay, is that when I bought five, the desirable pieces are three, and then wait a few days and send me two replacement, I do not know if it is a mistake of the shipper, if I was not in the courier there to see, go home to say no, the	Jingdong prefecture level city in Shandong
Fortune Shop	Green Intelligent Power Saver	This thing will only cost me more if I use it, but before I used it, it was less than a ~ electricity a month, and after I used it, it was more than two hundred kWh a month.	Jingdong prefecture level city in Shandong

Fortune Shop	Green Intelligent Power Saver	I just used it. I don't know how it is.	Jingdong prefecture level city in Shandong
Fortune Shop	Green Intelligent Power Saver	I've received the product and I'm using it, so I'll see how it goes in a month!	Jingdong prefecture level city in Shandong
Fortune Shop	Green Intelligent Power Saver	The goods have just received the effect do not know how, please look forward to the results.	Jingdong prefecture level city in Shandong
Fortune Shop	Green Intelligent Power Saver	Power saving device, the material is very good practical and durable, the size is suitable, packaging is very good, the price is cheap, logistics is very fast!	Jingdong prefecture level city in Shandong
Fortune Shop	Green Intelligent Power Saver	Very nice, will comment in detail after using it for a while.	Jingdong prefecture level city in Shandong
Fortune Shop	Green Intelligent Power Saver	Good stuff to trust and support	Jingdong prefecture level city in Shandong
Fortune Shop	Green Intelligent Power Saver	I'll comment after I use it, doubt if it really saves power no	Jingdong prefecture level city in Shandong
Fortune Shop	Green Intelligent Power Saver	It's good quality and works well!	Jingdong prefecture level city in Shandong
Fortune Shop	Green Intelligent Power Saver	hahaha just right hahahahahaha hiccup hahahaha	Jingdong prefecture level city in Shandong
Fortune Shop	Green Intelligent Power Saver	The real thing matches the picture and looks great!	Jingdong prefecture level city in Shandong
Fortune Shop	Green Intelligent Power Saver	Plugged it in and we'll see when we pay the bills at the end of the year!	Jingdong prefecture level city in Shandong
Fortune Shop	Green Intelligent Power Saver	False advertising. It doesn't work.	Jingdong prefecture level city in Shandong
Fortune Shop	Green Intelligent Power Saver	Goods received, good effect	Jingdong prefecture level city in Shandong
Fortune Shop	Green Intelligent Power Saver	It looks okay, we'll see if it saves electricity when the time comes	Jingdong prefecture level city in Shandong
Fortune Shop	Green Intelligent Power Saver	Logistics is very fast quality is very good, the price is very affordable, accessories are also complete, installed, satisfied with good use	Jingdong prefecture level city in Shandong
Fortune Shop	Green Intelligent Power Saver	I've received the power saver. I'm looking forward to the savings.	Jingdong prefecture level city in Shandong
Fortune Shop	Green Intelligent Power Saver	Received, have not yet used, after use and then evaluate	Jingdong prefecture level city in Shandong
Fortune Shop	Green Intelligent Power Saver	I received the item, but I don't know if it saves power or not.	Jingdong prefecture level city in Shandong
Fortune Shop	Green Intelligent Power Saver	On Trial	Jingdong prefecture level city in Shandong
Fortune Shop	Green Intelligent Power Saver	Use it and see how it goes.	Jingdong prefecture level city in Shandong
Fortune Shop	Green Intelligent Power Saver	Quality is very good, no odor, fine workmanship, grain color are very like, satisfied!	Jingdong prefecture level city in Shandong
Fortune Shop	Green Intelligent Power Saver	I'll give you a good review. I don't know if it'll work.	Jingdong prefecture level city in Shandong
Fortune Shop	Green Intelligent Power Saver	The logistics is very fast and the package is intact. Good use of affordable. The effect is very good, is genuine	Jingdong prefecture level city in Shandong
Fortune Shop	Green Intelligent Power Saver	Nothing happens when you plug it in.	Jingdong prefecture level city in Shandong
Fortune Shop	Green Intelligent Power Saver	I don't know if it's as good as it sounds.	Jingdong prefecture level city in Shandong
Fortune Shop	Green Intelligent Power Saver	The quality of the stuff is very good, exactly the same as the seller's description, very satisfied!	Jingdong prefecture level city in Shandong
Fortune Shop	Green Intelligent Power Saver	The saver works great, it really works, it's affordable and worth buying.	Jingdong prefecture level city in Shandong
Fortune Shop	Green Intelligent Power Saver	Just used it for a couple days. I don't know yet.	Jingdong prefecture level city in Shandong
Fortune Shop	Green Intelligent Power Saver	The quality is quite good, cheaper than the physical store, good, next time will come again!	Jingdong prefecture level city in Shandong
Fortune Shop	Green Intelligent Power Saver	Good quality, cheap and affordable worth recommending to buy!	Jingdong prefecture level city in Shandong
Fortune Shop	Green Intelligent Power Saver	Very good quality, well made, works well! Satisfied	Jingdong prefecture level city in Shandong
Fortune Shop	Green Intelligent Power Saver	The logistics is very fast quality is very good very pleasant a shopping	Jingdong prefecture level city in Shandong
Fortune Shop	Green Intelligent Power Saver	Use it and then come back to evaluate!	Jingdong prefecture level city in Shandong
Fortune Shop	Green Intelligent Power Saver	# It's no use lying at all #	Jingdong prefecture level city in Shandong
Fortune Shop	Green Intelligent Power Saver	I can't get a voltage drop.	Jingdong prefecture level city in Shandong
Fortune Shop	Green Intelligent Power Saver	Evaluate after use	Jingdong prefecture level city in Shandong
Fortune Shop	Green Intelligent Power Saver	Still on trial.	Jingdong prefecture level city in Shandong
Fortune Shop	Green Intelligent Power Saver	ineffective	Jingdong prefecture level city in Shandong
Fortune Shop	Green Intelligent Power Saver	Satisfaction Shopping	Jingdong prefecture level city in Shandong
Fortune Shop	Green Intelligent Power Saver	Lies.	Jingdong prefecture level city in Shandong
Fortune Shop	Green Intelligent Power Saver	It's a real power saver.	Jingdong prefecture level city in Shandong
Fortune Shop	Green Intelligent Power Saver	in use	Jingdong prefecture level city in Shandong
Fortune Shop	Green Intelligent Power Saver	I don't know yet.	Jingdong prefecture level city in Shandong
Fortune Shop	Green Intelligent Power Saver	not care about anything	Jingdong prefecture level city in Shandong
Fortune Shop	Green Intelligent Power Saver	It doesn't seem to have much effect	Jingdong prefecture level city in Shandong
Fortune Shop	Green Intelligent Power Saver	Let the merchant see for himself, does the plug positive and negative terminals look like this?	Jingdong prefecture level city in Shandong
Fortune Shop	Green Intelligent Power Saver	It doesn't work well, the electricity bill is less before using it, but more after using it, contacting customer service, the service is still not good, and returning it is slow	Jingdong prefecture level city in Shandong
Fortune Shop	Green Intelligent Power Saver	With the advertisement product does not match, above the effective negative, power saving rate of two rows of words it, the product looks very thin, with 3 days to see the meter number is still with the previous no difference, which is obviously fraudulent behavior!	Jingdong prefecture level city in Shandong
Fortune Shop	Green Intelligent Power Saver	Doesn't save any power at all. Not much use.	Jingdong prefecture level city in Shandong
Fortune Shop	Green Intelligent Power Saver	The plugs are all loose, they're all the same length and they don't work at all	Jingdong prefecture level city in Shandong
Fortune Shop	Green Intelligent Power Saver	It's bad 👎 it's not effective at all, you'd be crazy to sell this garbage on the internet.	Jingdong prefecture level city in Shandong

Fortune Shop	Green Intelligent Power Saver	It doesn't save any power at all, so I hope the old timers don't fall for this scam again	Jingdong prefecture level city in Shandong
Fortune Shop	Green Intelligent Power Saver	I've been using it for over a month now and haven't noticed any power savings.	Jingdong prefecture level city in Shandong
Fortune Shop	Green Intelligent Power Saver	It doesn't work at all. The electricity bill is higher when you use it.	Jingdong prefecture level city in Shandong
Fortune Shop	Green Intelligent Power Saver	It doesn't save any power, it's still as bad as ever.	Jingdong prefecture level city in Shandong
Fortune Shop	Green Intelligent Power Saver	Last night before leaving work to look at the table, and then plugged into the node device, this morning and then look at the table, than the original save a few degrees of electricity, very good to have a power-saving effect, easy to use, satisfied with the shopping praise.	Jingdong prefecture level city in Shandong
Fortune Shop	Green Intelligent Power Saver	The product is real and effective, the electricity bill is reduced, really good, thanks to the merchants have so many good products, the product is real and effective, the electricity bill is reduced, want to save electricity friends quickly buy it, early use early to save money.	Jingdong prefecture level city in Shandong
Fortune Shop	Green Intelligent Power Saver	Saver saves time and money, baby appearance is beautiful, the quality of goods is good, and the same as the business description, good service attitude, packaging is complete, logistics is very fast, praise to	Jingdong prefecture level city in Shandong
Fortune Shop	Green Intelligent Power Saver	The effect is good, summer, home electricity is relatively large, buy and use a few days, feel the effect is very good, originally ten dollars a day electricity bill, now eight dollars a day, quite good	Jingdong prefecture level city in Shandong
Fortune Shop	Green Intelligent Power Saver	Indeed can save electricity and money, the first time I know this or from a friend's home to see, but also do not believe it, and now they use it to know that there is this good thing.	Jingdong prefecture level city in Shandong
Fortune Shop	Green Intelligent Power Saver	I have received the Smart Power Saver. Perfectly packaged. Fast delivery. Boss customer service attitude is also very good. It's worth buying. I plugged it in and tried it and it really saves more power than the original. I'll buy it again next time.	Jingdong prefecture level city in Shandong
Fortune Shop	Green Intelligent Power Saver	It's nice to save a dozen bucks a night on two air conditioners that used to take 30 or 40 degrees a night, but now it's only 10 or so degrees a night	Jingdong prefecture level city in Shandong
Fortune Shop	Green Intelligent Power Saver	Product no manufacturer's name no product qualification certificate, try two days to see the effect of	Jingdong prefecture level city in Shandong
Fortune Shop	Green Intelligent Power Saver	I've been using it for over a month and have saved over $80, so I've repurchased a few more back to save together	Jingdong prefecture level city in Shandong
Fortune Shop	Green Intelligent Power Saver	I haven't used it yet and will comment when I do, but it's well made.	Jingdong prefecture level city in Shandong
Fortune Shop	Green Intelligent Power Saver	It's quite energy efficient. It's really a boon for the home.	Jingdong prefecture level city in Shandong
Fortune Shop	Green Intelligent Power Saver	Energy-saving socket received a month, after using it for a month proved that it is indeed energy-saving, electricity bill saving, will buy it again, thank you!	Jingdong prefecture level city in Shandong
Fortune Shop	Green Intelligent Power Saver	There was a similar product many years ago. Now technology has evolved, buy one to try, according to the instructions in the box, it takes one or two dozen days to show results. Check your electric bill and see what happens.	Jingdong prefecture level city in Shandong
Fortune Shop	Green Intelligent Power Saver	Jingdong logistics is really fast, the same day the order arrived on the same day, more convenient than the supermarket, and a wide range of choices, the	Jingdong prefecture level city in Shandong
Fortune Shop	Green Intelligent Power Saver	I've been using this for a month or so and I can't see any results, it really is a scam as they say.	Jingdong prefecture level city in Shandong
Fortune Shop	Green Intelligent Power Saver	Very good use, a month can save a few degrees of power loss of electricity, introduced to the relatives to buy back to try.	Jingdong prefecture level city in Shandong
Fortune Shop	Green Intelligent Power Saver	It's really powerful! With this air conditioner can be turned on at any time without the pressure of paying the electricity bill! It's good for the people!	Jingdong prefecture level city in Shandong
Fortune Shop	Green Intelligent Power Saver	This business is a big ** products are not qualified, with a month of electricity more than one hundred percent of thirty, the purchase agreed to return the goods but do not receive a refund after receiving the goods, looking for the official side does not help, the Jingdong is a big **, I have never shopped in	Jingdong prefecture level city in Shandong
Fortune Shop	Green Intelligent Power Saver	It's good. I tried it. It saves power.	Jingdong prefecture level city in Shandong
Fortune Shop	Green Intelligent Power Saver	Nothing**, it seems like there's more	Jingdong prefecture level city in Shandong
Fortune Shop	Green Intelligent Power Saver	Power saving device received, well packaged, good quality, fast logistics	Jingdong prefecture level city in Shandong
Fortune Shop	Green Intelligent Power Saver	The service is okay, is that when I bought five, the desirable pieces are three, and then wait a few days and send me two replacement, I do not know if it is a mistake of the shipper, if I was not in the courier there to see, go home to say no, the	Jingdong prefecture level city in Shandong
Fortune Shop	Green Intelligent Power Saver	This thing will only cost me more if I use it, but before I used it, it was less than a ~ electricity a month, and after I used it, it was more than two hundred kWh a month.	Jingdong prefecture level city in Shandong
Fortune Shop	Green Intelligent Power Saver	I just used it. I don't know how it is.	Jingdong prefecture level city in Shandong
Fortune Shop	Green Intelligent Power Saver	I've received the product and I'm using it, so I'll see how it goes in a month!	Jingdong prefecture level city in Shandong
Fortune Shop	Green Intelligent Power Saver	The goods have just received the effect do not know how, please look forward to the results.	Jingdong prefecture level city in Shandong
Fortune Shop	Green Intelligent Power Saver	Power saving device, the material is very good practical and durable, the size is suitable, packaging is very good, the price is cheap, logistics is very fast!	Jingdong prefecture level city in Shandong
Fortune Shop	Green Intelligent Power Saver	Very nice, will comment in detail after using it for a while.	Jingdong prefecture level city in Shandong
Fortune Shop	Green Intelligent Power Saver	Good stuff to trust and support	Jingdong prefecture level city in Shandong
Fortune Shop	Green Intelligent Power Saver	I'll comment after I use it, doubt if it really saves power no	Jingdong prefecture level city in Shandong
Fortune Shop	Green Intelligent Power Saver	It's good quality and works well!	Jingdong prefecture level city in Shandong
Fortune Shop	Green Intelligent Power Saver	hahaha just right hahahahahaha hiccup hahahaha	Jingdong prefecture level city in Shandong
Fortune Shop	Green Intelligent Power Saver	The real thing matches the picture and looks great!	Jingdong prefecture level city in Shandong
Fortune Shop	Green Intelligent Power Saver	Plugged it in and we'll see when we pay the bills at the end of the year!	Jingdong prefecture level city in Shandong
Fortune Shop	Green Intelligent Power Saver	False advertising. It doesn't work.	Jingdong prefecture level city in Shandong
Fortune Shop	Green Intelligent Power Saver	Goods received, good effect	Jingdong prefecture level city in Shandong
Fortune Shop	Green Intelligent Power Saver	It looks okay, we'll see if it saves electricity when the time comes	Jingdong prefecture level city in Shandong
Fortune Shop	Green Intelligent Power Saver	Logistics is very fast quality is very good, the price is very affordable, accessories are also complete, installed, satisfied with good use	Jingdong prefecture level city in Shandong
Fortune Shop	Green Intelligent Power Saver	I've received the power saver. I'm looking forward to the savings.	Jingdong prefecture level city in Shandong
Fortune Shop	Green Intelligent Power Saver	Received, have not yet used, after use and then evaluate	Jingdong prefecture level city in Shandong
Fortune Shop	Green Intelligent Power Saver	I received the item, but I don't know if it saves power or not.	Jingdong prefecture level city in Shandong
Fortune Shop	Green Intelligent Power Saver	On Trial	Jingdong prefecture level city in Shandong
Fortune Shop	Green Intelligent Power Saver	Use it and see how it goes.	Jingdong prefecture level city in Shandong
Fortune Shop	Green Intelligent Power Saver	Quality is very good, no odor, fine workmanship, grain color are very like, satisfied!	Jingdong prefecture level city in Shandong

Fortune Shop	Green Intelligent Power Saver	I'll give you a good review. I don't know if it'll work.	Jingdong prefecture level city in Shandong
Fortune Shop	Green Intelligent Power Saver	The logistics is very fast and the package is intact. Good use of affordable. The effect is very good, is genuine	Jingdong prefecture level city in Shandong
Fortune Shop	Green Intelligent Power Saver	Nothing happens when you plug it in.	Jingdong prefecture level city in Shandong
Fortune Shop	Green Intelligent Power Saver	I don't know if it's as good as it sounds.	Jingdong prefecture level city in Shandong
Fortune Shop	Green Intelligent Power Saver	The quality of the stuff is very good, exactly the same as the seller's description, very satisfied!	Jingdong prefecture level city in Shandong
Fortune Shop	Green Intelligent Power Saver	The saver works great, it really works, it's affordable and worth buying.	Jingdong prefecture level city in Shandong
Fortune Shop	Green Intelligent Power Saver	Just used it for a couple days. I don't know yet.	Jingdong prefecture level city in Shandong
Fortune Shop	Green Intelligent Power Saver	The quality is quite good, cheaper than the physical store, good, next time will come again!	Jingdong prefecture level city in Shandong
Fortune Shop	Green Intelligent Power Saver	Good quality, cheap and affordable worth recommending to buy!	Jingdong prefecture level city in Shandong
Fortune Shop	Green Intelligent Power Saver	Very good quality, well made, works well! Satisfied	Jingdong prefecture level city in Shandong
Fortune Shop	Green Intelligent Power Saver	The logistics is very fast quality is very good very pleasant a shopping	Jingdong prefecture level city in Shandong
Fortune Shop	Green Intelligent Power Saver	Use it and then come back to evaluate!	Jingdong prefecture level city in Shandong
Fortune Shop	Green Intelligent Power Saver	# It's no use lying at all #	Jingdong prefecture level city in Shandong
Fortune Shop	Green Intelligent Power Saver	I can't get a voltage drop.	Jingdong prefecture level city in Shandong
Fortune Shop	Green Intelligent Power Saver	Evaluate after use	Jingdong prefecture level city in Shandong
Fortune Shop	Green Intelligent Power Saver	Still on trial.	Jingdong prefecture level city in Shandong
Fortune Shop	Green Intelligent Power Saver	ineffective	Jingdong prefecture level city in Shandong
Fortune Shop	Green Intelligent Power Saver	Satisfaction Shopping	Jingdong prefecture level city in Shandong
Fortune Shop	Green Intelligent Power Saver	Lies.	Jingdong prefecture level city in Shandong
Fortune Shop	Green Intelligent Power Saver	It's a real power saver.	Jingdong prefecture level city in Shandong
Fortune Shop	Green Intelligent Power Saver	in use	Jingdong prefecture level city in Shandong
Fortune Shop	Green Intelligent Power Saver	I don't know yet.	Jingdong prefecture level city in Shandong
Fortune Shop	Green Intelligent Power Saver	not care about anything	Jingdong prefecture level city in Shandong
Fortune Shop	Green Intelligent Power Saver	It doesn't seem to have much effect	Jingdong prefecture level city in Shandong
Fortune Shop	Green Intelligent Power Saver	Let the merchant see for himself, does the plug positive and negative terminals look like this?	Jingdong prefecture level city in Shandong
Fortune Shop	Green Intelligent Power Saver	It doesn't work well, the electricity bill is less before using it, but more after using it, contacting customer service, the service is still not good, and returning it is slow	Jingdong prefecture level city in Shandong
Fortune Shop	Green Intelligent Power Saver	With the advertisement product does not match, above the effective negative, power saving rate of two rows of words it, the product looks very thin, with 3 days to see the meter number is still with the previous no difference, which is obviously fraudulent behavior!	Jingdong prefecture level city in Shandong
Fortune Shop	Green Intelligent Power Saver	Doesn't save any power at all. Not much use.	Jingdong prefecture level city in Shandong
Fortune Shop	Green Intelligent Power Saver	The plugs are all loose, they're all the same length and they don't work at all	Jingdong prefecture level city in Shandong
Fortune Shop	Green Intelligent Power Saver	It's bad 👎 it's not effective at all, you'd be crazy to sell this garbage on the internet.	Jingdong prefecture level city in Shandong
Fortune Shop	Green Intelligent Power Saver	It doesn't save any power at all, so I hope the old timers don't fall for this scam again	Jingdong prefecture level city in Shandong
Fortune Shop	Green Intelligent Power Saver	I've been using it for over a month now and haven't noticed any power savings.	Jingdong prefecture level city in Shandong
Fortune Shop	Green Intelligent Power Saver	It doesn't work at all. The electricity bill is higher when you use it.	Jingdong prefecture level city in Shandong
Fortune Shop	Green Intelligent Power Saver	It doesn't save any power, it's still as bad as ever.	Jingdong prefecture level city in Shandong
Fortune Shop	Green Intelligent Power Saver	Last night before leaving work to look at the table, and then plugged into the node device, this morning and then look at the table, than the original save a few degrees of electricity, very good to have a power-saving effect, easy to use, satisfied with the shopping praise.	Jingdong prefecture level city in Shandong
Fortune Shop	Green Intelligent Power Saver	The product is real and effective, the electricity bill is reduced, really good, thanks to the merchants have so many good products, the product is real and effective, the electricity bill is reduced, want to save electricity friends quickly buy it, early use early to save money.	Jingdong prefecture level city in Shandong
Fortune Shop	Green Intelligent Power Saver	Saver saves time and money, baby appearance is beautiful, the quality of goods is good, and the same as the business description, good service attitude, packaging is complete, logistics is very fast, praise to	Jingdong prefecture level city in Shandong
Fortune Shop	Green Intelligent Power Saver	The effect is good, summer, home electricity is relatively large, buy and use a few days, feel the effect is very good, originally ten dollars a day electricity bill, now eight dollars a day, quite good	Jingdong prefecture level city in Shandong
Fortune Shop	Green Intelligent Power Saver	Indeed can save electricity and money, the first time I know this or from a friend's home to see, but also do not believe it, and now they use it to know that there is this good thing.	Jingdong prefecture level city in Shandong
Fortune Shop	Green Intelligent Power Saver	I have received the Smart Power Saver. Perfectly packaged. Fast delivery. Boss customer service attitude is also very good. It's worth buying. I plugged it in and tried it and it really saves more power than the original. I'll buy it again next time.	Jingdong prefecture level city in Shandong
Fortune Shop	Green Intelligent Power Saver	It's nice to save a dozen bucks a night on two air conditioners that used to take 30 or 40 degrees a night, but now it's only 10 or so degrees a night	Jingdong prefecture level city in Shandong
Fortune Shop	Green Intelligent Power Saver	Product no manufacturer's name no product qualification certificate, try two days to see the effect of	Jingdong prefecture level city in Shandong
Fortune Shop	Green Intelligent Power Saver	I've been using it for over a month and have saved over $80, so I've repurchased a few more back to save together	Jingdong prefecture level city in Shandong
Fortune Shop	Green Intelligent Power Saver	I haven't used it yet and will comment when I do, but it's well made.	Jingdong prefecture level city in Shandong
Fortune Shop	Green Intelligent Power Saver	It's quite energy efficient. It's really a boon for the home.	Jingdong prefecture level city in Shandong
Fortune Shop	Green Intelligent Power Saver	Energy-saving socket received a month, after using it for a month proved that it is indeed energy-saving, electricity bill saving, will buy it again, thank you!	Jingdong prefecture level city in Shandong
Fortune Shop	Green Intelligent Power Saver	There was a similar product many years ago. Now technology has evolved, buy one to try, according to the instructions in the box, it takes one or two dozen days to show results. Check your electric bill and see what happens.	Jingdong prefecture level city in Shandong
Fortune Shop	Green Intelligent Power Saver	Jingdong logistics is really fast, the same day the order arrived on the same day, more convenient than the supermarket, and a wide range of choices, the	Jingdong prefecture level city in Shandong
Fortune Shop	Green Intelligent Power Saver	I've been using this for a month or so and I can't see any results, it really is a scam as they say.	Jingdong prefecture level city in Shandong

Fortune Shop	Green Intelligent Power Saver	Very good use, a month can save a few degrees of power loss of electricity, introduced to the relatives to buy back to try.	Jingdong prefecture level city in Shandong
Fortune Shop	Green Intelligent Power Saver	It's really powerful! With this air conditioner can be turned on at any time without the pressure of paying the electricity bill! It's good for the people!	Jingdong prefecture level city in Shandong
Fortune Shop	Green Intelligent Power Saver	This business is a big ** products are not qualified, with a month of electricity more than one hundred percent of thirty, the purchase agreed to return the goods but do not receive a refund after receiving the goods, looking for the official side does not help, the Jingdong is a big **, I have never shopped in	Jingdong prefecture level city in Shandong
Fortune Shop	Green Intelligent Power Saver	It's good. I tried it. It saves power.	Jingdong prefecture level city in Shandong
Fortune Shop	Green Intelligent Power Saver	Nothing**, it seems like there's more	Jingdong prefecture level city in Shandong
Fortune Shop	Green Intelligent Power Saver	Power saving device received, well packaged, good quality, fast logistics	Jingdong prefecture level city in Shandong
Fortune Shop	Green Intelligent Power Saver	The service is okay, is that when I bought five, the desirable pieces are three, and then wait a few days and send me two replacement, I do not know if it is a mistake of the shipper, if I was not in the courier there to see, go home to say no, the	Jingdong prefecture level city in Shandong
Fortune Shop	Green Intelligent Power Saver	This thing will only cost me more if I use it, but before I used it, it was less than a ~ electricity a month, and after I used it, it was more than two hundred kWh a month.	Jingdong prefecture level city in Shandong
Fortune Shop	Green Intelligent Power Saver	I just used it. I don't know how it is.	Jingdong prefecture level city in Shandong
Fortune Shop	Green Intelligent Power Saver	I've received the product and I'm using it, so I'll see how it goes in a month!	Jingdong prefecture level city in Shandong
Fortune Shop	Green Intelligent Power Saver	The goods have just received the effect do not know how, please look forward to the results.	Jingdong prefecture level city in Shandong
Fortune Shop	Green Intelligent Power Saver	Power saving device, the material is very good practical and durable, the size is suitable, packaging is very good, the price is cheap, logistics is very fast!	Jingdong prefecture level city in Shandong
Fortune Shop	Green Intelligent Power Saver	Very nice, will comment in detail after using it for a while.	Jingdong prefecture level city in Shandong
Fortune Shop	Green Intelligent Power Saver	Good stuff to trust and support	Jingdong prefecture level city in Shandong
Fortune Shop	Green Intelligent Power Saver	I'll comment after I use it, doubt if it really saves power no	Jingdong prefecture level city in Shandong
Fortune Shop	Green Intelligent Power Saver	It's good quality and works well!	Jingdong prefecture level city in Shandong
Fortune Shop	Green Intelligent Power Saver	hahaha just right hahahahahaha hiccup hahahaha	Jingdong prefecture level city in Shandong
Fortune Shop	Green Intelligent Power Saver	The real thing matches the picture and looks great!	Jingdong prefecture level city in Shandong
Fortune Shop	Green Intelligent Power Saver	Plugged it in and we'll see when we pay the bills at the end of the year!	Jingdong prefecture level city in Shandong
Fortune Shop	Green Intelligent Power Saver	False advertising. It doesn't work.	Jingdong prefecture level city in Shandong
Fortune Shop	Green Intelligent Power Saver	Goods received, good effect	Jingdong prefecture level city in Shandong
Fortune Shop	Green Intelligent Power Saver	It looks okay, we'll see if it saves electricity when the time comes	Jingdong prefecture level city in Shandong
Fortune Shop	Green Intelligent Power Saver	Logistics is very fast quality is very good, the price is very affordable, accessories are also complete, installed, satisfied with good use	Jingdong prefecture level city in Shandong
Fortune Shop	Green Intelligent Power Saver	I've received the power saver. I'm looking forward to the savings.	Jingdong prefecture level city in Shandong
Fortune Shop	Green Intelligent Power Saver	Received, have not yet used, after use and then evaluate	Jingdong prefecture level city in Shandong
Fortune Shop	Green Intelligent Power Saver	I received the item, but I don't know if it saves power or not.	Jingdong prefecture level city in Shandong
Fortune Shop	Green Intelligent Power Saver	On Trial	Jingdong prefecture level city in Shandong
Fortune Shop	Green Intelligent Power Saver	Use it and see how it goes.	Jingdong prefecture level city in Shandong
Fortune Shop	Green Intelligent Power Saver	Quality is very good, no odor, fine workmanship, grain color are very like, satisfied!	Jingdong prefecture level city in Shandong
Fortune Shop	Green Intelligent Power Saver	I'll give you a good review. I don't know if it'll work.	Jingdong prefecture level city in Shandong
Fortune Shop	Green Intelligent Power Saver	The logistics is very fast and the package is intact. Good use of affordable. The effect is very good, is genuine	Jingdong prefecture level city in Shandong
Fortune Shop	Green Intelligent Power Saver	Nothing happens when you plug it in.	Jingdong prefecture level city in Shandong
Fortune Shop	Green Intelligent Power Saver	I don't know if it's as good as it sounds.	Jingdong prefecture level city in Shandong
Fortune Shop	Green Intelligent Power Saver	The quality of the stuff is very good, exactly the same as the seller's description, very satisfied!	Jingdong prefecture level city in Shandong
Fortune Shop	Green Intelligent Power Saver	The saver works great, it really works, it's affordable and worth buying.	Jingdong prefecture level city in Shandong
Fortune Shop	Green Intelligent Power Saver	Just used it for a couple days. I don't know yet.	Jingdong prefecture level city in Shandong
Fortune Shop	Green Intelligent Power Saver	The quality is quite good, cheaper than the physical store, good, next time will come again!	Jingdong prefecture level city in Shandong
Fortune Shop	Green Intelligent Power Saver	Good quality, cheap and affordable worth recommending to buy!	Jingdong prefecture level city in Shandong
Fortune Shop	Green Intelligent Power Saver	Very good quality, well made, works well! Satisfied	Jingdong prefecture level city in Shandong
Fortune Shop	Green Intelligent Power Saver	The logistics is very fast quality is very good very pleasant a shopping	Jingdong prefecture level city in Shandong
Fortune Shop	Green Intelligent Power Saver	Use it and then come back to evaluate!	Jingdong prefecture level city in Shandong
Fortune Shop	Green Intelligent Power Saver	# It's no use lying at all #	Jingdong prefecture level city in Shandong
Fortune Shop	Green Intelligent Power Saver	I can't get a voltage drop.	Jingdong prefecture level city in Shandong
Fortune Shop	Green Intelligent Power Saver	Evaluate after use	Jingdong prefecture level city in Shandong
Fortune Shop	Green Intelligent Power Saver	Still on trial.	Jingdong prefecture level city in Shandong
Fortune Shop	Green Intelligent Power Saver	ineffective	Jingdong prefecture level city in Shandong
Fortune Shop	Green Intelligent Power Saver	Satisfaction Shopping	Jingdong prefecture level city in Shandong
Fortune Shop	Green Intelligent Power Saver	Lies.	Jingdong prefecture level city in Shandong
Fortune Shop	Green Intelligent Power Saver	It's a real power saver.	Jingdong prefecture level city in Shandong
Fortune Shop	Green Intelligent Power Saver	in use	Jingdong prefecture level city in Shandong

Fortune Shop	Green Intelligent Power Saver	I don't know yet.	Jingdong prefecture level city in Shandong
Fortune Shop	Green Intelligent Power Saver	not care about anything	Jingdong prefecture level city in Shandong
Fortune Shop	Green Intelligent Power Saver	It doesn't seem to have much effect	Jingdong prefecture level city in Shandong
Fortune Shop	Green Intelligent Power Saver	Let the merchant see for himself, does the plug positive and negative terminals look like this?	Jingdong prefecture level city in Shandong
Fortune Shop	Green Intelligent Power Saver	It doesn't work well, the electricity bill is less before using it, but more after using it, contacting customer service, the service is still not good, and returning it is slow	Jingdong prefecture level city in Shandong
Fortune Shop	Green Intelligent Power Saver	With the advertisement product does not match, above the effective negative, power saving rate of two rows of words it, the product looks very thin, with 3 days to see the meter number is still with the previous no difference, which is obviously fraudulent behavior!	Jingdong prefecture level city in Shandong
Fortune Shop	Green Intelligent Power Saver	Doesn't save any power at all. Not much use.	Jingdong prefecture level city in Shandong
Fortune Shop	Green Intelligent Power Saver	The plugs are all loose, they're all the same length and they don't work at all	Jingdong prefecture level city in Shandong
Fortune Shop	Green Intelligent Power Saver	It's bad 👎 it's not effective at all, you'd be crazy to sell this garbage on the internet.	Jingdong prefecture level city in Shandong
Fortune Shop	Green Intelligent Power Saver	It doesn't save any power at all, so I hope the old timers don't fall for this scam again	Jingdong prefecture level city in Shandong
Fortune Shop	Green Intelligent Power Saver	I've been using it for over a month now and haven't noticed any power savings.	Jingdong prefecture level city in Shandong
Fortune Shop	Green Intelligent Power Saver	It doesn't work at all. The electricity bill is higher when you use it.	Jingdong prefecture level city in Shandong
Fortune Shop	Green Intelligent Power Saver	It doesn't save any power, it's still as bad as ever.	Jingdong prefecture level city in Shandong
Fortune Shop	Green Intelligent Power Saver	Last night before leaving work to look at the table, and then plugged into the node device, this morning and then look at the table, than the original save a few degrees of electricity, very good to have a power-saving effect, easy to use, satisfied with the shopping praise.	Jingdong prefecture level city in Shandong
Fortune Shop	Green Intelligent Power Saver	The product is real and effective, the electricity bill is reduced, really good, thanks to the merchants have so many good products, the product is real and effective, the electricity bill is reduced, want to save electricity friends quickly buy it, early use early to save money.	Jingdong prefecture level city in Shandong
Fortune Shop	Green Intelligent Power Saver	Saver saves time and money, baby appearance is beautiful, the quality of goods is good, and the same as the business description, good service attitude, packaging is complete, logistics is very fast, praise to	Jingdong prefecture level city in Shandong
Fortune Shop	Green Intelligent Power Saver	The effect is good, summer, home electricity is relatively large, buy and use a few days, feel the effect is very good, originally ten dollars a day electricity bill, now eight dollars a day, quite good	Jingdong prefecture level city in Shandong
Fortune Shop	Green Intelligent Power Saver	Indeed can save electricity and money, the first time I know this or from a friend's home to see, but also do not believe it, and now they use it to know that there is this good thing.	Jingdong prefecture level city in Shandong
Fortune Shop	Green Intelligent Power Saver	I have received the Smart Power Saver. Perfectly packaged. Fast delivery. Boss customer service attitude is also very good. It's worth buying. I plugged it in and tried it and it really saves more power than the original. I'll buy it again next time.	Jingdong prefecture level city in Shandong
Fortune Shop	Green Intelligent Power Saver	It's nice to save a dozen bucks a night on two air conditioners that used to take 30 or 40 degrees a night, but now it's only 10 or so degrees a night	Jingdong prefecture level city in Shandong
Fortune Shop	Green Intelligent Power Saver	Product no manufacturer's name no product qualification certificate, try two days to see the effect of	Jingdong prefecture level city in Shandong
Fortune Shop	Green Intelligent Power Saver	I've been using it for over a month and have saved over $80, so I've repurchased a few more back to save together	Jingdong prefecture level city in Shandong
Fortune Shop	Green Intelligent Power Saver	I haven't used it yet and will comment when I do, but it's well made.	Jingdong prefecture level city in Shandong
Fortune Shop	Green Intelligent Power Saver	It's quite energy efficient. It's really a boon for the home.	Jingdong prefecture level city in Shandong
Fortune Shop	Green Intelligent Power Saver	Energy-saving socket received a month, after using it for a month proved that it is indeed energy-saving, electricity bill saving, will buy it again, thank you!	Jingdong prefecture level city in Shandong
Fortune Shop	Green Intelligent Power Saver	There was a similar product many years ago. Now technology has evolved, buy one to try, according to the instructions in the box, it takes one or two dozen days to show results. Check your electric bill and see what happens.	Jingdong prefecture level city in Shandong
Fortune Shop	Green Intelligent Power Saver	Jingdong logistics is really fast, the same day the order arrived on the same day, more convenient than the supermarket, and a wide range of choices, the	Jingdong prefecture level city in Shandong
Fortune Shop	Green Intelligent Power Saver	I've been using this for a month or so and I can't see any results, it really is a scam as they say.	Jingdong prefecture level city in Shandong
Fortune Shop	Green Intelligent Power Saver	Very good use, a month can save a few degrees of power loss of electricity, introduced to the relatives to buy back to try.	Jingdong prefecture level city in Shandong
Fortune Shop	Green Intelligent Power Saver	It's really powerful! With this air conditioner can be turned on at any time without the pressure of paying the electricity bill! It's good for the people!	Jingdong prefecture level city in Shandong
Fortune Shop	Green Intelligent Power Saver	This business is a big ** products are not qualified, with a month of electricity more than one hundred percent of thirty, the purchase agreed to return the goods but do not receive a refund after receiving the goods, looking for the official side does not help, the Jingdong is a big **, I have never shopped in	Jingdong prefecture level city in Shandong
Fortune Shop	Green Intelligent Power Saver	It's good. I tried it. It saves power.	Jingdong prefecture level city in Shandong
Fortune Shop	Green Intelligent Power Saver	Nothing**, it seems like there's more	Jingdong prefecture level city in Shandong
Fortune Shop	Green Intelligent Power Saver	Power saving device received, well packaged, good quality, fast logistics	Jingdong prefecture level city in Shandong
Fortune Shop	Green Intelligent Power Saver	The service is okay, is that when I bought five, the desirable pieces are three, and then wait a few days and send me two replacement, I do not know if it is a mistake of the shipper, if I was not in the courier there to see, go home to say no, the	Jingdong prefecture level city in Shandong
Fortune Shop	Green Intelligent Power Saver	This thing will only cost me more if I use it, but before I used it, it was less than a ~ electricity a month, and after I used it, it was more than two hundred kWh a month.	Jingdong prefecture level city in Shandong
Fortune Shop	Green Intelligent Power Saver	I just used it. I don't know how it is.	Jingdong prefecture level city in Shandong
Fortune Shop	Green Intelligent Power Saver	I've received the product and I'm using it, so I'll see how it goes in a month!	Jingdong prefecture level city in Shandong
Fortune Shop	Green Intelligent Power Saver	The goods have just received the effect do not know how, please look forward to the results.	Jingdong prefecture level city in Shandong
Fortune Shop	Green Intelligent Power Saver	Power saving device, the material is very good practical and durable, the size is suitable, packaging is very good, the price is cheap, logistics is very fast!	Jingdong prefecture level city in Shandong
Fortune Shop	Green Intelligent Power Saver	Very nice, will comment in detail after using it for a while.	Jingdong prefecture level city in Shandong
Fortune Shop	Green Intelligent Power Saver	Good stuff to trust and support	Jingdong prefecture level city in Shandong
Fortune Shop	Green Intelligent Power Saver	I'll comment after I use it, doubt if it really saves power no	Jingdong prefecture level city in Shandong
Fortune Shop	Green Intelligent Power Saver	It's good quality and works well!	Jingdong prefecture level city in Shandong
Fortune Shop	Green Intelligent Power Saver	hahaha just right hahahahahaha hiccup hahahaha	Jingdong prefecture level city in Shandong
Fortune Shop	Green Intelligent Power Saver	The real thing matches the picture and looks great!	Jingdong prefecture level city in Shandong
Fortune Shop	Green Intelligent Power Saver	Plugged it in and we'll see when we pay the bills at the end of the year!	Jingdong prefecture level city in Shandong
Fortune Shop	Green Intelligent Power Saver	False advertising. It doesn't work.	Jingdong prefecture level city in Shandong

Fortune Shop	Green Intelligent Power Saver	Goods received, good effect	Jingdong prefecture level city in Shandong
Fortune Shop	Green Intelligent Power Saver	It looks okay, we'll see if it saves electricity when the time comes	Jingdong prefecture level city in Shandong
Fortune Shop	Green Intelligent Power Saver	Logistics is very fast quality is very good, the price is very affordable, accessories are also complete, installed, satisfied with good use	Jingdong prefecture level city in Shandong
Fortune Shop	Green Intelligent Power Saver	I've received the power saver. I'm looking forward to the savings.	Jingdong prefecture level city in Shandong
Fortune Shop	Green Intelligent Power Saver	Received, have not yet used, after use and then evaluate	Jingdong prefecture level city in Shandong
Fortune Shop	Green Intelligent Power Saver	I received the item, but I don't know if it saves power or not.	Jingdong prefecture level city in Shandong
Fortune Shop	Green Intelligent Power Saver	On Trial	Jingdong prefecture level city in Shandong
Fortune Shop	Green Intelligent Power Saver	Use it and see how it goes.	Jingdong prefecture level city in Shandong
Fortune Shop	Green Intelligent Power Saver	Quality is very good, no odor, fine workmanship, grain color are very like, satisfied!	Jingdong prefecture level city in Shandong
Fortune Shop	Green Intelligent Power Saver	I'll give you a good review. I don't know if it'll work.	Jingdong prefecture level city in Shandong
Fortune Shop	Green Intelligent Power Saver	The logistics is very fast and the package is intact. Good use of affordable. The effect is very good, is genuine	Jingdong prefecture level city in Shandong
Fortune Shop	Green Intelligent Power Saver	Nothing happens when you plug it in.	Jingdong prefecture level city in Shandong
Fortune Shop	Green Intelligent Power Saver	I don't know if it's as good as it sounds.	Jingdong prefecture level city in Shandong
Fortune Shop	Green Intelligent Power Saver	The quality of the stuff is very good, exactly the same as the seller's description, very satisfied!	Jingdong prefecture level city in Shandong
Fortune Shop	Green Intelligent Power Saver	The saver works great, it really works, it's affordable and worth buying.	Jingdong prefecture level city in Shandong
Fortune Shop	Green Intelligent Power Saver	Just used it for a couple days. I don't know yet.	Jingdong prefecture level city in Shandong
Fortune Shop	Green Intelligent Power Saver	The quality is quite good, cheaper than the physical store, good, next time will come again!	Jingdong prefecture level city in Shandong
Fortune Shop	Green Intelligent Power Saver	Good quality, cheap and affordable worth recommending to buy!	Jingdong prefecture level city in Shandong
Fortune Shop	Green Intelligent Power Saver	Very good quality, well made, works well! Satisfied	Jingdong prefecture level city in Shandong
Fortune Shop	Green Intelligent Power Saver	The logistics is very fast quality is very good very pleasant a shopping	Jingdong prefecture level city in Shandong
Fortune Shop	Green Intelligent Power Saver	Use it and then come back to evaluate!	Jingdong prefecture level city in Shandong
Fortune Shop	Green Intelligent Power Saver	# It's no use lying at all #	Jingdong prefecture level city in Shandong
Fortune Shop	Green Intelligent Power Saver	I can't get a voltage drop.	Jingdong prefecture level city in Shandong
Fortune Shop	Green Intelligent Power Saver	Evaluate after use	Jingdong prefecture level city in Shandong
Fortune Shop	Green Intelligent Power Saver	Still on trial.	Jingdong prefecture level city in Shandong
Fortune Shop	Green Intelligent Power Saver	ineffective	Jingdong prefecture level city in Shandong
Fortune Shop	Green Intelligent Power Saver	Satisfaction Shopping	Jingdong prefecture level city in Shandong
Fortune Shop	Green Intelligent Power Saver	Lies.	Jingdong prefecture level city in Shandong
Fortune Shop	Green Intelligent Power Saver	It's a real power saver.	Jingdong prefecture level city in Shandong
Fortune Shop	Green Intelligent Power Saver	in use	Jingdong prefecture level city in Shandong
Fortune Shop	Green Intelligent Power Saver	I don't know yet.	Jingdong prefecture level city in Shandong
Fortune Shop	Green Intelligent Power Saver	not care about anything	Jingdong prefecture level city in Shandong
Fortune Shop	Green Intelligent Power Saver	It doesn't seem to have much effect	Jingdong prefecture level city in Shandong
Fortune Shop	Green Intelligent Power Saver	Let the merchant see for himself, does the plug positive and negative terminals look like this?	Jingdong prefecture level city in Shandong
Fortune Shop	Green Intelligent Power Saver	It doesn't work well, the electricity bill is less before using it, but more after using it, contacting customer service, the service is still not good, and returning it is slow	Jingdong prefecture level city in Shandong
Fortune Shop	Green Intelligent Power Saver	With the advertisement product does not match, above the effective negative, power saving rate of two rows of words it, the product looks very thin, with 3 days to see the meter number is still with the previous no difference, which is obviously fraudulent behavior!	Jingdong prefecture level city in Shandong
Fortune Shop	Green Intelligent Power Saver	Doesn't save any power at all. Not much use.	Jingdong prefecture level city in Shandong
Fortune Shop	Green Intelligent Power Saver	The plugs are all loose, they're all the same length and they don't work at all	Jingdong prefecture level city in Shandong
Fortune Shop	Green Intelligent Power Saver	It's bad 👎 it's not effective at all, you'd be crazy to sell this garbage on the internet.	Jingdong prefecture level city in Shandong
Fortune Shop	Green Intelligent Power Saver	It doesn't save any power at all, so I hope the old timers don't fall for this scam again	Jingdong prefecture level city in Shandong
Fortune Shop	Green Intelligent Power Saver	I've been using it for over a month now and haven't noticed any power savings.	Jingdong prefecture level city in Shandong
Fortune Shop	Green Intelligent Power Saver	It doesn't work at all. The electricity bill is higher when you use it.	Jingdong prefecture level city in Shandong
Fortune Shop	Green Intelligent Power Saver	It doesn't save any power, it's still as bad as ever.	Jingdong prefecture level city in Shandong
Fortune Shop	Green Intelligent Power Saver	Last night before leaving work to look at the table, and then plugged into the node device, this morning and then look at the table, than the original save a few degrees of electricity, very good to have a power-saving effect, easy to use, satisfied with the shopping praise.	Jingdong prefecture level city in Shandong
Fortune Shop	Green Intelligent Power Saver	The product is real and effective, the electricity bill is reduced, really good, thanks to the merchants have so many good products, the product is real and effective, the electricity bill is reduced, want to save electricity friends quickly buy it, early use early to save money.	Jingdong prefecture level city in Shandong
Fortune Shop	Green Intelligent Power Saver	Saver saves time and money, baby appearance is beautiful, the quality of goods is good, and the same as the business description, good service attitude, packaging is complete, logistics is very fast, praise to	Jingdong prefecture level city in Shandong
Fortune Shop	Green Intelligent Power Saver	The effect is good, summer, home electricity is relatively large, buy and use a few days, feel the effect is very good, originally ten dollars a day electricity bill, now eight dollars a day, quite good	Jingdong prefecture level city in Shandong
Fortune Shop	Green Intelligent Power Saver	Indeed can save electricity and money, the first time I know this or from a friend's home to see, but also do not believe it, and now they use it to know that there is this good thing.	Jingdong prefecture level city in Shandong
Fortune Shop	Green Intelligent Power Saver	I have received the Smart Power Saver. Perfectly packaged. Fast delivery. Boss customer service attitude is also very good. It's worth buying. I plugged it in and tried it and it really saves more power than the original. I'll buy it again next time.	Jingdong prefecture level city in Shandong

Fortune Shop	Green Intelligent Power Saver	It's nice to save a dozen bucks a night on two air conditioners that used to take 30 or 40 degrees a night, but now it's only 10 or so degrees a night	Jingdong prefecture level city in Shandong
Fortune Shop	Green Intelligent Power Saver	Product no manufacturer's name no product qualification certificate, try two days to see the effect of	Jingdong prefecture level city in Shandong
Fortune Shop	Green Intelligent Power Saver	I've been using it for over a month and have saved over $80, so I've repurchased a few more back to save together	Jingdong prefecture level city in Shandong
Fortune Shop	Green Intelligent Power Saver	I haven't used it yet and will comment when I do, but it's well made.	Jingdong prefecture level city in Shandong
Fortune Shop	Green Intelligent Power Saver	It's quite energy efficient. It's really a boon for the home.	Jingdong prefecture level city in Shandong
Fortune Shop	Green Intelligent Power Saver	Energy-saving socket received a month, after using it for a month proved that it is indeed energy-saving, electricity bill saving, will buy it again, thank you!	Jingdong prefecture level city in Shandong
Fortune Shop	Green Intelligent Power Saver	There was a similar product many years ago. Now technology has evolved, buy one to try, according to the instructions in the box, it takes one or two dozen days to show results. Check your electric bill and see what happens.	Jingdong prefecture level city in Shandong
Fortune Shop	Green Intelligent Power Saver	Jingdong logistics is really fast, the same day the order arrived on the same day, more convenient than the supermarket, and a wide range of choices, the	Jingdong prefecture level city in Shandong
Fortune Shop	Green Intelligent Power Saver	I've been using this for a month or so and I can't see any results, it really is a scam as they say.	Jingdong prefecture level city in Shandong
Fortune Shop	Green Intelligent Power Saver	Very good use, a month can save a few degrees of power loss of electricity, introduced to the relatives to buy back to try.	Jingdong prefecture level city in Shandong
Fortune Shop	Green Intelligent Power Saver	It's really powerful! With this air conditioner can be turned on at any time without the pressure of paying the electricity bill! It's good for the people!	Jingdong prefecture level city in Shandong
Fortune Shop	Green Intelligent Power Saver	This business is a big ** products are not qualified, with a month of electricity more than one hundred percent of thirty, the purchase agreed to return the goods but do not receive a refund after receiving the goods, looking for the official side does not help, the Jingdong is a big **, I have never shopped in	Jingdong prefecture level city in Shandong
Fortune Shop	Green Intelligent Power Saver	It's good. I tried it. It saves power.	Jingdong prefecture level city in Shandong
Fortune Shop	Green Intelligent Power Saver	Nothing**, it seems like there's more	Jingdong prefecture level city in Shandong
Fortune Shop	Green Intelligent Power Saver	Power saving device received, well packaged, good quality, fast logistics	Jingdong prefecture level city in Shandong
Fortune Shop	Green Intelligent Power Saver	The service is okay, is that when I bought five, the desirable pieces are three, and then wait a few days and send me two replacement, I do not know if it is a mistake of the shipper, if I was not in the courier there to see, go home to say no, the	Jingdong prefecture level city in Shandong
Fortune Shop	Green Intelligent Power Saver	This thing will only cost me more if I use it, but before I used it, it was less than a ~ electricity a month, and after I used it, it was more than two hundred kWh a month.	Jingdong prefecture level city in Shandong
Fortune Shop	Green Intelligent Power Saver	I just used it. I don't know how it is.	Jingdong prefecture level city in Shandong
Fortune Shop	Green Intelligent Power Saver	I've received the product and I'm using it, so I'll see how it goes in a month!	Jingdong prefecture level city in Shandong
Fortune Shop	Green Intelligent Power Saver	The goods have just received the effect do not know how, please look forward to the results.	Jingdong prefecture level city in Shandong
Fortune Shop	Green Intelligent Power Saver	Power saving device, the material is very good practical and durable, the size is suitable, packaging is very good, the price is cheap, logistics is very fast!	Jingdong prefecture level city in Shandong
Fortune Shop	Green Intelligent Power Saver	Very nice, will comment in detail after using it for a while.	Jingdong prefecture level city in Shandong
Fortune Shop	Green Intelligent Power Saver	Good stuff to trust and support	Jingdong prefecture level city in Shandong
Fortune Shop	Green Intelligent Power Saver	I'll comment after I use it, doubt if it really saves power no	Jingdong prefecture level city in Shandong
Fortune Shop	Green Intelligent Power Saver	It's good quality and works well!	Jingdong prefecture level city in Shandong
Fortune Shop	Green Intelligent Power Saver	hahaha just right hahahahahaha hiccup hahahaha	Jingdong prefecture level city in Shandong
Fortune Shop	Green Intelligent Power Saver	The real thing matches the picture and looks great!	Jingdong prefecture level city in Shandong
Fortune Shop	Green Intelligent Power Saver	Plugged it in and we'll see when we pay the bills at the end of the year!	Jingdong prefecture level city in Shandong
Fortune Shop	Green Intelligent Power Saver	False advertising. It doesn't work.	Jingdong prefecture level city in Shandong
Fortune Shop	Green Intelligent Power Saver	Goods received, good effect	Jingdong prefecture level city in Shandong
Fortune Shop	Green Intelligent Power Saver	It looks okay, we'll see if it saves electricity when the time comes	Jingdong prefecture level city in Shandong
Fortune Shop	Green Intelligent Power Saver	Logistics is very fast quality is very good, the price is very affordable, accessories are also complete, installed, satisfied with good use	Jingdong prefecture level city in Shandong
Fortune Shop	Green Intelligent Power Saver	I've received the power saver. I'm looking forward to the savings.	Jingdong prefecture level city in Shandong
Fortune Shop	Green Intelligent Power Saver	Received, have not yet used, after use and then evaluate	Jingdong prefecture level city in Shandong
Fortune Shop	Green Intelligent Power Saver	I received the item, but I don't know if it saves power or not.	Jingdong prefecture level city in Shandong
Fortune Shop	Green Intelligent Power Saver	On Trial	Jingdong prefecture level city in Shandong
Fortune Shop	Green Intelligent Power Saver	Use it and see how it goes.	Jingdong prefecture level city in Shandong
Fortune Shop	Green Intelligent Power Saver	Quality is very good, no odor, fine workmanship, grain color are very like, satisfied!	Jingdong prefecture level city in Shandong
Fortune Shop	Green Intelligent Power Saver	I'll give you a good review. I don't know if it'll work.	Jingdong prefecture level city in Shandong
Fortune Shop	Green Intelligent Power Saver	The logistics is very fast and the package is intact. Good use of affordable. The effect is very good, is genuine	Jingdong prefecture level city in Shandong
Fortune Shop	Green Intelligent Power Saver	Nothing happens when you plug it in.	Jingdong prefecture level city in Shandong
Fortune Shop	Green Intelligent Power Saver	I don't know if it's as good as it sounds.	Jingdong prefecture level city in Shandong
Fortune Shop	Green Intelligent Power Saver	The quality of the stuff is very good, exactly the same as the seller's description, very satisfied!	Jingdong prefecture level city in Shandong
Fortune Shop	Green Intelligent Power Saver	The saver works great, it really works, it's affordable and worth buying.	Jingdong prefecture level city in Shandong
Fortune Shop	Green Intelligent Power Saver	Just used it for a couple days. I don't know yet.	Jingdong prefecture level city in Shandong
Fortune Shop	Green Intelligent Power Saver	The quality is quite good, cheaper than the physical store, good, next time will come again!	Jingdong prefecture level city in Shandong
Fortune Shop	Green Intelligent Power Saver	Good quality, cheap and affordable worth recommending to buy!	Jingdong prefecture level city in Shandong
Fortune Shop	Green Intelligent Power Saver	Very good quality, well made, works well! Satisfied	Jingdong prefecture level city in Shandong
Fortune Shop	Green Intelligent Power Saver	The logistics is very fast quality is very good very pleasant a shopping	Jingdong prefecture level city in Shandong
Fortune Shop	Green Intelligent Power Saver	Use it and then come back to evaluate!	Jingdong prefecture level city in Shandong

Fortune Shop	Green Intelligent Power Saver	# It's no use lying at all #	Jingdong prefecture level city in Shandong
Fortune Shop	Green Intelligent Power Saver	I can't get a voltage drop.	Jingdong prefecture level city in Shandong
Fortune Shop	Green Intelligent Power Saver	Evaluate after use	Jingdong prefecture level city in Shandong
Fortune Shop	Green Intelligent Power Saver	Still on trial.	Jingdong prefecture level city in Shandong
Fortune Shop	Green Intelligent Power Saver	ineffective	Jingdong prefecture level city in Shandong
Fortune Shop	Green Intelligent Power Saver	Satisfaction Shopping	Jingdong prefecture level city in Shandong
Fortune Shop	Green Intelligent Power Saver	Lies.	Jingdong prefecture level city in Shandong
Fortune Shop	Green Intelligent Power Saver	It's a real power saver.	Jingdong prefecture level city in Shandong
Fortune Shop	Green Intelligent Power Saver	in use	Jingdong prefecture level city in Shandong
Fortune Shop	Green Intelligent Power Saver	I don't know yet.	Jingdong prefecture level city in Shandong
Fortune Shop	Green Intelligent Power Saver	not care about anything	Jingdong prefecture level city in Shandong
Fortune Shop	Green Intelligent Power Saver	It doesn't seem to have much effect	Jingdong prefecture level city in Shandong
Fortune Shop	Green Intelligent Power Saver	Let the merchant see for himself, does the plug positive and negative terminals look like this?	Jingdong prefecture level city in Shandong
Fortune Shop	Green Intelligent Power Saver	It doesn't work well, the electricity bill is less before using it, but more after using it, contacting customer service, the service is still not good, and returning it is slow	Jingdong prefecture level city in Shandong
Fortune Shop	Green Intelligent Power Saver	With the advertisement product does not match, above the effective negative, power saving rate of two rows of words it, the product looks very thin, with 3 days to see the meter number is still with the previous no difference, which is obviously fraudulent behavior!	Jingdong prefecture level city in Shandong
Fortune Shop	Green Intelligent Power Saver	Doesn't save any power at all. Not much use.	Jingdong prefecture level city in Shandong
Fortune Shop	Green Intelligent Power Saver	The plugs are all loose, they're all the same length and they don't work at all	Jingdong prefecture level city in Shandong
Fortune Shop	Green Intelligent Power Saver	It's bad 👎 it's not effective at all, you'd be crazy to sell this garbage on the internet.	Jingdong prefecture level city in Shandong
Fortune Shop	Green Intelligent Power Saver	It doesn't save any power at all, so I hope the old timers don't fall for this scam again	Jingdong prefecture level city in Shandong
Fortune Shop	Green Intelligent Power Saver	I've been using it for over a month now and haven't noticed any power savings.	Jingdong prefecture level city in Shandong
Fortune Shop	Green Intelligent Power Saver	It doesn't work at all. The electricity bill is higher when you use it.	Jingdong prefecture level city in Shandong
Fortune Shop	Green Intelligent Power Saver	It doesn't save any power, it's still as bad as ever.	Jingdong prefecture level city in Shandong
Fortune Shop	Green Intelligent Power Saver	Last night before leaving work to look at the table, and then plugged into the node device, this morning and then look at the table, than the original save a few degrees of electricity, very good to have a power-saving effect, easy to use, satisfied with the shopping praise.	Jingdong prefecture level city in Shandong
Fortune Shop	Green Intelligent Power Saver	The product is real and effective, the electricity bill is reduced, really good, thanks to the merchants have so many good products, the product is real and effective, the electricity bill is reduced, want to save electricity friends quickly buy it, early use early to save money.	Jingdong prefecture level city in Shandong
Fortune Shop	Green Intelligent Power Saver	Saver saves time and money, baby appearance is beautiful, the quality of goods is good, and the same as the business description, good service attitude, packaging is complete, logistics is very fast, praise to	Jingdong prefecture level city in Shandong
Fortune Shop	Green Intelligent Power Saver	The effect is good, summer, home electricity is relatively large, buy and use a few days, feel the effect is very good, originally ten dollars a day electricity bill, now eight dollars a day, quite good	Jingdong prefecture level city in Shandong
Fortune Shop	Green Intelligent Power Saver	Indeed can save electricity and money, the first time I know this or from a friend's home to see, but also do not believe it, and now they use it to know that there is this good thing.	Jingdong prefecture level city in Shandong
Fortune Shop	Green Intelligent Power Saver	I have received the Smart Power Saver. Perfectly packaged. Fast delivery. Boss customer service attitude is also very good. It's worth buying. I plugged it in and tried it and it really saves more power than the original. I'll buy it again next time.	Jingdong prefecture level city in Shandong
Fortune Shop	Green Intelligent Power Saver	It's nice to save a dozen bucks a night on two air conditioners that used to take 30 or 40 degrees a night, but now it's only 10 or so degrees a night	Jingdong prefecture level city in Shandong
Fortune Shop	Green Intelligent Power Saver	Product no manufacturer's name no product qualification certificate, try two days to see the effect of	Jingdong prefecture level city in Shandong
Fortune Shop	Green Intelligent Power Saver	I've been using it for over a month and have saved over $80, so I've repurchased a few more back to save together	Jingdong prefecture level city in Shandong
Fortune Shop	Green Intelligent Power Saver	I haven't used it yet and will comment when I do, but it's well made.	Jingdong prefecture level city in Shandong
Fortune Shop	Green Intelligent Power Saver	It's quite energy efficient. It's really a boon for the home.	Jingdong prefecture level city in Shandong
Fortune Shop	Green Intelligent Power Saver	Energy-saving socket received a month, after using it for a month proved that it is indeed energy-saving, electricity bill saving, will buy it again, thank you!	Jingdong prefecture level city in Shandong
Fortune Shop	Green Intelligent Power Saver	There was a similar product many years ago. Now technology has evolved, buy one to try, according to the instructions in the box, it takes one or two dozen days to show results. Check your electric bill and see what happens.	Jingdong prefecture level city in Shandong
Fortune Shop	Green Intelligent Power Saver	Jingdong logistics is really fast, the same day the order arrived on the same day, more convenient than the supermarket, and a wide range of choices, the	Jingdong prefecture level city in Shandong
Fortune Shop	Green Intelligent Power Saver	I've been using this for a month or so and I can't see any results, it really is a scam as they say.	Jingdong prefecture level city in Shandong
Fortune Shop	Green Intelligent Power Saver	Very good use, a month can save a few degrees of power loss of electricity, introduced to the relatives to buy back to try.	Jingdong prefecture level city in Shandong
Fortune Shop	Green Intelligent Power Saver	It's really powerful! With this air conditioner can be turned on at any time without the pressure of paying the electricity bill! It's good for the people!	Jingdong prefecture level city in Shandong
Fortune Shop	Green Intelligent Power Saver	This business is a big ** products are not qualified, with a month of electricity more than one hundred percent of thirty, the purchase agreed to return the goods but do not receive a refund after receiving the goods, looking for the official side does not help, the Jingdong is a big **, I have never shopped in	Jingdong prefecture level city in Shandong
Fortune Shop	Green Intelligent Power Saver	It's good. I tried it. It saves power.	Jingdong prefecture level city in Shandong
Fortune Shop	Green Intelligent Power Saver	Nothing**, it seems like there's more	Jingdong prefecture level city in Shandong
Fortune Shop	Green Intelligent Power Saver	Power saving device received, well packaged, good quality, fast logistics	Jingdong prefecture level city in Shandong
Fortune Shop	Green Intelligent Power Saver	The service is okay, is that when I bought five, the desirable pieces are three, and then wait a few days and send me two replacement, I do not know if it is a mistake of the shipper, if I was not in the courier there to see, go home to say no, the	Jingdong prefecture level city in Shandong
Fortune Shop	Green Intelligent Power Saver	This thing will only cost me more if I use it, but before I used it, it was less than a ~ electricity a month, and after I used it, it was more than two hundred kWh a month.	Jingdong prefecture level city in Shandong
Fortune Shop	Green Intelligent Power Saver	I just used it. I don't know how it is.	Jingdong prefecture level city in Shandong
Fortune Shop	Green Intelligent Power Saver	I've received the product and I'm using it, so I'll see how it goes in a month!	Jingdong prefecture level city in Shandong
Fortune Shop	Green Intelligent Power Saver	The goods have just received the effect do not know how, please look forward to the results.	Jingdong prefecture level city in Shandong

Fortune Shop	Green Intelligent Power Saver	Power saving device, the material is very good practical and durable, the size is suitable, packaging is very good, the price is cheap, logistics is very fast!	Jingdong prefecture level city in Shandong
Fortune Shop	Green Intelligent Power Saver	Very nice, will comment in detail after using it for a while.	Jingdong prefecture level city in Shandong
Fortune Shop	Green Intelligent Power Saver	Good stuff to trust and support	Jingdong prefecture level city in Shandong
Fortune Shop	Green Intelligent Power Saver	I'll comment after I use it, doubt if it really saves power no	Jingdong prefecture level city in Shandong
Fortune Shop	Green Intelligent Power Saver	It's good quality and works well!	Jingdong prefecture level city in Shandong
Fortune Shop	Green Intelligent Power Saver	hahaha just right hahahahahaha hiccup hahahaha	Jingdong prefecture level city in Shandong
Fortune Shop	Green Intelligent Power Saver	The real thing matches the picture and looks great!	Jingdong prefecture level city in Shandong
Fortune Shop	Green Intelligent Power Saver	Plugged it in and we'll see when we pay the bills at the end of the year!	Jingdong prefecture level city in Shandong
Fortune Shop	Green Intelligent Power Saver	False advertising. It doesn't work.	Jingdong prefecture level city in Shandong
Fortune Shop	Green Intelligent Power Saver	Goods received, good effect	Jingdong prefecture level city in Shandong
Fortune Shop	Green Intelligent Power Saver	It looks okay, we'll see if it saves electricity when the time comes	Jingdong prefecture level city in Shandong
Fortune Shop	Green Intelligent Power Saver	Logistics is very fast quality is very good, the price is very affordable, accessories are also complete, installed, satisfied with good use	Jingdong prefecture level city in Shandong
Fortune Shop	Green Intelligent Power Saver	I've received the power saver. I'm looking forward to the savings.	Jingdong prefecture level city in Shandong
Fortune Shop	Green Intelligent Power Saver	Received, have not yet used, after use and then evaluate	Jingdong prefecture level city in Shandong
Fortune Shop	Green Intelligent Power Saver	I received the item, but I don't know if it saves power or not.	Jingdong prefecture level city in Shandong
Fortune Shop	Green Intelligent Power Saver	On Trial	Jingdong prefecture level city in Shandong
Fortune Shop	Green Intelligent Power Saver	Use it and see how it goes.	Jingdong prefecture level city in Shandong
Fortune Shop	Green Intelligent Power Saver	Quality is very good, no odor, fine workmanship, grain color are very like, satisfied!	Jingdong prefecture level city in Shandong
Fortune Shop	Green Intelligent Power Saver	I'll give you a good review. I don't know if it'll work.	Jingdong prefecture level city in Shandong
Fortune Shop	Green Intelligent Power Saver	The logistics is very fast and the package is intact. Good use of affordable. The effect is very good, is genuine	Jingdong prefecture level city in Shandong
Fortune Shop	Green Intelligent Power Saver	Nothing happens when you plug it in.	Jingdong prefecture level city in Shandong
Fortune Shop	Green Intelligent Power Saver	I don't know if it's as good as it sounds.	Jingdong prefecture level city in Shandong
Fortune Shop	Green Intelligent Power Saver	The quality of the stuff is very good, exactly the same as the seller's description, very satisfied!	Jingdong prefecture level city in Shandong
Fortune Shop	Green Intelligent Power Saver	The saver works great, it really works, it's affordable and worth buying.	Jingdong prefecture level city in Shandong
Fortune Shop	Green Intelligent Power Saver	Just used it for a couple days. I don't know yet.	Jingdong prefecture level city in Shandong
Fortune Shop	Green Intelligent Power Saver	The quality is quite good, cheaper than the physical store, good, next time will come again!	Jingdong prefecture level city in Shandong
Fortune Shop	Green Intelligent Power Saver	Good quality, cheap and affordable worth recommending to buy!	Jingdong prefecture level city in Shandong
Fortune Shop	Green Intelligent Power Saver	Very good quality, well made, works well! Satisfied	Jingdong prefecture level city in Shandong
Fortune Shop	Green Intelligent Power Saver	The logistics is very fast quality is very good very pleasant a shopping	Jingdong prefecture level city in Shandong
Fortune Shop	Green Intelligent Power Saver	Use it and then come back to evaluate!	Jingdong prefecture level city in Shandong
Fortune Shop	Green Intelligent Power Saver	# It's no use lying at all #	Jingdong prefecture level city in Shandong
Fortune Shop	Green Intelligent Power Saver	I can't get a voltage drop.	Jingdong prefecture level city in Shandong
Fortune Shop	Green Intelligent Power Saver	Evaluate after use	Jingdong prefecture level city in Shandong
Fortune Shop	Green Intelligent Power Saver	Still on trial.	Jingdong prefecture level city in Shandong
Fortune Shop	Green Intelligent Power Saver	ineffective	Jingdong prefecture level city in Shandong
Fortune Shop	Green Intelligent Power Saver	Satisfaction Shopping	Jingdong prefecture level city in Shandong
Fortune Shop	Green Intelligent Power Saver	Lies.	Jingdong prefecture level city in Shandong
Fortune Shop	Green Intelligent Power Saver	It's a real power saver.	Jingdong prefecture level city in Shandong
Fortune Shop	Green Intelligent Power Saver	in use	Jingdong prefecture level city in Shandong
Fortune Shop	Green Intelligent Power Saver	I don't know yet.	Jingdong prefecture level city in Shandong
Fortune Shop	Green Intelligent Power Saver	not care about anything	Jingdong prefecture level city in Shandong
Fortune Shop	Green Intelligent Power Saver	It doesn't seem to have much effect	Jingdong prefecture level city in Shandong
Fortune Shop	Green Intelligent Power Saver	Let the merchant see for himself, does the plug positive and negative terminals look like this?	Jingdong prefecture level city in Shandong
Fortune Shop	Green Intelligent Power Saver	It doesn't work well, the electricity bill is less before using it, but more after using it, contacting customer service, the service is still not good, and returning it is slow	Jingdong prefecture level city in Shandong
Fortune Shop	Green Intelligent Power Saver	With the advertisement product does not match, above the effective negative, power saving rate of two rows of words it, the product looks very thin, with 3 days to see the meter number is still with the previous no difference, which is obviously fraudulent behavior!	Jingdong prefecture level city in Shandong
Fortune Shop	Green Intelligent Power Saver	Doesn't save any power at all. Not much use.	Jingdong prefecture level city in Shandong
Fortune Shop	Green Intelligent Power Saver	The plugs are all loose, they're all the same length and they don't work at all	Jingdong prefecture level city in Shandong
Fortune Shop	Green Intelligent Power Saver	It's bad 👎 it's not effective at all, you'd be crazy to sell this garbage on the internet.	Jingdong prefecture level city in Shandong
Fortune Shop	Green Intelligent Power Saver	It doesn't save any power at all, so I hope the old timers don't fall for this scam again	Jingdong prefecture level city in Shandong
Fortune Shop	Green Intelligent Power Saver	I've been using it for over a month now and haven't noticed any power savings.	Jingdong prefecture level city in Shandong
Fortune Shop	Green Intelligent Power Saver	It doesn't work at all. The electricity bill is higher when you use it.	Jingdong prefecture level city in Shandong

Fortune Shop	Green Intelligent Power Saver	It doesn't save any power, it's still as bad as ever.	Jingdong prefecture level city in Shandong
Fortune Shop	Green Intelligent Power Saver	Last night before leaving work to look at the table, and then plugged into the node device, this morning and then look at the table, than the original save a few degrees of electricity, very good to have a power-saving effect, easy to use, satisfied with the shopping praise.	Jingdong prefecture level city in Shandong
Fortune Shop	Green Intelligent Power Saver	The product is real and effective, the electricity bill is reduced, really good, thanks to the merchants have so many good products, the product is real and effective, the electricity bill is reduced, want to save electricity friends quickly buy it, early use early to save money.	Jingdong prefecture level city in Shandong
Fortune Shop	Green Intelligent Power Saver	Saver saves time and money, baby appearance is beautiful, the quality of goods is good, and the same as the business description, good service attitude, packaging is complete, logistics is very fast, praise to	Jingdong prefecture level city in Shandong
Fortune Shop	Green Intelligent Power Saver	The effect is good, summer, home electricity is relatively large, buy and use a few days, feel the effect is very good, originally ten dollars a day electricity bill, now eight dollars a day, quite good	Jingdong prefecture level city in Shandong
Fortune Shop	Green Intelligent Power Saver	Indeed can save electricity and money, the first time I know this or from a friend's home to see, but also do not believe it, and now they use it to know that there is this good thing.	Jingdong prefecture level city in Shandong
Fortune Shop	Green Intelligent Power Saver	I have received the Smart Power Saver. Perfectly packaged. Fast delivery. Boss customer service attitude is also very good. It's worth buying. I plugged it in and tried it and it really saves more power than the original. I'll buy it again next time.	Jingdong prefecture level city in Shandong
Fortune Shop	Green Intelligent Power Saver	It's nice to save a dozen bucks a night on two air conditioners that used to take 30 or 40 degrees a night, but now it's only 10 or so degrees a night	Jingdong prefecture level city in Shandong
Fortune Shop	Green Intelligent Power Saver	Product no manufacturer's name no product qualification certificate, try two days to see the effect of	Jingdong prefecture level city in Shandong
Fortune Shop	Green Intelligent Power Saver	I've been using it for over a month and have saved over $80, so I've repurchased a few more back to save together	Jingdong prefecture level city in Shandong
Fortune Shop	Green Intelligent Power Saver	I haven't used it yet and will comment when I do, but it's well made.	Jingdong prefecture level city in Shandong
Fortune Shop	Green Intelligent Power Saver	It's quite energy efficient. It's really a boon for the home.	Jingdong prefecture level city in Shandong
Fortune Shop	Green Intelligent Power Saver	Energy-saving socket received a month, after using it for a month proved that it is indeed energy-saving, electricity bill saving, will buy it again, thank you!	Jingdong prefecture level city in Shandong
Fortune Shop	Green Intelligent Power Saver	There was a similar product many years ago. Now technology has evolved, buy one to try, according to the instructions in the box, it takes one or two dozen days to show results. Check your electric bill and see what happens.	Jingdong prefecture level city in Shandong
Fortune Shop	Green Intelligent Power Saver	Jingdong logistics is really fast, the same day the order arrived on the same day, more convenient than the supermarket, and a wide range of choices, the	Jingdong prefecture level city in Shandong
Fortune Shop	Green Intelligent Power Saver	I've been using this for a month or so and I can't see any results, it really is a scam as they say.	Jingdong prefecture level city in Shandong
Fortune Shop	Green Intelligent Power Saver	Very good use, a month can save a few degrees of power loss of electricity, introduced to the relatives to buy back to try.	Jingdong prefecture level city in Shandong
Fortune Shop	Green Intelligent Power Saver	It's really powerful! With this air conditioner can be turned on at any time without the pressure of paying the electricity bill! It's good for the people!	Jingdong prefecture level city in Shandong
Fortune Shop	Green Intelligent Power Saver	This business is a big ** products are not qualified, with a month of electricity more than one hundred percent of thirty, the purchase agreed to return the goods but do not receive a refund after receiving the goods, looking for the official side does not help, the Jingdong is a big **, I have never shopped in	Jingdong prefecture level city in Shandong
Fortune Shop	Green Intelligent Power Saver	It's good. I tried it. It saves power.	Jingdong prefecture level city in Shandong
Fortune Shop	Green Intelligent Power Saver	Nothing**, it seems like there's more	Jingdong prefecture level city in Shandong
Fortune Shop	Green Intelligent Power Saver	Power saving device received, well packaged, good quality, fast logistics	Jingdong prefecture level city in Shandong
Fortune Shop	Green Intelligent Power Saver	The service is okay, is that when I bought five, the desirable pieces are three, and then wait a few days and send me two replacement, I do not know if it is a mistake of the shipper, if I was not in the courier there to see, go home to say no, the	Jingdong prefecture level city in Shandong
Fortune Shop	Green Intelligent Power Saver	This thing will only cost me more if I use it, but before I used it, it was less than a ~ electricity a month, and after I used it, it was more than two hundred kWh a month.	Jingdong prefecture level city in Shandong
Fortune Shop	Green Intelligent Power Saver	I just used it. I don't know how it is.	Jingdong prefecture level city in Shandong
Fortune Shop	Green Intelligent Power Saver	I've received the product and I'm using it, so I'll see how it goes in a month!	Jingdong prefecture level city in Shandong
Fortune Shop	Green Intelligent Power Saver	The goods have just received the effect do not know how, please look forward to the results.	Jingdong prefecture level city in Shandong
Fortune Shop	Green Intelligent Power Saver	Power saving device, the material is very good practical and durable, the size is suitable, packaging is very good, the price is cheap, logistics is very fast!	Jingdong prefecture level city in Shandong
Fortune Shop	Green Intelligent Power Saver	Very nice, will comment in detail after using it for a while.	Jingdong prefecture level city in Shandong
Fortune Shop	Green Intelligent Power Saver	Good stuff to trust and support	Jingdong prefecture level city in Shandong
Fortune Shop	Green Intelligent Power Saver	I'll comment after I use it, doubt if it really saves power no	Jingdong prefecture level city in Shandong
Fortune Shop	Green Intelligent Power Saver	It's good quality and works well!	Jingdong prefecture level city in Shandong
Fortune Shop	Green Intelligent Power Saver	hahaha just right hahahahahaha hiccup hahahaha	Jingdong prefecture level city in Shandong
Fortune Shop	Green Intelligent Power Saver	The real thing matches the picture and looks great!	Jingdong prefecture level city in Shandong
Fortune Shop	Green Intelligent Power Saver	Plugged it in and we'll see when we pay the bills at the end of the year!	Jingdong prefecture level city in Shandong
Fortune Shop	Green Intelligent Power Saver	False advertising. It doesn't work.	Jingdong prefecture level city in Shandong
Fortune Shop	Green Intelligent Power Saver	Goods received, good effect	Jingdong prefecture level city in Shandong
Fortune Shop	Green Intelligent Power Saver	It looks okay, we'll see if it saves electricity when the time comes	Jingdong prefecture level city in Shandong
Fortune Shop	Green Intelligent Power Saver	Logistics is very fast quality is very good, the price is very affordable, accessories are also complete, installed, satisfied with good use	Jingdong prefecture level city in Shandong
Fortune Shop	Green Intelligent Power Saver	I've received the power saver. I'm looking forward to the savings.	Jingdong prefecture level city in Shandong
Fortune Shop	Green Intelligent Power Saver	Received, have not yet used, after use and then evaluate	Jingdong prefecture level city in Shandong
Fortune Shop	Green Intelligent Power Saver	I received the item, but I don't know if it saves power or not.	Jingdong prefecture level city in Shandong
Fortune Shop	Green Intelligent Power Saver	On Trial	Jingdong prefecture level city in Shandong
Fortune Shop	Green Intelligent Power Saver	Use it and see how it goes.	Jingdong prefecture level city in Shandong
Fortune Shop	Green Intelligent Power Saver	Quality is very good, no odor, fine workmanship, grain color are very like, satisfied!	Jingdong prefecture level city in Shandong
Fortune Shop	Green Intelligent Power Saver	I'll give you a good review. I don't know if it'll work.	Jingdong prefecture level city in Shandong
Fortune Shop	Green Intelligent Power Saver	The logistics is very fast and the package is intact. Good use of affordable. The effect is very good, is genuine	Jingdong prefecture level city in Shandong
Fortune Shop	Green Intelligent Power Saver	Nothing happens when you plug it in.	Jingdong prefecture level city in Shandong

Fortune Shop	Green Intelligent Power Saver	I don't know if it's as good as it sounds.	Jingdong prefecture level city in Shandong
Fortune Shop	Green Intelligent Power Saver	The quality of the stuff is very good, exactly the same as the seller's description, very satisfied!	Jingdong prefecture level city in Shandong
Fortune Shop	Green Intelligent Power Saver	The saver works great, it really works, it's affordable and worth buying.	Jingdong prefecture level city in Shandong
Fortune Shop	Green Intelligent Power Saver	Just used it for a couple days. I don't know yet.	Jingdong prefecture level city in Shandong
Fortune Shop	Green Intelligent Power Saver	The quality is quite good, cheaper than the physical store, good, next time will come again!	Jingdong prefecture level city in Shandong
Fortune Shop	Green Intelligent Power Saver	Good quality, cheap and affordable worth recommending to buy!	Jingdong prefecture level city in Shandong
Fortune Shop	Green Intelligent Power Saver	Very good quality, well made, works well! Satisfied	Jingdong prefecture level city in Shandong
Fortune Shop	Green Intelligent Power Saver	The logistics is very fast quality is very good very pleasant a shopping	Jingdong prefecture level city in Shandong
Fortune Shop	Green Intelligent Power Saver	Use it and then come back to evaluate!	Jingdong prefecture level city in Shandong
Fortune Shop	Green Intelligent Power Saver	# It's no use lying at all #	Jingdong prefecture level city in Shandong
Fortune Shop	Green Intelligent Power Saver	I can't get a voltage drop.	Jingdong prefecture level city in Shandong
Fortune Shop	Green Intelligent Power Saver	Evaluate after use	Jingdong prefecture level city in Shandong
Fortune Shop	Green Intelligent Power Saver	Still on trial.	Jingdong prefecture level city in Shandong
Fortune Shop	Green Intelligent Power Saver	ineffective	Jingdong prefecture level city in Shandong
Fortune Shop	Green Intelligent Power Saver	Satisfaction Shopping	Jingdong prefecture level city in Shandong
Fortune Shop	Green Intelligent Power Saver	Lies.	Jingdong prefecture level city in Shandong
Fortune Shop	Green Intelligent Power Saver	It's a real power saver.	Jingdong prefecture level city in Shandong
Fortune Shop	Green Intelligent Power Saver	in use	Jingdong prefecture level city in Shandong
Fortune Shop	Green Intelligent Power Saver	I don't know yet.	Jingdong prefecture level city in Shandong
Fortune Shop	Green Intelligent Power Saver	not care about anything	Jingdong prefecture level city in Shandong
Fortune Shop	Green Intelligent Power Saver	It doesn't seem to have much effect	Jingdong prefecture level city in Shandong
Fortune Shop	Green Intelligent Power Saver	Let the merchant see for himself, does the plug positive and negative terminals look like this?	Jingdong prefecture level city in Shandong
Fortune Shop	Green Intelligent Power Saver	It doesn't work well, the electricity bill is less before using it, but more after using it, contacting customer service, the service is still not good, and returning it is slow	Jingdong prefecture level city in Shandong
Fortune Shop	Green Intelligent Power Saver	With the advertisement product does not match, above the effective negative, power saving rate of two rows of words it, the product looks very thin, with 3 days to see the meter number is still with the previous no difference, which is obviously fraudulent behavior!	Jingdong prefecture level city in Shandong
Fortune Shop	Green Intelligent Power Saver	Doesn't save any power at all. Not much use.	Jingdong prefecture level city in Shandong
Fortune Shop	Green Intelligent Power Saver	The plugs are all loose, they're all the same length and they don't work at all	Jingdong prefecture level city in Shandong
Fortune Shop	Green Intelligent Power Saver	It's bad 👎 it's not effective at all, you'd be crazy to sell this garbage on the internet.	Jingdong prefecture level city in Shandong
Fortune Shop	Green Intelligent Power Saver	It doesn't save any power at all, so I hope the old timers don't fall for this scam again	Jingdong prefecture level city in Shandong
Fortune Shop	Green Intelligent Power Saver	I've been using it for over a month now and haven't noticed any power savings.	Jingdong prefecture level city in Shandong
Fortune Shop	Green Intelligent Power Saver	It doesn't work at all. The electricity bill is higher when you use it.	Jingdong prefecture level city in Shandong
Fortune Shop	Green Intelligent Power Saver	It doesn't save any power, it's still as bad as ever.	Jingdong prefecture level city in Shandong
Fortune Shop	Green Intelligent Power Saver	Last night before leaving work to look at the table, and then plugged into the node device, this morning and then look at the table, the original save a few degrees of electricity, very good to have a power-saving effect, easy to use, satisfied with the shopping praise.	Jingdong prefecture level city in Shandong
Fortune Shop	Green Intelligent Power Saver	The product is real and effective, the electricity bill is reduced, really good, thanks to the merchants have so many good products, the product is real and effective, the electricity bill is reduced, want to save electricity friends quickly buy it, early use early to save money.	Jingdong prefecture level city in Shandong
Fortune Shop	Green Intelligent Power Saver	Saver saves time and money, baby appearance is beautiful, the quality of goods is good, and the same as the business description, good service attitude, packaging is complete, logistics is very fast, praise to	Jingdong prefecture level city in Shandong
Fortune Shop	Green Intelligent Power Saver	The effect is good, summer, home electricity is relatively large, buy and use a few days, feel the effect is very good, originally ten dollars a day electricity bill, now eight dollars a day, quite good	Jingdong prefecture level city in Shandong
Fortune Shop	Green Intelligent Power Saver	Indeed can save electricity and money, the first time I know this or from a friend's home to see, but also do not believe it, and now they use it to know that there is this good thing.	Jingdong prefecture level city in Shandong
Fortune Shop	Green Intelligent Power Saver	I have received the Smart Power Saver. Perfectly packaged. Fast delivery. Boss customer service attitude is also very good. It's worth buying. I plugged it in and tried it and it really saves more power than the original. I'll buy it again next time.	Jingdong prefecture level city in Shandong
Fortune Shop	Green Intelligent Power Saver	It's nice to save a dozen bucks a night on two air conditioners that used to take 30 or 40 degrees a night, but now it's only 10 or so degrees a night	Jingdong prefecture level city in Shandong
Fortune Shop	Green Intelligent Power Saver	Product no manufacturer's name no product qualification certificate, try two days to see the effect of	Jingdong prefecture level city in Shandong
Fortune Shop	Green Intelligent Power Saver	I've been using it for over a month and have saved over $80, so I've repurchased a few more back to save together	Jingdong prefecture level city in Shandong
Fortune Shop	Green Intelligent Power Saver	I haven't used it yet and will comment when I do, but it's well made.	Jingdong prefecture level city in Shandong
Fortune Shop	Green Intelligent Power Saver	It's quite energy efficient. It's really a boon for the home.	Jingdong prefecture level city in Shandong
Fortune Shop	Green Intelligent Power Saver	Energy-saving socket received a month, after using it for a month proved that it is indeed energy-saving, electricity bill saving, will buy it again, thank you!	Jingdong prefecture level city in Shandong
Fortune Shop	Green Intelligent Power Saver	There was a similar product many years ago. Now technology has evolved, buy one to try, according to the instructions in the box, it takes one or two dozen days to show results. Check your electric bill and see what happens.	Jingdong prefecture level city in Shandong
Fortune Shop	Green Intelligent Power Saver	Jingdong logistics is really fast, the same day the order arrived on the same day, more convenient than the supermarket, and a wide range of choices, the	Jingdong prefecture level city in Shandong
Fortune Shop	Green Intelligent Power Saver	I've been using this for a month or so and I can't see any results, it really is a scam as they say.	Jingdong prefecture level city in Shandong
Fortune Shop	Green Intelligent Power Saver	Very good use, a month can save a few degrees of power loss of electricity, introduced to the relatives to buy back to try.	Jingdong prefecture level city in Shandong
Fortune Shop	Green Intelligent Power Saver	It's really powerful! With this air conditioner can be turned on at any time without the pressure of paying the electricity bill! It's good for the people!	Jingdong prefecture level city in Shandong

Fortune Shop	Green Intelligent Power Saver	This business is a big ** products are not qualified, with a month of electricity more than one hundred percent of thirty, the purchase agreed to return the goods but do not receive a refund after receiving the goods, looking for the official side does not help, the Jingdong is a big **, I have never shopped in	Jingdong prefecture level city in Shandong
Fortune Shop	Green Intelligent Power Saver	It's good. I tried it. It saves power.	Jingdong prefecture level city in Shandong
Fortune Shop	Green Intelligent Power Saver	Nothing**, it seems like there's more	Jingdong prefecture level city in Shandong
Fortune Shop	Green Intelligent Power Saver	Power saving device received, well packaged, good quality, fast logistics	Jingdong prefecture level city in Shandong
Fortune Shop	Green Intelligent Power Saver	The service is okay, is that when I bought five, the desirable pieces are three, and then wait a few days and send me two replacement, I do not know if it is a mistake of the shipper, if I was not in the courier there to see, go home to say no, the	Jingdong prefecture level city in Shandong
Fortune Shop	Green Intelligent Power Saver	This thing will only cost me more if I use it, but before I used it, it was less than a ~ electricity a month, and after I used it, it was more than two hundred kWh a month.	Jingdong prefecture level city in Shandong
Fortune Shop	Green Intelligent Power Saver	I just used it. I don't know how it is.	Jingdong prefecture level city in Shandong
Fortune Shop	Green Intelligent Power Saver	I've received the product and I'm using it, so I'll see how it goes in a month!	Jingdong prefecture level city in Shandong
Fortune Shop	Green Intelligent Power Saver	The goods have just received the effect do not know how, please look forward to the results.	Jingdong prefecture level city in Shandong
Fortune Shop	Green Intelligent Power Saver	Power saving device, the material is very good practical and durable, the size is suitable, packaging is very good, the price is cheap, logistics is very fast!	Jingdong prefecture level city in Shandong
Fortune Shop	Green Intelligent Power Saver	Very nice, will comment in detail after using it for a while.	Jingdong prefecture level city in Shandong
Fortune Shop	Green Intelligent Power Saver	Good stuff to trust and support	Jingdong prefecture level city in Shandong
Fortune Shop	Green Intelligent Power Saver	I'll comment after I use it, doubt if it really saves power no	Jingdong prefecture level city in Shandong
Fortune Shop	Green Intelligent Power Saver	It's good quality and works well!	Jingdong prefecture level city in Shandong
Fortune Shop	Green Intelligent Power Saver	hahaha just right hahahahahaha hiccup hahahaha	Jingdong prefecture level city in Shandong
Fortune Shop	Green Intelligent Power Saver	The real thing matches the picture and looks great!	Jingdong prefecture level city in Shandong
Fortune Shop	Green Intelligent Power Saver	Plugged it in and we'll see when we pay the bills at the end of the year!	Jingdong prefecture level city in Shandong
Fortune Shop	Green Intelligent Power Saver	False advertising. It doesn't work.	Jingdong prefecture level city in Shandong
Fortune Shop	Green Intelligent Power Saver	Goods received, good effect	Jingdong prefecture level city in Shandong
Fortune Shop	Green Intelligent Power Saver	It looks okay, we'll see if it saves electricity when the time comes	Jingdong prefecture level city in Shandong
Fortune Shop	Green Intelligent Power Saver	Logistics is very fast quality is very good, the price is very affordable, accessories are also complete, installed, satisfied with good use	Jingdong prefecture level city in Shandong
Fortune Shop	Green Intelligent Power Saver	I've received the power saver. I'm looking forward to the savings.	Jingdong prefecture level city in Shandong
Fortune Shop	Green Intelligent Power Saver	Received, have not yet used, after use and then evaluate	Jingdong prefecture level city in Shandong
Fortune Shop	Green Intelligent Power Saver	I received the item, but I don't know if it saves power or not.	Jingdong prefecture level city in Shandong
Fortune Shop	Green Intelligent Power Saver	On Trial	Jingdong prefecture level city in Shandong
Fortune Shop	Green Intelligent Power Saver	Use it and see how it goes.	Jingdong prefecture level city in Shandong
Fortune Shop	Green Intelligent Power Saver	Quality is very good, no odor, fine workmanship, grain color are very like, satisfied!	Jingdong prefecture level city in Shandong
Fortune Shop	Green Intelligent Power Saver	I'll give you a good review. I don't know if it'll work.	Jingdong prefecture level city in Shandong
Fortune Shop	Green Intelligent Power Saver	The logistics is very fast and the package is intact. Good use of affordable. The effect is very good, is genuine	Jingdong prefecture level city in Shandong
Fortune Shop	Green Intelligent Power Saver	Nothing happens when you plug it in.	Jingdong prefecture level city in Shandong
Fortune Shop	Green Intelligent Power Saver	I don't know if it's as good as it sounds.	Jingdong prefecture level city in Shandong
Fortune Shop	Green Intelligent Power Saver	The quality of the stuff is very good, exactly the same as the seller's description, very satisfied!	Jingdong prefecture level city in Shandong
Fortune Shop	Green Intelligent Power Saver	The saver works great, it really works, it's affordable and worth buying.	Jingdong prefecture level city in Shandong
Fortune Shop	Green Intelligent Power Saver	Just used it for a couple days. I don't know yet.	Jingdong prefecture level city in Shandong
Fortune Shop	Green Intelligent Power Saver	The quality is quite good, cheaper than the physical store, good, next time will come again!	Jingdong prefecture level city in Shandong
Fortune Shop	Green Intelligent Power Saver	Good quality, cheap and affordable worth recommending to buy!	Jingdong prefecture level city in Shandong
Fortune Shop	Green Intelligent Power Saver	Very good quality, well made, works well! Satisfied	Jingdong prefecture level city in Shandong
Fortune Shop	Green Intelligent Power Saver	The logistics is very fast quality is very good very pleasant a shopping	Jingdong prefecture level city in Shandong
Fortune Shop	Green Intelligent Power Saver	Use it and then come back to evaluate!	Jingdong prefecture level city in Shandong
Fortune Shop	Green Intelligent Power Saver	# It's no use lying at all #	Jingdong prefecture level city in Shandong
Fortune Shop	Green Intelligent Power Saver	I can't get a voltage drop.	Jingdong prefecture level city in Shandong
Fortune Shop	Green Intelligent Power Saver	Evaluate after use	Jingdong prefecture level city in Shandong
Fortune Shop	Green Intelligent Power Saver	Still on trial.	Jingdong prefecture level city in Shandong
Fortune Shop	Green Intelligent Power Saver	ineffective	Jingdong prefecture level city in Shandong
Fortune Shop	Green Intelligent Power Saver	Satisfaction Shopping	Jingdong prefecture level city in Shandong
Fortune Shop	Green Intelligent Power Saver	Lies.	Jingdong prefecture level city in Shandong
Fortune Shop	Green Intelligent Power Saver	It's a real power saver.	Jingdong prefecture level city in Shandong
Fortune Shop	Green Intelligent Power Saver	in use	Jingdong prefecture level city in Shandong
Fortune Shop	Green Intelligent Power Saver	I don't know yet.	Jingdong prefecture level city in Shandong
Fortune Shop	Green Intelligent Power Saver	not care about anything	Jingdong prefecture level city in Shandong

Fortune Shop	Green Intelligent Power Saver	It doesn't seem to have much effect	Jingdong prefecture level city in Shandong
Fortune Shop	Green Intelligent Power Saver	Let the merchant see for himself, does the plug positive and negative terminals look like this?	Jingdong prefecture level city in Shandong
Fortune Shop	Green Intelligent Power Saver	It doesn't work well, the electricity bill is less before using it, but more after using it, contacting customer service, the service is still not good, and returning it is slow	Jingdong prefecture level city in Shandong
Fortune Shop	Green Intelligent Power Saver	With the advertisement product does not match, above the effective negative, power saving rate of two rows of words it, the product looks very thin, with 3 days to see the meter number is still with the previous no difference, which is obviously fraudulent behavior!	Jingdong prefecture level city in Shandong
Fortune Shop	Green Intelligent Power Saver	Doesn't save any power at all. Not much use.	Jingdong prefecture level city in Shandong
Fortune Shop	Green Intelligent Power Saver	The plugs are all loose, they're all the same length and they don't work at all	Jingdong prefecture level city in Shandong
Fortune Shop	Green Intelligent Power Saver	It's bad 👎 it's not effective at all, you'd be crazy to sell this garbage on the internet.	Jingdong prefecture level city in Shandong
Fortune Shop	Green Intelligent Power Saver	It doesn't save any power at all, so I hope the old timers don't fall for this scam again	Jingdong prefecture level city in Shandong
Fortune Shop	Green Intelligent Power Saver	I've been using it for over a month now and haven't noticed any power savings.	Jingdong prefecture level city in Shandong
Fortune Shop	Green Intelligent Power Saver	It doesn't work at all. The electricity bill is higher when you use it.	Jingdong prefecture level city in Shandong
Fortune Shop	Green Intelligent Power Saver	It doesn't save any power, it's still as bad as ever.	Jingdong prefecture level city in Shandong
Fortune Shop	Green Intelligent Power Saver	Last night before leaving work to look at the table, and then plugged into the node device, this morning and then look at the table, than the original save a few degrees of electricity, very good to have a power-saving effect, easy to use, satisfied with the shopping praise.	Jingdong prefecture level city in Shandong
Fortune Shop	Green Intelligent Power Saver	The product is real and effective, the electricity bill is reduced, really good, thanks to the merchants have so many good products, the product is real and effective, the electricity bill is reduced, want to save electricity friends quickly buy it, early use early to save money.	Jingdong prefecture level city in Shandong
Fortune Shop	Green Intelligent Power Saver	Saver saves time and money, baby appearance is beautiful, the quality of goods is good, and the same as the business description, good service attitude, packaging is complete, logistics is very fast, praise to	Jingdong prefecture level city in Shandong
Fortune Shop	Green Intelligent Power Saver	The effect is good, summer, home electricity is relatively large, buy and use a few days, feel the effect is very good, originally ten dollars a day electricity bill, now eight dollars a day, quite good	Jingdong prefecture level city in Shandong
Fortune Shop	Green Intelligent Power Saver	Indeed can save electricity and money, the first time I know this or from a friend's home to see, but also do not believe it, and now they use it to know that there is this good thing.	Jingdong prefecture level city in Shandong
Fortune Shop	Green Intelligent Power Saver	I have received the Smart Power Saver. Perfectly packaged. Fast delivery. Boss customer service attitude is also very good. It's worth buying. I plugged it in and tried it and it really saves more power than the original. I'll buy it again next time.	Jingdong prefecture level city in Shandong
Fortune Shop	Green Intelligent Power Saver	It's nice to save a dozen bucks a night on two air conditioners that used to take 30 or 40 degrees a night, but now it's only 10 or so degrees a night	Jingdong prefecture level city in Shandong
Fortune Shop	Green Intelligent Power Saver	Product no manufacturer's name no product qualification certificate, try two days to see the effect of	Jingdong prefecture level city in Shandong
Fortune Shop	Green Intelligent Power Saver	I've been using it for over a month and have saved over $80, so I've repurchased a few more back to save together	Jingdong prefecture level city in Shandong
Fortune Shop	Green Intelligent Power Saver	I haven't used it yet and will comment when I do, but it's well made.	Jingdong prefecture level city in Shandong
Fortune Shop	Green Intelligent Power Saver	It's quite energy efficient. It's really a boon for the home.	Jingdong prefecture level city in Shandong
Fortune Shop	Green Intelligent Power Saver	Energy-saving socket received a month, after using it for a month proved that it is indeed energy-saving, electricity bill saving, will buy it again, thank you!	Jingdong prefecture level city in Shandong
Fortune Shop	Green Intelligent Power Saver	There was a similar product many years ago. Now technology has evolved, buy one to try, according to the instructions in the box, it takes one or two dozen days to show results. Check your electric bill and see what happens.	Jingdong prefecture level city in Shandong
Fortune Shop	Green Intelligent Power Saver	Jingdong logistics is really fast, the same day the order arrived on the same day, more convenient than the supermarket, and a wide range of choices, the	Jingdong prefecture level city in Shandong
Fortune Shop	Green Intelligent Power Saver	I've been using this for a month or so and I can't see any results, it really is a scam as they say.	Jingdong prefecture level city in Shandong
Fortune Shop	Green Intelligent Power Saver	Very good use, a month can save a few degrees of power loss of electricity, introduced to the relatives to buy back to try.	Jingdong prefecture level city in Shandong
Fortune Shop	Green Intelligent Power Saver	It's really powerful! With this air conditioner can be turned on at any time without the pressure of paying the electricity bill! It's good for the people!	Jingdong prefecture level city in Shandong
Fortune Shop	Green Intelligent Power Saver	This business is a big ** products are not qualified, with a month of electricity more than one hundred percent of thirty, the purchase agreed to return the goods but do not receive a refund after receiving the goods, looking for the official side does not help, the Jingdong is a big **, I have never shopped in	Jingdong prefecture level city in Shandong
Fortune Shop	Green Intelligent Power Saver	It's good. I tried it. It saves power.	Jingdong prefecture level city in Shandong
Fortune Shop	Green Intelligent Power Saver	Nothing**, it seems like there's more	Jingdong prefecture level city in Shandong
Fortune Shop	Green Intelligent Power Saver	Power saving device received, well packaged, good quality, fast logistics	Jingdong prefecture level city in Shandong
Fortune Shop	Green Intelligent Power Saver	The service is okay, is that when I bought five, the desirable pieces are three, and then wait a few days and send me two replacement, I do not know if it is a mistake of the shipper, if I was not in the courier there to see, go home to say no, the	Jingdong prefecture level city in Shandong
Fortune Shop	Green Intelligent Power Saver	This thing will only cost me more if I use it, but before I used it, it was less than a ~ electricity a month, and after I used it, it was more than two hundred kWh a month.	Jingdong prefecture level city in Shandong
Fortune Shop	Green Intelligent Power Saver	I just used it. I don't know how it is.	Jingdong prefecture level city in Shandong
Fortune Shop	Green Intelligent Power Saver	I've received the product and I'm using it, so I'll see how it goes in a month!	Jingdong prefecture level city in Shandong
Fortune Shop	Green Intelligent Power Saver	The goods have just received the effect do not know how, please look forward to the results.	Jingdong prefecture level city in Shandong
Fortune Shop	Green Intelligent Power Saver	Power saving device, the material is very good practical and durable, the size is suitable, packaging is very good, the price is cheap, logistics is very fast!	Jingdong prefecture level city in Shandong
Fortune Shop	Green Intelligent Power Saver	Very nice, will comment in detail after using it for a while.	Jingdong prefecture level city in Shandong
Fortune Shop	Green Intelligent Power Saver	Good stuff to trust and support	Jingdong prefecture level city in Shandong
Fortune Shop	Green Intelligent Power Saver	I'll comment after I use it, doubt if it really saves power no	Jingdong prefecture level city in Shandong
Fortune Shop	Green Intelligent Power Saver	It's good quality and works well!	Jingdong prefecture level city in Shandong
Fortune Shop	Green Intelligent Power Saver	hahaha just right hahahahahaha hiccup hahahaha	Jingdong prefecture level city in Shandong
Fortune Shop	Green Intelligent Power Saver	The real thing matches the picture and looks great!	Jingdong prefecture level city in Shandong
Fortune Shop	Green Intelligent Power Saver	Plugged it in and we'll see when we pay the bills at the end of the year!	Jingdong prefecture level city in Shandong
Fortune Shop	Green Intelligent Power Saver	False advertising. It doesn't work.	Jingdong prefecture level city in Shandong
Fortune Shop	Green Intelligent Power Saver	Goods received, good effect	Jingdong prefecture level city in Shandong
Fortune Shop	Green Intelligent Power Saver	It looks okay, we'll see if it saves electricity when the time comes	Jingdong prefecture level city in Shandong

Fortune Shop	Green Intelligent Power Saver	Logistics is very fast quality is very good, the price is very affordable, accessories are also complete, installed, satisfied with good use	Jingdong prefecture level city in Shandong
Fortune Shop	Green Intelligent Power Saver	I've received the power saver. I'm looking forward to the savings.	Jingdong prefecture level city in Shandong
Fortune Shop	Green Intelligent Power Saver	Received, have not yet used, after use and then evaluate	Jingdong prefecture level city in Shandong
Fortune Shop	Green Intelligent Power Saver	I received the item, but I don't know if it saves power or not.	Jingdong prefecture level city in Shandong
Fortune Shop	Green Intelligent Power Saver	On Trial	Jingdong prefecture level city in Shandong
Fortune Shop	Green Intelligent Power Saver	Use it and see how it goes.	Jingdong prefecture level city in Shandong
Fortune Shop	Green Intelligent Power Saver	Quality is very good, no odor, fine workmanship, grain color are very like, satisfied!	Jingdong prefecture level city in Shandong
Fortune Shop	Green Intelligent Power Saver	I'll give you a good review. I don't know if it'll work.	Jingdong prefecture level city in Shandong
Fortune Shop	Green Intelligent Power Saver	The logistics is very fast and the package is intact. Good use of affordable. The effect is very good, is genuine	Jingdong prefecture level city in Shandong
Fortune Shop	Green Intelligent Power Saver	Nothing happens when you plug it in.	Jingdong prefecture level city in Shandong
Fortune Shop	Green Intelligent Power Saver	I don't know if it's as good as it sounds.	Jingdong prefecture level city in Shandong
Fortune Shop	Green Intelligent Power Saver	The quality of the stuff is very good, exactly the same as the seller's description, very satisfied!	Jingdong prefecture level city in Shandong
Fortune Shop	Green Intelligent Power Saver	The saver works great, it really works, it's affordable and worth buying.	Jingdong prefecture level city in Shandong
Fortune Shop	Green Intelligent Power Saver	Just used it for a couple days. I don't know yet.	Jingdong prefecture level city in Shandong
Fortune Shop	Green Intelligent Power Saver	The quality is quite good, cheaper than the physical store, good, next time will come again!	Jingdong prefecture level city in Shandong
Fortune Shop	Green Intelligent Power Saver	Good quality, cheap and affordable worth recommending to buy!	Jingdong prefecture level city in Shandong
Fortune Shop	Green Intelligent Power Saver	Very good quality, well made, works well! Satisfied	Jingdong prefecture level city in Shandong
Fortune Shop	Green Intelligent Power Saver	The logistics is very fast quality is very good very pleasant a shopping	Jingdong prefecture level city in Shandong
Fortune Shop	Green Intelligent Power Saver	Use it and then come back to evaluate!	Jingdong prefecture level city in Shandong
Fortune Shop	Green Intelligent Power Saver	# It's no use lying at all #	Jingdong prefecture level city in Shandong
Fortune Shop	Green Intelligent Power Saver	I can't get a voltage drop.	Jingdong prefecture level city in Shandong
Fortune Shop	Green Intelligent Power Saver	Evaluate after use	Jingdong prefecture level city in Shandong
Fortune Shop	Green Intelligent Power Saver	Still on trial.	Jingdong prefecture level city in Shandong
Fortune Shop	Green Intelligent Power Saver	ineffective	Jingdong prefecture level city in Shandong
Fortune Shop	Green Intelligent Power Saver	Satisfaction Shopping	Jingdong prefecture level city in Shandong
Fortune Shop	Green Intelligent Power Saver	Lies.	Jingdong prefecture level city in Shandong
Fortune Shop	Green Intelligent Power Saver	It's a real power saver.	Jingdong prefecture level city in Shandong
Fortune Shop	Green Intelligent Power Saver	in use	Jingdong prefecture level city in Shandong
Fortune Shop	Green Intelligent Power Saver	I don't know yet.	Jingdong prefecture level city in Shandong
Fortune Shop	Green Intelligent Power Saver	not care about anything	Jingdong prefecture level city in Shandong
Fortune Shop	Green Intelligent Power Saver	It doesn't seem to have much effect	Jingdong prefecture level city in Shandong
Fortune Shop	Green Intelligent Power Saver	Let the merchant see for himself, does the plug positive and negative terminals look like this?	Jingdong prefecture level city in Shandong
Fortune Shop	Green Intelligent Power Saver	It doesn't work well, the electricity bill is less before using it, but more after using it, contacting customer service, the service is still not good, and returning it is slow	Jingdong prefecture level city in Shandong
Fortune Shop	Green Intelligent Power Saver	With the advertisement product does not match, above the effective negative, power saving rate of two rows of words it, the product looks very thin, with 3 days to see the meter number is still with the previous no difference, which is obviously fraudulent behavior!	Jingdong prefecture level city in Shandong
Fortune Shop	Green Intelligent Power Saver	Doesn't save any power at all. Not much use.	Jingdong prefecture level city in Shandong
Fortune Shop	Green Intelligent Power Saver	The plugs are all loose, they're all the same length and they don't work at all	Jingdong prefecture level city in Shandong
Fortune Shop	Green Intelligent Power Saver	It's bad 👎 it's not effective at all, you'd be crazy to sell this garbage on the internet.	Jingdong prefecture level city in Shandong
Fortune Shop	Green Intelligent Power Saver	It doesn't save any power at all, so I hope the old timers don't fall for this scam again	Jingdong prefecture level city in Shandong
Fortune Shop	Green Intelligent Power Saver	I've been using it for over a month now and haven't noticed any power savings.	Jingdong prefecture level city in Shandong
Fortune Shop	Green Intelligent Power Saver	It doesn't work at all. The electricity bill is higher when you use it.	Jingdong prefecture level city in Shandong
Fortune Shop	Green Intelligent Power Saver	It doesn't save any power, it's still as bad as ever.	Jingdong prefecture level city in Shandong
Fortune Shop	Green Intelligent Power Saver	Last night before leaving work to look at the table, and then plugged into the node device, this morning and then look at the table, than the original save a few degrees of electricity, very good to have a power-saving effect, easy to use, satisfied with the shopping praise.	Jingdong prefecture level city in Shandong
Fortune Shop	Green Intelligent Power Saver	The product is real and effective, the electricity bill is reduced, really good, thanks to the merchants have so many good products, the product is real and effective, the electricity bill is reduced, want to save electricity friends quickly buy it, early use early to save money.	Jingdong prefecture level city in Shandong
Fortune Shop	Green Intelligent Power Saver	Saver saves time and money, baby appearance is beautiful, the quality of goods is good, and the same as the business description, good service attitude, packaging is complete, logistics is very fast, praise to	Jingdong prefecture level city in Shandong
Fortune Shop	Green Intelligent Power Saver	The effect is good, summer, home electricity is relatively large, buy and use a few days, feel the effect is very good, originally ten dollars a day electricity bill, now eight dollars a day, quite good	Jingdong prefecture level city in Shandong
Fortune Shop	Green Intelligent Power Saver	Indeed can save electricity and money, the first time I know this or from a friend's home to see, but also do not believe it, and now they use it to know that there is this good thing.	Jingdong prefecture level city in Shandong
Fortune Shop	Green Intelligent Power Saver	I have received the Smart Power Saver. Perfectly packaged. Fast delivery. Boss customer service attitude is also very good. It's worth buying. I plugged it in and tried it and it really saves more power than the original. I'll buy it again next time.	Jingdong prefecture level city in Shandong
Fortune Shop	Green Intelligent Power Saver	It's nice to save a dozen bucks a night on two air conditioners that used to take 30 or 40 degrees a night, but now it's only 10 or so degrees a night	Jingdong prefecture level city in Shandong
Fortune Shop	Green Intelligent Power Saver	Product no manufacturer's name no product qualification certificate, try two days to see the effect of	Jingdong prefecture level city in Shandong

Fortune Shop	Green Intelligent Power Saver	I've been using it for over a month and have saved over $80, so I've repurchased a few more back to save together	Jingdong prefecture level city in Shandong
Fortune Shop	Green Intelligent Power Saver	I haven't used it yet and will comment when I do, but it's well made.	Jingdong prefecture level city in Shandong
Fortune Shop	Green Intelligent Power Saver	It's quite energy efficient. It's really a boon for the home.	Jingdong prefecture level city in Shandong
Fortune Shop	Green Intelligent Power Saver	Energy-saving socket received a month, after using it for a month proved that it is indeed energy-saving, electricity bill saving, will buy it again, thank you!	Jingdong prefecture level city in Shandong
Fortune Shop	Green Intelligent Power Saver	There was a similar product many years ago. Now technology has evolved, buy one to try, according to the instructions in the box, it takes one or two dozen days to show results. Check your electric bill and see what happens.	Jingdong prefecture level city in Shandong
Fortune Shop	Green Intelligent Power Saver	Jingdong logistics is really fast, the same day the order arrived on the same day, more convenient than the supermarket, and a wide range of choices, the	Jingdong prefecture level city in Shandong
Fortune Shop	Green Intelligent Power Saver	I've been using this for a month or so and I can't see any results, it really is a scam as they say.	Jingdong prefecture level city in Shandong
Fortune Shop	Green Intelligent Power Saver	Very good use, a month can save a few degrees of power loss of electricity, introduced to the relatives to buy back to try.	Jingdong prefecture level city in Shandong
Fortune Shop	Green Intelligent Power Saver	It's really powerful! With this air conditioner can be turned on at any time without the pressure of paying the electricity bill! It's good for the people!	Jingdong prefecture level city in Shandong
Fortune Shop	Green Intelligent Power Saver	This business is a big ** products are not qualified, with a month of electricity more than one hundred percent of thirty, the purchase agreed to return the goods but do not receive a refund after receiving the goods, looking for the official side does not help, the Jingdong is a big **, I have never shopped in	Jingdong prefecture level city in Shandong
Fortune Shop	Green Intelligent Power Saver	It's good. I tried it. It saves power.	Jingdong prefecture level city in Shandong
Fortune Shop	Green Intelligent Power Saver	Nothing**, it seems like there's more	Jingdong prefecture level city in Shandong
Fortune Shop	Green Intelligent Power Saver	Power saving device received, well packaged, good quality, fast logistics	Jingdong prefecture level city in Shandong
Fortune Shop	Green Intelligent Power Saver	The service is okay, is that when I bought five, the desirable pieces are three, and then wait a few days and send me two replacement, I do not know if it is a mistake of the shipper, if I was not in the courier there to see, go home to say no, the	Jingdong prefecture level city in Shandong
Fortune Shop	Green Intelligent Power Saver	This thing will only cost me more if I use it, but before I used it, it was less than a ~ electricity a month, and after I used it, it was more than two hundred kWh a month.	Jingdong prefecture level city in Shandong
Fortune Shop	Green Intelligent Power Saver	I just used it. I don't know how it is.	Jingdong prefecture level city in Shandong
Fortune Shop	Green Intelligent Power Saver	I've received the product and I'm using it, so I'll see how it goes in a month!	Jingdong prefecture level city in Shandong
Fortune Shop	Green Intelligent Power Saver	The goods have just received the effect do not know how, please look forward to the results.	Jingdong prefecture level city in Shandong
Fortune Shop	Green Intelligent Power Saver	Power saving device, the material is very good practical and durable, the size is suitable, packaging is very good, the price is cheap, logistics is very fast!	Jingdong prefecture level city in Shandong
Fortune Shop	Green Intelligent Power Saver	Very nice, will comment in detail after using it for a while.	Jingdong prefecture level city in Shandong
Fortune Shop	Green Intelligent Power Saver	Good stuff to trust and support	Jingdong prefecture level city in Shandong
Fortune Shop	Green Intelligent Power Saver	I'll comment after I use it, doubt if it really saves power no	Jingdong prefecture level city in Shandong
Fortune Shop	Green Intelligent Power Saver	It's good quality and works well!	Jingdong prefecture level city in Shandong
Fortune Shop	Green Intelligent Power Saver	hahaha just right hahahahahaha hiccup hahahaha	Jingdong prefecture level city in Shandong
Fortune Shop	Green Intelligent Power Saver	The real thing matches the picture and looks great!	Jingdong prefecture level city in Shandong
Fortune Shop	Green Intelligent Power Saver	Plugged it in and we'll see when we pay the bills at the end of the year!	Jingdong prefecture level city in Shandong
Fortune Shop	Green Intelligent Power Saver	False advertising. It doesn't work.	Jingdong prefecture level city in Shandong
Fortune Shop	Green Intelligent Power Saver	Goods received, good effect	Jingdong prefecture level city in Shandong
Fortune Shop	Green Intelligent Power Saver	It looks okay, we'll see if it saves electricity when the time comes	Jingdong prefecture level city in Shandong
Fortune Shop	Green Intelligent Power Saver	Logistics is very fast quality is very good, the price is very affordable, accessories are also complete, installed, satisfied with good use	Jingdong prefecture level city in Shandong
Fortune Shop	Green Intelligent Power Saver	I've received the power saver. I'm looking forward to the savings.	Jingdong prefecture level city in Shandong
Fortune Shop	Green Intelligent Power Saver	Received, have not yet used, after use and then evaluate	Jingdong prefecture level city in Shandong
Fortune Shop	Green Intelligent Power Saver	I received the item, but I don't know if it saves power or not.	Jingdong prefecture level city in Shandong
Fortune Shop	Green Intelligent Power Saver	On Trial	Jingdong prefecture level city in Shandong
Fortune Shop	Green Intelligent Power Saver	Use it and see how it goes.	Jingdong prefecture level city in Shandong
Fortune Shop	Green Intelligent Power Saver	Quality is very good, no odor, fine workmanship, grain color are very like, satisfied!	Jingdong prefecture level city in Shandong
Fortune Shop	Green Intelligent Power Saver	I'll give you a good review. I don't know if it'll work.	Jingdong prefecture level city in Shandong
Fortune Shop	Green Intelligent Power Saver	The logistics is very fast and the package is intact. Good use of affordable. The effect is very good, is genuine	Jingdong prefecture level city in Shandong
Fortune Shop	Green Intelligent Power Saver	Nothing happens when you plug it in.	Jingdong prefecture level city in Shandong
Fortune Shop	Green Intelligent Power Saver	I don't know if it's as good as it sounds.	Jingdong prefecture level city in Shandong
Fortune Shop	Green Intelligent Power Saver	The quality of the stuff is very good, exactly the same as the seller's description, very satisfied!	Jingdong prefecture level city in Shandong
Fortune Shop	Green Intelligent Power Saver	The saver works great, it really works, it's affordable and worth buying.	Jingdong prefecture level city in Shandong
Fortune Shop	Green Intelligent Power Saver	Just used it for a couple days. I don't know yet.	Jingdong prefecture level city in Shandong
Fortune Shop	Green Intelligent Power Saver	The quality is quite good, cheaper than the physical store, good, next time will come again!	Jingdong prefecture level city in Shandong
Fortune Shop	Green Intelligent Power Saver	Good quality, cheap and affordable worth recommending to buy!	Jingdong prefecture level city in Shandong
Fortune Shop	Green Intelligent Power Saver	Very good quality, well made, works well! Satisfied	Jingdong prefecture level city in Shandong
Fortune Shop	Green Intelligent Power Saver	The logistics is very fast quality is very good very pleasant a shopping	Jingdong prefecture level city in Shandong
Fortune Shop	Green Intelligent Power Saver	Use it and then come back to evaluate!	Jingdong prefecture level city in Shandong
Fortune Shop	Green Intelligent Power Saver	# It's no use lying at all #	Jingdong prefecture level city in Shandong
Fortune Shop	Green Intelligent Power Saver	I can't get a voltage drop.	Jingdong prefecture level city in Shandong

Fortune Shop	Green Intelligent Power Saver	Evaluate after use	Jingdong prefecture level city in Shandong
Fortune Shop	Green Intelligent Power Saver	Still on trial.	Jingdong prefecture level city in Shandong
Fortune Shop	Green Intelligent Power Saver	ineffective	Jingdong prefecture level city in Shandong
Fortune Shop	Green Intelligent Power Saver	Satisfaction Shopping	Jingdong prefecture level city in Shandong
Fortune Shop	Green Intelligent Power Saver	Lies.	Jingdong prefecture level city in Shandong
Fortune Shop	Green Intelligent Power Saver	It's a real power saver.	Jingdong prefecture level city in Shandong
Fortune Shop	Green Intelligent Power Saver	in use	Jingdong prefecture level city in Shandong
Fortune Shop	Green Intelligent Power Saver	I don't know yet.	Jingdong prefecture level city in Shandong
Fortune Shop	Green Intelligent Power Saver	not care about anything	Jingdong prefecture level city in Shandong
Fortune Shop	Green Intelligent Power Saver	It doesn't seem to have much effect	Jingdong prefecture level city in Shandong
Fortune Shop	Green Intelligent Power Saver	Let the merchant see for himself, does the plug positive and negative terminals look like this?	Jingdong prefecture level city in Shandong
Fortune Shop	Green Intelligent Power Saver	It doesn't work well, the electricity bill is less before using it, but more after using it, contacting customer service, the service is still not good, and returning it is slow	Jingdong prefecture level city in Shandong
Fortune Shop	Green Intelligent Power Saver	With the advertisement product does not match, above the effective negative, power saving rate of two rows of words it, the product looks very thin, with 3 days to see the meter number is still with the previous no difference, which is obviously fraudulent behavior!	Jingdong prefecture level city in Shandong
Fortune Shop	Green Intelligent Power Saver	Doesn't save any power at all. Not much use.	Jingdong prefecture level city in Shandong
Fortune Shop	Green Intelligent Power Saver	The plugs are all loose, they're all the same length and they don't work at all	Jingdong prefecture level city in Shandong
Fortune Shop	Green Intelligent Power Saver	It's bad 👎 it's not effective at all, you'd be crazy to sell this garbage on the internet.	Jingdong prefecture level city in Shandong
Fortune Shop	Green Intelligent Power Saver	It doesn't save any power at all, so I hope the old timers don't fall for this scam again	Jingdong prefecture level city in Shandong
Fortune Shop	Green Intelligent Power Saver	I've been using it for over a month now and haven't noticed any power savings.	Jingdong prefecture level city in Shandong
Fortune Shop	Green Intelligent Power Saver	It doesn't work at all. The electricity bill is higher when you use it.	Jingdong prefecture level city in Shandong
Fortune Shop	Green Intelligent Power Saver	It doesn't save any power, it's still as bad as ever.	Jingdong prefecture level city in Shandong
Fortune Shop	Green Intelligent Power Saver	Last night before leaving work to look at the table, and then plugged into the node device, this morning and then look at the table, than the original save a few degrees of electricity, very good to have a power-saving effect, easy to use, satisfied with the shopping praise.	Jingdong prefecture level city in Shandong
Fortune Shop	Green Intelligent Power Saver	The product is real and effective, the electricity bill is reduced, really good, thanks to the merchants have so many good products, the product is real and effective, the electricity bill is reduced, want to save electricity friends quickly buy it, early use early to save money.	Jingdong prefecture level city in Shandong
Fortune Shop	Green Intelligent Power Saver	Saver saves time and money, baby appearance is beautiful, the quality of goods is good, and the same as the business description, good service attitude, packaging is complete, logistics is very fast, praise to	Jingdong prefecture level city in Shandong
Fortune Shop	Green Intelligent Power Saver	The effect is good, summer, home electricity is relatively large, buy and use a few days, feel the effect is very good, originally ten dollars a day electricity bill, now eight dollars a day, quite good	Jingdong prefecture level city in Shandong
Fortune Shop	Green Intelligent Power Saver	Indeed can save electricity and money, the first time I know this or from a friend's home to see, but also do not believe it, and now they use it to know that there is this good thing.	Jingdong prefecture level city in Shandong
Fortune Shop	Green Intelligent Power Saver	I have received the Smart Power Saver. Perfectly packaged. Fast delivery. Boss customer service attitude is also very good. It's worth buying. I plugged it in and tried it and it really saves more power than the original. I'll buy it again next time.	Jingdong prefecture level city in Shandong
Fortune Shop	Green Intelligent Power Saver	It's nice to save a dozen bucks a night on two air conditioners that used to take 30 or 40 degrees a night, but now it's only 10 or so degrees a night	Jingdong prefecture level city in Shandong
Fortune Shop	Green Intelligent Power Saver	Product no manufacturer's name no product qualification certificate, try two days to see the effect of	Jingdong prefecture level city in Shandong
Fortune Shop	Green Intelligent Power Saver	I've been using it for over a month and have saved over $80, so I've repurchased a few more back to save together	Jingdong prefecture level city in Shandong
Fortune Shop	Green Intelligent Power Saver	I haven't used it yet and will comment when I do, but it's well made.	Jingdong prefecture level city in Shandong
Fortune Shop	Green Intelligent Power Saver	It's quite energy efficient. It's really a boon for the home.	Jingdong prefecture level city in Shandong
Fortune Shop	Green Intelligent Power Saver	Energy-saving socket received a month, after using it for a month proved that it is indeed energy-saving, electricity bill saving, will buy it again, thank you!	Jingdong prefecture level city in Shandong
Fortune Shop	Green Intelligent Power Saver	There was a similar product many years ago. Now technology has evolved, buy one to try, according to the instructions in the box, it takes one or two dozen days to show results. Check your electric bill and see what happens.	Jingdong prefecture level city in Shandong
Fortune Shop	Green Intelligent Power Saver	Jingdong logistics is really fast, the same day the order arrived on the same day, more convenient than the supermarket, and a wide range of choices, the	Jingdong prefecture level city in Shandong
Fortune Shop	Green Intelligent Power Saver	I've been using this for a month or so and I can't see any results, it really is a scam as they say.	Jingdong prefecture level city in Shandong
Fortune Shop	Green Intelligent Power Saver	Very good use, a month can save a few degrees of power loss of electricity, introduced to the relatives to buy back to try.	Jingdong prefecture level city in Shandong
Fortune Shop	Green Intelligent Power Saver	It's really powerful! With this air conditioner can be turned on at any time without the pressure of paying the electricity bill! It's good for the people!	Jingdong prefecture level city in Shandong
Fortune Shop	Green Intelligent Power Saver	This business is a big ** products are not qualified, with a month of electricity more than one hundred percent of thirty, the purchase agreed to return the goods but do not receive a refund after receiving the goods, looking for the official side does not help, the Jingdong is a big **, I have never shopped in	Jingdong prefecture level city in Shandong
Fortune Shop	Green Intelligent Power Saver	It's good. I tried it. It saves power.	Jingdong prefecture level city in Shandong
Fortune Shop	Green Intelligent Power Saver	Nothing**, it seems like there's more	Jingdong prefecture level city in Shandong
Fortune Shop	Green Intelligent Power Saver	Power saving device received, well packaged, good quality, fast logistics	Jingdong prefecture level city in Shandong
Fortune Shop	Green Intelligent Power Saver	The service is okay, is that when I bought five, the desirable pieces are three, and then wait a few days and send me two replacement, I do not know if it is a mistake of the shipper, if I was not in the courier there to see, go home to say no, the	Jingdong prefecture level city in Shandong
Fortune Shop	Green Intelligent Power Saver	This thing will only cost me more if I use it, but before I used it, it was less than a ~ electricity a month, and after I used it, it was more than two hundred kWh a month.	Jingdong prefecture level city in Shandong
Fortune Shop	Green Intelligent Power Saver	I just used it. I don't know how it is.	Jingdong prefecture level city in Shandong
Fortune Shop	Green Intelligent Power Saver	I've received the product and I'm using it, so I'll see how it goes in a month!	Jingdong prefecture level city in Shandong
Fortune Shop	Green Intelligent Power Saver	The goods have just received the effect do not know how, please look forward to the results.	Jingdong prefecture level city in Shandong
Fortune Shop	Green Intelligent Power Saver	Power saving device, the material is very good practical and durable, the size is suitable, packaging is very good, the price is cheap, logistics is very fast!	Jingdong prefecture level city in Shandong
Fortune Shop	Green Intelligent Power Saver	Very nice, will comment in detail after using it for a while.	Jingdong prefecture level city in Shandong

Fortune Shop	Green Intelligent Power Saver	Good stuff to trust and support	Jingdong prefecture level city in Shandong
Fortune Shop	Green Intelligent Power Saver	I'll comment after I use it, doubt if it really saves power no	Jingdong prefecture level city in Shandong
Fortune Shop	Green Intelligent Power Saver	It's good quality and works well!	Jingdong prefecture level city in Shandong
Fortune Shop	Green Intelligent Power Saver	hahaha just right hahahahahaha hiccup hahahaha	Jingdong prefecture level city in Shandong
Fortune Shop	Green Intelligent Power Saver	The real thing matches the picture and looks great!	Jingdong prefecture level city in Shandong
Fortune Shop	Green Intelligent Power Saver	Plugged it in and we'll see when we pay the bills at the end of the year!	Jingdong prefecture level city in Shandong
Fortune Shop	Green Intelligent Power Saver	False advertising. It doesn't work.	Jingdong prefecture level city in Shandong
Fortune Shop	Green Intelligent Power Saver	Goods received, good effect	Jingdong prefecture level city in Shandong
Fortune Shop	Green Intelligent Power Saver	It looks okay, we'll see if it saves electricity when the time comes	Jingdong prefecture level city in Shandong
Fortune Shop	Green Intelligent Power Saver	Logistics is very fast quality is very good, the price is very affordable, accessories are also complete, installed, satisfied with good use	Jingdong prefecture level city in Shandong
Fortune Shop	Green Intelligent Power Saver	I've received the power saver. I'm looking forward to the savings.	Jingdong prefecture level city in Shandong
Fortune Shop	Green Intelligent Power Saver	Received, have not yet used, after use and then evaluate	Jingdong prefecture level city in Shandong
Fortune Shop	Green Intelligent Power Saver	I received the item, but I don't know if it saves power or not.	Jingdong prefecture level city in Shandong
Fortune Shop	Green Intelligent Power Saver	On Trial	Jingdong prefecture level city in Shandong
Fortune Shop	Green Intelligent Power Saver	Use it and see how it goes.	Jingdong prefecture level city in Shandong
Fortune Shop	Green Intelligent Power Saver	Quality is very good, no odor, fine workmanship, grain color are very like, satisfied!	Jingdong prefecture level city in Shandong
Fortune Shop	Green Intelligent Power Saver	I'll give you a good review. I don't know if it'll work.	Jingdong prefecture level city in Shandong
Fortune Shop	Green Intelligent Power Saver	The logistics is very fast and the package is intact. Good use of affordable. The effect is very good, is genuine	Jingdong prefecture level city in Shandong
Fortune Shop	Green Intelligent Power Saver	Nothing happens when you plug it in.	Jingdong prefecture level city in Shandong
Fortune Shop	Green Intelligent Power Saver	I don't know if it's as good as it sounds.	Jingdong prefecture level city in Shandong
Fortune Shop	Green Intelligent Power Saver	The quality of the stuff is very good, exactly the same as the seller's description, very satisfied!	Jingdong prefecture level city in Shandong
Fortune Shop	Green Intelligent Power Saver	The saver works great, it really works, it's affordable and worth buying.	Jingdong prefecture level city in Shandong
Fortune Shop	Green Intelligent Power Saver	Just used it for a couple days. I don't know yet.	Jingdong prefecture level city in Shandong
Fortune Shop	Green Intelligent Power Saver	The quality is quite good, cheaper than the physical store, good, next time will come again!	Jingdong prefecture level city in Shandong
Fortune Shop	Green Intelligent Power Saver	Good quality, cheap and affordable worth recommending to buy!	Jingdong prefecture level city in Shandong
Fortune Shop	Green Intelligent Power Saver	Very good quality, well made, works well! Satisfied	Jingdong prefecture level city in Shandong
Fortune Shop	Green Intelligent Power Saver	The logistics is very fast quality is very good very pleasant a shopping	Jingdong prefecture level city in Shandong
Fortune Shop	Green Intelligent Power Saver	Use it and then come back to evaluate!	Jingdong prefecture level city in Shandong
Fortune Shop	Green Intelligent Power Saver	# It's no use lying at all #	Jingdong prefecture level city in Shandong
Fortune Shop	Green Intelligent Power Saver	I can't get a voltage drop.	Jingdong prefecture level city in Shandong
Fortune Shop	Green Intelligent Power Saver	Evaluate after use	Jingdong prefecture level city in Shandong
Fortune Shop	Green Intelligent Power Saver	Still on trial.	Jingdong prefecture level city in Shandong
Fortune Shop	Green Intelligent Power Saver	ineffective	Jingdong prefecture level city in Shandong
Fortune Shop	Green Intelligent Power Saver	Satisfaction Shopping	Jingdong prefecture level city in Shandong
Fortune Shop	Green Intelligent Power Saver	Lies.	Jingdong prefecture level city in Shandong
Fortune Shop	Green Intelligent Power Saver	It's a real power saver.	Jingdong prefecture level city in Shandong
Fortune Shop	Green Intelligent Power Saver	in use	Jingdong prefecture level city in Shandong
Fortune Shop	Green Intelligent Power Saver	I don't know yet.	Jingdong prefecture level city in Shandong
Fortune Shop	Green Intelligent Power Saver	not care about anything	Jingdong prefecture level city in Shandong
Fortune Shop	Green Intelligent Power Saver	It doesn't seem to have much effect	Jingdong prefecture level city in Shandong
Fortune Shop	Green Intelligent Power Saver	Let the merchant see for himself, does the plug positive and negative terminals look like this?	Jingdong prefecture level city in Shandong
Fortune Shop	Green Intelligent Power Saver	It doesn't work well, the electricity bill is less before using it, but more after using it, contacting customer service, the service is still not good, and returning it is slow	Jingdong prefecture level city in Shandong
Fortune Shop	Green Intelligent Power Saver	With the advertisement product does not match, above the effective negative, power saving rate of two rows of words it, the product looks very thin, with 3 days to see the meter number is still with the previous no difference, which is obviously fraudulent behavior!	Jingdong prefecture level city in Shandong
Fortune Shop	Green Intelligent Power Saver	Doesn't save any power at all. Not much use.	Jingdong prefecture level city in Shandong
Fortune Shop	Green Intelligent Power Saver	The plugs are all loose, they're all the same length and they don't work at all	Jingdong prefecture level city in Shandong
Fortune Shop	Green Intelligent Power Saver	It's bad 👎 it's not effective at all, you'd be crazy to sell this garbage on the internet.	Jingdong prefecture level city in Shandong
Fortune Shop	Green Intelligent Power Saver	It doesn't save any power at all, so I hope the old timers don't fall for this scam again	Jingdong prefecture level city in Shandong
Fortune Shop	Green Intelligent Power Saver	I've been using it for over a month now and haven't noticed any power savings.	Jingdong prefecture level city in Shandong
Fortune Shop	Green Intelligent Power Saver	It doesn't work at all. The electricity bill is higher when you use it.	Jingdong prefecture level city in Shandong
Fortune Shop	Green Intelligent Power Saver	It doesn't save any power, it's still as bad as ever.	Jingdong prefecture level city in Shandong

Fortune Shop	Green Intelligent Power Saver	Last night before leaving work to look at the table, and then plugged into the node device, this morning and then look at the table, than the original save a few degrees of electricity, very good to have a power-saving effect, easy to use, satisfied with the shopping praise.	Jingdong prefecture level city in Shandong
Fortune Shop	Green Intelligent Power Saver	The product is real and effective, the electricity bill is reduced, really good, thanks to the merchants have so many good products, the product is real and effective, the electricity bill is reduced, want to save electricity friends quickly buy it, early use early to save money.	Jingdong prefecture level city in Shandong
Fortune Shop	Green Intelligent Power Saver	Saver saves time and money, baby appearance is beautiful, the quality of goods is good, and the same as the business description, good service attitude, packaging is complete, logistics is very fast, praise to	Jingdong prefecture level city in Shandong
Fortune Shop	Green Intelligent Power Saver	The effect is good, summer, home electricity is relatively large, buy and use a few days, feel the effect is very good, originally ten dollars a day electricity bill, now eight dollars a day, quite good	Jingdong prefecture level city in Shandong
Fortune Shop	Green Intelligent Power Saver	Indeed can save electricity and money, the first time I know this or from a friend's home to see, but also do not believe it, and now they use it to know that there is this good thing.	Jingdong prefecture level city in Shandong
Fortune Shop	Green Intelligent Power Saver	I have received the Smart Power Saver. Perfectly packaged. Fast delivery. Boss customer service attitude is also very good. It's worth buying. I plugged it in and tried it and it really saves more power than the original. I'll buy it again next time.	Jingdong prefecture level city in Shandong
Fortune Shop	Green Intelligent Power Saver	It's nice to save a dozen bucks a night on two air conditioners that used to take 30 or 40 degrees a night, but now it's only 10 or so degrees a night	Jingdong prefecture level city in Shandong
Fortune Shop	Green Intelligent Power Saver	Product no manufacturer's name no product qualification certificate, try two days to see the effect of	Jingdong prefecture level city in Shandong
Fortune Shop	Green Intelligent Power Saver	I've been using it for over a month and have saved over $80, so I've repurchased a few more back to save together	Jingdong prefecture level city in Shandong
Fortune Shop	Green Intelligent Power Saver	I haven't used it yet and will comment when I do, but it's well made.	Jingdong prefecture level city in Shandong
Fortune Shop	Green Intelligent Power Saver	It's quite energy efficient. It's really a boon for the home.	Jingdong prefecture level city in Shandong
Fortune Shop	Green Intelligent Power Saver	Energy-saving socket received a month, after using it for a month proved that it is indeed energy-saving, electricity bill saving, will buy it again, thank you!	Jingdong prefecture level city in Shandong
Fortune Shop	Green Intelligent Power Saver	There was a similar product many years ago. Now technology has evolved, buy one to try, according to the instructions in the box, it takes one or two dozen days to show results. Check your electric bill and see what happens.	Jingdong prefecture level city in Shandong
Fortune Shop	Green Intelligent Power Saver	Jingdong logistics is really fast, the same day the order arrived on the same day, more convenient than the supermarket, and a wide range of choices, the	Jingdong prefecture level city in Shandong
Fortune Shop	Green Intelligent Power Saver	I've been using this for a month or so and I can't see any results, it really is a scam as they say.	Jingdong prefecture level city in Shandong
Fortune Shop	Green Intelligent Power Saver	Very good use, a month can save a few degrees of power loss of electricity, introduced to the relatives to buy back to try.	Jingdong prefecture level city in Shandong
Fortune Shop	Green Intelligent Power Saver	It's really powerful! With this air conditioner can be turned on at any time without the pressure of paying the electricity bill! It's good for the people!	Jingdong prefecture level city in Shandong
Fortune Shop	Green Intelligent Power Saver	This business is a big ** products are not qualified, with a month of electricity more than one hundred percent of thirty, the purchase agreed to return the goods but do not receive a refund after receiving the goods, looking for the official side does not help, the Jingdong is a big **, I have never shopped in	Jingdong prefecture level city in Shandong
Fortune Shop	Green Intelligent Power Saver	It's good. I tried it. It saves power.	Jingdong prefecture level city in Shandong
Fortune Shop	Green Intelligent Power Saver	Nothing**, it seems like there's more	Jingdong prefecture level city in Shandong
Fortune Shop	Green Intelligent Power Saver	Power saving device received, well packaged, good quality, fast logistics	Jingdong prefecture level city in Shandong
Fortune Shop	Green Intelligent Power Saver	The service is okay, is that when I bought five, the desirable pieces are three, and then wait a few days and send me two replacement, I do not know if it is a mistake of the shipper, if I was not in the courier there to see, go home to say no, the	Jingdong prefecture level city in Shandong
Fortune Shop	Green Intelligent Power Saver	This thing will only cost me more if I use it, but before I used it, it was less than a ~ electricity a month, and after I used it, it was more than two hundred kWh a month.	Jingdong prefecture level city in Shandong
Fortune Shop	Green Intelligent Power Saver	I just used it. I don't know how it is.	Jingdong prefecture level city in Shandong
Fortune Shop	Green Intelligent Power Saver	I've received the product and I'm using it, so I'll see how it goes in a month!	Jingdong prefecture level city in Shandong
Fortune Shop	Green Intelligent Power Saver	The goods have just received the effect do not know how, please look forward to the results.	Jingdong prefecture level city in Shandong
Fortune Shop	Green Intelligent Power Saver	Power saving device, the material is very good practical and durable, the size is suitable, packaging is very good, the price is cheap, logistics is very fast!	Jingdong prefecture level city in Shandong
Fortune Shop	Green Intelligent Power Saver	Very nice, will comment in detail after using it for a while.	Jingdong prefecture level city in Shandong
Fortune Shop	Green Intelligent Power Saver	Good stuff to trust and support	Jingdong prefecture level city in Shandong
Fortune Shop	Green Intelligent Power Saver	I'll comment after I use it, doubt if it really saves power no	Jingdong prefecture level city in Shandong
Fortune Shop	Green Intelligent Power Saver	It's good quality and works well!	Jingdong prefecture level city in Shandong
Fortune Shop	Green Intelligent Power Saver	hahaha just right hahahahahaha hiccup hahahaha	Jingdong prefecture level city in Shandong
Fortune Shop	Green Intelligent Power Saver	The real thing matches the picture and looks great!	Jingdong prefecture level city in Shandong
Fortune Shop	Green Intelligent Power Saver	Plugged it in and we'll see when we pay the bills at the end of the year!	Jingdong prefecture level city in Shandong
Fortune Shop	Green Intelligent Power Saver	False advertising. It doesn't work.	Jingdong prefecture level city in Shandong
Fortune Shop	Green Intelligent Power Saver	Goods received, good effect	Jingdong prefecture level city in Shandong
Fortune Shop	Green Intelligent Power Saver	It looks okay, we'll see if it saves electricity when the time comes	Jingdong prefecture level city in Shandong
Fortune Shop	Green Intelligent Power Saver	Logistics is very fast quality is very good, the price is very affordable, accessories are also complete, installed, satisfied with good use	Jingdong prefecture level city in Shandong
Fortune Shop	Green Intelligent Power Saver	I've received the power saver. I'm looking forward to the savings.	Jingdong prefecture level city in Shandong
Fortune Shop	Green Intelligent Power Saver	Received, have not yet used, after use and then evaluate	Jingdong prefecture level city in Shandong
Fortune Shop	Green Intelligent Power Saver	I received the item, but I don't know if it saves power or not.	Jingdong prefecture level city in Shandong
Fortune Shop	Green Intelligent Power Saver	On Trial	Jingdong prefecture level city in Shandong
Fortune Shop	Green Intelligent Power Saver	Use it and see how it goes.	Jingdong prefecture level city in Shandong
Fortune Shop	Green Intelligent Power Saver	Quality is very good, no odor, fine workmanship, grain color are very like, satisfied!	Jingdong prefecture level city in Shandong
Fortune Shop	Green Intelligent Power Saver	I'll give you a good review. I don't know if it'll work.	Jingdong prefecture level city in Shandong
Fortune Shop	Green Intelligent Power Saver	The logistics is very fast and the package is intact. Good use of affordable. The effect is very good, is genuine	Jingdong prefecture level city in Shandong
Fortune Shop	Green Intelligent Power Saver	Nothing happens when you plug it in.	Jingdong prefecture level city in Shandong
Fortune Shop	Green Intelligent Power Saver	I don't know if it's as good as it sounds.	Jingdong prefecture level city in Shandong

Fortune Shop	Green Intelligent Power Saver	The quality of the stuff is very good, exactly the same as the seller's description, very satisfied!	Jingdong prefecture level city in Shandong
Fortune Shop	Green Intelligent Power Saver	The saver works great, it really works, it's affordable and worth buying.	Jingdong prefecture level city in Shandong
Fortune Shop	Green Intelligent Power Saver	Just used it for a couple days. I don't know yet.	Jingdong prefecture level city in Shandong
Fortune Shop	Green Intelligent Power Saver	The quality is quite good, cheaper than the physical store, good, next time will come again!	Jingdong prefecture level city in Shandong
Fortune Shop	Green Intelligent Power Saver	Good quality, cheap and affordable worth recommending to buy!	Jingdong prefecture level city in Shandong
Fortune Shop	Green Intelligent Power Saver	Very good quality, well made, works well! Satisfied	Jingdong prefecture level city in Shandong
Fortune Shop	Green Intelligent Power Saver	The logistics is very fast quality is very good very pleasant a shopping	Jingdong prefecture level city in Shandong
Fortune Shop	Green Intelligent Power Saver	Use it and then come back to evaluate!	Jingdong prefecture level city in Shandong
Fortune Shop	Green Intelligent Power Saver	# It's no use lying at all #	Jingdong prefecture level city in Shandong
Fortune Shop	Green Intelligent Power Saver	I can't get a voltage drop.	Jingdong prefecture level city in Shandong
Fortune Shop	Green Intelligent Power Saver	Evaluate after use	Jingdong prefecture level city in Shandong
Fortune Shop	Green Intelligent Power Saver	Still on trial.	Jingdong prefecture level city in Shandong
Fortune Shop	Green Intelligent Power Saver	ineffective	Jingdong prefecture level city in Shandong
Fortune Shop	Green Intelligent Power Saver	Satisfaction Shopping	Jingdong prefecture level city in Shandong
Fortune Shop	Green Intelligent Power Saver	Lies.	Jingdong prefecture level city in Shandong
Fortune Shop	Green Intelligent Power Saver	It's a real power saver.	Jingdong prefecture level city in Shandong
Fortune Shop	Green Intelligent Power Saver	in use	Jingdong prefecture level city in Shandong
Fortune Shop	Green Intelligent Power Saver	I don't know yet.	Jingdong prefecture level city in Shandong
Fortune Shop	Green Intelligent Power Saver	not care about anything	Jingdong prefecture level city in Shandong
Fortune Shop	Green Intelligent Power Saver	It doesn't seem to have much effect	Jingdong prefecture level city in Shandong
Fortune Shop	Green Intelligent Power Saver	Let the merchant see for himself, does the plug positive and negative terminals look like this?	Jingdong prefecture level city in Shandong
Fortune Shop	Green Intelligent Power Saver	It doesn't work well, the electricity bill is less before using it, but more after using it, contacting customer service, the service is still not good, and returning it is slow	Jingdong prefecture level city in Shandong
Fortune Shop	Green Intelligent Power Saver	With the advertisement product does not match, above the effective negative, power saving rate of two rows of words it, the product looks very thin, with 3 days to see the meter number is still with the previous no difference, which is obviously fraudulent behavior!	Jingdong prefecture level city in Shandong
Fortune Shop	Green Intelligent Power Saver	Doesn't save any power at all. Not much use.	Jingdong prefecture level city in Shandong
Fortune Shop	Green Intelligent Power Saver	The plugs are all loose, they're all the same length and they don't work at all	Jingdong prefecture level city in Shandong
Fortune Shop	Green Intelligent Power Saver	It's bad 👎 it's not effective at all, you'd be crazy to sell this garbage on the internet.	Jingdong prefecture level city in Shandong
Fortune Shop	Green Intelligent Power Saver	It doesn't save any power at all, so I hope the old timers don't fall for this scam again	Jingdong prefecture level city in Shandong
Fortune Shop	Green Intelligent Power Saver	I've been using it for over a month now and haven't noticed any power savings.	Jingdong prefecture level city in Shandong
Fortune Shop	Green Intelligent Power Saver	It doesn't work at all. The electricity bill is higher when you use it.	Jingdong prefecture level city in Shandong
Fortune Shop	Green Intelligent Power Saver	It doesn't save any power, it's still as bad as ever.	Jingdong prefecture level city in Shandong
Fortune Shop	Green Intelligent Power Saver	Last night before leaving work to look at the table, and then plugged into the node device, this morning and then look at the table, the original save a few degrees of electricity, very good to have a power-saving effect, easy to use, satisfied with the shopping praise.	Jingdong prefecture level city in Shandong
Fortune Shop	Green Intelligent Power Saver	The product is real and effective, the electricity bill is reduced, really good, thanks to the merchants have so many good products, the product is real and effective, the electricity bill is reduced, want to save electricity friends quickly buy it, early use early to save money.	Jingdong prefecture level city in Shandong
Fortune Shop	Green Intelligent Power Saver	Saver saves time and money, baby appearance is beautiful, the quality of goods is good, and the same as the business description, good service attitude, packaging is complete, logistics is very fast, praise to	Jingdong prefecture level city in Shandong
Fortune Shop	Green Intelligent Power Saver	The effect is good, summer, home electricity is relatively large, buy and use a few days, feel the effect is very good, originally ten dollars a day electricity bill, now eight dollars a day, quite good	Jingdong prefecture level city in Shandong
Fortune Shop	Green Intelligent Power Saver	Indeed can save electricity and money, the first time I know this or from a friend's home to see, but also do not believe it, and now they use it to know that there is this good thing.	Jingdong prefecture level city in Shandong
Fortune Shop	Green Intelligent Power Saver	I have received the Smart Power Saver. Perfectly packaged. Fast delivery. Boss customer service attitude is also very good. It's worth buying. I plugged it in and tried it and it really saves more power than the original. I'll buy it again next time.	Jingdong prefecture level city in Shandong
Fortune Shop	Green Intelligent Power Saver	It's nice to save a dozen bucks a night on two air conditioners that used to take 30 or 40 degrees a night, but now it's only 10 or so degrees a night	Jingdong prefecture level city in Shandong
Fortune Shop	Green Intelligent Power Saver	Product no manufacturer's name no product qualification certificate, try two days to see the effect of	Jingdong prefecture level city in Shandong
Fortune Shop	Green Intelligent Power Saver	I've been using it for over a month and have saved over $80, so I've repurchased a few more back to save together	Jingdong prefecture level city in Shandong
Fortune Shop	Green Intelligent Power Saver	I haven't used it yet and will comment when I do, but it's well made.	Jingdong prefecture level city in Shandong
Fortune Shop	Green Intelligent Power Saver	It's quite energy efficient. It's really a boon for the home.	Jingdong prefecture level city in Shandong
Fortune Shop	Green Intelligent Power Saver	Energy-saving socket received a month, after using it for a month proved that it is indeed energy-saving, electricity bill saving, will buy it again, thank you!	Jingdong prefecture level city in Shandong
Fortune Shop	Green Intelligent Power Saver	There was a similar product many years ago. Now technology has evolved, buy one to try, according to the instructions in the box, it takes one or two dozen days to show results. Check your electric bill and see what happens.	Jingdong prefecture level city in Shandong
Fortune Shop	Green Intelligent Power Saver	Jingdong logistics is really fast, the same day the order arrived on the same day, more convenient than the supermarket, and a wide range of choices, the	Jingdong prefecture level city in Shandong
Fortune Shop	Green Intelligent Power Saver	I've been using this for a month or so and I can't see any results, it really is a scam as they say.	Jingdong prefecture level city in Shandong
Fortune Shop	Green Intelligent Power Saver	Very good use, a month can save a few degrees of power loss of electricity, introduced to the relatives to buy back to try.	Jingdong prefecture level city in Shandong
Fortune Shop	Green Intelligent Power Saver	It's really powerful! With this air conditioner can be turned on at any time without the pressure of paying the electricity bill! It's good for the people!	Jingdong prefecture level city in Shandong
Fortune Shop	Green Intelligent Power Saver	This business is a big ** products are not qualified, with a month of electricity more than one hundred percent of thirty, the purchase agreed to return the goods but do not receive a refund after receiving the goods, looking for the official side does not help, the Jingdong is a big **, I have never shopped in	Jingdong prefecture level city in Shandong

Fortune Shop	Green Intelligent Power Saver	It's good. I tried it. It saves power.	Jingdong prefecture level city in Shandong
Fortune Shop	Green Intelligent Power Saver	Nothing**, it seems like there's more	Jingdong prefecture level city in Shandong
Fortune Shop	Green Intelligent Power Saver	Power saving device received, well packaged, good quality, fast logistics	Jingdong prefecture level city in Shandong
Fortune Shop	Green Intelligent Power Saver	The service is okay, is that when I bought five, the desirable pieces are three, and then wait a few days and send me two replacement, I do not know if it is a mistake of the shipper, if I was not in the courier there to see, go home to say no, the	Jingdong prefecture level city in Shandong
Fortune Shop	Green Intelligent Power Saver	This thing will only cost me more if I use it, but before I used it, it was less than a ~ electricity a month, and after I used it, it was more than two hundred kWh a month.	Jingdong prefecture level city in Shandong
Fortune Shop	Green Intelligent Power Saver	I just used it. I don't know how it is.	Jingdong prefecture level city in Shandong
Fortune Shop	Green Intelligent Power Saver	I've received the product and I'm using it, so I'll see how it goes in a month!	Jingdong prefecture level city in Shandong
Fortune Shop	Green Intelligent Power Saver	The goods have just received the effect do not know how, please look forward to the results.	Jingdong prefecture level city in Shandong
Fortune Shop	Green Intelligent Power Saver	Power saving device, the material is very good practical and durable, the size is suitable, packaging is very good, the price is cheap, logistics is very fast!	Jingdong prefecture level city in Shandong
Fortune Shop	Green Intelligent Power Saver	Very nice, will comment in detail after using it for a while.	Jingdong prefecture level city in Shandong
Fortune Shop	Green Intelligent Power Saver	Good stuff to trust and support	Jingdong prefecture level city in Shandong
Fortune Shop	Green Intelligent Power Saver	I'll comment after I use it, doubt if it really saves power no	Jingdong prefecture level city in Shandong
Fortune Shop	Green Intelligent Power Saver	It's good quality and works well!	Jingdong prefecture level city in Shandong
Fortune Shop	Green Intelligent Power Saver	hahaha just right hahahahahaha hiccup hahahaha	Jingdong prefecture level city in Shandong
Fortune Shop	Green Intelligent Power Saver	The real thing matches the picture and looks great!	Jingdong prefecture level city in Shandong
Fortune Shop	Green Intelligent Power Saver	Plugged it in and we'll see when we pay the bills at the end of the year!	Jingdong prefecture level city in Shandong
Fortune Shop	Green Intelligent Power Saver	False advertising. It doesn't work.	Jingdong prefecture level city in Shandong
Fortune Shop	Green Intelligent Power Saver	Goods received, good effect	Jingdong prefecture level city in Shandong
Fortune Shop	Green Intelligent Power Saver	It looks okay, we'll see if it saves electricity when the time comes	Jingdong prefecture level city in Shandong
Fortune Shop	Green Intelligent Power Saver	Logistics is very fast quality is very good, the price is very affordable, accessories are also complete, installed, satisfied with good use	Jingdong prefecture level city in Shandong
Fortune Shop	Green Intelligent Power Saver	I've received the power saver. I'm looking forward to the savings.	Jingdong prefecture level city in Shandong
Fortune Shop	Green Intelligent Power Saver	Received, have not yet used, after use and then evaluate	Jingdong prefecture level city in Shandong
Fortune Shop	Green Intelligent Power Saver	I received the item, but I don't know if it saves power or not.	Jingdong prefecture level city in Shandong
Fortune Shop	Green Intelligent Power Saver	On Trial	Jingdong prefecture level city in Shandong
Fortune Shop	Green Intelligent Power Saver	Use it and see how it goes.	Jingdong prefecture level city in Shandong
Fortune Shop	Green Intelligent Power Saver	Quality is very good, no odor, fine workmanship, grain color are very like, satisfied!	Jingdong prefecture level city in Shandong
Fortune Shop	Green Intelligent Power Saver	I'll give you a good review. I don't know if it'll work.	Jingdong prefecture level city in Shandong
Fortune Shop	Green Intelligent Power Saver	The logistics is very fast and the package is intact. Good use of affordable. The effect is very good, is genuine	Jingdong prefecture level city in Shandong
Fortune Shop	Green Intelligent Power Saver	Nothing happens when you plug it in.	Jingdong prefecture level city in Shandong
Fortune Shop	Green Intelligent Power Saver	I don't know if it's as good as it sounds.	Jingdong prefecture level city in Shandong
Fortune Shop	Green Intelligent Power Saver	The quality of the stuff is very good, exactly the same as the seller's description, very satisfied!	Jingdong prefecture level city in Shandong
Fortune Shop	Green Intelligent Power Saver	The saver works great, it really works, it's affordable and worth buying.	Jingdong prefecture level city in Shandong
Fortune Shop	Green Intelligent Power Saver	Just used it for a couple days. I don't know yet.	Jingdong prefecture level city in Shandong
Fortune Shop	Green Intelligent Power Saver	The quality is quite good, cheaper than the physical store, good, next time will come again!	Jingdong prefecture level city in Shandong
Fortune Shop	Green Intelligent Power Saver	Good quality, cheap and affordable worth recommending to buy!	Jingdong prefecture level city in Shandong
Fortune Shop	Green Intelligent Power Saver	Very good quality, well made, works well! Satisfied	Jingdong prefecture level city in Shandong
Fortune Shop	Green Intelligent Power Saver	The logistics is very fast quality is very good very pleasant a shopping	Jingdong prefecture level city in Shandong
Fortune Shop	Green Intelligent Power Saver	Use it and then come back to evaluate!	Jingdong prefecture level city in Shandong
Fortune Shop	Green Intelligent Power Saver	# It's no use lying at all #	Jingdong prefecture level city in Shandong
Fortune Shop	Green Intelligent Power Saver	I can't get a voltage drop.	Jingdong prefecture level city in Shandong
Fortune Shop	Green Intelligent Power Saver	Evaluate after use	Jingdong prefecture level city in Shandong
Fortune Shop	Green Intelligent Power Saver	Still on trial.	Jingdong prefecture level city in Shandong
Fortune Shop	Green Intelligent Power Saver	ineffective	Jingdong prefecture level city in Shandong
Fortune Shop	Green Intelligent Power Saver	Satisfaction Shopping	Jingdong prefecture level city in Shandong
Fortune Shop	Green Intelligent Power Saver	Lies.	Jingdong prefecture level city in Shandong
Fortune Shop	Green Intelligent Power Saver	It's a real power saver.	Jingdong prefecture level city in Shandong
Fortune Shop	Green Intelligent Power Saver	in use	Jingdong prefecture level city in Shandong
Fortune Shop	Green Intelligent Power Saver	I don't know yet.	Jingdong prefecture level city in Shandong
Fortune Shop	Green Intelligent Power Saver	not care about anything	Jingdong prefecture level city in Shandong
Fortune Shop	Green Intelligent Power Saver	It doesn't seem to have much effect	Jingdong prefecture level city in Shandong
Fortune Shop	Green Intelligent Power Saver	Let the merchant see for himself, does the plug positive and negative terminals look like this?	Jingdong prefecture level city in Shandong

Fortune Shop	Green Intelligent Power Saver	It doesn't work well, the electricity bill is less before using it, but more after using it, contacting customer service, the service is still not good, and returning it is slow	Jingdong prefecture level city in Shandong
Fortune Shop	Green Intelligent Power Saver	With the advertisement product does not match, above the effective negative, power saving rate of two rows of words it, the product looks very thin, with 3 days to see the meter number is still with the previous no difference, which is obviously fraudulent behavior!	Jingdong prefecture level city in Shandong
Fortune Shop	Green Intelligent Power Saver	Doesn't save any power at all. Not much use.	Jingdong prefecture level city in Shandong
Fortune Shop	Green Intelligent Power Saver	The plugs are all loose, they're all the same length and they don't work at all	Jingdong prefecture level city in Shandong
Fortune Shop	Green Intelligent Power Saver	It's bad 👎 it's not effective at all, you'd be crazy to sell this garbage on the internet.	Jingdong prefecture level city in Shandong
Fortune Shop	Green Intelligent Power Saver	It doesn't save any power at all, so I hope the old timers don't fall for this scam again	Jingdong prefecture level city in Shandong
Fortune Shop	Green Intelligent Power Saver	I've been using it for over a month now and haven't noticed any power savings.	Jingdong prefecture level city in Shandong
Fortune Shop	Green Intelligent Power Saver	It doesn't work at all. The electricity bill is higher when you use it.	Jingdong prefecture level city in Shandong
Fortune Shop	Green Intelligent Power Saver	It doesn't save any power, it's still as bad as ever.	Jingdong prefecture level city in Shandong
Fortune Shop	Green Intelligent Power Saver	Last night before leaving work to look at the table, and then plugged into the node device, this morning and then look at the table, than the original save a few degrees of electricity, very good to have a power-saving effect, easy to use, satisfied with the shopping praise.	Jingdong prefecture level city in Shandong
Fortune Shop	Green Intelligent Power Saver	The product is real and effective, the electricity bill is reduced, really good, thanks to the merchants have so many good products, the product is real and effective, the electricity bill is reduced, want to save electricity friends quickly buy it, early use early to save money.	Jingdong prefecture level city in Shandong
Fortune Shop	Green Intelligent Power Saver	Saver saves time and money, baby appearance is beautiful, the quality of goods is good, and the same as the business description, good service attitude, packaging is complete, logistics is very fast, praise to	Jingdong prefecture level city in Shandong
Fortune Shop	Green Intelligent Power Saver	The effect is good, summer, home electricity is relatively large, buy and use a few days, feel the effect is very good, originally ten dollars a day electricity bill, now eight dollars a day, quite good	Jingdong prefecture level city in Shandong
Fortune Shop	Green Intelligent Power Saver	Indeed can save electricity and money, the first time I know this or from a friend's home to see, but also do not believe it, and now they use it to know that there is this good thing.	Jingdong prefecture level city in Shandong
Fortune Shop	Green Intelligent Power Saver	I have received the Smart Power Saver. Perfectly packaged. Fast delivery. Boss customer service attitude is also very good. It's worth buying. I plugged it in and tried it and it really saves more power than the original. I'll buy it again next time.	Jingdong prefecture level city in Shandong
Fortune Shop	Green Intelligent Power Saver	It's nice to save a dozen bucks a night on two air conditioners that used to take 30 or 40 degrees a night, but now it's only 10 or so degrees a night	Jingdong prefecture level city in Shandong
Fortune Shop	Green Intelligent Power Saver	Product no manufacturer's name no product qualification certificate, try two days to see the effect of	Jingdong prefecture level city in Shandong
Fortune Shop	Green Intelligent Power Saver	I've been using it for over a month and have saved over $80, so I've repurchased a few more back to save together	Jingdong prefecture level city in Shandong
Fortune Shop	Green Intelligent Power Saver	I haven't used it yet and will comment when I do, but it's well made.	Jingdong prefecture level city in Shandong
Fortune Shop	Green Intelligent Power Saver	It's quite energy efficient. It's really a boon for the home.	Jingdong prefecture level city in Shandong
Fortune Shop	Green Intelligent Power Saver	Energy-saving socket received a month, after using it for a month proved that it is indeed energy-saving, electricity bill saving, will buy it again, thank you!	Jingdong prefecture level city in Shandong
Fortune Shop	Green Intelligent Power Saver	There was a similar product many years ago. Now technology has evolved, buy one to try, according to the instructions in the box, it takes one or two dozen days to show results. Check your electric bill and see what happens.	Jingdong prefecture level city in Shandong
Fortune Shop	Green Intelligent Power Saver	Jingdong logistics is really fast, the same day the order arrived on the same day, more convenient than the supermarket, and a wide range of choices, the	Jingdong prefecture level city in Shandong
Fortune Shop	Green Intelligent Power Saver	I've been using this for a month or so and I can't see any results, it really is a scam as they say.	Jingdong prefecture level city in Shandong
Fortune Shop	Green Intelligent Power Saver	Very good use, a month can save a few degrees of power loss of electricity, introduced to the relatives to buy back to try.	Jingdong prefecture level city in Shandong
Fortune Shop	Green Intelligent Power Saver	It's really powerful! With this air conditioner can be turned on at any time without the pressure of paying the electricity bill! It's good for the people!	Jingdong prefecture level city in Shandong
Fortune Shop	Green Intelligent Power Saver	This business is a big ** products are not qualified, with a month of electricity more than one hundred percent of thirty, the purchase agreed to return the goods but do not receive a refund after receiving the goods, looking for the official side does not help, the Jingdong is a big **, I have never shopped in	Jingdong prefecture level city in Shandong
Fortune Shop	Green Intelligent Power Saver	It's good. I tried it. It saves power.	Jingdong prefecture level city in Shandong
Fortune Shop	Green Intelligent Power Saver	Nothing**, it seems like there's more	Jingdong prefecture level city in Shandong
Fortune Shop	Green Intelligent Power Saver	Power saving device received, well packaged, good quality, fast logistics	Jingdong prefecture level city in Shandong
Fortune Shop	Green Intelligent Power Saver	The service is okay, is that when I bought five, the desirable pieces are three, and then wait a few days and send me two replacement, I do not know if it is a mistake of the shipper, if I was not in the courier there to see, go home to say no, the	Jingdong prefecture level city in Shandong
Fortune Shop	Green Intelligent Power Saver	This thing will only cost me more if I use it, but before I used it, it was less than a ~ electricity a month, and after I used it, it was more than two hundred kWh a month.	Jingdong prefecture level city in Shandong
Fortune Shop	Green Intelligent Power Saver	I just used it. I don't know how it is.	Jingdong prefecture level city in Shandong
Fortune Shop	Green Intelligent Power Saver	I've received the product and I'm using it, so I'll see how it goes in a month!	Jingdong prefecture level city in Shandong
Fortune Shop	Green Intelligent Power Saver	The goods have just received the effect do not know how, please look forward to the results.	Jingdong prefecture level city in Shandong
Fortune Shop	Green Intelligent Power Saver	Power saving device, the material is very good practical and durable, the size is suitable, packaging is very good, the price is cheap, logistics is very fast!	Jingdong prefecture level city in Shandong
Fortune Shop	Green Intelligent Power Saver	Very nice, will comment in detail after using it for a while.	Jingdong prefecture level city in Shandong
Fortune Shop	Green Intelligent Power Saver	Good stuff to trust and support	Jingdong prefecture level city in Shandong
Fortune Shop	Green Intelligent Power Saver	I'll comment after I use it, doubt if it really saves power no	Jingdong prefecture level city in Shandong
Fortune Shop	Green Intelligent Power Saver	It's good quality and works well!	Jingdong prefecture level city in Shandong
Fortune Shop	Green Intelligent Power Saver	hahaha just right hahahahahaha hiccup hahahaha	Jingdong prefecture level city in Shandong
Fortune Shop	Green Intelligent Power Saver	The real thing matches the picture and looks great!	Jingdong prefecture level city in Shandong
Fortune Shop	Green Intelligent Power Saver	Plugged it in and we'll see when we pay the bills at the end of the year!	Jingdong prefecture level city in Shandong
Fortune Shop	Green Intelligent Power Saver	False advertising. It doesn't work.	Jingdong prefecture level city in Shandong
Fortune Shop	Green Intelligent Power Saver	Goods received, good effect	Jingdong prefecture level city in Shandong
Fortune Shop	Green Intelligent Power Saver	It looks okay, we'll see if it saves electricity when the time comes	Jingdong prefecture level city in Shandong
Fortune Shop	Green Intelligent Power Saver	Logistics is very fast quality is very good, the price is very affordable, accessories are also complete, installed, satisfied with good use	Jingdong prefecture level city in Shandong
Fortune Shop	Green Intelligent Power Saver	I've received the power saver. I'm looking forward to the savings.	Jingdong prefecture level city in Shandong

Fortune Shop	Green Intelligent Power Saver	Received, have not yet used, after use and then evaluate	Jingdong prefecture level city in Shandong
Fortune Shop	Green Intelligent Power Saver	I received the item, but I don't know if it saves power or not.	Jingdong prefecture level city in Shandong
Fortune Shop	Green Intelligent Power Saver	On Trial	Jingdong prefecture level city in Shandong
Fortune Shop	Green Intelligent Power Saver	Use it and see how it goes.	Jingdong prefecture level city in Shandong
Fortune Shop	Green Intelligent Power Saver	Quality is very good, no odor, fine workmanship, grain color are very like, satisfied!	Jingdong prefecture level city in Shandong
Fortune Shop	Green Intelligent Power Saver	I'll give you a good review. I don't know if it'll work.	Jingdong prefecture level city in Shandong
Fortune Shop	Green Intelligent Power Saver	The logistics is very fast and the package is intact. Good use of affordable. The effect is very good, is genuine	Jingdong prefecture level city in Shandong
Fortune Shop	Green Intelligent Power Saver	Nothing happens when you plug it in.	Jingdong prefecture level city in Shandong
Fortune Shop	Green Intelligent Power Saver	I don't know if it's as good as it sounds.	Jingdong prefecture level city in Shandong
Fortune Shop	Green Intelligent Power Saver	The quality of the stuff is very good, exactly the same as the seller's description, very satisfied!	Jingdong prefecture level city in Shandong
Fortune Shop	Green Intelligent Power Saver	The saver works great, it really works, it's affordable and worth buying.	Jingdong prefecture level city in Shandong
Fortune Shop	Green Intelligent Power Saver	Just used it for a couple days. I don't know yet.	Jingdong prefecture level city in Shandong
Fortune Shop	Green Intelligent Power Saver	The quality is quite good, cheaper than the physical store, good, next time will come again!	Jingdong prefecture level city in Shandong
Fortune Shop	Green Intelligent Power Saver	Good quality, cheap and affordable worth recommending to buy!	Jingdong prefecture level city in Shandong
Fortune Shop	Green Intelligent Power Saver	Very good quality, well made, works well! Satisfied	Jingdong prefecture level city in Shandong
Fortune Shop	Green Intelligent Power Saver	The logistics is very fast quality is very good very pleasant a shopping	Jingdong prefecture level city in Shandong
Fortune Shop	Green Intelligent Power Saver	Use it and then come back to evaluate!	Jingdong prefecture level city in Shandong
Fortune Shop	Green Intelligent Power Saver	# It's no use lying at all #	Jingdong prefecture level city in Shandong
Fortune Shop	Green Intelligent Power Saver	I can't get a voltage drop.	Jingdong prefecture level city in Shandong
Fortune Shop	Green Intelligent Power Saver	Evaluate after use	Jingdong prefecture level city in Shandong
Fortune Shop	Green Intelligent Power Saver	Still on trial.	Jingdong prefecture level city in Shandong
Fortune Shop	Green Intelligent Power Saver	ineffective	Jingdong prefecture level city in Shandong
Fortune Shop	Green Intelligent Power Saver	Satisfaction Shopping	Jingdong prefecture level city in Shandong
Fortune Shop	Green Intelligent Power Saver	Lies.	Jingdong prefecture level city in Shandong
Fortune Shop	Green Intelligent Power Saver	It's a real power saver.	Jingdong prefecture level city in Shandong
Fortune Shop	Green Intelligent Power Saver	in use	Jingdong prefecture level city in Shandong
Fortune Shop	Green Intelligent Power Saver	I don't know yet.	Jingdong prefecture level city in Shandong
Fortune Shop	Green Intelligent Power Saver	not care about anything	Jingdong prefecture level city in Shandong
Fortune Shop	Green Intelligent Power Saver	It doesn't seem to have much effect	Jingdong prefecture level city in Shandong
Fortune Shop	Green Intelligent Power Saver	Let the merchant see for himself, does the plug positive and negative terminals look like this?	Jingdong prefecture level city in Shandong
Fortune Shop	Green Intelligent Power Saver	It doesn't work well, the electricity bill is less before using it, but more after using it, contacting customer service, the service is still not good, and returning it is slow	Jingdong prefecture level city in Shandong
Fortune Shop	Green Intelligent Power Saver	With the advertisement product does not match, above the effective negative, power saving rate of two rows of words it, the product looks very thin, with 3 days to see the meter number is still with the previous no difference, which is obviously fraudulent behavior!	Jingdong prefecture level city in Shandong
Fortune Shop	Green Intelligent Power Saver	Doesn't save any power at all. Not much use.	Jingdong prefecture level city in Shandong
Fortune Shop	Green Intelligent Power Saver	The plugs are all loose, they're all the same length and they don't work at all	Jingdong prefecture level city in Shandong
Fortune Shop	Green Intelligent Power Saver	It's bad 👎 it's not effective at all, you'd be crazy to sell this garbage on the internet.	Jingdong prefecture level city in Shandong
Fortune Shop	Green Intelligent Power Saver	It doesn't save any power at all, so I hope the old timers don't fall for this scam again	Jingdong prefecture level city in Shandong
Fortune Shop	Green Intelligent Power Saver	I've been using it for over a month now and haven't noticed any power savings.	Jingdong prefecture level city in Shandong
Fortune Shop	Green Intelligent Power Saver	It doesn't work at all. The electricity bill is higher when you use it.	Jingdong prefecture level city in Shandong
Fortune Shop	Green Intelligent Power Saver	It doesn't save any power, it's still as bad as ever.	Jingdong prefecture level city in Shandong
Fortune Shop	Green Intelligent Power Saver	Last night before leaving work to look at the table, and then plugged into the node device, this morning and then look at the table, than the original save a few degrees of electricity, very good to have a power-saving effect, easy to use, satisfied with the shopping praise.	Jingdong prefecture level city in Shandong
Fortune Shop	Green Intelligent Power Saver	The product is real and effective, the electricity bill is reduced, really good, thanks to the merchants have so many good products, the product is real and effective, the electricity bill is reduced, want to save electricity friends quickly buy it, early use early to save money.	Jingdong prefecture level city in Shandong
Fortune Shop	Green Intelligent Power Saver	Saver saves time and money, baby appearance is beautiful, the quality of goods is good, and the same as the business description, good service attitude, packaging is complete, logistics is very fast, praise to	Jingdong prefecture level city in Shandong
Fortune Shop	Green Intelligent Power Saver	The effect is good, summer, home electricity is relatively large, buy and use a few days, feel the effect is very good, originally ten dollars a day electricity bill, now eight dollars a day, quite good	Jingdong prefecture level city in Shandong
Fortune Shop	Green Intelligent Power Saver	Indeed can save electricity and money, the first time I know this or from a friend's home to see, but also do not believe it, and now they use it to know that there is this good thing.	Jingdong prefecture level city in Shandong
Fortune Shop	Green Intelligent Power Saver	I have received the Smart Power Saver. Perfectly packaged. Fast delivery. Boss customer service attitude is also very good. It's worth buying. I plugged it in and tried it and it really saves more power than the original. I'll buy it again next time.	Jingdong prefecture level city in Shandong
Fortune Shop	Green Intelligent Power Saver	It's nice to save a dozen bucks a night on two air conditioners that used to take 30 or 40 degrees a night, but now it's only 10 or so degrees a night	Jingdong prefecture level city in Shandong
Fortune Shop	Green Intelligent Power Saver	Product no manufacturer's name no product qualification certificate, try two days to see the effect of	Jingdong prefecture level city in Shandong
Fortune Shop	Green Intelligent Power Saver	I've been using it for over a month and have saved over $80, so I've repurchased a few more back to save together	Jingdong prefecture level city in Shandong
Fortune Shop	Green Intelligent Power Saver	I haven't used it yet and will comment when I do, but it's well made.	Jingdong prefecture level city in Shandong

Fortune Shop	Green Intelligent Power Saver	It's quite energy efficient. It's really a boon for the home.	Jingdong prefecture level city in Shandong
Fortune Shop	Green Intelligent Power Saver	Energy-saving socket received a month, after using it for a month proved that it is indeed energy-saving, electricity bill saving, will buy it again, thank you!	Jingdong prefecture level city in Shandong
Fortune Shop	Green Intelligent Power Saver	There was a similar product many years ago. Now technology has evolved, buy one to try, according to the instructions in the box, it takes one or two dozen days to show results. Check your electric bill and see what happens.	Jingdong prefecture level city in Shandong
Fortune Shop	Green Intelligent Power Saver	Jingdong logistics is really fast, the same day the order arrived on the same day, more convenient than the supermarket, and a wide range of choices, the	Jingdong prefecture level city in Shandong
Fortune Shop	Green Intelligent Power Saver	I've been using this for a month or so and I can't see any results, it really is a scam as they say.	Jingdong prefecture level city in Shandong
Fortune Shop	Green Intelligent Power Saver	Very good use, a month can save a few degrees of power loss of electricity, introduced to the relatives to buy back to try.	Jingdong prefecture level city in Shandong
Fortune Shop	Green Intelligent Power Saver	It's really powerful! With this air conditioner can be turned on at any time without the pressure of paying the electricity bill! It's good for the people!	Jingdong prefecture level city in Shandong
Fortune Shop	Green Intelligent Power Saver	This business is a big ** products are not qualified, with a month of electricity more than one hundred percent of thirty, the purchase agreed to return the goods but do not receive a refund after receiving the goods, looking for the official side does not help, the Jingdong is a big **, I have never shopped in	Jingdong prefecture level city in Shandong
Fortune Shop	Green Intelligent Power Saver	It's good. I tried it. It saves power.	Jingdong prefecture level city in Shandong
Fortune Shop	Green Intelligent Power Saver	Nothing**, it seems like there's more	Jingdong prefecture level city in Shandong
Fortune Shop	Green Intelligent Power Saver	Power saving device received, well packaged, good quality, fast logistics	Jingdong prefecture level city in Shandong
Fortune Shop	Green Intelligent Power Saver	The service is okay, is that when I bought five, the desirable pieces are three, and then wait a few days and send me two replacement, I do not know if it is a mistake of the shipper, if I was not in the courier there to see, go home to say no, the	Jingdong prefecture level city in Shandong
Fortune Shop	Green Intelligent Power Saver	This thing will only cost me more if I use it, but before I used it, it was less than a ~ electricity a month, and after I used it, it was more than two hundred kWh a month.	Jingdong prefecture level city in Shandong
Fortune Shop	Green Intelligent Power Saver	I just used it. I don't know how it is.	Jingdong prefecture level city in Shandong
Fortune Shop	Green Intelligent Power Saver	I've received the product and I'm using it, so I'll see how it goes in a month!	Jingdong prefecture level city in Shandong
Fortune Shop	Green Intelligent Power Saver	The goods have just received the effect do not know how, please look forward to the results.	Jingdong prefecture level city in Shandong
Fortune Shop	Green Intelligent Power Saver	Power saving device, the material is very good practical and durable, the size is suitable, packaging is very good, the price is cheap, logistics is very fast!	Jingdong prefecture level city in Shandong
Fortune Shop	Green Intelligent Power Saver	Very nice, will comment in detail after using it for a while.	Jingdong prefecture level city in Shandong
Fortune Shop	Green Intelligent Power Saver	Good stuff to trust and support	Jingdong prefecture level city in Shandong
Fortune Shop	Green Intelligent Power Saver	I'll comment after I use it, doubt if it really saves power no	Jingdong prefecture level city in Shandong
Fortune Shop	Green Intelligent Power Saver	It's good quality and works well!	Jingdong prefecture level city in Shandong
Fortune Shop	Green Intelligent Power Saver	hahaha just right hahahahahaha hiccup hahahaha	Jingdong prefecture level city in Shandong
Fortune Shop	Green Intelligent Power Saver	The real thing matches the picture and looks great!	Jingdong prefecture level city in Shandong
Fortune Shop	Green Intelligent Power Saver	Plugged it in and we'll see when we pay the bills at the end of the year!	Jingdong prefecture level city in Shandong
Fortune Shop	Green Intelligent Power Saver	False advertising. It doesn't work.	Jingdong prefecture level city in Shandong
Fortune Shop	Green Intelligent Power Saver	Goods received, good effect	Jingdong prefecture level city in Shandong
Fortune Shop	Green Intelligent Power Saver	It looks okay, we'll see if it saves electricity when the time comes	Jingdong prefecture level city in Shandong
Fortune Shop	Green Intelligent Power Saver	Logistics is very fast quality is very good, the price is very affordable, accessories are also complete, installed, satisfied with good use	Jingdong prefecture level city in Shandong
Fortune Shop	Green Intelligent Power Saver	I've received the power saver. I'm looking forward to the savings.	Jingdong prefecture level city in Shandong
Fortune Shop	Green Intelligent Power Saver	Received, have not yet used, after use and then evaluate	Jingdong prefecture level city in Shandong
Fortune Shop	Green Intelligent Power Saver	I received the item, but I don't know if it saves power or not.	Jingdong prefecture level city in Shandong
Fortune Shop	Green Intelligent Power Saver	On Trial	Jingdong prefecture level city in Shandong
Fortune Shop	Green Intelligent Power Saver	Use it and see how it goes.	Jingdong prefecture level city in Shandong
Fortune Shop	Green Intelligent Power Saver	Quality is very good, no odor, fine workmanship, grain color are very like, satisfied!	Jingdong prefecture level city in Shandong
Fortune Shop	Green Intelligent Power Saver	I'll give you a good review. I don't know if it'll work.	Jingdong prefecture level city in Shandong
Fortune Shop	Green Intelligent Power Saver	The logistics is very fast and the package is intact. Good use of affordable. The effect is very good, is genuine	Jingdong prefecture level city in Shandong
Fortune Shop	Green Intelligent Power Saver	Nothing happens when you plug it in.	Jingdong prefecture level city in Shandong
Fortune Shop	Green Intelligent Power Saver	I don't know if it's as good as it sounds.	Jingdong prefecture level city in Shandong
Fortune Shop	Green Intelligent Power Saver	The quality of the stuff is very good, exactly the same as the seller's description, very satisfied!	Jingdong prefecture level city in Shandong
Fortune Shop	Green Intelligent Power Saver	The saver works great, it really works, it's affordable and worth buying.	Jingdong prefecture level city in Shandong
Fortune Shop	Green Intelligent Power Saver	Just used it for a couple days. I don't know yet.	Jingdong prefecture level city in Shandong
Fortune Shop	Green Intelligent Power Saver	The quality is quite good, cheaper than the physical store, good, next time will come again!	Jingdong prefecture level city in Shandong
Fortune Shop	Green Intelligent Power Saver	Good quality, cheap and affordable worth recommending to buy!	Jingdong prefecture level city in Shandong
Fortune Shop	Green Intelligent Power Saver	Very good quality, well made, works well! Satisfied	Jingdong prefecture level city in Shandong
Fortune Shop	Green Intelligent Power Saver	The logistics is very fast quality is very good very pleasant a shopping	Jingdong prefecture level city in Shandong
Fortune Shop	Green Intelligent Power Saver	Use it and then come back to evaluate!	Jingdong prefecture level city in Shandong
Fortune Shop	Green Intelligent Power Saver	# It's no use lying at all #	Jingdong prefecture level city in Shandong
Fortune Shop	Green Intelligent Power Saver	I can't get a voltage drop.	Jingdong prefecture level city in Shandong
Fortune Shop	Green Intelligent Power Saver	Evaluate after use	Jingdong prefecture level city in Shandong
Fortune Shop	Green Intelligent Power Saver	Still on trial.	Jingdong prefecture level city in Shandong

Fortune Shop	Green Intelligent Power Saver	ineffective	Jingdong prefecture level city in Shandong
Fortune Shop	Green Intelligent Power Saver	Satisfaction Shopping	Jingdong prefecture level city in Shandong
Fortune Shop	Green Intelligent Power Saver	Lies.	Jingdong prefecture level city in Shandong
Fortune Shop	Green Intelligent Power Saver	It's a real power saver.	Jingdong prefecture level city in Shandong
Fortune Shop	Green Intelligent Power Saver	in use	Jingdong prefecture level city in Shandong
Fortune Shop	Green Intelligent Power Saver	I don't know yet.	Jingdong prefecture level city in Shandong
Fortune Shop	Green Intelligent Power Saver	not care about anything	Jingdong prefecture level city in Shandong
Fortune Shop	Green Intelligent Power Saver	It doesn't seem to have much effect	Jingdong prefecture level city in Shandong
Fortune Shop	Green Intelligent Power Saver	Let the merchant see for himself, does the plug positive and negative terminals look like this?	Jingdong prefecture level city in Shandong
Fortune Shop	Green Intelligent Power Saver	It doesn't work well, the electricity bill is less before using it, but more after using it, contacting customer service, the service is still not good, and returning it is slow	Jingdong prefecture level city in Shandong
Fortune Shop	Green Intelligent Power Saver	With the advertisement product does not match, above the effective negative, power saving rate of two rows of words it, the product looks very thin, with 3 days to see the meter number is still with the previous no difference, which is obviously fraudulent behavior!	Jingdong prefecture level city in Shandong
Fortune Shop	Green Intelligent Power Saver	Doesn't save any power at all. Not much use.	Jingdong prefecture level city in Shandong
Fortune Shop	Green Intelligent Power Saver	The plugs are all loose, they're all the same length and they don't work at all	Jingdong prefecture level city in Shandong
Fortune Shop	Green Intelligent Power Saver	It's bad 👎 it's not effective at all, you'd be crazy to sell this garbage on the internet.	Jingdong prefecture level city in Shandong
Fortune Shop	Green Intelligent Power Saver	It doesn't save any power at all, so I hope the old timers don't fall for this scam again	Jingdong prefecture level city in Shandong
Fortune Shop	Green Intelligent Power Saver	I've been using it for over a month now and haven't noticed any power savings.	Jingdong prefecture level city in Shandong
Fortune Shop	Green Intelligent Power Saver	It doesn't work at all. The electricity bill is higher when you use it.	Jingdong prefecture level city in Shandong
Fortune Shop	Green Intelligent Power Saver	It doesn't save any power, it's still as bad as ever.	Jingdong prefecture level city in Shandong
Fortune Shop	Green Intelligent Power Saver	Last night before leaving work to look at the table, and then plugged into the node device, this morning and then look at the table, than the original save a few degrees of electricity, very good to have a power-saving effect, easy to use, satisfied with the shopping praise.	Jingdong prefecture level city in Shandong
Fortune Shop	Green Intelligent Power Saver	The product is real and effective, the electricity bill is reduced, really good, thanks to the merchants have so many good products, the product is real and effective, the electricity bill is reduced, want to save electricity friends quickly buy it, early use early to save money.	Jingdong prefecture level city in Shandong
Fortune Shop	Green Intelligent Power Saver	Saver saves time and money, baby appearance is beautiful, the quality of goods is good, and the same as the business description, good service attitude, packaging is complete, logistics is very fast, praise to	Jingdong prefecture level city in Shandong
Fortune Shop	Green Intelligent Power Saver	The effect is good, summer, home electricity is relatively large, buy and use a few days, feel the effect is very good, originally ten dollars a day electricity bill, now eight dollars a day, quite good	Jingdong prefecture level city in Shandong
Fortune Shop	Green Intelligent Power Saver	Indeed can save electricity and money, the first time I know this or from a friend's home to see, but also do not believe it, and now they use it to know that there is this good thing.	Jingdong prefecture level city in Shandong
Fortune Shop	Green Intelligent Power Saver	I have received the Smart Power Saver. Perfectly packaged. Fast delivery. Boss customer service attitude is also very good. It's worth buying. I plugged it in and tried it and it really saves more power than the original. I'll buy it again next time.	Jingdong prefecture level city in Shandong
Fortune Shop	Green Intelligent Power Saver	It's nice to save a dozen bucks a night on two air conditioners that used to take 30 or 40 degrees a night, but now it's only 10 or so degrees a night	Jingdong prefecture level city in Shandong
Fortune Shop	Green Intelligent Power Saver	Product no manufacturer's name no product qualification certificate, try two days to see the effect of	Jingdong prefecture level city in Shandong
Fortune Shop	Green Intelligent Power Saver	I've been using it for over a month and have saved over $80, so I've repurchased a few more back to save together	Jingdong prefecture level city in Shandong
Fortune Shop	Green Intelligent Power Saver	I haven't used it yet and will comment when I do, but it's well made.	Jingdong prefecture level city in Shandong
Fortune Shop	Green Intelligent Power Saver	It's quite energy efficient. It's really a boon for the home.	Jingdong prefecture level city in Shandong
Fortune Shop	Green Intelligent Power Saver	Energy-saving socket received a month, after using it for a month proved that it is indeed energy-saving, electricity bill saving, will buy it again, thank you!	Jingdong prefecture level city in Shandong
Fortune Shop	Green Intelligent Power Saver	There was a similar product many years ago. Now technology has evolved, buy one to try, according to the instructions in the box, it takes one or two dozen days to show results. Check your electric bill and see what happens.	Jingdong prefecture level city in Shandong
Fortune Shop	Green Intelligent Power Saver	Jingdong logistics is really fast, the same day the order arrived on the same day, more convenient than the supermarket, and a wide range of choices, the	Jingdong prefecture level city in Shandong
Fortune Shop	Green Intelligent Power Saver	I've been using this for a month or so and I can't see any results, it really is a scam as they say.	Jingdong prefecture level city in Shandong
Fortune Shop	Green Intelligent Power Saver	Very good use, a month can save a few degrees of power loss of electricity, introduced to the relatives to buy back to try.	Jingdong prefecture level city in Shandong
Fortune Shop	Green Intelligent Power Saver	It's really powerful! With this air conditioner can be turned on at any time without the pressure of paying the electricity bill! It's good for the people!	Jingdong prefecture level city in Shandong
Fortune Shop	Green Intelligent Power Saver	This business is a big ** products are not qualified, with a month of electricity more than one hundred percent of thirty, the purchase agreed to return the goods but do not receive a refund after receiving the goods, looking for the official side does not help, the Jingdong is a big **, I have never shopped in	Jingdong prefecture level city in Shandong
Fortune Shop	Green Intelligent Power Saver	It's good. I tried it. It saves power.	Jingdong prefecture level city in Shandong
Fortune Shop	Green Intelligent Power Saver	Nothing**, it seems like there's more	Jingdong prefecture level city in Shandong
Fortune Shop	Green Intelligent Power Saver	Power saving device received, well packaged, good quality, fast logistics	Jingdong prefecture level city in Shandong
Fortune Shop	Green Intelligent Power Saver	The service is okay, is that when I bought five, the desirable pieces are three, and then wait a few days and send me two replacement, I do not know if it is a mistake of the shipper, if I was not in the courier there to see, go home to say no, the	Jingdong prefecture level city in Shandong
Fortune Shop	Green Intelligent Power Saver	This thing will only cost me more if I use it, but before I used it, it was less than a ~ electricity a month, and after I used it, it was more than two hundred kWh a month.	Jingdong prefecture level city in Shandong
Fortune Shop	Green Intelligent Power Saver	I just used it. I don't know how it is.	Jingdong prefecture level city in Shandong
Fortune Shop	Green Intelligent Power Saver	I've received the product and I'm using it, so I'll see how it goes in a month!	Jingdong prefecture level city in Shandong
Fortune Shop	Green Intelligent Power Saver	The goods have just received the effect do not know how, please look forward to the results.	Jingdong prefecture level city in Shandong
Fortune Shop	Green Intelligent Power Saver	Power saving device, the material is very good practical and durable, the size is suitable, packaging is very good, the price is cheap, logistics is very fast!	Jingdong prefecture level city in Shandong
Fortune Shop	Green Intelligent Power Saver	Very nice, will comment in detail after using it for a while.	Jingdong prefecture level city in Shandong
Fortune Shop	Green Intelligent Power Saver	Good stuff to trust and support	Jingdong prefecture level city in Shandong
Fortune Shop	Green Intelligent Power Saver	I'll comment after I use it, doubt if it really saves power no	Jingdong prefecture level city in Shandong

Fortune Shop	Green Intelligent Power Saver	It's good quality and works well!	Jingdong prefecture level city in Shandong
Fortune Shop	Green Intelligent Power Saver	hahaha just right hahahahahaha hiccup hahahaha	Jingdong prefecture level city in Shandong
Fortune Shop	Green Intelligent Power Saver	The real thing matches the picture and looks great!	Jingdong prefecture level city in Shandong
Fortune Shop	Green Intelligent Power Saver	Plugged it in and we'll see when we pay the bills at the end of the year!	Jingdong prefecture level city in Shandong
Fortune Shop	Green Intelligent Power Saver	False advertising. It doesn't work.	Jingdong prefecture level city in Shandong
Fortune Shop	Green Intelligent Power Saver	Goods received, good effect	Jingdong prefecture level city in Shandong
Fortune Shop	Green Intelligent Power Saver	It looks okay, we'll see if it saves electricity when the time comes	Jingdong prefecture level city in Shandong
Fortune Shop	Green Intelligent Power Saver	Logistics is very fast quality is very good, the price is very affordable, accessories are also complete, installed, satisfied with good use	Jingdong prefecture level city in Shandong
Fortune Shop	Green Intelligent Power Saver	I've received the power saver. I'm looking forward to the savings.	Jingdong prefecture level city in Shandong
Fortune Shop	Green Intelligent Power Saver	Received, have not yet used, after use and then evaluate	Jingdong prefecture level city in Shandong
Fortune Shop	Green Intelligent Power Saver	I received the item, but I don't know if it saves power or not.	Jingdong prefecture level city in Shandong
Fortune Shop	Green Intelligent Power Saver	On Trial	Jingdong prefecture level city in Shandong
Fortune Shop	Green Intelligent Power Saver	Use it and see how it goes.	Jingdong prefecture level city in Shandong
Fortune Shop	Green Intelligent Power Saver	Quality is very good, no odor, fine workmanship, grain color are very like, satisfied!	Jingdong prefecture level city in Shandong
Fortune Shop	Green Intelligent Power Saver	I'll give you a good review. I don't know if it'll work.	Jingdong prefecture level city in Shandong
Fortune Shop	Green Intelligent Power Saver	The logistics is very fast and the package is intact. Good use of affordable. The effect is very good, is genuine	Jingdong prefecture level city in Shandong
Fortune Shop	Green Intelligent Power Saver	Nothing happens when you plug it in.	Jingdong prefecture level city in Shandong
Fortune Shop	Green Intelligent Power Saver	I don't know if it's as good as it sounds.	Jingdong prefecture level city in Shandong
Fortune Shop	Green Intelligent Power Saver	The quality of the stuff is very good, exactly the same as the seller's description, very satisfied!	Jingdong prefecture level city in Shandong
Fortune Shop	Green Intelligent Power Saver	The saver works great, it really works, it's affordable and worth buying.	Jingdong prefecture level city in Shandong
Fortune Shop	Green Intelligent Power Saver	Just used it for a couple days. I don't know yet.	Jingdong prefecture level city in Shandong
Fortune Shop	Green Intelligent Power Saver	The quality is quite good, cheaper than the physical store, good, next time will come again!	Jingdong prefecture level city in Shandong
Fortune Shop	Green Intelligent Power Saver	Good quality, cheap and affordable worth recommending to buy!	Jingdong prefecture level city in Shandong
Fortune Shop	Green Intelligent Power Saver	Very good quality, well made, works well! Satisfied	Jingdong prefecture level city in Shandong
Fortune Shop	Green Intelligent Power Saver	The logistics is very fast quality is very good very pleasant a shopping	Jingdong prefecture level city in Shandong
Fortune Shop	Green Intelligent Power Saver	Use it and then come back to evaluate!	Jingdong prefecture level city in Shandong
Fortune Shop	Green Intelligent Power Saver	# It's no use lying at all #	Jingdong prefecture level city in Shandong
Fortune Shop	Green Intelligent Power Saver	I can't get a voltage drop.	Jingdong prefecture level city in Shandong
Fortune Shop	Green Intelligent Power Saver	Evaluate after use	Jingdong prefecture level city in Shandong
Fortune Shop	Green Intelligent Power Saver	Still on trial.	Jingdong prefecture level city in Shandong
Fortune Shop	Green Intelligent Power Saver	ineffective	Jingdong prefecture level city in Shandong
Fortune Shop	Green Intelligent Power Saver	Satisfaction Shopping	Jingdong prefecture level city in Shandong
Fortune Shop	Green Intelligent Power Saver	Lies.	Jingdong prefecture level city in Shandong
Fortune Shop	Green Intelligent Power Saver	It's a real power saver.	Jingdong prefecture level city in Shandong
Fortune Shop	Green Intelligent Power Saver	in use	Jingdong prefecture level city in Shandong
Fortune Shop	Green Intelligent Power Saver	I don't know yet.	Jingdong prefecture level city in Shandong
Fortune Shop	Green Intelligent Power Saver	not care about anything	Jingdong prefecture level city in Shandong
Fortune Shop	Green Intelligent Power Saver	It doesn't seem to have much effect	Jingdong prefecture level city in Shandong
Fortune Shop	Green Intelligent Power Saver	Let the merchant see for himself, does the plug positive and negative terminals look like this?	Jingdong prefecture level city in Shandong
Fortune Shop	Green Intelligent Power Saver	It doesn't work well, the electricity bill is less before using it, but more after using it, contacting customer service, the service is still not good, and returning it is slow	Jingdong prefecture level city in Shandong
Fortune Shop	Green Intelligent Power Saver	With the advertisement product does not match, above the effective negative, power saving rate of two rows of words it, the product looks very thin, with 3 days to see the meter number is still with the previous no difference, which is obviously fraudulent behavior!	Jingdong prefecture level city in Shandong
Fortune Shop	Green Intelligent Power Saver	Doesn't save any power at all. Not much use.	Jingdong prefecture level city in Shandong
Fortune Shop	Green Intelligent Power Saver	The plugs are all loose, they're all the same length and they don't work at all	Jingdong prefecture level city in Shandong
Fortune Shop	Green Intelligent Power Saver	It's bad 👎 it's not effective at all, you'd be crazy to sell this garbage on the internet.	Jingdong prefecture level city in Shandong
Fortune Shop	Green Intelligent Power Saver	It doesn't save any power at all, so I hope the old timers don't fall for this scam again	Jingdong prefecture level city in Shandong
Fortune Shop	Green Intelligent Power Saver	I've been using it for over a month now and haven't noticed any power savings.	Jingdong prefecture level city in Shandong
Fortune Shop	Green Intelligent Power Saver	It doesn't work at all. The electricity bill is higher when you use it.	Jingdong prefecture level city in Shandong
Fortune Shop	Green Intelligent Power Saver	It doesn't save any power, it's still as bad as ever.	Jingdong prefecture level city in Shandong
Fortune Shop	Green Intelligent Power Saver	Last night before leaving work to look at the table, and then plugged into the node device, this morning and then look at the table, than the original save a few degrees of electricity, very good to have a power-saving effect, easy to use, satisfied with the shopping praise.	Jingdong prefecture level city in Shandong
Fortune Shop	Green Intelligent Power Saver	The product is real and effective, the electricity bill is reduced, really good, thanks to the merchants have so many good products, the product is real and effective, the electricity bill is reduced, want to save electricity friends quickly buy it, early use early to save money.	Jingdong prefecture level city in Shandong

Fortune Shop	Green Intelligent Power Saver	Saver saves time and money, baby appearance is beautiful, the quality of goods is good, and the same as the business description, good service attitude, packaging is complete, logistics is very fast, praise to	Jingdong prefecture level city in Shandong
Fortune Shop	Green Intelligent Power Saver	The effect is good, summer, home electricity is relatively large, buy and use a few days, feel the effect is very good, originally ten dollars a day electricity bill, now eight dollars a day, quite good	Jingdong prefecture level city in Shandong
Fortune Shop	Green Intelligent Power Saver	Indeed can save electricity and money, the first time I know this or from a friend's home to see, but also do not believe it, and now they use it to know that there is this good thing.	Jingdong prefecture level city in Shandong
Fortune Shop	Green Intelligent Power Saver	I have received the Smart Power Saver. Perfectly packaged. Fast delivery. Boss customer service attitude is also very good. It's worth buying. I plugged it in and tried it and it really saves more power than the original. I'll buy it again next time.	Jingdong prefecture level city in Shandong
Fortune Shop	Green Intelligent Power Saver	It's nice to save a dozen bucks a night on two air conditioners that used to take 30 or 40 degrees a night, but now it's only 10 or so degrees a night	Jingdong prefecture level city in Shandong
Fortune Shop	Green Intelligent Power Saver	Product no manufacturer's name no product qualification certificate, try two days to see the effect of	Jingdong prefecture level city in Shandong
Fortune Shop	Green Intelligent Power Saver	I've been using it for over a month and have saved over $80, so I've repurchased a few more back to save together	Jingdong prefecture level city in Shandong
Fortune Shop	Green Intelligent Power Saver	I haven't used it yet and will comment when I do, but it's well made.	Jingdong prefecture level city in Shandong
Fortune Shop	Green Intelligent Power Saver	It's quite energy efficient. It's really a boon for the home.	Jingdong prefecture level city in Shandong
Fortune Shop	Green Intelligent Power Saver	Energy-saving socket received a month, after using it for a month proved that it is indeed energy-saving, electricity bill saving, will buy it again, thank you!	Jingdong prefecture level city in Shandong
Fortune Shop	Green Intelligent Power Saver	There was a similar product many years ago. Now technology has evolved, buy one to try, according to the instructions in the box, it takes one or two dozen days to show results. Check your electric bill and see what happens.	Jingdong prefecture level city in Shandong
Fortune Shop	Green Intelligent Power Saver	Jingdong logistics is really fast, the same day the order arrived on the same day, more convenient than the supermarket, and a wide range of choices, the	Jingdong prefecture level city in Shandong
Fortune Shop	Green Intelligent Power Saver	I've been using this for a month or so and I can't see any results, it really is a scam as they say.	Jingdong prefecture level city in Shandong
Fortune Shop	Green Intelligent Power Saver	Very good use, a month can save a few degrees of power loss of electricity, introduced to the relatives to buy back to try.	Jingdong prefecture level city in Shandong
Fortune Shop	Green Intelligent Power Saver	It's really powerful! With this air conditioner can be turned on at any time without the pressure of paying the electricity bill! It's good for the people!	Jingdong prefecture level city in Shandong
Fortune Shop	Green Intelligent Power Saver	This business is a big ** products are not qualified, with a month of electricity more than one hundred percent of thirty, the purchase agreed to return the goods but do not receive a refund after receiving the goods, looking for the official side does not help, the Jingdong is a big **, I have never shopped in	Jingdong prefecture level city in Shandong
Fortune Shop	Green Intelligent Power Saver	It's good. I tried it. It saves power.	Jingdong prefecture level city in Shandong
Fortune Shop	Green Intelligent Power Saver	Nothing**, it seems like there's more	Jingdong prefecture level city in Shandong
Fortune Shop	Green Intelligent Power Saver	Power saving device received, well packaged, good quality, fast logistics	Jingdong prefecture level city in Shandong
Fortune Shop	Green Intelligent Power Saver	The service is okay, is that when I bought five, the desirable pieces are three, and then wait a few days and send me two replacement, I do not know if it is a mistake of the shipper, if I was not in the courier there to see, go home to say no, the	Jingdong prefecture level city in Shandong
Fortune Shop	Green Intelligent Power Saver	This thing will only cost me more if I use it, but before I used it, it was less than a ` electricity a month, and after I used it, it was more than two hundred kWh a month.	Jingdong prefecture level city in Shandong
Fortune Shop	Green Intelligent Power Saver	I just used it. I don't know how it is.	Jingdong prefecture level city in Shandong
Fortune Shop	Green Intelligent Power Saver	I've received the product and I'm using it, so I'll see how it goes in a month!	Jingdong prefecture level city in Shandong
Fortune Shop	Green Intelligent Power Saver	The goods have just received the effect do not know how, please look forward to the results.	Jingdong prefecture level city in Shandong
Fortune Shop	Green Intelligent Power Saver	Power saving device, the material is very good practical and durable, the size is suitable, packaging is very good, the price is cheap, logistics is very fast!	Jingdong prefecture level city in Shandong
Fortune Shop	Green Intelligent Power Saver	Very nice, will comment in detail after using it for a while.	Jingdong prefecture level city in Shandong
Fortune Shop	Green Intelligent Power Saver	Good stuff to trust and support	Jingdong prefecture level city in Shandong
Fortune Shop	Green Intelligent Power Saver	I'll comment after I use it, doubt if it really saves power no	Jingdong prefecture level city in Shandong
Fortune Shop	Green Intelligent Power Saver	It's good quality and works well!	Jingdong prefecture level city in Shandong
Fortune Shop	Green Intelligent Power Saver	hahaha just right hahahahahaha hiccup hahahaha	Jingdong prefecture level city in Shandong
Fortune Shop	Green Intelligent Power Saver	The real thing matches the picture and looks great!	Jingdong prefecture level city in Shandong
Fortune Shop	Green Intelligent Power Saver	Plugged it in and we'll see when we pay the bills at the end of the year!	Jingdong prefecture level city in Shandong
Fortune Shop	Green Intelligent Power Saver	False advertising. It doesn't work.	Jingdong prefecture level city in Shandong
Fortune Shop	Green Intelligent Power Saver	Goods received, good effect	Jingdong prefecture level city in Shandong
Fortune Shop	Green Intelligent Power Saver	It looks okay, we'll see if it saves electricity when the time comes	Jingdong prefecture level city in Shandong
Fortune Shop	Green Intelligent Power Saver	Logistics is very fast quality is very good, the price is very affordable, accessories are also complete, installed, satisfied with good use	Jingdong prefecture level city in Shandong
Fortune Shop	Green Intelligent Power Saver	I've received the power saver. I'm looking forward to the savings.	Jingdong prefecture level city in Shandong
Fortune Shop	Green Intelligent Power Saver	Received, have not yet used, after use and then evaluate	Jingdong prefecture level city in Shandong
Fortune Shop	Green Intelligent Power Saver	I received the item, but I don't know if it saves power or not.	Jingdong prefecture level city in Shandong
Fortune Shop	Green Intelligent Power Saver	On Trial	Jingdong prefecture level city in Shandong
Fortune Shop	Green Intelligent Power Saver	Use it and see how it goes.	Jingdong prefecture level city in Shandong
Fortune Shop	Green Intelligent Power Saver	Quality is very good, no odor, fine workmanship, grain color are very like, satisfied!	Jingdong prefecture level city in Shandong
Fortune Shop	Green Intelligent Power Saver	I'll give you a good review. I don't know if it'll work.	Jingdong prefecture level city in Shandong
Fortune Shop	Green Intelligent Power Saver	The logistics is very fast and the package is intact. Good use of affordable. The effect is very good, is genuine	Jingdong prefecture level city in Shandong
Fortune Shop	Green Intelligent Power Saver	Nothing happens when you plug it in.	Jingdong prefecture level city in Shandong
Fortune Shop	Green Intelligent Power Saver	I don't know if it's as good as it sounds.	Jingdong prefecture level city in Shandong
Fortune Shop	Green Intelligent Power Saver	The quality of the stuff is very good, exactly the same as the seller's description, very satisfied!	Jingdong prefecture level city in Shandong
Fortune Shop	Green Intelligent Power Saver	The saver works great, it really works, it's affordable and worth buying.	Jingdong prefecture level city in Shandong
Fortune Shop	Green Intelligent Power Saver	Just used it for a couple days. I don't know yet.	Jingdong prefecture level city in Shandong

Fortune Shop	Green Intelligent Power Saver	The quality is quite good, cheaper than the physical store, good, next time will come again!	Jingdong prefecture level city in Shandong
Fortune Shop	Green Intelligent Power Saver	Good quality, cheap and affordable worth recommending to buy!	Jingdong prefecture level city in Shandong
Fortune Shop	Green Intelligent Power Saver	Very good quality, well made, works well! Satisfied	Jingdong prefecture level city in Shandong
Fortune Shop	Green Intelligent Power Saver	The logistics is very fast quality is very good very pleasant a shopping	Jingdong prefecture level city in Shandong
Fortune Shop	Green Intelligent Power Saver	Use it and then come back to evaluate!	Jingdong prefecture level city in Shandong
Fortune Shop	Green Intelligent Power Saver	# It's no use lying at all #	Jingdong prefecture level city in Shandong
Fortune Shop	Green Intelligent Power Saver	I can't get a voltage drop.	Jingdong prefecture level city in Shandong
Fortune Shop	Green Intelligent Power Saver	Evaluate after use	Jingdong prefecture level city in Shandong
Fortune Shop	Green Intelligent Power Saver	Still on trial.	Jingdong prefecture level city in Shandong
Fortune Shop	Green Intelligent Power Saver	ineffective	Jingdong prefecture level city in Shandong
Fortune Shop	Green Intelligent Power Saver	Satisfaction Shopping	Jingdong prefecture level city in Shandong
Fortune Shop	Green Intelligent Power Saver	Lies.	Jingdong prefecture level city in Shandong
Fortune Shop	Green Intelligent Power Saver	It's a real power saver.	Jingdong prefecture level city in Shandong
Fortune Shop	Green Intelligent Power Saver	in use	Jingdong prefecture level city in Shandong
Fortune Shop	Green Intelligent Power Saver	I don't know yet.	Jingdong prefecture level city in Shandong
Fortune Shop	Green Intelligent Power Saver	not care about anything	Jingdong prefecture level city in Shandong
Fortune Shop	Green Intelligent Power Saver	It doesn't seem to have much effect	Jingdong prefecture level city in Shandong
Fortune Shop	Green Intelligent Power Saver	Let the merchant see for himself, does the plug positive and negative terminals look like this?	Jingdong prefecture level city in Shandong
Fortune Shop	Green Intelligent Power Saver	It doesn't work well, the electricity bill is less before using it, but more after using it, contacting customer service, the service is still not good, and returning it is slow	Jingdong prefecture level city in Shandong
Fortune Shop	Green Intelligent Power Saver	With the advertisement product does not match, above the effective negative, power saving rate of two rows of words it, the product looks very thin, with 3 days to see the meter number is still with the previous no difference, which is obviously fraudulent behavior!	Jingdong prefecture level city in Shandong
Fortune Shop	Green Intelligent Power Saver	Doesn't save any power at all. Not much use.	Jingdong prefecture level city in Shandong
Fortune Shop	Green Intelligent Power Saver	The plugs are all loose, they're all the same length and they don't work at all	Jingdong prefecture level city in Shandong
Fortune Shop	Green Intelligent Power Saver	It's bad 👎 it's not effective at all, you'd be crazy to sell this garbage on the internet.	Jingdong prefecture level city in Shandong
Fortune Shop	Green Intelligent Power Saver	It doesn't save any power at all, so I hope the old timers don't fall for this scam again	Jingdong prefecture level city in Shandong
Fortune Shop	Green Intelligent Power Saver	I've been using it for over a month now and haven't noticed any power savings.	Jingdong prefecture level city in Shandong
Fortune Shop	Green Intelligent Power Saver	It doesn't work at all. The electricity bill is higher when you use it.	Jingdong prefecture level city in Shandong
Fortune Shop	Green Intelligent Power Saver	It doesn't save any power, it's still as bad as ever.	Jingdong prefecture level city in Shandong
Fortune Shop	Green Intelligent Power Saver	Last night before leaving work to look at the table, and then plugged into the node device, this morning and then look at the table, than the original save a few degrees of electricity, very good to have a power-saving effect, easy to use, satisfied with the shopping praise.	Jingdong prefecture level city in Shandong
Fortune Shop	Green Intelligent Power Saver	The product is real and effective, the electricity bill is reduced, really good, thanks to the merchants have so many good products, the product is real and effective, the electricity bill is reduced, want to save electricity friends quickly buy it, early use early to save money.	Jingdong prefecture level city in Shandong
Fortune Shop	Green Intelligent Power Saver	Saver saves time and money, baby appearance is beautiful, the quality of goods is good, and the same as the business description, good service attitude, packaging is complete, logistics is very fast, praise to	Jingdong prefecture level city in Shandong
Fortune Shop	Green Intelligent Power Saver	The effect is good, summer, home electricity is relatively large, buy and use a few days, feel the effect is very good, originally ten dollars a day electricity bill, now eight dollars a day, quite good	Jingdong prefecture level city in Shandong
Fortune Shop	Green Intelligent Power Saver	Indeed can save electricity and money, the first time I know this or from a friend's home to see, but also do not believe it, and now they use it to know that there is this good thing.	Jingdong prefecture level city in Shandong
Fortune Shop	Green Intelligent Power Saver	I have received the Smart Power Saver. Perfectly packaged. Fast delivery. Boss customer service attitude is also very good. It's worth buying. I plugged it in and tried it and it really saves more power than the original. I'll buy it again next time.	Jingdong prefecture level city in Shandong
Fortune Shop	Green Intelligent Power Saver	It's nice to save a dozen bucks a night on two air conditioners that used to take 30 or 40 degrees a night, but now it's only 10 or so degrees a night	Jingdong prefecture level city in Shandong
Fortune Shop	Green Intelligent Power Saver	Product no manufacturer's name no product qualification certificate, try two days to see the effect of	Jingdong prefecture level city in Shandong
Fortune Shop	Green Intelligent Power Saver	I've been using it for over a month and have saved over $80, so I've repurchased a few more back to save together	Jingdong prefecture level city in Shandong
Fortune Shop	Green Intelligent Power Saver	I haven't used it yet and will comment when I do, but it's well made.	Jingdong prefecture level city in Shandong
Fortune Shop	Green Intelligent Power Saver	It's quite energy efficient. It's really a boon for the home.	Jingdong prefecture level city in Shandong
Fortune Shop	Green Intelligent Power Saver	Energy-saving socket received a month, after using it for a month proved that it is indeed energy-saving, electricity bill saving, will buy it again, thank you!	Jingdong prefecture level city in Shandong
Fortune Shop	Green Intelligent Power Saver	There was a similar product many years ago. Now technology has evolved, buy one to try, according to the instructions in the box, it takes one or two dozen days to show results. Check your electric bill and see what happens.	Jingdong prefecture level city in Shandong
Fortune Shop	Green Intelligent Power Saver	Jingdong logistics is really fast, the same day the order arrived on the same day, more convenient than the supermarket, and a wide range of choices, the	Jingdong prefecture level city in Shandong
Fortune Shop	Green Intelligent Power Saver	I've been using this for a month or so and I can't see any results, it really is a scam as they say.	Jingdong prefecture level city in Shandong
Fortune Shop	Green Intelligent Power Saver	Very good use, a month can save a few degrees of power loss of electricity, introduced to the relatives to buy back to try.	Jingdong prefecture level city in Shandong
Fortune Shop	Green Intelligent Power Saver	It's really powerful! With this air conditioner can be turned on at any time without the pressure of paying the electricity bill! It's good for the people!	Jingdong prefecture level city in Shandong
Fortune Shop	Green Intelligent Power Saver	This business is a big ** products are not qualified, with a month of electricity more than one hundred percent of thirty, the purchase agreed to return the goods but do not receive a refund after receiving the goods, looking for the official side does not help, the Jingdong is a big **, I have never shopped in	Jingdong prefecture level city in Shandong
Fortune Shop	Green Intelligent Power Saver	It's good. I tried it. It saves power.	Jingdong prefecture level city in Shandong
Fortune Shop	Green Intelligent Power Saver	Nothing**, it seems like there's more	Jingdong prefecture level city in Shandong
Fortune Shop	Green Intelligent Power Saver	Power saving device received, well packaged, good quality, fast logistics	Jingdong prefecture level city in Shandong

Fortune Shop	Green Intelligent Power Saver	The service is okay, is that when I bought five, the desirable pieces are three, and then wait a few days and send me two replacement, I do not know if it is a mistake of the shipper, if I was not in the courier there to see, go home to say no, the	Jingdong prefecture level city in Shandong
Fortune Shop	Green Intelligent Power Saver	This thing will only cost me more if I use it, but before I used it, it was less than a ~ electricity a month, and after I used it, it was more than two hundred kWh a month.	Jingdong prefecture level city in Shandong
Fortune Shop	Green Intelligent Power Saver	I just used it. I don't know how it is.	Jingdong prefecture level city in Shandong
Fortune Shop	Green Intelligent Power Saver	I've received the product and I'm using it, so I'll see how it goes in a month!	Jingdong prefecture level city in Shandong
Fortune Shop	Green Intelligent Power Saver	The goods have just received the effect do not know how, please look forward to the results.	Jingdong prefecture level city in Shandong
Fortune Shop	Green Intelligent Power Saver	Power saving device, the material is very good practical and durable, the size is suitable, packaging is very good, the price is cheap, logistics is very fast!	Jingdong prefecture level city in Shandong
Fortune Shop	Green Intelligent Power Saver	Very nice, will comment in detail after using it for a while.	Jingdong prefecture level city in Shandong
Fortune Shop	Green Intelligent Power Saver	Good stuff to trust and support	Jingdong prefecture level city in Shandong
Fortune Shop	Green Intelligent Power Saver	I'll comment after I use it, doubt if it really saves power no	Jingdong prefecture level city in Shandong
Fortune Shop	Green Intelligent Power Saver	It's good quality and works well!	Jingdong prefecture level city in Shandong
Fortune Shop	Green Intelligent Power Saver	hahaha just right hahahahahaha hiccup hahahaha	Jingdong prefecture level city in Shandong
Fortune Shop	Green Intelligent Power Saver	The real thing matches the picture and looks great!	Jingdong prefecture level city in Shandong
Fortune Shop	Green Intelligent Power Saver	Plugged it in and we'll see when we pay the bills at the end of the year!	Jingdong prefecture level city in Shandong
Fortune Shop	Green Intelligent Power Saver	False advertising. It doesn't work.	Jingdong prefecture level city in Shandong
Fortune Shop	Green Intelligent Power Saver	Goods received, good effect	Jingdong prefecture level city in Shandong
Fortune Shop	Green Intelligent Power Saver	It looks okay, we'll see if it saves electricity when the time comes	Jingdong prefecture level city in Shandong
Fortune Shop	Green Intelligent Power Saver	Logistics is very fast quality is very good, the price is very affordable, accessories are also complete, installed, satisfied with good use	Jingdong prefecture level city in Shandong
Fortune Shop	Green Intelligent Power Saver	I've received the power saver. I'm looking forward to the savings.	Jingdong prefecture level city in Shandong
Fortune Shop	Green Intelligent Power Saver	Received, have not yet used, after use and then evaluate	Jingdong prefecture level city in Shandong
Fortune Shop	Green Intelligent Power Saver	I received the item, but I don't know if it saves power or not.	Jingdong prefecture level city in Shandong
Fortune Shop	Green Intelligent Power Saver	On Trial	Jingdong prefecture level city in Shandong
Fortune Shop	Green Intelligent Power Saver	Use it and see how it goes.	Jingdong prefecture level city in Shandong
Fortune Shop	Green Intelligent Power Saver	Quality is very good, no odor, fine workmanship, grain color are very like, satisfied!	Jingdong prefecture level city in Shandong
Fortune Shop	Green Intelligent Power Saver	I'll give you a good review. I don't know if it'll work.	Jingdong prefecture level city in Shandong
Fortune Shop	Green Intelligent Power Saver	The logistics is very fast and the package is intact. Good use of affordable. The effect is very good, is genuine	Jingdong prefecture level city in Shandong
Fortune Shop	Green Intelligent Power Saver	Nothing happens when you plug it in.	Jingdong prefecture level city in Shandong
Fortune Shop	Green Intelligent Power Saver	I don't know if it's as good as it sounds.	Jingdong prefecture level city in Shandong
Fortune Shop	Green Intelligent Power Saver	The quality of the stuff is very good, exactly the same as the seller's description, very satisfied!	Jingdong prefecture level city in Shandong
Fortune Shop	Green Intelligent Power Saver	The saver works great, it really works, it's affordable and worth buying.	Jingdong prefecture level city in Shandong
Fortune Shop	Green Intelligent Power Saver	Just used it for a couple days. I don't know yet.	Jingdong prefecture level city in Shandong
Fortune Shop	Green Intelligent Power Saver	The quality is quite good, cheaper than the physical store, good, next time will come again!	Jingdong prefecture level city in Shandong
Fortune Shop	Green Intelligent Power Saver	Good quality, cheap and affordable worth recommending to buy!	Jingdong prefecture level city in Shandong
Fortune Shop	Green Intelligent Power Saver	Very good quality, well made, works well! Satisfied	Jingdong prefecture level city in Shandong
Fortune Shop	Green Intelligent Power Saver	The logistics is very fast quality is very good very pleasant a shopping	Jingdong prefecture level city in Shandong
Fortune Shop	Green Intelligent Power Saver	Use it and then come back to evaluate!	Jingdong prefecture level city in Shandong
Fortune Shop	Green Intelligent Power Saver	# It's no use lying at all #	Jingdong prefecture level city in Shandong
Fortune Shop	Green Intelligent Power Saver	I can't get a voltage drop.	Jingdong prefecture level city in Shandong
Fortune Shop	Green Intelligent Power Saver	Evaluate after use	Jingdong prefecture level city in Shandong
Fortune Shop	Green Intelligent Power Saver	Still on trial.	Jingdong prefecture level city in Shandong
Fortune Shop	Green Intelligent Power Saver	ineffective	Jingdong prefecture level city in Shandong
Fortune Shop	Green Intelligent Power Saver	Satisfaction Shopping	Jingdong prefecture level city in Shandong
Fortune Shop	Green Intelligent Power Saver	Lies.	Jingdong prefecture level city in Shandong
Fortune Shop	Green Intelligent Power Saver	It's a real power saver.	Jingdong prefecture level city in Shandong
Fortune Shop	Green Intelligent Power Saver	in use	Jingdong prefecture level city in Shandong
Fortune Shop	Green Intelligent Power Saver	I don't know yet.	Jingdong prefecture level city in Shandong
Fortune Shop	Green Intelligent Power Saver	not care about anything	Jingdong prefecture level city in Shandong
Fortune Shop	Green Intelligent Power Saver	It doesn't seem to have much effect	Jingdong prefecture level city in Shandong
Fortune Shop	Green Intelligent Power Saver	Let the merchant see for himself, does the plug positive and negative terminals look like this?	Jingdong prefecture level city in Shandong
Fortune Shop	Green Intelligent Power Saver	It doesn't work well, the electricity bill is less before using it, but more after using it, contacting customer service, the service is still not good, and returning it is slow	Jingdong prefecture level city in Shandong
Fortune Shop	Green Intelligent Power Saver	With the advertisement product does not match, above the effective negative, power saving rate of two rows of words it, the product looks very thin, with 3 days to see the meter number is still with the previous no difference, which is obviously fraudulent behavior!	Jingdong prefecture level city in Shandong

Fortune Shop	Green Intelligent Power Saver	Doesn't save any power at all. Not much use.	Jingdong prefecture level city in Shandong
Fortune Shop	Green Intelligent Power Saver	The plugs are all loose, they're all the same length and they don't work at all	Jingdong prefecture level city in Shandong
Fortune Shop	Green Intelligent Power Saver	It's bad 👎 it's not effective at all, you'd be crazy to sell this garbage on the internet.	Jingdong prefecture level city in Shandong
Fortune Shop	Green Intelligent Power Saver	It doesn't save any power at all, so I hope the old timers don't fall for this scam again	Jingdong prefecture level city in Shandong
Fortune Shop	Green Intelligent Power Saver	I've been using it for over a month now and haven't noticed any power savings.	Jingdong prefecture level city in Shandong
Fortune Shop	Green Intelligent Power Saver	It doesn't work at all. The electricity bill is higher when you use it.	Jingdong prefecture level city in Shandong
Fortune Shop	Green Intelligent Power Saver	It doesn't save any power, it's still as bad as ever.	Jingdong prefecture level city in Shandong
Fortune Shop	Green Intelligent Power Saver	Last night before leaving work to look at the table, and then plugged into the node device, this morning and then look at the table, than the original save a few degrees of electricity, very good to have a power-saving effect, easy to use, satisfied with the shopping praise.	Jingdong prefecture level city in Shandong
Fortune Shop	Green Intelligent Power Saver	The product is real and effective, the electricity bill is reduced, really good, thanks to the merchants have so many good products, the product is real and effective, the electricity bill is reduced, want to save electricity friends quickly buy it, early use early to save money.	Jingdong prefecture level city in Shandong
Fortune Shop	Green Intelligent Power Saver	Saver saves time and money, baby appearance is beautiful, the quality of goods is good, and the same as the business description, good service attitude, packaging is complete, logistics is very fast, praise to	Jingdong prefecture level city in Shandong
Fortune Shop	Green Intelligent Power Saver	The effect is good, summer, home electricity is relatively large, buy and use a few days, feel the effect is very good, originally ten dollars a day electricity bill, now eight dollars a day, quite good	Jingdong prefecture level city in Shandong
Fortune Shop	Green Intelligent Power Saver	Indeed can save electricity and money, the first time I know this or from a friend's home to see, but also do not believe it, and now they use it to know that there is this good thing.	Jingdong prefecture level city in Shandong
Fortune Shop	Green Intelligent Power Saver	I have received the Smart Power Saver. Perfectly packaged. Fast delivery. Boss customer service attitude is also very good. It's worth buying. I plugged it in and tried it and it really saves more power than the original. I'll buy it again next time.	Jingdong prefecture level city in Shandong
Fortune Shop	Green Intelligent Power Saver	It's nice to save a dozen bucks a night on two air conditioners that used to take 30 or 40 degrees a night, but now it's only 10 or so degrees a night	Jingdong prefecture level city in Shandong
Fortune Shop	Green Intelligent Power Saver	Product no manufacturer's name no product qualification certificate, try two days to see the effect of	Jingdong prefecture level city in Shandong
Fortune Shop	Green Intelligent Power Saver	I've been using it for over a month and have saved over $80, so I've repurchased a few more back to save together	Jingdong prefecture level city in Shandong
Fortune Shop	Green Intelligent Power Saver	I haven't used it yet and will comment when I do, but it's well made.	Jingdong prefecture level city in Shandong
Fortune Shop	Green Intelligent Power Saver	It's quite energy efficient. It's really a boon for the home.	Jingdong prefecture level city in Shandong
Fortune Shop	Green Intelligent Power Saver	Energy-saving socket received a month, after using it for a month proved that it is indeed energy-saving, electricity bill saving, will buy it again, thank you!	Jingdong prefecture level city in Shandong
Fortune Shop	Green Intelligent Power Saver	There was a similar product many years ago. Now technology has evolved, buy one to try, according to the instructions in the box, it takes one or two dozen days to show results. Check your electric bill and see what happens.	Jingdong prefecture level city in Shandong
Fortune Shop	Green Intelligent Power Saver	Jingdong logistics is really fast, the same day the order arrived on the same day, more convenient than the supermarket, and a wide range of choices, the	Jingdong prefecture level city in Shandong
Fortune Shop	Green Intelligent Power Saver	I've been using this for a month or so and I can't see any results, it really is a scam as they say.	Jingdong prefecture level city in Shandong
Fortune Shop	Green Intelligent Power Saver	Very good use, a month can save a few degrees of power loss of electricity, introduced to the relatives to buy back to try.	Jingdong prefecture level city in Shandong
Fortune Shop	Green Intelligent Power Saver	It's really powerful! With this air conditioner can be turned on at any time without the pressure of paying the electricity bill! It's good for the people!	Jingdong prefecture level city in Shandong
Fortune Shop	Green Intelligent Power Saver	This business is a big ** products are not qualified, with a month of electricity more than one hundred percent of thirty, the purchase agreed to return the goods but do not receive a refund after receiving the goods, looking for the official side does not help, the Jingdong is a big **, I have never shopped in	Jingdong prefecture level city in Shandong
Fortune Shop	Green Intelligent Power Saver	It's good. I tried it. It saves power.	Jingdong prefecture level city in Shandong
Fortune Shop	Green Intelligent Power Saver	Nothing**, it seems like there's more	Jingdong prefecture level city in Shandong
Fortune Shop	Green Intelligent Power Saver	Power saving device received, well packaged, good quality, fast logistics	Jingdong prefecture level city in Shandong
Fortune Shop	Green Intelligent Power Saver	The service is okay, is that when I bought five, the desirable pieces are three, and then wait a few days and send me two replacement, I do not know if it is a mistake of the shipper, if I was not in the courier there to see, go home to say no, the	Jingdong prefecture level city in Shandong
Fortune Shop	Green Intelligent Power Saver	This thing will only cost me more if I use it, but before I used it, it was less than a ~ electricity a month, and after I used it, it was more than two hundred kWh a month.	Jingdong prefecture level city in Shandong
Fortune Shop	Green Intelligent Power Saver	I just used it. I don't know how it is.	Jingdong prefecture level city in Shandong
Fortune Shop	Green Intelligent Power Saver	I've received the product and I'm using it, so I'll see how it goes in a month!	Jingdong prefecture level city in Shandong
Fortune Shop	Green Intelligent Power Saver	The goods have just received the effect do not know how, please look forward to the results.	Jingdong prefecture level city in Shandong
Fortune Shop	Green Intelligent Power Saver	Power saving device, the material is very good practical and durable, the size is suitable, packaging is very good, the price is cheap, logistics is very fast!	Jingdong prefecture level city in Shandong
Fortune Shop	Green Intelligent Power Saver	Very nice, will comment in detail after using it for a while.	Jingdong prefecture level city in Shandong
Fortune Shop	Green Intelligent Power Saver	Good stuff to trust and support	Jingdong prefecture level city in Shandong
Fortune Shop	Green Intelligent Power Saver	I'll comment after I use it, doubt if it really saves power no	Jingdong prefecture level city in Shandong
Fortune Shop	Green Intelligent Power Saver	It's good quality and works well!	Jingdong prefecture level city in Shandong
Fortune Shop	Green Intelligent Power Saver	hahaha just right hahahahahaha hiccup hahahaha	Jingdong prefecture level city in Shandong
Fortune Shop	Green Intelligent Power Saver	The real thing matches the picture and looks great!	Jingdong prefecture level city in Shandong
Fortune Shop	Green Intelligent Power Saver	Plugged it in and we'll see when we pay the bills at the end of the year!	Jingdong prefecture level city in Shandong
Fortune Shop	Green Intelligent Power Saver	False advertising. It doesn't work.	Jingdong prefecture level city in Shandong
Fortune Shop	Green Intelligent Power Saver	Goods received, good effect	Jingdong prefecture level city in Shandong
Fortune Shop	Green Intelligent Power Saver	It looks okay, we'll see if it saves electricity when the time comes	Jingdong prefecture level city in Shandong
Fortune Shop	Green Intelligent Power Saver	Logistics is very fast quality is very good, the price is very affordable, accessories are also complete, installed, satisfied with good use	Jingdong prefecture level city in Shandong
Fortune Shop	Green Intelligent Power Saver	I've received the power saver. I'm looking forward to the savings.	Jingdong prefecture level city in Shandong
Fortune Shop	Green Intelligent Power Saver	Received, have not yet used, after use and then evaluate	Jingdong prefecture level city in Shandong
Fortune Shop	Green Intelligent Power Saver	I received the item, but I don't know if it saves power or not.	Jingdong prefecture level city in Shandong

Fortune Shop	Green Intelligent Power Saver	On Trial	Jingdong prefecture level city in Shandong
Fortune Shop	Green Intelligent Power Saver	Use it and see how it goes.	Jingdong prefecture level city in Shandong
Fortune Shop	Green Intelligent Power Saver	Quality is very good, no odor, fine workmanship, grain color are very like, satisfied!	Jingdong prefecture level city in Shandong
Fortune Shop	Green Intelligent Power Saver	I'll give you a good review. I don't know if it'll work.	Jingdong prefecture level city in Shandong
Fortune Shop	Green Intelligent Power Saver	The logistics is very fast and the package is intact. Good use of affordable. The effect is very good, is genuine	Jingdong prefecture level city in Shandong
Fortune Shop	Green Intelligent Power Saver	Nothing happens when you plug it in.	Jingdong prefecture level city in Shandong
Fortune Shop	Green Intelligent Power Saver	I don't know if it's as good as it sounds.	Jingdong prefecture level city in Shandong
Fortune Shop	Green Intelligent Power Saver	The quality of the stuff is very good, exactly the same as the seller's description, very satisfied!	Jingdong prefecture level city in Shandong
Fortune Shop	Green Intelligent Power Saver	The saver works great, it really works, it's affordable and worth buying.	Jingdong prefecture level city in Shandong
Fortune Shop	Green Intelligent Power Saver	Just used it for a couple days. I don't know yet.	Jingdong prefecture level city in Shandong
Fortune Shop	Green Intelligent Power Saver	The quality is quite good, cheaper than the physical store, good, next time will come again!	Jingdong prefecture level city in Shandong
Fortune Shop	Green Intelligent Power Saver	Good quality, cheap and affordable worth recommending to buy!	Jingdong prefecture level city in Shandong
Fortune Shop	Green Intelligent Power Saver	Very good quality, well made, works well! Satisfied	Jingdong prefecture level city in Shandong
Fortune Shop	Green Intelligent Power Saver	The logistics is very fast quality is very good very pleasant a shopping	Jingdong prefecture level city in Shandong
Fortune Shop	Green Intelligent Power Saver	Use it and then come back to evaluate!	Jingdong prefecture level city in Shandong
Fortune Shop	Green Intelligent Power Saver	# It's no use lying at all #	Jingdong prefecture level city in Shandong
Fortune Shop	Green Intelligent Power Saver	I can't get a voltage drop.	Jingdong prefecture level city in Shandong
Fortune Shop	Green Intelligent Power Saver	Evaluate after use	Jingdong prefecture level city in Shandong
Fortune Shop	Green Intelligent Power Saver	Still on trial.	Jingdong prefecture level city in Shandong
Fortune Shop	Green Intelligent Power Saver	ineffective	Jingdong prefecture level city in Shandong
Fortune Shop	Green Intelligent Power Saver	Satisfaction Shopping	Jingdong prefecture level city in Shandong
Fortune Shop	Green Intelligent Power Saver	Lies.	Jingdong prefecture level city in Shandong
Fortune Shop	Green Intelligent Power Saver	It's a real power saver.	Jingdong prefecture level city in Shandong
Fortune Shop	Green Intelligent Power Saver	in use	Jingdong prefecture level city in Shandong
Fortune Shop	Green Intelligent Power Saver	I don't know yet.	Jingdong prefecture level city in Shandong
Fortune Shop	Green Intelligent Power Saver	not care about anything	Jingdong prefecture level city in Shandong
Fortune Shop	Green Intelligent Power Saver	It doesn't seem to have much effect	Jingdong prefecture level city in Shandong
Fortune Shop	Green Intelligent Power Saver	Let the merchant see for himself, does the plug positive and negative terminals look like this?	Jingdong prefecture level city in Shandong
Fortune Shop	Green Intelligent Power Saver	It doesn't work well, the electricity bill is less before using it, but more after using it, contacting customer service, the service is still not good, and returning it is slow	Jingdong prefecture level city in Shandong
Fortune Shop	Green Intelligent Power Saver	With the advertisement product does not match, above the effective negative, power saving rate of two rows of words it, the product looks very thin, with 3 days to see the meter number is still with the previous no difference, which is obviously fraudulent behavior!	Jingdong prefecture level city in Shandong
Fortune Shop	Green Intelligent Power Saver	Doesn't save any power at all. Not much use.	Jingdong prefecture level city in Shandong
Fortune Shop	Green Intelligent Power Saver	The plugs are all loose, they're all the same length and they don't work at all	Jingdong prefecture level city in Shandong
Fortune Shop	Green Intelligent Power Saver	It's bad 👎 it's not effective at all, you'd be crazy to sell this garbage on the internet.	Jingdong prefecture level city in Shandong
Fortune Shop	Green Intelligent Power Saver	It doesn't save any power at all, so I hope the old timers don't fall for this scam again	Jingdong prefecture level city in Shandong
Fortune Shop	Green Intelligent Power Saver	I've been using it for over a month now and haven't noticed any power savings.	Jingdong prefecture level city in Shandong
Fortune Shop	Green Intelligent Power Saver	It doesn't work at all. The electricity bill is higher when you use it.	Jingdong prefecture level city in Shandong
Fortune Shop	Green Intelligent Power Saver	It doesn't save any power, it's still as bad as ever.	Jingdong prefecture level city in Shandong
Fortune Shop	Green Intelligent Power Saver	Last night before leaving work to look at the table, and then plugged into the node device, this morning and then look at the table, than the original save a few degrees of electricity, very good to have a power-saving effect, easy to use, satisfied with the shopping praise.	Jingdong prefecture level city in Shandong
Fortune Shop	Green Intelligent Power Saver	The product is real and effective, the electricity bill is reduced, really good, thanks to the merchants have so many good products, the product is real and effective, the electricity bill is reduced, want to save electricity friends quickly buy it, early use early to save money.	Jingdong prefecture level city in Shandong
Fortune Shop	Green Intelligent Power Saver	Saver saves time and money, baby appearance is beautiful, the quality of goods is good, and the same as the business description, good service attitude, packaging is complete, logistics is very fast, praise to	Jingdong prefecture level city in Shandong
Fortune Shop	Green Intelligent Power Saver	The effect is good, summer, home electricity is relatively large, buy and use a few days, feel the effect is very good, originally ten dollars a day electricity bill, now eight dollars a day, quite good	Jingdong prefecture level city in Shandong
Fortune Shop	Green Intelligent Power Saver	Indeed can save electricity and money, the first time I know this or from a friend's home to see, but also do not believe it, and now they use it to know that there is this good thing.	Jingdong prefecture level city in Shandong
Fortune Shop	Green Intelligent Power Saver	I have received the Smart Power Saver. Perfectly packaged. Fast delivery. Boss customer service attitude is also very good. It's worth buying. I plugged it in and tried it and it really saves more power than the original. I'll buy it again next time.	Jingdong prefecture level city in Shandong
Fortune Shop	Green Intelligent Power Saver	It's nice to save a dozen bucks a night on two air conditioners that used to take 30 or 40 degrees a night, but now it's only 10 or so degrees a night	Jingdong prefecture level city in Shandong
Fortune Shop	Green Intelligent Power Saver	Product no manufacturer's name no product qualification certificate, try two days to see the effect of	Jingdong prefecture level city in Shandong
Fortune Shop	Green Intelligent Power Saver	I've been using it for over a month and have saved over $80, so I've repurchased a few more back to save together	Jingdong prefecture level city in Shandong
Fortune Shop	Green Intelligent Power Saver	I haven't used it yet and will comment when I do, but it's well made.	Jingdong prefecture level city in Shandong
Fortune Shop	Green Intelligent Power Saver	It's quite energy efficient. It's really a boon for the home.	Jingdong prefecture level city in Shandong
Fortune Shop	Green Intelligent Power Saver	Energy-saving socket received a month, after using it for a month proved that it is indeed energy-saving, electricity bill saving, will buy it again, thank you!	Jingdong prefecture level city in Shandong

Fortune Shop	Green Intelligent Power Saver	There was a similar product many years ago. Now technology has evolved, buy one to try, according to the instructions in the box, it takes one or two dozen days to show results. Check your electric bill and see what happens.	Jingdong prefecture level city in Shandong
Fortune Shop	Green Intelligent Power Saver	Jingdong logistics is really fast, the same day the order arrived on the same day, more convenient than the supermarket, and a wide range of choices, the	Jingdong prefecture level city in Shandong
Fortune Shop	Green Intelligent Power Saver	I've been using this for a month or so and I can't see any results, it really is a scam as they say.	Jingdong prefecture level city in Shandong
Fortune Shop	Green Intelligent Power Saver	Very good use, a month can save a few degrees of power loss of electricity, introduced to the relatives to buy back to try.	Jingdong prefecture level city in Shandong
Fortune Shop	Green Intelligent Power Saver	It's really powerful! With this air conditioner can be turned on at any time without the pressure of paying the electricity bill! It's good for the people!	Jingdong prefecture level city in Shandong
Fortune Shop	Green Intelligent Power Saver	This business is a big ** products are not qualified, with a month of electricity more than one hundred percent of thirty, the purchase agreed to return the goods but do not receive a refund after receiving the goods, looking for the official side does not help, the Jingdong is a big **, I have never shopped in	Jingdong prefecture level city in Shandong
Fortune Shop	Green Intelligent Power Saver	It's good. I tried it. It saves power.	Jingdong prefecture level city in Shandong
Fortune Shop	Green Intelligent Power Saver	Nothing**, it seems like there's more	Jingdong prefecture level city in Shandong
Fortune Shop	Green Intelligent Power Saver	Power saving device received, well packaged, good quality, fast logistics	Jingdong prefecture level city in Shandong
Fortune Shop	Green Intelligent Power Saver	The service is okay, is that when I bought five, the desirable pieces are three, and then wait a few days and send me two replacement, I do not know if it is a mistake of the shipper, if I was not in the courier there to see, go home to say no, the	Jingdong prefecture level city in Shandong
Fortune Shop	Green Intelligent Power Saver	This thing will only cost me more if I use it, but before I used it, it was less than a ~ electricity a month, and after I used it, it was more than two hundred kWh a month.	Jingdong prefecture level city in Shandong
Fortune Shop	Green Intelligent Power Saver	I just used it. I don't know how it is.	Jingdong prefecture level city in Shandong
Fortune Shop	Green Intelligent Power Saver	I've received the product and I'm using it, so I'll see how it goes in a month!	Jingdong prefecture level city in Shandong
Fortune Shop	Green Intelligent Power Saver	The goods have just received the effect do not know how, please look forward to the results.	Jingdong prefecture level city in Shandong
Fortune Shop	Green Intelligent Power Saver	Power saving device, the material is very good practical and durable, the size is suitable, packaging is very good, the price is cheap, logistics is very fast!	Jingdong prefecture level city in Shandong
Fortune Shop	Green Intelligent Power Saver	Very nice, will comment in detail after using it for a while.	Jingdong prefecture level city in Shandong
Fortune Shop	Green Intelligent Power Saver	Good stuff to trust and support	Jingdong prefecture level city in Shandong
Fortune Shop	Green Intelligent Power Saver	I'll comment after I use it, doubt if it really saves power no	Jingdong prefecture level city in Shandong
Fortune Shop	Green Intelligent Power Saver	It's good quality and works well!	Jingdong prefecture level city in Shandong
Fortune Shop	Green Intelligent Power Saver	hahaha just right hahahahahaha hiccup hahahaha	Jingdong prefecture level city in Shandong
Fortune Shop	Green Intelligent Power Saver	The real thing matches the picture and looks great!	Jingdong prefecture level city in Shandong
Fortune Shop	Green Intelligent Power Saver	Plugged it in and we'll see when we pay the bills at the end of the year!	Jingdong prefecture level city in Shandong
Fortune Shop	Green Intelligent Power Saver	False advertising. It doesn't work.	Jingdong prefecture level city in Shandong
Fortune Shop	Green Intelligent Power Saver	Goods received, good effect	Jingdong prefecture level city in Shandong
Fortune Shop	Green Intelligent Power Saver	It looks okay, we'll see if it saves electricity when the time comes	Jingdong prefecture level city in Shandong
Fortune Shop	Green Intelligent Power Saver	Logistics is very fast quality is very good, the price is very affordable, accessories are also complete, installed, satisfied with good use	Jingdong prefecture level city in Shandong
Fortune Shop	Green Intelligent Power Saver	I've received the power saver. I'm looking forward to the savings.	Jingdong prefecture level city in Shandong
Fortune Shop	Green Intelligent Power Saver	Received, have not yet used, after use and then evaluate	Jingdong prefecture level city in Shandong
Fortune Shop	Green Intelligent Power Saver	I received the item, but I don't know if it saves power or not.	Jingdong prefecture level city in Shandong
Fortune Shop	Green Intelligent Power Saver	On Trial	Jingdong prefecture level city in Shandong
Fortune Shop	Green Intelligent Power Saver	Use it and see how it goes.	Jingdong prefecture level city in Shandong
Fortune Shop	Green Intelligent Power Saver	Quality is very good, no odor, fine workmanship, grain color are very like, satisfied!	Jingdong prefecture level city in Shandong
Fortune Shop	Green Intelligent Power Saver	I'll give you a good review. I don't know if it'll work.	Jingdong prefecture level city in Shandong
Fortune Shop	Green Intelligent Power Saver	The logistics is very fast and the package is intact. Good use of affordable. The effect is very good, is genuine	Jingdong prefecture level city in Shandong
Fortune Shop	Green Intelligent Power Saver	Nothing happens when you plug it in.	Jingdong prefecture level city in Shandong
Fortune Shop	Green Intelligent Power Saver	I don't know if it's as good as it sounds.	Jingdong prefecture level city in Shandong
Fortune Shop	Green Intelligent Power Saver	The quality of the stuff is very good, exactly the same as the seller's description, very satisfied!	Jingdong prefecture level city in Shandong
Fortune Shop	Green Intelligent Power Saver	The saver works great, it really works, it's affordable and worth buying.	Jingdong prefecture level city in Shandong
Fortune Shop	Green Intelligent Power Saver	Just used it for a couple days. I don't know yet.	Jingdong prefecture level city in Shandong
Fortune Shop	Green Intelligent Power Saver	The quality is quite good, cheaper than the physical store, good, next time will come again!	Jingdong prefecture level city in Shandong
Fortune Shop	Green Intelligent Power Saver	Good quality, cheap and affordable worth recommending to buy!	Jingdong prefecture level city in Shandong
Fortune Shop	Green Intelligent Power Saver	Very good quality, well made, works well! Satisfied	Jingdong prefecture level city in Shandong
Fortune Shop	Green Intelligent Power Saver	The logistics is very fast quality is very good very pleasant a shopping	Jingdong prefecture level city in Shandong
Fortune Shop	Green Intelligent Power Saver	Use it and then come back to evaluate!	Jingdong prefecture level city in Shandong
Fortune Shop	Green Intelligent Power Saver	# It's no use lying at all #	Jingdong prefecture level city in Shandong
Fortune Shop	Green Intelligent Power Saver	I can't get a voltage drop.	Jingdong prefecture level city in Shandong
Fortune Shop	Green Intelligent Power Saver	Evaluate after use	Jingdong prefecture level city in Shandong
Fortune Shop	Green Intelligent Power Saver	Still on trial.	Jingdong prefecture level city in Shandong
Fortune Shop	Green Intelligent Power Saver	ineffective	Jingdong prefecture level city in Shandong
Fortune Shop	Green Intelligent Power Saver	Satisfaction Shopping	Jingdong prefecture level city in Shandong

Fortune Shop	Green Intelligent Power Saver	Lies.	Jingdong prefecture level city in Shandong
Fortune Shop	Green Intelligent Power Saver	It's a real power saver.	Jingdong prefecture level city in Shandong
Fortune Shop	Green Intelligent Power Saver	in use	Jingdong prefecture level city in Shandong
Fortune Shop	Green Intelligent Power Saver	I don't know yet.	Jingdong prefecture level city in Shandong
Fortune Shop	Green Intelligent Power Saver	not care about anything	Jingdong prefecture level city in Shandong
Fortune Shop	Green Intelligent Power Saver	It doesn't seem to have much effect	Jingdong prefecture level city in Shandong
Fortune Shop	Green Intelligent Power Saver	Let the merchant see for himself, does the plug positive and negative terminals look like this?	Jingdong prefecture level city in Shandong
Fortune Shop	Green Intelligent Power Saver	It doesn't work well, the electricity bill is less before using it, but more after using it, contacting customer service, the service is still not good, and returning it is slow	Jingdong prefecture level city in Shandong
Fortune Shop	Green Intelligent Power Saver	With the advertisement product does not match, above the effective negative, power saving rate of two rows of words it, the product looks very thin, with 3 days to see the meter number is still with the previous no difference, which is obviously fraudulent behavior!	Jingdong prefecture level city in Shandong
Fortune Shop	Green Intelligent Power Saver	Doesn't save any power at all. Not much use.	Jingdong prefecture level city in Shandong
Fortune Shop	Green Intelligent Power Saver	The plugs are all loose, they're all the same length and they don't work at all	Jingdong prefecture level city in Shandong
Fortune Shop	Green Intelligent Power Saver	It's bad 👎 it's not effective at all, you'd be crazy to sell this garbage on the internet.	Jingdong prefecture level city in Shandong
Fortune Shop	Green Intelligent Power Saver	It doesn't save any power at all, so I hope the old timers don't fall for this scam again	Jingdong prefecture level city in Shandong
Fortune Shop	Green Intelligent Power Saver	I've been using it for over a month now and haven't noticed any power savings.	Jingdong prefecture level city in Shandong
Fortune Shop	Green Intelligent Power Saver	It doesn't work at all. The electricity bill is higher when you use it.	Jingdong prefecture level city in Shandong
Fortune Shop	Green Intelligent Power Saver	It doesn't save any power, it's still as bad as ever.	Jingdong prefecture level city in Shandong
Fortune Shop	Green Intelligent Power Saver	Last night before leaving work to look at the table, and then plugged into the node device, this morning and then look at the table, than the original save a few degrees of electricity, very good to have a power-saving effect, easy to use, satisfied with the shopping praise.	Jingdong prefecture level city in Shandong
Fortune Shop	Green Intelligent Power Saver	The product is real and effective, the electricity bill is reduced, really good, thanks to the merchants have so many good products, the product is real and effective, the electricity bill is reduced, want to save electricity friends quickly buy it, early use early to save money.	Jingdong prefecture level city in Shandong
Fortune Shop	Green Intelligent Power Saver	Saver saves time and money, baby appearance is beautiful, the quality of goods is good, and the same as the business description, good service attitude, packaging is complete, logistics is very fast, praise to	Jingdong prefecture level city in Shandong
Fortune Shop	Green Intelligent Power Saver	The effect is good, summer, home electricity is relatively large, buy and use a few days, feel the effect is very good, originally ten dollars a day electricity bill, now eight dollars a day, quite good	Jingdong prefecture level city in Shandong
Fortune Shop	Green Intelligent Power Saver	Indeed can save electricity and money, the first time I know this or from a friend's home to see, but also do not believe it, and now they use it to know that there is this good thing.	Jingdong prefecture level city in Shandong
Fortune Shop	Green Intelligent Power Saver	I have received the Smart Power Saver. Perfectly packaged. Fast delivery. Boss customer service attitude is also very good. It's worth buying. I plugged it in and tried it and it really saves more power than the original. I'll buy it again next time.	Jingdong prefecture level city in Shandong
Fortune Shop	Green Intelligent Power Saver	It's nice to save a dozen bucks a night on two air conditioners that used to take 30 or 40 degrees a night, but now it's only 10 or so degrees a night	Jingdong prefecture level city in Shandong
Fortune Shop	Green Intelligent Power Saver	Product no manufacturer's name no product qualification certificate, try two days to see the effect of	Jingdong prefecture level city in Shandong
Fortune Shop	Green Intelligent Power Saver	I've been using it for over a month and have saved over $80, so I've repurchased a few more back to save together	Jingdong prefecture level city in Shandong
Fortune Shop	Green Intelligent Power Saver	I haven't used it yet and will comment when I do, but it's well made.	Jingdong prefecture level city in Shandong
Fortune Shop	Green Intelligent Power Saver	It's quite energy efficient. It's really a boon for the home.	Jingdong prefecture level city in Shandong
Fortune Shop	Green Intelligent Power Saver	Energy-saving socket received a month, after using it for a month proved that it is indeed energy-saving, electricity bill saving, will buy it again, thank you!	Jingdong prefecture level city in Shandong
Fortune Shop	Green Intelligent Power Saver	There was a similar product many years ago. Now technology has evolved, buy one to try, according to the instructions in the box, it takes one or two dozen days to show results. Check your electric bill and see what happens.	Jingdong prefecture level city in Shandong
Fortune Shop	Green Intelligent Power Saver	Jingdong logistics is really fast, the same day the order arrived on the same day, more convenient than the supermarket, and a wide range of choices, the	Jingdong prefecture level city in Shandong
Fortune Shop	Green Intelligent Power Saver	I've been using this for a month or so and I can't see any results, it really is a scam as they say.	Jingdong prefecture level city in Shandong
Fortune Shop	Green Intelligent Power Saver	Very good use, a month can save a few degrees of power loss of electricity, introduced to the relatives to buy back to try.	Jingdong prefecture level city in Shandong
Fortune Shop	Green Intelligent Power Saver	It's really powerful! With this air conditioner can be turned on at any time without the pressure of paying the electricity bill! It's good for the people!	Jingdong prefecture level city in Shandong
Fortune Shop	Green Intelligent Power Saver	This business is a big ** products are not qualified, with a month of electricity more than one hundred percent of thirty, the purchase agreed to return the goods but do not receive a refund after receiving the goods, looking for the official side does not help, the Jingdong is a big **, I have never shopped in	Jingdong prefecture level city in Shandong
Fortune Shop	Green Intelligent Power Saver	It's good. I tried it. It saves power.	Jingdong prefecture level city in Shandong
Fortune Shop	Green Intelligent Power Saver	Nothing**, it seems like there's more	Jingdong prefecture level city in Shandong
Fortune Shop	Green Intelligent Power Saver	Power saving device received, well packaged, good quality, fast logistics	Jingdong prefecture level city in Shandong
Fortune Shop	Green Intelligent Power Saver	The service is okay, is that when I bought five, the desirable pieces are three, and then wait a few days and send me two replacement, I do not know if it is a mistake of the shipper, if I was not in the courier there to see, go home to say no, the	Jingdong prefecture level city in Shandong
Fortune Shop	Green Intelligent Power Saver	This thing will only cost me more if I use it, but before I used it, it was less than a ~ electricity a month, and after I used it, it was more than two hundred kWh a month.	Jingdong prefecture level city in Shandong
Fortune Shop	Green Intelligent Power Saver	I just used it. I don't know how it is.	Jingdong prefecture level city in Shandong
Fortune Shop	Green Intelligent Power Saver	I've received the product and I'm using it, so I'll see how it goes in a month!	Jingdong prefecture level city in Shandong
Fortune Shop	Green Intelligent Power Saver	The goods have just received the effect do not know how, please look forward to the results.	Jingdong prefecture level city in Shandong
Fortune Shop	Green Intelligent Power Saver	Power saving device, the material is very good practical and durable, the size is suitable, packaging is very good, the price is cheap, logistics is very fast!	Jingdong prefecture level city in Shandong
Fortune Shop	Green Intelligent Power Saver	Very nice, will comment in detail after using it for a while.	Jingdong prefecture level city in Shandong
Fortune Shop	Green Intelligent Power Saver	Good stuff to trust and support	Jingdong prefecture level city in Shandong
Fortune Shop	Green Intelligent Power Saver	I'll comment after I use it, doubt if it really saves power no	Jingdong prefecture level city in Shandong
Fortune Shop	Green Intelligent Power Saver	It's good quality and works well!	Jingdong prefecture level city in Shandong
Fortune Shop	Green Intelligent Power Saver	hahaha just right hahahahahaha hiccup hahahaha	Jingdong prefecture level city in Shandong

Fortune Shop	Green Intelligent Power Saver	The real thing matches the picture and looks great!	Jingdong prefecture level city in Shandong
Fortune Shop	Green Intelligent Power Saver	Plugged it in and we'll see when we pay the bills at the end of the year!	Jingdong prefecture level city in Shandong
Fortune Shop	Green Intelligent Power Saver	False advertising. It doesn't work.	Jingdong prefecture level city in Shandong
Fortune Shop	Green Intelligent Power Saver	Goods received, good effect	Jingdong prefecture level city in Shandong
Fortune Shop	Green Intelligent Power Saver	It looks okay, we'll see if it saves electricity when the time comes	Jingdong prefecture level city in Shandong
Fortune Shop	Green Intelligent Power Saver	Logistics is very fast quality is very good, the price is very affordable, accessories are also complete, installed, satisfied with good use	Jingdong prefecture level city in Shandong
Fortune Shop	Green Intelligent Power Saver	I've received the power saver. I'm looking forward to the savings.	Jingdong prefecture level city in Shandong
Fortune Shop	Green Intelligent Power Saver	Received, have not yet used, after use and then evaluate	Jingdong prefecture level city in Shandong
Fortune Shop	Green Intelligent Power Saver	I received the item, but I don't know if it saves power or not.	Jingdong prefecture level city in Shandong
Fortune Shop	Green Intelligent Power Saver	On Trial	Jingdong prefecture level city in Shandong
Fortune Shop	Green Intelligent Power Saver	Use it and see how it goes.	Jingdong prefecture level city in Shandong
Fortune Shop	Green Intelligent Power Saver	Quality is very good, no odor, fine workmanship, grain color are very like, satisfied!	Jingdong prefecture level city in Shandong
Fortune Shop	Green Intelligent Power Saver	I'll give you a good review. I don't know if it'll work.	Jingdong prefecture level city in Shandong
Fortune Shop	Green Intelligent Power Saver	The logistics is very fast and the package is intact. Good use of affordable. The effect is very good, is genuine	Jingdong prefecture level city in Shandong
Fortune Shop	Green Intelligent Power Saver	Nothing happens when you plug it in.	Jingdong prefecture level city in Shandong
Fortune Shop	Green Intelligent Power Saver	I don't know if it's as good as it sounds.	Jingdong prefecture level city in Shandong
Fortune Shop	Green Intelligent Power Saver	The quality of the stuff is very good, exactly the same as the seller's description, very satisfied!	Jingdong prefecture level city in Shandong
Fortune Shop	Green Intelligent Power Saver	The saver works great, it really works, it's affordable and worth buying.	Jingdong prefecture level city in Shandong
Fortune Shop	Green Intelligent Power Saver	Just used it for a couple days. I don't know yet.	Jingdong prefecture level city in Shandong
Fortune Shop	Green Intelligent Power Saver	The quality is quite good, cheaper than the physical store, good, next time will come again!	Jingdong prefecture level city in Shandong
Fortune Shop	Green Intelligent Power Saver	Good quality, cheap and affordable worth recommending to buy!	Jingdong prefecture level city in Shandong
Fortune Shop	Green Intelligent Power Saver	Very good quality, well made, works well! Satisfied	Jingdong prefecture level city in Shandong
Fortune Shop	Green Intelligent Power Saver	The logistics is very fast quality is very good very pleasant a shopping	Jingdong prefecture level city in Shandong
Fortune Shop	Green Intelligent Power Saver	Use it and then come back to evaluate!	Jingdong prefecture level city in Shandong
Fortune Shop	Green Intelligent Power Saver	# It's no use lying at all #	Jingdong prefecture level city in Shandong
Fortune Shop	Green Intelligent Power Saver	I can't get a voltage drop.	Jingdong prefecture level city in Shandong
Fortune Shop	Green Intelligent Power Saver	Evaluate after use	Jingdong prefecture level city in Shandong
Fortune Shop	Green Intelligent Power Saver	Still on trial.	Jingdong prefecture level city in Shandong
Fortune Shop	Green Intelligent Power Saver	ineffective	Jingdong prefecture level city in Shandong
Fortune Shop	Green Intelligent Power Saver	Satisfaction Shopping	Jingdong prefecture level city in Shandong
Fortune Shop	Green Intelligent Power Saver	Lies.	Jingdong prefecture level city in Shandong
Fortune Shop	Green Intelligent Power Saver	It's a real power saver.	Jingdong prefecture level city in Shandong
Fortune Shop	Green Intelligent Power Saver	in use	Jingdong prefecture level city in Shandong
Fortune Shop	Green Intelligent Power Saver	I don't know yet.	Jingdong prefecture level city in Shandong
Fortune Shop	Green Intelligent Power Saver	not care about anything	Jingdong prefecture level city in Shandong
Fortune Shop	Green Intelligent Power Saver	It doesn't seem to have much effect	Jingdong prefecture level city in Shandong
Fortune Shop	Green Intelligent Power Saver	Let the merchant see for himself, does the plug positive and negative terminals look like this?	Jingdong prefecture level city in Shandong
Fortune Shop	Green Intelligent Power Saver	It doesn't work well, the electricity bill is less before using it, but more after using it, contacting customer service, the service is still not good, and returning it is slow	Jingdong prefecture level city in Shandong
Fortune Shop	Green Intelligent Power Saver	With the advertisement product does not match, above the effective negative, power saving rate of two rows of words it, the product looks very thin, with 3 days to see the meter number is still with the previous no difference, which is obviously fraudulent behavior!	Jingdong prefecture level city in Shandong
Fortune Shop	Green Intelligent Power Saver	Doesn't save any power at all. Not much use.	Jingdong prefecture level city in Shandong
Fortune Shop	Green Intelligent Power Saver	The plugs are all loose, they're all the same length and they don't work at all	Jingdong prefecture level city in Shandong
Fortune Shop	Green Intelligent Power Saver	It's bad 👎 it's not effective at all, you'd be crazy to sell this garbage on the internet.	Jingdong prefecture level city in Shandong
Fortune Shop	Green Intelligent Power Saver	It doesn't save any power at all, so I hope the old timers don't fall for this scam again	Jingdong prefecture level city in Shandong
Fortune Shop	Green Intelligent Power Saver	I've been using it for over a month now and haven't noticed any power savings.	Jingdong prefecture level city in Shandong
Fortune Shop	Green Intelligent Power Saver	It doesn't work at all. The electricity bill is higher when you use it.	Jingdong prefecture level city in Shandong
Fortune Shop	Green Intelligent Power Saver	It doesn't save any power, it's still as bad as ever.	Jingdong prefecture level city in Shandong
Fortune Shop	Green Intelligent Power Saver	Last night before leaving work to look at the table, and then plugged into the node device, this morning and then look at the table, than the original save a few degrees of electricity, very good to have a power-saving effect, easy to use, satisfied with the shopping praise.	Jingdong prefecture level city in Shandong
Fortune Shop	Green Intelligent Power Saver	The product is real and effective, the electricity bill is reduced, really good, thanks to the merchants have so many good products, the product is real and effective, the electricity bill is reduced, want to save electricity friends quickly buy it, early use early to save money.	Jingdong prefecture level city in Shandong
Fortune Shop	Green Intelligent Power Saver	Saver saves time and money, baby appearance is beautiful, the quality of goods is good, and the same as the business description, good service attitude, packaging is complete, logistics is very fast, praise to	Jingdong prefecture level city in Shandong
Fortune Shop	Green Intelligent Power Saver	The effect is good, summer, home electricity is relatively large, buy and use a few days, feel the effect is very good, originally ten dollars a day electricity bill, now eight dollars a day, quite good	Jingdong prefecture level city in Shandong

Fortune Shop	Green Intelligent Power Saver	Indeed can save electricity and money, the first time I know this or from a friend's home to see, but also do not believe it, and now they use it to know that there is this good thing.	Jingdong prefecture level city in Shandong
Fortune Shop	Green Intelligent Power Saver	I have received the Smart Power Saver. Perfectly packaged. Fast delivery. Boss customer service attitude is also very good. It's worth buying. I plugged it in and tried it and it really saves more power than the original. I'll buy it again next time.	Jingdong prefecture level city in Shandong
Fortune Shop	Green Intelligent Power Saver	It's nice to save a dozen bucks a night on two air conditioners that used to take 30 or 40 degrees a night, but now it's only 10 or so degrees a night	Jingdong prefecture level city in Shandong
Fortune Shop	Green Intelligent Power Saver	Product no manufacturer's name no product qualification certificate, try two days to see the effect of	Jingdong prefecture level city in Shandong
Fortune Shop	Green Intelligent Power Saver	I've been using it for over a month and have saved over $80, so I've repurchased a few more back to save together	Jingdong prefecture level city in Shandong
Fortune Shop	Green Intelligent Power Saver	I haven't used it yet and will comment when I do, but it's well made.	Jingdong prefecture level city in Shandong
Fortune Shop	Green Intelligent Power Saver	It's quite energy efficient. It's really a boon for the home.	Jingdong prefecture level city in Shandong
Fortune Shop	Green Intelligent Power Saver	Energy-saving socket received a month, after using it for a month proved that it is indeed energy-saving, electricity bill saving, will buy it again, thank you!	Jingdong prefecture level city in Shandong
Fortune Shop	Green Intelligent Power Saver	There was a similar product many years ago. Now technology has evolved, buy one to try, according to the instructions in the box, it takes one or two dozen days to show results. Check your electric bill and see what happens.	Jingdong prefecture level city in Shandong
Fortune Shop	Green Intelligent Power Saver	Jingdong logistics is really fast, the same day the order arrived on the same day, more convenient than the supermarket, and a wide range of choices, the	Jingdong prefecture level city in Shandong
Fortune Shop	Green Intelligent Power Saver	I've been using this for a month or so and I can't see any results, it really is a scam as they say.	Jingdong prefecture level city in Shandong
Fortune Shop	Green Intelligent Power Saver	Very good use, a month can save a few degrees of power loss of electricity, introduced to the relatives to buy back to try.	Jingdong prefecture level city in Shandong
Fortune Shop	Green Intelligent Power Saver	It's really powerful! With this air conditioner can be turned on at any time without the pressure of paying the electricity bill! It's good for the people!	Jingdong prefecture level city in Shandong
Fortune Shop	Green Intelligent Power Saver	This business is a big ** products are not qualified, with a month of electricity more than one hundred percent of thirty, the purchase agreed to return the goods but do not receive a refund after receiving the goods, looking for the official side does not help, the Jingdong is a big **, I have never shopped in	Jingdong prefecture level city in Shandong
Fortune Shop	Green Intelligent Power Saver	It's good. I tried it. It saves power.	Jingdong prefecture level city in Shandong
Fortune Shop	Green Intelligent Power Saver	Nothing**, it seems like there's more	Jingdong prefecture level city in Shandong
Fortune Shop	Green Intelligent Power Saver	Power saving device received, well packaged, good quality, fast logistics	Jingdong prefecture level city in Shandong
Fortune Shop	Green Intelligent Power Saver	The service is okay, is that when I bought five, the desirable pieces are three, and then wait a few days and send me two replacement, I do not know if it is a mistake of the shipper, if I was not in the courier there to see, go home to say no, the	Jingdong prefecture level city in Shandong
Fortune Shop	Green Intelligent Power Saver	This thing will only cost me more if I use it, but before I used it, it was less than a ~ electricity a month, and after I used it, it was more than two hundred kWh a month.	Jingdong prefecture level city in Shandong
Fortune Shop	Green Intelligent Power Saver	I just used it. I don't know how it is.	Jingdong prefecture level city in Shandong
Fortune Shop	Green Intelligent Power Saver	I've received the product and I'm using it, so I'll see how it goes in a month!	Jingdong prefecture level city in Shandong
Fortune Shop	Green Intelligent Power Saver	The goods have just received the effect do not know how, please look forward to the results.	Jingdong prefecture level city in Shandong
Fortune Shop	Green Intelligent Power Saver	Power saving device, the material is very good practical and durable, the size is suitable, packaging is very good, the price is cheap, logistics is very fast!	Jingdong prefecture level city in Shandong
Fortune Shop	Green Intelligent Power Saver	Very nice, will comment in detail after using it for a while.	Jingdong prefecture level city in Shandong
Fortune Shop	Green Intelligent Power Saver	Good stuff to trust and support	Jingdong prefecture level city in Shandong
Fortune Shop	Green Intelligent Power Saver	I'll comment after I use it, doubt if it really saves power no	Jingdong prefecture level city in Shandong
Fortune Shop	Green Intelligent Power Saver	It's good quality and works well!	Jingdong prefecture level city in Shandong
Fortune Shop	Green Intelligent Power Saver	hahaha just right hahahahahaha hiccup hahahaha	Jingdong prefecture level city in Shandong
Fortune Shop	Green Intelligent Power Saver	The real thing matches the picture and looks great!	Jingdong prefecture level city in Shandong
Fortune Shop	Green Intelligent Power Saver	Plugged it in and we'll see when we pay the bills at the end of the year!	Jingdong prefecture level city in Shandong
Fortune Shop	Green Intelligent Power Saver	False advertising. It doesn't work.	Jingdong prefecture level city in Shandong
Fortune Shop	Green Intelligent Power Saver	Goods received, good effect	Jingdong prefecture level city in Shandong
Fortune Shop	Green Intelligent Power Saver	It looks okay, we'll see if it saves electricity when the time comes	Jingdong prefecture level city in Shandong
Fortune Shop	Green Intelligent Power Saver	Logistics is very fast quality is very good, the price is very affordable, accessories are also complete, installed, satisfied with good use	Jingdong prefecture level city in Shandong
Fortune Shop	Green Intelligent Power Saver	I've received the power saver. I'm looking forward to the savings.	Jingdong prefecture level city in Shandong
Fortune Shop	Green Intelligent Power Saver	Received, have not yet used, after use and then evaluate	Jingdong prefecture level city in Shandong
Fortune Shop	Green Intelligent Power Saver	I received the item, but I don't know if it saves power or not.	Jingdong prefecture level city in Shandong
Fortune Shop	Green Intelligent Power Saver	On Trial	Jingdong prefecture level city in Shandong
Fortune Shop	Green Intelligent Power Saver	Use it and see how it goes.	Jingdong prefecture level city in Shandong
Fortune Shop	Green Intelligent Power Saver	Quality is very good, no odor, fine workmanship, grain color are very like, satisfied!	Jingdong prefecture level city in Shandong
Fortune Shop	Green Intelligent Power Saver	I'll give you a good review. I don't know if it'll work.	Jingdong prefecture level city in Shandong
Fortune Shop	Green Intelligent Power Saver	The logistics is very fast and the package is intact. Good use of affordable. The effect is very good, is genuine	Jingdong prefecture level city in Shandong
Fortune Shop	Green Intelligent Power Saver	Nothing happens when you plug it in.	Jingdong prefecture level city in Shandong
Fortune Shop	Green Intelligent Power Saver	I don't know if it's as good as it sounds.	Jingdong prefecture level city in Shandong
Fortune Shop	Green Intelligent Power Saver	The quality of the stuff is very good, exactly the same as the seller's description, very satisfied!	Jingdong prefecture level city in Shandong
Fortune Shop	Green Intelligent Power Saver	The saver works great, it really works, it's affordable and worth buying.	Jingdong prefecture level city in Shandong
Fortune Shop	Green Intelligent Power Saver	Just used it for a couple days. I don't know yet.	Jingdong prefecture level city in Shandong
Fortune Shop	Green Intelligent Power Saver	The quality is quite good, cheaper than the physical store, good, next time will come again!	Jingdong prefecture level city in Shandong
Fortune Shop	Green Intelligent Power Saver	Good quality, cheap and affordable worth recommending to buy!	Jingdong prefecture level city in Shandong

Fortune Shop	Green Intelligent Power Saver	Very good quality, well made, works well! Satisfied	Jingdong prefecture level city in Shandong
Fortune Shop	Green Intelligent Power Saver	The logistics is very fast quality is very good very pleasant a shopping	Jingdong prefecture level city in Shandong
Fortune Shop	Green Intelligent Power Saver	Use it and then come back to evaluate!	Jingdong prefecture level city in Shandong
Fortune Shop	Green Intelligent Power Saver	# It's no use lying at all #	Jingdong prefecture level city in Shandong
Fortune Shop	Green Intelligent Power Saver	I can't get a voltage drop.	Jingdong prefecture level city in Shandong
Fortune Shop	Green Intelligent Power Saver	Evaluate after use	Jingdong prefecture level city in Shandong
Fortune Shop	Green Intelligent Power Saver	Still on trial.	Jingdong prefecture level city in Shandong
Fortune Shop	Green Intelligent Power Saver	ineffective	Jingdong prefecture level city in Shandong
Fortune Shop	Green Intelligent Power Saver	Satisfaction Shopping	Jingdong prefecture level city in Shandong
Fortune Shop	Green Intelligent Power Saver	Lies.	Jingdong prefecture level city in Shandong
Fortune Shop	Green Intelligent Power Saver	It's a real power saver.	Jingdong prefecture level city in Shandong
Fortune Shop	Green Intelligent Power Saver	in use	Jingdong prefecture level city in Shandong
Fortune Shop	Green Intelligent Power Saver	I don't know yet.	Jingdong prefecture level city in Shandong
Fortune Shop	Green Intelligent Power Saver	not care about anything	Jingdong prefecture level city in Shandong
Fortune Shop	Green Intelligent Power Saver	It doesn't seem to have much effect	Jingdong prefecture level city in Shandong
Fortune Shop	Green Intelligent Power Saver	Let the merchant see for himself, does the plug positive and negative terminals look like this?	Jingdong prefecture level city in Shandong
Fortune Shop	Green Intelligent Power Saver	It doesn't work well, the electricity bill is less before using it, but more after using it, contacting customer service, the service is still not good, and returning it is slow	Jingdong prefecture level city in Shandong
Fortune Shop	Green Intelligent Power Saver	With the advertisement product does not match, above the effective negative, power saving rate of two rows of words it, the product looks very thin, with 3 days to see the meter number is still with the previous no difference, which is obviously fraudulent behavior!	Jingdong prefecture level city in Shandong
Fortune Shop	Green Intelligent Power Saver	Doesn't save any power at all. Not much use.	Jingdong prefecture level city in Shandong
Fortune Shop	Green Intelligent Power Saver	The plugs are all loose, they're all the same length and they don't work at all	Jingdong prefecture level city in Shandong
Fortune Shop	Green Intelligent Power Saver	It's bad 👎 it's not effective at all, you'd be crazy to sell this garbage on the internet.	Jingdong prefecture level city in Shandong
Fortune Shop	Green Intelligent Power Saver	It doesn't save any power at all, so I hope the old timers don't fall for this scam again	Jingdong prefecture level city in Shandong
Fortune Shop	Green Intelligent Power Saver	I've been using it for over a month now and haven't noticed any power savings.	Jingdong prefecture level city in Shandong
Fortune Shop	Green Intelligent Power Saver	It doesn't work at all. The electricity bill is higher when you use it.	Jingdong prefecture level city in Shandong
Fortune Shop	Green Intelligent Power Saver	It doesn't save any power, it's still as bad as ever.	Jingdong prefecture level city in Shandong
Fortune Shop	Green Intelligent Power Saver	Last night before leaving work to look at the table, and then plugged into the node device, this morning and then look at the table, than the original save a few degrees of electricity, very good to have a power-saving effect, easy to use, satisfied with the shopping praise.	Jingdong prefecture level city in Shandong
Fortune Shop	Green Intelligent Power Saver	The product is real and effective, the electricity bill is reduced, really good, thanks to the merchants have so many good products, the product is real and effective, the electricity bill is reduced, want to save electricity friends quickly buy it, early use early to save money.	Jingdong prefecture level city in Shandong
Fortune Shop	Green Intelligent Power Saver	Saver saves time and money, baby appearance is beautiful, the quality of goods is good, and the same as the business description, good service attitude, packaging is complete, logistics is very fast, praise to	Jingdong prefecture level city in Shandong
Fortune Shop	Green Intelligent Power Saver	The effect is good, summer, home electricity is relatively large, buy and use a few days, feel the effect is very good, originally ten dollars a day electricity bill, now eight dollars a day, quite good	Jingdong prefecture level city in Shandong
Fortune Shop	Green Intelligent Power Saver	Indeed can save electricity and money, the first time I know this or from a friend's home to see, but also do not believe it, and now they use it to know that there is this good thing.	Jingdong prefecture level city in Shandong
Fortune Shop	Green Intelligent Power Saver	I have received the Smart Power Saver. Perfectly packaged. Fast delivery. Boss customer service attitude is also very good. It's worth buying. I plugged it in and tried it and it really saves more power than the original. I'll buy it again next time.	Jingdong prefecture level city in Shandong
Fortune Shop	Green Intelligent Power Saver	It's nice to save a dozen bucks a night on two air conditioners that used to take 30 or 40 degrees a night, but now it's only 10 or so degrees a night	Jingdong prefecture level city in Shandong
Fortune Shop	Green Intelligent Power Saver	Product no manufacturer's name no product qualification certificate, try two days to see the effect of	Jingdong prefecture level city in Shandong
Fortune Shop	Green Intelligent Power Saver	I've been using it for over a month and have saved over $80, so I've repurchased a few more back to save together	Jingdong prefecture level city in Shandong
Fortune Shop	Green Intelligent Power Saver	I haven't used it yet and will comment when I do, but it's well made.	Jingdong prefecture level city in Shandong
Fortune Shop	Green Intelligent Power Saver	It's quite energy efficient. It's really a boon for the home.	Jingdong prefecture level city in Shandong
Fortune Shop	Green Intelligent Power Saver	Energy-saving socket received a month, after using it for a month proved that it is indeed energy-saving, electricity bill saving, will buy it again, thank you!	Jingdong prefecture level city in Shandong
Fortune Shop	Green Intelligent Power Saver	There was a similar product many years ago. Now technology has evolved, buy one to try, according to the instructions in the box, it takes one or two dozen days to show results. Check your electric bill and see what happens.	Jingdong prefecture level city in Shandong
Fortune Shop	Green Intelligent Power Saver	Jingdong logistics is really fast, the same day the order arrived on the same day, more convenient than the supermarket, and a wide range of choices, the	Jingdong prefecture level city in Shandong
Fortune Shop	Green Intelligent Power Saver	I've been using this for a month or so and I can't see any results, it really is a scam as they say.	Jingdong prefecture level city in Shandong
Fortune Shop	Green Intelligent Power Saver	Very good use, a month can save a few degrees of power loss of electricity, introduced to the relatives to buy back to try.	Jingdong prefecture level city in Shandong
Fortune Shop	Green Intelligent Power Saver	It's really powerful! With this air conditioner can be turned on at any time without the pressure of paying the electricity bill! It's good for the people!	Jingdong prefecture level city in Shandong
Fortune Shop	Green Intelligent Power Saver	This business is a big ** products are not qualified, with a month of electricity more than one hundred percent of thirty, the purchase agreed to return the goods but do not receive a refund after receiving the goods, looking for the official side does not help, the Jingdong is a big **, I have never shopped in	Jingdong prefecture level city in Shandong
Fortune Shop	Green Intelligent Power Saver	It's good. I tried it. It saves power.	Jingdong prefecture level city in Shandong
Fortune Shop	Green Intelligent Power Saver	Nothing**, it seems like there's more	Jingdong prefecture level city in Shandong
Fortune Shop	Green Intelligent Power Saver	Power saving device received, well packaged, good quality, fast logistics	Jingdong prefecture level city in Shandong
Fortune Shop	Green Intelligent Power Saver	The service is okay, is that when I bought five, the desirable pieces are three, and then wait a few days and send me two replacement, I do not know if it is a mistake of the shipper, if I was not in the courier there to see, go home to say no, the	Jingdong prefecture level city in Shandong
Fortune Shop	Green Intelligent Power Saver	This thing will only cost me more if I use it, but before I used it, it was less than a ~ electricity a month, and after I used it, it was more than two hundred kWh a month.	Jingdong prefecture level city in Shandong

Fortune Shop	Green Intelligent Power Saver	I just used it. I don't know how it is.	Jingdong prefecture level city in Shandong
Fortune Shop	Green Intelligent Power Saver	I've received the product and I'm using it, so I'll see how it goes in a month!	Jingdong prefecture level city in Shandong
Fortune Shop	Green Intelligent Power Saver	The goods have just received the effect do not know how, please look forward to the results.	Jingdong prefecture level city in Shandong
Fortune Shop	Green Intelligent Power Saver	Power saving device, the material is very good practical and durable, the size is suitable, packaging is very good, the price is cheap, logistics is very fast!	Jingdong prefecture level city in Shandong
Fortune Shop	Green Intelligent Power Saver	Very nice, will comment in detail after using it for a while.	Jingdong prefecture level city in Shandong
Fortune Shop	Green Intelligent Power Saver	Good stuff to trust and support	Jingdong prefecture level city in Shandong
Fortune Shop	Green Intelligent Power Saver	I'll comment after I use it, doubt if it really saves power no	Jingdong prefecture level city in Shandong
Fortune Shop	Green Intelligent Power Saver	It's good quality and works well!	Jingdong prefecture level city in Shandong
Fortune Shop	Green Intelligent Power Saver	hahaha just right hahahahahaha hiccup hahahaha	Jingdong prefecture level city in Shandong
Fortune Shop	Green Intelligent Power Saver	The real thing matches the picture and looks great!	Jingdong prefecture level city in Shandong
Fortune Shop	Green Intelligent Power Saver	Plugged it in and we'll see when we pay the bills at the end of the year!	Jingdong prefecture level city in Shandong
Fortune Shop	Green Intelligent Power Saver	False advertising. It doesn't work.	Jingdong prefecture level city in Shandong
Fortune Shop	Green Intelligent Power Saver	Goods received, good effect	Jingdong prefecture level city in Shandong
Fortune Shop	Green Intelligent Power Saver	It looks okay, we'll see if it saves electricity when the time comes	Jingdong prefecture level city in Shandong
Fortune Shop	Green Intelligent Power Saver	Last night before leaving work to look at the table, and then plugged into the node device, this morning and then look at the table, than the original save a few degrees of electricity, very good to have a power-saving effect, easy to use, satisfied with the shopping praise.	Jingdong prefecture level city in Shandong
Fortune Shop	Green Intelligent Power Saver	The product is real and effective, the electricity bill is reduced, really good, thanks to the merchants have so many good products, the product is real and effective, the electricity bill is reduced, want to save electricity friends quickly buy it, early use early to save money.	Jingdong prefecture level city in Shandong
Fortune Shop	Green Intelligent Power Saver	Saver saves time and money, baby appearance is beautiful, the quality of goods is good, and the same as the business description, good service attitude, packaging is complete, logistics is very fast, praise to	Jingdong prefecture level city in Shandong
Fortune Shop	Green Intelligent Power Saver	The effect is good, summer, home electricity is relatively large, buy and use a few days, feel the effect is very good, originally ten dollars a day electricity bill, now eight dollars a day, quite good	Jingdong prefecture level city in Shandong
Fortune Shop	Green Intelligent Power Saver	Indeed can save electricity and money, the first time I know this or from a friend's home to see, but also do not believe it, and now they use it to know that there is this good thing.	Jingdong prefecture level city in Shandong
Fortune Shop	Green Intelligent Power Saver	I have received the Smart Power Saver. Perfectly packaged. Fast delivery. Boss customer service attitude is also very good. It's worth buying. I plugged it in and tried it and it really saves more power than the original. I'll buy it again next time.	Jingdong prefecture level city in Shandong
Fortune Shop	Green Intelligent Power Saver	It's nice to save a dozen bucks a night on two air conditioners that used to take 30 or 40 degrees a night, but now it's only 10 or so degrees a night	Jingdong prefecture level city in Shandong
Fortune Shop	Green Intelligent Power Saver	Product no manufacturer's name no product qualification certificate, try two days to see the effect of	Jingdong prefecture level city in Shandong
Fortune Shop	Green Intelligent Power Saver	I've been using it for over a month and have saved over $80, so I've repurchased a few more back to save together	Jingdong prefecture level city in Shandong
Fortune Shop	Green Intelligent Power Saver	I haven't used it yet and will comment when I do, but it's well made.	Jingdong prefecture level city in Shandong
Fortune Shop	Green Intelligent Power Saver	Last night before leaving work to look at the table, and then plugged into the node device, this morning and then look at the table, than the original save a few degrees of electricity, very good to have a power-saving effect, easy to use, satisfied with the shopping praise.	Jingdong prefecture level city in Shandong
Fortune Shop	Green Intelligent Power Saver	The product is real and effective, the electricity bill is reduced, really good, thanks to the merchants have so many good products, the product is real and effective, the electricity bill is reduced, want to save electricity friends quickly buy it, early use early to save money.	Jingdong prefecture level city in Shandong
Fortune Shop	Green Intelligent Power Saver	Saver saves time and money, baby appearance is beautiful, the quality of goods is good, and the same as the business description, good service attitude, packaging is complete, logistics is very fast, praise to	Jingdong prefecture level city in Shandong
Fortune Shop	Green Intelligent Power Saver	The effect is good, summer, home electricity is relatively large, buy and use a few days, feel the effect is very good, originally ten dollars a day electricity bill, now eight dollars a day, quite good	Jingdong prefecture level city in Shandong
Fortune Shop	Green Intelligent Power Saver	Indeed can save electricity and money, the first time I know this or from a friend's home to see, but also do not believe it, and now they use it to know that there is this good thing.	Jingdong prefecture level city in Shandong
Fortune Shop	Green Intelligent Power Saver	I have received the Smart Power Saver. Perfectly packaged. Fast delivery. Boss customer service attitude is also very good. It's worth buying. I plugged it in and tried it and it really saves more power than the original. I'll buy it again next time.	Jingdong prefecture level city in Shandong
Fortune Shop	Green Intelligent Power Saver	It's nice to save a dozen bucks a night on two air conditioners that used to take 30 or 40 degrees a night, but now it's only 10 or so degrees a night	Jingdong prefecture level city in Shandong
Fortune Shop	Green Intelligent Power Saver	Product no manufacturer's name no product qualification certificate, try two days to see the effect of	Jingdong prefecture level city in Shandong
Fortune Shop	Green Intelligent Power Saver	I've been using it for over a month and have saved over $80, so I've repurchased a few more back to save together	Jingdong prefecture level city in Shandong
Fortune Shop	Green Intelligent Power Saver	I haven't used it yet and will comment when I do, but it's well made.	Jingdong prefecture level city in Shandong
Fortune Shop	Green Intelligent Power Saver	It's quite energy efficient. It's really a boon for the home.	Jingdong prefecture level city in Shandong
Fortune Shop	Green Intelligent Power Saver	Energy-saving socket received a month, after using it for a month proved that it is indeed energy-saving, electricity bill saving, will buy it again, thank you!	Jingdong prefecture level city in Shandong
Fortune Shop	Green Intelligent Power Saver	There was a similar product many years ago. Now technology has evolved, buy one to try, according to the instructions in the box, it takes one or two dozen days to show results. Check your electric bill and see what happens.	Jingdong prefecture level city in Shandong
Fortune Shop	Green Intelligent Power Saver	Jingdong logistics is really fast, the same day the order arrived on the same day, more convenient than the supermarket, and a wide range of choices, the	Jingdong prefecture level city in Shandong
Fortune Shop	Green Intelligent Power Saver	I've been using this for a month or so and I can't see any results, it really is a scam as they say.	Jingdong prefecture level city in Shandong
Fortune Shop	Green Intelligent Power Saver	Very good use, a month can save a few degrees of power loss of electricity, introduced to the relatives to buy back to try.	Jingdong prefecture level city in Shandong
Fortune Shop	Green Intelligent Power Saver	It's really powerful! With this air conditioner can be turned on at any time without the pressure of paying the electricity bill! It's good for the people!	Jingdong prefecture level city in Shandong
Fortune Shop	Green Intelligent Power Saver	This business is a big ** products are not qualified, with a month of electricity more than one hundred percent of thirty, the purchase agreed to return the goods but do not receive a refund after receiving the goods, looking for the official side does not help, the Jingdong is a big **, I have never shopped in	Jingdong prefecture level city in Shandong
Fortune Shop	Green Intelligent Power Saver	It's good. I tried it. It saves power.	Jingdong prefecture level city in Shandong
Fortune Shop	Green Intelligent Power Saver	Nothing**, it seems like there's more	Jingdong prefecture level city in Shandong
Fortune Shop	Green Intelligent Power Saver	Power saving device received, well packaged, good quality, fast logistics	Jingdong prefecture level city in Shandong
Fortune Shop	Green Intelligent Power Saver	The service is okay, is that when I bought five, the desirable pieces are three, and then wait a few days and send me two replacement, I do not know if it is a mistake of the shipper, if I was not in the courier there to see, go home to say no, the	Jingdong prefecture level city in Shandong
Fortune Shop	Green Intelligent Power Saver	This thing will only cost me more if I use it, but before I used it, it was less than a ~ electricity a month, and after I used it, it was more than two hundred kWh a month.	Jingdong prefecture level city in Shandong

Fortune Shop	Green Intelligent Power Saver	I just used it. I don't know how it is.	Jingdong prefecture level city in Shandong
Fortune Shop	Green Intelligent Power Saver	I've received the product and I'm using it, so I'll see how it goes in a month!	Jingdong prefecture level city in Shandong
Fortune Shop	Green Intelligent Power Saver	The goods have just received the effect do not know how, please look forward to the results.	Jingdong prefecture level city in Shandong
Fortune Shop	Green Intelligent Power Saver	Power saving device, the material is very good practical and durable, the size is suitable, packaging is very good, the price is cheap, logistics is very fast!	Jingdong prefecture level city in Shandong
Fortune Shop	Green Intelligent Power Saver	Very nice, will comment in detail after using it for a while.	Jingdong prefecture level city in Shandong
Fortune Shop	Green Intelligent Power Saver	Good stuff to trust and support	Jingdong prefecture level city in Shandong
Fortune Shop	Green Intelligent Power Saver	I'll comment after I use it, doubt if it really saves power no	Jingdong prefecture level city in Shandong
Fortune Shop	Green Intelligent Power Saver	It's good quality and works well!	Jingdong prefecture level city in Shandong
Fortune Shop	Green Intelligent Power Saver	hahaha just right hahahahahaha hiccup hahahaha	Jingdong prefecture level city in Shandong
Fortune Shop	Green Intelligent Power Saver	The real thing matches the picture and looks great!	Jingdong prefecture level city in Shandong
Fortune Shop	Green Intelligent Power Saver	Plugged it in and we'll see when we pay the bills at the end of the year!	Jingdong prefecture level city in Shandong
Fortune Shop	Green Intelligent Power Saver	False advertising. It doesn't work.	Jingdong prefecture level city in Shandong
Fortune Shop	Green Intelligent Power Saver	Goods received, good effect	Jingdong prefecture level city in Shandong
Fortune Shop	Green Intelligent Power Saver	It looks okay, we'll see if it saves electricity when the time comes	Jingdong prefecture level city in Shandong
Fortune Shop	Green Intelligent Power Saver	Logistics is very fast quality is very good, the price is very affordable, accessories are also complete, installed, satisfied with good use	Jingdong prefecture level city in Shandong
Fortune Shop	Green Intelligent Power Saver	I've received the power saver. I'm looking forward to the savings.	Jingdong prefecture level city in Shandong
Fortune Shop	Green Intelligent Power Saver	Received, have not yet used, after use and then evaluate	Jingdong prefecture level city in Shandong
Fortune Shop	Green Intelligent Power Saver	I received the item, but I don't know if it saves power or not.	Jingdong prefecture level city in Shandong
Fortune Shop	Green Intelligent Power Saver	On Trial	Jingdong prefecture level city in Shandong
Fortune Shop	Green Intelligent Power Saver	Use it and see how it goes.	Jingdong prefecture level city in Shandong
Fortune Shop	Green Intelligent Power Saver	Quality is very good, no odor, fine workmanship, grain color are very like, satisfied!	Jingdong prefecture level city in Shandong
Fortune Shop	Green Intelligent Power Saver	I'll give you a good review. I don't know if it'll work.	Jingdong prefecture level city in Shandong
Fortune Shop	Green Intelligent Power Saver	The logistics is very fast and the package is intact. Good use of affordable. The effect is very good, is genuine	Jingdong prefecture level city in Shandong
Fortune Shop	Green Intelligent Power Saver	Nothing happens when you plug it in.	Jingdong prefecture level city in Shandong
Fortune Shop	Green Intelligent Power Saver	I don't know if it's as good as it sounds.	Jingdong prefecture level city in Shandong
Fortune Shop	Green Intelligent Power Saver	The quality of the stuff is very good, exactly the same as the seller's description, very satisfied!	Jingdong prefecture level city in Shandong
Fortune Shop	Green Intelligent Power Saver	The saver works great, it really works, it's affordable and worth buying.	Jingdong prefecture level city in Shandong
Fortune Shop	Green Intelligent Power Saver	Just used it for a couple days. I don't know yet.	Jingdong prefecture level city in Shandong
Fortune Shop	Green Intelligent Power Saver	The quality is quite good, cheaper than the physical store, good, next time will come again!	Jingdong prefecture level city in Shandong
Fortune Shop	Green Intelligent Power Saver	Good quality, cheap and affordable worth recommending to buy!	Jingdong prefecture level city in Shandong
Fortune Shop	Green Intelligent Power Saver	Very good quality, well made, works well! Satisfied	Jingdong prefecture level city in Shandong
Fortune Shop	Green Intelligent Power Saver	The logistics is very fast quality is very good very pleasant a shopping	Jingdong prefecture level city in Shandong
Fortune Shop	Green Intelligent Power Saver	Use it and then come back to evaluate!	Jingdong prefecture level city in Shandong
Fortune Shop	Green Intelligent Power Saver	# It's no use lying at all #	Jingdong prefecture level city in Shandong
Fortune Shop	Green Intelligent Power Saver	I can't get a voltage drop.	Jingdong prefecture level city in Shandong
Fortune Shop	Green Intelligent Power Saver	Evaluate after use	Jingdong prefecture level city in Shandong
Fortune Shop	Green Intelligent Power Saver	Still on trial.	Jingdong prefecture level city in Shandong
Fortune Shop	Green Intelligent Power Saver	ineffective	Jingdong prefecture level city in Shandong
Fortune Shop	Green Intelligent Power Saver	Satisfaction Shopping	Jingdong prefecture level city in Shandong
Fortune Shop	Green Intelligent Power Saver	Lies.	Jingdong prefecture level city in Shandong
Fortune Shop	Green Intelligent Power Saver	It's a real power saver.	Jingdong prefecture level city in Shandong
Fortune Shop	Green Intelligent Power Saver	in use	Jingdong prefecture level city in Shandong
Fortune Shop	Green Intelligent Power Saver	I don't know yet.	Jingdong prefecture level city in Shandong
Fortune Shop	Green Intelligent Power Saver	not care about anything	Jingdong prefecture level city in Shandong
Fortune Shop	Green Intelligent Power Saver	It doesn't seem to have much effect	Jingdong prefecture level city in Shandong
Fortune Shop	Green Intelligent Power Saver	Let the merchant see for himself, does the plug positive and negative terminals look like this?	Jingdong prefecture level city in Shandong
Fortune Shop	Green Intelligent Power Saver	It doesn't work well, the electricity bill is less before using it, but more after using it, contacting customer service, the service is still not good, and returning it is slow	Jingdong prefecture level city in Shandong
Fortune Shop	Green Intelligent Power Saver	With the advertisement product does not match, above the effective negative, power saving rate of two rows of words it, the product looks very thin, with 3 days to see the meter number is still with the previous no difference, which is obviously fraudulent behavior!	Jingdong prefecture level city in Shandong
Fortune Shop	Green Intelligent Power Saver	Doesn't save any power at all. Not much use.	Jingdong prefecture level city in Shandong
Fortune Shop	Green Intelligent Power Saver	The plugs are all loose, they're all the same length and they don't work at all	Jingdong prefecture level city in Shandong
Fortune Shop	Green Intelligent Power Saver	It's bad 👎 it's not effective at all, you'd be crazy to sell this garbage on the internet.	Jingdong prefecture level city in Shandong

Fortune Shop	Green Intelligent Power Saver	It doesn't save any power at all, so I hope the old timers don't fall for this scam again	Jingdong prefecture level city in Shandong
Fortune Shop	Green Intelligent Power Saver	I've been using it for over a month now and haven't noticed any power savings.	Jingdong prefecture level city in Shandong
Fortune Shop	Green Intelligent Power Saver	It doesn't work at all. The electricity bill is higher when you use it.	Jingdong prefecture level city in Shandong
Fortune Shop	Green Intelligent Power Saver	It doesn't save any power, it's still as bad as ever.	Jingdong prefecture level city in Shandong
Fortune Shop	Green Intelligent Power Saver	Last night before leaving work to look at the table, and then plugged into the node device, this morning and then look at the table, than the original save a few degrees of electricity, very good to have a power-saving effect, easy to use, satisfied with the shopping praise.	Jingdong prefecture level city in Shandong
Fortune Shop	Green Intelligent Power Saver	The product is real and effective, the electricity bill is reduced, really good, thanks to the merchants have so many good products, the product is real and effective, the electricity bill is reduced, want to save electricity friends quickly buy it, early use early to save money.	Jingdong prefecture level city in Shandong
Fortune Shop	Green Intelligent Power Saver	Saver saves time and money, baby appearance is beautiful, the quality of goods is good, and the same as the business description, good service attitude, packaging is complete, logistics is very fast, praise to	Jingdong prefecture level city in Shandong
Fortune Shop	Green Intelligent Power Saver	The effect is good, summer, home electricity is relatively large, buy and use a few days, feel the effect is very good, originally ten dollars a day electricity bill, now eight dollars a day, quite good	Jingdong prefecture level city in Shandong
Fortune Shop	Green Intelligent Power Saver	Indeed can save electricity and money, the first time I know this or from a friend's home to see, but also do not believe it, and now they use it to know that there is this good thing.	Jingdong prefecture level city in Shandong
Fortune Shop	Green Intelligent Power Saver	I have received the Smart Power Saver. Perfectly packaged. Fast delivery. Boss customer service attitude is also very good. It's worth buying. I plugged it in and tried it and it really saves more power than the original. I'll buy it again next time.	Jingdong prefecture level city in Shandong
Fortune Shop	Green Intelligent Power Saver	It's nice to save a dozen bucks a night on two air conditioners that used to take 30 or 40 degrees a night, but now it's only 10 or so degrees a night	Jingdong prefecture level city in Shandong
Fortune Shop	Green Intelligent Power Saver	Product no manufacturer's name no product qualification certificate, try two days to see the effect of	Jingdong prefecture level city in Shandong
Fortune Shop	Green Intelligent Power Saver	I've been using it for over a month and have saved over $80, so I've repurchased a few more back to save together	Jingdong prefecture level city in Shandong
Fortune Shop	Green Intelligent Power Saver	I haven't used it yet and will comment when I do, but it's well made.	Jingdong prefecture level city in Shandong
Fortune Shop	Green Intelligent Power Saver	It's quite energy efficient. It's really a boon for the home.	Jingdong prefecture level city in Shandong
Fortune Shop	Green Intelligent Power Saver	Energy-saving socket received a month, after using it for a month proved that it is indeed energy-saving, electricity bill saving, will buy it again, thank you!	Jingdong prefecture level city in Shandong
Fortune Shop	Green Intelligent Power Saver	There was a similar product many years ago. Now technology has evolved, buy one to try, according to the instructions in the box, it takes one or two dozen days to show results. Check your electric bill and see what happens.	Jingdong prefecture level city in Shandong
Fortune Shop	Green Intelligent Power Saver	Jingdong logistics is really fast, the same day the order arrived on the same day, more convenient than the supermarket, and a wide range of choices, the	Jingdong prefecture level city in Shandong
Fortune Shop	Green Intelligent Power Saver	I've been using this for a month or so and I can't see any results, it really is a scam as they say.	Jingdong prefecture level city in Shandong
Fortune Shop	Green Intelligent Power Saver	Very good use, a month can save a few degrees of power loss of electricity, introduced to the relatives to buy back to try.	Jingdong prefecture level city in Shandong
Fortune Shop	Green Intelligent Power Saver	It's really powerful! With this air conditioner can be turned on at any time without the pressure of paying the electricity bill! It's good for the people!	Jingdong prefecture level city in Shandong
Fortune Shop	Green Intelligent Power Saver	This business is a big ** products are not qualified, with a month of electricity more than one hundred percent of thirty, the purchase agreed to return the goods but do not receive a refund after receiving the goods, looking for the official side does not help, the Jingdong is a big **, I have never shopped in	Jingdong prefecture level city in Shandong
Fortune Shop	Green Intelligent Power Saver	It's good. I tried it. It saves power.	Jingdong prefecture level city in Shandong
Fortune Shop	Green Intelligent Power Saver	Nothing**, it seems like there's more	Jingdong prefecture level city in Shandong
Fortune Shop	Green Intelligent Power Saver	Power saving device received, well packaged, good quality, fast logistics	Jingdong prefecture level city in Shandong
Fortune Shop	Green Intelligent Power Saver	The service is okay, is that when I bought five, the desirable pieces are three, and then wait a few days and send me two replacement, I do not know if it is a mistake of the shipper, if I was not in the courier there to see, go home to say no, the	Jingdong prefecture level city in Shandong
Fortune Shop	Green Intelligent Power Saver	This thing will only cost me more if I use it, but before I used it, it was less than a ~ electricity a month, and after I used it, it was more than two hundred kWh a month.	Jingdong prefecture level city in Shandong
Fortune Shop	Green Intelligent Power Saver	I just used it. I don't know how it is.	Jingdong prefecture level city in Shandong
Fortune Shop	Green Intelligent Power Saver	I've received the product and I'm using it, so I'll see how it goes in a month!	Jingdong prefecture level city in Shandong
Fortune Shop	Green Intelligent Power Saver	The goods have just received the effect do not know how, please look forward to the results.	Jingdong prefecture level city in Shandong
Fortune Shop	Green Intelligent Power Saver	Power saving device, the material is very good practical and durable, the size is suitable, packaging is very good, the price is cheap, logistics is very fast!	Jingdong prefecture level city in Shandong
Fortune Shop	Green Intelligent Power Saver	Very nice, will comment in detail after using it for a while.	Jingdong prefecture level city in Shandong
Fortune Shop	Green Intelligent Power Saver	Good stuff to trust and support	Jingdong prefecture level city in Shandong
Fortune Shop	Green Intelligent Power Saver	I'll comment after I use it, doubt if it really saves power no	Jingdong prefecture level city in Shandong
Fortune Shop	Green Intelligent Power Saver	It's good quality and works well!	Jingdong prefecture level city in Shandong
Fortune Shop	Green Intelligent Power Saver	hahaha just right hahahahahaha hiccup hahahaha	Jingdong prefecture level city in Shandong
Fortune Shop	Green Intelligent Power Saver	The real thing matches the picture and looks great!	Jingdong prefecture level city in Shandong
Fortune Shop	Green Intelligent Power Saver	Plugged it in and we'll see when we pay the bills at the end of the year!	Jingdong prefecture level city in Shandong
Fortune Shop	Green Intelligent Power Saver	False advertising. It doesn't work.	Jingdong prefecture level city in Shandong
Fortune Shop	Green Intelligent Power Saver	Goods received, good effect	Jingdong prefecture level city in Shandong
Fortune Shop	Green Intelligent Power Saver	It looks okay, we'll see if it saves electricity when the time comes	Jingdong prefecture level city in Shandong
Fortune Shop	Green Intelligent Power Saver	Logistics is very fast quality is very good, the price is very affordable, accessories are also complete, installed, satisfied with good use	Jingdong prefecture level city in Shandong
Fortune Shop	Green Intelligent Power Saver	I've received the power saver. I'm looking forward to the savings.	Jingdong prefecture level city in Shandong
Fortune Shop	Green Intelligent Power Saver	Received, have not yet used, after use and then evaluate	Jingdong prefecture level city in Shandong
Fortune Shop	Green Intelligent Power Saver	I received the item, but I don't know if it saves power or not.	Jingdong prefecture level city in Shandong
Fortune Shop	Green Intelligent Power Saver	On Trial	Jingdong prefecture level city in Shandong
Fortune Shop	Green Intelligent Power Saver	Use it and see how it goes.	Jingdong prefecture level city in Shandong
Fortune Shop	Green Intelligent Power Saver	Quality is very good, no odor, fine workmanship, grain color are very like, satisfied!	Jingdong prefecture level city in Shandong

Fortune Shop	Green Intelligent Power Saver	I'll give you a good review. I don't know if it'll work.	Jingdong prefecture level city in Shandong
Fortune Shop	Green Intelligent Power Saver	The logistics is very fast and the package is intact. Good use of affordable. The effect is very good, is genuine	Jingdong prefecture level city in Shandong
Fortune Shop	Green Intelligent Power Saver	Nothing happens when you plug it in.	Jingdong prefecture level city in Shandong
Fortune Shop	Green Intelligent Power Saver	I don't know if it's as good as it sounds.	Jingdong prefecture level city in Shandong
Fortune Shop	Green Intelligent Power Saver	The quality of the stuff is very good, exactly the same as the seller's description, very satisfied!	Jingdong prefecture level city in Shandong
Fortune Shop	Green Intelligent Power Saver	The saver works great, it really works, it's affordable and worth buying.	Jingdong prefecture level city in Shandong
Fortune Shop	Green Intelligent Power Saver	Just used it for a couple days. I don't know yet.	Jingdong prefecture level city in Shandong
Fortune Shop	Green Intelligent Power Saver	The quality is quite good, cheaper than the physical store, good, next time will come again!	Jingdong prefecture level city in Shandong
Fortune Shop	Green Intelligent Power Saver	Good quality, cheap and affordable worth recommending to buy!	Jingdong prefecture level city in Shandong
Fortune Shop	Green Intelligent Power Saver	Very good quality, well made, works well! Satisfied	Jingdong prefecture level city in Shandong
Fortune Shop	Green Intelligent Power Saver	The logistics is very fast quality is very good very pleasant a shopping	Jingdong prefecture level city in Shandong
Fortune Shop	Green Intelligent Power Saver	Use it and then come back to evaluate!	Jingdong prefecture level city in Shandong
Fortune Shop	Green Intelligent Power Saver	# It's no use lying at all #	Jingdong prefecture level city in Shandong
Fortune Shop	Green Intelligent Power Saver	I can't get a voltage drop.	Jingdong prefecture level city in Shandong
Fortune Shop	Green Intelligent Power Saver	Evaluate after use	Jingdong prefecture level city in Shandong
Fortune Shop	Green Intelligent Power Saver	Still on trial.	Jingdong prefecture level city in Shandong
Fortune Shop	Green Intelligent Power Saver	ineffective	Jingdong prefecture level city in Shandong
Fortune Shop	Green Intelligent Power Saver	Satisfaction Shopping	Jingdong prefecture level city in Shandong
Fortune Shop	Green Intelligent Power Saver	Lies.	Jingdong prefecture level city in Shandong
Fortune Shop	Green Intelligent Power Saver	It's a real power saver.	Jingdong prefecture level city in Shandong
Fortune Shop	Green Intelligent Power Saver	in use	Jingdong prefecture level city in Shandong
Fortune Shop	Green Intelligent Power Saver	I don't know yet.	Jingdong prefecture level city in Shandong
Fortune Shop	Green Intelligent Power Saver	not care about anything	Jingdong prefecture level city in Shandong
Fortune Shop	Green Intelligent Power Saver	It doesn't seem to have much effect	Jingdong prefecture level city in Shandong
Fortune Shop	Green Intelligent Power Saver	Let the merchant see for himself, does the plug positive and negative terminals look like this?	Jingdong prefecture level city in Shandong
Fortune Shop	Green Intelligent Power Saver	It doesn't work well, the electricity bill is less before using it, but more after using it, contacting customer service, the service is still not good, and returning it is slow	Jingdong prefecture level city in Shandong
Fortune Shop	Green Intelligent Power Saver	With the advertisement product does not match, above the effective negative, power saving rate of two rows of words it, the product looks very thin, with 3 days to see the meter number is still with the previous no difference, which is obviously fraudulent behavior!	Jingdong prefecture level city in Shandong
Fortune Shop	Green Intelligent Power Saver	Doesn't save any power at all. Not much use.	Jingdong prefecture level city in Shandong
Fortune Shop	Green Intelligent Power Saver	The plugs are all loose, they're all the same length and they don't work at all	Jingdong prefecture level city in Shandong
Fortune Shop	Green Intelligent Power Saver	It's bad 👎 it's not effective at all, you'd be crazy to sell this garbage on the internet.	Jingdong prefecture level city in Shandong
Fortune Shop	Green Intelligent Power Saver	It doesn't save any power at all, so I hope the old timers don't fall for this scam again	Jingdong prefecture level city in Shandong
Fortune Shop	Green Intelligent Power Saver	I've been using it for over a month now and haven't noticed any power savings.	Jingdong prefecture level city in Shandong
Fortune Shop	Green Intelligent Power Saver	It doesn't work at all. The electricity bill is higher when you use it.	Jingdong prefecture level city in Shandong
Fortune Shop	Green Intelligent Power Saver	It doesn't save any power, it's still as bad as ever.	Jingdong prefecture level city in Shandong
Fortune Shop	Green Intelligent Power Saver	Last night before leaving work to look at the table, and then plugged into the node device, this morning and then look at the table, than the original save a few degrees of electricity, very good to have a power-saving effect, easy to use, satisfied with the shopping praise.	Jingdong prefecture level city in Shandong
Fortune Shop	Green Intelligent Power Saver	The product is real and effective, the electricity bill is reduced, really good, thanks to the merchants have so many good products, the product is real and effective, the electricity bill is reduced, want to save electricity friends quickly buy it, early use early to save money.	Jingdong prefecture level city in Shandong
Fortune Shop	Green Intelligent Power Saver	Saver saves time and money, baby appearance is beautiful, the quality of goods is good, and the same as the business description, good service attitude, packaging is complete, logistics is very fast, praise to	Jingdong prefecture level city in Shandong
Fortune Shop	Green Intelligent Power Saver	The effect is good, summer, home electricity is relatively large, buy and use a few days, feel the effect is very good, originally ten dollars a day electricity bill, now eight dollars a day, quite good	Jingdong prefecture level city in Shandong
Fortune Shop	Green Intelligent Power Saver	Indeed can save electricity and money, the first time I know this or from a friend's home to see, but also do not believe it, and now they use it to know that there is this good thing.	Jingdong prefecture level city in Shandong
Fortune Shop	Green Intelligent Power Saver	I have received the Smart Power Saver. Perfectly packaged. Fast delivery. Boss customer service attitude is also very good. It's worth buying. I plugged it in and tried it and it really saves more power than the original. I'll buy it again next time.	Jingdong prefecture level city in Shandong
Fortune Shop	Green Intelligent Power Saver	It's nice to save a dozen bucks a night on two air conditioners that used to take 30 or 40 degrees a night, but now it's only 10 or so degrees a night	Jingdong prefecture level city in Shandong
Fortune Shop	Green Intelligent Power Saver	Product no manufacturer's name no product qualification certificate, try two days to see the effect of	Jingdong prefecture level city in Shandong
Fortune Shop	Green Intelligent Power Saver	I've been using it for over a month and have saved over $80, so I've repurchased a few more back to save together	Jingdong prefecture level city in Shandong
Fortune Shop	Green Intelligent Power Saver	I haven't used it yet and will comment when I do, but it's well made.	Jingdong prefecture level city in Shandong
Fortune Shop	Green Intelligent Power Saver	It's quite energy efficient. It's really a boon for the home.	Jingdong prefecture level city in Shandong
Fortune Shop	Green Intelligent Power Saver	Energy-saving socket received a month, after using it for a month proved that it is indeed energy-saving, electricity bill saving, will buy it again, thank you!	Jingdong prefecture level city in Shandong
Fortune Shop	Green Intelligent Power Saver	There was a similar product many years ago. Now technology has evolved, buy one to try, according to the instructions in the box, it takes one or two dozen days to show results. Check your electric bill and see what happens.	Jingdong prefecture level city in Shandong
Fortune Shop	Green Intelligent Power Saver	Jingdong logistics is really fast, the same day the order arrived on the same day, more convenient than the supermarket, and a wide range of choices, the	Jingdong prefecture level city in Shandong
Fortune Shop	Green Intelligent Power Saver	I've been using this for a month or so and I can't see any results, it really is a scam as they say.	Jingdong prefecture level city in Shandong

Fortune Shop	Green Intelligent Power Saver	Very good use, a month can save a few degrees of power loss of electricity, introduced to the relatives to buy back to try.	Jingdong prefecture level city in Shandong
Fortune Shop	Green Intelligent Power Saver	It's really powerful! With this air conditioner can be turned on at any time without the pressure of paying the electricity bill! It's good for the people!	Jingdong prefecture level city in Shandong
Fortune Shop	Green Intelligent Power Saver	This business is a big ** products are not qualified, with a month of electricity more than one hundred percent of thirty, the purchase agreed to return the goods but do not receive a refund after receiving the goods, looking for the official side does not help, the Jingdong is a big **, I have never shopped in	Jingdong prefecture level city in Shandong
Fortune Shop	Green Intelligent Power Saver	It's good. I tried it. It saves power.	Jingdong prefecture level city in Shandong
Fortune Shop	Green Intelligent Power Saver	Nothing**, it seems like there's more	Jingdong prefecture level city in Shandong
Fortune Shop	Green Intelligent Power Saver	Power saving device received, well packaged, good quality, fast logistics	Jingdong prefecture level city in Shandong
Fortune Shop	Green Intelligent Power Saver	The service is okay, is that when I bought five, the desirable pieces are three, and then wait a few days and send me two replacement, I do not know if it is a mistake of the shipper, if I was not in the courier there to see, go home to say no, the	Jingdong prefecture level city in Shandong
Fortune Shop	Green Intelligent Power Saver	This thing will only cost me more if I use it, but before I used it, it was less than a ~ electricity a month, and after I used it, it was more than two hundred kWh a month.	Jingdong prefecture level city in Shandong
Fortune Shop	Green Intelligent Power Saver	I just used it. I don't know how it is.	Jingdong prefecture level city in Shandong
Fortune Shop	Green Intelligent Power Saver	I've received the product and I'm using it, so I'll see how it goes in a month!	Jingdong prefecture level city in Shandong
Fortune Shop	Green Intelligent Power Saver	The goods have just received the effect do not know how, please look forward to the results.	Jingdong prefecture level city in Shandong
Fortune Shop	Green Intelligent Power Saver	Power saving device, the material is very good practical and durable, the size is suitable, packaging is very good, the price is cheap, logistics is very fast!	Jingdong prefecture level city in Shandong
Fortune Shop	Green Intelligent Power Saver	Very nice, will comment in detail after using it for a while.	Jingdong prefecture level city in Shandong
Fortune Shop	Green Intelligent Power Saver	Good stuff to trust and support	Jingdong prefecture level city in Shandong
Fortune Shop	Green Intelligent Power Saver	I'll comment after I use it, doubt if it really saves power no	Jingdong prefecture level city in Shandong
Fortune Shop	Green Intelligent Power Saver	It's good quality and works well!	Jingdong prefecture level city in Shandong
Fortune Shop	Green Intelligent Power Saver	hahaha just right hahahahahaha hiccup hahahaha	Jingdong prefecture level city in Shandong
Fortune Shop	Green Intelligent Power Saver	The real thing matches the picture and looks great!	Jingdong prefecture level city in Shandong
Fortune Shop	Green Intelligent Power Saver	Plugged it in and we'll see when we pay the bills at the end of the year!	Jingdong prefecture level city in Shandong
Fortune Shop	Green Intelligent Power Saver	False advertising. It doesn't work.	Jingdong prefecture level city in Shandong
Fortune Shop	Green Intelligent Power Saver	Goods received, good effect	Jingdong prefecture level city in Shandong
Fortune Shop	Green Intelligent Power Saver	It looks okay, we'll see if it saves electricity when the time comes	Jingdong prefecture level city in Shandong
Fortune Shop	Green Intelligent Power Saver	Logistics is very fast quality is very good, the price is very affordable, accessories are also complete, installed, satisfied with good use	Jingdong prefecture level city in Shandong
Fortune Shop	Green Intelligent Power Saver	I've received the power saver. I'm looking forward to the savings.	Jingdong prefecture level city in Shandong
Fortune Shop	Green Intelligent Power Saver	Received, have not yet used, after use and then evaluate	Jingdong prefecture level city in Shandong
Fortune Shop	Green Intelligent Power Saver	I received the item, but I don't know if it saves power or not.	Jingdong prefecture level city in Shandong
Fortune Shop	Green Intelligent Power Saver	On Trial	Jingdong prefecture level city in Shandong
Fortune Shop	Green Intelligent Power Saver	Use it and see how it goes.	Jingdong prefecture level city in Shandong
Fortune Shop	Green Intelligent Power Saver	Quality is very good, no odor, fine workmanship, grain color are very like, satisfied!	Jingdong prefecture level city in Shandong
Fortune Shop	Green Intelligent Power Saver	I'll give you a good review. I don't know if it'll work.	Jingdong prefecture level city in Shandong
Fortune Shop	Green Intelligent Power Saver	The logistics is very fast and the package is intact. Good use of affordable. The effect is very good, is genuine	Jingdong prefecture level city in Shandong
Fortune Shop	Green Intelligent Power Saver	Nothing happens when you plug it in.	Jingdong prefecture level city in Shandong
Fortune Shop	Green Intelligent Power Saver	I don't know if it's as good as it sounds.	Jingdong prefecture level city in Shandong
Fortune Shop	Green Intelligent Power Saver	The quality of the stuff is very good, exactly the same as the seller's description, very satisfied!	Jingdong prefecture level city in Shandong
Fortune Shop	Green Intelligent Power Saver	The saver works great, it really works, it's affordable and worth buying.	Jingdong prefecture level city in Shandong
Fortune Shop	Green Intelligent Power Saver	Just used it for a couple days. I don't know yet.	Jingdong prefecture level city in Shandong
Fortune Shop	Green Intelligent Power Saver	The quality is quite good, cheaper than the physical store, good, next time will come again!	Jingdong prefecture level city in Shandong
Fortune Shop	Green Intelligent Power Saver	Good quality, cheap and affordable worth recommending to buy!	Jingdong prefecture level city in Shandong
Fortune Shop	Green Intelligent Power Saver	Very good quality, well made, works well! Satisfied	Jingdong prefecture level city in Shandong
Fortune Shop	Green Intelligent Power Saver	The logistics is very fast quality is very good very pleasant a shopping	Jingdong prefecture level city in Shandong
Fortune Shop	Green Intelligent Power Saver	Use it and then come back to evaluate!	Jingdong prefecture level city in Shandong
Fortune Shop	Green Intelligent Power Saver	# It's no use lying at all #	Jingdong prefecture level city in Shandong
Fortune Shop	Green Intelligent Power Saver	I can't get a voltage drop.	Jingdong prefecture level city in Shandong
Fortune Shop	Green Intelligent Power Saver	Evaluate after use	Jingdong prefecture level city in Shandong
Fortune Shop	Green Intelligent Power Saver	Still on trial.	Jingdong prefecture level city in Shandong
Fortune Shop	Green Intelligent Power Saver	ineffective	Jingdong prefecture level city in Shandong
Fortune Shop	Green Intelligent Power Saver	Satisfaction Shopping	Jingdong prefecture level city in Shandong
Fortune Shop	Green Intelligent Power Saver	Lies.	Jingdong prefecture level city in Shandong
Fortune Shop	Green Intelligent Power Saver	It's a real power saver.	Jingdong prefecture level city in Shandong
Fortune Shop	Green Intelligent Power Saver	in use	Jingdong prefecture level city in Shandong

Fortune Shop	Green Intelligent Power Saver	I don't know yet.	Jingdong prefecture level city in Shandong
Fortune Shop	Green Intelligent Power Saver	not care about anything	Jingdong prefecture level city in Shandong
Fortune Shop	Green Intelligent Power Saver	It doesn't seem to have much effect	Jingdong prefecture level city in Shandong
Fortune Shop	Green Intelligent Power Saver	Let the merchant see for himself, does the plug positive and negative terminals look like this?	Jingdong prefecture level city in Shandong
Fortune Shop	Green Intelligent Power Saver	It doesn't work well, the electricity bill is less before using it, but more after using it, contacting customer service, the service is still not good, and returning it is slow	Jingdong prefecture level city in Shandong
Fortune Shop	Green Intelligent Power Saver	With the advertisement product does not match, above the effective negative, power saving rate of two rows of words it, the product looks very thin, with 3 days to see the meter number is still with the previous no difference, which is obviously fraudulent behavior!	Jingdong prefecture level city in Shandong
Fortune Shop	Green Intelligent Power Saver	Doesn't save any power at all. Not much use.	Jingdong prefecture level city in Shandong
Fortune Shop	Green Intelligent Power Saver	The plugs are all loose, they're all the same length and they don't work at all	Jingdong prefecture level city in Shandong
Fortune Shop	Green Intelligent Power Saver	It's bad 👎 it's not effective at all, you'd be crazy to sell this garbage on the internet.	Jingdong prefecture level city in Shandong
Fortune Shop	Green Intelligent Power Saver	It doesn't save any power at all, so I hope the old timers don't fall for this scam again	Jingdong prefecture level city in Shandong
Fortune Shop	Green Intelligent Power Saver	I've been using it for over a month now and haven't noticed any power savings.	Jingdong prefecture level city in Shandong
Fortune Shop	Green Intelligent Power Saver	It doesn't work at all. The electricity bill is higher when you use it.	Jingdong prefecture level city in Shandong
Fortune Shop	Green Intelligent Power Saver	It doesn't save any power, it's still as bad as ever.	Jingdong prefecture level city in Shandong
Fortune Shop	Green Intelligent Power Saver	Last night before leaving work to look at the table, and then plugged into the node device, this morning and then look at the table, than the original save a few degrees of electricity, very good to have a power-saving effect, easy to use, satisfied with the shopping praise.	Jingdong prefecture level city in Shandong
Fortune Shop	Green Intelligent Power Saver	The product is real and effective, the electricity bill is reduced, really good, thanks to the merchants have so many good products, the product is real and effective, the electricity bill is reduced, want to save electricity friends quickly buy it, early use early to save money.	Jingdong prefecture level city in Shandong
Fortune Shop	Green Intelligent Power Saver	Saver saves time and money, baby appearance is beautiful, the quality of goods is good, and the same as the business description, good service attitude, packaging is complete, logistics is very fast, praise to	Jingdong prefecture level city in Shandong
Fortune Shop	Green Intelligent Power Saver	The effect is good, summer, home electricity is relatively large, buy and use a few days, feel the effect is very good, originally ten dollars a day electricity bill, now eight dollars a day, quite good	Jingdong prefecture level city in Shandong
Fortune Shop	Green Intelligent Power Saver	Indeed can save electricity and money, the first time I know this or from a friend's home to see, but also do not believe it, and now they use it to know that there is this good thing.	Jingdong prefecture level city in Shandong
Fortune Shop	Green Intelligent Power Saver	I have received the Smart Power Saver. Perfectly packaged. Fast delivery. Boss customer service attitude is also very good. It's worth buying. I plugged it in and tried it and it really saves more power than the original. I'll buy it again next time.	Jingdong prefecture level city in Shandong
Fortune Shop	Green Intelligent Power Saver	It's nice to save a dozen bucks a night on two air conditioners that used to take 30 or 40 degrees a night, but now it's only 10 or so degrees a night	Jingdong prefecture level city in Shandong
Fortune Shop	Green Intelligent Power Saver	Product no manufacturer's name no product qualification certificate, try two days to see the effect of	Jingdong prefecture level city in Shandong
Fortune Shop	Green Intelligent Power Saver	I've been using it for over a month and have saved over $80, so I've repurchased a few more back to save together	Jingdong prefecture level city in Shandong
Fortune Shop	Green Intelligent Power Saver	I haven't used it yet and will comment when I do, but it's well made.	Jingdong prefecture level city in Shandong
Fortune Shop	Green Intelligent Power Saver	It's quite energy efficient. It's really a boon for the home.	Jingdong prefecture level city in Shandong
Fortune Shop	Green Intelligent Power Saver	Energy-saving socket received a month, after using it for a month proved that it is indeed energy-saving, electricity bill saving, will buy it again, thank you!	Jingdong prefecture level city in Shandong
Fortune Shop	Green Intelligent Power Saver	There was a similar product many years ago. Now technology has evolved, buy one to try, according to the instructions in the box, it takes one or two dozen days to show results. Check your electric bill and see what happens.	Jingdong prefecture level city in Shandong
Fortune Shop	Green Intelligent Power Saver	Jingdong logistics is really fast, the same day the order arrived on the same day, more convenient than the supermarket, and a wide range of choices, the	Jingdong prefecture level city in Shandong
Fortune Shop	Green Intelligent Power Saver	I've been using this for a month or so and I can't see any results, it really is a scam as they say.	Jingdong prefecture level city in Shandong
Fortune Shop	Green Intelligent Power Saver	Very good use, a month can save a few degrees of power loss of electricity, introduced to the relatives to buy back to try.	Jingdong prefecture level city in Shandong
Fortune Shop	Green Intelligent Power Saver	It's really powerful! With this air conditioner can be turned on at any time without the pressure of paying the electricity bill! It's good for the people!	Jingdong prefecture level city in Shandong
Fortune Shop	Green Intelligent Power Saver	This business is a big ** products are not qualified, with a month of electricity more than one hundred percent of thirty, the purchase agreed to return the goods but do not receive a refund after receiving the goods, looking for the official side does not help, the Jingdong is a big **, I have never shopped in	Jingdong prefecture level city in Shandong
Fortune Shop	Green Intelligent Power Saver	It's good. I tried it. It saves power.	Jingdong prefecture level city in Shandong
Fortune Shop	Green Intelligent Power Saver	Nothing**, it seems like there's more	Jingdong prefecture level city in Shandong
Fortune Shop	Green Intelligent Power Saver	Power saving device received, well packaged, good quality, fast logistics	Jingdong prefecture level city in Shandong
Fortune Shop	Green Intelligent Power Saver	The service is okay, is that when I bought five, the desirable pieces are three, and then wait a few days and send me two replacement, I do not know if it is a mistake of the shipper, if I was not in the courier there to see, go home to say no, the	Jingdong prefecture level city in Shandong
Fortune Shop	Green Intelligent Power Saver	This thing will only cost me more if I use it, but before I used it, it was less than a ~ electricity a month, and after I used it, it was more than two hundred kWh a month.	Jingdong prefecture level city in Shandong
Fortune Shop	Green Intelligent Power Saver	I just used it. I don't know how it is.	Jingdong prefecture level city in Shandong
Fortune Shop	Green Intelligent Power Saver	I've received the product and I'm using it, so I'll see how it goes in a month!	Jingdong prefecture level city in Shandong
Fortune Shop	Green Intelligent Power Saver	The goods have just received the effect do not know how, please look forward to the results.	Jingdong prefecture level city in Shandong
Fortune Shop	Green Intelligent Power Saver	Power saving device, the material is very good practical and durable, the size is suitable, packaging is very good, the price is cheap, logistics is very fast!	Jingdong prefecture level city in Shandong
Fortune Shop	Green Intelligent Power Saver	Very nice, will comment in detail after using it for a while.	Jingdong prefecture level city in Shandong
Fortune Shop	Green Intelligent Power Saver	Good stuff to trust and support	Jingdong prefecture level city in Shandong
Fortune Shop	Green Intelligent Power Saver	I'll comment after I use it, doubt if it really saves power no	Jingdong prefecture level city in Shandong
Fortune Shop	Green Intelligent Power Saver	It's good quality and works well!	Jingdong prefecture level city in Shandong
Fortune Shop	Green Intelligent Power Saver	hahaha just right hahahahahaha hiccup hahahaha	Jingdong prefecture level city in Shandong
Fortune Shop	Green Intelligent Power Saver	The real thing matches the picture and looks great!	Jingdong prefecture level city in Shandong
Fortune Shop	Green Intelligent Power Saver	Plugged it in and we'll see when we pay the bills at the end of the year!	Jingdong prefecture level city in Shandong
Fortune Shop	Green Intelligent Power Saver	False advertising. It doesn't work.	Jingdong prefecture level city in Shandong

Fortune Shop	Green Intelligent Power Saver	Goods received, good effect	Jingdong prefecture level city in Shandong
Fortune Shop	Green Intelligent Power Saver	It looks okay, we'll see if it saves electricity when the time comes	Jingdong prefecture level city in Shandong
Fortune Shop	Green Intelligent Power Saver	Logistics is very fast quality is very good, the price is very affordable, accessories are also complete, installed, satisfied with good use	Jingdong prefecture level city in Shandong
Fortune Shop	Green Intelligent Power Saver	I've received the power saver. I'm looking forward to the savings.	Jingdong prefecture level city in Shandong
Fortune Shop	Green Intelligent Power Saver	Received, have not yet used, after use and then evaluate	Jingdong prefecture level city in Shandong
Fortune Shop	Green Intelligent Power Saver	I received the item, but I don't know if it saves power or not.	Jingdong prefecture level city in Shandong
Fortune Shop	Green Intelligent Power Saver	On Trial	Jingdong prefecture level city in Shandong
Fortune Shop	Green Intelligent Power Saver	Use it and see how it goes.	Jingdong prefecture level city in Shandong
Fortune Shop	Green Intelligent Power Saver	Quality is very good, no odor, fine workmanship, grain color are very like, satisfied!	Jingdong prefecture level city in Shandong
Fortune Shop	Green Intelligent Power Saver	I'll give you a good review. I don't know if it'll work.	Jingdong prefecture level city in Shandong
Fortune Shop	Green Intelligent Power Saver	The logistics is very fast and the package is intact. Good use of affordable. The effect is very good, is genuine	Jingdong prefecture level city in Shandong
Fortune Shop	Green Intelligent Power Saver	Nothing happens when you plug it in.	Jingdong prefecture level city in Shandong
Fortune Shop	Green Intelligent Power Saver	I don't know if it's as good as it sounds.	Jingdong prefecture level city in Shandong
Fortune Shop	Green Intelligent Power Saver	The quality of the stuff is very good, exactly the same as the seller's description, very satisfied!	Jingdong prefecture level city in Shandong
Fortune Shop	Green Intelligent Power Saver	The saver works great, it really works, it's affordable and worth buying.	Jingdong prefecture level city in Shandong
Fortune Shop	Green Intelligent Power Saver	Just used it for a couple days. I don't know yet.	Jingdong prefecture level city in Shandong
Fortune Shop	Green Intelligent Power Saver	The quality is quite good, cheaper than the physical store, good, next time will come again!	Jingdong prefecture level city in Shandong
Fortune Shop	Green Intelligent Power Saver	Good quality, cheap and affordable worth recommending to buy!	Jingdong prefecture level city in Shandong
Fortune Shop	Green Intelligent Power Saver	Very good quality, well made, works well! Satisfied	Jingdong prefecture level city in Shandong
Fortune Shop	Green Intelligent Power Saver	The logistics is very fast quality is very good very pleasant a shopping	Jingdong prefecture level city in Shandong
Fortune Shop	Green Intelligent Power Saver	Use it and then come back to evaluate!	Jingdong prefecture level city in Shandong
Fortune Shop	Green Intelligent Power Saver	# It's no use lying at all #	Jingdong prefecture level city in Shandong
Fortune Shop	Green Intelligent Power Saver	I can't get a voltage drop.	Jingdong prefecture level city in Shandong
Fortune Shop	Green Intelligent Power Saver	Evaluate after use	Jingdong prefecture level city in Shandong
Fortune Shop	Green Intelligent Power Saver	Still on trial.	Jingdong prefecture level city in Shandong
Fortune Shop	Green Intelligent Power Saver	ineffective	Jingdong prefecture level city in Shandong
Fortune Shop	Green Intelligent Power Saver	Satisfaction Shopping	Jingdong prefecture level city in Shandong
Fortune Shop	Green Intelligent Power Saver	Lies.	Jingdong prefecture level city in Shandong
Fortune Shop	Green Intelligent Power Saver	It's a real power saver.	Jingdong prefecture level city in Shandong
Fortune Shop	Green Intelligent Power Saver	in use	Jingdong prefecture level city in Shandong
Fortune Shop	Green Intelligent Power Saver	I don't know yet.	Jingdong prefecture level city in Shandong
Fortune Shop	Green Intelligent Power Saver	not care about anything	Jingdong prefecture level city in Shandong
Fortune Shop	Green Intelligent Power Saver	It doesn't seem to have much effect	Jingdong prefecture level city in Shandong
Fortune Shop	Green Intelligent Power Saver	Let the merchant see for himself, does the plug positive and negative terminals look like this?	Jingdong prefecture level city in Shandong
Fortune Shop	Green Intelligent Power Saver	It doesn't work well, the electricity bill is less before using it, but more after using it, contacting customer service, the service is still not good, and returning it is slow	Jingdong prefecture level city in Shandong
Fortune Shop	Green Intelligent Power Saver	With the advertisement product does not match, above the effective negative, power saving rate of two rows of words it, the product looks very thin, with 3 days to see the meter number is still with the previous no difference, which is obviously fraudulent behavior!	Jingdong prefecture level city in Shandong
Fortune Shop	Green Intelligent Power Saver	Doesn't save any power at all. Not much use.	Jingdong prefecture level city in Shandong
Fortune Shop	Green Intelligent Power Saver	The plugs are all loose, they're all the same length and they don't work at all	Jingdong prefecture level city in Shandong
Fortune Shop	Green Intelligent Power Saver	It's bad 👎 it's not effective at all, you'd be crazy to sell this garbage on the internet.	Jingdong prefecture level city in Shandong
Fortune Shop	Green Intelligent Power Saver	It doesn't save any power at all, so I hope the old timers don't fall for this scam again	Jingdong prefecture level city in Shandong
Fortune Shop	Green Intelligent Power Saver	I've been using it for over a month now and haven't noticed any power savings.	Jingdong prefecture level city in Shandong
Fortune Shop	Green Intelligent Power Saver	It doesn't work at all. The electricity bill is higher when you use it.	Jingdong prefecture level city in Shandong
Fortune Shop	Green Intelligent Power Saver	It doesn't save any power, it's still as bad as ever.	Jingdong prefecture level city in Shandong
Fortune Shop	Green Intelligent Power Saver	Last night before leaving work to look at the table, and then plugged into the node device, this morning and then look at the table, than the original save a few degrees of electricity, very good to have a power-saving effect, easy to use, satisfied with the shopping praise.	Jingdong prefecture level city in Shandong
Fortune Shop	Green Intelligent Power Saver	The product is real and effective, the electricity bill is reduced, really good, thanks to the merchants have so many good products, the product is real and effective, the electricity bill is reduced, want to save electricity friends quickly buy it, early use early to save money.	Jingdong prefecture level city in Shandong
Fortune Shop	Green Intelligent Power Saver	Saver saves time and money, baby appearance is beautiful, the quality of goods is good, and the same as the business description, good service attitude, packaging is complete, logistics is very fast, praise to	Jingdong prefecture level city in Shandong
Fortune Shop	Green Intelligent Power Saver	The effect is good, summer, home electricity is relatively large, buy and use a few days, feel the effect is very good, originally ten dollars a day electricity bill, now eight dollars a day, quite good	Jingdong prefecture level city in Shandong
Fortune Shop	Green Intelligent Power Saver	Indeed can save electricity and money, the first time I know this or from a friend's home to see, but also do not believe it, and now they use it to know that there is this good thing.	Jingdong prefecture level city in Shandong
Fortune Shop	Green Intelligent Power Saver	I have received the Smart Power Saver. Perfectly packaged. Fast delivery. Boss customer service attitude is also very good. It's worth buying. I plugged it in and tried it and it really saves more power than the original. I'll buy it again next time.	Jingdong prefecture level city in Shandong

Fortune Shop	Green Intelligent Power Saver	It's nice to save a dozen bucks a night on two air conditioners that used to take 30 or 40 degrees a night, but now it's only 10 or so degrees a night	Jingdong prefecture level city in Shandong
Fortune Shop	Green Intelligent Power Saver	Product no manufacturer's name no product qualification certificate, try two days to see the effect of	Jingdong prefecture level city in Shandong
Fortune Shop	Green Intelligent Power Saver	I've been using it for over a month and have saved over $80, so I've repurchased a few more back to save together	Jingdong prefecture level city in Shandong
Fortune Shop	Green Intelligent Power Saver	I haven't used it yet and will comment when I do, but it's well made.	Jingdong prefecture level city in Shandong
Fortune Shop	Green Intelligent Power Saver	It's quite energy efficient. It's really a boon for the home.	Jingdong prefecture level city in Shandong
Fortune Shop	Green Intelligent Power Saver	Energy-saving socket received a month, after using it for a month proved that it is indeed energy-saving, electricity bill saving, will buy it again, thank you!	Jingdong prefecture level city in Shandong
Fortune Shop	Green Intelligent Power Saver	There was a similar product many years ago. Now technology has evolved, buy one to try, according to the instructions in the box, it takes one or two dozen days to show results. Check your electric bill and see what happens.	Jingdong prefecture level city in Shandong
Fortune Shop	Green Intelligent Power Saver	Jingdong logistics is really fast, the same day the order arrived on the same day, more convenient than the supermarket, and a wide range of choices, the	Jingdong prefecture level city in Shandong
Fortune Shop	Green Intelligent Power Saver	I've been using this for a month or so and I can't see any results, it really is a scam as they say.	Jingdong prefecture level city in Shandong
Fortune Shop	Green Intelligent Power Saver	Very good use, a month can save a few degrees of power loss of electricity, introduced to the relatives to buy back to try.	Jingdong prefecture level city in Shandong
Fortune Shop	Green Intelligent Power Saver	It's really powerful! With this air conditioner can be turned on at any time without the pressure of paying the electricity bill! It's good for the people!	Jingdong prefecture level city in Shandong
Fortune Shop	Green Intelligent Power Saver	This business is a big ** products are not qualified, with a month of electricity more than one hundred percent of thirty, the purchase agreed to return the goods but do not receive a refund after receiving the goods, looking for the official side does not help, the Jingdong is a big **, I have never shopped in	Jingdong prefecture level city in Shandong
Fortune Shop	Green Intelligent Power Saver	It's good. I tried it. It saves power.	Jingdong prefecture level city in Shandong
Fortune Shop	Green Intelligent Power Saver	Nothing**, it seems like there's more	Jingdong prefecture level city in Shandong
Fortune Shop	Green Intelligent Power Saver	Power saving device received, well packaged, good quality, fast logistics	Jingdong prefecture level city in Shandong
Fortune Shop	Green Intelligent Power Saver	The service is okay, is that when I bought five, the desirable pieces are three, and then wait a few days and send me two replacement, I do not know if it is a mistake of the shipper, if I was not in the courier there to see, go home to say no, the	Jingdong prefecture level city in Shandong
Fortune Shop	Green Intelligent Power Saver	This thing will only cost me more if I use it, but before I used it, it was less than a ~ electricity a month, and after I used it, it was more than two hundred kWh a month.	Jingdong prefecture level city in Shandong
Fortune Shop	Green Intelligent Power Saver	I just used it. I don't know how it is.	Jingdong prefecture level city in Shandong
Fortune Shop	Green Intelligent Power Saver	I've received the product and I'm using it, so I'll see how it goes in a month!	Jingdong prefecture level city in Shandong
Fortune Shop	Green Intelligent Power Saver	The goods have just received the effect do not know how, please look forward to the results.	Jingdong prefecture level city in Shandong
Fortune Shop	Green Intelligent Power Saver	Power saving device, the material is very good practical and durable, the size is suitable, packaging is very good, the price is cheap, logistics is very fast!	Jingdong prefecture level city in Shandong
Fortune Shop	Green Intelligent Power Saver	Very nice, will comment in detail after using it for a while.	Jingdong prefecture level city in Shandong
Fortune Shop	Green Intelligent Power Saver	Good stuff to trust and support	Jingdong prefecture level city in Shandong
Fortune Shop	Green Intelligent Power Saver	I'll comment after I use it, doubt if it really saves power no	Jingdong prefecture level city in Shandong
Fortune Shop	Green Intelligent Power Saver	It's good quality and works well!	Jingdong prefecture level city in Shandong
Fortune Shop	Green Intelligent Power Saver	hahaha just right hahahahahaha hiccup hahahaha	Jingdong prefecture level city in Shandong
Fortune Shop	Green Intelligent Power Saver	The real thing matches the picture and looks great!	Jingdong prefecture level city in Shandong
Fortune Shop	Green Intelligent Power Saver	Plugged it in and we'll see when we pay the bills at the end of the year!	Jingdong prefecture level city in Shandong
Fortune Shop	Green Intelligent Power Saver	False advertising. It doesn't work.	Jingdong prefecture level city in Shandong
Fortune Shop	Green Intelligent Power Saver	Goods received, good effect	Jingdong prefecture level city in Shandong
Fortune Shop	Green Intelligent Power Saver	It looks okay, we'll see if it saves electricity when the time comes	Jingdong prefecture level city in Shandong
Fortune Shop	Green Intelligent Power Saver	Logistics is very fast quality is very good, the price is very affordable, accessories are also complete, installed, satisfied with good use	Jingdong prefecture level city in Shandong
Fortune Shop	Green Intelligent Power Saver	I've received the power saver. I'm looking forward to the savings.	Jingdong prefecture level city in Shandong
Fortune Shop	Green Intelligent Power Saver	Received, have not yet used, after use and then evaluate	Jingdong prefecture level city in Shandong
Fortune Shop	Green Intelligent Power Saver	I received the item, but I don't know if it saves power or not.	Jingdong prefecture level city in Shandong
Fortune Shop	Green Intelligent Power Saver	On Trial	Jingdong prefecture level city in Shandong
Fortune Shop	Green Intelligent Power Saver	Use it and see how it goes.	Jingdong prefecture level city in Shandong
Fortune Shop	Green Intelligent Power Saver	Quality is very good, no odor, fine workmanship, grain color are very like, satisfied!	Jingdong prefecture level city in Shandong
Fortune Shop	Green Intelligent Power Saver	I'll give you a good review. I don't know if it'll work.	Jingdong prefecture level city in Shandong
Fortune Shop	Green Intelligent Power Saver	The logistics is very fast and the package is intact. Good use of affordable. The effect is very good, is genuine	Jingdong prefecture level city in Shandong
Fortune Shop	Green Intelligent Power Saver	Nothing happens when you plug it in.	Jingdong prefecture level city in Shandong
Fortune Shop	Green Intelligent Power Saver	I don't know if it's as good as it sounds.	Jingdong prefecture level city in Shandong
Fortune Shop	Green Intelligent Power Saver	The quality of the stuff is very good, exactly the same as the seller's description, very satisfied!	Jingdong prefecture level city in Shandong
Fortune Shop	Green Intelligent Power Saver	The saver works great, it really works, it's affordable and worth buying.	Jingdong prefecture level city in Shandong
Fortune Shop	Green Intelligent Power Saver	Just used it for a couple days. I don't know yet.	Jingdong prefecture level city in Shandong
Fortune Shop	Green Intelligent Power Saver	The quality is quite good, cheaper than the physical store, good, next time will come again!	Jingdong prefecture level city in Shandong
Fortune Shop	Green Intelligent Power Saver	Good quality, cheap and affordable worth recommending to buy!	Jingdong prefecture level city in Shandong
Fortune Shop	Green Intelligent Power Saver	Very good quality, well made, works well! Satisfied	Jingdong prefecture level city in Shandong
Fortune Shop	Green Intelligent Power Saver	The logistics is very fast quality is very good very pleasant a shopping	Jingdong prefecture level city in Shandong
Fortune Shop	Green Intelligent Power Saver	Use it and then come back to evaluate!	Jingdong prefecture level city in Shandong

Fortune Shop	Green Intelligent Power Saver	# It's no use lying at all #	Jingdong prefecture level city in Shandong
Fortune Shop	Green Intelligent Power Saver	I can't get a voltage drop.	Jingdong prefecture level city in Shandong
Fortune Shop	Green Intelligent Power Saver	Evaluate after use	Jingdong prefecture level city in Shandong
Fortune Shop	Green Intelligent Power Saver	Still on trial.	Jingdong prefecture level city in Shandong
Fortune Shop	Green Intelligent Power Saver	ineffective	Jingdong prefecture level city in Shandong
Fortune Shop	Green Intelligent Power Saver	Satisfaction Shopping	Jingdong prefecture level city in Shandong
Fortune Shop	Green Intelligent Power Saver	Lies.	Jingdong prefecture level city in Shandong
Fortune Shop	Green Intelligent Power Saver	It's a real power saver.	Jingdong prefecture level city in Shandong
Fortune Shop	Green Intelligent Power Saver	in use	Jingdong prefecture level city in Shandong
Fortune Shop	Green Intelligent Power Saver	I don't know yet.	Jingdong prefecture level city in Shandong
Fortune Shop	Green Intelligent Power Saver	not care about anything	Jingdong prefecture level city in Shandong
Fortune Shop	Green Intelligent Power Saver	It doesn't seem to have much effect	Jingdong prefecture level city in Shandong
Fortune Shop	Green Intelligent Power Saver	Let the merchant see for himself, does the plug positive and negative terminals look like this?	Jingdong prefecture level city in Shandong
Fortune Shop	Green Intelligent Power Saver	It doesn't work well, the electricity bill is less before using it, but more after using it, contacting customer service, the service is still not good, and returning it is slow	Jingdong prefecture level city in Shandong
Fortune Shop	Green Intelligent Power Saver	With the advertisement product does not match, above the effective negative, power saving rate of two rows of words it, the product looks very thin, with 3 days to see the meter number is still with the previous no difference, which is obviously fraudulent behavior!	Jingdong prefecture level city in Shandong
Fortune Shop	Green Intelligent Power Saver	Doesn't save any power at all. Not much use.	Jingdong prefecture level city in Shandong
Fortune Shop	Green Intelligent Power Saver	The plugs are all loose, they're all the same length and they don't work at all	Jingdong prefecture level city in Shandong
Fortune Shop	Green Intelligent Power Saver	It's bad 👎 it's not effective at all, you'd be crazy to sell this garbage on the internet.	Jingdong prefecture level city in Shandong
Fortune Shop	Green Intelligent Power Saver	It doesn't save any power at all, so I hope the old timers don't fall for this scam again	Jingdong prefecture level city in Shandong
Fortune Shop	Green Intelligent Power Saver	I've been using it for over a month now and haven't noticed any power savings.	Jingdong prefecture level city in Shandong
Fortune Shop	Green Intelligent Power Saver	It doesn't work at all. The electricity bill is higher when you use it.	Jingdong prefecture level city in Shandong
Fortune Shop	Green Intelligent Power Saver	It doesn't save any power, it's still as bad as ever.	Jingdong prefecture level city in Shandong
Fortune Shop	Green Intelligent Power Saver	Last night before leaving work to look at the table, and then plugged into the node device, this morning and then look at the table, than the original save a few degrees of electricity, very good to have a power-saving effect, easy to use, satisfied with the shopping praise.	Jingdong prefecture level city in Shandong
Fortune Shop	Green Intelligent Power Saver	The product is real and effective, the electricity bill is reduced, really good, thanks to the merchants have so many good products, the product is real and effective, the electricity bill is reduced, want to save electricity friends quickly buy it, early use early to save money.	Jingdong prefecture level city in Shandong
Fortune Shop	Green Intelligent Power Saver	Saver saves time and money, baby appearance is beautiful, the quality of goods is good, and the same as the business description, good service attitude, packaging is complete, logistics is very fast, praise to	Jingdong prefecture level city in Shandong
Fortune Shop	Green Intelligent Power Saver	The effect is good, summer, home electricity is relatively large, buy and use a few days, feel the effect is very good, originally ten dollars a day electricity bill, now eight dollars a day, quite good	Jingdong prefecture level city in Shandong
Fortune Shop	Green Intelligent Power Saver	Indeed can save electricity and money, the first time I know this or from a friend's home to see, but also do not believe it, and now they use it to know that there is this good thing.	Jingdong prefecture level city in Shandong
Fortune Shop	Green Intelligent Power Saver	I have received the Smart Power Saver. Perfectly packaged. Fast delivery. Boss customer service attitude is also very good. It's worth buying. I plugged it in and tried it and it really saves more power than the original. I'll buy it again next time.	Jingdong prefecture level city in Shandong
Fortune Shop	Green Intelligent Power Saver	It's nice to save a dozen bucks a night on two air conditioners that used to take 30 or 40 degrees a night, but now it's only 10 or so degrees a night	Jingdong prefecture level city in Shandong
Fortune Shop	Green Intelligent Power Saver	Product no manufacturer's name no product qualification certificate, try two days to see the effect of	Jingdong prefecture level city in Shandong
Fortune Shop	Green Intelligent Power Saver	I've been using it for over a month and have saved over $80, so I've repurchased a few more back to save together	Jingdong prefecture level city in Shandong
Fortune Shop	Green Intelligent Power Saver	I haven't used it yet and will comment when I do, but it's well made.	Jingdong prefecture level city in Shandong
Fortune Shop	Green Intelligent Power Saver	It's quite energy efficient. It's really a boon for the home.	Jingdong prefecture level city in Shandong
Fortune Shop	Green Intelligent Power Saver	Energy-saving socket received a month, after using it for a month proved that it is indeed energy-saving, electricity bill saving, will buy it again, thank you!	Jingdong prefecture level city in Shandong
Fortune Shop	Green Intelligent Power Saver	There was a similar product many years ago. Now technology has evolved, buy one to try, according to the instructions in the box, it takes one or two dozen days to show results. Check your electric bill and see what happens.	Jingdong prefecture level city in Shandong
Fortune Shop	Green Intelligent Power Saver	Jingdong logistics is really fast, the same day the order arrived on the same day, more convenient than the supermarket, and a wide range of choices, the	Jingdong prefecture level city in Shandong
Fortune Shop	Green Intelligent Power Saver	I've been using this for a month or so and I can't see any results, it really is a scam as they say.	Jingdong prefecture level city in Shandong
Fortune Shop	Green Intelligent Power Saver	Very good use, a month can save a few degrees of power loss of electricity, introduced to the relatives to buy back to try.	Jingdong prefecture level city in Shandong
Fortune Shop	Green Intelligent Power Saver	It's really powerful! With this air conditioner can be turned on at any time without the pressure of paying the electricity bill! It's good for the people!	Jingdong prefecture level city in Shandong
Fortune Shop	Green Intelligent Power Saver	This business is a big ** products are not qualified, with a month of electricity more than one hundred percent of thirty, the purchase agreed to return the goods but do not receive a refund after receiving the goods, looking for the official side does not help, the Jingdong is a big **, I have never shopped in	Jingdong prefecture level city in Shandong
Fortune Shop	Green Intelligent Power Saver	It's good. I tried it. It saves power.	Jingdong prefecture level city in Shandong
Fortune Shop	Green Intelligent Power Saver	Nothing**, it seems like there's more	Jingdong prefecture level city in Shandong
Fortune Shop	Green Intelligent Power Saver	Power saving device received, well packaged, good quality, fast logistics	Jingdong prefecture level city in Shandong
Fortune Shop	Green Intelligent Power Saver	The service is okay, is that when I bought five, the desirable pieces are three, and then wait a few days and send me two replacement, I do not know if it is a mistake of the shipper, if I was not in the courier there to see, go home to say no, the	Jingdong prefecture level city in Shandong
Fortune Shop	Green Intelligent Power Saver	This thing will only cost me more if I use it, but before I used it, it was less than a ~ electricity a month, and after I used it, it was more than two hundred kWh a month.	Jingdong prefecture level city in Shandong
Fortune Shop	Green Intelligent Power Saver	I just used it. I don't know how it is.	Jingdong prefecture level city in Shandong
Fortune Shop	Green Intelligent Power Saver	I've received the product and I'm using it, so I'll see how it goes in a month!	Jingdong prefecture level city in Shandong
Fortune Shop	Green Intelligent Power Saver	The goods have just received the effect do not know how, please look forward to the results.	Jingdong prefecture level city in Shandong

Fortune Shop	Green Intelligent Power Saver	Power saving device, the material is very good practical and durable, the size is suitable, packaging is very good, the price is cheap, logistics is very fast!	Jingdong prefecture level city in Shandong
Fortune Shop	Green Intelligent Power Saver	Very nice, will comment in detail after using it for a while.	Jingdong prefecture level city in Shandong
Fortune Shop	Green Intelligent Power Saver	Good stuff to trust and support	Jingdong prefecture level city in Shandong
Fortune Shop	Green Intelligent Power Saver	I'll comment after I use it, doubt if it really saves power no	Jingdong prefecture level city in Shandong
Fortune Shop	Green Intelligent Power Saver	It's good quality and works well!	Jingdong prefecture level city in Shandong
Fortune Shop	Green Intelligent Power Saver	hahaha just right hahahahahaha hiccup hahahaha	Jingdong prefecture level city in Shandong
Fortune Shop	Green Intelligent Power Saver	The real thing matches the picture and looks great!	Jingdong prefecture level city in Shandong
Fortune Shop	Green Intelligent Power Saver	Plugged it in and we'll see when we pay the bills at the end of the year!	Jingdong prefecture level city in Shandong
Fortune Shop	Green Intelligent Power Saver	False advertising. It doesn't work.	Jingdong prefecture level city in Shandong
Fortune Shop	Green Intelligent Power Saver	Goods received, good effect	Jingdong prefecture level city in Shandong
Fortune Shop	Green Intelligent Power Saver	It looks okay, we'll see if it saves electricity when the time comes	Jingdong prefecture level city in Shandong
Fortune Shop	Green Intelligent Power Saver	Logistics is very fast quality is very good, the price is very affordable, accessories are also complete, installed, satisfied with good use	Jingdong prefecture level city in Shandong
Fortune Shop	Green Intelligent Power Saver	I've received the power saver. I'm looking forward to the savings.	Jingdong prefecture level city in Shandong
Fortune Shop	Green Intelligent Power Saver	Received, have not yet used, after use and then evaluate	Jingdong prefecture level city in Shandong
Fortune Shop	Green Intelligent Power Saver	I received the item, but I don't know if it saves power or not.	Jingdong prefecture level city in Shandong
Fortune Shop	Green Intelligent Power Saver	On Trial	Jingdong prefecture level city in Shandong
Fortune Shop	Green Intelligent Power Saver	Use it and see how it goes.	Jingdong prefecture level city in Shandong
Fortune Shop	Green Intelligent Power Saver	Quality is very good, no odor, fine workmanship, grain color are very like, satisfied!	Jingdong prefecture level city in Shandong
Fortune Shop	Green Intelligent Power Saver	I'll give you a good review. I don't know if it'll work.	Jingdong prefecture level city in Shandong
Fortune Shop	Green Intelligent Power Saver	The logistics is very fast and the package is intact. Good use of affordable. The effect is very good, is genuine	Jingdong prefecture level city in Shandong
Fortune Shop	Green Intelligent Power Saver	Nothing happens when you plug it in.	Jingdong prefecture level city in Shandong
Fortune Shop	Green Intelligent Power Saver	I don't know if it's as good as it sounds.	Jingdong prefecture level city in Shandong
Fortune Shop	Green Intelligent Power Saver	The quality of the stuff is very good, exactly the same as the seller's description, very satisfied!	Jingdong prefecture level city in Shandong
Fortune Shop	Green Intelligent Power Saver	The saver works great, it really works, it's affordable and worth buying.	Jingdong prefecture level city in Shandong
Fortune Shop	Green Intelligent Power Saver	Just used it for a couple days. I don't know yet.	Jingdong prefecture level city in Shandong
Fortune Shop	Green Intelligent Power Saver	The quality is quite good, cheaper than the physical store, good, next time will come again!	Jingdong prefecture level city in Shandong
Fortune Shop	Green Intelligent Power Saver	Good quality, cheap and affordable worth recommending to buy!	Jingdong prefecture level city in Shandong
Fortune Shop	Green Intelligent Power Saver	Very good quality, well made, works well! Satisfied	Jingdong prefecture level city in Shandong
Fortune Shop	Green Intelligent Power Saver	The logistics is very fast quality is very good very pleasant a shopping	Jingdong prefecture level city in Shandong
Fortune Shop	Green Intelligent Power Saver	Use it and then come back to evaluate!	Jingdong prefecture level city in Shandong
Fortune Shop	Green Intelligent Power Saver	# It's no use lying at all #	Jingdong prefecture level city in Shandong
Fortune Shop	Green Intelligent Power Saver	I can't get a voltage drop.	Jingdong prefecture level city in Shandong
Fortune Shop	Green Intelligent Power Saver	Evaluate after use	Jingdong prefecture level city in Shandong
Fortune Shop	Green Intelligent Power Saver	Still on trial.	Jingdong prefecture level city in Shandong
Fortune Shop	Green Intelligent Power Saver	ineffective	Jingdong prefecture level city in Shandong
Fortune Shop	Green Intelligent Power Saver	Satisfaction Shopping	Jingdong prefecture level city in Shandong
Fortune Shop	Green Intelligent Power Saver	Lies.	Jingdong prefecture level city in Shandong
Fortune Shop	Green Intelligent Power Saver	It's a real power saver.	Jingdong prefecture level city in Shandong
Fortune Shop	Green Intelligent Power Saver	in use	Jingdong prefecture level city in Shandong
Fortune Shop	Green Intelligent Power Saver	I don't know yet.	Jingdong prefecture level city in Shandong
Fortune Shop	Green Intelligent Power Saver	not care about anything	Jingdong prefecture level city in Shandong
Fortune Shop	Green Intelligent Power Saver	It doesn't seem to have much effect	Jingdong prefecture level city in Shandong
Fortune Shop	Green Intelligent Power Saver	Let the merchant see for himself, does the plug positive and negative terminals look like this?	Jingdong prefecture level city in Shandong
Fortune Shop	Green Intelligent Power Saver	It doesn't work well, the electricity bill is less before using it, but more after using it, contacting customer service, the service is still not good, and returning it is slow	Jingdong prefecture level city in Shandong
Fortune Shop	Green Intelligent Power Saver	With the advertisement product does not match, above the effective negative, power saving rate of two rows of words it, the product looks very thin, with 3 days to see the meter number is still with the previous no difference, which is obviously fraudulent behavior!	Jingdong prefecture level city in Shandong
Fortune Shop	Green Intelligent Power Saver	Doesn't save any power at all. Not much use.	Jingdong prefecture level city in Shandong
Fortune Shop	Green Intelligent Power Saver	The plugs are all loose, they're all the same length and they don't work at all	Jingdong prefecture level city in Shandong
Fortune Shop	Green Intelligent Power Saver	It's bad 👎 it's not effective at all, you'd be crazy to sell this garbage on the internet.	Jingdong prefecture level city in Shandong
Fortune Shop	Green Intelligent Power Saver	It doesn't save any power at all, so I hope the old timers don't fall for this scam again	Jingdong prefecture level city in Shandong
Fortune Shop	Green Intelligent Power Saver	I've been using it for over a month now and haven't noticed any power savings.	Jingdong prefecture level city in Shandong
Fortune Shop	Green Intelligent Power Saver	It doesn't work at all. The electricity bill is higher when you use it.	Jingdong prefecture level city in Shandong

Fortune Shop	Green Intelligent Power Saver	It doesn't save any power, it's still as bad as ever.	Jingdong prefecture level city in Shandong
Fortune Shop	Green Intelligent Power Saver	Last night before leaving work to look at the table, and then plugged into the node device, this morning and then look at the table, than the original save a few degrees of electricity, very good to have a power-saving effect, easy to use, satisfied with the shopping praise.	Jingdong prefecture level city in Shandong
Fortune Shop	Green Intelligent Power Saver	The product is real and effective, the electricity bill is reduced, really good, thanks to the merchants have so many good products, the product is real and effective, the electricity bill is reduced, want to save electricity friends quickly buy it, early use early to save money.	Jingdong prefecture level city in Shandong
Fortune Shop	Green Intelligent Power Saver	Saver saves time and money, baby appearance is beautiful, the quality of goods is good, and the same as the business description, good service attitude, packaging is complete, logistics is very fast, praise to	Jingdong prefecture level city in Shandong
Fortune Shop	Green Intelligent Power Saver	The effect is good, summer, home electricity is relatively large, buy and use a few days, feel the effect is very good, originally ten dollars a day electricity bill, now eight dollars a day, quite good	Jingdong prefecture level city in Shandong
Fortune Shop	Green Intelligent Power Saver	Indeed can save electricity and money, the first time I know this or from a friend's home to see, but also do not believe it, and now they use it to know that there is this good thing.	Jingdong prefecture level city in Shandong
Fortune Shop	Green Intelligent Power Saver	I have received the Smart Power Saver. Perfectly packaged. Fast delivery. Boss customer service attitude is also very good. It's worth buying. I plugged it in and tried it and it really saves more power than the original. I'll buy it again next time.	Jingdong prefecture level city in Shandong
Fortune Shop	Green Intelligent Power Saver	It's nice to save a dozen bucks a night on two air conditioners that used to take 30 or 40 degrees a night, but now it's only 10 or so degrees a night	Jingdong prefecture level city in Shandong
Fortune Shop	Green Intelligent Power Saver	Product no manufacturer's name no product qualification certificate, try two days to see the effect of	Jingdong prefecture level city in Shandong
Fortune Shop	Green Intelligent Power Saver	I've been using it for over a month and have saved over $80, so I've repurchased a few more back to save together	Jingdong prefecture level city in Shandong
Fortune Shop	Green Intelligent Power Saver	I haven't used it yet and will comment when I do, but it's well made.	Jingdong prefecture level city in Shandong
Fortune Shop	Green Intelligent Power Saver	It's quite energy efficient. It's really a boon for the home.	Jingdong prefecture level city in Shandong
Fortune Shop	Green Intelligent Power Saver	Energy-saving socket received a month, after using it for a month proved that it is indeed energy-saving, electricity bill saving, will buy it again, thank you!	Jingdong prefecture level city in Shandong
Fortune Shop	Green Intelligent Power Saver	There was a similar product many years ago. Now technology has evolved, buy one to try, according to the instructions in the box, it takes one or two dozen days to show results. Check your electric bill and see what happens.	Jingdong prefecture level city in Shandong
Fortune Shop	Green Intelligent Power Saver	Jingdong logistics is really fast, the same day the order arrived on the same day, more convenient than the supermarket, and a wide range of choices, the	Jingdong prefecture level city in Shandong
Fortune Shop	Green Intelligent Power Saver	I've been using this for a month or so and I can't see any results, it really is a scam as they say.	Jingdong prefecture level city in Shandong
Fortune Shop	Green Intelligent Power Saver	Very good use, a month can save a few degrees of power loss of electricity, introduced to the relatives to buy back to try.	Jingdong prefecture level city in Shandong
Fortune Shop	Green Intelligent Power Saver	It's really powerful! With this air conditioner can be turned on at any time without the pressure of paying the electricity bill! It's good for the people!	Jingdong prefecture level city in Shandong
Fortune Shop	Green Intelligent Power Saver	This business is a big ** products are not qualified, with a month of electricity more than one hundred percent of thirty, the purchase agreed to return the goods but do not receive a refund after receiving the goods, looking for the official side does not help, the Jingdong is a big **, I have never shopped in	Jingdong prefecture level city in Shandong
Fortune Shop	Green Intelligent Power Saver	It's good. I tried it. It saves power.	Jingdong prefecture level city in Shandong
Fortune Shop	Green Intelligent Power Saver	Nothing**, it seems like there's more	Jingdong prefecture level city in Shandong
Fortune Shop	Green Intelligent Power Saver	Power saving device received, well packaged, good quality, fast logistics	Jingdong prefecture level city in Shandong
Fortune Shop	Green Intelligent Power Saver	The service is okay, is that when I bought five, the desirable pieces are three, and then wait a few days and send me two replacement, I do not know if it is a mistake of the shipper, if I was not in the courier there to see, go home to say no, the	Jingdong prefecture level city in Shandong
Fortune Shop	Green Intelligent Power Saver	This thing will only cost me more if I use it, but before I used it, it was less than a ~ electricity a month, and after I used it, it was more than two hundred kWh a month.	Jingdong prefecture level city in Shandong
Fortune Shop	Green Intelligent Power Saver	I just used it. I don't know how it is.	Jingdong prefecture level city in Shandong
Fortune Shop	Green Intelligent Power Saver	I've received the product and I'm using it, so I'll see how it goes in a month!	Jingdong prefecture level city in Shandong
Fortune Shop	Green Intelligent Power Saver	The goods have just received the effect do not know how, please look forward to the results.	Jingdong prefecture level city in Shandong
Fortune Shop	Green Intelligent Power Saver	Power saving device, the material is very good practical and durable, the size is suitable, packaging is very good, the price is cheap, logistics is very fast!	Jingdong prefecture level city in Shandong
Fortune Shop	Green Intelligent Power Saver	Very nice, will comment in detail after using it for a while.	Jingdong prefecture level city in Shandong
Fortune Shop	Green Intelligent Power Saver	Good stuff to trust and support	Jingdong prefecture level city in Shandong
Fortune Shop	Green Intelligent Power Saver	I'll comment after I use it, doubt if it really saves power no	Jingdong prefecture level city in Shandong
Fortune Shop	Green Intelligent Power Saver	It's good quality and works well!	Jingdong prefecture level city in Shandong
Fortune Shop	Green Intelligent Power Saver	hahaha just right hahahahahaha hiccup hahahaha	Jingdong prefecture level city in Shandong
Fortune Shop	Green Intelligent Power Saver	The real thing matches the picture and looks great!	Jingdong prefecture level city in Shandong
Fortune Shop	Green Intelligent Power Saver	Plugged it in and we'll see when we pay the bills at the end of the year!	Jingdong prefecture level city in Shandong
Fortune Shop	Green Intelligent Power Saver	False advertising. It doesn't work.	Jingdong prefecture level city in Shandong
Fortune Shop	Green Intelligent Power Saver	Goods received, good effect	Jingdong prefecture level city in Shandong
Fortune Shop	Green Intelligent Power Saver	It looks okay, we'll see if it saves electricity when the time comes	Jingdong prefecture level city in Shandong
Fortune Shop	Green Intelligent Power Saver	Logistics is very fast quality is very good, the price is very affordable, accessories are also complete, installed, satisfied with good use	Jingdong prefecture level city in Shandong
Fortune Shop	Green Intelligent Power Saver	I've received the power saver. I'm looking forward to the savings.	Jingdong prefecture level city in Shandong
Fortune Shop	Green Intelligent Power Saver	Received, have not yet used, after use and then evaluate	Jingdong prefecture level city in Shandong
Fortune Shop	Green Intelligent Power Saver	I received the item, but I don't know if it saves power or not.	Jingdong prefecture level city in Shandong
Fortune Shop	Green Intelligent Power Saver	On Trial	Jingdong prefecture level city in Shandong
Fortune Shop	Green Intelligent Power Saver	Use it and see how it goes.	Jingdong prefecture level city in Shandong
Fortune Shop	Green Intelligent Power Saver	Quality is very good, no odor, fine workmanship, grain color are very like, satisfied!	Jingdong prefecture level city in Shandong
Fortune Shop	Green Intelligent Power Saver	I'll give you a good review. I don't know if it'll work.	Jingdong prefecture level city in Shandong
Fortune Shop	Green Intelligent Power Saver	The logistics is very fast and the package is intact. Good use of affordable. The effect is very good, is genuine	Jingdong prefecture level city in Shandong
Fortune Shop	Green Intelligent Power Saver	Nothing happens when you plug it in.	Jingdong prefecture level city in Shandong

Fortune Shop	Green Intelligent Power Saver	I don't know if it's as good as it sounds.	Jingdong prefecture level city in Shandong
Fortune Shop	Green Intelligent Power Saver	The quality of the stuff is very good, exactly the same as the seller's description, very satisfied!	Jingdong prefecture level city in Shandong
Fortune Shop	Green Intelligent Power Saver	The saver works great, it really works, it's affordable and worth buying.	Jingdong prefecture level city in Shandong
Fortune Shop	Green Intelligent Power Saver	Just used it for a couple days. I don't know yet.	Jingdong prefecture level city in Shandong
Fortune Shop	Green Intelligent Power Saver	The quality is quite good, cheaper than the physical store, good, next time will come again!	Jingdong prefecture level city in Shandong
Fortune Shop	Green Intelligent Power Saver	Good quality, cheap and affordable worth recommending to buy!	Jingdong prefecture level city in Shandong
Fortune Shop	Green Intelligent Power Saver	Very good quality, well made, works well! Satisfied	Jingdong prefecture level city in Shandong
Fortune Shop	Green Intelligent Power Saver	The logistics is very fast quality is very good very pleasant a shopping	Jingdong prefecture level city in Shandong
Fortune Shop	Green Intelligent Power Saver	Use it and then come back to evaluate!	Jingdong prefecture level city in Shandong
Fortune Shop	Green Intelligent Power Saver	# It's no use lying at all #	Jingdong prefecture level city in Shandong
Fortune Shop	Green Intelligent Power Saver	I can't get a voltage drop.	Jingdong prefecture level city in Shandong
Fortune Shop	Green Intelligent Power Saver	Evaluate after use	Jingdong prefecture level city in Shandong
Fortune Shop	Green Intelligent Power Saver	Still on trial.	Jingdong prefecture level city in Shandong
Fortune Shop	Green Intelligent Power Saver	ineffective	Jingdong prefecture level city in Shandong
Fortune Shop	Green Intelligent Power Saver	Satisfaction Shopping	Jingdong prefecture level city in Shandong
Fortune Shop	Green Intelligent Power Saver	Lies.	Jingdong prefecture level city in Shandong
Fortune Shop	Green Intelligent Power Saver	It's a real power saver.	Jingdong prefecture level city in Shandong
Fortune Shop	Green Intelligent Power Saver	in use	Jingdong prefecture level city in Shandong
Fortune Shop	Green Intelligent Power Saver	I don't know yet.	Jingdong prefecture level city in Shandong
Fortune Shop	Green Intelligent Power Saver	not care about anything	Jingdong prefecture level city in Shandong
Fortune Shop	Green Intelligent Power Saver	It doesn't seem to have much effect	Jingdong prefecture level city in Shandong
Fortune Shop	Green Intelligent Power Saver	Let the merchant see for himself, does the plug positive and negative terminals look like this?	Jingdong prefecture level city in Shandong
Fortune Shop	Green Intelligent Power Saver	It doesn't work well, the electricity bill is less before using it, but more after using it, contacting customer service, the service is still not good, and returning it is slow	Jingdong prefecture level city in Shandong
Fortune Shop	Green Intelligent Power Saver	With the advertisement product does not match, above the effective negative, power saving rate of two rows of words it, the product looks very thin, with 3 days to see the meter number is still with the previous no difference, which is obviously fraudulent behavior!	Jingdong prefecture level city in Shandong
Fortune Shop	Green Intelligent Power Saver	Doesn't save any power at all. Not much use.	Jingdong prefecture level city in Shandong
Fortune Shop	Green Intelligent Power Saver	The plugs are all loose, they're all the same length and they don't work at all	Jingdong prefecture level city in Shandong
Fortune Shop	Green Intelligent Power Saver	It's bad 👎 it's not effective at all, you'd be crazy to sell this garbage on the internet.	Jingdong prefecture level city in Shandong
Fortune Shop	Green Intelligent Power Saver	It doesn't save any power at all, so I hope the old timers don't fall for this scam again	Jingdong prefecture level city in Shandong
Fortune Shop	Green Intelligent Power Saver	I've been using it for over a month now and haven't noticed any power savings.	Jingdong prefecture level city in Shandong
Fortune Shop	Green Intelligent Power Saver	It doesn't work at all. The electricity bill is higher when you use it.	Jingdong prefecture level city in Shandong
Fortune Shop	Green Intelligent Power Saver	It doesn't save any power, it's still as bad as ever.	Jingdong prefecture level city in Shandong
Fortune Shop	Green Intelligent Power Saver	Last night before leaving work to look at the table, and then plugged into the node device, this morning and then look at the table, than the original save a few degrees of electricity, very good to have a power-saving effect, easy to use, satisfied with the shopping praise.	Jingdong prefecture level city in Shandong
Fortune Shop	Green Intelligent Power Saver	The product is real and effective, the electricity bill is reduced, really good, thanks to the merchants have so many good products, the product is real and effective, the electricity bill is reduced, want to save electricity friends quickly buy it, early use early to save money.	Jingdong prefecture level city in Shandong
Fortune Shop	Green Intelligent Power Saver	Saver saves time and money, baby appearance is beautiful, the quality of goods is good, and the same as the business description, good service attitude, packaging is complete, logistics is very fast, praise to	Jingdong prefecture level city in Shandong
Fortune Shop	Green Intelligent Power Saver	The effect is good, summer, home electricity is relatively large, buy and use a few days, feel the effect is very good, originally ten dollars a day electricity bill, now eight dollars a day, quite good	Jingdong prefecture level city in Shandong
Fortune Shop	Green Intelligent Power Saver	Indeed can save electricity and money, the first time I know this or from a friend's home to see, but also do not believe it, and now they use it to know that there is this good thing.	Jingdong prefecture level city in Shandong
Fortune Shop	Green Intelligent Power Saver	I have received the Smart Power Saver. Perfectly packaged. Fast delivery. Boss customer service attitude is also very good. It's worth buying. I plugged it in and tried it and it really saves more power than the original. I'll buy it again next time.	Jingdong prefecture level city in Shandong
Fortune Shop	Green Intelligent Power Saver	It's nice to save a dozen bucks a night on two air conditioners that used to take 30 or 40 degrees a night, but now it's only 10 or so degrees a night	Jingdong prefecture level city in Shandong
Fortune Shop	Green Intelligent Power Saver	Product no manufacturer's name no product qualification certificate, try two days to see the effect of	Jingdong prefecture level city in Shandong
Fortune Shop	Green Intelligent Power Saver	I've been using it for over a month and have saved over $80, so I've repurchased a few more back to save together	Jingdong prefecture level city in Shandong
Fortune Shop	Green Intelligent Power Saver	I haven't used it yet and will comment when I do, but it's well made.	Jingdong prefecture level city in Shandong
Fortune Shop	Green Intelligent Power Saver	It's quite energy efficient. It's really a boon for the home.	Jingdong prefecture level city in Shandong
Fortune Shop	Green Intelligent Power Saver	Energy-saving socket received a month, after using it for a month proved that it is indeed energy-saving, electricity bill saving, will buy it again, thank you!	Jingdong prefecture level city in Shandong
Fortune Shop	Green Intelligent Power Saver	There was a similar product many years ago. Now technology has evolved, buy one to try, according to the instructions in the box, it takes one or two dozen days to show results. Check your electric bill and see what happens.	Jingdong prefecture level city in Shandong
Fortune Shop	Green Intelligent Power Saver	Jingdong logistics is really fast, the same day the order arrived on the same day, more convenient than the supermarket, and a wide range of choices, the	Jingdong prefecture level city in Shandong
Fortune Shop	Green Intelligent Power Saver	I've been using this for a month or so and I can't see any results, it really is a scam as they say.	Jingdong prefecture level city in Shandong
Fortune Shop	Green Intelligent Power Saver	Very good use, a month can save a few degrees of power loss of electricity, introduced to the relatives to buy back to try.	Jingdong prefecture level city in Shandong
Fortune Shop	Green Intelligent Power Saver	It's really powerful! With this air conditioner can be turned on at any time without the pressure of paying the electricity bill! It's good for the people!	Jingdong prefecture level city in Shandong

Fortune Shop	Green Intelligent Power Saver	This business is a big ** products are not qualified, with a month of electricity more than one hundred percent of thirty, the purchase agreed to return the goods but do not receive a refund after receiving the goods, looking for the official side does not help, the Jingdong is a big **, I have never shopped in	Jingdong prefecture level city in Shandong
Fortune Shop	Green Intelligent Power Saver	It's good. I tried it. It saves power.	Jingdong prefecture level city in Shandong
Fortune Shop	Green Intelligent Power Saver	Nothing**, it seems like there's more	Jingdong prefecture level city in Shandong
Fortune Shop	Green Intelligent Power Saver	Power saving device received, well packaged, good quality, fast logistics	Jingdong prefecture level city in Shandong
Fortune Shop	Green Intelligent Power Saver	The service is okay, is that when I bought five, the desirable pieces are three, and then wait a few days and send me two replacement, I do not know if it is a mistake of the shipper, if I was not in the courier there to see, go home to say no, the	Jingdong prefecture level city in Shandong
Fortune Shop	Green Intelligent Power Saver	This thing will only cost me more if I use it, but before I used it, it was less than a ~ electricity a month, and after I used it, it was more than two hundred kWh a month.	Jingdong prefecture level city in Shandong
Fortune Shop	Green Intelligent Power Saver	I just used it. I don't know how it is.	Jingdong prefecture level city in Shandong
Fortune Shop	Green Intelligent Power Saver	I've received the product and I'm using it, so I'll see how it goes in a month!	Jingdong prefecture level city in Shandong
Fortune Shop	Green Intelligent Power Saver	The goods have just received the effect do not know how, please look forward to the results.	Jingdong prefecture level city in Shandong
Fortune Shop	Green Intelligent Power Saver	Power saving device, the material is very good practical and durable, the size is suitable, packaging is very good, the price is cheap, logistics is very fast!	Jingdong prefecture level city in Shandong
Fortune Shop	Green Intelligent Power Saver	Very nice, will comment in detail after using it for a while.	Jingdong prefecture level city in Shandong
Fortune Shop	Green Intelligent Power Saver	Good stuff to trust and support	Jingdong prefecture level city in Shandong
Fortune Shop	Green Intelligent Power Saver	I'll comment after I use it, doubt if it really saves power no	Jingdong prefecture level city in Shandong
Fortune Shop	Green Intelligent Power Saver	It's good quality and works well!	Jingdong prefecture level city in Shandong
Fortune Shop	Green Intelligent Power Saver	hahaha just right hahahahahaha hiccup hahahaha	Jingdong prefecture level city in Shandong
Fortune Shop	Green Intelligent Power Saver	The real thing matches the picture and looks great!	Jingdong prefecture level city in Shandong
Fortune Shop	Green Intelligent Power Saver	Plugged it in and we'll see when we pay the bills at the end of the year!	Jingdong prefecture level city in Shandong
Fortune Shop	Green Intelligent Power Saver	False advertising. It doesn't work.	Jingdong prefecture level city in Shandong
Fortune Shop	Green Intelligent Power Saver	Goods received, good effect	Jingdong prefecture level city in Shandong
Fortune Shop	Green Intelligent Power Saver	It looks okay, we'll see if it saves electricity when the time comes	Jingdong prefecture level city in Shandong
Fortune Shop	Green Intelligent Power Saver	Logistics is very fast quality is very good, the price is very affordable, accessories are also complete, installed, satisfied with good use	Jingdong prefecture level city in Shandong
Fortune Shop	Green Intelligent Power Saver	I've received the power saver. I'm looking forward to the savings.	Jingdong prefecture level city in Shandong
Fortune Shop	Green Intelligent Power Saver	Received, have not yet used, after use and then evaluate	Jingdong prefecture level city in Shandong
Fortune Shop	Green Intelligent Power Saver	I received the item, but I don't know if it saves power or not.	Jingdong prefecture level city in Shandong
Fortune Shop	Green Intelligent Power Saver	On Trial	Jingdong prefecture level city in Shandong
Fortune Shop	Green Intelligent Power Saver	Use it and see how it goes.	Jingdong prefecture level city in Shandong
Fortune Shop	Green Intelligent Power Saver	Quality is very good, no odor, fine workmanship, grain color are very like, satisfied!	Jingdong prefecture level city in Shandong
Fortune Shop	Green Intelligent Power Saver	I'll give you a good review. I don't know if it'll work.	Jingdong prefecture level city in Shandong
Fortune Shop	Green Intelligent Power Saver	The logistics is very fast and the package is intact. Good use of affordable. The effect is very good, is genuine	Jingdong prefecture level city in Shandong
Fortune Shop	Green Intelligent Power Saver	Nothing happens when you plug it in.	Jingdong prefecture level city in Shandong
Fortune Shop	Green Intelligent Power Saver	I don't know if it's as good as it sounds.	Jingdong prefecture level city in Shandong
Fortune Shop	Green Intelligent Power Saver	The quality of the stuff is very good, exactly the same as the seller's description, very satisfied!	Jingdong prefecture level city in Shandong
Fortune Shop	Green Intelligent Power Saver	The saver works great, it really works, it's affordable and worth buying.	Jingdong prefecture level city in Shandong
Fortune Shop	Green Intelligent Power Saver	Just used it for a couple days. I don't know yet.	Jingdong prefecture level city in Shandong
Fortune Shop	Green Intelligent Power Saver	The quality is quite good, cheaper than the physical store, good, next time will come again!	Jingdong prefecture level city in Shandong
Fortune Shop	Green Intelligent Power Saver	Good quality, cheap and affordable worth recommending to buy!	Jingdong prefecture level city in Shandong
Fortune Shop	Green Intelligent Power Saver	Very good quality, well made, works well! Satisfied	Jingdong prefecture level city in Shandong
Fortune Shop	Green Intelligent Power Saver	The logistics is very fast quality is very good very pleasant a shopping	Jingdong prefecture level city in Shandong
Fortune Shop	Green Intelligent Power Saver	Use it and then come back to evaluate!	Jingdong prefecture level city in Shandong
Fortune Shop	Green Intelligent Power Saver	# It's no use lying at all #	Jingdong prefecture level city in Shandong
Fortune Shop	Green Intelligent Power Saver	I can't get a voltage drop.	Jingdong prefecture level city in Shandong
Fortune Shop	Green Intelligent Power Saver	Evaluate after use	Jingdong prefecture level city in Shandong
Fortune Shop	Green Intelligent Power Saver	Still on trial.	Jingdong prefecture level city in Shandong
Fortune Shop	Green Intelligent Power Saver	ineffective	Jingdong prefecture level city in Shandong
Fortune Shop	Green Intelligent Power Saver	Satisfaction Shopping	Jingdong prefecture level city in Shandong
Fortune Shop	Green Intelligent Power Saver	Lies.	Jingdong prefecture level city in Shandong
Fortune Shop	Green Intelligent Power Saver	It's a real power saver.	Jingdong prefecture level city in Shandong
Fortune Shop	Green Intelligent Power Saver	in use	Jingdong prefecture level city in Shandong
Fortune Shop	Green Intelligent Power Saver	I don't know yet.	Jingdong prefecture level city in Shandong
Fortune Shop	Green Intelligent Power Saver	not care about anything	Jingdong prefecture level city in Shandong

Fortune Shop	Green Intelligent Power Saver	It doesn't seem to have much effect	Jingdong prefecture level city in Shandong
Fortune Shop	Green Intelligent Power Saver	Let the merchant see for himself, does the plug positive and negative terminals look like this?	Jingdong prefecture level city in Shandong
Fortune Shop	Green Intelligent Power Saver	It doesn't work well, the electricity bill is less before using it, but more after using it, contacting customer service, the service is still not good, and returning it is slow	Jingdong prefecture level city in Shandong
Fortune Shop	Green Intelligent Power Saver	With the advertisement product does not match, above the effective negative, power saving rate of two rows of words it, the product looks very thin, with 3 days to see the meter number is still with the previous no difference, which is obviously fraudulent behavior!	Jingdong prefecture level city in Shandong
Fortune Shop	Green Intelligent Power Saver	Doesn't save any power at all. Not much use.	Jingdong prefecture level city in Shandong
Fortune Shop	Green Intelligent Power Saver	The plugs are all loose, they're all the same length and they don't work at all	Jingdong prefecture level city in Shandong
Fortune Shop	Green Intelligent Power Saver	It's bad 👎 it's not effective at all, you'd be crazy to sell this garbage on the internet.	Jingdong prefecture level city in Shandong
Fortune Shop	Green Intelligent Power Saver	It doesn't save any power at all, so I hope the old timers don't fall for this scam again	Jingdong prefecture level city in Shandong
Fortune Shop	Green Intelligent Power Saver	I've been using it for over a month now and haven't noticed any power savings.	Jingdong prefecture level city in Shandong
Fortune Shop	Green Intelligent Power Saver	It doesn't work at all. The electricity bill is higher when you use it.	Jingdong prefecture level city in Shandong
Fortune Shop	Green Intelligent Power Saver	It doesn't save any power, it's still as bad as ever.	Jingdong prefecture level city in Shandong
Fortune Shop	Green Intelligent Power Saver	Last night before leaving work to look at the table, and then plugged into the node device, this morning and then look at the table, than the original save a few degrees of electricity, very good to have a power-saving effect, easy to use, satisfied with the shopping praise.	Jingdong prefecture level city in Shandong
Fortune Shop	Green Intelligent Power Saver	The product is real and effective, the electricity bill is reduced, really good, thanks to the merchants have so many good products, the product is real and effective, the electricity bill is reduced, want to save electricity friends quickly buy it, early use early to save money.	Jingdong prefecture level city in Shandong
Fortune Shop	Green Intelligent Power Saver	Saver saves time and money, baby appearance is beautiful, the quality of goods is good, and the same as the business description, good service attitude, packaging is complete, logistics is very fast, praise to	Jingdong prefecture level city in Shandong
Fortune Shop	Green Intelligent Power Saver	The effect is good, summer, home electricity is relatively large, buy and use a few days, feel the effect is very good, originally ten dollars a day electricity bill, now eight dollars a day, quite good	Jingdong prefecture level city in Shandong
Fortune Shop	Green Intelligent Power Saver	Indeed can save electricity and money, the first time I know this or from a friend's home to see, but also do not believe it, and now they use it to know that there is this good thing.	Jingdong prefecture level city in Shandong
Fortune Shop	Green Intelligent Power Saver	I have received the Smart Power Saver. Perfectly packaged. Fast delivery. Boss customer service attitude is also very good. It's worth buying. I plugged it in and tried it and it really saves more power than the original. I'll buy it again next time.	Jingdong prefecture level city in Shandong
Fortune Shop	Green Intelligent Power Saver	It's nice to save a dozen bucks a night on two air conditioners that used to take 30 or 40 degrees a night, but now it's only 10 or so degrees a night	Jingdong prefecture level city in Shandong
Fortune Shop	Green Intelligent Power Saver	Product no manufacturer's name no product qualification certificate, try two days to see the effect of	Jingdong prefecture level city in Shandong
Fortune Shop	Green Intelligent Power Saver	I've been using it for over a month and have saved over $80, so I've repurchased a few more back to save together	Jingdong prefecture level city in Shandong
Fortune Shop	Green Intelligent Power Saver	I haven't used it yet and will comment when I do, but it's well made.	Jingdong prefecture level city in Shandong
Fortune Shop	Green Intelligent Power Saver	It's quite energy efficient. It's really a boon for the home.	Jingdong prefecture level city in Shandong
Fortune Shop	Green Intelligent Power Saver	Energy-saving socket received a month, after using it for a month proved that it is indeed energy-saving, electricity bill saving, will buy it again, thank you!	Jingdong prefecture level city in Shandong
Fortune Shop	Green Intelligent Power Saver	There was a similar product many years ago. Now technology has evolved, buy one to try, according to the instructions in the box, it takes one or two dozen days to show results. Check your electric bill and see what happens.	Jingdong prefecture level city in Shandong
Fortune Shop	Green Intelligent Power Saver	Jingdong logistics is really fast, the same day the order arrived on the same day, more convenient than the supermarket, and a wide range of choices, the	Jingdong prefecture level city in Shandong
Fortune Shop	Green Intelligent Power Saver	I've been using this for a month or so and I can't see any results, it really is a scam as they say.	Jingdong prefecture level city in Shandong
Fortune Shop	Green Intelligent Power Saver	Very good use, a month can save a few degrees of power loss of electricity, introduced to the relatives to buy back to try.	Jingdong prefecture level city in Shandong
Fortune Shop	Green Intelligent Power Saver	It's really powerful! With this air conditioner can be turned on at any time without the pressure of paying the electricity bill! It's good for the people!	Jingdong prefecture level city in Shandong
Fortune Shop	Green Intelligent Power Saver	This business is a big ** products are not qualified, with a month of electricity more than one hundred percent of thirty, the purchase agreed to return the goods but do not receive a refund after receiving the goods, looking for the official side does not help, the Jingdong is a big **, I have never shopped in	Jingdong prefecture level city in Shandong
Fortune Shop	Green Intelligent Power Saver	It's good. I tried it. It saves power.	Jingdong prefecture level city in Shandong
Fortune Shop	Green Intelligent Power Saver	Nothing**, it seems like there's more	Jingdong prefecture level city in Shandong
Fortune Shop	Green Intelligent Power Saver	Power saving device received, well packaged, good quality, fast logistics	Jingdong prefecture level city in Shandong
Fortune Shop	Green Intelligent Power Saver	The service is okay, is that when I bought five, the desirable pieces are three, and then wait a few days and send me two replacement, I do not know if it is a mistake of the shipper, if I was not in the courier there to see, go home to say no, the	Jingdong prefecture level city in Shandong
Fortune Shop	Green Intelligent Power Saver	This thing will only cost me more if I use it, but before I used it, it was less than a ~ electricity a month, and after I used it, it was more than two hundred kWh a month.	Jingdong prefecture level city in Shandong
Fortune Shop	Green Intelligent Power Saver	I just used it. I don't know how it is.	Jingdong prefecture level city in Shandong
Fortune Shop	Green Intelligent Power Saver	I've received the product and I'm using it, so I'll see how it goes in a month!	Jingdong prefecture level city in Shandong
Fortune Shop	Green Intelligent Power Saver	The goods have just received the effect do not know how, please look forward to the results.	Jingdong prefecture level city in Shandong
Fortune Shop	Green Intelligent Power Saver	Power saving device, the material is very good practical and durable, the size is suitable, packaging is very good, the price is cheap, logistics is very fast!	Jingdong prefecture level city in Shandong
Fortune Shop	Green Intelligent Power Saver	Very nice, will comment in detail after using it for a while.	Jingdong prefecture level city in Shandong
Fortune Shop	Green Intelligent Power Saver	Good stuff to trust and support	Jingdong prefecture level city in Shandong
Fortune Shop	Green Intelligent Power Saver	I'll comment after I use it, doubt if it really saves power no	Jingdong prefecture level city in Shandong
Fortune Shop	Green Intelligent Power Saver	It's good quality and works well!	Jingdong prefecture level city in Shandong
Fortune Shop	Green Intelligent Power Saver	hahaha just right hahahahahaha hiccup hahahaha	Jingdong prefecture level city in Shandong
Fortune Shop	Green Intelligent Power Saver	The real thing matches the picture and looks great!	Jingdong prefecture level city in Shandong
Fortune Shop	Green Intelligent Power Saver	Plugged it in and we'll see when we pay the bills at the end of the year!	Jingdong prefecture level city in Shandong
Fortune Shop	Green Intelligent Power Saver	False advertising. It doesn't work.	Jingdong prefecture level city in Shandong
Fortune Shop	Green Intelligent Power Saver	Goods received, good effect	Jingdong prefecture level city in Shandong
Fortune Shop	Green Intelligent Power Saver	It looks okay, we'll see if it saves electricity when the time comes	Jingdong prefecture level city in Shandong

Fortune Shop	Green Intelligent Power Saver	Logistics is very fast quality is very good, the price is very affordable, accessories are also complete, installed, satisfied with good use	Jingdong prefecture level city in Shandong
Fortune Shop	Green Intelligent Power Saver	I've received the power saver. I'm looking forward to the savings.	Jingdong prefecture level city in Shandong
Fortune Shop	Green Intelligent Power Saver	Received, have not yet used, after use and then evaluate	Jingdong prefecture level city in Shandong
Fortune Shop	Green Intelligent Power Saver	I received the item, but I don't know if it saves power or not.	Jingdong prefecture level city in Shandong
Fortune Shop	Green Intelligent Power Saver	On Trial	Jingdong prefecture level city in Shandong
Fortune Shop	Green Intelligent Power Saver	Use it and see how it goes.	Jingdong prefecture level city in Shandong
Fortune Shop	Green Intelligent Power Saver	Quality is very good, no odor, fine workmanship, grain color are very like, satisfied!	Jingdong prefecture level city in Shandong
Fortune Shop	Green Intelligent Power Saver	I'll give you a good review. I don't know if it'll work.	Jingdong prefecture level city in Shandong
Fortune Shop	Green Intelligent Power Saver	The logistics is very fast and the package is intact. Good use of affordable. The effect is very good, is genuine	Jingdong prefecture level city in Shandong
Fortune Shop	Green Intelligent Power Saver	Nothing happens when you plug it in.	Jingdong prefecture level city in Shandong
Fortune Shop	Green Intelligent Power Saver	I don't know if it's as good as it sounds.	Jingdong prefecture level city in Shandong
Fortune Shop	Green Intelligent Power Saver	The quality of the stuff is very good, exactly the same as the seller's description, very satisfied!	Jingdong prefecture level city in Shandong
Fortune Shop	Green Intelligent Power Saver	The saver works great, it really works, it's affordable and worth buying.	Jingdong prefecture level city in Shandong
Fortune Shop	Green Intelligent Power Saver	Just used it for a couple days. I don't know yet.	Jingdong prefecture level city in Shandong
Fortune Shop	Green Intelligent Power Saver	The quality is quite good, cheaper than the physical store, good, next time will come again!	Jingdong prefecture level city in Shandong
Fortune Shop	Green Intelligent Power Saver	Good quality, cheap and affordable worth recommending to buy!	Jingdong prefecture level city in Shandong
Fortune Shop	Green Intelligent Power Saver	Very good quality, well made, works well! Satisfied	Jingdong prefecture level city in Shandong
Fortune Shop	Green Intelligent Power Saver	The logistics is very fast quality is very good very pleasant a shopping	Jingdong prefecture level city in Shandong
Fortune Shop	Green Intelligent Power Saver	Use it and then come back to evaluate!	Jingdong prefecture level city in Shandong
Fortune Shop	Green Intelligent Power Saver	# It's no use lying at all #	Jingdong prefecture level city in Shandong
Fortune Shop	Green Intelligent Power Saver	I can't get a voltage drop.	Jingdong prefecture level city in Shandong
Fortune Shop	Green Intelligent Power Saver	Evaluate after use	Jingdong prefecture level city in Shandong
Fortune Shop	Green Intelligent Power Saver	Still on trial.	Jingdong prefecture level city in Shandong
Fortune Shop	Green Intelligent Power Saver	ineffective	Jingdong prefecture level city in Shandong
Fortune Shop	Green Intelligent Power Saver	Satisfaction Shopping	Jingdong prefecture level city in Shandong
Fortune Shop	Green Intelligent Power Saver	Lies.	Jingdong prefecture level city in Shandong
Fortune Shop	Green Intelligent Power Saver	It's a real power saver.	Jingdong prefecture level city in Shandong
Fortune Shop	Green Intelligent Power Saver	in use	Jingdong prefecture level city in Shandong
Fortune Shop	Green Intelligent Power Saver	I don't know yet.	Jingdong prefecture level city in Shandong
Fortune Shop	Green Intelligent Power Saver	not care about anything	Jingdong prefecture level city in Shandong
Fortune Shop	Green Intelligent Power Saver	It doesn't seem to have much effect	Jingdong prefecture level city in Shandong
Fortune Shop	Green Intelligent Power Saver	Let the merchant see for himself, does the plug positive and negative terminals look like this?	Jingdong prefecture level city in Shandong
Fortune Shop	Green Intelligent Power Saver	It doesn't work well, the electricity bill is less before using it, but more after using it, contacting customer service, the service is still not good, and returning it is slow	Jingdong prefecture level city in Shandong
Fortune Shop	Green Intelligent Power Saver	With the advertisement product does not match, above the effective negative, power saving rate of two rows of words it, the product looks very thin, with 3 days to see the meter number is still with the previous no difference, which is obviously fraudulent behavior!	Jingdong prefecture level city in Shandong
Fortune Shop	Green Intelligent Power Saver	Doesn't save any power at all. Not much use.	Jingdong prefecture level city in Shandong
Fortune Shop	Green Intelligent Power Saver	The plugs are all loose, they're all the same length and they don't work at all	Jingdong prefecture level city in Shandong
Fortune Shop	Green Intelligent Power Saver	It's bad 👎 it's not effective at all, you'd be crazy to sell this garbage on the internet.	Jingdong prefecture level city in Shandong
Fortune Shop	Green Intelligent Power Saver	It doesn't save any power at all, so I hope the old timers don't fall for this scam again	Jingdong prefecture level city in Shandong
Fortune Shop	Green Intelligent Power Saver	I've been using it for over a month now and haven't noticed any power savings.	Jingdong prefecture level city in Shandong
Fortune Shop	Green Intelligent Power Saver	It doesn't work at all. The electricity bill is higher when you use it.	Jingdong prefecture level city in Shandong
Fortune Shop	Green Intelligent Power Saver	It doesn't save any power, it's still as bad as ever.	Jingdong prefecture level city in Shandong
Fortune Shop	Green Intelligent Power Saver	Last night before leaving work to look at the table, and then plugged into the node device, this morning and then look at the table, than the original save a few degrees of electricity, very good to have a power-saving effect, easy to use, satisfied with the shopping praise.	Jingdong prefecture level city in Shandong
Fortune Shop	Green Intelligent Power Saver	The product is real and effective, the electricity bill is reduced, really good, thanks to the merchants have so many good products, the product is real and effective, the electricity bill is reduced, want to save electricity friends quickly buy it, early use early to save money.	Jingdong prefecture level city in Shandong
Fortune Shop	Green Intelligent Power Saver	Saver saves time and money, baby appearance is beautiful, the quality of goods is good, and the same as the business description, good service attitude, packaging is complete, logistics is very fast, praise to	Jingdong prefecture level city in Shandong
Fortune Shop	Green Intelligent Power Saver	The effect is good, summer, home electricity is relatively large, buy and use a few days, feel the effect is very good, originally ten dollars a day electricity bill, now eight dollars a day, quite good	Jingdong prefecture level city in Shandong
Fortune Shop	Green Intelligent Power Saver	Indeed can save electricity and money, the first time I know this or from a friend's home to see, but also do not believe it, and now they use it to know that there is this good thing.	Jingdong prefecture level city in Shandong
Fortune Shop	Green Intelligent Power Saver	I have received the Smart Power Saver. Perfectly packaged. Fast delivery. Boss customer service attitude is also very good. It's worth buying. I plugged it in and tried it and it really saves more power than the original. I'll buy it again next time.	Jingdong prefecture level city in Shandong
Fortune Shop	Green Intelligent Power Saver	It's nice to save a dozen bucks a night on two air conditioners that used to take 30 or 40 degrees a night, but now it's only 10 or so degrees a night	Jingdong prefecture level city in Shandong
Fortune Shop	Green Intelligent Power Saver	Product no manufacturer's name no product qualification certificate, try two days to see the effect of	Jingdong prefecture level city in Shandong

Fortune Shop	Green Intelligent Power Saver	I've been using it for over a month and have saved over $80, so I've repurchased a few more back to save together	Jingdong prefecture level city in Shandong
Fortune Shop	Green Intelligent Power Saver	I haven't used it yet and will comment when I do, but it's well made.	Jingdong prefecture level city in Shandong
Fortune Shop	Green Intelligent Power Saver	It's quite energy efficient. It's really a boon for the home.	Jingdong prefecture level city in Shandong
Fortune Shop	Green Intelligent Power Saver	Energy-saving socket received a month, after using it for a month proved that it is indeed energy-saving, electricity bill saving, will buy it again, thank you!	Jingdong prefecture level city in Shandong
Fortune Shop	Green Intelligent Power Saver	There was a similar product many years ago. Now technology has evolved, buy one to try, according to the instructions in the box, it takes one or two dozen days to show results. Check your electric bill and see what happens.	Jingdong prefecture level city in Shandong
Fortune Shop	Green Intelligent Power Saver	Jingdong logistics is really fast, the same day the order arrived on the same day, more convenient than the supermarket, and a wide range of choices, the	Jingdong prefecture level city in Shandong
Fortune Shop	Green Intelligent Power Saver	I've been using this for a month or so and I can't see any results, it really is a scam as they say.	Jingdong prefecture level city in Shandong
Fortune Shop	Green Intelligent Power Saver	Very good use, a month can save a few degrees of power loss of electricity, introduced to the relatives to buy back to try.	Jingdong prefecture level city in Shandong
Fortune Shop	Green Intelligent Power Saver	It's really powerful! With this air conditioner can be turned on at any time without the pressure of paying the electricity bill! It's good for the people!	Jingdong prefecture level city in Shandong
Fortune Shop	Green Intelligent Power Saver	This business is a big ** products are not qualified, with a month of electricity more than one hundred percent of thirty, the purchase agreed to return the goods but do not receive a refund after receiving the goods, looking for the official side does not help, the Jingdong is a big **, I have never shopped in	Jingdong prefecture level city in Shandong
Fortune Shop	Green Intelligent Power Saver	It's good. I tried it. It saves power.	Jingdong prefecture level city in Shandong
Fortune Shop	Green Intelligent Power Saver	Nothing**, it seems like there's more	Jingdong prefecture level city in Shandong
Fortune Shop	Green Intelligent Power Saver	Power saving device received, well packaged, good quality, fast logistics	Jingdong prefecture level city in Shandong
Fortune Shop	Green Intelligent Power Saver	The service is okay, is that when I bought five, the desirable pieces are three, and then wait a few days and send me two replacement, I do not know if it is a mistake of the shipper, if I was not in the courier there to see, go home to say no, the	Jingdong prefecture level city in Shandong
Fortune Shop	Green Intelligent Power Saver	This thing will only cost me more if I use it, but before I used it, it was less than a ~ electricity a month, and after I used it, it was more than two hundred kWh a month.	Jingdong prefecture level city in Shandong
Fortune Shop	Green Intelligent Power Saver	I just used it. I don't know how it is.	Jingdong prefecture level city in Shandong
Fortune Shop	Green Intelligent Power Saver	I've received the product and I'm using it, so I'll see how it goes in a month!	Jingdong prefecture level city in Shandong
Fortune Shop	Green Intelligent Power Saver	The goods have just received the effect do not know how, please look forward to the results.	Jingdong prefecture level city in Shandong
Fortune Shop	Green Intelligent Power Saver	Power saving device, the material is very good practical and durable, the size is suitable, packaging is very good, the price is cheap, logistics is very fast!	Jingdong prefecture level city in Shandong
Fortune Shop	Green Intelligent Power Saver	Very nice, will comment in detail after using it for a while.	Jingdong prefecture level city in Shandong
Fortune Shop	Green Intelligent Power Saver	Good stuff to trust and support	Jingdong prefecture level city in Shandong
Fortune Shop	Green Intelligent Power Saver	I'll comment after I use it, doubt if it really saves power no	Jingdong prefecture level city in Shandong
Fortune Shop	Green Intelligent Power Saver	It's good quality and works well!	Jingdong prefecture level city in Shandong
Fortune Shop	Green Intelligent Power Saver	hahaha just right hahahahahaha hiccup hahahaha	Jingdong prefecture level city in Shandong
Fortune Shop	Green Intelligent Power Saver	The real thing matches the picture and looks great!	Jingdong prefecture level city in Shandong
Fortune Shop	Green Intelligent Power Saver	Plugged it in and we'll see when we pay the bills at the end of the year!	Jingdong prefecture level city in Shandong
Fortune Shop	Green Intelligent Power Saver	False advertising. It doesn't work.	Jingdong prefecture level city in Shandong
Fortune Shop	Green Intelligent Power Saver	Goods received, good effect	Jingdong prefecture level city in Shandong
Fortune Shop	Green Intelligent Power Saver	It looks okay, we'll see if it saves electricity when the time comes	Jingdong prefecture level city in Shandong
Fortune Shop	Green Intelligent Power Saver	Logistics is very fast quality is very good, the price is very affordable, accessories are also complete, installed, satisfied with good use	Jingdong prefecture level city in Shandong
Fortune Shop	Green Intelligent Power Saver	I've received the power saver. I'm looking forward to the savings.	Jingdong prefecture level city in Shandong
Fortune Shop	Green Intelligent Power Saver	Received, have not yet used, after use and then evaluate	Jingdong prefecture level city in Shandong
Fortune Shop	Green Intelligent Power Saver	I received the item, but I don't know if it saves power or not.	Jingdong prefecture level city in Shandong
Fortune Shop	Green Intelligent Power Saver	On Trial	Jingdong prefecture level city in Shandong
Fortune Shop	Green Intelligent Power Saver	Use it and see how it goes.	Jingdong prefecture level city in Shandong
Fortune Shop	Green Intelligent Power Saver	Quality is very good, no odor, fine workmanship, grain color are very like, satisfied!	Jingdong prefecture level city in Shandong
Fortune Shop	Green Intelligent Power Saver	I'll give you a good review. I don't know if it'll work.	Jingdong prefecture level city in Shandong
Fortune Shop	Green Intelligent Power Saver	The logistics is very fast and the package is intact. Good use of affordable. The effect is very good, is genuine	Jingdong prefecture level city in Shandong
Fortune Shop	Green Intelligent Power Saver	Nothing happens when you plug it in.	Jingdong prefecture level city in Shandong
Fortune Shop	Green Intelligent Power Saver	I don't know if it's as good as it sounds.	Jingdong prefecture level city in Shandong
Fortune Shop	Green Intelligent Power Saver	The quality of the stuff is very good, exactly the same as the seller's description, very satisfied!	Jingdong prefecture level city in Shandong
Fortune Shop	Green Intelligent Power Saver	The saver works great, it really works, it's affordable and worth buying.	Jingdong prefecture level city in Shandong
Fortune Shop	Green Intelligent Power Saver	Just used it for a couple days. I don't know yet.	Jingdong prefecture level city in Shandong
Fortune Shop	Green Intelligent Power Saver	The quality is quite good, cheaper than the physical store, good, next time will come again!	Jingdong prefecture level city in Shandong
Fortune Shop	Green Intelligent Power Saver	Good quality, cheap and affordable worth recommending to buy!	Jingdong prefecture level city in Shandong
Fortune Shop	Green Intelligent Power Saver	Very good quality, well made, works well! Satisfied	Jingdong prefecture level city in Shandong
Fortune Shop	Green Intelligent Power Saver	The logistics is very fast quality is very good very pleasant a shopping	Jingdong prefecture level city in Shandong
Fortune Shop	Green Intelligent Power Saver	Use it and then come back to evaluate!	Jingdong prefecture level city in Shandong
Fortune Shop	Green Intelligent Power Saver	# It's no use lying at all #	Jingdong prefecture level city in Shandong
Fortune Shop	Green Intelligent Power Saver	I can't get a voltage drop.	Jingdong prefecture level city in Shandong

Fortune Shop	Green Intelligent Power Saver	Evaluate after use	Jingdong prefecture level city in Shandong
Fortune Shop	Green Intelligent Power Saver	Still on trial.	Jingdong prefecture level city in Shandong
Fortune Shop	Green Intelligent Power Saver	ineffective	Jingdong prefecture level city in Shandong
Fortune Shop	Green Intelligent Power Saver	Satisfaction Shopping	Jingdong prefecture level city in Shandong
Fortune Shop	Green Intelligent Power Saver	Lies.	Jingdong prefecture level city in Shandong
Fortune Shop	Green Intelligent Power Saver	It's a real power saver.	Jingdong prefecture level city in Shandong
Fortune Shop	Green Intelligent Power Saver	in use	Jingdong prefecture level city in Shandong
Fortune Shop	Green Intelligent Power Saver	I don't know yet.	Jingdong prefecture level city in Shandong
Fortune Shop	Green Intelligent Power Saver	not care about anything	Jingdong prefecture level city in Shandong
Fortune Shop	Green Intelligent Power Saver	It doesn't seem to have much effect	Jingdong prefecture level city in Shandong
Fortune Shop	Green Intelligent Power Saver	Let the merchant see for himself, does the plug positive and negative terminals look like this?	Jingdong prefecture level city in Shandong
Fortune Shop	Green Intelligent Power Saver	It doesn't work well, the electricity bill is less before using it, but more after using it, contacting customer service, the service is still not good, and returning it is slow	Jingdong prefecture level city in Shandong
Fortune Shop	Green Intelligent Power Saver	With the advertisement product does not match, above the effective negative, power saving rate of two rows of words it, the product looks very thin, with 3 days to see the meter number is still with the previous no difference, which is obviously fraudulent behavior!	Jingdong prefecture level city in Shandong
Fortune Shop	Green Intelligent Power Saver	Doesn't save any power at all. Not much use.	Jingdong prefecture level city in Shandong
Fortune Shop	Green Intelligent Power Saver	The plugs are all loose, they're all the same length and they don't work at all	Jingdong prefecture level city in Shandong
Fortune Shop	Green Intelligent Power Saver	It's bad 👎 it's not effective at all, you'd be crazy to sell this garbage on the internet.	Jingdong prefecture level city in Shandong
Fortune Shop	Green Intelligent Power Saver	It doesn't save any power at all, so I hope the old timers don't fall for this scam again	Jingdong prefecture level city in Shandong
Fortune Shop	Green Intelligent Power Saver	I've been using it for over a month now and haven't noticed any power savings.	Jingdong prefecture level city in Shandong
Fortune Shop	Green Intelligent Power Saver	It doesn't work at all. The electricity bill is higher when you use it.	Jingdong prefecture level city in Shandong
Fortune Shop	Green Intelligent Power Saver	It doesn't save any power, it's still as bad as ever.	Jingdong prefecture level city in Shandong
Fortune Shop	Green Intelligent Power Saver	Last night before leaving work to look at the table, and then plugged into the node device, this morning and then look at the table, than the original save a few degrees of electricity, very good to have a power-saving effect, easy to use, satisfied with the shopping praise.	Jingdong prefecture level city in Shandong
Fortune Shop	Green Intelligent Power Saver	The product is real and effective, the electricity bill is reduced, really good, thanks to the merchants have so many good products, the product is real and effective, the electricity bill is reduced, want to save electricity friends quickly buy it, early use early to save money.	Jingdong prefecture level city in Shandong
Fortune Shop	Green Intelligent Power Saver	Saver saves time and money, baby appearance is beautiful, the quality of goods is good, and the same as the business description, good service attitude, packaging is complete, logistics is very fast, praise to	Jingdong prefecture level city in Shandong
Fortune Shop	Green Intelligent Power Saver	The effect is good, summer, home electricity is relatively large, buy and use a few days, feel the effect is very good, originally ten dollars a day electricity bill, now eight dollars a day, quite good	Jingdong prefecture level city in Shandong
Fortune Shop	Green Intelligent Power Saver	Indeed can save electricity and money, the first time I know this or from a friend's home to see, but also do not believe it, and now they use it to know that there is this good thing.	Jingdong prefecture level city in Shandong
Fortune Shop	Green Intelligent Power Saver	I have received the Smart Power Saver. Perfectly packaged. Fast delivery. Boss customer service attitude is also very good. It's worth buying. I plugged it in and tried it and it really saves more power than the original. I'll buy it again next time.	Jingdong prefecture level city in Shandong
Fortune Shop	Green Intelligent Power Saver	It's nice to save a dozen bucks a night on two air conditioners that used to take 30 or 40 degrees a night, but now it's only 10 or so degrees a night	Jingdong prefecture level city in Shandong
Fortune Shop	Green Intelligent Power Saver	Product no manufacturer's name no product qualification certificate, try two days to see the effect of	Jingdong prefecture level city in Shandong
Fortune Shop	Green Intelligent Power Saver	I've been using it for over a month and have saved over $80, so I've repurchased a few more back to save together	Jingdong prefecture level city in Shandong
Fortune Shop	Green Intelligent Power Saver	I haven't used it yet and will comment when I do, but it's well made.	Jingdong prefecture level city in Shandong
Fortune Shop	Green Intelligent Power Saver	It's quite energy efficient. It's really a boon for the home.	Jingdong prefecture level city in Shandong
Fortune Shop	Green Intelligent Power Saver	Energy-saving socket received a month, after using it for a month proved that it is indeed energy-saving, electricity bill saving, will buy it again, thank you!	Jingdong prefecture level city in Shandong
Fortune Shop	Green Intelligent Power Saver	There was a similar product many years ago. Now technology has evolved, buy one to try, according to the instructions in the box, it takes one or two dozen days to show results. Check your electric bill and see what happens.	Jingdong prefecture level city in Shandong
Fortune Shop	Green Intelligent Power Saver	Jingdong logistics is really fast, the same day the order arrived on the same day, more convenient than the supermarket, and a wide range of choices, the	Jingdong prefecture level city in Shandong
Fortune Shop	Green Intelligent Power Saver	I've been using this for a month or so and I can't see any results, it really is a scam as they say.	Jingdong prefecture level city in Shandong
Fortune Shop	Green Intelligent Power Saver	Very good use, a month can save a few degrees of power loss of electricity, introduced to the relatives to buy back to try.	Jingdong prefecture level city in Shandong
Fortune Shop	Green Intelligent Power Saver	It's really powerful! With this air conditioner can be turned on at any time without the pressure of paying the electricity bill! It's good for the people!	Jingdong prefecture level city in Shandong
Fortune Shop	Green Intelligent Power Saver	This business is a big ** products are not qualified, with a month of electricity more than one hundred percent of thirty, the purchase agreed to return the goods but do not receive a refund after receiving the goods, looking for the official side does not help, the Jingdong is a big **, I have never shopped in	Jingdong prefecture level city in Shandong
Fortune Shop	Green Intelligent Power Saver	It's good. I tried it. It saves power.	Jingdong prefecture level city in Shandong
Fortune Shop	Green Intelligent Power Saver	Nothing**, it seems like there's more	Jingdong prefecture level city in Shandong
Fortune Shop	Green Intelligent Power Saver	Power saving device received, well packaged, good quality, fast logistics	Jingdong prefecture level city in Shandong
Fortune Shop	Green Intelligent Power Saver	The service is okay, is that when I bought five, the desirable pieces are three, and then wait a few days and send me two replacement, I do not know if it is a mistake of the shipper, if I was not in the courier there to see, go home to say no, the	Jingdong prefecture level city in Shandong
Fortune Shop	Green Intelligent Power Saver	This thing will only cost me more if I use it, but before I used it, it was less than a ~ electricity a month, and after I used it, it was more than two hundred kWh a month.	Jingdong prefecture level city in Shandong
Fortune Shop	Green Intelligent Power Saver	I just used it. I don't know how it is.	Jingdong prefecture level city in Shandong
Fortune Shop	Green Intelligent Power Saver	I've received the product and I'm using it, so I'll see how it goes in a month!	Jingdong prefecture level city in Shandong
Fortune Shop	Green Intelligent Power Saver	The goods have just received the effect do not know how, please look forward to the results.	Jingdong prefecture level city in Shandong
Fortune Shop	Green Intelligent Power Saver	Power saving device, the material is very good practical and durable, the size is suitable, packaging is very good, the price is cheap, logistics is very fast!	Jingdong prefecture level city in Shandong
Fortune Shop	Green Intelligent Power Saver	Very nice, will comment in detail after using it for a while.	Jingdong prefecture level city in Shandong

Fortune Shop	Green Intelligent Power Saver	Good stuff to trust and support	Jingdong prefecture level city in Shandong
Fortune Shop	Green Intelligent Power Saver	I'll comment after I use it, doubt if it really saves power no	Jingdong prefecture level city in Shandong
Fortune Shop	Green Intelligent Power Saver	It's good quality and works well!	Jingdong prefecture level city in Shandong
Fortune Shop	Green Intelligent Power Saver	hahaha just right hahahahahaha hiccup hahahaha	Jingdong prefecture level city in Shandong
Fortune Shop	Green Intelligent Power Saver	The real thing matches the picture and looks great!	Jingdong prefecture level city in Shandong
Fortune Shop	Green Intelligent Power Saver	Plugged it in and we'll see when we pay the bills at the end of the year!	Jingdong prefecture level city in Shandong
Fortune Shop	Green Intelligent Power Saver	False advertising. It doesn't work.	Jingdong prefecture level city in Shandong
Fortune Shop	Green Intelligent Power Saver	Goods received, good effect	Jingdong prefecture level city in Shandong
Fortune Shop	Green Intelligent Power Saver	It looks okay, we'll see if it saves electricity when the time comes	Jingdong prefecture level city in Shandong
Fortune Shop	Green Intelligent Power Saver	Logistics is very fast quality is very good, the price is very affordable, accessories are also complete, installed, satisfied with good use	Jingdong prefecture level city in Shandong
Fortune Shop	Green Intelligent Power Saver	I've received the power saver. I'm looking forward to the savings.	Jingdong prefecture level city in Shandong
Fortune Shop	Green Intelligent Power Saver	Received, have not yet used, after use and then evaluate	Jingdong prefecture level city in Shandong
Fortune Shop	Green Intelligent Power Saver	I received the item, but I don't know if it saves power or not.	Jingdong prefecture level city in Shandong
Fortune Shop	Green Intelligent Power Saver	On Trial	Jingdong prefecture level city in Shandong
Fortune Shop	Green Intelligent Power Saver	Use it and see how it goes.	Jingdong prefecture level city in Shandong
Fortune Shop	Green Intelligent Power Saver	Quality is very good, no odor, fine workmanship, grain color are very like, satisfied!	Jingdong prefecture level city in Shandong
Fortune Shop	Green Intelligent Power Saver	I'll give you a good review. I don't know if it'll work.	Jingdong prefecture level city in Shandong
Fortune Shop	Green Intelligent Power Saver	The logistics is very fast and the package is intact. Good use of affordable. The effect is very good, is genuine	Jingdong prefecture level city in Shandong
Fortune Shop	Green Intelligent Power Saver	Nothing happens when you plug it in.	Jingdong prefecture level city in Shandong
Fortune Shop	Green Intelligent Power Saver	I don't know if it's as good as it sounds.	Jingdong prefecture level city in Shandong
Fortune Shop	Green Intelligent Power Saver	The quality of the stuff is very good, exactly the same as the seller's description, very satisfied!	Jingdong prefecture level city in Shandong
Fortune Shop	Green Intelligent Power Saver	The saver works great, it really works, it's affordable and worth buying.	Jingdong prefecture level city in Shandong
Fortune Shop	Green Intelligent Power Saver	Just used it for a couple days. I don't know yet.	Jingdong prefecture level city in Shandong
Fortune Shop	Green Intelligent Power Saver	The quality is quite good, cheaper than the physical store, good, next time will come again!	Jingdong prefecture level city in Shandong
Fortune Shop	Green Intelligent Power Saver	Good quality, cheap and affordable worth recommending to buy!	Jingdong prefecture level city in Shandong
Fortune Shop	Green Intelligent Power Saver	Very good quality, well made, works well! Satisfied	Jingdong prefecture level city in Shandong
Fortune Shop	Green Intelligent Power Saver	The logistics is very fast quality is very good very pleasant a shopping	Jingdong prefecture level city in Shandong
Fortune Shop	Green Intelligent Power Saver	Use it and then come back to evaluate!	Jingdong prefecture level city in Shandong
Fortune Shop	Green Intelligent Power Saver	# It's no use lying at all #	Jingdong prefecture level city in Shandong
Fortune Shop	Green Intelligent Power Saver	I can't get a voltage drop.	Jingdong prefecture level city in Shandong
Fortune Shop	Green Intelligent Power Saver	Evaluate after use	Jingdong prefecture level city in Shandong
Fortune Shop	Green Intelligent Power Saver	Still on trial.	Jingdong prefecture level city in Shandong
Fortune Shop	Green Intelligent Power Saver	ineffective	Jingdong prefecture level city in Shandong
Fortune Shop	Green Intelligent Power Saver	Satisfaction Shopping	Jingdong prefecture level city in Shandong
Fortune Shop	Green Intelligent Power Saver	Lies.	Jingdong prefecture level city in Shandong
Fortune Shop	Green Intelligent Power Saver	It's a real power saver.	Jingdong prefecture level city in Shandong
Fortune Shop	Green Intelligent Power Saver	in use	Jingdong prefecture level city in Shandong
Fortune Shop	Green Intelligent Power Saver	I don't know yet.	Jingdong prefecture level city in Shandong
Fortune Shop	Green Intelligent Power Saver	not care about anything	Jingdong prefecture level city in Shandong
Fortune Shop	Green Intelligent Power Saver	It doesn't seem to have much effect	Jingdong prefecture level city in Shandong
Fortune Shop	Green Intelligent Power Saver	Let the merchant see for himself, does the plug positive and negative terminals look like this?	Jingdong prefecture level city in Shandong
Fortune Shop	Green Intelligent Power Saver	It doesn't work well, the electricity bill is less before using it, but more after using it, contacting customer service, the service is still not good, and returning it is slow	Jingdong prefecture level city in Shandong
Fortune Shop	Green Intelligent Power Saver	With the advertisement product does not match, above the effective negative, power saving rate of two rows of words it, the product looks very thin, with 3 days to see the meter number is still with the previous no difference, which is obviously fraudulent behavior!	Jingdong prefecture level city in Shandong
Fortune Shop	Green Intelligent Power Saver	Doesn't save any power at all. Not much use.	Jingdong prefecture level city in Shandong
Fortune Shop	Green Intelligent Power Saver	The plugs are all loose, they're all the same length and they don't work at all	Jingdong prefecture level city in Shandong
Fortune Shop	Green Intelligent Power Saver	It's bad 👎 it's not effective at all, you'd be crazy to sell this garbage on the internet.	Jingdong prefecture level city in Shandong
Fortune Shop	Green Intelligent Power Saver	It doesn't save any power at all, so I hope the old timers don't fall for this scam again	Jingdong prefecture level city in Shandong
Fortune Shop	Green Intelligent Power Saver	I've been using it for over a month now and haven't noticed any power savings.	Jingdong prefecture level city in Shandong
Fortune Shop	Green Intelligent Power Saver	It doesn't work at all. The electricity bill is higher when you use it.	Jingdong prefecture level city in Shandong
Fortune Shop	Green Intelligent Power Saver	It doesn't save any power, it's still as bad as ever.	Jingdong prefecture level city in Shandong

Fortune Shop	White Intelligent Power Saver	Has arrived, usually reduce energy consumption is limited, but can save a little is a little, but also to protect household appliances, stabilize the current voltage, the main plug can be used. Winter home two air conditioning a refrigerator, a day down the electricity bill is almost a hundred dollars, with this after an average of two dozen dollars a day to spend less money to enjoy more, it's really very good!	Jingdong prefecture level city in Shandong
Fortune Shop	White Intelligent Power Saver	Goods have been received, the quality is very good, logistics is very fast, very satisfied with a shopping, thanks to the seller, praise!	Jingdong prefecture level city in Shandong
Fortune Shop	White Intelligent Power Saver	Goods received, feel very formal things, this should be very useful. Home electricity bill a month more than a hundred. Should be able to save a lot. Good value for the money packaging is not broken. Satisfied	Jingdong prefecture level city in Shandong
Fortune Shop	White Intelligent Power Saver	Second time I've bought from myself! Pro-test useful, if you find out early to save a lot of money ah! Help the country to promote energy saving, to help their own home to save electricity, so that energy saving into thousands of households, this store did! Good products are willing to share to benefit	Jingdong prefecture level city in Shandong
Fortune Shop	White Intelligent Power Saver	Goods received, feel very formal things, this should be very useful. Home electricity bill a month more than a hundred. Should be able to save a lot. Good value for the money packaging is not broken. Satisfied	Jingdong prefecture level city in Shandong
Fortune Shop	White Intelligent Power Saver	Product received, immediately try, see the meter speed has obvious warm slow. Work normally, the appearance is quite exquisite, fine workmanship. There should be a certain effect, there is a need to rest assured that you can buy.	Jingdong prefecture level city in Shandong
Fortune Shop	White Intelligent Power Saver	It did save over a hundred kWh after using it than before. It is true that you don't know anything until you try it. Personally, I am very satisfied.	Jingdong prefecture level city in Shandong
Fortune Shop	White Intelligent Power Saver	Pipe play, really, about 30% one 50% with a three-horsepower air conditioner trying to	Jingdong prefecture level city in Shandong
Fortune Shop	White Intelligent Power Saver	Actually can really save electricity, only used half a month to save more than 50, recommended to the neighbors to use together!	Jingdong prefecture level city in Shandong
Fortune Shop	White Intelligent Power Saver	Save what you can. Isn't it more cost effective to buy one of these than to pay the electric bill?	Jingdong prefecture level city in Shandong
Fortune Shop	White Intelligent Power Saver	Received, a use do not know how, use a month to see the effect is good, the effect is good then recommended, not good then I just do not recommend.	Jingdong prefecture level city in Shandong
Fortune Shop	White Intelligent Power Saver	Just received the goods, is a voltage regulator, next month to evaluate whether it is really power saving	Jingdong prefecture level city in Shandong
Fortune Shop	White Intelligent Power Saver	Very good, used a few days obviously feel that the electricity bill is much less, wait a month to see how much can be saved!	Jingdong prefecture level city in Shandong
Fortune Shop	White Intelligent Power Saver	Received the stuff used for more than a month without a little use, it feels like it uses more electricity than before	Jingdong prefecture level city in Shandong
Fortune Shop	White Intelligent Power Saver	The quality is very good, completely exceeded the expectations, the delivery speed is very fast, the packaging is very careful and strict, very satisfied with a shopping	Jingdong prefecture level city in Shandong
Fortune Shop	White Intelligent Power Saver	Fake, doesn't work at all. Don't even sell it.	Jingdong prefecture level city in Shandong
Fortune Shop	White Intelligent Power Saver	This item doesn't seem to have much effect?	Jingdong prefecture level city in Shandong
Fortune Shop	White Intelligent Power Saver	This production is a bit fake, just look at the focus on Ming	Jingdong prefecture level city in Shandong
Fortune Shop	White Intelligent Power Saver	The logistics is fast the service attitude is very good.	Jingdong prefecture level city in Shandong
Fortune Shop	White Intelligent Power Saver	The meter is good quality, sturdy and durable. It is accurate. Easy to use. Saves a lot of things.	Jingdong prefecture level city in Shandong
Fortune Shop	White Intelligent Power Saver	Just received the goods, don't know how it is yet	Jingdong prefecture level city in Shandong
Fortune Shop	White Intelligent Power Saver	Energy saver received, logistics is very fast, the quality is very good, the appearance is also very beautiful power saving is the most important.	Jingdong prefecture level city in Shandong
Fortune Shop	White Intelligent Power Saver	It's good to talk about it, it's not practical to use it.	Jingdong prefecture level city in Shandong
Fortune Shop	White Intelligent Power Saver	I don't know if it's good for now, I'll comment after using it	Jingdong prefecture level city in Shandong
Fortune Shop	White Intelligent Power Saver	I've been using it for two or three days. It's not energy efficient.	Jingdong prefecture level city in Shandong
Fortune Shop	White Intelligent Power Saver	Packaging is intact, logistics is also fast, cost-effective, inexpensive, very satisfied with a purchase	Jingdong prefecture level city in Shandong
Fortune Shop	White Intelligent Power Saver	In use to be observed!	Jingdong prefecture level city in Shandong
Fortune Shop	White Intelligent Power Saver	Use it for a while at a flat rate	Jingdong prefecture level city in Shandong
Fortune Shop	White Intelligent Power Saver	Very trustworthy brand, quality is guaranteed, the price is still affordable, quite satisfied!	Jingdong prefecture level city in Shandong
Fortune Shop	White Intelligent Power Saver	Great quality, great operation, easy to install, great price!	Jingdong prefecture level city in Shandong
Fortune Shop	White Intelligent Power Saver	I came back and installed it, it really saves electricity, much more than before, the	Jingdong prefecture level city in Shandong
Fortune Shop	White Intelligent Power Saver	Fast logistics, good quality, very good packaging, good customer service	Jingdong prefecture level city in Shandong
Fortune Shop	White Intelligent Power Saver	Very good quality, especially good, effective, satisfactory	Jingdong prefecture level city in Shandong
Fortune Shop	White Intelligent Power Saver	I don't know how well it works.	Jingdong prefecture level city in Shandong
Fortune Shop	White Intelligent Power Saver	Good value for money, recommend it haha	Jingdong prefecture level city in Shandong
Fortune Shop	White Intelligent Power Saver	Good. Good. Very good.	Jingdong prefecture level city in Shandong
Fortune Shop	White Intelligent Power Saver	It's not working.	Jingdong prefecture level city in Shandong
Fortune Shop	White Intelligent Power Saver	It's not working.	Jingdong prefecture level city in Shandong
Fortune Shop	White Intelligent Power Saver	In use	Jingdong prefecture level city in Shandong
Fortune Shop	White Intelligent Power Saver	I'll try it later.	Jingdong prefecture level city in Shandong
Fortune Shop	White Intelligent Power Saver	It's not much use.	Jingdong prefecture level city in Shandong
Fortune Shop	White Intelligent Power Saver	It doesn't save electricity.	Jingdong prefecture level city in Shandong
Fortune Shop	White Intelligent Power Saver	It's not much use.	Jingdong prefecture level city in Shandong
Fortune Shop	White Intelligent Power Saver	Tried it for a few days and tracked the record, and there is no difference from before.	Jingdong prefecture level city in Shandong
Fortune Shop	White Intelligent Power Saver	It's not very credible, so just do it yourself and turn off the lights diligently!	Jingdong prefecture level city in Shandong
Fortune Shop	White Intelligent Power Saver	Too bad, no power saving at all, scam people don't fall for it!	Jingdong prefecture level city in Shandong
Fortune Shop	White Intelligent Power Saver	It's not working at all.	Jingdong prefecture level city in Shandong
Fortune Shop	White Intelligent Power Saver	general	Jingdong prefecture level city in Shandong
Fortune Shop	White Intelligent Power Saver	(of an unmarried couple) be close	Jingdong prefecture level city in Shandong

Fortune Shop	White Intelligent Power Saver	of no use	Jingdong prefecture level city in Shandong
Fortune Shop	White Intelligent Power Saver	Actually can really save electricity, only used half a month to save more than 50, recommended to the neighbors to use together!	Jingdong prefecture level city in Shandong
Fortune Shop	White Intelligent Power Saver	Save what you can. Isn't it more cost effective to buy one of these than to pay the electric bill?	Jingdong prefecture level city in Shandong
Fortune Shop	White Intelligent Power Saver	Received, a use do not know how, use a month to see the effect is good, the effect is good then recommended, not good then I just do not recommend.	Jingdong prefecture level city in Shandong
Fortune Shop	White Intelligent Power Saver	Just received the goods, is a voltage regulator, next month to evaluate whether it is really power saving	Jingdong prefecture level city in Shandong
Fortune Shop	White Intelligent Power Saver	Very good, used a few days obviously feel that the electricity bill is much less, wait a month to see how much can be saved!	Jingdong prefecture level city in Shandong
Fortune Shop	White Intelligent Power Saver	Received the stuff used for more than a month without a little use, it feels like it uses more electricity than before	Jingdong prefecture level city in Shandong
Fortune Shop	White Intelligent Power Saver	The quality is very good, completely exceeded the expectations, the delivery speed is very fast, the packaging is very careful and strict, very satisfied with a shopping	Jingdong prefecture level city in Shandong
Fortune Shop	White Intelligent Power Saver	Fake, doesn't work at all. Don't even sell it.	Jingdong prefecture level city in Shandong
Fortune Shop	White Intelligent Power Saver	This item doesn't seem to have much effect?	Jingdong prefecture level city in Shandong
Fortune Shop	White Intelligent Power Saver	This production is a bit fake, just look at the focus on Ming	Jingdong prefecture level city in Shandong
Fortune Shop	White Intelligent Power Saver	The logistics is fast the service attitude is very good.	Jingdong prefecture level city in Shandong
Fortune Shop	White Intelligent Power Saver	The meter is good quality, sturdy and durable. It is accurate. Easy to use. Saves a lot of things.	Jingdong prefecture level city in Shandong
Fortune Shop	White Intelligent Power Saver	Just received the goods, don't know how it is yet	Jingdong prefecture level city in Shandong
Fortune Shop	White Intelligent Power Saver	Energy saver received, logistics is very fast, the quality is very good, the appearance is also very beautiful power saving is the most important.	Jingdong prefecture level city in Shandong
Fortune Shop	White Intelligent Power Saver	It's good to talk about it, it's not practical to use it.	Jingdong prefecture level city in Shandong
Fortune Shop	White Intelligent Power Saver	I don't know if it's good for now, I'll comment after using it	Jingdong prefecture level city in Shandong
Fortune Shop	White Intelligent Power Saver	I've been using it for two or three days. It's not energy efficient.	Jingdong prefecture level city in Shandong
Fortune Shop	White Intelligent Power Saver	Packaging is intact, logistics is also fast, cost-effective, inexpensive, very satisfied with a purchase	Jingdong prefecture level city in Shandong
Fortune Shop	White Intelligent Power Saver	In use to be observed!	Jingdong prefecture level city in Shandong
Fortune Shop	White Intelligent Power Saver	Use it for a while at a flat rate	Jingdong prefecture level city in Shandong
Fortune Shop	White Intelligent Power Saver	Very trustworthy brand, quality is guaranteed, the price is still affordable, quite satisfied!	Jingdong prefecture level city in Shandong
Fortune Shop	White Intelligent Power Saver	Great quality, great operation, easy to install, great price!	Jingdong prefecture level city in Shandong
Fortune Shop	White Intelligent Power Saver	I came back and installed it, it really saves electricity, much more than before, the	Jingdong prefecture level city in Shandong
Fortune Shop	White Intelligent Power Saver	Fast logistics, good quality, very good packaging, good customer service	Jingdong prefecture level city in Shandong
Fortune Shop	White Intelligent Power Saver	Very good quality, especially good, effective, satisfactory	Jingdong prefecture level city in Shandong
Fortune Shop	White Intelligent Power Saver	I don't know how well it works.	Jingdong prefecture level city in Shandong
Fortune Shop	White Intelligent Power Saver	Good value for money, recommend it haha	Jingdong prefecture level city in Shandong
Fortune Shop	White Intelligent Power Saver	Good. Good. Very good.	Jingdong prefecture level city in Shandong
Fortune Shop	White Intelligent Power Saver	It's not working.	Jingdong prefecture level city in Shandong
Fortune Shop	White Intelligent Power Saver	It's not working.	Jingdong prefecture level city in Shandong
Fortune Shop	White Intelligent Power Saver	In use	Jingdong prefecture level city in Shandong
Fortune Shop	White Intelligent Power Saver	I'll try it later.	Jingdong prefecture level city in Shandong
Fortune Shop	White Intelligent Power Saver	It's not much use.	Jingdong prefecture level city in Shandong
Fortune Shop	White Intelligent Power Saver	It doesn't save electricity.	Jingdong prefecture level city in Shandong
Fortune Shop	White Intelligent Power Saver	It's not much use.	Jingdong prefecture level city in Shandong
Fortune Shop	White Intelligent Power Saver	Tried it for a few days and tracked the record, and there is no difference from before.	Jingdong prefecture level city in Shandong
Fortune Shop	White Intelligent Power Saver	It's not very credible, so just do it yourself and turn off the lights diligently!	Jingdong prefecture level city in Shandong
Fortune Shop	White Intelligent Power Saver	Too bad, no power saving at all, scam people don't fall for it!	Jingdong prefecture level city in Shandong
Fortune Shop	White Intelligent Power Saver	It's not working at all.	Jingdong prefecture level city in Shandong
Fortune Shop	White Intelligent Power Saver	general	Jingdong prefecture level city in Shandong
Fortune Shop	White Intelligent Power Saver	(of an unmarried couple) be close	Jingdong prefecture level city in Shandong
Fortune Shop	White Intelligent Power Saver	of no use	Jingdong prefecture level city in Shandong
Fortune Shop	White Intelligent Power Saver	Actually can really save electricity, only used half a month to save more than 50, recommended to the neighbors to use together!	Jingdong prefecture level city in Shandong
Fortune Shop	White Intelligent Power Saver	Save what you can. Isn't it more cost effective to buy one of these than to pay the electric bill?	Jingdong prefecture level city in Shandong
Fortune Shop	White Intelligent Power Saver	Received, a use do not know how, use a month to see the effect is good, the effect is good then recommended, not good then I just do not recommend.	Jingdong prefecture level city in Shandong
Fortune Shop	White Intelligent Power Saver	Just received the goods, is a voltage regulator, next month to evaluate whether it is really power saving	Jingdong prefecture level city in Shandong
Fortune Shop	White Intelligent Power Saver	Very good, used a few days obviously feel that the electricity bill is much less, wait a month to see how much can be saved!	Jingdong prefecture level city in Shandong
Fortune Shop	White Intelligent Power Saver	Received the stuff used for more than a month without a little use, it feels like it uses more electricity than before	Jingdong prefecture level city in Shandong
Fortune Shop	White Intelligent Power Saver	The quality is very good, completely exceeded the expectations, the delivery speed is very fast, the packaging is very careful and strict, very satisfied with a shopping	Jingdong prefecture level city in Shandong
Fortune Shop	White Intelligent Power Saver	Fake, doesn't work at all. Don't even sell it.	Jingdong prefecture level city in Shandong

Fortune Shop	White Intelligent Power Saver	This item doesn't seem to have much effect?	Jingdong prefecture level city in Shandong
Fortune Shop	White Intelligent Power Saver	This production is a bit fake, just look at the focus on Ming	Jingdong prefecture level city in Shandong
Fortune Shop	White Intelligent Power Saver	The logistics is fast the service attitude is very good.	Jingdong prefecture level city in Shandong
Fortune Shop	White Intelligent Power Saver	The meter is good quality, sturdy and durable. It is accurate. Easy to use. Saves a lot of things.	Jingdong prefecture level city in Shandong
Fortune Shop	White Intelligent Power Saver	Just received the goods, don't know how it is yet	Jingdong prefecture level city in Shandong
Fortune Shop	White Intelligent Power Saver	Energy saver received, logistics is very fast, the quality is very good, the appearance is also very beautiful power saving is the most important.	Jingdong prefecture level city in Shandong
Fortune Shop	White Intelligent Power Saver	It's good to talk about it, it's not practical to use it.	Jingdong prefecture level city in Shandong
Fortune Shop	White Intelligent Power Saver	I don't know if it's good for now, I'll comment after using it	Jingdong prefecture level city in Shandong
Fortune Shop	White Intelligent Power Saver	I've been using it for two or three days. It's not energy efficient.	Jingdong prefecture level city in Shandong
Fortune Shop	White Intelligent Power Saver	Packaging is intact, logistics is also fast, cost-effective, inexpensive, very satisfied with a purchase	Jingdong prefecture level city in Shandong
Fortune Shop	White Intelligent Power Saver	In use to be observed!	Jingdong prefecture level city in Shandong
Fortune Shop	White Intelligent Power Saver	Use it for a while at a flat rate	Jingdong prefecture level city in Shandong
Fortune Shop	White Intelligent Power Saver	Very trustworthy brand, quality is guaranteed, the price is still affordable, quite satisfied!	Jingdong prefecture level city in Shandong
Fortune Shop	White Intelligent Power Saver	Great quality, great operation, easy to install, great price!	Jingdong prefecture level city in Shandong
Fortune Shop	White Intelligent Power Saver	I came back and installed it, it really saves electricity, much more than before, the	Jingdong prefecture level city in Shandong
Fortune Shop	White Intelligent Power Saver	Fast logistics, good quality, very good packaging, good customer service	Jingdong prefecture level city in Shandong
Fortune Shop	White Intelligent Power Saver	Very good quality, especially good, effective, satisfactory	Jingdong prefecture level city in Shandong
Fortune Shop	White Intelligent Power Saver	I don't know how well it works.	Jingdong prefecture level city in Shandong
Fortune Shop	White Intelligent Power Saver	Good value for money, recommend it haha	Jingdong prefecture level city in Shandong
Fortune Shop	White Intelligent Power Saver	Good. Good. Very good.	Jingdong prefecture level city in Shandong
Fortune Shop	White Intelligent Power Saver	It's not working.	Jingdong prefecture level city in Shandong
Fortune Shop	White Intelligent Power Saver	It's not working.	Jingdong prefecture level city in Shandong
Fortune Shop	White Intelligent Power Saver	In use	Jingdong prefecture level city in Shandong
Fortune Shop	White Intelligent Power Saver	I'll try it later.	Jingdong prefecture level city in Shandong
Fortune Shop	White Intelligent Power Saver	It's not much use.	Jingdong prefecture level city in Shandong
Fortune Shop	White Intelligent Power Saver	It doesn't save electricity.	Jingdong prefecture level city in Shandong
Fortune Shop	White Intelligent Power Saver	It's not much use.	Jingdong prefecture level city in Shandong
Fortune Shop	White Intelligent Power Saver	Tried it for a few days and tracked the record, and there is no difference from before.	Jingdong prefecture level city in Shandong
Fortune Shop	White Intelligent Power Saver	It's not very credible, so just do it yourself and turn off the lights diligently!	Jingdong prefecture level city in Shandong
Fortune Shop	White Intelligent Power Saver	Too bad, no power saving at all, scam people don't fall for it!	Jingdong prefecture level city in Shandong
Fortune Shop	White Intelligent Power Saver	It's not working at all.	Jingdong prefecture level city in Shandong
Fortune Shop	White Intelligent Power Saver	general	Jingdong prefecture level city in Shandong
Fortune Shop	White Intelligent Power Saver	(of an unmarried couple) be close	Jingdong prefecture level city in Shandong
Fortune Shop	White Intelligent Power Saver	of no use	Jingdong prefecture level city in Shandong
Fortune Shop	White Intelligent Power Saver	Actually can really save electricity, only used half a month to save more than 50, recommended to the neighbors to use together!	Jingdong prefecture level city in Shandong
Fortune Shop	White Intelligent Power Saver	Save what you can. Isn't it more cost effective to buy one of these than to pay the electric bill?	Jingdong prefecture level city in Shandong
Fortune Shop	White Intelligent Power Saver	Received, a use do not know how, use a month to see the effect is good, the effect is good then recommended, not good then I just do not recommend.	Jingdong prefecture level city in Shandong
Fortune Shop	White Intelligent Power Saver	Just received the goods, is a voltage regulator, next month to evaluate whether it is really power saving	Jingdong prefecture level city in Shandong
Fortune Shop	White Intelligent Power Saver	Very good, used a few days obviously feel that the electricity bill is much less, wait a month to see how much can be saved!	Jingdong prefecture level city in Shandong
Fortune Shop	White Intelligent Power Saver	Received the stuff used for more than a month without a little use, it feels like it uses more electricity than before	Jingdong prefecture level city in Shandong
Fortune Shop	White Intelligent Power Saver	The quality is very good, completely exceeded the expectations, the delivery speed is very fast, the packaging is very careful and strict, very satisfied with a shopping	Jingdong prefecture level city in Shandong
Fortune Shop	White Intelligent Power Saver	Fake, doesn't work at all. Don't even sell it.	Jingdong prefecture level city in Shandong
Fortune Shop	White Intelligent Power Saver	This item doesn't seem to have much effect?	Jingdong prefecture level city in Shandong
Fortune Shop	White Intelligent Power Saver	This production is a bit fake, just look at the focus on Ming	Jingdong prefecture level city in Shandong
Fortune Shop	White Intelligent Power Saver	The logistics is fast the service attitude is very good.	Jingdong prefecture level city in Shandong
Fortune Shop	White Intelligent Power Saver	The meter is good quality, sturdy and durable. It is accurate. Easy to use. Saves a lot of things.	Jingdong prefecture level city in Shandong
Fortune Shop	White Intelligent Power Saver	Just received the goods, don't know how it is yet	Jingdong prefecture level city in Shandong
Fortune Shop	White Intelligent Power Saver	Energy saver received, logistics is very fast, the quality is very good, the appearance is also very beautiful power saving is the most important.	Jingdong prefecture level city in Shandong
Fortune Shop	White Intelligent Power Saver	It's good to talk about it, it's not practical to use it.	Jingdong prefecture level city in Shandong
Fortune Shop	White Intelligent Power Saver	I don't know if it's good for now, I'll comment after using it	Jingdong prefecture level city in Shandong
Fortune Shop	White Intelligent Power Saver	I've been using it for two or three days. It's not energy efficient.	Jingdong prefecture level city in Shandong

Fortune Shop	White Intelligent Power Saver	Packaging is intact, logistics is also fast, cost-effective, inexpensive, very satisfied with a purchase	Jingdong prefecture level city in Shandong
Fortune Shop	White Intelligent Power Saver	In use to be observed!	Jingdong prefecture level city in Shandong
Fortune Shop	White Intelligent Power Saver	Use it for a while at a flat rate	Jingdong prefecture level city in Shandong
Fortune Shop	White Intelligent Power Saver	Very trustworthy brand, quality is guaranteed, the price is still affordable, quite satisfied!	Jingdong prefecture level city in Shandong
Fortune Shop	White Intelligent Power Saver	Great quality, great operation, easy to install, great price!	Jingdong prefecture level city in Shandong
Fortune Shop	White Intelligent Power Saver	I came back and installed it, it really saves electricity, much more than before, the	Jingdong prefecture level city in Shandong
Fortune Shop	White Intelligent Power Saver	Fast logistics, good quality, very good packaging, good customer service	Jingdong prefecture level city in Shandong
Fortune Shop	White Intelligent Power Saver	Very good quality, especially good, effective, satisfactory	Jingdong prefecture level city in Shandong
Fortune Shop	White Intelligent Power Saver	I don't know how well it works.	Jingdong prefecture level city in Shandong
Fortune Shop	White Intelligent Power Saver	Good value for money, recommend it haha	Jingdong prefecture level city in Shandong
Fortune Shop	White Intelligent Power Saver	Good. Good. Very good.	Jingdong prefecture level city in Shandong
Fortune Shop	White Intelligent Power Saver	It's not working.	Jingdong prefecture level city in Shandong
Fortune Shop	White Intelligent Power Saver	It's not working.	Jingdong prefecture level city in Shandong
Fortune Shop	White Intelligent Power Saver	In use	Jingdong prefecture level city in Shandong
Fortune Shop	White Intelligent Power Saver	It's not much use.	Jingdong prefecture level city in Shandong
Fortune Shop	White Intelligent Power Saver	I'll try it later.	Jingdong prefecture level city in Shandong
Fortune Shop	White Intelligent Power Saver	It doesn't save electricity.	Jingdong prefecture level city in Shandong
Fortune Shop	White Intelligent Power Saver	It's not much use.	Jingdong prefecture level city in Shandong
Fortune Shop	White Intelligent Power Saver	Tried it for a few days and tracked the record, and there is no difference from before.	Jingdong prefecture level city in Shandong
Fortune Shop	White Intelligent Power Saver	It's not very credible, so just do it yourself and turn off the lights diligently!	Jingdong prefecture level city in Shandong
Fortune Shop	White Intelligent Power Saver	Too bad, no power saving at all, scam people don't fall for it!	Jingdong prefecture level city in Shandong
Fortune Shop	White Intelligent Power Saver	It's not working at all.	Jingdong prefecture level city in Shandong
Fortune Shop	White Intelligent Power Saver	(of an unmarried couple) be close	Jingdong prefecture level city in Shandong
Fortune Shop	White Intelligent Power Saver	general	Jingdong prefecture level city in Shandong
Fortune Shop	White Intelligent Power Saver	of no use	Jingdong prefecture level city in Shandong
Fortune Shop	White Intelligent Power Saver	Actually can really save electricity, only used half a month to save more than 50, recommended to the neighbors to use together!	Jingdong prefecture level city in Shandong
Fortune Shop	White Intelligent Power Saver	Save what you can. Isn't it more cost effective to buy one of these than to pay the electric bill?	Jingdong prefecture level city in Shandong
Fortune Shop	White Intelligent Power Saver	Received, a use do not know how, use a month to see the effect is good, the effect is good then recommended, not good then I just do not recommend.	Jingdong prefecture level city in Shandong
Fortune Shop	White Intelligent Power Saver	Just received the goods, is a voltage regulator, next month to evaluate whether it is really power saving	Jingdong prefecture level city in Shandong
Fortune Shop	White Intelligent Power Saver	Very good, used a few days obviously feel that the electricity bill is much less, wait a month to see how much can be saved!	Jingdong prefecture level city in Shandong
Fortune Shop	White Intelligent Power Saver	Received the stuff used for more than a month without a little use, it feels like it uses more electricity than before	Jingdong prefecture level city in Shandong
Fortune Shop	White Intelligent Power Saver	The quality is very good, completely exceeded the expectations, the delivery speed is very fast, the packaging is very careful and strict, very satisfied with a shopping	Jingdong prefecture level city in Shandong
Fortune Shop	White Intelligent Power Saver	Fake, doesn't work at all. Don't even sell it.	Jingdong prefecture level city in Shandong
Fortune Shop	White Intelligent Power Saver	This item doesn't seem to have much effect?	Jingdong prefecture level city in Shandong
Fortune Shop	White Intelligent Power Saver	This production is a bit fake, just look at the focus on Ming	Jingdong prefecture level city in Shandong
Fortune Shop	White Intelligent Power Saver	The logistics is fast the service attitude is very good.	Jingdong prefecture level city in Shandong
Fortune Shop	White Intelligent Power Saver	The meter is good quality, sturdy and durable. It is accurate. Easy to use. Saves a lot of things.	Jingdong prefecture level city in Shandong
Fortune Shop	White Intelligent Power Saver	Just received the goods, don't know how it is yet	Jingdong prefecture level city in Shandong
Fortune Shop	White Intelligent Power Saver	Energy saver received, logistics is very fast, the quality is very good, the appearance is also very beautiful power saving is the most important.	Jingdong prefecture level city in Shandong
Fortune Shop	White Intelligent Power Saver	It's good to talk about it, it's not practical to use it.	Jingdong prefecture level city in Shandong
Fortune Shop	White Intelligent Power Saver	I don't know if it's good for now, I'll comment after using it	Jingdong prefecture level city in Shandong
Fortune Shop	White Intelligent Power Saver	I've been using it for two or three days. It's not energy efficient.	Jingdong prefecture level city in Shandong
Fortune Shop	White Intelligent Power Saver	Packaging is intact, logistics is also fast, cost-effective, inexpensive, very satisfied with a purchase	Jingdong prefecture level city in Shandong
Fortune Shop	White Intelligent Power Saver	In use to be observed!	Jingdong prefecture level city in Shandong
Fortune Shop	White Intelligent Power Saver	Use it for a while at a flat rate	Jingdong prefecture level city in Shandong
Fortune Shop	White Intelligent Power Saver	Very trustworthy brand, quality is guaranteed, the price is still affordable, quite satisfied!	Jingdong prefecture level city in Shandong
Fortune Shop	White Intelligent Power Saver	Great quality, great operation, easy to install, great price!	Jingdong prefecture level city in Shandong
Fortune Shop	White Intelligent Power Saver	I came back and installed it, it really saves electricity, much more than before, the	Jingdong prefecture level city in Shandong
Fortune Shop	White Intelligent Power Saver	Fast logistics, good quality, very good packaging, good customer service	Jingdong prefecture level city in Shandong
Fortune Shop	White Intelligent Power Saver	Very good quality, especially good, effective, satisfactory	Jingdong prefecture level city in Shandong
Fortune Shop	White Intelligent Power Saver	I don't know how well it works.	Jingdong prefecture level city in Shandong

Fortune Shop	White Intelligent Power Saver	Good value for money, recommend it haha	Jingdong prefecture level city in Shandong
Fortune Shop	White Intelligent Power Saver	Good. Good. Very good.	Jingdong prefecture level city in Shandong
Fortune Shop	White Intelligent Power Saver	It's not working.	Jingdong prefecture level city in Shandong
Fortune Shop	White Intelligent Power Saver	It's not working.	Jingdong prefecture level city in Shandong
Fortune Shop	White Intelligent Power Saver	In use	Jingdong prefecture level city in Shandong
Fortune Shop	White Intelligent Power Saver	I'll try it later.	Jingdong prefecture level city in Shandong
Fortune Shop	White Intelligent Power Saver	It's not much use.	Jingdong prefecture level city in Shandong
Fortune Shop	White Intelligent Power Saver	It doesn't save electricity.	Jingdong prefecture level city in Shandong
Fortune Shop	White Intelligent Power Saver	It's not much use.	Jingdong prefecture level city in Shandong
Fortune Shop	White Intelligent Power Saver	Tried it for a few days and tracked the record, and there is no difference from before.	Jingdong prefecture level city in Shandong
Fortune Shop	White Intelligent Power Saver	It's not very credible, so just do it yourself and turn off the lights diligently!	Jingdong prefecture level city in Shandong
Fortune Shop	White Intelligent Power Saver	Too bad, no power saving at all, scam people don't fall for it!	Jingdong prefecture level city in Shandong
Fortune Shop	White Intelligent Power Saver	It's not working at all.	Jingdong prefecture level city in Shandong
Fortune Shop	White Intelligent Power Saver	general	Jingdong prefecture level city in Shandong
Fortune Shop	White Intelligent Power Saver	(of an unmarried couple) be close	Jingdong prefecture level city in Shandong
Fortune Shop	White Intelligent Power Saver	of no use	Jingdong prefecture level city in Shandong
Fortune Shop	White Intelligent Power Saver	Actually can really save electricity, only used half a month to save more than 50, recommended to the neighbors to use together!	Jingdong prefecture level city in Shandong
Fortune Shop	White Intelligent Power Saver	Save what you can. Isn't it more cost effective to buy one of these than to pay the electric bill?	Jingdong prefecture level city in Shandong
Fortune Shop	White Intelligent Power Saver	Received, a use do not know how, use a month to see the effect is good, the effect is good then recommended, not good then I just do not recommend.	Jingdong prefecture level city in Shandong
Fortune Shop	White Intelligent Power Saver	Just received the goods, is a voltage regulator, next month to evaluate whether it is really power saving	Jingdong prefecture level city in Shandong
Fortune Shop	White Intelligent Power Saver	Very good, used a few days obviously feel that the electricity bill is much less, wait a month to see how much can be saved!	Jingdong prefecture level city in Shandong
Fortune Shop	White Intelligent Power Saver	Received the stuff used for more than a month without a little use, it feels like it uses more electricity than before	Jingdong prefecture level city in Shandong
Fortune Shop	White Intelligent Power Saver	The quality is very good, completely exceeded the expectations, the delivery speed is very fast, the packaging is very careful and strict, very satisfied with a shopping	Jingdong prefecture level city in Shandong
Fortune Shop	White Intelligent Power Saver	Fake, doesn't work at all. Don't even sell it.	Jingdong prefecture level city in Shandong
Fortune Shop	White Intelligent Power Saver	This item doesn't seem to have much effect?	Jingdong prefecture level city in Shandong
Fortune Shop	White Intelligent Power Saver	This production is a bit fake, just look at the focus on Ming	Jingdong prefecture level city in Shandong
Fortune Shop	White Intelligent Power Saver	The logistics is fast the service attitude is very good.	Jingdong prefecture level city in Shandong
Fortune Shop	White Intelligent Power Saver	The meter is good quality, sturdy and durable. It is accurate. Easy to use. Saves a lot of things.	Jingdong prefecture level city in Shandong
Fortune Shop	White Intelligent Power Saver	Just received the goods, don't know how it is yet	Jingdong prefecture level city in Shandong
Fortune Shop	White Intelligent Power Saver	Energy saver received, logistics is very fast, the quality is very good, the appearance is also very beautiful power saving is the most important.	Jingdong prefecture level city in Shandong
Fortune Shop	White Intelligent Power Saver	It's good to talk about it, it's not practical to use it.	Jingdong prefecture level city in Shandong
Fortune Shop	White Intelligent Power Saver	I don't know if it's good for now, I'll comment after using it	Jingdong prefecture level city in Shandong
Fortune Shop	White Intelligent Power Saver	I've been using it for two or three days. It's not energy efficient.	Jingdong prefecture level city in Shandong
Fortune Shop	White Intelligent Power Saver	Packaging is intact, logistics is also fast, cost-effective, inexpensive, very satisfied with a purchase	Jingdong prefecture level city in Shandong
Fortune Shop	White Intelligent Power Saver	In use to be observed!	Jingdong prefecture level city in Shandong
Fortune Shop	White Intelligent Power Saver	Use it for a while at a flat rate	Jingdong prefecture level city in Shandong
Fortune Shop	White Intelligent Power Saver	Very trustworthy brand, quality is guaranteed, the price is still affordable, quite satisfied!	Jingdong prefecture level city in Shandong
Fortune Shop	White Intelligent Power Saver	Great quality, great operation, easy to install, great price!	Jingdong prefecture level city in Shandong
Fortune Shop	White Intelligent Power Saver	I came back and installed it, it really saves electricity, much more than before, the	Jingdong prefecture level city in Shandong
Fortune Shop	White Intelligent Power Saver	Fast logistics, good quality, very good packaging, good customer service	Jingdong prefecture level city in Shandong
Fortune Shop	White Intelligent Power Saver	Very good quality, especially good, effective, satisfactory	Jingdong prefecture level city in Shandong
Fortune Shop	White Intelligent Power Saver	I don't know how well it works.	Jingdong prefecture level city in Shandong
Fortune Shop	White Intelligent Power Saver	Good value for money, recommend it haha	Jingdong prefecture level city in Shandong
Fortune Shop	White Intelligent Power Saver	Good. Good. Very good.	Jingdong prefecture level city in Shandong
Fortune Shop	White Intelligent Power Saver	It's not working.	Jingdong prefecture level city in Shandong
Fortune Shop	White Intelligent Power Saver	It's not working.	Jingdong prefecture level city in Shandong
Fortune Shop	White Intelligent Power Saver	In use	Jingdong prefecture level city in Shandong
Fortune Shop	White Intelligent Power Saver	I'll try it later.	Jingdong prefecture level city in Shandong
Fortune Shop	White Intelligent Power Saver	It's not much use.	Jingdong prefecture level city in Shandong
Fortune Shop	White Intelligent Power Saver	It doesn't save electricity.	Jingdong prefecture level city in Shandong
Fortune Shop	White Intelligent Power Saver	It's not much use.	Jingdong prefecture level city in Shandong

Fortune Shop	White Intelligent Power Saver	Tried it for a few days and tracked the record, and there is no difference from before.	Jingdong prefecture level city in Shandong
Fortune Shop	White Intelligent Power Saver	It's not very credible, so just do it yourself and turn off the lights diligently!	Jingdong prefecture level city in Shandong
Fortune Shop	White Intelligent Power Saver	Too bad, no power saving at all, scam people don't fall for it!	Jingdong prefecture level city in Shandong
Fortune Shop	White Intelligent Power Saver	It's not working at all.	Jingdong prefecture level city in Shandong
Fortune Shop	White Intelligent Power Saver	general	Jingdong prefecture level city in Shandong
Fortune Shop	White Intelligent Power Saver	(of an unmarried couple) be close	Jingdong prefecture level city in Shandong
Fortune Shop	White Intelligent Power Saver	of no use	Jingdong prefecture level city in Shandong
Fortune Shop	White Intelligent Power Saver	Actually can really save electricity, only used half a month to save more than 50, recommended to the neighbors to use together!	Jingdong prefecture level city in Shandong
Fortune Shop	White Intelligent Power Saver	Save what you can. Isn't it more cost effective to buy one of these than to pay the electric bill?	Jingdong prefecture level city in Shandong
Fortune Shop	White Intelligent Power Saver	Received, a use do not know how, use a month to see the effect is good, the effect is good then recommended, not good then I just do not recommend.	Jingdong prefecture level city in Shandong
Fortune Shop	White Intelligent Power Saver	Just received the goods, is a voltage regulator, next month to evaluate whether it is really power saving	Jingdong prefecture level city in Shandong
Fortune Shop	White Intelligent Power Saver	Very good, used a few days obviously feel that the electricity bill is much less, wait a month to see how much can be saved!	Jingdong prefecture level city in Shandong
Fortune Shop	White Intelligent Power Saver	Received the stuff used for more than a month without a little use, it feels like it uses more electricity than before	Jingdong prefecture level city in Shandong
Fortune Shop	White Intelligent Power Saver	The quality is very good, completely exceeded the expectations, the delivery speed is very fast, the packaging is very careful and strict, very satisfied with a shopping	Jingdong prefecture level city in Shandong
Fortune Shop	White Intelligent Power Saver	Fake, doesn't work at all. Don't even sell it.	Jingdong prefecture level city in Shandong
Fortune Shop	White Intelligent Power Saver	This item doesn't seem to have much effect?	Jingdong prefecture level city in Shandong
Fortune Shop	White Intelligent Power Saver	This production is a bit fake, just look at the focus on Ming	Jingdong prefecture level city in Shandong
Fortune Shop	White Intelligent Power Saver	The logistics is fast the service attitude is very good.	Jingdong prefecture level city in Shandong
Fortune Shop	White Intelligent Power Saver	The meter is good quality, sturdy and durable. It is accurate. Easy to use. Saves a lot of things.	Jingdong prefecture level city in Shandong
Fortune Shop	White Intelligent Power Saver	Just received the goods, don't know how it is yet	Jingdong prefecture level city in Shandong
Fortune Shop	White Intelligent Power Saver	Energy saver received, logistics is very fast, the quality is very good, the appearance is also very beautiful power saving is the most important.	Jingdong prefecture level city in Shandong
Fortune Shop	White Intelligent Power Saver	It's good to talk about it, it's not practical to use it.	Jingdong prefecture level city in Shandong
Fortune Shop	White Intelligent Power Saver	I don't know if it's good for now, I'll comment after using it	Jingdong prefecture level city in Shandong
Fortune Shop	White Intelligent Power Saver	I've been using it for two or three days. It's not energy efficient.	Jingdong prefecture level city in Shandong
Fortune Shop	White Intelligent Power Saver	Packaging is intact, logistics is also fast, cost-effective, inexpensive, very satisfied with a purchase	Jingdong prefecture level city in Shandong
Fortune Shop	White Intelligent Power Saver	In use to be observed!	Jingdong prefecture level city in Shandong
Fortune Shop	White Intelligent Power Saver	Use it for a while at a flat rate	Jingdong prefecture level city in Shandong
Fortune Shop	White Intelligent Power Saver	Very trustworthy brand, quality is guaranteed, the price is still affordable, quite satisfied!	Jingdong prefecture level city in Shandong
Fortune Shop	White Intelligent Power Saver	Great quality, great operation, easy to install, great price!	Jingdong prefecture level city in Shandong
Fortune Shop	White Intelligent Power Saver	I came back and installed it, it really saves electricity, much more than before, the	Jingdong prefecture level city in Shandong
Fortune Shop	White Intelligent Power Saver	Fast logistics, good quality, very good packaging, good customer service	Jingdong prefecture level city in Shandong
Fortune Shop	White Intelligent Power Saver	Very good quality, especially good, effective, satisfactory	Jingdong prefecture level city in Shandong
Fortune Shop	White Intelligent Power Saver	I don't know how well it works.	Jingdong prefecture level city in Shandong
Fortune Shop	White Intelligent Power Saver	Good value for money, recommend it haha	Jingdong prefecture level city in Shandong
Fortune Shop	White Intelligent Power Saver	Good. Good. Very good.	Jingdong prefecture level city in Shandong
Fortune Shop	White Intelligent Power Saver	It's not working.	Jingdong prefecture level city in Shandong
Fortune Shop	White Intelligent Power Saver	It's not working.	Jingdong prefecture level city in Shandong
Fortune Shop	White Intelligent Power Saver	In use	Jingdong prefecture level city in Shandong
Fortune Shop	White Intelligent Power Saver	I'll try it later.	Jingdong prefecture level city in Shandong
Fortune Shop	White Intelligent Power Saver	It's not much use.	Jingdong prefecture level city in Shandong
Fortune Shop	White Intelligent Power Saver	It's not much use.	Jingdong prefecture level city in Shandong
Fortune Shop	White Intelligent Power Saver	It doesn't save electricity.	Jingdong prefecture level city in Shandong
Fortune Shop	White Intelligent Power Saver	Tried it for a few days and tracked the record, and there is no difference from before.	Jingdong prefecture level city in Shandong
Fortune Shop	White Intelligent Power Saver	It's not very credible, so just do it yourself and turn off the lights diligently!	Jingdong prefecture level city in Shandong
Fortune Shop	White Intelligent Power Saver	Too bad, no power saving at all, scam people don't fall for it!	Jingdong prefecture level city in Shandong
Fortune Shop	White Intelligent Power Saver	general	Jingdong prefecture level city in Shandong
Fortune Shop	White Intelligent Power Saver	It's not working at all.	Jingdong prefecture level city in Shandong
Fortune Shop	White Intelligent Power Saver	(of an unmarried couple) be close	Jingdong prefecture level city in Shandong
Fortune Shop	White Intelligent Power Saver	of no use	Jingdong prefecture level city in Shandong
Fortune Shop	White Intelligent Power Saver	Actually can really save electricity, only used half a month to save more than 50, recommended to the neighbors to use together!	Jingdong prefecture level city in Shandong
Fortune Shop	White Intelligent Power Saver	Save what you can. Isn't it more cost effective to buy one of these than to pay the electric bill?	Jingdong prefecture level city in Shandong

Fortune Shop	White Intelligent Power Saver	Received, a use do not know how, use a month to see the effect is good, the effect is good then recommended, not good then I just do not recommend.	Jingdong prefecture level city in Shandong
Fortune Shop	White Intelligent Power Saver	Just received the goods, is a voltage regulator, next month to evaluate whether it is really power saving	Jingdong prefecture level city in Shandong
Fortune Shop	White Intelligent Power Saver	Very good, used a few days obviously feel that the electricity bill is much less, wait a month to see how much can be saved!	Jingdong prefecture level city in Shandong
Fortune Shop	White Intelligent Power Saver	Received the stuff used for more than a month without a little use, it feels like it uses more electricity than before	Jingdong prefecture level city in Shandong
Fortune Shop	White Intelligent Power Saver	The quality is very good, completely exceeded the expectations, the delivery speed is very fast, the packaging is very careful and strict, very satisfied with a shopping	Jingdong prefecture level city in Shandong
Fortune Shop	White Intelligent Power Saver	Fake, doesn't work at all. Don't even sell it.	Jingdong prefecture level city in Shandong
Fortune Shop	White Intelligent Power Saver	This item doesn't seem to have much effect?	Jingdong prefecture level city in Shandong
Fortune Shop	White Intelligent Power Saver	This production is a bit fake, just look at the focus on Ming	Jingdong prefecture level city in Shandong
Fortune Shop	White Intelligent Power Saver	The logistics is fast the service attitude is very good.	Jingdong prefecture level city in Shandong
Fortune Shop	White Intelligent Power Saver	The meter is good quality, sturdy and durable. It is accurate. Easy to use. Saves a lot of things.	Jingdong prefecture level city in Shandong
Fortune Shop	White Intelligent Power Saver	Just received the goods, don't know how it is yet	Jingdong prefecture level city in Shandong
Fortune Shop	White Intelligent Power Saver	Energy saver received, logistics is very fast, the quality is very good, the appearance is also very beautiful power saving is the most important.	Jingdong prefecture level city in Shandong
Fortune Shop	White Intelligent Power Saver	It's good to talk about it, it's not practical to use it.	Jingdong prefecture level city in Shandong
Fortune Shop	White Intelligent Power Saver	I don't know if it's good for now, I'll comment after using it	Jingdong prefecture level city in Shandong
Fortune Shop	White Intelligent Power Saver	I've been using it for two or three days. It's not energy efficient.	Jingdong prefecture level city in Shandong
Fortune Shop	White Intelligent Power Saver	Packaging is intact, logistics is also fast, cost-effective, inexpensive, very satisfied with a purchase	Jingdong prefecture level city in Shandong
Fortune Shop	White Intelligent Power Saver	In use to be observed!	Jingdong prefecture level city in Shandong
Fortune Shop	White Intelligent Power Saver	Use it for a while at a flat rate	Jingdong prefecture level city in Shandong
Fortune Shop	White Intelligent Power Saver	Very trustworthy brand, quality is guaranteed, the price is still affordable, quite satisfied!	Jingdong prefecture level city in Shandong
Fortune Shop	White Intelligent Power Saver	Great quality, great operation, easy to install, great price!	Jingdong prefecture level city in Shandong
Fortune Shop	White Intelligent Power Saver	I came back and installed it, it really saves electricity, much more than before, the	Jingdong prefecture level city in Shandong
Fortune Shop	White Intelligent Power Saver	Fast logistics, good quality, very good packaging, good customer service	Jingdong prefecture level city in Shandong
Fortune Shop	White Intelligent Power Saver	Very good quality, especially good, effective, satisfactory	Jingdong prefecture level city in Shandong
Fortune Shop	White Intelligent Power Saver	I don't know how well it works.	Jingdong prefecture level city in Shandong
Fortune Shop	White Intelligent Power Saver	Good value for money, recommend it haha	Jingdong prefecture level city in Shandong
Fortune Shop	White Intelligent Power Saver	Good. Good. Very good.	Jingdong prefecture level city in Shandong
Fortune Shop	White Intelligent Power Saver	It's not working.	Jingdong prefecture level city in Shandong
Fortune Shop	White Intelligent Power Saver	It's not working.	Jingdong prefecture level city in Shandong
Fortune Shop	White Intelligent Power Saver	In use	Jingdong prefecture level city in Shandong
Fortune Shop	White Intelligent Power Saver	I'll try it later.	Jingdong prefecture level city in Shandong
Fortune Shop	White Intelligent Power Saver	It's not much use.	Jingdong prefecture level city in Shandong
Fortune Shop	White Intelligent Power Saver	It doesn't save electricity.	Jingdong prefecture level city in Shandong
Fortune Shop	White Intelligent Power Saver	It's not much use.	Jingdong prefecture level city in Shandong
Fortune Shop	White Intelligent Power Saver	Tried it for a few days and tracked the record, and there is no difference from before.	Jingdong prefecture level city in Shandong
Fortune Shop	White Intelligent Power Saver	It's not very credible, so just do it yourself and turn off the lights diligently!	Jingdong prefecture level city in Shandong
Fortune Shop	White Intelligent Power Saver	Too bad, no power saving at all, scam people don't fall for it!	Jingdong prefecture level city in Shandong
Fortune Shop	White Intelligent Power Saver	It's not working at all.	Jingdong prefecture level city in Shandong
Fortune Shop	White Intelligent Power Saver	general	Jingdong prefecture level city in Shandong
Fortune Shop	White Intelligent Power Saver	(of an unmarried couple) be close	Jingdong prefecture level city in Shandong
Fortune Shop	White Intelligent Power Saver	of no use	Jingdong prefecture level city in Shandong
Fortune Shop	White Intelligent Power Saver	Actually can really save electricity, only used half a month to save more than 50, recommended to the neighbors to use together!	Jingdong prefecture level city in Shandong
Fortune Shop	White Intelligent Power Saver	Save what you can. Isn't it more cost effective to buy one of these than to pay the electric bill?	Jingdong prefecture level city in Shandong
Fortune Shop	White Intelligent Power Saver	Received, a use do not know how, use a month to see the effect is good, the effect is good then recommended, not good then I just do not recommend.	Jingdong prefecture level city in Shandong
Fortune Shop	White Intelligent Power Saver	Just received the goods, is a voltage regulator, next month to evaluate whether it is really power saving	Jingdong prefecture level city in Shandong
Fortune Shop	White Intelligent Power Saver	Very good, used a few days obviously feel that the electricity bill is much less, wait a month to see how much can be saved!	Jingdong prefecture level city in Shandong
Fortune Shop	White Intelligent Power Saver	Received the stuff used for more than a month without a little use, it feels like it uses more electricity than before	Jingdong prefecture level city in Shandong
Fortune Shop	White Intelligent Power Saver	The quality is very good, completely exceeded the expectations, the delivery speed is very fast, the packaging is very careful and strict, very satisfied with a shopping	Jingdong prefecture level city in Shandong
Fortune Shop	White Intelligent Power Saver	This item doesn't seem to have much effect?	Jingdong prefecture level city in Shandong
Fortune Shop	White Intelligent Power Saver	Fake, doesn't work at all. Don't even sell it.	Jingdong prefecture level city in Shandong
Fortune Shop	White Intelligent Power Saver	This production is a bit fake, just look at the focus on Ming	Jingdong prefecture level city in Shandong
Fortune Shop	White Intelligent Power Saver	The logistics is fast the service attitude is very good.	Jingdong prefecture level city in Shandong

Fortune Shop	White Intelligent Power Saver	The meter is good quality, sturdy and durable. It is accurate. Easy to use. Saves a lot of things.	Jingdong prefecture level city in Shandong
Fortune Shop	White Intelligent Power Saver	Just received the goods, don't know how it is yet	Jingdong prefecture level city in Shandong
Fortune Shop	White Intelligent Power Saver	Energy saver received, logistics is very fast, the quality is very good, the appearance is also very beautiful power saving is the most important.	Jingdong prefecture level city in Shandong
Fortune Shop	White Intelligent Power Saver	It's good to talk about it, it's not practical to use it.	Jingdong prefecture level city in Shandong
Fortune Shop	White Intelligent Power Saver	I don't know if it's good for now, I'll comment after using it	Jingdong prefecture level city in Shandong
Fortune Shop	White Intelligent Power Saver	I've been using it for two or three days. It's not energy efficient.	Jingdong prefecture level city in Shandong
Fortune Shop	White Intelligent Power Saver	Packaging is intact, logistics is also fast, cost-effective, inexpensive, very satisfied with a purchase	Jingdong prefecture level city in Shandong
Fortune Shop	White Intelligent Power Saver	In use to be observed!	Jingdong prefecture level city in Shandong
Fortune Shop	White Intelligent Power Saver	Use it for a while at a flat rate	Jingdong prefecture level city in Shandong
Fortune Shop	White Intelligent Power Saver	Very trustworthy brand, quality is guaranteed, the price is still affordable, quite satisfied!	Jingdong prefecture level city in Shandong
Fortune Shop	White Intelligent Power Saver	Great quality, great operation, easy to install, great price!	Jingdong prefecture level city in Shandong
Fortune Shop	White Intelligent Power Saver	I came back and installed it, it really saves electricity, much more than before, the	Jingdong prefecture level city in Shandong
Fortune Shop	White Intelligent Power Saver	Fast logistics, good quality, very good packaging, good customer service	Jingdong prefecture level city in Shandong
Fortune Shop	White Intelligent Power Saver	Very good quality, especially good, effective, satisfactory	Jingdong prefecture level city in Shandong
Fortune Shop	White Intelligent Power Saver	I don't know how well it works.	Jingdong prefecture level city in Shandong
Fortune Shop	White Intelligent Power Saver	Good value for money, recommend it haha	Jingdong prefecture level city in Shandong
Fortune Shop	White Intelligent Power Saver	Good. Good. Very good.	Jingdong prefecture level city in Shandong
Fortune Shop	White Intelligent Power Saver	It's not working.	Jingdong prefecture level city in Shandong
Fortune Shop	White Intelligent Power Saver	It's not working.	Jingdong prefecture level city in Shandong
Fortune Shop	White Intelligent Power Saver	In use	Jingdong prefecture level city in Shandong
Fortune Shop	White Intelligent Power Saver	I'll try it later.	Jingdong prefecture level city in Shandong
Fortune Shop	White Intelligent Power Saver	It's not much use.	Jingdong prefecture level city in Shandong
Fortune Shop	White Intelligent Power Saver	It doesn't save electricity.	Jingdong prefecture level city in Shandong
Fortune Shop	White Intelligent Power Saver	It's not much use.	Jingdong prefecture level city in Shandong
Fortune Shop	White Intelligent Power Saver	Tried it for a few days and tracked the record, and there is no difference from before.	Jingdong prefecture level city in Shandong
Fortune Shop	White Intelligent Power Saver	It's not very credible, so just do it yourself and turn off the lights diligently!	Jingdong prefecture level city in Shandong
Fortune Shop	White Intelligent Power Saver	Too bad, no power saving at all, scam people don't fall for it!	Jingdong prefecture level city in Shandong
Fortune Shop	White Intelligent Power Saver	It's not working at all.	Jingdong prefecture level city in Shandong
Fortune Shop	White Intelligent Power Saver	general	Jingdong prefecture level city in Shandong
Fortune Shop	White Intelligent Power Saver	(of an unmarried couple) be close	Jingdong prefecture level city in Shandong
Fortune Shop	White Intelligent Power Saver	of no use	Jingdong prefecture level city in Shandong
Fortune Shop	White Intelligent Power Saver	Actually can really save electricity, only used half a month to save more than 50, recommended to the neighbors to use together!	Jingdong prefecture level city in Shandong
Fortune Shop	White Intelligent Power Saver	Save what you can. Isn't it more cost effective to buy one of these than to pay the electric bill?	Jingdong prefecture level city in Shandong
Fortune Shop	White Intelligent Power Saver	Received, a use do not know how, use a month to see the effect is good, the effect is good then recommended, not good then I just do not recommend.	Jingdong prefecture level city in Shandong
Fortune Shop	White Intelligent Power Saver	Just received the goods, is a voltage regulator, next month to evaluate whether it is really power saving	Jingdong prefecture level city in Shandong
Fortune Shop	White Intelligent Power Saver	Very good, used a few days obviously feel that the electricity bill is much less, wait a month to see how much can be saved!	Jingdong prefecture level city in Shandong
Fortune Shop	White Intelligent Power Saver	Received the stuff used for more than a month without a little use, it feels like it uses more electricity than before	Jingdong prefecture level city in Shandong
Fortune Shop	White Intelligent Power Saver	The quality is very good, completely exceeded the expectations, the delivery speed is very fast, the packaging is very careful and strict, very satisfied with a shopping	Jingdong prefecture level city in Shandong
Fortune Shop	White Intelligent Power Saver	Fake, doesn't work at all. Don't even sell it.	Jingdong prefecture level city in Shandong
Fortune Shop	White Intelligent Power Saver	This item doesn't seem to have much effect?	Jingdong prefecture level city in Shandong
Fortune Shop	White Intelligent Power Saver	This production is a bit fake, just look at the focus on Ming	Jingdong prefecture level city in Shandong
Fortune Shop	White Intelligent Power Saver	The logistics is fast the service attitude is very good.	Jingdong prefecture level city in Shandong
Fortune Shop	White Intelligent Power Saver	The meter is good quality, sturdy and durable. It is accurate. Easy to use. Saves a lot of things.	Jingdong prefecture level city in Shandong
Fortune Shop	White Intelligent Power Saver	Just received the goods, don't know how it is yet	Jingdong prefecture level city in Shandong
Fortune Shop	White Intelligent Power Saver	Energy saver received, logistics is very fast, the quality is very good, the appearance is also very beautiful power saving is the most important.	Jingdong prefecture level city in Shandong
Fortune Shop	White Intelligent Power Saver	It's good to talk about it, it's not practical to use it.	Jingdong prefecture level city in Shandong
Fortune Shop	White Intelligent Power Saver	I don't know if it's good for now, I'll comment after using it	Jingdong prefecture level city in Shandong
Fortune Shop	White Intelligent Power Saver	I've been using it for two or three days. It's not energy efficient.	Jingdong prefecture level city in Shandong
Fortune Shop	White Intelligent Power Saver	Packaging is intact, logistics is also fast, cost-effective, inexpensive, very satisfied with a purchase	Jingdong prefecture level city in Shandong
Fortune Shop	White Intelligent Power Saver	In use to be observed!	Jingdong prefecture level city in Shandong
Fortune Shop	White Intelligent Power Saver	Use it for a while at a flat rate	Jingdong prefecture level city in Shandong

Fortune Shop	White Intelligent Power Saver	Very trustworthy brand, quality is guaranteed, the price is still affordable, quite satisfied!	Jingdong prefecture level city in Shandong
Fortune Shop	White Intelligent Power Saver	Great quality, great operation, easy to install, great price!	Jingdong prefecture level city in Shandong
Fortune Shop	White Intelligent Power Saver	I came back and installed it, it really saves electricity, much more than before, the	Jingdong prefecture level city in Shandong
Fortune Shop	White Intelligent Power Saver	Fast logistics, good quality, very good packaging, good customer service	Jingdong prefecture level city in Shandong
Fortune Shop	White Intelligent Power Saver	Very good quality, especially good, effective, satisfactory	Jingdong prefecture level city in Shandong
Fortune Shop	White Intelligent Power Saver	I don't know how well it works.	Jingdong prefecture level city in Shandong
Fortune Shop	White Intelligent Power Saver	Good value for money, recommend it haha	Jingdong prefecture level city in Shandong
Fortune Shop	White Intelligent Power Saver	Good. Good. Very good.	Jingdong prefecture level city in Shandong
Fortune Shop	White Intelligent Power Saver	It's not working.	Jingdong prefecture level city in Shandong
Fortune Shop	White Intelligent Power Saver	It's not working.	Jingdong prefecture level city in Shandong
Fortune Shop	White Intelligent Power Saver	In use	Jingdong prefecture level city in Shandong
Fortune Shop	White Intelligent Power Saver	I'll try it later.	Jingdong prefecture level city in Shandong
Fortune Shop	White Intelligent Power Saver	It's not much use.	Jingdong prefecture level city in Shandong
Fortune Shop	White Intelligent Power Saver	It doesn't save electricity.	Jingdong prefecture level city in Shandong
Fortune Shop	White Intelligent Power Saver	It's not much use.	Jingdong prefecture level city in Shandong
Fortune Shop	White Intelligent Power Saver	Tried it for a few days and tracked the record, and there is no difference from before.	Jingdong prefecture level city in Shandong
Fortune Shop	White Intelligent Power Saver	It's not very credible, so just do it yourself and turn off the lights diligently!	Jingdong prefecture level city in Shandong
Fortune Shop	White Intelligent Power Saver	Too bad, no power saving at all, scam people don't fall for it!	Jingdong prefecture level city in Shandong
Fortune Shop	White Intelligent Power Saver	It's not working at all.	Jingdong prefecture level city in Shandong
Fortune Shop	White Intelligent Power Saver	general	Jingdong prefecture level city in Shandong
Fortune Shop	White Intelligent Power Saver	(of an unmarried couple) be close	Jingdong prefecture level city in Shandong
Fortune Shop	White Intelligent Power Saver	of no use	Jingdong prefecture level city in Shandong
Fortune Shop	White Intelligent Power Saver	Actually can really save electricity, only used half a month to save more than 50, recommended to the neighbors to use together!	Jingdong prefecture level city in Shandong
Fortune Shop	White Intelligent Power Saver	Save what you can. Isn't it more cost effective to buy one of these than to pay the electric bill?	Jingdong prefecture level city in Shandong
Fortune Shop	White Intelligent Power Saver	Received, a use do not know how, use a month to see the effect is good, the effect is good then recommended, not good then I just do not recommend.	Jingdong prefecture level city in Shandong
Fortune Shop	White Intelligent Power Saver	Just received the goods, is a voltage regulator, next month to evaluate whether it is really power saving	Jingdong prefecture level city in Shandong
Fortune Shop	White Intelligent Power Saver	Very good, used a few days obviously feel that the electricity bill is much less, wait a month to see how much can be saved!	Jingdong prefecture level city in Shandong
Fortune Shop	White Intelligent Power Saver	Received the stuff used for more than a month without a little use, it feels like it uses more electricity than before	Jingdong prefecture level city in Shandong
Fortune Shop	White Intelligent Power Saver	The quality is very good, completely exceeded the expectations, the delivery speed is very fast, the packaging is very careful and strict, very satisfied with a shopping	Jingdong prefecture level city in Shandong
Fortune Shop	White Intelligent Power Saver	Fake, doesn't work at all. Don't even sell it.	Jingdong prefecture level city in Shandong
Fortune Shop	White Intelligent Power Saver	This item doesn't seem to have much effect?	Jingdong prefecture level city in Shandong
Fortune Shop	White Intelligent Power Saver	This production is a bit fake, just look at the focus on Ming	Jingdong prefecture level city in Shandong
Fortune Shop	White Intelligent Power Saver	The logistics is fast the service attitude is very good.	Jingdong prefecture level city in Shandong
Fortune Shop	White Intelligent Power Saver	The meter is good quality, sturdy and durable. It is accurate. Easy to use. Saves a lot of things.	Jingdong prefecture level city in Shandong
Fortune Shop	White Intelligent Power Saver	Just received the goods, don't know how it is yet	Jingdong prefecture level city in Shandong
Fortune Shop	White Intelligent Power Saver	Energy saver received, logistics is very fast, the quality is very good, the appearance is also very beautiful power saving is the most important.	Jingdong prefecture level city in Shandong
Fortune Shop	White Intelligent Power Saver	It's good to talk about it, it's not practical to use it.	Jingdong prefecture level city in Shandong
Fortune Shop	White Intelligent Power Saver	I don't know if it's good for now, I'll comment after using it	Jingdong prefecture level city in Shandong
Fortune Shop	White Intelligent Power Saver	I've been using it for two or three days. It's not energy efficient.	Jingdong prefecture level city in Shandong
Fortune Shop	White Intelligent Power Saver	Packaging is intact, logistics is also fast, cost-effective, inexpensive, very satisfied with a purchase	Jingdong prefecture level city in Shandong
Fortune Shop	White Intelligent Power Saver	In use to be observed!	Jingdong prefecture level city in Shandong
Fortune Shop	White Intelligent Power Saver	Use it for a while at a flat rate	Jingdong prefecture level city in Shandong
Fortune Shop	White Intelligent Power Saver	Very trustworthy brand, quality is guaranteed, the price is still affordable, quite satisfied!	Jingdong prefecture level city in Shandong
Fortune Shop	White Intelligent Power Saver	Great quality, great operation, easy to install, great price!	Jingdong prefecture level city in Shandong
Fortune Shop	White Intelligent Power Saver	I came back and installed it, it really saves electricity, much more than before, the	Jingdong prefecture level city in Shandong
Fortune Shop	White Intelligent Power Saver	Fast logistics, good quality, very good packaging, good customer service	Jingdong prefecture level city in Shandong
Fortune Shop	White Intelligent Power Saver	Very good quality, especially good, effective, satisfactory	Jingdong prefecture level city in Shandong
Fortune Shop	White Intelligent Power Saver	I don't know how well it works.	Jingdong prefecture level city in Shandong
Fortune Shop	White Intelligent Power Saver	Good value for money, recommend it haha	Jingdong prefecture level city in Shandong
Fortune Shop	White Intelligent Power Saver	Good. Good. Very good.	Jingdong prefecture level city in Shandong
Fortune Shop	White Intelligent Power Saver	It's not working.	Jingdong prefecture level city in Shandong

Fortune Shop	White Intelligent Power Saver	It's not working.	Jingdong prefecture level city in Shandong
Fortune Shop	White Intelligent Power Saver	In use	Jingdong prefecture level city in Shandong
Fortune Shop	White Intelligent Power Saver	I'll try it later.	Jingdong prefecture level city in Shandong
Fortune Shop	White Intelligent Power Saver	It's not much use.	Jingdong prefecture level city in Shandong
Fortune Shop	White Intelligent Power Saver	It doesn't save electricity.	Jingdong prefecture level city in Shandong
Fortune Shop	White Intelligent Power Saver	It's not much use.	Jingdong prefecture level city in Shandong
Fortune Shop	White Intelligent Power Saver	Tried it for a few days and tracked the record, and there is no difference from before.	Jingdong prefecture level city in Shandong
Fortune Shop	White Intelligent Power Saver	It's not very credible, so just do it yourself and turn off the lights diligently!	Jingdong prefecture level city in Shandong
Fortune Shop	White Intelligent Power Saver	Too bad, no power saving at all, scam people don't fall for it!	Jingdong prefecture level city in Shandong
Fortune Shop	White Intelligent Power Saver	It's not working at all.	Jingdong prefecture level city in Shandong
Fortune Shop	White Intelligent Power Saver	general	Jingdong prefecture level city in Shandong
Fortune Shop	White Intelligent Power Saver	(of an unmarried couple) be close	Jingdong prefecture level city in Shandong
Fortune Shop	White Intelligent Power Saver	of no use	Jingdong prefecture level city in Shandong
Fortune Shop	White Intelligent Power Saver	Actually can really save electricity, only used half a month to save more than 50, recommended to the neighbors to use together!	Jingdong prefecture level city in Shandong
Fortune Shop	White Intelligent Power Saver	Save what you can. Isn't it more cost effective to buy one of these than to pay the electric bill?	Jingdong prefecture level city in Shandong
Fortune Shop	White Intelligent Power Saver	Received, a use do not know how, use a month to see the effect is good, the effect is good then recommended, not good then I just do not recommend.	Jingdong prefecture level city in Shandong
Fortune Shop	White Intelligent Power Saver	Just received the goods, is a voltage regulator, next month to evaluate whether it is really power saving	Jingdong prefecture level city in Shandong
Fortune Shop	White Intelligent Power Saver	Very good, used a few days obviously feel that the electricity bill is much less, wait a month to see how much can be saved!	Jingdong prefecture level city in Shandong
Fortune Shop	White Intelligent Power Saver	Received the stuff used for more than a month without a little use, it feels like it uses more electricity than before	Jingdong prefecture level city in Shandong
Fortune Shop	White Intelligent Power Saver	The quality is very good, completely exceeded the expectations, the delivery speed is very fast, the packaging is very careful and strict, very satisfied with a shopping	Jingdong prefecture level city in Shandong
Fortune Shop	White Intelligent Power Saver	Fake, doesn't work at all. Don't even sell it.	Jingdong prefecture level city in Shandong
Fortune Shop	White Intelligent Power Saver	This item doesn't seem to have much effect?	Jingdong prefecture level city in Shandong
Fortune Shop	White Intelligent Power Saver	This production is a bit fake, just look at the focus on Ming	Jingdong prefecture level city in Shandong
Fortune Shop	White Intelligent Power Saver	The logistics is fast the service attitude is very good.	Jingdong prefecture level city in Shandong
Fortune Shop	White Intelligent Power Saver	The meter is good quality, sturdy and durable. It is accurate. Easy to use. Saves a lot of things.	Jingdong prefecture level city in Shandong
Fortune Shop	White Intelligent Power Saver	Just received the goods, don't know how it is yet	Jingdong prefecture level city in Shandong
Fortune Shop	White Intelligent Power Saver	Energy saver received, logistics is very fast, the quality is very good, the appearance is also very beautiful power saving is the most important.	Jingdong prefecture level city in Shandong
Fortune Shop	White Intelligent Power Saver	It's good to talk about it, it's not practical to use it.	Jingdong prefecture level city in Shandong
Fortune Shop	White Intelligent Power Saver	I don't know if it's good for now, I'll comment after using it	Jingdong prefecture level city in Shandong
Fortune Shop	White Intelligent Power Saver	I've been using it for two or three days. It's not energy efficient.	Jingdong prefecture level city in Shandong
Fortune Shop	White Intelligent Power Saver	Packaging is intact, logistics is also fast, cost-effective, inexpensive, very satisfied with a purchase	Jingdong prefecture level city in Shandong
Fortune Shop	White Intelligent Power Saver	In use to be observed!	Jingdong prefecture level city in Shandong
Fortune Shop	White Intelligent Power Saver	Use it for a while at a flat rate	Jingdong prefecture level city in Shandong
Fortune Shop	White Intelligent Power Saver	Very trustworthy brand, quality is guaranteed, the price is still affordable, quite satisfied!	Jingdong prefecture level city in Shandong
Fortune Shop	White Intelligent Power Saver	Great quality, great operation, easy to install, great price!	Jingdong prefecture level city in Shandong
Fortune Shop	White Intelligent Power Saver	I came back and installed it, it really saves electricity, much more than before, the	Jingdong prefecture level city in Shandong
Fortune Shop	White Intelligent Power Saver	Fast logistics, good quality, very good packaging, good customer service	Jingdong prefecture level city in Shandong
Fortune Shop	White Intelligent Power Saver	Very good quality, especially good, effective, satisfactory	Jingdong prefecture level city in Shandong
Fortune Shop	White Intelligent Power Saver	I don't know how well it works.	Jingdong prefecture level city in Shandong
Fortune Shop	White Intelligent Power Saver	Good value for money, recommend it haha	Jingdong prefecture level city in Shandong
Fortune Shop	White Intelligent Power Saver	Good. Good. Very good.	Jingdong prefecture level city in Shandong
Fortune Shop	White Intelligent Power Saver	It's not working.	Jingdong prefecture level city in Shandong
Fortune Shop	White Intelligent Power Saver	It's not working.	Jingdong prefecture level city in Shandong
Fortune Shop	White Intelligent Power Saver	In use	Jingdong prefecture level city in Shandong
Fortune Shop	White Intelligent Power Saver	I'll try it later.	Jingdong prefecture level city in Shandong
Fortune Shop	White Intelligent Power Saver	It's not much use.	Jingdong prefecture level city in Shandong
Fortune Shop	White Intelligent Power Saver	It doesn't save electricity.	Jingdong prefecture level city in Shandong
Fortune Shop	White Intelligent Power Saver	It's not much use.	Jingdong prefecture level city in Shandong
Fortune Shop	White Intelligent Power Saver	Tried it for a few days and tracked the record, and there is no difference from before.	Jingdong prefecture level city in Shandong
Fortune Shop	White Intelligent Power Saver	It's not very credible, so just do it yourself and turn off the lights diligently!	Jingdong prefecture level city in Shandong
Fortune Shop	White Intelligent Power Saver	Too bad, no power saving at all, scam people don't fall for it!	Jingdong prefecture level city in Shandong

Fortune Shop	White Intelligent Power Saver	It's not working at all.	Jingdong prefecture level city in Shandong
Fortune Shop	White Intelligent Power Saver	general	Jingdong prefecture level city in Shandong
Fortune Shop	White Intelligent Power Saver	(of an unmarried couple) be close	Jingdong prefecture level city in Shandong
Fortune Shop	White Intelligent Power Saver	of no use	Jingdong prefecture level city in Shandong
Fortune Shop	White Intelligent Power Saver	Actually can really save electricity, only used half a month to save more than 50, recommended to the neighbors to use together!	Jingdong prefecture level city in Shandong
Fortune Shop	White Intelligent Power Saver	Save what you can. Isn't it more cost effective to buy one of these than to pay the electric bill?	Jingdong prefecture level city in Shandong
Fortune Shop	White Intelligent Power Saver	Received, a use do not know how, use a month to see the effect is good, the effect is good then recommended, not good then I just do not recommend.	Jingdong prefecture level city in Shandong
Fortune Shop	White Intelligent Power Saver	Just received the goods, is a voltage regulator, next month to evaluate whether it is really power saving	Jingdong prefecture level city in Shandong
Fortune Shop	White Intelligent Power Saver	Very good, used a few days obviously feel that the electricity bill is much less, wait a month to see how much can be saved!	Jingdong prefecture level city in Shandong
Fortune Shop	White Intelligent Power Saver	Received the stuff used for more than a month without a little use, it feels like it uses more electricity than before	Jingdong prefecture level city in Shandong
Fortune Shop	White Intelligent Power Saver	The quality is very good, completely exceeded the expectations, the delivery speed is very fast, the packaging is very careful and strict, very satisfied with a shopping	Jingdong prefecture level city in Shandong
Fortune Shop	White Intelligent Power Saver	Fake, doesn't work at all. Don't even sell it.	Jingdong prefecture level city in Shandong
Fortune Shop	White Intelligent Power Saver	This item doesn't seem to have much effect?	Jingdong prefecture level city in Shandong
Fortune Shop	White Intelligent Power Saver	This production is a bit fake, just look at the focus on Ming	Jingdong prefecture level city in Shandong
Fortune Shop	White Intelligent Power Saver	The logistics is fast the service attitude is very good.	Jingdong prefecture level city in Shandong
Fortune Shop	White Intelligent Power Saver	The meter is good quality, sturdy and durable. It is accurate. Easy to use. Saves a lot of things.	Jingdong prefecture level city in Shandong
Fortune Shop	White Intelligent Power Saver	Just received the goods, don't know how it is yet	Jingdong prefecture level city in Shandong
Fortune Shop	White Intelligent Power Saver	Energy saver received, logistics is very fast, the quality is very good, the appearance is also very beautiful power saving is the most important.	Jingdong prefecture level city in Shandong
Fortune Shop	White Intelligent Power Saver	It's good to talk about it, it's not practical to use it.	Jingdong prefecture level city in Shandong
Fortune Shop	White Intelligent Power Saver	I don't know if it's good for now, I'll comment after using it	Jingdong prefecture level city in Shandong
Fortune Shop	White Intelligent Power Saver	I've been using it for two or three days. It's not energy efficient.	Jingdong prefecture level city in Shandong
Fortune Shop	White Intelligent Power Saver	Packaging is intact, logistics is also fast, cost-effective, inexpensive, very satisfied with a purchase	Jingdong prefecture level city in Shandong
Fortune Shop	White Intelligent Power Saver	In use to be observed!	Jingdong prefecture level city in Shandong
Fortune Shop	White Intelligent Power Saver	Use it for a while at a flat rate	Jingdong prefecture level city in Shandong
Fortune Shop	White Intelligent Power Saver	Very trustworthy brand, quality is guaranteed, the price is still affordable, quite satisfied!	Jingdong prefecture level city in Shandong
Fortune Shop	White Intelligent Power Saver	Great quality, great operation, easy to install, great price!	Jingdong prefecture level city in Shandong
Fortune Shop	White Intelligent Power Saver	I came back and installed it, it really saves electricity, much more than before, the	Jingdong prefecture level city in Shandong
Fortune Shop	White Intelligent Power Saver	Fast logistics, good quality, very good packaging, good customer service	Jingdong prefecture level city in Shandong
Fortune Shop	White Intelligent Power Saver	Very good quality, especially good, effective, satisfactory	Jingdong prefecture level city in Shandong
Fortune Shop	White Intelligent Power Saver	I don't know how well it works.	Jingdong prefecture level city in Shandong
Fortune Shop	White Intelligent Power Saver	Good value for money, recommend it haha	Jingdong prefecture level city in Shandong
Fortune Shop	White Intelligent Power Saver	Good. Good. Very good.	Jingdong prefecture level city in Shandong
Fortune Shop	White Intelligent Power Saver	It's not working.	Jingdong prefecture level city in Shandong
Fortune Shop	White Intelligent Power Saver	It's not working.	Jingdong prefecture level city in Shandong
Fortune Shop	White Intelligent Power Saver	In use	Jingdong prefecture level city in Shandong
Fortune Shop	White Intelligent Power Saver	I'll try it later.	Jingdong prefecture level city in Shandong
Fortune Shop	White Intelligent Power Saver	It's not much use.	Jingdong prefecture level city in Shandong
Fortune Shop	White Intelligent Power Saver	It doesn't save electricity.	Jingdong prefecture level city in Shandong
Fortune Shop	White Intelligent Power Saver	It's not much use.	Jingdong prefecture level city in Shandong
Fortune Shop	White Intelligent Power Saver	Tried it for a few days and tracked the record, and there is no difference from before.	Jingdong prefecture level city in Shandong
Fortune Shop	White Intelligent Power Saver	It's not very credible, so just do it yourself and turn off the lights diligently!	Jingdong prefecture level city in Shandong
Fortune Shop	White Intelligent Power Saver	Too bad, no power saving at all, scam people don't fall for it!	Jingdong prefecture level city in Shandong
Fortune Shop	White Intelligent Power Saver	It's not working at all.	Jingdong prefecture level city in Shandong
Fortune Shop	White Intelligent Power Saver	general	Jingdong prefecture level city in Shandong
Fortune Shop	White Intelligent Power Saver	(of an unmarried couple) be close	Jingdong prefecture level city in Shandong
Fortune Shop	White Intelligent Power Saver	of no use	Jingdong prefecture level city in Shandong
Fortune Shop	White Intelligent Power Saver	Actually can really save electricity, only used half a month to save more than 50, recommended to the neighbors to use together!	Jingdong prefecture level city in Shandong
Fortune Shop	White Intelligent Power Saver	Save what you can. Isn't it more cost effective to buy one of these than to pay the electric bill?	Jingdong prefecture level city in Shandong
Fortune Shop	White Intelligent Power Saver	Received, a use do not know how, use a month to see the effect is good, the effect is good then recommended, not good then I just do not recommend.	Jingdong prefecture level city in Shandong
Fortune Shop	White Intelligent Power Saver	Just received the goods, is a voltage regulator, next month to evaluate whether it is really power saving	Jingdong prefecture level city in Shandong
Fortune Shop	White Intelligent Power Saver	Very good, used a few days obviously feel that the electricity bill is much less, wait a month to see how much can be saved!	Jingdong prefecture level city in Shandong

Fortune Shop	White Intelligent Power Saver	Received the stuff used for more than a month without a little use, it feels like it uses more electricity than before	Jingdong prefecture level city in Shandong
Fortune Shop	White Intelligent Power Saver	The quality is very good, completely exceeded the expectations, the delivery speed is very fast, the packaging is very careful and strict, very satisfied with a shopping	Jingdong prefecture level city in Shandong
Fortune Shop	White Intelligent Power Saver	Fake, doesn't work at all. Don't even sell it.	Jingdong prefecture level city in Shandong
Fortune Shop	White Intelligent Power Saver	This item doesn't seem to have much effect?	Jingdong prefecture level city in Shandong
Fortune Shop	White Intelligent Power Saver	This production is a bit fake, just look at the focus on Ming	Jingdong prefecture level city in Shandong
Fortune Shop	White Intelligent Power Saver	The logistics is fast the service attitude is very good.	Jingdong prefecture level city in Shandong
Fortune Shop	White Intelligent Power Saver	The meter is good quality, sturdy and durable. It is accurate. Easy to use. Saves a lot of things.	Jingdong prefecture level city in Shandong
Fortune Shop	White Intelligent Power Saver	Just received the goods, don't know how it is yet	Jingdong prefecture level city in Shandong
Fortune Shop	White Intelligent Power Saver	Energy saver received, logistics is very fast, the quality is very good, the appearance is also very beautiful power saving is the most important.	Jingdong prefecture level city in Shandong
Fortune Shop	White Intelligent Power Saver	It's good to talk about it, it's not practical to use it.	Jingdong prefecture level city in Shandong
Fortune Shop	White Intelligent Power Saver	I don't know if it's good for now, I'll comment after using it	Jingdong prefecture level city in Shandong
Fortune Shop	White Intelligent Power Saver	I've been using it for two or three days. It's not energy efficient.	Jingdong prefecture level city in Shandong
Fortune Shop	White Intelligent Power Saver	Packaging is intact, logistics is also fast, cost-effective, inexpensive, very satisfied with a purchase	Jingdong prefecture level city in Shandong
Fortune Shop	White Intelligent Power Saver	In use to be observed!	Jingdong prefecture level city in Shandong
Fortune Shop	White Intelligent Power Saver	Use it for a while at a flat rate	Jingdong prefecture level city in Shandong
Fortune Shop	White Intelligent Power Saver	Very trustworthy brand, quality is guaranteed, the price is still affordable, quite satisfied!	Jingdong prefecture level city in Shandong
Fortune Shop	White Intelligent Power Saver	Great quality, great operation, easy to install, great price!	Jingdong prefecture level city in Shandong
Fortune Shop	White Intelligent Power Saver	I came back and installed it, it really saves electricity, much more than before, the	Jingdong prefecture level city in Shandong
Fortune Shop	White Intelligent Power Saver	Fast logistics, good quality, very good packaging, good customer service	Jingdong prefecture level city in Shandong
Fortune Shop	White Intelligent Power Saver	Very good quality, especially good, effective, satisfactory	Jingdong prefecture level city in Shandong
Fortune Shop	White Intelligent Power Saver	I don't know how well it works.	Jingdong prefecture level city in Shandong
Fortune Shop	White Intelligent Power Saver	Good value for money, recommend it haha	Jingdong prefecture level city in Shandong
Fortune Shop	White Intelligent Power Saver	Good. Good. Very good.	Jingdong prefecture level city in Shandong
Fortune Shop	White Intelligent Power Saver	It's not working.	Jingdong prefecture level city in Shandong
Fortune Shop	White Intelligent Power Saver	It's not working.	Jingdong prefecture level city in Shandong
Fortune Shop	White Intelligent Power Saver	In use	Jingdong prefecture level city in Shandong
Fortune Shop	White Intelligent Power Saver	I'll try it later.	Jingdong prefecture level city in Shandong
Fortune Shop	White Intelligent Power Saver	It's not much use.	Jingdong prefecture level city in Shandong
Fortune Shop	White Intelligent Power Saver	It doesn't save electricity.	Jingdong prefecture level city in Shandong
Fortune Shop	White Intelligent Power Saver	It's not much use.	Jingdong prefecture level city in Shandong
Fortune Shop	White Intelligent Power Saver	Tried it for a few days and tracked the record, and there is no difference from before.	Jingdong prefecture level city in Shandong
Fortune Shop	White Intelligent Power Saver	It's not very credible, so just do it yourself and turn off the lights diligently!	Jingdong prefecture level city in Shandong
Fortune Shop	White Intelligent Power Saver	Too bad, no power saving at all, scam people don't fall for it!	Jingdong prefecture level city in Shandong
Fortune Shop	White Intelligent Power Saver	It's not working at all.	Jingdong prefecture level city in Shandong
Fortune Shop	White Intelligent Power Saver	general	Jingdong prefecture level city in Shandong
Fortune Shop	White Intelligent Power Saver	(of an unmarried couple) be close	Jingdong prefecture level city in Shandong
Fortune Shop	White Intelligent Power Saver	of no use	Jingdong prefecture level city in Shandong
Fortune Shop	White Intelligent Power Saver	Actually can really save electricity, only used half a month to save more than 50, recommended to the neighbors to use together!	Jingdong prefecture level city in Shandong
Fortune Shop	White Intelligent Power Saver	Save what you can. Isn't it more cost effective to buy one of these than to pay the electric bill?	Jingdong prefecture level city in Shandong
Fortune Shop	White Intelligent Power Saver	Received, a use do not know how, use a month to see the effect is good, the effect is good then recommended, not good then I just do not recommend.	Jingdong prefecture level city in Shandong
Fortune Shop	White Intelligent Power Saver	Just received the goods, is a voltage regulator, next month to evaluate whether it is really power saving	Jingdong prefecture level city in Shandong
Fortune Shop	White Intelligent Power Saver	Very good, used a few days obviously feel that the electricity bill is much less, wait a month to see how much can be saved!	Jingdong prefecture level city in Shandong
Fortune Shop	White Intelligent Power Saver	Received the stuff used for more than a month without a little use, it feels like it uses more electricity than before	Jingdong prefecture level city in Shandong
Fortune Shop	White Intelligent Power Saver	The quality is very good, completely exceeded the expectations, the delivery speed is very fast, the packaging is very careful and strict, very satisfied with a shopping	Jingdong prefecture level city in Shandong
Fortune Shop	White Intelligent Power Saver	Fake, doesn't work at all. Don't even sell it.	Jingdong prefecture level city in Shandong
Fortune Shop	White Intelligent Power Saver	This item doesn't seem to have much effect?	Jingdong prefecture level city in Shandong
Fortune Shop	White Intelligent Power Saver	This production is a bit fake, just look at the focus on Ming	Jingdong prefecture level city in Shandong
Fortune Shop	White Intelligent Power Saver	The logistics is fast the service attitude is very good.	Jingdong prefecture level city in Shandong
Fortune Shop	White Intelligent Power Saver	The meter is good quality, sturdy and durable. It is accurate. Easy to use. Saves a lot of things.	Jingdong prefecture level city in Shandong
Fortune Shop	White Intelligent Power Saver	Just received the goods, don't know how it is yet	Jingdong prefecture level city in Shandong
Fortune Shop	White Intelligent Power Saver	Energy saver received, logistics is very fast, the quality is very good, the appearance is also very beautiful power saving is the most important.	Jingdong prefecture level city in Shandong

Fortune Shop	White Intelligent Power Saver	It's good to talk about it, it's not practical to use it.	Jingdong prefecture level city in Shandong
Fortune Shop	White Intelligent Power Saver	I don't know if it's good for now, I'll comment after using it	Jingdong prefecture level city in Shandong
Fortune Shop	White Intelligent Power Saver	I've been using it for two or three days. It's not energy efficient.	Jingdong prefecture level city in Shandong
Fortune Shop	White Intelligent Power Saver	Packaging is intact, logistics is also fast, cost-effective, inexpensive, very satisfied with a purchase	Jingdong prefecture level city in Shandong
Fortune Shop	White Intelligent Power Saver	In use to be observed!	Jingdong prefecture level city in Shandong
Fortune Shop	White Intelligent Power Saver	Use it for a while at a flat rate	Jingdong prefecture level city in Shandong
Fortune Shop	White Intelligent Power Saver	Very trustworthy brand, quality is guaranteed, the price is still affordable, quite satisfied!	Jingdong prefecture level city in Shandong
Fortune Shop	White Intelligent Power Saver	Great quality, great operation, easy to install, great price!	Jingdong prefecture level city in Shandong
Fortune Shop	White Intelligent Power Saver	I came back and installed it, it really saves electricity, much more than before, the	Jingdong prefecture level city in Shandong
Fortune Shop	White Intelligent Power Saver	Fast logistics, good quality, very good packaging, good customer service	Jingdong prefecture level city in Shandong
Fortune Shop	White Intelligent Power Saver	Very good quality, especially good, effective, satisfactory	Jingdong prefecture level city in Shandong
Fortune Shop	White Intelligent Power Saver	I don't know how well it works.	Jingdong prefecture level city in Shandong
Fortune Shop	White Intelligent Power Saver	Good value for money, recommend it haha	Jingdong prefecture level city in Shandong
Fortune Shop	White Intelligent Power Saver	Good. Good. Very good.	Jingdong prefecture level city in Shandong
Fortune Shop	White Intelligent Power Saver	It's not working.	Jingdong prefecture level city in Shandong
Fortune Shop	White Intelligent Power Saver	It's not working.	Jingdong prefecture level city in Shandong
Fortune Shop	White Intelligent Power Saver	In use	Jingdong prefecture level city in Shandong
Fortune Shop	White Intelligent Power Saver	I'll try it later.	Jingdong prefecture level city in Shandong
Fortune Shop	White Intelligent Power Saver	It's not much use.	Jingdong prefecture level city in Shandong
Fortune Shop	White Intelligent Power Saver	It doesn't save electricity.	Jingdong prefecture level city in Shandong
Fortune Shop	White Intelligent Power Saver	It's not much use.	Jingdong prefecture level city in Shandong
Fortune Shop	White Intelligent Power Saver	Tried it for a few days and tracked the record, and there is no difference from before.	Jingdong prefecture level city in Shandong
Fortune Shop	White Intelligent Power Saver	It's not very credible, so just do it yourself and turn off the lights diligently!	Jingdong prefecture level city in Shandong
Fortune Shop	White Intelligent Power Saver	Too bad, no power saving at all, scam people don't fall for it!	Jingdong prefecture level city in Shandong
Fortune Shop	White Intelligent Power Saver	It's not working at all.	Jingdong prefecture level city in Shandong
Fortune Shop	White Intelligent Power Saver	general	Jingdong prefecture level city in Shandong
Fortune Shop	White Intelligent Power Saver	(of an unmarried couple) be close	Jingdong prefecture level city in Shandong
Fortune Shop	White Intelligent Power Saver	of no use	Jingdong prefecture level city in Shandong
Fortune Shop	White Intelligent Power Saver	Actually can really save electricity, only used half a month to save more than 50, recommended to the neighbors to use together!	Jingdong prefecture level city in Shandong
Fortune Shop	White Intelligent Power Saver	Save what you can. Isn't it more cost effective to buy one of these than to pay the electric bill?	Jingdong prefecture level city in Shandong
Fortune Shop	White Intelligent Power Saver	Received, a use do not know how, use a month to see the effect is good, the effect is good then recommended, not good then I just do not recommend.	Jingdong prefecture level city in Shandong
Fortune Shop	White Intelligent Power Saver	Just received the goods, is a voltage regulator, next month to evaluate whether it is really power saving	Jingdong prefecture level city in Shandong
Fortune Shop	White Intelligent Power Saver	Very good, used a few days obviously feel that the electricity bill is much less, wait a month to see how much can be saved!	Jingdong prefecture level city in Shandong
Fortune Shop	White Intelligent Power Saver	Received the stuff used for more than a month without a little use, it feels like it uses more electricity than before	Jingdong prefecture level city in Shandong
Fortune Shop	White Intelligent Power Saver	The quality is very good, completely exceeded the expectations, the delivery speed is very fast, the packaging is very careful and strict, very satisfied with a shopping	Jingdong prefecture level city in Shandong
Fortune Shop	White Intelligent Power Saver	Fake, doesn't work at all. Don't even sell it.	Jingdong prefecture level city in Shandong
Fortune Shop	White Intelligent Power Saver	This item doesn't seem to have much effect?	Jingdong prefecture level city in Shandong
Fortune Shop	White Intelligent Power Saver	This production is a bit fake, just look at the focus on Ming	Jingdong prefecture level city in Shandong
Fortune Shop	White Intelligent Power Saver	The logistics is fast the service attitude is very good.	Jingdong prefecture level city in Shandong
Fortune Shop	White Intelligent Power Saver	The meter is good quality, sturdy and durable. It is accurate. Easy to use. Saves a lot of things.	Jingdong prefecture level city in Shandong
Fortune Shop	White Intelligent Power Saver	Just received the goods, don't know how it is yet	Jingdong prefecture level city in Shandong
Fortune Shop	White Intelligent Power Saver	Energy saver received, logistics is very fast, the quality is very good, the appearance is also very beautiful power saving is the most important.	Jingdong prefecture level city in Shandong
Fortune Shop	White Intelligent Power Saver	It's good to talk about it, it's not practical to use it.	Jingdong prefecture level city in Shandong
Fortune Shop	White Intelligent Power Saver	I don't know if it's good for now, I'll comment after using it	Jingdong prefecture level city in Shandong
Fortune Shop	White Intelligent Power Saver	I've been using it for two or three days. It's not energy efficient.	Jingdong prefecture level city in Shandong
Fortune Shop	White Intelligent Power Saver	Packaging is intact, logistics is also fast, cost-effective, inexpensive, very satisfied with a purchase	Jingdong prefecture level city in Shandong
Fortune Shop	White Intelligent Power Saver	In use to be observed!	Jingdong prefecture level city in Shandong
Fortune Shop	White Intelligent Power Saver	Use it for a while at a flat rate	Jingdong prefecture level city in Shandong
Fortune Shop	White Intelligent Power Saver	Very trustworthy brand, quality is guaranteed, the price is still affordable, quite satisfied!	Jingdong prefecture level city in Shandong
Fortune Shop	White Intelligent Power Saver	Great quality, great operation, easy to install, great price!	Jingdong prefecture level city in Shandong
Fortune Shop	White Intelligent Power Saver	I came back and installed it, it really saves electricity, much more than before, the	Jingdong prefecture level city in Shandong

Fortune Shop	White Intelligent Power Saver	Fast logistics, good quality, very good packaging, good customer service	Jingdong prefecture level city in Shandong
Fortune Shop	White Intelligent Power Saver	Very good quality, especially good, effective, satisfactory	Jingdong prefecture level city in Shandong
Fortune Shop	White Intelligent Power Saver	I don't know how well it works.	Jingdong prefecture level city in Shandong
Fortune Shop	White Intelligent Power Saver	Good value for money, recommend it haha	Jingdong prefecture level city in Shandong
Fortune Shop	White Intelligent Power Saver	Good. Good. Very good.	Jingdong prefecture level city in Shandong
Fortune Shop	White Intelligent Power Saver	It's not working.	Jingdong prefecture level city in Shandong
Fortune Shop	White Intelligent Power Saver	It's not working.	Jingdong prefecture level city in Shandong
Fortune Shop	White Intelligent Power Saver	In use	Jingdong prefecture level city in Shandong
Fortune Shop	White Intelligent Power Saver	I'll try it later.	Jingdong prefecture level city in Shandong
Fortune Shop	White Intelligent Power Saver	It's not much use.	Jingdong prefecture level city in Shandong
Fortune Shop	White Intelligent Power Saver	It doesn't save electricity.	Jingdong prefecture level city in Shandong
Fortune Shop	White Intelligent Power Saver	It's not much use.	Jingdong prefecture level city in Shandong
Fortune Shop	White Intelligent Power Saver	Tried it for a few days and tracked the record, and there is no difference from before.	Jingdong prefecture level city in Shandong
Fortune Shop	White Intelligent Power Saver	It's not very credible, so just do it yourself and turn off the lights diligently!	Jingdong prefecture level city in Shandong
Fortune Shop	White Intelligent Power Saver	Too bad, no power saving at all, scam people don't fall for it!	Jingdong prefecture level city in Shandong
Fortune Shop	White Intelligent Power Saver	It's not working at all.	Jingdong prefecture level city in Shandong
Fortune Shop	White Intelligent Power Saver	general	Jingdong prefecture level city in Shandong
Fortune Shop	White Intelligent Power Saver	(of an unmarried couple) be close	Jingdong prefecture level city in Shandong
Fortune Shop	White Intelligent Power Saver	of no use	Jingdong prefecture level city in Shandong
Fortune Shop	White Intelligent Power Saver	Actually can really save electricity, only used half a month to save more than 50, recommended to the neighbors to use together!	Jingdong prefecture level city in Shandong
Fortune Shop	White Intelligent Power Saver	Save what you can. Isn't it more cost effective to buy one of these than to pay the electric bill?	Jingdong prefecture level city in Shandong
Fortune Shop	White Intelligent Power Saver	Received, a use do not know how, use a month to see the effect is good, the effect is good then recommended, not good then I just do not recommend.	Jingdong prefecture level city in Shandong
Fortune Shop	White Intelligent Power Saver	Just received the goods, is a voltage regulator, next month to evaluate whether it is really power saving	Jingdong prefecture level city in Shandong
Fortune Shop	White Intelligent Power Saver	Very good, used a few days obviously feel that the electricity bill is much less, wait a month to see how much can be saved!	Jingdong prefecture level city in Shandong
Fortune Shop	White Intelligent Power Saver	Received the stuff used for more than a month without a little use, it feels like it uses more electricity than before	Jingdong prefecture level city in Shandong
Fortune Shop	White Intelligent Power Saver	The quality is very good, completely exceeded the expectations, the delivery speed is very fast, the packaging is very careful and strict, very satisfied with a shopping	Jingdong prefecture level city in Shandong
Fortune Shop	White Intelligent Power Saver	Fake, doesn't work at all. Don't even sell it.	Jingdong prefecture level city in Shandong
Fortune Shop	White Intelligent Power Saver	This item doesn't seem to have much effect?	Jingdong prefecture level city in Shandong
Fortune Shop	White Intelligent Power Saver	This production is a bit fake, just look at the focus on Ming	Jingdong prefecture level city in Shandong
Fortune Shop	White Intelligent Power Saver	The logistics is fast the service attitude is very good.	Jingdong prefecture level city in Shandong
Fortune Shop	White Intelligent Power Saver	The meter is good quality, sturdy and durable. It is accurate. Easy to use. Saves a lot of things.	Jingdong prefecture level city in Shandong
Fortune Shop	White Intelligent Power Saver	Just received the goods, don't know how it is yet	Jingdong prefecture level city in Shandong
Fortune Shop	White Intelligent Power Saver	Energy saver received, logistics is very fast, the quality is very good, the appearance is also very beautiful power saving is the most important.	Jingdong prefecture level city in Shandong
Fortune Shop	White Intelligent Power Saver	It's good to talk about it, it's not practical to use it.	Jingdong prefecture level city in Shandong
Fortune Shop	White Intelligent Power Saver	I don't know if it's good for now, I'll comment after using it	Jingdong prefecture level city in Shandong
Fortune Shop	White Intelligent Power Saver	I've been using it for two or three days. It's not energy efficient.	Jingdong prefecture level city in Shandong
Fortune Shop	White Intelligent Power Saver	Packaging is intact, logistics is also fast, cost-effective, inexpensive, very satisfied with a purchase	Jingdong prefecture level city in Shandong
Fortune Shop	White Intelligent Power Saver	In use to be observed!	Jingdong prefecture level city in Shandong
Fortune Shop	White Intelligent Power Saver	Use it for a while at a flat rate	Jingdong prefecture level city in Shandong
Fortune Shop	White Intelligent Power Saver	Very trustworthy brand, quality is guaranteed, the price is still affordable, quite satisfied!	Jingdong prefecture level city in Shandong
Fortune Shop	White Intelligent Power Saver	Great quality, great operation, easy to install, great price!	Jingdong prefecture level city in Shandong
Fortune Shop	White Intelligent Power Saver	I came back and installed it, it really saves electricity, much more than before, the	Jingdong prefecture level city in Shandong
Fortune Shop	White Intelligent Power Saver	Fast logistics, good quality, very good packaging, good customer service	Jingdong prefecture level city in Shandong
Fortune Shop	White Intelligent Power Saver	Very good quality, especially good, effective, satisfactory	Jingdong prefecture level city in Shandong
Fortune Shop	White Intelligent Power Saver	I don't know how well it works.	Jingdong prefecture level city in Shandong
Fortune Shop	White Intelligent Power Saver	Good value for money, recommend it haha	Jingdong prefecture level city in Shandong
Fortune Shop	White Intelligent Power Saver	Good. Good. Very good.	Jingdong prefecture level city in Shandong
Fortune Shop	White Intelligent Power Saver	It's not working.	Jingdong prefecture level city in Shandong
Fortune Shop	White Intelligent Power Saver	It's not working.	Jingdong prefecture level city in Shandong
Fortune Shop	White Intelligent Power Saver	In use	Jingdong prefecture level city in Shandong
Fortune Shop	White Intelligent Power Saver	I'll try it later.	Jingdong prefecture level city in Shandong

Fortune Shop	White Intelligent Power Saver	It's not much use.	Jingdong prefecture level city in Shandong
Fortune Shop	White Intelligent Power Saver	It doesn't save electricity.	Jingdong prefecture level city in Shandong
Fortune Shop	White Intelligent Power Saver	It's not much use.	Jingdong prefecture level city in Shandong
Fortune Shop	White Intelligent Power Saver	Tried it for a few days and tracked the record, and there is no difference from before.	Jingdong prefecture level city in Shandong
Fortune Shop	White Intelligent Power Saver	It's not very credible, so just do it yourself and turn off the lights diligently!	Jingdong prefecture level city in Shandong
Fortune Shop	White Intelligent Power Saver	Too bad, no power saving at all, scam people don't fall for it!	Jingdong prefecture level city in Shandong
Fortune Shop	White Intelligent Power Saver	It's not working at all.	Jingdong prefecture level city in Shandong
Fortune Shop	White Intelligent Power Saver	general	Jingdong prefecture level city in Shandong
Fortune Shop	White Intelligent Power Saver	(of an unmarried couple) be close	Jingdong prefecture level city in Shandong
Fortune Shop	White Intelligent Power Saver	of no use	Jingdong prefecture level city in Shandong
Fortune Shop	White Intelligent Power Saver	Actually can really save electricity, only used half a month to save more than 50, recommended to the neighbors to use together!	Jingdong prefecture level city in Shandong
Fortune Shop	White Intelligent Power Saver	Save what you can. Isn't it more cost effective to buy one of these than to pay the electric bill?	Jingdong prefecture level city in Shandong
Fortune Shop	White Intelligent Power Saver	Received, a use do not know how, use a month to see the effect is good, the effect is good then recommended, not good then I just do not recommend.	Jingdong prefecture level city in Shandong
Fortune Shop	White Intelligent Power Saver	Just received the goods, is a voltage regulator, next month to evaluate whether it is really power saving	Jingdong prefecture level city in Shandong
Fortune Shop	White Intelligent Power Saver	Very good, used a few days obviously feel that the electricity bill is much less, wait a month to see how much can be saved!	Jingdong prefecture level city in Shandong
Fortune Shop	White Intelligent Power Saver	Received the stuff used for more than a month without a little use, it feels like it uses more electricity than before	Jingdong prefecture level city in Shandong
Fortune Shop	White Intelligent Power Saver	The quality is very good, completely exceeded the expectations, the delivery speed is very fast, the packaging is very careful and strict, very satisfied with a shopping	Jingdong prefecture level city in Shandong
Fortune Shop	White Intelligent Power Saver	Fake, doesn't work at all. Don't even sell it.	Jingdong prefecture level city in Shandong
Fortune Shop	White Intelligent Power Saver	This item doesn't seem to have much effect?	Jingdong prefecture level city in Shandong
Fortune Shop	White Intelligent Power Saver	This production is a bit fake, just look at the focus on Ming	Jingdong prefecture level city in Shandong
Fortune Shop	White Intelligent Power Saver	The logistics is fast the service attitude is very good.	Jingdong prefecture level city in Shandong
Fortune Shop	White Intelligent Power Saver	The meter is good quality, sturdy and durable. It is accurate. Easy to use. Saves a lot of things.	Jingdong prefecture level city in Shandong
Fortune Shop	White Intelligent Power Saver	Just received the goods, don't know how it is yet	Jingdong prefecture level city in Shandong
Fortune Shop	White Intelligent Power Saver	Energy saver received, logistics is very fast, the quality is very good, the appearance is also very beautiful power saving is the most important.	Jingdong prefecture level city in Shandong
Fortune Shop	White Intelligent Power Saver	It's good to talk about it, it's not practical to use it.	Jingdong prefecture level city in Shandong
Fortune Shop	White Intelligent Power Saver	I don't know if it's good for now, I'll comment after using it	Jingdong prefecture level city in Shandong
Fortune Shop	White Intelligent Power Saver	I've been using it for two or three days. It's not energy efficient.	Jingdong prefecture level city in Shandong
Fortune Shop	White Intelligent Power Saver	Packaging is intact, logistics is also fast, cost-effective, inexpensive, very satisfied with a purchase	Jingdong prefecture level city in Shandong
Fortune Shop	White Intelligent Power Saver	In use to be observed!	Jingdong prefecture level city in Shandong
Fortune Shop	White Intelligent Power Saver	Use it for a while at a flat rate	Jingdong prefecture level city in Shandong
Fortune Shop	White Intelligent Power Saver	Very trustworthy brand, quality is guaranteed, the price is still affordable, quite satisfied!	Jingdong prefecture level city in Shandong
Fortune Shop	White Intelligent Power Saver	Great quality, great operation, easy to install, great price!	Jingdong prefecture level city in Shandong
Fortune Shop	White Intelligent Power Saver	I came back and installed it, it really saves electricity, much more than before, the	Jingdong prefecture level city in Shandong
Fortune Shop	White Intelligent Power Saver	Fast logistics, good quality, very good packaging, good customer service	Jingdong prefecture level city in Shandong
Fortune Shop	White Intelligent Power Saver	Very good quality, especially good, effective, satisfactory	Jingdong prefecture level city in Shandong
Fortune Shop	White Intelligent Power Saver	I don't know how well it works.	Jingdong prefecture level city in Shandong
Fortune Shop	White Intelligent Power Saver	Good value for money, recommend it haha	Jingdong prefecture level city in Shandong
Fortune Shop	White Intelligent Power Saver	Good. Good. Very good.	Jingdong prefecture level city in Shandong
Fortune Shop	White Intelligent Power Saver	It's not working.	Jingdong prefecture level city in Shandong
Fortune Shop	White Intelligent Power Saver	It's not working.	Jingdong prefecture level city in Shandong
Fortune Shop	White Intelligent Power Saver	In use	Jingdong prefecture level city in Shandong
Fortune Shop	White Intelligent Power Saver	I'll try it later.	Jingdong prefecture level city in Shandong
Fortune Shop	White Intelligent Power Saver	It's not much use.	Jingdong prefecture level city in Shandong
Fortune Shop	White Intelligent Power Saver	It doesn't save electricity.	Jingdong prefecture level city in Shandong
Fortune Shop	White Intelligent Power Saver	It's not much use.	Jingdong prefecture level city in Shandong
Fortune Shop	White Intelligent Power Saver	Tried it for a few days and tracked the record, and there is no difference from before.	Jingdong prefecture level city in Shandong
Fortune Shop	White Intelligent Power Saver	It's not very credible, so just do it yourself and turn off the lights diligently!	Jingdong prefecture level city in Shandong
Fortune Shop	White Intelligent Power Saver	Too bad, no power saving at all, scam people don't fall for it!	Jingdong prefecture level city in Shandong
Fortune Shop	White Intelligent Power Saver	It's not working at all.	Jingdong prefecture level city in Shandong
Fortune Shop	White Intelligent Power Saver	general	Jingdong prefecture level city in Shandong
Fortune Shop	White Intelligent Power Saver	(of an unmarried couple) be close	Jingdong prefecture level city in Shandong

Fortune Shop	White Intelligent Power Saver	of no use	Jingdong prefecture level city in Shandong
Fortune Shop	White Intelligent Power Saver	Actually can really save electricity, only used half a month to save more than 50, recommended to the neighbors to use together!	Jingdong prefecture level city in Shandong
Fortune Shop	White Intelligent Power Saver	Save what you can. Isn't it more cost effective to buy one of these than to pay the electric bill?	Jingdong prefecture level city in Shandong
Fortune Shop	White Intelligent Power Saver	Received, a use do not know how, use a month to see the effect is good, the effect is good then recommended, not good then I just do not recommend.	Jingdong prefecture level city in Shandong
Fortune Shop	White Intelligent Power Saver	Just received the goods, is a voltage regulator, next month to evaluate whether it is really power saving	Jingdong prefecture level city in Shandong
Fortune Shop	White Intelligent Power Saver	Very good, used a few days obviously feel that the electricity bill is much less, wait a month to see how much can be saved!	Jingdong prefecture level city in Shandong
Fortune Shop	White Intelligent Power Saver	Received the stuff used for more than a month without a little use, it feels like it uses more electricity than before	Jingdong prefecture level city in Shandong
Fortune Shop	White Intelligent Power Saver	The quality is very good, completely exceeded the expectations, the delivery speed is very fast, the packaging is very careful and strict, very satisfied with a shopping	Jingdong prefecture level city in Shandong
Fortune Shop	White Intelligent Power Saver	Fake, doesn't work at all. Don't even sell it.	Jingdong prefecture level city in Shandong
Fortune Shop	White Intelligent Power Saver	This item doesn't seem to have much effect?	Jingdong prefecture level city in Shandong
Fortune Shop	White Intelligent Power Saver	This production is a bit fake, just look at the focus on Ming	Jingdong prefecture level city in Shandong
Fortune Shop	White Intelligent Power Saver	The logistics is fast the service attitude is very good.	Jingdong prefecture level city in Shandong
Fortune Shop	White Intelligent Power Saver	The meter is good quality, sturdy and durable. It is accurate. Easy to use. Saves a lot of things.	Jingdong prefecture level city in Shandong
Fortune Shop	White Intelligent Power Saver	Just received the goods, don't know how it is yet	Jingdong prefecture level city in Shandong
Fortune Shop	White Intelligent Power Saver	Energy saver received, logistics is very fast, the quality is very good, the appearance is also very beautiful power saving is the most important.	Jingdong prefecture level city in Shandong
Fortune Shop	White Intelligent Power Saver	It's good to talk about it, it's not practical to use it.	Jingdong prefecture level city in Shandong
Fortune Shop	White Intelligent Power Saver	I don't know if it's good for now, I'll comment after using it	Jingdong prefecture level city in Shandong
Fortune Shop	White Intelligent Power Saver	I've been using it for two or three days. It's not energy efficient.	Jingdong prefecture level city in Shandong
Fortune Shop	White Intelligent Power Saver	Packaging is intact, logistics is also fast, cost-effective, inexpensive, very satisfied with a purchase	Jingdong prefecture level city in Shandong
Fortune Shop	White Intelligent Power Saver	In use to be observed!	Jingdong prefecture level city in Shandong
Fortune Shop	White Intelligent Power Saver	Use it for a while at a flat rate	Jingdong prefecture level city in Shandong
Fortune Shop	White Intelligent Power Saver	Very trustworthy brand, quality is guaranteed, the price is still affordable, quite satisfied!	Jingdong prefecture level city in Shandong
Fortune Shop	White Intelligent Power Saver	Great quality, great operation, easy to install, great price!	Jingdong prefecture level city in Shandong
Fortune Shop	White Intelligent Power Saver	I came back and installed it, it really saves electricity, much more than before, the	Jingdong prefecture level city in Shandong
Fortune Shop	White Intelligent Power Saver	Fast logistics, good quality, very good packaging, good customer service	Jingdong prefecture level city in Shandong
Fortune Shop	White Intelligent Power Saver	Very good quality, especially good, effective, satisfactory	Jingdong prefecture level city in Shandong
Fortune Shop	White Intelligent Power Saver	I don't know how well it works.	Jingdong prefecture level city in Shandong
Fortune Shop	White Intelligent Power Saver	Good value for money, recommend it haha	Jingdong prefecture level city in Shandong
Fortune Shop	White Intelligent Power Saver	Good. Good. Very good.	Jingdong prefecture level city in Shandong
Fortune Shop	White Intelligent Power Saver	It's not working.	Jingdong prefecture level city in Shandong
Fortune Shop	White Intelligent Power Saver	It's not working.	Jingdong prefecture level city in Shandong
Fortune Shop	White Intelligent Power Saver	In use	Jingdong prefecture level city in Shandong
Fortune Shop	White Intelligent Power Saver	I'll try it later.	Jingdong prefecture level city in Shandong
Fortune Shop	White Intelligent Power Saver	It's not much use.	Jingdong prefecture level city in Shandong
Fortune Shop	White Intelligent Power Saver	It doesn't save electricity.	Jingdong prefecture level city in Shandong
Fortune Shop	White Intelligent Power Saver	It's not much use.	Jingdong prefecture level city in Shandong
Fortune Shop	White Intelligent Power Saver	Tried it for a few days and tracked the record, and there is no difference from before.	Jingdong prefecture level city in Shandong
Fortune Shop	White Intelligent Power Saver	It's not very credible, so just do it yourself and turn off the lights diligently!	Jingdong prefecture level city in Shandong
Fortune Shop	White Intelligent Power Saver	Too bad, no power saving at all, scam people don't fall for it!	Jingdong prefecture level city in Shandong
Fortune Shop	White Intelligent Power Saver	It's not working at all.	Jingdong prefecture level city in Shandong
Fortune Shop	White Intelligent Power Saver	general	Jingdong prefecture level city in Shandong
Fortune Shop	White Intelligent Power Saver	(of an unmarried couple) be close	Jingdong prefecture level city in Shandong
Fortune Shop	White Intelligent Power Saver	of no use	Jingdong prefecture level city in Shandong
Fortune Shop	White Intelligent Power Saver	Actually can really save electricity, only used half a month to save more than 50, recommended to the neighbors to use together!	Jingdong prefecture level city in Shandong
Fortune Shop	White Intelligent Power Saver	Save what you can. Isn't it more cost effective to buy one of these than to pay the electric bill?	Jingdong prefecture level city in Shandong
Fortune Shop	White Intelligent Power Saver	Received, a use do not know how, use a month to see the effect is good, the effect is good then recommended, not good then I just do not recommend.	Jingdong prefecture level city in Shandong
Fortune Shop	White Intelligent Power Saver	Just received the goods, is a voltage regulator, next month to evaluate whether it is really power saving	Jingdong prefecture level city in Shandong
Fortune Shop	White Intelligent Power Saver	Very good, used a few days obviously feel that the electricity bill is much less, wait a month to see how much can be saved!	Jingdong prefecture level city in Shandong
Fortune Shop	White Intelligent Power Saver	Received the stuff used for more than a month without a little use, it feels like it uses more electricity than before	Jingdong prefecture level city in Shandong
Fortune Shop	White Intelligent Power Saver	The quality is very good, completely exceeded the expectations, the delivery speed is very fast, the packaging is very careful and strict, very satisfied with a shopping	Jingdong prefecture level city in Shandong
Fortune Shop	White Intelligent Power Saver	Fake, doesn't work at all. Don't even sell it.	Jingdong prefecture level city in Shandong

Fortune Shop	White Intelligent Power Saver	This item doesn't seem to have much effect?	Jingdong prefecture level city in Shandong
Fortune Shop	White Intelligent Power Saver	This production is a bit fake, just look at the focus on Ming	Jingdong prefecture level city in Shandong
Fortune Shop	White Intelligent Power Saver	The logistics is fast the service attitude is very good.	Jingdong prefecture level city in Shandong
Fortune Shop	White Intelligent Power Saver	The meter is good quality, sturdy and durable. It is accurate. Easy to use. Saves a lot of things.	Jingdong prefecture level city in Shandong
Fortune Shop	White Intelligent Power Saver	Just received the goods, don't know how it is yet	Jingdong prefecture level city in Shandong
Fortune Shop	White Intelligent Power Saver	Energy saver received, logistics is very fast, the quality is very good, the appearance is also very beautiful power saving is the most important.	Jingdong prefecture level city in Shandong
Fortune Shop	White Intelligent Power Saver	It's good to talk about it, it's not practical to use it.	Jingdong prefecture level city in Shandong
Fortune Shop	White Intelligent Power Saver	I don't know if it's good for now, I'll comment after using it	Jingdong prefecture level city in Shandong
Fortune Shop	White Intelligent Power Saver	I've been using it for two or three days. It's not energy efficient.	Jingdong prefecture level city in Shandong
Fortune Shop	White Intelligent Power Saver	Packaging is intact, logistics is also fast, cost-effective, inexpensive, very satisfied with a purchase	Jingdong prefecture level city in Shandong
Fortune Shop	White Intelligent Power Saver	In use to be observed!	Jingdong prefecture level city in Shandong
Fortune Shop	White Intelligent Power Saver	Use it for a while at a flat rate	Jingdong prefecture level city in Shandong
Fortune Shop	White Intelligent Power Saver	Very trustworthy brand, quality is guaranteed, the price is still affordable, quite satisfied!	Jingdong prefecture level city in Shandong
Fortune Shop	White Intelligent Power Saver	Great quality, great operation, easy to install, great price!	Jingdong prefecture level city in Shandong
Fortune Shop	White Intelligent Power Saver	I came back and installed it, it really saves electricity, much more than before, the	Jingdong prefecture level city in Shandong
Fortune Shop	White Intelligent Power Saver	Fast logistics, good quality, very good packaging, good customer service	Jingdong prefecture level city in Shandong
Fortune Shop	White Intelligent Power Saver	Very good quality, especially good, effective, satisfactory	Jingdong prefecture level city in Shandong
Fortune Shop	White Intelligent Power Saver	I don't know how well it works.	Jingdong prefecture level city in Shandong
Fortune Shop	White Intelligent Power Saver	Good value for money, recommend it haha	Jingdong prefecture level city in Shandong
Fortune Shop	White Intelligent Power Saver	Good. Good. Very good.	Jingdong prefecture level city in Shandong
Fortune Shop	White Intelligent Power Saver	It's not working.	Jingdong prefecture level city in Shandong
Fortune Shop	White Intelligent Power Saver	It's not working.	Jingdong prefecture level city in Shandong
Fortune Shop	White Intelligent Power Saver	In use	Jingdong prefecture level city in Shandong
Fortune Shop	White Intelligent Power Saver	I'll try it later.	Jingdong prefecture level city in Shandong
Fortune Shop	White Intelligent Power Saver	It's not much use.	Jingdong prefecture level city in Shandong
Fortune Shop	White Intelligent Power Saver	It doesn't save electricity.	Jingdong prefecture level city in Shandong
Fortune Shop	White Intelligent Power Saver	It's not much use.	Jingdong prefecture level city in Shandong
Fortune Shop	White Intelligent Power Saver	Tried it for a few days and tracked the record, and there is no difference from before.	Jingdong prefecture level city in Shandong
Fortune Shop	White Intelligent Power Saver	It's not very credible, so just do it yourself and turn off the lights diligently!	Jingdong prefecture level city in Shandong
Fortune Shop	White Intelligent Power Saver	Too bad, no power saving at all, scam people don't fall for it!	Jingdong prefecture level city in Shandong
Fortune Shop	White Intelligent Power Saver	It's not working at all.	Jingdong prefecture level city in Shandong
Fortune Shop	White Intelligent Power Saver	general	Jingdong prefecture level city in Shandong
Fortune Shop	White Intelligent Power Saver	(of an unmarried couple) be close	Jingdong prefecture level city in Shandong
Fortune Shop	White Intelligent Power Saver	of no use	Jingdong prefecture level city in Shandong
Fortune Shop	White Intelligent Power Saver	Actually can really save electricity, only used half a month to save more than 50, recommended to the neighbors to use together!	Jingdong prefecture level city in Shandong
Fortune Shop	White Intelligent Power Saver	Save what you can. Isn't it more cost effective to buy one of these than to pay the electric bill?	Jingdong prefecture level city in Shandong
Fortune Shop	White Intelligent Power Saver	Received, a use do not know how, use a month to see the effect is good, the effect is good then recommended, not good then I just do not recommend.	Jingdong prefecture level city in Shandong
Fortune Shop	White Intelligent Power Saver	Just received the goods, is a voltage regulator, next month to evaluate whether it is really power saving	Jingdong prefecture level city in Shandong
Fortune Shop	White Intelligent Power Saver	Very good, used a few days obviously feel that the electricity bill is much less, wait a month to see how much can be saved!	Jingdong prefecture level city in Shandong
Fortune Shop	White Intelligent Power Saver	Received the stuff used for more than a month without a little use, it feels like it uses more electricity than before	Jingdong prefecture level city in Shandong
Fortune Shop	White Intelligent Power Saver	The quality is very good, completely exceeded the expectations, the delivery speed is very fast, the packaging is very careful and strict, very satisfied with a shopping	Jingdong prefecture level city in Shandong
Fortune Shop	White Intelligent Power Saver	Fake, doesn't work at all. Don't even sell it.	Jingdong prefecture level city in Shandong
Fortune Shop	White Intelligent Power Saver	This item doesn't seem to have much effect?	Jingdong prefecture level city in Shandong
Fortune Shop	White Intelligent Power Saver	This production is a bit fake, just look at the focus on Ming	Jingdong prefecture level city in Shandong
Fortune Shop	White Intelligent Power Saver	The logistics is fast the service attitude is very good.	Jingdong prefecture level city in Shandong
Fortune Shop	White Intelligent Power Saver	The meter is good quality, sturdy and durable. It is accurate. Easy to use. Saves a lot of things.	Jingdong prefecture level city in Shandong
Fortune Shop	White Intelligent Power Saver	Just received the goods, don't know how it is yet	Jingdong prefecture level city in Shandong
Fortune Shop	White Intelligent Power Saver	Energy saver received, logistics is very fast, the quality is very good, the appearance is also very beautiful power saving is the most important.	Jingdong prefecture level city in Shandong
Fortune Shop	White Intelligent Power Saver	It's good to talk about it, it's not practical to use it.	Jingdong prefecture level city in Shandong
Fortune Shop	White Intelligent Power Saver	I don't know if it's good for now, I'll comment after using it	Jingdong prefecture level city in Shandong
Fortune Shop	White Intelligent Power Saver	I've been using it for two or three days. It's not energy efficient.	Jingdong prefecture level city in Shandong

Fortune Shop	White Intelligent Power Saver	Packaging is intact, logistics is also fast, cost-effective, inexpensive, very satisfied with a purchase	Jingdong prefecture level city in Shandong
Fortune Shop	White Intelligent Power Saver	In use to be observed!	Jingdong prefecture level city in Shandong
Fortune Shop	White Intelligent Power Saver	Use it for a while at a flat rate	Jingdong prefecture level city in Shandong
Fortune Shop	White Intelligent Power Saver	Very trustworthy brand, quality is guaranteed, the price is still affordable, quite satisfied!	Jingdong prefecture level city in Shandong
Fortune Shop	White Intelligent Power Saver	Great quality, great operation, easy to install, great price!	Jingdong prefecture level city in Shandong
Fortune Shop	White Intelligent Power Saver	I came back and installed it, it really saves electricity, much more than before, the	Jingdong prefecture level city in Shandong
Fortune Shop	White Intelligent Power Saver	Fast logistics, good quality, very good packaging, good customer service	Jingdong prefecture level city in Shandong
Fortune Shop	White Intelligent Power Saver	Very good quality, especially good, effective, satisfactory	Jingdong prefecture level city in Shandong
Fortune Shop	White Intelligent Power Saver	I don't know how well it works.	Jingdong prefecture level city in Shandong
Fortune Shop	White Intelligent Power Saver	Good value for money, recommend it haha	Jingdong prefecture level city in Shandong
Fortune Shop	White Intelligent Power Saver	Good. Good. Very good.	Jingdong prefecture level city in Shandong
Fortune Shop	White Intelligent Power Saver	It's not working.	Jingdong prefecture level city in Shandong
Fortune Shop	White Intelligent Power Saver	It's not working.	Jingdong prefecture level city in Shandong
Fortune Shop	White Intelligent Power Saver	In use	Jingdong prefecture level city in Shandong
Fortune Shop	White Intelligent Power Saver	I'll try it later.	Jingdong prefecture level city in Shandong
Fortune Shop	White Intelligent Power Saver	It's not much use.	Jingdong prefecture level city in Shandong
Fortune Shop	White Intelligent Power Saver	It doesn't save electricity.	Jingdong prefecture level city in Shandong
Fortune Shop	White Intelligent Power Saver	It's not much use.	Jingdong prefecture level city in Shandong
Fortune Shop	White Intelligent Power Saver	Tried it for a few days and tracked the record, and there is no difference from before.	Jingdong prefecture level city in Shandong
Fortune Shop	White Intelligent Power Saver	It's not very credible, so just do it yourself and turn off the lights diligently!	Jingdong prefecture level city in Shandong
Fortune Shop	White Intelligent Power Saver	Too bad, no power saving at all, scam people don't fall for it!	Jingdong prefecture level city in Shandong
Fortune Shop	White Intelligent Power Saver	It's not working at all.	Jingdong prefecture level city in Shandong
Fortune Shop	White Intelligent Power Saver	general	Jingdong prefecture level city in Shandong
Fortune Shop	White Intelligent Power Saver	(of an unmarried couple) be close	Jingdong prefecture level city in Shandong
Fortune Shop	White Intelligent Power Saver	of no use	Jingdong prefecture level city in Shandong
Fortune Shop	White Intelligent Power Saver	Actually can really save electricity, only used half a month to save more than 50, recommended to the neighbors to use together!	Jingdong prefecture level city in Shandong
Fortune Shop	White Intelligent Power Saver	Save what you can. Isn't it more cost effective to buy one of these than to pay the electric bill?	Jingdong prefecture level city in Shandong
Fortune Shop	White Intelligent Power Saver	Received, a use do not know how, use a month to see the effect is good, the effect is good then recommended, not good then I just do not recommend.	Jingdong prefecture level city in Shandong
Fortune Shop	White Intelligent Power Saver	Just received the goods, is a voltage regulator, next month to evaluate whether it is really power saving	Jingdong prefecture level city in Shandong
Fortune Shop	White Intelligent Power Saver	Very good, used a few days obviously feel that the electricity bill is much less, wait a month to see how much can be saved!	Jingdong prefecture level city in Shandong
Fortune Shop	White Intelligent Power Saver	Received the stuff used for more than a month without a little use, it feels like it uses more electricity than before	Jingdong prefecture level city in Shandong
Fortune Shop	White Intelligent Power Saver	The quality is very good, completely exceeded the expectations, the delivery speed is very fast, the packaging is very careful and strict, very satisfied with a shopping	Jingdong prefecture level city in Shandong
Fortune Shop	White Intelligent Power Saver	Fake, doesn't work at all. Don't even sell it.	Jingdong prefecture level city in Shandong
Fortune Shop	White Intelligent Power Saver	This item doesn't seem to have much effect?	Jingdong prefecture level city in Shandong
Fortune Shop	White Intelligent Power Saver	This production is a bit fake, just look at the focus on Ming	Jingdong prefecture level city in Shandong
Fortune Shop	White Intelligent Power Saver	The logistics is fast the service attitude is very good.	Jingdong prefecture level city in Shandong
Fortune Shop	White Intelligent Power Saver	The meter is good quality, sturdy and durable. It is accurate. Easy to use. Saves a lot of things.	Jingdong prefecture level city in Shandong
Fortune Shop	White Intelligent Power Saver	Just received the goods, don't know how it is yet	Jingdong prefecture level city in Shandong
Fortune Shop	White Intelligent Power Saver	Energy saver received, logistics is very fast, the quality is very good, the appearance is also very beautiful power saving is the most important.	Jingdong prefecture level city in Shandong
Fortune Shop	White Intelligent Power Saver	It's good to talk about it, it's not practical to use it.	Jingdong prefecture level city in Shandong
Fortune Shop	White Intelligent Power Saver	I don't know if it's good for now, I'll comment after using it	Jingdong prefecture level city in Shandong
Fortune Shop	White Intelligent Power Saver	I've been using it for two or three days. It's not energy efficient.	Jingdong prefecture level city in Shandong
Fortune Shop	White Intelligent Power Saver	Packaging is intact, logistics is also fast, cost-effective, inexpensive, very satisfied with a purchase	Jingdong prefecture level city in Shandong
Fortune Shop	White Intelligent Power Saver	In use to be observed!	Jingdong prefecture level city in Shandong
Fortune Shop	White Intelligent Power Saver	Use it for a while at a flat rate	Jingdong prefecture level city in Shandong
Fortune Shop	White Intelligent Power Saver	Very trustworthy brand, quality is guaranteed, the price is still affordable, quite satisfied!	Jingdong prefecture level city in Shandong
Fortune Shop	White Intelligent Power Saver	Great quality, great operation, easy to install, great price!	Jingdong prefecture level city in Shandong
Fortune Shop	White Intelligent Power Saver	I came back and installed it, it really saves electricity, much more than before, the	Jingdong prefecture level city in Shandong
Fortune Shop	White Intelligent Power Saver	Fast logistics, good quality, very good packaging, good customer service	Jingdong prefecture level city in Shandong
Fortune Shop	White Intelligent Power Saver	Very good quality, especially good, effective, satisfactory	Jingdong prefecture level city in Shandong
Fortune Shop	White Intelligent Power Saver	I don't know how well it works.	Jingdong prefecture level city in Shandong

Fortune Shop	White Intelligent Power Saver	Good value for money, recommend it haha	Jingdong prefecture level city in Shandong
Fortune Shop	White Intelligent Power Saver	Good. Good. Very good.	Jingdong prefecture level city in Shandong
Fortune Shop	White Intelligent Power Saver	It's not working.	Jingdong prefecture level city in Shandong
Fortune Shop	White Intelligent Power Saver	It's not working.	Jingdong prefecture level city in Shandong
Fortune Shop	White Intelligent Power Saver	In use	Jingdong prefecture level city in Shandong
Fortune Shop	White Intelligent Power Saver	I'll try it later.	Jingdong prefecture level city in Shandong
Fortune Shop	White Intelligent Power Saver	It's not much use.	Jingdong prefecture level city in Shandong
Fortune Shop	White Intelligent Power Saver	It doesn't save electricity.	Jingdong prefecture level city in Shandong
Fortune Shop	White Intelligent Power Saver	It's not much use.	Jingdong prefecture level city in Shandong
Fortune Shop	White Intelligent Power Saver	Tried it for a few days and tracked the record, and there is no difference from before.	Jingdong prefecture level city in Shandong
Fortune Shop	White Intelligent Power Saver	It's not very credible, so just do it yourself and turn off the lights diligently!	Jingdong prefecture level city in Shandong
Fortune Shop	White Intelligent Power Saver	Too bad, no power saving at all, scam people don't fall for it!	Jingdong prefecture level city in Shandong
Fortune Shop	White Intelligent Power Saver	It's not working at all.	Jingdong prefecture level city in Shandong
Fortune Shop	White Intelligent Power Saver	general	Jingdong prefecture level city in Shandong
Fortune Shop	White Intelligent Power Saver	(of an unmarried couple) be close	Jingdong prefecture level city in Shandong
Fortune Shop	White Intelligent Power Saver	of no use	Jingdong prefecture level city in Shandong
Fortune Shop	White Intelligent Power Saver	Actually can really save electricity, only used half a month to save more than 50, recommended to the neighbors to use together!	Jingdong prefecture level city in Shandong
Fortune Shop	White Intelligent Power Saver	Save what you can. Isn't it more cost effective to buy one of these than to pay the electric bill?	Jingdong prefecture level city in Shandong
Fortune Shop	White Intelligent Power Saver	Received, a use do not know how, use a month to see the effect is good, the effect is good then recommended, not good then I just do not recommend.	Jingdong prefecture level city in Shandong
Fortune Shop	White Intelligent Power Saver	Just received the goods, is a voltage regulator, next month to evaluate whether it is really power saving	Jingdong prefecture level city in Shandong
Fortune Shop	White Intelligent Power Saver	Very good, used a few days obviously feel that the electricity bill is much less, wait a month to see how much can be saved!	Jingdong prefecture level city in Shandong
Fortune Shop	White Intelligent Power Saver	Received the stuff used for more than a month without a little use, it feels like it uses more electricity than before	Jingdong prefecture level city in Shandong
Fortune Shop	White Intelligent Power Saver	The quality is very good, completely exceeded the expectations, the delivery speed is very fast, the packaging is very careful and strict, very satisfied with a shopping	Jingdong prefecture level city in Shandong
Fortune Shop	White Intelligent Power Saver	Fake, doesn't work at all. Don't even sell it.	Jingdong prefecture level city in Shandong
Fortune Shop	White Intelligent Power Saver	This item doesn't seem to have much effect?	Jingdong prefecture level city in Shandong
Fortune Shop	White Intelligent Power Saver	This production is a bit fake, just look at the focus on Ming	Jingdong prefecture level city in Shandong
Fortune Shop	White Intelligent Power Saver	The logistics is fast the service attitude is very good.	Jingdong prefecture level city in Shandong
Fortune Shop	White Intelligent Power Saver	The meter is good quality, sturdy and durable. It is accurate. Easy to use. Saves a lot of things.	Jingdong prefecture level city in Shandong
Fortune Shop	White Intelligent Power Saver	Just received the goods, don't know how it is yet	Jingdong prefecture level city in Shandong
Fortune Shop	White Intelligent Power Saver	Energy saver received, logistics is very fast, the quality is very good, the appearance is also very beautiful power saving is the most important.	Jingdong prefecture level city in Shandong
Fortune Shop	White Intelligent Power Saver	It's good to talk about it, it's not practical to use it.	Jingdong prefecture level city in Shandong
Fortune Shop	White Intelligent Power Saver	I don't know if it's good for now, I'll comment after using it	Jingdong prefecture level city in Shandong
Fortune Shop	White Intelligent Power Saver	I've been using it for two or three days. It's not energy efficient.	Jingdong prefecture level city in Shandong
Fortune Shop	White Intelligent Power Saver	Packaging is intact, logistics is also fast, cost-effective, inexpensive, very satisfied with a purchase	Jingdong prefecture level city in Shandong
Fortune Shop	White Intelligent Power Saver	In use to be observed!	Jingdong prefecture level city in Shandong
Fortune Shop	White Intelligent Power Saver	Use it for a while at a flat rate	Jingdong prefecture level city in Shandong
Fortune Shop	White Intelligent Power Saver	Very trustworthy brand, quality is guaranteed, the price is still affordable, quite satisfied!	Jingdong prefecture level city in Shandong
Fortune Shop	White Intelligent Power Saver	Great quality, great operation, easy to install, great price!	Jingdong prefecture level city in Shandong
Fortune Shop	White Intelligent Power Saver	I came back and installed it, it really saves electricity, much more than before, the	Jingdong prefecture level city in Shandong
Fortune Shop	White Intelligent Power Saver	Fast logistics, good quality, very good packaging, good customer service	Jingdong prefecture level city in Shandong
Fortune Shop	White Intelligent Power Saver	Very good quality, especially good, effective, satisfactory	Jingdong prefecture level city in Shandong
Fortune Shop	White Intelligent Power Saver	I don't know how well it works.	Jingdong prefecture level city in Shandong
Fortune Shop	White Intelligent Power Saver	Good value for money, recommend it haha	Jingdong prefecture level city in Shandong
Fortune Shop	White Intelligent Power Saver	Good. Good. Very good.	Jingdong prefecture level city in Shandong
Fortune Shop	White Intelligent Power Saver	It's not working.	Jingdong prefecture level city in Shandong
Fortune Shop	White Intelligent Power Saver	It's not working.	Jingdong prefecture level city in Shandong
Fortune Shop	White Intelligent Power Saver	In use	Jingdong prefecture level city in Shandong
Fortune Shop	White Intelligent Power Saver	I'll try it later.	Jingdong prefecture level city in Shandong
Fortune Shop	White Intelligent Power Saver	It's not much use.	Jingdong prefecture level city in Shandong
Fortune Shop	White Intelligent Power Saver	It doesn't save electricity.	Jingdong prefecture level city in Shandong
Fortune Shop	White Intelligent Power Saver	It's not much use.	Jingdong prefecture level city in Shandong

Fortune Shop	White Intelligent Power Saver	Tried it for a few days and tracked the record, and there is no difference from before.	Jingdong prefecture level city in Shandong
Fortune Shop	White Intelligent Power Saver	It's not very credible, so just do it yourself and turn off the lights diligently!	Jingdong prefecture level city in Shandong
Fortune Shop	White Intelligent Power Saver	Too bad, no power saving at all, scam people don't fall for it!	Jingdong prefecture level city in Shandong
Fortune Shop	White Intelligent Power Saver	It's not working at all.	Jingdong prefecture level city in Shandong
Fortune Shop	White Intelligent Power Saver	general	Jingdong prefecture level city in Shandong
Fortune Shop	White Intelligent Power Saver	(of an unmarried couple) be close	Jingdong prefecture level city in Shandong
Fortune Shop	White Intelligent Power Saver	of no use	Jingdong prefecture level city in Shandong
Fortune Shop	White Intelligent Power Saver	Actually can really save electricity, only used half a month to save more than 50, recommended to the neighbors to use together!	Jingdong prefecture level city in Shandong
Fortune Shop	White Intelligent Power Saver	Save what you can. Isn't it more cost effective to buy one of these than to pay the electric bill?	Jingdong prefecture level city in Shandong
Fortune Shop	White Intelligent Power Saver	Received, a use do not know how, use a month to see the effect is good, the effect is good then recommended, not good then I just do not recommend.	Jingdong prefecture level city in Shandong
Fortune Shop	White Intelligent Power Saver	Just received the goods, is a voltage regulator, next month to evaluate whether it is really power saving	Jingdong prefecture level city in Shandong
Fortune Shop	White Intelligent Power Saver	Very good, used a few days obviously feel that the electricity bill is much less, wait a month to see how much can be saved!	Jingdong prefecture level city in Shandong
Fortune Shop	White Intelligent Power Saver	Received the stuff used for more than a month without a little use, it feels like it uses more electricity than before	Jingdong prefecture level city in Shandong
Fortune Shop	White Intelligent Power Saver	The quality is very good, completely exceeded the expectations, the delivery speed is very fast, the packaging is very careful and strict, very satisfied with a shopping	Jingdong prefecture level city in Shandong
Fortune Shop	White Intelligent Power Saver	Fake, doesn't work at all. Don't even sell it.	Jingdong prefecture level city in Shandong
Fortune Shop	White Intelligent Power Saver	This item doesn't seem to have much effect?	Jingdong prefecture level city in Shandong
Fortune Shop	White Intelligent Power Saver	This production is a bit fake, just look at the focus on Ming	Jingdong prefecture level city in Shandong
Fortune Shop	White Intelligent Power Saver	The logistics is fast the service attitude is very good.	Jingdong prefecture level city in Shandong
Fortune Shop	White Intelligent Power Saver	The meter is good quality, sturdy and durable. It is accurate. Easy to use. Saves a lot of things.	Jingdong prefecture level city in Shandong
Fortune Shop	White Intelligent Power Saver	Just received the goods, don't know how it is yet	Jingdong prefecture level city in Shandong
Fortune Shop	White Intelligent Power Saver	Energy saver received, logistics is very fast, the quality is very good, the appearance is also very beautiful power saving is the most important.	Jingdong prefecture level city in Shandong
Fortune Shop	White Intelligent Power Saver	It's good to talk about it, it's not practical to use it.	Jingdong prefecture level city in Shandong
Fortune Shop	White Intelligent Power Saver	I don't know if it's good for now, I'll comment after using it	Jingdong prefecture level city in Shandong
Fortune Shop	White Intelligent Power Saver	I've been using it for two or three days. It's not energy efficient.	Jingdong prefecture level city in Shandong
Fortune Shop	White Intelligent Power Saver	Packaging is intact, logistics is also fast, cost-effective, inexpensive, very satisfied with a purchase	Jingdong prefecture level city in Shandong
Fortune Shop	White Intelligent Power Saver	In use to be observed!	Jingdong prefecture level city in Shandong
Fortune Shop	White Intelligent Power Saver	Use it for a while at a flat rate	Jingdong prefecture level city in Shandong
Fortune Shop	White Intelligent Power Saver	Very trustworthy brand, quality is guaranteed, the price is still affordable, quite satisfied!	Jingdong prefecture level city in Shandong
Fortune Shop	White Intelligent Power Saver	Great quality, great operation, easy to install, great price!	Jingdong prefecture level city in Shandong
Fortune Shop	White Intelligent Power Saver	I came back and installed it, it really saves electricity, much more than before, the	Jingdong prefecture level city in Shandong
Fortune Shop	White Intelligent Power Saver	Fast logistics, good quality, very good packaging, good customer service	Jingdong prefecture level city in Shandong
Fortune Shop	White Intelligent Power Saver	Very good quality, especially good, effective, satisfactory	Jingdong prefecture level city in Shandong
Fortune Shop	White Intelligent Power Saver	I don't know how well it works.	Jingdong prefecture level city in Shandong
Fortune Shop	White Intelligent Power Saver	Good value for money, recommend it haha	Jingdong prefecture level city in Shandong
Fortune Shop	White Intelligent Power Saver	Good. Good. Very good.	Jingdong prefecture level city in Shandong
Fortune Shop	White Intelligent Power Saver	It's not working.	Jingdong prefecture level city in Shandong
Fortune Shop	White Intelligent Power Saver	It's not working.	Jingdong prefecture level city in Shandong
Fortune Shop	White Intelligent Power Saver	In use	Jingdong prefecture level city in Shandong
Fortune Shop	White Intelligent Power Saver	I'll try it later.	Jingdong prefecture level city in Shandong
Fortune Shop	White Intelligent Power Saver	It's not much use.	Jingdong prefecture level city in Shandong
Fortune Shop	White Intelligent Power Saver	It doesn't save electricity.	Jingdong prefecture level city in Shandong
Fortune Shop	White Intelligent Power Saver	It's not much use.	Jingdong prefecture level city in Shandong
Fortune Shop	White Intelligent Power Saver	Tried it for a few days and tracked the record, and there is no difference from before.	Jingdong prefecture level city in Shandong
Fortune Shop	White Intelligent Power Saver	It's not very credible, so just do it yourself and turn off the lights diligently!	Jingdong prefecture level city in Shandong
Fortune Shop	White Intelligent Power Saver	Too bad, no power saving at all, scam people don't fall for it!	Jingdong prefecture level city in Shandong
Fortune Shop	White Intelligent Power Saver	It's not working at all.	Jingdong prefecture level city in Shandong
Fortune Shop	White Intelligent Power Saver	general	Jingdong prefecture level city in Shandong
Fortune Shop	White Intelligent Power Saver	(of an unmarried couple) be close	Jingdong prefecture level city in Shandong
Fortune Shop	White Intelligent Power Saver	of no use	Jingdong prefecture level city in Shandong
Fortune Shop	White Intelligent Power Saver	Actually can really save electricity, only used half a month to save more than 50, recommended to the neighbors to use together!	Jingdong prefecture level city in Shandong
Fortune Shop	White Intelligent Power Saver	Save what you can. Isn't it more cost effective to buy one of these than to pay the electric bill?	Jingdong prefecture level city in Shandong

Fortune Shop	White Intelligent Power Saver	Received, a use do not know how, use a month to see the effect is good, the effect is good then recommended, not good then I just do not recommend.	Jingdong prefecture level city in Shandong
Fortune Shop	White Intelligent Power Saver	Just received the goods, is a voltage regulator, next month to evaluate whether it is really power saving	Jingdong prefecture level city in Shandong
Fortune Shop	White Intelligent Power Saver	Very good, used a few days obviously feel that the electricity bill is much less, wait a month to see how much can be saved!	Jingdong prefecture level city in Shandong
Fortune Shop	White Intelligent Power Saver	Received the stuff used for more than a month without a little use, it feels like it uses more electricity than before	Jingdong prefecture level city in Shandong
Fortune Shop	White Intelligent Power Saver	The quality is very good, completely exceeded the expectations, the delivery speed is very fast, the packaging is very careful and strict, very satisfied with a shopping	Jingdong prefecture level city in Shandong
Fortune Shop	White Intelligent Power Saver	Fake, doesn't work at all. Don't even sell it.	Jingdong prefecture level city in Shandong
Fortune Shop	White Intelligent Power Saver	This item doesn't seem to have much effect?	Jingdong prefecture level city in Shandong
Fortune Shop	White Intelligent Power Saver	This production is a bit fake, just look at the focus on Ming	Jingdong prefecture level city in Shandong
Fortune Shop	White Intelligent Power Saver	The logistics is fast the service attitude is very good.	Jingdong prefecture level city in Shandong
Fortune Shop	White Intelligent Power Saver	The meter is good quality, sturdy and durable. It is accurate. Easy to use. Saves a lot of things.	Jingdong prefecture level city in Shandong
Fortune Shop	White Intelligent Power Saver	Just received the goods, don't know how it is yet	Jingdong prefecture level city in Shandong
Fortune Shop	White Intelligent Power Saver	Energy saver received, logistics is very fast, the quality is very good, the appearance is also very beautiful power saving is the most important.	Jingdong prefecture level city in Shandong
Fortune Shop	White Intelligent Power Saver	It's good to talk about it, it's not practical to use it.	Jingdong prefecture level city in Shandong
Fortune Shop	White Intelligent Power Saver	I don't know if it's good for now, I'll comment after using it	Jingdong prefecture level city in Shandong
Fortune Shop	White Intelligent Power Saver	I've been using it for two or three days. It's not energy efficient.	Jingdong prefecture level city in Shandong
Fortune Shop	White Intelligent Power Saver	Packaging is intact, logistics is also fast, cost-effective, inexpensive, very satisfied with a purchase	Jingdong prefecture level city in Shandong
Fortune Shop	White Intelligent Power Saver	In use to be observed!	Jingdong prefecture level city in Shandong
Fortune Shop	White Intelligent Power Saver	Use it for a while at a flat rate	Jingdong prefecture level city in Shandong
Fortune Shop	White Intelligent Power Saver	Very trustworthy brand, quality is guaranteed, the price is still affordable, quite satisfied!	Jingdong prefecture level city in Shandong
Fortune Shop	White Intelligent Power Saver	Great quality, great operation, easy to install, great price!	Jingdong prefecture level city in Shandong
Fortune Shop	White Intelligent Power Saver	I came back and installed it, it really saves electricity, much more than before, the	Jingdong prefecture level city in Shandong
Fortune Shop	White Intelligent Power Saver	Fast logistics, good quality, very good packaging, good customer service	Jingdong prefecture level city in Shandong
Fortune Shop	White Intelligent Power Saver	Very good quality, especially good, effective, satisfactory	Jingdong prefecture level city in Shandong
Fortune Shop	White Intelligent Power Saver	I don't know how well it works.	Jingdong prefecture level city in Shandong
Fortune Shop	White Intelligent Power Saver	Good value for money, recommend it haha	Jingdong prefecture level city in Shandong
Fortune Shop	White Intelligent Power Saver	Good. Good. Very good.	Jingdong prefecture level city in Shandong
Fortune Shop	White Intelligent Power Saver	It's not working.	Jingdong prefecture level city in Shandong
Fortune Shop	White Intelligent Power Saver	It's not working.	Jingdong prefecture level city in Shandong
Fortune Shop	White Intelligent Power Saver	In use	Jingdong prefecture level city in Shandong
Fortune Shop	White Intelligent Power Saver	I'll try it later.	Jingdong prefecture level city in Shandong
Fortune Shop	White Intelligent Power Saver	It's not much use.	Jingdong prefecture level city in Shandong
Fortune Shop	White Intelligent Power Saver	It doesn't save electricity.	Jingdong prefecture level city in Shandong
Fortune Shop	White Intelligent Power Saver	It's not much use.	Jingdong prefecture level city in Shandong
Fortune Shop	White Intelligent Power Saver	Tried it for a few days and tracked the record, and there is no difference from before.	Jingdong prefecture level city in Shandong
Fortune Shop	White Intelligent Power Saver	It's not very credible, so just do it yourself and turn off the lights diligently!	Jingdong prefecture level city in Shandong
Fortune Shop	White Intelligent Power Saver	Too bad, no power saving at all, scam people don't fall for it!	Jingdong prefecture level city in Shandong
Fortune Shop	White Intelligent Power Saver	It's not working at all.	Jingdong prefecture level city in Shandong
Fortune Shop	White Intelligent Power Saver	general	Jingdong prefecture level city in Shandong
Fortune Shop	White Intelligent Power Saver	(of an unmarried couple) be close	Jingdong prefecture level city in Shandong
Fortune Shop	White Intelligent Power Saver	of no use	Jingdong prefecture level city in Shandong
Fortune Shop	White Intelligent Power Saver	Actually can really save electricity, only used half a month to save more than 50, recommended to the neighbors to use together!	Jingdong prefecture level city in Shandong
Fortune Shop	White Intelligent Power Saver	Save what you can. Isn't it more cost effective to buy one of these than to pay the electric bill?	Jingdong prefecture level city in Shandong
Fortune Shop	White Intelligent Power Saver	Received, a use do not know how, use a month to see the effect is good, the effect is good then recommended, not good then I just do not recommend.	Jingdong prefecture level city in Shandong
Fortune Shop	White Intelligent Power Saver	Just received the goods, is a voltage regulator, next month to evaluate whether it is really power saving	Jingdong prefecture level city in Shandong
Fortune Shop	White Intelligent Power Saver	Very good, used a few days obviously feel that the electricity bill is much less, wait a month to see how much can be saved!	Jingdong prefecture level city in Shandong
Fortune Shop	White Intelligent Power Saver	Received the stuff used for more than a month without a little use, it feels like it uses more electricity than before	Jingdong prefecture level city in Shandong
Fortune Shop	White Intelligent Power Saver	The quality is very good, completely exceeded the expectations, the delivery speed is very fast, the packaging is very careful and strict, very satisfied with a shopping	Jingdong prefecture level city in Shandong
Fortune Shop	White Intelligent Power Saver	Fake, doesn't work at all. Don't even sell it.	Jingdong prefecture level city in Shandong
Fortune Shop	White Intelligent Power Saver	This item doesn't seem to have much effect?	Jingdong prefecture level city in Shandong
Fortune Shop	White Intelligent Power Saver	This production is a bit fake, just look at the focus on Ming	Jingdong prefecture level city in Shandong
Fortune Shop	White Intelligent Power Saver	The logistics is fast the service attitude is very good.	Jingdong prefecture level city in Shandong

Fortune Shop	White Intelligent Power Saver	The meter is good quality, sturdy and durable. It is accurate. Easy to use. Saves a lot of things.	Jingdong prefecture level city in Shandong
Fortune Shop	White Intelligent Power Saver	Just received the goods, don't know how it is yet	Jingdong prefecture level city in Shandong
Fortune Shop	White Intelligent Power Saver	Energy saver received, logistics is very fast, the quality is very good, the appearance is also very beautiful power saving is the most important.	Jingdong prefecture level city in Shandong
Fortune Shop	White Intelligent Power Saver	It's good to talk about it, it's not practical to use it.	Jingdong prefecture level city in Shandong
Fortune Shop	White Intelligent Power Saver	I don't know if it's good for now, I'll comment after using it	Jingdong prefecture level city in Shandong
Fortune Shop	White Intelligent Power Saver	I've been using it for two or three days. It's not energy efficient.	Jingdong prefecture level city in Shandong
Fortune Shop	White Intelligent Power Saver	Packaging is intact, logistics is also fast, cost-effective, inexpensive, very satisfied with a purchase	Jingdong prefecture level city in Shandong
Fortune Shop	White Intelligent Power Saver	In use to be observed!	Jingdong prefecture level city in Shandong
Fortune Shop	White Intelligent Power Saver	Use it for a while at a flat rate	Jingdong prefecture level city in Shandong
Fortune Shop	White Intelligent Power Saver	Very trustworthy brand, quality is guaranteed, the price is still affordable, quite satisfied!	Jingdong prefecture level city in Shandong
Fortune Shop	White Intelligent Power Saver	Great quality, great operation, easy to install, great price!	Jingdong prefecture level city in Shandong
Fortune Shop	White Intelligent Power Saver	I came back and installed it, it really saves electricity, much more than before, the	Jingdong prefecture level city in Shandong
Fortune Shop	White Intelligent Power Saver	Fast logistics, good quality, very good packaging, good customer service	Jingdong prefecture level city in Shandong
Fortune Shop	White Intelligent Power Saver	Very good quality, especially good, effective, satisfactory	Jingdong prefecture level city in Shandong
Fortune Shop	White Intelligent Power Saver	I don't know how well it works.	Jingdong prefecture level city in Shandong
Fortune Shop	White Intelligent Power Saver	Good value for money, recommend it haha	Jingdong prefecture level city in Shandong
Fortune Shop	White Intelligent Power Saver	Good. Good. Very good.	Jingdong prefecture level city in Shandong
Fortune Shop	White Intelligent Power Saver	It's not working.	Jingdong prefecture level city in Shandong
Fortune Shop	White Intelligent Power Saver	It's not working.	Jingdong prefecture level city in Shandong
Fortune Shop	White Intelligent Power Saver	In use	Jingdong prefecture level city in Shandong
Fortune Shop	White Intelligent Power Saver	I'll try it later.	Jingdong prefecture level city in Shandong
Fortune Shop	White Intelligent Power Saver	It's not much use.	Jingdong prefecture level city in Shandong
Fortune Shop	White Intelligent Power Saver	It doesn't save electricity.	Jingdong prefecture level city in Shandong
Fortune Shop	White Intelligent Power Saver	It's not much use.	Jingdong prefecture level city in Shandong
Fortune Shop	White Intelligent Power Saver	Tried it for a few days and tracked the record, and there is no difference from before.	Jingdong prefecture level city in Shandong
Fortune Shop	White Intelligent Power Saver	It's not very credible, so just do it yourself and turn off the lights diligently!	Jingdong prefecture level city in Shandong
Fortune Shop	White Intelligent Power Saver	Too bad, no power saving at all, scam people don't fall for it!	Jingdong prefecture level city in Shandong
Fortune Shop	White Intelligent Power Saver	It's not working at all.	Jingdong prefecture level city in Shandong
Fortune Shop	White Intelligent Power Saver	general	Jingdong prefecture level city in Shandong
Fortune Shop	White Intelligent Power Saver	(of an unmarried couple) be close	Jingdong prefecture level city in Shandong
Fortune Shop	White Intelligent Power Saver	of no use	Jingdong prefecture level city in Shandong
Fortune Shop	White Intelligent Power Saver	Actually can really save electricity, only used half a month to save more than 50, recommended to the neighbors to use together!	Jingdong prefecture level city in Shandong
Fortune Shop	White Intelligent Power Saver	Save what you can. Isn't it more cost effective to buy one of these than to pay the electric bill?	Jingdong prefecture level city in Shandong
Fortune Shop	White Intelligent Power Saver	Received, a use do not know how, use a month to see the effect is good, the effect is good then recommended, not good then I just do not recommend.	Jingdong prefecture level city in Shandong
Fortune Shop	White Intelligent Power Saver	Just received the goods, is a voltage regulator, next month to evaluate whether it is really power saving	Jingdong prefecture level city in Shandong
Fortune Shop	White Intelligent Power Saver	Very good, used a few days obviously feel that the electricity bill is much less, wait a month to see how much can be saved!	Jingdong prefecture level city in Shandong
Fortune Shop	White Intelligent Power Saver	Received the stuff used for more than a month without a little use, it feels like it uses more electricity than before	Jingdong prefecture level city in Shandong
Fortune Shop	White Intelligent Power Saver	The quality is very good, completely exceeded the expectations, the delivery speed is very fast, the packaging is very careful and strict, very satisfied with a shopping	Jingdong prefecture level city in Shandong
Fortune Shop	White Intelligent Power Saver	Fake, doesn't work at all. Don't even sell it.	Jingdong prefecture level city in Shandong
Fortune Shop	White Intelligent Power Saver	This item doesn't seem to have much effect?	Jingdong prefecture level city in Shandong
Fortune Shop	White Intelligent Power Saver	This production is a bit fake, just look at the focus on Ming	Jingdong prefecture level city in Shandong
Fortune Shop	White Intelligent Power Saver	The logistics is fast the service attitude is very good.	Jingdong prefecture level city in Shandong
Fortune Shop	White Intelligent Power Saver	The meter is good quality, sturdy and durable. It is accurate. Easy to use. Saves a lot of things.	Jingdong prefecture level city in Shandong
Fortune Shop	White Intelligent Power Saver	Just received the goods, don't know how it is yet	Jingdong prefecture level city in Shandong
Fortune Shop	White Intelligent Power Saver	Energy saver received, logistics is very fast, the quality is very good, the appearance is also very beautiful power saving is the most important.	Jingdong prefecture level city in Shandong
Fortune Shop	White Intelligent Power Saver	It's good to talk about it, it's not practical to use it.	Jingdong prefecture level city in Shandong
Fortune Shop	White Intelligent Power Saver	I don't know if it's good for now, I'll comment after using it	Jingdong prefecture level city in Shandong
Fortune Shop	White Intelligent Power Saver	I've been using it for two or three days. It's not energy efficient.	Jingdong prefecture level city in Shandong
Fortune Shop	White Intelligent Power Saver	Packaging is intact, logistics is also fast, cost-effective, inexpensive, very satisfied with a purchase	Jingdong prefecture level city in Shandong
Fortune Shop	White Intelligent Power Saver	In use to be observed!	Jingdong prefecture level city in Shandong
Fortune Shop	White Intelligent Power Saver	Use it for a while at a flat rate	Jingdong prefecture level city in Shandong

Fortune Shop	White Intelligent Power Saver	Very trustworthy brand, quality is guaranteed, the price is still affordable, quite satisfied!	Jingdong prefecture level city in Shandong
Fortune Shop	White Intelligent Power Saver	Great quality, great operation, easy to install, great price!	Jingdong prefecture level city in Shandong
Fortune Shop	White Intelligent Power Saver	I came back and installed it, it really saves electricity, much more than before, the	Jingdong prefecture level city in Shandong
Fortune Shop	White Intelligent Power Saver	Fast logistics, good quality, very good packaging, good customer service	Jingdong prefecture level city in Shandong
Fortune Shop	White Intelligent Power Saver	Very good quality, especially good, effective, satisfactory	Jingdong prefecture level city in Shandong
Fortune Shop	White Intelligent Power Saver	I don't know how well it works.	Jingdong prefecture level city in Shandong
Fortune Shop	White Intelligent Power Saver	Good value for money, recommend it haha	Jingdong prefecture level city in Shandong
Fortune Shop	White Intelligent Power Saver	Good. Good. Very good.	Jingdong prefecture level city in Shandong
Fortune Shop	White Intelligent Power Saver	It's not working.	Jingdong prefecture level city in Shandong
Fortune Shop	White Intelligent Power Saver	It's not working.	Jingdong prefecture level city in Shandong
Fortune Shop	White Intelligent Power Saver	In use	Jingdong prefecture level city in Shandong
Fortune Shop	White Intelligent Power Saver	I'll try it later.	Jingdong prefecture level city in Shandong
Fortune Shop	White Intelligent Power Saver	It's not much use.	Jingdong prefecture level city in Shandong
Fortune Shop	White Intelligent Power Saver	It doesn't save electricity.	Jingdong prefecture level city in Shandong
Fortune Shop	White Intelligent Power Saver	It's not much use.	Jingdong prefecture level city in Shandong
Fortune Shop	White Intelligent Power Saver	Tried it for a few days and tracked the record, and there is no difference from before.	Jingdong prefecture level city in Shandong
Fortune Shop	White Intelligent Power Saver	It's not very credible, so just do it yourself and turn off the lights diligently!	Jingdong prefecture level city in Shandong
Fortune Shop	White Intelligent Power Saver	Too bad, no power saving at all, scam people don't fall for it!	Jingdong prefecture level city in Shandong
Fortune Shop	White Intelligent Power Saver	It's not working at all.	Jingdong prefecture level city in Shandong
Fortune Shop	White Intelligent Power Saver	general	Jingdong prefecture level city in Shandong
Fortune Shop	White Intelligent Power Saver	(of an unmarried couple) be close	Jingdong prefecture level city in Shandong
Fortune Shop	White Intelligent Power Saver	of no use	Jingdong prefecture level city in Shandong
Fortune Shop	White Intelligent Power Saver	Actually can really save electricity, only used half a month to save more than 50, recommended to the neighbors to use together!	Jingdong prefecture level city in Shandong
Fortune Shop	White Intelligent Power Saver	Save what you can. Isn't it more cost effective to buy one of these than to pay the electric bill?	Jingdong prefecture level city in Shandong
Fortune Shop	White Intelligent Power Saver	Received, a use do not know how, use a month to see the effect is good, the effect is good then recommended, not good then I just do not recommend.	Jingdong prefecture level city in Shandong
Fortune Shop	White Intelligent Power Saver	Just received the goods, is a voltage regulator, next month to evaluate whether it is really power saving	Jingdong prefecture level city in Shandong
Fortune Shop	White Intelligent Power Saver	Very good, used a few days obviously feel that the electricity bill is much less, wait a month to see how much can be saved!	Jingdong prefecture level city in Shandong
Fortune Shop	White Intelligent Power Saver	Received the stuff used for more than a month without a little use, it feels like it uses more electricity than before	Jingdong prefecture level city in Shandong
Fortune Shop	White Intelligent Power Saver	The quality is very good, completely exceeded the expectations, the delivery speed is very fast, the packaging is very careful and strict, very satisfied with a shopping	Jingdong prefecture level city in Shandong
Fortune Shop	White Intelligent Power Saver	Fake, doesn't work at all. Don't even sell it.	Jingdong prefecture level city in Shandong
Fortune Shop	White Intelligent Power Saver	This item doesn't seem to have much effect?	Jingdong prefecture level city in Shandong
Fortune Shop	White Intelligent Power Saver	This production is a bit fake, just look at the focus on Ming	Jingdong prefecture level city in Shandong
Fortune Shop	White Intelligent Power Saver	The logistics is fast the service attitude is very good.	Jingdong prefecture level city in Shandong
Fortune Shop	White Intelligent Power Saver	The meter is good quality, sturdy and durable. It is accurate. Easy to use. Saves a lot of things.	Jingdong prefecture level city in Shandong
Fortune Shop	White Intelligent Power Saver	Just received the goods, don't know how it is yet	Jingdong prefecture level city in Shandong
Fortune Shop	White Intelligent Power Saver	Energy saver received, logistics is very fast, the quality is very good, the appearance is also very beautiful power saving is the most important.	Jingdong prefecture level city in Shandong
Fortune Shop	White Intelligent Power Saver	It's good to talk about it, it's not practical to use it.	Jingdong prefecture level city in Shandong
Fortune Shop	White Intelligent Power Saver	I don't know if it's good for now, I'll comment after using it	Jingdong prefecture level city in Shandong
Fortune Shop	White Intelligent Power Saver	I've been using it for two or three days. It's not energy efficient.	Jingdong prefecture level city in Shandong
Fortune Shop	White Intelligent Power Saver	Packaging is intact, logistics is also fast, cost-effective, inexpensive, very satisfied with a purchase	Jingdong prefecture level city in Shandong
Fortune Shop	White Intelligent Power Saver	In use to be observed!	Jingdong prefecture level city in Shandong
Fortune Shop	White Intelligent Power Saver	Use it for a while at a flat rate	Jingdong prefecture level city in Shandong
Fortune Shop	White Intelligent Power Saver	Very trustworthy brand, quality is guaranteed, the price is still affordable, quite satisfied!	Jingdong prefecture level city in Shandong
Fortune Shop	White Intelligent Power Saver	Great quality, great operation, easy to install, great price!	Jingdong prefecture level city in Shandong
Fortune Shop	White Intelligent Power Saver	I came back and installed it, it really saves electricity, much more than before, the	Jingdong prefecture level city in Shandong
Fortune Shop	White Intelligent Power Saver	Fast logistics, good quality, very good packaging, good customer service	Jingdong prefecture level city in Shandong
Fortune Shop	White Intelligent Power Saver	Very good quality, especially good, effective, satisfactory	Jingdong prefecture level city in Shandong
Fortune Shop	White Intelligent Power Saver	I don't know how well it works.	Jingdong prefecture level city in Shandong
Fortune Shop	White Intelligent Power Saver	Good value for money, recommend it haha	Jingdong prefecture level city in Shandong
Fortune Shop	White Intelligent Power Saver	Good. Good. Very good.	Jingdong prefecture level city in Shandong
Fortune Shop	White Intelligent Power Saver	It's not working.	Jingdong prefecture level city in Shandong

Fortune Shop	White Intelligent Power Saver	It's not working.	Jingdong prefecture level city in Shandong
Fortune Shop	White Intelligent Power Saver	In use	Jingdong prefecture level city in Shandong
Fortune Shop	White Intelligent Power Saver	I'll try it later.	Jingdong prefecture level city in Shandong
Fortune Shop	White Intelligent Power Saver	It's not much use.	Jingdong prefecture level city in Shandong
Fortune Shop	White Intelligent Power Saver	It doesn't save electricity.	Jingdong prefecture level city in Shandong
Fortune Shop	White Intelligent Power Saver	It's not much use.	Jingdong prefecture level city in Shandong
Fortune Shop	White Intelligent Power Saver	Tried it for a few days and tracked the record, and there is no difference from before.	Jingdong prefecture level city in Shandong
Fortune Shop	White Intelligent Power Saver	It's not very credible, so just do it yourself and turn off the lights diligently!	Jingdong prefecture level city in Shandong
Fortune Shop	White Intelligent Power Saver	Too bad, no power saving at all, scam people don't fall for it!	Jingdong prefecture level city in Shandong
Fortune Shop	White Intelligent Power Saver	It's not working at all.	Jingdong prefecture level city in Shandong
Fortune Shop	White Intelligent Power Saver	general	Jingdong prefecture level city in Shandong
Fortune Shop	White Intelligent Power Saver	(of an unmarried couple) be close	Jingdong prefecture level city in Shandong
Fortune Shop	White Intelligent Power Saver	of no use	Jingdong prefecture level city in Shandong
Fortune Shop	White Intelligent Power Saver	Actually can really save electricity, only used half a month to save more than 50, recommended to the neighbors to use together!	Jingdong prefecture level city in Shandong
Fortune Shop	White Intelligent Power Saver	Save what you can. Isn't it more cost effective to buy one of these than to pay the electric bill?	Jingdong prefecture level city in Shandong
Fortune Shop	White Intelligent Power Saver	Received, a use do not know how, use a month to see the effect is good, the effect is good then recommended, not good then I just do not recommend.	Jingdong prefecture level city in Shandong
Fortune Shop	White Intelligent Power Saver	Just received the goods, is a voltage regulator, next month to evaluate whether it is really power saving	Jingdong prefecture level city in Shandong
Fortune Shop	White Intelligent Power Saver	Very good, used a few days obviously feel that the electricity bill is much less, wait a month to see how much can be saved!	Jingdong prefecture level city in Shandong
Fortune Shop	White Intelligent Power Saver	Received the stuff used for more than a month without a little use, it feels like it uses more electricity than before	Jingdong prefecture level city in Shandong
Fortune Shop	White Intelligent Power Saver	The quality is very good, completely exceeded the expectations, the delivery speed is very fast, the packaging is very careful and strict, very satisfied with a shopping	Jingdong prefecture level city in Shandong
Fortune Shop	White Intelligent Power Saver	Fake, doesn't work at all. Don't even sell it.	Jingdong prefecture level city in Shandong
Fortune Shop	White Intelligent Power Saver	This item doesn't seem to have much effect?	Jingdong prefecture level city in Shandong
Fortune Shop	White Intelligent Power Saver	This production is a bit fake, just look at the focus on Ming	Jingdong prefecture level city in Shandong
Fortune Shop	White Intelligent Power Saver	The logistics is fast the service attitude is very good.	Jingdong prefecture level city in Shandong
Fortune Shop	White Intelligent Power Saver	The meter is good quality, sturdy and durable. It is accurate. Easy to use. Saves a lot of things.	Jingdong prefecture level city in Shandong
Fortune Shop	White Intelligent Power Saver	Just received the goods, don't know how it is yet	Jingdong prefecture level city in Shandong
Fortune Shop	White Intelligent Power Saver	Energy saver received, logistics is very fast, the quality is very good, the appearance is also very beautiful power saving is the most important.	Jingdong prefecture level city in Shandong
Fortune Shop	White Intelligent Power Saver	It's good to talk about it, it's not practical to use it.	Jingdong prefecture level city in Shandong
Fortune Shop	White Intelligent Power Saver	I don't know if it's good for now, I'll comment after using it	Jingdong prefecture level city in Shandong
Fortune Shop	White Intelligent Power Saver	I've been using it for two or three days. It's not energy efficient.	Jingdong prefecture level city in Shandong
Fortune Shop	White Intelligent Power Saver	Packaging is intact, logistics is also fast, cost-effective, inexpensive, very satisfied with a purchase	Jingdong prefecture level city in Shandong
Fortune Shop	White Intelligent Power Saver	In use to be observed!	Jingdong prefecture level city in Shandong
Fortune Shop	White Intelligent Power Saver	Use it for a while at a flat rate	Jingdong prefecture level city in Shandong
Fortune Shop	White Intelligent Power Saver	Very trustworthy brand, quality is guaranteed, the price is still affordable, quite satisfied!	Jingdong prefecture level city in Shandong
Fortune Shop	White Intelligent Power Saver	Great quality, great operation, easy to install, great price!	Jingdong prefecture level city in Shandong
Fortune Shop	White Intelligent Power Saver	I came back and installed it, it really saves electricity, much more than before, the	Jingdong prefecture level city in Shandong
Fortune Shop	White Intelligent Power Saver	Fast logistics, good quality, very good packaging, good customer service	Jingdong prefecture level city in Shandong
Fortune Shop	White Intelligent Power Saver	Very good quality, especially good, effective, satisfactory	Jingdong prefecture level city in Shandong
Fortune Shop	White Intelligent Power Saver	I don't know how well it works.	Jingdong prefecture level city in Shandong
Fortune Shop	White Intelligent Power Saver	Good value for money, recommend it haha	Jingdong prefecture level city in Shandong
Fortune Shop	White Intelligent Power Saver	Good. Good. Very good.	Jingdong prefecture level city in Shandong
Fortune Shop	White Intelligent Power Saver	It's not working.	Jingdong prefecture level city in Shandong
Fortune Shop	White Intelligent Power Saver	It's not working.	Jingdong prefecture level city in Shandong
Fortune Shop	White Intelligent Power Saver	In use	Jingdong prefecture level city in Shandong
Fortune Shop	White Intelligent Power Saver	I'll try it later.	Jingdong prefecture level city in Shandong
Fortune Shop	White Intelligent Power Saver	It's not much use.	Jingdong prefecture level city in Shandong
Fortune Shop	White Intelligent Power Saver	It doesn't save electricity.	Jingdong prefecture level city in Shandong
Fortune Shop	White Intelligent Power Saver	It's not much use.	Jingdong prefecture level city in Shandong
Fortune Shop	White Intelligent Power Saver	Tried it for a few days and tracked the record, and there is no difference from before.	Jingdong prefecture level city in Shandong
Fortune Shop	White Intelligent Power Saver	It's not very credible, so just do it yourself and turn off the lights diligently!	Jingdong prefecture level city in Shandong
Fortune Shop	White Intelligent Power Saver	Too bad, no power saving at all, scam people don't fall for it!	Jingdong prefecture level city in Shandong

Fortune Shop	White Intelligent Power Saver	It's not working at all.	Jingdong prefecture level city in Shandong
Fortune Shop	White Intelligent Power Saver	general	Jingdong prefecture level city in Shandong
Fortune Shop	White Intelligent Power Saver	(of an unmarried couple) be close	Jingdong prefecture level city in Shandong
Fortune Shop	White Intelligent Power Saver	of no use	Jingdong prefecture level city in Shandong
Fortune Shop	White Intelligent Power Saver	Actually can really save electricity, only used half a month to save more than 50, recommended to the neighbors to use together!	Jingdong prefecture level city in Shandong
Fortune Shop	White Intelligent Power Saver	Save what you can. Isn't it more cost effective to buy one of these than to pay the electric bill?	Jingdong prefecture level city in Shandong
Fortune Shop	White Intelligent Power Saver	Received, a use do not know how, use a month to see the effect is good, the effect is good then recommended, not good then I just do not recommend.	Jingdong prefecture level city in Shandong
Fortune Shop	White Intelligent Power Saver	Just received the goods, is a voltage regulator, next month to evaluate whether it is really power saving	Jingdong prefecture level city in Shandong
Fortune Shop	White Intelligent Power Saver	Very good, used a few days obviously feel that the electricity bill is much less, wait a month to see how much can be saved!	Jingdong prefecture level city in Shandong
Fortune Shop	White Intelligent Power Saver	Received the stuff used for more than a month without a little use, it feels like it uses more electricity than before	Jingdong prefecture level city in Shandong
Fortune Shop	White Intelligent Power Saver	The quality is very good, completely exceeded the expectations, the delivery speed is very fast, the packaging is very careful and strict, very satisfied with a shopping	Jingdong prefecture level city in Shandong
Fortune Shop	White Intelligent Power Saver	Fake, doesn't work at all. Don't even sell it.	Jingdong prefecture level city in Shandong
Fortune Shop	White Intelligent Power Saver	This item doesn't seem to have much effect?	Jingdong prefecture level city in Shandong
Fortune Shop	White Intelligent Power Saver	This production is a bit fake, just look at the focus on Ming	Jingdong prefecture level city in Shandong
Fortune Shop	White Intelligent Power Saver	The logistics is fast the service attitude is very good.	Jingdong prefecture level city in Shandong
Fortune Shop	White Intelligent Power Saver	The meter is good quality, sturdy and durable. It is accurate. Easy to use. Saves a lot of things.	Jingdong prefecture level city in Shandong
Fortune Shop	White Intelligent Power Saver	Just received the goods, don't know how it is yet	Jingdong prefecture level city in Shandong
Fortune Shop	White Intelligent Power Saver	Energy saver received, logistics is very fast, the quality is very good, the appearance is also very beautiful power saving is the most important.	Jingdong prefecture level city in Shandong
Fortune Shop	White Intelligent Power Saver	It's good to talk about it, it's not practical to use it.	Jingdong prefecture level city in Shandong
Fortune Shop	White Intelligent Power Saver	I don't know if it's good for now, I'll comment after using it	Jingdong prefecture level city in Shandong
Fortune Shop	White Intelligent Power Saver	I've been using it for two or three days. It's not energy efficient.	Jingdong prefecture level city in Shandong
Fortune Shop	White Intelligent Power Saver	Packaging is intact, logistics is also fast, cost-effective, inexpensive, very satisfied with a purchase	Jingdong prefecture level city in Shandong
Fortune Shop	White Intelligent Power Saver	In use to be observed!	Jingdong prefecture level city in Shandong
Fortune Shop	White Intelligent Power Saver	Use it for a while at a flat rate	Jingdong prefecture level city in Shandong
Fortune Shop	White Intelligent Power Saver	Very trustworthy brand, quality is guaranteed, the price is still affordable, quite satisfied!	Jingdong prefecture level city in Shandong
Fortune Shop	White Intelligent Power Saver	Great quality, great operation, easy to install, great price!	Jingdong prefecture level city in Shandong
Fortune Shop	White Intelligent Power Saver	I came back and installed it, it really saves electricity, much more than before, the	Jingdong prefecture level city in Shandong
Fortune Shop	White Intelligent Power Saver	Fast logistics, good quality, very good packaging, good customer service	Jingdong prefecture level city in Shandong
Fortune Shop	White Intelligent Power Saver	Very good quality, especially good, effective, satisfactory	Jingdong prefecture level city in Shandong
Fortune Shop	White Intelligent Power Saver	I don't know how well it works.	Jingdong prefecture level city in Shandong
Fortune Shop	White Intelligent Power Saver	Good value for money, recommend it haha	Jingdong prefecture level city in Shandong
Fortune Shop	White Intelligent Power Saver	Good. Good. Very good.	Jingdong prefecture level city in Shandong
Fortune Shop	White Intelligent Power Saver	It's not working.	Jingdong prefecture level city in Shandong
Fortune Shop	White Intelligent Power Saver	It's not working.	Jingdong prefecture level city in Shandong
Fortune Shop	White Intelligent Power Saver	In use	Jingdong prefecture level city in Shandong
Fortune Shop	White Intelligent Power Saver	I'll try it later.	Jingdong prefecture level city in Shandong
Fortune Shop	White Intelligent Power Saver	It's not much use.	Jingdong prefecture level city in Shandong
Fortune Shop	White Intelligent Power Saver	It doesn't save electricity.	Jingdong prefecture level city in Shandong
Fortune Shop	White Intelligent Power Saver	It's not much use.	Jingdong prefecture level city in Shandong
Fortune Shop	White Intelligent Power Saver	Tried it for a few days and tracked the record, and there is no difference from before.	Jingdong prefecture level city in Shandong
Fortune Shop	White Intelligent Power Saver	It's not very credible, so just do it yourself and turn off the lights diligently!	Jingdong prefecture level city in Shandong
Fortune Shop	White Intelligent Power Saver	Too bad, no power saving at all, scam people don't fall for it!	Jingdong prefecture level city in Shandong
Fortune Shop	White Intelligent Power Saver	It's not working at all.	Jingdong prefecture level city in Shandong
Fortune Shop	White Intelligent Power Saver	general	Jingdong prefecture level city in Shandong
Fortune Shop	White Intelligent Power Saver	(of an unmarried couple) be close	Jingdong prefecture level city in Shandong
Fortune Shop	White Intelligent Power Saver	of no use	Jingdong prefecture level city in Shandong
Fortune Shop	White Intelligent Power Saver	Actually can really save electricity, only used half a month to save more than 50, recommended to the neighbors to use together!	Jingdong prefecture level city in Shandong
Fortune Shop	White Intelligent Power Saver	Save what you can. Isn't it more cost effective to buy one of these than to pay the electric bill?	Jingdong prefecture level city in Shandong
Fortune Shop	White Intelligent Power Saver	Received, a use do not know how, use a month to see the effect is good, the effect is good then recommended, not good then I just do not recommend.	Jingdong prefecture level city in Shandong
Fortune Shop	White Intelligent Power Saver	Just received the goods, is a voltage regulator, next month to evaluate whether it is really power saving	Jingdong prefecture level city in Shandong
Fortune Shop	White Intelligent Power Saver	Very good, used a few days obviously feel that the electricity bill is much less, wait a month to see how much can be saved!	Jingdong prefecture level city in Shandong

Fortune Shop	White Intelligent Power Saver	Received the stuff used for more than a month without a little use, it feels like it uses more electricity than before	Jingdong prefecture level city in Shandong
Fortune Shop	White Intelligent Power Saver	The quality is very good, completely exceeded the expectations, the delivery speed is very fast, the packaging is very careful and strict, very satisfied with a shopping	Jingdong prefecture level city in Shandong
Fortune Shop	White Intelligent Power Saver	Fake, doesn't work at all. Don't even sell it.	Jingdong prefecture level city in Shandong
Fortune Shop	White Intelligent Power Saver	This item doesn't seem to have much effect?	Jingdong prefecture level city in Shandong
Fortune Shop	White Intelligent Power Saver	This production is a bit fake, just look at the focus on Ming	Jingdong prefecture level city in Shandong
Fortune Shop	White Intelligent Power Saver	The logistics is fast the service attitude is very good.	Jingdong prefecture level city in Shandong
Fortune Shop	White Intelligent Power Saver	The meter is good quality, sturdy and durable. It is accurate. Easy to use. Saves a lot of things.	Jingdong prefecture level city in Shandong
Fortune Shop	White Intelligent Power Saver	Just received the goods, don't know how it is yet	Jingdong prefecture level city in Shandong
Fortune Shop	White Intelligent Power Saver	Energy saver received, logistics is very fast, the quality is very good, the appearance is also very beautiful power saving is the most important.	Jingdong prefecture level city in Shandong
Fortune Shop	White Intelligent Power Saver	It's good to talk about it, it's not practical to use it.	Jingdong prefecture level city in Shandong
Fortune Shop	White Intelligent Power Saver	I don't know if it's good for now, I'll comment after using it	Jingdong prefecture level city in Shandong
Fortune Shop	White Intelligent Power Saver	I've been using it for two or three days. It's not energy efficient.	Jingdong prefecture level city in Shandong
Fortune Shop	White Intelligent Power Saver	Packaging is intact, logistics is also fast, cost-effective, inexpensive, very satisfied with a purchase	Jingdong prefecture level city in Shandong
Fortune Shop	White Intelligent Power Saver	In use to be observed!	Jingdong prefecture level city in Shandong
Fortune Shop	White Intelligent Power Saver	Use it for a while at a flat rate	Jingdong prefecture level city in Shandong
Fortune Shop	White Intelligent Power Saver	Very trustworthy brand, quality is guaranteed, the price is still affordable, quite satisfied!	Jingdong prefecture level city in Shandong
Fortune Shop	White Intelligent Power Saver	Great quality, great operation, easy to install, great price!	Jingdong prefecture level city in Shandong
Fortune Shop	White Intelligent Power Saver	I came back and installed it, it really saves electricity, much more than before, the	Jingdong prefecture level city in Shandong
Fortune Shop	White Intelligent Power Saver	Fast logistics, good quality, very good packaging, good customer service	Jingdong prefecture level city in Shandong
Fortune Shop	White Intelligent Power Saver	Very good quality, especially good, effective, satisfactory	Jingdong prefecture level city in Shandong
Fortune Shop	White Intelligent Power Saver	I don't know how well it works.	Jingdong prefecture level city in Shandong
Fortune Shop	White Intelligent Power Saver	Good value for money, recommend it haha	Jingdong prefecture level city in Shandong
Fortune Shop	White Intelligent Power Saver	Good. Good. Very good.	Jingdong prefecture level city in Shandong
Fortune Shop	White Intelligent Power Saver	It's not working.	Jingdong prefecture level city in Shandong
Fortune Shop	White Intelligent Power Saver	It's not working.	Jingdong prefecture level city in Shandong
Fortune Shop	White Intelligent Power Saver	In use	Jingdong prefecture level city in Shandong
Fortune Shop	White Intelligent Power Saver	I'll try it later.	Jingdong prefecture level city in Shandong
Fortune Shop	White Intelligent Power Saver	It's not much use.	Jingdong prefecture level city in Shandong
Fortune Shop	White Intelligent Power Saver	It doesn't save electricity.	Jingdong prefecture level city in Shandong
Fortune Shop	White Intelligent Power Saver	It's not much use.	Jingdong prefecture level city in Shandong
Fortune Shop	White Intelligent Power Saver	Tried it for a few days and tracked the record, and there is no difference from before.	Jingdong prefecture level city in Shandong
Fortune Shop	White Intelligent Power Saver	It's not very credible, so just do it yourself and turn off the lights diligently!	Jingdong prefecture level city in Shandong
Fortune Shop	White Intelligent Power Saver	Too bad, no power saving at all, scam people don't fall for it!	Jingdong prefecture level city in Shandong
Fortune Shop	White Intelligent Power Saver	It's not working at all.	Jingdong prefecture level city in Shandong
Fortune Shop	White Intelligent Power Saver	general	Jingdong prefecture level city in Shandong
Fortune Shop	White Intelligent Power Saver	(of an unmarried couple) be close	Jingdong prefecture level city in Shandong
Fortune Shop	White Intelligent Power Saver	of no use	Jingdong prefecture level city in Shandong
Fortune Shop	White Intelligent Power Saver	Actually can really save electricity, only used half a month to save more than 50, recommended to the neighbors to use together!	Jingdong prefecture level city in Shandong
Fortune Shop	White Intelligent Power Saver	Save what you can. Isn't it more cost effective to buy one of these than to pay the electric bill?	Jingdong prefecture level city in Shandong
Fortune Shop	White Intelligent Power Saver	Received, a use do not know how, use a month to see the effect is good, the effect is good then recommended, not good then I just do not recommend.	Jingdong prefecture level city in Shandong
Fortune Shop	White Intelligent Power Saver	Just received the goods, is a voltage regulator, next month to evaluate whether it is really power saving	Jingdong prefecture level city in Shandong
Fortune Shop	White Intelligent Power Saver	Very good, used a few days obviously feel that the electricity bill is much less, wait a month to see how much can be saved!	Jingdong prefecture level city in Shandong
Fortune Shop	White Intelligent Power Saver	Received the stuff used for more than a month without a little use, it feels like it uses more electricity than before	Jingdong prefecture level city in Shandong
Fortune Shop	White Intelligent Power Saver	The quality is very good, completely exceeded the expectations, the delivery speed is very fast, the packaging is very careful and strict, very satisfied with a shopping	Jingdong prefecture level city in Shandong
Fortune Shop	White Intelligent Power Saver	Fake, doesn't work at all. Don't even sell it.	Jingdong prefecture level city in Shandong
Fortune Shop	White Intelligent Power Saver	This item doesn't seem to have much effect?	Jingdong prefecture level city in Shandong
Fortune Shop	White Intelligent Power Saver	This production is a bit fake, just look at the focus on Ming	Jingdong prefecture level city in Shandong
Fortune Shop	White Intelligent Power Saver	The logistics is fast the service attitude is very good.	Jingdong prefecture level city in Shandong
Fortune Shop	White Intelligent Power Saver	The meter is good quality, sturdy and durable. It is accurate. Easy to use. Saves a lot of things.	Jingdong prefecture level city in Shandong
Fortune Shop	White Intelligent Power Saver	Just received the goods, don't know how it is yet	Jingdong prefecture level city in Shandong
Fortune Shop	White Intelligent Power Saver	Energy saver received, logistics is very fast, the quality is very good, the appearance is also very beautiful power saving is the most important.	Jingdong prefecture level city in Shandong

Fortune Shop	White Intelligent Power Saver	It's good to talk about it, it's not practical to use it.	Jingdong prefecture level city in Shandong
Fortune Shop	White Intelligent Power Saver	I don't know if it's good for now, I'll comment after using it	Jingdong prefecture level city in Shandong
Fortune Shop	White Intelligent Power Saver	I've been using it for two or three days. It's not energy efficient.	Jingdong prefecture level city in Shandong
Fortune Shop	White Intelligent Power Saver	Packaging is intact, logistics is also fast, cost-effective, inexpensive, very satisfied with a purchase	Jingdong prefecture level city in Shandong
Fortune Shop	White Intelligent Power Saver	In use to be observed!	Jingdong prefecture level city in Shandong
Fortune Shop	White Intelligent Power Saver	Use it for a while at a flat rate	Jingdong prefecture level city in Shandong
Fortune Shop	White Intelligent Power Saver	Very trustworthy brand, quality is guaranteed, the price is still affordable, quite satisfied!	Jingdong prefecture level city in Shandong
Fortune Shop	White Intelligent Power Saver	Great quality, great operation, easy to install, great price!	Jingdong prefecture level city in Shandong
Fortune Shop	White Intelligent Power Saver	I came back and installed it, it really saves electricity, much more than before, the	Jingdong prefecture level city in Shandong
Fortune Shop	White Intelligent Power Saver	Fast logistics, good quality, very good packaging, good customer service	Jingdong prefecture level city in Shandong
Fortune Shop	White Intelligent Power Saver	Very good quality, especially good, effective, satisfactory	Jingdong prefecture level city in Shandong
Fortune Shop	White Intelligent Power Saver	I don't know how well it works.	Jingdong prefecture level city in Shandong
Fortune Shop	White Intelligent Power Saver	Good value for money, recommend it haha	Jingdong prefecture level city in Shandong
Fortune Shop	White Intelligent Power Saver	Good. Good. Very good.	Jingdong prefecture level city in Shandong
Fortune Shop	White Intelligent Power Saver	It's not working.	Jingdong prefecture level city in Shandong
Fortune Shop	White Intelligent Power Saver	It's not working.	Jingdong prefecture level city in Shandong
Fortune Shop	White Intelligent Power Saver	In use	Jingdong prefecture level city in Shandong
Fortune Shop	White Intelligent Power Saver	I'll try it later.	Jingdong prefecture level city in Shandong
Fortune Shop	White Intelligent Power Saver	It's not much use.	Jingdong prefecture level city in Shandong
Fortune Shop	White Intelligent Power Saver	It doesn't save electricity.	Jingdong prefecture level city in Shandong
Fortune Shop	White Intelligent Power Saver	It's not much use.	Jingdong prefecture level city in Shandong
Fortune Shop	White Intelligent Power Saver	Tried it for a few days and tracked the record, and there is no difference from before.	Jingdong prefecture level city in Shandong
Fortune Shop	White Intelligent Power Saver	It's not very credible, so just do it yourself and turn off the lights diligently!	Jingdong prefecture level city in Shandong
Fortune Shop	White Intelligent Power Saver	Too bad, no power saving at all, scam people don't fall for it!	Jingdong prefecture level city in Shandong
Fortune Shop	White Intelligent Power Saver	It's not working at all.	Jingdong prefecture level city in Shandong
Fortune Shop	White Intelligent Power Saver	general	Jingdong prefecture level city in Shandong
Fortune Shop	White Intelligent Power Saver	(of an unmarried couple) be close	Jingdong prefecture level city in Shandong
Fortune Shop	White Intelligent Power Saver	of no use	Jingdong prefecture level city in Shandong
Fortune Shop	White Intelligent Power Saver	Actually can really save electricity, only used half a month to save more than 50, recommended to the neighbors to use together!	Jingdong prefecture level city in Shandong
Fortune Shop	White Intelligent Power Saver	Save what you can. Isn't it more cost effective to buy one of these than to pay the electric bill?	Jingdong prefecture level city in Shandong
Fortune Shop	White Intelligent Power Saver	Received, a use do not know how, use a month to see the effect is good, the effect is good then recommended, not good then I just do not recommend.	Jingdong prefecture level city in Shandong
Fortune Shop	White Intelligent Power Saver	Just received the goods, is a voltage regulator, next month to evaluate whether it is really power saving	Jingdong prefecture level city in Shandong
Fortune Shop	White Intelligent Power Saver	Very good, used a few days obviously feel that the electricity bill is much less, wait a month to see how much can be saved!	Jingdong prefecture level city in Shandong
Fortune Shop	White Intelligent Power Saver	Received the stuff used for more than a month without a little use, it feels like it uses more electricity than before	Jingdong prefecture level city in Shandong
Fortune Shop	White Intelligent Power Saver	The quality is very good, completely exceeded the expectations, the delivery speed is very fast, the packaging is very careful and strict, very satisfied with a shopping	Jingdong prefecture level city in Shandong
Fortune Shop	White Intelligent Power Saver	Fake, doesn't work at all. Don't even sell it.	Jingdong prefecture level city in Shandong
Fortune Shop	White Intelligent Power Saver	This item doesn't seem to have much effect?	Jingdong prefecture level city in Shandong
Fortune Shop	White Intelligent Power Saver	This production is a bit fake, just look at the focus on Ming	Jingdong prefecture level city in Shandong
Fortune Shop	White Intelligent Power Saver	The logistics is fast the service attitude is very good.	Jingdong prefecture level city in Shandong
Fortune Shop	White Intelligent Power Saver	The meter is good quality, sturdy and durable. It is accurate. Easy to use. Saves a lot of things.	Jingdong prefecture level city in Shandong
Fortune Shop	White Intelligent Power Saver	Just received the goods, don't know how it is yet	Jingdong prefecture level city in Shandong
Fortune Shop	White Intelligent Power Saver	Energy saver received, logistics is very fast, the quality is very good, the appearance is also very beautiful power saving is the most important.	Jingdong prefecture level city in Shandong
Fortune Shop	White Intelligent Power Saver	It's good to talk about it, it's not practical to use it.	Jingdong prefecture level city in Shandong
Fortune Shop	White Intelligent Power Saver	I don't know if it's good for now, I'll comment after using it	Jingdong prefecture level city in Shandong
Fortune Shop	White Intelligent Power Saver	I've been using it for two or three days. It's not energy efficient.	Jingdong prefecture level city in Shandong
Fortune Shop	White Intelligent Power Saver	Packaging is intact, logistics is also fast, cost-effective, inexpensive, very satisfied with a purchase	Jingdong prefecture level city in Shandong
Fortune Shop	White Intelligent Power Saver	In use to be observed!	Jingdong prefecture level city in Shandong
Fortune Shop	White Intelligent Power Saver	Use it for a while at a flat rate	Jingdong prefecture level city in Shandong
Fortune Shop	White Intelligent Power Saver	Very trustworthy brand, quality is guaranteed, the price is still affordable, quite satisfied!	Jingdong prefecture level city in Shandong
Fortune Shop	White Intelligent Power Saver	Great quality, great operation, easy to install, great price!	Jingdong prefecture level city in Shandong
Fortune Shop	White Intelligent Power Saver	I came back and installed it, it really saves electricity, much more than before, the	Jingdong prefecture level city in Shandong

Fortune Shop	White Intelligent Power Saver	Fast logistics, good quality, very good packaging, good customer service	Jingdong prefecture level city in Shandong
Fortune Shop	White Intelligent Power Saver	Very good quality, especially good, effective, satisfactory	Jingdong prefecture level city in Shandong
Fortune Shop	White Intelligent Power Saver	I don't know how well it works.	Jingdong prefecture level city in Shandong
Fortune Shop	White Intelligent Power Saver	Good value for money, recommend it haha	Jingdong prefecture level city in Shandong
Fortune Shop	White Intelligent Power Saver	Good. Good. Very good.	Jingdong prefecture level city in Shandong
Fortune Shop	White Intelligent Power Saver	It's not working.	Jingdong prefecture level city in Shandong
Fortune Shop	White Intelligent Power Saver	It's not working.	Jingdong prefecture level city in Shandong
Fortune Shop	White Intelligent Power Saver	In use	Jingdong prefecture level city in Shandong
Fortune Shop	White Intelligent Power Saver	I'll try it later.	Jingdong prefecture level city in Shandong
Fortune Shop	White Intelligent Power Saver	It's not much use.	Jingdong prefecture level city in Shandong
Fortune Shop	White Intelligent Power Saver	It doesn't save electricity.	Jingdong prefecture level city in Shandong
Fortune Shop	White Intelligent Power Saver	It's not much use.	Jingdong prefecture level city in Shandong
Fortune Shop	White Intelligent Power Saver	Tried it for a few days and tracked the record, and there is no difference from before.	Jingdong prefecture level city in Shandong
Fortune Shop	White Intelligent Power Saver	It's not very credible, so just do it yourself and turn off the lights diligently!	Jingdong prefecture level city in Shandong
Fortune Shop	White Intelligent Power Saver	Too bad, no power saving at all, scam people don't fall for it!	Jingdong prefecture level city in Shandong
Fortune Shop	White Intelligent Power Saver	It's not working at all.	Jingdong prefecture level city in Shandong
Fortune Shop	White Intelligent Power Saver	general	Jingdong prefecture level city in Shandong
Fortune Shop	White Intelligent Power Saver	(of an unmarried couple) be close	Jingdong prefecture level city in Shandong
Fortune Shop	White Intelligent Power Saver	of no use	Jingdong prefecture level city in Shandong
Fortune Shop	White Intelligent Power Saver	Actually can really save electricity, only used half a month to save more than 50, recommended to the neighbors to use together!	Jingdong prefecture level city in Shandong
Fortune Shop	White Intelligent Power Saver	Save what you can. Isn't it more cost effective to buy one of these than to pay the electric bill?	Jingdong prefecture level city in Shandong
Fortune Shop	White Intelligent Power Saver	Received, a use do not know how, use a month to see the effect is good, the effect is good then recommended, not good then I just do not recommend.	Jingdong prefecture level city in Shandong
Fortune Shop	White Intelligent Power Saver	Just received the goods, is a voltage regulator, next month to evaluate whether it is really power saving	Jingdong prefecture level city in Shandong
Fortune Shop	White Intelligent Power Saver	Very good, used a few days obviously feel that the electricity bill is much less, wait a month to see how much can be saved!	Jingdong prefecture level city in Shandong
Fortune Shop	White Intelligent Power Saver	Received the stuff used for more than a month without a little use, it feels like it uses more electricity than before	Jingdong prefecture level city in Shandong
Fortune Shop	White Intelligent Power Saver	The quality is very good, completely exceeded the expectations, the delivery speed is very fast, the packaging is very careful and strict, very satisfied with a shopping	Jingdong prefecture level city in Shandong
Fortune Shop	White Intelligent Power Saver	Fake, doesn't work at all. Don't even sell it.	Jingdong prefecture level city in Shandong
Fortune Shop	White Intelligent Power Saver	This item doesn't seem to have much effect?	Jingdong prefecture level city in Shandong
Fortune Shop	White Intelligent Power Saver	This production is a bit fake, just look at the focus on Ming	Jingdong prefecture level city in Shandong
Fortune Shop	White Intelligent Power Saver	The logistics is fast the service attitude is very good.	Jingdong prefecture level city in Shandong
Fortune Shop	White Intelligent Power Saver	The meter is good quality, sturdy and durable. It is accurate. Easy to use. Saves a lot of things.	Jingdong prefecture level city in Shandong
Fortune Shop	White Intelligent Power Saver	Just received the goods, don't know how it is yet	Jingdong prefecture level city in Shandong
Fortune Shop	White Intelligent Power Saver	Energy saver received, logistics is very fast, the quality is very good, the appearance is also very beautiful power saving is the most important.	Jingdong prefecture level city in Shandong
Fortune Shop	White Intelligent Power Saver	It's good to talk about it, it's not practical to use it.	Jingdong prefecture level city in Shandong
Fortune Shop	White Intelligent Power Saver	I don't know if it's good for now, I'll comment after using it	Jingdong prefecture level city in Shandong
Fortune Shop	White Intelligent Power Saver	I've been using it for two or three days. It's not energy efficient.	Jingdong prefecture level city in Shandong
Fortune Shop	White Intelligent Power Saver	Packaging is intact, logistics is also fast, cost-effective, inexpensive, very satisfied with a purchase	Jingdong prefecture level city in Shandong
Fortune Shop	White Intelligent Power Saver	In use to be observed!	Jingdong prefecture level city in Shandong
Fortune Shop	White Intelligent Power Saver	Use it for a while at a flat rate	Jingdong prefecture level city in Shandong
Fortune Shop	White Intelligent Power Saver	Very trustworthy brand, quality is guaranteed, the price is still affordable, quite satisfied!	Jingdong prefecture level city in Shandong
Fortune Shop	White Intelligent Power Saver	Great quality, great operation, easy to install, great price!	Jingdong prefecture level city in Shandong
Fortune Shop	White Intelligent Power Saver	I came back and installed it, it really saves electricity, much more than before, the	Jingdong prefecture level city in Shandong
Fortune Shop	White Intelligent Power Saver	Fast logistics, good quality, very good packaging, good customer service	Jingdong prefecture level city in Shandong
Fortune Shop	White Intelligent Power Saver	Very good quality, especially good, effective, satisfactory	Jingdong prefecture level city in Shandong
Fortune Shop	White Intelligent Power Saver	I don't know how well it works.	Jingdong prefecture level city in Shandong
Fortune Shop	White Intelligent Power Saver	Good value for money, recommend it haha	Jingdong prefecture level city in Shandong
Fortune Shop	White Intelligent Power Saver	Good. Good. Very good.	Jingdong prefecture level city in Shandong
Fortune Shop	White Intelligent Power Saver	It's not working.	Jingdong prefecture level city in Shandong
Fortune Shop	White Intelligent Power Saver	It's not working.	Jingdong prefecture level city in Shandong
Fortune Shop	White Intelligent Power Saver	In use	Jingdong prefecture level city in Shandong
Fortune Shop	White Intelligent Power Saver	I'll try it later.	Jingdong prefecture level city in Shandong

Fortune Shop	White Intelligent Power Saver	It's not much use.	Jingdong prefecture level city in Shandong
Fortune Shop	White Intelligent Power Saver	It doesn't save electricity.	Jingdong prefecture level city in Shandong
Fortune Shop	White Intelligent Power Saver	It's not much use.	Jingdong prefecture level city in Shandong
Fortune Shop	White Intelligent Power Saver	Tried it for a few days and tracked the record, and there is no difference from before.	Jingdong prefecture level city in Shandong
Fortune Shop	White Intelligent Power Saver	It's not very credible, so just do it yourself and turn off the lights diligently!	Jingdong prefecture level city in Shandong
Fortune Shop	White Intelligent Power Saver	Too bad, no power saving at all, scam people don't fall for it!	Jingdong prefecture level city in Shandong
Fortune Shop	White Intelligent Power Saver	It's not working at all.	Jingdong prefecture level city in Shandong
Fortune Shop	White Intelligent Power Saver	general	Jingdong prefecture level city in Shandong
Fortune Shop	White Intelligent Power Saver	(of an unmarried couple) be close	Jingdong prefecture level city in Shandong
Fortune Shop	White Intelligent Power Saver	of no use	Jingdong prefecture level city in Shandong
Fortune Shop	White Intelligent Power Saver	Actually can really save electricity, only used half a month to save more than 50, recommended to the neighbors to use together!	Jingdong prefecture level city in Shandong
Fortune Shop	White Intelligent Power Saver	Save what you can. Isn't it more cost effective to buy one of these than to pay the electric bill?	Jingdong prefecture level city in Shandong
Fortune Shop	White Intelligent Power Saver	Received, a use do not know how, use a month to see the effect is good, the effect is good then recommended, not good then I just do not recommend.	Jingdong prefecture level city in Shandong
Fortune Shop	White Intelligent Power Saver	Just received the goods, is a voltage regulator, next month to evaluate whether it is really power saving	Jingdong prefecture level city in Shandong
Fortune Shop	White Intelligent Power Saver	Very good, used a few days obviously feel that the electricity bill is much less, wait a month to see how much can be saved!	Jingdong prefecture level city in Shandong
Fortune Shop	White Intelligent Power Saver	Received the stuff used for more than a month without a little use, it feels like it uses more electricity than before	Jingdong prefecture level city in Shandong
Fortune Shop	White Intelligent Power Saver	The quality is very good, completely exceeded the expectations, the delivery speed is very fast, the packaging is very careful and strict, very satisfied with a shopping	Jingdong prefecture level city in Shandong
Fortune Shop	White Intelligent Power Saver	Fake, doesn't work at all. Don't even sell it.	Jingdong prefecture level city in Shandong
Fortune Shop	White Intelligent Power Saver	This item doesn't seem to have much effect?	Jingdong prefecture level city in Shandong
Fortune Shop	White Intelligent Power Saver	This production is a bit fake, just look at the focus on Ming	Jingdong prefecture level city in Shandong
Fortune Shop	White Intelligent Power Saver	The logistics is fast the service attitude is very good.	Jingdong prefecture level city in Shandong
Fortune Shop	White Intelligent Power Saver	The meter is good quality, sturdy and durable. It is accurate. Easy to use. Saves a lot of things.	Jingdong prefecture level city in Shandong
Fortune Shop	White Intelligent Power Saver	Just received the goods, don't know how it is yet	Jingdong prefecture level city in Shandong
Fortune Shop	White Intelligent Power Saver	Energy saver received, logistics is very fast, the quality is very good, the appearance is also very beautiful power saving is the most important.	Jingdong prefecture level city in Shandong
Fortune Shop	White Intelligent Power Saver	It's good to talk about it, it's not practical to use it.	Jingdong prefecture level city in Shandong
Fortune Shop	White Intelligent Power Saver	I don't know if it's good for now, I'll comment after using it	Jingdong prefecture level city in Shandong
Fortune Shop	White Intelligent Power Saver	I've been using it for two or three days. It's not energy efficient.	Jingdong prefecture level city in Shandong
Fortune Shop	White Intelligent Power Saver	Packaging is intact, logistics is also fast, cost-effective, inexpensive, very satisfied with a purchase	Jingdong prefecture level city in Shandong
Fortune Shop	White Intelligent Power Saver	In use to be observed!	Jingdong prefecture level city in Shandong
Fortune Shop	White Intelligent Power Saver	Use it for a while at a flat rate	Jingdong prefecture level city in Shandong
Fortune Shop	White Intelligent Power Saver	Very trustworthy brand, quality is guaranteed, the price is still affordable, quite satisfied!	Jingdong prefecture level city in Shandong
Fortune Shop	White Intelligent Power Saver	Great quality, great operation, easy to install, great price!	Jingdong prefecture level city in Shandong
Fortune Shop	White Intelligent Power Saver	I came back and installed it, it really saves electricity, much more than before, the	Jingdong prefecture level city in Shandong
Fortune Shop	White Intelligent Power Saver	Fast logistics, good quality, very good packaging, good customer service	Jingdong prefecture level city in Shandong
Fortune Shop	White Intelligent Power Saver	Very good quality, especially good, effective, satisfactory	Jingdong prefecture level city in Shandong
Fortune Shop	White Intelligent Power Saver	I don't know how well it works.	Jingdong prefecture level city in Shandong
Fortune Shop	White Intelligent Power Saver	Good value for money, recommend it haha	Jingdong prefecture level city in Shandong
Fortune Shop	White Intelligent Power Saver	Good. Good. Very good.	Jingdong prefecture level city in Shandong
Fortune Shop	White Intelligent Power Saver	It's not working.	Jingdong prefecture level city in Shandong
Fortune Shop	White Intelligent Power Saver	It's not working.	Jingdong prefecture level city in Shandong
Fortune Shop	White Intelligent Power Saver	In use	Jingdong prefecture level city in Shandong
Fortune Shop	White Intelligent Power Saver	I'll try it later.	Jingdong prefecture level city in Shandong
Fortune Shop	White Intelligent Power Saver	It's not much use.	Jingdong prefecture level city in Shandong
Fortune Shop	White Intelligent Power Saver	It doesn't save electricity.	Jingdong prefecture level city in Shandong
Fortune Shop	White Intelligent Power Saver	It's not much use.	Jingdong prefecture level city in Shandong
Fortune Shop	White Intelligent Power Saver	Tried it for a few days and tracked the record, and there is no difference from before.	Jingdong prefecture level city in Shandong
Fortune Shop	White Intelligent Power Saver	It's not very credible, so just do it yourself and turn off the lights diligently!	Jingdong prefecture level city in Shandong
Fortune Shop	White Intelligent Power Saver	Too bad, no power saving at all, scam people don't fall for it!	Jingdong prefecture level city in Shandong
Fortune Shop	White Intelligent Power Saver	It's not working at all.	Jingdong prefecture level city in Shandong
Fortune Shop	White Intelligent Power Saver	general	Jingdong prefecture level city in Shandong
Fortune Shop	White Intelligent Power Saver	(of an unmarried couple) be close	Jingdong prefecture level city in Shandong

Fortune Shop	White Intelligent Power Saver	of no use	Jingdong prefecture level city in Shandong
Fortune Shop	White Intelligent Power Saver	Actually can really save electricity, only used half a month to save more than 50, recommended to the neighbors to use together!	Jingdong prefecture level city in Shandong
Fortune Shop	White Intelligent Power Saver	Save what you can. Isn't it more cost effective to buy one of these than to pay the electric bill?	Jingdong prefecture level city in Shandong
Fortune Shop	White Intelligent Power Saver	Received, a use do not know how, use a month to see the effect is good, the effect is good then recommended, not good then I just do not recommend.	Jingdong prefecture level city in Shandong
Fortune Shop	White Intelligent Power Saver	Just received the goods, is a voltage regulator, next month to evaluate whether it is really power saving	Jingdong prefecture level city in Shandong
Fortune Shop	White Intelligent Power Saver	Very good, used a few days obviously feel that the electricity bill is much less, wait a month to see how much can be saved!	Jingdong prefecture level city in Shandong
Fortune Shop	White Intelligent Power Saver	Received the stuff used for more than a month without a little use, it feels like it uses more electricity than before	Jingdong prefecture level city in Shandong
Fortune Shop	White Intelligent Power Saver	The quality is very good, completely exceeded the expectations, the delivery speed is very fast, the packaging is very careful and strict, very satisfied with a shopping	Jingdong prefecture level city in Shandong
Fortune Shop	White Intelligent Power Saver	Fake, doesn't work at all. Don't even sell it.	Jingdong prefecture level city in Shandong
Fortune Shop	White Intelligent Power Saver	This item doesn't seem to have much effect?	Jingdong prefecture level city in Shandong
Fortune Shop	White Intelligent Power Saver	This production is a bit fake, just look at the focus on Ming	Jingdong prefecture level city in Shandong
Fortune Shop	White Intelligent Power Saver	The logistics is fast the service attitude is very good.	Jingdong prefecture level city in Shandong
Fortune Shop	White Intelligent Power Saver	The meter is good quality, sturdy and durable. It is accurate. Easy to use. Saves a lot of things.	Jingdong prefecture level city in Shandong
Fortune Shop	White Intelligent Power Saver	Just received the goods, don't know how it is yet	Jingdong prefecture level city in Shandong
Fortune Shop	White Intelligent Power Saver	Energy saver received, logistics is very fast, the quality is very good, the appearance is also very beautiful power saving is the most important.	Jingdong prefecture level city in Shandong
Fortune Shop	White Intelligent Power Saver	It's good to talk about it, it's not practical to use it.	Jingdong prefecture level city in Shandong
Fortune Shop	White Intelligent Power Saver	I don't know if it's good for now, I'll comment after using it	Jingdong prefecture level city in Shandong
Fortune Shop	White Intelligent Power Saver	I've been using it for two or three days. It's not energy efficient.	Jingdong prefecture level city in Shandong
Fortune Shop	White Intelligent Power Saver	Packaging is intact, logistics is also fast, cost-effective, inexpensive, very satisfied with a purchase	Jingdong prefecture level city in Shandong
Fortune Shop	White Intelligent Power Saver	In use to be observed!	Jingdong prefecture level city in Shandong
Fortune Shop	White Intelligent Power Saver	Use it for a while at a flat rate	Jingdong prefecture level city in Shandong
Fortune Shop	White Intelligent Power Saver	Very trustworthy brand, quality is guaranteed, the price is still affordable, quite satisfied!	Jingdong prefecture level city in Shandong
Fortune Shop	White Intelligent Power Saver	Great quality, great operation, easy to install, great price!	Jingdong prefecture level city in Shandong
Fortune Shop	White Intelligent Power Saver	I came back and installed it, it really saves electricity, much more than before, the	Jingdong prefecture level city in Shandong
Fortune Shop	White Intelligent Power Saver	Fast logistics, good quality, very good packaging, good customer service	Jingdong prefecture level city in Shandong
Fortune Shop	White Intelligent Power Saver	Very good quality, especially good, effective, satisfactory	Jingdong prefecture level city in Shandong
Fortune Shop	White Intelligent Power Saver	I don't know how well it works.	Jingdong prefecture level city in Shandong
Fortune Shop	White Intelligent Power Saver	Good value for money, recommend it haha	Jingdong prefecture level city in Shandong
Fortune Shop	White Intelligent Power Saver	Good. Good. Very good.	Jingdong prefecture level city in Shandong
Fortune Shop	White Intelligent Power Saver	It's not working.	Jingdong prefecture level city in Shandong
Fortune Shop	White Intelligent Power Saver	It's not working.	Jingdong prefecture level city in Shandong
Fortune Shop	White Intelligent Power Saver	In use	Jingdong prefecture level city in Shandong
Fortune Shop	White Intelligent Power Saver	I'll try it later.	Jingdong prefecture level city in Shandong
Fortune Shop	White Intelligent Power Saver	It's not much use.	Jingdong prefecture level city in Shandong
Fortune Shop	White Intelligent Power Saver	It doesn't save electricity.	Jingdong prefecture level city in Shandong
Fortune Shop	White Intelligent Power Saver	It's not much use.	Jingdong prefecture level city in Shandong
Fortune Shop	White Intelligent Power Saver	Tried it for a few days and tracked the record, and there is no difference from before.	Jingdong prefecture level city in Shandong
Fortune Shop	White Intelligent Power Saver	It's not very credible, so just do it yourself and turn off the lights diligently!	Jingdong prefecture level city in Shandong
Fortune Shop	White Intelligent Power Saver	Too bad, no power saving at all, scam people don't fall for it!	Jingdong prefecture level city in Shandong
Fortune Shop	White Intelligent Power Saver	It's not working at all.	Jingdong prefecture level city in Shandong
Fortune Shop	White Intelligent Power Saver	general	Jingdong prefecture level city in Shandong
Fortune Shop	White Intelligent Power Saver	(of an unmarried couple) be close	Jingdong prefecture level city in Shandong
Fortune Shop	White Intelligent Power Saver	of no use	Jingdong prefecture level city in Shandong
Fortune Shop	White Intelligent Power Saver	Actually can really save electricity, only used half a month to save more than 50, recommended to the neighbors to use together!	Jingdong prefecture level city in Shandong
Fortune Shop	White Intelligent Power Saver	Save what you can. Isn't it more cost effective to buy one of these than to pay the electric bill?	Jingdong prefecture level city in Shandong
Fortune Shop	White Intelligent Power Saver	Received, a use do not know how, use a month to see the effect is good, the effect is good then recommended, not good then I just do not recommend.	Jingdong prefecture level city in Shandong
Fortune Shop	White Intelligent Power Saver	Just received the goods, is a voltage regulator, next month to evaluate whether it is really power saving	Jingdong prefecture level city in Shandong
Fortune Shop	White Intelligent Power Saver	Very good, used a few days obviously feel that the electricity bill is much less, wait a month to see how much can be saved!	Jingdong prefecture level city in Shandong
Fortune Shop	White Intelligent Power Saver	Received the stuff used for more than a month without a little use, it feels like it uses more electricity than before	Jingdong prefecture level city in Shandong
Fortune Shop	White Intelligent Power Saver	The quality is very good, completely exceeded the expectations, the delivery speed is very fast, the packaging is very careful and strict, very satisfied with a shopping	Jingdong prefecture level city in Shandong
Fortune Shop	White Intelligent Power Saver	Fake, doesn't work at all. Don't even sell it.	Jingdong prefecture level city in Shandong

Fortune Shop	White Intelligent Power Saver	This item doesn't seem to have much effect?	Jingdong prefecture level city in Shandong
Fortune Shop	White Intelligent Power Saver	This production is a bit fake, just look at the focus on Ming	Jingdong prefecture level city in Shandong
Fortune Shop	White Intelligent Power Saver	The logistics is fast the service attitude is very good.	Jingdong prefecture level city in Shandong
Fortune Shop	White Intelligent Power Saver	The meter is good quality, sturdy and durable. It is accurate. Easy to use. Saves a lot of things.	Jingdong prefecture level city in Shandong
Fortune Shop	White Intelligent Power Saver	Just received the goods, don't know how it is yet	Jingdong prefecture level city in Shandong
Fortune Shop	White Intelligent Power Saver	Energy saver received, logistics is very fast, the quality is very good, the appearance is also very beautiful power saving is the most important.	Jingdong prefecture level city in Shandong
Fortune Shop	White Intelligent Power Saver	It's good to talk about it, it's not practical to use it.	Jingdong prefecture level city in Shandong
Fortune Shop	White Intelligent Power Saver	I don't know if it's good for now, I'll comment after using it	Jingdong prefecture level city in Shandong
Fortune Shop	White Intelligent Power Saver	I've been using it for two or three days. It's not energy efficient.	Jingdong prefecture level city in Shandong
Fortune Shop	White Intelligent Power Saver	Packaging is intact, logistics is also fast, cost-effective, inexpensive, very satisfied with a purchase	Jingdong prefecture level city in Shandong
Fortune Shop	White Intelligent Power Saver	In use to be observed!	Jingdong prefecture level city in Shandong
Fortune Shop	White Intelligent Power Saver	Use it for a while at a flat rate	Jingdong prefecture level city in Shandong
Fortune Shop	White Intelligent Power Saver	Very trustworthy brand, quality is guaranteed, the price is still affordable, quite satisfied!	Jingdong prefecture level city in Shandong
Fortune Shop	White Intelligent Power Saver	Great quality, great operation, easy to install, great price!	Jingdong prefecture level city in Shandong
Fortune Shop	White Intelligent Power Saver	I came back and installed it, it really saves electricity, much more than before, the	Jingdong prefecture level city in Shandong
Fortune Shop	White Intelligent Power Saver	Fast logistics, good quality, very good packaging, good customer service	Jingdong prefecture level city in Shandong
Fortune Shop	White Intelligent Power Saver	Very good quality, especially good, effective, satisfactory	Jingdong prefecture level city in Shandong
Fortune Shop	White Intelligent Power Saver	I don't know how well it works.	Jingdong prefecture level city in Shandong
Fortune Shop	White Intelligent Power Saver	Good value for money, recommend it haha	Jingdong prefecture level city in Shandong
Fortune Shop	White Intelligent Power Saver	Good. Good. Very good.	Jingdong prefecture level city in Shandong
Fortune Shop	White Intelligent Power Saver	It's not working.	Jingdong prefecture level city in Shandong
Fortune Shop	White Intelligent Power Saver	It's not working.	Jingdong prefecture level city in Shandong
Fortune Shop	White Intelligent Power Saver	In use	Jingdong prefecture level city in Shandong
Fortune Shop	White Intelligent Power Saver	I'll try it later.	Jingdong prefecture level city in Shandong
Fortune Shop	White Intelligent Power Saver	It's not much use.	Jingdong prefecture level city in Shandong
Fortune Shop	White Intelligent Power Saver	It doesn't save electricity.	Jingdong prefecture level city in Shandong
Fortune Shop	White Intelligent Power Saver	It's not much use.	Jingdong prefecture level city in Shandong
Fortune Shop	White Intelligent Power Saver	Tried it for a few days and tracked the record, and there is no difference from before.	Jingdong prefecture level city in Shandong
Fortune Shop	White Intelligent Power Saver	It's not very credible, so just do it yourself and turn off the lights diligently!	Jingdong prefecture level city in Shandong
Fortune Shop	White Intelligent Power Saver	Too bad, no power saving at all, scam people don't fall for it!	Jingdong prefecture level city in Shandong
Fortune Shop	White Intelligent Power Saver	It's not working at all.	Jingdong prefecture level city in Shandong
Fortune Shop	White Intelligent Power Saver	general	Jingdong prefecture level city in Shandong
Fortune Shop	White Intelligent Power Saver	(of an unmarried couple) be close	Jingdong prefecture level city in Shandong
Fortune Shop	White Intelligent Power Saver	of no use	Jingdong prefecture level city in Shandong
Fortune Shop	White Intelligent Power Saver	Actually can really save electricity, only used half a month to save more than 50, recommended to the neighbors to use together!	Jingdong prefecture level city in Shandong
Fortune Shop	White Intelligent Power Saver	Save what you can. Isn't it more cost effective to buy one of these than to pay the electric bill?	Jingdong prefecture level city in Shandong
Fortune Shop	White Intelligent Power Saver	Received, a use do not know how, use a month to see the effect is good, the effect is good then recommended, not good then I just do not recommend.	Jingdong prefecture level city in Shandong
Fortune Shop	White Intelligent Power Saver	Just received the goods, is a voltage regulator, next month to evaluate whether it is really power saving	Jingdong prefecture level city in Shandong
Fortune Shop	White Intelligent Power Saver	Very good, used a few days obviously feel that the electricity bill is much less, wait a month to see how much can be saved!	Jingdong prefecture level city in Shandong
Fortune Shop	White Intelligent Power Saver	Received the stuff used for more than a month without a little use, it feels like it uses more electricity than before	Jingdong prefecture level city in Shandong
Fortune Shop	White Intelligent Power Saver	The quality is very good, completely exceeded the expectations, the delivery speed is very fast, the packaging is very careful and strict, very satisfied with a shopping	Jingdong prefecture level city in Shandong
Fortune Shop	White Intelligent Power Saver	Fake, doesn't work at all. Don't even sell it.	Jingdong prefecture level city in Shandong
Fortune Shop	White Intelligent Power Saver	This item doesn't seem to have much effect?	Jingdong prefecture level city in Shandong
Fortune Shop	White Intelligent Power Saver	This production is a bit fake, just look at the focus on Ming	Jingdong prefecture level city in Shandong
Fortune Shop	White Intelligent Power Saver	The logistics is fast the service attitude is very good.	Jingdong prefecture level city in Shandong
Fortune Shop	White Intelligent Power Saver	The meter is good quality, sturdy and durable. It is accurate. Easy to use. Saves a lot of things.	Jingdong prefecture level city in Shandong
Fortune Shop	White Intelligent Power Saver	Just received the goods, don't know how it is yet	Jingdong prefecture level city in Shandong
Fortune Shop	White Intelligent Power Saver	Energy saver received, logistics is very fast, the quality is very good, the appearance is also very beautiful power saving is the most important.	Jingdong prefecture level city in Shandong
Fortune Shop	White Intelligent Power Saver	It's good to talk about it, it's not practical to use it.	Jingdong prefecture level city in Shandong
Fortune Shop	White Intelligent Power Saver	I don't know if it's good for now, I'll comment after using it	Jingdong prefecture level city in Shandong
Fortune Shop	White Intelligent Power Saver	I've been using it for two or three days. It's not energy efficient.	Jingdong prefecture level city in Shandong

Fortune Shop	White Intelligent Power Saver	Packaging is intact, logistics is also fast, cost-effective, inexpensive, very satisfied with a purchase	Jingdong prefecture level city in Shandong
Fortune Shop	White Intelligent Power Saver	In use to be observed!	Jingdong prefecture level city in Shandong
Fortune Shop	White Intelligent Power Saver	Use it for a while at a flat rate	Jingdong prefecture level city in Shandong
Fortune Shop	White Intelligent Power Saver	Very trustworthy brand, quality is guaranteed, the price is still affordable, quite satisfied!	Jingdong prefecture level city in Shandong
Fortune Shop	White Intelligent Power Saver	Great quality, great operation, easy to install, great price!	Jingdong prefecture level city in Shandong
Fortune Shop	White Intelligent Power Saver	I came back and installed it, it really saves electricity, much more than before, the	Jingdong prefecture level city in Shandong
Fortune Shop	White Intelligent Power Saver	Fast logistics, good quality, very good packaging, good customer service	Jingdong prefecture level city in Shandong
Fortune Shop	White Intelligent Power Saver	Very good quality, especially good, effective, satisfactory	Jingdong prefecture level city in Shandong
Fortune Shop	White Intelligent Power Saver	I don't know how well it works.	Jingdong prefecture level city in Shandong
Fortune Shop	White Intelligent Power Saver	Good value for money, recommend it haha	Jingdong prefecture level city in Shandong
Fortune Shop	White Intelligent Power Saver	Good. Good. Very good.	Jingdong prefecture level city in Shandong
Fortune Shop	White Intelligent Power Saver	It's not working.	Jingdong prefecture level city in Shandong
Fortune Shop	White Intelligent Power Saver	It's not working.	Jingdong prefecture level city in Shandong
Fortune Shop	White Intelligent Power Saver	In use	Jingdong prefecture level city in Shandong
Fortune Shop	White Intelligent Power Saver	I'll try it later.	Jingdong prefecture level city in Shandong
Fortune Shop	White Intelligent Power Saver	It's not much use.	Jingdong prefecture level city in Shandong
Fortune Shop	White Intelligent Power Saver	It doesn't save electricity.	Jingdong prefecture level city in Shandong
Fortune Shop	White Intelligent Power Saver	It's not much use.	Jingdong prefecture level city in Shandong
Fortune Shop	White Intelligent Power Saver	Tried it for a few days and tracked the record, and there is no difference from before.	Jingdong prefecture level city in Shandong
Fortune Shop	White Intelligent Power Saver	It's not very credible, so just do it yourself and turn off the lights diligently!	Jingdong prefecture level city in Shandong
Fortune Shop	White Intelligent Power Saver	Too bad, no power saving at all, scam people don't fall for it!	Jingdong prefecture level city in Shandong
Fortune Shop	White Intelligent Power Saver	It's not working at all.	Jingdong prefecture level city in Shandong
Fortune Shop	White Intelligent Power Saver	general	Jingdong prefecture level city in Shandong
Fortune Shop	White Intelligent Power Saver	(of an unmarried couple) be close	Jingdong prefecture level city in Shandong
Fortune Shop	White Intelligent Power Saver	of no use	Jingdong prefecture level city in Shandong
Fortune Shop	White Intelligent Power Saver	Actually can really save electricity, only used half a month to save more than 50, recommended to the neighbors to use together!	Jingdong prefecture level city in Shandong
Fortune Shop	White Intelligent Power Saver	Save what you can. Isn't it more cost effective to buy one of these than to pay the electric bill?	Jingdong prefecture level city in Shandong
Fortune Shop	White Intelligent Power Saver	Received, a use do not know how, use a month to see the effect is good, the effect is good then recommended, not good then I just do not recommend.	Jingdong prefecture level city in Shandong
Fortune Shop	White Intelligent Power Saver	Just received the goods, is a voltage regulator, next month to evaluate whether it is really power saving	Jingdong prefecture level city in Shandong
Fortune Shop	White Intelligent Power Saver	Very good, used a few days obviously feel that the electricity bill is much less, wait a month to see how much can be saved!	Jingdong prefecture level city in Shandong
Fortune Shop	White Intelligent Power Saver	Received the stuff used for more than a month without a little use, it feels like it uses more electricity than before	Jingdong prefecture level city in Shandong
Fortune Shop	White Intelligent Power Saver	The quality is very good, completely exceeded the expectations, the delivery speed is very fast, the packaging is very careful and strict, very satisfied with a shopping	Jingdong prefecture level city in Shandong
Fortune Shop	White Intelligent Power Saver	Fake, doesn't work at all. Don't even sell it.	Jingdong prefecture level city in Shandong
Fortune Shop	White Intelligent Power Saver	This item doesn't seem to have much effect?	Jingdong prefecture level city in Shandong
Fortune Shop	White Intelligent Power Saver	This production is a bit fake, just look at the focus on Ming	Jingdong prefecture level city in Shandong
Fortune Shop	White Intelligent Power Saver	The logistics is fast the service attitude is very good.	Jingdong prefecture level city in Shandong
Fortune Shop	White Intelligent Power Saver	The meter is good quality, sturdy and durable. It is accurate. Easy to use. Saves a lot of things.	Jingdong prefecture level city in Shandong
Fortune Shop	White Intelligent Power Saver	Just received the goods, don't know how it is yet	Jingdong prefecture level city in Shandong
Fortune Shop	White Intelligent Power Saver	Energy saver received, logistics is very fast, the quality is very good, the appearance is also very beautiful power saving is the most important.	Jingdong prefecture level city in Shandong
Fortune Shop	White Intelligent Power Saver	It's good to talk about it, it's not practical to use it.	Jingdong prefecture level city in Shandong
Fortune Shop	White Intelligent Power Saver	I don't know if it's good for now, I'll comment after using it	Jingdong prefecture level city in Shandong
Fortune Shop	White Intelligent Power Saver	I've been using it for two or three days. It's not energy efficient.	Jingdong prefecture level city in Shandong
Fortune Shop	White Intelligent Power Saver	Packaging is intact, logistics is also fast, cost-effective, inexpensive, very satisfied with a purchase	Jingdong prefecture level city in Shandong
Fortune Shop	White Intelligent Power Saver	In use to be observed!	Jingdong prefecture level city in Shandong
Fortune Shop	White Intelligent Power Saver	Use it for a while at a flat rate	Jingdong prefecture level city in Shandong
Fortune Shop	White Intelligent Power Saver	Very trustworthy brand, quality is guaranteed, the price is still affordable, quite satisfied!	Jingdong prefecture level city in Shandong
Fortune Shop	White Intelligent Power Saver	Great quality, great operation, easy to install, great price!	Jingdong prefecture level city in Shandong
Fortune Shop	White Intelligent Power Saver	I came back and installed it, it really saves electricity, much more than before, the	Jingdong prefecture level city in Shandong
Fortune Shop	White Intelligent Power Saver	Fast logistics, good quality, very good packaging, good customer service	Jingdong prefecture level city in Shandong
Fortune Shop	White Intelligent Power Saver	Very good quality, especially good, effective, satisfactory	Jingdong prefecture level city in Shandong
Fortune Shop	White Intelligent Power Saver	I don't know how well it works.	Jingdong prefecture level city in Shandong

Fortune Shop	White Intelligent Power Saver	Good value for money, recommend it haha	Jingdong prefecture level city in Shandong
Fortune Shop	White Intelligent Power Saver	Good. Good. Very good.	Jingdong prefecture level city in Shandong
Fortune Shop	White Intelligent Power Saver	It's not working.	Jingdong prefecture level city in Shandong
Fortune Shop	White Intelligent Power Saver	It's not working.	Jingdong prefecture level city in Shandong
Fortune Shop	White Intelligent Power Saver	In use	Jingdong prefecture level city in Shandong
Fortune Shop	White Intelligent Power Saver	I'll try it later.	Jingdong prefecture level city in Shandong
Fortune Shop	White Intelligent Power Saver	It's not much use.	Jingdong prefecture level city in Shandong
Fortune Shop	White Intelligent Power Saver	It doesn't save electricity.	Jingdong prefecture level city in Shandong
Fortune Shop	White Intelligent Power Saver	It's not much use.	Jingdong prefecture level city in Shandong
Fortune Shop	White Intelligent Power Saver	Tried it for a few days and tracked the record, and there is no difference from before.	Jingdong prefecture level city in Shandong
Fortune Shop	White Intelligent Power Saver	It's not very credible, so just do it yourself and turn off the lights diligently!	Jingdong prefecture level city in Shandong
Fortune Shop	White Intelligent Power Saver	Too bad, no power saving at all, scam people don't fall for it!	Jingdong prefecture level city in Shandong
Fortune Shop	White Intelligent Power Saver	It's not working at all.	Jingdong prefecture level city in Shandong
Fortune Shop	White Intelligent Power Saver	general	Jingdong prefecture level city in Shandong
Fortune Shop	White Intelligent Power Saver	(of an unmarried couple) be close	Jingdong prefecture level city in Shandong
Fortune Shop	White Intelligent Power Saver	of no use	Jingdong prefecture level city in Shandong
Fortune Shop	White Intelligent Power Saver	Actually can really save electricity, only used half a month to save more than 50, recommended to the neighbors to use together!	Jingdong prefecture level city in Shandong
Fortune Shop	White Intelligent Power Saver	Save what you can. Isn't it more cost effective to buy one of these than to pay the electric bill?	Jingdong prefecture level city in Shandong
Fortune Shop	White Intelligent Power Saver	Received, a use do not know how, use a month to see the effect is good, the effect is good then recommended, not good then I just do not recommend.	Jingdong prefecture level city in Shandong
Fortune Shop	White Intelligent Power Saver	Just received the goods, is a voltage regulator, next month to evaluate whether it is really power saving	Jingdong prefecture level city in Shandong
Fortune Shop	White Intelligent Power Saver	Very good, used a few days obviously feel that the electricity bill is much less, wait a month to see how much can be saved!	Jingdong prefecture level city in Shandong
Fortune Shop	White Intelligent Power Saver	Received the stuff used for more than a month without a little use, it feels like it uses more electricity than before	Jingdong prefecture level city in Shandong
Fortune Shop	White Intelligent Power Saver	The quality is very good, completely exceeded the expectations, the delivery speed is very fast, the packaging is very careful and strict, very satisfied with a shopping	Jingdong prefecture level city in Shandong
Fortune Shop	White Intelligent Power Saver	Fake, doesn't work at all. Don't even sell it.	Jingdong prefecture level city in Shandong
Fortune Shop	White Intelligent Power Saver	This item doesn't seem to have much effect?	Jingdong prefecture level city in Shandong
Fortune Shop	White Intelligent Power Saver	This production is a bit fake, just look at the focus on Ming	Jingdong prefecture level city in Shandong
Fortune Shop	White Intelligent Power Saver	The logistics is fast the service attitude is very good.	Jingdong prefecture level city in Shandong
Fortune Shop	White Intelligent Power Saver	The meter is good quality, sturdy and durable. It is accurate. Easy to use. Saves a lot of things.	Jingdong prefecture level city in Shandong
Fortune Shop	White Intelligent Power Saver	Just received the goods, don't know how it is yet	Jingdong prefecture level city in Shandong
Fortune Shop	White Intelligent Power Saver	Energy saver received, logistics is very fast, the quality is very good, the appearance is also very beautiful power saving is the most important.	Jingdong prefecture level city in Shandong
Fortune Shop	White Intelligent Power Saver	It's good to talk about it, it's not practical to use it.	Jingdong prefecture level city in Shandong
Fortune Shop	White Intelligent Power Saver	I don't know if it's good for now, I'll comment after using it	Jingdong prefecture level city in Shandong
Fortune Shop	White Intelligent Power Saver	I've been using it for two or three days. It's not energy efficient.	Jingdong prefecture level city in Shandong
Fortune Shop	White Intelligent Power Saver	Packaging is intact, logistics is also fast, cost-effective, inexpensive, very satisfied with a purchase	Jingdong prefecture level city in Shandong
Fortune Shop	White Intelligent Power Saver	In use to be observed!	Jingdong prefecture level city in Shandong
Fortune Shop	White Intelligent Power Saver	Use it for a while at a flat rate	Jingdong prefecture level city in Shandong
Fortune Shop	White Intelligent Power Saver	Very trustworthy brand, quality is guaranteed, the price is still affordable, quite satisfied!	Jingdong prefecture level city in Shandong
Fortune Shop	White Intelligent Power Saver	Great quality, great operation, easy to install, great price!	Jingdong prefecture level city in Shandong
Fortune Shop	White Intelligent Power Saver	I came back and installed it, it really saves electricity, much more than before, the	Jingdong prefecture level city in Shandong
Fortune Shop	White Intelligent Power Saver	Fast logistics, good quality, very good packaging, good customer service	Jingdong prefecture level city in Shandong
Fortune Shop	White Intelligent Power Saver	Very good quality, especially good, effective, satisfactory	Jingdong prefecture level city in Shandong
Fortune Shop	White Intelligent Power Saver	I don't know how well it works.	Jingdong prefecture level city in Shandong
Fortune Shop	White Intelligent Power Saver	Good value for money, recommend it haha	Jingdong prefecture level city in Shandong
Fortune Shop	White Intelligent Power Saver	Good. Good. Very good.	Jingdong prefecture level city in Shandong
Fortune Shop	White Intelligent Power Saver	It's not working.	Jingdong prefecture level city in Shandong
Fortune Shop	White Intelligent Power Saver	It's not working.	Jingdong prefecture level city in Shandong
Fortune Shop	White Intelligent Power Saver	In use	Jingdong prefecture level city in Shandong
Fortune Shop	White Intelligent Power Saver	I'll try it later.	Jingdong prefecture level city in Shandong
Fortune Shop	White Intelligent Power Saver	It's not much use.	Jingdong prefecture level city in Shandong
Fortune Shop	White Intelligent Power Saver	It doesn't save electricity.	Jingdong prefecture level city in Shandong
Fortune Shop	White Intelligent Power Saver	It's not much use.	Jingdong prefecture level city in Shandong

Fortune Shop	White Intelligent Power Saver	Tried it for a few days and tracked the record, and there is no difference from before.	Jingdong prefecture level city in Shandong
Fortune Shop	White Intelligent Power Saver	It's not very credible, so just do it yourself and turn off the lights diligently!	Jingdong prefecture level city in Shandong
Fortune Shop	White Intelligent Power Saver	Too bad, no power saving at all, scam people don't fall for it!	Jingdong prefecture level city in Shandong
Fortune Shop	White Intelligent Power Saver	It's not working at all.	Jingdong prefecture level city in Shandong
Fortune Shop	White Intelligent Power Saver	general	Jingdong prefecture level city in Shandong
Fortune Shop	White Intelligent Power Saver	(of an unmarried couple) be close	Jingdong prefecture level city in Shandong
Fortune Shop	White Intelligent Power Saver	of no use	Jingdong prefecture level city in Shandong
Fortune Shop	White Intelligent Power Saver	Actually can really save electricity, only used half a month to save more than 50, recommended to the neighbors to use together!	Jingdong prefecture level city in Shandong
Fortune Shop	White Intelligent Power Saver	Save what you can. Isn't it more cost effective to buy one of these than to pay the electric bill?	Jingdong prefecture level city in Shandong
Fortune Shop	White Intelligent Power Saver	Received, a use do not know how, use a month to see the effect is good, the effect is good then recommended, not good then I just do not recommend.	Jingdong prefecture level city in Shandong
Fortune Shop	White Intelligent Power Saver	Just received the goods, is a voltage regulator, next month to evaluate whether it is really power saving	Jingdong prefecture level city in Shandong
Fortune Shop	White Intelligent Power Saver	Very good, used a few days obviously feel that the electricity bill is much less, wait a month to see how much can be saved!	Jingdong prefecture level city in Shandong
Fortune Shop	White Intelligent Power Saver	Received the stuff used for more than a month without a little use, it feels like it uses more electricity than before	Jingdong prefecture level city in Shandong
Fortune Shop	White Intelligent Power Saver	The quality is very good, completely exceeded the expectations, the delivery speed is very fast, the packaging is very careful and strict, very satisfied with a shopping	Jingdong prefecture level city in Shandong
Fortune Shop	White Intelligent Power Saver	Fake, doesn't work at all. Don't even sell it.	Jingdong prefecture level city in Shandong
Fortune Shop	White Intelligent Power Saver	This item doesn't seem to have much effect?	Jingdong prefecture level city in Shandong
Fortune Shop	White Intelligent Power Saver	This production is a bit fake, just look at the focus on Ming	Jingdong prefecture level city in Shandong
Fortune Shop	White Intelligent Power Saver	The logistics is fast the service attitude is very good.	Jingdong prefecture level city in Shandong
Fortune Shop	White Intelligent Power Saver	The meter is good quality, sturdy and durable. It is accurate. Easy to use. Saves a lot of things.	Jingdong prefecture level city in Shandong
Fortune Shop	White Intelligent Power Saver	Just received the goods, don't know how it is yet	Jingdong prefecture level city in Shandong
Fortune Shop	White Intelligent Power Saver	Energy saver received, logistics is very fast, the quality is very good, the appearance is also very beautiful power saving is the most important.	Jingdong prefecture level city in Shandong
Fortune Shop	White Intelligent Power Saver	It's good to talk about it, it's not practical to use it.	Jingdong prefecture level city in Shandong
Fortune Shop	White Intelligent Power Saver	I don't know if it's good for now, I'll comment after using it	Jingdong prefecture level city in Shandong
Fortune Shop	White Intelligent Power Saver	I've been using it for two or three days. It's not energy efficient.	Jingdong prefecture level city in Shandong
Fortune Shop	White Intelligent Power Saver	Packaging is intact, logistics is also fast, cost-effective, inexpensive, very satisfied with a purchase	Jingdong prefecture level city in Shandong
Fortune Shop	White Intelligent Power Saver	In use to be observed!	Jingdong prefecture level city in Shandong
Fortune Shop	White Intelligent Power Saver	Use it for a while at a flat rate	Jingdong prefecture level city in Shandong
Fortune Shop	White Intelligent Power Saver	Very trustworthy brand, quality is guaranteed, the price is still affordable, quite satisfied!	Jingdong prefecture level city in Shandong
Fortune Shop	White Intelligent Power Saver	Great quality, great operation, easy to install, great price!	Jingdong prefecture level city in Shandong
Fortune Shop	White Intelligent Power Saver	I came back and installed it, it really saves electricity, much more than before, the	Jingdong prefecture level city in Shandong
Fortune Shop	White Intelligent Power Saver	Fast logistics, good quality, very good packaging, good customer service	Jingdong prefecture level city in Shandong
Fortune Shop	White Intelligent Power Saver	Very good quality, especially good, effective, satisfactory	Jingdong prefecture level city in Shandong
Fortune Shop	White Intelligent Power Saver	I don't know how well it works.	Jingdong prefecture level city in Shandong
Fortune Shop	White Intelligent Power Saver	Good value for money, recommend it haha	Jingdong prefecture level city in Shandong
Fortune Shop	White Intelligent Power Saver	Good. Good. Very good.	Jingdong prefecture level city in Shandong
Fortune Shop	White Intelligent Power Saver	It's not working.	Jingdong prefecture level city in Shandong
Fortune Shop	White Intelligent Power Saver	It's not working.	Jingdong prefecture level city in Shandong
Fortune Shop	White Intelligent Power Saver	In use	Jingdong prefecture level city in Shandong
Fortune Shop	White Intelligent Power Saver	I'll try it later.	Jingdong prefecture level city in Shandong
Fortune Shop	White Intelligent Power Saver	It's not much use.	Jingdong prefecture level city in Shandong
Fortune Shop	White Intelligent Power Saver	It doesn't save electricity.	Jingdong prefecture level city in Shandong
Fortune Shop	White Intelligent Power Saver	It's not much use.	Jingdong prefecture level city in Shandong
Fortune Shop	White Intelligent Power Saver	Tried it for a few days and tracked the record, and there is no difference from before.	Jingdong prefecture level city in Shandong
Fortune Shop	White Intelligent Power Saver	It's not very credible, so just do it yourself and turn off the lights diligently!	Jingdong prefecture level city in Shandong
Fortune Shop	White Intelligent Power Saver	Too bad, no power saving at all, scam people don't fall for it!	Jingdong prefecture level city in Shandong
Fortune Shop	White Intelligent Power Saver	It's not working at all.	Jingdong prefecture level city in Shandong
Fortune Shop	White Intelligent Power Saver	general	Jingdong prefecture level city in Shandong
Fortune Shop	White Intelligent Power Saver	(of an unmarried couple) be close	Jingdong prefecture level city in Shandong
Fortune Shop	White Intelligent Power Saver	of no use	Jingdong prefecture level city in Shandong
Fortune Shop	White Intelligent Power Saver	Actually can really save electricity, only used half a month to save more than 50, recommended to the neighbors to use together!	Jingdong prefecture level city in Shandong
Fortune Shop	White Intelligent Power Saver	Save what you can. Isn't it more cost effective to buy one of these than to pay the electric bill?	Jingdong prefecture level city in Shandong

Fortune Shop	White Intelligent Power Saver	Received, a use do not know how, use a month to see the effect is good, the effect is good then recommended, not good then I just do not recommend.	Jingdong prefecture level city in Shandong
Fortune Shop	White Intelligent Power Saver	Just received the goods, is a voltage regulator, next month to evaluate whether it is really power saving	Jingdong prefecture level city in Shandong
Fortune Shop	White Intelligent Power Saver	Very good, used a few days obviously feel that the electricity bill is much less, wait a month to see how much can be saved!	Jingdong prefecture level city in Shandong
Fortune Shop	White Intelligent Power Saver	Received the stuff used for more than a month without a little use, it feels like it uses more electricity than before	Jingdong prefecture level city in Shandong
Fortune Shop	White Intelligent Power Saver	The quality is very good, completely exceeded the expectations, the delivery speed is very fast, the packaging is very careful and strict, very satisfied with a shopping	Jingdong prefecture level city in Shandong
Fortune Shop	White Intelligent Power Saver	Fake, doesn't work at all. Don't even sell it.	Jingdong prefecture level city in Shandong
Fortune Shop	White Intelligent Power Saver	This item doesn't seem to have much effect?	Jingdong prefecture level city in Shandong
Fortune Shop	White Intelligent Power Saver	This production is a bit fake, just look at the focus on Ming	Jingdong prefecture level city in Shandong
Fortune Shop	White Intelligent Power Saver	The logistics is fast the service attitude is very good.	Jingdong prefecture level city in Shandong
Fortune Shop	White Intelligent Power Saver	The meter is good quality, sturdy and durable. It is accurate. Easy to use. Saves a lot of things.	Jingdong prefecture level city in Shandong
Fortune Shop	White Intelligent Power Saver	Just received the goods, don't know how it is yet	Jingdong prefecture level city in Shandong
Fortune Shop	White Intelligent Power Saver	Energy saver received, logistics is very fast, the quality is very good, the appearance is also very beautiful power saving is the most important.	Jingdong prefecture level city in Shandong
Fortune Shop	White Intelligent Power Saver	It's good to talk about it, it's not practical to use it.	Jingdong prefecture level city in Shandong
Fortune Shop	White Intelligent Power Saver	I don't know if it's good for now, I'll comment after using it	Jingdong prefecture level city in Shandong
Fortune Shop	White Intelligent Power Saver	I've been using it for two or three days. It's not energy efficient.	Jingdong prefecture level city in Shandong
Fortune Shop	White Intelligent Power Saver	Packaging is intact, logistics is also fast, cost-effective, inexpensive, very satisfied with a purchase	Jingdong prefecture level city in Shandong
Fortune Shop	White Intelligent Power Saver	In use to be observed!	Jingdong prefecture level city in Shandong
Fortune Shop	White Intelligent Power Saver	Use it for a while at a flat rate	Jingdong prefecture level city in Shandong
Fortune Shop	White Intelligent Power Saver	Very trustworthy brand, quality is guaranteed, the price is still affordable, quite satisfied!	Jingdong prefecture level city in Shandong
Fortune Shop	White Intelligent Power Saver	Great quality, great operation, easy to install, great price!	Jingdong prefecture level city in Shandong
Fortune Shop	White Intelligent Power Saver	I came back and installed it, it really saves electricity, much more than before, the	Jingdong prefecture level city in Shandong
Fortune Shop	White Intelligent Power Saver	Fast logistics, good quality, very good packaging, good customer service	Jingdong prefecture level city in Shandong
Fortune Shop	White Intelligent Power Saver	Very good quality, especially good, effective, satisfactory	Jingdong prefecture level city in Shandong
Fortune Shop	White Intelligent Power Saver	I don't know how well it works.	Jingdong prefecture level city in Shandong
Fortune Shop	White Intelligent Power Saver	Good value for money, recommend it haha	Jingdong prefecture level city in Shandong
Fortune Shop	White Intelligent Power Saver	Good. Good. Very good.	Jingdong prefecture level city in Shandong
Fortune Shop	White Intelligent Power Saver	It's not working.	Jingdong prefecture level city in Shandong
Fortune Shop	White Intelligent Power Saver	It's not working.	Jingdong prefecture level city in Shandong
Fortune Shop	White Intelligent Power Saver	In use	Jingdong prefecture level city in Shandong
Fortune Shop	White Intelligent Power Saver	I'll try it later.	Jingdong prefecture level city in Shandong
Fortune Shop	White Intelligent Power Saver	It's not much use.	Jingdong prefecture level city in Shandong
Fortune Shop	White Intelligent Power Saver	It doesn't save electricity.	Jingdong prefecture level city in Shandong
Fortune Shop	White Intelligent Power Saver	It's not much use.	Jingdong prefecture level city in Shandong
Fortune Shop	White Intelligent Power Saver	Tried it for a few days and tracked the record, and there is no difference from before.	Jingdong prefecture level city in Shandong
Fortune Shop	White Intelligent Power Saver	It's not very credible, so just do it yourself and turn off the lights diligently!	Jingdong prefecture level city in Shandong
Fortune Shop	White Intelligent Power Saver	Too bad, no power saving at all, scam people don't fall for it!	Jingdong prefecture level city in Shandong
Fortune Shop	White Intelligent Power Saver	It's not working at all.	Jingdong prefecture level city in Shandong
Fortune Shop	White Intelligent Power Saver	general	Jingdong prefecture level city in Shandong
Fortune Shop	White Intelligent Power Saver	(of an unmarried couple) be close	Jingdong prefecture level city in Shandong
Fortune Shop	White Intelligent Power Saver	of no use	Jingdong prefecture level city in Shandong
Fortune Shop	White Intelligent Power Saver	Actually can really save electricity, only used half a month to save more than 50, recommended to the neighbors to use together!	Jingdong prefecture level city in Shandong
Fortune Shop	White Intelligent Power Saver	Save what you can. Isn't it more cost effective to buy one of these than to pay the electric bill?	Jingdong prefecture level city in Shandong
Fortune Shop	White Intelligent Power Saver	Received, a use do not know how, use a month to see the effect is good, the effect is good then recommended, not good then I just do not recommend.	Jingdong prefecture level city in Shandong
Fortune Shop	White Intelligent Power Saver	Just received the goods, is a voltage regulator, next month to evaluate whether it is really power saving	Jingdong prefecture level city in Shandong
Fortune Shop	White Intelligent Power Saver	Very good, used a few days obviously feel that the electricity bill is much less, wait a month to see how much can be saved!	Jingdong prefecture level city in Shandong
Fortune Shop	White Intelligent Power Saver	Received the stuff used for more than a month without a little use, it feels like it uses more electricity than before	Jingdong prefecture level city in Shandong
Fortune Shop	White Intelligent Power Saver	The quality is very good, completely exceeded the expectations, the delivery speed is very fast, the packaging is very careful and strict, very satisfied with a shopping	Jingdong prefecture level city in Shandong
Fortune Shop	White Intelligent Power Saver	Fake, doesn't work at all. Don't even sell it.	Jingdong prefecture level city in Shandong
Fortune Shop	White Intelligent Power Saver	This item doesn't seem to have much effect?	Jingdong prefecture level city in Shandong
Fortune Shop	White Intelligent Power Saver	This production is a bit fake, just look at the focus on Ming	Jingdong prefecture level city in Shandong
Fortune Shop	White Intelligent Power Saver	The logistics is fast the service attitude is very good.	Jingdong prefecture level city in Shandong

Fortune Shop	White Intelligent Power Saver	The meter is good quality, sturdy and durable. It is accurate. Easy to use. Saves a lot of things.	Jingdong prefecture level city in Shandong
Fortune Shop	White Intelligent Power Saver	Just received the goods, don't know how it is yet	Jingdong prefecture level city in Shandong
Fortune Shop	White Intelligent Power Saver	Energy saver received, logistics is very fast, the quality is very good, the appearance is also very beautiful power saving is the most important.	Jingdong prefecture level city in Shandong
Fortune Shop	White Intelligent Power Saver	It's good to talk about it, it's not practical to use it.	Jingdong prefecture level city in Shandong
Fortune Shop	White Intelligent Power Saver	I don't know if it's good for now, I'll comment after using it	Jingdong prefecture level city in Shandong
Fortune Shop	White Intelligent Power Saver	I've been using it for two or three days. It's not energy efficient.	Jingdong prefecture level city in Shandong
Fortune Shop	White Intelligent Power Saver	Packaging is intact, logistics is also fast, cost-effective, inexpensive, very satisfied with a purchase	Jingdong prefecture level city in Shandong
Fortune Shop	White Intelligent Power Saver	In use to be observed!	Jingdong prefecture level city in Shandong
Fortune Shop	White Intelligent Power Saver	Use it for a while at a flat rate	Jingdong prefecture level city in Shandong
Fortune Shop	White Intelligent Power Saver	Very trustworthy brand, quality is guaranteed, the price is still affordable, quite satisfied!	Jingdong prefecture level city in Shandong
Fortune Shop	White Intelligent Power Saver	Great quality, great operation, easy to install, great price!	Jingdong prefecture level city in Shandong
Fortune Shop	White Intelligent Power Saver	I came back and installed it, it really saves electricity, much more than before, the	Jingdong prefecture level city in Shandong
Fortune Shop	White Intelligent Power Saver	Fast logistics, good quality, very good packaging, good customer service	Jingdong prefecture level city in Shandong
Fortune Shop	White Intelligent Power Saver	Very good quality, especially good, effective, satisfactory	Jingdong prefecture level city in Shandong
Fortune Shop	White Intelligent Power Saver	I don't know how well it works.	Jingdong prefecture level city in Shandong
Fortune Shop	White Intelligent Power Saver	Good value for money, recommend it haha	Jingdong prefecture level city in Shandong
Fortune Shop	White Intelligent Power Saver	Good. Good. Very good.	Jingdong prefecture level city in Shandong
Fortune Shop	White Intelligent Power Saver	It's not working.	Jingdong prefecture level city in Shandong
Fortune Shop	White Intelligent Power Saver	It's not working.	Jingdong prefecture level city in Shandong
Fortune Shop	White Intelligent Power Saver	In use	Jingdong prefecture level city in Shandong
Fortune Shop	White Intelligent Power Saver	I'll try it later.	Jingdong prefecture level city in Shandong
Fortune Shop	White Intelligent Power Saver	It's not much use.	Jingdong prefecture level city in Shandong
Fortune Shop	White Intelligent Power Saver	It doesn't save electricity.	Jingdong prefecture level city in Shandong
Fortune Shop	White Intelligent Power Saver	It's not much use.	Jingdong prefecture level city in Shandong
Fortune Shop	White Intelligent Power Saver	Tried it for a few days and tracked the record, and there is no difference from before.	Jingdong prefecture level city in Shandong
Fortune Shop	White Intelligent Power Saver	It's not very credible, so just do it yourself and turn off the lights diligently!	Jingdong prefecture level city in Shandong
Fortune Shop	White Intelligent Power Saver	Too bad, no power saving at all, scam people don't fall for it!	Jingdong prefecture level city in Shandong
Fortune Shop	White Intelligent Power Saver	It's not working at all.	Jingdong prefecture level city in Shandong
Fortune Shop	White Intelligent Power Saver	general	Jingdong prefecture level city in Shandong
Fortune Shop	White Intelligent Power Saver	(of an unmarried couple) be close	Jingdong prefecture level city in Shandong
Fortune Shop	White Intelligent Power Saver	of no use	Jingdong prefecture level city in Shandong
Fortune Shop	White Intelligent Power Saver	Actually can really save electricity, only used half a month to save more than 50, recommended to the neighbors to use together!	Jingdong prefecture level city in Shandong
Fortune Shop	White Intelligent Power Saver	Save what you can. Isn't it more cost effective to buy one of these than to pay the electric bill?	Jingdong prefecture level city in Shandong
Fortune Shop	White Intelligent Power Saver	Received, a use do not know how, use a month to see the effect is good, the effect is good then recommended, not good then I just do not recommend.	Jingdong prefecture level city in Shandong
Fortune Shop	White Intelligent Power Saver	Just received the goods, is a voltage regulator, next month to evaluate whether it is really power saving	Jingdong prefecture level city in Shandong
Fortune Shop	White Intelligent Power Saver	Very good, used a few days obviously feel that the electricity bill is much less, wait a month to see how much can be saved!	Jingdong prefecture level city in Shandong
Fortune Shop	White Intelligent Power Saver	Received the stuff used for more than a month without a little use, it feels like it uses more electricity than before	Jingdong prefecture level city in Shandong
Fortune Shop	White Intelligent Power Saver	The quality is very good, completely exceeded the expectations, the delivery speed is very fast, the packaging is very careful and strict, very satisfied with a shopping	Jingdong prefecture level city in Shandong
Fortune Shop	White Intelligent Power Saver	Fake, doesn't work at all. Don't even sell it.	Jingdong prefecture level city in Shandong
Fortune Shop	White Intelligent Power Saver	This item doesn't seem to have much effect?	Jingdong prefecture level city in Shandong
Fortune Shop	White Intelligent Power Saver	This production is a bit fake, just look at the focus on Ming	Jingdong prefecture level city in Shandong
Fortune Shop	White Intelligent Power Saver	The logistics is fast the service attitude is very good.	Jingdong prefecture level city in Shandong
Fortune Shop	White Intelligent Power Saver	The meter is good quality, sturdy and durable. It is accurate. Easy to use. Saves a lot of things.	Jingdong prefecture level city in Shandong
Fortune Shop	White Intelligent Power Saver	Just received the goods, don't know how it is yet	Jingdong prefecture level city in Shandong
Fortune Shop	White Intelligent Power Saver	Energy saver received, logistics is very fast, the quality is very good, the appearance is also very beautiful power saving is the most important.	Jingdong prefecture level city in Shandong
Fortune Shop	White Intelligent Power Saver	It's good to talk about it, it's not practical to use it.	Jingdong prefecture level city in Shandong
Fortune Shop	White Intelligent Power Saver	I don't know if it's good for now, I'll comment after using it	Jingdong prefecture level city in Shandong
Fortune Shop	White Intelligent Power Saver	I've been using it for two or three days. It's not energy efficient.	Jingdong prefecture level city in Shandong
Fortune Shop	White Intelligent Power Saver	Packaging is intact, logistics is also fast, cost-effective, inexpensive, very satisfied with a purchase	Jingdong prefecture level city in Shandong
Fortune Shop	White Intelligent Power Saver	In use to be observed!	Jingdong prefecture level city in Shandong
Fortune Shop	White Intelligent Power Saver	Use it for a while at a flat rate	Jingdong prefecture level city in Shandong

Fortune Shop	White Intelligent Power Saver	Very trustworthy brand, quality is guaranteed, the price is still affordable, quite satisfied!	Jingdong prefecture level city in Shandong
Fortune Shop	White Intelligent Power Saver	Great quality, great operation, easy to install, great price!	Jingdong prefecture level city in Shandong
Fortune Shop	White Intelligent Power Saver	I came back and installed it, it really saves electricity, much more than before, the	Jingdong prefecture level city in Shandong
Fortune Shop	White Intelligent Power Saver	Fast logistics, good quality, very good packaging, good customer service	Jingdong prefecture level city in Shandong
Fortune Shop	White Intelligent Power Saver	Very good quality, especially good, effective, satisfactory	Jingdong prefecture level city in Shandong
Fortune Shop	White Intelligent Power Saver	I don't know how well it works.	Jingdong prefecture level city in Shandong
Fortune Shop	White Intelligent Power Saver	Good value for money, recommend it haha	Jingdong prefecture level city in Shandong
Fortune Shop	White Intelligent Power Saver	Good. Good. Very good.	Jingdong prefecture level city in Shandong
Fortune Shop	White Intelligent Power Saver	It's not working.	Jingdong prefecture level city in Shandong
Fortune Shop	White Intelligent Power Saver	It's not working.	Jingdong prefecture level city in Shandong
Fortune Shop	White Intelligent Power Saver	In use	Jingdong prefecture level city in Shandong
Fortune Shop	White Intelligent Power Saver	I'll try it later.	Jingdong prefecture level city in Shandong
Fortune Shop	White Intelligent Power Saver	It's not much use.	Jingdong prefecture level city in Shandong
Fortune Shop	White Intelligent Power Saver	It doesn't save electricity.	Jingdong prefecture level city in Shandong
Fortune Shop	White Intelligent Power Saver	It's not much use.	Jingdong prefecture level city in Shandong
Fortune Shop	White Intelligent Power Saver	Tried it for a few days and tracked the record, and there is no difference from before.	Jingdong prefecture level city in Shandong
Fortune Shop	White Intelligent Power Saver	It's not very credible, so just do it yourself and turn off the lights diligently!	Jingdong prefecture level city in Shandong
Fortune Shop	White Intelligent Power Saver	Too bad, no power saving at all, scam people don't fall for it!	Jingdong prefecture level city in Shandong
Fortune Shop	White Intelligent Power Saver	It's not working at all.	Jingdong prefecture level city in Shandong
Fortune Shop	White Intelligent Power Saver	general	Jingdong prefecture level city in Shandong
Fortune Shop	White Intelligent Power Saver	(of an unmarried couple) be close	Jingdong prefecture level city in Shandong
Fortune Shop	White Intelligent Power Saver	of no use	Jingdong prefecture level city in Shandong
Fortune Shop	White Intelligent Power Saver	Actually can really save electricity, only used half a month to save more than 50, recommended to the neighbors to use together!	Jingdong prefecture level city in Shandong
Fortune Shop	White Intelligent Power Saver	Save what you can. Isn't it more cost effective to buy one of these than to pay the electric bill?	Jingdong prefecture level city in Shandong
Fortune Shop	White Intelligent Power Saver	Received, a use do not know how, use a month to see the effect is good, the effect is good then recommended, not good then I just do not recommend.	Jingdong prefecture level city in Shandong
Fortune Shop	White Intelligent Power Saver	Just received the goods, is a voltage regulator, next month to evaluate whether it is really power saving	Jingdong prefecture level city in Shandong
Fortune Shop	White Intelligent Power Saver	Very good, used a few days obviously feel that the electricity bill is much less, wait a month to see how much can be saved!	Jingdong prefecture level city in Shandong
Fortune Shop	White Intelligent Power Saver	Received the stuff used for more than a month without a little use, it feels like it uses more electricity than before	Jingdong prefecture level city in Shandong
Fortune Shop	White Intelligent Power Saver	The quality is very good, completely exceeded the expectations, the delivery speed is very fast, the packaging is very careful and strict, very satisfied with a shopping	Jingdong prefecture level city in Shandong
Fortune Shop	White Intelligent Power Saver	Fake, doesn't work at all. Don't even sell it.	Jingdong prefecture level city in Shandong
Fortune Shop	White Intelligent Power Saver	This item doesn't seem to have much effect?	Jingdong prefecture level city in Shandong
Fortune Shop	White Intelligent Power Saver	This production is a bit fake, just look at the focus on Ming	Jingdong prefecture level city in Shandong
Fortune Shop	White Intelligent Power Saver	The logistics is fast the service attitude is very good.	Jingdong prefecture level city in Shandong
Fortune Shop	White Intelligent Power Saver	The meter is good quality, sturdy and durable. It is accurate. Easy to use. Saves a lot of things.	Jingdong prefecture level city in Shandong
Fortune Shop	White Intelligent Power Saver	Just received the goods, don't know how it is yet	Jingdong prefecture level city in Shandong
Fortune Shop	White Intelligent Power Saver	Energy saver received, logistics is very fast, the quality is very good, the appearance is also very beautiful power saving is the most important.	Jingdong prefecture level city in Shandong
Fortune Shop	White Intelligent Power Saver	It's good to talk about it, it's not practical to use it.	Jingdong prefecture level city in Shandong
Fortune Shop	White Intelligent Power Saver	I don't know if it's good for now, I'll comment after using it	Jingdong prefecture level city in Shandong
Fortune Shop	White Intelligent Power Saver	I've been using it for two or three days. It's not energy efficient.	Jingdong prefecture level city in Shandong
Fortune Shop	White Intelligent Power Saver	Packaging is intact, logistics is also fast, cost-effective, inexpensive, very satisfied with a purchase	Jingdong prefecture level city in Shandong
Fortune Shop	White Intelligent Power Saver	In use to be observed!	Jingdong prefecture level city in Shandong
Fortune Shop	White Intelligent Power Saver	Use it for a while at a flat rate	Jingdong prefecture level city in Shandong
Fortune Shop	White Intelligent Power Saver	Very trustworthy brand, quality is guaranteed, the price is still affordable, quite satisfied!	Jingdong prefecture level city in Shandong
Fortune Shop	White Intelligent Power Saver	Great quality, great operation, easy to install, great price!	Jingdong prefecture level city in Shandong
Fortune Shop	White Intelligent Power Saver	I came back and installed it, it really saves electricity, much more than before, the	Jingdong prefecture level city in Shandong
Fortune Shop	White Intelligent Power Saver	Fast logistics, good quality, very good packaging, good customer service	Jingdong prefecture level city in Shandong
Fortune Shop	White Intelligent Power Saver	Very good quality, especially good, effective, satisfactory	Jingdong prefecture level city in Shandong
Fortune Shop	White Intelligent Power Saver	I don't know how well it works.	Jingdong prefecture level city in Shandong
Fortune Shop	White Intelligent Power Saver	Good value for money, recommend it haha	Jingdong prefecture level city in Shandong
Fortune Shop	White Intelligent Power Saver	Good. Good. Very good.	Jingdong prefecture level city in Shandong
Fortune Shop	White Intelligent Power Saver	It's not working.	Jingdong prefecture level city in Shandong

Fortune Shop	White Intelligent Power Saver	It's not working.	Jingdong prefecture level city in Shandong
Fortune Shop	White Intelligent Power Saver	In use	Jingdong prefecture level city in Shandong
Fortune Shop	White Intelligent Power Saver	I'll try it later.	Jingdong prefecture level city in Shandong
Fortune Shop	White Intelligent Power Saver	It's not much use.	Jingdong prefecture level city in Shandong
Fortune Shop	White Intelligent Power Saver	It doesn't save electricity.	Jingdong prefecture level city in Shandong
Fortune Shop	White Intelligent Power Saver	It's not much use.	Jingdong prefecture level city in Shandong
Fortune Shop	White Intelligent Power Saver	Tried it for a few days and tracked the record, and there is no difference from before.	Jingdong prefecture level city in Shandong
Fortune Shop	White Intelligent Power Saver	It's not very credible, so just do it yourself and turn off the lights diligently!	Jingdong prefecture level city in Shandong
Fortune Shop	White Intelligent Power Saver	Too bad, no power saving at all, scam people don't fall for it!	Jingdong prefecture level city in Shandong
Fortune Shop	White Intelligent Power Saver	It's not working at all.	Jingdong prefecture level city in Shandong
Fortune Shop	White Intelligent Power Saver	general	Jingdong prefecture level city in Shandong
Fortune Shop	White Intelligent Power Saver	(of an unmarried couple) be close	Jingdong prefecture level city in Shandong
Fortune Shop	White Intelligent Power Saver	of no use	Jingdong prefecture level city in Shandong
Fortune Shop	White Intelligent Power Saver	Actually can really save electricity, only used half a month to save more than 50, recommended to the neighbors to use together!	Jingdong prefecture level city in Shandong
Fortune Shop	White Intelligent Power Saver	Save what you can. Isn't it more cost effective to buy one of these than to pay the electric bill?	Jingdong prefecture level city in Shandong
Fortune Shop	White Intelligent Power Saver	Received, a use do not know how, use a month to see the effect is good, the effect is good then recommended, not good then I just do not recommend.	Jingdong prefecture level city in Shandong
Fortune Shop	White Intelligent Power Saver	Just received the goods, is a voltage regulator, next month to evaluate whether it is really power saving	Jingdong prefecture level city in Shandong
Fortune Shop	White Intelligent Power Saver	Very good, used a few days obviously feel that the electricity bill is much less, wait a month to see how much can be saved!	Jingdong prefecture level city in Shandong
Fortune Shop	White Intelligent Power Saver	Received the stuff used for more than a month without a little use, it feels like it uses more electricity than before	Jingdong prefecture level city in Shandong
Fortune Shop	White Intelligent Power Saver	The quality is very good, completely exceeded the expectations, the delivery speed is very fast, the packaging is very careful and strict, very satisfied with a shopping	Jingdong prefecture level city in Shandong
Fortune Shop	White Intelligent Power Saver	Fake, doesn't work at all. Don't even sell it.	Jingdong prefecture level city in Shandong
Fortune Shop	White Intelligent Power Saver	This item doesn't seem to have much effect?	Jingdong prefecture level city in Shandong
Fortune Shop	White Intelligent Power Saver	This production is a bit fake, just look at the focus on Ming	Jingdong prefecture level city in Shandong
Fortune Shop	White Intelligent Power Saver	The logistics is fast the service attitude is very good.	Jingdong prefecture level city in Shandong
Fortune Shop	White Intelligent Power Saver	The meter is good quality, sturdy and durable. It is accurate. Easy to use. Saves a lot of things.	Jingdong prefecture level city in Shandong
Fortune Shop	White Intelligent Power Saver	Just received the goods, don't know how it is yet	Jingdong prefecture level city in Shandong
Fortune Shop	White Intelligent Power Saver	Energy saver received, logistics is very fast, the quality is very good, the appearance is also very beautiful power saving is the most important.	Jingdong prefecture level city in Shandong
Fortune Shop	White Intelligent Power Saver	It's good to talk about it, it's not practical to use it.	Jingdong prefecture level city in Shandong
Fortune Shop	White Intelligent Power Saver	I don't know if it's good for now, I'll comment after using it	Jingdong prefecture level city in Shandong
Fortune Shop	White Intelligent Power Saver	I've been using it for two or three days. It's not energy efficient.	Jingdong prefecture level city in Shandong
Fortune Shop	White Intelligent Power Saver	Packaging is intact, logistics is also fast, cost-effective, inexpensive, very satisfied with a purchase	Jingdong prefecture level city in Shandong
Fortune Shop	White Intelligent Power Saver	In use to be observed!	Jingdong prefecture level city in Shandong
Fortune Shop	White Intelligent Power Saver	Use it for a while at a flat rate	Jingdong prefecture level city in Shandong
Fortune Shop	White Intelligent Power Saver	Very trustworthy brand, quality is guaranteed, the price is still affordable, quite satisfied!	Jingdong prefecture level city in Shandong
Fortune Shop	White Intelligent Power Saver	Great quality, great operation, easy to install, great price!	Jingdong prefecture level city in Shandong
Fortune Shop	White Intelligent Power Saver	I came back and installed it, it really saves electricity, much more than before, the	Jingdong prefecture level city in Shandong
Fortune Shop	White Intelligent Power Saver	Fast logistics, good quality, very good packaging, good customer service	Jingdong prefecture level city in Shandong
Fortune Shop	White Intelligent Power Saver	Very good quality, especially good, effective, satisfactory	Jingdong prefecture level city in Shandong
Fortune Shop	White Intelligent Power Saver	I don't know how well it works.	Jingdong prefecture level city in Shandong
Fortune Shop	White Intelligent Power Saver	Good value for money, recommend it haha	Jingdong prefecture level city in Shandong
Fortune Shop	White Intelligent Power Saver	Good. Good. Very good.	Jingdong prefecture level city in Shandong
Fortune Shop	White Intelligent Power Saver	It's not working.	Jingdong prefecture level city in Shandong
Fortune Shop	White Intelligent Power Saver	It's not working.	Jingdong prefecture level city in Shandong
Fortune Shop	White Intelligent Power Saver	In use	Jingdong prefecture level city in Shandong
Fortune Shop	White Intelligent Power Saver	I'll try it later.	Jingdong prefecture level city in Shandong
Fortune Shop	White Intelligent Power Saver	It's not much use.	Jingdong prefecture level city in Shandong
Fortune Shop	White Intelligent Power Saver	It doesn't save electricity.	Jingdong prefecture level city in Shandong
Fortune Shop	White Intelligent Power Saver	It's not much use.	Jingdong prefecture level city in Shandong
Fortune Shop	White Intelligent Power Saver	Tried it for a few days and tracked the record, and there is no difference from before.	Jingdong prefecture level city in Shandong
Fortune Shop	White Intelligent Power Saver	It's not very credible, so just do it yourself and turn off the lights diligently!	Jingdong prefecture level city in Shandong
Fortune Shop	White Intelligent Power Saver	Too bad, no power saving at all, scam people don't fall for it!	Jingdong prefecture level city in Shandong

Fortune Shop	White Intelligent Power Saver	It's not working at all.	Jingdong prefecture level city in Shandong
Fortune Shop	White Intelligent Power Saver	general	Jingdong prefecture level city in Shandong
Fortune Shop	White Intelligent Power Saver	(of an unmarried couple) be close	Jingdong prefecture level city in Shandong
Fortune Shop	White Intelligent Power Saver	of no use	Jingdong prefecture level city in Shandong
Fortune Shop	White Intelligent Power Saver	Actually can really save electricity, only used half a month to save more than 50, recommended to the neighbors to use together!	Jingdong prefecture level city in Shandong
Fortune Shop	White Intelligent Power Saver	Save what you can. Isn't it more cost effective to buy one of these than to pay the electric bill?	Jingdong prefecture level city in Shandong
Fortune Shop	White Intelligent Power Saver	Received, a use do not know how, use a month to see the effect is good, the effect is good then recommended, not good then I just do not recommend.	Jingdong prefecture level city in Shandong
Fortune Shop	White Intelligent Power Saver	Just received the goods, is a voltage regulator, next month to evaluate whether it is really power saving	Jingdong prefecture level city in Shandong
Fortune Shop	White Intelligent Power Saver	Very good, used a few days obviously feel that the electricity bill is much less, wait a month to see how much can be saved!	Jingdong prefecture level city in Shandong
Fortune Shop	White Intelligent Power Saver	Received the stuff used for more than a month without a little use, it feels like it uses more electricity than before	Jingdong prefecture level city in Shandong
Fortune Shop	White Intelligent Power Saver	The quality is very good, completely exceeded the expectations, the delivery speed is very fast, the packaging is very careful and strict, very satisfied with a shopping	Jingdong prefecture level city in Shandong
Fortune Shop	White Intelligent Power Saver	Fake, doesn't work at all. Don't even sell it.	Jingdong prefecture level city in Shandong
Fortune Shop	White Intelligent Power Saver	This item doesn't seem to have much effect?	Jingdong prefecture level city in Shandong
Fortune Shop	White Intelligent Power Saver	This production is a bit fake, just look at the focus on Ming	Jingdong prefecture level city in Shandong
Fortune Shop	White Intelligent Power Saver	The logistics is fast the service attitude is very good.	Jingdong prefecture level city in Shandong
Fortune Shop	White Intelligent Power Saver	The meter is good quality, sturdy and durable. It is accurate. Easy to use. Saves a lot of things.	Jingdong prefecture level city in Shandong
Fortune Shop	White Intelligent Power Saver	Just received the goods, don't know how it is yet	Jingdong prefecture level city in Shandong
Fortune Shop	White Intelligent Power Saver	Energy saver received, logistics is very fast, the quality is very good, the appearance is also very beautiful power saving is the most important.	Jingdong prefecture level city in Shandong
Fortune Shop	White Intelligent Power Saver	It's good to talk about it, it's not practical to use it.	Jingdong prefecture level city in Shandong
Fortune Shop	White Intelligent Power Saver	I don't know if it's good for now, I'll comment after using it	Jingdong prefecture level city in Shandong
Fortune Shop	White Intelligent Power Saver	I've been using it for two or three days. It's not energy efficient.	Jingdong prefecture level city in Shandong
Fortune Shop	White Intelligent Power Saver	Packaging is intact, logistics is also fast, cost-effective, inexpensive, very satisfied with a purchase	Jingdong prefecture level city in Shandong
Fortune Shop	White Intelligent Power Saver	In use to be observed!	Jingdong prefecture level city in Shandong
Fortune Shop	White Intelligent Power Saver	Use it for a while at a flat rate	Jingdong prefecture level city in Shandong
Fortune Shop	White Intelligent Power Saver	Very trustworthy brand, quality is guaranteed, the price is still affordable, quite satisfied!	Jingdong prefecture level city in Shandong
Fortune Shop	White Intelligent Power Saver	Great quality, great operation, easy to install, great price!	Jingdong prefecture level city in Shandong
Fortune Shop	White Intelligent Power Saver	I came back and installed it, it really saves electricity, much more than before, the	Jingdong prefecture level city in Shandong
Fortune Shop	White Intelligent Power Saver	Fast logistics, good quality, very good packaging, good customer service	Jingdong prefecture level city in Shandong
Fortune Shop	White Intelligent Power Saver	Very good quality, especially good, effective, satisfactory	Jingdong prefecture level city in Shandong
Fortune Shop	White Intelligent Power Saver	I don't know how well it works.	Jingdong prefecture level city in Shandong
Fortune Shop	White Intelligent Power Saver	Good value for money, recommend it haha	Jingdong prefecture level city in Shandong
Fortune Shop	White Intelligent Power Saver	Good. Good. Very good.	Jingdong prefecture level city in Shandong
Fortune Shop	White Intelligent Power Saver	It's not working.	Jingdong prefecture level city in Shandong
Fortune Shop	White Intelligent Power Saver	It's not working.	Jingdong prefecture level city in Shandong
Fortune Shop	White Intelligent Power Saver	In use	Jingdong prefecture level city in Shandong
Fortune Shop	White Intelligent Power Saver	I'll try it later.	Jingdong prefecture level city in Shandong
Fortune Shop	White Intelligent Power Saver	It's not much use.	Jingdong prefecture level city in Shandong
Fortune Shop	White Intelligent Power Saver	It doesn't save electricity.	Jingdong prefecture level city in Shandong
Fortune Shop	White Intelligent Power Saver	It's not much use.	Jingdong prefecture level city in Shandong
Fortune Shop	White Intelligent Power Saver	Tried it for a few days and tracked the record, and there is no difference from before.	Jingdong prefecture level city in Shandong
Fortune Shop	White Intelligent Power Saver	It's not very credible, so just do it yourself and turn off the lights diligently!	Jingdong prefecture level city in Shandong
Fortune Shop	White Intelligent Power Saver	Too bad, no power saving at all, scam people don't fall for it!	Jingdong prefecture level city in Shandong
Fortune Shop	White Intelligent Power Saver	It's not working at all.	Jingdong prefecture level city in Shandong
Fortune Shop	White Intelligent Power Saver	general	Jingdong prefecture level city in Shandong
Fortune Shop	White Intelligent Power Saver	(of an unmarried couple) be close	Jingdong prefecture level city in Shandong
Fortune Shop	White Intelligent Power Saver	of no use	Jingdong prefecture level city in Shandong
Fortune Shop	White Intelligent Power Saver	Actually can really save electricity, only used half a month to save more than 50, recommended to the neighbors to use together!	Jingdong prefecture level city in Shandong
Fortune Shop	White Intelligent Power Saver	Save what you can. Isn't it more cost effective to buy one of these than to pay the electric bill?	Jingdong prefecture level city in Shandong
Fortune Shop	White Intelligent Power Saver	Received, a use do not know how, use a month to see the effect is good, the effect is good then recommended, not good then I just do not recommend.	Jingdong prefecture level city in Shandong
Fortune Shop	White Intelligent Power Saver	Just received the goods, is a voltage regulator, next month to evaluate whether it is really power saving	Jingdong prefecture level city in Shandong
Fortune Shop	White Intelligent Power Saver	Very good, used a few days obviously feel that the electricity bill is much less, wait a month to see how much can be saved!	Jingdong prefecture level city in Shandong

Fortune Shop	White Intelligent Power Saver	Received the stuff used for more than a month without a little use, it feels like it uses more electricity than before	Jingdong prefecture level city in Shandong
Fortune Shop	White Intelligent Power Saver	The quality is very good, completely exceeded the expectations, the delivery speed is very fast, the packaging is very careful and strict, very satisfied with a shopping	Jingdong prefecture level city in Shandong
Fortune Shop	White Intelligent Power Saver	Fake, doesn't work at all. Don't even sell it.	Jingdong prefecture level city in Shandong
Fortune Shop	White Intelligent Power Saver	This item doesn't seem to have much effect?	Jingdong prefecture level city in Shandong
Fortune Shop	White Intelligent Power Saver	This production is a bit fake, just look at the focus on Ming	Jingdong prefecture level city in Shandong
Fortune Shop	White Intelligent Power Saver	The logistics is fast the service attitude is very good.	Jingdong prefecture level city in Shandong
Fortune Shop	White Intelligent Power Saver	The meter is good quality, sturdy and durable. It is accurate. Easy to use. Saves a lot of things.	Jingdong prefecture level city in Shandong
Fortune Shop	White Intelligent Power Saver	Just received the goods, don't know how it is yet	Jingdong prefecture level city in Shandong
Fortune Shop	White Intelligent Power Saver	Energy saver received, logistics is very fast, the quality is very good, the appearance is also very beautiful power saving is the most important.	Jingdong prefecture level city in Shandong
Fortune Shop	White Intelligent Power Saver	It's good to talk about it, it's not practical to use it.	Jingdong prefecture level city in Shandong
Fortune Shop	White Intelligent Power Saver	I don't know if it's good for now, I'll comment after using it	Jingdong prefecture level city in Shandong
Fortune Shop	White Intelligent Power Saver	I've been using it for two or three days. It's not energy efficient.	Jingdong prefecture level city in Shandong
Fortune Shop	White Intelligent Power Saver	Packaging is intact, logistics is also fast, cost-effective, inexpensive, very satisfied with a purchase	Jingdong prefecture level city in Shandong
Fortune Shop	White Intelligent Power Saver	In use to be observed!	Jingdong prefecture level city in Shandong
Fortune Shop	White Intelligent Power Saver	Use it for a while at a flat rate	Jingdong prefecture level city in Shandong
Fortune Shop	White Intelligent Power Saver	Very trustworthy brand, quality is guaranteed, the price is still affordable, quite satisfied!	Jingdong prefecture level city in Shandong
Fortune Shop	White Intelligent Power Saver	Great quality, great operation, easy to install, great price!	Jingdong prefecture level city in Shandong
Fortune Shop	White Intelligent Power Saver	I came back and installed it, it really saves electricity, much more than before, the	Jingdong prefecture level city in Shandong
Fortune Shop	White Intelligent Power Saver	Fast logistics, good quality, very good packaging, good customer service	Jingdong prefecture level city in Shandong
Fortune Shop	White Intelligent Power Saver	Very good quality, especially good, effective, satisfactory	Jingdong prefecture level city in Shandong
Fortune Shop	White Intelligent Power Saver	I don't know how well it works.	Jingdong prefecture level city in Shandong
Fortune Shop	White Intelligent Power Saver	Good value for money, recommend it haha	Jingdong prefecture level city in Shandong
Fortune Shop	White Intelligent Power Saver	Good. Good. Very good.	Jingdong prefecture level city in Shandong
Fortune Shop	White Intelligent Power Saver	It's not working.	Jingdong prefecture level city in Shandong
Fortune Shop	White Intelligent Power Saver	It's not working.	Jingdong prefecture level city in Shandong
Fortune Shop	White Intelligent Power Saver	In use	Jingdong prefecture level city in Shandong
Fortune Shop	White Intelligent Power Saver	I'll try it later.	Jingdong prefecture level city in Shandong
Fortune Shop	White Intelligent Power Saver	It's not much use.	Jingdong prefecture level city in Shandong
Fortune Shop	White Intelligent Power Saver	It doesn't save electricity.	Jingdong prefecture level city in Shandong
Fortune Shop	White Intelligent Power Saver	It's not much use.	Jingdong prefecture level city in Shandong
Fortune Shop	White Intelligent Power Saver	Tried it for a few days and tracked the record, and there is no difference from before.	Jingdong prefecture level city in Shandong
Fortune Shop	White Intelligent Power Saver	It's not very credible, so just do it yourself and turn off the lights diligently!	Jingdong prefecture level city in Shandong
Fortune Shop	White Intelligent Power Saver	Too bad, no power saving at all, scam people don't fall for it!	Jingdong prefecture level city in Shandong
Fortune Shop	White Intelligent Power Saver	It's not working at all.	Jingdong prefecture level city in Shandong
Fortune Shop	White Intelligent Power Saver	general	Jingdong prefecture level city in Shandong
Fortune Shop	White Intelligent Power Saver	(of an unmarried couple) be close	Jingdong prefecture level city in Shandong
Fortune Shop	White Intelligent Power Saver	of no use	Jingdong prefecture level city in Shandong
Fortune Shop	White Intelligent Power Saver	Actually can really save electricity, only used half a month to save more than 50, recommended to the neighbors to use together!	Jingdong prefecture level city in Shandong
Fortune Shop	White Intelligent Power Saver	Save what you can. Isn't it more cost effective to buy one of these than to pay the electric bill?	Jingdong prefecture level city in Shandong
Fortune Shop	White Intelligent Power Saver	Received, a use do not know how, use a month to see the effect is good, the effect is good then recommended, not good then I just do not recommend.	Jingdong prefecture level city in Shandong
Fortune Shop	White Intelligent Power Saver	Just received the goods, is a voltage regulator, next month to evaluate whether it is really power saving	Jingdong prefecture level city in Shandong
Fortune Shop	White Intelligent Power Saver	Very good, used a few days obviously feel that the electricity bill is much less, wait a month to see how much can be saved!	Jingdong prefecture level city in Shandong
Fortune Shop	White Intelligent Power Saver	Received the stuff used for more than a month without a little use, it feels like it uses more electricity than before	Jingdong prefecture level city in Shandong
Fortune Shop	White Intelligent Power Saver	The quality is very good, completely exceeded the expectations, the delivery speed is very fast, the packaging is very careful and strict, very satisfied with a shopping	Jingdong prefecture level city in Shandong
Fortune Shop	White Intelligent Power Saver	Fake, doesn't work at all. Don't even sell it.	Jingdong prefecture level city in Shandong
Fortune Shop	White Intelligent Power Saver	This item doesn't seem to have much effect?	Jingdong prefecture level city in Shandong
Fortune Shop	White Intelligent Power Saver	This production is a bit fake, just look at the focus on Ming	Jingdong prefecture level city in Shandong
Fortune Shop	White Intelligent Power Saver	The logistics is fast the service attitude is very good.	Jingdong prefecture level city in Shandong
Fortune Shop	White Intelligent Power Saver	The meter is good quality, sturdy and durable. It is accurate. Easy to use. Saves a lot of things.	Jingdong prefecture level city in Shandong
Fortune Shop	White Intelligent Power Saver	Just received the goods, don't know how it is yet	Jingdong prefecture level city in Shandong
Fortune Shop	White Intelligent Power Saver	Energy saver received, logistics is very fast, the quality is very good, the appearance is also very beautiful power saving is the most important.	Jingdong prefecture level city in Shandong

Fortune Shop	White Intelligent Power Saver	It's good to talk about it, it's not practical to use it.	Jingdong prefecture level city in Shandong
Fortune Shop	White Intelligent Power Saver	I don't know if it's good for now, I'll comment after using it	Jingdong prefecture level city in Shandong
Fortune Shop	White Intelligent Power Saver	I've been using it for two or three days. It's not energy efficient.	Jingdong prefecture level city in Shandong
Fortune Shop	White Intelligent Power Saver	Packaging is intact, logistics is also fast, cost-effective, inexpensive, very satisfied with a purchase	Jingdong prefecture level city in Shandong
Fortune Shop	White Intelligent Power Saver	In use to be observed!	Jingdong prefecture level city in Shandong
Fortune Shop	White Intelligent Power Saver	Use it for a while at a flat rate	Jingdong prefecture level city in Shandong
Fortune Shop	White Intelligent Power Saver	Very trustworthy brand, quality is guaranteed, the price is still affordable, quite satisfied!	Jingdong prefecture level city in Shandong
Fortune Shop	White Intelligent Power Saver	Great quality, great operation, easy to install, great price!	Jingdong prefecture level city in Shandong
Fortune Shop	White Intelligent Power Saver	I came back and installed it, it really saves electricity, much more than before, the	Jingdong prefecture level city in Shandong
Fortune Shop	White Intelligent Power Saver	Fast logistics, good quality, very good packaging, good customer service	Jingdong prefecture level city in Shandong
Fortune Shop	White Intelligent Power Saver	Very good quality, especially good, effective, satisfactory	Jingdong prefecture level city in Shandong
Fortune Shop	White Intelligent Power Saver	I don't know how well it works.	Jingdong prefecture level city in Shandong
Fortune Shop	White Intelligent Power Saver	Good value for money, recommend it haha	Jingdong prefecture level city in Shandong
Fortune Shop	White Intelligent Power Saver	Good. Good. Very good.	Jingdong prefecture level city in Shandong
Fortune Shop	White Intelligent Power Saver	It's not working.	Jingdong prefecture level city in Shandong
Fortune Shop	White Intelligent Power Saver	It's not working.	Jingdong prefecture level city in Shandong
Fortune Shop	White Intelligent Power Saver	In use	Jingdong prefecture level city in Shandong
Fortune Shop	White Intelligent Power Saver	I'll try it later.	Jingdong prefecture level city in Shandong
Fortune Shop	White Intelligent Power Saver	It's not much use.	Jingdong prefecture level city in Shandong
Fortune Shop	White Intelligent Power Saver	It doesn't save electricity.	Jingdong prefecture level city in Shandong
Fortune Shop	White Intelligent Power Saver	It's not much use.	Jingdong prefecture level city in Shandong
Fortune Shop	White Intelligent Power Saver	Tried it for a few days and tracked the record, and there is no difference from before.	Jingdong prefecture level city in Shandong
Fortune Shop	White Intelligent Power Saver	It's not very credible, so just do it yourself and turn off the lights diligently!	Jingdong prefecture level city in Shandong
Fortune Shop	White Intelligent Power Saver	Too bad, no power saving at all, scam people don't fall for it!	Jingdong prefecture level city in Shandong
Fortune Shop	White Intelligent Power Saver	It's not working at all.	Jingdong prefecture level city in Shandong
Fortune Shop	White Intelligent Power Saver	general	Jingdong prefecture level city in Shandong
Fortune Shop	White Intelligent Power Saver	(of an unmarried couple) be close	Jingdong prefecture level city in Shandong
Fortune Shop	White Intelligent Power Saver	of no use	Jingdong prefecture level city in Shandong
Fortune Shop	White Intelligent Power Saver	Actually can really save electricity, only used half a month to save more than 50, recommended to the neighbors to use together!	Jingdong prefecture level city in Shandong
Fortune Shop	White Intelligent Power Saver	Save what you can. Isn't it more cost effective to buy one of these than to pay the electric bill?	Jingdong prefecture level city in Shandong
Fortune Shop	White Intelligent Power Saver	Received, a use do not know how, use a month to see the effect is good, the effect is good then recommended, not good then I just do not recommend.	Jingdong prefecture level city in Shandong
Fortune Shop	White Intelligent Power Saver	Just received the goods, is a voltage regulator, next month to evaluate whether it is really power saving	Jingdong prefecture level city in Shandong
Fortune Shop	White Intelligent Power Saver	Very good, used a few days obviously feel that the electricity bill is much less, wait a month to see how much can be saved!	Jingdong prefecture level city in Shandong
Fortune Shop	White Intelligent Power Saver	Received the stuff used for more than a month without a little use, it feels like it uses more electricity than before	Jingdong prefecture level city in Shandong
Fortune Shop	White Intelligent Power Saver	The quality is very good, completely exceeded the expectations, the delivery speed is very fast, the packaging is very careful and strict, very satisfied with a shopping	Jingdong prefecture level city in Shandong
Fortune Shop	White Intelligent Power Saver	Fake, doesn't work at all. Don't even sell it.	Jingdong prefecture level city in Shandong
Fortune Shop	White Intelligent Power Saver	This item doesn't seem to have much effect?	Jingdong prefecture level city in Shandong
Fortune Shop	White Intelligent Power Saver	This production is a bit fake, just look at the focus on Ming	Jingdong prefecture level city in Shandong
Fortune Shop	White Intelligent Power Saver	The logistics is fast the service attitude is very good.	Jingdong prefecture level city in Shandong
Fortune Shop	White Intelligent Power Saver	The meter is good quality, sturdy and durable. It is accurate. Easy to use. Saves a lot of things.	Jingdong prefecture level city in Shandong
Fortune Shop	White Intelligent Power Saver	Just received the goods, don't know how it is yet	Jingdong prefecture level city in Shandong
Fortune Shop	White Intelligent Power Saver	Energy saver received, logistics is very fast, the quality is very good, the appearance is also very beautiful power saving is the most important.	Jingdong prefecture level city in Shandong
Fortune Shop	White Intelligent Power Saver	It's good to talk about it, it's not practical to use it.	Jingdong prefecture level city in Shandong
Fortune Shop	White Intelligent Power Saver	I don't know if it's good for now, I'll comment after using it	Jingdong prefecture level city in Shandong
Fortune Shop	White Intelligent Power Saver	I've been using it for two or three days. It's not energy efficient.	Jingdong prefecture level city in Shandong
Fortune Shop	White Intelligent Power Saver	Packaging is intact, logistics is also fast, cost-effective, inexpensive, very satisfied with a purchase	Jingdong prefecture level city in Shandong
Fortune Shop	White Intelligent Power Saver	In use to be observed!	Jingdong prefecture level city in Shandong
Fortune Shop	White Intelligent Power Saver	Use it for a while at a flat rate	Jingdong prefecture level city in Shandong
Fortune Shop	White Intelligent Power Saver	Very trustworthy brand, quality is guaranteed, the price is still affordable, quite satisfied!	Jingdong prefecture level city in Shandong
Fortune Shop	White Intelligent Power Saver	Great quality, great operation, easy to install, great price!	Jingdong prefecture level city in Shandong
Fortune Shop	White Intelligent Power Saver	I came back and installed it, it really saves electricity, much more than before, the	Jingdong prefecture level city in Shandong

Fortune Shop	White Intelligent Power Saver	Fast logistics, good quality, very good packaging, good customer service	Jingdong prefecture level city in Shandong
Fortune Shop	White Intelligent Power Saver	Very good quality, especially good, effective, satisfactory	Jingdong prefecture level city in Shandong
Fortune Shop	White Intelligent Power Saver	I don't know how well it works.	Jingdong prefecture level city in Shandong
Fortune Shop	White Intelligent Power Saver	Good value for money, recommend it haha	Jingdong prefecture level city in Shandong
Fortune Shop	White Intelligent Power Saver	Good. Good. Very good.	Jingdong prefecture level city in Shandong
Fortune Shop	White Intelligent Power Saver	It's not working.	Jingdong prefecture level city in Shandong
Fortune Shop	White Intelligent Power Saver	It's not working.	Jingdong prefecture level city in Shandong
Fortune Shop	White Intelligent Power Saver	In use	Jingdong prefecture level city in Shandong
Fortune Shop	White Intelligent Power Saver	I'll try it later.	Jingdong prefecture level city in Shandong
Fortune Shop	White Intelligent Power Saver	It's not much use.	Jingdong prefecture level city in Shandong
Fortune Shop	White Intelligent Power Saver	It doesn't save electricity.	Jingdong prefecture level city in Shandong
Fortune Shop	White Intelligent Power Saver	It's not much use.	Jingdong prefecture level city in Shandong
Fortune Shop	White Intelligent Power Saver	Tried it for a few days and tracked the record, and there is no difference from before.	Jingdong prefecture level city in Shandong
Fortune Shop	White Intelligent Power Saver	It's not very credible, so just do it yourself and turn off the lights diligently!	Jingdong prefecture level city in Shandong
Fortune Shop	White Intelligent Power Saver	Too bad, no power saving at all, scam people don't fall for it!	Jingdong prefecture level city in Shandong
Fortune Shop	White Intelligent Power Saver	It's not working at all.	Jingdong prefecture level city in Shandong
Fortune Shop	White Intelligent Power Saver	general	Jingdong prefecture level city in Shandong
Fortune Shop	White Intelligent Power Saver	(of an unmarried couple) be close	Jingdong prefecture level city in Shandong
Fortune Shop	White Intelligent Power Saver	of no use	Jingdong prefecture level city in Shandong
Fortune Shop	White Intelligent Power Saver	Actually can really save electricity, only used half a month to save more than 50, recommended to the neighbors to use together!	Jingdong prefecture level city in Shandong
Fortune Shop	White Intelligent Power Saver	Save what you can. Isn't it more cost effective to buy one of these than to pay the electric bill?	Jingdong prefecture level city in Shandong
Fortune Shop	White Intelligent Power Saver	Received, a use do not know how, use a month to see the effect is good, the effect is good then recommended, not good then I just do not recommend.	Jingdong prefecture level city in Shandong
Fortune Shop	White Intelligent Power Saver	Just received the goods, is a voltage regulator, next month to evaluate whether it is really power saving	Jingdong prefecture level city in Shandong
Fortune Shop	White Intelligent Power Saver	Very good, used a few days obviously feel that the electricity bill is much less, wait a month to see how much can be saved!	Jingdong prefecture level city in Shandong
Fortune Shop	White Intelligent Power Saver	Received the stuff used for more than a month without a little use, it feels like it uses more electricity than before	Jingdong prefecture level city in Shandong
Fortune Shop	White Intelligent Power Saver	The quality is very good, completely exceeded the expectations, the delivery speed is very fast, the packaging is very careful and strict, very satisfied with a shopping	Jingdong prefecture level city in Shandong
Fortune Shop	White Intelligent Power Saver	Fake, doesn't work at all. Don't even sell it.	Jingdong prefecture level city in Shandong
Fortune Shop	White Intelligent Power Saver	This item doesn't seem to have much effect?	Jingdong prefecture level city in Shandong
Fortune Shop	White Intelligent Power Saver	This production is a bit fake, just look at the focus on Ming	Jingdong prefecture level city in Shandong
Fortune Shop	White Intelligent Power Saver	The logistics is fast the service attitude is very good.	Jingdong prefecture level city in Shandong
Fortune Shop	White Intelligent Power Saver	The meter is good quality, sturdy and durable. It is accurate. Easy to use. Saves a lot of things.	Jingdong prefecture level city in Shandong
Fortune Shop	White Intelligent Power Saver	Just received the goods, don't know how it is yet	Jingdong prefecture level city in Shandong
Fortune Shop	White Intelligent Power Saver	Energy saver received, logistics is very fast, the quality is very good, the appearance is also very beautiful power saving is the most important.	Jingdong prefecture level city in Shandong
Fortune Shop	White Intelligent Power Saver	It's good to talk about it, it's not practical to use it.	Jingdong prefecture level city in Shandong
Fortune Shop	White Intelligent Power Saver	I don't know if it's good for now, I'll comment after using it	Jingdong prefecture level city in Shandong
Fortune Shop	White Intelligent Power Saver	I've been using it for two or three days. It's not energy efficient.	Jingdong prefecture level city in Shandong
Fortune Shop	White Intelligent Power Saver	Packaging is intact, logistics is also fast, cost-effective, inexpensive, very satisfied with a purchase	Jingdong prefecture level city in Shandong
Fortune Shop	White Intelligent Power Saver	In use to be observed!	Jingdong prefecture level city in Shandong
Fortune Shop	White Intelligent Power Saver	Use it for a while at a flat rate	Jingdong prefecture level city in Shandong
Fortune Shop	White Intelligent Power Saver	Very trustworthy brand, quality is guaranteed, the price is still affordable, quite satisfied!	Jingdong prefecture level city in Shandong
Fortune Shop	White Intelligent Power Saver	Great quality, great operation, easy to install, great price!	Jingdong prefecture level city in Shandong
Fortune Shop	White Intelligent Power Saver	I came back and installed it, it really saves electricity, much more than before, the	Jingdong prefecture level city in Shandong
Fortune Shop	White Intelligent Power Saver	Fast logistics, good quality, very good packaging, good customer service	Jingdong prefecture level city in Shandong
Fortune Shop	White Intelligent Power Saver	Very good quality, especially good, effective, satisfactory	Jingdong prefecture level city in Shandong
Fortune Shop	White Intelligent Power Saver	I don't know how well it works.	Jingdong prefecture level city in Shandong
Fortune Shop	White Intelligent Power Saver	Good value for money, recommend it haha	Jingdong prefecture level city in Shandong
Fortune Shop	White Intelligent Power Saver	Good. Good. Very good.	Jingdong prefecture level city in Shandong
Fortune Shop	White Intelligent Power Saver	It's not working.	Jingdong prefecture level city in Shandong
Fortune Shop	White Intelligent Power Saver	It's not working.	Jingdong prefecture level city in Shandong
Fortune Shop	White Intelligent Power Saver	In use	Jingdong prefecture level city in Shandong
Fortune Shop	White Intelligent Power Saver	I'll try it later.	Jingdong prefecture level city in Shandong

Fortune Shop	White Intelligent Power Saver	It's not much use.	Jingdong prefecture level city in Shandong
Fortune Shop	White Intelligent Power Saver	It doesn't save electricity.	Jingdong prefecture level city in Shandong
Fortune Shop	White Intelligent Power Saver	It's not much use.	Jingdong prefecture level city in Shandong
Fortune Shop	White Intelligent Power Saver	Tried it for a few days and tracked the record, and there is no difference from before.	Jingdong prefecture level city in Shandong
Fortune Shop	White Intelligent Power Saver	It's not very credible, so just do it yourself and turn off the lights diligently!	Jingdong prefecture level city in Shandong
Fortune Shop	White Intelligent Power Saver	Too bad, no power saving at all, scam people don't fall for it!	Jingdong prefecture level city in Shandong
Fortune Shop	White Intelligent Power Saver	It's not working at all.	Jingdong prefecture level city in Shandong
Fortune Shop	White Intelligent Power Saver	general	Jingdong prefecture level city in Shandong
Fortune Shop	White Intelligent Power Saver	(of an unmarried couple) be close	Jingdong prefecture level city in Shandong
Fortune Shop	White Intelligent Power Saver	of no use	Jingdong prefecture level city in Shandong
Fortune Shop	White Intelligent Power Saver	Actually can really save electricity, only used half a month to save more than 50, recommended to the neighbors to use together!	Jingdong prefecture level city in Shandong
Fortune Shop	White Intelligent Power Saver	Save what you can. Isn't it more cost effective to buy one of these than to pay the electric bill?	Jingdong prefecture level city in Shandong
Fortune Shop	White Intelligent Power Saver	Received, a use do not know how, use a month to see the effect is good, the effect is good then recommended, not good then I just do not recommend.	Jingdong prefecture level city in Shandong
Fortune Shop	White Intelligent Power Saver	Just received the goods, is a voltage regulator, next month to evaluate whether it is really power saving	Jingdong prefecture level city in Shandong
Fortune Shop	White Intelligent Power Saver	Very good, used a few days obviously feel that the electricity bill is much less, wait a month to see how much can be saved!	Jingdong prefecture level city in Shandong
Fortune Shop	White Intelligent Power Saver	Received the stuff used for more than a month without a little use, it feels like it uses more electricity than before	Jingdong prefecture level city in Shandong
Fortune Shop	White Intelligent Power Saver	The quality is very good, completely exceeded the expectations, the delivery speed is very fast, the packaging is very careful and strict, very satisfied with a shopping	Jingdong prefecture level city in Shandong
Fortune Shop	White Intelligent Power Saver	Fake, doesn't work at all. Don't even sell it.	Jingdong prefecture level city in Shandong
Fortune Shop	White Intelligent Power Saver	This item doesn't seem to have much effect?	Jingdong prefecture level city in Shandong
Fortune Shop	White Intelligent Power Saver	This production is a bit fake, just look at the focus on Ming	Jingdong prefecture level city in Shandong
Fortune Shop	White Intelligent Power Saver	The logistics is fast the service attitude is very good.	Jingdong prefecture level city in Shandong
Fortune Shop	White Intelligent Power Saver	The meter is good quality, sturdy and durable. It is accurate. Easy to use. Saves a lot of things.	Jingdong prefecture level city in Shandong
Fortune Shop	White Intelligent Power Saver	Just received the goods, don't know how it is yet	Jingdong prefecture level city in Shandong
Fortune Shop	White Intelligent Power Saver	Energy saver received, logistics is very fast, the quality is very good, the appearance is also very beautiful power saving is the most important.	Jingdong prefecture level city in Shandong
Fortune Shop	White Intelligent Power Saver	It's good to talk about it, it's not practical to use it.	Jingdong prefecture level city in Shandong
Fortune Shop	White Intelligent Power Saver	I don't know if it's good for now, I'll comment after using it	Jingdong prefecture level city in Shandong
Fortune Shop	White Intelligent Power Saver	I've been using it for two or three days. It's not energy efficient.	Jingdong prefecture level city in Shandong
Fortune Shop	White Intelligent Power Saver	Packaging is intact, logistics is also fast, cost-effective, inexpensive, very satisfied with a purchase	Jingdong prefecture level city in Shandong
Fortune Shop	White Intelligent Power Saver	In use to be observed!	Jingdong prefecture level city in Shandong
Fortune Shop	White Intelligent Power Saver	Use it for a while at a flat rate	Jingdong prefecture level city in Shandong
Fortune Shop	White Intelligent Power Saver	Very trustworthy brand, quality is guaranteed, the price is still affordable, quite satisfied!	Jingdong prefecture level city in Shandong
Fortune Shop	White Intelligent Power Saver	Great quality, great operation, easy to install, great price!	Jingdong prefecture level city in Shandong
Fortune Shop	White Intelligent Power Saver	I came back and installed it, it really saves electricity, much more than before, the	Jingdong prefecture level city in Shandong
Fortune Shop	White Intelligent Power Saver	Fast logistics, good quality, very good packaging, good customer service	Jingdong prefecture level city in Shandong
Fortune Shop	White Intelligent Power Saver	Very good quality, especially good, effective, satisfactory	Jingdong prefecture level city in Shandong
Fortune Shop	White Intelligent Power Saver	I don't know how well it works.	Jingdong prefecture level city in Shandong
Fortune Shop	White Intelligent Power Saver	Good value for money, recommend it haha	Jingdong prefecture level city in Shandong
Fortune Shop	White Intelligent Power Saver	Good. Good. Very good.	Jingdong prefecture level city in Shandong
Fortune Shop	White Intelligent Power Saver	It's not working.	Jingdong prefecture level city in Shandong
Fortune Shop	White Intelligent Power Saver	It's not working.	Jingdong prefecture level city in Shandong
Fortune Shop	White Intelligent Power Saver	In use	Jingdong prefecture level city in Shandong
Fortune Shop	White Intelligent Power Saver	I'll try it later.	Jingdong prefecture level city in Shandong
Fortune Shop	White Intelligent Power Saver	It's not much use.	Jingdong prefecture level city in Shandong
Fortune Shop	White Intelligent Power Saver	It doesn't save electricity.	Jingdong prefecture level city in Shandong
Fortune Shop	White Intelligent Power Saver	It's not much use.	Jingdong prefecture level city in Shandong
Fortune Shop	White Intelligent Power Saver	Tried it for a few days and tracked the record, and there is no difference from before.	Jingdong prefecture level city in Shandong
Fortune Shop	White Intelligent Power Saver	It's not very credible, so just do it yourself and turn off the lights diligently!	Jingdong prefecture level city in Shandong
Fortune Shop	White Intelligent Power Saver	Too bad, no power saving at all, scam people don't fall for it!	Jingdong prefecture level city in Shandong
Fortune Shop	White Intelligent Power Saver	It's not working at all.	Jingdong prefecture level city in Shandong
Fortune Shop	White Intelligent Power Saver	general	Jingdong prefecture level city in Shandong
Fortune Shop	White Intelligent Power Saver	(of an unmarried couple) be close	Jingdong prefecture level city in Shandong

Fortune Shop	White Intelligent Power Saver	of no use	Jingdong prefecture level city in Shandong
Fortune Shop	White Intelligent Power Saver	Actually can really save electricity, only used half a month to save more than 50, recommended to the neighbors to use together!	Jingdong prefecture level city in Shandong
Fortune Shop	White Intelligent Power Saver	Save what you can. Isn't it more cost effective to buy one of these than to pay the electric bill?	Jingdong prefecture level city in Shandong
Fortune Shop	White Intelligent Power Saver	Received, a use do not know how, use a month to see the effect is good, the effect is good then recommended, not good then I just do not recommend.	Jingdong prefecture level city in Shandong
Fortune Shop	White Intelligent Power Saver	Just received the goods, is a voltage regulator, next month to evaluate whether it is really power saving	Jingdong prefecture level city in Shandong
Fortune Shop	White Intelligent Power Saver	Very good, used a few days obviously feel that the electricity bill is much less, wait a month to see how much can be saved!	Jingdong prefecture level city in Shandong
Fortune Shop	White Intelligent Power Saver	Received the stuff used for more than a month without a little use, it feels like it uses more electricity than before	Jingdong prefecture level city in Shandong
Fortune Shop	White Intelligent Power Saver	The quality is very good, completely exceeded the expectations, the delivery speed is very fast, the packaging is very careful and strict, very satisfied with a shopping	Jingdong prefecture level city in Shandong
Fortune Shop	White Intelligent Power Saver	Fake, doesn't work at all. Don't even sell it.	Jingdong prefecture level city in Shandong
Fortune Shop	White Intelligent Power Saver	This item doesn't seem to have much effect?	Jingdong prefecture level city in Shandong
Fortune Shop	White Intelligent Power Saver	This production is a bit fake, just look at the focus on Ming	Jingdong prefecture level city in Shandong
Fortune Shop	White Intelligent Power Saver	The logistics is fast the service attitude is very good.	Jingdong prefecture level city in Shandong
Fortune Shop	White Intelligent Power Saver	The meter is good quality, sturdy and durable. It is accurate. Easy to use. Saves a lot of things.	Jingdong prefecture level city in Shandong
Fortune Shop	White Intelligent Power Saver	Just received the goods, don't know how it is yet	Jingdong prefecture level city in Shandong
Fortune Shop	White Intelligent Power Saver	Energy saver received, logistics is very fast, the quality is very good, the appearance is also very beautiful power saving is the most important.	Jingdong prefecture level city in Shandong
Fortune Shop	White Intelligent Power Saver	It's good to talk about it, it's not practical to use it.	Jingdong prefecture level city in Shandong
Fortune Shop	White Intelligent Power Saver	I don't know if it's good for now, I'll comment after using it	Jingdong prefecture level city in Shandong
Fortune Shop	White Intelligent Power Saver	I've been using it for two or three days. It's not energy efficient.	Jingdong prefecture level city in Shandong
Fortune Shop	White Intelligent Power Saver	Packaging is intact, logistics is also fast, cost-effective, inexpensive, very satisfied with a purchase	Jingdong prefecture level city in Shandong
Fortune Shop	White Intelligent Power Saver	In use to be observed!	Jingdong prefecture level city in Shandong
Fortune Shop	White Intelligent Power Saver	Use it for a while at a flat rate	Jingdong prefecture level city in Shandong
Fortune Shop	White Intelligent Power Saver	Very trustworthy brand, quality is guaranteed, the price is still affordable, quite satisfied!	Jingdong prefecture level city in Shandong
Fortune Shop	White Intelligent Power Saver	Great quality, great operation, easy to install, great price!	Jingdong prefecture level city in Shandong
Fortune Shop	White Intelligent Power Saver	I came back and installed it, it really saves electricity, much more than before, the	Jingdong prefecture level city in Shandong
Fortune Shop	White Intelligent Power Saver	Fast logistics, good quality, very good packaging, good customer service	Jingdong prefecture level city in Shandong
Fortune Shop	White Intelligent Power Saver	Very good quality, especially good, effective, satisfactory	Jingdong prefecture level city in Shandong
Fortune Shop	White Intelligent Power Saver	I don't know how well it works.	Jingdong prefecture level city in Shandong
Fortune Shop	White Intelligent Power Saver	Good value for money, recommend it haha	Jingdong prefecture level city in Shandong
Fortune Shop	White Intelligent Power Saver	Good. Good. Very good.	Jingdong prefecture level city in Shandong
Fortune Shop	White Intelligent Power Saver	It's not working.	Jingdong prefecture level city in Shandong
Fortune Shop	White Intelligent Power Saver	It's not working.	Jingdong prefecture level city in Shandong
Fortune Shop	White Intelligent Power Saver	In use	Jingdong prefecture level city in Shandong
Fortune Shop	White Intelligent Power Saver	I'll try it later.	Jingdong prefecture level city in Shandong
Fortune Shop	White Intelligent Power Saver	It's not much use.	Jingdong prefecture level city in Shandong
Fortune Shop	White Intelligent Power Saver	It doesn't save electricity.	Jingdong prefecture level city in Shandong
Fortune Shop	White Intelligent Power Saver	It's not much use.	Jingdong prefecture level city in Shandong
Fortune Shop	White Intelligent Power Saver	Tried it for a few days and tracked the record, and there is no difference from before.	Jingdong prefecture level city in Shandong
Fortune Shop	White Intelligent Power Saver	It's not very credible, so just do it yourself and turn off the lights diligently!	Jingdong prefecture level city in Shandong
Fortune Shop	White Intelligent Power Saver	Too bad, no power saving at all, scam people don't fall for it!	Jingdong prefecture level city in Shandong
Fortune Shop	White Intelligent Power Saver	It's not working at all.	Jingdong prefecture level city in Shandong
Fortune Shop	White Intelligent Power Saver	general	Jingdong prefecture level city in Shandong
Fortune Shop	White Intelligent Power Saver	(of an unmarried couple) be close	Jingdong prefecture level city in Shandong
Fortune Shop	White Intelligent Power Saver	of no use	Jingdong prefecture level city in Shandong
Fortune Shop	White Intelligent Power Saver	Actually can really save electricity, only used half a month to save more than 50, recommended to the neighbors to use together!	Jingdong prefecture level city in Shandong
Fortune Shop	White Intelligent Power Saver	Save what you can. Isn't it more cost effective to buy one of these than to pay the electric bill?	Jingdong prefecture level city in Shandong
Fortune Shop	White Intelligent Power Saver	Received, a use do not know how, use a month to see the effect is good, the effect is good then recommended, not good then I just do not recommend.	Jingdong prefecture level city in Shandong
Fortune Shop	White Intelligent Power Saver	Just received the goods, is a voltage regulator, next month to evaluate whether it is really power saving	Jingdong prefecture level city in Shandong
Fortune Shop	White Intelligent Power Saver	Very good, used a few days obviously feel that the electricity bill is much less, wait a month to see how much can be saved!	Jingdong prefecture level city in Shandong
Fortune Shop	White Intelligent Power Saver	Received the stuff used for more than a month without a little use, it feels like it uses more electricity than before	Jingdong prefecture level city in Shandong
Fortune Shop	White Intelligent Power Saver	The quality is very good, completely exceeded the expectations, the delivery speed is very fast, the packaging is very careful and strict, very satisfied with a shopping	Jingdong prefecture level city in Shandong
Fortune Shop	White Intelligent Power Saver	Fake, doesn't work at all. Don't even sell it.	Jingdong prefecture level city in Shandong

Fortune Shop	White Intelligent Power Saver	This item doesn't seem to have much effect?	Jingdong prefecture level city in Shandong
Fortune Shop	White Intelligent Power Saver	This production is a bit fake, just look at the focus on Ming	Jingdong prefecture level city in Shandong
Fortune Shop	White Intelligent Power Saver	The logistics is fast the service attitude is very good.	Jingdong prefecture level city in Shandong
Fortune Shop	White Intelligent Power Saver	The meter is good quality, sturdy and durable. It is accurate. Easy to use. Saves a lot of things.	Jingdong prefecture level city in Shandong
Fortune Shop	White Intelligent Power Saver	Just received the goods, don't know how it is yet	Jingdong prefecture level city in Shandong
Fortune Shop	White Intelligent Power Saver	Energy saver received, logistics is very fast, the quality is very good, the appearance is also very beautiful power saving is the most important.	Jingdong prefecture level city in Shandong
Fortune Shop	White Intelligent Power Saver	It's good to talk about it, it's not practical to use it.	Jingdong prefecture level city in Shandong
Fortune Shop	White Intelligent Power Saver	I don't know if it's good for now, I'll comment after using it	Jingdong prefecture level city in Shandong
Fortune Shop	White Intelligent Power Saver	I've been using it for two or three days. It's not energy efficient.	Jingdong prefecture level city in Shandong
Fortune Shop	White Intelligent Power Saver	Packaging is intact, logistics is also fast, cost-effective, inexpensive, very satisfied with a purchase	Jingdong prefecture level city in Shandong
Fortune Shop	White Intelligent Power Saver	In use to be observed!	Jingdong prefecture level city in Shandong
Fortune Shop	White Intelligent Power Saver	Use it for a while at a flat rate	Jingdong prefecture level city in Shandong
Fortune Shop	White Intelligent Power Saver	Very trustworthy brand, quality is guaranteed, the price is still affordable, quite satisfied!	Jingdong prefecture level city in Shandong
Fortune Shop	White Intelligent Power Saver	Great quality, great operation, easy to install, great price!	Jingdong prefecture level city in Shandong
Fortune Shop	White Intelligent Power Saver	I came back and installed it, it really saves electricity, much more than before, the	Jingdong prefecture level city in Shandong
Fortune Shop	White Intelligent Power Saver	Fast logistics, good quality, very good packaging, good customer service	Jingdong prefecture level city in Shandong
Fortune Shop	White Intelligent Power Saver	Very good quality, especially good, effective, satisfactory	Jingdong prefecture level city in Shandong
Fortune Shop	White Intelligent Power Saver	I don't know how well it works.	Jingdong prefecture level city in Shandong
Fortune Shop	White Intelligent Power Saver	Good value for money, recommend it haha	Jingdong prefecture level city in Shandong
Fortune Shop	White Intelligent Power Saver	Good. Good. Very good.	Jingdong prefecture level city in Shandong
Fortune Shop	White Intelligent Power Saver	It's not working.	Jingdong prefecture level city in Shandong
Fortune Shop	White Intelligent Power Saver	It's not working.	Jingdong prefecture level city in Shandong
Fortune Shop	White Intelligent Power Saver	In use	Jingdong prefecture level city in Shandong
Fortune Shop	White Intelligent Power Saver	I'll try it later.	Jingdong prefecture level city in Shandong
Fortune Shop	White Intelligent Power Saver	It's not much use.	Jingdong prefecture level city in Shandong
Fortune Shop	White Intelligent Power Saver	It doesn't save electricity.	Jingdong prefecture level city in Shandong
Fortune Shop	White Intelligent Power Saver	It's not much use.	Jingdong prefecture level city in Shandong
Fortune Shop	White Intelligent Power Saver	Tried it for a few days and tracked the record, and there is no difference from before.	Jingdong prefecture level city in Shandong
Fortune Shop	White Intelligent Power Saver	It's not very credible, so just do it yourself and turn off the lights diligently!	Jingdong prefecture level city in Shandong
Fortune Shop	White Intelligent Power Saver	Too bad, no power saving at all, scam people don't fall for it!	Jingdong prefecture level city in Shandong
Fortune Shop	White Intelligent Power Saver	It's not working at all.	Jingdong prefecture level city in Shandong
Fortune Shop	White Intelligent Power Saver	general	Jingdong prefecture level city in Shandong
Fortune Shop	White Intelligent Power Saver	(of an unmarried couple) be close	Jingdong prefecture level city in Shandong
Fortune Shop	White Intelligent Power Saver	of no use	Jingdong prefecture level city in Shandong
Fortune Shop	White Intelligent Power Saver	Actually can really save electricity, only used half a month to save more than 50, recommended to the neighbors to use together!	Jingdong prefecture level city in Shandong
Fortune Shop	White Intelligent Power Saver	Save what you can. Isn't it more cost effective to buy one of these than to pay the electric bill?	Jingdong prefecture level city in Shandong
Fortune Shop	White Intelligent Power Saver	Received, a use do not know how, use a month to see the effect is good, the effect is good then recommended, not good then I just do not recommend.	Jingdong prefecture level city in Shandong
Fortune Shop	White Intelligent Power Saver	Just received the goods, is a voltage regulator, next month to evaluate whether it is really power saving	Jingdong prefecture level city in Shandong
Fortune Shop	White Intelligent Power Saver	Very good, used a few days obviously feel that the electricity bill is much less, wait a month to see how much can be saved!	Jingdong prefecture level city in Shandong
Fortune Shop	White Intelligent Power Saver	Received the stuff used for more than a month without a little use, it feels like it uses more electricity than before	Jingdong prefecture level city in Shandong
Fortune Shop	White Intelligent Power Saver	The quality is very good, completely exceeded the expectations, the delivery speed is very fast, the packaging is very careful and strict, very satisfied with a shopping	Jingdong prefecture level city in Shandong
Fortune Shop	White Intelligent Power Saver	Fake, doesn't work at all. Don't even sell it.	Jingdong prefecture level city in Shandong
Fortune Shop	White Intelligent Power Saver	This item doesn't seem to have much effect?	Jingdong prefecture level city in Shandong
Fortune Shop	White Intelligent Power Saver	This production is a bit fake, just look at the focus on Ming	Jingdong prefecture level city in Shandong
Fortune Shop	White Intelligent Power Saver	The logistics is fast the service attitude is very good.	Jingdong prefecture level city in Shandong
Fortune Shop	White Intelligent Power Saver	The meter is good quality, sturdy and durable. It is accurate. Easy to use. Saves a lot of things.	Jingdong prefecture level city in Shandong
Fortune Shop	White Intelligent Power Saver	Just received the goods, don't know how it is yet	Jingdong prefecture level city in Shandong
Fortune Shop	White Intelligent Power Saver	Energy saver received, logistics is very fast, the quality is very good, the appearance is also very beautiful power saving is the most important.	Jingdong prefecture level city in Shandong
Fortune Shop	White Intelligent Power Saver	It's good to talk about it, it's not practical to use it.	Jingdong prefecture level city in Shandong
Fortune Shop	White Intelligent Power Saver	I don't know if it's good for now, I'll comment after using it	Jingdong prefecture level city in Shandong
Fortune Shop	White Intelligent Power Saver	I've been using it for two or three days. It's not energy efficient.	Jingdong prefecture level city in Shandong

Fortune Shop	White Intelligent Power Saver	Packaging is intact, logistics is also fast, cost-effective, inexpensive, very satisfied with a purchase	Jingdong prefecture level city in Shandong
Fortune Shop	White Intelligent Power Saver	In use to be observed!	Jingdong prefecture level city in Shandong
Fortune Shop	White Intelligent Power Saver	Use it for a while at a flat rate	Jingdong prefecture level city in Shandong
Fortune Shop	White Intelligent Power Saver	Very trustworthy brand, quality is guaranteed, the price is still affordable, quite satisfied!	Jingdong prefecture level city in Shandong
Fortune Shop	White Intelligent Power Saver	Great quality, great operation, easy to install, great price!	Jingdong prefecture level city in Shandong
Fortune Shop	White Intelligent Power Saver	I came back and installed it, it really saves electricity, much more than before, the	Jingdong prefecture level city in Shandong
Fortune Shop	White Intelligent Power Saver	Fast logistics, good quality, very good packaging, good customer service	Jingdong prefecture level city in Shandong
Fortune Shop	White Intelligent Power Saver	Very good quality, especially good, effective, satisfactory	Jingdong prefecture level city in Shandong
Fortune Shop	White Intelligent Power Saver	I don't know how well it works.	Jingdong prefecture level city in Shandong
Fortune Shop	White Intelligent Power Saver	Good value for money, recommend it haha	Jingdong prefecture level city in Shandong
Fortune Shop	White Intelligent Power Saver	Good. Good. Very good.	Jingdong prefecture level city in Shandong
Fortune Shop	White Intelligent Power Saver	It's not working.	Jingdong prefecture level city in Shandong
Fortune Shop	White Intelligent Power Saver	It's not working.	Jingdong prefecture level city in Shandong
Fortune Shop	White Intelligent Power Saver	In use	Jingdong prefecture level city in Shandong
Fortune Shop	White Intelligent Power Saver	I'll try it later.	Jingdong prefecture level city in Shandong
Fortune Shop	White Intelligent Power Saver	It's not much use.	Jingdong prefecture level city in Shandong
Fortune Shop	White Intelligent Power Saver	It doesn't save electricity.	Jingdong prefecture level city in Shandong
Fortune Shop	White Intelligent Power Saver	It's not much use.	Jingdong prefecture level city in Shandong
Fortune Shop	White Intelligent Power Saver	Tried it for a few days and tracked the record, and there is no difference from before.	Jingdong prefecture level city in Shandong
Fortune Shop	White Intelligent Power Saver	It's not very credible, so just do it yourself and turn off the lights diligently!	Jingdong prefecture level city in Shandong
Fortune Shop	White Intelligent Power Saver	Too bad, no power saving at all, scam people don't fall for it!	Jingdong prefecture level city in Shandong
Fortune Shop	White Intelligent Power Saver	It's not working at all.	Jingdong prefecture level city in Shandong
Fortune Shop	White Intelligent Power Saver	general	Jingdong prefecture level city in Shandong
Fortune Shop	White Intelligent Power Saver	(of an unmarried couple) be close	Jingdong prefecture level city in Shandong
Fortune Shop	White Intelligent Power Saver	of no use	Jingdong prefecture level city in Shandong
Fortune Shop	White Intelligent Power Saver	Actually can really save electricity, only used half a month to save more than 50, recommended to the neighbors to use together!	Jingdong prefecture level city in Shandong
Fortune Shop	White Intelligent Power Saver	Save what you can. Isn't it more cost effective to buy one of these than to pay the electric bill?	Jingdong prefecture level city in Shandong
Fortune Shop	White Intelligent Power Saver	Received, a use do not know how, use a month to see the effect is good, the effect is good then recommended, not good then I just do not recommend.	Jingdong prefecture level city in Shandong
Fortune Shop	White Intelligent Power Saver	Just received the goods, is a voltage regulator, next month to evaluate whether it is really power saving	Jingdong prefecture level city in Shandong
Fortune Shop	White Intelligent Power Saver	Very good, used a few days obviously feel that the electricity bill is much less, wait a month to see how much can be saved!	Jingdong prefecture level city in Shandong
Fortune Shop	White Intelligent Power Saver	Received the stuff used for more than a month without a little use, it feels like it uses more electricity than before	Jingdong prefecture level city in Shandong
Fortune Shop	White Intelligent Power Saver	The quality is very good, completely exceeded the expectations, the delivery speed is very fast, the packaging is very careful and strict, very satisfied with a shopping	Jingdong prefecture level city in Shandong
Fortune Shop	White Intelligent Power Saver	Fake, doesn't work at all. Don't even sell it.	Jingdong prefecture level city in Shandong
Fortune Shop	White Intelligent Power Saver	This item doesn't seem to have much effect?	Jingdong prefecture level city in Shandong
Fortune Shop	White Intelligent Power Saver	This production is a bit fake, just look at the focus on Ming	Jingdong prefecture level city in Shandong
Fortune Shop	White Intelligent Power Saver	The logistics is fast the service attitude is very good.	Jingdong prefecture level city in Shandong
Fortune Shop	White Intelligent Power Saver	The meter is good quality, sturdy and durable. It is accurate. Easy to use. Saves a lot of things.	Jingdong prefecture level city in Shandong
Fortune Shop	White Intelligent Power Saver	Just received the goods, don't know how it is yet	Jingdong prefecture level city in Shandong
Fortune Shop	White Intelligent Power Saver	Energy saver received, logistics is very fast, the quality is very good, the appearance is also very beautiful power saving is the most important.	Jingdong prefecture level city in Shandong
Fortune Shop	White Intelligent Power Saver	It's good to talk about it, it's not practical to use it.	Jingdong prefecture level city in Shandong
Fortune Shop	White Intelligent Power Saver	I don't know if it's good for now, I'll comment after using it	Jingdong prefecture level city in Shandong
Fortune Shop	White Intelligent Power Saver	I've been using it for two or three days. It's not energy efficient.	Jingdong prefecture level city in Shandong
Fortune Shop	White Intelligent Power Saver	Packaging is intact, logistics is also fast, cost-effective, inexpensive, very satisfied with a purchase	Jingdong prefecture level city in Shandong
Fortune Shop	White Intelligent Power Saver	In use to be observed!	Jingdong prefecture level city in Shandong
Fortune Shop	White Intelligent Power Saver	Use it for a while at a flat rate	Jingdong prefecture level city in Shandong
Fortune Shop	White Intelligent Power Saver	Very trustworthy brand, quality is guaranteed, the price is still affordable, quite satisfied!	Jingdong prefecture level city in Shandong
Fortune Shop	White Intelligent Power Saver	Great quality, great operation, easy to install, great price!	Jingdong prefecture level city in Shandong
Fortune Shop	White Intelligent Power Saver	I came back and installed it, it really saves electricity, much more than before, the	Jingdong prefecture level city in Shandong
Fortune Shop	White Intelligent Power Saver	Fast logistics, good quality, very good packaging, good customer service	Jingdong prefecture level city in Shandong
Fortune Shop	White Intelligent Power Saver	Very good quality, especially good, effective, satisfactory	Jingdong prefecture level city in Shandong
Fortune Shop	White Intelligent Power Saver	I don't know how well it works.	Jingdong prefecture level city in Shandong

Fortune Shop	White Intelligent Power Saver	Good value for money, recommend it haha	Jingdong prefecture level city in Shandong
Fortune Shop	White Intelligent Power Saver	Good. Good. Very good.	Jingdong prefecture level city in Shandong
Fortune Shop	White Intelligent Power Saver	It's not working.	Jingdong prefecture level city in Shandong
Fortune Shop	White Intelligent Power Saver	It's not working.	Jingdong prefecture level city in Shandong
Fortune Shop	White Intelligent Power Saver	In use	Jingdong prefecture level city in Shandong
Fortune Shop	White Intelligent Power Saver	I'll try it later.	Jingdong prefecture level city in Shandong
Fortune Shop	White Intelligent Power Saver	It's not much use.	Jingdong prefecture level city in Shandong
Fortune Shop	White Intelligent Power Saver	It doesn't save electricity.	Jingdong prefecture level city in Shandong
Fortune Shop	White Intelligent Power Saver	It's not much use.	Jingdong prefecture level city in Shandong
Fortune Shop	White Intelligent Power Saver	Tried it for a few days and tracked the record, and there is no difference from before.	Jingdong prefecture level city in Shandong
Fortune Shop	White Intelligent Power Saver	It's not very credible, so just do it yourself and turn off the lights diligently!	Jingdong prefecture level city in Shandong
Fortune Shop	White Intelligent Power Saver	Too bad, no power saving at all, scam people don't fall for it!	Jingdong prefecture level city in Shandong
Fortune Shop	White Intelligent Power Saver	It's not working at all.	Jingdong prefecture level city in Shandong
Fortune Shop	White Intelligent Power Saver	general	Jingdong prefecture level city in Shandong
Fortune Shop	White Intelligent Power Saver	(of an unmarried couple) be close	Jingdong prefecture level city in Shandong
Fortune Shop	White Intelligent Power Saver	of no use	Jingdong prefecture level city in Shandong
Fortune Shop	White Intelligent Power Saver	Actually can really save electricity, only used half a month to save more than 50, recommended to the neighbors to use together!	Jingdong prefecture level city in Shandong
Fortune Shop	White Intelligent Power Saver	Save what you can. Isn't it more cost effective to buy one of these than to pay the electric bill?	Jingdong prefecture level city in Shandong
Fortune Shop	White Intelligent Power Saver	Received, a use do not know how, use a month to see the effect is good, the effect is good then recommended, not good then I just do not recommend.	Jingdong prefecture level city in Shandong
Fortune Shop	White Intelligent Power Saver	Just received the goods, is a voltage regulator, next month to evaluate whether it is really power saving	Jingdong prefecture level city in Shandong
Fortune Shop	White Intelligent Power Saver	Very good, used a few days obviously feel that the electricity bill is much less, wait a month to see how much can be saved!	Jingdong prefecture level city in Shandong
Fortune Shop	White Intelligent Power Saver	Received the stuff used for more than a month without a little use, it feels like it uses more electricity than before	Jingdong prefecture level city in Shandong
Fortune Shop	White Intelligent Power Saver	The quality is very good, completely exceeded the expectations, the delivery speed is very fast, the packaging is very careful and strict, very satisfied with a shopping	Jingdong prefecture level city in Shandong
Fortune Shop	White Intelligent Power Saver	Fake, doesn't work at all. Don't even sell it.	Jingdong prefecture level city in Shandong
Fortune Shop	White Intelligent Power Saver	This item doesn't seem to have much effect?	Jingdong prefecture level city in Shandong
Fortune Shop	White Intelligent Power Saver	This production is a bit fake, just look at the focus on Ming	Jingdong prefecture level city in Shandong
Fortune Shop	White Intelligent Power Saver	The logistics is fast the service attitude is very good.	Jingdong prefecture level city in Shandong
Fortune Shop	White Intelligent Power Saver	The meter is good quality, sturdy and durable. It is accurate. Easy to use. Saves a lot of things.	Jingdong prefecture level city in Shandong
Fortune Shop	White Intelligent Power Saver	Just received the goods, don't know how it is yet	Jingdong prefecture level city in Shandong
Fortune Shop	White Intelligent Power Saver	Energy saver received, logistics is very fast, the quality is very good, the appearance is also very beautiful power saving is the most important.	Jingdong prefecture level city in Shandong
Fortune Shop	White Intelligent Power Saver	It's good to talk about it, it's not practical to use it.	Jingdong prefecture level city in Shandong
Fortune Shop	White Intelligent Power Saver	I don't know if it's good for now, I'll comment after using it	Jingdong prefecture level city in Shandong
Fortune Shop	White Intelligent Power Saver	I've been using it for two or three days. It's not energy efficient.	Jingdong prefecture level city in Shandong
Fortune Shop	White Intelligent Power Saver	Packaging is intact, logistics is also fast, cost-effective, inexpensive, very satisfied with a purchase	Jingdong prefecture level city in Shandong
Fortune Shop	White Intelligent Power Saver	In use to be observed!	Jingdong prefecture level city in Shandong
Fortune Shop	White Intelligent Power Saver	Use it for a while at a flat rate	Jingdong prefecture level city in Shandong
Fortune Shop	White Intelligent Power Saver	Very trustworthy brand, quality is guaranteed, the price is still affordable, quite satisfied!	Jingdong prefecture level city in Shandong
Fortune Shop	White Intelligent Power Saver	Great quality, great operation, easy to install, great price!	Jingdong prefecture level city in Shandong
Fortune Shop	White Intelligent Power Saver	I came back and installed it, it really saves electricity, much more than before, the	Jingdong prefecture level city in Shandong
Fortune Shop	White Intelligent Power Saver	Fast logistics, good quality, very good packaging, good customer service	Jingdong prefecture level city in Shandong
Fortune Shop	White Intelligent Power Saver	Very good quality, especially good, effective, satisfactory	Jingdong prefecture level city in Shandong
Fortune Shop	White Intelligent Power Saver	I don't know how well it works.	Jingdong prefecture level city in Shandong
Fortune Shop	White Intelligent Power Saver	Good value for money, recommend it haha	Jingdong prefecture level city in Shandong
Fortune Shop	White Intelligent Power Saver	Good. Good. Very good.	Jingdong prefecture level city in Shandong
Fortune Shop	White Intelligent Power Saver	It's not working.	Jingdong prefecture level city in Shandong
Fortune Shop	White Intelligent Power Saver	It's not working.	Jingdong prefecture level city in Shandong
Fortune Shop	White Intelligent Power Saver	In use	Jingdong prefecture level city in Shandong
Fortune Shop	White Intelligent Power Saver	I'll try it later.	Jingdong prefecture level city in Shandong
Fortune Shop	White Intelligent Power Saver	It's not much use.	Jingdong prefecture level city in Shandong
Fortune Shop	White Intelligent Power Saver	It doesn't save electricity.	Jingdong prefecture level city in Shandong
Fortune Shop	White Intelligent Power Saver	It's not much use.	Jingdong prefecture level city in Shandong

Fortune Shop	White Intelligent Power Saver	Tried it for a few days and tracked the record, and there is no difference from before.	Jingdong prefecture level city in Shandong
Fortune Shop	White Intelligent Power Saver	It's not very credible, so just do it yourself and turn off the lights diligently!	Jingdong prefecture level city in Shandong
Fortune Shop	White Intelligent Power Saver	Too bad, no power saving at all, scam people don't fall for it!	Jingdong prefecture level city in Shandong
Fortune Shop	White Intelligent Power Saver	It's not working at all.	Jingdong prefecture level city in Shandong
Fortune Shop	White Intelligent Power Saver	general	Jingdong prefecture level city in Shandong
Fortune Shop	White Intelligent Power Saver	(of an unmarried couple) be close	Jingdong prefecture level city in Shandong
Fortune Shop	White Intelligent Power Saver	of no use	Jingdong prefecture level city in Shandong
Fortune Shop	White Intelligent Power Saver	Actually can really save electricity, only used half a month to save more than 50, recommended to the neighbors to use together!	Jingdong prefecture level city in Shandong
Fortune Shop	White Intelligent Power Saver	Save what you can. Isn't it more cost effective to buy one of these than to pay the electric bill?	Jingdong prefecture level city in Shandong
Fortune Shop	White Intelligent Power Saver	Received, a use do not know how, use a month to see the effect is good, the effect is good then recommended, not good then I just do not recommend.	Jingdong prefecture level city in Shandong
Fortune Shop	White Intelligent Power Saver	Just received the goods, is a voltage regulator, next month to evaluate whether it is really power saving	Jingdong prefecture level city in Shandong
Fortune Shop	White Intelligent Power Saver	Very good, used a few days obviously feel that the electricity bill is much less, wait a month to see how much can be saved!	Jingdong prefecture level city in Shandong
Fortune Shop	White Intelligent Power Saver	Received the stuff used for more than a month without a little use, it feels like it uses more electricity than before	Jingdong prefecture level city in Shandong
Fortune Shop	White Intelligent Power Saver	The quality is very good, completely exceeded the expectations, the delivery speed is very fast, the packaging is very careful and strict, very satisfied with a shopping	Jingdong prefecture level city in Shandong
Fortune Shop	White Intelligent Power Saver	Fake, doesn't work at all. Don't even sell it.	Jingdong prefecture level city in Shandong
Fortune Shop	White Intelligent Power Saver	This item doesn't seem to have much effect?	Jingdong prefecture level city in Shandong
Fortune Shop	White Intelligent Power Saver	This production is a bit fake, just look at the focus on Ming	Jingdong prefecture level city in Shandong
Fortune Shop	White Intelligent Power Saver	The logistics is fast the service attitude is very good.	Jingdong prefecture level city in Shandong
Fortune Shop	White Intelligent Power Saver	The meter is good quality, sturdy and durable. It is accurate. Easy to use. Saves a lot of things.	Jingdong prefecture level city in Shandong
Fortune Shop	White Intelligent Power Saver	Just received the goods, don't know how it is yet	Jingdong prefecture level city in Shandong
Fortune Shop	White Intelligent Power Saver	Energy saver received, logistics is very fast, the quality is very good, the appearance is also very beautiful power saving is the most important.	Jingdong prefecture level city in Shandong
Fortune Shop	White Intelligent Power Saver	It's good to talk about it, it's not practical to use it.	Jingdong prefecture level city in Shandong
Fortune Shop	White Intelligent Power Saver	I don't know if it's good for now, I'll comment after using it	Jingdong prefecture level city in Shandong
Fortune Shop	White Intelligent Power Saver	I've been using it for two or three days. It's not energy efficient.	Jingdong prefecture level city in Shandong
Fortune Shop	White Intelligent Power Saver	Packaging is intact, logistics is also fast, cost-effective, inexpensive, very satisfied with a purchase	Jingdong prefecture level city in Shandong
Fortune Shop	White Intelligent Power Saver	In use to be observed!	Jingdong prefecture level city in Shandong
Fortune Shop	White Intelligent Power Saver	Use it for a while at a flat rate	Jingdong prefecture level city in Shandong
Fortune Shop	White Intelligent Power Saver	Very trustworthy brand, quality is guaranteed, the price is still affordable, quite satisfied!	Jingdong prefecture level city in Shandong
Fortune Shop	White Intelligent Power Saver	Great quality, great operation, easy to install, great price!	Jingdong prefecture level city in Shandong
Fortune Shop	White Intelligent Power Saver	I came back and installed it, it really saves electricity, much more than before, the	Jingdong prefecture level city in Shandong
Fortune Shop	White Intelligent Power Saver	Fast logistics, good quality, very good packaging, good customer service	Jingdong prefecture level city in Shandong
Fortune Shop	White Intelligent Power Saver	Very good quality, especially good, effective, satisfactory	Jingdong prefecture level city in Shandong
Fortune Shop	White Intelligent Power Saver	I don't know how well it works.	Jingdong prefecture level city in Shandong
Fortune Shop	White Intelligent Power Saver	Good value for money, recommend it haha	Jingdong prefecture level city in Shandong
Fortune Shop	White Intelligent Power Saver	Good. Good. Very good.	Jingdong prefecture level city in Shandong
Fortune Shop	White Intelligent Power Saver	It's not working.	Jingdong prefecture level city in Shandong
Fortune Shop	White Intelligent Power Saver	It's not working.	Jingdong prefecture level city in Shandong
Fortune Shop	White Intelligent Power Saver	In use	Jingdong prefecture level city in Shandong
Fortune Shop	White Intelligent Power Saver	I'll try it later.	Jingdong prefecture level city in Shandong
Fortune Shop	White Intelligent Power Saver	It's not much use.	Jingdong prefecture level city in Shandong
Fortune Shop	White Intelligent Power Saver	It doesn't save electricity.	Jingdong prefecture level city in Shandong
Fortune Shop	White Intelligent Power Saver	It's not much use.	Jingdong prefecture level city in Shandong
Fortune Shop	White Intelligent Power Saver	Tried it for a few days and tracked the record, and there is no difference from before.	Jingdong prefecture level city in Shandong
Fortune Shop	White Intelligent Power Saver	It's not very credible, so just do it yourself and turn off the lights diligently!	Jingdong prefecture level city in Shandong
Fortune Shop	White Intelligent Power Saver	Too bad, no power saving at all, scam people don't fall for it!	Jingdong prefecture level city in Shandong
Fortune Shop	White Intelligent Power Saver	It's not working at all.	Jingdong prefecture level city in Shandong
Fortune Shop	White Intelligent Power Saver	general	Jingdong prefecture level city in Shandong
Fortune Shop	White Intelligent Power Saver	(of an unmarried couple) be close	Jingdong prefecture level city in Shandong
Fortune Shop	White Intelligent Power Saver	of no use	Jingdong prefecture level city in Shandong
Fortune Shop	White Intelligent Power Saver	Actually can really save electricity, only used half a month to save more than 50, recommended to the neighbors to use together!	Jingdong prefecture level city in Shandong
Fortune Shop	White Intelligent Power Saver	Save what you can. Isn't it more cost effective to buy one of these than to pay the electric bill?	Jingdong prefecture level city in Shandong

Fortune Shop	White Intelligent Power Saver	Received, a use do not know how, use a month to see the effect is good, the effect is good then recommended, not good then I just do not recommend.	Jingdong prefecture level city in Shandong
Fortune Shop	White Intelligent Power Saver	Just received the goods, is a voltage regulator, next month to evaluate whether it is really power saving	Jingdong prefecture level city in Shandong
Fortune Shop	White Intelligent Power Saver	Very good, used a few days obviously feel that the electricity bill is much less, wait a month to see how much can be saved!	Jingdong prefecture level city in Shandong
Fortune Shop	White Intelligent Power Saver	Received the stuff used for more than a month without a little use, it feels like it uses more electricity than before	Jingdong prefecture level city in Shandong
Fortune Shop	White Intelligent Power Saver	The quality is very good, completely exceeded the expectations, the delivery speed is very fast, the packaging is very careful and strict, very satisfied with a shopping	Jingdong prefecture level city in Shandong
Fortune Shop	White Intelligent Power Saver	Fake, doesn't work at all. Don't even sell it.	Jingdong prefecture level city in Shandong
Fortune Shop	White Intelligent Power Saver	This item doesn't seem to have much effect?	Jingdong prefecture level city in Shandong
Fortune Shop	White Intelligent Power Saver	This production is a bit fake, just look at the focus on Ming	Jingdong prefecture level city in Shandong
Fortune Shop	White Intelligent Power Saver	The logistics is fast the service attitude is very good.	Jingdong prefecture level city in Shandong
Fortune Shop	White Intelligent Power Saver	The meter is good quality, sturdy and durable. It is accurate. Easy to use. Saves a lot of things.	Jingdong prefecture level city in Shandong
Fortune Shop	White Intelligent Power Saver	Just received the goods, don't know how it is yet	Jingdong prefecture level city in Shandong
Fortune Shop	White Intelligent Power Saver	Energy saver received, logistics is very fast, the quality is very good, the appearance is also very beautiful power saving is the most important.	Jingdong prefecture level city in Shandong
Fortune Shop	White Intelligent Power Saver	It's good to talk about it, it's not practical to use it.	Jingdong prefecture level city in Shandong
Fortune Shop	White Intelligent Power Saver	I don't know if it's good for now, I'll comment after using it	Jingdong prefecture level city in Shandong
Fortune Shop	White Intelligent Power Saver	I've been using it for two or three days. It's not energy efficient.	Jingdong prefecture level city in Shandong
Fortune Shop	White Intelligent Power Saver	Packaging is intact, logistics is also fast, cost-effective, inexpensive, very satisfied with a purchase	Jingdong prefecture level city in Shandong
Fortune Shop	White Intelligent Power Saver	In use to be observed!	Jingdong prefecture level city in Shandong
Fortune Shop	White Intelligent Power Saver	Use it for a while at a flat rate	Jingdong prefecture level city in Shandong
Fortune Shop	White Intelligent Power Saver	Very trustworthy brand, quality is guaranteed, the price is still affordable, quite satisfied!	Jingdong prefecture level city in Shandong
Fortune Shop	White Intelligent Power Saver	Great quality, great operation, easy to install, great price!	Jingdong prefecture level city in Shandong
Fortune Shop	White Intelligent Power Saver	I came back and installed it, it really saves electricity, much more than before, the	Jingdong prefecture level city in Shandong
Fortune Shop	White Intelligent Power Saver	Fast logistics, good quality, very good packaging, good customer service	Jingdong prefecture level city in Shandong
Fortune Shop	White Intelligent Power Saver	Very good quality, especially good, effective, satisfactory	Jingdong prefecture level city in Shandong
Fortune Shop	White Intelligent Power Saver	I don't know how well it works.	Jingdong prefecture level city in Shandong
Fortune Shop	White Intelligent Power Saver	Good value for money, recommend it haha	Jingdong prefecture level city in Shandong
Fortune Shop	White Intelligent Power Saver	Good. Good. Very good.	Jingdong prefecture level city in Shandong
Fortune Shop	White Intelligent Power Saver	It's not working.	Jingdong prefecture level city in Shandong
Fortune Shop	White Intelligent Power Saver	It's not working.	Jingdong prefecture level city in Shandong
Fortune Shop	White Intelligent Power Saver	In use	Jingdong prefecture level city in Shandong
Fortune Shop	White Intelligent Power Saver	I'll try it later.	Jingdong prefecture level city in Shandong
Fortune Shop	White Intelligent Power Saver	It's not much use.	Jingdong prefecture level city in Shandong
Fortune Shop	White Intelligent Power Saver	It doesn't save electricity.	Jingdong prefecture level city in Shandong
Fortune Shop	White Intelligent Power Saver	It's not much use.	Jingdong prefecture level city in Shandong
Fortune Shop	White Intelligent Power Saver	Tried it for a few days and tracked the record, and there is no difference from before.	Jingdong prefecture level city in Shandong
Fortune Shop	White Intelligent Power Saver	It's not very credible, so just do it yourself and turn off the lights diligently!	Jingdong prefecture level city in Shandong
Fortune Shop	White Intelligent Power Saver	Too bad, no power saving at all, scam people don't fall for it!	Jingdong prefecture level city in Shandong
Fortune Shop	White Intelligent Power Saver	It's not working at all.	Jingdong prefecture level city in Shandong
Fortune Shop	White Intelligent Power Saver	general	Jingdong prefecture level city in Shandong
Fortune Shop	White Intelligent Power Saver	(of an unmarried couple) be close	Jingdong prefecture level city in Shandong
Fortune Shop	White Intelligent Power Saver	of no use	Jingdong prefecture level city in Shandong
Fortune Shop	White Intelligent Power Saver	Actually can really save electricity, only used half a month to save more than 50, recommended to the neighbors to use together!	Jingdong prefecture level city in Shandong
Fortune Shop	White Intelligent Power Saver	Save what you can. Isn't it more cost effective to buy one of these than to pay the electric bill?	Jingdong prefecture level city in Shandong
Fortune Shop	White Intelligent Power Saver	Received, a use do not know how, use a month to see the effect is good, the effect is good then recommended, not good then I just do not recommend.	Jingdong prefecture level city in Shandong
Fortune Shop	White Intelligent Power Saver	Just received the goods, is a voltage regulator, next month to evaluate whether it is really power saving	Jingdong prefecture level city in Shandong
Fortune Shop	White Intelligent Power Saver	Very good, used a few days obviously feel that the electricity bill is much less, wait a month to see how much can be saved!	Jingdong prefecture level city in Shandong
Fortune Shop	White Intelligent Power Saver	Received the stuff used for more than a month without a little use, it feels like it uses more electricity than before	Jingdong prefecture level city in Shandong
Fortune Shop	White Intelligent Power Saver	The quality is very good, completely exceeded the expectations, the delivery speed is very fast, the packaging is very careful and strict, very satisfied with a shopping	Jingdong prefecture level city in Shandong
Fortune Shop	White Intelligent Power Saver	Fake, doesn't work at all. Don't even sell it.	Jingdong prefecture level city in Shandong
Fortune Shop	White Intelligent Power Saver	This item doesn't seem to have much effect?	Jingdong prefecture level city in Shandong
Fortune Shop	White Intelligent Power Saver	This production is a bit fake, just look at the focus on Ming	Jingdong prefecture level city in Shandong
Fortune Shop	White Intelligent Power Saver	The logistics is fast the service attitude is very good.	Jingdong prefecture level city in Shandong

Fortune Shop	White Intelligent Power Saver	The meter is good quality, sturdy and durable. It is accurate. Easy to use. Saves a lot of things.	Jingdong prefecture level city in Shandong
Fortune Shop	White Intelligent Power Saver	Just received the goods, don't know how it is yet	Jingdong prefecture level city in Shandong
Fortune Shop	White Intelligent Power Saver	Energy saver received, logistics is very fast, the quality is very good, the appearance is also very beautiful power saving is the most important.	Jingdong prefecture level city in Shandong
Fortune Shop	White Intelligent Power Saver	It's good to talk about it, it's not practical to use it.	Jingdong prefecture level city in Shandong
Fortune Shop	White Intelligent Power Saver	I don't know if it's good for now, I'll comment after using it	Jingdong prefecture level city in Shandong
Fortune Shop	White Intelligent Power Saver	I've been using it for two or three days. It's not energy efficient.	Jingdong prefecture level city in Shandong
Fortune Shop	White Intelligent Power Saver	Packaging is intact, logistics is also fast, cost-effective, inexpensive, very satisfied with a purchase	Jingdong prefecture level city in Shandong
Fortune Shop	White Intelligent Power Saver	In use to be observed!	Jingdong prefecture level city in Shandong
Fortune Shop	White Intelligent Power Saver	Use it for a while at a flat rate	Jingdong prefecture level city in Shandong
Fortune Shop	White Intelligent Power Saver	Very trustworthy brand, quality is guaranteed, the price is still affordable, quite satisfied!	Jingdong prefecture level city in Shandong
Fortune Shop	White Intelligent Power Saver	Great quality, great operation, easy to install, great price!	Jingdong prefecture level city in Shandong
Fortune Shop	White Intelligent Power Saver	I came back and installed it, it really saves electricity, much more than before, the	Jingdong prefecture level city in Shandong
Fortune Shop	White Intelligent Power Saver	Fast logistics, good quality, very good packaging, good customer service	Jingdong prefecture level city in Shandong
Fortune Shop	White Intelligent Power Saver	Very good quality, especially good, effective, satisfactory	Jingdong prefecture level city in Shandong
Fortune Shop	White Intelligent Power Saver	I don't know how well it works.	Jingdong prefecture level city in Shandong
Fortune Shop	White Intelligent Power Saver	Good value for money, recommend it haha	Jingdong prefecture level city in Shandong
Fortune Shop	White Intelligent Power Saver	Good. Good. Very good.	Jingdong prefecture level city in Shandong
Fortune Shop	White Intelligent Power Saver	It's not working.	Jingdong prefecture level city in Shandong
Fortune Shop	White Intelligent Power Saver	It's not working.	Jingdong prefecture level city in Shandong
Fortune Shop	White Intelligent Power Saver	In use	Jingdong prefecture level city in Shandong
Fortune Shop	White Intelligent Power Saver	I'll try it later.	Jingdong prefecture level city in Shandong
Fortune Shop	White Intelligent Power Saver	It's not much use.	Jingdong prefecture level city in Shandong
Fortune Shop	White Intelligent Power Saver	It doesn't save electricity.	Jingdong prefecture level city in Shandong
Fortune Shop	White Intelligent Power Saver	It's not much use.	Jingdong prefecture level city in Shandong
Fortune Shop	White Intelligent Power Saver	Tried it for a few days and tracked the record, and there is no difference from before.	Jingdong prefecture level city in Shandong
Fortune Shop	White Intelligent Power Saver	It's not very credible, so just do it yourself and turn off the lights diligently!	Jingdong prefecture level city in Shandong
Fortune Shop	White Intelligent Power Saver	Too bad, no power saving at all, scam people don't fall for it!	Jingdong prefecture level city in Shandong
Fortune Shop	White Intelligent Power Saver	It's not working at all.	Jingdong prefecture level city in Shandong
Fortune Shop	White Intelligent Power Saver	general	Jingdong prefecture level city in Shandong
Fortune Shop	White Intelligent Power Saver	(of an unmarried couple) be close	Jingdong prefecture level city in Shandong
Fortune Shop	White Intelligent Power Saver	of no use	Jingdong prefecture level city in Shandong
Fortune Shop	White Intelligent Power Saver	Actually can really save electricity, only used half a month to save more than 50, recommended to the neighbors to use together!	Jingdong prefecture level city in Shandong
Fortune Shop	White Intelligent Power Saver	Save what you can. Isn't it more cost effective to buy one of these than to pay the electric bill?	Jingdong prefecture level city in Shandong
Fortune Shop	White Intelligent Power Saver	Received, a use do not know how, use a month to see the effect is good, the effect is good then recommended, not good then I just do not recommend.	Jingdong prefecture level city in Shandong
Fortune Shop	White Intelligent Power Saver	Just received the goods, is a voltage regulator, next month to evaluate whether it is really power saving	Jingdong prefecture level city in Shandong
Fortune Shop	White Intelligent Power Saver	Very good, used a few days obviously feel that the electricity bill is much less, wait a month to see how much can be saved!	Jingdong prefecture level city in Shandong
Fortune Shop	White Intelligent Power Saver	Received the stuff used for more than a month without a little use, it feels like it uses more electricity than before	Jingdong prefecture level city in Shandong
Fortune Shop	White Intelligent Power Saver	The quality is very good, completely exceeded the expectations, the delivery speed is very fast, the packaging is very careful and strict, very satisfied with a shopping	Jingdong prefecture level city in Shandong
Fortune Shop	White Intelligent Power Saver	Fake, doesn't work at all. Don't even sell it.	Jingdong prefecture level city in Shandong
Fortune Shop	White Intelligent Power Saver	This item doesn't seem to have much effect?	Jingdong prefecture level city in Shandong
Fortune Shop	White Intelligent Power Saver	This production is a bit fake, just look at the focus on Ming	Jingdong prefecture level city in Shandong
Fortune Shop	White Intelligent Power Saver	The logistics is fast the service attitude is very good.	Jingdong prefecture level city in Shandong
Fortune Shop	White Intelligent Power Saver	The meter is good quality, sturdy and durable. It is accurate. Easy to use. Saves a lot of things.	Jingdong prefecture level city in Shandong
Fortune Shop	White Intelligent Power Saver	Just received the goods, don't know how it is yet	Jingdong prefecture level city in Shandong
Fortune Shop	White Intelligent Power Saver	Energy saver received, logistics is very fast, the quality is very good, the appearance is also very beautiful power saving is the most important.	Jingdong prefecture level city in Shandong
Fortune Shop	White Intelligent Power Saver	It's good to talk about it, it's not practical to use it.	Jingdong prefecture level city in Shandong
Fortune Shop	White Intelligent Power Saver	I don't know if it's good for now, I'll comment after using it	Jingdong prefecture level city in Shandong
Fortune Shop	White Intelligent Power Saver	I've been using it for two or three days. It's not energy efficient.	Jingdong prefecture level city in Shandong
Fortune Shop	White Intelligent Power Saver	Packaging is intact, logistics is also fast, cost-effective, inexpensive, very satisfied with a purchase	Jingdong prefecture level city in Shandong
Fortune Shop	White Intelligent Power Saver	In use to be observed!	Jingdong prefecture level city in Shandong
Fortune Shop	White Intelligent Power Saver	Use it for a while at a flat rate	Jingdong prefecture level city in Shandong

Fortune Shop	White Intelligent Power Saver	Very trustworthy brand, quality is guaranteed, the price is still affordable, quite satisfied!	Jingdong prefecture level city in Shandong
Fortune Shop	White Intelligent Power Saver	Great quality, great operation, easy to install, great price!	Jingdong prefecture level city in Shandong
Fortune Shop	White Intelligent Power Saver	I came back and installed it, it really saves electricity, much more than before, the	Jingdong prefecture level city in Shandong
Fortune Shop	White Intelligent Power Saver	Fast logistics, good quality, very good packaging, good customer service	Jingdong prefecture level city in Shandong
Fortune Shop	White Intelligent Power Saver	Very good quality, especially good, effective, satisfactory	Jingdong prefecture level city in Shandong
Fortune Shop	White Intelligent Power Saver	I don't know how well it works.	Jingdong prefecture level city in Shandong
Fortune Shop	White Intelligent Power Saver	Good value for money, recommend it haha	Jingdong prefecture level city in Shandong
Fortune Shop	White Intelligent Power Saver	Good. Good. Very good.	Jingdong prefecture level city in Shandong
Fortune Shop	White Intelligent Power Saver	It's not working.	Jingdong prefecture level city in Shandong
Fortune Shop	White Intelligent Power Saver	It's not working.	Jingdong prefecture level city in Shandong
Fortune Shop	White Intelligent Power Saver	In use	Jingdong prefecture level city in Shandong
Fortune Shop	White Intelligent Power Saver	I'll try it later.	Jingdong prefecture level city in Shandong
Fortune Shop	White Intelligent Power Saver	It's not much use.	Jingdong prefecture level city in Shandong
Fortune Shop	White Intelligent Power Saver	It doesn't save electricity.	Jingdong prefecture level city in Shandong
Fortune Shop	White Intelligent Power Saver	It's not much use.	Jingdong prefecture level city in Shandong
Fortune Shop	White Intelligent Power Saver	Tried it for a few days and tracked the record, and there is no difference from before.	Jingdong prefecture level city in Shandong
Fortune Shop	White Intelligent Power Saver	It's not very credible, so just do it yourself and turn off the lights diligently!	Jingdong prefecture level city in Shandong
Fortune Shop	White Intelligent Power Saver	Too bad, no power saving at all, scam people don't fall for it!	Jingdong prefecture level city in Shandong
Fortune Shop	White Intelligent Power Saver	It's not working at all.	Jingdong prefecture level city in Shandong
Fortune Shop	White Intelligent Power Saver	general	Jingdong prefecture level city in Shandong
Fortune Shop	White Intelligent Power Saver	(of an unmarried couple) be close	Jingdong prefecture level city in Shandong
Fortune Shop	White Intelligent Power Saver	of no use	Jingdong prefecture level city in Shandong
Fortune Shop	White Intelligent Power Saver	Actually can really save electricity, only used half a month to save more than 50, recommended to the neighbors to use together!	Jingdong prefecture level city in Shandong
Fortune Shop	White Intelligent Power Saver	Save what you can. Isn't it more cost effective to buy one of these than to pay the electric bill?	Jingdong prefecture level city in Shandong
Fortune Shop	White Intelligent Power Saver	Received, a use do not know how, use a month to see the effect is good, the effect is good then recommended, not good then I just do not recommend.	Jingdong prefecture level city in Shandong
Fortune Shop	White Intelligent Power Saver	Just received the goods, is a voltage regulator, next month to evaluate whether it is really power saving	Jingdong prefecture level city in Shandong
Fortune Shop	White Intelligent Power Saver	Very good, used a few days obviously feel that the electricity bill is much less, wait a month to see how much can be saved!	Jingdong prefecture level city in Shandong
Fortune Shop	White Intelligent Power Saver	Received the stuff used for more than a month without a little use, it feels like it uses more electricity than before	Jingdong prefecture level city in Shandong
Fortune Shop	White Intelligent Power Saver	The quality is very good, completely exceeded the expectations, the delivery speed is very fast, the packaging is very careful and strict, very satisfied with a shopping	Jingdong prefecture level city in Shandong
Fortune Shop	White Intelligent Power Saver	Fake, doesn't work at all. Don't even sell it.	Jingdong prefecture level city in Shandong
Fortune Shop	White Intelligent Power Saver	This item doesn't seem to have much effect?	Jingdong prefecture level city in Shandong
Fortune Shop	White Intelligent Power Saver	This production is a bit fake, just look at the focus on Ming	Jingdong prefecture level city in Shandong
Fortune Shop	White Intelligent Power Saver	The logistics is fast the service attitude is very good.	Jingdong prefecture level city in Shandong
Fortune Shop	White Intelligent Power Saver	The meter is good quality, sturdy and durable. It is accurate. Easy to use. Saves a lot of things.	Jingdong prefecture level city in Shandong
Fortune Shop	White Intelligent Power Saver	Just received the goods, don't know how it is yet	Jingdong prefecture level city in Shandong
Fortune Shop	White Intelligent Power Saver	Energy saver received, logistics is very fast, the quality is very good, the appearance is also very beautiful power saving is the most important.	Jingdong prefecture level city in Shandong
Fortune Shop	White Intelligent Power Saver	It's good to talk about it, it's not practical to use it.	Jingdong prefecture level city in Shandong
Fortune Shop	White Intelligent Power Saver	I don't know if it's good for now, I'll comment after using it	Jingdong prefecture level city in Shandong
Fortune Shop	White Intelligent Power Saver	I've been using it for two or three days. It's not energy efficient.	Jingdong prefecture level city in Shandong
Fortune Shop	White Intelligent Power Saver	Packaging is intact, logistics is also fast, cost-effective, inexpensive, very satisfied with a purchase	Jingdong prefecture level city in Shandong
Fortune Shop	White Intelligent Power Saver	In use to be observed!	Jingdong prefecture level city in Shandong
Fortune Shop	White Intelligent Power Saver	Use it for a while at a flat rate	Jingdong prefecture level city in Shandong
Fortune Shop	White Intelligent Power Saver	Very trustworthy brand, quality is guaranteed, the price is still affordable, quite satisfied!	Jingdong prefecture level city in Shandong
Fortune Shop	White Intelligent Power Saver	Great quality, great operation, easy to install, great price!	Jingdong prefecture level city in Shandong
Fortune Shop	White Intelligent Power Saver	I came back and installed it, it really saves electricity, much more than before, the	Jingdong prefecture level city in Shandong
Fortune Shop	White Intelligent Power Saver	Fast logistics, good quality, very good packaging, good customer service	Jingdong prefecture level city in Shandong
Fortune Shop	White Intelligent Power Saver	Very good quality, especially good, effective, satisfactory	Jingdong prefecture level city in Shandong
Fortune Shop	White Intelligent Power Saver	I don't know how well it works.	Jingdong prefecture level city in Shandong
Fortune Shop	White Intelligent Power Saver	Good value for money, recommend it haha	Jingdong prefecture level city in Shandong
Fortune Shop	White Intelligent Power Saver	Good. Good. Very good.	Jingdong prefecture level city in Shandong
Fortune Shop	White Intelligent Power Saver	It's not working.	Jingdong prefecture level city in Shandong

Fortune Shop	White Intelligent Power Saver	It's not working.	Jingdong prefecture level city in Shandong
Fortune Shop	White Intelligent Power Saver	In use	Jingdong prefecture level city in Shandong
Fortune Shop	White Intelligent Power Saver	I'll try it later.	Jingdong prefecture level city in Shandong
Fortune Shop	White Intelligent Power Saver	It's not much use.	Jingdong prefecture level city in Shandong
Fortune Shop	White Intelligent Power Saver	It doesn't save electricity.	Jingdong prefecture level city in Shandong
Fortune Shop	White Intelligent Power Saver	It's not much use.	Jingdong prefecture level city in Shandong
Fortune Shop	White Intelligent Power Saver	Tried it for a few days and tracked the record, and there is no difference from before.	Jingdong prefecture level city in Shandong
Fortune Shop	White Intelligent Power Saver	It's not very credible, so just do it yourself and turn off the lights diligently!	Jingdong prefecture level city in Shandong
Fortune Shop	White Intelligent Power Saver	Too bad, no power saving at all, scam people don't fall for it!	Jingdong prefecture level city in Shandong
Fortune Shop	White Intelligent Power Saver	It's not working at all.	Jingdong prefecture level city in Shandong
Fortune Shop	White Intelligent Power Saver	general	Jingdong prefecture level city in Shandong
Fortune Shop	White Intelligent Power Saver	(of an unmarried couple) be close	Jingdong prefecture level city in Shandong
Fortune Shop	White Intelligent Power Saver	of no use	Jingdong prefecture level city in Shandong
Fortune Shop	White Intelligent Power Saver	Actually can really save electricity, only used half a month to save more than 50, recommended to the neighbors to use together!	Jingdong prefecture level city in Shandong
Fortune Shop	White Intelligent Power Saver	Save what you can. Isn't it more cost effective to buy one of these than to pay the electric bill?	Jingdong prefecture level city in Shandong
Fortune Shop	White Intelligent Power Saver	Received, a use do not know how, use a month to see the effect is good, the effect is good then recommended, not good then I just do not recommend.	Jingdong prefecture level city in Shandong
Fortune Shop	White Intelligent Power Saver	Just received the goods, is a voltage regulator, next month to evaluate whether it is really power saving	Jingdong prefecture level city in Shandong
Fortune Shop	White Intelligent Power Saver	Very good, used a few days obviously feel that the electricity bill is much less, wait a month to see how much can be saved!	Jingdong prefecture level city in Shandong
Fortune Shop	White Intelligent Power Saver	Received the stuff used for more than a month without a little use, it feels like it uses more electricity than before	Jingdong prefecture level city in Shandong
Fortune Shop	White Intelligent Power Saver	The quality is very good, completely exceeded the expectations, the delivery speed is very fast, the packaging is very careful and strict, very satisfied with a shopping	Jingdong prefecture level city in Shandong
Fortune Shop	White Intelligent Power Saver	Fake, doesn't work at all. Don't even sell it.	Jingdong prefecture level city in Shandong
Fortune Shop	White Intelligent Power Saver	This item doesn't seem to have much effect?	Jingdong prefecture level city in Shandong
Fortune Shop	White Intelligent Power Saver	This production is a bit fake, just look at the focus on Ming	Jingdong prefecture level city in Shandong
Fortune Shop	White Intelligent Power Saver	The logistics is fast the service attitude is very good.	Jingdong prefecture level city in Shandong
Fortune Shop	White Intelligent Power Saver	The meter is good quality, sturdy and durable. It is accurate. Easy to use. Saves a lot of things.	Jingdong prefecture level city in Shandong
Fortune Shop	White Intelligent Power Saver	Just received the goods, don't know how it is yet	Jingdong prefecture level city in Shandong
Fortune Shop	White Intelligent Power Saver	Energy saver received, logistics is very fast, the quality is very good, the appearance is also very beautiful power saving is the most important.	Jingdong prefecture level city in Shandong
Fortune Shop	White Intelligent Power Saver	It's good to talk about it, it's not practical to use it.	Jingdong prefecture level city in Shandong
Fortune Shop	White Intelligent Power Saver	I don't know if it's good for now, I'll comment after using it	Jingdong prefecture level city in Shandong
Fortune Shop	White Intelligent Power Saver	I've been using it for two or three days. It's not energy efficient.	Jingdong prefecture level city in Shandong
Fortune Shop	White Intelligent Power Saver	Packaging is intact, logistics is also fast, cost-effective, inexpensive, very satisfied with a purchase	Jingdong prefecture level city in Shandong
Fortune Shop	White Intelligent Power Saver	In use to be observed!	Jingdong prefecture level city in Shandong
Fortune Shop	White Intelligent Power Saver	Use it for a while at a flat rate	Jingdong prefecture level city in Shandong
Fortune Shop	White Intelligent Power Saver	Very trustworthy brand, quality is guaranteed, the price is still affordable, quite satisfied!	Jingdong prefecture level city in Shandong
Fortune Shop	White Intelligent Power Saver	Great quality, great operation, easy to install, great price!	Jingdong prefecture level city in Shandong
Fortune Shop	White Intelligent Power Saver	I came back and installed it, it really saves electricity, much more than before, the	Jingdong prefecture level city in Shandong
Fortune Shop	White Intelligent Power Saver	Fast logistics, good quality, very good packaging, good customer service	Jingdong prefecture level city in Shandong
Fortune Shop	White Intelligent Power Saver	Very good quality, especially good, effective, satisfactory	Jingdong prefecture level city in Shandong
Fortune Shop	White Intelligent Power Saver	I don't know how well it works.	Jingdong prefecture level city in Shandong
Fortune Shop	White Intelligent Power Saver	Good value for money, recommend it haha	Jingdong prefecture level city in Shandong
Fortune Shop	White Intelligent Power Saver	Good. Good. Very good.	Jingdong prefecture level city in Shandong
Fortune Shop	White Intelligent Power Saver	It's not working.	Jingdong prefecture level city in Shandong
Fortune Shop	White Intelligent Power Saver	It's not working.	Jingdong prefecture level city in Shandong
Fortune Shop	White Intelligent Power Saver	In use	Jingdong prefecture level city in Shandong
Fortune Shop	White Intelligent Power Saver	I'll try it later.	Jingdong prefecture level city in Shandong
Fortune Shop	White Intelligent Power Saver	It's not much use.	Jingdong prefecture level city in Shandong
Fortune Shop	White Intelligent Power Saver	It doesn't save electricity.	Jingdong prefecture level city in Shandong
Fortune Shop	White Intelligent Power Saver	It's not much use.	Jingdong prefecture level city in Shandong
Fortune Shop	White Intelligent Power Saver	Tried it for a few days and tracked the record, and there is no difference from before.	Jingdong prefecture level city in Shandong
Fortune Shop	White Intelligent Power Saver	It's not very credible, so just do it yourself and turn off the lights diligently!	Jingdong prefecture level city in Shandong
Fortune Shop	White Intelligent Power Saver	Too bad, no power saving at all, scam people don't fall for it!	Jingdong prefecture level city in Shandong

Fortune Shop	White Intelligent Power Saver	It's not working at all.	Jingdong prefecture level city in Shandong
Fortune Shop	White Intelligent Power Saver	general	Jingdong prefecture level city in Shandong
Fortune Shop	White Intelligent Power Saver	(of an unmarried couple) be close	Jingdong prefecture level city in Shandong
Fortune Shop	White Intelligent Power Saver	of no use	Jingdong prefecture level city in Shandong
Fortune Shop	White Intelligent Power Saver	Actually can really save electricity, only used half a month to save more than 50, recommended to the neighbors to use together!	Jingdong prefecture level city in Shandong
Fortune Shop	White Intelligent Power Saver	Save what you can. Isn't it more cost effective to buy one of these than to pay the electric bill?	Jingdong prefecture level city in Shandong
Fortune Shop	White Intelligent Power Saver	Received, a use do not know how, use a month to see the effect is good, the effect is good then recommended, not good then I just do not recommend.	Jingdong prefecture level city in Shandong
Fortune Shop	White Intelligent Power Saver	Just received the goods, is a voltage regulator, next month to evaluate whether it is really power saving	Jingdong prefecture level city in Shandong
Fortune Shop	White Intelligent Power Saver	Very good, used a few days obviously feel that the electricity bill is much less, wait a month to see how much can be saved!	Jingdong prefecture level city in Shandong
Fortune Shop	White Intelligent Power Saver	Received the stuff used for more than a month without a little use, it feels like it uses more electricity than before	Jingdong prefecture level city in Shandong
Fortune Shop	White Intelligent Power Saver	The quality is very good, completely exceeded the expectations, the delivery speed is very fast, the packaging is very careful and strict, very satisfied with a shopping	Jingdong prefecture level city in Shandong
Fortune Shop	White Intelligent Power Saver	Fake, doesn't work at all. Don't even sell it.	Jingdong prefecture level city in Shandong
Fortune Shop	White Intelligent Power Saver	This item doesn't seem to have much effect?	Jingdong prefecture level city in Shandong
Fortune Shop	White Intelligent Power Saver	This production is a bit fake, just look at the focus on Ming	Jingdong prefecture level city in Shandong
Fortune Shop	White Intelligent Power Saver	The logistics is fast the service attitude is very good.	Jingdong prefecture level city in Shandong
Fortune Shop	White Intelligent Power Saver	The meter is good quality, sturdy and durable. It is accurate. Easy to use. Saves a lot of things.	Jingdong prefecture level city in Shandong
Fortune Shop	White Intelligent Power Saver	Just received the goods, don't know how it is yet	Jingdong prefecture level city in Shandong
Fortune Shop	White Intelligent Power Saver	Energy saver received, logistics is very fast, the quality is very good, the appearance is also very beautiful power saving is the most important.	Jingdong prefecture level city in Shandong
Fortune Shop	White Intelligent Power Saver	It's good to talk about it, it's not practical to use it.	Jingdong prefecture level city in Shandong
Fortune Shop	White Intelligent Power Saver	I don't know if it's good for now, I'll comment after using it	Jingdong prefecture level city in Shandong
Fortune Shop	White Intelligent Power Saver	I've been using it for two or three days. It's not energy efficient.	Jingdong prefecture level city in Shandong
Fortune Shop	White Intelligent Power Saver	Packaging is intact, logistics is also fast, cost-effective, inexpensive, very satisfied with a purchase	Jingdong prefecture level city in Shandong
Fortune Shop	White Intelligent Power Saver	In use to be observed!	Jingdong prefecture level city in Shandong
Fortune Shop	White Intelligent Power Saver	Use it for a while at a flat rate	Jingdong prefecture level city in Shandong
Fortune Shop	White Intelligent Power Saver	Very trustworthy brand, quality is guaranteed, the price is still affordable, quite satisfied!	Jingdong prefecture level city in Shandong
Fortune Shop	White Intelligent Power Saver	Great quality, great operation, easy to install, great price!	Jingdong prefecture level city in Shandong
Fortune Shop	White Intelligent Power Saver	I came back and installed it, it really saves electricity, much more than before, the	Jingdong prefecture level city in Shandong
Fortune Shop	White Intelligent Power Saver	Fast logistics, good quality, very good packaging, good customer service	Jingdong prefecture level city in Shandong
Fortune Shop	White Intelligent Power Saver	Very good quality, especially good, effective, satisfactory	Jingdong prefecture level city in Shandong
Fortune Shop	White Intelligent Power Saver	I don't know how well it works.	Jingdong prefecture level city in Shandong
Fortune Shop	White Intelligent Power Saver	Good value for money, recommend it haha	Jingdong prefecture level city in Shandong
Fortune Shop	White Intelligent Power Saver	Good. Good. Very good.	Jingdong prefecture level city in Shandong
Fortune Shop	White Intelligent Power Saver	It's not working.	Jingdong prefecture level city in Shandong
Fortune Shop	White Intelligent Power Saver	It's not working.	Jingdong prefecture level city in Shandong
Fortune Shop	White Intelligent Power Saver	In use	Jingdong prefecture level city in Shandong
Fortune Shop	White Intelligent Power Saver	I'll try it later.	Jingdong prefecture level city in Shandong
Fortune Shop	White Intelligent Power Saver	It's not much use.	Jingdong prefecture level city in Shandong
Fortune Shop	White Intelligent Power Saver	It doesn't save electricity.	Jingdong prefecture level city in Shandong
Fortune Shop	White Intelligent Power Saver	It's not much use.	Jingdong prefecture level city in Shandong
Fortune Shop	White Intelligent Power Saver	Tried it for a few days and tracked the record, and there is no difference from before.	Jingdong prefecture level city in Shandong
Fortune Shop	White Intelligent Power Saver	It's not very credible, so just do it yourself and turn off the lights diligently!	Jingdong prefecture level city in Shandong
Fortune Shop	White Intelligent Power Saver	Too bad, no power saving at all, scam people don't fall for it!	Jingdong prefecture level city in Shandong
Fortune Shop	White Intelligent Power Saver	It's not working at all.	Jingdong prefecture level city in Shandong
Fortune Shop	White Intelligent Power Saver	general	Jingdong prefecture level city in Shandong
Fortune Shop	White Intelligent Power Saver	(of an unmarried couple) be close	Jingdong prefecture level city in Shandong
Fortune Shop	White Intelligent Power Saver	of no use	Jingdong prefecture level city in Shandong
Fortune Shop	White Intelligent Power Saver	Actually can really save electricity, only used half a month to save more than 50, recommended to the neighbors to use together!	Jingdong prefecture level city in Shandong
Fortune Shop	White Intelligent Power Saver	Save what you can. Isn't it more cost effective to buy one of these than to pay the electric bill?	Jingdong prefecture level city in Shandong
Fortune Shop	White Intelligent Power Saver	Received, a use do not know how, use a month to see the effect is good, the effect is good then recommended, not good then I just do not recommend.	Jingdong prefecture level city in Shandong
Fortune Shop	White Intelligent Power Saver	Just received the goods, is a voltage regulator, next month to evaluate whether it is really power saving	Jingdong prefecture level city in Shandong
Fortune Shop	White Intelligent Power Saver	Very good, used a few days obviously feel that the electricity bill is much less, wait a month to see how much can be saved!	Jingdong prefecture level city in Shandong

Fortune Shop	White Intelligent Power Saver	Received the stuff used for more than a month without a little use, it feels like it uses more electricity than before	Jingdong prefecture level city in Shandong
Fortune Shop	White Intelligent Power Saver	The quality is very good, completely exceeded the expectations, the delivery speed is very fast, the packaging is very careful and strict, very satisfied with a shopping	Jingdong prefecture level city in Shandong
Fortune Shop	White Intelligent Power Saver	Fake, doesn't work at all. Don't even sell it.	Jingdong prefecture level city in Shandong
Fortune Shop	White Intelligent Power Saver	This item doesn't seem to have much effect?	Jingdong prefecture level city in Shandong
Fortune Shop	White Intelligent Power Saver	This production is a bit fake, just look at the focus on Ming	Jingdong prefecture level city in Shandong
Fortune Shop	White Intelligent Power Saver	The logistics is fast the service attitude is very good.	Jingdong prefecture level city in Shandong
Fortune Shop	White Intelligent Power Saver	The meter is good quality, sturdy and durable. It is accurate. Easy to use. Saves a lot of things.	Jingdong prefecture level city in Shandong
Fortune Shop	White Intelligent Power Saver	Just received the goods, don't know how it is yet	Jingdong prefecture level city in Shandong
Fortune Shop	White Intelligent Power Saver	Energy saver received, logistics is very fast, the quality is very good, the appearance is also very beautiful power saving is the most important.	Jingdong prefecture level city in Shandong
Fortune Shop	White Intelligent Power Saver	It's good to talk about it, it's not practical to use it.	Jingdong prefecture level city in Shandong
Fortune Shop	White Intelligent Power Saver	I don't know if it's good for now, I'll comment after using it	Jingdong prefecture level city in Shandong
Fortune Shop	White Intelligent Power Saver	I've been using it for two or three days. It's not energy efficient.	Jingdong prefecture level city in Shandong
Fortune Shop	White Intelligent Power Saver	Packaging is intact, logistics is also fast, cost-effective, inexpensive, very satisfied with a purchase	Jingdong prefecture level city in Shandong
Fortune Shop	White Intelligent Power Saver	In use to be observed!	Jingdong prefecture level city in Shandong
Fortune Shop	White Intelligent Power Saver	Use it for a while at a flat rate	Jingdong prefecture level city in Shandong
Fortune Shop	White Intelligent Power Saver	Very trustworthy brand, quality is guaranteed, the price is still affordable, quite satisfied!	Jingdong prefecture level city in Shandong
Fortune Shop	White Intelligent Power Saver	Great quality, great operation, easy to install, great price!	Jingdong prefecture level city in Shandong
Fortune Shop	White Intelligent Power Saver	I came back and installed it, it really saves electricity, much more than before, the	Jingdong prefecture level city in Shandong
Fortune Shop	White Intelligent Power Saver	Fast logistics, good quality, very good packaging, good customer service	Jingdong prefecture level city in Shandong
Fortune Shop	White Intelligent Power Saver	Very good quality, especially good, effective, satisfactory	Jingdong prefecture level city in Shandong
Fortune Shop	White Intelligent Power Saver	I don't know how well it works.	Jingdong prefecture level city in Shandong
Fortune Shop	White Intelligent Power Saver	Good value for money, recommend it haha	Jingdong prefecture level city in Shandong
Fortune Shop	White Intelligent Power Saver	Good. Good. Very good.	Jingdong prefecture level city in Shandong
Fortune Shop	White Intelligent Power Saver	It's not working.	Jingdong prefecture level city in Shandong
Fortune Shop	White Intelligent Power Saver	It's not working.	Jingdong prefecture level city in Shandong
Fortune Shop	White Intelligent Power Saver	In use	Jingdong prefecture level city in Shandong
Fortune Shop	White Intelligent Power Saver	I'll try it later.	Jingdong prefecture level city in Shandong
Fortune Shop	White Intelligent Power Saver	It's not much use.	Jingdong prefecture level city in Shandong
Fortune Shop	White Intelligent Power Saver	It doesn't save electricity.	Jingdong prefecture level city in Shandong
Fortune Shop	White Intelligent Power Saver	It's not much use.	Jingdong prefecture level city in Shandong
Fortune Shop	White Intelligent Power Saver	Tried it for a few days and tracked the record, and there is no difference from before.	Jingdong prefecture level city in Shandong
Fortune Shop	White Intelligent Power Saver	It's not very credible, so just do it yourself and turn off the lights diligently!	Jingdong prefecture level city in Shandong
Fortune Shop	White Intelligent Power Saver	Too bad, no power saving at all, scam people don't fall for it!	Jingdong prefecture level city in Shandong
Fortune Shop	White Intelligent Power Saver	It's not working at all.	Jingdong prefecture level city in Shandong
Fortune Shop	White Intelligent Power Saver	general	Jingdong prefecture level city in Shandong
Fortune Shop	White Intelligent Power Saver	(of an unmarried couple) be close	Jingdong prefecture level city in Shandong
Fortune Shop	White Intelligent Power Saver	of no use	Jingdong prefecture level city in Shandong
Fortune Shop	White Intelligent Power Saver	Actually can really save electricity, only used half a month to save more than 50, recommended to the neighbors to use together!	Jingdong prefecture level city in Shandong
Fortune Shop	White Intelligent Power Saver	Save what you can. Isn't it more cost effective to buy one of these than to pay the electric bill?	Jingdong prefecture level city in Shandong
Fortune Shop	White Intelligent Power Saver	Received, a use do not know how, use a month to see the effect is good, the effect is good then recommended, not good then I just do not recommend.	Jingdong prefecture level city in Shandong
Fortune Shop	White Intelligent Power Saver	Just received the goods, is a voltage regulator, next month to evaluate whether it is really power saving	Jingdong prefecture level city in Shandong
Fortune Shop	White Intelligent Power Saver	Very good, used a few days obviously feel that the electricity bill is much less, wait a month to see how much can be saved!	Jingdong prefecture level city in Shandong
Fortune Shop	White Intelligent Power Saver	Received the stuff used for more than a month without a little use, it feels like it uses more electricity than before	Jingdong prefecture level city in Shandong
Fortune Shop	White Intelligent Power Saver	The quality is very good, completely exceeded the expectations, the delivery speed is very fast, the packaging is very careful and strict, very satisfied with a shopping	Jingdong prefecture level city in Shandong
Fortune Shop	White Intelligent Power Saver	Fake, doesn't work at all. Don't even sell it.	Jingdong prefecture level city in Shandong
Fortune Shop	White Intelligent Power Saver	This item doesn't seem to have much effect?	Jingdong prefecture level city in Shandong
Fortune Shop	White Intelligent Power Saver	This production is a bit fake, just look at the focus on Ming	Jingdong prefecture level city in Shandong
Fortune Shop	White Intelligent Power Saver	The logistics is fast the service attitude is very good.	Jingdong prefecture level city in Shandong
Fortune Shop	White Intelligent Power Saver	The meter is good quality, sturdy and durable. It is accurate. Easy to use. Saves a lot of things.	Jingdong prefecture level city in Shandong
Fortune Shop	White Intelligent Power Saver	Just received the goods, don't know how it is yet	Jingdong prefecture level city in Shandong
Fortune Shop	White Intelligent Power Saver	Energy saver received, logistics is very fast, the quality is very good, the appearance is also very beautiful power saving is the most important.	Jingdong prefecture level city in Shandong

Fortune Shop	White Intelligent Power Saver	It's good to talk about it, it's not practical to use it.	Jingdong prefecture level city in Shandong
Fortune Shop	White Intelligent Power Saver	I don't know if it's good for now, I'll comment after using it	Jingdong prefecture level city in Shandong
Fortune Shop	White Intelligent Power Saver	I've been using it for two or three days. It's not energy efficient.	Jingdong prefecture level city in Shandong
Fortune Shop	White Intelligent Power Saver	Packaging is intact, logistics is also fast, cost-effective, inexpensive, very satisfied with a purchase	Jingdong prefecture level city in Shandong
Fortune Shop	White Intelligent Power Saver	In use to be observed!	Jingdong prefecture level city in Shandong
Fortune Shop	White Intelligent Power Saver	Use it for a while at a flat rate	Jingdong prefecture level city in Shandong
Fortune Shop	White Intelligent Power Saver	Very trustworthy brand, quality is guaranteed, the price is still affordable, quite satisfied!	Jingdong prefecture level city in Shandong
Fortune Shop	White Intelligent Power Saver	Great quality, great operation, easy to install, great price!	Jingdong prefecture level city in Shandong
Fortune Shop	White Intelligent Power Saver	I came back and installed it, it really saves electricity, much more than before, the	Jingdong prefecture level city in Shandong
Fortune Shop	White Intelligent Power Saver	Fast logistics, good quality, very good packaging, good customer service	Jingdong prefecture level city in Shandong
Fortune Shop	White Intelligent Power Saver	Very good quality, especially good, effective, satisfactory	Jingdong prefecture level city in Shandong
Fortune Shop	White Intelligent Power Saver	I don't know how well it works.	Jingdong prefecture level city in Shandong
Fortune Shop	White Intelligent Power Saver	Good value for money, recommend it haha	Jingdong prefecture level city in Shandong
Fortune Shop	White Intelligent Power Saver	Good. Good. Very good.	Jingdong prefecture level city in Shandong
Fortune Shop	White Intelligent Power Saver	It's not working.	Jingdong prefecture level city in Shandong
Fortune Shop	White Intelligent Power Saver	It's not working.	Jingdong prefecture level city in Shandong
Fortune Shop	White Intelligent Power Saver	In use	Jingdong prefecture level city in Shandong
Fortune Shop	White Intelligent Power Saver	I'll try it later.	Jingdong prefecture level city in Shandong
Fortune Shop	White Intelligent Power Saver	It's not much use.	Jingdong prefecture level city in Shandong
Fortune Shop	White Intelligent Power Saver	It doesn't save electricity.	Jingdong prefecture level city in Shandong
Fortune Shop	White Intelligent Power Saver	It's not much use.	Jingdong prefecture level city in Shandong
Fortune Shop	White Intelligent Power Saver	Tried it for a few days and tracked the record, and there is no difference from before.	Jingdong prefecture level city in Shandong
Fortune Shop	White Intelligent Power Saver	It's not very credible, so just do it yourself and turn off the lights diligently!	Jingdong prefecture level city in Shandong
Fortune Shop	White Intelligent Power Saver	Too bad, no power saving at all, scam people don't fall for it!	Jingdong prefecture level city in Shandong
Fortune Shop	White Intelligent Power Saver	It's not working at all.	Jingdong prefecture level city in Shandong
Fortune Shop	White Intelligent Power Saver	general	Jingdong prefecture level city in Shandong
Fortune Shop	White Intelligent Power Saver	(of an unmarried couple) be close	Jingdong prefecture level city in Shandong
Fortune Shop	White Intelligent Power Saver	of no use	Jingdong prefecture level city in Shandong
Fortune Shop	White Intelligent Power Saver	Actually can really save electricity, only used half a month to save more than 50, recommended to the neighbors to use together!	Jingdong prefecture level city in Shandong
Fortune Shop	White Intelligent Power Saver	Save what you can. Isn't it more cost effective to buy one of these than to pay the electric bill?	Jingdong prefecture level city in Shandong
Fortune Shop	White Intelligent Power Saver	Received, a use do not know how, use a month to see the effect is good, the effect is good then recommended, not good then I just do not recommend.	Jingdong prefecture level city in Shandong
Fortune Shop	White Intelligent Power Saver	Just received the goods, is a voltage regulator, next month to evaluate whether it is really power saving	Jingdong prefecture level city in Shandong
Fortune Shop	White Intelligent Power Saver	Very good, used a few days obviously feel that the electricity bill is much less, wait a month to see how much can be saved!	Jingdong prefecture level city in Shandong
Fortune Shop	White Intelligent Power Saver	Received the stuff used for more than a month without a little use, it feels like it uses more electricity than before	Jingdong prefecture level city in Shandong
Fortune Shop	White Intelligent Power Saver	The quality is very good, completely exceeded the expectations, the delivery speed is very fast, the packaging is very careful and strict, very satisfied with a shopping	Jingdong prefecture level city in Shandong
Fortune Shop	White Intelligent Power Saver	Fake, doesn't work at all. Don't even sell it.	Jingdong prefecture level city in Shandong
Fortune Shop	White Intelligent Power Saver	This item doesn't seem to have much effect?	Jingdong prefecture level city in Shandong
Fortune Shop	White Intelligent Power Saver	This production is a bit fake, just look at the focus on Ming	Jingdong prefecture level city in Shandong
Fortune Shop	White Intelligent Power Saver	The logistics is fast the service attitude is very good.	Jingdong prefecture level city in Shandong
Fortune Shop	White Intelligent Power Saver	The meter is good quality, sturdy and durable. It is accurate. Easy to use. Saves a lot of things.	Jingdong prefecture level city in Shandong
Fortune Shop	White Intelligent Power Saver	Just received the goods, don't know how it is yet	Jingdong prefecture level city in Shandong
Fortune Shop	White Intelligent Power Saver	Energy saver received, logistics is very fast, the quality is very good, the appearance is also very beautiful power saving is the most important.	Jingdong prefecture level city in Shandong
Fortune Shop	White Intelligent Power Saver	It's good to talk about it, it's not practical to use it.	Jingdong prefecture level city in Shandong
Fortune Shop	White Intelligent Power Saver	I don't know if it's good for now, I'll comment after using it	Jingdong prefecture level city in Shandong
Fortune Shop	White Intelligent Power Saver	I've been using it for two or three days. It's not energy efficient.	Jingdong prefecture level city in Shandong
Fortune Shop	White Intelligent Power Saver	Packaging is intact, logistics is also fast, cost-effective, inexpensive, very satisfied with a purchase	Jingdong prefecture level city in Shandong
Fortune Shop	White Intelligent Power Saver	In use to be observed!	Jingdong prefecture level city in Shandong
Fortune Shop	White Intelligent Power Saver	Use it for a while at a flat rate	Jingdong prefecture level city in Shandong
Fortune Shop	White Intelligent Power Saver	Very trustworthy brand, quality is guaranteed, the price is still affordable, quite satisfied!	Jingdong prefecture level city in Shandong
Fortune Shop	White Intelligent Power Saver	Great quality, great operation, easy to install, great price!	Jingdong prefecture level city in Shandong
Fortune Shop	White Intelligent Power Saver	I came back and installed it, it really saves electricity, much more than before, the	Jingdong prefecture level city in Shandong

Fortune Shop	White Intelligent Power Saver	Fast logistics, good quality, very good packaging, good customer service	Jingdong prefecture level city in Shandong
Fortune Shop	White Intelligent Power Saver	Very good quality, especially good, effective, satisfactory	Jingdong prefecture level city in Shandong
Fortune Shop	White Intelligent Power Saver	I don't know how well it works.	Jingdong prefecture level city in Shandong
Fortune Shop	White Intelligent Power Saver	Good value for money, recommend it haha	Jingdong prefecture level city in Shandong
Fortune Shop	White Intelligent Power Saver	Good. Good. Very good.	Jingdong prefecture level city in Shandong
Fortune Shop	White Intelligent Power Saver	It's not working.	Jingdong prefecture level city in Shandong
Fortune Shop	White Intelligent Power Saver	It's not working.	Jingdong prefecture level city in Shandong
Fortune Shop	White Intelligent Power Saver	In use	Jingdong prefecture level city in Shandong
Fortune Shop	White Intelligent Power Saver	I'll try it later.	Jingdong prefecture level city in Shandong
Fortune Shop	White Intelligent Power Saver	It's not much use.	Jingdong prefecture level city in Shandong
Fortune Shop	White Intelligent Power Saver	It doesn't save electricity.	Jingdong prefecture level city in Shandong
Fortune Shop	White Intelligent Power Saver	It's not much use.	Jingdong prefecture level city in Shandong
Fortune Shop	White Intelligent Power Saver	Tried it for a few days and tracked the record, and there is no difference from before.	Jingdong prefecture level city in Shandong
Fortune Shop	White Intelligent Power Saver	It's not very credible, so just do it yourself and turn off the lights diligently!	Jingdong prefecture level city in Shandong
Fortune Shop	White Intelligent Power Saver	Too bad, no power saving at all, scam people don't fall for it!	Jingdong prefecture level city in Shandong
Fortune Shop	White Intelligent Power Saver	It's not working at all.	Jingdong prefecture level city in Shandong
Fortune Shop	White Intelligent Power Saver	general	Jingdong prefecture level city in Shandong
Fortune Shop	White Intelligent Power Saver	(of an unmarried couple) be close	Jingdong prefecture level city in Shandong
Fortune Shop	White Intelligent Power Saver	of no use	Jingdong prefecture level city in Shandong
Fortune Shop	White Intelligent Power Saver	Actually can really save electricity, only used half a month to save more than 50, recommended to the neighbors to use together!	Jingdong prefecture level city in Shandong
Fortune Shop	White Intelligent Power Saver	Save what you can. Isn't it more cost effective to buy one of these than to pay the electric bill?	Jingdong prefecture level city in Shandong
Fortune Shop	White Intelligent Power Saver	Received, a use do not know how, use a month to see the effect is good, the effect is good then recommended, not good then I just do not recommend.	Jingdong prefecture level city in Shandong
Fortune Shop	White Intelligent Power Saver	Just received the goods, is a voltage regulator, next month to evaluate whether it is really power saving	Jingdong prefecture level city in Shandong
Fortune Shop	White Intelligent Power Saver	Very good, used a few days obviously feel that the electricity bill is much less, wait a month to see how much can be saved!	Jingdong prefecture level city in Shandong
Fortune Shop	White Intelligent Power Saver	Received the stuff used for more than a month without a little use, it feels like it uses more electricity than before	Jingdong prefecture level city in Shandong
Fortune Shop	White Intelligent Power Saver	The quality is very good, completely exceeded the expectations, the delivery speed is very fast, the packaging is very careful and strict, very satisfied with a shopping	Jingdong prefecture level city in Shandong
Fortune Shop	White Intelligent Power Saver	Fake, doesn't work at all. Don't even sell it.	Jingdong prefecture level city in Shandong
Fortune Shop	White Intelligent Power Saver	This item doesn't seem to have much effect?	Jingdong prefecture level city in Shandong
Fortune Shop	White Intelligent Power Saver	This production is a bit fake, just look at the focus on Ming	Jingdong prefecture level city in Shandong
Fortune Shop	White Intelligent Power Saver	The logistics is fast the service attitude is very good.	Jingdong prefecture level city in Shandong
Fortune Shop	White Intelligent Power Saver	The meter is good quality, sturdy and durable. It is accurate. Easy to use. Saves a lot of things.	Jingdong prefecture level city in Shandong
Fortune Shop	White Intelligent Power Saver	Just received the goods, don't know how it is yet	Jingdong prefecture level city in Shandong
Fortune Shop	White Intelligent Power Saver	Energy saver received, logistics is very fast, the quality is very good, the appearance is also very beautiful power saving is the most important.	Jingdong prefecture level city in Shandong
Fortune Shop	White Intelligent Power Saver	It's good to talk about it, it's not practical to use it.	Jingdong prefecture level city in Shandong
Fortune Shop	White Intelligent Power Saver	I don't know if it's good for now, I'll comment after using it	Jingdong prefecture level city in Shandong
Fortune Shop	White Intelligent Power Saver	I've been using it for two or three days. It's not energy efficient.	Jingdong prefecture level city in Shandong
Fortune Shop	White Intelligent Power Saver	Packaging is intact, logistics is also fast, cost-effective, inexpensive, very satisfied with a purchase	Jingdong prefecture level city in Shandong
Fortune Shop	White Intelligent Power Saver	In use to be observed!	Jingdong prefecture level city in Shandong
Fortune Shop	White Intelligent Power Saver	Use it for a while at a flat rate	Jingdong prefecture level city in Shandong
Fortune Shop	White Intelligent Power Saver	Very trustworthy brand, quality is guaranteed, the price is still affordable, quite satisfied!	Jingdong prefecture level city in Shandong
Fortune Shop	White Intelligent Power Saver	Great quality, great operation, easy to install, great price!	Jingdong prefecture level city in Shandong
Fortune Shop	White Intelligent Power Saver	I came back and installed it, it really saves electricity, much more than before, the	Jingdong prefecture level city in Shandong
Fortune Shop	White Intelligent Power Saver	Fast logistics, good quality, very good packaging, good customer service	Jingdong prefecture level city in Shandong
Fortune Shop	White Intelligent Power Saver	Very good quality, especially good, effective, satisfactory	Jingdong prefecture level city in Shandong
Fortune Shop	White Intelligent Power Saver	I don't know how well it works.	Jingdong prefecture level city in Shandong
Fortune Shop	White Intelligent Power Saver	Good value for money, recommend it haha	Jingdong prefecture level city in Shandong
Fortune Shop	White Intelligent Power Saver	Good. Good. Very good.	Jingdong prefecture level city in Shandong
Fortune Shop	White Intelligent Power Saver	It's not working.	Jingdong prefecture level city in Shandong
Fortune Shop	White Intelligent Power Saver	It's not working.	Jingdong prefecture level city in Shandong
Fortune Shop	White Intelligent Power Saver	In use	Jingdong prefecture level city in Shandong
Fortune Shop	White Intelligent Power Saver	I'll try it later.	Jingdong prefecture level city in Shandong

Fortune Shop	White Intelligent Power Saver	It's not much use.	Jingdong prefecture level city in Shandong
Fortune Shop	White Intelligent Power Saver	It doesn't save electricity.	Jingdong prefecture level city in Shandong
Fortune Shop	White Intelligent Power Saver	It's not much use.	Jingdong prefecture level city in Shandong
Fortune Shop	White Intelligent Power Saver	Tried it for a few days and tracked the record, and there is no difference from before.	Jingdong prefecture level city in Shandong
Fortune Shop	White Intelligent Power Saver	It's not very credible, so just do it yourself and turn off the lights diligently!	Jingdong prefecture level city in Shandong
Fortune Shop	White Intelligent Power Saver	Too bad, no power saving at all, scam people don't fall for it!	Jingdong prefecture level city in Shandong
Fortune Shop	White Intelligent Power Saver	It's not working at all.	Jingdong prefecture level city in Shandong
Fortune Shop	White Intelligent Power Saver	general	Jingdong prefecture level city in Shandong
Fortune Shop	White Intelligent Power Saver	(of an unmarried couple) be close	Jingdong prefecture level city in Shandong
Fortune Shop	White Intelligent Power Saver	of no use	Jingdong prefecture level city in Shandong
Fortune Shop	White Intelligent Power Saver	Actually can really save electricity, only used half a month to save more than 50, recommended to the neighbors to use together!	Jingdong prefecture level city in Shandong
Fortune Shop	White Intelligent Power Saver	Save what you can. Isn't it more cost effective to buy one of these than to pay the electric bill?	Jingdong prefecture level city in Shandong
Fortune Shop	White Intelligent Power Saver	Received, a use do not know how, use a month to see the effect is good, the effect is good then recommended, not good then I just do not recommend.	Jingdong prefecture level city in Shandong
Fortune Shop	White Intelligent Power Saver	Just received the goods, is a voltage regulator, next month to evaluate whether it is really power saving	Jingdong prefecture level city in Shandong
Fortune Shop	White Intelligent Power Saver	Very good, used a few days obviously feel that the electricity bill is much less, wait a month to see how much can be saved!	Jingdong prefecture level city in Shandong
Fortune Shop	White Intelligent Power Saver	Received the stuff used for more than a month without a little use, it feels like it uses more electricity than before	Jingdong prefecture level city in Shandong
Fortune Shop	White Intelligent Power Saver	The quality is very good, completely exceeded the expectations, the delivery speed is very fast, the packaging is very careful and strict, very satisfied with a shopping	Jingdong prefecture level city in Shandong
Fortune Shop	White Intelligent Power Saver	Fake, doesn't work at all. Don't even sell it.	Jingdong prefecture level city in Shandong
Fortune Shop	White Intelligent Power Saver	This item doesn't seem to have much effect?	Jingdong prefecture level city in Shandong
Fortune Shop	White Intelligent Power Saver	This production is a bit fake, just look at the focus on Ming	Jingdong prefecture level city in Shandong
Fortune Shop	White Intelligent Power Saver	The logistics is fast the service attitude is very good.	Jingdong prefecture level city in Shandong
Fortune Shop	White Intelligent Power Saver	The meter is good quality, sturdy and durable. It is accurate. Easy to use. Saves a lot of things.	Jingdong prefecture level city in Shandong
Fortune Shop	White Intelligent Power Saver	Just received the goods, don't know how it is yet	Jingdong prefecture level city in Shandong
Fortune Shop	White Intelligent Power Saver	Energy saver received, logistics is very fast, the quality is very good, the appearance is also very beautiful power saving is the most important.	Jingdong prefecture level city in Shandong
Fortune Shop	White Intelligent Power Saver	It's good to talk about it, it's not practical to use it.	Jingdong prefecture level city in Shandong
Fortune Shop	White Intelligent Power Saver	I don't know if it's good for now, I'll comment after using it	Jingdong prefecture level city in Shandong
Fortune Shop	White Intelligent Power Saver	I've been using it for two or three days. It's not energy efficient.	Jingdong prefecture level city in Shandong
Fortune Shop	White Intelligent Power Saver	Packaging is intact, logistics is also fast, cost-effective, inexpensive, very satisfied with a purchase	Jingdong prefecture level city in Shandong
Fortune Shop	White Intelligent Power Saver	In use to be observed!	Jingdong prefecture level city in Shandong
Fortune Shop	White Intelligent Power Saver	Use it for a while at a flat rate	Jingdong prefecture level city in Shandong
Fortune Shop	White Intelligent Power Saver	Very trustworthy brand, quality is guaranteed, the price is still affordable, quite satisfied!	Jingdong prefecture level city in Shandong
Fortune Shop	White Intelligent Power Saver	Great quality, great operation, easy to install, great price!	Jingdong prefecture level city in Shandong
Fortune Shop	White Intelligent Power Saver	I came back and installed it, it really saves electricity, much more than before, the	Jingdong prefecture level city in Shandong
Fortune Shop	White Intelligent Power Saver	Fast logistics, good quality, very good packaging, good customer service	Jingdong prefecture level city in Shandong
Fortune Shop	White Intelligent Power Saver	Very good quality, especially good, effective, satisfactory	Jingdong prefecture level city in Shandong
Fortune Shop	White Intelligent Power Saver	I don't know how well it works.	Jingdong prefecture level city in Shandong
Fortune Shop	White Intelligent Power Saver	Good value for money, recommend it haha	Jingdong prefecture level city in Shandong
Fortune Shop	White Intelligent Power Saver	Good. Good. Very good.	Jingdong prefecture level city in Shandong
Fortune Shop	White Intelligent Power Saver	It's not working.	Jingdong prefecture level city in Shandong
Fortune Shop	White Intelligent Power Saver	It's not working.	Jingdong prefecture level city in Shandong
Fortune Shop	White Intelligent Power Saver	In use	Jingdong prefecture level city in Shandong
Fortune Shop	White Intelligent Power Saver	I'll try it later.	Jingdong prefecture level city in Shandong
Fortune Shop	White Intelligent Power Saver	It's not much use.	Jingdong prefecture level city in Shandong
Fortune Shop	White Intelligent Power Saver	It doesn't save electricity.	Jingdong prefecture level city in Shandong
Fortune Shop	White Intelligent Power Saver	It's not much use.	Jingdong prefecture level city in Shandong
Fortune Shop	White Intelligent Power Saver	Tried it for a few days and tracked the record, and there is no difference from before.	Jingdong prefecture level city in Shandong
Fortune Shop	White Intelligent Power Saver	It's not very credible, so just do it yourself and turn off the lights diligently!	Jingdong prefecture level city in Shandong
Fortune Shop	White Intelligent Power Saver	Too bad, no power saving at all, scam people don't fall for it!	Jingdong prefecture level city in Shandong
Fortune Shop	White Intelligent Power Saver	It's not working at all.	Jingdong prefecture level city in Shandong
Fortune Shop	White Intelligent Power Saver	general	Jingdong prefecture level city in Shandong
Fortune Shop	White Intelligent Power Saver	(of an unmarried couple) be close	Jingdong prefecture level city in Shandong

Fortune Shop	White Intelligent Power Saver	of no use	Jingdong prefecture level city in Shandong
Fortune Shop	White Intelligent Power Saver	Actually can really save electricity, only used half a month to save more than 50, recommended to the neighbors to use together!	Jingdong prefecture level city in Shandong
Fortune Shop	White Intelligent Power Saver	Save what you can. Isn't it more cost effective to buy one of these than to pay the electric bill?	Jingdong prefecture level city in Shandong
Fortune Shop	White Intelligent Power Saver	Received, a use do not know how, use a month to see the effect is good, the effect is good then recommended, not good then I just do not recommend.	Jingdong prefecture level city in Shandong
Fortune Shop	White Intelligent Power Saver	Just received the goods, is a voltage regulator, next month to evaluate whether it is really power saving	Jingdong prefecture level city in Shandong
Fortune Shop	White Intelligent Power Saver	Very good, used a few days obviously feel that the electricity bill is much less, wait a month to see how much can be saved!	Jingdong prefecture level city in Shandong
Fortune Shop	White Intelligent Power Saver	Received the stuff used for more than a month without a little use, it feels like it uses more electricity than before	Jingdong prefecture level city in Shandong
Fortune Shop	White Intelligent Power Saver	The quality is very good, completely exceeded the expectations, the delivery speed is very fast, the packaging is very careful and strict, very satisfied with a shopping	Jingdong prefecture level city in Shandong
Fortune Shop	White Intelligent Power Saver	Fake, doesn't work at all. Don't even sell it.	Jingdong prefecture level city in Shandong
Fortune Shop	White Intelligent Power Saver	This item doesn't seem to have much effect?	Jingdong prefecture level city in Shandong
Fortune Shop	White Intelligent Power Saver	This production is a bit fake, just look at the focus on Ming	Jingdong prefecture level city in Shandong
Fortune Shop	White Intelligent Power Saver	The logistics is fast the service attitude is very good.	Jingdong prefecture level city in Shandong
Fortune Shop	White Intelligent Power Saver	The meter is good quality, sturdy and durable. It is accurate. Easy to use. Saves a lot of things.	Jingdong prefecture level city in Shandong
Fortune Shop	White Intelligent Power Saver	Just received the goods, don't know how it is yet	Jingdong prefecture level city in Shandong
Fortune Shop	White Intelligent Power Saver	Energy saver received, logistics is very fast, the quality is very good, the appearance is also very beautiful power saving is the most important.	Jingdong prefecture level city in Shandong
Fortune Shop	White Intelligent Power Saver	It's good to talk about it, it's not practical to use it.	Jingdong prefecture level city in Shandong
Fortune Shop	White Intelligent Power Saver	I don't know if it's good for now, I'll comment after using it	Jingdong prefecture level city in Shandong
Fortune Shop	White Intelligent Power Saver	I've been using it for two or three days. It's not energy efficient.	Jingdong prefecture level city in Shandong
Fortune Shop	White Intelligent Power Saver	Packaging is intact, logistics is also fast, cost-effective, inexpensive, very satisfied with a purchase	Jingdong prefecture level city in Shandong
Fortune Shop	White Intelligent Power Saver	In use to be observed!	Jingdong prefecture level city in Shandong
Fortune Shop	White Intelligent Power Saver	Use it for a while at a flat rate	Jingdong prefecture level city in Shandong
Fortune Shop	White Intelligent Power Saver	Very trustworthy brand, quality is guaranteed, the price is still affordable, quite satisfied!	Jingdong prefecture level city in Shandong
Fortune Shop	White Intelligent Power Saver	Great quality, great operation, easy to install, great price!	Jingdong prefecture level city in Shandong
Fortune Shop	White Intelligent Power Saver	I came back and installed it, it really saves electricity, much more than before, the	Jingdong prefecture level city in Shandong
Fortune Shop	White Intelligent Power Saver	Fast logistics, good quality, very good packaging, good customer service	Jingdong prefecture level city in Shandong
Fortune Shop	White Intelligent Power Saver	Very good quality, especially good, effective, satisfactory	Jingdong prefecture level city in Shandong
Fortune Shop	White Intelligent Power Saver	I don't know how well it works.	Jingdong prefecture level city in Shandong
Fortune Shop	White Intelligent Power Saver	Good value for money, recommend it haha	Jingdong prefecture level city in Shandong
Fortune Shop	White Intelligent Power Saver	Good. Good. Very good.	Jingdong prefecture level city in Shandong
Fortune Shop	White Intelligent Power Saver	It's not working.	Jingdong prefecture level city in Shandong
Fortune Shop	White Intelligent Power Saver	It's not working.	Jingdong prefecture level city in Shandong
Fortune Shop	White Intelligent Power Saver	In use	Jingdong prefecture level city in Shandong
Fortune Shop	White Intelligent Power Saver	I'll try it later.	Jingdong prefecture level city in Shandong
Fortune Shop	White Intelligent Power Saver	It's not much use.	Jingdong prefecture level city in Shandong
Fortune Shop	White Intelligent Power Saver	It doesn't save electricity.	Jingdong prefecture level city in Shandong
Fortune Shop	White Intelligent Power Saver	It's not much use.	Jingdong prefecture level city in Shandong
Fortune Shop	White Intelligent Power Saver	Tried it for a few days and tracked the record, and there is no difference from before.	Jingdong prefecture level city in Shandong
Fortune Shop	White Intelligent Power Saver	It's not very credible, so just do it yourself and turn off the lights diligently!	Jingdong prefecture level city in Shandong
Fortune Shop	White Intelligent Power Saver	Too bad, no power saving at all, scam people don't fall for it!	Jingdong prefecture level city in Shandong
Fortune Shop	White Intelligent Power Saver	It's not working at all.	Jingdong prefecture level city in Shandong
Fortune Shop	White Intelligent Power Saver	general	Jingdong prefecture level city in Shandong
Fortune Shop	White Intelligent Power Saver	(of an unmarried couple) be close	Jingdong prefecture level city in Shandong
Fortune Shop	White Intelligent Power Saver	of no use	Jingdong prefecture level city in Shandong
Fortune Shop	White Intelligent Power Saver	Actually can really save electricity, only used half a month to save more than 50, recommended to the neighbors to use together!	Jingdong prefecture level city in Shandong
Fortune Shop	White Intelligent Power Saver	Save what you can. Isn't it more cost effective to buy one of these than to pay the electric bill?	Jingdong prefecture level city in Shandong
Fortune Shop	White Intelligent Power Saver	Received, a use do not know how, use a month to see the effect is good, the effect is good then recommended, not good then I just do not recommend.	Jingdong prefecture level city in Shandong
Fortune Shop	White Intelligent Power Saver	Just received the goods, is a voltage regulator, next month to evaluate whether it is really power saving	Jingdong prefecture level city in Shandong
Fortune Shop	White Intelligent Power Saver	Very good, used a few days obviously feel that the electricity bill is much less, wait a month to see how much can be saved!	Jingdong prefecture level city in Shandong
Fortune Shop	White Intelligent Power Saver	Received the stuff used for more than a month without a little use, it feels like it uses more electricity than before	Jingdong prefecture level city in Shandong
Fortune Shop	White Intelligent Power Saver	The quality is very good, completely exceeded the expectations, the delivery speed is very fast, the packaging is very careful and strict, very satisfied with a shopping	Jingdong prefecture level city in Shandong
Fortune Shop	White Intelligent Power Saver	Fake, doesn't work at all. Don't even sell it.	Jingdong prefecture level city in Shandong

Fortune Shop	White Intelligent Power Saver	This item doesn't seem to have much effect?	Jingdong prefecture level city in Shandong
Fortune Shop	White Intelligent Power Saver	This production is a bit fake, just look at the focus on Ming	Jingdong prefecture level city in Shandong
Fortune Shop	White Intelligent Power Saver	The logistics is fast the service attitude is very good.	Jingdong prefecture level city in Shandong
Fortune Shop	White Intelligent Power Saver	The meter is good quality, sturdy and durable. It is accurate. Easy to use. Saves a lot of things.	Jingdong prefecture level city in Shandong
Fortune Shop	White Intelligent Power Saver	Just received the goods, don't know how it is yet	Jingdong prefecture level city in Shandong
Fortune Shop	White Intelligent Power Saver	Energy saver received, logistics is very fast, the quality is very good, the appearance is also very beautiful power saving is the most important.	Jingdong prefecture level city in Shandong
Fortune Shop	White Intelligent Power Saver	It's good to talk about it, it's not practical to use it.	Jingdong prefecture level city in Shandong
Fortune Shop	White Intelligent Power Saver	I don't know if it's good for now, I'll comment after using it	Jingdong prefecture level city in Shandong
Fortune Shop	White Intelligent Power Saver	I've been using it for two or three days. It's not energy efficient.	Jingdong prefecture level city in Shandong
Fortune Shop	White Intelligent Power Saver	Packaging is intact, logistics is also fast, cost-effective, inexpensive, very satisfied with a purchase	Jingdong prefecture level city in Shandong
Fortune Shop	White Intelligent Power Saver	In use to be observed!	Jingdong prefecture level city in Shandong
Fortune Shop	White Intelligent Power Saver	Use it for a while at a flat rate	Jingdong prefecture level city in Shandong
Fortune Shop	White Intelligent Power Saver	Very trustworthy brand, quality is guaranteed, the price is still affordable, quite satisfied!	Jingdong prefecture level city in Shandong
Fortune Shop	White Intelligent Power Saver	Great quality, great operation, easy to install, great price!	Jingdong prefecture level city in Shandong
Fortune Shop	White Intelligent Power Saver	I came back and installed it, it really saves electricity, much more than before, the	Jingdong prefecture level city in Shandong
Fortune Shop	White Intelligent Power Saver	Fast logistics, good quality, very good packaging, good customer service	Jingdong prefecture level city in Shandong
Fortune Shop	White Intelligent Power Saver	Very good quality, especially good, effective, satisfactory	Jingdong prefecture level city in Shandong
Fortune Shop	White Intelligent Power Saver	I don't know how well it works.	Jingdong prefecture level city in Shandong
Fortune Shop	White Intelligent Power Saver	Good value for money, recommend it haha	Jingdong prefecture level city in Shandong
Fortune Shop	White Intelligent Power Saver	Good. Good. Very good.	Jingdong prefecture level city in Shandong
Fortune Shop	White Intelligent Power Saver	It's not working.	Jingdong prefecture level city in Shandong
Fortune Shop	White Intelligent Power Saver	It's not working.	Jingdong prefecture level city in Shandong
Fortune Shop	White Intelligent Power Saver	In use	Jingdong prefecture level city in Shandong
Fortune Shop	White Intelligent Power Saver	I'll try it later.	Jingdong prefecture level city in Shandong
Fortune Shop	White Intelligent Power Saver	It's not much use.	Jingdong prefecture level city in Shandong
Fortune Shop	White Intelligent Power Saver	It doesn't save electricity.	Jingdong prefecture level city in Shandong
Fortune Shop	White Intelligent Power Saver	It's not much use.	Jingdong prefecture level city in Shandong
Fortune Shop	White Intelligent Power Saver	Tried it for a few days and tracked the record, and there is no difference from before.	Jingdong prefecture level city in Shandong
Fortune Shop	White Intelligent Power Saver	It's not very credible, so just do it yourself and turn off the lights diligently!	Jingdong prefecture level city in Shandong
Fortune Shop	White Intelligent Power Saver	Too bad, no power saving at all, scam people don't fall for it!	Jingdong prefecture level city in Shandong
Fortune Shop	White Intelligent Power Saver	It's not working at all.	Jingdong prefecture level city in Shandong
Fortune Shop	White Intelligent Power Saver	general	Jingdong prefecture level city in Shandong
Fortune Shop	White Intelligent Power Saver	(of an unmarried couple) be close	Jingdong prefecture level city in Shandong
Fortune Shop	White Intelligent Power Saver	of no use	Jingdong prefecture level city in Shandong
Fortune Shop	White Intelligent Power Saver	Actually can really save electricity, only used half a month to save more than 50, recommended to the neighbors to use together!	Jingdong prefecture level city in Shandong
Fortune Shop	White Intelligent Power Saver	Save what you can. Isn't it more cost effective to buy one of these than to pay the electric bill?	Jingdong prefecture level city in Shandong
Fortune Shop	White Intelligent Power Saver	Received, a use do not know how, use a month to see the effect is good, the effect is good then recommended, not good then I just do not recommend.	Jingdong prefecture level city in Shandong
Fortune Shop	White Intelligent Power Saver	Just received the goods, is a voltage regulator, next month to evaluate whether it is really power saving	Jingdong prefecture level city in Shandong
Fortune Shop	White Intelligent Power Saver	Very good, used a few days obviously feel that the electricity bill is much less, wait a month to see how much can be saved!	Jingdong prefecture level city in Shandong
Fortune Shop	White Intelligent Power Saver	Received the stuff used for more than a month without a little use, it feels like it uses more electricity than before	Jingdong prefecture level city in Shandong
Fortune Shop	White Intelligent Power Saver	The quality is very good, completely exceeded the expectations, the delivery speed is very fast, the packaging is very careful and strict, very satisfied with a shopping	Jingdong prefecture level city in Shandong
Fortune Shop	White Intelligent Power Saver	Fake, doesn't work at all. Don't even sell it.	Jingdong prefecture level city in Shandong
Fortune Shop	White Intelligent Power Saver	This item doesn't seem to have much effect?	Jingdong prefecture level city in Shandong
Fortune Shop	White Intelligent Power Saver	This production is a bit fake, just look at the focus on Ming	Jingdong prefecture level city in Shandong
Fortune Shop	White Intelligent Power Saver	The logistics is fast the service attitude is very good.	Jingdong prefecture level city in Shandong
Fortune Shop	White Intelligent Power Saver	The meter is good quality, sturdy and durable. It is accurate. Easy to use. Saves a lot of things.	Jingdong prefecture level city in Shandong
Fortune Shop	White Intelligent Power Saver	Just received the goods, don't know how it is yet	Jingdong prefecture level city in Shandong
Fortune Shop	White Intelligent Power Saver	Energy saver received, logistics is very fast, the quality is very good, the appearance is also very beautiful power saving is the most important.	Jingdong prefecture level city in Shandong
Fortune Shop	White Intelligent Power Saver	It's good to talk about it, it's not practical to use it.	Jingdong prefecture level city in Shandong
Fortune Shop	White Intelligent Power Saver	I don't know if it's good for now, I'll comment after using it	Jingdong prefecture level city in Shandong
Fortune Shop	White Intelligent Power Saver	I've been using it for two or three days. It's not energy efficient.	Jingdong prefecture level city in Shandong

Fortune Shop	White Intelligent Power Saver	Packaging is intact, logistics is also fast, cost-effective, inexpensive, very satisfied with a purchase	Jingdong prefecture level city in Shandong
Fortune Shop	White Intelligent Power Saver	In use to be observed!	Jingdong prefecture level city in Shandong
Fortune Shop	White Intelligent Power Saver	Use it for a while at a flat rate	Jingdong prefecture level city in Shandong
Fortune Shop	White Intelligent Power Saver	Very trustworthy brand, quality is guaranteed, the price is still affordable, quite satisfied!	Jingdong prefecture level city in Shandong
Fortune Shop	White Intelligent Power Saver	Great quality, great operation, easy to install, great price!	Jingdong prefecture level city in Shandong
Fortune Shop	White Intelligent Power Saver	I came back and installed it, it really saves electricity, much more than before, the	Jingdong prefecture level city in Shandong
Fortune Shop	White Intelligent Power Saver	Fast logistics, good quality, very good packaging, good customer service	Jingdong prefecture level city in Shandong
Fortune Shop	White Intelligent Power Saver	Very good quality, especially good, effective, satisfactory	Jingdong prefecture level city in Shandong
Fortune Shop	White Intelligent Power Saver	I don't know how well it works.	Jingdong prefecture level city in Shandong
Fortune Shop	White Intelligent Power Saver	Good value for money, recommend it haha	Jingdong prefecture level city in Shandong
Fortune Shop	White Intelligent Power Saver	Good. Good. Very good.	Jingdong prefecture level city in Shandong
Fortune Shop	White Intelligent Power Saver	It's not working.	Jingdong prefecture level city in Shandong
Fortune Shop	White Intelligent Power Saver	It's not working.	Jingdong prefecture level city in Shandong
Fortune Shop	White Intelligent Power Saver	In use	Jingdong prefecture level city in Shandong
Fortune Shop	White Intelligent Power Saver	I'll try it later.	Jingdong prefecture level city in Shandong
Fortune Shop	White Intelligent Power Saver	It's not much use.	Jingdong prefecture level city in Shandong
Fortune Shop	White Intelligent Power Saver	It doesn't save electricity.	Jingdong prefecture level city in Shandong
Fortune Shop	White Intelligent Power Saver	It's not much use.	Jingdong prefecture level city in Shandong
Fortune Shop	White Intelligent Power Saver	Tried it for a few days and tracked the record, and there is no difference from before.	Jingdong prefecture level city in Shandong
Fortune Shop	White Intelligent Power Saver	It's not very credible, so just do it yourself and turn off the lights diligently!	Jingdong prefecture level city in Shandong
Fortune Shop	White Intelligent Power Saver	Too bad, no power saving at all, scam people don't fall for it!	Jingdong prefecture level city in Shandong
Fortune Shop	White Intelligent Power Saver	It's not working at all.	Jingdong prefecture level city in Shandong
Fortune Shop	White Intelligent Power Saver	general	Jingdong prefecture level city in Shandong
Fortune Shop	White Intelligent Power Saver	(of an unmarried couple) be close	Jingdong prefecture level city in Shandong
Fortune Shop	White Intelligent Power Saver	of no use	Jingdong prefecture level city in Shandong
Fortune Shop	White Intelligent Power Saver	Actually can really save electricity, only used half a month to save more than 50, recommended to the neighbors to use together!	Jingdong prefecture level city in Shandong
Fortune Shop	White Intelligent Power Saver	Save what you can. Isn't it more cost effective to buy one of these than to pay the electric bill?	Jingdong prefecture level city in Shandong
Fortune Shop	White Intelligent Power Saver	Received, a use do not know how, use a month to see the effect is good, the effect is good then recommended, not good then I just do not recommend.	Jingdong prefecture level city in Shandong
Fortune Shop	White Intelligent Power Saver	Just received the goods, is a voltage regulator, next month to evaluate whether it is really power saving	Jingdong prefecture level city in Shandong
Fortune Shop	White Intelligent Power Saver	Very good, used a few days obviously feel that the electricity bill is much less, wait a month to see how much can be saved!	Jingdong prefecture level city in Shandong
Fortune Shop	White Intelligent Power Saver	Received the stuff used for more than a month without a little use, it feels like it uses more electricity than before	Jingdong prefecture level city in Shandong
Fortune Shop	White Intelligent Power Saver	The quality is very good, completely exceeded the expectations, the delivery speed is very fast, the packaging is very careful and strict, very satisfied with a shopping	Jingdong prefecture level city in Shandong
Fortune Shop	White Intelligent Power Saver	Fake, doesn't work at all. Don't even sell it.	Jingdong prefecture level city in Shandong
Fortune Shop	White Intelligent Power Saver	This item doesn't seem to have much effect?	Jingdong prefecture level city in Shandong
Fortune Shop	White Intelligent Power Saver	This production is a bit fake, just look at the focus on Ming	Jingdong prefecture level city in Shandong
Fortune Shop	White Intelligent Power Saver	The logistics is fast the service attitude is very good.	Jingdong prefecture level city in Shandong
Fortune Shop	White Intelligent Power Saver	The meter is good quality, sturdy and durable. It is accurate. Easy to use. Saves a lot of things.	Jingdong prefecture level city in Shandong
Fortune Shop	White Intelligent Power Saver	Just received the goods, don't know how it is yet	Jingdong prefecture level city in Shandong
Fortune Shop	White Intelligent Power Saver	Energy saver received, logistics is very fast, the quality is very good, the appearance is also very beautiful power saving is the most important.	Jingdong prefecture level city in Shandong
Fortune Shop	White Intelligent Power Saver	It's good to talk about it, it's not practical to use it.	Jingdong prefecture level city in Shandong
Fortune Shop	White Intelligent Power Saver	I don't know if it's good for now, I'll comment after using it	Jingdong prefecture level city in Shandong
Fortune Shop	White Intelligent Power Saver	I've been using it for two or three days. It's not energy efficient.	Jingdong prefecture level city in Shandong
Fortune Shop	White Intelligent Power Saver	Packaging is intact, logistics is also fast, cost-effective, inexpensive, very satisfied with a purchase	Jingdong prefecture level city in Shandong
Fortune Shop	White Intelligent Power Saver	In use to be observed!	Jingdong prefecture level city in Shandong
Fortune Shop	White Intelligent Power Saver	Use it for a while at a flat rate	Jingdong prefecture level city in Shandong
Fortune Shop	White Intelligent Power Saver	Very trustworthy brand, quality is guaranteed, the price is still affordable, quite satisfied!	Jingdong prefecture level city in Shandong
Fortune Shop	White Intelligent Power Saver	Great quality, great operation, easy to install, great price!	Jingdong prefecture level city in Shandong
Fortune Shop	White Intelligent Power Saver	I came back and installed it, it really saves electricity, much more than before, the	Jingdong prefecture level city in Shandong
Fortune Shop	White Intelligent Power Saver	Fast logistics, good quality, very good packaging, good customer service	Jingdong prefecture level city in Shandong
Fortune Shop	White Intelligent Power Saver	Very good quality, especially good, effective, satisfactory	Jingdong prefecture level city in Shandong
Fortune Shop	White Intelligent Power Saver	I don't know how well it works.	Jingdong prefecture level city in Shandong

Fortune Shop	White Intelligent Power Saver	Good value for money, recommend it haha	Jingdong prefecture level city in Shandong
Fortune Shop	White Intelligent Power Saver	Good. Good. Very good.	Jingdong prefecture level city in Shandong
Fortune Shop	White Intelligent Power Saver	It's not working.	Jingdong prefecture level city in Shandong
Fortune Shop	White Intelligent Power Saver	It's not working.	Jingdong prefecture level city in Shandong
Fortune Shop	White Intelligent Power Saver	In use	Jingdong prefecture level city in Shandong
Fortune Shop	White Intelligent Power Saver	I'll try it later.	Jingdong prefecture level city in Shandong
Fortune Shop	White Intelligent Power Saver	It's not much use.	Jingdong prefecture level city in Shandong
Fortune Shop	White Intelligent Power Saver	It doesn't save electricity.	Jingdong prefecture level city in Shandong
Fortune Shop	White Intelligent Power Saver	It's not much use.	Jingdong prefecture level city in Shandong
Fortune Shop	White Intelligent Power Saver	Tried it for a few days and tracked the record, and there is no difference from before.	Jingdong prefecture level city in Shandong
Fortune Shop	White Intelligent Power Saver	It's not very credible, so just do it yourself and turn off the lights diligently!	Jingdong prefecture level city in Shandong
Fortune Shop	White Intelligent Power Saver	Too bad, no power saving at all, scam people don't fall for it!	Jingdong prefecture level city in Shandong
Fortune Shop	White Intelligent Power Saver	It's not working at all.	Jingdong prefecture level city in Shandong
Fortune Shop	White Intelligent Power Saver	general	Jingdong prefecture level city in Shandong
Fortune Shop	White Intelligent Power Saver	(of an unmarried couple) be close	Jingdong prefecture level city in Shandong
Fortune Shop	White Intelligent Power Saver	of no use	Jingdong prefecture level city in Shandong
Fortune Shop	White Intelligent Power Saver	Actually can really save electricity, only used half a month to save more than 50, recommended to the neighbors to use together!	Jingdong prefecture level city in Shandong
Fortune Shop	White Intelligent Power Saver	Save what you can. Isn't it more cost effective to buy one of these than to pay the electric bill?	Jingdong prefecture level city in Shandong
Fortune Shop	White Intelligent Power Saver	Received, a use do not know how, use a month to see the effect is good, the effect is good then recommended, not good then I just do not recommend.	Jingdong prefecture level city in Shandong
Fortune Shop	White Intelligent Power Saver	Just received the goods, is a voltage regulator, next month to evaluate whether it is really power saving	Jingdong prefecture level city in Shandong
Fortune Shop	White Intelligent Power Saver	Very good, used a few days obviously feel that the electricity bill is much less, wait a month to see how much can be saved!	Jingdong prefecture level city in Shandong
Fortune Shop	White Intelligent Power Saver	Received the stuff used for more than a month without a little use, it feels like it uses more electricity than before	Jingdong prefecture level city in Shandong
Fortune Shop	White Intelligent Power Saver	The quality is very good, completely exceeded the expectations, the delivery speed is very fast, the packaging is very careful and strict, very satisfied with a shopping	Jingdong prefecture level city in Shandong
Fortune Shop	White Intelligent Power Saver	Fake, doesn't work at all. Don't even sell it.	Jingdong prefecture level city in Shandong
Fortune Shop	White Intelligent Power Saver	This item doesn't seem to have much effect?	Jingdong prefecture level city in Shandong
Fortune Shop	White Intelligent Power Saver	This production is a bit fake, just look at the focus on Ming	Jingdong prefecture level city in Shandong
Fortune Shop	White Intelligent Power Saver	The logistics is fast the service attitude is very good.	Jingdong prefecture level city in Shandong
Fortune Shop	White Intelligent Power Saver	The meter is good quality, sturdy and durable. It is accurate. Easy to use. Saves a lot of things.	Jingdong prefecture level city in Shandong
Fortune Shop	White Intelligent Power Saver	Just received the goods, don't know how it is yet	Jingdong prefecture level city in Shandong
Fortune Shop	White Intelligent Power Saver	Energy saver received, logistics is very fast, the quality is very good, the appearance is also very beautiful power saving is the most important.	Jingdong prefecture level city in Shandong
Fortune Shop	White Intelligent Power Saver	It's good to talk about it, it's not practical to use it.	Jingdong prefecture level city in Shandong
Fortune Shop	White Intelligent Power Saver	I don't know if it's good for now, I'll comment after using it	Jingdong prefecture level city in Shandong
Fortune Shop	White Intelligent Power Saver	I've been using it for two or three days. It's not energy efficient.	Jingdong prefecture level city in Shandong
Fortune Shop	White Intelligent Power Saver	Packaging is intact, logistics is also fast, cost-effective, inexpensive, very satisfied with a purchase	Jingdong prefecture level city in Shandong
Fortune Shop	White Intelligent Power Saver	In use to be observed!	Jingdong prefecture level city in Shandong
Fortune Shop	White Intelligent Power Saver	Use it for a while at a flat rate	Jingdong prefecture level city in Shandong
Fortune Shop	White Intelligent Power Saver	Very trustworthy brand, quality is guaranteed, the price is still affordable, quite satisfied!	Jingdong prefecture level city in Shandong
Fortune Shop	White Intelligent Power Saver	Great quality, great operation, easy to install, great price!	Jingdong prefecture level city in Shandong
Fortune Shop	White Intelligent Power Saver	I came back and installed it, it really saves electricity, much more than before, the	Jingdong prefecture level city in Shandong
Fortune Shop	White Intelligent Power Saver	Fast logistics, good quality, very good packaging, good customer service	Jingdong prefecture level city in Shandong
Fortune Shop	White Intelligent Power Saver	Very good quality, especially good, effective, satisfactory	Jingdong prefecture level city in Shandong
Fortune Shop	White Intelligent Power Saver	I don't know how well it works.	Jingdong prefecture level city in Shandong
Fortune Shop	White Intelligent Power Saver	Good value for money, recommend it haha	Jingdong prefecture level city in Shandong
Fortune Shop	White Intelligent Power Saver	Good. Good. Very good.	Jingdong prefecture level city in Shandong
Fortune Shop	White Intelligent Power Saver	It's not working.	Jingdong prefecture level city in Shandong
Fortune Shop	White Intelligent Power Saver	It's not working.	Jingdong prefecture level city in Shandong
Fortune Shop	White Intelligent Power Saver	In use	Jingdong prefecture level city in Shandong
Fortune Shop	White Intelligent Power Saver	I'll try it later.	Jingdong prefecture level city in Shandong
Fortune Shop	White Intelligent Power Saver	It's not much use.	Jingdong prefecture level city in Shandong
Fortune Shop	White Intelligent Power Saver	It doesn't save electricity.	Jingdong prefecture level city in Shandong
Fortune Shop	White Intelligent Power Saver	It's not much use.	Jingdong prefecture level city in Shandong

Fortune Shop	White Intelligent Power Saver	Tried it for a few days and tracked the record, and there is no difference from before.	Jingdong prefecture level city in Shandong
Fortune Shop	White Intelligent Power Saver	It's not very credible, so just do it yourself and turn off the lights diligently!	Jingdong prefecture level city in Shandong
Fortune Shop	White Intelligent Power Saver	Too bad, no power saving at all, scam people don't fall for it!	Jingdong prefecture level city in Shandong
Fortune Shop	White Intelligent Power Saver	It's not working at all.	Jingdong prefecture level city in Shandong
Fortune Shop	White Intelligent Power Saver	general	Jingdong prefecture level city in Shandong
Fortune Shop	White Intelligent Power Saver	(of an unmarried couple) be close	Jingdong prefecture level city in Shandong
Fortune Shop	White Intelligent Power Saver	of no use	Jingdong prefecture level city in Shandong
Fortune Shop	White Intelligent Power Saver	Actually can really save electricity, only used half a month to save more than 50, recommended to the neighbors to use together!	Jingdong prefecture level city in Shandong
Fortune Shop	White Intelligent Power Saver	Save what you can. Isn't it more cost effective to buy one of these than to pay the electric bill?	Jingdong prefecture level city in Shandong
Fortune Shop	White Intelligent Power Saver	Received, a use do not know how, use a month to see the effect is good, the effect is good then recommended, not good then I just do not recommend.	Jingdong prefecture level city in Shandong
Fortune Shop	White Intelligent Power Saver	Just received the goods, is a voltage regulator, next month to evaluate whether it is really power saving	Jingdong prefecture level city in Shandong
Fortune Shop	White Intelligent Power Saver	Very good, used a few days obviously feel that the electricity bill is much less, wait a month to see how much can be saved!	Jingdong prefecture level city in Shandong
Fortune Shop	White Intelligent Power Saver	Received the stuff used for more than a month without a little use, it feels like it uses more electricity than before	Jingdong prefecture level city in Shandong
Fortune Shop	White Intelligent Power Saver	The quality is very good, completely exceeded the expectations, the delivery speed is very fast, the packaging is very careful and strict, very satisfied with a shopping	Jingdong prefecture level city in Shandong
Fortune Shop	White Intelligent Power Saver	Fake, doesn't work at all. Don't even sell it.	Jingdong prefecture level city in Shandong
Fortune Shop	White Intelligent Power Saver	This item doesn't seem to have much effect?	Jingdong prefecture level city in Shandong
Fortune Shop	White Intelligent Power Saver	This production is a bit fake, just look at the focus on Ming	Jingdong prefecture level city in Shandong
Fortune Shop	White Intelligent Power Saver	The logistics is fast the service attitude is very good.	Jingdong prefecture level city in Shandong
Fortune Shop	White Intelligent Power Saver	The meter is good quality, sturdy and durable. It is accurate. Easy to use. Saves a lot of things.	Jingdong prefecture level city in Shandong
Fortune Shop	White Intelligent Power Saver	Just received the goods, don't know how it is yet	Jingdong prefecture level city in Shandong
Fortune Shop	White Intelligent Power Saver	Energy saver received, logistics is very fast, the quality is very good, the appearance is also very beautiful power saving is the most important.	Jingdong prefecture level city in Shandong
Fortune Shop	White Intelligent Power Saver	It's good to talk about it, it's not practical to use it.	Jingdong prefecture level city in Shandong
Fortune Shop	White Intelligent Power Saver	I don't know if it's good for now, I'll comment after using it	Jingdong prefecture level city in Shandong
Fortune Shop	White Intelligent Power Saver	I've been using it for two or three days. It's not energy efficient.	Jingdong prefecture level city in Shandong
Fortune Shop	White Intelligent Power Saver	Packaging is intact, logistics is also fast, cost-effective, inexpensive, very satisfied with a purchase	Jingdong prefecture level city in Shandong
Fortune Shop	White Intelligent Power Saver	In use to be observed!	Jingdong prefecture level city in Shandong
Fortune Shop	White Intelligent Power Saver	Use it for a while at a flat rate	Jingdong prefecture level city in Shandong
Fortune Shop	White Intelligent Power Saver	Very trustworthy brand, quality is guaranteed, the price is still affordable, quite satisfied!	Jingdong prefecture level city in Shandong
Fortune Shop	White Intelligent Power Saver	Great quality, great operation, easy to install, great price!	Jingdong prefecture level city in Shandong
Fortune Shop	White Intelligent Power Saver	I came back and installed it, it really saves electricity, much more than before, the	Jingdong prefecture level city in Shandong
Fortune Shop	White Intelligent Power Saver	Fast logistics, good quality, very good packaging, good customer service	Jingdong prefecture level city in Shandong
Fortune Shop	White Intelligent Power Saver	Very good quality, especially good, effective, satisfactory	Jingdong prefecture level city in Shandong
Fortune Shop	White Intelligent Power Saver	I don't know how well it works.	Jingdong prefecture level city in Shandong
Fortune Shop	White Intelligent Power Saver	Good value for money, recommend it haha	Jingdong prefecture level city in Shandong
Fortune Shop	White Intelligent Power Saver	Good. Good. Very good.	Jingdong prefecture level city in Shandong
Fortune Shop	White Intelligent Power Saver	It's not working.	Jingdong prefecture level city in Shandong
Fortune Shop	White Intelligent Power Saver	It's not working.	Jingdong prefecture level city in Shandong
Fortune Shop	White Intelligent Power Saver	In use	Jingdong prefecture level city in Shandong
Fortune Shop	White Intelligent Power Saver	I'll try it later.	Jingdong prefecture level city in Shandong
Fortune Shop	White Intelligent Power Saver	It's not much use.	Jingdong prefecture level city in Shandong
Fortune Shop	White Intelligent Power Saver	It doesn't save electricity.	Jingdong prefecture level city in Shandong
Fortune Shop	White Intelligent Power Saver	It's not much use.	Jingdong prefecture level city in Shandong
Fortune Shop	White Intelligent Power Saver	Tried it for a few days and tracked the record, and there is no difference from before.	Jingdong prefecture level city in Shandong
Fortune Shop	White Intelligent Power Saver	It's not very credible, so just do it yourself and turn off the lights diligently!	Jingdong prefecture level city in Shandong
Fortune Shop	White Intelligent Power Saver	Too bad, no power saving at all, scam people don't fall for it!	Jingdong prefecture level city in Shandong
Fortune Shop	White Intelligent Power Saver	It's not working at all.	Jingdong prefecture level city in Shandong
Fortune Shop	White Intelligent Power Saver	general	Jingdong prefecture level city in Shandong
Fortune Shop	White Intelligent Power Saver	(of an unmarried couple) be close	Jingdong prefecture level city in Shandong
Fortune Shop	White Intelligent Power Saver	of no use	Jingdong prefecture level city in Shandong
Fortune Shop	White Intelligent Power Saver	Actually can really save electricity, only used half a month to save more than 50, recommended to the neighbors to use together!	Jingdong prefecture level city in Shandong
Fortune Shop	White Intelligent Power Saver	Save what you can. Isn't it more cost effective to buy one of these than to pay the electric bill?	Jingdong prefecture level city in Shandong

Fortune Shop	White Intelligent Power Saver	Received, a use do not know how, use a month to see the effect is good, the effect is good then recommended, not good then I just do not recommend.	Jingdong prefecture level city in Shandong
Fortune Shop	White Intelligent Power Saver	Just received the goods, is a voltage regulator, next month to evaluate whether it is really power saving	Jingdong prefecture level city in Shandong
Fortune Shop	White Intelligent Power Saver	Very good, used a few days obviously feel that the electricity bill is much less, wait a month to see how much can be saved!	Jingdong prefecture level city in Shandong
Fortune Shop	White Intelligent Power Saver	Received the stuff used for more than a month without a little use, it feels like it uses more electricity than before	Jingdong prefecture level city in Shandong
Fortune Shop	White Intelligent Power Saver	The quality is very good, completely exceeded the expectations, the delivery speed is very fast, the packaging is very careful and strict, very satisfied with a shopping	Jingdong prefecture level city in Shandong
Fortune Shop	White Intelligent Power Saver	Fake, doesn't work at all. Don't even sell it.	Jingdong prefecture level city in Shandong
Fortune Shop	White Intelligent Power Saver	This item doesn't seem to have much effect?	Jingdong prefecture level city in Shandong
Fortune Shop	White Intelligent Power Saver	This production is a bit fake, just look at the focus on Ming	Jingdong prefecture level city in Shandong
Fortune Shop	White Intelligent Power Saver	The logistics is fast the service attitude is very good.	Jingdong prefecture level city in Shandong
Fortune Shop	White Intelligent Power Saver	The meter is good quality, sturdy and durable. It is accurate. Easy to use. Saves a lot of things.	Jingdong prefecture level city in Shandong
Fortune Shop	White Intelligent Power Saver	Just received the goods, don't know how it is yet	Jingdong prefecture level city in Shandong
Fortune Shop	White Intelligent Power Saver	Energy saver received, logistics is very fast, the quality is very good, the appearance is also very beautiful power saving is the most important.	Jingdong prefecture level city in Shandong
Fortune Shop	White Intelligent Power Saver	It's good to talk about it, it's not practical to use it.	Jingdong prefecture level city in Shandong
Fortune Shop	White Intelligent Power Saver	I don't know if it's good for now, I'll comment after using it	Jingdong prefecture level city in Shandong
Fortune Shop	White Intelligent Power Saver	I've been using it for two or three days. It's not energy efficient.	Jingdong prefecture level city in Shandong
Fortune Shop	White Intelligent Power Saver	Packaging is intact, logistics is also fast, cost-effective, inexpensive, very satisfied with a purchase	Jingdong prefecture level city in Shandong
Fortune Shop	White Intelligent Power Saver	In use to be observed!	Jingdong prefecture level city in Shandong
Fortune Shop	White Intelligent Power Saver	Use it for a while at a flat rate	Jingdong prefecture level city in Shandong
Fortune Shop	White Intelligent Power Saver	Very trustworthy brand, quality is guaranteed, the price is still affordable, quite satisfied!	Jingdong prefecture level city in Shandong
Fortune Shop	White Intelligent Power Saver	Great quality, great operation, easy to install, great price!	Jingdong prefecture level city in Shandong
Fortune Shop	White Intelligent Power Saver	I came back and installed it, it really saves electricity, much more than before, the	Jingdong prefecture level city in Shandong
Fortune Shop	White Intelligent Power Saver	Fast logistics, good quality, very good packaging, good customer service	Jingdong prefecture level city in Shandong
Fortune Shop	White Intelligent Power Saver	Very good quality, especially good, effective, satisfactory	Jingdong prefecture level city in Shandong
Fortune Shop	White Intelligent Power Saver	I don't know how well it works.	Jingdong prefecture level city in Shandong
Fortune Shop	White Intelligent Power Saver	Good value for money, recommend it haha	Jingdong prefecture level city in Shandong
Fortune Shop	White Intelligent Power Saver	Good. Good. Very good.	Jingdong prefecture level city in Shandong
Fortune Shop	White Intelligent Power Saver	It's not working.	Jingdong prefecture level city in Shandong
Fortune Shop	White Intelligent Power Saver	It's not working.	Jingdong prefecture level city in Shandong
Fortune Shop	White Intelligent Power Saver	In use	Jingdong prefecture level city in Shandong
Fortune Shop	White Intelligent Power Saver	I'll try it later.	Jingdong prefecture level city in Shandong
Fortune Shop	White Intelligent Power Saver	It's not much use.	Jingdong prefecture level city in Shandong
Fortune Shop	White Intelligent Power Saver	It doesn't save electricity.	Jingdong prefecture level city in Shandong
Fortune Shop	White Intelligent Power Saver	It's not much use.	Jingdong prefecture level city in Shandong
Fortune Shop	White Intelligent Power Saver	Tried it for a few days and tracked the record, and there is no difference from before.	Jingdong prefecture level city in Shandong
Fortune Shop	White Intelligent Power Saver	It's not very credible, so just do it yourself and turn off the lights diligently!	Jingdong prefecture level city in Shandong
Fortune Shop	White Intelligent Power Saver	Too bad, no power saving at all, scam people don't fall for it!	Jingdong prefecture level city in Shandong
Fortune Shop	White Intelligent Power Saver	It's not working at all.	Jingdong prefecture level city in Shandong
Fortune Shop	White Intelligent Power Saver	general	Jingdong prefecture level city in Shandong
Fortune Shop	White Intelligent Power Saver	(of an unmarried couple) be close	Jingdong prefecture level city in Shandong
Fortune Shop	White Intelligent Power Saver	of no use	Jingdong prefecture level city in Shandong
Fortune Shop	White Intelligent Power Saver	Actually can really save electricity, only used half a month to save more than 50, recommended to the neighbors to use together!	Jingdong prefecture level city in Shandong
Fortune Shop	White Intelligent Power Saver	Save what you can. Isn't it more cost effective to buy one of these than to pay the electric bill?	Jingdong prefecture level city in Shandong
Fortune Shop	White Intelligent Power Saver	Received, a use do not know how, use a month to see the effect is good, the effect is good then recommended, not good then I just do not recommend.	Jingdong prefecture level city in Shandong
Fortune Shop	White Intelligent Power Saver	Just received the goods, is a voltage regulator, next month to evaluate whether it is really power saving	Jingdong prefecture level city in Shandong
Fortune Shop	White Intelligent Power Saver	Very good, used a few days obviously feel that the electricity bill is much less, wait a month to see how much can be saved!	Jingdong prefecture level city in Shandong
Fortune Shop	White Intelligent Power Saver	Received the stuff used for more than a month without a little use, it feels like it uses more electricity than before	Jingdong prefecture level city in Shandong
Fortune Shop	White Intelligent Power Saver	The quality is very good, completely exceeded the expectations, the delivery speed is very fast, the packaging is very careful and strict, very satisfied with a shopping	Jingdong prefecture level city in Shandong
Fortune Shop	White Intelligent Power Saver	Fake, doesn't work at all. Don't even sell it.	Jingdong prefecture level city in Shandong
Fortune Shop	White Intelligent Power Saver	This item doesn't seem to have much effect?	Jingdong prefecture level city in Shandong
Fortune Shop	White Intelligent Power Saver	This production is a bit fake, just look at the focus on Ming	Jingdong prefecture level city in Shandong
Fortune Shop	White Intelligent Power Saver	The logistics is fast the service attitude is very good.	Jingdong prefecture level city in Shandong

Fortune Shop	White Intelligent Power Saver	The meter is good quality, sturdy and durable. It is accurate. Easy to use. Saves a lot of things.	Jingdong prefecture level city in Shandong
Fortune Shop	White Intelligent Power Saver	Just received the goods, don't know how it is yet	Jingdong prefecture level city in Shandong
Fortune Shop	White Intelligent Power Saver	Energy saver received, logistics is very fast, the quality is very good, the appearance is also very beautiful power saving is the most important.	Jingdong prefecture level city in Shandong
Fortune Shop	White Intelligent Power Saver	It's good to talk about it, it's not practical to use it.	Jingdong prefecture level city in Shandong
Fortune Shop	White Intelligent Power Saver	I don't know if it's good for now, I'll comment after using it	Jingdong prefecture level city in Shandong
Fortune Shop	White Intelligent Power Saver	I've been using it for two or three days. It's not energy efficient.	Jingdong prefecture level city in Shandong
Fortune Shop	White Intelligent Power Saver	Packaging is intact, logistics is also fast, cost-effective, inexpensive, very satisfied with a purchase	Jingdong prefecture level city in Shandong
Fortune Shop	White Intelligent Power Saver	In use to be observed!	Jingdong prefecture level city in Shandong
Fortune Shop	White Intelligent Power Saver	Use it for a while at a flat rate	Jingdong prefecture level city in Shandong
Fortune Shop	White Intelligent Power Saver	Very trustworthy brand, quality is guaranteed, the price is still affordable, quite satisfied!	Jingdong prefecture level city in Shandong
Fortune Shop	White Intelligent Power Saver	Great quality, great operation, easy to install, great price!	Jingdong prefecture level city in Shandong
Fortune Shop	White Intelligent Power Saver	I came back and installed it, it really saves electricity, much more than before, the	Jingdong prefecture level city in Shandong
Fortune Shop	White Intelligent Power Saver	Fast logistics, good quality, very good packaging, good customer service	Jingdong prefecture level city in Shandong
Fortune Shop	White Intelligent Power Saver	Very good quality, especially good, effective, satisfactory	Jingdong prefecture level city in Shandong
Fortune Shop	White Intelligent Power Saver	I don't know how well it works.	Jingdong prefecture level city in Shandong
Fortune Shop	White Intelligent Power Saver	Good value for money, recommend it haha	Jingdong prefecture level city in Shandong
Fortune Shop	White Intelligent Power Saver	Good. Good. Very good.	Jingdong prefecture level city in Shandong
Fortune Shop	White Intelligent Power Saver	It's not working.	Jingdong prefecture level city in Shandong
Fortune Shop	White Intelligent Power Saver	It's not working.	Jingdong prefecture level city in Shandong
Fortune Shop	White Intelligent Power Saver	In use	Jingdong prefecture level city in Shandong
Fortune Shop	White Intelligent Power Saver	I'll try it later.	Jingdong prefecture level city in Shandong
Fortune Shop	White Intelligent Power Saver	It's not much use.	Jingdong prefecture level city in Shandong
Fortune Shop	White Intelligent Power Saver	It doesn't save electricity.	Jingdong prefecture level city in Shandong
Fortune Shop	White Intelligent Power Saver	It's not much use.	Jingdong prefecture level city in Shandong
Fortune Shop	White Intelligent Power Saver	Tried it for a few days and tracked the record, and there is no difference from before.	Jingdong prefecture level city in Shandong
Fortune Shop	White Intelligent Power Saver	It's not very credible, so just do it yourself and turn off the lights diligently!	Jingdong prefecture level city in Shandong
Fortune Shop	White Intelligent Power Saver	Too bad, no power saving at all, scam people don't fall for it!	Jingdong prefecture level city in Shandong
Fortune Shop	White Intelligent Power Saver	It's not working at all.	Jingdong prefecture level city in Shandong
Fortune Shop	White Intelligent Power Saver	general	Jingdong prefecture level city in Shandong
Fortune Shop	White Intelligent Power Saver	(of an unmarried couple) be close	Jingdong prefecture level city in Shandong
Fortune Shop	White Intelligent Power Saver	of no use	Jingdong prefecture level city in Shandong
Fortune Shop	White Intelligent Power Saver	Actually can really save electricity, only used half a month to save more than 50, recommended to the neighbors to use together!	Jingdong prefecture level city in Shandong
Fortune Shop	White Intelligent Power Saver	Save what you can. Isn't it more cost effective to buy one of these than to pay the electric bill?	Jingdong prefecture level city in Shandong
Fortune Shop	White Intelligent Power Saver	Received, a use do not know how, use a month to see the effect is good, the effect is good then recommended, not good then I just do not recommend.	Jingdong prefecture level city in Shandong
Fortune Shop	White Intelligent Power Saver	Just received the goods, is a voltage regulator, next month to evaluate whether it is really power saving	Jingdong prefecture level city in Shandong
Fortune Shop	White Intelligent Power Saver	Very good, used a few days obviously feel that the electricity bill is much less, wait a month to see how much can be saved!	Jingdong prefecture level city in Shandong
Fortune Shop	White Intelligent Power Saver	Received the stuff used for more than a month without a little use, it feels like it uses more electricity than before	Jingdong prefecture level city in Shandong
Fortune Shop	White Intelligent Power Saver	The quality is very good, completely exceeded the expectations, the delivery speed is very fast, the packaging is very careful and strict, very satisfied with a shopping	Jingdong prefecture level city in Shandong
Fortune Shop	White Intelligent Power Saver	Fake, doesn't work at all. Don't even sell it.	Jingdong prefecture level city in Shandong
Fortune Shop	White Intelligent Power Saver	This item doesn't seem to have much effect?	Jingdong prefecture level city in Shandong
Fortune Shop	White Intelligent Power Saver	This production is a bit fake, just look at the focus on Ming	Jingdong prefecture level city in Shandong
Fortune Shop	White Intelligent Power Saver	The logistics is fast the service attitude is very good.	Jingdong prefecture level city in Shandong
Fortune Shop	White Intelligent Power Saver	The meter is good quality, sturdy and durable. It is accurate. Easy to use. Saves a lot of things.	Jingdong prefecture level city in Shandong
Fortune Shop	White Intelligent Power Saver	Just received the goods, don't know how it is yet	Jingdong prefecture level city in Shandong
Fortune Shop	White Intelligent Power Saver	Energy saver received, logistics is very fast, the quality is very good, the appearance is also very beautiful power saving is the most important.	Jingdong prefecture level city in Shandong
Fortune Shop	White Intelligent Power Saver	It's good to talk about it, it's not practical to use it.	Jingdong prefecture level city in Shandong
Fortune Shop	White Intelligent Power Saver	I don't know if it's good for now, I'll comment after using it	Jingdong prefecture level city in Shandong
Fortune Shop	White Intelligent Power Saver	I've been using it for two or three days. It's not energy efficient.	Jingdong prefecture level city in Shandong
Fortune Shop	White Intelligent Power Saver	Packaging is intact, logistics is also fast, cost-effective, inexpensive, very satisfied with a purchase	Jingdong prefecture level city in Shandong
Fortune Shop	White Intelligent Power Saver	In use to be observed!	Jingdong prefecture level city in Shandong
Fortune Shop	White Intelligent Power Saver	Use it for a while at a flat rate	Jingdong prefecture level city in Shandong

Fortune Shop	White Intelligent Power Saver	Very trustworthy brand, quality is guaranteed, the price is still affordable, quite satisfied!	Jingdong prefecture level city in Shandong
Fortune Shop	White Intelligent Power Saver	Great quality, great operation, easy to install, great price!	Jingdong prefecture level city in Shandong
Fortune Shop	White Intelligent Power Saver	I came back and installed it, it really saves electricity, much more than before, the	Jingdong prefecture level city in Shandong
Fortune Shop	White Intelligent Power Saver	Fast logistics, good quality, very good packaging, good customer service	Jingdong prefecture level city in Shandong
Fortune Shop	White Intelligent Power Saver	Very good quality, especially good, effective, satisfactory	Jingdong prefecture level city in Shandong
Fortune Shop	White Intelligent Power Saver	I don't know how well it works.	Jingdong prefecture level city in Shandong
Fortune Shop	White Intelligent Power Saver	Good value for money, recommend it haha	Jingdong prefecture level city in Shandong
Fortune Shop	White Intelligent Power Saver	Good. Good. Very good.	Jingdong prefecture level city in Shandong
Fortune Shop	White Intelligent Power Saver	It's not working.	Jingdong prefecture level city in Shandong
Fortune Shop	White Intelligent Power Saver	It's not working.	Jingdong prefecture level city in Shandong
Fortune Shop	White Intelligent Power Saver	In use	Jingdong prefecture level city in Shandong
Fortune Shop	White Intelligent Power Saver	I'll try it later.	Jingdong prefecture level city in Shandong
Fortune Shop	White Intelligent Power Saver	It's not much use.	Jingdong prefecture level city in Shandong
Fortune Shop	White Intelligent Power Saver	It doesn't save electricity.	Jingdong prefecture level city in Shandong
Fortune Shop	White Intelligent Power Saver	It's not much use.	Jingdong prefecture level city in Shandong
Fortune Shop	White Intelligent Power Saver	Tried it for a few days and tracked the record, and there is no difference from before.	Jingdong prefecture level city in Shandong
Fortune Shop	White Intelligent Power Saver	It's not very credible, so just do it yourself and turn off the lights diligently!	Jingdong prefecture level city in Shandong
Fortune Shop	White Intelligent Power Saver	Too bad, no power saving at all, scam people don't fall for it!	Jingdong prefecture level city in Shandong
Fortune Shop	White Intelligent Power Saver	It's not working at all.	Jingdong prefecture level city in Shandong
Fortune Shop	White Intelligent Power Saver	general	Jingdong prefecture level city in Shandong
Fortune Shop	White Intelligent Power Saver	(of an unmarried couple) be close	Jingdong prefecture level city in Shandong
Fortune Shop	White Intelligent Power Saver	of no use	Jingdong prefecture level city in Shandong
Fortune Shop	White Intelligent Power Saver	Actually can really save electricity, only used half a month to save more than 50, recommended to the neighbors to use together!	Jingdong prefecture level city in Shandong
Fortune Shop	White Intelligent Power Saver	Save what you can. Isn't it more cost effective to buy one of these than to pay the electric bill?	Jingdong prefecture level city in Shandong
Fortune Shop	White Intelligent Power Saver	Received, a use do not know how, use a month to see the effect is good, the effect is good then recommended, not good then I just do not recommend.	Jingdong prefecture level city in Shandong
Fortune Shop	White Intelligent Power Saver	Just received the goods, is a voltage regulator, next month to evaluate whether it is really power saving	Jingdong prefecture level city in Shandong
Fortune Shop	White Intelligent Power Saver	Very good, used a few days obviously feel that the electricity bill is much less, wait a month to see how much can be saved!	Jingdong prefecture level city in Shandong
Fortune Shop	White Intelligent Power Saver	Received the stuff used for more than a month without a little use, it feels like it uses more electricity than before	Jingdong prefecture level city in Shandong
Fortune Shop	White Intelligent Power Saver	The quality is very good, completely exceeded the expectations, the delivery speed is very fast, the packaging is very careful and strict, very satisfied with a shopping	Jingdong prefecture level city in Shandong
Fortune Shop	White Intelligent Power Saver	Fake, doesn't work at all. Don't even sell it.	Jingdong prefecture level city in Shandong
Fortune Shop	White Intelligent Power Saver	This item doesn't seem to have much effect?	Jingdong prefecture level city in Shandong
Fortune Shop	White Intelligent Power Saver	This production is a bit fake, just look at the focus on Ming	Jingdong prefecture level city in Shandong
Fortune Shop	White Intelligent Power Saver	The logistics is fast the service attitude is very good.	Jingdong prefecture level city in Shandong
Fortune Shop	White Intelligent Power Saver	The meter is good quality, sturdy and durable. It is accurate. Easy to use. Saves a lot of things.	Jingdong prefecture level city in Shandong
Fortune Shop	White Intelligent Power Saver	Just received the goods, don't know how it is yet	Jingdong prefecture level city in Shandong
Fortune Shop	White Intelligent Power Saver	Energy saver received, logistics is very fast, the quality is very good, the appearance is also very beautiful power saving is the most important.	Jingdong prefecture level city in Shandong
Fortune Shop	White Intelligent Power Saver	It's good to talk about it, it's not practical to use it.	Jingdong prefecture level city in Shandong
Fortune Shop	White Intelligent Power Saver	I don't know if it's good for now, I'll comment after using it	Jingdong prefecture level city in Shandong
Fortune Shop	White Intelligent Power Saver	I've been using it for two or three days. It's not energy efficient.	Jingdong prefecture level city in Shandong
Fortune Shop	White Intelligent Power Saver	Packaging is intact, logistics is also fast, cost-effective, inexpensive, very satisfied with a purchase	Jingdong prefecture level city in Shandong
Fortune Shop	White Intelligent Power Saver	In use to be observed!	Jingdong prefecture level city in Shandong
Fortune Shop	White Intelligent Power Saver	Use it for a while at a flat rate	Jingdong prefecture level city in Shandong
Fortune Shop	White Intelligent Power Saver	Very trustworthy brand, quality is guaranteed, the price is still affordable, quite satisfied!	Jingdong prefecture level city in Shandong
Fortune Shop	White Intelligent Power Saver	Great quality, great operation, easy to install, great price!	Jingdong prefecture level city in Shandong
Fortune Shop	White Intelligent Power Saver	I came back and installed it, it really saves electricity, much more than before, the	Jingdong prefecture level city in Shandong
Fortune Shop	White Intelligent Power Saver	Fast logistics, good quality, very good packaging, good customer service	Jingdong prefecture level city in Shandong
Fortune Shop	White Intelligent Power Saver	Very good quality, especially good, effective, satisfactory	Jingdong prefecture level city in Shandong
Fortune Shop	White Intelligent Power Saver	I don't know how well it works.	Jingdong prefecture level city in Shandong
Fortune Shop	White Intelligent Power Saver	Good value for money, recommend it haha	Jingdong prefecture level city in Shandong
Fortune Shop	White Intelligent Power Saver	Good. Good. Very good.	Jingdong prefecture level city in Shandong
Fortune Shop	White Intelligent Power Saver	It's not working.	Jingdong prefecture level city in Shandong

Fortune Shop	White Intelligent Power Saver	It's not working.	Jingdong prefecture level city in Shandong
Fortune Shop	White Intelligent Power Saver	In use	Jingdong prefecture level city in Shandong
Fortune Shop	White Intelligent Power Saver	I'll try it later.	Jingdong prefecture level city in Shandong
Fortune Shop	White Intelligent Power Saver	It's not much use.	Jingdong prefecture level city in Shandong
Fortune Shop	White Intelligent Power Saver	It doesn't save electricity.	Jingdong prefecture level city in Shandong
Fortune Shop	White Intelligent Power Saver	It's not much use.	Jingdong prefecture level city in Shandong
Fortune Shop	White Intelligent Power Saver	Tried it for a few days and tracked the record, and there is no difference from before.	Jingdong prefecture level city in Shandong
Fortune Shop	White Intelligent Power Saver	It's not very credible, so just do it yourself and turn off the lights diligently!	Jingdong prefecture level city in Shandong
Fortune Shop	White Intelligent Power Saver	Too bad, no power saving at all, scam people don't fall for it!	Jingdong prefecture level city in Shandong
Fortune Shop	White Intelligent Power Saver	It's not working at all.	Jingdong prefecture level city in Shandong
Fortune Shop	White Intelligent Power Saver	general	Jingdong prefecture level city in Shandong
Fortune Shop	White Intelligent Power Saver	(of an unmarried couple) be close	Jingdong prefecture level city in Shandong
Fortune Shop	White Intelligent Power Saver	of no use	Jingdong prefecture level city in Shandong
Fortune Shop	White Intelligent Power Saver	Actually can really save electricity, only used half a month to save more than 50, recommended to the neighbors to use together!	Jingdong prefecture level city in Shandong
Fortune Shop	White Intelligent Power Saver	Save what you can. Isn't it more cost effective to buy one of these than to pay the electric bill?	Jingdong prefecture level city in Shandong
Fortune Shop	White Intelligent Power Saver	Received, a use do not know how, use a month to see the effect is good, the effect is good then recommended, not good then I just do not recommend.	Jingdong prefecture level city in Shandong
Fortune Shop	White Intelligent Power Saver	Just received the goods, is a voltage regulator, next month to evaluate whether it is really power saving	Jingdong prefecture level city in Shandong
Fortune Shop	White Intelligent Power Saver	Very good, used a few days obviously feel that the electricity bill is much less, wait a month to see how much can be saved!	Jingdong prefecture level city in Shandong
Fortune Shop	White Intelligent Power Saver	Received the stuff used for more than a month without a little use, it feels like it uses more electricity than before	Jingdong prefecture level city in Shandong
Fortune Shop	White Intelligent Power Saver	The quality is very good, completely exceeded the expectations, the delivery speed is very fast, the packaging is very careful and strict, very satisfied with a shopping	Jingdong prefecture level city in Shandong
Fortune Shop	White Intelligent Power Saver	Fake, doesn't work at all. Don't even sell it.	Jingdong prefecture level city in Shandong
Fortune Shop	White Intelligent Power Saver	This item doesn't seem to have much effect?	Jingdong prefecture level city in Shandong
Fortune Shop	White Intelligent Power Saver	This production is a bit fake, just look at the focus on Ming	Jingdong prefecture level city in Shandong
Fortune Shop	White Intelligent Power Saver	The logistics is fast the service attitude is very good.	Jingdong prefecture level city in Shandong
Fortune Shop	White Intelligent Power Saver	The meter is good quality, sturdy and durable. It is accurate. Easy to use. Saves a lot of things.	Jingdong prefecture level city in Shandong
Fortune Shop	White Intelligent Power Saver	Just received the goods, don't know how it is yet	Jingdong prefecture level city in Shandong
Fortune Shop	White Intelligent Power Saver	Energy saver received, logistics is very fast, the quality is very good, the appearance is also very beautiful power saving is the most important.	Jingdong prefecture level city in Shandong
Fortune Shop	White Intelligent Power Saver	It's good to talk about it, it's not practical to use it.	Jingdong prefecture level city in Shandong
Fortune Shop	White Intelligent Power Saver	I don't know if it's good for now, I'll comment after using it	Jingdong prefecture level city in Shandong
Fortune Shop	White Intelligent Power Saver	I've been using it for two or three days. It's not energy efficient.	Jingdong prefecture level city in Shandong
Fortune Shop	White Intelligent Power Saver	Packaging is intact, logistics is also fast, cost-effective, inexpensive, very satisfied with a purchase	Jingdong prefecture level city in Shandong
Fortune Shop	White Intelligent Power Saver	In use to be observed!	Jingdong prefecture level city in Shandong
Fortune Shop	White Intelligent Power Saver	Use it for a while at a flat rate	Jingdong prefecture level city in Shandong
Fortune Shop	White Intelligent Power Saver	Very trustworthy brand, quality is guaranteed, the price is still affordable, quite satisfied!	Jingdong prefecture level city in Shandong
Fortune Shop	White Intelligent Power Saver	Great quality, great operation, easy to install, great price!	Jingdong prefecture level city in Shandong
Fortune Shop	White Intelligent Power Saver	I came back and installed it, it really saves electricity, much more than before, the	Jingdong prefecture level city in Shandong
Fortune Shop	White Intelligent Power Saver	Fast logistics, good quality, very good packaging, good customer service	Jingdong prefecture level city in Shandong
Fortune Shop	White Intelligent Power Saver	Very good quality, especially good, effective, satisfactory	Jingdong prefecture level city in Shandong
Fortune Shop	White Intelligent Power Saver	I don't know how well it works.	Jingdong prefecture level city in Shandong
Fortune Shop	White Intelligent Power Saver	Good value for money, recommend it haha	Jingdong prefecture level city in Shandong
Fortune Shop	White Intelligent Power Saver	Good. Good. Very good.	Jingdong prefecture level city in Shandong
Fortune Shop	White Intelligent Power Saver	It's not working.	Jingdong prefecture level city in Shandong
Fortune Shop	White Intelligent Power Saver	It's not working.	Jingdong prefecture level city in Shandong
Fortune Shop	White Intelligent Power Saver	In use	Jingdong prefecture level city in Shandong
Fortune Shop	White Intelligent Power Saver	I'll try it later.	Jingdong prefecture level city in Shandong
Fortune Shop	White Intelligent Power Saver	It's not much use.	Jingdong prefecture level city in Shandong
Fortune Shop	White Intelligent Power Saver	It doesn't save electricity.	Jingdong prefecture level city in Shandong
Fortune Shop	White Intelligent Power Saver	It's not much use.	Jingdong prefecture level city in Shandong
Fortune Shop	White Intelligent Power Saver	Tried it for a few days and tracked the record, and there is no difference from before.	Jingdong prefecture level city in Shandong
Fortune Shop	White Intelligent Power Saver	It's not very credible, so just do it yourself and turn off the lights diligently!	Jingdong prefecture level city in Shandong
Fortune Shop	White Intelligent Power Saver	Too bad, no power saving at all, scam people don't fall for it!	Jingdong prefecture level city in Shandong

Fortune Shop	White Intelligent Power Saver	It's not working at all.	Jingdong prefecture level city in Shandong
Fortune Shop	White Intelligent Power Saver	general	Jingdong prefecture level city in Shandong
Fortune Shop	White Intelligent Power Saver	(of an unmarried couple) be close	Jingdong prefecture level city in Shandong
Fortune Shop	White Intelligent Power Saver	of no use	Jingdong prefecture level city in Shandong
Fortune Shop	White Intelligent Power Saver	Actually can really save electricity, only used half a month to save more than 50, recommended to the neighbors to use together!	Jingdong prefecture level city in Shandong
Fortune Shop	White Intelligent Power Saver	Save what you can. Isn't it more cost effective to buy one of these than to pay the electric bill?	Jingdong prefecture level city in Shandong
Fortune Shop	White Intelligent Power Saver	Received, a use do not know how, use a month to see the effect is good, the effect is good then recommended, not good then I just do not recommend.	Jingdong prefecture level city in Shandong
Fortune Shop	White Intelligent Power Saver	Just received the goods, is a voltage regulator, next month to evaluate whether it is really power saving	Jingdong prefecture level city in Shandong
Fortune Shop	White Intelligent Power Saver	Very good, used a few days obviously feel that the electricity bill is much less, wait a month to see how much can be saved!	Jingdong prefecture level city in Shandong
Fortune Shop	White Intelligent Power Saver	Received the stuff used for more than a month without a little use, it feels like it uses more electricity than before	Jingdong prefecture level city in Shandong
Fortune Shop	White Intelligent Power Saver	The quality is very good, completely exceeded the expectations, the delivery speed is very fast, the packaging is very careful and strict, very satisfied with a shopping	Jingdong prefecture level city in Shandong
Fortune Shop	White Intelligent Power Saver	Fake, doesn't work at all. Don't even sell it.	Jingdong prefecture level city in Shandong
Fortune Shop	White Intelligent Power Saver	This item doesn't seem to have much effect?	Jingdong prefecture level city in Shandong
Fortune Shop	White Intelligent Power Saver	This production is a bit fake, just look at the focus on Ming	Jingdong prefecture level city in Shandong
Fortune Shop	White Intelligent Power Saver	The logistics is fast the service attitude is very good.	Jingdong prefecture level city in Shandong
Fortune Shop	White Intelligent Power Saver	The meter is good quality, sturdy and durable. It is accurate. Easy to use. Saves a lot of things.	Jingdong prefecture level city in Shandong
Fortune Shop	White Intelligent Power Saver	Just received the goods, don't know how it is yet	Jingdong prefecture level city in Shandong
Fortune Shop	White Intelligent Power Saver	Energy saver received, logistics is very fast, the quality is very good, the appearance is also very beautiful power saving is the most important.	Jingdong prefecture level city in Shandong
Fortune Shop	White Intelligent Power Saver	It's good to talk about it, it's not practical to use it.	Jingdong prefecture level city in Shandong
Fortune Shop	White Intelligent Power Saver	I don't know if it's good for now, I'll comment after using it	Jingdong prefecture level city in Shandong
Fortune Shop	White Intelligent Power Saver	I've been using it for two or three days. It's not energy efficient.	Jingdong prefecture level city in Shandong
Fortune Shop	White Intelligent Power Saver	Packaging is intact, logistics is also fast, cost-effective, inexpensive, very satisfied with a purchase	Jingdong prefecture level city in Shandong
Fortune Shop	White Intelligent Power Saver	In use to be observed!	Jingdong prefecture level city in Shandong
Fortune Shop	White Intelligent Power Saver	Use it for a while at a flat rate	Jingdong prefecture level city in Shandong
Fortune Shop	White Intelligent Power Saver	Very trustworthy brand, quality is guaranteed, the price is still affordable, quite satisfied!	Jingdong prefecture level city in Shandong
Fortune Shop	White Intelligent Power Saver	Great quality, great operation, easy to install, great price!	Jingdong prefecture level city in Shandong
Fortune Shop	White Intelligent Power Saver	I came back and installed it, it really saves electricity, much more than before, the	Jingdong prefecture level city in Shandong
Fortune Shop	White Intelligent Power Saver	Fast logistics, good quality, very good packaging, good customer service	Jingdong prefecture level city in Shandong
Fortune Shop	White Intelligent Power Saver	Very good quality, especially good, effective, satisfactory	Jingdong prefecture level city in Shandong
Fortune Shop	White Intelligent Power Saver	I don't know how well it works.	Jingdong prefecture level city in Shandong
Fortune Shop	White Intelligent Power Saver	Good value for money, recommend it haha	Jingdong prefecture level city in Shandong
Fortune Shop	White Intelligent Power Saver	Good. Good. Very good.	Jingdong prefecture level city in Shandong
Fortune Shop	White Intelligent Power Saver	It's not working.	Jingdong prefecture level city in Shandong
Fortune Shop	White Intelligent Power Saver	It's not working.	Jingdong prefecture level city in Shandong
Fortune Shop	White Intelligent Power Saver	In use	Jingdong prefecture level city in Shandong
Fortune Shop	White Intelligent Power Saver	I'll try it later.	Jingdong prefecture level city in Shandong
Fortune Shop	White Intelligent Power Saver	It's not much use.	Jingdong prefecture level city in Shandong
Fortune Shop	White Intelligent Power Saver	It doesn't save electricity.	Jingdong prefecture level city in Shandong
Fortune Shop	White Intelligent Power Saver	It's not much use.	Jingdong prefecture level city in Shandong
Fortune Shop	White Intelligent Power Saver	Tried it for a few days and tracked the record, and there is no difference from before.	Jingdong prefecture level city in Shandong
Fortune Shop	White Intelligent Power Saver	It's not very credible, so just do it yourself and turn off the lights diligently!	Jingdong prefecture level city in Shandong
Fortune Shop	White Intelligent Power Saver	Too bad, no power saving at all, scam people don't fall for it!	Jingdong prefecture level city in Shandong
Fortune Shop	White Intelligent Power Saver	It's not working at all.	Jingdong prefecture level city in Shandong
Fortune Shop	White Intelligent Power Saver	general	Jingdong prefecture level city in Shandong
Fortune Shop	White Intelligent Power Saver	(of an unmarried couple) be close	Jingdong prefecture level city in Shandong
Fortune Shop	White Intelligent Power Saver	of no use	Jingdong prefecture level city in Shandong
Fortune Shop	White Intelligent Power Saver	Actually can really save electricity, only used half a month to save more than 50, recommended to the neighbors to use together!	Jingdong prefecture level city in Shandong
Fortune Shop	White Intelligent Power Saver	Save what you can. Isn't it more cost effective to buy one of these than to pay the electric bill?	Jingdong prefecture level city in Shandong
Fortune Shop	White Intelligent Power Saver	Received, a use do not know how, use a month to see the effect is good, the effect is good then recommended, not good then I just do not recommend.	Jingdong prefecture level city in Shandong
Fortune Shop	White Intelligent Power Saver	Just received the goods, is a voltage regulator, next month to evaluate whether it is really power saving	Jingdong prefecture level city in Shandong
Fortune Shop	White Intelligent Power Saver	Very good, used a few days obviously feel that the electricity bill is much less, wait a month to see how much can be saved!	Jingdong prefecture level city in Shandong

Fortune Shop	White Intelligent Power Saver	Received the stuff used for more than a month without a little use, it feels like it uses more electricity than before	Jingdong prefecture level city in Shandong
Fortune Shop	White Intelligent Power Saver	The quality is very good, completely exceeded the expectations, the delivery speed is very fast, the packaging is very careful and strict, very satisfied with a shopping	Jingdong prefecture level city in Shandong
Fortune Shop	White Intelligent Power Saver	Fake, doesn't work at all. Don't even sell it.	Jingdong prefecture level city in Shandong
Fortune Shop	White Intelligent Power Saver	This item doesn't seem to have much effect?	Jingdong prefecture level city in Shandong
Fortune Shop	White Intelligent Power Saver	This production is a bit fake, just look at the focus on Ming	Jingdong prefecture level city in Shandong
Fortune Shop	White Intelligent Power Saver	The logistics is fast the service attitude is very good.	Jingdong prefecture level city in Shandong
Fortune Shop	White Intelligent Power Saver	The meter is good quality, sturdy and durable. It is accurate. Easy to use. Saves a lot of things.	Jingdong prefecture level city in Shandong
Fortune Shop	White Intelligent Power Saver	Just received the goods, don't know how it is yet	Jingdong prefecture level city in Shandong
Fortune Shop	White Intelligent Power Saver	Energy saver received, logistics is very fast, the quality is very good, the appearance is also very beautiful power saving is the most important.	Jingdong prefecture level city in Shandong
Fortune Shop	White Intelligent Power Saver	It's good to talk about it, it's not practical to use it.	Jingdong prefecture level city in Shandong
Fortune Shop	White Intelligent Power Saver	I don't know if it's good for now, I'll comment after using it	Jingdong prefecture level city in Shandong
Fortune Shop	White Intelligent Power Saver	I've been using it for two or three days. It's not energy efficient.	Jingdong prefecture level city in Shandong
Fortune Shop	White Intelligent Power Saver	Packaging is intact, logistics is also fast, cost-effective, inexpensive, very satisfied with a purchase	Jingdong prefecture level city in Shandong
Fortune Shop	White Intelligent Power Saver	In use to be observed!	Jingdong prefecture level city in Shandong
Fortune Shop	White Intelligent Power Saver	Use it for a while at a flat rate	Jingdong prefecture level city in Shandong
Fortune Shop	White Intelligent Power Saver	Very trustworthy brand, quality is guaranteed, the price is still affordable, quite satisfied!	Jingdong prefecture level city in Shandong
Fortune Shop	White Intelligent Power Saver	Great quality, great operation, easy to install, great price!	Jingdong prefecture level city in Shandong
Fortune Shop	White Intelligent Power Saver	I came back and installed it, it really saves electricity, much more than before, the	Jingdong prefecture level city in Shandong
Fortune Shop	White Intelligent Power Saver	Fast logistics, good quality, very good packaging, good customer service	Jingdong prefecture level city in Shandong
Fortune Shop	White Intelligent Power Saver	Very good quality, especially good, effective, satisfactory	Jingdong prefecture level city in Shandong
Fortune Shop	White Intelligent Power Saver	I don't know how well it works.	Jingdong prefecture level city in Shandong
Fortune Shop	White Intelligent Power Saver	Good value for money, recommend it haha	Jingdong prefecture level city in Shandong
Fortune Shop	White Intelligent Power Saver	Good. Good. Very good.	Jingdong prefecture level city in Shandong
Fortune Shop	White Intelligent Power Saver	It's not working.	Jingdong prefecture level city in Shandong
Fortune Shop	White Intelligent Power Saver	It's not working.	Jingdong prefecture level city in Shandong
Fortune Shop	White Intelligent Power Saver	In use	Jingdong prefecture level city in Shandong
Fortune Shop	White Intelligent Power Saver	I'll try it later.	Jingdong prefecture level city in Shandong
Fortune Shop	White Intelligent Power Saver	It's not much use.	Jingdong prefecture level city in Shandong
Fortune Shop	White Intelligent Power Saver	It doesn't save electricity.	Jingdong prefecture level city in Shandong
Fortune Shop	White Intelligent Power Saver	It's not much use.	Jingdong prefecture level city in Shandong
Fortune Shop	White Intelligent Power Saver	Tried it for a few days and tracked the record, and there is no difference from before.	Jingdong prefecture level city in Shandong
Fortune Shop	White Intelligent Power Saver	It's not very credible, so just do it yourself and turn off the lights diligently!	Jingdong prefecture level city in Shandong
Fortune Shop	White Intelligent Power Saver	Too bad, no power saving at all, scam people don't fall for it!	Jingdong prefecture level city in Shandong
Fortune Shop	White Intelligent Power Saver	It's not working at all.	Jingdong prefecture level city in Shandong
Fortune Shop	White Intelligent Power Saver	general	Jingdong prefecture level city in Shandong
Fortune Shop	White Intelligent Power Saver	(of an unmarried couple) be close	Jingdong prefecture level city in Shandong
Fortune Shop	White Intelligent Power Saver	of no use	Jingdong prefecture level city in Shandong
Fortune Shop	White Intelligent Power Saver	Actually can really save electricity, only used half a month to save more than 50, recommended to the neighbors to use together!	Jingdong prefecture level city in Shandong
Fortune Shop	White Intelligent Power Saver	Save what you can. Isn't it more cost effective to buy one of these than to pay the electric bill?	Jingdong prefecture level city in Shandong
Fortune Shop	White Intelligent Power Saver	Received, a use do not know how, use a month to see the effect is good, the effect is good then recommended, not good then I just do not recommend.	Jingdong prefecture level city in Shandong
Fortune Shop	White Intelligent Power Saver	Just received the goods, is a voltage regulator, next month to evaluate whether it is really power saving	Jingdong prefecture level city in Shandong
Fortune Shop	White Intelligent Power Saver	Very good, used a few days obviously feel that the electricity bill is much less, wait a month to see how much can be saved!	Jingdong prefecture level city in Shandong
Fortune Shop	White Intelligent Power Saver	Received the stuff used for more than a month without a little use, it feels like it uses more electricity than before	Jingdong prefecture level city in Shandong
Fortune Shop	White Intelligent Power Saver	The quality is very good, completely exceeded the expectations, the delivery speed is very fast, the packaging is very careful and strict, very satisfied with a shopping	Jingdong prefecture level city in Shandong
Fortune Shop	White Intelligent Power Saver	Fake, doesn't work at all. Don't even sell it.	Jingdong prefecture level city in Shandong
Fortune Shop	White Intelligent Power Saver	This item doesn't seem to have much effect?	Jingdong prefecture level city in Shandong
Fortune Shop	White Intelligent Power Saver	This production is a bit fake, just look at the focus on Ming	Jingdong prefecture level city in Shandong
Fortune Shop	White Intelligent Power Saver	The logistics is fast the service attitude is very good.	Jingdong prefecture level city in Shandong
Fortune Shop	White Intelligent Power Saver	The meter is good quality, sturdy and durable. It is accurate. Easy to use. Saves a lot of things.	Jingdong prefecture level city in Shandong
Fortune Shop	White Intelligent Power Saver	Just received the goods, don't know how it is yet	Jingdong prefecture level city in Shandong
Fortune Shop	White Intelligent Power Saver	Energy saver received, logistics is very fast, the quality is very good, the appearance is also very beautiful power saving is the most important.	Jingdong prefecture level city in Shandong

Fortune Shop	White Intelligent Power Saver	It's good to talk about it, it's not practical to use it.	Jingdong prefecture level city in Shandong
Fortune Shop	White Intelligent Power Saver	I don't know if it's good for now, I'll comment after using it	Jingdong prefecture level city in Shandong
Fortune Shop	White Intelligent Power Saver	I've been using it for two or three days. It's not energy efficient.	Jingdong prefecture level city in Shandong
Fortune Shop	White Intelligent Power Saver	Packaging is intact, logistics is also fast, cost-effective, inexpensive, very satisfied with a purchase	Jingdong prefecture level city in Shandong
Fortune Shop	White Intelligent Power Saver	In use to be observed!	Jingdong prefecture level city in Shandong
Fortune Shop	White Intelligent Power Saver	Use it for a while at a flat rate	Jingdong prefecture level city in Shandong
Fortune Shop	White Intelligent Power Saver	Very trustworthy brand, quality is guaranteed, the price is still affordable, quite satisfied!	Jingdong prefecture level city in Shandong
Fortune Shop	White Intelligent Power Saver	Great quality, great operation, easy to install, great price!	Jingdong prefecture level city in Shandong
Fortune Shop	White Intelligent Power Saver	I came back and installed it, it really saves electricity, much more than before, the	Jingdong prefecture level city in Shandong
Fortune Shop	White Intelligent Power Saver	Fast logistics, good quality, very good packaging, good customer service	Jingdong prefecture level city in Shandong
Fortune Shop	White Intelligent Power Saver	Very good quality, especially good, effective, satisfactory	Jingdong prefecture level city in Shandong
Fortune Shop	White Intelligent Power Saver	I don't know how well it works.	Jingdong prefecture level city in Shandong
Fortune Shop	White Intelligent Power Saver	Good value for money, recommend it haha	Jingdong prefecture level city in Shandong
Fortune Shop	White Intelligent Power Saver	Good. Good. Very good.	Jingdong prefecture level city in Shandong
Fortune Shop	White Intelligent Power Saver	It's not working.	Jingdong prefecture level city in Shandong
Fortune Shop	White Intelligent Power Saver	It's not working.	Jingdong prefecture level city in Shandong
Fortune Shop	White Intelligent Power Saver	In use	Jingdong prefecture level city in Shandong
Fortune Shop	White Intelligent Power Saver	I'll try it later.	Jingdong prefecture level city in Shandong
Fortune Shop	White Intelligent Power Saver	It's not much use.	Jingdong prefecture level city in Shandong
Fortune Shop	White Intelligent Power Saver	It doesn't save electricity.	Jingdong prefecture level city in Shandong
Fortune Shop	White Intelligent Power Saver	It's not much use.	Jingdong prefecture level city in Shandong
Fortune Shop	White Intelligent Power Saver	Tried it for a few days and tracked the record, and there is no difference from before.	Jingdong prefecture level city in Shandong
Fortune Shop	White Intelligent Power Saver	It's not very credible, so just do it yourself and turn off the lights diligently!	Jingdong prefecture level city in Shandong
Fortune Shop	White Intelligent Power Saver	Too bad, no power saving at all, scam people don't fall for it!	Jingdong prefecture level city in Shandong
Fortune Shop	White Intelligent Power Saver	It's not working at all.	Jingdong prefecture level city in Shandong
Fortune Shop	White Intelligent Power Saver	general	Jingdong prefecture level city in Shandong
Fortune Shop	White Intelligent Power Saver	(of an unmarried couple) be close	Jingdong prefecture level city in Shandong
Fortune Shop	White Intelligent Power Saver	of no use	Jingdong prefecture level city in Shandong
Fortune Shop	White Intelligent Power Saver	Actually can really save electricity, only used half a month to save more than 50, recommended to the neighbors to use together!	Jingdong prefecture level city in Shandong
Fortune Shop	White Intelligent Power Saver	Save what you can. Isn't it more cost effective to buy one of these than to pay the electric bill?	Jingdong prefecture level city in Shandong
Fortune Shop	White Intelligent Power Saver	Received, a use do not know how, use a month to see the effect is good, the effect is good then recommended, not good then I just do not recommend.	Jingdong prefecture level city in Shandong
Fortune Shop	White Intelligent Power Saver	Just received the goods, is a voltage regulator, next month to evaluate whether it is really power saving	Jingdong prefecture level city in Shandong
Fortune Shop	White Intelligent Power Saver	Very good, used a few days obviously feel that the electricity bill is much less, wait a month to see how much can be saved!	Jingdong prefecture level city in Shandong
Fortune Shop	White Intelligent Power Saver	Received the stuff used for more than a month without a little use, it feels like it uses more electricity than before	Jingdong prefecture level city in Shandong
Fortune Shop	White Intelligent Power Saver	The quality is very good, completely exceeded the expectations, the delivery speed is very fast, the packaging is very careful and strict, very satisfied with a shopping	Jingdong prefecture level city in Shandong
Fortune Shop	White Intelligent Power Saver	Fake, doesn't work at all. Don't even sell it.	Jingdong prefecture level city in Shandong
Fortune Shop	White Intelligent Power Saver	This item doesn't seem to have much effect?	Jingdong prefecture level city in Shandong
Fortune Shop	White Intelligent Power Saver	This production is a bit fake, just look at the focus on Ming	Jingdong prefecture level city in Shandong
Fortune Shop	White Intelligent Power Saver	The logistics is fast the service attitude is very good.	Jingdong prefecture level city in Shandong
Fortune Shop	White Intelligent Power Saver	The meter is good quality, sturdy and durable. It is accurate. Easy to use. Saves a lot of things.	Jingdong prefecture level city in Shandong
Fortune Shop	White Intelligent Power Saver	Just received the goods, don't know how it is yet	Jingdong prefecture level city in Shandong
Fortune Shop	White Intelligent Power Saver	Energy saver received, logistics is very fast, the quality is very good, the appearance is also very beautiful power saving is the most important.	Jingdong prefecture level city in Shandong
Fortune Shop	White Intelligent Power Saver	It's good to talk about it, it's not practical to use it.	Jingdong prefecture level city in Shandong
Fortune Shop	White Intelligent Power Saver	I don't know if it's good for now, I'll comment after using it	Jingdong prefecture level city in Shandong
Fortune Shop	White Intelligent Power Saver	I've been using it for two or three days. It's not energy efficient.	Jingdong prefecture level city in Shandong
Fortune Shop	White Intelligent Power Saver	Packaging is intact, logistics is also fast, cost-effective, inexpensive, very satisfied with a purchase	Jingdong prefecture level city in Shandong
Fortune Shop	White Intelligent Power Saver	In use to be observed!	Jingdong prefecture level city in Shandong
Fortune Shop	White Intelligent Power Saver	Use it for a while at a flat rate	Jingdong prefecture level city in Shandong
Fortune Shop	White Intelligent Power Saver	Very trustworthy brand, quality is guaranteed, the price is still affordable, quite satisfied!	Jingdong prefecture level city in Shandong
Fortune Shop	White Intelligent Power Saver	Great quality, great operation, easy to install, great price!	Jingdong prefecture level city in Shandong
Fortune Shop	White Intelligent Power Saver	I came back and installed it, it really saves electricity, much more than before, the	Jingdong prefecture level city in Shandong

Fortune Shop	White Intelligent Power Saver	Fast logistics, good quality, very good packaging, good customer service	Jingdong prefecture level city in Shandong
Fortune Shop	White Intelligent Power Saver	Very good quality, especially good, effective, satisfactory	Jingdong prefecture level city in Shandong
Fortune Shop	White Intelligent Power Saver	I don't know how well it works.	Jingdong prefecture level city in Shandong
Fortune Shop	White Intelligent Power Saver	Good value for money, recommend it haha	Jingdong prefecture level city in Shandong
Fortune Shop	White Intelligent Power Saver	Good. Good. Very good.	Jingdong prefecture level city in Shandong
Fortune Shop	White Intelligent Power Saver	It's not working.	Jingdong prefecture level city in Shandong
Fortune Shop	White Intelligent Power Saver	It's not working.	Jingdong prefecture level city in Shandong
Fortune Shop	White Intelligent Power Saver	In use	Jingdong prefecture level city in Shandong
Fortune Shop	White Intelligent Power Saver	I'll try it later.	Jingdong prefecture level city in Shandong
Fortune Shop	White Intelligent Power Saver	It's not much use.	Jingdong prefecture level city in Shandong
Fortune Shop	White Intelligent Power Saver	It doesn't save electricity.	Jingdong prefecture level city in Shandong
Fortune Shop	White Intelligent Power Saver	It's not much use.	Jingdong prefecture level city in Shandong
Fortune Shop	White Intelligent Power Saver	Tried it for a few days and tracked the record, and there is no difference from before.	Jingdong prefecture level city in Shandong
Fortune Shop	White Intelligent Power Saver	It's not very credible, so just do it yourself and turn off the lights diligently!	Jingdong prefecture level city in Shandong
Fortune Shop	White Intelligent Power Saver	Too bad, no power saving at all, scam people don't fall for it!	Jingdong prefecture level city in Shandong
Fortune Shop	White Intelligent Power Saver	It's not working at all.	Jingdong prefecture level city in Shandong
Fortune Shop	White Intelligent Power Saver	general	Jingdong prefecture level city in Shandong
Fortune Shop	White Intelligent Power Saver	(of an unmarried couple) be close	Jingdong prefecture level city in Shandong
Fortune Shop	White Intelligent Power Saver	of no use	Jingdong prefecture level city in Shandong
Fortune Shop	White Intelligent Power Saver	Actually can really save electricity, only used half a month to save more than 50, recommended to the neighbors to use together!	Jingdong prefecture level city in Shandong
Fortune Shop	White Intelligent Power Saver	Save what you can. Isn't it more cost effective to buy one of these than to pay the electric bill?	Jingdong prefecture level city in Shandong
Fortune Shop	White Intelligent Power Saver	Received, a use do not know how, use a month to see the effect is good, the effect is good then recommended, not good then I just do not recommend.	Jingdong prefecture level city in Shandong
Fortune Shop	White Intelligent Power Saver	Just received the goods, is a voltage regulator, next month to evaluate whether it is really power saving	Jingdong prefecture level city in Shandong
Fortune Shop	White Intelligent Power Saver	Very good, used a few days obviously feel that the electricity bill is much less, wait a month to see how much can be saved!	Jingdong prefecture level city in Shandong
Fortune Shop	White Intelligent Power Saver	Received the stuff used for more than a month without a little use, it feels like it uses more electricity than before	Jingdong prefecture level city in Shandong
Fortune Shop	White Intelligent Power Saver	The quality is very good, completely exceeded the expectations, the delivery speed is very fast, the packaging is very careful and strict, very satisfied with a shopping	Jingdong prefecture level city in Shandong
Fortune Shop	White Intelligent Power Saver	Fake, doesn't work at all. Don't even sell it.	Jingdong prefecture level city in Shandong
Fortune Shop	White Intelligent Power Saver	This item doesn't seem to have much effect?	Jingdong prefecture level city in Shandong
Fortune Shop	White Intelligent Power Saver	This production is a bit fake, just look at the focus on Ming	Jingdong prefecture level city in Shandong
Fortune Shop	White Intelligent Power Saver	The logistics is fast the service attitude is very good.	Jingdong prefecture level city in Shandong
Fortune Shop	White Intelligent Power Saver	The meter is good quality, sturdy and durable. It is accurate. Easy to use. Saves a lot of things.	Jingdong prefecture level city in Shandong
Fortune Shop	White Intelligent Power Saver	Just received the goods, don't know how it is yet	Jingdong prefecture level city in Shandong
Fortune Shop	White Intelligent Power Saver	Energy saver received, logistics is very fast, the quality is very good, the appearance is also very beautiful power saving is the most important.	Jingdong prefecture level city in Shandong
Fortune Shop	White Intelligent Power Saver	It's good to talk about it, it's not practical to use it.	Jingdong prefecture level city in Shandong
Fortune Shop	White Intelligent Power Saver	I don't know if it's good for now, I'll comment after using it	Jingdong prefecture level city in Shandong
Fortune Shop	White Intelligent Power Saver	I've been using it for two or three days. It's not energy efficient.	Jingdong prefecture level city in Shandong
Fortune Shop	White Intelligent Power Saver	Packaging is intact, logistics is also fast, cost-effective, inexpensive, very satisfied with a purchase	Jingdong prefecture level city in Shandong
Fortune Shop	White Intelligent Power Saver	In use to be observed!	Jingdong prefecture level city in Shandong
Fortune Shop	White Intelligent Power Saver	Use it for a while at a flat rate	Jingdong prefecture level city in Shandong
Fortune Shop	White Intelligent Power Saver	Very trustworthy brand, quality is guaranteed, the price is still affordable, quite satisfied!	Jingdong prefecture level city in Shandong
Fortune Shop	White Intelligent Power Saver	Great quality, great operation, easy to install, great price!	Jingdong prefecture level city in Shandong
Fortune Shop	White Intelligent Power Saver	I came back and installed it, it really saves electricity, much more than before, the	Jingdong prefecture level city in Shandong
Fortune Shop	White Intelligent Power Saver	Fast logistics, good quality, very good packaging, good customer service	Jingdong prefecture level city in Shandong
Fortune Shop	White Intelligent Power Saver	Very good quality, especially good, effective, satisfactory	Jingdong prefecture level city in Shandong
Fortune Shop	White Intelligent Power Saver	I don't know how well it works.	Jingdong prefecture level city in Shandong
Fortune Shop	White Intelligent Power Saver	Good value for money, recommend it haha	Jingdong prefecture level city in Shandong
Fortune Shop	White Intelligent Power Saver	Good. Good. Very good.	Jingdong prefecture level city in Shandong
Fortune Shop	White Intelligent Power Saver	It's not working.	Jingdong prefecture level city in Shandong
Fortune Shop	White Intelligent Power Saver	It's not working.	Jingdong prefecture level city in Shandong
Fortune Shop	White Intelligent Power Saver	In use	Jingdong prefecture level city in Shandong
Fortune Shop	White Intelligent Power Saver	I'll try it later.	Jingdong prefecture level city in Shandong

Fortune Shop	White Intelligent Power Saver	It's not much use.	Jingdong prefecture level city in Shandong
Fortune Shop	White Intelligent Power Saver	It doesn't save electricity.	Jingdong prefecture level city in Shandong
Fortune Shop	White Intelligent Power Saver	It's not much use.	Jingdong prefecture level city in Shandong
Fortune Shop	White Intelligent Power Saver	Tried it for a few days and tracked the record, and there is no difference from before.	Jingdong prefecture level city in Shandong
Fortune Shop	White Intelligent Power Saver	It's not very credible, so just do it yourself and turn off the lights diligently!	Jingdong prefecture level city in Shandong
Fortune Shop	White Intelligent Power Saver	Too bad, no power saving at all, scam people don't fall for it!	Jingdong prefecture level city in Shandong
Fortune Shop	White Intelligent Power Saver	It's not working at all.	Jingdong prefecture level city in Shandong
Fortune Shop	White Intelligent Power Saver	general	Jingdong prefecture level city in Shandong
Fortune Shop	White Intelligent Power Saver	(of an unmarried couple) be close	Jingdong prefecture level city in Shandong
Fortune Shop	White Intelligent Power Saver	of no use	Jingdong prefecture level city in Shandong
Fortune Shop	White Intelligent Power Saver	Actually can really save electricity, only used half a month to save more than 50, recommended to the neighbors to use together!	Jingdong prefecture level city in Shandong
Fortune Shop	White Intelligent Power Saver	Save what you can. Isn't it more cost effective to buy one of these than to pay the electric bill?	Jingdong prefecture level city in Shandong
Fortune Shop	White Intelligent Power Saver	Received, a use do not know how, use a month to see the effect is good, the effect is good then recommended, not good then I just do not recommend.	Jingdong prefecture level city in Shandong
Fortune Shop	White Intelligent Power Saver	Just received the goods, is a voltage regulator, next month to evaluate whether it is really power saving	Jingdong prefecture level city in Shandong
Fortune Shop	White Intelligent Power Saver	Very good, used a few days obviously feel that the electricity bill is much less, wait a month to see how much can be saved!	Jingdong prefecture level city in Shandong
Fortune Shop	White Intelligent Power Saver	Received the stuff used for more than a month without a little use, it feels like it uses more electricity than before	Jingdong prefecture level city in Shandong
Fortune Shop	White Intelligent Power Saver	The quality is very good, completely exceeded the expectations, the delivery speed is very fast, the packaging is very careful and strict, very satisfied with a shopping	Jingdong prefecture level city in Shandong
Fortune Shop	White Intelligent Power Saver	Fake, doesn't work at all. Don't even sell it.	Jingdong prefecture level city in Shandong
Fortune Shop	White Intelligent Power Saver	This item doesn't seem to have much effect?	Jingdong prefecture level city in Shandong
Fortune Shop	White Intelligent Power Saver	This production is a bit fake, just look at the focus on Ming	Jingdong prefecture level city in Shandong
Fortune Shop	White Intelligent Power Saver	The logistics is fast the service attitude is very good.	Jingdong prefecture level city in Shandong
Fortune Shop	White Intelligent Power Saver	The meter is good quality, sturdy and durable. It is accurate. Easy to use. Saves a lot of things.	Jingdong prefecture level city in Shandong
Fortune Shop	White Intelligent Power Saver	Just received the goods, don't know how it is yet	Jingdong prefecture level city in Shandong
Fortune Shop	White Intelligent Power Saver	Energy saver received, logistics is very fast, the quality is very good, the appearance is also very beautiful power saving is the most important.	Jingdong prefecture level city in Shandong
Fortune Shop	White Intelligent Power Saver	It's good to talk about it, it's not practical to use it.	Jingdong prefecture level city in Shandong
Fortune Shop	White Intelligent Power Saver	I don't know if it's good for now, I'll comment after using it	Jingdong prefecture level city in Shandong
Fortune Shop	White Intelligent Power Saver	I've been using it for two or three days. It's not energy efficient.	Jingdong prefecture level city in Shandong
Fortune Shop	White Intelligent Power Saver	Packaging is intact, logistics is also fast, cost-effective, inexpensive, very satisfied with a purchase	Jingdong prefecture level city in Shandong
Fortune Shop	White Intelligent Power Saver	In use to be observed!	Jingdong prefecture level city in Shandong
Fortune Shop	White Intelligent Power Saver	Use it for a while at a flat rate	Jingdong prefecture level city in Shandong
Fortune Shop	White Intelligent Power Saver	Very trustworthy brand, quality is guaranteed, the price is still affordable, quite satisfied!	Jingdong prefecture level city in Shandong
Fortune Shop	White Intelligent Power Saver	Great quality, great operation, easy to install, great price!	Jingdong prefecture level city in Shandong
Fortune Shop	White Intelligent Power Saver	I came back and installed it, it really saves electricity, much more than before, the	Jingdong prefecture level city in Shandong
Fortune Shop	White Intelligent Power Saver	Fast logistics, good quality, very good packaging, good customer service	Jingdong prefecture level city in Shandong
Fortune Shop	White Intelligent Power Saver	Very good quality, especially good, effective, satisfactory	Jingdong prefecture level city in Shandong
Fortune Shop	White Intelligent Power Saver	I don't know how well it works.	Jingdong prefecture level city in Shandong
Fortune Shop	White Intelligent Power Saver	Good value for money, recommend it haha	Jingdong prefecture level city in Shandong
Fortune Shop	White Intelligent Power Saver	Good. Good. Very good.	Jingdong prefecture level city in Shandong
Fortune Shop	White Intelligent Power Saver	It's not working.	Jingdong prefecture level city in Shandong
Fortune Shop	White Intelligent Power Saver	It's not working.	Jingdong prefecture level city in Shandong
Fortune Shop	White Intelligent Power Saver	In use	Jingdong prefecture level city in Shandong
Fortune Shop	White Intelligent Power Saver	I'll try it later.	Jingdong prefecture level city in Shandong
Fortune Shop	White Intelligent Power Saver	It's not much use.	Jingdong prefecture level city in Shandong
Fortune Shop	White Intelligent Power Saver	It doesn't save electricity.	Jingdong prefecture level city in Shandong
Fortune Shop	White Intelligent Power Saver	It's not much use.	Jingdong prefecture level city in Shandong
Fortune Shop	White Intelligent Power Saver	Tried it for a few days and tracked the record, and there is no difference from before.	Jingdong prefecture level city in Shandong
Fortune Shop	White Intelligent Power Saver	It's not very credible, so just do it yourself and turn off the lights diligently!	Jingdong prefecture level city in Shandong
Fortune Shop	White Intelligent Power Saver	Too bad, no power saving at all, scam people don't fall for it!	Jingdong prefecture level city in Shandong
Fortune Shop	White Intelligent Power Saver	It's not working at all.	Jingdong prefecture level city in Shandong
Fortune Shop	White Intelligent Power Saver	general	Jingdong prefecture level city in Shandong
Fortune Shop	White Intelligent Power Saver	(of an unmarried couple) be close	Jingdong prefecture level city in Shandong

Fortune Shop	White Intelligent Power Saver	of no use	Jingdong prefecture level city in Shandong
Fortune Shop	White Intelligent Power Saver	Actually can really save electricity, only used half a month to save more than 50, recommended to the neighbors to use together!	Jingdong prefecture level city in Shandong
Fortune Shop	White Intelligent Power Saver	Save what you can. Isn't it more cost effective to buy one of these than to pay the electric bill?	Jingdong prefecture level city in Shandong
Fortune Shop	White Intelligent Power Saver	Received, a use do not know how, use a month to see the effect is good, the effect is good then recommended, not good then I just do not recommend.	Jingdong prefecture level city in Shandong
Fortune Shop	White Intelligent Power Saver	Just received the goods, is a voltage regulator, next month to evaluate whether it is really power saving	Jingdong prefecture level city in Shandong
Fortune Shop	White Intelligent Power Saver	Very good, used a few days obviously feel that the electricity bill is much less, wait a month to see how much can be saved!	Jingdong prefecture level city in Shandong
Fortune Shop	White Intelligent Power Saver	Received the stuff used for more than a month without a little use, it feels like it uses more electricity than before	Jingdong prefecture level city in Shandong
Fortune Shop	White Intelligent Power Saver	The quality is very good, completely exceeded the expectations, the delivery speed is very fast, the packaging is very careful and strict, very satisfied with a shopping	Jingdong prefecture level city in Shandong
Fortune Shop	White Intelligent Power Saver	Fake, doesn't work at all. Don't even sell it.	Jingdong prefecture level city in Shandong
Fortune Shop	White Intelligent Power Saver	This item doesn't seem to have much effect?	Jingdong prefecture level city in Shandong
Fortune Shop	White Intelligent Power Saver	This production is a bit fake, just look at the focus on Ming	Jingdong prefecture level city in Shandong
Fortune Shop	White Intelligent Power Saver	The logistics is fast the service attitude is very good.	Jingdong prefecture level city in Shandong
Fortune Shop	White Intelligent Power Saver	The meter is good quality, sturdy and durable. It is accurate. Easy to use. Saves a lot of things.	Jingdong prefecture level city in Shandong
Fortune Shop	White Intelligent Power Saver	Just received the goods, don't know how it is yet	Jingdong prefecture level city in Shandong
Fortune Shop	White Intelligent Power Saver	Energy saver received, logistics is very fast, the quality is very good, the appearance is also very beautiful power saving is the most important.	Jingdong prefecture level city in Shandong
Fortune Shop	White Intelligent Power Saver	It's good to talk about it, it's not practical to use it.	Jingdong prefecture level city in Shandong
Fortune Shop	White Intelligent Power Saver	I don't know if it's good for now, I'll comment after using it	Jingdong prefecture level city in Shandong
Fortune Shop	White Intelligent Power Saver	I've been using it for two or three days. It's not energy efficient.	Jingdong prefecture level city in Shandong
Fortune Shop	White Intelligent Power Saver	Packaging is intact, logistics is also fast, cost-effective, inexpensive, very satisfied with a purchase	Jingdong prefecture level city in Shandong
Fortune Shop	White Intelligent Power Saver	In use to be observed!	Jingdong prefecture level city in Shandong
Fortune Shop	White Intelligent Power Saver	Use it for a while at a flat rate	Jingdong prefecture level city in Shandong
Fortune Shop	White Intelligent Power Saver	Very trustworthy brand, quality is guaranteed, the price is still affordable, quite satisfied!	Jingdong prefecture level city in Shandong
Fortune Shop	White Intelligent Power Saver	Great quality, great operation, easy to install, great price!	Jingdong prefecture level city in Shandong
Fortune Shop	White Intelligent Power Saver	I came back and installed it, it really saves electricity, much more than before, the	Jingdong prefecture level city in Shandong
Fortune Shop	White Intelligent Power Saver	Fast logistics, good quality, very good packaging, good customer service	Jingdong prefecture level city in Shandong
Fortune Shop	White Intelligent Power Saver	Very good quality, especially good, effective, satisfactory	Jingdong prefecture level city in Shandong
Fortune Shop	White Intelligent Power Saver	I don't know how well it works.	Jingdong prefecture level city in Shandong
Fortune Shop	White Intelligent Power Saver	Good value for money, recommend it haha	Jingdong prefecture level city in Shandong
Fortune Shop	White Intelligent Power Saver	Good. Good. Very good.	Jingdong prefecture level city in Shandong
Fortune Shop	White Intelligent Power Saver	It's not working.	Jingdong prefecture level city in Shandong
Fortune Shop	White Intelligent Power Saver	It's not working.	Jingdong prefecture level city in Shandong
Fortune Shop	White Intelligent Power Saver	In use	Jingdong prefecture level city in Shandong
Fortune Shop	White Intelligent Power Saver	I'll try it later.	Jingdong prefecture level city in Shandong
Fortune Shop	White Intelligent Power Saver	It's not much use.	Jingdong prefecture level city in Shandong
Fortune Shop	White Intelligent Power Saver	It doesn't save electricity.	Jingdong prefecture level city in Shandong
Fortune Shop	White Intelligent Power Saver	It's not much use.	Jingdong prefecture level city in Shandong
Fortune Shop	White Intelligent Power Saver	Tried it for a few days and tracked the record, and there is no difference from before.	Jingdong prefecture level city in Shandong
Fortune Shop	White Intelligent Power Saver	It's not very credible, so just do it yourself and turn off the lights diligently!	Jingdong prefecture level city in Shandong
Fortune Shop	White Intelligent Power Saver	Too bad, no power saving at all, scam people don't fall for it!	Jingdong prefecture level city in Shandong
Fortune Shop	White Intelligent Power Saver	It's not working at all.	Jingdong prefecture level city in Shandong
Fortune Shop	White Intelligent Power Saver	general	Jingdong prefecture level city in Shandong
Fortune Shop	White Intelligent Power Saver	(of an unmarried couple) be close	Jingdong prefecture level city in Shandong
Fortune Shop	White Intelligent Power Saver	of no use	Jingdong prefecture level city in Shandong
Fortune Shop	White Intelligent Power Saver	Actually can really save electricity, only used half a month to save more than 50, recommended to the neighbors to use together!	Jingdong prefecture level city in Shandong
Fortune Shop	White Intelligent Power Saver	Save what you can. Isn't it more cost effective to buy one of these than to pay the electric bill?	Jingdong prefecture level city in Shandong
Fortune Shop	White Intelligent Power Saver	Received, a use do not know how, use a month to see the effect is good, the effect is good then recommended, not good then I just do not recommend.	Jingdong prefecture level city in Shandong
Fortune Shop	White Intelligent Power Saver	Just received the goods, is a voltage regulator, next month to evaluate whether it is really power saving	Jingdong prefecture level city in Shandong
Fortune Shop	White Intelligent Power Saver	Very good, used a few days obviously feel that the electricity bill is much less, wait a month to see how much can be saved!	Jingdong prefecture level city in Shandong
Fortune Shop	White Intelligent Power Saver	Received the stuff used for more than a month without a little use, it feels like it uses more electricity than before	Jingdong prefecture level city in Shandong
Fortune Shop	White Intelligent Power Saver	The quality is very good, completely exceeded the expectations, the delivery speed is very fast, the packaging is very careful and strict, very satisfied with a shopping	Jingdong prefecture level city in Shandong
Fortune Shop	White Intelligent Power Saver	Fake, doesn't work at all. Don't even sell it.	Jingdong prefecture level city in Shandong

Fortune Shop	White Intelligent Power Saver	This item doesn't seem to have much effect?	Jingdong prefecture level city in Shandong
Fortune Shop	White Intelligent Power Saver	This production is a bit fake, just look at the focus on Ming	Jingdong prefecture level city in Shandong
Fortune Shop	White Intelligent Power Saver	The logistics is fast the service attitude is very good.	Jingdong prefecture level city in Shandong
Fortune Shop	White Intelligent Power Saver	The meter is good quality, sturdy and durable. It is accurate. Easy to use. Saves a lot of things.	Jingdong prefecture level city in Shandong
Fortune Shop	White Intelligent Power Saver	Just received the goods, don't know how it is yet	Jingdong prefecture level city in Shandong
Fortune Shop	White Intelligent Power Saver	Energy saver received, logistics is very fast, the quality is very good, the appearance is also very beautiful power saving is the most important.	Jingdong prefecture level city in Shandong
Fortune Shop	White Intelligent Power Saver	It's good to talk about it, it's not practical to use it.	Jingdong prefecture level city in Shandong
Fortune Shop	White Intelligent Power Saver	I don't know if it's good for now, I'll comment after using it	Jingdong prefecture level city in Shandong
Fortune Shop	White Intelligent Power Saver	I've been using it for two or three days. It's not energy efficient.	Jingdong prefecture level city in Shandong
Fortune Shop	White Intelligent Power Saver	Packaging is intact, logistics is also fast, cost-effective, inexpensive, very satisfied with a purchase	Jingdong prefecture level city in Shandong
Fortune Shop	White Intelligent Power Saver	In use to be observed!	Jingdong prefecture level city in Shandong
Fortune Shop	White Intelligent Power Saver	Use it for a while at a flat rate	Jingdong prefecture level city in Shandong
Fortune Shop	White Intelligent Power Saver	Very trustworthy brand, quality is guaranteed, the price is still affordable, quite satisfied!	Jingdong prefecture level city in Shandong
Fortune Shop	White Intelligent Power Saver	Great quality, great operation, easy to install, great price!	Jingdong prefecture level city in Shandong
Fortune Shop	White Intelligent Power Saver	I came back and installed it, it really saves electricity, much more than before, the	Jingdong prefecture level city in Shandong
Fortune Shop	White Intelligent Power Saver	Fast logistics, good quality, very good packaging, good customer service	Jingdong prefecture level city in Shandong
Fortune Shop	White Intelligent Power Saver	Very good quality, especially good, effective, satisfactory	Jingdong prefecture level city in Shandong
Fortune Shop	White Intelligent Power Saver	I don't know how well it works.	Jingdong prefecture level city in Shandong
Fortune Shop	White Intelligent Power Saver	Good value for money, recommend it haha	Jingdong prefecture level city in Shandong
Fortune Shop	White Intelligent Power Saver	Good. Good. Very good.	Jingdong prefecture level city in Shandong
Fortune Shop	White Intelligent Power Saver	It's not working.	Jingdong prefecture level city in Shandong
Fortune Shop	White Intelligent Power Saver	It's not working.	Jingdong prefecture level city in Shandong
Fortune Shop	White Intelligent Power Saver	In use	Jingdong prefecture level city in Shandong
Fortune Shop	White Intelligent Power Saver	I'll try it later.	Jingdong prefecture level city in Shandong
Fortune Shop	White Intelligent Power Saver	It's not much use.	Jingdong prefecture level city in Shandong
Fortune Shop	White Intelligent Power Saver	It doesn't save electricity.	Jingdong prefecture level city in Shandong
Fortune Shop	White Intelligent Power Saver	It's not much use.	Jingdong prefecture level city in Shandong
Fortune Shop	White Intelligent Power Saver	Tried it for a few days and tracked the record, and there is no difference from before.	Jingdong prefecture level city in Shandong
Fortune Shop	White Intelligent Power Saver	It's not very credible, so just do it yourself and turn off the lights diligently!	Jingdong prefecture level city in Shandong
Fortune Shop	White Intelligent Power Saver	Too bad, no power saving at all, scam people don't fall for it!	Jingdong prefecture level city in Shandong
Fortune Shop	White Intelligent Power Saver	It's not working at all.	Jingdong prefecture level city in Shandong
Fortune Shop	White Intelligent Power Saver	general	Jingdong prefecture level city in Shandong
Fortune Shop	White Intelligent Power Saver	(of an unmarried couple) be close	Jingdong prefecture level city in Shandong
Fortune Shop	White Intelligent Power Saver	of no use	Jingdong prefecture level city in Shandong
Fortune Shop	White Intelligent Power Saver	Actually can really save electricity, only used half a month to save more than 50, recommended to the neighbors to use together!	Jingdong prefecture level city in Shandong
Fortune Shop	White Intelligent Power Saver	Save what you can. Isn't it more cost effective to buy one of these than to pay the electric bill?	Jingdong prefecture level city in Shandong
Fortune Shop	White Intelligent Power Saver	Received, a use do not know how, use a month to see the effect is good, the effect is good then recommended, not good then I just do not recommend.	Jingdong prefecture level city in Shandong
Fortune Shop	White Intelligent Power Saver	Just received the goods, is a voltage regulator, next month to evaluate whether it is really power saving	Jingdong prefecture level city in Shandong
Fortune Shop	White Intelligent Power Saver	Very good, used a few days obviously feel that the electricity bill is much less, wait a month to see how much can be saved!	Jingdong prefecture level city in Shandong
Fortune Shop	White Intelligent Power Saver	Received the stuff used for more than a month without a little use, it feels like it uses more electricity than before	Jingdong prefecture level city in Shandong
Fortune Shop	White Intelligent Power Saver	The quality is very good, completely exceeded the expectations, the delivery speed is very fast, the packaging is very careful and strict, very satisfied with a shopping	Jingdong prefecture level city in Shandong
Fortune Shop	White Intelligent Power Saver	Fake, doesn't work at all. Don't even sell it.	Jingdong prefecture level city in Shandong
Fortune Shop	White Intelligent Power Saver	This item doesn't seem to have much effect?	Jingdong prefecture level city in Shandong
Fortune Shop	White Intelligent Power Saver	This production is a bit fake, just look at the focus on Ming	Jingdong prefecture level city in Shandong
Fortune Shop	White Intelligent Power Saver	The logistics is fast the service attitude is very good.	Jingdong prefecture level city in Shandong
Fortune Shop	White Intelligent Power Saver	The meter is good quality, sturdy and durable. It is accurate. Easy to use. Saves a lot of things.	Jingdong prefecture level city in Shandong
Fortune Shop	White Intelligent Power Saver	Just received the goods, don't know how it is yet	Jingdong prefecture level city in Shandong
Fortune Shop	White Intelligent Power Saver	Energy saver received, logistics is very fast, the quality is very good, the appearance is also very beautiful power saving is the most important.	Jingdong prefecture level city in Shandong
Fortune Shop	White Intelligent Power Saver	It's good to talk about it, it's not practical to use it.	Jingdong prefecture level city in Shandong
Fortune Shop	White Intelligent Power Saver	I don't know if it's good for now, I'll comment after using it	Jingdong prefecture level city in Shandong
Fortune Shop	White Intelligent Power Saver	I've been using it for two or three days. It's not energy efficient.	Jingdong prefecture level city in Shandong

Fortune Shop	White Intelligent Power Saver	Packaging is intact, logistics is also fast, cost-effective, inexpensive, very satisfied with a purchase	Jingdong prefecture level city in Shandong
Fortune Shop	White Intelligent Power Saver	In use to be observed!	Jingdong prefecture level city in Shandong
Fortune Shop	White Intelligent Power Saver	Use it for a while at a flat rate	Jingdong prefecture level city in Shandong
Fortune Shop	White Intelligent Power Saver	Very trustworthy brand, quality is guaranteed, the price is still affordable, quite satisfied!	Jingdong prefecture level city in Shandong
Fortune Shop	White Intelligent Power Saver	Great quality, great operation, easy to install, great price!	Jingdong prefecture level city in Shandong
Fortune Shop	White Intelligent Power Saver	I came back and installed it, it really saves electricity, much more than before, the	Jingdong prefecture level city in Shandong
Fortune Shop	White Intelligent Power Saver	Fast logistics, good quality, very good packaging, good customer service	Jingdong prefecture level city in Shandong
Fortune Shop	White Intelligent Power Saver	Very good quality, especially good, effective, satisfactory	Jingdong prefecture level city in Shandong
Fortune Shop	White Intelligent Power Saver	I don't know how well it works.	Jingdong prefecture level city in Shandong
Fortune Shop	White Intelligent Power Saver	Good value for money, recommend it haha	Jingdong prefecture level city in Shandong
Fortune Shop	White Intelligent Power Saver	Good. Good. Very good.	Jingdong prefecture level city in Shandong
Fortune Shop	White Intelligent Power Saver	It's not working.	Jingdong prefecture level city in Shandong
Fortune Shop	White Intelligent Power Saver	It's not working.	Jingdong prefecture level city in Shandong
Fortune Shop	White Intelligent Power Saver	In use	Jingdong prefecture level city in Shandong
Fortune Shop	White Intelligent Power Saver	I'll try it later.	Jingdong prefecture level city in Shandong
Fortune Shop	White Intelligent Power Saver	It's not much use.	Jingdong prefecture level city in Shandong
Fortune Shop	White Intelligent Power Saver	It doesn't save electricity.	Jingdong prefecture level city in Shandong
Fortune Shop	White Intelligent Power Saver	It's not much use.	Jingdong prefecture level city in Shandong
Fortune Shop	White Intelligent Power Saver	Tried it for a few days and tracked the record, and there is no difference from before.	Jingdong prefecture level city in Shandong
Fortune Shop	White Intelligent Power Saver	It's not very credible, so just do it yourself and turn off the lights diligently!	Jingdong prefecture level city in Shandong
Fortune Shop	White Intelligent Power Saver	Too bad, no power saving at all, scam people don't fall for it!	Jingdong prefecture level city in Shandong
Fortune Shop	White Intelligent Power Saver	It's not working at all.	Jingdong prefecture level city in Shandong
Fortune Shop	White Intelligent Power Saver	general	Jingdong prefecture level city in Shandong
Fortune Shop	White Intelligent Power Saver	(of an unmarried couple) be close	Jingdong prefecture level city in Shandong
Fortune Shop	White Intelligent Power Saver	of no use	Jingdong prefecture level city in Shandong
Fortune Shop	White Intelligent Power Saver	Actually can really save electricity, only used half a month to save more than 50, recommended to the neighbors to use together!	Jingdong prefecture level city in Shandong
Fortune Shop	White Intelligent Power Saver	Save what you can. Isn't it more cost effective to buy one of these than to pay the electric bill?	Jingdong prefecture level city in Shandong
Fortune Shop	White Intelligent Power Saver	Received, a use do not know how, use a month to see the effect is good, the effect is good then recommended, not good then I just do not recommend.	Jingdong prefecture level city in Shandong
Fortune Shop	White Intelligent Power Saver	Just received the goods, is a voltage regulator, next month to evaluate whether it is really power saving	Jingdong prefecture level city in Shandong
Fortune Shop	White Intelligent Power Saver	Very good, used a few days obviously feel that the electricity bill is much less, wait a month to see how much can be saved!	Jingdong prefecture level city in Shandong
Fortune Shop	White Intelligent Power Saver	Received the stuff used for more than a month without a little use, it feels like it uses more electricity than before	Jingdong prefecture level city in Shandong
Fortune Shop	White Intelligent Power Saver	The quality is very good, completely exceeded the expectations, the delivery speed is very fast, the packaging is very careful and strict, very satisfied with a shopping	Jingdong prefecture level city in Shandong
Fortune Shop	White Intelligent Power Saver	Fake, doesn't work at all. Don't even sell it.	Jingdong prefecture level city in Shandong
Fortune Shop	White Intelligent Power Saver	This item doesn't seem to have much effect?	Jingdong prefecture level city in Shandong
Fortune Shop	White Intelligent Power Saver	This production is a bit fake, just look at the focus on Ming	Jingdong prefecture level city in Shandong
Fortune Shop	White Intelligent Power Saver	The logistics is fast the service attitude is very good.	Jingdong prefecture level city in Shandong
Fortune Shop	White Intelligent Power Saver	The meter is good quality, sturdy and durable. It is accurate. Easy to use. Saves a lot of things.	Jingdong prefecture level city in Shandong
Fortune Shop	White Intelligent Power Saver	Just received the goods, don't know how it is yet	Jingdong prefecture level city in Shandong
Fortune Shop	White Intelligent Power Saver	Energy saver received, logistics is very fast, the quality is very good, the appearance is also very beautiful power saving is the most important.	Jingdong prefecture level city in Shandong
Fortune Shop	White Intelligent Power Saver	It's good to talk about it, it's not practical to use it.	Jingdong prefecture level city in Shandong
Fortune Shop	White Intelligent Power Saver	I don't know if it's good for now, I'll comment after using it	Jingdong prefecture level city in Shandong
Fortune Shop	White Intelligent Power Saver	I've been using it for two or three days. It's not energy efficient.	Jingdong prefecture level city in Shandong
Fortune Shop	White Intelligent Power Saver	Packaging is intact, logistics is also fast, cost-effective, inexpensive, very satisfied with a purchase	Jingdong prefecture level city in Shandong
Fortune Shop	White Intelligent Power Saver	In use to be observed!	Jingdong prefecture level city in Shandong
Fortune Shop	White Intelligent Power Saver	Use it for a while at a flat rate	Jingdong prefecture level city in Shandong
Fortune Shop	White Intelligent Power Saver	Very trustworthy brand, quality is guaranteed, the price is still affordable, quite satisfied!	Jingdong prefecture level city in Shandong
Fortune Shop	White Intelligent Power Saver	Great quality, great operation, easy to install, great price!	Jingdong prefecture level city in Shandong
Fortune Shop	White Intelligent Power Saver	I came back and installed it, it really saves electricity, much more than before, the	Jingdong prefecture level city in Shandong
Fortune Shop	White Intelligent Power Saver	Fast logistics, good quality, very good packaging, good customer service	Jingdong prefecture level city in Shandong
Fortune Shop	White Intelligent Power Saver	Very good quality, especially good, effective, satisfactory	Jingdong prefecture level city in Shandong
Fortune Shop	White Intelligent Power Saver	I don't know how well it works.	Jingdong prefecture level city in Shandong

Fortune Shop	White Intelligent Power Saver	Good value for money, recommend it haha	Jingdong prefecture level city in Shandong
Fortune Shop	White Intelligent Power Saver	Good. Good. Very good.	Jingdong prefecture level city in Shandong
Fortune Shop	White Intelligent Power Saver	It's not working.	Jingdong prefecture level city in Shandong
Fortune Shop	White Intelligent Power Saver	It's not working.	Jingdong prefecture level city in Shandong
Fortune Shop	White Intelligent Power Saver	In use	Jingdong prefecture level city in Shandong
Fortune Shop	White Intelligent Power Saver	I'll try it later.	Jingdong prefecture level city in Shandong
Fortune Shop	White Intelligent Power Saver	It's not much use.	Jingdong prefecture level city in Shandong
Fortune Shop	White Intelligent Power Saver	It doesn't save electricity.	Jingdong prefecture level city in Shandong
Fortune Shop	White Intelligent Power Saver	It's not much use.	Jingdong prefecture level city in Shandong
Fortune Shop	White Intelligent Power Saver	Tried it for a few days and tracked the record, and there is no difference from before.	Jingdong prefecture level city in Shandong
Fortune Shop	White Intelligent Power Saver	It's not very credible, so just do it yourself and turn off the lights diligently!	Jingdong prefecture level city in Shandong
Fortune Shop	White Intelligent Power Saver	Too bad, no power saving at all, scam people don't fall for it!	Jingdong prefecture level city in Shandong
Fortune Shop	White Intelligent Power Saver	It's not working at all.	Jingdong prefecture level city in Shandong
Fortune Shop	White Intelligent Power Saver	general	Jingdong prefecture level city in Shandong
Fortune Shop	White Intelligent Power Saver	(of an unmarried couple) be close	Jingdong prefecture level city in Shandong
Fortune Shop	White Intelligent Power Saver	of no use	Jingdong prefecture level city in Shandong
Fortune Shop	White Intelligent Power Saver	Actually can really save electricity, only used half a month to save more than 50, recommended to the neighbors to use together!	Jingdong prefecture level city in Shandong
Fortune Shop	White Intelligent Power Saver	Save what you can. Isn't it more cost effective to buy one of these than to pay the electric bill?	Jingdong prefecture level city in Shandong
Fortune Shop	White Intelligent Power Saver	Received, a use do not know how, use a month to see the effect is good, the effect is good then recommended, not good then I just do not recommend.	Jingdong prefecture level city in Shandong
Fortune Shop	White Intelligent Power Saver	Just received the goods, is a voltage regulator, next month to evaluate whether it is really power saving	Jingdong prefecture level city in Shandong
Fortune Shop	White Intelligent Power Saver	Very good, used a few days obviously feel that the electricity bill is much less, wait a month to see how much can be saved!	Jingdong prefecture level city in Shandong
Fortune Shop	White Intelligent Power Saver	Received the stuff used for more than a month without a little use, it feels like it uses more electricity than before	Jingdong prefecture level city in Shandong
Fortune Shop	White Intelligent Power Saver	The quality is very good, completely exceeded the expectations, the delivery speed is very fast, the packaging is very careful and strict, very satisfied with a shopping	Jingdong prefecture level city in Shandong
Fortune Shop	White Intelligent Power Saver	Fake, doesn't work at all. Don't even sell it.	Jingdong prefecture level city in Shandong
Fortune Shop	White Intelligent Power Saver	This item doesn't seem to have much effect?	Jingdong prefecture level city in Shandong
Fortune Shop	White Intelligent Power Saver	This production is a bit fake, just look at the focus on Ming	Jingdong prefecture level city in Shandong
Fortune Shop	White Intelligent Power Saver	The logistics is fast the service attitude is very good.	Jingdong prefecture level city in Shandong
Fortune Shop	White Intelligent Power Saver	The meter is good quality, sturdy and durable. It is accurate. Easy to use. Saves a lot of things.	Jingdong prefecture level city in Shandong
Fortune Shop	White Intelligent Power Saver	Just received the goods, don't know how it is yet	Jingdong prefecture level city in Shandong
Fortune Shop	White Intelligent Power Saver	Energy saver received, logistics is very fast, the quality is very good, the appearance is also very beautiful power saving is the most important.	Jingdong prefecture level city in Shandong
Fortune Shop	White Intelligent Power Saver	It's good to talk about it, it's not practical to use it.	Jingdong prefecture level city in Shandong
Fortune Shop	White Intelligent Power Saver	I don't know if it's good for now, I'll comment after using it	Jingdong prefecture level city in Shandong
Fortune Shop	White Intelligent Power Saver	I've been using it for two or three days. It's not energy efficient.	Jingdong prefecture level city in Shandong
Fortune Shop	White Intelligent Power Saver	Packaging is intact, logistics is also fast, cost-effective, inexpensive, very satisfied with a purchase	Jingdong prefecture level city in Shandong
Fortune Shop	White Intelligent Power Saver	In use to be observed!	Jingdong prefecture level city in Shandong
Fortune Shop	White Intelligent Power Saver	Use it for a while at a flat rate	Jingdong prefecture level city in Shandong
Fortune Shop	White Intelligent Power Saver	Very trustworthy brand, quality is guaranteed, the price is still affordable, quite satisfied!	Jingdong prefecture level city in Shandong
Fortune Shop	White Intelligent Power Saver	Great quality, great operation, easy to install, great price!	Jingdong prefecture level city in Shandong
Fortune Shop	White Intelligent Power Saver	I came back and installed it, it really saves electricity, much more than before, the	Jingdong prefecture level city in Shandong
Fortune Shop	White Intelligent Power Saver	Fast logistics, good quality, very good packaging, good customer service	Jingdong prefecture level city in Shandong
Fortune Shop	White Intelligent Power Saver	Very good quality, especially good, effective, satisfactory	Jingdong prefecture level city in Shandong
Fortune Shop	White Intelligent Power Saver	I don't know how well it works.	Jingdong prefecture level city in Shandong
Fortune Shop	White Intelligent Power Saver	Good value for money, recommend it haha	Jingdong prefecture level city in Shandong
Fortune Shop	White Intelligent Power Saver	Good. Good. Very good.	Jingdong prefecture level city in Shandong
Fortune Shop	White Intelligent Power Saver	It's not working.	Jingdong prefecture level city in Shandong
Fortune Shop	White Intelligent Power Saver	It's not working.	Jingdong prefecture level city in Shandong
Fortune Shop	White Intelligent Power Saver	In use	Jingdong prefecture level city in Shandong
Fortune Shop	White Intelligent Power Saver	I'll try it later.	Jingdong prefecture level city in Shandong
Fortune Shop	White Intelligent Power Saver	It's not much use.	Jingdong prefecture level city in Shandong
Fortune Shop	White Intelligent Power Saver	It doesn't save electricity.	Jingdong prefecture level city in Shandong
Fortune Shop	White Intelligent Power Saver	It's not much use.	Jingdong prefecture level city in Shandong

Fortune Shop	White Intelligent Power Saver	Tried it for a few days and tracked the record, and there is no difference from before.	Jingdong prefecture level city in Shandong
Fortune Shop	White Intelligent Power Saver	It's not very credible, so just do it yourself and turn off the lights diligently!	Jingdong prefecture level city in Shandong
Fortune Shop	White Intelligent Power Saver	Too bad, no power saving at all, scam people don't fall for it!	Jingdong prefecture level city in Shandong
Fortune Shop	White Intelligent Power Saver	It's not working at all.	Jingdong prefecture level city in Shandong
Fortune Shop	White Intelligent Power Saver	general	Jingdong prefecture level city in Shandong
Fortune Shop	White Intelligent Power Saver	(of an unmarried couple) be close	Jingdong prefecture level city in Shandong
Fortune Shop	White Intelligent Power Saver	of no use	Jingdong prefecture level city in Shandong
Fortune Shop	White Intelligent Power Saver	Actually can really save electricity, only used half a month to save more than 50, recommended to the neighbors to use together!	Jingdong prefecture level city in Shandong
Fortune Shop	White Intelligent Power Saver	Save what you can. Isn't it more cost effective to buy one of these than to pay the electric bill?	Jingdong prefecture level city in Shandong
Fortune Shop	White Intelligent Power Saver	Received, a use do not know how, use a month to see the effect is good, the effect is good then recommended, not good then I just do not recommend.	Jingdong prefecture level city in Shandong
Fortune Shop	White Intelligent Power Saver	Just received the goods, is a voltage regulator, next month to evaluate whether it is really power saving	Jingdong prefecture level city in Shandong
Fortune Shop	White Intelligent Power Saver	Very good, used a few days obviously feel that the electricity bill is much less, wait a month to see how much can be saved!	Jingdong prefecture level city in Shandong
Fortune Shop	White Intelligent Power Saver	Received the stuff used for more than a month without a little use, it feels like it uses more electricity than before	Jingdong prefecture level city in Shandong
Fortune Shop	White Intelligent Power Saver	The quality is very good, completely exceeded the expectations, the delivery speed is very fast, the packaging is very careful and strict, very satisfied with a shopping	Jingdong prefecture level city in Shandong
Fortune Shop	White Intelligent Power Saver	Fake, doesn't work at all. Don't even sell it.	Jingdong prefecture level city in Shandong
Fortune Shop	White Intelligent Power Saver	This item doesn't seem to have much effect?	Jingdong prefecture level city in Shandong
Fortune Shop	White Intelligent Power Saver	This production is a bit fake, just look at the focus on Ming	Jingdong prefecture level city in Shandong
Fortune Shop	White Intelligent Power Saver	The logistics is fast the service attitude is very good.	Jingdong prefecture level city in Shandong
Fortune Shop	White Intelligent Power Saver	The meter is good quality, sturdy and durable. It is accurate. Easy to use. Saves a lot of things.	Jingdong prefecture level city in Shandong
Fortune Shop	White Intelligent Power Saver	Just received the goods, don't know how it is yet	Jingdong prefecture level city in Shandong
Fortune Shop	White Intelligent Power Saver	Energy saver received, logistics is very fast, the quality is very good, the appearance is also very beautiful power saving is the most important.	Jingdong prefecture level city in Shandong
Fortune Shop	White Intelligent Power Saver	It's good to talk about it, it's not practical to use it.	Jingdong prefecture level city in Shandong
Fortune Shop	White Intelligent Power Saver	I don't know if it's good for now, I'll comment after using it	Jingdong prefecture level city in Shandong
Fortune Shop	White Intelligent Power Saver	I've been using it for two or three days. It's not energy efficient.	Jingdong prefecture level city in Shandong
Fortune Shop	White Intelligent Power Saver	Packaging is intact, logistics is also fast, cost-effective, inexpensive, very satisfied with a purchase	Jingdong prefecture level city in Shandong
Fortune Shop	White Intelligent Power Saver	In use to be observed!	Jingdong prefecture level city in Shandong
Fortune Shop	White Intelligent Power Saver	Use it for a while at a flat rate	Jingdong prefecture level city in Shandong
Fortune Shop	White Intelligent Power Saver	Very trustworthy brand, quality is guaranteed, the price is still affordable, quite satisfied!	Jingdong prefecture level city in Shandong
Fortune Shop	White Intelligent Power Saver	Great quality, great operation, easy to install, great price!	Jingdong prefecture level city in Shandong
Fortune Shop	White Intelligent Power Saver	I came back and installed it, it really saves electricity, much more than before, the	Jingdong prefecture level city in Shandong
Fortune Shop	White Intelligent Power Saver	Fast logistics, good quality, very good packaging, good customer service	Jingdong prefecture level city in Shandong
Fortune Shop	White Intelligent Power Saver	Very good quality, especially good, effective, satisfactory	Jingdong prefecture level city in Shandong
Fortune Shop	White Intelligent Power Saver	I don't know how well it works.	Jingdong prefecture level city in Shandong
Fortune Shop	White Intelligent Power Saver	Good value for money, recommend it haha	Jingdong prefecture level city in Shandong
Fortune Shop	White Intelligent Power Saver	Good. Good. Very good.	Jingdong prefecture level city in Shandong
Fortune Shop	White Intelligent Power Saver	It's not working.	Jingdong prefecture level city in Shandong
Fortune Shop	White Intelligent Power Saver	It's not working.	Jingdong prefecture level city in Shandong
Fortune Shop	White Intelligent Power Saver	In use	Jingdong prefecture level city in Shandong
Fortune Shop	White Intelligent Power Saver	I'll try it later.	Jingdong prefecture level city in Shandong
Fortune Shop	White Intelligent Power Saver	It's not much use.	Jingdong prefecture level city in Shandong
Fortune Shop	White Intelligent Power Saver	It doesn't save electricity.	Jingdong prefecture level city in Shandong
Fortune Shop	White Intelligent Power Saver	It's not much use.	Jingdong prefecture level city in Shandong
Fortune Shop	White Intelligent Power Saver	Tried it for a few days and tracked the record, and there is no difference from before.	Jingdong prefecture level city in Shandong
Fortune Shop	White Intelligent Power Saver	It's not very credible, so just do it yourself and turn off the lights diligently!	Jingdong prefecture level city in Shandong
Fortune Shop	White Intelligent Power Saver	Too bad, no power saving at all, scam people don't fall for it!	Jingdong prefecture level city in Shandong
Fortune Shop	White Intelligent Power Saver	It's not working at all.	Jingdong prefecture level city in Shandong
Fortune Shop	White Intelligent Power Saver	general	Jingdong prefecture level city in Shandong
Fortune Shop	White Intelligent Power Saver	(of an unmarried couple) be close	Jingdong prefecture level city in Shandong
Fortune Shop	White Intelligent Power Saver	of no use	Jingdong prefecture level city in Shandong
Fortune Shop	White Intelligent Power Saver	Actually can really save electricity, only used half a month to save more than 50, recommended to the neighbors to use together!	Jingdong prefecture level city in Shandong
Fortune Shop	White Intelligent Power Saver	Save what you can. Isn't it more cost effective to buy one of these than to pay the electric bill?	Jingdong prefecture level city in Shandong

Fortune Shop	White Intelligent Power Saver	Received, a use do not know how, use a month to see the effect is good, the effect is good then recommended, not good then I just do not recommend.	Jingdong prefecture level city in Shandong
Fortune Shop	White Intelligent Power Saver	Just received the goods, is a voltage regulator, next month to evaluate whether it is really power saving	Jingdong prefecture level city in Shandong
Fortune Shop	White Intelligent Power Saver	Very good, used a few days obviously feel that the electricity bill is much less, wait a month to see how much can be saved!	Jingdong prefecture level city in Shandong
Fortune Shop	White Intelligent Power Saver	Received the stuff used for more than a month without a little use, it feels like it uses more electricity than before	Jingdong prefecture level city in Shandong
Fortune Shop	White Intelligent Power Saver	The quality is very good, completely exceeded the expectations, the delivery speed is very fast, the packaging is very careful and strict, very satisfied with a shopping	Jingdong prefecture level city in Shandong
Fortune Shop	White Intelligent Power Saver	Fake, doesn't work at all. Don't even sell it.	Jingdong prefecture level city in Shandong
Fortune Shop	White Intelligent Power Saver	This item doesn't seem to have much effect?	Jingdong prefecture level city in Shandong
Fortune Shop	White Intelligent Power Saver	This production is a bit fake, just look at the focus on Ming	Jingdong prefecture level city in Shandong
Fortune Shop	White Intelligent Power Saver	The logistics is fast the service attitude is very good.	Jingdong prefecture level city in Shandong
Fortune Shop	White Intelligent Power Saver	The meter is good quality, sturdy and durable. It is accurate. Easy to use. Saves a lot of things.	Jingdong prefecture level city in Shandong
Fortune Shop	White Intelligent Power Saver	Just received the goods, don't know how it is yet	Jingdong prefecture level city in Shandong
Fortune Shop	White Intelligent Power Saver	Energy saver received, logistics is very fast, the quality is very good, the appearance is also very beautiful power saving is the most important.	Jingdong prefecture level city in Shandong
Fortune Shop	White Intelligent Power Saver	It's good to talk about it, it's not practical to use it.	Jingdong prefecture level city in Shandong
Fortune Shop	White Intelligent Power Saver	I don't know if it's good for now, I'll comment after using it	Jingdong prefecture level city in Shandong
Fortune Shop	White Intelligent Power Saver	I've been using it for two or three days. It's not energy efficient.	Jingdong prefecture level city in Shandong
Fortune Shop	White Intelligent Power Saver	Packaging is intact, logistics is also fast, cost-effective, inexpensive, very satisfied with a purchase	Jingdong prefecture level city in Shandong
Fortune Shop	White Intelligent Power Saver	In use to be observed!	Jingdong prefecture level city in Shandong
Fortune Shop	White Intelligent Power Saver	Use it for a while at a flat rate	Jingdong prefecture level city in Shandong
Fortune Shop	White Intelligent Power Saver	Very trustworthy brand, quality is guaranteed, the price is still affordable, quite satisfied!	Jingdong prefecture level city in Shandong
Fortune Shop	White Intelligent Power Saver	Great quality, great operation, easy to install, great price!	Jingdong prefecture level city in Shandong
Fortune Shop	White Intelligent Power Saver	I came back and installed it, it really saves electricity, much more than before, the	Jingdong prefecture level city in Shandong
Fortune Shop	White Intelligent Power Saver	Fast logistics, good quality, very good packaging, good customer service	Jingdong prefecture level city in Shandong
Fortune Shop	White Intelligent Power Saver	Very good quality, especially good, effective, satisfactory	Jingdong prefecture level city in Shandong
Fortune Shop	White Intelligent Power Saver	I don't know how well it works.	Jingdong prefecture level city in Shandong
Fortune Shop	White Intelligent Power Saver	Good value for money, recommend it haha	Jingdong prefecture level city in Shandong
Fortune Shop	White Intelligent Power Saver	Good. Good. Very good.	Jingdong prefecture level city in Shandong
Fortune Shop	White Intelligent Power Saver	It's not working.	Jingdong prefecture level city in Shandong
Fortune Shop	White Intelligent Power Saver	It's not working.	Jingdong prefecture level city in Shandong
Fortune Shop	White Intelligent Power Saver	In use	Jingdong prefecture level city in Shandong
Fortune Shop	White Intelligent Power Saver	I'll try it later.	Jingdong prefecture level city in Shandong
Fortune Shop	White Intelligent Power Saver	It's not much use.	Jingdong prefecture level city in Shandong
Fortune Shop	White Intelligent Power Saver	It doesn't save electricity.	Jingdong prefecture level city in Shandong
Fortune Shop	White Intelligent Power Saver	It's not much use.	Jingdong prefecture level city in Shandong
Fortune Shop	White Intelligent Power Saver	Tried it for a few days and tracked the record, and there is no difference from before.	Jingdong prefecture level city in Shandong
Fortune Shop	White Intelligent Power Saver	It's not very credible, so just do it yourself and turn off the lights diligently!	Jingdong prefecture level city in Shandong
Fortune Shop	White Intelligent Power Saver	Too bad, no power saving at all, scam people don't fall for it!	Jingdong prefecture level city in Shandong
Fortune Shop	White Intelligent Power Saver	It's not working at all.	Jingdong prefecture level city in Shandong
Fortune Shop	White Intelligent Power Saver	general	Jingdong prefecture level city in Shandong
Fortune Shop	White Intelligent Power Saver	(of an unmarried couple) be close	Jingdong prefecture level city in Shandong
Fortune Shop	White Intelligent Power Saver	of no use	Jingdong prefecture level city in Shandong
Fortune Shop	White Intelligent Power Saver	Actually can really save electricity, only used half a month to save more than 50, recommended to the neighbors to use together!	Jingdong prefecture level city in Shandong
Fortune Shop	White Intelligent Power Saver	Save what you can. Isn't it more cost effective to buy one of these than to pay the electric bill?	Jingdong prefecture level city in Shandong
Fortune Shop	White Intelligent Power Saver	Received, a use do not know how, use a month to see the effect is good, the effect is good then recommended, not good then I just do not recommend.	Jingdong prefecture level city in Shandong
Fortune Shop	White Intelligent Power Saver	Just received the goods, is a voltage regulator, next month to evaluate whether it is really power saving	Jingdong prefecture level city in Shandong
Fortune Shop	White Intelligent Power Saver	Very good, used a few days obviously feel that the electricity bill is much less, wait a month to see how much can be saved!	Jingdong prefecture level city in Shandong
Fortune Shop	White Intelligent Power Saver	Received the stuff used for more than a month without a little use, it feels like it uses more electricity than before	Jingdong prefecture level city in Shandong
Fortune Shop	White Intelligent Power Saver	The quality is very good, completely exceeded the expectations, the delivery speed is very fast, the packaging is very careful and strict, very satisfied with a shopping	Jingdong prefecture level city in Shandong
Fortune Shop	White Intelligent Power Saver	Fake, doesn't work at all. Don't even sell it.	Jingdong prefecture level city in Shandong
Fortune Shop	White Intelligent Power Saver	This item doesn't seem to have much effect?	Jingdong prefecture level city in Shandong
Fortune Shop	White Intelligent Power Saver	This production is a bit fake, just look at the focus on Ming	Jingdong prefecture level city in Shandong
Fortune Shop	White Intelligent Power Saver	The logistics is fast the service attitude is very good.	Jingdong prefecture level city in Shandong

Fortune Shop	White Intelligent Power Saver	The meter is good quality, sturdy and durable. It is accurate. Easy to use. Saves a lot of things.	Jingdong prefecture level city in Shandong
Fortune Shop	White Intelligent Power Saver	Just received the goods, don't know how it is yet	Jingdong prefecture level city in Shandong
Fortune Shop	White Intelligent Power Saver	Energy saver received, logistics is very fast, the quality is very good, the appearance is also very beautiful power saving is the most important.	Jingdong prefecture level city in Shandong
Fortune Shop	White Intelligent Power Saver	It's good to talk about it, it's not practical to use it.	Jingdong prefecture level city in Shandong
Fortune Shop	White Intelligent Power Saver	I don't know if it's good for now, I'll comment after using it	Jingdong prefecture level city in Shandong
Fortune Shop	White Intelligent Power Saver	I've been using it for two or three days. It's not energy efficient.	Jingdong prefecture level city in Shandong
Fortune Shop	White Intelligent Power Saver	Packaging is intact, logistics is also fast, cost-effective, inexpensive, very satisfied with a purchase	Jingdong prefecture level city in Shandong
Fortune Shop	White Intelligent Power Saver	In use to be observed!	Jingdong prefecture level city in Shandong
Fortune Shop	White Intelligent Power Saver	Use it for a while at a flat rate	Jingdong prefecture level city in Shandong
Fortune Shop	White Intelligent Power Saver	Very trustworthy brand, quality is guaranteed, the price is still affordable, quite satisfied!	Jingdong prefecture level city in Shandong
Fortune Shop	White Intelligent Power Saver	Great quality, great operation, easy to install, great price!	Jingdong prefecture level city in Shandong
Fortune Shop	White Intelligent Power Saver	I came back and installed it, it really saves electricity, much more than before, the	Jingdong prefecture level city in Shandong
Fortune Shop	White Intelligent Power Saver	Fast logistics, good quality, very good packaging, good customer service	Jingdong prefecture level city in Shandong
Fortune Shop	White Intelligent Power Saver	Very good quality, especially good, effective, satisfactory	Jingdong prefecture level city in Shandong
Fortune Shop	White Intelligent Power Saver	I don't know how well it works.	Jingdong prefecture level city in Shandong
Fortune Shop	White Intelligent Power Saver	Good value for money, recommend it haha	Jingdong prefecture level city in Shandong
Fortune Shop	White Intelligent Power Saver	Good. Good. Very good.	Jingdong prefecture level city in Shandong
Fortune Shop	White Intelligent Power Saver	It's not working.	Jingdong prefecture level city in Shandong
Fortune Shop	White Intelligent Power Saver	It's not working.	Jingdong prefecture level city in Shandong
Fortune Shop	White Intelligent Power Saver	In use	Jingdong prefecture level city in Shandong
Fortune Shop	White Intelligent Power Saver	I'll try it later.	Jingdong prefecture level city in Shandong
Fortune Shop	White Intelligent Power Saver	It's not much use.	Jingdong prefecture level city in Shandong
Fortune Shop	White Intelligent Power Saver	It doesn't save electricity.	Jingdong prefecture level city in Shandong
Fortune Shop	White Intelligent Power Saver	It's not much use.	Jingdong prefecture level city in Shandong
Fortune Shop	White Intelligent Power Saver	Tried it for a few days and tracked the record, and there is no difference from before.	Jingdong prefecture level city in Shandong
Fortune Shop	White Intelligent Power Saver	It's not very credible, so just do it yourself and turn off the lights diligently!	Jingdong prefecture level city in Shandong
Fortune Shop	White Intelligent Power Saver	Too bad, no power saving at all, scam people don't fall for it!	Jingdong prefecture level city in Shandong
Fortune Shop	White Intelligent Power Saver	It's not working at all.	Jingdong prefecture level city in Shandong
Fortune Shop	White Intelligent Power Saver	general	Jingdong prefecture level city in Shandong
Fortune Shop	White Intelligent Power Saver	(of an unmarried couple) be close	Jingdong prefecture level city in Shandong
Fortune Shop	White Intelligent Power Saver	of no use	Jingdong prefecture level city in Shandong
Fortune Shop	White Intelligent Power Saver	Actually can really save electricity, only used half a month to save more than 50, recommended to the neighbors to use together!	Jingdong prefecture level city in Shandong
Fortune Shop	White Intelligent Power Saver	Save what you can. Isn't it more cost effective to buy one of these than to pay the electric bill?	Jingdong prefecture level city in Shandong
Fortune Shop	White Intelligent Power Saver	Received, a use do not know how, use a month to see the effect is good, the effect is good then recommended, not good then I just do not recommend.	Jingdong prefecture level city in Shandong
Fortune Shop	White Intelligent Power Saver	Just received the goods, is a voltage regulator, next month to evaluate whether it is really power saving	Jingdong prefecture level city in Shandong
Fortune Shop	White Intelligent Power Saver	Very good, used a few days obviously feel that the electricity bill is much less, wait a month to see how much can be saved!	Jingdong prefecture level city in Shandong
Fortune Shop	White Intelligent Power Saver	Received the stuff used for more than a month without a little use, it feels like it uses more electricity than before	Jingdong prefecture level city in Shandong
Fortune Shop	White Intelligent Power Saver	The quality is very good, completely exceeded the expectations, the delivery speed is very fast, the packaging is very careful and strict, very satisfied with a shopping	Jingdong prefecture level city in Shandong
Fortune Shop	White Intelligent Power Saver	Fake, doesn't work at all. Don't even sell it.	Jingdong prefecture level city in Shandong
Fortune Shop	White Intelligent Power Saver	This item doesn't seem to have much effect?	Jingdong prefecture level city in Shandong
Fortune Shop	White Intelligent Power Saver	This production is a bit fake, just look at the focus on Ming	Jingdong prefecture level city in Shandong
Fortune Shop	White Intelligent Power Saver	The logistics is fast the service attitude is very good.	Jingdong prefecture level city in Shandong
Fortune Shop	White Intelligent Power Saver	The meter is good quality, sturdy and durable. It is accurate. Easy to use. Saves a lot of things.	Jingdong prefecture level city in Shandong
Fortune Shop	White Intelligent Power Saver	Just received the goods, don't know how it is yet	Jingdong prefecture level city in Shandong
Fortune Shop	White Intelligent Power Saver	Energy saver received, logistics is very fast, the quality is very good, the appearance is also very beautiful power saving is the most important.	Jingdong prefecture level city in Shandong
Fortune Shop	White Intelligent Power Saver	It's good to talk about it, it's not practical to use it.	Jingdong prefecture level city in Shandong
Fortune Shop	White Intelligent Power Saver	I don't know if it's good for now, I'll comment after using it	Jingdong prefecture level city in Shandong
Fortune Shop	White Intelligent Power Saver	I've been using it for two or three days. It's not energy efficient.	Jingdong prefecture level city in Shandong
Fortune Shop	White Intelligent Power Saver	Packaging is intact, logistics is also fast, cost-effective, inexpensive, very satisfied with a purchase	Jingdong prefecture level city in Shandong
Fortune Shop	White Intelligent Power Saver	In use to be observed!	Jingdong prefecture level city in Shandong
Fortune Shop	White Intelligent Power Saver	Use it for a while at a flat rate	Jingdong prefecture level city in Shandong

Fortune Shop	White Intelligent Power Saver	Very trustworthy brand, quality is guaranteed, the price is still affordable, quite satisfied!	Jingdong prefecture level city in Shandong
Fortune Shop	White Intelligent Power Saver	Great quality, great operation, easy to install, great price!	Jingdong prefecture level city in Shandong
Fortune Shop	White Intelligent Power Saver	I came back and installed it, it really saves electricity, much more than before, the	Jingdong prefecture level city in Shandong
Fortune Shop	White Intelligent Power Saver	Fast logistics, good quality, very good packaging, good customer service	Jingdong prefecture level city in Shandong
Fortune Shop	White Intelligent Power Saver	Very good quality, especially good, effective, satisfactory	Jingdong prefecture level city in Shandong
Fortune Shop	White Intelligent Power Saver	I don't know how well it works.	Jingdong prefecture level city in Shandong
Fortune Shop	White Intelligent Power Saver	Good value for money, recommend it haha	Jingdong prefecture level city in Shandong
Fortune Shop	White Intelligent Power Saver	Good. Good. Very good.	Jingdong prefecture level city in Shandong
Fortune Shop	White Intelligent Power Saver	It's not working.	Jingdong prefecture level city in Shandong
Fortune Shop	White Intelligent Power Saver	It's not working.	Jingdong prefecture level city in Shandong
Fortune Shop	White Intelligent Power Saver	In use	Jingdong prefecture level city in Shandong
Fortune Shop	White Intelligent Power Saver	I'll try it later.	Jingdong prefecture level city in Shandong
Fortune Shop	White Intelligent Power Saver	It's not much use.	Jingdong prefecture level city in Shandong
Fortune Shop	White Intelligent Power Saver	It doesn't save electricity.	Jingdong prefecture level city in Shandong
Fortune Shop	White Intelligent Power Saver	It's not much use.	Jingdong prefecture level city in Shandong
Fortune Shop	White Intelligent Power Saver	Tried it for a few days and tracked the record, and there is no difference from before.	Jingdong prefecture level city in Shandong
Fortune Shop	White Intelligent Power Saver	It's not very credible, so just do it yourself and turn off the lights diligently!	Jingdong prefecture level city in Shandong
Fortune Shop	White Intelligent Power Saver	Too bad, no power saving at all, scam people don't fall for it!	Jingdong prefecture level city in Shandong
Fortune Shop	White Intelligent Power Saver	It's not working at all.	Jingdong prefecture level city in Shandong
Fortune Shop	White Intelligent Power Saver	general	Jingdong prefecture level city in Shandong
Fortune Shop	White Intelligent Power Saver	(of an unmarried couple) be close	Jingdong prefecture level city in Shandong
Fortune Shop	White Intelligent Power Saver	of no use	Jingdong prefecture level city in Shandong
Fortune Shop	White Intelligent Power Saver	Actually can really save electricity, only used half a month to save more than 50, recommended to the neighbors to use together!	Jingdong prefecture level city in Shandong
Fortune Shop	White Intelligent Power Saver	Save what you can. Isn't it more cost effective to buy one of these than to pay the electric bill?	Jingdong prefecture level city in Shandong
Fortune Shop	White Intelligent Power Saver	Received, a use do not know how, use a month to see the effect is good, the effect is good then recommended, not good then I just do not recommend.	Jingdong prefecture level city in Shandong
Fortune Shop	White Intelligent Power Saver	Just received the goods, is a voltage regulator, next month to evaluate whether it is really power saving	Jingdong prefecture level city in Shandong
Fortune Shop	White Intelligent Power Saver	Very good, used a few days obviously feel that the electricity bill is much less, wait a month to see how much can be saved!	Jingdong prefecture level city in Shandong
Fortune Shop	White Intelligent Power Saver	Received the stuff used for more than a month without a little use, it feels like it uses more electricity than before	Jingdong prefecture level city in Shandong
Fortune Shop	White Intelligent Power Saver	The quality is very good, completely exceeded the expectations, the delivery speed is very fast, the packaging is very careful and strict, very satisfied with a shopping	Jingdong prefecture level city in Shandong
Fortune Shop	White Intelligent Power Saver	Fake, doesn't work at all. Don't even sell it.	Jingdong prefecture level city in Shandong
Fortune Shop	White Intelligent Power Saver	This item doesn't seem to have much effect?	Jingdong prefecture level city in Shandong
Fortune Shop	White Intelligent Power Saver	This production is a bit fake, just look at the focus on Ming	Jingdong prefecture level city in Shandong
Fortune Shop	White Intelligent Power Saver	The logistics is fast the service attitude is very good.	Jingdong prefecture level city in Shandong
Fortune Shop	White Intelligent Power Saver	The meter is good quality, sturdy and durable. It is accurate. Easy to use. Saves a lot of things.	Jingdong prefecture level city in Shandong
Fortune Shop	White Intelligent Power Saver	Just received the goods, don't know how it is yet	Jingdong prefecture level city in Shandong
Fortune Shop	White Intelligent Power Saver	Energy saver received, logistics is very fast, the quality is very good, the appearance is also very beautiful power saving is the most important.	Jingdong prefecture level city in Shandong
Fortune Shop	White Intelligent Power Saver	It's good to talk about it, it's not practical to use it.	Jingdong prefecture level city in Shandong
Fortune Shop	White Intelligent Power Saver	I don't know if it's good for now, I'll comment after using it	Jingdong prefecture level city in Shandong
Fortune Shop	White Intelligent Power Saver	I've been using it for two or three days. It's not energy efficient.	Jingdong prefecture level city in Shandong
Fortune Shop	White Intelligent Power Saver	Packaging is intact, logistics is also fast, cost-effective, inexpensive, very satisfied with a purchase	Jingdong prefecture level city in Shandong
Fortune Shop	White Intelligent Power Saver	In use to be observed!	Jingdong prefecture level city in Shandong
Fortune Shop	White Intelligent Power Saver	Use it for a while at a flat rate	Jingdong prefecture level city in Shandong
Fortune Shop	White Intelligent Power Saver	Very trustworthy brand, quality is guaranteed, the price is still affordable, quite satisfied!	Jingdong prefecture level city in Shandong
Fortune Shop	White Intelligent Power Saver	Great quality, great operation, easy to install, great price!	Jingdong prefecture level city in Shandong
Fortune Shop	White Intelligent Power Saver	I came back and installed it, it really saves electricity, much more than before, the	Jingdong prefecture level city in Shandong
Fortune Shop	White Intelligent Power Saver	Fast logistics, good quality, very good packaging, good customer service	Jingdong prefecture level city in Shandong
Fortune Shop	White Intelligent Power Saver	Very good quality, especially good, effective, satisfactory	Jingdong prefecture level city in Shandong
Fortune Shop	White Intelligent Power Saver	I don't know how well it works.	Jingdong prefecture level city in Shandong
Fortune Shop	White Intelligent Power Saver	Good value for money, recommend it haha	Jingdong prefecture level city in Shandong
Fortune Shop	White Intelligent Power Saver	Good. Good. Very good.	Jingdong prefecture level city in Shandong
Fortune Shop	White Intelligent Power Saver	It's not working.	Jingdong prefecture level city in Shandong

Fortune Shop	White Intelligent Power Saver	It's not working.	Jingdong prefecture level city in Shandong
Fortune Shop	White Intelligent Power Saver	In use	Jingdong prefecture level city in Shandong
Fortune Shop	White Intelligent Power Saver	I'll try it later.	Jingdong prefecture level city in Shandong
Fortune Shop	White Intelligent Power Saver	It's not much use.	Jingdong prefecture level city in Shandong
Fortune Shop	White Intelligent Power Saver	It doesn't save electricity.	Jingdong prefecture level city in Shandong
Fortune Shop	White Intelligent Power Saver	It's not much use.	Jingdong prefecture level city in Shandong
Fortune Shop	White Intelligent Power Saver	Tried it for a few days and tracked the record, and there is no difference from before.	Jingdong prefecture level city in Shandong
Fortune Shop	White Intelligent Power Saver	It's not very credible, so just do it yourself and turn off the lights diligently!	Jingdong prefecture level city in Shandong
Fortune Shop	White Intelligent Power Saver	Too bad, no power saving at all, scam people don't fall for it!	Jingdong prefecture level city in Shandong
Fortune Shop	White Intelligent Power Saver	It's not working at all.	Jingdong prefecture level city in Shandong
Fortune Shop	White Intelligent Power Saver	general	Jingdong prefecture level city in Shandong
Fortune Shop	White Intelligent Power Saver	(of an unmarried couple) be close	Jingdong prefecture level city in Shandong
Fortune Shop	White Intelligent Power Saver	of no use	Jingdong prefecture level city in Shandong
Fortune Shop	White Intelligent Power Saver	Actually can really save electricity, only used half a month to save more than 50, recommended to the neighbors to use together!	Jingdong prefecture level city in Shandong
Fortune Shop	White Intelligent Power Saver	Save what you can. Isn't it more cost effective to buy one of these than to pay the electric bill?	Jingdong prefecture level city in Shandong
Fortune Shop	White Intelligent Power Saver	Received, a use do not know how, use a month to see the effect is good, the effect is good then recommended, not good then I just do not recommend.	Jingdong prefecture level city in Shandong
Fortune Shop	White Intelligent Power Saver	Just received the goods, is a voltage regulator, next month to evaluate whether it is really power saving	Jingdong prefecture level city in Shandong
Fortune Shop	White Intelligent Power Saver	Very good, used a few days obviously feel that the electricity bill is much less, wait a month to see how much can be saved!	Jingdong prefecture level city in Shandong
Fortune Shop	White Intelligent Power Saver	Received the stuff used for more than a month without a little use, it feels like it uses more electricity than before	Jingdong prefecture level city in Shandong
Fortune Shop	White Intelligent Power Saver	The quality is very good, completely exceeded the expectations, the delivery speed is very fast, the packaging is very careful and strict, very satisfied with a shopping	Jingdong prefecture level city in Shandong
Fortune Shop	White Intelligent Power Saver	Fake, doesn't work at all. Don't even sell it.	Jingdong prefecture level city in Shandong
Fortune Shop	White Intelligent Power Saver	This item doesn't seem to have much effect?	Jingdong prefecture level city in Shandong
Fortune Shop	White Intelligent Power Saver	This production is a bit fake, just look at the focus on Ming	Jingdong prefecture level city in Shandong
Fortune Shop	White Intelligent Power Saver	The logistics is fast the service attitude is very good.	Jingdong prefecture level city in Shandong
Fortune Shop	White Intelligent Power Saver	The meter is good quality, sturdy and durable. It is accurate. Easy to use. Saves a lot of things.	Jingdong prefecture level city in Shandong
Fortune Shop	White Intelligent Power Saver	Just received the goods, don't know how it is yet	Jingdong prefecture level city in Shandong
Fortune Shop	White Intelligent Power Saver	Energy saver received, logistics is very fast, the quality is very good, the appearance is also very beautiful power saving is the most important.	Jingdong prefecture level city in Shandong
Fortune Shop	White Intelligent Power Saver	It's good to talk about it, it's not practical to use it.	Jingdong prefecture level city in Shandong
Fortune Shop	White Intelligent Power Saver	I don't know if it's good for now, I'll comment after using it	Jingdong prefecture level city in Shandong
Fortune Shop	White Intelligent Power Saver	I've been using it for two or three days. It's not energy efficient.	Jingdong prefecture level city in Shandong
Fortune Shop	White Intelligent Power Saver	Packaging is intact, logistics is also fast, cost-effective, inexpensive, very satisfied with a purchase	Jingdong prefecture level city in Shandong
Fortune Shop	White Intelligent Power Saver	In use to be observed!	Jingdong prefecture level city in Shandong
Fortune Shop	White Intelligent Power Saver	Use it for a while at a flat rate	Jingdong prefecture level city in Shandong
Fortune Shop	White Intelligent Power Saver	Very trustworthy brand, quality is guaranteed, the price is still affordable, quite satisfied!	Jingdong prefecture level city in Shandong
Fortune Shop	White Intelligent Power Saver	Great quality, great operation, easy to install, great price!	Jingdong prefecture level city in Shandong
Fortune Shop	White Intelligent Power Saver	I came back and installed it, it really saves electricity, much more than before, the	Jingdong prefecture level city in Shandong
Fortune Shop	White Intelligent Power Saver	Fast logistics, good quality, very good packaging, good customer service	Jingdong prefecture level city in Shandong
Fortune Shop	White Intelligent Power Saver	Very good quality, especially good, effective, satisfactory	Jingdong prefecture level city in Shandong
Fortune Shop	White Intelligent Power Saver	I don't know how well it works.	Jingdong prefecture level city in Shandong
Fortune Shop	White Intelligent Power Saver	Good value for money, recommend it haha	Jingdong prefecture level city in Shandong
Fortune Shop	White Intelligent Power Saver	Good. Good. Very good.	Jingdong prefecture level city in Shandong
Fortune Shop	White Intelligent Power Saver	It's not working.	Jingdong prefecture level city in Shandong
Fortune Shop	White Intelligent Power Saver	It's not working.	Jingdong prefecture level city in Shandong
Fortune Shop	White Intelligent Power Saver	In use	Jingdong prefecture level city in Shandong
Fortune Shop	White Intelligent Power Saver	I'll try it later.	Jingdong prefecture level city in Shandong
Fortune Shop	White Intelligent Power Saver	It's not much use.	Jingdong prefecture level city in Shandong
Fortune Shop	White Intelligent Power Saver	It doesn't save electricity.	Jingdong prefecture level city in Shandong
Fortune Shop	White Intelligent Power Saver	It's not much use.	Jingdong prefecture level city in Shandong
Fortune Shop	White Intelligent Power Saver	Tried it for a few days and tracked the record, and there is no difference from before.	Jingdong prefecture level city in Shandong
Fortune Shop	White Intelligent Power Saver	It's not very credible, so just do it yourself and turn off the lights diligently!	Jingdong prefecture level city in Shandong
Fortune Shop	White Intelligent Power Saver	Too bad, no power saving at all, scam people don't fall for it!	Jingdong prefecture level city in Shandong

Fortune Shop	White Intelligent Power Saver	It's not working at all.	Jingdong prefecture level city in Shandong
Fortune Shop	White Intelligent Power Saver	general	Jingdong prefecture level city in Shandong
Fortune Shop	White Intelligent Power Saver	(of an unmarried couple) be close	Jingdong prefecture level city in Shandong
Fortune Shop	White Intelligent Power Saver	of no use	Jingdong prefecture level city in Shandong
Fortune Shop	White Intelligent Power Saver	Actually can really save electricity, only used half a month to save more than 50, recommended to the neighbors to use together!	Jingdong prefecture level city in Shandong
Fortune Shop	White Intelligent Power Saver	Save what you can. Isn't it more cost effective to buy one of these than to pay the electric bill?	Jingdong prefecture level city in Shandong
Fortune Shop	White Intelligent Power Saver	Received, a use do not know how, use a month to see the effect is good, the effect is good then recommended, not good then I just do not recommend.	Jingdong prefecture level city in Shandong
Fortune Shop	White Intelligent Power Saver	Just received the goods, is a voltage regulator, next month to evaluate whether it is really power saving	Jingdong prefecture level city in Shandong
Fortune Shop	White Intelligent Power Saver	Very good, used a few days obviously feel that the electricity bill is much less, wait a month to see how much can be saved!	Jingdong prefecture level city in Shandong
Fortune Shop	White Intelligent Power Saver	Received the stuff used for more than a month without a little use, it feels like it uses more electricity than before	Jingdong prefecture level city in Shandong
Fortune Shop	White Intelligent Power Saver	The quality is very good, completely exceeded the expectations, the delivery speed is very fast, the packaging is very careful and strict, very satisfied with a shopping	Jingdong prefecture level city in Shandong
Fortune Shop	White Intelligent Power Saver	Fake, doesn't work at all. Don't even sell it.	Jingdong prefecture level city in Shandong
Fortune Shop	White Intelligent Power Saver	This item doesn't seem to have much effect?	Jingdong prefecture level city in Shandong
Fortune Shop	White Intelligent Power Saver	This production is a bit fake, just look at the focus on Ming	Jingdong prefecture level city in Shandong
Fortune Shop	White Intelligent Power Saver	The logistics is fast the service attitude is very good.	Jingdong prefecture level city in Shandong
Fortune Shop	White Intelligent Power Saver	The meter is good quality, sturdy and durable. It is accurate. Easy to use. Saves a lot of things.	Jingdong prefecture level city in Shandong
Fortune Shop	White Intelligent Power Saver	Just received the goods, don't know how it is yet	Jingdong prefecture level city in Shandong
Fortune Shop	White Intelligent Power Saver	Energy saver received, logistics is very fast, the quality is very good, the appearance is also very beautiful power saving is the most important.	Jingdong prefecture level city in Shandong
Fortune Shop	White Intelligent Power Saver	It's good to talk about it, it's not practical to use it.	Jingdong prefecture level city in Shandong
Fortune Shop	White Intelligent Power Saver	I don't know if it's good for now, I'll comment after using it	Jingdong prefecture level city in Shandong
Fortune Shop	White Intelligent Power Saver	I've been using it for two or three days. It's not energy efficient.	Jingdong prefecture level city in Shandong
Fortune Shop	White Intelligent Power Saver	Packaging is intact, logistics is also fast, cost-effective, inexpensive, very satisfied with a purchase	Jingdong prefecture level city in Shandong
Fortune Shop	White Intelligent Power Saver	In use to be observed!	Jingdong prefecture level city in Shandong
Fortune Shop	White Intelligent Power Saver	Use it for a while at a flat rate	Jingdong prefecture level city in Shandong
Fortune Shop	White Intelligent Power Saver	Very trustworthy brand, quality is guaranteed, the price is still affordable, quite satisfied!	Jingdong prefecture level city in Shandong
Fortune Shop	White Intelligent Power Saver	Great quality, great operation, easy to install, great price!	Jingdong prefecture level city in Shandong
Fortune Shop	White Intelligent Power Saver	I came back and installed it, it really saves electricity, much more than before, the	Jingdong prefecture level city in Shandong
Fortune Shop	White Intelligent Power Saver	Fast logistics, good quality, very good packaging, good customer service	Jingdong prefecture level city in Shandong
Fortune Shop	White Intelligent Power Saver	Very good quality, especially good, effective, satisfactory	Jingdong prefecture level city in Shandong
Fortune Shop	White Intelligent Power Saver	I don't know how well it works.	Jingdong prefecture level city in Shandong
Fortune Shop	White Intelligent Power Saver	Good value for money, recommend it haha	Jingdong prefecture level city in Shandong
Fortune Shop	White Intelligent Power Saver	Good. Good. Very good.	Jingdong prefecture level city in Shandong
Fortune Shop	White Intelligent Power Saver	It's not working.	Jingdong prefecture level city in Shandong
Fortune Shop	White Intelligent Power Saver	It's not working.	Jingdong prefecture level city in Shandong
Fortune Shop	White Intelligent Power Saver	In use	Jingdong prefecture level city in Shandong
Fortune Shop	White Intelligent Power Saver	I'll try it later.	Jingdong prefecture level city in Shandong
Fortune Shop	White Intelligent Power Saver	It's not much use.	Jingdong prefecture level city in Shandong
Fortune Shop	White Intelligent Power Saver	It doesn't save electricity.	Jingdong prefecture level city in Shandong
Fortune Shop	White Intelligent Power Saver	It's not much use.	Jingdong prefecture level city in Shandong
Fortune Shop	White Intelligent Power Saver	Tried it for a few days and tracked the record, and there is no difference from before.	Jingdong prefecture level city in Shandong
Fortune Shop	White Intelligent Power Saver	It's not very credible, so just do it yourself and turn off the lights diligently!	Jingdong prefecture level city in Shandong
Fortune Shop	White Intelligent Power Saver	Too bad, no power saving at all, scam people don't fall for it!	Jingdong prefecture level city in Shandong
Fortune Shop	White Intelligent Power Saver	It's not working at all.	Jingdong prefecture level city in Shandong
Fortune Shop	White Intelligent Power Saver	general	Jingdong prefecture level city in Shandong
Fortune Shop	White Intelligent Power Saver	(of an unmarried couple) be close	Jingdong prefecture level city in Shandong
Fortune Shop	White Intelligent Power Saver	of no use	Jingdong prefecture level city in Shandong
Fortune Shop	White Intelligent Power Saver	Actually can really save electricity, only used half a month to save more than 50, recommended to the neighbors to use together!	Jingdong prefecture level city in Shandong
Fortune Shop	White Intelligent Power Saver	Save what you can. Isn't it more cost effective to buy one of these than to pay the electric bill?	Jingdong prefecture level city in Shandong
Fortune Shop	White Intelligent Power Saver	Received, a use do not know how, use a month to see the effect is good, the effect is good then recommended, not good then I just do not recommend.	Jingdong prefecture level city in Shandong
Fortune Shop	White Intelligent Power Saver	Just received the goods, is a voltage regulator, next month to evaluate whether it is really power saving	Jingdong prefecture level city in Shandong
Fortune Shop	White Intelligent Power Saver	Very good, used a few days obviously feel that the electricity bill is much less, wait a month to see how much can be saved!	Jingdong prefecture level city in Shandong

Fortune Shop	White Intelligent Power Saver	Received the stuff used for more than a month without a little use, it feels like it uses more electricity than before	Jingdong prefecture level city in Shandong
Fortune Shop	White Intelligent Power Saver	The quality is very good, completely exceeded the expectations, the delivery speed is very fast, the packaging is very careful and strict, very satisfied with a shopping	Jingdong prefecture level city in Shandong
Fortune Shop	White Intelligent Power Saver	Fake, doesn't work at all. Don't even sell it.	Jingdong prefecture level city in Shandong
Fortune Shop	White Intelligent Power Saver	This item doesn't seem to have much effect?	Jingdong prefecture level city in Shandong
Fortune Shop	White Intelligent Power Saver	This production is a bit fake, just look at the focus on Ming	Jingdong prefecture level city in Shandong
Fortune Shop	White Intelligent Power Saver	The logistics is fast the service attitude is very good.	Jingdong prefecture level city in Shandong
Fortune Shop	White Intelligent Power Saver	The meter is good quality, sturdy and durable. It is accurate. Easy to use. Saves a lot of things.	Jingdong prefecture level city in Shandong
Fortune Shop	White Intelligent Power Saver	Just received the goods, don't know how it is yet	Jingdong prefecture level city in Shandong
Fortune Shop	White Intelligent Power Saver	Energy saver received, logistics is very fast, the quality is very good, the appearance is also very beautiful power saving is the most important.	Jingdong prefecture level city in Shandong
Fortune Shop	White Intelligent Power Saver	It's good to talk about it, it's not practical to use it.	Jingdong prefecture level city in Shandong
Fortune Shop	White Intelligent Power Saver	I don't know if it's good for now, I'll comment after using it	Jingdong prefecture level city in Shandong
Fortune Shop	White Intelligent Power Saver	I've been using it for two or three days. It's not energy efficient.	Jingdong prefecture level city in Shandong
Fortune Shop	White Intelligent Power Saver	Packaging is intact, logistics is also fast, cost-effective, inexpensive, very satisfied with a purchase	Jingdong prefecture level city in Shandong
Fortune Shop	White Intelligent Power Saver	In use to be observed!	Jingdong prefecture level city in Shandong
Fortune Shop	White Intelligent Power Saver	Use it for a while at a flat rate	Jingdong prefecture level city in Shandong
Fortune Shop	White Intelligent Power Saver	Very trustworthy brand, quality is guaranteed, the price is still affordable, quite satisfied!	Jingdong prefecture level city in Shandong
Fortune Shop	White Intelligent Power Saver	Great quality, great operation, easy to install, great price!	Jingdong prefecture level city in Shandong
Fortune Shop	White Intelligent Power Saver	I came back and installed it, it really saves electricity, much more than before, the	Jingdong prefecture level city in Shandong
Fortune Shop	White Intelligent Power Saver	Fast logistics, good quality, very good packaging, good customer service	Jingdong prefecture level city in Shandong
Fortune Shop	White Intelligent Power Saver	Very good quality, especially good, effective, satisfactory	Jingdong prefecture level city in Shandong
Fortune Shop	White Intelligent Power Saver	I don't know how well it works.	Jingdong prefecture level city in Shandong
Fortune Shop	White Intelligent Power Saver	Good value for money, recommend it haha	Jingdong prefecture level city in Shandong
Fortune Shop	White Intelligent Power Saver	Good. Good. Very good.	Jingdong prefecture level city in Shandong
Fortune Shop	White Intelligent Power Saver	It's not working.	Jingdong prefecture level city in Shandong
Fortune Shop	White Intelligent Power Saver	It's not working.	Jingdong prefecture level city in Shandong
Fortune Shop	White Intelligent Power Saver	In use	Jingdong prefecture level city in Shandong
Fortune Shop	White Intelligent Power Saver	I'll try it later.	Jingdong prefecture level city in Shandong
Fortune Shop	White Intelligent Power Saver	It's not much use.	Jingdong prefecture level city in Shandong
Fortune Shop	White Intelligent Power Saver	It doesn't save electricity.	Jingdong prefecture level city in Shandong
Fortune Shop	White Intelligent Power Saver	It's not much use.	Jingdong prefecture level city in Shandong
Fortune Shop	White Intelligent Power Saver	Tried it for a few days and tracked the record, and there is no difference from before.	Jingdong prefecture level city in Shandong
Fortune Shop	White Intelligent Power Saver	It's not very credible, so just do it yourself and turn off the lights diligently!	Jingdong prefecture level city in Shandong
Fortune Shop	White Intelligent Power Saver	Too bad, no power saving at all, scam people don't fall for it!	Jingdong prefecture level city in Shandong
Fortune Shop	White Intelligent Power Saver	It's not working at all.	Jingdong prefecture level city in Shandong
Fortune Shop	White Intelligent Power Saver	general	Jingdong prefecture level city in Shandong
Fortune Shop	White Intelligent Power Saver	(of an unmarried couple) be close	Jingdong prefecture level city in Shandong
Fortune Shop	White Intelligent Power Saver	of no use	Jingdong prefecture level city in Shandong
Fortune Shop	King of Power Saving	Quality is very good with the effect is also very practical, the product in the home living room kitchen with special savings savings, product equipment environmental protection mute, is also highlighting the importance of the product, the product is also with automatic multi-functional power, for the family is also more energy-saving, the effect is particularly obvious, but also very affordable, the price in the	Jingdong prefecture level city in Shandong
Fortune Shop	King of Power Saving	I can't believe there is such an awesome product, can really do to save electricity, our home power consumption is relatively large, electricity is too expensive, with this you can just turn on the air	Jingdong prefecture level city in Shandong
Fortune Shop	King of Power Saving	A friend introduced a product, with the description, the price is not expensive, logistics is very fast!	Jingdong prefecture level city in Shandong
Fortune Shop	King of Power Saving	It is genuine, and the same as the physical store, the price is also favorable, decisively turn powder	Jingdong prefecture level city in Shandong
Fortune Shop	King of Power Saving	Looks great, feels good in the hand, easy to use, quick, thanks!	Jingdong prefecture level city in Shandong
Fortune Shop	King of Power Saving	After receiving the goods, immediately plugged into the power outlet on, carefully observe a little, feel very satisfied, the logistics packaging services are to play a five-star evaluation, the actual has been very letter lazy Jingdong.	Jingdong prefecture level city in Shandong
Fortune Shop	King of Power Saving	The home uses too much electricity, the electricity bill really can not eat, with this magic tool a month less two hundred dollars of electricity, really thank you!	Jingdong prefecture level city in Shandong
Fortune Shop	King of Power Saving	Nice feel, easy to use and affordable!	Jingdong prefecture level city in Shandong
Fortune Shop	King of Power Saving	Hello, this does not save electricity at all, can I tease the goods?	Jingdong prefecture level city in Shandong
Fortune Shop	King of Power Saving	It doesn't save any power at all over the course of a month	Jingdong prefecture level city in Shandong
Fortune Shop	In-situ compensated economizer	not have	Jingdong prefecture level city in Shandong

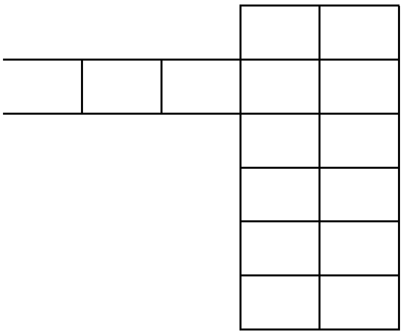

